# Genome-wide protein-protein interactions and protein function exploration in cyanobacteria

**DOI:** 10.1038/srep15519

**Published:** 2015-10-22

**Authors:** Qi Lv, Weimin Ma, Hui Liu, Jiang Li, Huan Wang, Fang Lu, Chen Zhao, Tieliu Shi

**Affiliations:** 1 Center for Bioinformatics and Computational Biology, and the Institute of Biomedical Sciences, School of Life Sciences, East China Normal University, 500 Dongchuan Road, Shanghai, 200241, China; 2 College of Life and Environment Sciences, Shanghai Normal University, 100 Guilin Road, Shanghai, 200234, China; 3 The institute of plant physiology and ecology, Shanghai Institutes for Biological Sciences, Chinese Acedamy of Sciences, 300 Fenglin Road, Shanghai 200032, China

## Abstract

Genome-wide network analysis is well implemented to study proteins of unknown function. Here, we effectively explored protein functions and the biological mechanism based on inferred high confident protein-protein interaction (PPI) network in cyanobacteria. We integrated data from seven different sources and predicted 1,997 PPIs, which were evaluated by experiments in molecular mechanism, text mining of literatures in proved direct/indirect evidences, and “interologs” in conservation. Combined the predicted PPIs with known PPIs, we obtained 4,715 no-redundant PPIs (involving 3,231 proteins covering over 90% of genome) to generate the PPI network. Based on the PPI network, terms in Gene ontology (GO) were assigned to function-unknown proteins. Functional modules were identified by dissecting the PPI network into sub-networks and analyzing pathway enrichment, with which we investigated novel function of underlying proteins in protein complexes and pathways. Examples of photosynthesis and DNA repair indicate that the network approach is a powerful tool in protein function analysis. Overall, this systems biology approach provides a new insight into posterior functional analysis of PPIs in cyanobacteria.

Cyanobacteria, the only known prokaryotes capable of oxygenic photosynthesis, are one of the most popular model organisms for photosynthesis, respiration, energy metabolism and regulatory function researches. Many studies have indicated that cyanobacteria could be applied in the wastewater treatment^1^, and significantly produce renewable energy source, like ethanol, biodiesel, hydrogen, etc.^2^,^3^,^4^. To date, our understanding to the molecular mechanisms underlying these biological functions is incomplete. For example, up to 60% of the proteins in *Synechocystis* sp. strain PCC 6803 are annotated as “unknown function” or “hypothetical protein”, although this organism is the first phototrophic organism fully sequenced and commonly selected in proteome analysis. To gain new insight into the important biological processes in cyanobacteria, protein-protein interaction (PPI) network construction and network-based protein function prediction are essential by providing a global understanding of protein relationships^5^,^6^. Experimental methods are focusing on genome-wide PPIs detection with yeast two-hybrid (Y2H) system and tandem affinity purification (TAP) coupled with mass spectroscopy^5^,^7^,^8^. Specifically, a Y2H screening system identified 3,236 interactions that provides new insight for gene function analyses in *Synechocystis* sp. strain PCC 6803^9^. However, these experimental methods have their own limitations^10^. Firstly, they are labor- and time-intensive associated with high cost. Secondly, the experimental methods are prone to false positives. Thirdly, they are condition-specific and method-specific, which lead to a lower overlap even with the same species in the same platform.

Alternatively, computational methods have been widely used to effectively infer genome-wide PPIs and provide insight into protein properties in biological systems^11^,^12^,^13^. Such studies were also undertaken in *Synechocystis* sp. strain PCC 6803, such as SynechoNET database that integrated PPIs by domain information^14^ and InteroPORC database that inferred highly conserved PPIs^15^. However, the data from single source are bias in predicting PPIs, thus it is critical to integrate data computationally from multiple sources to construct high quality and coverage PPI network of an organism. For example, integration of multiple independent positive training datasets to predict PPIs can effectively reduce bias originally from single dataset by giving confidence scores for PPIs^16^,^17^. Also, in model plant Arabidopsis, integration of indirect evidences from multiple datasets by either Bayesian approach^18^ or support vector machine model^19^ has identified genome-wide PPIs with high reliability. Multiple datasets of indirect evidences to predict PPIs include genomic, evolutionary, domain, expression profiles and Gene Ontology (GO) information. Genomic context method contains gene neighborhood conservation, gene fusion and gene cluster. The assumption of gene fusion is that homologs of some interacting protein pairs in another species fuse into a single protein chain^20^,^21^. Gene neighborhood method presumes that the genes encoding interacting protein pairs are closely located and this closeness is conserved across different genomes^22^. Gene clusters assume that proteins, transcribed from a single functional unit (operon), are likely to have functional relation^23^. The evolutionary information, phylogenetic profile, assumes that functional related proteins are conserved in other organism^24^. Domain based information applies known interacting domains to predict potential protein interactions^25^. Besides, expression profiles and Gene Ontology (GO) annotation are also efficiently used to predict PPIs^18^.

Insights to the function of proteins and the mechanisms of biological processes can be gained by systematic analyses of large scale PPI network. A great number of studies predicted protein functions based on the assumption that functional similar proteins would cluster together in network and that interacting protein partners share similar function^6^. For example, the assignment of proteins to functional classes can be determined by simulated annealing method based on global optimization which minimizes the number of protein interactions among different functional classes^26^. This method solves the complicated computational problem resulting from global minimization from complex network and is the recommendatory method in global protein function prediction from PPI network.

In this work, we proposed a systematic approach to construct a high confident PPI network with predicted PPIs by integrating seven different datasets and known PPIs in *Synechocystis* sp. strain PCC 6803 (Fig. 1a). The quality of this network was evaluated by Y2H experiments, text mining and conserved interologs. We then conducted subsequent functional analysis based on the PPI network to deeply explore the annotation of function-unknown proteins, novel latent proteins in protein complexes and relative mechanisms of functional modules. Examples closely to biological processes of cyanobacteria were illustrated, suggesting that this systems biology approach is a powerful tool in PPI analysis of *Synechocystis* sp. strain PCC 6803.

## Results

### PPI prediction in *Synechocystis* sp. strain PCC 6803

To predict genome-wide PPIs in *Synechocystis* sp. strain PCC 6803, we first constructed gold standard datasets of PPIs. For gold standard positive dataset (GSP), we collected 2,718 known PPIs among 1,048 proteins confirmed by Y2H experiments^9^ (Supplementary Table S1). For the gold standard negative dataset (GSN) which means the protein pairs without interactions, we first generated protein pairs by collecting proteins from different cellular locations according to GO. After removing proteins with possible mobility or secretion, we obtained final GSN with 18,919 protein pairs (Supplementary Table S2).

We predicted PPIs with seven different data: gene clusters^23^, microarray gene expression profiles^12^, smallest shared biological process (SSBP)^12^, gene fusion^20^,^21^, gene neighborhood^20^,^21^, gene co-occurrences or phylogenetic profile^21^,^27^ and domain interaction^12^,^20^. Using GSP and GSN sets as positive and negative data, we calculated the likelihood ratio (LR) for each PPI in each data source, and then integrated these LRs with Naïve Bayes approach to generate high confident PPIs with integrated LR scores and posterior odds (see method). Finally, we obtained 1,997 high confident PPIs involving 2,765 proteins (Supplementary Table S3).

**Figure 1:**
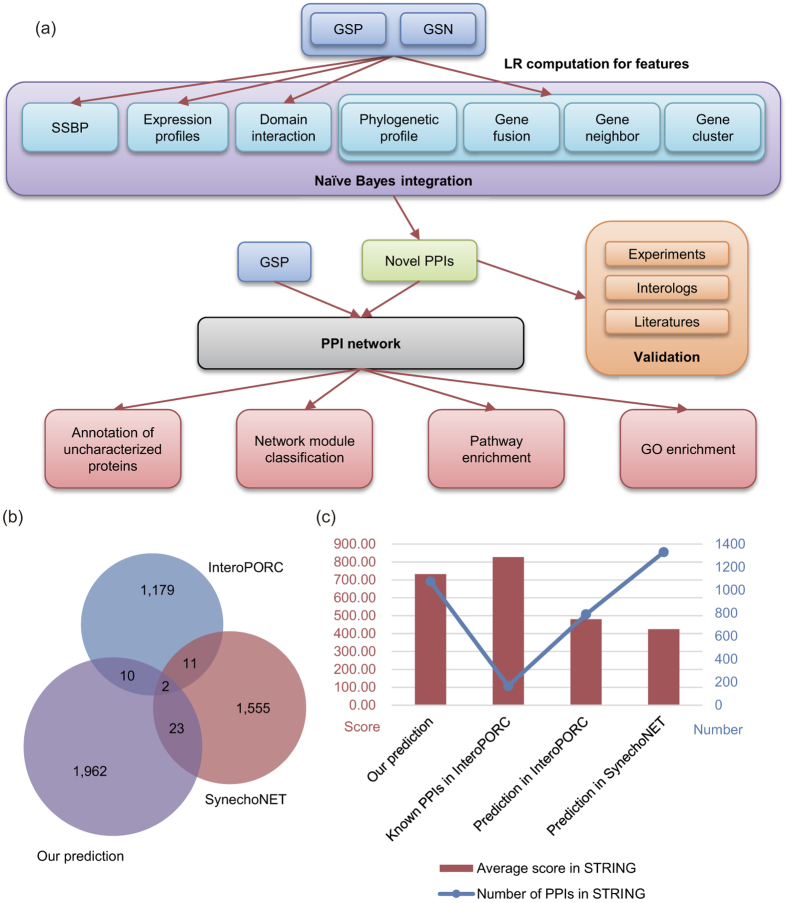
Workflow of the study and the comparisons with other data. (**a**) Workflow of protein-protein interaction prediction by Naïve Bayes integration and PPI network analyses. PPIs were predicted with seven different features: smallest shared biological process (SSBP), microarray gene expression profiles, domain interaction, phylogenetic profile, gene fusion, gene neighborhood and gene cluster. Using gold standard positive dataset (GSP) and gold standard negative dataset (GSN) sets as positive and negative data, we calculated the likelihood ratio (LR) for each PPI in each feature, and then integrated these LRs with Naïve Bayes approach. (**b**) The overlapped and unique PPIs predicted in this study and two other similar studies, InteroPORC and SynechoNET. (**c**) Evaluation and comparison of our prediction, InteroPORC and SynechoNET using an independent data, STRING scores.

Compared with two former studies on PPI prediction in *Synechocystis* sp. strain PCC 6803, we found a quite small overlap between the three datasets (Fig. 1b), indicating that different predictive method has its own inherent bias on PPI prediction, which is consistent with previous study^28^. To evaluate these different prediction datasets, we applied an independent data as benchmarks to evaluate the PPIs from different sources (Fig. 1c): the scores of PPIs in STRING database that contains global PPIs across nearly 100 fully sequenced genomes^29^. Our high confident PPIs were compared with three PPI datasets: an independent positive data in InteroPORC (182 PPIs), predicted PPIs in both InteroPORC and SynechoNET. The average score of our data (732.17) is more closer to that of the independent positive data (827.54) than that of predicted PPIs in other two databases (InteroPORC: 479.94; SynechoNET: 424.67). More remarkable, the performance of our result is better than the prediction in InteroPORC, although these independent positive data were used to infer PPIs in InteroPORC. All these results support the view that our prediction method is effective in generating high score PPIs. Furthermore, nearly half of our predicted PPIs are not contained in STRING database, suggesting the value of our data in novel PPI discovery.

### Evaluation of high confident PPIs using experiments, text mining and interologs

To evaluate these high confident PPIs, we applied three approaches from different aspects: experiments in physiological interaction evidences, text mining of literatures in proved direct/indirect evidences and “interologs” in conservation evidences.

**Figure 2:**
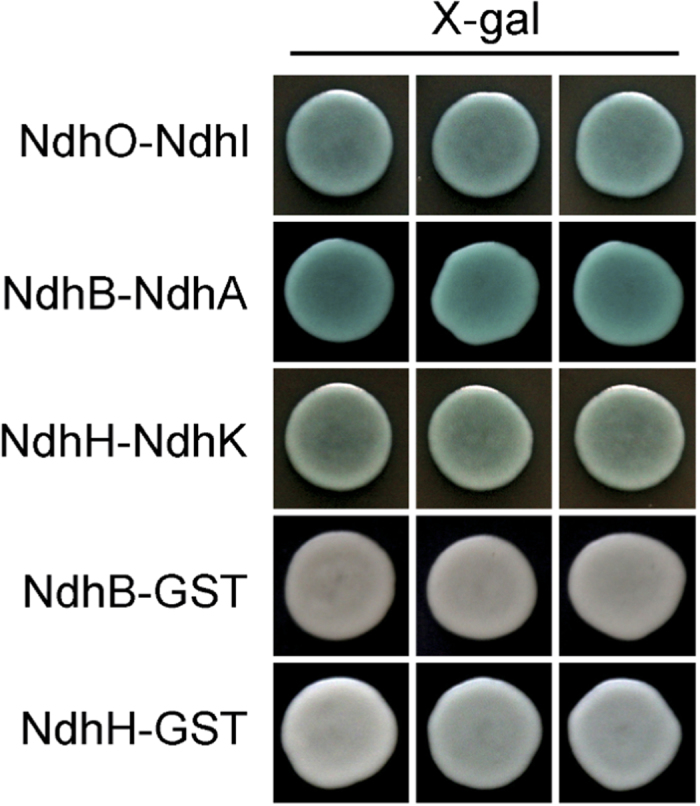
Yeast two-hybrid system for the interaction identification among Ndh subunits. NdhB, NdhH and NdhO were constructed into bait vectors, whereas NdhA, NdhI, NdhK and GST were constructed into prey vectors, respectively. Subsequently, they were transformed into the yeast strain EGY48. Transformed yeast was dropped onto X-gal medium. Blue precipitate represents accumulated β-galactosidase activity resulting from the activation of the lacZ reporter gene by protein-protein interaction. The induction plate was incubated at 30 °C for 22 h and then photographed. The interactions of NdhO-NdhI, NdhB-GST and NdhH-GST were assayed as one positive and two negative controls, respectively. At least six independent experiments were performed, and the result of one representative is shown. See Supplementary Figure S1 for full views of yeast two-hybrid results.

**Figure 3:**
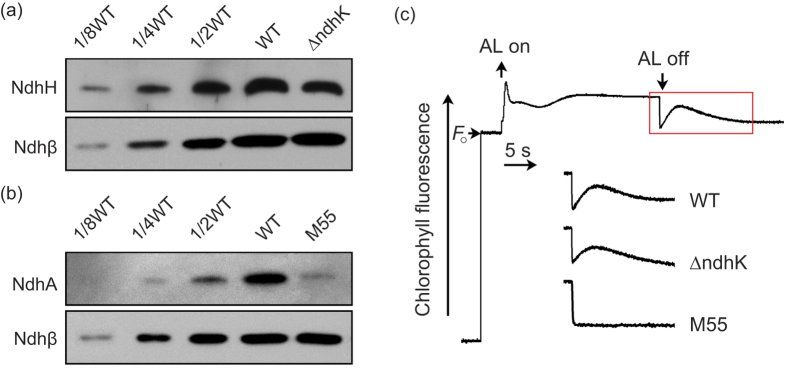
Functional analysis of PPI protein pairs. (**a**) Immunodetection of Ndh subunits in thylakoid membranes from the WT (including indicated serial dilutions) and ∆*ndhK* strains. Immunoblotting was performed with antibodies against NdhH subunit. Lanes were loaded with thylakoid membrane proteins corresponding to 1 μg chlorophyll *a* and ATPβ was used as a loading control. See Supplementary Figure S2 for full views of western blots. (**b**) Immunodetection of Ndh subunits in thylakoid membranes from the WT (including indicated serial dilutions) and M55 strains. Immunoblotting was performed with antibodies against NdhA subunit. Lanes were loaded with thylakoid membrane proteins corresponding to 1 μg chlorophyll a and ATPβ was used as a loading control. See Supplementary Figure S2 for full views of western blots. (**c**) Monitoring of NDH-CET activity using chlorophyll fluorescence analysis. The top curve shows a typical trace of chlorophyll fluorescence in the WT *Synechocystis* sp. strain PCC 6803. The chlorophyll *a* concentration was adjusted to 10 μg mL^−1^ before measurement. Cells were exposed to AL (620 nm; 45 μmol photons m^−2^s^−1^) for 30 s. AL was turned off, and the subsequent change in the chlorophyll fluorescence level was monitored as an indicator of NDH-CET activity.

**Figure 4:**
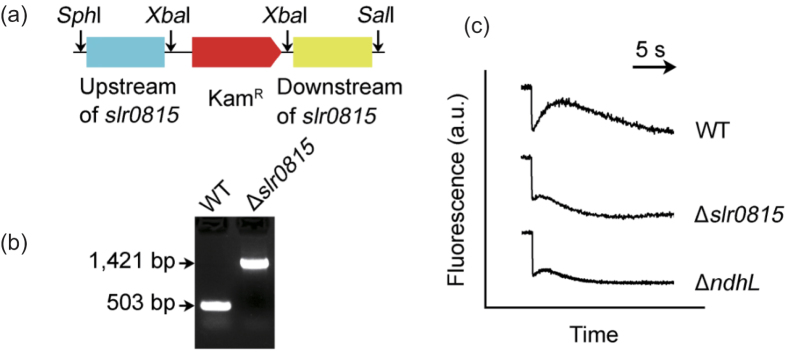
*slr0815* gene deletion mutation and its effect on NDH-CET. (**a**) Construction of plasmid used to generate the *slr0815* deletion mutant (∆*slr0815*). (**b**) PCR segregation analysis of the ∆*slr0815* mutant using the *slr0815*-G and *slr0815*-H primer sequences (Supplemental Table S4). See Supplementary Figure S3 for full views of DNA electrophoresis gels. (**c**) Monitoring of NDH-CET activity by chlorophyll fluorescence. Two-day-old cells were exposed to actinic light (45 μmol photons m^−2^s^−1^) for 30 sec. After illumination, the subsequent transient change in chlorophyll fluorescence was monitored as an indication of NDH-CET activity. a.u., arbitrary units.

We first carried out experiments to verify high confident PPIs between critical protein complex members (Supplementary Table S4). In *Synechocystis* sp. strain PCC 6803, functional distinct multiple NADPH dehydrogenase (NDH-1) complexes are essential for CO_2_ uptake, cyclic electron transport around photosystem I and respiration. Therefore, we tested the interactions between 6 proteins related to the large-size NDH-1 complex (NDH-1L) by yeast two-hybrid method (Sll0519, NdhA; Sll0223, NdhB; Slr0261, NdhH; Sll0520, NdhI; Slr1280, NdhK; and Ssl1690, NdhO). The experimental results proved the interactions of NdhO-NdhI, NdhH-NdhK and NdhA-NdhB (Fig. 2). All of these PPIs located either in hydrophilic or hydrophobic NDH-1L sub-complex, but not between them, indicating the reliability of these predicted interactions. To investigate the physiological significance of the interaction among the NDH-1 subunits, we tested the protein expression levels of two subunits, NdhH and NdhA using the mutant strains defective in the PPI pair NdhK and NdhB, respectively, by immunodetection. By comparison with wild type (WT), the accumulation of NdhH in the NdhK defective mutant ∆*ndhK* decreased to more than half (Fig. 3a) and the NdhA in the NdhB defective mutant M55 lowered to nearly 1/4 (Fig. 3b), suggesting the dependence of the PPI pair in protein accumulation. Further, we compared the activity of NDH-1-dependent cyclic electron flow around PSI (NDH-CET) via analyzing the post-illumination increase in chlorophyll fluorescence after termination of actinic light. NDH-CET was completely inactivated in M55, being consistent with previous studies^30^ and partly in ∆*ndhK* (Fig. 3c), indicating the PPI pairs of NDH-1 are involved in regulation of the NDH-CET activity. It is worth noting that predicted protein protein interaction through integrative bioinformatics approaches include both of physical protein or domain interactions and the functional associations between potentially interacting proteins^31^. For example, the product of gene *slr0815* was predicted to interact with NdhL (Ssr1386; Supplemental Table S3), and deletion of *slr0815* almost completely impaired NDH-CET activity (Fig. 4a–c), being consistent with the results of *ndhL*-deleted mutant (∆*ndhL*)^30^.

We then evaluated the high confident PPIs by mining published literature evidences of double-mutant phenotypes and Y2H results (Supplementary Table S5). Totally, 6 and 19 PPIs were verified by double-mutants and Y2H results with significance (p < 1e–4) that were computed by probability according to a hypergeometric model^15^. On one hand, we examined whether double-mutants lose normal function compared with the WT, which suggests intermolecular interactions between proteins^32^. For instance, in NDH-1 family, the predicted interaction between Slr0331 (NdhD1) and Slr1291 (NdhD2) is consistent with the double mutant ∆*ndhD1/ndhD2* (∆*D1/D2*), which loses capability to grow under photoheterotrophic conditions and exhibits low respiration rate^33^. Another predicted pair of NDH-1 family members, Sll1733 (NdhD3) and Sll0027 (NdhD4), is also verified by their double mutant, ∆*ndhD3/ndhD4* (∆*D3/D4*), which grows slowly in air and cannot uptake CO_2_^34^. As for the photosystem II (PSII) complex, under situation of absence of glucose, the double mutant ∆*psbO/psbV* strain lost the ability to grow^35^, supporting the high confident PPI, Sll0258 (PsbV) and Sll0427 (PsbO). Moreover, high confident PPI between Sll0258 (PsbV) and Sll1194 (PsbU) was verified by double deletion mutant ∆*psbU/psbV*, which cannot grow in the environment of absence Ca^2+^ or Cl^−^, compared with the ∆*psbU* and WT strains^36^. Similarly, experimental evidence for the ∆*cpcG1/cpcG2* double mutant also confirmed the predicted functional linkage between Slr2051 (CpcG1) and Sll1471 (CpcG2)^37^. On the other hand, text mining of Y2H experiments provides direct evidence of the high confident PPIs. For example, Y2H experiments verified two PPIs between PSII complex members: Sll1867 (PsbA3, photosystem II D1 protein) and Slr1311 (PratA, photosynthesis and respiration PSII)^38^; Sll0698 (Hik33, two-component sensor histidine kinase) and Ssl3451 (SipA, hypothetical protein)^39^. In addition, the formation of a reaction center pre-complex with D1 and Psbl^40^ also supported our prediction: Sml0001(Psbl, PSII reaction center PsbI protein) interacts with Sll1867, Slr1311 and Slr1181 (all these three proteins are PSII proteins). As for PSI complex members, experimental results of physical interactions proved the reliability of two PPIs: Slr0737 (PsaD) and Slr1655 (PsaL), Ssr2831 (PsaE) and Sll0819 (PsaF)^41^.

Last, we utilized the “conserved interologs” of PPIs from 25 organisms to verify the interactions between conserved proteins, based on the assumption that interactions between ortholog pairs are conserved across different organisms^42^. On average, 17.7% of predictions were verified in interologs across the 25 species (Supplementary Figure S4) and the union of all conserved PPIs is 449. For example, one predicted interaction pair, Slr0543 (TrpB, tryptophan synthase subunit beta) and slr0966 (trpA, tryptophan synthase subunit alpha), has the related physical interacting interolog in *E.coli,* NP_415777 with NP_415776^43^. Similarly, another predicted interaction pair, Slr1199 (MutL, DNA mismatch repair protein) and sll1165 (MutS, DNA mismatch repair protein) has the corresponding experimental confirmed interolog in *E. coli*, NP_418591 and NP_417213^44^. Interologs could also suggest that some predicted PPIs form complexes, such as Sll1260 (Rps2, 30S ribosomal protein S2) with Ssl3432 (Rps19, 30S ribosomal protein S19), and Sll1260 with Sll1097 (Rps7, 30S ribosomal protein S7), both confirmed between their orthologs NP_011859 with NP_014435, and NP_011859 with NP_012647 in *S. Cerevisae*^45^. Actually, NP_011859 is mitochondrial 37S ribosomal protein MRP4, NP_014435 represents mitochondrial 37S ribosomal protein S19 and NP_014435 means mitochondrial 37S ribosomal protein S7. The three proteins are all ribosomal subunits involving in cellular process of translation.

**Figure 5:**
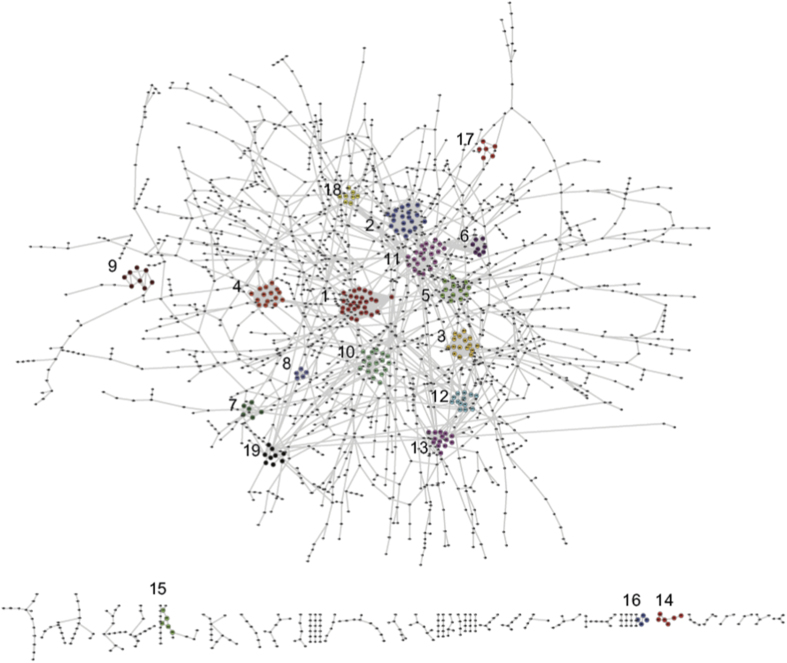
19 sub-networks in PPI network. The PPI network (4,715 protein pairs involving 3,231 proteins) was constructed with high confident predicted PPIs and GSP (known confirmed PPIs). The whole PPI network was partitioned by the molecular complex detection algorithm (MCODE) with the default parameter in Cytoscape into 19 dense protein sets, which were extended by adding the first-layer neighbor proteins in PPI network to generate the final 19 sub-networks that were marked with numbers.

Overall, the above evaluation suggests the reliability of our high confident PPIs that could be used in further analysis. Since GSP is considered as known confirmed PPIs, we combined them with our high confident PPIs, making final 4,715 protein pairs involving 3,231 proteins after eliminating redundancy to construct PPIs network (Fig. 5) for subsequently PPI network analysis and unknown protein function annotation.

### GO terms assignment of proteins with unknown function

Traditional homology method employed protein function alignment from one protein to its cousins, which were assumedly descended from the same ancestor to roughly assign functions of almost 40%–70% protein in a genome^46^. However, there are extremely less proteins with unknown function, which have homological proteins in proximal model species. In *Synechocystis* sp. strain PCC 6803, based on the existing protein annotation nearly 60% proteins are of unknown function, so we applied a non-homology method, simulated annealing algorithm, to annotate the proteins of unknown function based on PPI network (see method).

Totally, we annotated 1,391 proteins with 160 GO terms in biological process, 1,518 proteins with 10 GO terms in cellular component and 1,366 proteins with 55 GO terms in molecular function (Supplementary Table S6). Those function-unknown proteins were mainly assigned for metabolic process, carbohydrate biosynthetic process, catalytic activity and transporter activity. For instance, one hypothetical protein (Sll1252) was predicted to be involved in ion trans-membrane transporter activity (GO:0015075), monovalent inorganic cation trans-membrane transporter activity (GO:0015077). Recently, Sll1252 was reported closely related to redox sensing of the plastoquinone pool to balance the photosynthetic electron flow and cope with global environmental stresses^47^. Another hypothetical protein (Sll0822) was predicted involving in regulation of gene expression (GO:0010468), nitrogen compound metabolic process (GO:0006807), cellular response to stimulus (GO:0051716), stress (GO:0006950), and extracellular stimulus (GO:0009991). Experiment result suggests that this protein is involved in the regulation of nitrogen uptake systems and acts as a repressor, or as part of a repressor complex^48^.

### Identification and function annotation of function modules in PPI network

Function modules are important in revealing biological mechanism of complex PPI network. Well defined and annotated function modules could be achieved by extracting sub-network (a form of module which is effective in sparse topological relationship among proteins rather than merely pair) and pathway (a group of proteins with similar functions which cooperate with each other in related biological processes) from PPI network. Therefore, we identified sub-networks annotated by pathway enrichment analysis to define function modules for further analysis.

We first partitioned the whole PPI network by the molecular complex detection algorithm (MCODE) with the default parameter in Cytoscape^49^, and got 19 dense protein sets, which were extended by adding the first-layer neighbor proteins in PPI network to generate the final 19 sub-networks (Supplementary Table S7). We then undertook pathway enrichment analysis for each sub-network. 98 pathways were obtained from KEGG, most of which are involved in metabolic pathway (syn01100), biosynthesis of secondary metabolites (syn01110), ABC transporters (syn02010) and porphyrin and chlorophyll metabolism (syn00860). Through pathway enrichment analysis, 19 sub-networks were significantly enriched in 5 pathways, including metabolic pathways (syn01100), photosynthesis (syn00195), two-component system (syn02020) and photosynthesis-antenna proteins (syn00196). For example, sub-network 4 was enriched in photosynthesis-antenna proteins. And this sub-network includes 15 proteins, Slr2051 (CpcG), Sll1051 (CpcF), Slr2067 (ApcA), Slr1459 (ApcF), Sll1577 (CpcB), Slr1986 (ApcB), Ssr3383 (ApcC), Slr1878 (CpcE), Sll1580 (CpcC), Sll1579 (CpcC), Ssl3093 (CpcD), Sll1471 (CpcG), Sll1578 (CpcA), Sll0928 (ApcD) and Slr0335 (ApcE). These proteins are phycobili-proteins serving for external antenna proteins for PSII. Another example is sub-network 10 that was enriched in the two-component system pathway (syn02020). Two of sub-network 10 members, Sll1229 (Hik41) and Sll1228 (Hik4), were speculated to participate in the current two-component system in KEGG. In fact, both of the two proteins have been annotated as two-component hybrid sensor and regulator in Cyanobase.

### Mining PPI mechanism in function modules

Based on annotated function-unknown proteins and well-defined function modules, we could efficiently mining PPI mechanism. Focusing on purine metabolism and carbon dioxide, we deeply explored biological mechanism based on function modules.

The first example is sub-network 14. In this sub-network, six function unknown proteins (Slr1660, Slr1658, Slr1657, Sll0537, Slr1659 and Sll0536) were assigned to GO terms: catalytic activity (GO:0003824), cellular ketone metabolic process (GO:0042180), regulation of cellular process (GO:0050794), ligase activity (GO:0016874) and hydrolase activity (GO:0016787). These proteins with unknown function have no homologs in model organisms, so we explored their functions by transferring the function of proteins with the same GO terms from other species rather than sequence similarity. In *E. coli*, we found seven functional similar proteins (b0480, bifunctional UDP-sugar hydrolase/5'-nucleotidase; b0522, N5-carboxyaminoimidazole ribonucleotide synthase; b1849, phosphoribosylglycinamide formyltransferase 2; b3397, adenosine nucleotide hydrolase; b4005, phosphoribosylglycinamide synthetase phosphoribosylamine-glycine ligase; b4006, fused IMP cyclohydrolase/phosphoribosylaminoimidazolecarboxamide formyltransferase; and b4213, 2'-3'-cyclic-nucleotide 2'-phosphodiesterase), which were enriched in pathway purine metabolism (eco00230). By mapping same purine metabolism in *E.coli* and *Synechocystis* sp. PCC6803, we found two enzymes in purine metabolism pathway that could have the potential function of six proteins with unknown function in *Synechocystis* sp. PCC6803 (see Supplementary Figure S5).

**Figure 6:**
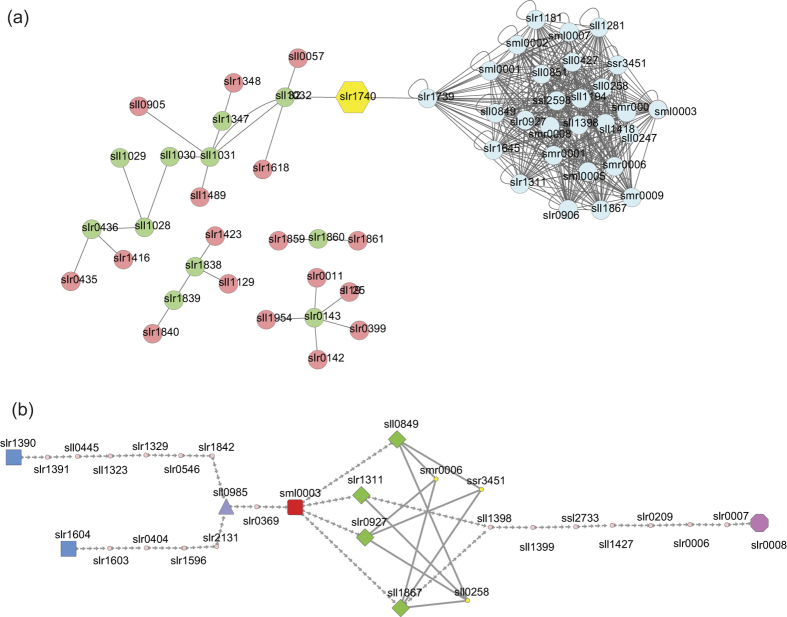
Examples of functional linkages in PPI network. (**a**) Protein-protein interactions in functional linkage of CO_2_ uptake and photosystem II. Sub-network 1 is enriched in photosynthesis, especially photosystem II and carbon dioxide. A key protein, slr1740 (appA, oligopeptide binding protein of ABC transporter) structured a highly important linkage between modules of photosynthesis and carbon dioxide, suggesting the potential function of slr1740 in carbon dioxide uptake and photosystem II. Blue nodes, proteins involved in the photosystem II process; green nodes, proteins involved in CO_2_ uptake; pink nodes, first neighbor of CO_2_ uptake proteins; yellow node, appA, oligopeptide binding protein of ABC transporter. (**b**) Protein-protein interactions in biological processes of photosystem II reaction center subunits under UV irradiation. Copies of FtsH (slr1390 and slr1640) separately interacting with their own relative proteins could both link with sml0003 (PS II reaction center M protein), which also interacts with D1 and D2 protein. D1 and D2 proteins then interact with cytochrome proteins, and finally affect slr0008 (CtpA, Carboxyl-terminal processing protease) by a series of PPIs. Blue nodes, FtsH copies; red node photosystem II reaction center M protein; green nodes, protein members of D1 and D2; yellow nodes, cytochrome proteins; pink node, CtpA.

The second example is sub-network 1, which was extremely enriched with photosynthesis, especially photosystem II and the biological process of carbon dioxide. Based on PPIs network, we found a key protein, Slr1740 (AppA, oligopeptide binding protein of ABC transporter) structuring a highly important linkage between modules of photosynthesis and carbon dioxide, suggesting the potential function of Slr1740 that relates carbon dioxide uptake to photosystem II (see Fig. 6a). In fact, ABC transporters are critical membrane proteins responsible for transport of manganese (Mn, composing functional Mn cluster in the photosystem II)^50^ and HCO_3_^−^ (accumulated in carbon dioxide uptake systems)^51^.

## Discussion

Here we present a comprehensive systems biology approach of integrating data from different sources, identifying high confident PPIs and undertaking a series downstream functional analyses based on PPI network to investigate protein function, especially proteins with unknown function. This method is verified to be efficient in predicting PPIs and understanding the biological mechanism of proteins, protein complexes and their molecular interactions.

Our work has advantages in data source, integrating method, PPIs qualities and PPI network analysis. Firstly, we applied seven different sources data including not only domain information, but also genome context, evolutionary and function information. Secondly, we applied Naïve Bayes approach to statistically integrate results by providing PPI probabilities. In addition, to enhance the power of prediction, we applied GSP and GSN to appraise the performance of each protein pairs with quantitative score. Thirdly, our high confident PPIs have higher coverage in genome and have been evaluated by different evidences. Specifically, we predicted 1,997 high confident PPIs involving 2,765 proteins, the reliability of which was confirmed by Y2H experiments, and assessed by text mining and conserved interologs. Furthermore, we tested the physiological significance of the PPI pairs of NDH-1 (Fig. 3). Fourthly, we constructed PPI network, and carried out subsequent functional analysis based on the PPI network to infer function of uncharacterized proteins and explore the mechanism of PPIs. As the result, functional modules not only strengthen the reliability of high confident PPIs, but also present underlying functional relationships between PPIs, particularly for protein family members in a certain pathway or cell complex (component).

In contrast, previous studies only predicted information-bias PPIs. For example, Kim *et al.* predicted less high confident PPIs (1,591 pairs involving 509 proteins) with simply combining domain biased information together^14^, and only 3 of their high confident PPIs overlap with yeast two-hybrid experimental results^9^; Michaut *et al.* only applied interologs to infer PPIs, making the prediction highly biased toward conservation and dependent on its unique data source^15^.

Besides informative examples in results, we take one more intriguing instance to demonstrate the application of our result by explaining biological processes of photosystem II reaction center subunits (both D1 and D2 proteins) under UV irradiation. Microorganisms, such as cyanobacteria, possess a range of compounds that absorb UV so that they are proposed to function as sunscreen^52^. Energy transfer, tetrapyrrole synthesis nitrate and ammonium uptake and cell differentiation would be negatively affected by UV light. UV-B irradiation results in the loss of steady state oxygen evolution and a parallel loss of the photosystem II reaction center subunits^53^. Additionally, in parallel with the transcripts of D1-processing protease (CtpA) and D1-degrading FtsH protease, wholly biosynthesis processing and degradation of D1 are coordinated^54^. By mining the whole biosynthesis and degradation of D1 protein pathway in our PPI network, we found a comparative intact path with UV irradiation (Fig. 6b). Copies of FtsH (Slr1390 and Slr1640) through separately interacting with their own relative proteins, could both link with Sml0003 (PS II reaction center M protein), which also interacts with D1 and D2 protein. D1 and D2 proteins then interact with cytochrome proteins, and finally affect Slr0008 (CtpA, Carboxyl-terminal processing protease) by a series of PPIs.

For future work, we will focus on high confident PPIs that have not been proved by existing evidence and try to explore their underlying biological function, such as Sll0654 (PhoA) and Sll0656 (NucH) in sub-network 17, Sll0320 (probable ribonuclease D) and Sll0319 (periplasmic protein, function unknown) in sub-network 15, Sll0431, Sll0651, Sll0201, Sll0700 (all are putative transposases) in sub-network 9.

As discussed above, our systems biology approach is efficient in predicting PPIs. And the resulting PPI network includes plenty of validated functional linkages among protein pairs and sub-networks, which could be viewed as a novel resource to investigate the potential mechanisms of PPIs in *Synechocystis* sp. PCC6803. This systems biology approach provides the first step to explore functional linkage of cellular network, and is expected to extent the coverage and accuracy of the PPIs accompanying with more experimental data in the future.

## Materials and Methods

### Data preparation and features calculation

In gene expression, we first analyzed 20 microarrays datasets (GSE10708, GSE11970, GSE14410, GSE16162, GSE1695, GSE21133, GSE24882, GSE27406, GSE3682, GSE3703, GSE3715, GSE3716, GSE3717, GSE3755, GSE4019, GSE4604, GSE4606, GSE4613, GSE5391 and GSE9577) which were collected from GEO. Pearson correlation co-efficiencies were computed for each dataset to find out co-expressed gene pairs by cor function in R. We then maintained only three datasets (GSE4613, GSE37482 and GSE1695) where co-expression was positively correlated with interacting proteins. The three datasets were time course response to dehydration/desiccation and UV irradiation (GSE4613), response to inorganic carbon limitation (GSE1695) and transcriptomic response of *Synechocystis* 6803 encapsulated in silica gel (GSE37482).

GO annotation were collected from Gene Ontology Consortium^55^ to compute smallest shared biological process (SSBP) for each protein pair, assuming that protein pairs having the same GO term annotation should be more likely to interact with each other.

The genome context methods (gene fusion, gene neighborhood, phylogenetic profile and gene cluster) were analyzed by InPrePPI^28^. Domain data were obtained from DOMINE database (Database of Protein Domain Interactions, http://domine.utdallas.edu/cgi-bin/Domine) and Pfam database (http://pfam.sanger.ac.uk/).

Orthologs of 23 organisms were derived from InParanoid database (http://inparanoid.sbc.su.se/cgi-bin/index.cgi). For two species, *E. coli* and *S. cerevisae* with no data in InParanoid database, orthologs were obtained with reciprocal BLASTP between proteins with E-value < 1e-10. PPIs data of these species were downloaded from Database of Interacting Proteins (DIP, http://dip.doe-mbi.ucla.edu) and STRING (http://string-db.org/).

### Data integration method: Naïve Bayes approach

This approach, depending on the prior odds of protein-protein pairs, computed the likelihood ratio of each individual approach (or non-redundancy) and integrated them into a global likelihood. According to basic Bayes rules, we obtained eventual scores which represent the probability of each protein pair.

The prior odds were defined as equation (1). Here, *P*(*pos*) was the probability of a pair of proteins sharing an interaction and *P*(*neg*) was the probability of finding a non-interacting protein pairs.


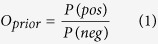


The likelihood ratio of individual approach was defined as equation (2), where *f*_i_ was a pair of proteins and *n* was the number of all possible protein pairs in each feature. Pr( *f*_1_…*f*_n_|*GSP*) was the probability based on the condition of GSP and so was it with Pr( *f*_1_…*f*_n_|*GSN*). When each element in the model was considered as independent (or non-redundancy), the global likelihood ratio could be calculated easily as the product of several individual likelihood ratio as equation (3).


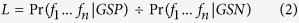



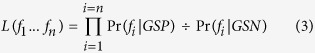


According to Bayes rule, the posterior odds could be computed according to equation (4) and equation (5).


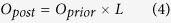



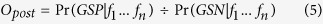


Equation (5) could be recognized as ultimate result of predicted protein-protein interaction. It meant, based on known condition of pairs of proteins, the probability belonging to GSP versus the probability belonging to GSN. The higher score they had, the more probability they might interact. We defined high confident PPIs as posterior odds larger than 5, which was stricter than previously defined posterior odds cutoff^17^.

### Yeast two-hybrid analysis

Several pairs of protein interactions were validated by yeast two-hybrid experiments. The encoding sequences of these proteins were amplified using primers listed in (Supplementary Table S4). The PCR products were digested with *Eco*RI/*Xho*I and unidirectionally inserted into pJG45 and pB42AD plasmids to construct baits and preys, respectively. Combinations of bait, prey and the reporter vector pSH18-34 were co-transformed into yeast strain EGY48 according to previously described procedures. The selection of transformants and the analysis of the galactosidase were performed as the former described procedure^56^.

### Isolation of crude thylakoid membranes

The cell cultures (800 mL) were harvested at the logarithmic phase (*A*_730_ = 0.6–0.8) and washed twice by suspending in 50 mL of fresh BG-11 medium, and the thylakoid membranes were isolated according to Gombos *et al.*^57^ with some modifications as follows. The cells suspended in 5 mL of disruption buffer (10 mM HEPES-NaOH, 5 mM sodium phosphate, pH7.5, 10 mM MgCl_2_, 10 mM NaCl, and 25% glycerol (v/v)) were supplemented by zirconia/silica beads and broken by vortexing 15 times at the highest speed for 20 s at 4 °C with 5 min cooling on ice between the runs. The crude extract was centrifuged at 5,000 × g for 5 min to remove the glass beads and unbroken cells. By further centrifugation at 20,000 × g for 30 min, we obtained crude thylakoid membranes from the precipitation.

### Electrophoresis, immunoblotting and chlorophyll fluorescence

SDS-PAGE of *Synechocystis* sp. strain PCC 6803 crude thylakoid membranes was carried out on 12% polyacrylamide gel with 6 M urea as described earlier^58^.

For immunoblotting, the proteins were electrotransferred to a polyvinylidene difluoride (PVDF) membrane (Immobilon-P; Millipore, Bedford, MA) and detected by protein-specific antibodies using an ECL assay kit (Amersham Pharmacia, NJ) according to the manufacturer’s protocol. The NDH-1 complexes were detected using the antibodies against NdhA and NdhH, respectively, which were previously raised in our laboratory^59^. Antibody against ATPβ was purchased from Agrisera Co. (Cännäs, Sweden).

The transient increase in chlorophyll fluorescence after actinic light turned off was monitored as described^59^.

### Construction of Δ*slr0815* mutant

Δ*slr0815* mutant was constructed as follows. The upstream and downstream regions of an unknown encoding gene *slr0815* were amplified by PCR creating appropriate restriction sites. A DNA fragment encoding a kanamycin resistance (Kam^R^) cassette was also amplified by PCR creating XbaI sites using appropriate PCR primers, *slr0815*-C and *slr0815*-D (Supplemental Table S4). These three PCR products were ligated into the MCS of pUC19 (Fig. 4a) and was used to transform the WT cells of *Synechocystis* sp. strain PCC 6803 to generate the Δ*slr0815* mutant. The transformants were spread on agar plates containing BG-11 medium and kanamycin (10 μg mL^−1^) buffered at pH 8.0, and the plates were incubated in 2% (v/v) CO_2_ in air under illumination by fluorescent lamps at 40 μmol photons m^–2^s^–1^. The mutated *slr0815* in the transformants was segregated to homogeneity (by successive streak purification) as determined by PCR amplification analysis (Fig. 4b).

### Function annotations with GO terms

GO terms were used to infer the annotations of proteins with unknown function. We selected the 5th upper GO terms as the level for proper functional annotation. Simulated annealing algorithm was applied to detect proteins with unknown function based on association rules with the whole network^26^. With the simulated annealing algorithm, each protein in the network would be assigned several GO terms with simulated frequency. Partial assignments were caused by randomly selected processes rather than true functional annotation. To minimize the false assignments, 10% of total results were randomly selected to compute a background noise and simulate the process with 100 times repeat. At last, GO terms, whose frequencies were larger than the background noise, were selected and viewed as true function annotation to proteins with unknown function.

### Pathway and GO enrichment analysis

Pathway data of *Synechocystis* sp. strain PCC 6803 was downloaded from KEGG (http://www.genome.jp/kegg/). With the annotation of gene and its participated pathway, using the hypergeometric distribution method, we compared the pathway information with genes in each sub-network and identified statistic significant function of each sub-network. One protein complex was selected to be enriched by one certain pathway if any pathway mapped to the complex with adjusted p-value <0.05. The statistical analysis was applied with R. The whole process of GO enrichment analysis was executed by the online tools AmiGO.

## Additional Information

**How to cite this article**: Lv, Q. *et al.* Genome-wide protein-protein interactions and protein function exploration in cyanobacteria. *Sci. Rep.* **5**, 15519; doi: 10.1038/srep15519 (2015).

## Supplementary Material

Supplementary Information
