# Supplementary material for: Genome-wide protein-protein interactions and protein function exploration in cyanobacteria
Source: Sci Rep. 2015 Oct 22;5:15519. doi: 10.1038/srep15519 (PMC4614683; doi:10.1038/srep15519)
Supplement: Supplementary Information [file srep15519-s1.pdf]

Genome-wide protein-protein interactions and protein function exploration in cyanobacteria

Qi Lv†, Weimin Ma†, Hui Liu, Jiang Li, Huan Wang, Fang Lu, Chen Zhao & Tieliu Shi*

†These authors contributed equally to this work.

*Corresponding author, E-mail, tieliushi01@gmail.com

Affiliations

Center for Bioinformatics and Computational Biology, and the Institute of Biomedical Sciences, School of Life Science, East China Normal University, 500 Dongchuan Road, Shanghai, 200241, China

Qi Lv, Hui Liu, Jiang Li, Huan Wang, Chen Zhao & Tieliu Shi

College of Life and Environment Sciences, Shanghai Normal University, 100 Guilin Road, Shanghai, 200234, China

Weimin Ma & Fang Lu

The institute of plant physiology and ecology, Shanghai Institutes for Biological Sciences, Chinese Acedamy of Sciences, 300 Fenglin Road, Shanghai 200032, China

Tieliu Shi

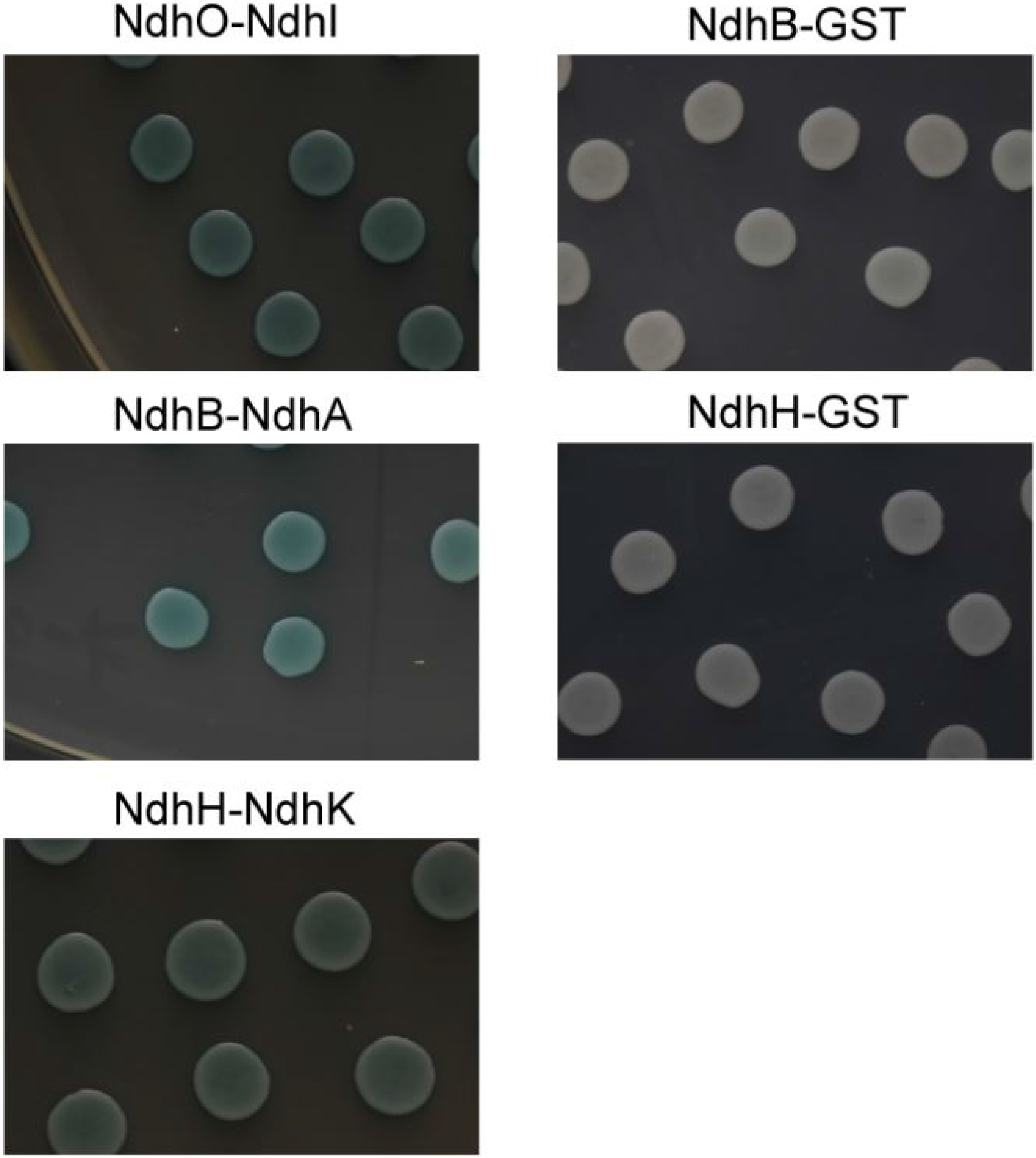

Supplementary Figure S1 Full views of yeast two-hybrid results in Figure 2.

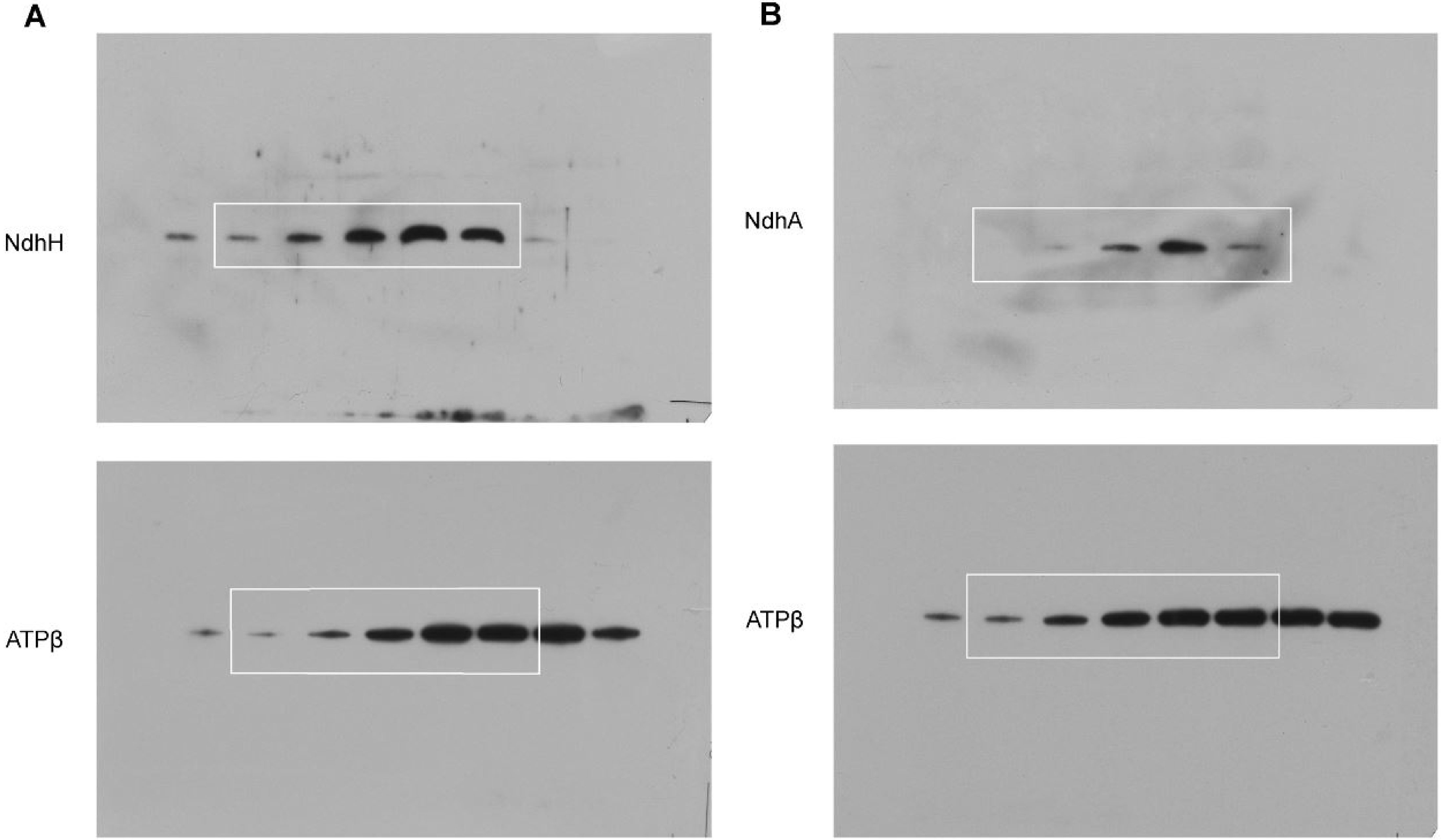

Supplementary Figure S2 Full views of western blots. Rectangles delimit cropped areas used in the indicated Figure 3.

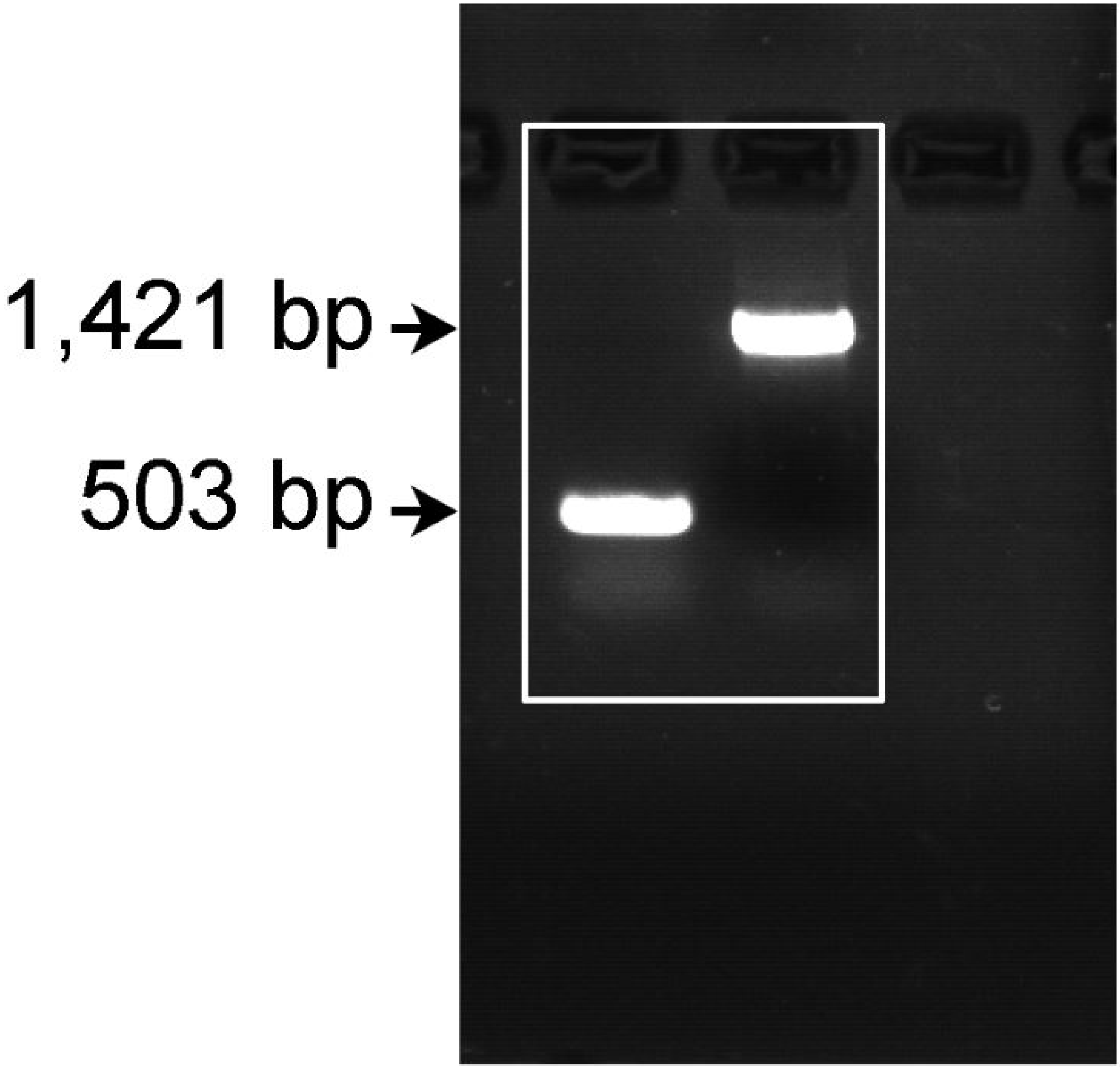

Supplementary Figure S3 Full views of DNA electrophoresis gels. Rectangles delimit cropped areas used in the indicated Figure 4.

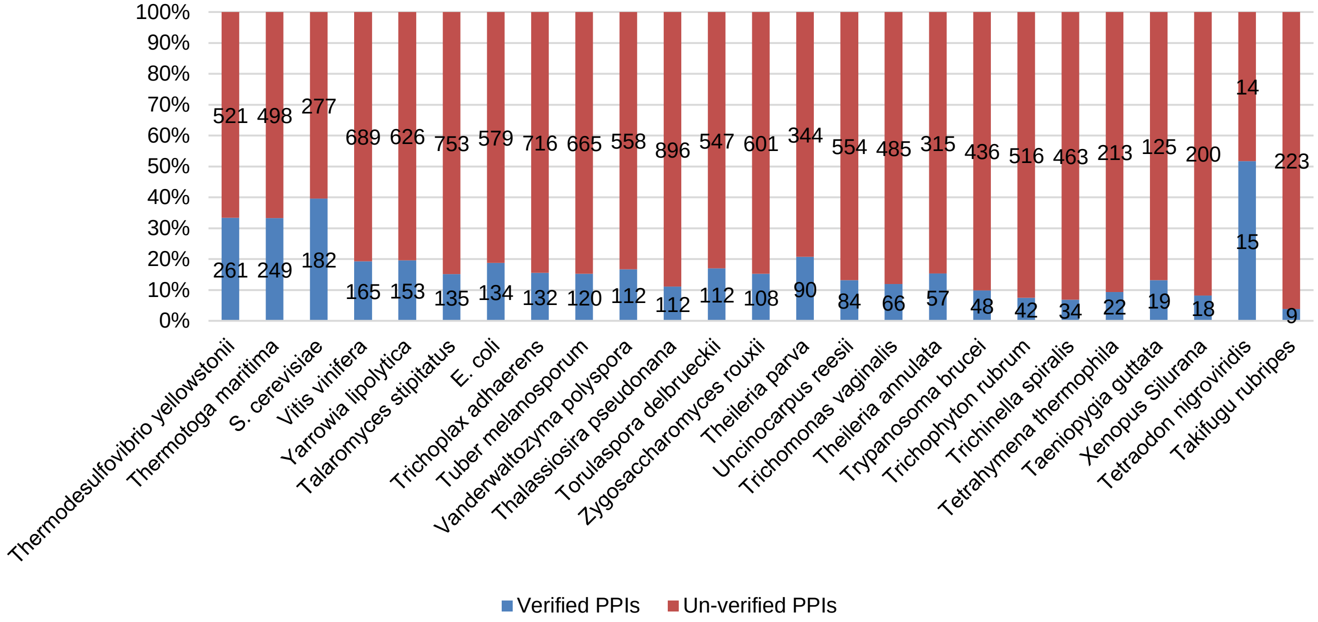

Supplementary Figure S4 Interolog verification of interactions based on conserved proteins among 25 organisms.

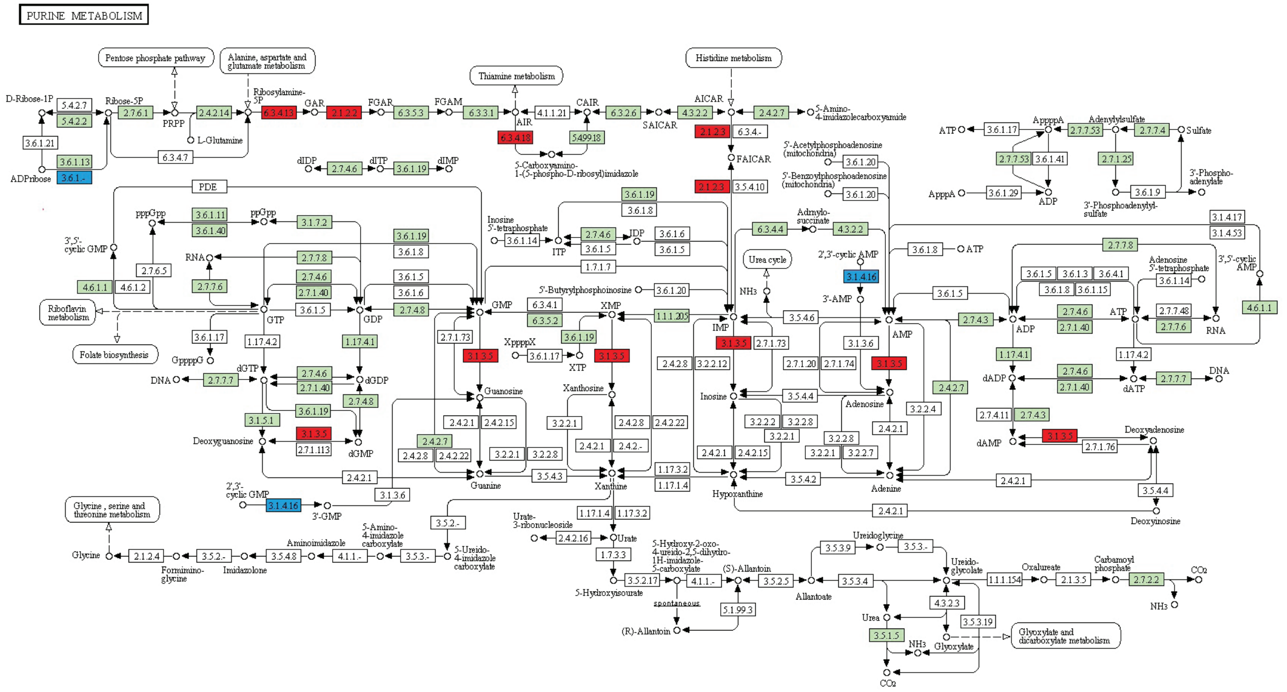

Supplemental Figure 5 In purine metabolism two enzymes were assigned as the potential function of function-unknown proteins in sub-network 14 by mapping the same GO annotation of sub-network in E.coli. (Red nodes represent conserved function in E.coli and Synechocystis sp PCC 6803, Blue nodes represent novel nodes in Synechocystis sp PCC 6803, based on conserved function sub-network analysis.)

Supplemental Table S1. PPIs as gold standard positive dataset (GSP).

P1	P2	P1	P2	P1	P2	P1	P2	P1	P2
sll0080	slr1619	sll1219	slr0084	sll1751	sll1858	sll0380	sll0508	sll0041	sll1383
sll0534	slr0542	sll1942	slr1277	sll1446	sll1546	slr1878	ssr2009	sll1614	ssr2142
sll0361	slr0665	sll0861	slr0994	sll1322	slr0722	slr0994	slr1842	slr0653	slr0668
slr0199	slr0585	slr0794	slr1971	slr1032	slr7023	sll0920	slr1203	sll0024	slr1495
sll1121	slr0836	sll1424	slr0536	sll1317	slr1710	slr0594	slr1215	sll1371	slr1530
slr0550	slr1544	sll0647	slr0890	sll1334	slr0553	sll0355	sll1143	sll0266	slr2098
slr0280	slr1025	sll0865	slr0373	sll0507	slr0473	sll0446	sll1193	sll0833	sll1601
sll0647	sll1274	slr0733	slr1974	sll1775	slr1403	sll0195	ssr1258	slr0565	sml0003
slr0467	ssl0242	sll0336	slr0480	slr1212	slr2087	sll1440	sll2005	sll1143	slr0922
slr1074	slr1618	sll1275	sll1623	slr0076	slr1614	sll0812	slr0491	sll0142	sll1214
sll0178	ssr2439	sll1621	slr0280	sll1354	slr1926	sll1165	slr0060	sll0702	sll1265
slr0506	slr1644	sll0732	slr0337	slr0820	slr1568	sll1739	ssr3154	sll0780	sll0781
sll0656	slr0528	sll0170	slr2016	sll0909	sll0910	slr1106	slr1918	sll1354	slr1565
sll1614	slr0404	sll0902	sll1995	sll0606	slr1230	sll1638	slr1204	sll1525	sll1987
sll1343	slr1239	slr0358	slr1855	slr1431	ssr1256	slr1591	slr1854	slr1706	slr2038
sll1586	sll1679	slr1044	slr1215	sll0779	sll1814	slr0738	ssl2542	slr1222	slr1656
sll1920	ssr2142	slr0514	slr1767	sll0783	slr1542	sll1568	slr1417	sll1079	slr1592
sll0754	slr1471	sll0002	smr0010	sll1868	slr1533	sll0621	slr0798	sll0418	sll0676
sll0702	slr2018	sll1757	slr0506	sll1625	slr1847	sll0519	sll1614	sll0237	ssl2542
sll0272	slr0963	sll0414	sll1632	sll0641	slr1673	slr1811	ssr0854	sll0524	sll1584
slr0615	slr1865	sll0263	slr0981	slr0294	slr0392	slr0553	ssl0750	sll0982	slr0377
sll1614	slr1676	sll0445	sll1064	slr1192	slr7054	sll0252	sll0272	sll0864	slr0821
slr0789	slr1243	slr1044	slr1726	sll0147	ssr2016	slr0006	slr0204	slr1742	ssl2542
sll0149	sll0767	slr0359	slr1307	sll0823	slr0023	sll0631	sll2007	slr1505	slr2037
slr0008	slr0067	sll1654	sll1933	sll0310	sll1356	sll0423	slr2026	sll1119	slr1196
sll0176	ssl0312	sll1737	sll2003	sll1653	slr0597	slr1215	slr1728	slr0008	slr1278
sll0414	sll1563	sll0488	ssr1528	sll1630	sll1679	sll1317	slr0593	sll1578	slr0697
sll1040	smr0004	sll0267	sll1925	sll0261	sll0413	slr0114	ssr3451	slr0936	slr1262
sll1418	slr0454	slr0467	ssl1045	sll0262	slr0798	sll0398	sll0518	sll1864	slr1413
slr0653	slr1055	sll0445	slr1567	sll0912	ssr3532	slr1604	slr1774	sll0740	slr1913
sll0021	slr1774	slr0675	slr1751	slr0543	slr1194	sll0238	slr0798	slr0479	slr0804
sll1679	slr1699	sll1866	sll1933	slr2018	slr2038	sll0865	sll1961	sll1841	ssr1256
sll0149	sll0586	sll0933	slr1647	slr0653	slr0750	sll1299	slr1591	sll0252	slr1204
sll0194	sll1614	sll1934	slr0452	sll1621	slr1938	sll0140	slr0108	sll0412	sll1237
slr0642	slr1403	slr1204	slr1827	slr0659	ssr2049	sll0296	ssr3532	sll1362	slr0935
slr0733	ssr1258	sll1482	sll1864	ssl3389	ssr3451	sll0623	sll1667	sll0778	slr2034
sll0558	slr1731	sll1586	slr0980	ssl0738	ssl0739	sll1530	slr1511	slr1928	ssl2920
sll0872	slr0695	sll1942	ssl0294	slr0603	ssr2551	sll0865	ssl0242	slr1773	slr1795
sll0242	slr0343	sll0616	sll1360	slr0876	slr0889	sll0242	sll1724	slr1699	slr2089
sll0606	sll0915	sll1427	slr0737	sll1426	slr0871	slr0467	slr0729	sll0427	sll0779
sll0141	slr1773	sll0577	sll1166	slr0214	ssl3291	slr0861	ssl0750	slr0091	slr0880
sll1323	sll1324	sll0322	sll1864	sll1513	slr1660	sll7055	slr0990	sll0281	slr0263
sll1913	slr0280	sll1802	sll1817	slr0143	ssl0452	sll0080	slr2092	slr0844	slr1128
sll0812	slr1305	sll0783	sll1360	sll1995	slr0960	sll0185	sll0854	sll1946	ssr1698
slr1152	slr1505	slr1053	ssl3389	sll0149	ssl3432	sll1675	slr1285	sll1098	sll1240
sll1733	sll1967	sll1715	slr1113	sll0865	sll1659	sll1965	slr0653	sll1858	slr1644
slr0514	slr2111	sll1241	ssr2049	sll0418	slr1127	slr1047	slr1594	slr0770	ssr2754
sll1946	slr1572	slr0111	slr0156	sll1098	slr1759	sll1595	slr1840	sll1733	sll1950
slr0606	slr2102	sll0861	slr1506	slr0364	ssl0750	sll1418	slr0533	sll1400	ssl2733

sll0645	slr0589	sll0837	slr2144	sll0905	sll1031	sll1424	slr0653	sll1558	sll1763
slr0633	slr1095	slr0751	slr1045	slr0408	ssl0750	slr0468	slr0545	sll0779	sll1656
sll0656	slr1032	sll0547	slr1185	slr0797	slr1978	sll1572	slr1726	slr1857	ssl0483
slr0077	slr0214	sll0180	slr0076	sll1231	slr1573	slr1239	ssl0353	sll1130	slr0937
sll1736	slr0408	sll0427	slr1729	sll0405	sll0406	sll0149	sll1488	sll0602	slr1959
slr1969	ssr2843	sll1096	slr0366	sll0016	slr1204	slr0399	slr0487	slr1787	ssr3159
slr1667	slr1668	sll1455	slr0280	sll1981	ssl1577	sll1428	sll1482	slr0473	ssr1375
sll0661	slr2077	slr0801	slr1719	sll1201	slr1895	sll1967	slr1788	sll0447	slr1800
sll0865	ssl2999	sll0099	sll1072	sll1934	slr0534	slr0557	slr1966	sll1174	sll1371
slr1648	ssl0483	sll0641	ssr2595	slr2001	ssr1256	slr0208	slr1992	sll0789	ssr3571
sll0488	slr1084	slr1098	slr1565	sll1193	slr1274	sll0641	sll1129	slr1821	slr1957
sll1614	slr1052	sll1614	slr1518	sll1084	sll1902	slr0594	smr0010	sll0865	slr1974
sll1772	slr0529	sll1732	slr0798	sll1356	ssl0832	slr0527	slr1958	sll1371	slr1110
sll0336	sll1324	sll0898	sll1143	sll0262	slr0923	slr0305	slr0798	sll1687	slr0653
sll1614	slr0305	sll0623	slr0191	sll0996	slr1364	sll2012	slr0653	sll1270	ssl2615
slr1278	ssl0452	slr0214	slr0871	slr0919	slr1235	sll1446	slr1090	slr1644	slr1710
slr1077	ssr3154	sll0102	slr1471	sll1564	ssr1391	sll0981	slr0257	sll1040	smr0010
sll0927	slr0280	sll0996	slr1613	sll1939	slr1674	sll1611	slr0923	sll0288	ssl1255
sll1513	slr0640	sll1427	slr0370	sll1441	slr2077	slr1330	slr1743	sll0776	ssr1391
slr0143	slr1871	sll1621	slr1744	sll0931	slr1693	slr0467	slr1694	sll0068	slr1530
sll0564	sll1699	sll0418	ssl2996	sll1466	sll1730	slr0482	slr0921	slr1028	ssr1552
sll0198	slr1210	sll1354	sll1642	sll0010	sll1398	slr0394	slr1289	sll1217	slr1353
sll1353	slr1084	sll1743	slr0969	slr0733	slr1900	sll0182	sll0505	sll1614	slr0533
sll1623	slr0680	slr1910	ssl3692	sll1800	sll1802	sll0848	sll1135	sll1228	sll1542
sll0611	sll2012	slr0525	ssl0787	slr0020	slr2122	sll1472	slr0875	sll0671	sll0897
sll0314	slr0990	sll0096	sll1629	slr0063	ssr2009	sll1614	sll1733	sll0404	sll0864
sll0669	sll1454	sll1434	slr1992	sll1526	slr1762	slr0551	slr1778	sll0446	slr1198
sll1454	sll1512	slr1330	slr1498	sll1578	slr1300	sll1318	slr0733	sll0445	slr1429
sll0350	slr0250	slr0213	slr0536	slr0386	slr1319	sll0441	slr0955	sll0269	slr0602
sll0103	sll0529	slr0733	ssr2962	sll0861	slr2034	slr1866	smr0010	slr1676	ssl0787
slr0108	slr0942	sll1003	sll1908	slr0537	slr1028	slr0431	slr1032	sll0436	sll0647
slr1044	slr1618	slr1567	slr1809	sll1418	slr2077	sll0310	sll0776	sll0066	slr2087
slr0789	slr1971	sll0180	sll1500	sll0068	slr1614	sll0947	sll1773	slr0774	ssr3451
slr0749	slr1956	sll1647	slr0476	sll0587	ssr2787	slr1037	slr1047	slr0288	ssr1238
slr0969	ssl1577	sll1463	slr1045	sll0156	slr1699	ssr2754	ssr2755	sll0558	sll1527
sll0864	sll2005	sll0178	slr1301	sll0776	slr0171	sll1104	slr1420	sll0776	slr1926
sll0661	slr0861	sll0053	sll0184	slr0073	slr0354	sll0198	slr0362	slr1697	ssr2781
slr0066	slr0435	slr1047	slr1397	sll0242	sll0616	slr1110	slr1743	sll0626	sll1129
slr0404	slr1596	slr0398	slr1068	sll2007	slr1762	sll0933	slr0687	sll0325	sll1021
sll0169	slr1644	slr0369	slr0642	slr0701	slr0773	sll0641	sll1761	sll1099	slr0687
sll0103	sll1614	slr0209	slr1476	sll0556	sll0735	sll0577	sll0865	sll0865	ssr1258
sll0547	sll1634	sll1353	ssl1377	sll1031	sll1500	sll0428	slr0638	sll0992	sll1599
sll0524	slr0204	sll1086	sll1488	sll0861	slr1519	sll0790	slr0975	sll0381	sll0445
sll0309	ssr2843	sll1455	slr1047	sll1434	slr2038	sll0236	sll1902	slr1575	slr1776
slr0793	slr1638	sll1323	slr0679	sll1267	slr0335	sll1783	slr0666	slr0249	slr0373
sll0788	slr1591	sll1076	sll1418	slr0658	slr2016	sll0169	ssr2142	slr0980	slr1041
sll0588	sll1721	sll0252	sll0624	sll1096	slr0742	sll1799	sll1971	sll1398	slr0143
sll0415	sll0424	slr0359	slr0787	sll1722	slr0609	sll0944	slr1793	sll0149	sll1096
slr0366	ssl3177	slr1147	ssl2502	sll1085	slr0770	sll2001	slr1636	slr0335	slr0844
slr0852	slr1705	sll1995	slr0491	sll1353	sll1737	slr0250	slr0872	sll0822	sll1511
sll0901	slr0550	sll0766	slr0361	slr0992	slr2098	sll0252	sll1608	sll0424	sll0703

sll1272	sll1530	sll0360	sll0804	slr0642	slr1471	sll0068	slr1992	slr0513	slr1511
slr0510	ssr2615	slr1367	slr1603	sll0669	slr1064	sll0669	slr2092	sll1868	ssr3154
sll0535	sll7075	sll1003	slr1974	slr0698	ssl1923	sll0804	slr0211	sll1281	slr1019
sll0322	slr0232	sll1562	slr0467	sll0931	sll1566	slr0157	slr2018	sll0178	slr1505
sll1902	slr1636	sll0148	slr0152	sll1037	slr0467	slr0528	slr1809	sll1424	slr1959
sll0819	slr1866	sll0763	slr1028	sll0280	slr0653	sll1079	sll1775	sll0191	sll0222
sll1304	sll1688	sll1858	ssl2996	slr0959	slr1971	slr0408	slr1431	slr0476	slr2130
sll0702	slr0191	slr0554	ssl2733	sll1614	slr1603	slr1732	slr1788	slr0695	slr1590
sll0931	slr1699	sll1699	slr0550	sll1802	slr0366	slr0794	slr2087	sll0021	slr1568
sll0175	slr0880	sll0920	slr0579	sll1814	slr0765	sll1173	slr1847	slr0905	ssl1045
sll0776	sll1653	sll0242	sll0293	sll1564	sll1821	sll0638	sll1633	sll0819	slr1710
slr0733	ssl1255	slr1139	slr1238	slr0163	slr0941	sll0584	sll0780	slr1343	slr1604
sll0178	sll0532	slr1788	ssr1552	sll0021	slr1591	sll1544	sll1902	slr0399	slr1305
sll0414	slr0861	sll1751	ssr1562	sll1241	slr0201	sll1372	slr1978	slr0066	slr1626
sll1614	slr1595	sll0296	sll1761	sll1500	slr0423	sll0041	slr1800	slr0115	slr1338
sll0821	slr1593	sll0785	slr0545	slr0978	slr2099	slr0008	slr0675	slr1081	slr2018
sll0737	slr1391	sll1229	slr0773	slr0423	slr0746	slr1727	slr1729	sll0996	sll1679
sll0149	slr0679	slr0162	ssl0452	slr1636	slr2128	sll0587	sll1293	sll0283	slr1767
slr0294	slr1913	sll0641	sll0756	sll0521	slr1612	sll1209	slr0740	sll1959	slr1229
slr0495	slr1104	sll0068	ssr1256	sll0095	sll1542	slr0793	slr2144	slr1603	slr1978
sll0445	sll1502	sll0827	sll0862	sll1629	slr1204	sll1317	sll1920	sll0142	sll1446
sll1875	slr1977	sll1737	slr0369	sll0754	slr0384	slr1444	slr2025	slr0774	slr2087
slr1199	slr1474	sll0242	slr1773	sll0398	sll0810	slr0417	slr0491	sll0199	slr0602
sll0041	slr2037	sll1621	sll1625	sll0022	sll0023	sll0039	sll0985	sll0055	sll0555
sll2012	slr1702	slr1215	slr2036	sll1218	slr0788	slr0076	slr1325	sll1632	slr0208
slr0688	slr1347	sll0508	sll1314	sll0819	slr0765	slr1235	slr1423	slr0232	slr1644
slr0023	slr0963	slr1164	ssr2803	sll1709	slr1686	sll0488	slr1851	sll0418	sll1214
sll5086	ssr1399	sll0743	sll1969	sll1841	slr0550	slr1791	slr1842	sll1908	slr0263
sll0175	sll0539	slr1612	ssl0109	slr1028	ssl0750	sll1294	sll1956	sll0698	ssl3451
slr0249	slr1122	sll0053	slr0309	sll0325	sll0471	sll0226	slr1239	sll8042	slr0172
sll0751	slr0467	sll0499	ssr1375	slr0351	slr1519	sll0252	slr1626	sll0295	sll1366
sll1647	slr0885	slr0399	slr0984	sll0398	slr1437	sll2005	slr1974	slr0534	ssr3154
sll0401	sll1950	sll0865	slr0376	sll0418	sll1956	sll0033	sll1693	slr1070	slr2098
sll0144	sll0910	sll0149	sll0708	sll0350	slr1423	sll0505	sll0914	slr0207	slr1636
sll0142	slr1644	sll1967	slr1278	slr0921	slr1048	sll0144	slr1690	sll1841	ssr0536
sll1689	slr1055	sll1920	slr0473	sll0242	sll0828	sll0428	slr0442	slr0451	slr0889
slr0171	slr1044	slr2131	ssl2598	sll1934	slr1031	sll1003	slr2005	sll0041	sll0043
sll0375	slr2046	sll1427	slr1259	sll1418	slr0794	sll0532	sll1003	sll1214	slr1300
sll0914	slr2131	slr1565	ssr3188	slr1593	slr2047	sll0381	sll1184	sll0861	slr1658
sll0754	slr1647	sll0920	slr1636	slr1198	slr2130	sll0910	sll8034	sll0638	slr1077
sll0216	sll1886	sll1575	ssr1114	slr0774	ssl2598	sll0804	sll1858	sll2012	slr0889
slr1651	ssl1255	sll0140	slr0887	sll0624	slr1495	slr1926	slr2073	slr0354	slr0887
sll1143	sml0006	sll1135	slr0839	slr1209	slr1210	sll0590	slr0642	sll0210	sll0558
sll0135	slr1964	sll0865	ssr2831	sll1614	slr1291	slr0364	slr2084	sll1025	sll1273
sll1317	slr2131	slr0208	slr1844	sll0026	slr1471	sll1967	slr0969	slr0994	slr2011
sll0688	slr0073	sll0703	slr0579	sll0350	slr0992	sll0418	slr0243	slr1492	slr1493
slr0091	slr0585	slr1090	slr1636	sll1621	slr0697	sll0923	sll1653	sll0865	slr1902
sll0923	sll1472	sll0821	slr0839	sll1814	slr1471	sll0672	slr1950	sll1770	slr0211
sll0933	slr0704	sll1272	ssr1720	slr0394	slr0943	sll1003	slr0392	sll1023	ssr3159
sll1180	smr0001	sll0284	slr0733	sll0985	slr1613	slr0602	slr1331	slr0213	slr0514
sll0071	slr0900	sll0985	slr2131	slr1353	ssr3188	slr0229	slr1659	sll0861	slr1493

sll0647	slr1192	sll1687	slr0941	sll1293	slr0280	sll0585	slr1926	sll1995	slr2100
sll0672	slr0774	slr0467	ssr3122	sll0616	sll2005	sll1908	slr0955	sll1912	slr0467
slr0341	slr0722	slr0192	ssr0693	slr0747	slr1608	sll1054	slr0733	sll1222	slr0442
sll1814	slr0454	sll0149	slr1306	sll0661	sll1553	sll0043	sll1691	sll0932	sll1614
sll1404	slr1198	sll0865	ssr2194	sll0606	sll0641	slr0798	slr1518	sll1165	sll1934
slr0665	ssr1698	sll0645	slr0476	slr0993	slr1774	sll1614	slr1747	slr1044	ssr2142
sll1500	sll1608	sll0544	sll1366	sll1491	slr0585	sll0459	slr0889	sll1925	slr0774
sll0639	slr0484	sll0499	slr1926	slr0871	ssr0536	sll0445	sll0601	sll0066	sll0071
sll0586	sll1251	sll0703	sll2007	sll0280	sll1508	sll1092	ssl2138	sll0499	ssr2553
sll1921	slr1773	sll1129	slr1330	sll0672	sll1002	sll1562	slr0877	sll1995	slr0580
sll0163	slr1362	sll0696	slr0659	sll0427	sll1614	slr1398	slr1878	sll0505	sll1656
sll0019	slr0152	sll1043	slr1204	slr1152	slr1803	sll2012	slr1545	sll0933	slr0638
sll0180	slr1881	sll0902	sll1193	sll0169	slr1629	slr0919	slr1076	sll0421	slr1330
sll0149	sll1527	sll1325	ssl2999	sll0823	slr0739	slr0431	ssl3044	sll0640	sll0862
sll0898	slr0704	sll0558	sll1370	slr1047	slr1109	sll1353	slr1812	slr0525	slr0810
sll1951	ssl1377	sll1231	ssr2201	slr1818	ssr2803	sll1253	ssr2201	sll1293	ssl2807
sll1383	sll1760	sll1669	slr0953	sll0851	slr1655	slr1423	slr1811	sll0418	slr1917
sll0182	slr0589	slr0615	slr0942	sll1132	sll1738	sll1247	slr1066	sll1143	slr1100
sll0057	slr1738	sll1599	slr1573	sll0865	ssl1762	sll1689	slr1702	sll1614	ssr1386
sll1178	sll2006	slr0536	slr0955	sll0521	slr0798	slr0423	slr1617	sll0864	slr1879
slr0451	slr1847	sll1987	slr0147	sll0270	slr0169	sll0149	sml0011	sll0223	slr1403
slr0630	slr0942	slr0280	slr1614	sll1746	sll7065	slr1417	slr1708	sll0208	slr0171
sll1029	slr0729	sll0596	slr1848	sll0779	slr0602	sll0815	slr0467	slr0473	slr0806
sll1343	slr0994	slr1222	ssl2996	sll0173	sll1253	sll0520	slr0452	slr0992	slr1755
sll0194	sll1411	sll0606	sll1945	sll0418	slr0975	slr1471	ssl0331	sll0208	sll1632
slr0157	slr0309	sll1291	ssr2962	sll1253	slr0739	slr0740	slr1204	sll1614	sll1814
slr1383	ssl1377	sll0861	slr0406	sll0319	slr0665	sll1174	slr1668	sll0269	slr1044
sll0584	slr2131	sll1981	slr0348	sll0041	sll1512	slr0008	slr0467	sll1305	slr0152
slr0378	slr1592	sll0027	sll1614	sll0832	sll1608	sll1500	slr0280	sll1003	slr2092
slr2084	ssl0832	sll0085	sll0473	sll0198	ssl3573	sll0723	sll1005	slr0978	slr1596
slr1122	slr2098	sll0672	slr2087	slr1098	slr1353	sll0985	slr1842	slr0662	slr0974
sll0865	ssr1375	sll1427	slr1880	sll0997	slr0066	sll0377	slr0611	sll0070	sll0424
sll0661	sll1563	sll0550	slr1385	sll1528	ssr0692	sll1001	sll1981	sll0502	sll0982
sll1250	slr0442	sll0318	slr0496	sll0228	slr1674	sll0445	sll1835	sll0676	slr1331
sll0493	sll1294	slr1225	smr0001	slr0589	slr1776	sll1653	sll1673	sll0602	ssl3382
sll0830	slr1198	sll0410	slr0383	sll0743	slr1626	slr1204	slr2131	sll0933	sll1601
sll0301	sll1858	slr0118	slr0479	sll1272	sll1949	slr0744	slr0872	sll1800	slr0445
sll1219	slr1756	sll1542	slr1494	sll1214	slr1656	sll1166	slr0798	slr0108	slr1330
slr0257	slr1641	sll0027	slr1471	sll0237	slr1431	sll1219	slr1161	sll7062	slr0169
sll0502	ssr3154	sll1263	sll1772	sll0854	slr1348	sll1912	slr0363	sll0980	slr1686
slr1300	ssr2755	sll0521	slr0114	slr1444	slr1636	sll0507	sll0533	sll0408	sll0783
sll1524	slr0008	sll1108	slr0302	sll0053	sll0185	sll0149	sll1097	sll0236	ssl0294
sll0909	ssr1736	sll1267	slr1140	sll1864	slr0798	sll0931	sll1841	sll1800	slr0020
sll0427	slr0774	sll0601	slr1204	sll1799	slr1338	slr1117	ssl0294	sll1739	slr2034
sll0872	slr1800	sll0048	slr0341	sll1995	slr1211	sll0053	slr1431	slr1767	ssl2733
sll0043	sll1170	sll0177	sll1886	sll0488	slr0147	sll0382	sll0861	sll0242	slr1906
sll0657	sll1513	sll1314	slr0165	sll0931	slr0679	sll0055	sll1513	sll1647	slr0407
sll1527	ssl2996	sll0811	sll0861	slr0359	slr1908	sll0606	slr1604	sll0243	sll0375
sll0370	sll0413	sll1383	ssl2542	sll1272	ssl1762	sll1418	slr0602	sll0819	slr0774
slr1393	slr2111	sll1787	slr1431	sll1721	sll1841	sll1434	slr0341	sll0149	sll1822
sll1675	slr1416	sll1317	slr0594	slr0585	ssl0294	sll1500	slr1959	sll0788	sll1902

sll1352	slr1436	sll1969	slr0702	sll0415	slr1643	slr1793	ssl3382	sll1841	slr0147
sll1905	slr1362	sll1969	slr1196	sll0096	slr1403	slr0343	slr0765	slr1207	slr1210
sll0488	slr0228	slr1901	slr2092	sll0103	slr0298	sll1498	slr0955	sll1800	slr1122
sll0456	sll1247	slr1603	slr1971	slr0317	slr0818	sll1950	slr2084	sll8020	slr2072
slr1272	slr1851	sll1800	slr0990	sll0222	slr2105	sll1291	slr1385	sll0982	slr1301
slr0798	slr2033	sll0178	sll1825	slr0299	slr1616	sll0865	slr0503	slr0868	slr1423
sll1920	slr0503	slr0876	slr1964	sll0782	ssr2551	slr1351	slr1565	slr1410	slr1840
sll1270	slr1046	sll0923	sll1671	slr0231	slr0467	slr1041	ssr2843	slr0818	slr0992
sll1323	slr0713	sll0590	slr1215	ssl0707	ssr2787	slr1243	slr2077	sll1578	slr0324
slr0599	slr0602	sll0282	slr0354	sll0754	sll1601	sll0382	slr1222	sll1584	slr0909
slr1986	ssl0242	slr0191	slr1774	sll1499	slr1272	sll1229	slr1747	sll1671	slr0572
sll0252	slr0845	slr0639	slr1471	sll1250	slr0924	sll0242	ssl0353	sll1586	slr1204
sll0656	sll1825	slr0854	slr1117	sll1035	slr1204	sll0584	slr0794	sll0687	sll0688
sll0933	sll1070	sll1343	slr1686	sll0702	sll1283	slr1471	slr1595	sll1687	slr1019
slr0306	slr1342	sll0735	sll1864	sll1270	slr1518	sll0513	sll1679	sll0743	sll1315
sll0242	sll1679	sll0149	sll1967	sll0754	slr0733	sll1737	slr0844	slr1288	slr1311
sll0485	sll1620	slr0798	slr2098	sll0558	sll1766	slr0798	ssr2142	slr1547	ssr2142
sll0534	slr1423	sll1614	slr0589	sll1027	ssr2843	slr0008	slr0440	sll0289	slr0937
sll0615	slr0765	slr0473	ssr2422	sll0732	slr1857	sll1426	slr0383	sll0149	slr0989
slr0280	slr0992	sll1383	sll1508	sll1291	ssl0109	sll1647	ssl0294	sll1611	slr1471
slr0602	slr2034	sll0488	sll1775	sll0375	slr0431	slr0467	ssl2781	sll1452	slr1865
sll0810	slr1139	slr1667	ssr0854	slr1778	ssr2975	slr0869	slr1800	slr1917	slr2131
sll1831	slr1966	sll1043	slr0942	sll0776	sll1318	sll0360	sll1614	sll0661	sll1555
sll0451	slr1708	sll0985	sll1905	sll0223	slr1729	sll1442	slr0366	sll0400	slr1204
slr1842	ssl2542	sll1541	ssl3712	sll0418	ssr2755	sll1484	slr1655	sll1053	slr0341
slr0797	slr0798	sll0544	slr0280	sll0191	slr1753	sll0656	slr2118	slr1248	slr1385
sll0336	sll0728	sll1546	slr0923	sll0149	sll1812	sll0616	sll1408	sll1696	ssl0294
sll0178	slr1653	sll0716	slr0994	sll2012	slr0207	sll0861	slr1369	slr0993	slr1472
sll0910	slr0304	sll0286	sll1003	sll0934	slr0060	sll1528	ssl0832	slr0023	slr0063
slr0602	ssr2781	sll1244	slr0473	slr1767	smr0014	sll0985	slr1983	sll1418	slr1443
sll1129	slr0354	sll0656	slr0719	slr0617	ssl0353	sll0022	slr1136	sll0854	slr2100
sll0779	slr1595	sll1775	slr0245	sll1736	sll1858	sll0301	slr0369	sll1360	slr0104
sll0915	slr0442	slr1508	ssl0832	sll1515	slr1756	slr0114	smr0004	slr1862	slr2098
sll1186	sll1879	sll1291	ssr2848	sll0554	sll0741	sll8049	slr0065	sll0980	slr0257
sll0156	slr1119	sll0728	sll8009	sll0325	slr0775	slr0366	slr2048	sll0727	sll1737
sll0208	sll1218	sll1714	sll1715	sll1165	ssr1256	slr0108	slr1343	sll0296	sll1250
sll0406	slr0467	sll0985	slr0719	sll0614	sll0865	sll1231	ssr0692	sll1324	slr0602
slr1204	slr1610	sll0763	slr0093	slr0697	slr1198	sll0502	slr0361	sll0508	slr1843
sll1579	slr1390	slr0937	slr2052	sll0455	sll1763	slr1204	slr2007	sll2005	slr1629
sll1884	slr1896	slr1046	slr1403	slr0226	slr1866	sll0377	ssr1256	sll0616	slr0704
sll0865	sll1749	slr0455	ssl3142	sll0322	slr0171	slr0080	slr1230	sll1908	slr1143
slr1216	slr1232	sll0913	sll2005	sll1222	sll1763	sll1925	slr0143	sll1489	slr1047
slr1638	ssl3076	sll0192	sll1405	sll0584	slr0594	slr0229	slr1034	sll0057	sll1434
slr0108	ssr0511	sll1736	slr0794	sll0295	slr0585	sll0413	sll1003	slr0423	slr1852
sll1737	slr1950	sll1031	slr1023	sll0068	ssr1399	slr1535	slr2098	slr0331	slr1729
sll0022	slr0191	sll1483	ssl8008	sll0149	sll1743	sll1864	sll1920	sll1527	sll1956
sll0057	sll1463	sll1874	ssl2598	sll0656	sll0985	slr1729	ssl2615	sll1577	slr0818
sll0851	slr0341	sll0283	slr1148	sll0055	sll1483	sll1528	slr1732	slr1062	slr1423
slr0399	slr1110	sll0892	slr1143	sll0446	sll0449	sll1167	ssl3441	sll2012	slr0880
slr0211	slr0220	sll0382	sll0915	sll0445	slr1391	sll1124	slr1329	sll1995	slr1423
slr1968	ssr0692	sll0413	slr0280	sll1902	slr1177	sll0596	sll1077	slr0459	slr1143

sll1222	sll1477
slr1661	slr9201
slr2025	slr2037
sll1253	ssl2084
sll0638	sll0827
slr0445	slr1055
sll0147	ssr1407
slr1908	slr2038
sll0931	slr2098
sll0286	sll1366
slr0774	slr1726
slr0287	slr0857
slr0925	slr1034
slr0545	slr2130
sll0445	slr1189
sll1110	sll1237
slr0439	ssl0750
sll0702	sll1699
sll0141	slr0649
sll1454	ssr3159
slr0871	slr1198
sll1774	slr1198
slr1293	slr1992
sll1513	slr0798
sll0283	slr1655
sll1653	sll2012
sll0359	slr1204
sll0230	ssl0750
sll1135	slr0483
sll0238	sll1614
sll1614	slr1215
slr1431	slr1799
sll1812	slr0722
sll1954	slr0143
sll1003	slr0546
slr0220	ssr3159
slr0208	slr0702
sll0413	slr0810
sll0149	sll0888
sll0148	ssr2755
sll0445	sll0446
sll1237	sll1865
sll0252	slr2122
sll1294	slr1636
sll0780	slr0787
sll0238	slr1205
sll1879	slr0734
slr1603	slr2057
sll1363	sll2012
sll1586	sll1967
sll1454	slr2084

sll0688	slr1423
sll0776	sll1488
sll0002	slr1652
slr0630	slr2088
sll1868	slr1732
sll0898	slr1075
sll0336	slr0958
slr0653	slr1974
sll0788	sll1864
slr1307	slr1857
sll1237	slr0592
sll0577	slr1376
sll0732	slr1595
sll1322	slr0343
sll0413	sll1945
slr1215	slr1301
sll0861	sll1774
sll1354	sll1934
slr0653	sml0011
slr0480	slr0942
slr0889	slr1739
sll1965	slr0213
sll0274	slr2131
slr0963	slr0980
slr0369	slr0797
sll0641	slr1667
sll0456	slr0962
sll0269	sll1814
sll0779	slr1655
sll1868	slr1398
sll1393	sll1502
sll1542	slr2032
sll0418	slr1906
slr0602	slr1644
sll1730	slr1204
sll0865	ssr2201
slr1651	ssl3389
sll0020	slr2098
sll0192	sll0413
sll0361	slr2013
sll0210	slr1117
sll0216	slr1880
sll0532	slr0321
sll1293	slr1028
sll0149	ssr1480
slr0565	slr1471
sll0540	sll1913
slr0359	ssl2996
sll0459	sll1061
sll1775	slr1420
sll0350	slr0950

sll0370	sll1864
sll0853	slr0351
slr0292	slr1756
sll1119	slr1966
sll0142	slr0331
slr0263	slr1484
sll0149	sll1651
sll1969	slr0280
slr0554	ssl3291
slr0263	slr0442
slr1699	slr1888
slr0798	smr0009
slr1966	slr1968
sll1913	slr0599
sll1236	slr1974
sll1129	slr1940
sll0985	slr1855
slr1821	slr1950
slr2038	ssr1473
sll1276	slr1529
slr0250	slr1664
sll1466	slr1194
slr0551	slr1774
sll0536	sll1797
slr0399	slr1920
sll0586	sll1143
sll0776	slr1032
slr0765	slr2087
slr0360	slr1732
slr0147	slr0676
sll1143	slr1629
sll1527	slr2120
sll1086	slr1708
sll0985	slr1541
slr1385	slr1984
sll1547	slr0957
sll0142	sll1752
sll0361	sll0641
sll0821	slr2053
sll0108	sll1920
sll1355	slr0733
slr1835	ssr2781
sll0535	ssr1513
sll5042	slr1902
sll0149	ssl0090
slr1761	slr1978
slr1272	slr1732
sll1527	ssl0331
sll1880	slr1240
sll0359	sll0822
slr0844	slr1204

sll0154	ssl3692
slr0348	slr1287
sll1353	ssl1784
sll1679	sll1951
sll2005	slr1431
sll1006	smr0006
slr0794	smr0001
slr0226	slr0774
sll0071	sll1441
sll0243	sll1262
sll1742	slr0050
sll0149	slr0383
sll0544	slr0446
sll0716	slr0794
sll2005	slr0994
sll0611	ssr1399
sll0350	sll0532
sll0320	slr1505
slr0288	slr1800
slr0346	slr1033
sll1296	ssl2999
sll1006	slr2033
sll1749	slr1641
sll0208	slr0243
sll1526	slr0484
sll1214	slr0797
sll1669	sll2005
sll0865	slr0992
sll0499	slr0121
sll0149	ssr2016
sll1317	slr0454
sll0053	sll0175
sll0804	ssr2142
sll1343	sll1946
sll1225	ssl2420
slr0650	slr0654
sll0871	slr1300
sll1536	slr0821
sll0169	slr1127
sll0865	ssl0352
sll0252	slr0549
slr0467	slr1919
slr2031	ssl3291
sll1751	slr0143
slr0211	slr1222
slr0919	slr1917
sll1797	slr7024
sll0572	sll1803
sll1965	ssl3719
sll0184	slr0889
sll0776	sll1435

sll0821	sll1763
sll2012	slr0668
sll0238	ssr1386
sll1841	slr1096
sll1427	ssl2733
slr0733	ssl1707
sll0380	sll1446
sll0763	slr1263
sll0162	slr0093
slr0733	slr1653
sll1214	slr0420
sll0149	ssr1736
slr1098	slr1974
sll0513	sll1003
sll1799	sll1802
sll0676	sll1831
sll0584	sll0672
sll0865	slr2076
sll0615	sll1614
sll0297	sll0821
sll0413	slr0844
slr0733	ssl2065
sll1831	ssl2996
sll0446	sll1555
slr0335	ssl3692
slr1176	slr1519
sll0932	sll1418
sll1001	sll1863
sll0053	slr0280
sll1366	ssr3154
sll0185	sll1614
sll0375	slr0366
sll0798	smr0010
sll1383	slr0280
sll0923	ssr0692
sll0267	slr1645
sll0641	slr1391
sll0596	sll1757
sll0985	ssr1375
sll1119	slr1968
slr0482	ssl2542
sll1394	slr1923
sll0178	ssl0294
sll0499	sll1101
slr1495	slr1848
slr0586	slr1110
slr1212	ssr2803
sll0022	slr1691
sll0588	slr1266
sll0584	sll1920
sll0547	slr1787

sll0022	sll0641
sll0454	slr1431
sll1231	slr1571
slr0280	slr0845
sll1912	slr0280
slr0942	slr1495
sll0301	sll1614
sll0897	sll1505
sll0169	slr1518
sll0833	ssr3451
slr0935	slr0994
sll0010	sll1272
sll0262	slr0369
sll0149	sll0286
slr0798	slr1052
sll0057	sll1180
sll1270	ssl0353
slr1440	ssr2201
sll1526	sll2001
slr0542	slr7037
sll0103	sll0985
sll1699	slr0909
sll0638	sll0779
sll0732	slr1229
slr1139	slr1699
slr1030	slr1777
sll1002	sll1614
sll0732	sll1154
sll0144	slr1062
sll1193	slr0604
sll0818	slr1140
sll1737	slr0640
slr1508	slr1885
sll0149	slr0318
sll0252	slr1431
slr1413	slr1918
sll1084	slr1083
slr0351	slr0615
slr0280	slr1223
sll0504	slr0302
slr1851	slr1968
sll0189	slr0171
slr0569	ssr3154
sll1239	sll1350
sll0536	slr1531
sll1318	slr0214
slr0543	slr0966
sll0252	slr0386
slr0369	sml0003
sll0149	ssl0739
slr1596	ssr2142

sll0310	ssr2803
sll1277	slr0992
sll0350	slr1799
slr0479	slr1349
slr0653	slr1098
sll1251	ssr1399
sll1595	slr1812
sll0252	sll1578
sll0173	slr1777
sll1841	sll1908
sll1613	slr0697
sll1356	ssl1255
slr1230	slr1812
sll0274	sll1229
slr0747	ssl3177
sll0242	ssr2049
sll0739	sll1393
sll0861	slr2115
sll0445	sll1563
slr0664	slr2098
sll0996	slr0460
sll0149	slr1974
slr1495	ssl2807
sll1613	slr1938
sll0735	slr1604
slr1248	ssr1386
sll1277	sll1442
sll0558	slr1022
sll1293	sll1586
slr0152	slr2102
slr0604	slr0740
slr2121	ssr1527
sll1614	smr0001
sll1129	slr1077
sll0497	slr0394
slr2080	ssr3532
slr1943	slr1959
sll1709	sll1967
sll1658	slr0525
slr1129	slr1557
sll0252	ssr3154
sll1262	slr0649
sll0178	sll1394
sll0784	sll1773
sll1689	sll1965
slr0971	slr1644
sll0191	sll1296
slr1090	slr1900
sll2005	slr0476
slr1661	slr2098
sll1001	slr0569

sll0723	sll1540
slr1194	slr1557
slr1275	slr1969
sll1455	sll1477
sll0609	slr2131
slr1136	ssr2194
sll0402	slr1441
sll0503	sll1743
slr0293	slr0810
sll1435	ssr1375
slr1303	ssl2542
sll1193	slr1932
slr0836	ssr1480
sll1222	slr0697
sll1920	slr0226
sll1446	slr1851
sll1864	slr0974
sll0068	sll1084
slr1495	ssr0536
sll0865	ssr2016
slr1855	ssl3044
sll0263	slr0191
slr0681	slr2087
sll1167	sll1411
sll1143	sll1835
slr1423	ssl0331
sll1614	sml0003
sll1942	slr1644
sll0042	slr1645
sll0065	sll0184
sll0199	slr0454
sll1173	slr0919
slr0852	slr1907
sll0041	slr1636
sll1653	slr1971
sll1087	sml0003
sll0445	slr0147
sll1738	ssl3379
sll1679	sll1772
sll1802	ssr1480
sll1751	slr1301
slr0519	slr0861
sll1954	sll2010
sll1527	ssr3300
sll0354	slr1866
sll0495	slr1235
sll0149	ssl2507
sll0507	slr0121
sll1625	ssr1698
slr0978	slr1400
slr0697	slr0980

slr0810	slr2099
slr0322	ssl1377
slr0510	slr1139
slr0602	ssr2194
sll1580	slr1932
sll0301	slr1376
slr0545	slr2067
sll0446	sll0461
sll1441	slr0962
sll0314	sll1835
sll1455	sll1625
slr1760	ssl3142
sll0010	slr0364
sll0898	slr0687
sll1354	slr1851
sll1186	slr1702
slr0179	slr1591
sll0932	sll1317
slr1591	slr1974
slr0938	slr1773
slr1227	slr1307
sll8019	slr1511
sll0590	slr2135
sll0192	slr1761
sll0540	slr1198
sll1315	slr0272
slr0842	ssr2615
slr0839	slr1235
sll0149	ssr0757
slr1760	slr1812
slr0280	slr1420
sll1564	sll1675
slr0868	slr1322
sll0271	slr0679
slr0676	slr2092
sll0507	ssr0536
sll0182	sll1614
slr0810	slr8023
slr0186	slr2121
sll0488	sll0781
sll0933	slr1471
slr1471	ssr1386
slr1538	slr1557
slr0607	slr2130
sll0621	sll1317
sll1623	sll1937
slr1044	slr2033
slr1533	slr2048
sll1774	slr0924
sll0261	ssl1762
sll0270	slr2092

sll0985	sll1612
sll0314	slr0103
sll1003	sll1252
slr1064	slr1431
slr0108	slr0844
sll2012	slr0506
sll1098	sll1864
slr1229	slr2092
sll0689	ssr2142
sll1446	slr1152
slr0978	slr1812
slr1530	slr2045
sll0488	ssr2781
sll0252	slr0157
sll1058	slr0038
sll0804	slr0765
slr1107	slr1369
slr0923	slr1356
sll0554	sll1799
sll1360	slr1533
sll1799	ssr0536
slr0348	slr1495
slr1761	slr1918
sll1800	slr1315
sll0398	slr2141
sll0242	ssr3300
slr0386	ssl2296
sll0322	slr1644
sll0136	sll0252
slr0305	slr0765
sll0252	slr1865
sll0728	sll1562
sll0053	sll1864
sll1614	slr0260
sll0822	slr1896
sll1463	slr1727
sll0865	slr1699
slr0454	smr0010
slr0794	slr0795
sll0319	slr1505
slr0104	slr1629
sll1653	sll1913
sll0985	ssl0707
sll1226	slr2025
sll1871	ssl2598
slr0073	slr0845
sll0584	sll1614
sll0017	ssl2542
sll1699	slr0551
sll0043	ssl3692
slr0280	slr0549

sll1371	slr2026
sll0169	sll0819
slr0848	slr1048
sll0172	sll0173
sll0616	slr1697
slr0795	slr0797
slr0451	slr1441
slr1455	slr1842
slr0404	slr1603
sll0944	slr1934
slr0774	slr0815
slr1239	slr1350
sll0070	sll0577
slr1364	slr1992
sll0779	slr1978
sll1371	slr0980
sll0252	slr1614
sll0418	sll0871
sll0178	slr1340
sll1231	slr0495
sll1679	slr1641
slr0876	slr1853
sll0198	slr2124
sll1556	sll1643
slr0476	slr2018
slr1584	slr1732
slr0431	ssl0750
sll0310	slr1571
sll0641	slr1266
sll1057	slr1896
sll1499	slr2098
slr0114	ssr2142
sll0377	sll1174
sll0763	slr1245
sll1803	slr1055
sll0007	sll1902
slr0707	ssr0692
sll1172	slr1369
sll0094	slr1385
sll1193	slr1550
sll0274	slr0369
sll0177	sll1218
sll1272	slr1258
sll1231	sll1749
sll1454	slr1353
slr0040	slr1821
sll0763	slr0520
sll0812	sll1800
sll1193	slr1641
sll0809	slr1759
slr0638	slr1603

sll0547	sll1426
slr0667	slr0668
sll1577	slr0280
sll0861	slr1071
sll1222	slr1025
sll1231	sll1658
sll0534	sll1562
slr0066	slr1614
sll1354	ssl1255
slr0662	slr2019
sll1920	sml0005
sll1885	slr0467
slr1608	slr1708
sll0177	slr0955
sll1092	slr2098
slr0112	slr0602
sll0169	sll0914
sll0702	slr1774
sll0188	slr0491
sll0865	sll1630
sll0408	slr2144
slr0909	ssr0692
sll1527	sll5006
sll0068	slr2125
sll1789	slr0418
sll1864	slr1343
sll1634	sll1817
sll0865	ssl3712
slr1956	ssr2803
sll1879	slr0192
sll0933	sll1166
sll1371	slr1812
slr1052	slr1444
sll0226	slr0615
sll1987	slr0006
sll0252	sll1830
sll0992	slr1544
sll0944	slr1842
slr0063	slr0609
slr0360	slr1847
sll2002	ssl3076
sll0912	sll1805
slr0798	slr1726
sll1929	slr1179
sll1874	slr1702
slr1127	slr1300
sll1062	ssl2148
sll0932	slr2011
sll0252	slr0586
sll1033	ssl2667
sll2012	slr1082

sll1858	slr1052
sll1084	slr0630
sll0985	slr1768
sll0804	slr1046
slr0445	slr1610
sll0914	sll1427
sll0071	slr1761
sll0911	slr1594
slr0942	slr1420
sll0915	ssl3076
sll0418	sll1752
slr0426	slr1699
sll0286	sll1178
sll0861	slr0315
slr0765	ssr2142
sll1488	sll1516
sll1143	slr0679
slr1274	slr1636
slr0733	ssr2439
sll0659	slr0442
sll1611	smr0007
sll0631	slr0994
sll0744	slr0810
sll1625	slr1992
sll0664	slr1045
sll0182	slr1107
slr0149	slr0495
sll1817	slr0053
sll0178	slr0192
slr0692	ssr2843
sll0865	ssl2065
slr0070	ssl1762
slr0930	slr1125
sll0754	sll1166
sll0007	slr1699
sll0194	sll1489
slr0496	slr1066
sll1058	slr0658
slr0451	ssl1784
sll1867	slr1596
sll0888	slr0467
ssl2733	ssr2962
slr0121	ssl0353
sll1037	ssl0452
sll0067	ssl0109
sll1427	slr1604
slr1863	ssr3571
slr1391	slr1812
sll1424	sll1660
slr0077	slr1834
sll0380	sll1174

sll0985	slr1329
sll1715	slr0467
sll1769	slr1048
sll0661	slr1992
slr0445	ssr2843
sll1787	sll2006
slr0243	slr1125
sll0274	slr0594
sll1231	sll1671
sll0671	slr1052
sll1917	slr1773
sll0985	slr1945
sll0540	sll1293
sll0752	slr1644
sll1516	slr2037
slr0846	slr1902
sll1063	slr1204
sll1186	slr1451
slr1556	ssl3044
sll0778	sll0933
slr0287	slr2113
sll1656	slr1645
slr1044	slr1052
sll0043	ssl2296
sll0586	sll0915
sll0189	slr0009
sll1703	slr1343
sll1987	slr0818
slr1834	ssl0563
slr2131	ssl2615
sll0252	slr0942
slr0287	slr1075
sll0350	slr0480
slr2114	ssl0109
sll0520	sll1938
slr0031	slr0479
slr1805	slr1900
sll1101	slr0846
slr0348	slr1235
sll1143	sll1193
sll0286	slr0733
sll1098	sll1293
sll1528	slr1254
sll0595	sll0861
sll0776	slr0602
sll1643	slr1934
sll0534	sll0861
sll1196	slr1139
sll1750	slr2098
slr1378	slr1854
sll1908	slr1591

slr0523	slr1023
sll1477	slr1198
sll1614	slr1655
sll0252	sll0997
slr1942	ssl0352
sll0020	sll0822
sll0616	sll1799
sll1689	ssl1707
slr0208	slr0444
sll0379	sll1525
slr0226	slr0496
slr0394	slr1773
sll1543	ssl2999
slr0280	slr1081
sll1352	slr1811
sll1135	sll1533
slr1239	slr1919
slr0848	slr1367
slr0744	slr1025
sll1247	slr1127
sll5052	slr0848
slr0467	slr0675
slr1020	slr2098
slr0341	slr0523
ssl2064	ssl2065
sll1427	slr0209
sll1614	ssl2615
sll1469	slr0467
sll1526	slr0467
slr1222	slr1609
sll1323	slr0602
slr0994	slr1966
sll0413	slr1641
slr0635	slr1417
sll0754	slr2036
sll0398	sll1968
sll0821	sll0985
sll0270	slr1431
sll0270	ssr2843
sll1653	slr1272
sll0944	slr0415
sll0070	slr1614
sll1272	slr0975
sll0519	sll1270
sll0933	sll1951
sll0735	slr1303
sll1802	sll1813
sll0445	sll1323
sll0871	sll1247
sll1120	sll1693
sll0865	slr1440

sll1305	slr1073
sll0270	slr1974
sll0418	slr0108
sll1124	sll1801
slr0854	slr1536
slr1431	slr1573
sll0043	slr1645
slr0058	slr0553
slr0774	smr0004
sll0041	sll0269
slr0565	slr0798
sll1343	slr1799
sll1920	ssl3076
slr0774	slr1215
sll0837	slr0467
sll1317	slr1376
sll0005	sll1699
slr0144	slr0147
slr0445	slr0733
sll0861	sll1864
sll1219	slr1031
slr1044	slr1257
sll0183	sll0381
sll0225	slr1334
sll0280	sll0502
slr0280	slr0942
sll1371	ssl0832
slr0209	slr1431
slr0143	slr0399
sll0554	sll1541
sll0301	slr1950
sll0776	slr1644
sll1323	slr0294
sll0586	ssr2787
slr0675	ssr0854
sll1231	slr0845
sll0821	slr1143
sll1967	slr1566
sll0169	slr1218
slr0377	slr1819
sll0354	ssl1972
sll1614	slr0565
slr0491	slr0599
sll1787	slr0653
slr1325	slr1674
slr1177	ssl1464
slr0546	slr1367
sll0182	sll1060
sll0149	ssr0692
slr1028	slr2084
sll0577	slr0602

sll0080	slr1612
sll1003	ssr2848
sll0418	slr1066
sll0208	sll1643
sll0108	sll0169
sll0023	sll1841
sll0085	sll1471
sll0735	slr2051
sll1356	ssr2201
slr0818	slr1827
slr0921	slr1305
slr1046	slr1471
sll1466	slr0923
sll1143	ssr2049
slr0144	slr2017
sll1912	slr0066
sll0243	ssr2848
sll1393	slr0579
slr0018	slr0392
slr0451	ssr1513
sll1578	slr2031
sll1946	ssr2848
sll2012	slr0707
sll0536	slr1659
slr1596	slr1702
slr1204	slr2005
slr0120	slr1812
sll0865	sll0898
sll1477	sll1763
sll0508	sll1396
sll1193	slr1887
sll0912	slr0653
sll0756	sll1699
sll0309	ssl0750
sll0931	sll1969
sll1394	sll1981
slr0758	slr1299
sll0804	slr1726
sll1669	slr0335
sll0861	slr1827
sll0149	sll1813
slr0103	slr1418
sll1218	slr0452
sll0654	sll0732
sll0865	slr0050
sll1214	slr1350
sll0898	slr0798
sll0525	ssl1004
slr0157	slr0280
sll0735	sll1076
sll0867	sll1959

sll0861	ssl2502
sll0861	slr1441
slr0665	slr1198
sll0554	sll1514
sll0709	slr0744
ssl2148	ssr2615
sll0615	sll0861
slr0006	slr0209
sll1344	slr1565
sll1189	sll1749
sll0149	ssr2787
slr0322	slr1777
slr0818	slr1534
sll1231	slr2005
sll0418	slr1609
slr1992	ssl0105
slr1053	ssl1255
sll1071	sll1353
sll1424	slr0320
sll0923	sll2007
sll1938	slr1025
sll1613	sll1647
sll0821	slr1196
sll0862	sll1440
slr1205	slr1353
sll1552	slr1844
sll1350	sll1751
slr0536	slr2098
sll0088	slr1444
sll1614	slr0343
slr1529	ssr2831
sll0524	slr0793
sll0272	slr2102
sll1614	slr1557
sll0263	sll1509
sll0149	sll1805
slr0204	slr1777
slr0653	slr1330
sll1699	ssl3291
sll1463	ssr1256
sll0354	slr1726
slr1727	slr2131
sll1253	ssr2615
slr1083	slr1998
sll0169	slr0171
sll0026	slr0798
sll0149	ssl0352
sll0985	sll1687
sll1483	slr1664
slr1429	slr2084
slr0309	slr0607

sll0754	slr0594
slr0493	ssr0482
sll1378	slr0280
sll0283	sll1601
slr0156	slr0604
sll1231	ssr1391
sll0861	slr1608
sll0149	sll1811
sll1343	slr1966
sll0449	sll0498
slr0954	slr2098
sll0754	sll1003
slr0165	slr0467
sll0023	sll1454
slr0358	slr2073
slr0244	slr1243
sll1130	ssl2245
sll0169	slr1235
slr1203	slr1325
sll1354	sll1763
sll1799	ssr3122
slr1977	ssl2814
slr1254	slr1638
sll1799	slr0609
sll0861	sll0926
slr1307	slr1443
slr0408	slr1862
ssr0335	ssr0336
slr0366	slr1677
sll0931	slr1885
slr1329	slr1648
sll0149	ssl1377
sll0447	slr1048
sll0057	ssl0105
slr0907	slr1699
sll1483	sll2005
sll1452	sll1660
sll1262	slr1799
sll0149	slr0114
slr0288	slr0744
sll1393	slr1959
slr0602	slr1804
sll1061	sll1424
slr1139	slr1759
sll0103	ssl0294
slr1415	ssr1386
sll1676	slr0351
sll0140	slr1530
slr0993	slr1275
slr1667	ssr0335
sll0182	slr0642

slr1090	ssl1577
sll0252	slr0435
sll1457	slr1194
sll1222	sll1594
slr0734	slr1204
sll1418	slr1593
slr0467	slr0841
slr0011	slr1256
slr0019	ssl2542
slr0598	slr1166
sll1408	slr1902
sll1858	slr0171
sll0223	slr0913
sll0781	slr2098
slr0697	slr1204
slr0451	slr0653
sll0053	slr0435
slr0496	slr1776
sll0355	sll1685
sll1443	ssr2551
slr0650	slr1262
sll0268	sll0915
sll0812	slr1102
sll0149	slr1100
sll1538	slr0451
sll0447	slr0415
sll0507	slr1052
sll0042	sll0572
sll0822	slr0758
sll0911	sll1544
sll0288	sll0289
sll1688	sll1830
sll0252	sll1994
sll0843	sll1396
sll1201	slr1991
sll1011	sll1222
sll0865	slr1437
sll0509	sll1398
sll0497	slr0469
slr0607	slr0697
slr1400	slr1818
slr0008	slr0801
sll1683	ssl1762
sll0641	sll0832
sll0785	ssr1698
sll0355	sll1614
sll0821	sll2007
sll1247	slr0243
sll1327	slr0844
slr1044	slr1045
sll1063	slr0807

slr0019	slr0317
sll0936	slr1906
sll1614	ssr3451
sll1704	sll1921
sll0869	ssl1377
sll1371	slr0199
sll0010	slr0838
sll2007	slr1932
sll1814	slr0798
sll1193	slr0491
sll0192	slr0454
sll1708	slr1896
slr0376	slr0456
sll0103	slr1128
sll0238	slr0343
sll1578	sll1969
sll0995	sll1072
sll1987	slr1035
sll1003	ssl2502
sll0005	slr1710
sll1579	slr0299
sll0782	slr1629
sll0252	sll1143
slr1280	ssr2781
sll0041	sll1225
slr0467	slr1818
sll1435	slr0707
sll0787	slr0015
sll1799	slr0909
sll1060	slr1710
sll1119	slr0919
sll1526	slr2080
sll1370	sll2015
slr1918	slr2077
slr1874	ssl2542
sll0656	sll0767
sll0252	slr0317
sll0362	slr0341
sll1446	slr0977
slr1931	slr2098
sll0149	slr0653
sll1875	slr2025
sll1699	slr0607
sll0310	slr0733
sll0148	sll0350
sll0286	sll0380
sll1601	slr0535
sll0995	slr1778
slr1462	slr2087
slr0054	slr1978
sll1669	slr1837

sll0563	sll1231
sll1580	slr0370
slr0280	slr0550
slr1267	ssl3379
sll1620	slr0733
slr0852	slr1906
sll0385	slr0317
slr0147	slr1073
slr2073	ssr2554
sll1763	slr0359
sll1439	slr0467
sll1441	sll1463
sll1799	slr1531
sll0861	slr0393
sll1861	ssl0331
slr1436	slr1462
slr1881	ssr3532
sll1214	slr1950
sll1555	slr0868
sll0738	slr1046
sll1761	sll1945
sll0488	ssr0854
sll1733	slr1729
sll0477	sll1060
sll0474	sll0933
sll0149	slr0525
sll0166	slr1143
sll0455	sll1813
sll1003	ssr2049
sll1643	ssl0750
sll1477	ssl2542
slr0304	slr0790
slr0280	slr0990
slr0818	slr0877
slr0108	slr0144
sll1428	slr2131
sll1006	slr0723
sll0408	slr1181
slr0408	ssl2065
sll1606	slr1398
sll0788	ssr2049
sll1250	sll1355
slr0214	slr0293
slr1169	slr1567
sll0020	sll1954
sll1679	slr1572
sll1873	ssl0707
sll0424	slr0734
sll0269	slr1052
sll1170	slr0083
slr0872	slr1778

slr1657	slr1659
sll1614	slr2087
sll1669	slr1048
slr1533	slr1857
sll1488	sll1775
sll0672	sll0898
sll0216	sll1496
sll1614	slr0642
sll1452	slr1756
slr0872	slr1963
sll0783	sll1037
sll1138	slr1204
sll1931	slr1198
slr0317	slr1702
sll0058	slr1385
sll0783	slr1420
sll0807	sll1679
slr1343	slr1536
slr0357	slr1626
slr0496	slr1747
sll1110	slr1266
sll1830	slr0697
slr0519	slr0633
sll0735	slr1579
sll0178	ssr2201
sll0272	slr1199
sll0656	ssr1238
sll0252	slr1287
sll1443	slr1023
sll1873	slr2032
slr0506	slr2043
sll0354	slr1609
sll0861	slr0434
sll1085	sll1774
sll1108	sll1933
sll0184	ssl3364
sll0779	slr0565
slr1307	slr1993
sll0898	slr0774
sll1096	slr2046
slr0165	slr0337
slr0848	slr0969
sll1353	slr0740
slr0359	slr1211
sll0794	slr0579
sll0754	slr1363
sll0533	slr1895
slr1812	ssr0757
sll1270	ssr2142
sll0217	sll0513
slr0978	slr1969

sll0614	slr1216
slr0796	slr1235
sll0639	sll1427
sll0149	sll0933
sll0194	slr1471
slr0838	ssl2542
sll2014	slr0299
sll0283	sll0456
sll1180	ssr3571
sll1680	slr0023
sll0208	slr1398
slr0484	slr1083
sll0535	slr1855
sll1129	slr0801
sll1442	slr0772
sll0505	slr1906
sll0223	slr0839
sll1614	slr2033
slr0810	slr1744
sll1143	ssr0692
sll1516	ssr2781
slr0765	slr1052
slr1254	slr5038
sll0909	sll1433
sll0068	ssl0739
sll1367	slr1549
sll0053	sll1366
sll1231	ssr2016
sll1414	sll1651
sll0272	sll1558
sll1029	slr1686
slr0399	slr1645
sll0008	sll0716
sll1632	slr1852
sll0350	sll1268
sll0519	slr0798
sll0053	sll1249
slr0423	slr1265
sll1427	sll1600
slr0261	ssr0692
slr0876	slr1656
sll0185	sll0735
sll0865	slr1464
slr0697	slr1699
sll0041	sll1582
sll1086	slr0993
sll1201	slr1811
slr1291	slr1350
sll0932	slr1215
slr0723	slr1198
sll2005	ssl0294

sll0615	sll1017
sll0380	sll1525
sll0443	sll1187
slr0579	slr1364
sll0354	sll0606
sll1354	slr2053
slr0214	slr0654
sll1477	sll1653
sll0488	sll1239
slr0415	ssl2084
slr0687	slr1110
sll0034	sll0310
sll0021	ssl2807
sll0068	slr0922
slr1992	slr2130
slr0556	slr2098
sll0071	sll1614
sll1643	slr2031
sll0149	ssr1238
slr0050	slr0543
slr0091	slr0384
slr1343	slr1916
sll0192	slr1107
slr1139	slr1454
sll1685	slr0642
sll1170	sll1237
sll0788	sll1472
sll0169	sll1880
sll1715	slr1777
slr0713	slr0877
slr0378	slr0550
sll0624	slr0073
sll1399	slr1590
sll0149	sll1435
sll1987	slr1209
sll0096	sll1557
slr0599	slr1710
sll0886	slr1398
sll1455	slr0697
sll0933	sll1614
slr0596	ssr0657
slr0384	slr1834
sll1611	slr0798
sll0606	slr2046
sll1360	slr0876
slr0546	slr1329
sll0737	slr1232
sll1514	sll1841
slr0969	slr1959
sll0931	sll1687
sll1930	slr0287

slr0317	slr1763
sll0865	slr1811
sll1135	slr0356
sll5052	slr0020
sll0149	slr1032
sll1967	slr1164
sll1198	ssl2542
sll1545	slr0089
sll1427	slr0406
slr0404	slr1471
sll0361	slr1708
sll0539	slr1067
sll0427	sll1477
sll0175	sll0821
sll0325	slr0611
sll0173	slr0408
sll0005	slr0867
sll0173	sll1443
sll0756	sll1104
sll0497	slr1417
sll0226	sll1180
sll0446	ssl3076
sll1272	slr0267
sll0350	slr1164
sll0666	slr0599
sll0149	ssr1391
sll0216	sll1995
sll1359	slr1673
slr0011	slr0143
sll0985	slr0369
sll1334	ssr2803
sll1442	ssl2807
sll0149	sll1398
slr0545	slr0913
sll0504	slr0941
sll1949	slr1277

slr0451	ssr1386
sll0641	sll1949
sll1886	slr0114
slr1774	ssl0109
slr0454	slr0503
sll0861	sll1169
sll0798	ssl3432
slr0602	smr0009
slr1107	ssl2598
slr0103	slr0551
sll2012	slr1848
slr0848	slr1549
slr0514	slr1289
sll0182	sll0474
slr0008	slr1550
sll1390	slr1376
sll0149	sll1054
sll1104	slr1914
sll2005	sll2007
sll1428	sll1614
sll0616	slr1521
sll1276	slr1727
sll0861	slr1744
sll1987	slr0740
sll1394	slr0884
sll1344	ssr1114
sll1861	slr1235
slr0646	slr1847
slr0787	ssr3154
sll0360	slr1471
sll1672	ssl0241
sll1787	slr1441
sll1703	slr1865
sll0068	sll1658
sll1293	sll1296
slr0653	ssr1258

sll0776	slr0152
sll1598	ssl3441
sll1614	smr0008
slr2077	slr2087
sll0775	sll1433
slr1103	ssl0294
sll0554	sll1103
sll0833	slr1192
slr1693	ssl0832
sll0314	sll0624
sll1679	slr0737
sll0005	sll1942
sll0057	sll1032
slr1964	ssr3572
slr0546	slr1842
sll0051	sll0739
sll0021	sll0022
sll0057	sll1643
sll0812	sll1802
sll0053	sll0309
sll1398	slr0733
sll2006	slr2018
slr0376	ssr1552
sll0488	sll1749
sll0057	slr0697
sll0585	slr0579
sll0671	slr0181
sll1367	sll1949
sll0096	sll1920
slr0165	slr1804
slr0697	slr1614
sll0819	slr0839
slr0519	slr1067
slr1645	slr1983
sll1219	sll1371
sll1401	slr1767

slr0305	slr0774
slr0204	slr0596
sll1871	ssl1577
sll0188	slr0992
slr1150	slr2128
sll1250	slr2092
slr0808	ssl3432
sll1546	sml0011
sll0985	slr1860
sll1553	sll1967
sll0267	ssl0787
sll1699	slr1205
sll0819	slr1288
sll0006	sll0513
sll0914	slr2105
sll0005	sll0992
sll1730	ssr3122
sll0252	sll1912
sll1553	slr1720
sll0513	slr1934
sll1620	slr1902
slr1215	slr1471
sll1671	slr1096
sll1865	slr1441
slr0467	slr0666
sll1864	slr2102
sll0252	sll0564
slr0304	slr2104
sll0242	slr1614
sll0584	sll1270
sll0142	sll1323
ssl0452	ssl0453
sll0584	slr1950
sll1423	sll1812
sll1611	sll1906
sll0149	sml0006

sll0647	sll1679
slr0280	slr0354
sll1507	slr1301
slr0467	slr0787
slr1369	slr2077
slr0452	ssl2542
sll1750	slr0066
slr1331	slr1804
sll1084	slr0658
sll0544	sll0910
slr2136	ssl0563
sll0861	slr0886
sll1787	sll2012
sll0631	slr1614
sll0043	sll0192
sll0320	sll1763
sll0540	slr2038
sll1864	ssl3291
sll1799	slr1978
sll0657	sll0790
slr0473	slr1529
slr0152	slr1604
sll1507	ssl2245
slr0214	ssl0331
sll1393	sll2005
sll1433	slr0942
slr0355	slr0940
sll0242	slr0579
slr1215	slr2131
sll0446	slr0668
sll1150	slr0642
sll0194	slr1225
sll2005	slr1557
sll0488	slr0442

Supplemental Table S2. PPIs as gold standard negative dataset (GSN).

P1	P2	P1	P2	P1	P2	P1	P2	P1	P2
sll0402	sll1322	sll1098	slr0261	sll1196	smr0003	slr0906	slr1510	sll1108	ssl3093
sll0728	sll1322	slr0261	slr0434	sll0745	smr0003	sll1043	slr0906	sll0362	ssl3093
sll0336	sll1322	sll1261	slr0261	sll1815	smr0003	slr0400	slr0906	slr0958	ssl3093
sll1299	sll1322	sll1099	slr0261	sll1059	smr0003	sll1415	slr0906	slr1720	ssl3093
sll1322	ssl2084	slr0261	slr0752	slr1123	smr0003	sll1909	slr0906	sll0179	ssl3093
sll1322	sll2001	slr0261	slr0952	sll1760	smr0003	sll0373	slr0906	sll0454	ssl3093
sll1322	sll1430	slr0261	slr1511	sll0469	smr0003	slr0906	slr2035	sll1553	ssl3093
sll0573	sll1322	sll1605	slr0261	slr1517	smr0003	slr0906	slr2132	slr0638	ssl3093
sll1322	slr1898	sll0990	slr0261	sll0869	smr0003	slr0906	slr0922	slr0220	ssl3093
sll0080	sll1322	sll1633	slr0261	slr1598	smr0003	slr0838	slr0906	slr0357	ssl3093
sll1322	slr1022	slr0018	slr0261	sll0868	smr0003	sll1823	slr0906	sll1362	ssl3093
sll1322	sll1883	sll0567	slr0261	slr0994	smr0003	sll1056	slr0906	slr1550	ssl3093
sll1322	slr1133	slr0261	slr0884	sll0379	smr0003	slr0520	slr0906	sll1074	ssl3093
sll1322	slr0444	sll1342	slr0261	sll1676	smr0003	sll0144	slr0906	slr0649	ssl3093
sll1322	slr2130	slr0261	slr1349	sll0927	smr0003	sll0467	slr0906	sll0495	ssl3093
sll1322	sll1669	sll0593	slr0261	sll0996	smr0003	slr0711	slr0906	sll1425	ssl3093
sll1322	slr0585	sll0220	slr0261	sll0844	smr0003	sll0754	slr0906	sll0502	ssl3093
sll1322	slr2075	sll0899	slr0261	sll1615	smr0003	sll0569	slr0906	slr1703	ssl3093
sll1322	slr2076	slr0261	slr1756	sll0202	smr0003	sll1277	slr0906	sll0078	ssl3093
sll0416	sll1322	sll1085	slr0261	slr0902	smr0003	sll1110	slr0906	slr0557	ssl3093
sll1322	slr0156	sll1931	slr0261	sll1144	smr0003	sll1865	slr0906	slr1884	ssl3093
sll1322	slr1641	sll0083	slr0261	slr0017	smr0003	slr0906	slr1228	slr1031	ssl3093
sll1322	slr0542	slr0261	slr1755	slr1424	smr0003	slr0808	slr0906	slr1793	ssl3093
sll0534	sll1322	sll0057	slr0261	slr1423	smr0003	slr0082	slr0906	slr0633	ssl3093
sll1322	slr0165	sll0017	slr0261	sll2010	smr0003	slr0742	slr0906	slr1278	ssl3093
sll0030	sll1322	sll1185	slr0261	slr0528	smr0003	sll0098	slr0906	slr0783	ssl3093
sll1322	slr0847	slr0261	slr0839	slr1351	smr0003	slr0906	slr1646	sll1198	ssl3093
sll1322	slr0553	sll1876	slr0261	sll0622	smr0003	slr0906	slr1130	sll0204	ssl3093
sll1322	slr0812	sll0900	slr0261	sll0631	smr0003	slr0080	slr0906	sll1750	ssl3093
sll1322	slr1347	slr0261	slr0608	slr0787	smr0003	slr0551	slr0906	sll0420	ssl3093
sll1322	slr1348	slr0261	slr0652	sll1852	smr0003	sll0145	slr0906	slr1256	ssl3093
sll1322	slr1791	slr0084	slr0261	slr1090	smr0003	sll0708	slr0906	sll1639	ssl3093
sll0594	sll1322	sll1893	slr0261	sll0902	smr0003	slr0679	slr0906	slr1899	ssl3093
sll1322	slr0550	slr0261	slr0500	slr0661	smr0003	slr0722	slr0906	sll0643	ssl3093
sll1058	sll1322	slr0261	slr1560	slr0526	smr0003	slr0072	slr0906	slr1844	ssl3093
sll1322	slr1665	sll1988	slr0261	sll1249	smr0003	sll1547	slr0906	sll0459	ssl3093
sll1322	slr0536	slr0261	slr1289	sll0892	smr0003	sll1234	slr0906	sll0865	ssl3093
sll1322	slr1874	sll1556	slr0261	sll0660	smr0003	sll0616	slr0906	slr0733	ssl3093
sll1322	sll1776	slr0261	ssl3441	slr1779	smr0003	slr0906	slr2073	sll1459	ssl3093
sll0848	sll1322	slr0261	slr0744	slr0394	smr0003	slr0906	slr1639	sll0905	ssl3093
sll0897	sll1322	slr0261	slr0974	slr2047	smr0003	sll1108	slr0906	sll0402	sll0616
sll1322	sll1933	slr0261	slr1622	slr0741	smr0003	sll0362	slr0906	sll0616	sll0728
sll1322	slr0965	sll1196	slr0261	slr1510	smr0003	slr0906	slr0958	sll0336	sll0616
sll1322	slr1463	sll0745	slr0261	sll1043	smr0003	slr0906	slr1720	sll0616	sll1299
sll1098	sll1322	sll1815	slr0261	slr0400	smr0003	sll0179	slr0906	sll0616	ssl2084
sll1322	slr0434	sll1059	slr0261	sll1415	smr0003	sll0454	slr0906	sll0616	sll2001
sll1261	sll1322	slr0261	slr1123	sll1909	smr0003	sll1553	slr0906	sll0616	sll1430
sll1099	sll1322	sll1760	slr0261	sll0373	smr0003	slr0638	slr0906	sll0573	sll0616
sll1322	slr0752	sll0469	slr0261	slr2035	smr0003	slr0220	slr0906	sll0616	slr1898

sll1322	slr0952	slr0261	slr1517	slr2132	smr0003	slr0357	slr0906	sll0080	sll0616
sll1322	slr1511	sll0869	slr0261	slr0922	smr0003	sll1362	slr0906	sll0616	slr1022
sll1322	sll1605	slr0261	slr1598	slr0838	smr0003	slr0906	slr1550	sll0616	sll1883
sll0990	sll1322	sll0868	slr0261	sll1823	smr0003	sll1074	slr0906	sll0616	slr1133
sll1322	sll1633	slr0261	slr0994	sll1056	smr0003	slr0649	slr0906	sll0616	slr0444
sll1322	slr0018	sll0379	slr0261	slr0520	smr0003	sll0495	slr0906	sll0616	slr2130
sll0567	sll1322	sll1676	slr0261	sll0144	smr0003	sll1425	slr0906	sll0616	sll1669
sll1322	slr0884	sll0927	slr0261	sll0467	smr0003	sll0502	slr0906	sll0616	slr0585
sll1322	sll1342	sll0996	slr0261	slr0711	smr0003	slr0906	slr1703	sll0616	slr2075
sll1322	slr1349	sll0844	slr0261	sll0754	smr0003	sll0078	slr0906	sll0616	slr2076
sll0593	sll1322	sll1615	slr0261	sll0569	smr0003	slr0557	slr0906	sll0416	sll0616
sll0220	sll1322	sll0202	slr0261	sll1277	smr0003	slr0906	slr1884	sll0616	slr0156
sll0899	sll1322	slr0261	slr0902	sll1110	smr0003	slr0906	slr1031	sll0616	slr1641
sll1322	slr1756	sll1144	slr0261	sll1865	smr0003	slr0906	slr1793	sll0616	slr0542
sll1085	sll1322	slr0017	slr0261	slr1228	smr0003	slr0633	slr0906	sll0534	sll0616
sll1322	sll1931	slr0261	slr1424	slr0808	smr0003	slr0906	slr1278	sll0616	slr0165
sll0083	sll1322	slr0261	slr1423	slr0082	smr0003	slr0783	slr0906	sll0030	sll0616
sll1322	slr1755	sll2010	slr0261	slr0742	smr0003	sll1198	slr0906	sll0616	slr0847
sll0057	sll1322	slr0261	slr0528	sll0098	smr0003	sll0204	slr0906	sll0616	slr0553
sll0017	sll1322	slr0261	slr1351	slr1646	smr0003	sll1750	slr0906	sll0616	slr0812
sll1185	sll1322	sll0622	slr0261	slr1130	smr0003	sll0420	slr0906	sll0616	slr1347
sll1322	slr0839	sll0631	slr0261	slr0080	smr0003	slr0906	slr1256	sll0616	slr1348
sll1322	sll1876	slr0261	slr0787	slr0551	smr0003	sll1639	slr0906	sll0616	slr1791
sll0900	sll1322	sll1852	slr0261	sll0145	smr0003	slr0906	slr1899	sll0594	sll0616
sll1322	slr0608	slr0261	slr1090	sll0708	smr0003	sll0643	slr0906	sll0616	slr0550
sll1322	slr0652	sll0902	slr0261	slr0679	smr0003	slr0906	slr1844	sll0616	sll1058
sll1322	slr0084	slr0261	slr0661	slr0722	smr0003	sll0459	slr0906	sll0616	slr1665
sll1322	sll1893	slr0261	slr0526	slr0072	smr0003	sll0865	slr0906	sll0616	slr0536
sll1322	slr0500	sll1249	slr0261	sll1547	smr0003	slr0733	slr0906	sll0616	slr1874
sll1322	slr1560	sll0892	slr0261	sll1234	smr0003	sll1459	slr0906	sll0616	sll1776
sll1322	sll1988	sll0660	slr0261	sll0616	smr0003	sll0905	slr0906	sll0616	sll0848
sll1322	slr1289	slr0261	slr1779	slr2073	smr0003	sll0402	sll0851	sll0616	sll0897
sll1322	sll1556	slr0261	slr0394	slr1639	smr0003	sll0728	sll0851	sll0616	sll1933
sll1322	ssl3441	slr0261	slr2047	sll1108	smr0003	sll0336	sll0851	sll0616	slr0965
sll1322	slr0744	slr0261	slr0741	sll0362	smr0003	sll0851	sll1299	sll0616	slr1463
sll1322	slr0974	slr0261	slr1510	slr0958	smr0003	sll0851	ssl2084	sll0616	sll1098
sll1322	slr1622	sll1043	slr0261	slr1720	smr0003	sll0851	sll2001	sll0616	slr0434
sll1196	sll1322	slr0261	slr0400	sll0179	smr0003	sll0851	sll1430	sll0616	sll1261
sll0745	sll1322	sll1415	slr0261	sll0454	smr0003	sll0573	sll0851	sll0616	sll1099
sll1322	sll1815	sll1909	slr0261	sll1553	smr0003	sll0851	slr1898	sll0616	slr0752
sll1059	sll1322	sll0373	slr0261	slr0638	smr0003	sll0080	sll0851	sll0616	slr0952
sll1322	slr1123	slr0261	slr2035	slr0220	smr0003	sll0851	slr1022	sll0616	slr1511
sll1322	sll1760	slr0261	slr2132	slr0357	smr0003	sll0851	sll1883	sll0616	sll1605
sll0469	sll1322	slr0261	slr0922	sll1362	smr0003	sll0851	slr1133	sll0616	sll0990
sll1322	slr1517	slr0261	slr0838	slr1550	smr0003	sll0851	slr0444	sll0616	sll1633
sll0869	sll1322	sll1823	slr0261	sll1074	smr0003	sll0851	slr2130	sll0616	slr0018
sll1322	slr1598	sll1056	slr0261	slr0649	smr0003	sll0851	sll1669	sll0567	sll0616
sll0868	sll1322	slr0261	slr0520	sll0495	smr0003	sll0851	slr0585	sll0616	slr0884
sll1322	slr0994	sll0144	slr0261	sll1425	smr0003	sll0851	slr2075	sll0616	sll1342
sll0379	sll1322	sll0467	slr0261	sll0502	smr0003	sll0851	slr2076	sll0616	slr1349
sll1322	sll1676	slr0261	slr0711	slr1703	smr0003	sll0416	sll0851	sll0593	sll0616

sll0927	sll1322
sll0996	sll1322
sll0844	sll1322
sll1322	sll1615
sll0202	sll1322
sll1322	slr0902
sll1144	sll1322
sll1322	slr0017
sll1322	slr1424
sll1322	slr1423
sll1322	sll2010
sll1322	slr0528
sll1322	slr1351
sll0622	sll1322
sll0631	sll1322
sll1322	slr0787
sll1322	sll1852
sll1322	slr1090
sll0902	sll1322
sll1322	slr0661
sll1322	slr0526
sll1249	sll1322
sll0892	sll1322
sll0660	sll1322
sll1322	slr1779
sll1322	slr0394
sll1322	slr2047
sll1322	slr0741
sll1322	slr1510
sll1043	sll1322
sll1322	slr0400
sll1322	sll1415
sll1322	sll1909
sll0373	sll1322
sll1322	slr2035
sll1322	slr2132
sll1322	slr0922
sll1322	slr0838
sll1322	sll1823
sll1056	sll1322
sll1322	slr0520
sll0144	sll1322
sll0467	sll1322
sll1322	slr0711
sll0754	sll1322
sll0569	sll1322
sll1277	sll1322
sll1110	sll1322
sll1322	sll1865
sll1322	slr1228
sll1322	slr0808

sll0754	slr0261
sll0569	slr0261
sll1277	slr0261
sll1110	slr0261
sll1865	slr0261
slr0261	slr1228
slr0261	slr0808
slr0082	slr0261
slr0261	slr0742
sll0098	slr0261
slr0261	slr1646
slr0261	slr1130
slr0080	slr0261
slr0261	slr0551
sll0145	slr0261
sll0708	slr0261
slr0261	slr0679
slr0261	slr0722
slr0072	slr0261
sll1547	slr0261
sll1234	slr0261
sll0616	slr0261
slr0261	slr2073
slr0261	slr1639
sll1108	slr0261
sll0362	slr0261
slr0261	slr0958
slr0261	slr1720
sll0179	slr0261
sll0454	slr0261
sll1553	slr0261
slr0261	slr0638
slr0220	slr0261
slr0261	slr0357
sll1362	slr0261
slr0261	slr1550
sll1074	slr0261
slr0261	slr0649
sll0495	slr0261
sll1425	slr0261
sll0502	slr0261
slr0261	slr1703
sll0078	slr0261
slr0261	slr0557
slr0261	slr1884
slr0261	slr1031
slr0261	slr1793
slr0261	slr0633
slr0261	slr1278
slr0261	slr0783
sll1198	slr0261

sll0078	smr0003
slr0557	smr0003
slr1884	smr0003
slr1031	smr0003
slr1793	smr0003
slr0633	smr0003
slr1278	smr0003
slr0783	smr0003
sll1198	smr0003
sll0204	smr0003
sll1750	smr0003
sll0420	smr0003
slr1256	smr0003
sll1639	smr0003
slr1899	smr0003
sll0643	smr0003
slr1844	smr0003
sll0459	smr0003
sll0865	smr0003
slr0733	smr0003
sll1459	smr0003
sll0905	smr0003
sll0402	sml0004
sll0728	sml0004
sll0336	sml0004
sll1299	sml0004
sml0004	ssl2084
sll2001	sml0004
sll1430	sml0004
sll0573	sml0004
slr1898	sml0004
sll0080	sml0004
slr1022	sml0004
sll1883	sml0004
slr1133	sml0004
slr0444	sml0004
slr2130	sml0004
sll1669	sml0004
slr0585	sml0004
slr2075	sml0004
slr2076	sml0004
sll0416	sml0004
slr0156	sml0004
slr1641	sml0004
slr0542	sml0004
sll0534	sml0004
slr0165	sml0004
sll0030	sml0004
slr0847	sml0004
slr0553	sml0004
slr0812	sml0004

sll0851	slr0156
sll0851	slr1641
sll0851	slr0542
sll0534	sll0851
sll0851	slr0165
sll0030	sll0851
sll0851	slr0847
sll0851	slr0553
sll0851	slr0812
sll0851	slr1347
sll0851	slr1348
sll0851	slr1791
sll0594	sll0851
sll0851	slr0550
sll0851	sll1058
sll0851	slr1665
sll0851	slr0536
sll0851	slr1874
sll0851	sll1776
sll0848	sll0851
sll0851	sll0897
sll0851	sll1933
sll0851	slr0965
sll0851	slr1463
sll0851	sll1098
sll0851	slr0434
sll0851	sll1261
sll0851	sll1099
sll0851	slr0752
sll0851	slr0952
sll0851	slr1511
sll0851	sll1605
sll0851	sll0990
sll0851	sll1633
sll0851	slr0018
sll0567	sll0851
sll0851	slr0884
sll0851	sll1342
sll0851	slr1349
sll0593	sll0851
sll0220	sll0851
sll0851	sll0899
sll0851	slr1756
sll0851	sll1085
sll0851	sll1931
sll0083	sll0851
sll0851	slr1755
sll0057	sll0851
sll0017	sll0851
sll0851	sll1185
sll0851	slr0839

sll0220	sll0616
sll0616	sll0899
sll0616	slr1756
sll0616	sll1085
sll0616	sll1931
sll0083	sll0616
sll0616	slr1755
sll0057	sll0616
sll0017	sll0616
sll0616	sll1185
sll0616	slr0839
sll0616	sll1876
sll0616	sll0900
sll0616	slr0608
sll0616	slr0652
sll0616	slr0084
sll0616	sll1893
sll0616	slr0500
sll0616	slr1560
sll0616	sll1988
sll0616	slr1289
sll0616	sll1556
sll0616	ssl3441
sll0616	slr0744
sll0616	slr0974
sll0616	slr1622
sll0616	sll1196
sll0616	sll0745
sll0616	sll1815
sll0616	sll1059
sll0616	slr1123
sll0616	sll1760
sll0469	sll0616
sll0616	slr1517
sll0616	sll0869
sll0616	slr1598
sll0616	sll0868
sll0616	slr0994
sll0379	sll0616
sll0616	sll1676
sll0616	sll0927
sll0616	sll0996
sll0616	sll0844
sll0616	sll1615
sll0202	sll0616
sll0616	slr0902
sll0616	sll1144
sll0616	slr0017
sll0616	slr1424
sll0616	slr1423
sll0616	sll2010

sll1322	slr0082	sll0204	slr0261	slr1347	sml0004	sll0851	sll1876	sll0616	slr0528
sll1322	slr0742	sll1750	slr0261	slr1348	sml0004	sll0851	sll0900	sll0616	slr1351
sll0098	sll1322	sll0420	slr0261	slr1791	sml0004	sll0851	slr0608	sll0616	sll0622
sll1322	slr1646	slr0261	slr1256	sll0594	sml0004	sll0851	slr0652	sll0616	sll0631
sll1322	slr1130	sll1639	slr0261	slr0550	sml0004	sll0851	slr0084	sll0616	slr0787
sll1322	slr0080	slr0261	slr1899	sll1058	sml0004	sll0851	sll1893	sll0616	sll1852
sll1322	slr0551	sll0643	slr0261	slr1665	sml0004	sll0851	slr0500	sll0616	slr1090
sll0145	sll1322	slr0261	slr1844	slr0536	sml0004	sll0851	slr1560	sll0616	sll0902
sll0708	sll1322	sll0459	slr0261	slr1874	sml0004	sll0851	sll1988	sll0616	slr0661
sll1322	slr0679	sll0865	slr0261	sll1776	sml0004	sll0851	slr1289	sll0616	slr0526
sll1322	slr0072	slr0261	slr0733	sll0848	sml0004	sll0851	sll1556	sll0616	sll1249
sll1322	sll1547	sll1459	slr0261	sll0897	sml0004	sll0851	ssl3441	sll0616	sll0892
sll1234	sll1322	sll0905	slr0261	sll1933	sml0004	sll0851	slr0744	sll0616	sll0660
sll0616	sll1322	sll0402	sll0520	slr0965	sml0004	sll0851	slr0974	sll0616	slr1779
sll1322	slr2073	sll0520	sll0728	slr1463	sml0004	sll0851	slr1622	sll0616	slr0394
sll1322	slr1639	sll0336	sll0520	sll1098	sml0004	sll0851	sll1196	sll0616	slr2047
sll1108	sll1322	sll0520	sll1299	slr0434	sml0004	sll0745	sll0851	sll0616	slr0741
sll0362	sll1322	sll0520	ssl2084	sll1261	sml0004	sll0851	sll1815	sll0616	slr1510
sll1322	slr0958	sll0520	sll2001	sll1099	sml0004	sll0851	sll1059	sll0616	sll1043
sll1322	slr1720	sll0520	sll1430	slr0752	sml0004	sll0851	slr1123	sll0616	slr0400
sll0179	sll1322	sll0520	sll0573	slr0952	sml0004	sll0851	sll1760	sll0616	sll1415
sll0454	sll1322	sll0520	slr1898	slr1511	sml0004	sll0469	sll0851	sll0616	sll1909
sll1322	sll1553	sll0080	sll0520	sll1605	sml0004	sll0851	slr1517	sll0373	sll0616
sll1322	slr0638	sll0520	slr1022	sll0990	sml0004	sll0851	sll0869	sll0616	slr2035
sll1322	slr0220	sll0520	sll1883	sll1633	sml0004	sll0851	slr1598	sll0616	slr2132
sll1322	slr0357	sll0520	slr1133	slr0018	sml0004	sll0851	sll0868	sll0616	slr0922
sll1322	sll1362	sll0520	slr0444	sll0567	sml0004	sll0851	slr0994	sll0616	slr0838
sll1322	slr1550	sll0520	slr2130	slr0884	sml0004	sll0379	sll0851	sll0616	sll1823
sll1074	sll1322	sll0520	sll1669	sll1342	sml0004	sll0851	sll1676	sll0616	sll1056
sll1322	slr0649	sll0520	slr0585	slr1349	sml0004	sll0851	sll0927	sll0616	slr0520
sll0495	sll1322	sll0520	slr2075	sll0593	sml0004	sll0851	sll0996	sll0144	sll0616
sll1322	sll1425	sll0520	slr2076	sll0220	sml0004	sll0844	sll0851	sll0467	sll0616
sll0502	sll1322	sll0416	sll0520	sll0899	sml0004	sll0851	sll1615	sll0616	slr0711
sll1322	slr1703	sll0520	slr0156	slr1756	sml0004	sll0202	sll0851	sll0616	sll0754
sll0078	sll1322	sll0520	slr1641	sll1085	sml0004	sll0851	slr0902	sll0569	sll0616
sll1322	slr0557	sll0520	slr0542	sll1931	sml0004	sll0851	sll1144	sll0616	sll1277
sll1322	slr1884	sll0520	sll0534	sll0083	sml0004	sll0851	slr0017	sll0616	sll1110
sll1322	slr1031	sll0520	slr0165	slr1755	sml0004	sll0851	slr1424	sll0616	sll1865
sll1322	slr1793	sll0030	sll0520	sll0057	sml0004	sll0851	slr1423	sll0616	slr1228
sll1322	slr0633	sll0520	slr0847	sll0017	sml0004	sll0851	sll2010	sll0616	slr0808
sll1322	slr1278	sll0520	slr0553	sll1185	sml0004	sll0851	slr0528	sll0616	slr0082
sll1322	slr0783	sll0520	slr0812	slr0839	sml0004	sll0851	slr1351	sll0616	slr0742
sll1198	sll1322	sll0520	slr1347	sll1876	sml0004	sll0622	sll0851	sll0098	sll0616
sll0204	sll1322	sll0520	slr1348	sll0900	sml0004	sll0631	sll0851	sll0616	slr1646
sll1322	sll1750	sll0520	slr1791	slr0608	sml0004	sll0851	slr0787	sll0616	slr1130
sll0420	sll1322	sll0520	sll0594	slr0652	sml0004	sll0851	sll1852	sll0616	slr0080
sll1322	slr1256	sll0520	slr0550	slr0084	sml0004	sll0851	slr1090	sll0616	slr0551
sll1322	sll1639	sll0520	sll1058	sll1893	sml0004	sll0851	sll0902	sll0145	sll0616
sll1322	slr1899	sll0520	slr1665	slr0500	sml0004	sll0851	slr0661	sll0616	sll0708
sll0643	sll1322	sll0520	slr0536	slr1560	sml0004	sll0851	slr0526	sll0616	slr0679
sll1322	slr1844	sll0520	slr1874	sll1988	sml0004	sll0851	sll1249	sll0616	slr0722

sll0459	sll1322	sll0520	sll1776	slr1289	sml0004	sll0851	sll0892	sll0616	slr0072
sll0865	sll1322	sll0520	sll0848	sll1556	sml0004	sll0660	sll0851	sll0616	sll1547
sll1322	slr0733	sll0520	sll0897	sml0004	ssl3441	sll0851	slr1779	sll0616	sll1234
sll1322	sll1459	sll0520	sll1933	slr0744	sml0004	sll0851	slr0394	sll0616	sll0616
sll0905	sll1322	sll0520	slr0965	slr0974	sml0004	sll0851	slr2047	sll0616	slr2073
sll0402	sll1326	sll0520	slr1463	slr1622	sml0004	sll0851	slr0741	sll0616	slr1639
sll0728	sll1326	sll0520	sll1098	sll1196	sml0004	sll0851	slr1510	sll0616	sll1108
sll0336	sll1326	sll0520	slr0434	sll0745	sml0004	sll0851	sll1043	sll0362	sll0616
sll1299	sll1326	sll0520	sll1261	sll1815	sml0004	sll0851	slr0400	sll0616	slr0958
sll1326	ssl2084	sll0520	sll1099	sll1059	sml0004	sll0851	sll1415	sll0616	slr1720
sll1326	sll2001	sll0520	slr0752	slr1123	sml0004	sll0851	sll1909	sll0179	sll0616
sll1326	sll1430	sll0520	slr0952	sll1760	sml0004	sll0373	sll0851	sll0454	sll0616
sll0573	sll1326	sll0520	slr1511	sll0469	sml0004	sll0851	slr2035	sll0616	sll1553
sll1326	slr1898	sll0520	sll1605	slr1517	sml0004	sll0851	slr2132	sll0616	slr0638
sll0080	sll1326	sll0520	sll0990	sll0869	sml0004	sll0851	slr0922	sll0616	slr0220
sll1326	slr1022	sll0520	sll1633	slr1598	sml0004	sll0851	slr0838	sll0616	slr0357
sll1326	sll1883	sll0520	slr0018	sll0868	sml0004	sll0851	sll1823	sll0616	sll1362
sll1326	slr1133	sll0520	sll0567	slr0994	sml0004	sll0851	sll1056	sll0616	slr1550
sll1326	slr0444	sll0520	slr0884	sll0379	sml0004	sll0851	slr0520	sll0616	sll1074
sll1326	slr2130	sll0520	sll1342	sll1676	sml0004	sll0144	sll0851	sll0616	slr0649
sll1326	sll1669	sll0520	slr1349	sll0927	sml0004	sll0467	sll0851	sll0495	sll0616
sll1326	slr0585	sll0520	sll0593	sll0996	sml0004	sll0851	slr0711	sll0616	sll1425
sll1326	slr2075	sll0220	sll0520	sll0844	sml0004	sll0754	sll0851	sll0502	sll0616
sll1326	slr2076	sll0520	sll0899	sll1615	sml0004	sll0569	sll0851	sll0616	slr1703
sll0416	sll1326	sll0520	slr1756	sll0202	sml0004	sll0851	sll1277	sll0078	sll0616
sll1326	slr0156	sll0520	sll1085	slr0902	sml0004	sll0851	sll1110	sll0616	slr0557
sll1326	slr1641	sll0520	sll1931	sll1144	sml0004	sll0851	sll1865	sll0616	slr1884
sll1326	slr0542	sll0083	sll0520	slr0017	sml0004	sll0851	slr1228	sll0616	slr1031
sll0534	sll1326	sll0520	slr1755	slr1424	sml0004	sll0851	slr0808	sll0616	slr1793
sll1326	slr0165	sll0057	sll0520	slr1423	sml0004	sll0851	slr0082	sll0616	slr0633
sll0030	sll1326	sll0017	sll0520	sll2010	sml0004	sll0851	slr0742	sll0616	slr1278
sll1326	slr0847	sll0520	sll1185	slr0528	sml0004	sll0098	sll0851	sll0616	slr0783
sll1326	slr0553	sll0520	slr0839	slr1351	sml0004	sll0851	slr1646	sll0616	sll1198
sll1326	slr0812	sll0520	sll1876	sll0622	sml0004	sll0851	slr1130	sll0204	sll0616
sll1326	slr1347	sll0520	sll0900	sll0631	sml0004	sll0851	slr0080	sll0616	sll1750
sll1326	slr1348	sll0520	slr0608	slr0787	sml0004	sll0851	slr0551	sll0420	sll0616
sll1326	slr1791	sll0520	slr0652	sll1852	sml0004	sll0145	sll0851	sll0616	slr1256
sll0594	sll1326	sll0520	slr0084	slr1090	sml0004	sll0708	sll0851	sll0616	sll1639
sll1326	slr0550	sll0520	sll1893	sll0902	sml0004	sll0851	slr0679	sll0616	slr1899
sll1058	sll1326	sll0520	slr0500	slr0661	sml0004	sll0851	slr0722	sll0616	sll0643
sll1326	slr1665	sll0520	slr1560	slr0526	sml0004	sll0851	slr0072	sll0616	slr1844
sll1326	slr0536	sll0520	sll1988	sll1249	sml0004	sll0851	sll1547	sll0459	sll0616
sll1326	slr1874	sll0520	slr1289	sll0892	sml0004	sll0851	sll1234	sll0616	sll0865
sll1326	sll1776	sll0520	sll1556	sll0660	sml0004	sll0616	sll0851	sll0616	slr0733
sll0848	sll1326	sll0520	ssl3441	slr1779	sml0004	sll0851	slr2073	sll0616	sll1459
sll0897	sll1326	sll0520	slr0744	slr0394	sml0004	sll0851	slr1639	sll0616	sll0905
sll1326	sll1933	sll0520	slr0974	slr2047	sml0004	sll0851	sll1108	sll0402	slr1034
sll1326	slr0965	sll0520	slr1622	slr0741	sml0004	sll0362	sll0851	sll0728	slr1034
sll1326	slr1463	sll0520	sll1196	slr1510	sml0004	sll0851	slr0958	sll0336	slr1034
sll1098	sll1326	sll0520	sll0745	sll1043	sml0004	sll0851	slr1720	sll1299	slr1034
sll1326	slr0434	sll0520	sll1815	slr0400	sml0004	sll0179	sll0851	slr1034	ssl2084

sll1261	sll1326	sll0520	sll1059	sll1415	sml0004	sll0454	sll0851	sll2001	slr1034
sll1099	sll1326	sll0520	slr1123	sll1909	sml0004	sll0851	sll1553	sll1430	slr1034
sll1326	slr0752	sll0520	sll1760	sll0373	sml0004	sll0851	slr0638	sll0573	slr1034
sll1326	slr0952	sll0469	sll0520	slr2035	sml0004	sll0851	slr0220	slr1034	slr1898
sll1326	slr1511	sll0520	slr1517	slr2132	sml0004	sll0851	slr0357	sll0080	slr1034
sll1326	sll1605	sll0520	sll0869	slr0922	sml0004	sll0851	sll1362	slr1022	slr1034
sll0990	sll1326	sll0520	slr1598	slr0838	sml0004	sll0851	slr1550	sll1883	slr1034
sll1326	sll1633	sll0520	sll0868	sll1823	sml0004	sll0851	sll1074	slr1034	slr1133
sll1326	slr0018	sll0520	slr0994	sll1056	sml0004	sll0851	slr0649	slr0444	slr1034
sll0567	sll1326	sll0379	sll0520	slr0520	sml0004	sll0495	sll0851	slr1034	slr2130
sll1326	slr0884	sll0520	sll1676	sll0144	sml0004	sll0851	sll1425	sll1669	slr1034
sll1326	sll1342	sll0520	sll0927	sll0467	sml0004	sll0502	sll0851	slr0585	slr1034
sll1326	slr1349	sll0520	sll0996	slr0711	sml0004	sll0851	slr1703	slr1034	slr2075
sll0593	sll1326	sll0520	sll0844	sll0754	sml0004	sll0078	sll0851	slr1034	slr2076
sll0220	sll1326	sll0520	sll1615	sll0569	sml0004	sll0851	slr0557	sll0416	slr1034
sll0899	sll1326	sll0202	sll0520	sll1277	sml0004	sll0851	slr1884	slr0156	slr1034
sll1326	slr1756	sll0520	slr0902	sll1110	sml0004	sll0851	slr1031	slr1034	slr1641
sll1085	sll1326	sll0520	sll1144	sll1865	sml0004	sll0851	slr1793	slr0542	slr1034
sll1326	sll1931	sll0520	slr0017	slr1228	sml0004	sll0851	slr0633	sll0534	slr1034
sll0083	sll1326	sll0520	slr1424	slr0808	sml0004	sll0851	slr1278	slr0165	slr1034
sll1326	slr1755	sll0520	slr1423	slr0082	sml0004	sll0851	slr0783	sll0030	slr1034
sll0057	sll1326	sll0520	sll2010	slr0742	sml0004	sll0851	sll1198	slr0847	slr1034
sll0017	sll1326	sll0520	slr0528	sll0098	sml0004	sll0204	sll0851	slr0553	slr1034
sll1185	sll1326	sll0520	slr1351	slr1646	sml0004	sll0851	sll1750	slr0812	slr1034
sll1326	slr0839	sll0520	sll0622	slr1130	sml0004	sll0420	sll0851	slr1034	slr1347
sll1326	sll1876	sll0520	sll0631	slr0080	sml0004	sll0851	slr1256	slr1034	slr1348
sll0900	sll1326	sll0520	slr0787	slr0551	sml0004	sll0851	sll1639	slr1034	slr1791
sll1326	slr0608	sll0520	sll1852	sll0145	sml0004	sll0851	slr1899	sll0594	slr1034
sll1326	slr0652	sll0520	slr1090	sll0708	sml0004	sll0643	sll0851	slr0550	slr1034
sll1326	slr0084	sll0520	sll0902	slr0679	sml0004	sll0851	slr1844	sll1058	slr1034
sll1326	sll1893	sll0520	slr0661	slr0722	sml0004	sll0459	sll0851	slr1034	slr1665
sll1326	slr0500	sll0520	slr0526	slr0072	sml0004	sll0851	sll0865	slr0536	slr1034
sll1326	slr1560	sll0520	sll1249	sll1547	sml0004	sll0851	slr0733	slr1034	slr1874
sll1326	sll1988	sll0520	sll0892	sll1234	sml0004	sll0851	sll1459	sll1776	slr1034
sll1326	slr1289	sll0520	sll0660	sll0616	sml0004	sll0851	sll0905	sll0848	slr1034
sll1326	sll1556	sll0520	slr1779	slr2073	sml0004	sll0402	ssr3451	sll0897	slr1034
sll1326	ssl3441	sll0520	slr0394	slr1639	sml0004	sll0728	ssr3451	sll1933	slr1034
sll1326	slr0744	sll0520	slr2047	sll1108	sml0004	sll0336	ssr3451	slr0965	slr1034
sll1326	slr0974	sll0520	slr0741	sll0362	sml0004	sll1299	ssr3451	slr1034	slr1463
sll1326	slr1622	sll0520	slr1510	slr0958	sml0004	ssl2084	ssr3451	sll1098	slr1034
sll1196	sll1326	sll0520	sll1043	slr1720	sml0004	sll2001	ssr3451	slr0434	slr1034
sll0745	sll1326	sll0520	slr0400	sll0179	sml0004	sll1430	ssr3451	sll1261	slr1034
sll1326	sll1815	sll0520	sll1415	sll0454	sml0004	sll0573	ssr3451	sll1099	slr1034
sll1059	sll1326	sll0520	sll1909	sll1553	sml0004	slr1898	ssr3451	slr0752	slr1034
sll1326	slr1123	sll0373	sll0520	slr0638	sml0004	sll0080	ssr3451	slr0952	slr1034
sll1326	sll1760	sll0520	slr2035	slr0220	sml0004	slr1022	ssr3451	slr1034	slr1511
sll0469	sll1326	sll0520	slr2132	slr0357	sml0004	sll1883	ssr3451	sll1605	slr1034
sll1326	slr1517	sll0520	slr0922	sll1362	sml0004	slr1133	ssr3451	sll0990	slr1034
sll0869	sll1326	sll0520	slr0838	slr1550	sml0004	slr0444	ssr3451	sll1633	slr1034
sll1326	slr1598	sll0520	sll1823	sll1074	sml0004	slr2130	ssr3451	slr0018	slr1034
sll0868	sll1326	sll0520	sll1056	slr0649	sml0004	sll1669	ssr3451	sll0567	slr1034

sll1326	slr0994	sll0520	slr0520	sll0495	sml0004	slr0585	ssr3451	slr0884	slr1034
sll0379	sll1326	sll0144	sll0520	sll1425	sml0004	slr2075	ssr3451	sll1342	slr1034
sll1326	sll1676	sll0467	sll0520	sll0502	sml0004	slr2076	ssr3451	slr1034	slr1349
sll0927	sll1326	sll0520	slr0711	slr1703	sml0004	sll0416	ssr3451	sll0593	slr1034
sll0996	sll1326	sll0520	sll0754	sll0078	sml0004	slr0156	ssr3451	sll0220	slr1034
sll0844	sll1326	sll0520	sll0569	slr0557	sml0004	slr1641	ssr3451	sll0899	slr1034
sll1326	sll1615	sll0520	sll1277	slr1884	sml0004	slr0542	ssr3451	slr1034	slr1756
sll0202	sll1326	sll0520	sll1110	slr1031	sml0004	sll0534	ssr3451	sll1085	slr1034
sll1326	slr0902	sll0520	sll1865	slr1793	sml0004	slr0165	ssr3451	sll1931	slr1034
sll1144	sll1326	sll0520	slr1228	slr0633	sml0004	sll0030	ssr3451	sll0083	slr1034
sll1326	slr0017	sll0520	slr0808	slr1278	sml0004	slr0847	ssr3451	slr1034	slr1755
sll1326	slr1424	sll0520	slr0082	slr0783	sml0004	slr0553	ssr3451	sll0057	slr1034
sll1326	slr1423	sll0520	slr0742	sll1198	sml0004	slr0812	ssr3451	sll0017	slr1034
sll1326	sll2010	sll0098	sll0520	sll0204	sml0004	slr1347	ssr3451	sll1185	slr1034
sll1326	slr0528	sll0520	slr1646	sll1750	sml0004	slr1348	ssr3451	slr0839	slr1034
sll1326	slr1351	sll0520	slr1130	sll0420	sml0004	slr1791	ssr3451	sll1876	slr1034
sll0622	sll1326	sll0520	slr0080	slr1256	sml0004	sll0594	ssr3451	sll0900	slr1034
sll0631	sll1326	sll0520	slr0551	sll1639	sml0004	slr0550	ssr3451	slr0608	slr1034
sll1326	slr0787	sll0145	sll0520	slr1899	sml0004	sll1058	ssr3451	slr0652	slr1034
sll1326	sll1852	sll0520	sll0708	sll0643	sml0004	slr1665	ssr3451	slr0084	slr1034
sll1326	slr1090	sll0520	slr0679	slr1844	sml0004	slr0536	ssr3451	sll1893	slr1034
sll0902	sll1326	sll0520	slr0722	sll0459	sml0004	slr1874	ssr3451	slr0500	slr1034
sll1326	slr0661	sll0520	slr0072	sll0865	sml0004	sll1776	ssr3451	slr1034	slr1560
sll1326	slr0526	sll0520	sll1547	slr0733	sml0004	sll0848	ssr3451	sll1988	slr1034
sll1249	sll1326	sll0520	sll1234	sll1459	sml0004	sll0897	ssr3451	slr1034	slr1289
sll0892	sll1326	sll0520	sll0616	sll0905	sml0004	sll1933	ssr3451	sll1556	slr1034
sll0660	sll1326	sll0520	slr2073	sll0402	slr2067	slr0965	ssr3451	slr1034	ssl3441
sll1326	slr1779	sll0520	slr1639	sll0728	slr2067	slr1463	ssr3451	slr0744	slr1034
sll1326	slr0394	sll0520	sll1108	sll0336	slr2067	sll1098	ssr3451	slr0974	slr1034
sll1326	slr2047	sll0362	sll0520	sll1299	slr2067	slr0434	ssr3451	slr1034	slr1622
sll1326	slr0741	sll0520	slr0958	slr2067	ssl2084	sll1261	ssr3451	sll1196	slr1034
sll1326	slr1510	sll0520	slr1720	sll2001	slr2067	sll1099	ssr3451	sll0745	slr1034
sll1043	sll1326	sll0179	sll0520	sll1430	slr2067	slr0752	ssr3451	sll1815	slr1034
sll1326	slr0400	sll0454	sll0520	sll0573	slr2067	slr0952	ssr3451	sll1059	slr1034
sll1326	sll1415	sll0520	sll1553	slr1898	slr2067	slr1511	ssr3451	slr1034	slr1123
sll1326	sll1909	sll0520	slr0638	sll0080	slr2067	sll1605	ssr3451	sll1760	slr1034
sll0373	sll1326	sll0520	slr0220	slr1022	slr2067	sll0990	ssr3451	sll0469	slr1034
sll1326	slr2035	sll0520	slr0357	sll1883	slr2067	sll1633	ssr3451	slr1034	slr1517
sll1326	slr2132	sll0520	sll1362	slr1133	slr2067	slr0018	ssr3451	sll0869	slr1034
sll1326	slr0922	sll0520	slr1550	slr0444	slr2067	sll0567	ssr3451	slr1034	slr1598
sll1326	slr0838	sll0520	sll1074	slr2067	slr2130	slr0884	ssr3451	sll0868	slr1034
sll1326	sll1823	sll0520	slr0649	sll1669	slr2067	sll1342	ssr3451	slr0994	slr1034
sll1056	sll1326	sll0495	sll0520	slr0585	slr2067	slr1349	ssr3451	sll0379	slr1034
sll1326	slr0520	sll0520	sll1425	slr2067	slr2075	sll0593	ssr3451	sll1676	slr1034
sll0144	sll1326	sll0502	sll0520	slr2067	slr2076	sll0220	ssr3451	sll0927	slr1034
sll0467	sll1326	sll0520	slr1703	sll0416	slr2067	sll0899	ssr3451	sll0996	slr1034
sll1326	slr0711	sll0078	sll0520	slr0156	slr2067	slr1756	ssr3451	sll0844	slr1034
sll0754	sll1326	sll0520	slr0557	slr1641	slr2067	sll1085	ssr3451	sll1615	slr1034
sll0569	sll1326	sll0520	slr1884	slr0542	slr2067	sll1931	ssr3451	sll0202	slr1034
sll1277	sll1326	sll0520	slr1031	sll0534	slr2067	sll0083	ssr3451	slr0902	slr1034
sll1110	sll1326	sll0520	slr1793	slr0165	slr2067	slr1755	ssr3451	sll1144	slr1034

sll1326	sll1865	sll0520	slr0633	sll0030	slr2067	sll0057	ssr3451	slr0017	slr1034
sll1326	slr1228	sll0520	slr1278	slr0847	slr2067	sll0017	ssr3451	slr1034	slr1424
sll1326	slr0808	sll0520	slr0783	slr0553	slr2067	sll1185	ssr3451	slr1034	slr1423
sll1326	slr0082	sll0520	sll1198	slr0812	slr2067	slr0839	ssr3451	sll2010	slr1034
sll1326	slr0742	sll0204	sll0520	slr1347	slr2067	sll1876	ssr3451	slr0528	slr1034
sll0098	sll1326	sll0520	sll1750	slr1348	slr2067	sll0900	ssr3451	slr1034	slr1351
sll1326	slr1646	sll0420	sll0520	slr1791	slr2067	slr0608	ssr3451	sll0622	slr1034
sll1326	slr1130	sll0520	slr1256	sll0594	slr2067	slr0652	ssr3451	sll0631	slr1034
sll1326	slr0080	sll0520	sll1639	slr0550	slr2067	slr0084	ssr3451	slr0787	slr1034
sll1326	slr0551	sll0520	slr1899	sll1058	slr2067	sll1893	ssr3451	sll1852	slr1034
sll0145	sll1326	sll0520	sll0643	slr1665	slr2067	slr0500	ssr3451	slr1034	slr1090
sll0708	sll1326	sll0520	slr1844	slr0536	slr2067	slr1560	ssr3451	sll0902	slr1034
sll1326	slr0679	sll0459	sll0520	slr1874	slr2067	sll1988	ssr3451	slr0661	slr1034
sll1326	slr0722	sll0520	sll0865	sll1776	slr2067	slr1289	ssr3451	slr0526	slr1034
sll1326	slr0072	sll0520	slr0733	sll0848	slr2067	sll1556	ssr3451	sll1249	slr1034
sll1326	sll1547	sll0520	sll1459	sll0897	slr2067	ssl3441	ssr3451	sll0892	slr1034
sll1234	sll1326	sll0520	sll0905	sll1933	slr2067	slr0744	ssr3451	sll0660	slr1034
sll0616	sll1326	sll0402	slr1281	slr0965	slr2067	slr0974	ssr3451	slr1034	slr1779
sll1326	slr2073	sll0728	slr1281	slr1463	slr2067	slr1622	ssr3451	slr0394	slr1034
sll1326	slr1639	sll0336	slr1281	sll1098	slr2067	sll1196	ssr3451	slr1034	slr2047
sll1108	sll1326	sll1299	slr1281	slr0434	slr2067	sll0745	ssr3451	slr0741	slr1034
sll0362	sll1326	slr1281	ssl2084	sll1261	slr2067	sll1815	ssr3451	slr1034	slr1510
sll1326	slr0958	sll2001	slr1281	sll1099	slr2067	sll1059	ssr3451	sll1043	slr1034
sll1326	slr1720	sll1430	slr1281	slr0752	slr2067	slr1123	ssr3451	slr0400	slr1034
sll0179	sll1326	sll0573	slr1281	slr0952	slr2067	sll1760	ssr3451	sll1415	slr1034
sll0454	sll1326	slr1281	slr1898	slr1511	slr2067	sll0469	ssr3451	sll1909	slr1034
sll1326	sll1553	sll0080	slr1281	sll1605	slr2067	slr1517	ssr3451	sll0373	slr1034
sll1326	slr0638	slr1022	slr1281	sll0990	slr2067	sll0869	ssr3451	slr1034	slr2035
sll1326	slr0220	sll1883	slr1281	sll1633	slr2067	slr1598	ssr3451	slr1034	slr2132
sll1326	slr0357	slr1133	slr1281	slr0018	slr2067	sll0868	ssr3451	slr0922	slr1034
sll1326	sll1362	slr0444	slr1281	sll0567	slr2067	slr0994	ssr3451	slr0838	slr1034
sll1326	slr1550	slr1281	slr2130	slr0884	slr2067	sll0379	ssr3451	sll1823	slr1034
sll1074	sll1326	sll1669	slr1281	sll1342	slr2067	sll1676	ssr3451	sll1056	slr1034
sll1326	slr0649	slr0585	slr1281	slr1349	slr2067	sll0927	ssr3451	slr0520	slr1034
sll0495	sll1326	slr1281	slr2075	sll0593	slr2067	sll0996	ssr3451	sll0144	slr1034
sll1326	sll1425	slr1281	slr2076	sll0220	slr2067	sll0844	ssr3451	sll0467	slr1034
sll0502	sll1326	sll0416	slr1281	sll0899	slr2067	sll1615	ssr3451	slr0711	slr1034
sll1326	slr1703	slr0156	slr1281	slr1756	slr2067	sll0202	ssr3451	sll0754	slr1034
sll0078	sll1326	slr1281	slr1641	sll1085	slr2067	slr0902	ssr3451	sll0569	slr1034
sll1326	slr0557	slr0542	slr1281	sll1931	slr2067	sll1144	ssr3451	sll1277	slr1034
sll1326	slr1884	sll0534	slr1281	sll0083	slr2067	slr0017	ssr3451	sll1110	slr1034
sll1326	slr1031	slr0165	slr1281	slr1755	slr2067	slr1424	ssr3451	sll1865	slr1034
sll1326	slr1793	sll0030	slr1281	sll0057	slr2067	slr1423	ssr3451	slr1034	slr1228
sll1326	slr0633	slr0847	slr1281	sll0017	slr2067	sll2010	ssr3451	slr0808	slr1034
sll1326	slr1278	slr0553	slr1281	sll1185	slr2067	slr0528	ssr3451	slr0082	slr1034
sll1326	slr0783	slr0812	slr1281	slr0839	slr2067	slr1351	ssr3451	slr0742	slr1034
sll1198	sll1326	slr1281	slr1347	sll1876	slr2067	sll0622	ssr3451	sll0098	slr1034
sll0204	sll1326	slr1281	slr1348	sll0900	slr2067	sll0631	ssr3451	slr1034	slr1646
sll1326	sll1750	slr1281	slr1791	slr0608	slr2067	slr0787	ssr3451	slr1034	slr1130
sll0420	sll1326	sll0594	slr1281	slr0652	slr2067	sll1852	ssr3451	slr0080	slr1034
sll1326	slr1256	slr0550	slr1281	slr0084	slr2067	slr1090	ssr3451	slr0551	slr1034

sll1326	sll1639	sll1058	slr1281	sll1893	slr2067	sll0902	ssr3451	sll0145	slr1034
sll1326	slr1899	slr1281	slr1665	slr0500	slr2067	slr0661	ssr3451	sll0708	slr1034
sll0643	sll1326	slr0536	slr1281	slr1560	slr2067	slr0526	ssr3451	slr0679	slr1034
sll1326	slr1844	slr1281	slr1874	sll1988	slr2067	sll1249	ssr3451	slr0722	slr1034
sll0459	sll1326	sll1776	slr1281	slr1289	slr2067	sll0892	ssr3451	slr0072	slr1034
sll0865	sll1326	sll0848	slr1281	sll1556	slr2067	sll0660	ssr3451	sll1547	slr1034
sll1326	slr0733	sll0897	slr1281	slr2067	ssl3441	slr1779	ssr3451	sll1234	slr1034
sll1326	sll1459	sll1933	slr1281	slr0744	slr2067	slr0394	ssr3451	sll0616	slr1034
sll0905	sll1326	slr0965	slr1281	slr0974	slr2067	slr2047	ssr3451	slr1034	slr2073
sll0402	slr1329	slr1281	slr1463	slr1622	slr2067	slr0741	ssr3451	slr1034	slr1639
sll0728	slr1329	sll1098	slr1281	sll1196	slr2067	slr1510	ssr3451	sll1108	slr1034
sll0336	slr1329	slr0434	slr1281	sll0745	slr2067	sll1043	ssr3451	sll0362	slr1034
sll1299	slr1329	sll1261	slr1281	sll1815	slr2067	slr0400	ssr3451	slr0958	slr1034
slr1329	ssl2084	sll1099	slr1281	sll1059	slr2067	sll1415	ssr3451	slr1034	slr1720
sll2001	slr1329	slr0752	slr1281	slr1123	slr2067	sll1909	ssr3451	sll0179	slr1034
sll1430	slr1329	slr0952	slr1281	sll1760	slr2067	sll0373	ssr3451	sll0454	slr1034
sll0573	slr1329	slr1281	slr1511	sll0469	slr2067	slr2035	ssr3451	sll1553	slr1034
slr1329	slr1898	sll1605	slr1281	slr1517	slr2067	slr2132	ssr3451	slr0638	slr1034
sll0080	slr1329	sll0990	slr1281	sll0869	slr2067	slr0922	ssr3451	slr0220	slr1034
slr1022	slr1329	sll1633	slr1281	slr1598	slr2067	slr0838	ssr3451	slr0357	slr1034
sll1883	slr1329	slr0018	slr1281	sll0868	slr2067	sll1823	ssr3451	sll1362	slr1034
slr1133	slr1329	sll0567	slr1281	slr0994	slr2067	sll1056	ssr3451	slr1034	slr1550
slr0444	slr1329	slr0884	slr1281	sll0379	slr2067	slr0520	ssr3451	sll1074	slr1034
slr1329	slr2130	sll1342	slr1281	sll1676	slr2067	sll0144	ssr3451	slr0649	slr1034
sll1669	slr1329	slr1281	slr1349	sll0927	slr2067	sll0467	ssr3451	sll0495	slr1034
slr0585	slr1329	sll0593	slr1281	sll0996	slr2067	slr0711	ssr3451	sll1425	slr1034
slr1329	slr2075	sll0220	slr1281	sll0844	slr2067	sll0754	ssr3451	sll0502	slr1034
slr1329	slr2076	sll0899	slr1281	sll1615	slr2067	sll0569	ssr3451	slr1034	slr1703
sll0416	slr1329	slr1281	slr1756	sll0202	slr2067	sll1277	ssr3451	sll0078	slr1034
slr0156	slr1329	sll1085	slr1281	slr0902	slr2067	sll1110	ssr3451	slr0557	slr1034
slr1329	slr1641	sll1931	slr1281	sll1144	slr2067	sll1865	ssr3451	slr1034	slr1884
slr0542	slr1329	sll0083	slr1281	slr0017	slr2067	slr1228	ssr3451	slr1031	slr1034
sll0534	slr1329	slr1281	slr1755	slr1424	slr2067	slr0808	ssr3451	slr1034	slr1793
slr0165	slr1329	sll0057	slr1281	slr1423	slr2067	slr0082	ssr3451	slr0633	slr1034
sll0030	slr1329	sll0017	slr1281	sll2010	slr2067	slr0742	ssr3451	slr1034	slr1278
slr0847	slr1329	sll1185	slr1281	slr0528	slr2067	sll0098	ssr3451	slr0783	slr1034
slr0553	slr1329	slr0839	slr1281	slr1351	slr2067	slr1646	ssr3451	sll1198	slr1034
slr0812	slr1329	sll1876	slr1281	sll0622	slr2067	slr1130	ssr3451	sll0204	slr1034
slr1329	slr1347	sll0900	slr1281	sll0631	slr2067	slr0080	ssr3451	sll1750	slr1034
slr1329	slr1348	slr0608	slr1281	slr0787	slr2067	slr0551	ssr3451	sll0420	slr1034
slr1329	slr1791	slr0652	slr1281	sll1852	slr2067	sll0145	ssr3451	slr1034	slr1256
sll0594	slr1329	slr0084	slr1281	slr1090	slr2067	sll0708	ssr3451	sll1639	slr1034
slr0550	slr1329	sll1893	slr1281	sll0902	slr2067	slr0679	ssr3451	slr1034	slr1899
sll1058	slr1329	slr0500	slr1281	slr0661	slr2067	slr0722	ssr3451	sll0643	slr1034
slr1329	slr1665	slr1281	slr1560	slr0526	slr2067	slr0072	ssr3451	slr1034	slr1844
slr0536	slr1329	sll1988	slr1281	sll1249	slr2067	sll1547	ssr3451	sll0459	slr1034
slr1329	slr1874	slr1281	slr1289	sll0892	slr2067	sll1234	ssr3451	sll0865	slr1034
sll1776	slr1329	sll1556	slr1281	sll0660	slr2067	sll0616	ssr3451	slr0733	slr1034
sll0848	slr1329	slr1281	ssl3441	slr1779	slr2067	slr2073	ssr3451	sll1459	slr1034
sll0897	slr1329	slr0744	slr1281	slr0394	slr2067	slr1639	ssr3451	sll0905	slr1034
sll1933	slr1329	slr0974	slr1281	slr2047	slr2067	sll1108	ssr3451	sll0402	slr1185

slr0965	slr1329	slr1281	slr1622	slr0741	slr2067	sll0362	ssr3451	sll0728	slr1185
slr1329	slr1463	sll1196	slr1281	slr1510	slr2067	slr0958	ssr3451	sll0336	slr1185
sll1098	slr1329	sll0745	slr1281	sll1043	slr2067	slr1720	ssr3451	sll1299	slr1185
slr0434	slr1329	sll1815	slr1281	slr0400	slr2067	sll0179	ssr3451	slr1185	ssl2084
sll1261	slr1329	sll1059	slr1281	sll1415	slr2067	sll0454	ssr3451	sll2001	slr1185
sll1099	slr1329	slr1123	slr1281	sll1909	slr2067	sll1553	ssr3451	sll1430	slr1185
slr0752	slr1329	sll1760	slr1281	sll0373	slr2067	slr0638	ssr3451	sll0573	slr1185
slr0952	slr1329	sll0469	slr1281	slr2035	slr2067	slr0220	ssr3451	slr1185	slr1898
slr1329	slr1511	slr1281	slr1517	slr2067	slr2132	slr0357	ssr3451	sll0080	slr1185
sll1605	slr1329	sll0869	slr1281	slr0922	slr2067	sll1362	ssr3451	slr1022	slr1185
sll0990	slr1329	slr1281	slr1598	slr0838	slr2067	slr1550	ssr3451	sll1883	slr1185
sll1633	slr1329	sll0868	slr1281	sll1823	slr2067	sll1074	ssr3451	slr1133	slr1185
slr0018	slr1329	slr0994	slr1281	sll1056	slr2067	slr0649	ssr3451	slr0444	slr1185
sll0567	slr1329	sll0379	slr1281	slr0520	slr2067	sll0495	ssr3451	slr1185	slr2130
slr0884	slr1329	sll1676	slr1281	sll0144	slr2067	sll1425	ssr3451	sll1669	slr1185
sll1342	slr1329	sll0927	slr1281	sll0467	slr2067	sll0502	ssr3451	slr0585	slr1185
slr1329	slr1349	sll0996	slr1281	slr0711	slr2067	slr1703	ssr3451	slr1185	slr2075
sll0593	slr1329	sll0844	slr1281	sll0754	slr2067	sll0078	ssr3451	slr1185	slr2076
sll0220	slr1329	sll1615	slr1281	sll0569	slr2067	slr0557	ssr3451	sll0416	slr1185
sll0899	slr1329	sll0202	slr1281	sll1277	slr2067	slr1884	ssr3451	slr0156	slr1185
slr1329	slr1756	slr0902	slr1281	sll1110	slr2067	slr1031	ssr3451	slr1185	slr1641
sll1085	slr1329	sll1144	slr1281	sll1865	slr2067	slr1793	ssr3451	slr0542	slr1185
sll1931	slr1329	slr0017	slr1281	slr1228	slr2067	slr0633	ssr3451	sll0534	slr1185
sll0083	slr1329	slr1281	slr1424	slr0808	slr2067	slr1278	ssr3451	slr0165	slr1185
slr1329	slr1755	slr1281	slr1423	slr0082	slr2067	slr0783	ssr3451	sll0030	slr1185
sll0057	slr1329	sll2010	slr1281	slr0742	slr2067	sll1198	ssr3451	slr0847	slr1185
sll0017	slr1329	slr0528	slr1281	sll0098	slr2067	sll0204	ssr3451	slr0553	slr1185
sll1185	slr1329	slr1281	slr1351	slr1646	slr2067	sll1750	ssr3451	slr0812	slr1185
slr0839	slr1329	sll0622	slr1281	slr1130	slr2067	sll0420	ssr3451	slr1185	slr1347
sll1876	slr1329	sll0631	slr1281	slr0080	slr2067	slr1256	ssr3451	slr1185	slr1348
sll0900	slr1329	slr0787	slr1281	slr0551	slr2067	sll1639	ssr3451	slr1185	slr1791
slr0608	slr1329	sll1852	slr1281	sll0145	slr2067	slr1899	ssr3451	sll0594	slr1185
slr0652	slr1329	slr1090	slr1281	sll0708	slr2067	sll0643	ssr3451	slr0550	slr1185
slr0084	slr1329	sll0902	slr1281	slr0679	slr2067	slr1844	ssr3451	sll1058	slr1185
sll1893	slr1329	slr0661	slr1281	slr0722	slr2067	sll0459	ssr3451	slr1185	slr1665
slr0500	slr1329	slr0526	slr1281	slr0072	slr2067	sll0865	ssr3451	slr0536	slr1185
slr1329	slr1560	sll1249	slr1281	sll1547	slr2067	slr0733	ssr3451	slr1185	slr1874
sll1988	slr1329	sll0892	slr1281	sll1234	slr2067	sll1459	ssr3451	sll1776	slr1185
slr1289	slr1329	sll0660	slr1281	sll0616	slr2067	sll0905	ssr3451	sll0848	slr1185
sll1556	slr1329	slr1281	slr1779	slr2067	slr2073	sll0402	smr0006	sll0897	slr1185
slr1329	ssl3441	slr0394	slr1281	slr1639	slr2067	sll0728	smr0006	sll1933	slr1185
slr0744	slr1329	slr1281	slr2047	sll1108	slr2067	sll0336	smr0006	slr0965	slr1185
slr0974	slr1329	slr0741	slr1281	sll0362	slr2067	sll1299	smr0006	slr1185	slr1463
slr1329	slr1622	slr1281	slr1510	slr0958	slr2067	smr0006	ssl2084	sll1098	slr1185
sll1196	slr1329	sll1043	slr1281	slr1720	slr2067	sll2001	smr0006	slr0434	slr1185
sll0745	slr1329	slr0400	slr1281	sll0179	slr2067	sll1430	smr0006	sll1261	slr1185
sll1815	slr1329	sll1415	slr1281	sll0454	slr2067	sll0573	smr0006	sll1099	slr1185
sll1059	slr1329	sll1909	slr1281	sll1553	slr2067	slr1898	smr0006	slr0752	slr1185
slr1123	slr1329	sll0373	slr1281	slr0638	slr2067	sll0080	smr0006	slr0952	slr1185
sll1760	slr1329	slr1281	slr2035	slr0220	slr2067	slr1022	smr0006	slr1185	slr1511
sll0469	slr1329	slr1281	slr2132	slr0357	slr2067	sll1883	smr0006	sll1605	slr1185

slr1329	slr1517	slr0922	slr1281	sll1362	slr2067	slr1133	smr0006	sll0990	slr1185
sll0869	slr1329	slr0838	slr1281	slr1550	slr2067	slr0444	smr0006	sll1633	slr1185
slr1329	slr1598	sll1823	slr1281	sll1074	slr2067	slr2130	smr0006	slr0018	slr1185
sll0868	slr1329	sll1056	slr1281	slr0649	slr2067	sll1669	smr0006	sll0567	slr1185
slr0994	slr1329	slr0520	slr1281	sll0495	slr2067	slr0585	smr0006	slr0884	slr1185
sll0379	slr1329	sll0144	slr1281	sll1425	slr2067	slr2075	smr0006	sll1342	slr1185
sll1676	slr1329	sll0467	slr1281	sll0502	slr2067	slr2076	smr0006	slr1185	slr1349
sll0927	slr1329	slr0711	slr1281	slr1703	slr2067	sll0416	smr0006	sll0593	slr1185
sll0996	slr1329	sll0754	slr1281	sll0078	slr2067	slr0156	smr0006	sll0220	slr1185
sll0844	slr1329	sll0569	slr1281	slr0557	slr2067	slr1641	smr0006	sll0899	slr1185
sll1615	slr1329	sll1277	slr1281	slr1884	slr2067	slr0542	smr0006	slr1185	slr1756
sll0202	slr1329	sll1110	slr1281	slr1031	slr2067	sll0534	smr0006	sll1085	slr1185
slr0902	slr1329	sll1865	slr1281	slr1793	slr2067	slr0165	smr0006	sll1931	slr1185
sll1144	slr1329	slr1228	slr1281	slr0633	slr2067	sll0030	smr0006	sll0083	slr1185
slr0017	slr1329	slr0808	slr1281	slr1278	slr2067	slr0847	smr0006	slr1185	slr1755
slr1329	slr1424	slr0082	slr1281	slr0783	slr2067	slr0553	smr0006	sll0057	slr1185
slr1329	slr1423	slr0742	slr1281	sll1198	slr2067	slr0812	smr0006	sll0017	slr1185
sll2010	slr1329	sll0098	slr1281	sll0204	slr2067	slr1347	smr0006	sll1185	slr1185
slr0528	slr1329	slr1281	slr1646	sll1750	slr2067	slr1348	smr0006	slr0839	slr1185
slr1329	slr1351	slr1130	slr1281	sll0420	slr2067	slr1791	smr0006	sll1876	slr1185
sll0622	slr1329	slr0080	slr1281	slr1256	slr2067	sll0594	smr0006	sll0900	slr1185
sll0631	slr1329	slr0551	slr1281	sll1639	slr2067	slr0550	smr0006	slr0608	slr1185
slr0787	slr1329	sll0145	slr1281	slr1899	slr2067	sll1058	smr0006	slr0652	slr1185
sll1852	slr1329	sll0708	slr1281	sll0643	slr2067	slr1665	smr0006	slr0084	slr1185
slr1090	slr1329	slr0679	slr1281	slr1844	slr2067	slr0536	smr0006	sll1893	slr1185
sll0902	slr1329	slr0722	slr1281	sll0459	slr2067	slr1874	smr0006	slr0500	slr1185
slr0661	slr1329	slr0072	slr1281	sll0865	slr2067	sll1776	smr0006	slr1185	slr1560
slr0526	slr1329	sll1547	slr1281	slr0733	slr2067	sll0848	smr0006	sll1988	slr1185
sll1249	slr1329	sll1234	slr1281	sll1459	slr2067	sll0897	smr0006	slr1185	slr1289
sll0892	slr1329	sll0616	slr1281	sll0905	slr2067	sll1933	smr0006	sll1556	slr1185
sll0660	slr1329	slr1281	slr2073	sll0402	slr1986	slr0965	smr0006	slr1185	ssl3441
slr1329	slr1779	slr1281	slr1639	sll0728	slr1986	slr1463	smr0006	slr0744	slr1185
slr0394	slr1329	sll1108	slr1281	sll0336	slr1986	sll1098	smr0006	slr0974	slr1185
slr1329	slr2047	sll0362	slr1281	sll1299	slr1986	slr0434	smr0006	slr1185	slr1622
slr0741	slr1329	slr0958	slr1281	slr1986	ssl2084	sll1261	smr0006	sll1196	slr1185
slr1329	slr1510	slr1281	slr1720	sll2001	slr1986	sll1099	smr0006	sll0745	slr1185
sll1043	slr1329	sll0179	slr1281	sll1430	slr1986	slr0752	smr0006	sll1815	slr1185
slr0400	slr1329	sll0454	slr1281	sll0573	slr1986	slr0952	smr0006	sll1059	slr1185
sll1415	slr1329	sll1553	slr1281	slr1898	slr1986	slr1511	smr0006	slr1123	slr1185
sll1909	slr1329	slr0638	slr1281	sll0080	slr1986	sll1605	smr0006	sll1760	slr1185
sll0373	slr1329	slr0220	slr1281	slr1022	slr1986	sll0990	smr0006	sll0469	slr1185
slr1329	slr2035	slr0357	slr1281	sll1883	slr1986	sll1633	smr0006	slr1185	slr1517
slr1329	slr2132	sll1362	slr1281	slr1133	slr1986	slr0018	smr0006	sll0869	slr1185
slr0922	slr1329	slr1281	slr1550	slr0444	slr1986	sll0567	smr0006	slr1185	slr1598
slr0838	slr1329	sll1074	slr1281	slr1986	slr2130	slr0884	smr0006	sll0868	slr1185
sll1823	slr1329	slr0649	slr1281	sll1669	slr1986	sll1342	smr0006	slr0994	slr1185
sll1056	slr1329	sll0495	slr1281	slr0585	slr1986	slr1349	smr0006	sll0379	slr1185
slr0520	slr1329	sll1425	slr1281	slr1986	slr2075	sll0593	smr0006	sll1676	slr1185
sll0144	slr1329	sll0502	slr1281	slr1986	slr2076	sll0220	smr0006	sll0927	slr1185
sll0467	slr1329	slr1281	slr1703	sll0416	slr1986	sll0899	smr0006	sll0996	slr1185
slr0711	slr1329	sll0078	slr1281	slr0156	slr1986	slr1756	smr0006	sll0844	slr1185

sll0754	slr1329	slr0557	slr1281	slr1641	slr1986	sll1085	smr0006	sll1615	slr1185
sll0569	slr1329	slr1281	slr1884	slr0542	slr1986	sll1931	smr0006	sll0202	slr1185
sll1277	slr1329	slr1031	slr1281	sll0534	slr1986	sll0083	smr0006	slr0902	slr1185
sll1110	slr1329	slr1281	slr1793	slr0165	slr1986	slr1755	smr0006	sll1144	slr1185
sll1865	slr1329	slr0633	slr1281	sll0030	slr1986	sll0057	smr0006	slr0017	slr1185
slr1228	slr1329	slr1278	slr1281	slr0847	slr1986	sll0017	smr0006	slr1185	slr1424
slr0808	slr1329	slr0783	slr1281	slr0553	slr1986	sll1185	smr0006	slr1185	slr1423
slr0082	slr1329	sll1198	slr1281	slr0812	slr1986	slr0839	smr0006	sll2010	slr1185
slr0742	slr1329	sll0204	slr1281	slr1347	slr1986	sll1876	smr0006	slr0528	slr1185
sll0098	slr1329	sll1750	slr1281	slr1348	slr1986	sll0900	smr0006	slr1185	slr1351
slr1329	slr1646	sll0420	slr1281	slr1791	slr1986	slr0608	smr0006	sll0622	slr1185
slr1130	slr1329	slr1256	slr1281	sll0594	slr1986	slr0652	smr0006	sll0631	slr1185
slr0080	slr1329	sll1639	slr1281	slr0550	slr1986	slr0084	smr0006	slr0787	slr1185
slr0551	slr1329	slr1281	slr1899	sll1058	slr1986	sll1893	smr0006	sll1852	slr1185
sll0145	slr1329	sll0643	slr1281	slr1665	slr1986	slr0500	smr0006	slr1090	slr1185
sll0708	slr1329	slr1281	slr1844	slr0536	slr1986	slr1560	smr0006	sll0902	slr1185
slr0679	slr1329	sll0459	slr1281	slr1874	slr1986	sll1988	smr0006	slr0661	slr1185
slr0722	slr1329	sll0865	slr1281	sll1776	slr1986	slr1289	smr0006	slr0526	slr1185
slr0072	slr1329	slr0733	slr1281	sll0848	slr1986	sll1556	smr0006	sll1249	slr1185
sll1547	slr1329	sll1459	slr1281	sll0897	slr1986	smr0006	ssl3441	sll0892	slr1185
sll1234	slr1329	sll0905	slr1281	sll1933	slr1986	slr0744	smr0006	sll0660	slr1185
sll0616	slr1329	sll0402	slr1280	slr0965	slr1986	slr0974	smr0006	slr1185	slr1779
slr1329	slr2073	sll0728	slr1280	slr1463	slr1986	slr1622	smr0006	slr0394	slr1185
slr1329	slr1639	sll0336	slr1280	sll1098	slr1986	sll1196	smr0006	slr1185	slr2047
sll1108	slr1329	sll1299	slr1280	slr0434	slr1986	sll0745	smr0006	slr0741	slr1185
sll0362	slr1329	slr1280	ssl2084	sll1261	slr1986	sll1815	smr0006	slr1185	slr1510
slr0958	slr1329	sll2001	slr1280	sll1099	slr1986	sll1059	smr0006	sll1043	slr1185
slr1329	slr1720	sll1430	slr1280	slr0752	slr1986	slr1123	smr0006	slr0400	slr1185
sll0179	slr1329	sll0573	slr1280	slr0952	slr1986	sll1760	smr0006	sll1415	slr1185
sll0454	slr1329	slr1280	slr1898	slr1511	slr1986	sll0469	smr0006	sll1909	slr1185
sll1553	slr1329	sll0080	slr1280	sll1605	slr1986	slr1517	smr0006	sll0373	slr1185
slr0638	slr1329	slr1022	slr1280	sll0990	slr1986	sll0869	smr0006	slr1185	slr2035
slr0220	slr1329	sll1883	slr1280	sll1633	slr1986	slr1598	smr0006	slr1185	slr2132
slr0357	slr1329	slr1133	slr1280	slr0018	slr1986	sll0868	smr0006	slr0922	slr1185
sll1362	slr1329	slr0444	slr1280	sll0567	slr1986	slr0994	smr0006	slr0838	slr1185
slr1329	slr1550	slr1280	slr2130	slr0884	slr1986	sll0379	smr0006	sll1823	slr1185
sll1074	slr1329	sll1669	slr1280	sll1342	slr1986	sll1676	smr0006	sll1056	slr1185
slr0649	slr1329	slr0585	slr1280	slr1349	slr1986	sll0927	smr0006	slr0520	slr1185
sll0495	slr1329	slr1280	slr2075	sll0593	slr1986	sll0996	smr0006	sll0144	slr1185
sll1425	slr1329	slr1280	slr2076	sll0220	slr1986	sll0844	smr0006	sll0467	slr1185
sll0502	slr1329	sll0416	slr1280	sll0899	slr1986	sll1615	smr0006	slr0711	slr1185
slr1329	slr1703	slr0156	slr1280	slr1756	slr1986	sll0202	smr0006	sll0754	slr1185
sll0078	slr1329	slr1280	slr1641	sll1085	slr1986	slr0902	smr0006	sll0569	slr1185
slr0557	slr1329	slr0542	slr1280	sll1931	slr1986	sll1144	smr0006	sll1277	slr1185
slr1329	slr1884	sll0534	slr1280	sll0083	slr1986	slr0017	smr0006	sll1110	slr1185
slr1031	slr1329	slr0165	slr1280	slr1755	slr1986	slr1424	smr0006	sll1865	slr1185
slr1329	slr1793	sll0030	slr1280	sll0057	slr1986	slr1423	smr0006	slr1185	slr1228
slr0633	slr1329	slr0847	slr1280	sll0017	slr1986	sll2010	smr0006	slr0808	slr1185
slr1278	slr1329	slr0553	slr1280	sll1185	slr1986	slr0528	smr0006	slr0082	slr1185
slr0783	slr1329	slr0812	slr1280	slr0839	slr1986	slr1351	smr0006	slr0742	slr1185
sll1198	slr1329	slr1280	slr1347	sll1876	slr1986	sll0622	smr0006	sll0098	slr1185

sll0204	slr1329	slr1280	slr1348	sll0900	slr1986	sll0631	smr0006	slr1185	slr1646
sll1750	slr1329	slr1280	slr1791	slr0608	slr1986	slr0787	smr0006	slr1130	slr1185
sll0420	slr1329	sll0594	slr1280	slr0652	slr1986	sll1852	smr0006	slr0080	slr1185
slr1256	slr1329	slr0550	slr1280	slr0084	slr1986	slr1090	smr0006	slr0551	slr1185
sll1639	slr1329	sll1058	slr1280	sll1893	slr1986	sll0902	smr0006	sll0145	slr1185
slr1329	slr1899	slr1280	slr1665	slr0500	slr1986	slr0661	smr0006	sll0708	slr1185
sll0643	slr1329	slr0536	slr1280	slr1560	slr1986	slr0526	smr0006	slr0679	slr1185
slr1329	slr1844	slr1280	slr1874	sll1988	slr1986	sll1249	smr0006	slr0722	slr1185
sll0459	slr1329	sll1776	slr1280	slr1289	slr1986	sll0892	smr0006	slr0072	slr1185
sll0865	slr1329	sll0848	slr1280	sll1556	slr1986	sll0660	smr0006	sll1547	slr1185
slr0733	slr1329	sll0897	slr1280	slr1986	ssl3441	slr1779	smr0006	sll1234	slr1185
sll1459	slr1329	sll1933	slr1280	slr0744	slr1986	slr0394	smr0006	sll0616	slr1185
sll0905	slr1329	slr0965	slr1280	slr0974	slr1986	slr2047	smr0006	slr1185	slr2073
sll0402	sll1325	slr1280	slr1463	slr1622	slr1986	slr0741	smr0006	slr1185	slr1639
sll0728	sll1325	sll1098	slr1280	sll1196	slr1986	slr1510	smr0006	sll1108	slr1185
sll0336	sll1325	slr0434	slr1280	sll0745	slr1986	sll1043	smr0006	sll0362	slr1185
sll1299	sll1325	sll1261	slr1280	sll1815	slr1986	slr0400	smr0006	slr0958	slr1185
sll1325	ssl2084	sll1099	slr1280	sll1059	slr1986	sll1415	smr0006	slr1185	slr1720
sll1325	sll2001	slr0752	slr1280	slr1123	slr1986	sll1909	smr0006	sll0179	slr1185
sll1325	sll1430	slr0952	slr1280	sll1760	slr1986	sll0373	smr0006	sll0454	slr1185
sll0573	sll1325	slr1280	slr1511	sll0469	slr1986	slr2035	smr0006	sll1553	slr1185
sll1325	slr1898	sll1605	slr1280	slr1517	slr1986	slr2132	smr0006	slr0638	slr1185
sll0080	sll1325	sll0990	slr1280	sll0869	slr1986	slr0922	smr0006	slr0220	slr1185
sll1325	slr1022	sll1633	slr1280	slr1598	slr1986	slr0838	smr0006	slr0357	slr1185
sll1325	sll1883	slr0018	slr1280	sll0868	slr1986	sll1823	smr0006	sll1362	slr1185
sll1325	slr1133	sll0567	slr1280	slr0994	slr1986	sll1056	smr0006	slr1185	slr1550
sll1325	slr0444	slr0884	slr1280	sll0379	slr1986	slr0520	smr0006	sll1074	slr1185
sll1325	slr2130	sll1342	slr1280	sll1676	slr1986	sll0144	smr0006	slr0649	slr1185
sll1325	sll1669	slr1280	slr1349	sll0927	slr1986	sll0467	smr0006	sll0495	slr1185
sll1325	slr0585	sll0593	slr1280	sll0996	slr1986	slr0711	smr0006	sll1425	slr1185
sll1325	slr2075	sll0220	slr1280	sll0844	slr1986	sll0754	smr0006	sll0502	slr1185
sll1325	slr2076	sll0899	slr1280	sll1615	slr1986	sll0569	smr0006	slr1185	slr1703
sll0416	sll1325	slr1280	slr1756	sll0202	slr1986	sll1277	smr0006	sll0078	slr1185
sll1325	slr0156	sll1085	slr1280	slr0902	slr1986	sll1110	smr0006	slr0557	slr1185
sll1325	slr1641	sll1931	slr1280	sll1144	slr1986	sll1865	smr0006	slr1185	slr1884
sll1325	slr0542	sll0083	slr1280	slr0017	slr1986	slr1228	smr0006	slr1031	slr1185
sll0534	sll1325	slr1280	slr1755	slr1424	slr1986	slr0808	smr0006	slr1185	slr1793
sll1325	slr0165	sll0057	slr1280	slr1423	slr1986	slr0082	smr0006	slr0633	slr1185
sll0030	sll1325	sll0017	slr1280	sll2010	slr1986	slr0742	smr0006	slr1185	slr1278
sll1325	slr0847	sll1185	slr1280	slr0528	slr1986	sll0098	smr0006	slr0783	slr1185
sll1325	slr0553	slr0839	slr1280	slr1351	slr1986	slr1646	smr0006	sll1198	slr1185
sll1325	slr0812	sll1876	slr1280	sll0622	slr1986	slr1130	smr0006	sll0204	slr1185
sll1325	slr1347	sll0900	slr1280	sll0631	slr1986	slr0080	smr0006	sll1750	slr1185
sll1325	slr1348	slr0608	slr1280	slr0787	slr1986	slr0551	smr0006	sll0420	slr1185
sll1325	slr1791	slr0652	slr1280	sll1852	slr1986	sll0145	smr0006	slr1185	slr1256
sll0594	sll1325	slr0084	slr1280	slr1090	slr1986	sll0708	smr0006	sll1639	slr1185
sll1325	slr0550	sll1893	slr1280	sll0902	slr1986	slr0679	smr0006	slr1185	slr1899
sll1058	sll1325	slr0500	slr1280	slr0661	slr1986	slr0722	smr0006	sll0643	slr1185
sll1325	slr1665	slr1280	slr1560	slr0526	slr1986	slr0072	smr0006	slr1185	slr1844
sll1325	slr0536	sll1988	slr1280	sll1249	slr1986	sll1547	smr0006	sll0459	slr1185
sll1325	slr1874	slr1280	slr1289	sll0892	slr1986	sll1234	smr0006	sll0865	slr1185

sll1325	sll1776	sll1556	slr1280	sll0660	slr1986	sll0616	smr0006	slr0733	slr1185
sll0848	sll1325	slr1280	ssl3441	slr1779	slr1986	slr2073	smr0006	sll1459	slr1185
sll0897	sll1325	slr0744	slr1280	slr0394	slr1986	slr1639	smr0006	sll0905	slr1185
sll1325	sll1933	slr0974	slr1280	slr1986	slr2047	sll1108	smr0006	sll0402	sll1316
sll1325	slr0965	slr1280	slr1622	slr0741	slr1986	sll0362	smr0006	sll0728	sll1316
sll1325	slr1463	sll1196	slr1280	slr1510	slr1986	slr0958	smr0006	sll0336	sll1316
sll1098	sll1325	sll0745	slr1280	sll1043	slr1986	slr1720	smr0006	sll1299	sll1316
sll1325	slr0434	sll1815	slr1280	slr0400	slr1986	sll0179	smr0006	sll1316	ssl2084
sll1261	sll1325	sll1059	slr1280	sll1415	slr1986	sll0454	smr0006	sll1316	sll2001
sll1099	sll1325	slr1123	slr1280	sll1909	slr1986	sll1553	smr0006	sll1316	sll1430
sll1325	slr0752	sll1760	slr1280	sll0373	slr1986	slr0638	smr0006	sll0573	sll1316
sll1325	slr0952	sll0469	slr1280	slr1986	slr2035	slr0220	smr0006	sll1316	slr1898
sll1325	slr1511	slr1280	slr1517	slr1986	slr2132	slr0357	smr0006	sll0080	sll1316
sll1325	sll1605	sll0869	slr1280	slr0922	slr1986	sll1362	smr0006	sll1316	slr1022
sll0990	sll1325	slr1280	slr1598	slr0838	slr1986	slr1550	smr0006	sll1316	sll1883
sll1325	sll1633	sll0868	slr1280	sll1823	slr1986	sll1074	smr0006	sll1316	slr1133
sll1325	slr0018	slr0994	slr1280	sll1056	slr1986	slr0649	smr0006	sll1316	slr0444
sll0567	sll1325	sll0379	slr1280	slr0520	slr1986	sll0495	smr0006	sll1316	slr2130
sll1325	slr0884	sll1676	slr1280	sll0144	slr1986	sll1425	smr0006	sll1316	sll1669
sll1325	sll1342	sll0927	slr1280	sll0467	slr1986	sll0502	smr0006	sll1316	slr0585
sll1325	slr1349	sll0996	slr1280	slr0711	slr1986	slr1703	smr0006	sll1316	slr2075
sll0593	sll1325	sll0844	slr1280	sll0754	slr1986	sll0078	smr0006	sll1316	slr2076
sll0220	sll1325	sll1615	slr1280	sll0569	slr1986	slr0557	smr0006	sll0416	sll1316
sll0899	sll1325	sll0202	slr1280	sll1277	slr1986	slr1884	smr0006	sll1316	slr0156
sll1325	slr1756	slr0902	slr1280	sll1110	slr1986	slr1031	smr0006	sll1316	slr1641
sll1085	sll1325	sll1144	slr1280	sll1865	slr1986	slr1793	smr0006	sll1316	slr0542
sll1325	sll1931	slr0017	slr1280	slr1228	slr1986	slr0633	smr0006	sll0534	sll1316
sll0083	sll1325	slr1280	slr1424	slr0808	slr1986	slr1278	smr0006	sll1316	slr0165
sll1325	slr1755	slr1280	slr1423	slr0082	slr1986	slr0783	smr0006	sll0030	sll1316
sll0057	sll1325	sll2010	slr1280	slr0742	slr1986	sll1198	smr0006	sll1316	slr0847
sll0017	sll1325	slr0528	slr1280	sll0098	slr1986	sll0204	smr0006	sll1316	slr0553
sll1185	sll1325	slr1280	slr1351	slr1646	slr1986	sll1750	smr0006	sll1316	slr0812
sll1325	slr0839	sll0622	slr1280	slr1130	slr1986	sll0420	smr0006	sll1316	slr1347
sll1325	sll1876	sll0631	slr1280	slr0080	slr1986	slr1256	smr0006	sll1316	slr1348
sll0900	sll1325	slr0787	slr1280	slr0551	slr1986	sll1639	smr0006	sll1316	slr1791
sll1325	slr0608	sll1852	slr1280	sll0145	slr1986	slr1899	smr0006	sll0594	sll1316
sll1325	slr0652	slr1090	slr1280	sll0708	slr1986	sll0643	smr0006	sll1316	slr0550
sll1325	slr0084	sll0902	slr1280	slr0679	slr1986	slr1844	smr0006	sll1058	sll1316
sll1325	sll1893	slr0661	slr1280	slr0722	slr1986	sll0459	smr0006	sll1316	slr1665
sll1325	slr0500	slr0526	slr1280	slr0072	slr1986	sll0865	smr0006	sll1316	slr0536
sll1325	slr1560	sll1249	slr1280	sll1547	slr1986	slr0733	smr0006	sll1316	slr1874
sll1325	sll1988	sll0892	slr1280	sll1234	slr1986	sll1459	smr0006	sll1316	sll1776
sll1325	slr1289	sll0660	slr1280	sll0616	slr1986	sll0905	smr0006	sll0848	sll1316
sll1325	sll1556	slr1280	slr1779	slr1986	slr2073	sll0402	ssl2598	sll0897	sll1316
sll1325	ssl3441	slr0394	slr1280	slr1639	slr1986	sll0728	ssl2598	sll1316	sll1933
sll1325	slr0744	slr1280	slr2047	sll1108	slr1986	sll0336	ssl2598	sll1316	slr0965
sll1325	slr0974	slr0741	slr1280	sll0362	slr1986	sll1299	ssl2598	sll1316	slr1463
sll1325	slr1622	slr1280	slr1510	slr0958	slr1986	ssl2084	ssl2598	sll1098	sll1316
sll1196	sll1325	sll1043	slr1280	slr1720	slr1986	sll2001	ssl2598	sll1316	slr0434
sll0745	sll1325	slr0400	slr1280	sll0179	slr1986	sll1430	ssl2598	sll1261	sll1316
sll1325	sll1815	sll1415	slr1280	sll0454	slr1986	sll0573	ssl2598	sll1099	sll1316

sll1059	sll1325	sll1909	slr1280	sll1553	slr1986	slr1898	ssl2598	sll1316	slr0752
sll1325	slr1123	sll0373	slr1280	slr0638	slr1986	sll0080	ssl2598	sll1316	slr0952
sll1325	sll1760	slr1280	slr2035	slr0220	slr1986	slr1022	ssl2598	sll1316	slr1511
sll0469	sll1325	slr1280	slr2132	slr0357	slr1986	sll1883	ssl2598	sll1316	sll1605
sll1325	slr1517	slr0922	slr1280	sll1362	slr1986	slr1133	ssl2598	sll0990	sll1316
sll0869	sll1325	slr0838	slr1280	slr1550	slr1986	slr0444	ssl2598	sll1316	sll1633
sll1325	slr1598	sll1823	slr1280	sll1074	slr1986	slr2130	ssl2598	sll1316	slr0018
sll0868	sll1325	sll1056	slr1280	slr0649	slr1986	sll1669	ssl2598	sll0567	sll1316
sll1325	slr0994	slr0520	slr1280	sll0495	slr1986	slr0585	ssl2598	sll1316	slr0884
sll0379	sll1325	sll0144	slr1280	sll1425	slr1986	slr2075	ssl2598	sll1316	sll1342
sll1325	sll1676	sll0467	slr1280	sll0502	slr1986	slr2076	ssl2598	sll1316	slr1349
sll0927	sll1325	slr0711	slr1280	slr1703	slr1986	sll0416	ssl2598	sll0593	sll1316
sll0996	sll1325	sll0754	slr1280	sll0078	slr1986	slr0156	ssl2598	sll0220	sll1316
sll0844	sll1325	sll0569	slr1280	slr0557	slr1986	slr1641	ssl2598	sll0899	sll1316
sll1325	sll1615	sll1277	slr1280	slr1884	slr1986	slr0542	ssl2598	sll1316	slr1756
sll0202	sll1325	sll1110	slr1280	slr1031	slr1986	sll0534	ssl2598	sll1085	sll1316
sll1325	slr0902	sll1865	slr1280	slr1793	slr1986	slr0165	ssl2598	sll1316	sll1931
sll1144	sll1325	slr1228	slr1280	slr0633	slr1986	sll0030	ssl2598	sll0083	sll1316
sll1325	slr0017	slr0808	slr1280	slr1278	slr1986	slr0847	ssl2598	sll1316	slr1755
sll1325	slr1424	slr0082	slr1280	slr0783	slr1986	slr0553	ssl2598	sll0057	sll1316
sll1325	slr1423	slr0742	slr1280	sll1198	slr1986	slr0812	ssl2598	sll0017	sll1316
sll1325	sll2010	sll0098	slr1280	sll0204	slr1986	slr1347	ssl2598	sll1185	sll1316
sll1325	slr0528	slr1280	slr1646	sll1750	slr1986	slr1348	ssl2598	sll1316	slr0839
sll1325	slr1351	slr1130	slr1280	sll0420	slr1986	slr1791	ssl2598	sll1316	sll1876
sll0622	sll1325	slr0080	slr1280	slr1256	slr1986	sll0594	ssl2598	sll0900	sll1316
sll0631	sll1325	slr0551	slr1280	sll1639	slr1986	slr0550	ssl2598	sll1316	slr0608
sll1325	slr0787	sll0145	slr1280	slr1899	slr1986	sll1058	ssl2598	sll1316	slr0652
sll1325	sll1852	sll0708	slr1280	sll0643	slr1986	slr1665	ssl2598	sll1316	slr0084
sll1325	slr1090	slr0679	slr1280	slr1844	slr1986	slr0536	ssl2598	sll1316	sll1893
sll0902	sll1325	slr0722	slr1280	sll0459	slr1986	slr1874	ssl2598	sll1316	slr0500
sll1325	slr0661	slr0072	slr1280	sll0865	slr1986	sll1776	ssl2598	sll1316	slr1560
sll1325	slr0526	sll1547	slr1280	slr0733	slr1986	sll0848	ssl2598	sll1316	sll1988
sll1249	sll1325	sll1234	slr1280	sll1459	slr1986	sll0897	ssl2598	sll1316	slr1289
sll0892	sll1325	sll0616	slr1280	sll0905	slr1986	sll1933	ssl2598	sll1316	sll1556
sll0660	sll1325	slr1280	slr2073	sll0402	sll1578	slr0965	ssl2598	sll1316	ssl3441
sll1325	slr1779	slr1280	slr1639	sll0728	sll1578	slr1463	ssl2598	sll1316	slr0744
sll1325	slr0394	sll1108	slr1280	sll0336	sll1578	sll1098	ssl2598	sll1316	slr0974
sll1325	slr2047	sll0362	slr1280	sll1299	sll1578	slr0434	ssl2598	sll1316	slr1622
sll1325	slr0741	slr0958	slr1280	sll1578	ssl2084	sll1261	ssl2598	sll1196	sll1316
sll1325	slr1510	slr1280	slr1720	sll1578	sll2001	sll1099	ssl2598	sll0745	sll1316
sll1043	sll1325	sll0179	slr1280	sll1430	sll1578	slr0752	ssl2598	sll1316	sll1815
sll1325	slr0400	sll0454	slr1280	sll0573	sll1578	slr0952	ssl2598	sll1059	sll1316
sll1325	sll1415	sll1553	slr1280	sll1578	slr1898	slr1511	ssl2598	sll1316	slr1123
sll1325	sll1909	slr0638	slr1280	sll0080	sll1578	sll1605	ssl2598	sll1316	sll1760
sll0373	sll1325	slr0220	slr1280	sll1578	slr1022	sll0990	ssl2598	sll0469	sll1316
sll1325	slr2035	slr0357	slr1280	sll1578	sll1883	sll1633	ssl2598	sll1316	slr1517
sll1325	slr2132	sll1362	slr1280	sll1578	slr1133	slr0018	ssl2598	sll0869	sll1316
sll1325	slr0922	slr1280	slr1550	sll1578	slr0444	sll0567	ssl2598	sll1316	slr1598
sll1325	slr0838	sll1074	slr1280	sll1578	slr2130	slr0884	ssl2598	sll0868	sll1316
sll1325	sll1823	slr0649	slr1280	sll1578	sll1669	sll1342	ssl2598	sll1316	slr0994
sll1056	sll1325	sll0495	slr1280	sll1578	slr0585	slr1349	ssl2598	sll0379	sll1316

sll1325	slr0520	sll1425	slr1280	sll1578	slr2075	sll0593	ssl2598	sll1316	sll1676
sll0144	sll1325	sll0502	slr1280	sll1578	slr2076	sll0220	ssl2598	sll0927	sll1316
sll0467	sll1325	slr1280	slr1703	sll0416	sll1578	sll0899	ssl2598	sll0996	sll1316
sll1325	slr0711	sll0078	slr1280	sll1578	slr0156	slr1756	ssl2598	sll0844	sll1316
sll0754	sll1325	slr0557	slr1280	sll1578	slr1641	sll1085	ssl2598	sll1316	sll1615
sll0569	sll1325	slr1280	slr1884	sll1578	slr0542	sll1931	ssl2598	sll0202	sll1316
sll1277	sll1325	slr1031	slr1280	sll0534	sll1578	sll0083	ssl2598	sll1316	slr0902
sll1110	sll1325	slr1280	slr1793	sll1578	slr0165	slr1755	ssl2598	sll1144	sll1316
sll1325	sll1865	slr0633	slr1280	sll0030	sll1578	sll0057	ssl2598	sll1316	slr0017
sll1325	slr1228	slr1278	slr1280	sll1578	slr0847	sll0017	ssl2598	sll1316	slr1424
sll1325	slr0808	slr0783	slr1280	sll1578	slr0553	sll1185	ssl2598	sll1316	slr1423
sll1325	slr0082	sll1198	slr1280	sll1578	slr0812	slr0839	ssl2598	sll1316	sll2010
sll1325	slr0742	sll0204	slr1280	sll1578	slr1347	sll1876	ssl2598	sll1316	slr0528
sll0098	sll1325	sll1750	slr1280	sll1578	slr1348	sll0900	ssl2598	sll1316	slr1351
sll1325	slr1646	sll0420	slr1280	sll1578	slr1791	slr0608	ssl2598	sll0622	sll1316
sll1325	slr1130	slr1256	slr1280	sll0594	sll1578	slr0652	ssl2598	sll0631	sll1316
sll1325	slr0080	sll1639	slr1280	sll1578	slr0550	slr0084	ssl2598	sll1316	slr0787
sll1325	slr0551	slr1280	slr1899	sll1058	sll1578	sll1893	ssl2598	sll1316	sll1852
sll0145	sll1325	sll0643	slr1280	sll1578	slr1665	slr0500	ssl2598	sll1316	slr1090
sll0708	sll1325	slr1280	slr1844	sll1578	slr0536	slr1560	ssl2598	sll0902	sll1316
sll1325	slr0679	sll0459	slr1280	sll1578	slr1874	sll1988	ssl2598	sll1316	slr0661
sll1325	slr0722	sll0865	slr1280	sll1578	sll1776	slr1289	ssl2598	sll1316	slr0526
sll1325	slr0072	slr0733	slr1280	sll0848	sll1578	sll1556	ssl2598	sll1249	sll1316
sll1325	sll1547	sll1459	slr1280	sll0897	sll1578	ssl2598	ssl3441	sll0892	sll1316
sll1234	sll1325	sll0905	slr1280	sll1578	sll1933	slr0744	ssl2598	sll0660	sll1316
sll0616	sll1325	ndhK2	sll0402	sll1578	slr0965	slr0974	ssl2598	sll1316	slr1779
sll1325	slr2073	ndhK2	sll0728	sll1578	slr1463	slr1622	ssl2598	sll1316	slr0394
sll1325	slr1639	ndhK2	sll0336	sll1098	sll1578	sll1196	ssl2598	sll1316	slr2047
sll1108	sll1325	ndhK2	sll1299	sll1578	slr0434	sll0745	ssl2598	sll1316	slr0741
sll0362	sll1325	ndhK2	ssl2084	sll1261	sll1578	sll1815	ssl2598	sll1316	slr1510
sll1325	slr0958	ndhK2	sll2001	sll1099	sll1578	sll1059	ssl2598	sll1043	sll1316
sll1325	slr1720	ndhK2	sll1430	sll1578	slr0752	slr1123	ssl2598	sll1316	slr0400
sll0179	sll1325	ndhK2	sll0573	sll1578	slr0952	sll1760	ssl2598	sll1316	sll1415
sll0454	sll1325	ndhK2	slr1898	sll1578	slr1511	sll0469	ssl2598	sll1316	sll1909
sll1325	sll1553	ndhK2	sll0080	sll1578	sll1605	slr1517	ssl2598	sll0373	sll1316
sll1325	slr0638	ndhK2	slr1022	sll0990	sll1578	sll0869	ssl2598	sll1316	slr2035
sll1325	slr0220	ndhK2	sll1883	sll1578	sll1633	slr1598	ssl2598	sll1316	slr2132
sll1325	slr0357	ndhK2	slr1133	sll1578	slr0018	sll0868	ssl2598	sll1316	slr0922
sll1325	sll1362	ndhK2	slr0444	sll0567	sll1578	slr0994	ssl2598	sll1316	slr0838
sll1325	slr1550	ndhK2	slr2130	sll1578	slr0884	sll0379	ssl2598	sll1316	sll1823
sll1074	sll1325	ndhK2	sll1669	sll1342	sll1578	sll1676	ssl2598	sll1056	sll1316
sll1325	slr0649	ndhK2	slr0585	sll1578	slr1349	sll0927	ssl2598	sll1316	slr0520
sll0495	sll1325	ndhK2	slr2075	sll0593	sll1578	sll0996	ssl2598	sll0144	sll1316
sll1325	sll1425	ndhK2	slr2076	sll0220	sll1578	sll0844	ssl2598	sll0467	sll1316
sll0502	sll1325	ndhK2	sll0416	sll0899	sll1578	sll1615	ssl2598	sll1316	slr0711
sll1325	slr1703	ndhK2	slr0156	sll1578	slr1756	sll0202	ssl2598	sll0754	sll1316
sll0078	sll1325	ndhK2	slr1641	sll1085	sll1578	slr0902	ssl2598	sll0569	sll1316
sll1325	slr0557	ndhK2	slr0542	sll1578	sll1931	sll1144	ssl2598	sll1277	sll1316
sll1325	slr1884	ndhK2	sll0534	sll0083	sll1578	slr0017	ssl2598	sll1110	sll1316
sll1325	slr1031	ndhK2	slr0165	sll1578	slr1755	slr1424	ssl2598	sll1316	sll1865
sll1325	slr1793	ndhK2	sll0030	sll0057	sll1578	slr1423	ssl2598	sll1316	slr1228

sll1325	slr0633	ndhK2	slr0847	sll0017	sll1578	sll2010	ssl2598	sll1316	slr0808
sll1325	slr1278	ndhK2	slr0553	sll1185	sll1578	slr0528	ssl2598	sll1316	slr0082
sll1325	slr0783	ndhK2	slr0812	sll1578	slr0839	slr1351	ssl2598	sll1316	slr0742
sll1198	sll1325	ndhK2	slr1347	sll1578	sll1876	sll0622	ssl2598	sll0098	sll1316
sll0204	sll1325	ndhK2	slr1348	sll0900	sll1578	sll0631	ssl2598	sll1316	slr1646
sll1325	sll1750	ndhK2	slr1791	sll1578	slr0608	slr0787	ssl2598	sll1316	slr1130
sll0420	sll1325	ndhK2	sll0594	sll1578	slr0652	sll1852	ssl2598	sll1316	slr0080
sll1325	slr1256	ndhK2	slr0550	sll1578	slr0084	slr1090	ssl2598	sll1316	slr0551
sll1325	sll1639	ndhK2	sll1058	sll1578	sll1893	sll0902	ssl2598	sll0145	sll1316
sll1325	slr1899	ndhK2	slr1665	sll1578	slr0500	slr0661	ssl2598	sll0708	sll1316
sll0643	sll1325	ndhK2	slr0536	sll1578	slr1560	slr0526	ssl2598	sll1316	slr0679
sll1325	slr1844	ndhK2	slr1874	sll1578	sll1988	sll1249	ssl2598	sll1316	slr0722
sll0459	sll1325	ndhK2	sll1776	sll1578	slr1289	sll0892	ssl2598	sll1316	slr0072
sll0865	sll1325	ndhK2	sll0848	sll1556	sll1578	sll0660	ssl2598	sll1316	sll1547
sll1325	slr0733	ndhK2	sll0897	sll1578	ssl3441	slr1779	ssl2598	sll1234	sll1316
sll1325	sll1459	ndhK2	sll1933	sll1578	slr0744	slr0394	ssl2598	sll0616	sll1316
sll0905	sll1325	ndhK2	slr0965	sll1578	slr0974	slr2047	ssl2598	sll1316	slr2073
sll0402	slr1330	ndhK2	slr1463	sll1578	slr1622	slr0741	ssl2598	sll1316	slr1639
sll0728	slr1330	ndhK2	sll1098	sll1196	sll1578	slr1510	ssl2598	sll1108	sll1316
sll0336	slr1330	ndhK2	slr0434	sll0745	sll1578	sll1043	ssl2598	sll0362	sll1316
sll1299	slr1330	ndhK2	sll1261	sll1578	sll1815	slr0400	ssl2598	sll1316	slr0958
slr1330	ssl2084	ndhK2	sll1099	sll1059	sll1578	sll1415	ssl2598	sll1316	slr1720
sll2001	slr1330	ndhK2	slr0752	sll1578	slr1123	sll1909	ssl2598	sll0179	sll1316
sll1430	slr1330	ndhK2	slr0952	sll1578	sll1760	sll0373	ssl2598	sll0454	sll1316
sll0573	slr1330	ndhK2	slr1511	sll0469	sll1578	slr2035	ssl2598	sll1316	sll1553
slr1330	slr1898	ndhK2	sll1605	sll1578	slr1517	slr2132	ssl2598	sll1316	slr0638
sll0080	slr1330	ndhK2	sll0990	sll0869	sll1578	slr0922	ssl2598	sll1316	slr0220
slr1022	slr1330	ndhK2	sll1633	sll1578	slr1598	slr0838	ssl2598	sll1316	slr0357
sll1883	slr1330	ndhK2	slr0018	sll0868	sll1578	sll1823	ssl2598	sll1316	sll1362
slr1133	slr1330	ndhK2	sll0567	sll1578	slr0994	sll1056	ssl2598	sll1316	slr1550
slr0444	slr1330	ndhK2	slr0884	sll0379	sll1578	slr0520	ssl2598	sll1074	sll1316
slr1330	slr2130	ndhK2	sll1342	sll1578	sll1676	sll0144	ssl2598	sll1316	slr0649
sll1669	slr1330	ndhK2	slr1349	sll0927	sll1578	sll0467	ssl2598	sll0495	sll1316
slr0585	slr1330	ndhK2	sll0593	sll0996	sll1578	slr0711	ssl2598	sll1316	sll1425
slr1330	slr2075	ndhK2	sll0220	sll0844	sll1578	sll0754	ssl2598	sll0502	sll1316
slr1330	slr2076	ndhK2	sll0899	sll1578	sll1615	sll0569	ssl2598	sll1316	slr1703
sll0416	slr1330	ndhK2	slr1756	sll0202	sll1578	sll1277	ssl2598	sll0078	sll1316
slr0156	slr1330	ndhK2	sll1085	sll1578	slr0902	sll1110	ssl2598	sll1316	slr0557
slr1330	slr1641	ndhK2	sll1931	sll1144	sll1578	sll1865	ssl2598	sll1316	slr1884
slr0542	slr1330	ndhK2	sll0083	sll1578	slr0017	slr1228	ssl2598	sll1316	slr1031
sll0534	slr1330	ndhK2	slr1755	sll1578	slr1424	slr0808	ssl2598	sll1316	slr1793
slr0165	slr1330	ndhK2	sll0057	sll1578	slr1423	slr0082	ssl2598	sll1316	slr0633
sll0030	slr1330	ndhK2	sll0017	sll1578	sll2010	slr0742	ssl2598	sll1316	slr1278
slr0847	slr1330	ndhK2	sll1185	sll1578	slr0528	sll0098	ssl2598	sll1316	slr0783
slr0553	slr1330	ndhK2	slr0839	sll1578	slr1351	slr1646	ssl2598	sll1198	sll1316
slr0812	slr1330	ndhK2	sll1876	sll0622	sll1578	slr1130	ssl2598	sll0204	sll1316
slr1330	slr1347	ndhK2	sll0900	sll0631	sll1578	slr0080	ssl2598	sll1316	sll1750
slr1330	slr1348	ndhK2	slr0608	sll1578	slr0787	slr0551	ssl2598	sll0420	sll1316
slr1330	slr1791	ndhK2	slr0652	sll1578	sll1852	sll0145	ssl2598	sll1316	slr1256
sll0594	slr1330	ndhK2	slr0084	sll1578	slr1090	sll0708	ssl2598	sll1316	sll1639
slr0550	slr1330	ndhK2	sll1893	sll0902	sll1578	slr0679	ssl2598	sll1316	slr1899

sll1058	slr1330	ndhK2	slr0500	sll1578	slr0661	slr0722	ssl2598	sll0643	sll1316
slr1330	slr1665	ndhK2	slr1560	sll1578	slr0526	slr0072	ssl2598	sll1316	slr1844
slr0536	slr1330	ndhK2	sll1988	sll1249	sll1578	sll1547	ssl2598	sll0459	sll1316
slr1330	slr1874	ndhK2	slr1289	sll0892	sll1578	sll1234	ssl2598	sll0865	sll1316
sll1776	slr1330	ndhK2	sll1556	sll0660	sll1578	sll0616	ssl2598	sll1316	slr0733
sll0848	slr1330	ndhK2	ssl3441	sll1578	slr1779	slr2073	ssl2598	sll1316	sll1459
sll0897	slr1330	ndhK2	slr0744	sll1578	slr0394	slr1639	ssl2598	sll0905	sll1316
sll1933	slr1330	ndhK2	slr0974	sll1578	slr2047	sll1108	ssl2598	sll0402	slr1513
slr0965	slr1330	ndhK2	slr1622	sll1578	slr0741	sll0362	ssl2598	sll0728	slr1513
slr1330	slr1463	ndhK2	sll1196	sll1578	slr1510	slr0958	ssl2598	sll0336	slr1513
sll1098	slr1330	ndhK2	sll0745	sll1043	sll1578	slr1720	ssl2598	sll1299	slr1513
slr0434	slr1330	ndhK2	sll1815	sll1578	slr0400	sll0179	ssl2598	slr1513	ssl2084
sll1261	slr1330	ndhK2	sll1059	sll1415	sll1578	sll0454	ssl2598	sll2001	slr1513
sll1099	slr1330	ndhK2	slr1123	sll1578	sll1909	sll1553	ssl2598	sll1430	slr1513
slr0752	slr1330	ndhK2	sll1760	sll0373	sll1578	slr0638	ssl2598	sll0573	slr1513
slr0952	slr1330	ndhK2	sll0469	sll1578	slr2035	slr0220	ssl2598	slr1513	slr1898
slr1330	slr1511	ndhK2	slr1517	sll1578	slr2132	slr0357	ssl2598	sll0080	slr1513
sll1605	slr1330	ndhK2	sll0869	sll1578	slr0922	sll1362	ssl2598	slr1022	slr1513
sll0990	slr1330	ndhK2	slr1598	sll1578	slr0838	slr1550	ssl2598	sll1883	slr1513
sll1633	slr1330	ndhK2	sll0868	sll1578	sll1823	sll1074	ssl2598	slr1133	slr1513
slr0018	slr1330	ndhK2	slr0994	sll1056	sll1578	slr0649	ssl2598	slr0444	slr1513
sll0567	slr1330	ndhK2	sll0379	sll1578	slr0520	sll0495	ssl2598	slr1513	slr2130
slr0884	slr1330	ndhK2	sll1676	sll0144	sll1578	sll1425	ssl2598	sll1669	slr1513
sll1342	slr1330	ndhK2	sll0927	sll0467	sll1578	sll0502	ssl2598	slr0585	slr1513
slr1330	slr1349	ndhK2	sll0996	sll1578	slr0711	slr1703	ssl2598	slr1513	slr2075
sll0593	slr1330	ndhK2	sll0844	sll0754	sll1578	sll0078	ssl2598	slr1513	slr2076
sll0220	slr1330	ndhK2	sll1615	sll0569	sll1578	slr0557	ssl2598	sll0416	slr1513
sll0899	slr1330	ndhK2	sll0202	sll1277	sll1578	slr1884	ssl2598	slr0156	slr1513
slr1330	slr1756	ndhK2	slr0902	sll1110	sll1578	slr1031	ssl2598	slr1513	slr1641
sll1085	slr1330	ndhK2	sll1144	sll1578	sll1865	slr1793	ssl2598	slr0542	slr1513
sll1931	slr1330	ndhK2	slr0017	sll1578	slr1228	slr0633	ssl2598	sll0534	slr1513
sll0083	slr1330	ndhK2	slr1424	sll1578	slr0808	slr1278	ssl2598	slr0165	slr1513
slr1330	slr1755	ndhK2	slr1423	sll1578	slr0082	slr0783	ssl2598	sll0030	slr1513
sll0057	slr1330	ndhK2	sll2010	sll1578	slr0742	sll1198	ssl2598	slr0847	slr1513
sll0017	slr1330	ndhK2	slr0528	sll0098	sll1578	sll0204	ssl2598	slr0553	slr1513
sll1185	slr1330	ndhK2	slr1351	sll1578	slr1646	sll1750	ssl2598	slr0812	slr1513
slr0839	slr1330	ndhK2	sll0622	sll1578	slr1130	sll0420	ssl2598	slr1347	slr1513
sll1876	slr1330	ndhK2	sll0631	sll1578	slr0080	slr1256	ssl2598	slr1348	slr1513
sll0900	slr1330	ndhK2	slr0787	sll1578	slr0551	sll1639	ssl2598	slr1513	slr1791
slr0608	slr1330	ndhK2	sll1852	sll0145	sll1578	slr1899	ssl2598	sll0594	slr1513
slr0652	slr1330	ndhK2	slr1090	sll0708	sll1578	sll0643	ssl2598	slr0550	slr1513
slr0084	slr1330	ndhK2	sll0902	sll1578	slr0679	slr1844	ssl2598	sll1058	slr1513
sll1893	slr1330	ndhK2	slr0661	sll1578	slr0722	sll0459	ssl2598	slr1513	slr1665
slr0500	slr1330	ndhK2	slr0526	sll1578	slr0072	sll0865	ssl2598	slr0536	slr1513
slr1330	slr1560	ndhK2	sll1249	sll1547	sll1578	slr0733	ssl2598	slr1513	slr1874
sll1988	slr1330	ndhK2	sll0892	sll1234	sll1578	sll1459	ssl2598	sll1776	slr1513
slr1289	slr1330	ndhK2	sll0660	sll0616	sll1578	sll0905	ssl2598	sll0848	slr1513
sll1556	slr1330	ndhK2	slr1779	sll1578	slr2073	sll0402	sml0001	sll0897	slr1513
slr1330	ssl3441	ndhK2	slr0394	sll1578	slr1639	sll0728	sml0001	sll1933	slr1513
slr0744	slr1330	ndhK2	slr2047	sll1108	sll1578	sll0336	sml0001	slr0965	slr1513
slr0974	slr1330	ndhK2	slr0741	sll0362	sll1578	sll1299	sml0001	slr1463	slr1513

slr1330	slr1622	ndhK2	slr1510	sll1578	slr0958	sml0001	ssl2084	sll1098	slr1513
sll1196	slr1330	ndhK2	sll1043	sll1578	slr1720	sll2001	sml0001	slr0434	slr1513
sll0745	slr1330	ndhK2	slr0400	sll0179	sll1578	sll1430	sml0001	sll1261	slr1513
sll1815	slr1330	ndhK2	sll1415	sll0454	sll1578	sll0573	sml0001	sll1099	slr1513
sll1059	slr1330	ndhK2	sll1909	sll1553	sll1578	slr1898	sml0001	slr0752	slr1513
slr1123	slr1330	ndhK2	sll0373	sll1578	slr0638	sll0080	sml0001	slr0952	slr1513
sll1760	slr1330	ndhK2	slr2035	sll1578	slr0220	slr1022	sml0001	slr1511	slr1513
sll0469	slr1330	ndhK2	slr2132	sll1578	slr0357	sll1883	sml0001	sll1605	slr1513
slr1330	slr1517	ndhK2	slr0922	sll1362	sll1578	slr1133	sml0001	sll0990	slr1513
sll0869	slr1330	ndhK2	slr0838	sll1578	slr1550	slr0444	sml0001	sll1633	slr1513
slr1330	slr1598	ndhK2	sll1823	sll1074	sll1578	slr2130	sml0001	slr0018	slr1513
sll0868	slr1330	ndhK2	sll1056	sll1578	slr0649	sll1669	sml0001	sll0567	slr1513
slr0994	slr1330	ndhK2	slr0520	sll0495	sll1578	slr0585	sml0001	slr0884	slr1513
sll0379	slr1330	ndhK2	sll0144	sll1425	sll1578	slr2075	sml0001	sll1342	slr1513
sll1676	slr1330	ndhK2	sll0467	sll0502	sll1578	slr2076	sml0001	slr1349	slr1513
sll0927	slr1330	ndhK2	slr0711	sll1578	slr1703	sll0416	sml0001	sll0593	slr1513
sll0996	slr1330	ndhK2	sll0754	sll0078	sll1578	slr0156	sml0001	sll0220	slr1513
sll0844	slr1330	ndhK2	sll0569	sll1578	slr0557	slr1641	sml0001	sll0899	slr1513
sll1615	slr1330	ndhK2	sll1277	sll1578	slr1884	slr0542	sml0001	slr1513	slr1756
sll0202	slr1330	ndhK2	sll1110	sll1578	slr1031	sll0534	sml0001	sll1085	slr1513
slr0902	slr1330	ndhK2	sll1865	sll1578	slr1793	slr0165	sml0001	sll1931	slr1513
sll1144	slr1330	ndhK2	slr1228	sll1578	slr0633	sll0030	sml0001	sll0083	slr1513
slr0017	slr1330	ndhK2	slr0808	sll1578	slr1278	slr0847	sml0001	slr1513	slr1755
slr1330	slr1424	ndhK2	slr0082	sll1578	slr0783	slr0553	sml0001	sll0057	slr1513
slr1330	slr1423	ndhK2	slr0742	sll1198	sll1578	slr0812	sml0001	sll0017	slr1513
sll2010	slr1330	ndhK2	sll0098	sll0204	sll1578	slr1347	sml0001	sll1185	slr1513
slr0528	slr1330	ndhK2	slr1646	sll1578	sll1750	slr1348	sml0001	slr0839	slr1513
slr1330	slr1351	ndhK2	slr1130	sll0420	sll1578	slr1791	sml0001	sll1876	slr1513
sll0622	slr1330	ndhK2	slr0080	sll1578	slr1256	sll0594	sml0001	sll0900	slr1513
sll0631	slr1330	ndhK2	slr0551	sll1578	sll1639	slr0550	sml0001	slr0608	slr1513
slr0787	slr1330	ndhK2	sll0145	sll1578	slr1899	sll1058	sml0001	slr0652	slr1513
sll1852	slr1330	ndhK2	sll0708	sll0643	sll1578	slr1665	sml0001	slr0084	slr1513
slr1090	slr1330	ndhK2	slr0679	sll1578	slr1844	slr0536	sml0001	sll1893	slr1513
sll0902	slr1330	ndhK2	slr0722	sll0459	sll1578	slr1874	sml0001	slr0500	slr1513
slr0661	slr1330	ndhK2	slr0072	sll0865	sll1578	sll1776	sml0001	slr1513	slr1560
slr0526	slr1330	ndhK2	sll1547	sll1578	slr0733	sll0848	sml0001	sll1988	slr1513
sll1249	slr1330	ndhK2	sll1234	sll1459	sll1578	sll0897	sml0001	slr1289	slr1513
sll0892	slr1330	ndhK2	sll0616	sll0905	sll1578	sll1933	sml0001	sll1556	slr1513
sll0660	slr1330	ndhK2	slr2073	sll0402	sll1577	slr0965	sml0001	slr1513	ssl3441
slr1330	slr1779	ndhK2	slr1639	sll0728	sll1577	slr1463	sml0001	slr0744	slr1513
slr0394	slr1330	ndhK2	sll1108	sll0336	sll1577	sll1098	sml0001	slr0974	slr1513
slr1330	slr2047	ndhK2	sll0362	sll1299	sll1577	slr0434	sml0001	slr1513	slr1622
slr0741	slr1330	ndhK2	slr0958	sll1577	ssl2084	sll1261	sml0001	sll1196	slr1513
slr1330	slr1510	ndhK2	slr1720	sll1577	sll2001	sll1099	sml0001	sll0745	slr1513
sll1043	slr1330	ndhK2	sll0179	sll1430	sll1577	slr0752	sml0001	sll1815	slr1513
slr0400	slr1330	ndhK2	sll0454	sll0573	sll1577	slr0952	sml0001	sll1059	slr1513
sll1415	slr1330	ndhK2	sll1553	sll1577	slr1898	slr1511	sml0001	slr1123	slr1513
sll1909	slr1330	ndhK2	slr0638	sll0080	sll1577	sll1605	sml0001	sll1760	slr1513
sll0373	slr1330	ndhK2	slr0220	sll1577	slr1022	sll0990	sml0001	sll0469	slr1513
slr1330	slr2035	ndhK2	slr0357	sll1577	sll1883	sll1633	sml0001	slr1513	slr1517
slr1330	slr2132	ndhK2	sll1362	sll1577	slr1133	slr0018	sml0001	sll0869	slr1513

slr0922	slr1330	ndhK2	slr1550	sll1577	slr0444	sll0567	sml0001	slr1513	slr1598
slr0838	slr1330	ndhK2	sll1074	sll1577	slr2130	slr0884	sml0001	sll0868	slr1513
sll1823	slr1330	ndhK2	slr0649	sll1577	sll1669	sll1342	sml0001	slr0994	slr1513
sll1056	slr1330	ndhK2	sll0495	sll1577	slr0585	slr1349	sml0001	sll0379	slr1513
slr0520	slr1330	ndhK2	sll1425	sll1577	slr2075	sll0593	sml0001	sll1676	slr1513
sll0144	slr1330	ndhK2	sll0502	sll1577	slr2076	sll0220	sml0001	sll0927	slr1513
sll0467	slr1330	ndhK2	slr1703	sll0416	sll1577	sll0899	sml0001	sll0996	slr1513
slr0711	slr1330	ndhK2	sll0078	sll1577	slr0156	slr1756	sml0001	sll0844	slr1513
sll0754	slr1330	ndhK2	slr0557	sll1577	slr1641	sll1085	sml0001	sll1615	slr1513
sll0569	slr1330	ndhK2	slr1884	sll1577	slr0542	sll1931	sml0001	sll0202	slr1513
sll1277	slr1330	ndhK2	slr1031	sll0534	sll1577	sll0083	sml0001	slr0902	slr1513
sll1110	slr1330	ndhK2	slr1793	sll1577	slr0165	slr1755	sml0001	sll1144	slr1513
sll1865	slr1330	ndhK2	slr0633	sll0030	sll1577	sll0057	sml0001	slr0017	slr1513
slr1228	slr1330	ndhK2	slr1278	sll1577	slr0847	sll0017	sml0001	slr1424	slr1513
slr0808	slr1330	ndhK2	slr0783	sll1577	slr0553	sll1185	sml0001	slr1423	slr1513
slr0082	slr1330	ndhK2	sll1198	sll1577	slr0812	slr0839	sml0001	sll2010	slr1513
slr0742	slr1330	ndhK2	sll0204	sll1577	slr1347	sll1876	sml0001	slr0528	slr1513
sll0098	slr1330	ndhK2	sll1750	sll1577	slr1348	sll0900	sml0001	slr1351	slr1513
slr1330	slr1646	ndhK2	sll0420	sll1577	slr1791	slr0608	sml0001	sll0622	slr1513
slr1130	slr1330	ndhK2	slr1256	sll0594	sll1577	slr0652	sml0001	sll0631	slr1513
slr0080	slr1330	ndhK2	sll1639	sll1577	slr0550	slr0084	sml0001	slr0787	slr1513
slr0551	slr1330	ndhK2	slr1899	sll1058	sll1577	sll1893	sml0001	sll1852	slr1513
sll0145	slr1330	ndhK2	sll0643	sll1577	slr1665	slr0500	sml0001	slr1090	slr1513
sll0708	slr1330	ndhK2	slr1844	sll1577	slr0536	slr1560	sml0001	sll0902	slr1513
slr0679	slr1330	ndhK2	sll0459	sll1577	slr1874	sll1988	sml0001	slr0661	slr1513
slr0722	slr1330	ndhK2	sll0865	sll1577	sll1776	slr1289	sml0001	slr0526	slr1513
slr0072	slr1330	ndhK2	slr0733	sll0848	sll1577	sll1556	sml0001	sll1249	slr1513
sll1547	slr1330	ndhK2	sll1459	sll0897	sll1577	sml0001	ssl3441	sll0892	slr1513
sll1234	slr1330	ndhK2	sll0905	sll1577	sll1933	slr0744	sml0001	sll0660	slr1513
sll0616	slr1330	sll0402	ssr1386	sll1577	slr0965	slr0974	sml0001	slr1513	slr1779
slr1330	slr2073	sll0728	ssr1386	sll1577	slr1463	slr1622	sml0001	slr0394	slr1513
slr1330	slr1639	sll0336	ssr1386	sll1098	sll1577	sll1196	sml0001	slr1513	slr2047
sll1108	slr1330	sll1299	ssr1386	sll1577	slr0434	sll0745	sml0001	slr0741	slr1513
sll0362	slr1330	ssl2084	ssr1386	sll1261	sll1577	sll1815	sml0001	slr1510	slr1513
slr0958	slr1330	sll2001	ssr1386	sll1099	sll1577	sll1059	sml0001	sll1043	slr1513
slr1330	slr1720	sll1430	ssr1386	sll1577	slr0752	slr1123	sml0001	slr0400	slr1513
sll0179	slr1330	sll0573	ssr1386	sll1577	slr0952	sll1760	sml0001	sll1415	slr1513
sll0454	slr1330	slr1898	ssr1386	sll1577	slr1511	sll0469	sml0001	sll1909	slr1513
sll1553	slr1330	sll0080	ssr1386	sll1577	sll1605	slr1517	sml0001	sll0373	slr1513
slr0638	slr1330	slr1022	ssr1386	sll0990	sll1577	sll0869	sml0001	slr1513	slr2035
slr0220	slr1330	sll1883	ssr1386	sll1577	sll1633	slr1598	sml0001	slr1513	slr2132
slr0357	slr1330	slr1133	ssr1386	sll1577	slr0018	sll0868	sml0001	slr0922	slr1513
sll1362	slr1330	slr0444	ssr1386	sll0567	sll1577	slr0994	sml0001	slr0838	slr1513
slr1330	slr1550	slr2130	ssr1386	sll1577	slr0884	sll0379	sml0001	sll1823	slr1513
sll1074	slr1330	sll1669	ssr1386	sll1342	sll1577	sll1676	sml0001	sll1056	slr1513
slr0649	slr1330	slr0585	ssr1386	sll1577	slr1349	sll0927	sml0001	slr0520	slr1513
sll0495	slr1330	slr2075	ssr1386	sll0593	sll1577	sll0996	sml0001	sll0144	slr1513
sll1425	slr1330	slr2076	ssr1386	sll0220	sll1577	sll0844	sml0001	sll0467	slr1513
sll0502	slr1330	sll0416	ssr1386	sll0899	sll1577	sll1615	sml0001	slr0711	slr1513
slr1330	slr1703	slr0156	ssr1386	sll1577	slr1756	sll0202	sml0001	sll0754	slr1513
sll0078	slr1330	slr1641	ssr1386	sll1085	sll1577	slr0902	sml0001	sll0569	slr1513

slr0557	slr1330	slr0542	ssr1386	sll1577	sll1931	sll1144	sml0001	sll1277	slr1513
slr1330	slr1884	sll0534	ssr1386	sll0083	sll1577	slr0017	sml0001	sll1110	slr1513
slr1031	slr1330	slr0165	ssr1386	sll1577	slr1755	slr1424	sml0001	sll1865	slr1513
slr1330	slr1793	sll0030	ssr1386	sll0057	sll1577	slr1423	sml0001	slr1228	slr1513
slr0633	slr1330	slr0847	ssr1386	sll0017	sll1577	sll2010	sml0001	slr0808	slr1513
slr1278	slr1330	slr0553	ssr1386	sll1185	sll1577	slr0528	sml0001	slr0082	slr1513
slr0783	slr1330	slr0812	ssr1386	sll1577	slr0839	slr1351	sml0001	slr0742	slr1513
sll1198	slr1330	slr1347	ssr1386	sll1577	sll1876	sll0622	sml0001	sll0098	slr1513
sll0204	slr1330	slr1348	ssr1386	sll0900	sll1577	sll0631	sml0001	slr1513	slr1646
sll1750	slr1330	slr1791	ssr1386	sll1577	slr0608	slr0787	sml0001	slr1130	slr1513
sll0420	slr1330	sll0594	ssr1386	sll1577	slr0652	sll1852	sml0001	slr0080	slr1513
slr1256	slr1330	slr0550	ssr1386	sll1577	slr0084	slr1090	sml0001	slr0551	slr1513
sll1639	slr1330	sll1058	ssr1386	sll1577	sll1893	sll0902	sml0001	sll0145	slr1513
slr1330	slr1899	slr1665	ssr1386	sll1577	slr0500	slr0661	sml0001	sll0708	slr1513
sll0643	slr1330	slr0536	ssr1386	sll1577	slr1560	slr0526	sml0001	slr0679	slr1513
slr1330	slr1844	slr1874	ssr1386	sll1577	sll1988	sll1249	sml0001	slr0722	slr1513
sll0459	slr1330	sll1776	ssr1386	sll1577	slr1289	sll0892	sml0001	slr0072	slr1513
sll0865	slr1330	sll0848	ssr1386	sll1556	sll1577	sll0660	sml0001	sll1547	slr1513
slr0733	slr1330	sll0897	ssr1386	sll1577	ssl3441	slr1779	sml0001	sll1234	slr1513
sll1459	slr1330	sll1933	ssr1386	sll1577	slr0744	slr0394	sml0001	sll0616	slr1513
sll0905	slr1330	slr0965	ssr1386	sll1577	slr0974	slr2047	sml0001	slr1513	slr2073
sll0402	sll1324	slr1463	ssr1386	sll1577	slr1622	slr0741	sml0001	slr1513	slr1639
sll0728	sll1324	sll1098	ssr1386	sll1196	sll1577	slr1510	sml0001	sll1108	slr1513
sll1299	sll1324	slr0434	ssr1386	sll0745	sll1577	sll1043	sml0001	sll0362	slr1513
sll1324	ssl2084	sll1261	ssr1386	sll1577	sll1815	slr0400	sml0001	slr0958	slr1513
sll1324	sll2001	sll1099	ssr1386	sll1059	sll1577	sll1415	sml0001	slr1513	slr1720
sll1324	sll1430	slr0752	ssr1386	sll1577	slr1123	sll1909	sml0001	sll0179	slr1513
sll0573	sll1324	slr0952	ssr1386	sll1577	sll1760	sll0373	sml0001	sll0454	slr1513
sll1324	slr1898	slr1511	ssr1386	sll0469	sll1577	slr2035	sml0001	sll1553	slr1513
sll0080	sll1324	sll1605	ssr1386	sll1577	slr1517	slr2132	sml0001	slr0638	slr1513
sll1324	slr1022	sll0990	ssr1386	sll0869	sll1577	slr0922	sml0001	slr0220	slr1513
sll1324	sll1883	sll1633	ssr1386	sll1577	slr1598	slr0838	sml0001	slr0357	slr1513
sll1324	slr1133	slr0018	ssr1386	sll0868	sll1577	sll1823	sml0001	sll1362	slr1513
sll1324	slr0444	sll0567	ssr1386	sll1577	slr0994	sll1056	sml0001	slr1513	slr1550
sll1324	slr2130	slr0884	ssr1386	sll0379	sll1577	slr0520	sml0001	sll1074	slr1513
sll1324	sll1669	sll1342	ssr1386	sll1577	sll1676	sll0144	sml0001	slr0649	slr1513
sll1324	slr0585	slr1349	ssr1386	sll0927	sll1577	sll0467	sml0001	sll0495	slr1513
sll1324	slr2075	sll0593	ssr1386	sll0996	sll1577	slr0711	sml0001	sll1425	slr1513
sll1324	slr2076	sll0220	ssr1386	sll0844	sll1577	sll0754	sml0001	sll0502	slr1513
sll0416	sll1324	sll0899	ssr1386	sll1577	sll1615	sll0569	sml0001	slr1513	slr1703
sll1324	slr0156	slr1756	ssr1386	sll0202	sll1577	sll1277	sml0001	sll0078	slr1513
sll1324	slr1641	sll1085	ssr1386	sll1577	slr0902	sll1110	sml0001	slr0557	slr1513
sll1324	slr0542	sll1931	ssr1386	sll1144	sll1577	sll1865	sml0001	slr1513	slr1884
sll0534	sll1324	sll0083	ssr1386	sll1577	slr0017	slr1228	sml0001	slr1031	slr1513
sll1324	slr0165	slr1755	ssr1386	sll1577	slr1424	slr0808	sml0001	slr1513	slr1793
sll0030	sll1324	sll0057	ssr1386	sll1577	slr1423	slr0082	sml0001	slr0633	slr1513
sll1324	slr0847	sll0017	ssr1386	sll1577	sll2010	slr0742	sml0001	slr1278	slr1513
sll1324	slr0553	sll1185	ssr1386	sll1577	slr0528	sll0098	sml0001	slr0783	slr1513
sll1324	slr0812	slr0839	ssr1386	sll1577	slr1351	slr1646	sml0001	sll1198	slr1513
sll1324	slr1347	sll1876	ssr1386	sll0622	sll1577	slr1130	sml0001	sll0204	slr1513
sll1324	slr1348	sll0900	ssr1386	sll0631	sll1577	slr0080	sml0001	sll1750	slr1513

sll1324	slr1791	slr0608	ssr1386	sll1577	slr0787	slr0551	sml0001	sll0420	slr1513
sll0594	sll1324	slr0652	ssr1386	sll1577	sll1852	sll0145	sml0001	slr1256	slr1513
sll1324	slr0550	slr0084	ssr1386	sll1577	slr1090	sll0708	sml0001	sll1639	slr1513
sll1058	sll1324	sll1893	ssr1386	sll0902	sll1577	slr0679	sml0001	slr1513	slr1899
sll1324	slr1665	slr0500	ssr1386	sll1577	slr0661	slr0722	sml0001	sll0643	slr1513
sll1324	slr0536	slr1560	ssr1386	sll1577	slr0526	slr0072	sml0001	slr1513	slr1844
sll1324	slr1874	sll1988	ssr1386	sll1249	sll1577	sll1547	sml0001	sll0459	slr1513
sll1324	sll1776	slr1289	ssr1386	sll0892	sll1577	sll1234	sml0001	sll0865	slr1513
sll0848	sll1324	sll1556	ssr1386	sll0660	sll1577	sll0616	sml0001	slr0733	slr1513
sll0897	sll1324	ssl3441	ssr1386	sll1577	slr1779	slr2073	sml0001	sll1459	slr1513
sll1324	sll1933	slr0744	ssr1386	sll1577	slr0394	slr1639	sml0001	sll0905	slr1513
sll1324	slr0965	slr0974	ssr1386	sll1577	slr2047	sll1108	sml0001	sll0402	sll1697
sll1324	slr1463	slr1622	ssr1386	sll1577	slr0741	sll0362	sml0001	sll0728	sll1697
sll1098	sll1324	sll1196	ssr1386	sll1577	slr1510	slr0958	sml0001	sll0336	sll1697
sll1324	slr0434	sll0745	ssr1386	sll1043	sll1577	slr1720	sml0001	sll1299	sll1697
sll1261	sll1324	sll1815	ssr1386	sll1577	slr0400	sll0179	sml0001	sll1697	ssl2084
sll1099	sll1324	sll1059	ssr1386	sll1415	sll1577	sll0454	sml0001	sll1697	sll2001
sll1324	slr0752	slr1123	ssr1386	sll1577	sll1909	sll1553	sml0001	sll1430	sll1697
sll1324	slr0952	sll1760	ssr1386	sll0373	sll1577	slr0638	sml0001	sll0573	sll1697
sll1324	slr1511	sll0469	ssr1386	sll1577	slr2035	slr0220	sml0001	sll1697	slr1898
sll1324	sll1605	slr1517	ssr1386	sll1577	slr2132	slr0357	sml0001	sll0080	sll1697
sll0990	sll1324	sll0869	ssr1386	sll1577	slr0922	sll1362	sml0001	sll1697	slr1022
sll1324	sll1633	slr1598	ssr1386	sll1577	slr0838	slr1550	sml0001	sll1697	sll1883
sll1324	slr0018	sll0868	ssr1386	sll1577	sll1823	sll1074	sml0001	sll1697	slr1133
sll0567	sll1324	slr0994	ssr1386	sll1056	sll1577	slr0649	sml0001	sll1697	slr0444
sll1324	slr0884	sll0379	ssr1386	sll1577	slr0520	sll0495	sml0001	sll1697	slr2130
sll1324	sll1342	sll1676	ssr1386	sll0144	sll1577	sll1425	sml0001	sll1669	sll1697
sll1324	slr1349	sll0927	ssr1386	sll0467	sll1577	sll0502	sml0001	sll1697	slr0585
sll0593	sll1324	sll0996	ssr1386	sll1577	slr0711	slr1703	sml0001	sll1697	slr2075
sll0220	sll1324	sll0844	ssr1386	sll0754	sll1577	sll0078	sml0001	sll1697	slr2076
sll0899	sll1324	sll1615	ssr1386	sll0569	sll1577	slr0557	sml0001	sll0416	sll1697
sll1324	slr1756	sll0202	ssr1386	sll1277	sll1577	slr1884	sml0001	sll1697	slr0156
sll1085	sll1324	slr0902	ssr1386	sll1110	sll1577	slr1031	sml0001	sll1697	slr1641
sll1324	sll1931	sll1144	ssr1386	sll1577	sll1865	slr1793	sml0001	sll1697	slr0542
sll0083	sll1324	slr0017	ssr1386	sll1577	slr1228	slr0633	sml0001	sll0534	sll1697
sll1324	slr1755	slr1424	ssr1386	sll1577	slr0808	slr1278	sml0001	sll1697	slr0165
sll0057	sll1324	slr1423	ssr1386	sll1577	slr0082	slr0783	sml0001	sll0030	sll1697
sll0017	sll1324	sll2010	ssr1386	sll1577	slr0742	sll1198	sml0001	sll1697	slr0847
sll1185	sll1324	slr0528	ssr1386	sll0098	sll1577	sll0204	sml0001	sll1697	slr0553
sll1324	slr0839	slr1351	ssr1386	sll1577	slr1646	sll1750	sml0001	sll1697	slr0812
sll1324	sll1876	sll0622	ssr1386	sll1577	slr1130	sll0420	sml0001	sll1697	slr1347
sll0900	sll1324	sll0631	ssr1386	sll1577	slr0080	slr1256	sml0001	sll1697	slr1348
sll1324	slr0608	slr0787	ssr1386	sll1577	slr0551	sll1639	sml0001	sll1697	slr1791
sll1324	slr0652	sll1852	ssr1386	sll0145	sll1577	slr1899	sml0001	sll0594	sll1697
sll1324	slr0084	slr1090	ssr1386	sll0708	sll1577	sll0643	sml0001	sll1697	slr0550
sll1324	sll1893	sll0902	ssr1386	sll1577	slr0679	slr1844	sml0001	sll1058	sll1697
sll1324	slr0500	slr0661	ssr1386	sll1577	slr0722	sll0459	sml0001	sll1697	slr1665
sll1324	slr1560	slr0526	ssr1386	sll1577	slr0072	sll0865	sml0001	sll1697	slr0536
sll1324	sll1988	sll1249	ssr1386	sll1547	sll1577	slr0733	sml0001	sll1697	slr1874
sll1324	slr1289	sll0892	ssr1386	sll1234	sll1577	sll1459	sml0001	sll1697	sll1776
sll1324	sll1556	sll0660	ssr1386	sll0616	sll1577	sll0905	sml0001	sll0848	sll1697

sll1324	ssl3441	slr1779	ssr1386	sll1577	slr2073	sll0402	smr0008	sll0897	sll1697
sll1324	slr0744	slr0394	ssr1386	sll1577	slr1639	sll0728	smr0008	sll1697	sll1933
sll1324	slr0974	slr2047	ssr1386	sll1108	sll1577	sll0336	smr0008	sll1697	slr0965
sll1324	slr1622	slr0741	ssr1386	sll0362	sll1577	sll1299	smr0008	sll1697	slr1463
sll1196	sll1324	slr1510	ssr1386	sll1577	slr0958	smr0008	ssl2084	sll1098	sll1697
sll0745	sll1324	sll1043	ssr1386	sll1577	slr1720	sll2001	smr0008	sll1697	slr0434
sll1324	sll1815	slr0400	ssr1386	sll0179	sll1577	sll1430	smr0008	sll1261	sll1697
sll1059	sll1324	sll1415	ssr1386	sll0454	sll1577	sll0573	smr0008	sll1099	sll1697
sll1324	slr1123	sll1909	ssr1386	sll1553	sll1577	slr1898	smr0008	sll1697	slr0752
sll1324	sll1760	sll0373	ssr1386	sll1577	slr0638	sll0080	smr0008	sll1697	slr0952
sll0469	sll1324	slr2035	ssr1386	sll1577	slr0220	slr1022	smr0008	sll1697	slr1511
sll1324	slr1517	slr2132	ssr1386	sll1577	slr0357	sll1883	smr0008	sll1605	sll1697
sll0869	sll1324	slr0922	ssr1386	sll1362	sll1577	slr1133	smr0008	sll0990	sll1697
sll1324	slr1598	slr0838	ssr1386	sll1577	slr1550	slr0444	smr0008	sll1633	sll1697
sll0868	sll1324	sll1823	ssr1386	sll1074	sll1577	slr2130	smr0008	sll1697	slr0018
sll1324	slr0994	sll1056	ssr1386	sll1577	slr0649	sll1669	smr0008	sll0567	sll1697
sll0379	sll1324	slr0520	ssr1386	sll0495	sll1577	slr0585	smr0008	sll1697	slr0884
sll1324	sll1676	sll0144	ssr1386	sll1425	sll1577	slr2075	smr0008	sll1342	sll1697
sll0927	sll1324	sll0467	ssr1386	sll0502	sll1577	slr2076	smr0008	sll1697	slr1349
sll0996	sll1324	slr0711	ssr1386	sll1577	slr1703	sll0416	smr0008	sll0593	sll1697
sll0844	sll1324	sll0754	ssr1386	sll0078	sll1577	slr0156	smr0008	sll0220	sll1697
sll1324	sll1615	sll0569	ssr1386	sll1577	slr0557	slr1641	smr0008	sll0899	sll1697
sll0202	sll1324	sll1277	ssr1386	sll1577	slr1884	slr0542	smr0008	sll1697	slr1756
sll1324	slr0902	sll1110	ssr1386	sll1577	slr1031	sll0534	smr0008	sll1085	sll1697
sll1144	sll1324	sll1865	ssr1386	sll1577	slr1793	slr0165	smr0008	sll1697	sll1931
sll1324	slr0017	slr1228	ssr1386	sll1577	slr0633	sll0030	smr0008	sll0083	sll1697
sll1324	slr1424	slr0808	ssr1386	sll1577	slr1278	slr0847	smr0008	sll1697	slr1755
sll1324	slr1423	slr0082	ssr1386	sll1577	slr0783	slr0553	smr0008	sll0057	sll1697
sll1324	sll2010	slr0742	ssr1386	sll1198	sll1577	slr0812	smr0008	sll0017	sll1697
sll1324	slr0528	sll0098	ssr1386	sll0204	sll1577	slr1347	smr0008	sll1185	sll1697
sll1324	slr1351	slr1646	ssr1386	sll1577	sll1750	slr1348	smr0008	sll1697	slr0839
sll0622	sll1324	slr1130	ssr1386	sll0420	sll1577	slr1791	smr0008	sll1697	sll1876
sll0631	sll1324	slr0080	ssr1386	sll1577	slr1256	sll0594	smr0008	sll0900	sll1697
sll1324	slr0787	slr0551	ssr1386	sll1577	sll1639	slr0550	smr0008	sll1697	slr0608
sll1324	sll1852	sll0145	ssr1386	sll1577	slr1899	sll1058	smr0008	sll1697	slr0652
sll1324	slr1090	sll0708	ssr1386	sll0643	sll1577	slr1665	smr0008	sll1697	slr0084
sll0902	sll1324	slr0679	ssr1386	sll1577	slr1844	slr0536	smr0008	sll1697	sll1893
sll1324	slr0661	slr0722	ssr1386	sll0459	sll1577	slr1874	smr0008	sll1697	slr0500
sll1324	slr0526	slr0072	ssr1386	sll0865	sll1577	sll1776	smr0008	sll1697	slr1560
sll1249	sll1324	sll1547	ssr1386	sll1577	slr0733	sll0848	smr0008	sll1697	sll1988
sll0892	sll1324	sll1234	ssr1386	sll1459	sll1577	sll0897	smr0008	sll1697	slr1289
sll0660	sll1324	sll0616	ssr1386	sll0905	sll1577	sll1933	smr0008	sll1556	sll1697
sll1324	slr1779	slr2073	ssr1386	sll0402	slr1834	slr0965	smr0008	sll1697	ssl3441
sll1324	slr0394	slr1639	ssr1386	sll0728	slr1834	slr1463	smr0008	sll1697	slr0744
sll1324	slr2047	sll1108	ssr1386	sll0336	slr1834	sll1098	smr0008	sll1697	slr0974
sll1324	slr0741	sll0362	ssr1386	sll1299	slr1834	slr0434	smr0008	sll1697	slr1622
sll1324	slr1510	slr0958	ssr1386	slr1834	ssl2084	sll1261	smr0008	sll1196	sll1697
sll1043	sll1324	slr1720	ssr1386	sll2001	slr1834	sll1099	smr0008	sll0745	sll1697
sll1324	slr0400	sll0179	ssr1386	sll1430	slr1834	slr0752	smr0008	sll1697	sll1815
sll1324	sll1415	sll0454	ssr1386	sll0573	slr1834	slr0952	smr0008	sll1059	sll1697
sll1324	sll1909	sll1553	ssr1386	slr1834	slr1898	slr1511	smr0008	sll1697	slr1123

sll0373	sll1324	slr0638	ssr1386	sll0080	slr1834	sll1605	smr0008	sll1697	sll1760
sll1324	slr2035	slr0220	ssr1386	slr1022	slr1834	sll0990	smr0008	sll0469	sll1697
sll1324	slr2132	slr0357	ssr1386	sll1883	slr1834	sll1633	smr0008	sll1697	slr1517
sll1324	slr0922	sll1362	ssr1386	slr1133	slr1834	slr0018	smr0008	sll0869	sll1697
sll1324	slr0838	slr1550	ssr1386	slr0444	slr1834	sll0567	smr0008	sll1697	slr1598
sll1324	sll1823	sll1074	ssr1386	slr1834	slr2130	slr0884	smr0008	sll0868	sll1697
sll1056	sll1324	slr0649	ssr1386	sll1669	slr1834	sll1342	smr0008	sll1697	slr0994
sll1324	slr0520	sll0495	ssr1386	slr0585	slr1834	slr1349	smr0008	sll0379	sll1697
sll0144	sll1324	sll1425	ssr1386	slr1834	slr2075	sll0593	smr0008	sll1676	sll1697
sll0467	sll1324	sll0502	ssr1386	slr1834	slr2076	sll0220	smr0008	sll0927	sll1697
sll1324	slr0711	slr1703	ssr1386	sll0416	slr1834	sll0899	smr0008	sll0996	sll1697
sll0754	sll1324	sll0078	ssr1386	slr0156	slr1834	slr1756	smr0008	sll0844	sll1697
sll0569	sll1324	slr0557	ssr1386	slr1641	slr1834	sll1085	smr0008	sll1615	sll1697
sll1277	sll1324	slr1884	ssr1386	slr0542	slr1834	sll1931	smr0008	sll0202	sll1697
sll1110	sll1324	slr1031	ssr1386	sll0534	slr1834	sll0083	smr0008	sll1697	slr0902
sll1324	sll1865	slr1793	ssr1386	slr0165	slr1834	slr1755	smr0008	sll1144	sll1697
sll1324	slr1228	slr0633	ssr1386	sll0030	slr1834	sll0057	smr0008	sll1697	slr0017
sll1324	slr0808	slr1278	ssr1386	slr0847	slr1834	sll0017	smr0008	sll1697	slr1424
sll1324	slr0082	slr0783	ssr1386	slr0553	slr1834	sll1185	smr0008	sll1697	slr1423
sll1324	slr0742	sll1198	ssr1386	slr0812	slr1834	slr0839	smr0008	sll1697	sll2010
sll0098	sll1324	sll0204	ssr1386	slr1347	slr1834	sll1876	smr0008	sll1697	slr0528
sll1324	slr1646	sll1750	ssr1386	slr1348	slr1834	sll0900	smr0008	sll1697	slr1351
sll1324	slr1130	sll0420	ssr1386	slr1791	slr1834	slr0608	smr0008	sll0622	sll1697
sll1324	slr0080	slr1256	ssr1386	sll0594	slr1834	slr0652	smr0008	sll0631	sll1697
sll1324	slr0551	sll1639	ssr1386	slr0550	slr1834	slr0084	smr0008	sll1697	slr0787
sll0145	sll1324	slr1899	ssr1386	sll1058	slr1834	sll1893	smr0008	sll1697	sll1852
sll0708	sll1324	sll0643	ssr1386	slr1665	slr1834	slr0500	smr0008	sll1697	slr1090
sll1324	slr0679	slr1844	ssr1386	slr0536	slr1834	slr1560	smr0008	sll0902	sll1697
sll1324	slr0722	sll0459	ssr1386	slr1834	slr1874	sll1988	smr0008	sll1697	slr0661
sll1324	slr0072	sll0865	ssr1386	sll1776	slr1834	slr1289	smr0008	sll1697	slr0526
sll1324	sll1547	slr0733	ssr1386	sll0848	slr1834	sll1556	smr0008	sll1249	sll1697
sll1234	sll1324	sll1459	ssr1386	sll0897	slr1834	smr0008	ssl3441	sll0892	sll1697
sll0616	sll1324	sll0905	ssr1386	sll1933	slr1834	slr0744	smr0008	sll0660	sll1697
sll1324	slr2073	sll0402	slr1623	slr0965	slr1834	slr0974	smr0008	sll1697	slr1779
sll1324	slr1639	sll0728	slr1623	slr1463	slr1834	slr1622	smr0008	sll1697	slr0394
sll1108	sll1324	sll0336	slr1623	sll1098	slr1834	sll1196	smr0008	sll1697	slr2047
sll0362	sll1324	sll1299	slr1623	slr0434	slr1834	sll0745	smr0008	sll1697	slr0741
sll1324	slr0958	slr1623	ssl2084	sll1261	slr1834	sll1815	smr0008	sll1697	slr1510
sll1324	slr1720	sll2001	slr1623	sll1099	slr1834	sll1059	smr0008	sll1043	sll1697
sll0179	sll1324	sll1430	slr1623	slr0752	slr1834	slr1123	smr0008	sll1697	slr0400
sll0454	sll1324	sll0573	slr1623	slr0952	slr1834	sll1760	smr0008	sll1415	sll1697
sll1324	sll1553	slr1623	slr1898	slr1511	slr1834	sll0469	smr0008	sll1697	sll1909
sll1324	slr0638	sll0080	slr1623	sll1605	slr1834	slr1517	smr0008	sll0373	sll1697
sll1324	slr0220	slr1022	slr1623	sll0990	slr1834	sll0869	smr0008	sll1697	slr2035
sll1324	slr0357	sll1883	slr1623	sll1633	slr1834	slr1598	smr0008	sll1697	slr2132
sll1324	sll1362	slr1133	slr1623	slr0018	slr1834	sll0868	smr0008	sll1697	slr0922
sll1324	slr1550	slr0444	slr1623	sll0567	slr1834	slr0994	smr0008	sll1697	slr0838
sll1074	sll1324	slr1623	slr2130	slr0884	slr1834	sll0379	smr0008	sll1697	sll1823
sll1324	slr0649	sll1669	slr1623	sll1342	slr1834	sll1676	smr0008	sll1056	sll1697
sll0495	sll1324	slr0585	slr1623	slr1349	slr1834	sll0927	smr0008	sll1697	slr0520
sll1324	sll1425	slr1623	slr2075	sll0593	slr1834	sll0996	smr0008	sll0144	sll1697

sll0502	sll1324	slr1623	slr2076	sll0220	slr1834	sll0844	smr0008	sll0467	sll1697
sll1324	slr1703	sll0416	slr1623	sll0899	slr1834	sll1615	smr0008	sll1697	slr0711
sll0078	sll1324	slr0156	slr1623	slr1756	slr1834	sll0202	smr0008	sll0754	sll1697
sll1324	slr0557	slr1623	slr1641	sll1085	slr1834	slr0902	smr0008	sll0569	sll1697
sll1324	slr1884	slr0542	slr1623	sll1931	slr1834	sll1144	smr0008	sll1277	sll1697
sll1324	slr1031	sll0534	slr1623	sll0083	slr1834	slr0017	smr0008	sll1110	sll1697
sll1324	slr1793	slr0165	slr1623	slr1755	slr1834	slr1424	smr0008	sll1697	sll1865
sll1324	slr0633	sll0030	slr1623	sll0057	slr1834	slr1423	smr0008	sll1697	slr1228
sll1324	slr1278	slr0847	slr1623	sll0017	slr1834	sll2010	smr0008	sll1697	slr0808
sll1324	slr0783	slr0553	slr1623	sll1185	slr1834	slr0528	smr0008	sll1697	slr0082
sll1198	sll1324	slr0812	slr1623	slr0839	slr1834	slr1351	smr0008	sll1697	slr0742
sll0204	sll1324	slr1347	slr1623	sll1876	slr1834	sll0622	smr0008	sll0098	sll1697
sll1324	sll1750	slr1348	slr1623	sll0900	slr1834	sll0631	smr0008	sll1697	slr1646
sll0420	sll1324	slr1623	slr1791	slr0608	slr1834	slr0787	smr0008	sll1697	slr1130
sll1324	slr1256	sll0594	slr1623	slr0652	slr1834	sll1852	smr0008	sll1697	slr0080
sll1324	sll1639	slr0550	slr1623	slr0084	slr1834	slr1090	smr0008	sll1697	slr0551
sll1324	slr1899	sll1058	slr1623	sll1893	slr1834	sll0902	smr0008	sll0145	sll1697
sll0643	sll1324	slr1623	slr1665	slr0500	slr1834	slr0661	smr0008	sll0708	sll1697
sll1324	slr1844	slr0536	slr1623	slr1560	slr1834	slr0526	smr0008	sll1697	slr0679
sll0459	sll1324	slr1623	slr1874	sll1988	slr1834	sll1249	smr0008	sll1697	slr0722
sll0865	sll1324	sll1776	slr1623	slr1289	slr1834	sll0892	smr0008	sll1697	slr0072
sll1324	slr0733	sll0848	slr1623	sll1556	slr1834	sll0660	smr0008	sll1547	sll1697
sll1324	sll1459	sll0897	slr1623	slr1834	ssl3441	slr1779	smr0008	sll1234	sll1697
sll0905	sll1324	sll1933	slr1623	slr0744	slr1834	slr0394	smr0008	sll0616	sll1697
sll0402	sll1327	slr0965	slr1623	slr0974	slr1834	slr2047	smr0008	sll1697	slr2073
sll0728	sll1327	slr1463	slr1623	slr1622	slr1834	slr0741	smr0008	sll1697	slr1639
sll0336	sll1327	sll1098	slr1623	sll1196	slr1834	slr1510	smr0008	sll1108	sll1697
sll1299	sll1327	slr0434	slr1623	sll0745	slr1834	sll1043	smr0008	sll0362	sll1697
sll1327	ssl2084	sll1261	slr1623	sll1815	slr1834	slr0400	smr0008	sll1697	slr0958
sll1327	sll2001	sll1099	slr1623	sll1059	slr1834	sll1415	smr0008	sll1697	slr1720
sll1327	sll1430	slr0752	slr1623	slr1123	slr1834	sll1909	smr0008	sll0179	sll1697
sll0573	sll1327	slr0952	slr1623	sll1760	slr1834	sll0373	smr0008	sll0454	sll1697
sll1327	slr1898	slr1511	slr1623	sll0469	slr1834	slr2035	smr0008	sll1553	sll1697
sll0080	sll1327	sll1605	slr1623	slr1517	slr1834	slr2132	smr0008	sll1697	slr0638
sll1327	slr1022	sll0990	slr1623	sll0869	slr1834	slr0922	smr0008	sll1697	slr0220
sll1327	sll1883	sll1633	slr1623	slr1598	slr1834	slr0838	smr0008	sll1697	slr0357
sll1327	slr1133	slr0018	slr1623	sll0868	slr1834	sll1823	smr0008	sll1362	sll1697
sll1327	slr0444	sll0567	slr1623	slr0994	slr1834	sll1056	smr0008	sll1697	slr1550
sll1327	slr2130	slr0884	slr1623	sll0379	slr1834	slr0520	smr0008	sll1074	sll1697
sll1327	sll1669	sll1342	slr1623	sll1676	slr1834	sll0144	smr0008	sll1697	slr0649
sll1327	slr0585	slr1349	slr1623	sll0927	slr1834	sll0467	smr0008	sll0495	sll1697
sll1327	slr2075	sll0593	slr1623	sll0996	slr1834	slr0711	smr0008	sll1425	sll1697
sll1327	slr2076	sll0220	slr1623	sll0844	slr1834	sll0754	smr0008	sll0502	sll1697
sll0416	sll1327	sll0899	slr1623	sll1615	slr1834	sll0569	smr0008	sll1697	slr1703
sll1327	slr0156	slr1623	slr1756	sll0202	slr1834	sll1277	smr0008	sll0078	sll1697
sll1327	slr1641	sll1085	slr1623	slr0902	slr1834	sll1110	smr0008	sll1697	slr0557
sll1327	slr0542	sll1931	slr1623	sll1144	slr1834	sll1865	smr0008	sll1697	slr1884
sll0534	sll1327	sll0083	slr1623	slr0017	slr1834	slr1228	smr0008	sll1697	slr1031
sll1327	slr0165	slr1623	slr1755	slr1424	slr1834	slr0808	smr0008	sll1697	slr1793
sll0030	sll1327	sll0057	slr1623	slr1423	slr1834	slr0082	smr0008	sll1697	slr0633
sll1327	slr0847	sll0017	slr1623	sll2010	slr1834	slr0742	smr0008	sll1697	slr1278

sll1327	slr0553	sll1185	slr1623	slr0528	slr1834	sll0098	smr0008	sll1697	slr0783
sll1327	slr0812	slr0839	slr1623	slr1351	slr1834	slr1646	smr0008	sll1198	sll1697
sll1327	slr1347	sll1876	slr1623	sll0622	slr1834	slr1130	smr0008	sll0204	sll1697
sll1327	slr1348	sll0900	slr1623	sll0631	slr1834	slr0080	smr0008	sll1697	sll1750
sll1327	slr1791	slr0608	slr1623	slr0787	slr1834	slr0551	smr0008	sll0420	sll1697
sll0594	sll1327	slr0652	slr1623	sll1852	slr1834	sll0145	smr0008	sll1697	slr1256
sll1327	slr0550	slr0084	slr1623	slr1090	slr1834	sll0708	smr0008	sll1639	sll1697
sll1058	sll1327	sll1893	slr1623	sll0902	slr1834	slr0679	smr0008	sll1697	slr1899
sll1327	slr1665	slr0500	slr1623	slr0661	slr1834	slr0722	smr0008	sll0643	sll1697
sll1327	slr0536	slr1560	slr1623	slr0526	slr1834	slr0072	smr0008	sll1697	slr1844
sll1327	slr1874	sll1988	slr1623	sll1249	slr1834	sll1547	smr0008	sll0459	sll1697
sll1327	sll1776	slr1289	slr1623	sll0892	slr1834	sll1234	smr0008	sll0865	sll1697
sll0848	sll1327	sll1556	slr1623	sll0660	slr1834	sll0616	smr0008	sll1697	slr0733
sll0897	sll1327	slr1623	ssl3441	slr1779	slr1834	slr2073	smr0008	sll1459	sll1697
sll1327	sll1933	slr0744	slr1623	slr0394	slr1834	slr1639	smr0008	sll0905	sll1697
sll1327	slr0965	slr0974	slr1623	slr1834	slr2047	sll1108	smr0008	sll0402	slr1796
sll1327	slr1463	slr1622	slr1623	slr0741	slr1834	sll0362	smr0008	sll0728	slr1796
sll1098	sll1327	sll1196	slr1623	slr1510	slr1834	slr0958	smr0008	sll0336	slr1796
sll1327	slr0434	sll0745	slr1623	sll1043	slr1834	slr1720	smr0008	sll1299	slr1796
sll1261	sll1327	sll1815	slr1623	slr0400	slr1834	sll0179	smr0008	slr1796	ssl2084
sll1099	sll1327	sll1059	slr1623	sll1415	slr1834	sll0454	smr0008	sll2001	slr1796
sll1327	slr0752	slr1123	slr1623	sll1909	slr1834	sll1553	smr0008	sll1430	slr1796
sll1327	slr0952	sll1760	slr1623	sll0373	slr1834	slr0638	smr0008	sll0573	slr1796
sll1327	slr1511	sll0469	slr1623	slr1834	slr2035	slr0220	smr0008	slr1796	slr1898
sll1327	sll1605	slr1517	slr1623	slr1834	slr2132	slr0357	smr0008	sll0080	slr1796
sll0990	sll1327	sll0869	slr1623	slr0922	slr1834	sll1362	smr0008	slr1022	slr1796
sll1327	sll1633	slr1598	slr1623	slr0838	slr1834	slr1550	smr0008	sll1883	slr1796
sll1327	slr0018	sll0868	slr1623	sll1823	slr1834	sll1074	smr0008	slr1133	slr1796
sll0567	sll1327	slr0994	slr1623	sll1056	slr1834	slr0649	smr0008	slr0444	slr1796
sll1327	slr0884	sll0379	slr1623	slr0520	slr1834	sll0495	smr0008	slr1796	slr2130
sll1327	sll1342	sll1676	slr1623	sll0144	slr1834	sll1425	smr0008	sll1669	slr1796
sll1327	slr1349	sll0927	slr1623	sll0467	slr1834	sll0502	smr0008	slr0585	slr1796
sll0593	sll1327	sll0996	slr1623	slr0711	slr1834	slr1703	smr0008	slr1796	slr2075
sll0220	sll1327	sll0844	slr1623	sll0754	slr1834	sll0078	smr0008	slr1796	slr2076
sll0899	sll1327	sll1615	slr1623	sll0569	slr1834	slr0557	smr0008	sll0416	slr1796
sll1327	slr1756	sll0202	slr1623	sll1277	slr1834	slr1884	smr0008	slr0156	slr1796
sll1085	sll1327	slr0902	slr1623	sll1110	slr1834	slr1031	smr0008	slr1641	slr1796
sll1327	sll1931	sll1144	slr1623	sll1865	slr1834	slr1793	smr0008	slr0542	slr1796
sll0083	sll1327	slr0017	slr1623	slr1228	slr1834	slr0633	smr0008	sll0534	slr1796
sll1327	slr1755	slr1424	slr1623	slr0808	slr1834	slr1278	smr0008	slr0165	slr1796
sll0057	sll1327	slr1423	slr1623	slr0082	slr1834	slr0783	smr0008	sll0030	slr1796
sll0017	sll1327	sll2010	slr1623	slr0742	slr1834	sll1198	smr0008	slr0847	slr1796
sll1185	sll1327	slr0528	slr1623	sll0098	slr1834	sll0204	smr0008	slr0553	slr1796
sll1327	slr0839	slr1351	slr1623	slr1646	slr1834	sll1750	smr0008	slr0812	slr1796
sll1327	sll1876	sll0622	slr1623	slr1130	slr1834	sll0420	smr0008	slr1347	slr1796
sll0900	sll1327	sll0631	slr1623	slr0080	slr1834	slr1256	smr0008	slr1348	slr1796
sll1327	slr0608	slr0787	slr1623	slr0551	slr1834	sll1639	smr0008	slr1791	slr1796
sll1327	slr0652	sll1852	slr1623	sll0145	slr1834	slr1899	smr0008	sll0594	slr1796
sll1327	slr0084	slr1090	slr1623	sll0708	slr1834	sll0643	smr0008	slr0550	slr1796
sll1327	sll1893	sll0902	slr1623	slr0679	slr1834	slr1844	smr0008	sll1058	slr1796
sll1327	slr0500	slr0661	slr1623	slr0722	slr1834	sll0459	smr0008	slr1665	slr1796

sll1327	slr1560	slr0526	slr1623	slr0072	slr1834	sll0865	smr0008	slr0536	slr1796
sll1327	sll1988	sll1249	slr1623	sll1547	slr1834	slr0733	smr0008	slr1796	slr1874
sll1327	slr1289	sll0892	slr1623	sll1234	slr1834	sll1459	smr0008	sll1776	slr1796
sll1327	sll1556	sll0660	slr1623	sll0616	slr1834	sll0905	smr0008	sll0848	slr1796
sll1327	ssl3441	slr1623	slr1779	slr1834	slr2073	sll0402	smr0007	sll0897	slr1796
sll1327	slr0744	slr0394	slr1623	slr1639	slr1834	sll0728	smr0007	sll1933	slr1796
sll1327	slr0974	slr1623	slr2047	sll1108	slr1834	sll0336	smr0007	slr0965	slr1796
sll1327	slr1622	slr0741	slr1623	sll0362	slr1834	sll1299	smr0007	slr1463	slr1796
sll1196	sll1327	slr1510	slr1623	slr0958	slr1834	smr0007	ssl2084	sll1098	slr1796
sll0745	sll1327	sll1043	slr1623	slr1720	slr1834	sll2001	smr0007	slr0434	slr1796
sll1327	sll1815	slr0400	slr1623	sll0179	slr1834	sll1430	smr0007	sll1261	slr1796
sll1059	sll1327	sll1415	slr1623	sll0454	slr1834	sll0573	smr0007	sll1099	slr1796
sll1327	slr1123	sll1909	slr1623	sll1553	slr1834	slr1898	smr0007	slr0752	slr1796
sll1327	sll1760	sll0373	slr1623	slr0638	slr1834	sll0080	smr0007	slr0952	slr1796
sll0469	sll1327	slr1623	slr2035	slr0220	slr1834	slr1022	smr0007	slr1511	slr1796
sll1327	slr1517	slr1623	slr2132	slr0357	slr1834	sll1883	smr0007	sll1605	slr1796
sll0869	sll1327	slr0922	slr1623	sll1362	slr1834	slr1133	smr0007	sll0990	slr1796
sll1327	slr1598	slr0838	slr1623	slr1550	slr1834	slr0444	smr0007	sll1633	slr1796
sll0868	sll1327	sll1823	slr1623	sll1074	slr1834	slr2130	smr0007	slr0018	slr1796
sll1327	slr0994	sll1056	slr1623	slr0649	slr1834	sll1669	smr0007	sll0567	slr1796
sll0379	sll1327	slr0520	slr1623	sll0495	slr1834	slr0585	smr0007	slr0884	slr1796
sll1327	sll1676	sll0144	slr1623	sll1425	slr1834	slr2075	smr0007	sll1342	slr1796
sll0927	sll1327	sll0467	slr1623	sll0502	slr1834	slr2076	smr0007	slr1349	slr1796
sll0996	sll1327	slr0711	slr1623	slr1703	slr1834	sll0416	smr0007	sll0593	slr1796
sll0844	sll1327	sll0754	slr1623	sll0078	slr1834	slr0156	smr0007	sll0220	slr1796
sll1327	sll1615	sll0569	slr1623	slr0557	slr1834	slr1641	smr0007	sll0899	slr1796
sll0202	sll1327	sll1277	slr1623	slr1834	slr1884	slr0542	smr0007	slr1756	slr1796
sll1327	slr0902	sll1110	slr1623	slr1031	slr1834	sll0534	smr0007	sll1085	slr1796
sll1144	sll1327	sll1865	slr1623	slr1793	slr1834	slr0165	smr0007	sll1931	slr1796
sll1327	slr0017	slr1228	slr1623	slr0633	slr1834	sll0030	smr0007	sll0083	slr1796
sll1327	slr1424	slr0808	slr1623	slr1278	slr1834	slr0847	smr0007	slr1755	slr1796
sll1327	slr1423	slr0082	slr1623	slr0783	slr1834	slr0553	smr0007	sll0057	slr1796
sll1327	sll2010	slr0742	slr1623	sll1198	slr1834	slr0812	smr0007	sll0017	slr1796
sll1327	slr0528	sll0098	slr1623	sll0204	slr1834	slr1347	smr0007	sll1185	slr1796
sll1327	slr1351	slr1623	slr1646	sll1750	slr1834	slr1348	smr0007	slr0839	slr1796
sll0622	sll1327	slr1130	slr1623	sll0420	slr1834	slr1791	smr0007	sll1876	slr1796
sll0631	sll1327	slr0080	slr1623	slr1256	slr1834	sll0594	smr0007	sll0900	slr1796
sll1327	slr0787	slr0551	slr1623	sll1639	slr1834	slr0550	smr0007	slr0608	slr1796
sll1327	sll1852	sll0145	slr1623	slr1834	slr1899	sll1058	smr0007	slr0652	slr1796
sll1327	slr1090	sll0708	slr1623	sll0643	slr1834	slr1665	smr0007	slr0084	slr1796
sll0902	sll1327	slr0679	slr1623	slr1834	slr1844	slr0536	smr0007	sll1893	slr1796
sll1327	slr0661	slr0722	slr1623	sll0459	slr1834	slr1874	smr0007	slr0500	slr1796
sll1327	slr0526	slr0072	slr1623	sll0865	slr1834	sll1776	smr0007	slr1560	slr1796
sll1249	sll1327	sll1547	slr1623	slr0733	slr1834	sll0848	smr0007	sll1988	slr1796
sll0892	sll1327	sll1234	slr1623	sll1459	slr1834	sll0897	smr0007	slr1289	slr1796
sll0660	sll1327	sll0616	slr1623	sll0905	slr1834	sll1933	smr0007	sll1556	slr1796
sll1327	slr1779	slr1623	slr2073	sll0402	slr1835	slr0965	smr0007	slr1796	ssl3441
sll1327	slr0394	slr1623	slr1639	sll0728	slr1835	slr1463	smr0007	slr0744	slr1796
sll1327	slr2047	sll1108	slr1623	sll0336	slr1835	sll1098	smr0007	slr0974	slr1796
sll1327	slr0741	sll0362	slr1623	sll1299	slr1835	slr0434	smr0007	slr1622	slr1796
sll1327	slr1510	slr0958	slr1623	slr1835	ssl2084	sll1261	smr0007	sll1196	slr1796

sll1043	sll1327	slr1623	slr1720	sll2001	slr1835	sll1099	smr0007	sll0745	slr1796
sll1327	slr0400	sll0179	slr1623	sll1430	slr1835	slr0752	smr0007	sll1815	slr1796
sll1327	sll1415	sll0454	slr1623	sll0573	slr1835	slr0952	smr0007	sll1059	slr1796
sll1327	sll1909	sll1553	slr1623	slr1835	slr1898	slr1511	smr0007	slr1123	slr1796
sll0373	sll1327	slr0638	slr1623	sll0080	slr1835	sll1605	smr0007	sll1760	slr1796
sll1327	slr2035	slr0220	slr1623	slr1022	slr1835	sll0990	smr0007	sll0469	slr1796
sll1327	slr2132	slr0357	slr1623	sll1883	slr1835	sll1633	smr0007	slr1517	slr1796
sll1327	slr0922	sll1362	slr1623	slr1133	slr1835	slr0018	smr0007	sll0869	slr1796
sll1327	slr0838	slr1550	slr1623	slr0444	slr1835	sll0567	smr0007	slr1598	slr1796
sll1327	sll1823	sll1074	slr1623	slr1835	slr2130	slr0884	smr0007	sll0868	slr1796
sll1056	sll1327	slr0649	slr1623	sll1669	slr1835	sll1342	smr0007	slr0994	slr1796
sll1327	slr0520	sll0495	slr1623	slr0585	slr1835	slr1349	smr0007	sll0379	slr1796
sll0144	sll1327	sll1425	slr1623	slr1835	slr2075	sll0593	smr0007	sll1676	slr1796
sll0467	sll1327	sll0502	slr1623	slr1835	slr2076	sll0220	smr0007	sll0927	slr1796
sll1327	slr0711	slr1623	slr1703	sll0416	slr1835	sll0899	smr0007	sll0996	slr1796
sll0754	sll1327	sll0078	slr1623	slr0156	slr1835	slr1756	smr0007	sll0844	slr1796
sll0569	sll1327	slr0557	slr1623	slr1641	slr1835	sll1085	smr0007	sll1615	slr1796
sll1277	sll1327	slr1623	slr1884	slr0542	slr1835	sll1931	smr0007	sll0202	slr1796
sll1110	sll1327	slr1031	slr1623	sll0534	slr1835	sll0083	smr0007	slr0902	slr1796
sll1327	sll1865	slr1623	slr1793	slr0165	slr1835	slr1755	smr0007	sll1144	slr1796
sll1327	slr1228	slr0633	slr1623	sll0030	slr1835	sll0057	smr0007	slr0017	slr1796
sll1327	slr0808	slr1278	slr1623	slr0847	slr1835	sll0017	smr0007	slr1424	slr1796
sll1327	slr0082	slr0783	slr1623	slr0553	slr1835	sll1185	smr0007	slr1423	slr1796
sll1327	slr0742	sll1198	slr1623	slr0812	slr1835	slr0839	smr0007	sll2010	slr1796
sll0098	sll1327	sll0204	slr1623	slr1347	slr1835	sll1876	smr0007	slr0528	slr1796
sll1327	slr1646	sll1750	slr1623	slr1348	slr1835	sll0900	smr0007	slr1351	slr1796
sll1327	slr1130	sll0420	slr1623	slr1791	slr1835	slr0608	smr0007	sll0622	slr1796
sll1327	slr0080	slr1256	slr1623	sll0594	slr1835	slr0652	smr0007	sll0631	slr1796
sll1327	slr0551	sll1639	slr1623	slr0550	slr1835	slr0084	smr0007	slr0787	slr1796
sll0145	sll1327	slr1623	slr1899	sll1058	slr1835	sll1893	smr0007	sll1852	slr1796
sll0708	sll1327	sll0643	slr1623	slr1665	slr1835	slr0500	smr0007	slr1090	slr1796
sll1327	slr0679	slr1623	slr1844	slr0536	slr1835	slr1560	smr0007	sll0902	slr1796
sll1327	slr0722	sll0459	slr1623	slr1835	slr1874	sll1988	smr0007	slr0661	slr1796
sll1327	slr0072	sll0865	slr1623	sll1776	slr1835	slr1289	smr0007	slr0526	slr1796
sll1327	sll1547	slr0733	slr1623	sll0848	slr1835	sll1556	smr0007	sll1249	slr1796
sll1234	sll1327	sll1459	slr1623	sll0897	slr1835	smr0007	ssl3441	sll0892	slr1796
sll0616	sll1327	sll0905	slr1623	sll1933	slr1835	slr0744	smr0007	sll0660	slr1796
sll1327	slr2073	sll0402	sll1262	slr0965	slr1835	slr0974	smr0007	slr1779	slr1796
sll1327	slr1639	sll0728	sll1262	slr1463	slr1835	slr1622	smr0007	slr0394	slr1796
sll1108	sll1327	sll0336	sll1262	sll1098	slr1835	sll1196	smr0007	slr1796	slr2047
sll0362	sll1327	sll1262	sll1299	slr0434	slr1835	sll0745	smr0007	slr0741	slr1796
sll1327	slr0958	sll1262	ssl2084	sll1261	slr1835	sll1815	smr0007	slr1510	slr1796
sll1327	slr1720	sll1262	sll2001	sll1099	slr1835	sll1059	smr0007	sll1043	slr1796
sll0179	sll1327	sll1262	sll1430	slr0752	slr1835	slr1123	smr0007	slr0400	slr1796
sll0454	sll1327	sll0573	sll1262	slr0952	slr1835	sll1760	smr0007	sll1415	slr1796
sll1327	sll1553	sll1262	slr1898	slr1511	slr1835	sll0469	smr0007	sll1909	slr1796
sll1327	slr0638	sll0080	sll1262	sll1605	slr1835	slr1517	smr0007	sll0373	slr1796
sll1327	slr0220	sll1262	slr1022	sll0990	slr1835	sll0869	smr0007	slr1796	slr2035
sll1327	slr0357	sll1262	sll1883	sll1633	slr1835	slr1598	smr0007	slr1796	slr2132
sll1327	sll1362	sll1262	slr1133	slr0018	slr1835	sll0868	smr0007	slr0922	slr1796
sll1327	slr1550	sll1262	slr0444	sll0567	slr1835	slr0994	smr0007	slr0838	slr1796

sll1074	sll1327	sll1262	slr2130	slr0884	slr1835	sll0379	smr0007	sll1823	slr1796
sll1327	slr0649	sll1262	sll1669	sll1342	slr1835	sll1676	smr0007	sll1056	slr1796
sll0495	sll1327	sll1262	slr0585	slr1349	slr1835	sll0927	smr0007	slr0520	slr1796
sll1327	sll1425	sll1262	slr2075	sll0593	slr1835	sll0996	smr0007	sll0144	slr1796
sll0502	sll1327	sll1262	slr2076	sll0220	slr1835	sll0844	smr0007	sll0467	slr1796
sll1327	slr1703	sll0416	sll1262	sll0899	slr1835	sll1615	smr0007	slr0711	slr1796
sll0078	sll1327	sll1262	slr0156	slr1756	slr1835	sll0202	smr0007	sll0754	slr1796
sll1327	slr0557	sll1262	slr1641	sll1085	slr1835	slr0902	smr0007	sll0569	slr1796
sll1327	slr1884	sll1262	slr0542	sll1931	slr1835	sll1144	smr0007	sll1277	slr1796
sll1327	slr1031	sll0534	sll1262	sll0083	slr1835	slr0017	smr0007	sll1110	slr1796
sll1327	slr1793	sll1262	slr0165	slr1755	slr1835	slr1424	smr0007	sll1865	slr1796
sll1327	slr0633	sll0030	sll1262	sll0057	slr1835	slr1423	smr0007	slr1228	slr1796
sll1327	slr1278	sll1262	slr0847	sll0017	slr1835	sll2010	smr0007	slr0808	slr1796
sll1327	slr0783	sll1262	slr0553	sll1185	slr1835	slr0528	smr0007	slr0082	slr1796
sll1198	sll1327	sll1262	slr0812	slr0839	slr1835	slr1351	smr0007	slr0742	slr1796
sll0204	sll1327	sll1262	slr1347	sll1876	slr1835	sll0622	smr0007	sll0098	slr1796
sll1327	sll1750	sll1262	slr1348	sll0900	slr1835	sll0631	smr0007	slr1646	slr1796
sll0420	sll1327	sll1262	slr1791	slr0608	slr1835	slr0787	smr0007	slr1130	slr1796
sll1327	slr1256	sll0594	sll1262	slr0652	slr1835	sll1852	smr0007	slr0080	slr1796
sll1327	sll1639	sll1262	slr0550	slr0084	slr1835	slr1090	smr0007	slr0551	slr1796
sll1327	slr1899	sll1058	sll1262	sll1893	slr1835	sll0902	smr0007	sll0145	slr1796
sll0643	sll1327	sll1262	slr1665	slr0500	slr1835	slr0661	smr0007	sll0708	slr1796
sll1327	slr1844	sll1262	slr0536	slr1560	slr1835	slr0526	smr0007	slr0679	slr1796
sll0459	sll1327	sll1262	slr1874	sll1988	slr1835	sll1249	smr0007	slr0722	slr1796
sll0865	sll1327	sll1262	sll1776	slr1289	slr1835	sll0892	smr0007	slr0072	slr1796
sll1327	slr0733	sll0848	sll1262	sll1556	slr1835	sll0660	smr0007	sll1547	slr1796
sll1327	sll1459	sll0897	sll1262	slr1835	ssl3441	slr1779	smr0007	sll1234	slr1796
sll0905	sll1327	sll1262	sll1933	slr0744	slr1835	slr0394	smr0007	sll0616	slr1796
sll0402	ssl2615	sll1262	slr0965	slr0974	slr1835	slr2047	smr0007	slr1796	slr2073
sll0728	ssl2615	sll1262	slr1463	slr1622	slr1835	slr0741	smr0007	slr1639	slr1796
sll0336	ssl2615	sll1098	sll1262	sll1196	slr1835	slr1510	smr0007	sll1108	slr1796
sll1299	ssl2615	sll1262	slr0434	sll0745	slr1835	sll1043	smr0007	sll0362	slr1796
ssl2084	ssl2615	sll1261	sll1262	sll1815	slr1835	slr0400	smr0007	slr0958	slr1796
sll2001	ssl2615	sll1099	sll1262	sll1059	slr1835	sll1415	smr0007	slr1720	slr1796
sll1430	ssl2615	sll1262	slr0752	slr1123	slr1835	sll1909	smr0007	sll0179	slr1796
sll0573	ssl2615	sll1262	slr0952	sll1760	slr1835	sll0373	smr0007	sll0454	slr1796
slr1898	ssl2615	sll1262	slr1511	sll0469	slr1835	slr2035	smr0007	sll1553	slr1796
sll0080	ssl2615	sll1262	sll1605	slr1517	slr1835	slr2132	smr0007	slr0638	slr1796
slr1022	ssl2615	sll0990	sll1262	sll0869	slr1835	slr0922	smr0007	slr0220	slr1796
sll1883	ssl2615	sll1262	sll1633	slr1598	slr1835	slr0838	smr0007	slr0357	slr1796
slr1133	ssl2615	sll1262	slr0018	sll0868	slr1835	sll1823	smr0007	sll1362	slr1796
slr0444	ssl2615	sll0567	sll1262	slr0994	slr1835	sll1056	smr0007	slr1550	slr1796
slr2130	ssl2615	sll1262	slr0884	sll0379	slr1835	slr0520	smr0007	sll1074	slr1796
sll1669	ssl2615	sll1262	sll1342	sll1676	slr1835	sll0144	smr0007	slr0649	slr1796
slr0585	ssl2615	sll1262	slr1349	sll0927	slr1835	sll0467	smr0007	sll0495	slr1796
slr2075	ssl2615	sll0593	sll1262	sll0996	slr1835	slr0711	smr0007	sll1425	slr1796
slr2076	ssl2615	sll0220	sll1262	sll0844	slr1835	sll0754	smr0007	sll0502	slr1796
sll0416	ssl2615	sll0899	sll1262	sll1615	slr1835	sll0569	smr0007	slr1703	slr1796
slr0156	ssl2615	sll1262	slr1756	sll0202	slr1835	sll1277	smr0007	sll0078	slr1796
slr1641	ssl2615	sll1085	sll1262	slr0902	slr1835	sll1110	smr0007	slr0557	slr1796
slr0542	ssl2615	sll1262	sll1931	sll1144	slr1835	sll1865	smr0007	slr1796	slr1884

sll0534	ssl2615	sll0083	sll1262	slr0017	slr1835	slr1228	smr0007	slr1031	slr1796
slr0165	ssl2615	sll1262	slr1755	slr1424	slr1835	slr0808	smr0007	slr1793	slr1796
sll0030	ssl2615	sll0057	sll1262	slr1423	slr1835	slr0082	smr0007	slr0633	slr1796
slr0847	ssl2615	sll0017	sll1262	sll2010	slr1835	slr0742	smr0007	slr1278	slr1796
slr0553	ssl2615	sll1185	sll1262	slr0528	slr1835	sll0098	smr0007	slr0783	slr1796
slr0812	ssl2615	sll1262	slr0839	slr1351	slr1835	slr1646	smr0007	sll1198	slr1796
slr1347	ssl2615	sll1262	sll1876	sll0622	slr1835	slr1130	smr0007	sll0204	slr1796
slr1348	ssl2615	sll0900	sll1262	sll0631	slr1835	slr0080	smr0007	sll1750	slr1796
slr1791	ssl2615	sll1262	slr0608	slr0787	slr1835	slr0551	smr0007	sll0420	slr1796
sll0594	ssl2615	sll1262	slr0652	sll1852	slr1835	sll0145	smr0007	slr1256	slr1796
slr0550	ssl2615	sll1262	slr0084	slr1090	slr1835	sll0708	smr0007	sll1639	slr1796
sll1058	ssl2615	sll1262	sll1893	sll0902	slr1835	slr0679	smr0007	slr1796	slr1899
slr1665	ssl2615	sll1262	slr0500	slr0661	slr1835	slr0722	smr0007	sll0643	slr1796
slr0536	ssl2615	sll1262	slr1560	slr0526	slr1835	slr0072	smr0007	slr1796	slr1844
slr1874	ssl2615	sll1262	sll1988	sll1249	slr1835	sll1547	smr0007	sll0459	slr1796
sll1776	ssl2615	sll1262	slr1289	sll0892	slr1835	sll1234	smr0007	sll0865	slr1796
sll0848	ssl2615	sll1262	sll1556	sll0660	slr1835	sll0616	smr0007	slr0733	slr1796
sll0897	ssl2615	sll1262	ssl3441	slr1779	slr1835	slr2073	smr0007	sll1459	slr1796
sll1933	ssl2615	sll1262	slr0744	slr0394	slr1835	slr1639	smr0007	sll0905	slr1796
slr0965	ssl2615	sll1262	slr0974	slr1835	slr2047	sll1108	smr0007	sll0402	slr1949
slr1463	ssl2615	sll1262	slr1622	slr0741	slr1835	sll0362	smr0007	sll0728	slr1949
sll1098	ssl2615	sll1196	sll1262	slr1510	slr1835	slr0958	smr0007	sll0336	slr1949
slr0434	ssl2615	sll0745	sll1262	sll1043	slr1835	slr1720	smr0007	sll1299	slr1949
sll1261	ssl2615	sll1262	sll1815	slr0400	slr1835	sll0179	smr0007	slr1949	ssl2084
sll1099	ssl2615	sll1059	sll1262	sll1415	slr1835	sll0454	smr0007	sll2001	slr1949
slr0752	ssl2615	sll1262	slr1123	sll1909	slr1835	sll1553	smr0007	sll1430	slr1949
slr0952	ssl2615	sll1262	sll1760	sll0373	slr1835	slr0638	smr0007	sll0573	slr1949
slr1511	ssl2615	sll0469	sll1262	slr1835	slr2035	slr0220	smr0007	slr1898	slr1949
sll1605	ssl2615	sll1262	slr1517	slr1835	slr2132	slr0357	smr0007	sll0080	slr1949
sll0990	ssl2615	sll0869	sll1262	slr0922	slr1835	sll1362	smr0007	slr1022	slr1949
sll1633	ssl2615	sll1262	slr1598	slr0838	slr1835	slr1550	smr0007	sll1883	slr1949
slr0018	ssl2615	sll0868	sll1262	sll1823	slr1835	sll1074	smr0007	slr1133	slr1949
sll0567	ssl2615	sll1262	slr0994	sll1056	slr1835	slr0649	smr0007	slr0444	slr1949
slr0884	ssl2615	sll0379	sll1262	slr0520	slr1835	sll0495	smr0007	slr1949	slr2130
sll1342	ssl2615	sll1262	sll1676	sll0144	slr1835	sll1425	smr0007	sll1669	slr1949
slr1349	ssl2615	sll0927	sll1262	sll0467	slr1835	sll0502	smr0007	slr0585	slr1949
sll0593	ssl2615	sll0996	sll1262	slr0711	slr1835	slr1703	smr0007	slr1949	slr2075
sll0220	ssl2615	sll0844	sll1262	sll0754	slr1835	sll0078	smr0007	slr1949	slr2076
sll0899	ssl2615	sll1262	sll1615	sll0569	slr1835	slr0557	smr0007	sll0416	slr1949
slr1756	ssl2615	sll0202	sll1262	sll1277	slr1835	slr1884	smr0007	slr0156	slr1949
sll1085	ssl2615	sll1262	slr0902	sll1110	slr1835	slr1031	smr0007	slr1641	slr1949
sll1931	ssl2615	sll1144	sll1262	sll1865	slr1835	slr1793	smr0007	slr0542	slr1949
sll0083	ssl2615	sll1262	slr0017	slr1228	slr1835	slr0633	smr0007	sll0534	slr1949
slr1755	ssl2615	sll1262	slr1424	slr0808	slr1835	slr1278	smr0007	slr0165	slr1949
sll0057	ssl2615	sll1262	slr1423	slr0082	slr1835	slr0783	smr0007	sll0030	slr1949
sll0017	ssl2615	sll1262	sll2010	slr0742	slr1835	sll1198	smr0007	slr0847	slr1949
sll1185	ssl2615	sll1262	slr0528	sll0098	slr1835	sll0204	smr0007	slr0553	slr1949
slr0839	ssl2615	sll1262	slr1351	slr1646	slr1835	sll1750	smr0007	slr0812	slr1949
sll1876	ssl2615	sll0622	sll1262	slr1130	slr1835	sll0420	smr0007	slr1347	slr1949
sll0900	ssl2615	sll0631	sll1262	slr0080	slr1835	slr1256	smr0007	slr1348	slr1949
slr0608	ssl2615	sll1262	slr0787	slr0551	slr1835	sll1639	smr0007	slr1791	slr1949

slr0652	ssl2615	sll1262	sll1852	sll0145	slr1835	slr1899	smr0007	sll0594	slr1949
slr0084	ssl2615	sll1262	slr1090	sll0708	slr1835	sll0643	smr0007	slr0550	slr1949
sll1893	ssl2615	sll0902	sll1262	slr0679	slr1835	slr1844	smr0007	sll1058	slr1949
slr0500	ssl2615	sll1262	slr0661	slr0722	slr1835	sll0459	smr0007	slr1665	slr1949
slr1560	ssl2615	sll1262	slr0526	slr0072	slr1835	sll0865	smr0007	slr0536	slr1949
sll1988	ssl2615	sll1249	sll1262	sll1547	slr1835	slr0733	smr0007	slr1874	slr1949
slr1289	ssl2615	sll0892	sll1262	sll1234	slr1835	sll1459	smr0007	sll1776	slr1949
sll1556	ssl2615	sll0660	sll1262	sll0616	slr1835	sll0905	smr0007	sll0848	slr1949
ssl2615	ssl3441	sll1262	slr1779	slr1835	slr2073	sll0402	sml0003	sll0897	slr1949
slr0744	ssl2615	sll1262	slr0394	slr1639	slr1835	sll0728	sml0003	sll1933	slr1949
slr0974	ssl2615	sll1262	slr2047	sll1108	slr1835	sll0336	sml0003	slr0965	slr1949
slr1622	ssl2615	sll1262	slr0741	sll0362	slr1835	sll1299	sml0003	slr1463	slr1949
sll1196	ssl2615	sll1262	slr1510	slr0958	slr1835	sml0003	ssl2084	sll1098	slr1949
sll0745	ssl2615	sll1043	sll1262	slr1720	slr1835	sll2001	sml0003	slr0434	slr1949
sll1815	ssl2615	sll1262	slr0400	sll0179	slr1835	sll1430	sml0003	sll1261	slr1949
sll1059	ssl2615	sll1262	sll1415	sll0454	slr1835	sll0573	sml0003	sll1099	slr1949
slr1123	ssl2615	sll1262	sll1909	sll1553	slr1835	slr1898	sml0003	slr0752	slr1949
sll1760	ssl2615	sll0373	sll1262	slr0638	slr1835	sll0080	sml0003	slr0952	slr1949
sll0469	ssl2615	sll1262	slr2035	slr0220	slr1835	slr1022	sml0003	slr1511	slr1949
slr1517	ssl2615	sll1262	slr2132	slr0357	slr1835	sll1883	sml0003	sll1605	slr1949
sll0869	ssl2615	sll1262	slr0922	sll1362	slr1835	slr1133	sml0003	sll0990	slr1949
slr1598	ssl2615	sll1262	slr0838	slr1550	slr1835	slr0444	sml0003	sll1633	slr1949
sll0868	ssl2615	sll1262	sll1823	sll1074	slr1835	slr2130	sml0003	slr0018	slr1949
slr0994	ssl2615	sll1056	sll1262	slr0649	slr1835	sll1669	sml0003	sll0567	slr1949
sll0379	ssl2615	sll1262	slr0520	sll0495	slr1835	slr0585	sml0003	slr0884	slr1949
sll1676	ssl2615	sll0144	sll1262	sll1425	slr1835	slr2075	sml0003	sll1342	slr1949
sll0927	ssl2615	sll0467	sll1262	sll0502	slr1835	slr2076	sml0003	slr1349	slr1949
sll0996	ssl2615	sll1262	slr0711	slr1703	slr1835	sll0416	sml0003	sll0593	slr1949
sll0844	ssl2615	sll0754	sll1262	sll0078	slr1835	slr0156	sml0003	sll0220	slr1949
sll1615	ssl2615	sll0569	sll1262	slr0557	slr1835	slr1641	sml0003	sll0899	slr1949
sll0202	ssl2615	sll1262	sll1277	slr1835	slr1884	slr0542	sml0003	slr1756	slr1949
slr0902	ssl2615	sll1110	sll1262	slr1031	slr1835	sll0534	sml0003	sll1085	slr1949
sll1144	ssl2615	sll1262	sll1865	slr1793	slr1835	slr0165	sml0003	sll1931	slr1949
slr0017	ssl2615	sll1262	slr1228	slr0633	slr1835	sll0030	sml0003	sll0083	slr1949
slr1424	ssl2615	sll1262	slr0808	slr1278	slr1835	slr0847	sml0003	slr1755	slr1949
slr1423	ssl2615	sll1262	slr0082	slr0783	slr1835	slr0553	sml0003	sll0057	slr1949
sll2010	ssl2615	sll1262	slr0742	sll1198	slr1835	slr0812	sml0003	sll0017	slr1949
slr0528	ssl2615	sll0098	sll1262	sll0204	slr1835	slr1347	sml0003	sll1185	slr1949
slr1351	ssl2615	sll1262	slr1646	sll1750	slr1835	slr1348	sml0003	slr0839	slr1949
sll0622	ssl2615	sll1262	slr1130	sll0420	slr1835	slr1791	sml0003	sll1876	slr1949
sll0631	ssl2615	sll1262	slr0080	slr1256	slr1835	sll0594	sml0003	sll0900	slr1949
slr0787	ssl2615	sll1262	slr0551	sll1639	slr1835	slr0550	sml0003	slr0608	slr1949
sll1852	ssl2615	sll0145	sll1262	slr1835	slr1899	sll1058	sml0003	slr0652	slr1949
slr1090	ssl2615	sll0708	sll1262	sll0643	slr1835	slr1665	sml0003	slr0084	slr1949
sll0902	ssl2615	sll1262	slr0679	slr1835	slr1844	slr0536	sml0003	sll1893	slr1949
slr0661	ssl2615	sll1262	slr0722	sll0459	slr1835	slr1874	sml0003	slr0500	slr1949
slr0526	ssl2615	sll1262	slr0072	sll0865	slr1835	sll1776	sml0003	slr1560	slr1949
sll1249	ssl2615	sll1262	sll1547	slr0733	slr1835	sll0848	sml0003	sll1988	slr1949
sll0892	ssl2615	sll1234	sll1262	sll1459	slr1835	sll0897	sml0003	slr1289	slr1949
sll0660	ssl2615	sll0616	sll1262	sll0905	slr1835	sll1933	sml0003	sll1556	slr1949
slr1779	ssl2615	sll1262	slr2073	sll0402	ssl0563	slr0965	sml0003	slr1949	ssl3441

slr0394	ssl2615	sll1262	slr1639	sll0728	ssl0563	slr1463	sml0003	slr0744	slr1949
slr2047	ssl2615	sll1108	sll1262	sll0336	ssl0563	sll1098	sml0003	slr0974	slr1949
slr0741	ssl2615	sll0362	sll1262	sll1299	ssl0563	slr0434	sml0003	slr1622	slr1949
slr1510	ssl2615	sll1262	slr0958	ssl0563	ssl2084	sll1261	sml0003	sll1196	slr1949
sll1043	ssl2615	sll1262	slr1720	sll2001	ssl0563	sll1099	sml0003	sll0745	slr1949
slr0400	ssl2615	sll0179	sll1262	sll1430	ssl0563	slr0752	sml0003	sll1815	slr1949
sll1415	ssl2615	sll0454	sll1262	sll0573	ssl0563	slr0952	sml0003	sll1059	slr1949
sll1909	ssl2615	sll1262	sll1553	slr1898	ssl0563	slr1511	sml0003	slr1123	slr1949
sll0373	ssl2615	sll1262	slr0638	sll0080	ssl0563	sll1605	sml0003	sll1760	slr1949
slr2035	ssl2615	sll1262	slr0220	slr1022	ssl0563	sll0990	sml0003	sll0469	slr1949
slr2132	ssl2615	sll1262	slr0357	sll1883	ssl0563	sll1633	sml0003	slr1517	slr1949
slr0922	ssl2615	sll1262	sll1362	slr1133	ssl0563	slr0018	sml0003	sll0869	slr1949
slr0838	ssl2615	sll1262	slr1550	slr0444	ssl0563	sll0567	sml0003	slr1598	slr1949
sll1823	ssl2615	sll1074	sll1262	slr2130	ssl0563	slr0884	sml0003	sll0868	slr1949
sll1056	ssl2615	sll0495	sll1262	sll1669	ssl0563	sll1342	sml0003	slr0994	slr1949
slr0520	ssl2615	sll1262	sll1425	slr0585	ssl0563	slr1349	sml0003	sll0379	slr1949
sll0144	ssl2615	sll0502	sll1262	slr2075	ssl0563	sll0593	sml0003	sll1676	slr1949
sll0467	ssl2615	sll1262	slr1703	slr2076	ssl0563	sll0220	sml0003	sll0927	slr1949
slr0711	ssl2615	sll0078	sll1262	sll0416	ssl0563	sll0899	sml0003	sll0996	slr1949
sll0754	ssl2615	sll1262	slr0557	slr0156	ssl0563	slr1756	sml0003	sll0844	slr1949
sll0569	ssl2615	sll1262	slr1884	slr1641	ssl0563	sll1085	sml0003	sll1615	slr1949
sll1277	ssl2615	sll1262	slr1031	slr0542	ssl0563	sll1931	sml0003	sll0202	slr1949
sll1110	ssl2615	sll1262	slr1793	sll0534	ssl0563	sll0083	sml0003	slr0902	slr1949
sll1865	ssl2615	sll1262	slr0633	slr0165	ssl0563	slr1755	sml0003	sll1144	slr1949
slr1228	ssl2615	sll1262	slr1278	sll0030	ssl0563	sll0057	sml0003	slr0017	slr1949
slr0808	ssl2615	sll1262	slr0783	slr0847	ssl0563	sll0017	sml0003	slr1424	slr1949
slr0082	ssl2615	sll1198	sll1262	slr0553	ssl0563	sll1185	sml0003	slr1423	slr1949
slr0742	ssl2615	sll0204	sll1262	slr0812	ssl0563	slr0839	sml0003	sll2010	slr1949
sll0098	ssl2615	sll1262	sll1750	slr1347	ssl0563	sll1876	sml0003	slr0528	slr1949
slr1646	ssl2615	sll0420	sll1262	slr1348	ssl0563	sll0900	sml0003	slr1351	slr1949
slr1130	ssl2615	sll1262	slr1256	slr1791	ssl0563	slr0608	sml0003	sll0622	slr1949
slr0080	ssl2615	sll1262	sll1639	sll0594	ssl0563	slr0652	sml0003	sll0631	slr1949
slr0551	ssl2615	sll1262	slr1899	slr0550	ssl0563	slr0084	sml0003	slr0787	slr1949
sll0145	ssl2615	sll0643	sll1262	sll1058	ssl0563	sll1893	sml0003	sll1852	slr1949
sll0708	ssl2615	sll1262	slr1844	slr1665	ssl0563	slr0500	sml0003	slr1090	slr1949
slr0679	ssl2615	sll0459	sll1262	slr0536	ssl0563	slr1560	sml0003	sll0902	slr1949
slr0722	ssl2615	sll0865	sll1262	slr1874	ssl0563	sll1988	sml0003	slr0661	slr1949
slr0072	ssl2615	sll1262	slr0733	sll1776	ssl0563	slr1289	sml0003	slr0526	slr1949
sll1547	ssl2615	sll1262	sll1459	sll0848	ssl0563	sll1556	sml0003	sll1249	slr1949
sll1234	ssl2615	sll0905	sll1262	sll0897	ssl0563	sml0003	ssl3441	sll0892	slr1949
sll0616	ssl2615	sll0402	ssl1690	sll1933	ssl0563	slr0744	sml0003	sll0660	slr1949
slr2073	ssl2615	sll0728	ssl1690	slr0965	ssl0563	slr0974	sml0003	slr1779	slr1949
slr1639	ssl2615	sll0336	ssl1690	slr1463	ssl0563	slr1622	sml0003	slr0394	slr1949
sll1108	ssl2615	sll1299	ssl1690	sll1098	ssl0563	sll1196	sml0003	slr1949	slr2047
sll0362	ssl2615	ssl1690	ssl2084	slr0434	ssl0563	sll0745	sml0003	slr0741	slr1949
slr0958	ssl2615	sll2001	ssl1690	sll1261	ssl0563	sll1815	sml0003	slr1510	slr1949
slr1720	ssl2615	sll1430	ssl1690	sll1099	ssl0563	sll1059	sml0003	sll1043	slr1949
sll0179	ssl2615	sll0573	ssl1690	slr0752	ssl0563	slr1123	sml0003	slr0400	slr1949
sll0454	ssl2615	slr1898	ssl1690	slr0952	ssl0563	sll1760	sml0003	sll1415	slr1949
sll1553	ssl2615	sll0080	ssl1690	slr1511	ssl0563	sll0469	sml0003	sll1909	slr1949
slr0638	ssl2615	slr1022	ssl1690	sll1605	ssl0563	slr1517	sml0003	sll0373	slr1949

slr0220	ssl2615	sll1883	ssl1690	sll0990	ssl0563	sll0869	sml0003	slr1949	slr2035
slr0357	ssl2615	slr1133	ssl1690	sll1633	ssl0563	slr1598	sml0003	slr1949	slr2132
sll1362	ssl2615	slr0444	ssl1690	slr0018	ssl0563	sll0868	sml0003	slr0922	slr1949
slr1550	ssl2615	slr2130	ssl1690	sll0567	ssl0563	slr0994	sml0003	slr0838	slr1949
sll1074	ssl2615	sll1669	ssl1690	slr0884	ssl0563	sll0379	sml0003	sll1823	slr1949
slr0649	ssl2615	slr0585	ssl1690	sll1342	ssl0563	sll1676	sml0003	sll1056	slr1949
sll0495	ssl2615	slr2075	ssl1690	slr1349	ssl0563	sll0927	sml0003	slr0520	slr1949
sll1425	ssl2615	slr2076	ssl1690	sll0593	ssl0563	sll0996	sml0003	sll0144	slr1949
sll0502	ssl2615	sll0416	ssl1690	sll0220	ssl0563	sll0844	sml0003	sll0467	slr1949
slr1703	ssl2615	slr0156	ssl1690	sll0899	ssl0563	sll1615	sml0003	slr0711	slr1949
sll0078	ssl2615	slr1641	ssl1690	slr1756	ssl0563	sll0202	sml0003	sll0754	slr1949
slr0557	ssl2615	slr0542	ssl1690	sll1085	ssl0563	slr0902	sml0003	sll0569	slr1949
slr1884	ssl2615	sll0534	ssl1690	sll1931	ssl0563	sll1144	sml0003	sll1277	slr1949
slr1031	ssl2615	slr0165	ssl1690	sll0083	ssl0563	slr0017	sml0003	sll1110	slr1949
slr1793	ssl2615	sll0030	ssl1690	slr1755	ssl0563	slr1424	sml0003	sll1865	slr1949
slr0633	ssl2615	slr0847	ssl1690	sll0057	ssl0563	slr1423	sml0003	slr1228	slr1949
slr1278	ssl2615	slr0553	ssl1690	sll0017	ssl0563	sll2010	sml0003	slr0808	slr1949
slr0783	ssl2615	slr0812	ssl1690	sll1185	ssl0563	slr0528	sml0003	slr0082	slr1949
sll1198	ssl2615	slr1347	ssl1690	slr0839	ssl0563	slr1351	sml0003	slr0742	slr1949
sll0204	ssl2615	slr1348	ssl1690	sll1876	ssl0563	sll0622	sml0003	sll0098	slr1949
sll1750	ssl2615	slr1791	ssl1690	sll0900	ssl0563	sll0631	sml0003	slr1646	slr1949
sll0420	ssl2615	sll0594	ssl1690	slr0608	ssl0563	slr0787	sml0003	slr1130	slr1949
slr1256	ssl2615	slr0550	ssl1690	slr0652	ssl0563	sll1852	sml0003	slr0080	slr1949
sll1639	ssl2615	sll1058	ssl1690	slr0084	ssl0563	slr1090	sml0003	slr0551	slr1949
slr1899	ssl2615	slr1665	ssl1690	sll1893	ssl0563	sll0902	sml0003	sll0145	slr1949
sll0643	ssl2615	slr0536	ssl1690	slr0500	ssl0563	slr0661	sml0003	sll0708	slr1949
slr1844	ssl2615	slr1874	ssl1690	slr1560	ssl0563	slr0526	sml0003	slr0679	slr1949
sll0459	ssl2615	sll1776	ssl1690	sll1988	ssl0563	sll1249	sml0003	slr0722	slr1949
sll0865	ssl2615	sll0848	ssl1690	slr1289	ssl0563	sll0892	sml0003	slr0072	slr1949
slr0733	ssl2615	sll0897	ssl1690	sll1556	ssl0563	sll0660	sml0003	sll1547	slr1949
sll1459	ssl2615	sll1933	ssl1690	ssl0563	ssl3441	slr1779	sml0003	sll1234	slr1949
sll0905	ssl2615	slr0965	ssl1690	slr0744	ssl0563	slr0394	sml0003	sll0616	slr1949
sll0402	sll1323	slr1463	ssl1690	slr0974	ssl0563	slr2047	sml0003	slr1949	slr2073
sll0728	sll1323	sll1098	ssl1690	slr1622	ssl0563	slr0741	sml0003	slr1639	slr1949
sll0336	sll1323	slr0434	ssl1690	sll1196	ssl0563	slr1510	sml0003	sll1108	slr1949
sll1299	sll1323	sll1261	ssl1690	sll0745	ssl0563	sll1043	sml0003	sll0362	slr1949
sll1323	ssl2084	sll1099	ssl1690	sll1815	ssl0563	slr0400	sml0003	slr0958	slr1949
sll1323	sll2001	slr0752	ssl1690	sll1059	ssl0563	sll1415	sml0003	slr1720	slr1949
sll1323	sll1430	slr0952	ssl1690	slr1123	ssl0563	sll1909	sml0003	sll0179	slr1949
sll0573	sll1323	slr1511	ssl1690	sll1760	ssl0563	sll0373	sml0003	sll0454	slr1949
sll1323	slr1898	sll1605	ssl1690	sll0469	ssl0563	slr2035	sml0003	sll1553	slr1949
sll0080	sll1323	sll0990	ssl1690	slr1517	ssl0563	slr2132	sml0003	slr0638	slr1949
sll1323	slr1022	sll1633	ssl1690	sll0869	ssl0563	slr0922	sml0003	slr0220	slr1949
sll1323	sll1883	slr0018	ssl1690	slr1598	ssl0563	slr0838	sml0003	slr0357	slr1949
sll1323	slr1133	sll0567	ssl1690	sll0868	ssl0563	sll1823	sml0003	sll1362	slr1949
sll1323	slr0444	slr0884	ssl1690	slr0994	ssl0563	sll1056	sml0003	slr1550	slr1949
sll1323	slr2130	sll1342	ssl1690	sll0379	ssl0563	slr0520	sml0003	sll1074	slr1949
sll1323	sll1669	slr1349	ssl1690	sll1676	ssl0563	sll0144	sml0003	slr0649	slr1949
sll1323	slr0585	sll0593	ssl1690	sll0927	ssl0563	sll0467	sml0003	sll0495	slr1949
sll1323	slr2075	sll0220	ssl1690	sll0996	ssl0563	slr0711	sml0003	sll1425	slr1949
sll1323	slr2076	sll0899	ssl1690	sll0844	ssl0563	sll0754	sml0003	sll0502	slr1949

sll0416	sll1323
sll1323	slr0156
sll1323	slr1641
sll1323	slr0542
sll0534	sll1323
sll1323	slr0165
sll0030	sll1323
sll1323	slr0847
sll1323	slr0553
sll1323	slr0812
sll1323	slr1347
sll1323	slr1348
sll1323	slr1791
sll0594	sll1323
sll1323	slr0550
sll1058	sll1323
sll1323	slr1665
sll1323	slr0536
sll1323	slr1874
sll1323	sll1776
sll0848	sll1323
sll0897	sll1323
sll1323	sll1933
sll1323	slr0965
sll1323	slr1463
sll1098	sll1323
sll1323	slr0434
sll1261	sll1323
sll1099	sll1323
sll1323	slr0752
sll1323	slr0952
sll1323	slr1511
sll1323	sll1605
sll0990	sll1323
sll1323	sll1633
sll1323	slr0018
sll0567	sll1323
sll1323	slr0884
sll1323	sll1342
sll1323	slr1349
sll0593	sll1323
sll0220	sll1323
sll0899	sll1323
sll1323	slr1756
sll1085	sll1323
sll1323	sll1931
sll0083	sll1323
sll1323	slr1755
sll0057	sll1323
sll0017	sll1323
sll1185	sll1323

slr1756	ssl1690
sll1085	ssl1690
sll1931	ssl1690
sll0083	ssl1690
slr1755	ssl1690
sll0057	ssl1690
sll0017	ssl1690
sll1185	ssl1690
slr0839	ssl1690
sll1876	ssl1690
sll0900	ssl1690
slr0608	ssl1690
slr0652	ssl1690
slr0084	ssl1690
sll1893	ssl1690
slr0500	ssl1690
slr1560	ssl1690
sll1988	ssl1690
slr1289	ssl1690
sll1556	ssl1690
ssl1690	ssl3441
slr0744	ssl1690
slr0974	ssl1690
slr1622	ssl1690
sll1196	ssl1690
sll0745	ssl1690
sll1815	ssl1690
sll1059	ssl1690
slr1123	ssl1690
sll1760	ssl1690
sll0469	ssl1690
slr1517	ssl1690
sll0869	ssl1690
slr1598	ssl1690
sll0868	ssl1690
slr0994	ssl1690
sll0379	ssl1690
sll1676	ssl1690
sll0927	ssl1690
sll0996	ssl1690
sll0844	ssl1690
sll1615	ssl1690
sll0202	ssl1690
slr0902	ssl1690
sll1144	ssl1690
slr0017	ssl1690
slr1424	ssl1690
slr1423	ssl1690
sll2010	ssl1690
slr0528	ssl1690
slr1351	ssl1690

sll1615	ssl0563
sll0202	ssl0563
slr0902	ssl0563
sll1144	ssl0563
slr0017	ssl0563
slr1424	ssl0563
slr1423	ssl0563
sll2010	ssl0563
slr0528	ssl0563
slr1351	ssl0563
sll0622	ssl0563
sll0631	ssl0563
slr0787	ssl0563
sll1852	ssl0563
slr1090	ssl0563
sll0902	ssl0563
slr0661	ssl0563
slr0526	ssl0563
sll1249	ssl0563
sll0892	ssl0563
sll0660	ssl0563
slr1779	ssl0563
slr0394	ssl0563
slr2047	ssl0563
slr0741	ssl0563
slr1510	ssl0563
sll1043	ssl0563
slr0400	ssl0563
sll1415	ssl0563
sll1909	ssl0563
sll0373	ssl0563
slr2035	ssl0563
slr2132	ssl0563
slr0922	ssl0563
slr0838	ssl0563
sll1823	ssl0563
sll1056	ssl0563
slr0520	ssl0563
sll0144	ssl0563
sll0467	ssl0563
slr0711	ssl0563
sll0754	ssl0563
sll0569	ssl0563
sll1277	ssl0563
sll1110	ssl0563
sll1865	ssl0563
slr1228	ssl0563
slr0808	ssl0563
slr0082	ssl0563
slr0742	ssl0563
sll0098	ssl0563

sll0569	sml0003
sll1277	sml0003
sll1110	sml0003
sll1865	sml0003
slr1228	sml0003
slr0808	sml0003
slr0082	sml0003
slr0742	sml0003
sll0098	sml0003
slr1646	sml0003
slr1130	sml0003
slr0080	sml0003
slr0551	sml0003
sll0145	sml0003
sll0708	sml0003
slr0679	sml0003
slr0722	sml0003
slr0072	sml0003
sll1547	sml0003
sll1234	sml0003
sll0616	sml0003
slr2073	sml0003
slr1639	sml0003
sll1108	sml0003
sll0362	sml0003
slr0958	sml0003
slr1720	sml0003
sll0179	sml0003
sll0454	sml0003
sll1553	sml0003
slr0638	sml0003
slr0220	sml0003
slr0357	sml0003
sll1362	sml0003
slr1550	sml0003
sll1074	sml0003
slr0649	sml0003
sll0495	sml0003
sll1425	sml0003
sll0502	sml0003
slr1703	sml0003
sll0078	sml0003
slr0557	sml0003
slr1884	sml0003
slr1031	sml0003
slr1793	sml0003
slr0633	sml0003
slr1278	sml0003
slr0783	sml0003
sll1198	sml0003
sll0204	sml0003

slr1703	slr1949
sll0078	slr1949
slr0557	slr1949
slr1884	slr1949
slr1031	slr1949
slr1793	slr1949
slr0633	slr1949
slr1278	slr1949
slr0783	slr1949
sll1198	slr1949
sll0204	slr1949
sll1750	slr1949
sll0420	slr1949
slr1256	slr1949
sll1639	slr1949
slr1899	slr1949
sll0643	slr1949
slr1844	slr1949
sll0459	slr1949
sll0865	slr1949
slr0733	slr1949
sll1459	slr1949
sll0905	slr1949
sll0402	ssl2009
sll0728	ssl2009
sll0336	ssl2009
sll1299	ssl2009
ssl2009	ssl2084
sll2001	ssl2009
sll1430	ssl2009
sll0573	ssl2009
slr1898	ssl2009
sll0080	ssl2009
slr1022	ssl2009
sll1883	ssl2009
slr1133	ssl2009
slr0444	ssl2009
slr2130	ssl2009
sll1669	ssl2009
slr0585	ssl2009
slr2075	ssl2009
slr2076	ssl2009
sll0416	ssl2009
slr0156	ssl2009
slr1641	ssl2009
slr0542	ssl2009
sll0534	ssl2009
slr0165	ssl2009
sll0030	ssl2009
slr0847	ssl2009
slr0553	ssl2009

sll1323	slr0839	sll0622	ssl1690	slr1646	ssl0563	sll1750	sml0003	slr0812	ssl2009
sll1323	sll1876	sll0631	ssl1690	slr1130	ssl0563	sll0420	sml0003	slr1347	ssl2009
sll0900	sll1323	slr0787	ssl1690	slr0080	ssl0563	slr1256	sml0003	slr1348	ssl2009
sll1323	slr0608	sll1852	ssl1690	slr0551	ssl0563	sll1639	sml0003	slr1791	ssl2009
sll1323	slr0652	slr1090	ssl1690	sll0145	ssl0563	slr1899	sml0003	sll0594	ssl2009
sll1323	slr0084	sll0902	ssl1690	sll0708	ssl0563	sll0643	sml0003	slr0550	ssl2009
sll1323	sll1893	slr0661	ssl1690	slr0679	ssl0563	slr1844	sml0003	sll1058	ssl2009
sll1323	slr0500	slr0526	ssl1690	slr0722	ssl0563	sll0459	sml0003	slr1665	ssl2009
sll1323	slr1560	sll1249	ssl1690	slr0072	ssl0563	sll0865	sml0003	slr0536	ssl2009
sll1323	sll1988	sll0892	ssl1690	sll1547	ssl0563	slr0733	sml0003	slr1874	ssl2009
sll1323	slr1289	sll0660	ssl1690	sll1234	ssl0563	sll1459	sml0003	sll1776	ssl2009
sll1323	sll1556	slr1779	ssl1690	sll0616	ssl0563	sll0905	sml0003	sll0848	ssl2009
sll1323	ssl3441	slr0394	ssl1690	slr2073	ssl0563	sll0402	smr0009	sll0897	ssl2009
sll1323	slr0744	slr2047	ssl1690	slr1639	ssl0563	sll0728	smr0009	sll1933	ssl2009
sll1323	slr0974	slr0741	ssl1690	sll1108	ssl0563	sll0336	smr0009	slr0965	ssl2009
sll1323	slr1622	slr1510	ssl1690	sll0362	ssl0563	sll1299	smr0009	slr1463	ssl2009
sll1196	sll1323	sll1043	ssl1690	slr0958	ssl0563	smr0009	ssl2084	sll1098	ssl2009
sll0745	sll1323	slr0400	ssl1690	slr1720	ssl0563	sll2001	smr0009	slr0434	ssl2009
sll1323	sll1815	sll1415	ssl1690	sll0179	ssl0563	sll1430	smr0009	sll1261	ssl2009
sll1059	sll1323	sll1909	ssl1690	sll0454	ssl0563	sll0573	smr0009	sll1099	ssl2009
sll1323	slr1123	sll0373	ssl1690	sll1553	ssl0563	slr1898	smr0009	slr0752	ssl2009
sll1323	sll1760	slr2035	ssl1690	slr0638	ssl0563	sll0080	smr0009	slr0952	ssl2009
sll0469	sll1323	slr2132	ssl1690	slr0220	ssl0563	slr1022	smr0009	slr1511	ssl2009
sll1323	slr1517	slr0922	ssl1690	slr0357	ssl0563	sll1883	smr0009	sll1605	ssl2009
sll0869	sll1323	slr0838	ssl1690	sll1362	ssl0563	slr1133	smr0009	sll0990	ssl2009
sll1323	slr1598	sll1823	ssl1690	slr1550	ssl0563	slr0444	smr0009	sll1633	ssl2009
sll0868	sll1323	sll1056	ssl1690	sll1074	ssl0563	slr2130	smr0009	slr0018	ssl2009
sll1323	slr0994	slr0520	ssl1690	slr0649	ssl0563	sll1669	smr0009	sll0567	ssl2009
sll0379	sll1323	sll0144	ssl1690	sll0495	ssl0563	slr0585	smr0009	slr0884	ssl2009
sll1323	sll1676	sll0467	ssl1690	sll1425	ssl0563	slr2075	smr0009	sll1342	ssl2009
sll0927	sll1323	slr0711	ssl1690	sll0502	ssl0563	slr2076	smr0009	slr1349	ssl2009
sll0996	sll1323	sll0754	ssl1690	slr1703	ssl0563	sll0416	smr0009	sll0593	ssl2009
sll0844	sll1323	sll0569	ssl1690	sll0078	ssl0563	slr0156	smr0009	sll0220	ssl2009
sll1323	sll1615	sll1277	ssl1690	slr0557	ssl0563	slr1641	smr0009	sll0899	ssl2009
sll0202	sll1323	sll1110	ssl1690	slr1884	ssl0563	slr0542	smr0009	slr1756	ssl2009
sll1323	slr0902	sll1865	ssl1690	slr1031	ssl0563	sll0534	smr0009	sll1085	ssl2009
sll1144	sll1323	slr1228	ssl1690	slr1793	ssl0563	slr0165	smr0009	sll1931	ssl2009
sll1323	slr0017	slr0808	ssl1690	slr0633	ssl0563	sll0030	smr0009	sll0083	ssl2009
sll1323	slr1424	slr0082	ssl1690	slr1278	ssl0563	slr0847	smr0009	slr1755	ssl2009
sll1323	slr1423	slr0742	ssl1690	slr0783	ssl0563	slr0553	smr0009	sll0057	ssl2009
sll1323	sll2010	sll0098	ssl1690	sll1198	ssl0563	slr0812	smr0009	sll0017	ssl2009
sll1323	slr0528	slr1646	ssl1690	sll0204	ssl0563	slr1347	smr0009	sll1185	ssl2009
sll1323	slr1351	slr1130	ssl1690	sll1750	ssl0563	slr1348	smr0009	slr0839	ssl2009
sll0622	sll1323	slr0080	ssl1690	sll0420	ssl0563	slr1791	smr0009	sll1876	ssl2009
sll0631	sll1323	slr0551	ssl1690	slr1256	ssl0563	sll0594	smr0009	sll0900	ssl2009
sll1323	slr0787	sll0145	ssl1690	sll1639	ssl0563	slr0550	smr0009	slr0608	ssl2009
sll1323	sll1852	sll0708	ssl1690	slr1899	ssl0563	sll1058	smr0009	slr0652	ssl2009
sll1323	slr1090	slr0679	ssl1690	sll0643	ssl0563	slr1665	smr0009	slr0084	ssl2009
sll0902	sll1323	slr0722	ssl1690	slr1844	ssl0563	slr0536	smr0009	sll1893	ssl2009
sll1323	slr0661	slr0072	ssl1690	sll0459	ssl0563	slr1874	smr0009	slr0500	ssl2009
sll1323	slr0526	sll1547	ssl1690	sll0865	ssl0563	sll1776	smr0009	slr1560	ssl2009

sll1249	sll1323	sll1234	ssl1690	slr0733	ssl0563	sll0848	smr0009	sll1988	ssl2009
sll0892	sll1323	sll0616	ssl1690	sll1459	ssl0563	sll0897	smr0009	slr1289	ssl2009
sll0660	sll1323	slr2073	ssl1690	sll0905	ssl0563	sll1933	smr0009	sll1556	ssl2009
sll1323	slr1779	slr1639	ssl1690	sll0402	ssr2831	slr0965	smr0009	ssl2009	ssl3441
sll1323	slr0394	sll1108	ssl1690	sll0728	ssr2831	slr1463	smr0009	slr0744	ssl2009
sll1323	slr2047	sll0362	ssl1690	sll0336	ssr2831	sll1098	smr0009	slr0974	ssl2009
sll1323	slr0741	slr0958	ssl1690	sll1299	ssr2831	slr0434	smr0009	slr1622	ssl2009
sll1323	slr1510	slr1720	ssl1690	ssl2084	ssr2831	sll1261	smr0009	sll1196	ssl2009
sll1043	sll1323	sll0179	ssl1690	sll2001	ssr2831	sll1099	smr0009	sll0745	ssl2009
sll1323	slr0400	sll0454	ssl1690	sll1430	ssr2831	slr0752	smr0009	sll1815	ssl2009
sll1323	sll1415	sll1553	ssl1690	sll0573	ssr2831	slr0952	smr0009	sll1059	ssl2009
sll1323	sll1909	slr0638	ssl1690	slr1898	ssr2831	slr1511	smr0009	slr1123	ssl2009
sll0373	sll1323	slr0220	ssl1690	sll0080	ssr2831	sll1605	smr0009	sll1760	ssl2009
sll1323	slr2035	slr0357	ssl1690	slr1022	ssr2831	sll0990	smr0009	sll0469	ssl2009
sll1323	slr2132	sll1362	ssl1690	sll1883	ssr2831	sll1633	smr0009	slr1517	ssl2009
sll1323	slr0922	slr1550	ssl1690	slr1133	ssr2831	slr0018	smr0009	sll0869	ssl2009
sll1323	slr0838	sll1074	ssl1690	slr0444	ssr2831	sll0567	smr0009	slr1598	ssl2009
sll1323	sll1823	slr0649	ssl1690	slr2130	ssr2831	slr0884	smr0009	sll0868	ssl2009
sll1056	sll1323	sll0495	ssl1690	sll1669	ssr2831	sll1342	smr0009	slr0994	ssl2009
sll1323	slr0520	sll1425	ssl1690	slr0585	ssr2831	slr1349	smr0009	sll0379	ssl2009
sll0144	sll1323	sll0502	ssl1690	slr2075	ssr2831	sll0593	smr0009	sll1676	ssl2009
sll0467	sll1323	slr1703	ssl1690	slr2076	ssr2831	sll0220	smr0009	sll0927	ssl2009
sll1323	slr0711	sll0078	ssl1690	sll0416	ssr2831	sll0899	smr0009	sll0996	ssl2009
sll0754	sll1323	slr0557	ssl1690	slr0156	ssr2831	slr1756	smr0009	sll0844	ssl2009
sll0569	sll1323	slr1884	ssl1690	slr1641	ssr2831	sll1085	smr0009	sll1615	ssl2009
sll1277	sll1323	slr1031	ssl1690	slr0542	ssr2831	sll1931	smr0009	sll0202	ssl2009
sll1110	sll1323	slr1793	ssl1690	sll0534	ssr2831	sll0083	smr0009	slr0902	ssl2009
sll1323	sll1865	slr0633	ssl1690	slr0165	ssr2831	slr1755	smr0009	sll1144	ssl2009
sll1323	slr1228	slr1278	ssl1690	sll0030	ssr2831	sll0057	smr0009	slr0017	ssl2009
sll1323	slr0808	slr0783	ssl1690	slr0847	ssr2831	sll0017	smr0009	slr1424	ssl2009
sll1323	slr0082	sll1198	ssl1690	slr0553	ssr2831	sll1185	smr0009	slr1423	ssl2009
sll1323	slr0742	sll0204	ssl1690	slr0812	ssr2831	slr0839	smr0009	sll2010	ssl2009
sll0098	sll1323	sll1750	ssl1690	slr1347	ssr2831	sll1876	smr0009	slr0528	ssl2009
sll1323	slr1646	sll0420	ssl1690	slr1348	ssr2831	sll0900	smr0009	slr1351	ssl2009
sll1323	slr1130	slr1256	ssl1690	slr1791	ssr2831	slr0608	smr0009	sll0622	ssl2009
sll1323	slr0080	sll1639	ssl1690	sll0594	ssr2831	slr0652	smr0009	sll0631	ssl2009
sll1323	slr0551	slr1899	ssl1690	slr0550	ssr2831	slr0084	smr0009	slr0787	ssl2009
sll0145	sll1323	sll0643	ssl1690	sll1058	ssr2831	sll1893	smr0009	sll1852	ssl2009
sll0708	sll1323	slr1844	ssl1690	slr1665	ssr2831	slr0500	smr0009	slr1090	ssl2009
sll1323	slr0722	sll0459	ssl1690	slr0536	ssr2831	slr1560	smr0009	sll0902	ssl2009
sll1323	slr0072	sll0865	ssl1690	slr1874	ssr2831	sll1988	smr0009	slr0661	ssl2009
sll1323	sll1547	slr0733	ssl1690	sll1776	ssr2831	slr1289	smr0009	slr0526	ssl2009
sll1234	sll1323	sll1459	ssl1690	sll0848	ssr2831	sll1556	smr0009	sll1249	ssl2009
sll0616	sll1323	sll0905	ssl1690	sll0897	ssr2831	smr0009	ssl3441	sll0892	ssl2009
sll1323	slr2073	sll0402	sll0519	sll1933	ssr2831	slr0744	smr0009	sll0660	ssl2009
sll1323	slr1639	sll0519	sll0728	slr0965	ssr2831	slr0974	smr0009	slr1779	ssl2009
sll1108	sll1323	sll0336	sll0519	slr1463	ssr2831	slr1622	smr0009	slr0394	ssl2009
sll0362	sll1323	sll0519	sll1299	sll1098	ssr2831	sll1196	smr0009	slr2047	ssl2009
sll1323	slr0958	sll0519	ssl2084	slr0434	ssr2831	sll0745	smr0009	slr0741	ssl2009
sll1323	slr1720	sll0519	sll2001	sll1261	ssr2831	sll1815	smr0009	slr1510	ssl2009
sll0179	sll1323	sll0519	sll1430	sll1099	ssr2831	sll1059	smr0009	sll1043	ssl2009

sll0454	sll1323	sll0519	sll0573	slr0752	ssr2831	slr1123	smr0009	slr0400	ssl2009
sll1323	sll1553	sll0519	slr1898	slr0952	ssr2831	sll1760	smr0009	sll1415	ssl2009
sll1323	slr0638	sll0080	sll0519	slr1511	ssr2831	sll0469	smr0009	sll1909	ssl2009
sll1323	slr0220	sll0519	slr1022	sll1605	ssr2831	slr1517	smr0009	sll0373	ssl2009
sll1323	slr0357	sll0519	sll1883	sll0990	ssr2831	sll0869	smr0009	slr2035	ssl2009
sll1323	sll1362	sll0519	slr1133	sll1633	ssr2831	slr1598	smr0009	slr2132	ssl2009
sll1323	slr1550	sll0519	slr0444	slr0018	ssr2831	sll0868	smr0009	slr0922	ssl2009
sll1074	sll1323	sll0519	slr2130	sll0567	ssr2831	slr0994	smr0009	slr0838	ssl2009
sll1323	slr0649	sll0519	sll1669	slr0884	ssr2831	sll0379	smr0009	sll1823	ssl2009
sll0495	sll1323	sll0519	slr0585	sll1342	ssr2831	sll1676	smr0009	sll1056	ssl2009
sll1323	sll1425	sll0519	slr2075	slr1349	ssr2831	sll0927	smr0009	slr0520	ssl2009
sll0502	sll1323	sll0519	slr2076	sll0593	ssr2831	sll0996	smr0009	sll0144	ssl2009
sll1323	slr1703	sll0416	sll0519	sll0220	ssr2831	sll0844	smr0009	sll0467	ssl2009
sll0078	sll1323	sll0519	slr0156	sll0899	ssr2831	sll1615	smr0009	slr0711	ssl2009
sll1323	slr0557	sll0519	slr1641	slr1756	ssr2831	sll0202	smr0009	sll0754	ssl2009
sll1323	slr1884	sll0519	slr0542	sll1085	ssr2831	slr0902	smr0009	sll0569	ssl2009
sll1323	slr1031	sll0519	sll0534	sll1931	ssr2831	sll1144	smr0009	sll1277	ssl2009
sll1323	slr1793	sll0519	slr0165	sll0083	ssr2831	slr0017	smr0009	sll1110	ssl2009
sll1323	slr0633	sll0030	sll0519	slr1755	ssr2831	slr1424	smr0009	sll1865	ssl2009
sll1323	slr1278	sll0519	slr0847	sll0057	ssr2831	slr1423	smr0009	slr1228	ssl2009
sll1323	slr0783	sll0519	slr0553	sll0017	ssr2831	sll2010	smr0009	slr0808	ssl2009
sll1198	sll1323	sll0519	slr0812	sll1185	ssr2831	slr0528	smr0009	slr0082	ssl2009
sll0204	sll1323	sll0519	slr1347	slr0839	ssr2831	slr1351	smr0009	slr0742	ssl2009
sll1323	sll1750	sll0519	slr1348	sll1876	ssr2831	sll0622	smr0009	sll0098	ssl2009
sll0420	sll1323	sll0519	slr1791	sll0900	ssr2831	sll0631	smr0009	slr1646	ssl2009
sll1323	slr1256	sll0519	sll0594	slr0608	ssr2831	slr0787	smr0009	slr1130	ssl2009
sll1323	sll1639	sll0519	slr0550	slr0652	ssr2831	sll1852	smr0009	slr0080	ssl2009
sll1323	slr1899	sll0519	sll1058	slr0084	ssr2831	slr1090	smr0009	slr0551	ssl2009
sll0643	sll1323	sll0519	slr1665	sll1893	ssr2831	sll0902	smr0009	sll0145	ssl2009
sll1323	slr1844	sll0519	slr0536	slr0500	ssr2831	slr0661	smr0009	sll0708	ssl2009
sll0459	sll1323	sll0519	slr1874	slr1560	ssr2831	slr0526	smr0009	slr0679	ssl2009
sll0865	sll1323	sll0519	sll1776	sll1988	ssr2831	sll1249	smr0009	slr0722	ssl2009
sll1323	slr0733	sll0519	sll0848	slr1289	ssr2831	sll0892	smr0009	slr0072	ssl2009
sll1323	sll1459	sll0519	sll0897	sll1556	ssr2831	sll0660	smr0009	sll1547	ssl2009
sll0905	sll1323	sll0519	sll1933	ssl3441	ssr2831	slr1779	smr0009	sll1234	ssl2009
sll0402	slr2087	sll0519	slr0965	slr0744	ssr2831	slr0394	smr0009	sll0616	ssl2009
sll0728	slr2087	sll0519	slr1463	slr0974	ssr2831	slr2047	smr0009	slr2073	ssl2009
sll0336	slr2087	sll0519	sll1098	slr1622	ssr2831	slr0741	smr0009	slr1639	ssl2009
sll1299	slr2087	sll0519	slr0434	sll1196	ssr2831	slr1510	smr0009	sll1108	ssl2009
slr2087	ssl2084	sll0519	sll1261	sll0745	ssr2831	sll1043	smr0009	sll0362	ssl2009
sll2001	slr2087	sll0519	sll1099	sll1815	ssr2831	slr0400	smr0009	slr0958	ssl2009
sll1430	slr2087	sll0519	slr0752	sll1059	ssr2831	sll1415	smr0009	slr1720	ssl2009
sll0573	slr2087	sll0519	slr0952	slr1123	ssr2831	sll1909	smr0009	sll0179	ssl2009
slr1898	slr2087	sll0519	slr1511	sll1760	ssr2831	sll0373	smr0009	sll0454	ssl2009
sll0080	slr2087	sll0519	sll1605	sll0469	ssr2831	slr2035	smr0009	sll1553	ssl2009
slr1022	slr2087	sll0519	sll0990	slr1517	ssr2831	slr2132	smr0009	slr0638	ssl2009
sll1883	slr2087	sll0519	sll1633	sll0869	ssr2831	slr0922	smr0009	slr0220	ssl2009
slr1133	slr2087	sll0519	slr0018	slr1598	ssr2831	slr0838	smr0009	slr0357	ssl2009
slr0444	slr2087	sll0519	sll0567	sll0868	ssr2831	sll1823	smr0009	sll1362	ssl2009
slr2087	slr2130	sll0519	slr0884	slr0994	ssr2831	sll1056	smr0009	slr1550	ssl2009
sll1669	slr2087	sll0519	sll1342	sll0379	ssr2831	slr0520	smr0009	sll1074	ssl2009

slr0585	slr2087	sll0519	slr1349	sll1676	ssr2831	sll0144	smr0009	slr0649	ssl2009
slr2075	slr2087	sll0519	sll0593	sll0927	ssr2831	sll0467	smr0009	sll0495	ssl2009
slr2076	slr2087	sll0220	sll0519	sll0996	ssr2831	slr0711	smr0009	sll1425	ssl2009
sll0416	slr2087	sll0519	sll0899	sll0844	ssr2831	sll0754	smr0009	sll0502	ssl2009
slr0156	slr2087	sll0519	slr1756	sll1615	ssr2831	sll0569	smr0009	slr1703	ssl2009
slr1641	slr2087	sll0519	sll1085	sll0202	ssr2831	sll1277	smr0009	sll0078	ssl2009
slr0542	slr2087	sll0519	sll1931	slr0902	ssr2831	sll1110	smr0009	slr0557	ssl2009
sll0534	slr2087	sll0083	sll0519	sll1144	ssr2831	sll1865	smr0009	slr1884	ssl2009
slr0165	slr2087	sll0519	slr1755	slr0017	ssr2831	slr1228	smr0009	slr1031	ssl2009
sll0030	slr2087	sll0057	sll0519	slr1424	ssr2831	slr0808	smr0009	slr1793	ssl2009
slr0847	slr2087	sll0017	sll0519	slr1423	ssr2831	slr0082	smr0009	slr0633	ssl2009
slr0553	slr2087	sll0519	sll1185	sll2010	ssr2831	slr0742	smr0009	slr1278	ssl2009
slr0812	slr2087	sll0519	slr0839	slr0528	ssr2831	sll0098	smr0009	slr0783	ssl2009
slr1347	slr2087	sll0519	sll1876	slr1351	ssr2831	slr1646	smr0009	sll1198	ssl2009
slr1348	slr2087	sll0519	sll0900	sll0622	ssr2831	slr1130	smr0009	sll0204	ssl2009
slr1791	slr2087	sll0519	slr0608	sll0631	ssr2831	slr0080	smr0009	sll1750	ssl2009
sll0594	slr2087	sll0519	slr0652	slr0787	ssr2831	slr0551	smr0009	sll0420	ssl2009
slr0550	slr2087	sll0519	slr0084	sll1852	ssr2831	sll0145	smr0009	slr1256	ssl2009
sll1058	slr2087	sll0519	sll1893	slr1090	ssr2831	sll0708	smr0009	sll1639	ssl2009
slr1665	slr2087	sll0519	slr0500	sll0902	ssr2831	slr0679	smr0009	slr1899	ssl2009
slr0536	slr2087	sll0519	slr1560	slr0661	ssr2831	slr0722	smr0009	sll0643	ssl2009
slr1874	slr2087	sll0519	sll1988	slr0526	ssr2831	slr0072	smr0009	slr1844	ssl2009
sll1776	slr2087	sll0519	slr1289	sll1249	ssr2831	sll1547	smr0009	sll0459	ssl2009
sll0848	slr2087	sll0519	sll1556	sll0892	ssr2831	sll1234	smr0009	sll0865	ssl2009
sll0897	slr2087	sll0519	ssl3441	sll0660	ssr2831	sll0616	smr0009	slr0733	ssl2009
sll1933	slr2087	sll0519	slr0744	slr1779	ssr2831	slr2073	smr0009	sll1459	ssl2009
slr0965	slr2087	sll0519	slr0974	slr0394	ssr2831	slr1639	smr0009	sll0905	ssl2009
slr1463	slr2087	sll0519	slr1622	slr2047	ssr2831	sll1108	smr0009	sll0402	ssr2422
sll1098	slr2087	sll0519	sll1196	slr0741	ssr2831	sll0362	smr0009	sll0728	ssr2422
slr0434	slr2087	sll0519	sll0745	slr1510	ssr2831	slr0958	smr0009	sll0336	ssr2422
sll1261	slr2087	sll0519	sll1815	sll1043	ssr2831	slr1720	smr0009	sll1299	ssr2422
sll1099	slr2087	sll0519	sll1059	slr0400	ssr2831	sll0179	smr0009	ssl2084	ssr2422
slr0752	slr2087	sll0519	slr1123	sll1415	ssr2831	sll0454	smr0009	sll2001	ssr2422
slr0952	slr2087	sll0519	sll1760	sll1909	ssr2831	sll1553	smr0009	sll1430	ssr2422
slr1511	slr2087	sll0469	sll0519	sll0373	ssr2831	slr0638	smr0009	sll0573	ssr2422
sll1605	slr2087	sll0519	slr1517	slr2035	ssr2831	slr0220	smr0009	slr1898	ssr2422
sll0990	slr2087	sll0519	sll0869	slr2132	ssr2831	slr0357	smr0009	sll0080	ssr2422
sll1633	slr2087	sll0519	slr1598	slr0922	ssr2831	sll1362	smr0009	slr1022	ssr2422
slr0018	slr2087	sll0519	sll0868	slr0838	ssr2831	slr1550	smr0009	sll1883	ssr2422
sll0567	slr2087	sll0519	slr0994	sll1823	ssr2831	sll1074	smr0009	slr1133	ssr2422
slr0884	slr2087	sll0379	sll0519	sll1056	ssr2831	slr0649	smr0009	slr0444	ssr2422
sll1342	slr2087	sll0519	sll1676	slr0520	ssr2831	sll0495	smr0009	slr2130	ssr2422
slr1349	slr2087	sll0519	sll0927	sll0144	ssr2831	sll1425	smr0009	sll1669	ssr2422
sll0593	slr2087	sll0519	sll0996	sll0467	ssr2831	sll0502	smr0009	slr0585	ssr2422
sll0220	slr2087	sll0519	sll0844	slr0711	ssr2831	slr1703	smr0009	slr2075	ssr2422
sll0899	slr2087	sll0519	sll1615	sll0754	ssr2831	sll0078	smr0009	slr2076	ssr2422
slr1756	slr2087	sll0202	sll0519	sll0569	ssr2831	slr0557	smr0009	sll0416	ssr2422
sll1085	slr2087	sll0519	slr0902	sll1277	ssr2831	slr1884	smr0009	slr0156	ssr2422
sll1931	slr2087	sll0519	sll1144	sll1110	ssr2831	slr1031	smr0009	slr1641	ssr2422
sll0083	slr2087	sll0519	slr0017	sll1865	ssr2831	slr1793	smr0009	slr0542	ssr2422
slr1755	slr2087	sll0519	slr1424	slr1228	ssr2831	slr0633	smr0009	sll0534	ssr2422

sll0057	slr2087	sll0519	slr1423	slr0808	ssr2831	slr1278	smr0009	slr0165	ssr2422
sll0017	slr2087	sll0519	sll2010	slr0082	ssr2831	slr0783	smr0009	sll0030	ssr2422
sll1185	slr2087	sll0519	slr0528	slr0742	ssr2831	sll1198	smr0009	slr0847	ssr2422
slr0839	slr2087	sll0519	slr1351	sll0098	ssr2831	sll0204	smr0009	slr0553	ssr2422
sll1876	slr2087	sll0519	sll0622	slr1646	ssr2831	sll1750	smr0009	slr0812	ssr2422
sll0900	slr2087	sll0519	sll0631	slr1130	ssr2831	sll0420	smr0009	slr1347	ssr2422
slr0608	slr2087	sll0519	slr0787	slr0080	ssr2831	slr1256	smr0009	slr1348	ssr2422
slr0652	slr2087	sll0519	sll1852	slr0551	ssr2831	sll1639	smr0009	slr1791	ssr2422
slr0084	slr2087	sll0519	slr1090	sll0145	ssr2831	slr1899	smr0009	sll0594	ssr2422
sll1893	slr2087	sll0519	sll0902	sll0708	ssr2831	sll0643	smr0009	slr0550	ssr2422
slr0500	slr2087	sll0519	slr0661	slr0679	ssr2831	slr1844	smr0009	sll1058	ssr2422
slr1560	slr2087	sll0519	slr0526	slr0722	ssr2831	sll0459	smr0009	slr1665	ssr2422
sll1988	slr2087	sll0519	sll1249	slr0072	ssr2831	sll0865	smr0009	slr0536	ssr2422
slr1289	slr2087	sll0519	sll0892	sll1547	ssr2831	slr0733	smr0009	slr1874	ssr2422
sll1556	slr2087	sll0519	sll0660	sll1234	ssr2831	sll1459	smr0009	sll1776	ssr2422
slr2087	ssl3441	sll0519	slr1779	sll0616	ssr2831	sll0905	smr0009	sll0848	ssr2422
slr0744	slr2087	sll0519	slr0394	slr2073	ssr2831	sll0402	smr0001	sll0897	ssr2422
slr0974	slr2087	sll0519	slr2047	slr1639	ssr2831	sll0728	smr0001	sll1933	ssr2422
slr1622	slr2087	sll0519	slr0741	sll1108	ssr2831	sll0336	smr0001	slr0965	ssr2422
sll1196	slr2087	sll0519	slr1510	sll0362	ssr2831	sll1299	smr0001	slr1463	ssr2422
sll0745	slr2087	sll0519	sll1043	slr0958	ssr2831	smr0001	ssl2084	sll1098	ssr2422
sll1815	slr2087	sll0519	slr0400	slr1720	ssr2831	sll2001	smr0001	slr0434	ssr2422
sll1059	slr2087	sll0519	sll1415	sll0179	ssr2831	sll1430	smr0001	sll1261	ssr2422
slr1123	slr2087	sll0519	sll1909	sll0454	ssr2831	sll0573	smr0001	sll1099	ssr2422
sll1760	slr2087	sll0373	sll0519	sll1553	ssr2831	slr1898	smr0001	slr0752	ssr2422
sll0469	slr2087	sll0519	slr2035	slr0638	ssr2831	sll0080	smr0001	slr0952	ssr2422
slr1517	slr2087	sll0519	slr2132	slr0220	ssr2831	slr1022	smr0001	slr1511	ssr2422
sll0869	slr2087	sll0519	slr0922	slr0357	ssr2831	sll1883	smr0001	sll1605	ssr2422
slr1598	slr2087	sll0519	slr0838	sll1362	ssr2831	slr1133	smr0001	sll0990	ssr2422
sll0868	slr2087	sll0519	sll1823	slr1550	ssr2831	slr0444	smr0001	sll1633	ssr2422
slr0994	slr2087	sll0519	sll1056	sll1074	ssr2831	slr2130	smr0001	slr0018	ssr2422
sll0379	slr2087	sll0519	slr0520	slr0649	ssr2831	sll1669	smr0001	sll0567	ssr2422
sll1676	slr2087	sll0144	sll0519	sll0495	ssr2831	slr0585	smr0001	slr0884	ssr2422
sll0927	slr2087	sll0467	sll0519	sll1425	ssr2831	slr2075	smr0001	sll1342	ssr2422
sll0996	slr2087	sll0519	slr0711	sll0502	ssr2831	slr2076	smr0001	slr1349	ssr2422
sll0844	slr2087	sll0519	sll0754	slr1703	ssr2831	sll0416	smr0001	sll0593	ssr2422
sll1615	slr2087	sll0519	sll0569	sll0078	ssr2831	slr0156	smr0001	sll0220	ssr2422
sll0202	slr2087	sll0519	sll1277	slr0557	ssr2831	slr1641	smr0001	sll0899	ssr2422
slr0902	slr2087	sll0519	sll1110	slr1884	ssr2831	slr0542	smr0001	slr1756	ssr2422
sll1144	slr2087	sll0519	sll1865	slr1031	ssr2831	sll0534	smr0001	sll1085	ssr2422
slr0017	slr2087	sll0519	slr1228	slr1793	ssr2831	slr0165	smr0001	sll1931	ssr2422
slr1424	slr2087	sll0519	slr0808	slr0633	ssr2831	sll0030	smr0001	sll0083	ssr2422
slr1423	slr2087	sll0519	slr0082	slr1278	ssr2831	slr0847	smr0001	slr1755	ssr2422
sll2010	slr2087	sll0519	slr0742	slr0783	ssr2831	slr0553	smr0001	sll0057	ssr2422
slr0528	slr2087	sll0098	sll0519	sll1198	ssr2831	slr0812	smr0001	sll0017	ssr2422
slr1351	slr2087	sll0519	slr1646	sll0204	ssr2831	slr1347	smr0001	sll1185	ssr2422
sll0622	slr2087	sll0519	slr1130	sll1750	ssr2831	slr1348	smr0001	slr0839	ssr2422
sll0631	slr2087	sll0519	slr0080	sll0420	ssr2831	slr1791	smr0001	sll1876	ssr2422
slr0787	slr2087	sll0519	slr0551	slr1256	ssr2831	sll0594	smr0001	sll0900	ssr2422
sll1852	slr2087	sll0145	sll0519	sll1639	ssr2831	slr0550	smr0001	slr0608	ssr2422
slr1090	slr2087	sll0519	sll0708	slr1899	ssr2831	sll1058	smr0001	slr0652	ssr2422

sll0902	slr2087	sll0519	slr0679	sll0643	ssr2831	slr1665	smr0001	slr0084	ssr2422
slr0661	slr2087	sll0519	slr0722	slr1844	ssr2831	slr0536	smr0001	sll1893	ssr2422
slr0526	slr2087	sll0519	slr0072	sll0459	ssr2831	slr1874	smr0001	slr0500	ssr2422
sll1249	slr2087	sll0519	sll1547	slr0733	ssr2831	sll1776	smr0001	slr1560	ssr2422
sll0892	slr2087	sll0519	sll1234	sll1459	ssr2831	sll0848	smr0001	sll1988	ssr2422
sll0660	slr2087	sll0519	sll0616	sll0905	ssr2831	sll0897	smr0001	slr1289	ssr2422
slr1779	slr2087	sll0519	slr2073	sll0402	smr0004	sll1933	smr0001	sll1556	ssr2422
slr0394	slr2087	sll0519	slr1639	sll0728	smr0004	slr0965	smr0001	ssl3441	ssr2422
slr2047	slr2087	sll0519	sll1108	sll0336	smr0004	slr1463	smr0001	slr0744	ssr2422
slr0741	slr2087	sll0362	sll0519	sll1299	smr0004	sll1098	smr0001	slr0974	ssr2422
slr1510	slr2087	sll0519	slr0958	smr0004	ssl2084	slr0434	smr0001	slr1622	ssr2422
sll1043	slr2087	sll0519	slr1720	sll2001	smr0004	sll1261	smr0001	sll1196	ssr2422
slr0400	slr2087	sll0179	sll0519	sll1430	smr0004	sll1099	smr0001	sll0745	ssr2422
sll1415	slr2087	sll0454	sll0519	sll0573	smr0004	slr0752	smr0001	sll1815	ssr2422
sll1909	slr2087	sll0519	sll1553	slr1898	smr0004	slr0952	smr0001	sll1059	ssr2422
sll0373	slr2087	sll0519	slr0638	sll0080	smr0004	slr1511	smr0001	slr1123	ssr2422
slr2035	slr2087	sll0519	slr0220	slr1022	smr0004	sll1605	smr0001	sll1760	ssr2422
slr2087	slr2132	sll0519	slr0357	sll1883	smr0004	sll0990	smr0001	sll0469	ssr2422
slr0922	slr2087	sll0519	sll1362	slr1133	smr0004	sll1633	smr0001	slr1517	ssr2422
slr0838	slr2087	sll0519	slr1550	slr0444	smr0004	slr0018	smr0001	sll0869	ssr2422
sll1823	slr2087	sll0519	sll1074	slr2130	smr0004	sll0567	smr0001	slr1598	ssr2422
sll1056	slr2087	sll0519	slr0649	sll1669	smr0004	slr0884	smr0001	sll0868	ssr2422
slr0520	slr2087	sll0495	sll0519	slr0585	smr0004	sll1342	smr0001	slr0994	ssr2422
sll0144	slr2087	sll0519	sll1425	slr2075	smr0004	slr1349	smr0001	sll0379	ssr2422
sll0467	slr2087	sll0502	sll0519	slr2076	smr0004	sll0593	smr0001	sll1676	ssr2422
slr0711	slr2087	sll0519	slr1703	sll0416	smr0004	sll0220	smr0001	sll0927	ssr2422
sll0754	slr2087	sll0078	sll0519	slr0156	smr0004	sll0899	smr0001	sll0996	ssr2422
sll0569	slr2087	sll0519	slr0557	slr1641	smr0004	slr1756	smr0001	sll0844	ssr2422
sll1277	slr2087	sll0519	slr1884	slr0542	smr0004	sll1085	smr0001	sll1615	ssr2422
sll1110	slr2087	sll0519	slr1031	sll0534	smr0004	sll1931	smr0001	sll0202	ssr2422
sll1865	slr2087	sll0519	slr1793	slr0165	smr0004	sll0083	smr0001	slr0902	ssr2422
slr1228	slr2087	sll0519	slr0633	sll0030	smr0004	slr1755	smr0001	sll1144	ssr2422
slr0808	slr2087	sll0519	slr1278	slr0847	smr0004	sll0057	smr0001	slr0017	ssr2422
slr0082	slr2087	sll0519	slr0783	slr0553	smr0004	sll0017	smr0001	slr1424	ssr2422
slr0742	slr2087	sll0519	sll1198	slr0812	smr0004	sll1185	smr0001	slr1423	ssr2422
sll0098	slr2087	sll0204	sll0519	slr1347	smr0004	slr0839	smr0001	sll2010	ssr2422
slr1646	slr2087	sll0519	sll1750	slr1348	smr0004	sll1876	smr0001	slr0528	ssr2422
slr1130	slr2087	sll0420	sll0519	slr1791	smr0004	sll0900	smr0001	slr1351	ssr2422
slr0080	slr2087	sll0519	slr1256	sll0594	smr0004	slr0608	smr0001	sll0622	ssr2422
slr0551	slr2087	sll0519	sll1639	slr0550	smr0004	slr0652	smr0001	sll0631	ssr2422
sll0145	slr2087	sll0519	slr1899	sll1058	smr0004	slr0084	smr0001	slr0787	ssr2422
sll0708	slr2087	sll0519	sll0643	slr1665	smr0004	sll1893	smr0001	sll1852	ssr2422
slr0679	slr2087	sll0519	slr1844	slr0536	smr0004	slr0500	smr0001	slr1090	ssr2422
slr0722	slr2087	sll0459	sll0519	slr1874	smr0004	slr1560	smr0001	sll0902	ssr2422
slr0072	slr2087	sll0519	sll0865	sll1776	smr0004	sll1988	smr0001	slr0661	ssr2422
sll1547	slr2087	sll0519	slr0733	sll0848	smr0004	slr1289	smr0001	slr0526	ssr2422
sll1234	slr2087	sll0519	sll1459	sll0897	smr0004	sll1556	smr0001	sll1249	ssr2422
sll0616	slr2087	sll0519	sll0905	sll1933	smr0004	smr0001	ssl3441	sll0892	ssr2422
slr2073	slr2087	sll0223	sll0402	slr0965	smr0004	slr0744	smr0001	sll0660	ssr2422
slr1639	slr2087	sll0223	sll0728	slr1463	smr0004	slr0974	smr0001	slr1779	ssr2422
sll1108	slr2087	sll0223	sll0336	sll1098	smr0004	slr1622	smr0001	slr0394	ssr2422

sll0362	slr2087	sll0223	sll1299	slr0434	smr0004	sll1196	smr0001	slr2047	ssr2422
slr0958	slr2087	sll0223	ssl2084	sll1261	smr0004	sll0745	smr0001	slr0741	ssr2422
slr1720	slr2087	sll0223	sll2001	sll1099	smr0004	sll1815	smr0001	slr1510	ssr2422
sll0179	slr2087	sll0223	sll1430	slr0752	smr0004	sll1059	smr0001	sll1043	ssr2422
sll0454	slr2087	sll0223	sll0573	slr0952	smr0004	slr1123	smr0001	slr0400	ssr2422
sll1553	slr2087	sll0223	slr1898	slr1511	smr0004	sll1760	smr0001	sll1415	ssr2422
slr0638	slr2087	sll0080	sll0223	sll1605	smr0004	sll0469	smr0001	sll1909	ssr2422
slr0220	slr2087	sll0223	slr1022	sll0990	smr0004	slr1517	smr0001	sll0373	ssr2422
slr0357	slr2087	sll0223	sll1883	sll1633	smr0004	sll0869	smr0001	slr2035	ssr2422
sll1362	slr2087	sll0223	slr1133	slr0018	smr0004	slr1598	smr0001	slr2132	ssr2422
slr1550	slr2087	sll0223	slr0444	sll0567	smr0004	sll0868	smr0001	slr0922	ssr2422
sll1074	slr2087	sll0223	slr2130	slr0884	smr0004	slr0994	smr0001	slr0838	ssr2422
slr0649	slr2087	sll0223	sll1669	sll1342	smr0004	sll0379	smr0001	sll1823	ssr2422
sll0495	slr2087	sll0223	slr0585	slr1349	smr0004	sll1676	smr0001	sll1056	ssr2422
sll1425	slr2087	sll0223	slr2075	sll0593	smr0004	sll0927	smr0001	slr0520	ssr2422
sll0502	slr2087	sll0223	slr2076	sll0220	smr0004	sll0996	smr0001	sll0144	ssr2422
slr1703	slr2087	sll0223	sll0416	sll0899	smr0004	sll0844	smr0001	sll0467	ssr2422
sll0078	slr2087	sll0223	slr0156	slr1756	smr0004	sll1615	smr0001	slr0711	ssr2422
slr0557	slr2087	sll0223	slr1641	sll1085	smr0004	sll0202	smr0001	sll0754	ssr2422
slr1884	slr2087	sll0223	slr0542	sll1931	smr0004	slr0902	smr0001	sll0569	ssr2422
slr1031	slr2087	sll0223	sll0534	sll0083	smr0004	sll1144	smr0001	sll1277	ssr2422
slr1793	slr2087	sll0223	slr0165	slr1755	smr0004	slr0017	smr0001	sll1110	ssr2422
slr0633	slr2087	sll0030	sll0223	sll0057	smr0004	slr1424	smr0001	sll1865	ssr2422
slr1278	slr2087	sll0223	slr0847	sll0017	smr0004	slr1423	smr0001	slr1228	ssr2422
slr0783	slr2087	sll0223	slr0553	sll1185	smr0004	sll2010	smr0001	slr0808	ssr2422
sll1198	slr2087	sll0223	slr0812	slr0839	smr0004	slr0528	smr0001	slr0082	ssr2422
sll0204	slr2087	sll0223	slr1347	sll1876	smr0004	slr1351	smr0001	slr0742	ssr2422
sll1750	slr2087	sll0223	slr1348	sll0900	smr0004	sll0622	smr0001	sll0098	ssr2422
sll0420	slr2087	sll0223	slr1791	slr0608	smr0004	sll0631	smr0001	slr1646	ssr2422
slr1256	slr2087	sll0223	sll0594	slr0652	smr0004	slr0787	smr0001	slr1130	ssr2422
sll1639	slr2087	sll0223	slr0550	slr0084	smr0004	sll1852	smr0001	slr0080	ssr2422
slr1899	slr2087	sll0223	sll1058	sll1893	smr0004	slr1090	smr0001	slr0551	ssr2422
sll0643	slr2087	sll0223	slr1665	slr0500	smr0004	sll0902	smr0001	sll0145	ssr2422
slr1844	slr2087	sll0223	slr0536	slr1560	smr0004	slr0661	smr0001	sll0708	ssr2422
sll0459	slr2087	sll0223	slr1874	sll1988	smr0004	slr0526	smr0001	slr0679	ssr2422
sll0865	slr2087	sll0223	sll1776	slr1289	smr0004	sll1249	smr0001	slr0722	ssr2422
slr0733	slr2087	sll0223	sll0848	sll1556	smr0004	sll0892	smr0001	slr0072	ssr2422
sll1459	slr2087	sll0223	sll0897	smr0004	ssl3441	sll0660	smr0001	sll1547	ssr2422
sll0905	slr2087	sll0223	sll1933	slr0744	smr0004	slr1779	smr0001	sll1234	ssr2422
sll0402	sll1513	sll0223	slr0965	slr0974	smr0004	slr0394	smr0001	sll0616	ssr2422
sll0728	sll1513	sll0223	slr1463	slr1622	smr0004	slr2047	smr0001	slr2073	ssr2422
sll0336	sll1513	sll0223	sll1098	sll1196	smr0004	slr0741	smr0001	slr1639	ssr2422
sll1299	sll1513	sll0223	slr0434	sll0745	smr0004	slr1510	smr0001	sll1108	ssr2422
sll1513	ssl2084	sll0223	sll1261	sll1815	smr0004	sll1043	smr0001	sll0362	ssr2422
sll1513	sll2001	sll0223	sll1099	sll1059	smr0004	slr0400	smr0001	slr0958	ssr2422
sll1430	sll1513	sll0223	slr0752	slr1123	smr0004	sll1415	smr0001	slr1720	ssr2422
sll0573	sll1513	sll0223	slr0952	sll1760	smr0004	sll1909	smr0001	sll0179	ssr2422
sll1513	slr1898	sll0223	slr1511	sll0469	smr0004	sll0373	smr0001	sll0454	ssr2422
sll0080	sll1513	sll0223	sll1605	slr1517	smr0004	slr2035	smr0001	sll1553	ssr2422
sll1513	slr1022	sll0223	sll0990	sll0869	smr0004	slr2132	smr0001	slr0638	ssr2422
sll1513	sll1883	sll0223	sll1633	slr1598	smr0004	slr0922	smr0001	slr0220	ssr2422

sll1513	slr1133	sll0223	slr0018	sll0868	smr0004	slr0838	smr0001	slr0357	ssr2422
sll1513	slr0444	sll0223	sll0567	slr0994	smr0004	sll1823	smr0001	sll1362	ssr2422
sll1513	slr2130	sll0223	slr0884	sll0379	smr0004	sll1056	smr0001	slr1550	ssr2422
sll1513	sll1669	sll0223	sll1342	sll1676	smr0004	slr0520	smr0001	sll1074	ssr2422
sll1513	slr0585	sll0223	slr1349	sll0927	smr0004	sll0144	smr0001	slr0649	ssr2422
sll1513	slr2075	sll0223	sll0593	sll0996	smr0004	sll0467	smr0001	sll0495	ssr2422
sll1513	slr2076	sll0220	sll0223	sll0844	smr0004	slr0711	smr0001	sll1425	ssr2422
sll0416	sll1513	sll0223	sll0899	sll1615	smr0004	sll0754	smr0001	sll0502	ssr2422
sll1513	slr0156	sll0223	slr1756	sll0202	smr0004	sll0569	smr0001	slr1703	ssr2422
sll1513	slr1641	sll0223	sll1085	slr0902	smr0004	sll1277	smr0001	sll0078	ssr2422
sll1513	slr0542	sll0223	sll1931	sll1144	smr0004	sll1110	smr0001	slr0557	ssr2422
sll0534	sll1513	sll0083	sll0223	slr0017	smr0004	sll1865	smr0001	slr1884	ssr2422
sll1513	slr0165	sll0223	slr1755	slr1424	smr0004	slr1228	smr0001	slr1031	ssr2422
sll0030	sll1513	sll0057	sll0223	slr1423	smr0004	slr0808	smr0001	slr1793	ssr2422
sll1513	slr0847	sll0017	sll0223	sll2010	smr0004	slr0082	smr0001	slr0633	ssr2422
sll1513	slr0553	sll0223	sll1185	slr0528	smr0004	slr0742	smr0001	slr1278	ssr2422
sll1513	slr0812	sll0223	sll1876	slr1351	smr0004	sll0098	smr0001	slr0783	ssr2422
sll1513	slr1347	sll0223	sll0900	sll0622	smr0004	slr1646	smr0001	sll1198	ssr2422
sll1513	slr1348	sll0223	slr0608	sll0631	smr0004	slr1130	smr0001	sll0204	ssr2422
sll1513	slr1791	sll0223	slr0652	slr0787	smr0004	slr0080	smr0001	sll1750	ssr2422
sll0594	sll1513	sll0223	slr0084	sll1852	smr0004	slr0551	smr0001	sll0420	ssr2422
sll1513	slr0550	sll0223	sll1893	slr1090	smr0004	sll0145	smr0001	slr1256	ssr2422
sll1058	sll1513	sll0223	slr0500	sll0902	smr0004	sll0708	smr0001	sll1639	ssr2422
sll1513	slr1665	sll0223	slr1560	slr0661	smr0004	slr0679	smr0001	slr1899	ssr2422
sll1513	slr0536	sll0223	sll1988	slr0526	smr0004	slr0722	smr0001	sll0643	ssr2422
sll1513	slr1874	sll0223	slr1289	sll1249	smr0004	slr0072	smr0001	slr1844	ssr2422
sll1513	sll1776	sll0223	sll1556	sll0892	smr0004	sll1547	smr0001	sll0459	ssr2422
sll0848	sll1513	sll0223	ssl3441	sll0660	smr0004	sll1234	smr0001	sll0865	ssr2422
sll0897	sll1513	sll0223	slr0744	slr1779	smr0004	sll0616	smr0001	slr0733	ssr2422
sll1513	sll1933	sll0223	slr0974	slr0394	smr0004	slr2073	smr0001	sll1459	ssr2422
sll1513	slr0965	sll0223	slr1622	slr2047	smr0004	slr1639	smr0001	sll0905	ssr2422
sll1513	slr1463	sll0223	sll1196	slr0741	smr0004	sll1108	smr0001	sll0402	ssl2501
sll1098	sll1513	sll0223	sll0745	slr1510	smr0004	sll0362	smr0001	sll0728	ssl2501
sll1513	slr0434	sll0223	sll1815	sll1043	smr0004	slr0958	smr0001	sll0336	ssl2501
sll1261	sll1513	sll0223	sll1059	slr0400	smr0004	slr1720	smr0001	sll1299	ssl2501
sll1099	sll1513	sll0223	slr1123	sll1415	smr0004	sll0179	smr0001	ssl2084	ssl2501
sll1513	slr0752	sll0223	sll1760	sll1909	smr0004	sll0454	smr0001	sll2001	ssl2501
sll1513	slr0952	sll0223	sll0469	sll0373	smr0004	sll1553	smr0001	sll1430	ssl2501
sll1513	slr1511	sll0223	slr1517	slr2035	smr0004	slr0638	smr0001	sll0573	ssl2501
sll1513	sll1605	sll0223	sll0869	slr2132	smr0004	slr0220	smr0001	slr1898	ssl2501
sll0990	sll1513	sll0223	slr1598	slr0922	smr0004	slr0357	smr0001	sll0080	ssl2501
sll1513	sll1633	sll0223	sll0868	slr0838	smr0004	sll1362	smr0001	slr1022	ssl2501
sll1513	slr0018	sll0223	slr0994	sll1823	smr0004	slr1550	smr0001	sll1883	ssl2501
sll0567	sll1513	sll0223	sll0379	sll1056	smr0004	sll1074	smr0001	slr1133	ssl2501
sll1513	slr0884	sll0223	sll1676	slr0520	smr0004	slr0649	smr0001	slr0444	ssl2501
sll1342	sll1513	sll0223	sll0927	sll0144	smr0004	sll0495	smr0001	slr2130	ssl2501
sll1513	slr1349	sll0223	sll0996	sll0467	smr0004	sll1425	smr0001	sll1669	ssl2501
sll0593	sll1513	sll0223	sll0844	slr0711	smr0004	sll0502	smr0001	slr0585	ssl2501
sll0220	sll1513	sll0223	sll1615	sll0754	smr0004	slr1703	smr0001	slr2075	ssl2501
sll0899	sll1513	sll0202	sll0223	sll0569	smr0004	sll0078	smr0001	slr2076	ssl2501
sll1513	slr1756	sll0223	slr0902	sll1277	smr0004	slr0557	smr0001	sll0416	ssl2501

sll1085	sll1513	sll0223	sll1144	sll1110	smr0004	slr1884	smr0001	slr0156	ssl2501
sll1513	sll1931	sll0223	slr0017	sll1865	smr0004	slr1031	smr0001	slr1641	ssl2501
sll0083	sll1513	sll0223	slr1424	slr1228	smr0004	slr1793	smr0001	slr0542	ssl2501
sll1513	slr1755	sll0223	slr1423	slr0808	smr0004	slr0633	smr0001	sll0534	ssl2501
sll0057	sll1513	sll0223	sll2010	slr0082	smr0004	slr1278	smr0001	slr0165	ssl2501
sll0017	sll1513	sll0223	slr0528	slr0742	smr0004	slr0783	smr0001	sll0030	ssl2501
sll1185	sll1513	sll0223	slr1351	sll0098	smr0004	sll1198	smr0001	slr0847	ssl2501
sll1513	slr0839	sll0223	sll0622	slr1646	smr0004	sll0204	smr0001	slr0553	ssl2501
sll1513	sll1876	sll0223	sll0631	slr1130	smr0004	sll1750	smr0001	slr0812	ssl2501
sll0900	sll1513	sll0223	slr0787	slr0080	smr0004	sll0420	smr0001	slr1347	ssl2501
sll1513	slr0608	sll0223	sll1852	slr0551	smr0004	slr1256	smr0001	slr1348	ssl2501
sll1513	slr0652	sll0223	slr1090	sll0145	smr0004	sll1639	smr0001	slr1791	ssl2501
sll1513	slr0084	sll0223	sll0902	sll0708	smr0004	slr1899	smr0001	sll0594	ssl2501
sll1513	sll1893	sll0223	slr0661	slr0679	smr0004	sll0643	smr0001	slr0550	ssl2501
sll1513	slr0500	sll0223	slr0526	slr0722	smr0004	slr1844	smr0001	sll1058	ssl2501
sll1513	slr1560	sll0223	sll1249	slr0072	smr0004	sll0459	smr0001	slr1665	ssl2501
sll1513	sll1988	sll0223	sll0892	sll1547	smr0004	sll0865	smr0001	slr0536	ssl2501
sll1513	slr1289	sll0223	sll0660	sll1234	smr0004	slr0733	smr0001	slr1874	ssl2501
sll1513	sll1556	sll0223	slr1779	sll0616	smr0004	sll1459	smr0001	sll1776	ssl2501
sll1513	ssl3441	sll0223	slr0394	slr2073	smr0004	sll0905	smr0001	sll0848	ssl2501
sll1513	slr0744	sll0223	slr2047	slr1639	smr0004	sll0402	sll1194	sll0897	ssl2501
sll1513	slr0974	sll0223	slr0741	sll1108	smr0004	sll0728	sll1194	sll1933	ssl2501
sll1513	slr1622	sll0223	slr1510	sll0362	smr0004	sll0336	sll1194	slr0965	ssl2501
sll1196	sll1513	sll0223	sll1043	slr0958	smr0004	sll1194	sll1299	slr1463	ssl2501
sll0745	sll1513	sll0223	slr0400	slr1720	smr0004	sll1194	ssl2084	sll1098	ssl2501
sll1513	sll1815	sll0223	sll1415	sll0179	smr0004	sll1194	sll2001	slr0434	ssl2501
sll1059	sll1513	sll0223	sll1909	sll0454	smr0004	sll1194	sll1430	sll1261	ssl2501
sll1513	slr1123	sll0223	sll0373	sll1553	smr0004	sll0573	sll1194	sll1099	ssl2501
sll1513	sll1760	sll0223	slr2035	slr0638	smr0004	sll1194	slr1898	slr0752	ssl2501
sll0469	sll1513	sll0223	slr2132	slr0220	smr0004	sll0080	sll1194	slr0952	ssl2501
sll1513	slr1517	sll0223	slr0922	slr0357	smr0004	sll1194	slr1022	slr1511	ssl2501
sll0869	sll1513	sll0223	slr0838	sll1362	smr0004	sll1194	sll1883	sll1605	ssl2501
sll1513	slr1598	sll0223	sll1823	slr1550	smr0004	sll1194	slr1133	sll0990	ssl2501
sll0868	sll1513	sll0223	sll1056	sll1074	smr0004	sll1194	slr0444	sll1633	ssl2501
sll1513	slr0994	sll0223	slr0520	slr0649	smr0004	sll1194	slr2130	slr0018	ssl2501
sll0379	sll1513	sll0144	sll0223	sll0495	smr0004	sll1194	sll1669	sll0567	ssl2501
sll1513	sll1676	sll0223	sll0467	sll1425	smr0004	sll1194	slr0585	slr0884	ssl2501
sll0927	sll1513	sll0223	slr0711	sll0502	smr0004	sll1194	slr2075	sll1342	ssl2501
sll0996	sll1513	sll0223	sll0754	slr1703	smr0004	sll1194	slr2076	slr1349	ssl2501
sll0844	sll1513	sll0223	sll0569	sll0078	smr0004	sll0416	sll1194	sll0593	ssl2501
sll1513	sll1615	sll0223	sll1277	slr0557	smr0004	sll1194	slr0156	sll0220	ssl2501
sll0202	sll1513	sll0223	sll1110	slr1884	smr0004	sll1194	slr1641	sll0899	ssl2501
sll1513	slr0902	sll0223	sll1865	slr1031	smr0004	sll1194	slr0542	slr1756	ssl2501
sll1144	sll1513	sll0223	slr1228	slr1793	smr0004	sll0534	sll1194	sll1085	ssl2501
sll1513	slr0017	sll0223	slr0808	slr0633	smr0004	sll1194	slr0165	sll1931	ssl2501
sll1513	slr1424	sll0223	slr0082	slr1278	smr0004	sll0030	sll1194	sll0083	ssl2501
sll1513	slr1423	sll0223	slr0742	slr0783	smr0004	sll1194	slr0847	slr1755	ssl2501
sll1513	sll2010	sll0098	sll0223	sll1198	smr0004	sll1194	slr0553	sll0057	ssl2501
sll1513	slr0528	sll0223	slr1646	sll0204	smr0004	sll1194	slr0812	sll0017	ssl2501
sll1513	slr1351	sll0223	slr1130	sll1750	smr0004	sll1194	slr1347	sll1185	ssl2501
sll0622	sll1513	sll0223	slr0080	sll0420	smr0004	sll1194	slr1348	slr0839	ssl2501

sll0631	sll1513	sll0223	slr0551	slr1256	smr0004	sll1194	slr1791	sll1876	ssl2501
sll1513	slr0787	sll0145	sll0223	sll1639	smr0004	sll0594	sll1194	sll0900	ssl2501
sll1513	sll1852	sll0223	sll0708	slr1899	smr0004	sll1194	slr0550	slr0608	ssl2501
sll1513	slr1090	sll0223	slr0679	sll0643	smr0004	sll1058	sll1194	slr0652	ssl2501
sll0902	sll1513	sll0223	slr0722	slr1844	smr0004	sll1194	slr1665	slr0084	ssl2501
sll1513	slr0661	sll0223	slr0072	sll0459	smr0004	sll1194	slr0536	sll1893	ssl2501
sll1513	slr0526	sll0223	sll1547	sll0865	smr0004	sll1194	slr1874	slr0500	ssl2501
sll1249	sll1513	sll0223	sll1234	slr0733	smr0004	sll1194	sll1776	slr1560	ssl2501
sll0892	sll1513	sll0223	sll0616	sll1459	smr0004	sll0848	sll1194	sll1988	ssl2501
sll0660	sll1513	sll0223	slr2073	sll0905	smr0004	sll0897	sll1194	slr1289	ssl2501
sll1513	slr1779	sll0223	slr1639	sll0402	sml0008	sll1194	sll1933	sll1556	ssl2501
sll1513	slr0394	sll0223	sll1108	sll0728	sml0008	sll1194	slr0965	ssl2501	ssl3441
sll1513	slr2047	sll0223	sll0362	sll0336	sml0008	sll1194	slr1463	slr0744	ssl2501
sll1513	slr0741	sll0223	slr0958	sll1299	sml0008	sll1098	sll1194	slr0974	ssl2501
sll1513	slr1510	sll0223	slr1720	sml0008	ssl2084	sll1194	slr0434	slr1622	ssl2501
sll1043	sll1513	sll0179	sll0223	sll2001	sml0008	sll1194	sll1261	sll1196	ssl2501
sll1513	slr0400	sll0223	sll0454	sll1430	sml0008	sll1099	sll1194	sll0745	ssl2501
sll1415	sll1513	sll0223	sll1553	sll0573	sml0008	sll1194	slr0752	sll1815	ssl2501
sll1513	sll1909	sll0223	slr0638	slr1898	sml0008	sll1194	slr0952	sll1059	ssl2501
sll0373	sll1513	sll0223	slr0220	sll0080	sml0008	sll1194	slr1511	slr1123	ssl2501
sll1513	slr2035	sll0223	slr0357	slr1022	sml0008	sll1194	sll1605	sll1760	ssl2501
sll1513	slr2132	sll0223	sll1362	sll1883	sml0008	sll0990	sll1194	sll0469	ssl2501
sll1513	slr0922	sll0223	slr1550	slr1133	sml0008	sll1194	sll1633	slr1517	ssl2501
sll1513	slr0838	sll0223	sll1074	slr0444	sml0008	sll1194	slr0018	sll0869	ssl2501
sll1513	sll1823	sll0223	slr0649	slr2130	sml0008	sll0567	sll1194	slr1598	ssl2501
sll1056	sll1513	sll0223	sll0495	sll1669	sml0008	sll1194	slr0884	sll0868	ssl2501
sll1513	slr0520	sll0223	sll1425	slr0585	sml0008	sll1194	sll1342	slr0994	ssl2501
sll0144	sll1513	sll0223	sll0502	slr2075	sml0008	sll1194	slr1349	sll0379	ssl2501
sll0467	sll1513	sll0223	slr1703	slr2076	sml0008	sll0593	sll1194	sll1676	ssl2501
sll1513	slr0711	sll0078	sll0223	sll0416	sml0008	sll0220	sll1194	sll0927	ssl2501
sll0754	sll1513	sll0223	slr0557	slr0156	sml0008	sll0899	sll1194	sll0996	ssl2501
sll0569	sll1513	sll0223	slr1884	slr1641	sml0008	sll1194	slr1756	sll0844	ssl2501
sll1277	sll1513	sll0223	slr1031	slr0542	sml0008	sll1085	sll1194	sll1615	ssl2501
sll1110	sll1513	sll0223	slr1793	sll0534	sml0008	sll1194	sll1931	sll0202	ssl2501
sll1513	sll1865	sll0223	slr0633	slr0165	sml0008	sll0083	sll1194	slr0902	ssl2501
sll1513	slr1228	sll0223	slr1278	sll0030	sml0008	sll1194	slr1755	sll1144	ssl2501
sll1513	slr0808	sll0223	slr0783	slr0847	sml0008	sll0057	sll1194	slr0017	ssl2501
sll1513	slr0082	sll0223	sll1198	slr0553	sml0008	sll0017	sll1194	slr1424	ssl2501
sll1513	slr0742	sll0204	sll0223	slr0812	sml0008	sll1185	sll1194	slr1423	ssl2501
sll0098	sll1513	sll0223	sll1750	slr1347	sml0008	sll1194	slr0839	sll2010	ssl2501
sll1513	slr1646	sll0223	sll0420	slr1348	sml0008	sll1194	sll1876	slr0528	ssl2501
sll1513	slr1130	sll0223	slr1256	slr1791	sml0008	sll0900	sll1194	slr1351	ssl2501
sll1513	slr0080	sll0223	sll1639	sll0594	sml0008	sll1194	slr0608	sll0622	ssl2501
sll1513	slr0551	sll0223	slr1899	slr0550	sml0008	sll1194	slr0652	sll0631	ssl2501
sll0145	sll1513	sll0223	sll0643	sll1058	sml0008	sll1194	slr0084	slr0787	ssl2501
sll0708	sll1513	sll0223	slr1844	slr1665	sml0008	sll1194	sll1893	sll1852	ssl2501
sll1513	slr0679	sll0223	sll0459	slr0536	sml0008	sll1194	slr0500	slr1090	ssl2501
sll1513	slr0722	sll0223	sll0865	slr1874	sml0008	sll1194	slr1560	sll0902	ssl2501
sll1513	slr0072	sll0223	slr0733	sll1776	sml0008	sll1194	sll1988	slr0661	ssl2501
sll1513	sll1547	sll0223	sll1459	sll0848	sml0008	sll1194	slr1289	slr0526	ssl2501
sll1234	sll1513	sll0223	sll0905	sll0897	sml0008	sll1194	sll1556	sll1249	ssl2501

sll0616	sll1513	sll0402	slr1279	sll1933	sml0008	sll1194	ssl3441	sll0892	ssl2501
sll1513	slr2073	sll0728	slr1279	slr0965	sml0008	sll1194	slr0744	sll0660	ssl2501
sll1513	slr1639	sll0336	slr1279	slr1463	sml0008	sll1194	slr0974	slr1779	ssl2501
sll1108	sll1513	sll1299	slr1279	sll1098	sml0008	sll1194	slr1622	slr0394	ssl2501
sll0362	sll1513	slr1279	ssl2084	slr0434	sml0008	sll1194	sll1196	slr2047	ssl2501
sll1513	slr0958	sll2001	slr1279	sll1261	sml0008	sll0745	sll1194	slr0741	ssl2501
sll1513	slr1720	sll1430	slr1279	sll1099	sml0008	sll1194	sll1815	slr1510	ssl2501
sll0179	sll1513	sll0573	slr1279	slr0752	sml0008	sll1059	sll1194	sll1043	ssl2501
sll0454	sll1513	slr1279	slr1898	slr0952	sml0008	sll1194	slr1123	slr0400	ssl2501
sll1513	sll1553	sll0080	slr1279	slr1511	sml0008	sll1194	sll1760	sll1415	ssl2501
sll1513	slr0638	slr1022	slr1279	sll1605	sml0008	sll0469	sll1194	sll1909	ssl2501
sll1513	slr0220	sll1883	slr1279	sll0990	sml0008	sll1194	slr1517	sll0373	ssl2501
sll1513	slr0357	slr1133	slr1279	sll1633	sml0008	sll0869	sll1194	slr2035	ssl2501
sll1362	sll1513	slr0444	slr1279	slr0018	sml0008	sll1194	slr1598	slr2132	ssl2501
sll1513	slr1550	slr1279	slr2130	sll0567	sml0008	sll0868	sll1194	slr0922	ssl2501
sll1074	sll1513	sll1669	slr1279	slr0884	sml0008	sll1194	slr0994	slr0838	ssl2501
sll1513	slr0649	slr0585	slr1279	sll1342	sml0008	sll0379	sll1194	sll1823	ssl2501
sll0495	sll1513	slr1279	slr2075	slr1349	sml0008	sll1194	sll1676	sll1056	ssl2501
sll1425	sll1513	slr1279	slr2076	sll0593	sml0008	sll0927	sll1194	slr0520	ssl2501
sll0502	sll1513	sll0416	slr1279	sll0220	sml0008	sll0996	sll1194	sll0144	ssl2501
sll1513	slr1703	slr0156	slr1279	sll0899	sml0008	sll0844	sll1194	sll0467	ssl2501
sll0078	sll1513	slr1279	slr1641	slr1756	sml0008	sll1194	sll1615	slr0711	ssl2501
sll1513	slr0557	slr0542	slr1279	sll1085	sml0008	sll0202	sll1194	sll0754	ssl2501
sll1513	slr1884	sll0534	slr1279	sll1931	sml0008	sll1194	slr0902	sll0569	ssl2501
sll1513	slr1031	slr0165	slr1279	sll0083	sml0008	sll1144	sll1194	sll1277	ssl2501
sll1513	slr1793	sll0030	slr1279	slr1755	sml0008	sll1194	slr0017	sll1110	ssl2501
sll1513	slr0633	slr0847	slr1279	sll0057	sml0008	sll1194	slr1424	sll1865	ssl2501
sll1513	slr1278	slr0553	slr1279	sll0017	sml0008	sll1194	slr1423	slr1228	ssl2501
sll1513	slr0783	slr0812	slr1279	sll1185	sml0008	sll1194	sll2010	slr0808	ssl2501
sll1198	sll1513	slr1279	slr1347	slr0839	sml0008	sll1194	slr0528	slr0082	ssl2501
sll0204	sll1513	slr1279	slr1348	sll1876	sml0008	sll1194	slr1351	slr0742	ssl2501
sll1513	sll1750	slr1279	slr1791	sll0900	sml0008	sll0622	sll1194	sll0098	ssl2501
sll0420	sll1513	sll0594	slr1279	slr0608	sml0008	sll0631	sll1194	slr1646	ssl2501
sll1513	slr1256	slr0550	slr1279	slr0652	sml0008	sll1194	slr0787	slr1130	ssl2501
sll1513	sll1639	sll1058	slr1279	slr0084	sml0008	sll1194	sll1852	slr0080	ssl2501
sll1513	slr1899	slr1279	slr1665	sll1893	sml0008	sll1194	slr1090	slr0551	ssl2501
sll0643	sll1513	slr0536	slr1279	slr0500	sml0008	sll0902	sll1194	sll0145	ssl2501
sll1513	slr1844	slr1279	slr1874	slr1560	sml0008	sll1194	slr0661	sll0708	ssl2501
sll0459	sll1513	sll1776	slr1279	sll1988	sml0008	sll1194	slr0526	slr0679	ssl2501
sll0865	sll1513	sll0848	slr1279	slr1289	sml0008	sll1194	sll1249	slr0722	ssl2501
sll1513	slr0733	sll0897	slr1279	sll1556	sml0008	sll0892	sll1194	slr0072	ssl2501
sll1459	sll1513	sll1933	slr1279	sml0008	ssl3441	sll0660	sll1194	sll1547	ssl2501
sll0905	sll1513	slr0965	slr1279	slr0744	sml0008	sll1194	slr1779	sll1234	ssl2501
sll0258	sll0402	slr1279	slr1463	slr0974	sml0008	sll1194	slr0394	sll0616	ssl2501
sll0258	sll0728	sll1098	slr1279	slr1622	sml0008	sll1194	slr2047	slr2073	ssl2501
sll0258	sll0336	slr0434	slr1279	sll1196	sml0008	sll1194	slr0741	slr1639	ssl2501
sll0258	sll1299	sll1261	slr1279	sll0745	sml0008	sll1194	slr1510	sll1108	ssl2501
sll0258	ssl2084	sll1099	slr1279	sll1815	sml0008	sll1043	sll1194	sll0362	ssl2501
sll0258	sll2001	slr0752	slr1279	sll1059	sml0008	sll1194	slr0400	slr0958	ssl2501
sll0258	sll1430	slr0952	slr1279	slr1123	sml0008	sll1194	sll1415	slr1720	ssl2501
sll0258	sll0573	slr1279	slr1511	sll1760	sml0008	sll1194	sll1909	sll0179	ssl2501

sll0258	slr1898	sll1605	slr1279	sll0469	sml0008	sll0373	sll1194	sll0454	ssl2501
sll0080	sll0258	sll0990	slr1279	slr1517	sml0008	sll1194	slr2035	sll1553	ssl2501
sll0258	slr1022	sll1633	slr1279	sll0869	sml0008	sll1194	slr2132	slr0638	ssl2501
sll0258	sll1883	slr0018	slr1279	slr1598	sml0008	sll1194	slr0922	slr0220	ssl2501
sll0258	slr1133	sll0567	slr1279	sll0868	sml0008	sll1194	slr0838	slr0357	ssl2501
sll0258	slr0444	slr0884	slr1279	slr0994	sml0008	sll1194	sll1823	sll1362	ssl2501
sll0258	slr2130	sll1342	slr1279	sll0379	sml0008	sll1056	sll1194	slr1550	ssl2501
sll0258	sll1669	slr1279	slr1349	sll1676	sml0008	sll1194	slr0520	sll1074	ssl2501
sll0258	slr0585	sll0593	slr1279	sll0927	sml0008	sll0144	sll1194	slr0649	ssl2501
sll0258	slr2075	sll0220	slr1279	sll0996	sml0008	sll0467	sll1194	sll0495	ssl2501
sll0258	slr2076	sll0899	slr1279	sll0844	sml0008	sll1194	slr0711	sll1425	ssl2501
sll0258	sll0416	slr1279	slr1756	sll1615	sml0008	sll0754	sll1194	sll0502	ssl2501
sll0258	slr0156	sll1085	slr1279	sll0202	sml0008	sll0569	sll1194	slr1703	ssl2501
sll0258	slr1641	sll1931	slr1279	slr0902	sml0008	sll1194	sll1277	sll0078	ssl2501
sll0258	slr0542	sll0083	slr1279	sll1144	sml0008	sll1110	sll1194	slr0557	ssl2501
sll0258	sll0534	slr1279	slr1755	slr0017	sml0008	sll1194	sll1865	slr1884	ssl2501
sll0258	slr0165	sll0057	slr1279	slr1424	sml0008	sll1194	slr1228	slr1031	ssl2501
sll0030	sll0258	sll0017	slr1279	slr1423	sml0008	sll1194	slr0808	slr1793	ssl2501
sll0258	slr0847	sll1185	slr1279	sll2010	sml0008	sll1194	slr0082	slr0633	ssl2501
sll0258	slr0553	slr0839	slr1279	slr0528	sml0008	sll1194	slr0742	slr1278	ssl2501
sll0258	slr0812	sll1876	slr1279	slr1351	sml0008	sll0098	sll1194	slr0783	ssl2501
sll0258	slr1347	sll0900	slr1279	sll0622	sml0008	sll1194	slr1646	sll1198	ssl2501
sll0258	slr1348	slr0608	slr1279	sll0631	sml0008	sll1194	slr1130	sll0204	ssl2501
sll0258	slr1791	slr0652	slr1279	slr0787	sml0008	sll1194	slr0080	sll1750	ssl2501
sll0258	sll0594	slr0084	slr1279	sll1852	sml0008	sll1194	slr0551	sll0420	ssl2501
sll0258	slr0550	sll1893	slr1279	slr1090	sml0008	sll0145	sll1194	slr1256	ssl2501
sll0258	sll1058	slr0500	slr1279	sll0902	sml0008	sll0708	sll1194	sll1639	ssl2501
sll0258	slr1665	slr1279	slr1560	slr0661	sml0008	sll1194	slr0679	slr1899	ssl2501
sll0258	slr0536	sll1988	slr1279	slr0526	sml0008	sll1194	slr0722	sll0643	ssl2501
sll0258	slr1874	slr1279	slr1289	sll1249	sml0008	sll1194	slr0072	slr1844	ssl2501
sll0258	sll1776	sll1556	slr1279	sll0892	sml0008	sll1194	sll1547	sll0459	ssl2501
sll0258	sll0848	slr1279	ssl3441	sll0660	sml0008	sll1194	sll1234	sll0865	ssl2501
sll0258	sll0897	slr0744	slr1279	slr1779	sml0008	sll0616	sll1194	slr0733	ssl2501
sll0258	sll1933	slr0974	slr1279	slr0394	sml0008	sll1194	slr2073	sll1459	ssl2501
sll0258	slr0965	slr1279	slr1622	slr2047	sml0008	sll1194	slr1639	sll0905	ssl2501
sll0258	slr1463	sll1196	slr1279	slr0741	sml0008	sll1108	sll1194	sll0402	sll0412
sll0258	sll1098	sll0745	slr1279	slr1510	sml0008	sll0362	sll1194	sll0412	sll0728
sll0258	slr0434	sll1815	slr1279	sll1043	sml0008	sll1194	slr0958	sll0336	sll0412
sll0258	sll1261	sll1059	slr1279	slr0400	sml0008	sll1194	slr1720	sll0412	sll1299
sll0258	sll1099	slr1123	slr1279	sll1415	sml0008	sll0179	sll1194	sll0412	ssl2084
sll0258	slr0752	sll1760	slr1279	sll1909	sml0008	sll0454	sll1194	sll0412	sll2001
sll0258	slr0952	sll0469	slr1279	sll0373	sml0008	sll1194	sll1553	sll0412	sll1430
sll0258	slr1511	slr1279	slr1517	slr2035	sml0008	sll1194	slr0638	sll0412	sll0573
sll0258	sll1605	sll0869	slr1279	slr2132	sml0008	sll1194	slr0220	sll0412	slr1898
sll0258	sll0990	slr1279	slr1598	slr0922	sml0008	sll1194	slr0357	sll0080	sll0412
sll0258	sll1633	sll0868	slr1279	slr0838	sml0008	sll1194	sll1362	sll0412	slr1022
sll0258	slr0018	slr0994	slr1279	sll1823	sml0008	sll1194	slr1550	sll0412	sll1883
sll0258	sll0567	sll0379	slr1279	sll1056	sml0008	sll1074	sll1194	sll0412	slr1133
sll0258	slr0884	sll1676	slr1279	slr0520	sml0008	sll1194	slr0649	sll0412	slr0444
sll0258	sll1342	sll0927	slr1279	sll0144	sml0008	sll0495	sll1194	sll0412	slr2130
sll0258	slr1349	sll0996	slr1279	sll0467	sml0008	sll1194	sll1425	sll0412	sll1669

sll0258	sll0593	sll0844	slr1279	slr0711	sml0008	sll0502	sll1194	sll0412	slr0585
sll0220	sll0258	sll1615	slr1279	sll0754	sml0008	sll1194	slr1703	sll0412	slr2075
sll0258	sll0899	sll0202	slr1279	sll0569	sml0008	sll0078	sll1194	sll0412	slr2076
sll0258	slr1756	slr0902	slr1279	sll1277	sml0008	sll1194	slr0557	sll0412	sll0416
sll0258	sll1085	sll1144	slr1279	sll1110	sml0008	sll1194	slr1884	sll0412	slr0156
sll0258	sll1931	slr0017	slr1279	sll1865	sml0008	sll1194	slr1031	sll0412	slr1641
sll0083	sll0258	slr1279	slr1424	slr1228	sml0008	sll1194	slr1793	sll0412	slr0542
sll0258	slr1755	slr1279	slr1423	slr0808	sml0008	sll1194	slr0633	sll0412	sll0534
sll0057	sll0258	sll2010	slr1279	slr0082	sml0008	sll1194	slr1278	sll0412	slr0165
sll0017	sll0258	slr0528	slr1279	slr0742	sml0008	sll1194	slr0783	sll0030	sll0412
sll0258	sll1185	slr1279	slr1351	sll0098	sml0008	sll1194	sll1198	sll0412	slr0847
sll0258	slr0839	sll0622	slr1279	slr1646	sml0008	sll0204	sll1194	sll0412	slr0553
sll0258	sll1876	sll0631	slr1279	slr1130	sml0008	sll1194	sll1750	sll0412	slr0812
sll0258	sll0900	slr0787	slr1279	slr0080	sml0008	sll0420	sll1194	sll0412	slr1347
sll0258	slr0608	sll1852	slr1279	slr0551	sml0008	sll1194	slr1256	sll0412	slr1348
sll0258	slr0652	slr1090	slr1279	sll0145	sml0008	sll1194	sll1639	sll0412	slr1791
sll0258	slr0084	sll0902	slr1279	sll0708	sml0008	sll1194	slr1899	sll0412	sll0594
sll0258	sll1893	slr0661	slr1279	slr0679	sml0008	sll0643	sll1194	sll0412	slr0550
sll0258	slr0500	slr0526	slr1279	slr0722	sml0008	sll1194	slr1844	sll0412	sll1058
sll0258	slr1560	sll1249	slr1279	slr0072	sml0008	sll0459	sll1194	sll0412	slr1665
sll0258	sll1988	sll0892	slr1279	sll1547	sml0008	sll0865	sll1194	sll0412	slr0536
sll0258	slr1289	sll0660	slr1279	sll1234	sml0008	sll1194	slr0733	sll0412	slr1874
sll0258	sll1556	slr1279	slr1779	sll0616	sml0008	sll1194	sll1459	sll0412	sll1776
sll0258	ssl3441	slr0394	slr1279	slr2073	sml0008	sll0905	sll1194	sll0412	sll0848
sll0258	slr0744	slr1279	slr2047	slr1639	sml0008	sll0402	sml0002	sll0412	sll0897
sll0258	slr0974	slr0741	slr1279	sll1108	sml0008	sll0728	sml0002	sll0412	sll1933
sll0258	slr1622	slr1279	slr1510	sll0362	sml0008	sll0336	sml0002	sll0412	slr0965
sll0258	sll1196	sll1043	slr1279	slr0958	sml0008	sll1299	sml0002	sll0412	slr1463
sll0258	sll0745	slr0400	slr1279	slr1720	sml0008	sml0002	ssl2084	sll0412	sll1098
sll0258	sll1815	sll1415	slr1279	sll0179	sml0008	sll2001	sml0002	sll0412	slr0434
sll0258	sll1059	sll1909	slr1279	sll0454	sml0008	sll1430	sml0002	sll0412	sll1261
sll0258	slr1123	sll0373	slr1279	sll1553	sml0008	sll0573	sml0002	sll0412	sll1099
sll0258	sll1760	slr1279	slr2035	slr0638	sml0008	slr1898	sml0002	sll0412	slr0752
sll0258	sll0469	slr1279	slr2132	slr0220	sml0008	sll0080	sml0002	sll0412	slr0952
sll0258	slr1517	slr0922	slr1279	slr0357	sml0008	slr1022	sml0002	sll0412	slr1511
sll0258	sll0869	slr0838	slr1279	sll1362	sml0008	sll1883	sml0002	sll0412	sll1605
sll0258	slr1598	sll1823	slr1279	slr1550	sml0008	slr1133	sml0002	sll0412	sll0990
sll0258	sll0868	sll1056	slr1279	sll1074	sml0008	slr0444	sml0002	sll0412	sll1633
sll0258	slr0994	slr0520	slr1279	slr0649	sml0008	slr2130	sml0002	sll0412	slr0018
sll0258	sll0379	sll0144	slr1279	sll0495	sml0008	sll1669	sml0002	sll0412	sll0567
sll0258	sll1676	sll0467	slr1279	sll1425	sml0008	slr0585	sml0002	sll0412	slr0884
sll0258	sll0927	slr0711	slr1279	sll0502	sml0008	slr2075	sml0002	sll0412	sll1342
sll0258	sll0996	sll0754	slr1279	slr1703	sml0008	slr2076	sml0002	sll0412	slr1349
sll0258	sll0844	sll0569	slr1279	sll0078	sml0008	sll0416	sml0002	sll0412	sll0593
sll0258	sll1615	sll1277	slr1279	slr0557	sml0008	slr0156	sml0002	sll0220	sll0412
sll0202	sll0258	sll1110	slr1279	slr1884	sml0008	slr1641	sml0002	sll0412	sll0899
sll0258	slr0902	sll1865	slr1279	slr1031	sml0008	slr0542	sml0002	sll0412	slr1756
sll0258	sll1144	slr1228	slr1279	slr1793	sml0008	sll0534	sml0002	sll0412	sll1085
sll0258	slr0017	slr0808	slr1279	slr0633	sml0008	slr0165	sml0002	sll0412	sll1931
sll0258	slr1424	slr0082	slr1279	slr1278	sml0008	sll0030	sml0002	sll0083	sll0412
sll0258	slr1423	slr0742	slr1279	slr0783	sml0008	slr0847	sml0002	sll0412	slr1755

sll0258	sll2010	sll0098	slr1279	sll1198	sml0008	slr0553	sml0002	sll0057	sll0412
sll0258	slr0528	slr1279	slr1646	sll0204	sml0008	slr0812	sml0002	sll0017	sll0412
sll0258	slr1351	slr1130	slr1279	sll1750	sml0008	slr1347	sml0002	sll0412	sll1185
sll0258	sll0622	slr0080	slr1279	sll0420	sml0008	slr1348	sml0002	sll0412	slr0839
sll0258	sll0631	slr0551	slr1279	slr1256	sml0008	slr1791	sml0002	sll0412	sll1876
sll0258	slr0787	sll0145	slr1279	sll1639	sml0008	sll0594	sml0002	sll0412	sll0900
sll0258	sll1852	sll0708	slr1279	slr1899	sml0008	slr0550	sml0002	sll0412	slr0608
sll0258	slr1090	slr0679	slr1279	sll0643	sml0008	sll1058	sml0002	sll0412	slr0652
sll0258	sll0902	slr0722	slr1279	slr1844	sml0008	slr1665	sml0002	sll0412	slr0084
sll0258	slr0661	slr0072	slr1279	sll0459	sml0008	slr0536	sml0002	sll0412	sll1893
sll0258	slr0526	sll1547	slr1279	sll0865	sml0008	slr1874	sml0002	sll0412	slr0500
sll0258	sll1249	sll1234	slr1279	slr0733	sml0008	sll1776	sml0002	sll0412	slr1560
sll0258	sll0892	sll0616	slr1279	sll1459	sml0008	sll0848	sml0002	sll0412	sll1988
sll0258	sll0660	slr1279	slr2073	sll0905	sml0008	sll0897	sml0002	sll0412	slr1289
sll0258	slr1779	slr1279	slr1639	sll0402	ssr0390	sll1933	sml0002	sll0412	sll1556
sll0258	slr0394	sll1108	slr1279	sll0728	ssr0390	slr0965	sml0002	sll0412	ssl3441
sll0258	slr2047	sll0362	slr1279	sll0336	ssr0390	slr1463	sml0002	sll0412	slr0744
sll0258	slr0741	slr0958	slr1279	sll1299	ssr0390	sll1098	sml0002	sll0412	slr0974
sll0258	slr1510	slr1279	slr1720	ssl2084	ssr0390	slr0434	sml0002	sll0412	slr1622
sll0258	sll1043	sll0179	slr1279	sll2001	ssr0390	sll1261	sml0002	sll0412	sll1196
sll0258	slr0400	sll0454	slr1279	sll1430	ssr0390	sll1099	sml0002	sll0412	sll0745
sll0258	sll1415	sll1553	slr1279	sll0573	ssr0390	slr0752	sml0002	sll0412	sll1815
sll0258	sll1909	slr0638	slr1279	slr1898	ssr0390	slr0952	sml0002	sll0412	sll1059
sll0258	sll0373	slr0220	slr1279	sll0080	ssr0390	slr1511	sml0002	sll0412	slr1123
sll0258	slr2035	slr0357	slr1279	slr1022	ssr0390	sll1605	sml0002	sll0412	sll1760
sll0258	slr2132	sll1362	slr1279	sll1883	ssr0390	sll0990	sml0002	sll0412	sll0469
sll0258	slr0922	slr1279	slr1550	slr1133	ssr0390	sll1633	sml0002	sll0412	slr1517
sll0258	slr0838	sll1074	slr1279	slr0444	ssr0390	slr0018	sml0002	sll0412	sll0869
sll0258	sll1823	slr0649	slr1279	slr2130	ssr0390	sll0567	sml0002	sll0412	slr1598
sll0258	sll1056	sll0495	slr1279	sll1669	ssr0390	slr0884	sml0002	sll0412	sll0868
sll0258	slr0520	sll1425	slr1279	slr0585	ssr0390	sll1342	sml0002	sll0412	slr0994
sll0144	sll0258	sll0502	slr1279	slr2075	ssr0390	slr1349	sml0002	sll0379	sll0412
sll0258	sll0467	slr1279	slr1703	slr2076	ssr0390	sll0593	sml0002	sll0412	sll1676
sll0258	slr0711	sll0078	slr1279	sll0416	ssr0390	sll0220	sml0002	sll0412	sll0927
sll0258	sll0754	slr0557	slr1279	slr0156	ssr0390	sll0899	sml0002	sll0412	sll0996
sll0258	sll0569	slr1279	slr1884	slr1641	ssr0390	slr1756	sml0002	sll0412	sll0844
sll0258	sll1277	slr1031	slr1279	slr0542	ssr0390	sll1085	sml0002	sll0412	sll1615
sll0258	sll1110	slr1279	slr1793	sll0534	ssr0390	sll1931	sml0002	sll0202	sll0412
sll0258	sll1865	slr0633	slr1279	slr0165	ssr0390	sll0083	sml0002	sll0412	slr0902
sll0258	slr1228	slr1278	slr1279	sll0030	ssr0390	slr1755	sml0002	sll0412	sll1144
sll0258	slr0808	slr0783	slr1279	slr0847	ssr0390	sll0057	sml0002	sll0412	slr0017
sll0258	slr0082	sll1198	slr1279	slr0553	ssr0390	sll0017	sml0002	sll0412	slr1424
sll0258	slr0742	sll0204	slr1279	slr0812	ssr0390	sll1185	sml0002	sll0412	slr1423
sll0098	sll0258	sll1750	slr1279	slr1347	ssr0390	slr0839	sml0002	sll0412	sll2010
sll0258	slr1646	sll0420	slr1279	slr1348	ssr0390	sll1876	sml0002	sll0412	slr0528
sll0258	slr1130	slr1256	slr1279	slr1791	ssr0390	sll0900	sml0002	sll0412	slr1351
sll0258	slr0080	sll1639	slr1279	sll0594	ssr0390	slr0608	sml0002	sll0412	sll0622
sll0258	slr0551	slr1279	slr1899	slr0550	ssr0390	slr0652	sml0002	sll0412	sll0631
sll0145	sll0258	sll0643	slr1279	sll1058	ssr0390	slr0084	sml0002	sll0412	slr0787
sll0258	sll0708	slr1279	slr1844	slr1665	ssr0390	sll1893	sml0002	sll0412	sll1852
sll0258	slr0679	sll0459	slr1279	slr0536	ssr0390	slr0500	sml0002	sll0412	slr1090

sll0258	slr0722	sll0865	slr1279	slr1874	ssr0390	slr1560	sml0002	sll0412	sll0902
sll0258	slr0072	slr0733	slr1279	sll1776	ssr0390	sll1988	sml0002	sll0412	slr0661
sll0258	sll1547	sll1459	slr1279	sll0848	ssr0390	slr1289	sml0002	sll0412	slr0526
sll0258	sll1234	sll0905	slr1279	sll0897	ssr0390	sll1556	sml0002	sll0412	sll1249
sll0258	sll0616	sll0402	slr0331	sll1933	ssr0390	sml0002	ssl3441	sll0412	sll0892
sll0258	slr2073	sll0728	slr0331	slr0965	ssr0390	slr0744	sml0002	sll0412	sll0660
sll0258	slr1639	sll0336	slr0331	slr1463	ssr0390	slr0974	sml0002	sll0412	slr1779
sll0258	sll1108	sll1299	slr0331	sll1098	ssr0390	slr1622	sml0002	sll0412	slr0394
sll0258	sll0362	slr0331	ssl2084	slr0434	ssr0390	sll1196	sml0002	sll0412	slr2047
sll0258	slr0958	sll2001	slr0331	sll1261	ssr0390	sll0745	sml0002	sll0412	slr0741
sll0258	slr1720	sll1430	slr0331	sll1099	ssr0390	sll1815	sml0002	sll0412	slr1510
sll0179	sll0258	sll0573	slr0331	slr0752	ssr0390	sll1059	sml0002	sll0412	sll1043
sll0258	sll0454	slr0331	slr1898	slr0952	ssr0390	slr1123	sml0002	sll0412	slr0400
sll0258	sll1553	sll0080	slr0331	slr1511	ssr0390	sll1760	sml0002	sll0412	sll1415
sll0258	slr0638	slr0331	slr1022	sll1605	ssr0390	sll0469	sml0002	sll0412	sll1909
sll0258	slr0220	sll1883	slr0331	sll0990	ssr0390	slr1517	sml0002	sll0373	sll0412
sll0258	slr0357	slr0331	slr1133	sll1633	ssr0390	sll0869	sml0002	sll0412	slr2035
sll0258	sll1362	slr0331	slr0444	slr0018	ssr0390	slr1598	sml0002	sll0412	slr2132
sll0258	slr1550	slr0331	slr2130	sll0567	ssr0390	sll0868	sml0002	sll0412	slr0922
sll0258	sll1074	sll1669	slr0331	slr0884	ssr0390	slr0994	sml0002	sll0412	slr0838
sll0258	slr0649	slr0331	slr0585	sll1342	ssr0390	sll0379	sml0002	sll0412	sll1823
sll0258	sll0495	slr0331	slr2075	slr1349	ssr0390	sll1676	sml0002	sll0412	sll1056
sll0258	sll1425	slr0331	slr2076	sll0593	ssr0390	sll0927	sml0002	sll0412	slr0520
sll0258	sll0502	sll0416	slr0331	sll0220	ssr0390	sll0996	sml0002	sll0144	sll0412
sll0258	slr1703	slr0156	slr0331	sll0899	ssr0390	sll0844	sml0002	sll0412	sll0467
sll0078	sll0258	slr0331	slr1641	slr1756	ssr0390	sll1615	sml0002	sll0412	slr0711
sll0258	slr0557	slr0331	slr0542	sll1085	ssr0390	sll0202	sml0002	sll0412	sll0754
sll0258	slr1884	sll0534	slr0331	sll1931	ssr0390	slr0902	sml0002	sll0412	sll0569
sll0258	slr1031	slr0165	slr0331	sll0083	ssr0390	sll1144	sml0002	sll0412	sll1277
sll0258	slr1793	sll0030	slr0331	slr1755	ssr0390	slr0017	sml0002	sll0412	sll1110
sll0258	slr0633	slr0331	slr0847	sll0057	ssr0390	slr1424	sml0002	sll0412	sll1865
sll0258	slr1278	slr0331	slr0553	sll0017	ssr0390	slr1423	sml0002	sll0412	slr1228
sll0258	slr0783	slr0331	slr0812	sll1185	ssr0390	sll2010	sml0002	sll0412	slr0808
sll0258	sll1198	slr0331	slr1347	slr0839	ssr0390	slr0528	sml0002	sll0412	slr0082
sll0204	sll0258	slr0331	slr1348	sll1876	ssr0390	slr1351	sml0002	sll0412	slr0742
sll0258	sll1750	slr0331	slr1791	sll0900	ssr0390	sll0622	sml0002	sll0098	sll0412
sll0258	sll0420	sll0594	slr0331	slr0608	ssr0390	sll0631	sml0002	sll0412	slr1646
sll0258	slr1256	slr0331	slr0550	slr0652	ssr0390	slr0787	sml0002	sll0412	slr1130
sll0258	sll1639	sll1058	slr0331	slr0084	ssr0390	sll1852	sml0002	sll0412	slr0080
sll0258	slr1899	slr0331	slr1665	sll1893	ssr0390	slr1090	sml0002	sll0412	slr0551
sll0258	sll0643	slr0331	slr0536	slr0500	ssr0390	sll0902	sml0002	sll0145	sll0412
sll0258	slr1844	slr0331	slr1874	slr1560	ssr0390	slr0661	sml0002	sll0412	sll0708
sll0258	sll0459	sll1776	slr0331	sll1988	ssr0390	slr0526	sml0002	sll0412	slr0679
sll0258	sll0865	sll0848	slr0331	slr1289	ssr0390	sll1249	sml0002	sll0412	slr0722
sll0258	slr0733	sll0897	slr0331	sll1556	ssr0390	sll0892	sml0002	sll0412	slr0072
sll0258	sll1459	sll1933	slr0331	ssl3441	ssr0390	sll0660	sml0002	sll0412	sll1547
sll0258	sll0905	slr0331	slr0965	slr0744	ssr0390	slr1779	sml0002	sll0412	sll1234
sll0402	slr0342	slr0331	slr1463	slr0974	ssr0390	slr0394	sml0002	sll0412	sll0616
sll0728	slr0342	sll1098	slr0331	slr1622	ssr0390	slr2047	sml0002	sll0412	slr2073
sll0336	slr0342	slr0331	slr0434	sll1196	ssr0390	slr0741	sml0002	sll0412	slr1639
sll1299	slr0342	sll1261	slr0331	sll0745	ssr0390	slr1510	sml0002	sll0412	sll1108

slr0342	ssl2084	sll1099	slr0331	sll1815	ssr0390	sll1043	sml0002	sll0362	sll0412
sll2001	slr0342	slr0331	slr0752	sll1059	ssr0390	slr0400	sml0002	sll0412	slr0958
sll1430	slr0342	slr0331	slr0952	slr1123	ssr0390	sll1415	sml0002	sll0412	slr1720
sll0573	slr0342	slr0331	slr1511	sll1760	ssr0390	sll1909	sml0002	sll0179	sll0412
slr0342	slr1898	sll1605	slr0331	sll0469	ssr0390	sll0373	sml0002	sll0412	sll0454
sll0080	slr0342	sll0990	slr0331	slr1517	ssr0390	slr2035	sml0002	sll0412	sll1553
slr0342	slr1022	sll1633	slr0331	sll0869	ssr0390	slr2132	sml0002	sll0412	slr0638
sll1883	slr0342	slr0018	slr0331	slr1598	ssr0390	slr0922	sml0002	sll0412	slr0220
slr0342	slr1133	sll0567	slr0331	sll0868	ssr0390	slr0838	sml0002	sll0412	slr0357
slr0342	slr0444	slr0331	slr0884	slr0994	ssr0390	sll1823	sml0002	sll0412	sll1362
slr0342	slr2130	sll1342	slr0331	sll0379	ssr0390	sll1056	sml0002	sll0412	slr1550
sll1669	slr0342	slr0331	slr1349	sll1676	ssr0390	slr0520	sml0002	sll0412	sll1074
slr0342	slr0585	sll0593	slr0331	sll0927	ssr0390	sll0144	sml0002	sll0412	slr0649
slr0342	slr2075	sll0220	slr0331	sll0996	ssr0390	sll0467	sml0002	sll0412	sll0495
slr0342	slr2076	sll0899	slr0331	sll0844	ssr0390	slr0711	sml0002	sll0412	sll1425
sll0416	slr0342	slr0331	slr1756	sll1615	ssr0390	sll0754	sml0002	sll0412	sll0502
slr0156	slr0342	sll1085	slr0331	sll0202	ssr0390	sll0569	sml0002	sll0412	slr1703
slr0342	slr1641	sll1931	slr0331	slr0902	ssr0390	sll1277	sml0002	sll0078	sll0412
slr0342	slr0542	sll0083	slr0331	sll1144	ssr0390	sll1110	sml0002	sll0412	slr0557
sll0534	slr0342	slr0331	slr1755	slr0017	ssr0390	sll1865	sml0002	sll0412	slr1884
slr0165	slr0342	sll0057	slr0331	slr1424	ssr0390	slr1228	sml0002	sll0412	slr1031
sll0030	slr0342	sll0017	slr0331	slr1423	ssr0390	slr0808	sml0002	sll0412	slr1793
slr0342	slr0847	sll1185	slr0331	sll2010	ssr0390	slr0082	sml0002	sll0412	slr0633
slr0342	slr0553	slr0331	slr0839	slr0528	ssr0390	slr0742	sml0002	sll0412	slr1278
slr0342	slr0812	sll1876	slr0331	slr1351	ssr0390	sll0098	sml0002	sll0412	slr0783
slr0342	slr1347	sll0900	slr0331	sll0622	ssr0390	slr1646	sml0002	sll0412	sll1198
slr0342	slr1348	slr0331	slr0608	sll0631	ssr0390	slr1130	sml0002	sll0204	sll0412
slr0342	slr1791	slr0331	slr0652	slr0787	ssr0390	slr0080	sml0002	sll0412	sll1750
sll0594	slr0342	slr0084	slr0331	sll1852	ssr0390	slr0551	sml0002	sll0412	sll0420
slr0342	slr0550	sll1893	slr0331	slr1090	ssr0390	sll0145	sml0002	sll0412	slr1256
sll1058	slr0342	slr0331	slr0500	sll0902	ssr0390	sll0708	sml0002	sll0412	sll1639
slr0342	slr1665	slr0331	slr1560	slr0661	ssr0390	slr0679	sml0002	sll0412	slr1899
slr0342	slr0536	sll1988	slr0331	slr0526	ssr0390	slr0722	sml0002	sll0412	sll0643
slr0342	slr1874	slr0331	slr1289	sll1249	ssr0390	slr0072	sml0002	sll0412	slr1844
sll1776	slr0342	sll1556	slr0331	sll0892	ssr0390	sll1547	sml0002	sll0412	sll0459
sll0848	slr0342	slr0331	ssl3441	sll0660	ssr0390	sll1234	sml0002	sll0412	sll0865
sll0897	slr0342	slr0331	slr0744	slr1779	ssr0390	sll0616	sml0002	sll0412	slr0733
sll1933	slr0342	slr0331	slr0974	slr0394	ssr0390	slr2073	sml0002	sll0412	sll1459
slr0342	slr0965	slr0331	slr1622	slr2047	ssr0390	slr1639	sml0002	sll0412	sll0905
slr0342	slr1463	sll1196	slr0331	slr0741	ssr0390	sll1108	sml0002	sll0402	slr0575
sll1098	slr0342	sll0745	slr0331	slr1510	ssr0390	sll0362	sml0002	sll0728	slr0575
slr0342	slr0434	sll1815	slr0331	sll1043	ssr0390	slr0958	sml0002	sll0336	slr0575
sll1261	slr0342	sll1059	slr0331	slr0400	ssr0390	slr1720	sml0002	sll1299	slr0575
sll1099	slr0342	slr0331	slr1123	sll1415	ssr0390	sll0179	sml0002	slr0575	ssl2084
slr0342	slr0752	sll1760	slr0331	sll1909	ssr0390	sll0454	sml0002	sll2001	slr0575
slr0342	slr0952	sll0469	slr0331	sll0373	ssr0390	sll1553	sml0002	sll1430	slr0575
slr0342	slr1511	slr0331	slr1517	slr2035	ssr0390	slr0638	sml0002	sll0573	slr0575
sll1605	slr0342	sll0869	slr0331	slr2132	ssr0390	slr0220	sml0002	slr0575	slr1898
sll0990	slr0342	slr0331	slr1598	slr0922	ssr0390	slr0357	sml0002	sll0080	slr0575
sll1633	slr0342	sll0868	slr0331	slr0838	ssr0390	sll1362	sml0002	slr0575	slr1022
slr0018	slr0342	slr0331	slr0994	sll1823	ssr0390	slr1550	sml0002	sll1883	slr0575

sll0567	slr0342	sll0379	slr0331	sll1056	ssr0390	sll1074	sml0002	slr0575	slr1133
slr0342	slr0884	sll1676	slr0331	slr0520	ssr0390	slr0649	sml0002	slr0444	slr0575
sll1342	slr0342	sll0927	slr0331	sll0144	ssr0390	sll0495	sml0002	slr0575	slr2130
slr0342	slr1349	sll0996	slr0331	sll0467	ssr0390	sll1425	sml0002	sll1669	slr0575
sll0593	slr0342	sll0844	slr0331	slr0711	ssr0390	sll0502	sml0002	slr0575	slr0585
sll0220	slr0342	sll1615	slr0331	sll0754	ssr0390	slr1703	sml0002	slr0575	slr2075
sll0899	slr0342	sll0202	slr0331	sll0569	ssr0390	sll0078	sml0002	slr0575	slr2076
slr0342	slr1756	slr0331	slr0902	sll1277	ssr0390	slr0557	sml0002	sll0416	slr0575
sll1085	slr0342	sll1144	slr0331	sll1110	ssr0390	slr1884	sml0002	slr0156	slr0575
sll1931	slr0342	slr0017	slr0331	sll1865	ssr0390	slr1031	sml0002	slr0575	slr1641
sll0083	slr0342	slr0331	slr1424	slr1228	ssr0390	slr1793	sml0002	slr0542	slr0575
slr0342	slr1755	slr0331	slr1423	slr0808	ssr0390	slr0633	sml0002	sll0534	slr0575
sll0057	slr0342	sll2010	slr0331	slr0082	ssr0390	slr1278	sml0002	slr0165	slr0575
sll0017	slr0342	slr0331	slr0528	slr0742	ssr0390	slr0783	sml0002	sll0030	slr0575
sll1185	slr0342	slr0331	slr1351	sll0098	ssr0390	sll1198	sml0002	slr0575	slr0847
slr0342	slr0839	sll0622	slr0331	slr1646	ssr0390	sll0204	sml0002	slr0553	slr0575
sll1876	slr0342	sll0631	slr0331	slr1130	ssr0390	sll1750	sml0002	slr0575	slr0812
sll0900	slr0342	slr0331	slr0787	slr0080	ssr0390	sll0420	sml0002	slr0575	slr1347
slr0342	slr0608	sll1852	slr0331	slr0551	ssr0390	slr1256	sml0002	slr0575	slr1348
slr0342	slr0652	slr0331	slr1090	sll0145	ssr0390	sll1639	sml0002	slr0575	slr1791
slr0084	slr0342	sll0902	slr0331	sll0708	ssr0390	slr1899	sml0002	sll0594	slr0575
sll1893	slr0342	slr0331	slr0661	slr0679	ssr0390	sll0643	sml0002	slr0550	slr0575
slr0342	slr0500	slr0331	slr0526	slr0722	ssr0390	slr1844	sml0002	sll1058	slr0575
slr0342	slr1560	sll1249	slr0331	slr0072	ssr0390	sll0459	sml0002	slr0575	slr1665
sll1988	slr0342	sll0892	slr0331	sll1547	ssr0390	sll0865	sml0002	slr0536	slr0575
slr0342	slr1289	sll0660	slr0331	sll1234	ssr0390	slr0733	sml0002	slr0575	slr1874
sll1556	slr0342	slr0331	slr1779	sll0616	ssr0390	sll1459	sml0002	sll1776	slr0575
slr0342	ssl3441	slr0331	slr0394	slr2073	ssr0390	sll0905	sml0002	sll0848	slr0575
slr0342	slr0744	slr0331	slr2047	slr1639	ssr0390	sll0402	sml0007	sll0897	slr0575
slr0342	slr0974	slr0331	slr0741	sll1108	ssr0390	sll0728	sml0007	sll1933	slr0575
slr0342	slr1622	slr0331	slr1510	sll0362	ssr0390	sll0336	sml0007	slr0575	slr0965
sll1196	slr0342	sll1043	slr0331	slr0958	ssr0390	sll1299	sml0007	slr0575	slr1463
sll0745	slr0342	slr0331	slr0400	slr1720	ssr0390	sml0007	ssl2084	sll1098	slr0575
sll1815	slr0342	sll1415	slr0331	sll0179	ssr0390	sll2001	sml0007	slr0434	slr0575
sll1059	slr0342	sll1909	slr0331	sll0454	ssr0390	sll1430	sml0007	sll1261	slr0575
slr0342	slr1123	sll0373	slr0331	sll1553	ssr0390	sll0573	sml0007	sll1099	slr0575
sll1760	slr0342	slr0331	slr2035	slr0638	ssr0390	slr1898	sml0007	slr0575	slr0752
sll0469	slr0342	slr0331	slr2132	slr0220	ssr0390	sll0080	sml0007	slr0575	slr0952
slr0342	slr1517	slr0331	slr0922	slr0357	ssr0390	slr1022	sml0007	slr0575	slr1511
sll0869	slr0342	slr0331	slr0838	sll1362	ssr0390	sll1883	sml0007	sll1605	slr0575
slr0342	slr1598	sll1823	slr0331	slr1550	ssr0390	slr1133	sml0007	sll0990	slr0575
sll0868	slr0342	sll1056	slr0331	sll1074	ssr0390	slr0444	sml0007	sll1633	slr0575
slr0342	slr0994	slr0331	slr0520	slr0649	ssr0390	slr2130	sml0007	slr0018	slr0575
sll0379	slr0342	sll0144	slr0331	sll0495	ssr0390	sll1669	sml0007	sll0567	slr0575
sll1676	slr0342	sll0467	slr0331	sll1425	ssr0390	slr0585	sml0007	slr0575	slr0884
sll0927	slr0342	slr0331	slr0711	sll0502	ssr0390	slr2075	sml0007	sll1342	slr0575
sll0996	slr0342	sll0754	slr0331	slr1703	ssr0390	slr2076	sml0007	slr0575	slr1349
sll0844	slr0342	sll0569	slr0331	sll0078	ssr0390	sll0416	sml0007	sll0593	slr0575
sll1615	slr0342	sll1277	slr0331	slr0557	ssr0390	slr0156	sml0007	sll0220	slr0575
sll0202	slr0342	sll1110	slr0331	slr1884	ssr0390	slr1641	sml0007	sll0899	slr0575
slr0342	slr0902	sll1865	slr0331	slr1031	ssr0390	slr0542	sml0007	slr0575	slr1756

sll1144	slr0342	slr0331	slr1228	slr1793	ssr0390	sll0534	sml0007	sll1085	slr0575
slr0017	slr0342	slr0331	slr0808	slr0633	ssr0390	slr0165	sml0007	sll1931	slr0575
slr0342	slr1424	slr0082	slr0331	slr1278	ssr0390	sll0030	sml0007	sll0083	slr0575
slr0342	slr1423	slr0331	slr0742	slr0783	ssr0390	slr0847	sml0007	slr0575	slr1755
sll2010	slr0342	sll0098	slr0331	sll1198	ssr0390	slr0553	sml0007	sll0057	slr0575
slr0342	slr0528	slr0331	slr1646	sll0204	ssr0390	slr0812	sml0007	sll0017	slr0575
slr0342	slr1351	slr0331	slr1130	sll1750	ssr0390	slr1347	sml0007	sll1185	slr0575
sll0622	slr0342	slr0080	slr0331	sll0420	ssr0390	slr1348	sml0007	slr0575	slr0839
sll0631	slr0342	slr0331	slr0551	slr1256	ssr0390	slr1791	sml0007	sll1876	slr0575
slr0342	slr0787	sll0145	slr0331	sll1639	ssr0390	sll0594	sml0007	sll0900	slr0575
sll1852	slr0342	sll0708	slr0331	slr1899	ssr0390	slr0550	sml0007	slr0575	slr0608
slr0342	slr1090	slr0331	slr0679	sll0643	ssr0390	sll1058	sml0007	slr0575	slr0652
sll0902	slr0342	slr0331	slr0722	slr1844	ssr0390	slr1665	sml0007	slr0084	slr0575
slr0342	slr0661	slr0072	slr0331	sll0459	ssr0390	slr0536	sml0007	sll1893	slr0575
slr0342	slr0526	sll1547	slr0331	sll0865	ssr0390	slr1874	sml0007	slr0500	slr0575
sll1249	slr0342	sll1234	slr0331	slr0733	ssr0390	sll1776	sml0007	slr0575	slr1560
sll0892	slr0342	sll0616	slr0331	sll1459	ssr0390	sll0848	sml0007	sll1988	slr0575
sll0660	slr0342	slr0331	slr2073	sll0905	ssr0390	sll0897	sml0007	slr0575	slr1289
slr0342	slr1779	slr0331	slr1639	sll0402	sll0629	sll1933	sml0007	sll1556	slr0575
slr0342	slr0394	sll1108	slr0331	sll0629	sll0728	slr0965	sml0007	slr0575	ssl3441
slr0342	slr2047	sll0362	slr0331	sll0336	sll0629	slr1463	sml0007	slr0575	slr0744
slr0342	slr0741	slr0331	slr0958	sll0629	sll1299	sll1098	sml0007	slr0575	slr0974
slr0342	slr1510	slr0331	slr1720	sll0629	ssl2084	slr0434	sml0007	slr0575	slr1622
sll1043	slr0342	sll0179	slr0331	sll0629	sll2001	sll1261	sml0007	sll1196	slr0575
slr0342	slr0400	sll0454	slr0331	sll0629	sll1430	sll1099	sml0007	sll0745	slr0575
sll1415	slr0342	sll1553	slr0331	sll0573	sll0629	slr0752	sml0007	sll1815	slr0575
sll1909	slr0342	slr0331	slr0638	sll0629	slr1898	slr0952	sml0007	sll1059	slr0575
sll0373	slr0342	slr0220	slr0331	sll0080	sll0629	slr1511	sml0007	slr0575	slr1123
slr0342	slr2035	slr0331	slr0357	sll0629	slr1022	sll1605	sml0007	sll1760	slr0575
slr0342	slr2132	sll1362	slr0331	sll0629	sll1883	sll0990	sml0007	sll0469	slr0575
slr0342	slr0922	slr0331	slr1550	sll0629	slr1133	sll1633	sml0007	slr0575	slr1517
slr0342	slr0838	sll1074	slr0331	sll0629	slr0444	slr0018	sml0007	sll0869	slr0575
sll1823	slr0342	slr0331	slr0649	sll0629	slr2130	sll0567	sml0007	slr0575	slr1598
sll1056	slr0342	sll0495	slr0331	sll0629	sll1669	slr0884	sml0007	sll0868	slr0575
slr0342	slr0520	sll1425	slr0331	sll0629	slr0585	sll1342	sml0007	slr0575	slr0994
sll0144	slr0342	sll0502	slr0331	sll0629	slr2075	slr1349	sml0007	sll0379	slr0575
sll0467	slr0342	slr0331	slr1703	sll0629	slr2076	sll0593	sml0007	sll1676	slr0575
slr0342	slr0711	sll0078	slr0331	sll0416	sll0629	sll0220	sml0007	sll0927	slr0575
sll0754	slr0342	slr0331	slr0557	sll0629	slr0156	sll0899	sml0007	sll0996	slr0575
sll0569	slr0342	slr0331	slr1884	sll0629	slr1641	slr1756	sml0007	sll0844	slr0575
sll1277	slr0342	slr0331	slr1031	sll0629	slr0542	sll1085	sml0007	sll1615	slr0575
sll1110	slr0342	slr0331	slr1793	sll0534	sll0629	sll1931	sml0007	sll0202	slr0575
sll1865	slr0342	slr0331	slr0633	sll0629	slr0165	sll0083	sml0007	slr0575	slr0902
slr0342	slr1228	slr0331	slr1278	sll0030	sll0629	slr1755	sml0007	sll1144	slr0575
slr0342	slr0808	slr0331	slr0783	sll0629	slr0847	sll0057	sml0007	slr0017	slr0575
slr0082	slr0342	sll1198	slr0331	sll0629	slr0553	sll0017	sml0007	slr0575	slr1424
slr0342	slr0742	sll0204	slr0331	sll0629	slr0812	sll1185	sml0007	slr0575	slr1423
sll0098	slr0342	sll1750	slr0331	sll0629	slr1347	slr0839	sml0007	sll2010	slr0575
slr0342	slr1646	sll0420	slr0331	sll0629	slr1348	sll1876	sml0007	slr0528	slr0575
slr0342	slr1130	slr0331	slr1256	sll0629	slr1791	sll0900	sml0007	slr0575	slr1351
slr0080	slr0342	sll1639	slr0331	sll0594	sll0629	slr0608	sml0007	sll0622	slr0575

slr0342	slr0551	slr0331	slr1899	sll0629	slr0550	slr0652	sml0007	sll0631	slr0575
sll0145	slr0342	sll0643	slr0331	sll0629	sll1058	slr0084	sml0007	slr0575	slr0787
sll0708	slr0342	slr0331	slr1844	sll0629	slr1665	sll1893	sml0007	sll1852	slr0575
slr0342	slr0679	sll0459	slr0331	sll0629	slr0536	slr0500	sml0007	slr0575	slr1090
slr0342	slr0722	sll0865	slr0331	sll0629	slr1874	slr1560	sml0007	sll0902	slr0575
slr0072	slr0342	slr0331	slr0733	sll0629	sll1776	sll1988	sml0007	slr0575	slr0661
sll1547	slr0342	sll1459	slr0331	sll0629	sll0848	slr1289	sml0007	slr0526	slr0575
sll1234	slr0342	sll0905	slr0331	sll0629	sll0897	sll1556	sml0007	sll1249	slr0575
sll0616	slr0342	sll0402	slr1291	sll0629	sll1933	sml0007	ssl3441	sll0892	slr0575
slr0342	slr2073	sll0728	slr1291	sll0629	slr0965	slr0744	sml0007	sll0660	slr0575
slr0342	slr1639	sll0336	slr1291	sll0629	slr1463	slr0974	sml0007	slr0575	slr1779
sll1108	slr0342	sll1299	slr1291	sll0629	sll1098	slr1622	sml0007	slr0394	slr0575
sll0362	slr0342	slr1291	ssl2084	sll0629	slr0434	sll1196	sml0007	slr0575	slr2047
slr0342	slr0958	sll2001	slr1291	sll0629	sll1261	sll0745	sml0007	slr0575	slr0741
slr0342	slr1720	sll1430	slr1291	sll0629	sll1099	sll1815	sml0007	slr0575	slr1510
sll0179	slr0342	sll0573	slr1291	sll0629	slr0752	sll1059	sml0007	sll1043	slr0575
sll0454	slr0342	slr1291	slr1898	sll0629	slr0952	slr1123	sml0007	slr0400	slr0575
sll1553	slr0342	sll0080	slr1291	sll0629	slr1511	sll1760	sml0007	sll1415	slr0575
slr0342	slr0638	slr1022	slr1291	sll0629	sll1605	sll0469	sml0007	sll1909	slr0575
slr0220	slr0342	sll1883	slr1291	sll0629	sll0990	slr1517	sml0007	sll0373	slr0575
slr0342	slr0357	slr1133	slr1291	sll0629	sll1633	sll0869	sml0007	slr0575	slr2035
sll1362	slr0342	slr0444	slr1291	sll0629	slr0018	slr1598	sml0007	slr0575	slr2132
slr0342	slr1550	slr1291	slr2130	sll0567	sll0629	sll0868	sml0007	slr0575	slr0922
sll1074	slr0342	sll1669	slr1291	sll0629	slr0884	slr0994	sml0007	slr0575	slr0838
slr0342	slr0649	slr0585	slr1291	sll0629	sll1342	sll0379	sml0007	sll1823	slr0575
sll0495	slr0342	slr1291	slr2075	sll0629	slr1349	sll1676	sml0007	sll1056	slr0575
sll1425	slr0342	slr1291	slr2076	sll0593	sll0629	sll0927	sml0007	slr0520	slr0575
sll0502	slr0342	sll0416	slr1291	sll0220	sll0629	sll0996	sml0007	sll0144	slr0575
slr0342	slr1703	slr0156	slr1291	sll0629	sll0899	sll0844	sml0007	sll0467	slr0575
sll0078	slr0342	slr1291	slr1641	sll0629	slr1756	sll1615	sml0007	slr0575	slr0711
slr0342	slr0557	slr0542	slr1291	sll0629	sll1085	sll0202	sml0007	sll0754	slr0575
slr0342	slr1884	sll0534	slr1291	sll0629	sll1931	slr0902	sml0007	sll0569	slr0575
slr0342	slr1031	slr0165	slr1291	sll0083	sll0629	sll1144	sml0007	sll1277	slr0575
slr0342	slr1793	sll0030	slr1291	sll0629	slr1755	slr0017	sml0007	sll1110	slr0575
slr0342	slr0633	slr0847	slr1291	sll0057	sll0629	slr1424	sml0007	sll1865	slr0575
slr0342	slr1278	slr0553	slr1291	sll0017	sll0629	slr1423	sml0007	slr0575	slr1228
slr0342	slr0783	slr0812	slr1291	sll0629	sll1185	sll2010	sml0007	slr0575	slr0808
sll1198	slr0342	slr1291	slr1347	sll0629	slr0839	slr0528	sml0007	slr0082	slr0575
sll0204	slr0342	slr1291	slr1348	sll0629	sll1876	slr1351	sml0007	slr0575	slr0742
sll1750	slr0342	slr1291	slr1791	sll0629	sll0900	sll0622	sml0007	sll0098	slr0575
sll0420	slr0342	sll0594	slr1291	sll0629	slr0608	sll0631	sml0007	slr0575	slr1646
slr0342	slr1256	slr0550	slr1291	sll0629	slr0652	slr0787	sml0007	slr0575	slr1130
sll1639	slr0342	sll1058	slr1291	sll0629	slr0084	sll1852	sml0007	slr0080	slr0575
slr0342	slr1899	slr1291	slr1665	sll0629	sll1893	slr1090	sml0007	slr0551	slr0575
sll0643	slr0342	slr0536	slr1291	sll0629	slr0500	sll0902	sml0007	sll0145	slr0575
slr0342	slr1844	slr1291	slr1874	sll0629	slr1560	slr0661	sml0007	sll0708	slr0575
sll0459	slr0342	sll1776	slr1291	sll0629	sll1988	slr0526	sml0007	slr0575	slr0679
sll0865	slr0342	sll0848	slr1291	sll0629	slr1289	sll1249	sml0007	slr0575	slr0722
slr0342	slr0733	sll0897	slr1291	sll0629	sll1556	sll0892	sml0007	slr0072	slr0575
sll1459	slr0342	sll1933	slr1291	sll0629	ssl3441	sll0660	sml0007	sll1547	slr0575
sll0905	slr0342	slr0965	slr1291	sll0629	slr0744	slr1779	sml0007	sll1234	slr0575

sll0402	sll1317	slr1291	slr1463	sll0629	slr0974	slr0394	sml0007	sll0616	slr0575
sll0728	sll1317	sll1098	slr1291	sll0629	slr1622	slr2047	sml0007	slr0575	slr2073
sll0336	sll1317	slr0434	slr1291	sll0629	sll1196	slr0741	sml0007	slr0575	slr1639
sll1299	sll1317	sll1261	slr1291	sll0629	sll0745	slr1510	sml0007	sll1108	slr0575
sll1317	ssl2084	sll1099	slr1291	sll0629	sll1815	sll1043	sml0007	sll0362	slr0575
sll1317	sll2001	slr0752	slr1291	sll0629	sll1059	slr0400	sml0007	slr0575	slr0958
sll1317	sll1430	slr0952	slr1291	sll0629	slr1123	sll1415	sml0007	slr0575	slr1720
sll0573	sll1317	slr1291	slr1511	sll0629	sll1760	sll1909	sml0007	sll0179	slr0575
sll1317	slr1898	sll1605	slr1291	sll0469	sll0629	sll0373	sml0007	sll0454	slr0575
sll0080	sll1317	sll0990	slr1291	sll0629	slr1517	slr2035	sml0007	sll1553	slr0575
sll1317	slr1022	sll1633	slr1291	sll0629	sll0869	slr2132	sml0007	slr0575	slr0638
sll1317	sll1883	slr0018	slr1291	sll0629	slr1598	slr0922	sml0007	slr0220	slr0575
sll1317	slr1133	sll0567	slr1291	sll0629	sll0868	slr0838	sml0007	slr0357	slr0575
sll1317	slr0444	slr0884	slr1291	sll0629	slr0994	sll1823	sml0007	sll1362	slr0575
sll1317	slr2130	sll1342	slr1291	sll0379	sll0629	sll1056	sml0007	slr0575	slr1550
sll1317	sll1669	slr1291	slr1349	sll0629	sll1676	slr0520	sml0007	sll1074	slr0575
sll1317	slr0585	sll0593	slr1291	sll0629	sll0927	sll0144	sml0007	slr0575	slr0649
sll1317	slr2075	sll0220	slr1291	sll0629	sll0996	sll0467	sml0007	sll0495	slr0575
sll1317	slr2076	sll0899	slr1291	sll0629	sll0844	slr0711	sml0007	sll1425	slr0575
sll0416	sll1317	slr1291	slr1756	sll0629	sll1615	sll0754	sml0007	sll0502	slr0575
sll1317	slr0156	sll1085	slr1291	sll0202	sll0629	sll0569	sml0007	slr0575	slr1703
sll1317	slr1641	sll1931	slr1291	sll0629	slr0902	sll1277	sml0007	sll0078	slr0575
sll1317	slr0542	sll0083	slr1291	sll0629	sll1144	sll1110	sml0007	slr0557	slr0575
sll0534	sll1317	slr1291	slr1755	sll0629	slr0017	sll1865	sml0007	slr0575	slr1884
sll1317	slr0165	sll0057	slr1291	sll0629	slr1424	slr1228	sml0007	slr0575	slr1031
sll0030	sll1317	sll0017	slr1291	sll0629	slr1423	slr0808	sml0007	slr0575	slr1793
sll1317	slr0847	sll1185	slr1291	sll0629	sll2010	slr0082	sml0007	slr0575	slr0633
sll1317	slr0553	slr0839	slr1291	sll0629	slr0528	slr0742	sml0007	slr0575	slr1278
sll1317	slr0812	sll1876	slr1291	sll0629	slr1351	sll0098	sml0007	slr0575	slr0783
sll1317	slr1347	sll0900	slr1291	sll0622	sll0629	slr1646	sml0007	sll1198	slr0575
sll1317	slr1348	slr0608	slr1291	sll0629	sll0631	slr1130	sml0007	sll0204	slr0575
sll1317	slr1791	slr0652	slr1291	sll0629	slr0787	slr0080	sml0007	sll1750	slr0575
sll0594	sll1317	slr0084	slr1291	sll0629	sll1852	slr0551	sml0007	sll0420	slr0575
sll1317	slr0550	sll1893	slr1291	sll0629	slr1090	sll0145	sml0007	slr0575	slr1256
sll1058	sll1317	slr0500	slr1291	sll0629	sll0902	sll0708	sml0007	sll1639	slr0575
sll1317	slr1665	slr1291	slr1560	sll0629	slr0661	slr0679	sml0007	slr0575	slr1899
sll1317	slr0536	sll1988	slr1291	sll0629	slr0526	slr0722	sml0007	sll0643	slr0575
sll1317	slr1874	slr1289	slr1291	sll0629	sll1249	slr0072	sml0007	slr0575	slr1844
sll1317	sll1776	sll1556	slr1291	sll0629	sll0892	sll1547	sml0007	sll0459	slr0575
sll0848	sll1317	slr1291	ssl3441	sll0629	sll0660	sll1234	sml0007	sll0865	slr0575
sll0897	sll1317	slr0744	slr1291	sll0629	slr1779	sll0616	sml0007	slr0575	slr0733
sll1317	sll1933	slr0974	slr1291	sll0629	slr0394	slr2073	sml0007	sll1459	slr0575
sll1317	slr0965	slr1291	slr1622	sll0629	slr2047	slr1639	sml0007	sll0905	slr0575
sll1317	slr1463	sll1196	slr1291	sll0629	slr0741	sll1108	sml0007	sll0402	slr0729
sll1098	sll1317	sll0745	slr1291	sll0629	slr1510	sll0362	sml0007	sll0728	slr0729
sll1317	slr0434	sll1815	slr1291	sll0629	sll1043	slr0958	sml0007	sll0336	slr0729
sll1261	sll1317	sll1059	slr1291	sll0629	slr0400	slr1720	sml0007	sll1299	slr0729
sll1099	sll1317	slr1123	slr1291	sll0629	sll1415	sll0179	sml0007	slr0729	ssl2084
sll1317	slr0752	sll1760	slr1291	sll0629	sll1909	sll0454	sml0007	sll2001	slr0729
sll1317	slr0952	sll0469	slr1291	sll0373	sll0629	sll1553	sml0007	sll1430	slr0729
sll1317	slr1511	slr1291	slr1517	sll0629	slr2035	slr0638	sml0007	sll0573	slr0729

sll1317	sll1605	sll0869	slr1291	sll0629	slr2132	slr0220	sml0007	slr0729	slr1898
sll0990	sll1317	slr1291	slr1598	sll0629	slr0922	slr0357	sml0007	sll0080	slr0729
sll1317	sll1633	sll0868	slr1291	sll0629	slr0838	sll1362	sml0007	slr0729	slr1022
sll1317	slr0018	slr0994	slr1291	sll0629	sll1823	slr1550	sml0007	sll1883	slr0729
sll0567	sll1317	sll0379	slr1291	sll0629	sll1056	sll1074	sml0007	slr0729	slr1133
sll1317	slr0884	sll1676	slr1291	sll0629	slr0520	slr0649	sml0007	slr0444	slr0729
sll1317	sll1342	sll0927	slr1291	sll0144	sll0629	sll0495	sml0007	slr0729	slr2130
sll1317	slr1349	sll0996	slr1291	sll0467	sll0629	sll1425	sml0007	sll1669	slr0729
sll0593	sll1317	sll0844	slr1291	sll0629	slr0711	sll0502	sml0007	slr0585	slr0729
sll0220	sll1317	sll1615	slr1291	sll0629	sll0754	slr1703	sml0007	slr0729	slr2075
sll0899	sll1317	sll0202	slr1291	sll0569	sll0629	sll0078	sml0007	slr0729	slr2076
sll1317	slr1756	slr0902	slr1291	sll0629	sll1277	slr0557	sml0007	sll0416	slr0729
sll1085	sll1317	sll1144	slr1291	sll0629	sll1110	slr1884	sml0007	slr0156	slr0729
sll1317	sll1931	slr0017	slr1291	sll0629	sll1865	slr1031	sml0007	slr0729	slr1641
sll0083	sll1317	slr1291	slr1424	sll0629	slr1228	slr1793	sml0007	slr0542	slr0729
sll1317	slr1755	slr1291	slr1423	sll0629	slr0808	slr0633	sml0007	sll0534	slr0729
sll0057	sll1317	sll2010	slr1291	sll0629	slr0082	slr1278	sml0007	slr0165	slr0729
sll0017	sll1317	slr0528	slr1291	sll0629	slr0742	slr0783	sml0007	sll0030	slr0729
sll1185	sll1317	slr1291	slr1351	sll0098	sll0629	sll1198	sml0007	slr0729	slr0847
sll1317	slr0839	sll0622	slr1291	sll0629	slr1646	sll0204	sml0007	slr0553	slr0729
sll1317	sll1876	sll0631	slr1291	sll0629	slr1130	sll1750	sml0007	slr0729	slr0812
sll0900	sll1317	slr0787	slr1291	sll0629	slr0080	sll0420	sml0007	slr0729	slr1347
sll1317	slr0608	sll1852	slr1291	sll0629	slr0551	slr1256	sml0007	slr0729	slr1348
sll1317	slr0652	slr1090	slr1291	sll0145	sll0629	sll1639	sml0007	slr0729	slr1791
sll1317	slr0084	sll0902	slr1291	sll0629	sll0708	slr1899	sml0007	sll0594	slr0729
sll1317	sll1893	slr0661	slr1291	sll0629	slr0679	sll0643	sml0007	slr0550	slr0729
sll1317	slr0500	slr0526	slr1291	sll0629	slr0722	slr1844	sml0007	sll1058	slr0729
sll1317	slr1560	sll1249	slr1291	sll0629	slr0072	sll0459	sml0007	slr0729	slr1665
sll1317	sll1988	sll0892	slr1291	sll0629	sll1547	sll0865	sml0007	slr0536	slr0729
sll1317	slr1289	sll0660	slr1291	sll0629	sll1234	slr0733	sml0007	slr0729	slr1874
sll1317	sll1556	slr1291	slr1779	sll0616	sll0629	sll1459	sml0007	sll1776	slr0729
sll1317	ssl3441	slr0394	slr1291	sll0629	slr2073	sll0905	sml0007	sll0848	slr0729
sll1317	slr0744	slr1291	slr2047	sll0629	slr1639	sll0402	sll1281	sll0897	slr0729
sll1317	slr0974	slr0741	slr1291	sll0629	sll1108	sll0728	sll1281	sll1933	slr0729
sll1317	slr1622	slr1291	slr1510	sll0362	sll0629	sll0336	sll1281	slr0729	slr0965
sll1196	sll1317	sll1043	slr1291	sll0629	slr0958	sll1281	sll1299	slr0729	slr1463
sll0745	sll1317	slr0400	slr1291	sll0629	slr1720	sll1281	ssl2084	sll1098	slr0729
sll1317	sll1815	sll1415	slr1291	sll0179	sll0629	sll1281	sll2001	slr0434	slr0729
sll1059	sll1317	sll1909	slr1291	sll0454	sll0629	sll1281	sll1430	sll1261	slr0729
sll1317	slr1123	sll0373	slr1291	sll0629	sll1553	sll0573	sll1281	sll1099	slr0729
sll1317	sll1760	slr1291	slr2035	sll0629	slr0638	sll1281	slr1898	slr0729	slr0752
sll0469	sll1317	slr1291	slr2132	sll0629	slr0220	sll0080	sll1281	slr0729	slr0952
sll1317	slr1517	slr0922	slr1291	sll0629	slr0357	sll1281	slr1022	slr0729	slr1511
sll0869	sll1317	slr0838	slr1291	sll0629	sll1362	sll1281	sll1883	sll1605	slr0729
sll1317	slr1598	sll1823	slr1291	sll0629	slr1550	sll1281	slr1133	sll0990	slr0729
sll0868	sll1317	sll1056	slr1291	sll0629	sll1074	sll1281	slr0444	sll1633	slr0729
sll1317	slr0994	slr0520	slr1291	sll0629	slr0649	sll1281	slr2130	slr0018	slr0729
sll0379	sll1317	sll0144	slr1291	sll0495	sll0629	sll1281	sll1669	sll0567	slr0729
sll1317	sll1676	sll0467	slr1291	sll0629	sll1425	sll1281	slr0585	slr0729	slr0884
sll0927	sll1317	slr0711	slr1291	sll0502	sll0629	sll1281	slr2075	sll1342	slr0729
sll0996	sll1317	sll0754	slr1291	sll0629	slr1703	sll1281	slr2076	slr0729	slr1349

sll0844	sll1317	sll0569	slr1291	sll0078	sll0629	sll0416	sll1281	sll0593	slr0729
sll1317	sll1615	sll1277	slr1291	sll0629	slr0557	sll1281	slr0156	sll0220	slr0729
sll0202	sll1317	sll1110	slr1291	sll0629	slr1884	sll1281	slr1641	sll0899	slr0729
sll1317	slr0902	sll1865	slr1291	sll0629	slr1031	sll1281	slr0542	slr0729	slr1756
sll1144	sll1317	slr1228	slr1291	sll0629	slr1793	sll0534	sll1281	sll1085	slr0729
sll1317	slr0017	slr0808	slr1291	sll0629	slr0633	sll1281	slr0165	sll1931	slr0729
sll1317	slr1424	slr0082	slr1291	sll0629	slr1278	sll0030	sll1281	sll0083	slr0729
sll1317	slr1423	slr0742	slr1291	sll0629	slr0783	sll1281	slr0847	slr0729	slr1755
sll1317	sll2010	sll0098	slr1291	sll0629	sll1198	sll1281	slr0553	sll0057	slr0729
sll1317	slr0528	slr1291	slr1646	sll0204	sll0629	sll1281	slr0812	sll0017	slr0729
sll1317	slr1351	slr1130	slr1291	sll0629	sll1750	sll1281	slr1347	sll1185	slr0729
sll0622	sll1317	slr0080	slr1291	sll0420	sll0629	sll1281	slr1348	slr0729	slr0839
sll0631	sll1317	slr0551	slr1291	sll0629	slr1256	sll1281	slr1791	sll1876	slr0729
sll1317	slr0787	sll0145	slr1291	sll0629	sll1639	sll0594	sll1281	sll0900	slr0729
sll1317	sll1852	sll0708	slr1291	sll0629	slr1899	sll1281	slr0550	slr0608	slr0729
sll1317	slr1090	slr0679	slr1291	sll0629	sll0643	sll1058	sll1281	slr0652	slr0729
sll0902	sll1317	slr0722	slr1291	sll0629	slr1844	sll1281	slr1665	slr0084	slr0729
sll1317	slr0661	slr0072	slr1291	sll0459	sll0629	sll1281	slr0536	sll1893	slr0729
sll1317	slr0526	sll1547	slr1291	sll0629	sll0865	sll1281	slr1874	slr0500	slr0729
sll1249	sll1317	sll1234	slr1291	sll0629	slr0733	sll1281	sll1776	slr0729	slr1560
sll0892	sll1317	sll0616	slr1291	sll0629	sll1459	sll0848	sll1281	sll1988	slr0729
sll0660	sll1317	slr1291	slr2073	sll0629	sll0905	sll0897	sll1281	slr0729	slr1289
sll1317	slr1779	slr1291	slr1639	sll0402	slr1655	sll1281	sll1933	sll1556	slr0729
sll1317	slr0394	sll1108	slr1291	sll0728	slr1655	sll1281	slr0965	slr0729	ssl3441
sll1317	slr2047	sll0362	slr1291	sll0336	slr1655	sll1281	slr1463	slr0729	slr0744
sll1317	slr0741	slr0958	slr1291	sll1299	slr1655	sll1098	sll1281	slr0729	slr0974
sll1317	slr1510	slr1291	slr1720	slr1655	ssl2084	sll1281	slr0434	slr0729	slr1622
sll1043	sll1317	sll0179	slr1291	sll2001	slr1655	sll1261	sll1281	sll1196	slr0729
sll1317	slr0400	sll0454	slr1291	sll1430	slr1655	sll1099	sll1281	sll0745	slr0729
sll1317	sll1415	sll1553	slr1291	sll0573	slr1655	sll1281	slr0752	sll1815	slr0729
sll1317	sll1909	slr0638	slr1291	slr1655	slr1898	sll1281	slr0952	sll1059	slr0729
sll0373	sll1317	slr0220	slr1291	sll0080	slr1655	sll1281	slr1511	slr0729	slr1123
sll1317	slr2035	slr0357	slr1291	slr1022	slr1655	sll1281	sll1605	sll1760	slr0729
sll1317	slr2132	sll1362	slr1291	sll1883	slr1655	sll0990	sll1281	sll0469	slr0729
sll1317	slr0922	slr1291	slr1550	slr1133	slr1655	sll1281	sll1633	slr0729	slr1517
sll1317	slr0838	sll1074	slr1291	slr0444	slr1655	sll1281	slr0018	sll0869	slr0729
sll1317	sll1823	slr0649	slr1291	slr1655	slr2130	sll0567	sll1281	slr0729	slr1598
sll1056	sll1317	sll0495	slr1291	sll1669	slr1655	sll1281	slr0884	sll0868	slr0729
sll1317	slr0520	sll1425	slr1291	slr0585	slr1655	sll1281	sll1342	slr0729	slr0994
sll0144	sll1317	sll0502	slr1291	slr1655	slr2075	sll1281	slr1349	sll0379	slr0729
sll0467	sll1317	slr1291	slr1703	slr1655	slr2076	sll0593	sll1281	sll1676	slr0729
sll1317	slr0711	sll0078	slr1291	sll0416	slr1655	sll0220	sll1281	sll0927	slr0729
sll0754	sll1317	slr0557	slr1291	slr0156	slr1655	sll0899	sll1281	sll0996	slr0729
sll0569	sll1317	slr1291	slr1884	slr1641	slr1655	sll1281	slr1756	sll0844	slr0729
sll1277	sll1317	slr1031	slr1291	slr0542	slr1655	sll1085	sll1281	sll1615	slr0729
sll1110	sll1317	slr1291	slr1793	sll0534	slr1655	sll1281	sll1931	sll0202	slr0729
sll1317	sll1865	slr0633	slr1291	slr0165	slr1655	sll0083	sll1281	slr0729	slr0902
sll1317	slr1228	slr1278	slr1291	sll0030	slr1655	sll1281	slr1755	sll1144	slr0729
sll1317	slr0808	slr0783	slr1291	slr0847	slr1655	sll0057	sll1281	slr0017	slr0729
sll1317	slr0082	sll1198	slr1291	slr0553	slr1655	sll0017	sll1281	slr0729	slr1424
sll1317	slr0742	sll0204	slr1291	slr0812	slr1655	sll1185	sll1281	slr0729	slr1423

sll0098	sll1317	sll1750	slr1291	slr1347	slr1655	sll1281	slr0839	sll2010	slr0729
sll1317	slr1646	sll0420	slr1291	slr1348	slr1655	sll1281	sll1876	slr0528	slr0729
sll1317	slr1130	slr1256	slr1291	slr1655	slr1791	sll0900	sll1281	slr0729	slr1351
sll1317	slr0080	sll1639	slr1291	sll0594	slr1655	sll1281	slr0608	sll0622	slr0729
sll1317	slr0551	slr1291	slr1899	slr0550	slr1655	sll1281	slr0652	sll0631	slr0729
sll0145	sll1317	sll0643	slr1291	sll1058	slr1655	sll1281	slr0084	slr0729	slr0787
sll0708	sll1317	slr1291	slr1844	slr1655	slr1665	sll1281	sll1893	sll1852	slr0729
sll1317	slr0679	sll0459	slr1291	slr0536	slr1655	sll1281	slr0500	slr0729	slr1090
sll1317	slr0722	sll0865	slr1291	slr1655	slr1874	sll1281	slr1560	sll0902	slr0729
sll1317	slr0072	slr0733	slr1291	sll1776	slr1655	sll1281	sll1988	slr0661	slr0729
sll1317	sll1547	sll1459	slr1291	sll0848	slr1655	sll1281	slr1289	slr0526	slr0729
sll1234	sll1317	sll0905	slr1291	sll0897	slr1655	sll1281	sll1556	sll1249	slr0729
sll0616	sll1317	sll0402	sll0522	sll1933	slr1655	sll1281	ssl3441	sll0892	slr0729
sll1317	slr2073	sll0522	sll0728	slr0965	slr1655	sll1281	slr0744	sll0660	slr0729
sll1317	slr1639	sll0336	sll0522	slr1463	slr1655	sll1281	slr0974	slr0729	slr1779
sll1108	sll1317	sll0522	sll1299	sll1098	slr1655	sll1281	slr1622	slr0394	slr0729
sll0362	sll1317	sll0522	ssl2084	slr0434	slr1655	sll1196	sll1281	slr0729	slr2047
sll1317	slr0958	sll0522	sll2001	sll1261	slr1655	sll0745	sll1281	slr0729	slr0741
sll1317	slr1720	sll0522	sll1430	sll1099	slr1655	sll1281	sll1815	slr0729	slr1510
sll0179	sll1317	sll0522	sll0573	slr0752	slr1655	sll1059	sll1281	sll1043	slr0729
sll0454	sll1317	sll0522	slr1898	slr0952	slr1655	sll1281	slr1123	slr0400	slr0729
sll1317	sll1553	sll0080	sll0522	slr1511	slr1655	sll1281	sll1760	sll1415	slr0729
sll1317	slr0638	sll0522	slr1022	sll1605	slr1655	sll0469	sll1281	sll1909	slr0729
sll1317	slr0220	sll0522	sll1883	sll0990	slr1655	sll1281	slr1517	sll0373	slr0729
sll1317	slr0357	sll0522	slr1133	sll1633	slr1655	sll0869	sll1281	slr0729	slr2035
sll1317	sll1362	sll0522	slr0444	slr0018	slr1655	sll1281	slr1598	slr0729	slr2132
sll1317	slr1550	sll0522	slr2130	sll0567	slr1655	sll0868	sll1281	slr0729	slr0922
sll1074	sll1317	sll0522	sll1669	slr0884	slr1655	sll1281	slr0994	slr0729	slr0838
sll1317	slr0649	sll0522	slr0585	sll1342	slr1655	sll0379	sll1281	sll1823	slr0729
sll0495	sll1317	sll0522	slr2075	slr1349	slr1655	sll1281	sll1676	sll1056	slr0729
sll1317	sll1425	sll0522	slr2076	sll0593	slr1655	sll0927	sll1281	slr0520	slr0729
sll0502	sll1317	sll0416	sll0522	sll0220	slr1655	sll0996	sll1281	sll0144	slr0729
sll1317	slr1703	sll0522	slr0156	sll0899	slr1655	sll0844	sll1281	sll0467	slr0729
sll0078	sll1317	sll0522	slr1641	slr1655	slr1756	sll1281	sll1615	slr0711	slr0729
sll1317	slr0557	sll0522	slr0542	sll1085	slr1655	sll0202	sll1281	sll0754	slr0729
sll1317	slr1884	sll0522	sll0534	sll1931	slr1655	sll1281	slr0902	sll0569	slr0729
sll1317	slr1031	sll0522	slr0165	sll0083	slr1655	sll1144	sll1281	sll1277	slr0729
sll1317	slr1793	sll0030	sll0522	slr1655	slr1755	sll1281	slr0017	sll1110	slr0729
sll1317	slr0633	sll0522	slr0847	sll0057	slr1655	sll1281	slr1424	sll1865	slr0729
sll1317	slr1278	sll0522	slr0553	sll0017	slr1655	sll1281	slr1423	slr0729	slr1228
sll1317	slr0783	sll0522	slr0812	sll1185	slr1655	sll1281	sll2010	slr0729	slr0808
sll1198	sll1317	sll0522	slr1347	slr0839	slr1655	sll1281	slr0528	slr0082	slr0729
sll0204	sll1317	sll0522	slr1348	sll1876	slr1655	sll1281	slr1351	slr0729	slr0742
sll1317	sll1750	sll0522	slr1791	sll0900	slr1655	sll0622	sll1281	sll0098	slr0729
sll0420	sll1317	sll0522	sll0594	slr0608	slr1655	sll0631	sll1281	slr0729	slr1646
sll1317	slr1256	sll0522	slr0550	slr0652	slr1655	sll1281	slr0787	slr0729	slr1130
sll1317	sll1639	sll0522	sll1058	slr0084	slr1655	sll1281	sll1852	slr0080	slr0729
sll1317	slr1899	sll0522	slr1665	sll1893	slr1655	sll1281	slr1090	slr0551	slr0729
sll0643	sll1317	sll0522	slr0536	slr0500	slr1655	sll0902	sll1281	sll0145	slr0729
sll1317	slr1844	sll0522	slr1874	slr1560	slr1655	sll1281	slr0661	sll0708	slr0729
sll0459	sll1317	sll0522	sll1776	sll1988	slr1655	sll1281	slr0526	slr0679	slr0729

sll0865	sll1317	sll0522	sll0848	slr1289	slr1655	sll1249	sll1281	slr0722	slr0729
sll1317	slr0733	sll0522	sll0897	sll1556	slr1655	sll0892	sll1281	slr0072	slr0729
sll1317	sll1459	sll0522	sll1933	slr1655	ssl3441	sll0660	sll1281	sll1547	slr0729
sll0905	sll1317	sll0522	slr0965	slr0744	slr1655	sll1281	slr1779	sll1234	slr0729
sll0402	slr1643	sll0522	slr1463	slr0974	slr1655	sll1281	slr0394	sll0616	slr0729
sll0728	slr1643	sll0522	sll1098	slr1622	slr1655	sll1281	slr2047	slr0729	slr2073
sll0336	slr1643	sll0522	slr0434	sll1196	slr1655	sll1281	slr0741	slr0729	slr1639
sll1299	slr1643	sll0522	sll1261	sll0745	slr1655	sll1281	slr1510	sll1108	slr0729
slr1643	ssl2084	sll0522	sll1099	sll1815	slr1655	sll1043	sll1281	sll0362	slr0729
sll2001	slr1643	sll0522	slr0752	sll1059	slr1655	sll1281	slr0400	slr0729	slr0958
sll1430	slr1643	sll0522	slr0952	slr1123	slr1655	sll1281	sll1415	slr0729	slr1720
sll0573	slr1643	sll0522	slr1511	sll1760	slr1655	sll1281	sll1909	sll0179	slr0729
slr1643	slr1898	sll0522	sll1605	sll0469	slr1655	sll0373	sll1281	sll0454	slr0729
sll0080	slr1643	sll0522	sll0990	slr1517	slr1655	sll1281	slr2035	sll1553	slr0729
slr1022	slr1643	sll0522	sll1633	sll0869	slr1655	sll1281	slr2132	slr0638	slr0729
sll1883	slr1643	sll0522	slr0018	slr1598	slr1655	sll1281	slr0922	slr0220	slr0729
slr1133	slr1643	sll0522	sll0567	sll0868	slr1655	sll1281	slr0838	slr0357	slr0729
slr0444	slr1643	sll0522	slr0884	slr0994	slr1655	sll1281	sll1823	sll1362	slr0729
slr1643	slr2130	sll0522	sll1342	sll0379	slr1655	sll1056	sll1281	slr0729	slr1550
sll1669	slr1643	sll0522	slr1349	sll1676	slr1655	sll1281	slr0520	sll1074	slr0729
slr0585	slr1643	sll0522	sll0593	sll0927	slr1655	sll0144	sll1281	slr0649	slr0729
slr1643	slr2075	sll0220	sll0522	sll0996	slr1655	sll0467	sll1281	sll0495	slr0729
slr1643	slr2076	sll0522	sll0899	sll0844	slr1655	sll1281	slr0711	sll1425	slr0729
sll0416	slr1643	sll0522	slr1756	sll1615	slr1655	sll0754	sll1281	sll0502	slr0729
slr0156	slr1643	sll0522	sll1085	sll0202	slr1655	sll0569	sll1281	slr0729	slr1703
slr1641	slr1643	sll0522	sll1931	slr0902	slr1655	sll1277	sll1281	sll0078	slr0729
slr0542	slr1643	sll0083	sll0522	sll1144	slr1655	sll1110	sll1281	slr0557	slr0729
sll0534	slr1643	sll0522	slr1755	slr0017	slr1655	sll1281	sll1865	slr0729	slr1884
slr0165	slr1643	sll0057	sll0522	slr1424	slr1655	sll1281	slr1228	slr0729	slr1031
sll0030	slr1643	sll0017	sll0522	slr1423	slr1655	sll1281	slr0808	slr0729	slr1793
slr0847	slr1643	sll0522	sll1185	sll2010	slr1655	sll1281	slr0082	slr0633	slr0729
slr0553	slr1643	sll0522	slr0839	slr0528	slr1655	sll1281	slr0742	slr0729	slr1278
slr0812	slr1643	sll0522	sll1876	slr1351	slr1655	sll0098	sll1281	slr0729	slr0783
slr1347	slr1643	sll0522	sll0900	sll0622	slr1655	sll1281	slr1646	sll1198	slr0729
slr1348	slr1643	sll0522	slr0608	sll0631	slr1655	sll1281	slr1130	sll0204	slr0729
slr1643	slr1791	sll0522	slr0652	slr0787	slr1655	sll1281	slr0080	sll1750	slr0729
sll0594	slr1643	sll0522	slr0084	sll1852	slr1655	sll1281	slr0551	sll0420	slr0729
slr0550	slr1643	sll0522	sll1893	slr1090	slr1655	sll0145	sll1281	slr0729	slr1256
sll1058	slr1643	sll0522	slr0500	sll0902	slr1655	sll0708	sll1281	sll1639	slr0729
slr1643	slr1665	sll0522	slr1560	slr0661	slr1655	sll1281	slr0679	slr0729	slr1899
slr0536	slr1643	sll0522	sll1988	slr0526	slr1655	sll1281	slr0722	sll0643	slr0729
slr1643	slr1874	sll0522	slr1289	sll1249	slr1655	sll1281	slr0072	slr0729	slr1844
sll1776	slr1643	sll0522	sll1556	sll0892	slr1655	sll1281	sll1547	sll0459	slr0729
sll0848	slr1643	sll0522	ssl3441	sll0660	slr1655	sll1234	sll1281	sll0865	slr0729
sll0897	slr1643	sll0522	slr0744	slr1655	slr1779	sll0616	sll1281	slr0729	slr0733
sll1933	slr1643	sll0522	slr0974	slr0394	slr1655	sll1281	slr2073	sll1459	slr0729
slr0965	slr1643	sll0522	slr1622	slr1655	slr2047	sll1281	slr1639	sll0905	slr0729
slr1463	slr1643	sll0522	sll1196	slr0741	slr1655	sll1108	sll1281	sll0402	sll0982
sll1098	slr1643	sll0522	sll0745	slr1510	slr1655	sll0362	sll1281	sll0728	sll0982
slr0434	slr1643	sll0522	sll1815	sll1043	slr1655	sll1281	slr0958	sll0336	sll0982
sll1261	slr1643	sll0522	sll1059	slr0400	slr1655	sll1281	slr1720	sll0982	sll1299

sll1099	slr1643	sll0522	slr1123	sll1415	slr1655	sll0179	sll1281	sll0982	ssl2084
slr0752	slr1643	sll0522	sll1760	sll1909	slr1655	sll0454	sll1281	sll0982	sll2001
slr0952	slr1643	sll0469	sll0522	sll0373	slr1655	sll1281	sll1553	sll0982	sll1430
slr1511	slr1643	sll0522	slr1517	slr1655	slr2035	sll1281	slr0638	sll0573	sll0982
sll1605	slr1643	sll0522	sll0869	slr1655	slr2132	sll1281	slr0220	sll0982	slr1898
sll0990	slr1643	sll0522	slr1598	slr0922	slr1655	sll1281	slr0357	sll0080	sll0982
sll1633	slr1643	sll0522	sll0868	slr0838	slr1655	sll1281	sll1362	sll0982	slr1022
slr0018	slr1643	sll0522	slr0994	sll1823	slr1655	sll1281	slr1550	sll0982	sll1883
sll0567	slr1643	sll0379	sll0522	sll1056	slr1655	sll1074	sll1281	sll0982	slr1133
slr0884	slr1643	sll0522	sll1676	slr0520	slr1655	sll1281	slr0649	sll0982	slr0444
sll1342	slr1643	sll0522	sll0927	sll0144	slr1655	sll0495	sll1281	sll0982	slr2130
slr1349	slr1643	sll0522	sll0996	sll0467	slr1655	sll1281	sll1425	sll0982	sll1669
sll0593	slr1643	sll0522	sll0844	slr0711	slr1655	sll0502	sll1281	sll0982	slr0585
sll0220	slr1643	sll0522	sll1615	sll0754	slr1655	sll1281	slr1703	sll0982	slr2075
sll0899	slr1643	sll0202	sll0522	sll0569	slr1655	sll0078	sll1281	sll0982	slr2076
slr1643	slr1756	sll0522	slr0902	sll1277	slr1655	sll1281	slr0557	sll0416	sll0982
sll1085	slr1643	sll0522	sll1144	sll1110	slr1655	sll1281	slr1884	sll0982	slr0156
sll1931	slr1643	sll0522	slr0017	sll1865	slr1655	sll1281	slr1031	sll0982	slr1641
sll0083	slr1643	sll0522	slr1424	slr1228	slr1655	sll1281	slr1793	sll0982	slr0542
slr1643	slr1755	sll0522	slr1423	slr0808	slr1655	sll1281	slr0633	sll0534	sll0982
sll0057	slr1643	sll0522	sll2010	slr0082	slr1655	sll1281	slr1278	sll0982	slr0165
sll0017	slr1643	sll0522	slr0528	slr0742	slr1655	sll1281	slr0783	sll0030	sll0982
sll1185	slr1643	sll0522	slr1351	sll0098	slr1655	sll1198	sll1281	sll0982	slr0847
slr0839	slr1643	sll0522	sll0622	slr1646	slr1655	sll0204	sll1281	sll0982	slr0553
sll1876	slr1643	sll0522	sll0631	slr1130	slr1655	sll1281	sll1750	sll0982	slr0812
sll0900	slr1643	sll0522	slr0787	slr0080	slr1655	sll0420	sll1281	sll0982	slr1347
slr0608	slr1643	sll0522	sll1852	slr0551	slr1655	sll1281	slr1256	sll0982	slr1348
slr0652	slr1643	sll0522	slr1090	sll0145	slr1655	sll1281	sll1639	sll0982	slr1791
slr0084	slr1643	sll0522	sll0902	sll0708	slr1655	sll1281	slr1899	sll0594	sll0982
sll1893	slr1643	sll0522	slr0661	slr0679	slr1655	sll0643	sll1281	sll0982	slr0550
slr0500	slr1643	sll0522	slr0526	slr0722	slr1655	sll1281	slr1844	sll0982	sll1058
slr1560	slr1643	sll0522	sll1249	slr0072	slr1655	sll0459	sll1281	sll0982	slr1665
sll1988	slr1643	sll0522	sll0892	sll1547	slr1655	sll0865	sll1281	sll0982	slr0536
slr1289	slr1643	sll0522	sll0660	sll1234	slr1655	sll1281	slr0733	sll0982	slr1874
sll1556	slr1643	sll0522	slr1779	sll0616	slr1655	sll1281	sll1459	sll0982	sll1776
slr1643	ssl3441	sll0522	slr0394	slr1655	slr2073	sll0905	sll1281	sll0848	sll0982
slr0744	slr1643	sll0522	slr2047	slr1639	slr1655	sll0402	ssr3383	sll0897	sll0982
slr0974	slr1643	sll0522	slr0741	sll1108	slr1655	sll0728	ssr3383	sll0982	sll1933
slr1622	slr1643	sll0522	slr1510	sll0362	slr1655	sll0336	ssr3383	sll0982	slr0965
sll1196	slr1643	sll0522	sll1043	slr0958	slr1655	sll1299	ssr3383	sll0982	slr1463
sll0745	slr1643	sll0522	slr0400	slr1655	slr1720	ssl2084	ssr3383	sll0982	sll1098
sll1815	slr1643	sll0522	sll1415	sll0179	slr1655	sll2001	ssr3383	sll0982	slr0434
sll1059	slr1643	sll0522	sll1909	sll0454	slr1655	sll1430	ssr3383	sll0982	sll1261
slr1123	slr1643	sll0373	sll0522	sll1553	slr1655	sll0573	ssr3383	sll0982	sll1099
sll1760	slr1643	sll0522	slr2035	slr0638	slr1655	slr1898	ssr3383	sll0982	slr0752
sll0469	slr1643	sll0522	slr2132	slr0220	slr1655	sll0080	ssr3383	sll0982	slr0952
slr1517	slr1643	sll0522	slr0922	slr0357	slr1655	slr1022	ssr3383	sll0982	slr1511
sll0869	slr1643	sll0522	slr0838	sll1362	slr1655	sll1883	ssr3383	sll0982	sll1605
slr1598	slr1643	sll0522	sll1823	slr1550	slr1655	slr1133	ssr3383	sll0982	sll0990
sll0868	slr1643	sll0522	sll1056	sll1074	slr1655	slr0444	ssr3383	sll0982	sll1633
slr0994	slr1643	sll0522	slr0520	slr0649	slr1655	slr2130	ssr3383	sll0982	slr0018

sll0379	slr1643	sll0144	sll0522	sll0495	slr1655	sll1669	ssr3383	sll0567	sll0982
sll1676	slr1643	sll0467	sll0522	sll1425	slr1655	slr0585	ssr3383	sll0982	slr0884
sll0927	slr1643	sll0522	slr0711	sll0502	slr1655	slr2075	ssr3383	sll0982	sll1342
sll0996	slr1643	sll0522	sll0754	slr1655	slr1703	slr2076	ssr3383	sll0982	slr1349
sll0844	slr1643	sll0522	sll0569	sll0078	slr1655	sll0416	ssr3383	sll0593	sll0982
sll1615	slr1643	sll0522	sll1277	slr0557	slr1655	slr0156	ssr3383	sll0220	sll0982
sll0202	slr1643	sll0522	sll1110	slr1655	slr1884	slr1641	ssr3383	sll0899	sll0982
slr0902	slr1643	sll0522	sll1865	slr1031	slr1655	slr0542	ssr3383	sll0982	slr1756
sll1144	slr1643	sll0522	slr1228	slr1655	slr1793	sll0534	ssr3383	sll0982	sll1085
slr0017	slr1643	sll0522	slr0808	slr0633	slr1655	slr0165	ssr3383	sll0982	sll1931
slr1424	slr1643	sll0522	slr0082	slr1278	slr1655	sll0030	ssr3383	sll0083	sll0982
slr1423	slr1643	sll0522	slr0742	slr0783	slr1655	slr0847	ssr3383	sll0982	slr1755
sll2010	slr1643	sll0098	sll0522	sll1198	slr1655	slr0553	ssr3383	sll0057	sll0982
slr0528	slr1643	sll0522	slr1646	sll0204	slr1655	slr0812	ssr3383	sll0017	sll0982
slr1351	slr1643	sll0522	slr1130	sll1750	slr1655	slr1347	ssr3383	sll0982	sll1185
sll0622	slr1643	sll0522	slr0080	sll0420	slr1655	slr1348	ssr3383	sll0982	slr0839
sll0631	slr1643	sll0522	slr0551	slr1256	slr1655	slr1791	ssr3383	sll0982	sll1876
slr0787	slr1643	sll0145	sll0522	sll1639	slr1655	sll0594	ssr3383	sll0900	sll0982
sll1852	slr1643	sll0522	sll0708	slr1655	slr1899	slr0550	ssr3383	sll0982	slr0608
slr1090	slr1643	sll0522	slr0679	sll0643	slr1655	sll1058	ssr3383	sll0982	slr0652
sll0902	slr1643	sll0522	slr0722	slr1655	slr1844	slr1665	ssr3383	sll0982	slr0084
slr0661	slr1643	sll0522	slr0072	sll0459	slr1655	slr0536	ssr3383	sll0982	sll1893
slr0526	slr1643	sll0522	sll1547	sll0865	slr1655	slr1874	ssr3383	sll0982	slr0500
sll1249	slr1643	sll0522	sll1234	slr0733	slr1655	sll1776	ssr3383	sll0982	slr1560
sll0892	slr1643	sll0522	sll0616	sll1459	slr1655	sll0848	ssr3383	sll0982	sll1988
sll0660	slr1643	sll0522	slr2073	sll0905	slr1655	sll0897	ssr3383	sll0982	slr1289
slr1643	slr1779	sll0522	slr1639	sll0402	smr0005	sll1933	ssr3383	sll0982	sll1556
slr0394	slr1643	sll0522	sll1108	sll0728	smr0005	slr0965	ssr3383	sll0982	ssl3441
slr1643	slr2047	sll0362	sll0522	sll0336	smr0005	slr1463	ssr3383	sll0982	slr0744
slr0741	slr1643	sll0522	slr0958	sll1299	smr0005	sll1098	ssr3383	sll0982	slr0974
slr1510	slr1643	sll0522	slr1720	smr0005	ssl2084	slr0434	ssr3383	sll0982	slr1622
sll1043	slr1643	sll0179	sll0522	sll2001	smr0005	sll1261	ssr3383	sll0982	sll1196
slr0400	slr1643	sll0454	sll0522	sll1430	smr0005	sll1099	ssr3383	sll0745	sll0982
sll1415	slr1643	sll0522	sll1553	sll0573	smr0005	slr0752	ssr3383	sll0982	sll1815
sll1909	slr1643	sll0522	slr0638	slr1898	smr0005	slr0952	ssr3383	sll0982	sll1059
sll0373	slr1643	sll0522	slr0220	sll0080	smr0005	slr1511	ssr3383	sll0982	slr1123
slr1643	slr2035	sll0522	slr0357	slr1022	smr0005	sll1605	ssr3383	sll0982	sll1760
slr1643	slr2132	sll0522	sll1362	sll1883	smr0005	sll0990	ssr3383	sll0469	sll0982
slr0922	slr1643	sll0522	slr1550	slr1133	smr0005	sll1633	ssr3383	sll0982	slr1517
slr0838	slr1643	sll0522	sll1074	slr0444	smr0005	slr0018	ssr3383	sll0869	sll0982
sll1823	slr1643	sll0522	slr0649	slr2130	smr0005	sll0567	ssr3383	sll0982	slr1598
sll1056	slr1643	sll0495	sll0522	sll1669	smr0005	slr0884	ssr3383	sll0868	sll0982
slr0520	slr1643	sll0522	sll1425	slr0585	smr0005	sll1342	ssr3383	sll0982	slr0994
sll0144	slr1643	sll0502	sll0522	slr2075	smr0005	slr1349	ssr3383	sll0379	sll0982
sll0467	slr1643	sll0522	slr1703	slr2076	smr0005	sll0593	ssr3383	sll0982	sll1676
slr0711	slr1643	sll0078	sll0522	sll0416	smr0005	sll0220	ssr3383	sll0927	sll0982
sll0754	slr1643	sll0522	slr0557	slr0156	smr0005	sll0899	ssr3383	sll0982	sll0996
sll0569	slr1643	sll0522	slr1884	slr1641	smr0005	slr1756	ssr3383	sll0844	sll0982
sll1277	slr1643	sll0522	slr1031	slr0542	smr0005	sll1085	ssr3383	sll0982	sll1615
sll1110	slr1643	sll0522	slr1793	sll0534	smr0005	sll1931	ssr3383	sll0202	sll0982
sll1865	slr1643	sll0522	slr0633	slr0165	smr0005	sll0083	ssr3383	sll0982	slr0902

slr1228	slr1643	sll0522	slr1278	sll0030	smr0005	slr1755	ssr3383	sll0982	sll1144
slr0808	slr1643	sll0522	slr0783	slr0847	smr0005	sll0057	ssr3383	sll0982	slr0017
slr0082	slr1643	sll0522	sll1198	slr0553	smr0005	sll0017	ssr3383	sll0982	slr1424
slr0742	slr1643	sll0204	sll0522	slr0812	smr0005	sll1185	ssr3383	sll0982	slr1423
sll0098	slr1643	sll0522	sll1750	slr1347	smr0005	slr0839	ssr3383	sll0982	sll2010
slr1643	slr1646	sll0420	sll0522	slr1348	smr0005	sll1876	ssr3383	sll0982	slr0528
slr1130	slr1643	sll0522	slr1256	slr1791	smr0005	sll0900	ssr3383	sll0982	slr1351
slr0080	slr1643	sll0522	sll1639	sll0594	smr0005	slr0608	ssr3383	sll0622	sll0982
slr0551	slr1643	sll0522	slr1899	slr0550	smr0005	slr0652	ssr3383	sll0631	sll0982
sll0145	slr1643	sll0522	sll0643	sll1058	smr0005	slr0084	ssr3383	sll0982	slr0787
sll0708	slr1643	sll0522	slr1844	slr1665	smr0005	sll1893	ssr3383	sll0982	sll1852
slr0679	slr1643	sll0459	sll0522	slr0536	smr0005	slr0500	ssr3383	sll0982	slr1090
slr0722	slr1643	sll0522	sll0865	slr1874	smr0005	slr1560	ssr3383	sll0902	sll0982
slr0072	slr1643	sll0522	slr0733	sll1776	smr0005	sll1988	ssr3383	sll0982	slr0661
sll1547	slr1643	sll0522	sll1459	sll0848	smr0005	slr1289	ssr3383	sll0982	slr0526
sll1234	slr1643	sll0522	sll0905	sll0897	smr0005	sll1556	ssr3383	sll0982	sll1249
sll0616	slr1643	sll0402	slr1963	sll1933	smr0005	ssl3441	ssr3383	sll0892	sll0982
slr1643	slr2073	sll0728	slr1963	slr0965	smr0005	slr0744	ssr3383	sll0660	sll0982
slr1639	slr1643	sll0336	slr1963	slr1463	smr0005	slr0974	ssr3383	sll0982	slr1779
sll1108	slr1643	sll1299	slr1963	sll1098	smr0005	slr1622	ssr3383	sll0982	slr0394
sll0362	slr1643	slr1963	ssl2084	slr0434	smr0005	sll1196	ssr3383	sll0982	slr2047
slr0958	slr1643	sll2001	slr1963	sll1261	smr0005	sll0745	ssr3383	sll0982	slr0741
slr1643	slr1720	sll1430	slr1963	sll1099	smr0005	sll1815	ssr3383	sll0982	slr1510
sll0179	slr1643	sll0573	slr1963	slr0752	smr0005	sll1059	ssr3383	sll0982	sll1043
sll0454	slr1643	slr1898	slr1963	slr0952	smr0005	slr1123	ssr3383	sll0982	slr0400
sll1553	slr1643	sll0080	slr1963	slr1511	smr0005	sll1760	ssr3383	sll0982	sll1415
slr0638	slr1643	slr1022	slr1963	sll1605	smr0005	sll0469	ssr3383	sll0982	sll1909
slr0220	slr1643	sll1883	slr1963	sll0990	smr0005	slr1517	ssr3383	sll0373	sll0982
slr0357	slr1643	slr1133	slr1963	sll1633	smr0005	sll0869	ssr3383	sll0982	slr2035
sll1362	slr1643	slr0444	slr1963	slr0018	smr0005	slr1598	ssr3383	sll0982	slr2132
slr1550	slr1643	slr1963	slr2130	sll0567	smr0005	sll0868	ssr3383	sll0982	slr0922
sll1074	slr1643	sll1669	slr1963	slr0884	smr0005	slr0994	ssr3383	sll0982	slr0838
slr0649	slr1643	slr0585	slr1963	sll1342	smr0005	sll0379	ssr3383	sll0982	sll1823
sll0495	slr1643	slr1963	slr2075	slr1349	smr0005	sll1676	ssr3383	sll0982	sll1056
sll1425	slr1643	slr1963	slr2076	sll0593	smr0005	sll0927	ssr3383	sll0982	slr0520
sll0502	slr1643	sll0416	slr1963	sll0220	smr0005	sll0996	ssr3383	sll0144	sll0982
slr1643	slr1703	slr0156	slr1963	sll0899	smr0005	sll0844	ssr3383	sll0467	sll0982
sll0078	slr1643	slr1641	slr1963	slr1756	smr0005	sll1615	ssr3383	sll0982	slr0711
slr0557	slr1643	slr0542	slr1963	sll1085	smr0005	sll0202	ssr3383	sll0754	sll0982
slr1643	slr1884	sll0534	slr1963	sll1931	smr0005	slr0902	ssr3383	sll0569	sll0982
slr1031	slr1643	slr0165	slr1963	sll0083	smr0005	sll1144	ssr3383	sll0982	sll1277
slr1643	slr1793	sll0030	slr1963	slr1755	smr0005	slr0017	ssr3383	sll0982	sll1110
slr0633	slr1643	slr0847	slr1963	sll0057	smr0005	slr1424	ssr3383	sll0982	sll1865
slr1278	slr1643	slr0553	slr1963	sll0017	smr0005	slr1423	ssr3383	sll0982	slr1228
slr0783	slr1643	slr0812	slr1963	sll1185	smr0005	sll2010	ssr3383	sll0982	slr0808
sll1198	slr1643	slr1347	slr1963	slr0839	smr0005	slr0528	ssr3383	sll0982	slr0082
sll0204	slr1643	slr1348	slr1963	sll1876	smr0005	slr1351	ssr3383	sll0982	slr0742
sll1750	slr1643	slr1791	slr1963	sll0900	smr0005	sll0622	ssr3383	sll0098	sll0982
sll0420	slr1643	sll0594	slr1963	slr0608	smr0005	sll0631	ssr3383	sll0982	slr1646
slr1256	slr1643	slr0550	slr1963	slr0652	smr0005	slr0787	ssr3383	sll0982	slr1130
sll1639	slr1643	sll1058	slr1963	slr0084	smr0005	sll1852	ssr3383	sll0982	slr0080

slr1643	slr1899	slr1665	slr1963	sll1893	smr0005	slr1090	ssr3383	sll0982	slr0551
sll0643	slr1643	slr0536	slr1963	slr0500	smr0005	sll0902	ssr3383	sll0145	sll0982
slr1643	slr1844	slr1874	slr1963	slr1560	smr0005	slr0661	ssr3383	sll0708	sll0982
sll0459	slr1643	sll1776	slr1963	sll1988	smr0005	slr0526	ssr3383	sll0982	slr0679
sll0865	slr1643	sll0848	slr1963	slr1289	smr0005	sll1249	ssr3383	sll0982	slr0722
slr0733	slr1643	sll0897	slr1963	sll1556	smr0005	sll0892	ssr3383	sll0982	slr0072
sll1459	slr1643	sll1933	slr1963	smr0005	ssl3441	sll0660	ssr3383	sll0982	sll1547
sll0905	slr1643	slr0965	slr1963	slr0744	smr0005	slr1779	ssr3383	sll0982	sll1234
sll0402	slr1295	slr1463	slr1963	slr0974	smr0005	slr0394	ssr3383	sll0616	sll0982
sll0728	slr1295	sll1098	slr1963	slr1622	smr0005	slr2047	ssr3383	sll0982	slr2073
sll0336	slr1295	slr0434	slr1963	sll1196	smr0005	slr0741	ssr3383	sll0982	slr1639
sll1299	slr1295	sll1261	slr1963	sll0745	smr0005	slr1510	ssr3383	sll0982	sll1108
slr1295	ssl2084	sll1099	slr1963	sll1815	smr0005	sll1043	ssr3383	sll0362	sll0982
sll2001	slr1295	slr0752	slr1963	sll1059	smr0005	slr0400	ssr3383	sll0982	slr0958
sll1430	slr1295	slr0952	slr1963	slr1123	smr0005	sll1415	ssr3383	sll0982	slr1720
sll0573	slr1295	slr1511	slr1963	sll1760	smr0005	sll1909	ssr3383	sll0179	sll0982
slr1295	slr1898	sll1605	slr1963	sll0469	smr0005	sll0373	ssr3383	sll0454	sll0982
sll0080	slr1295	sll0990	slr1963	slr1517	smr0005	slr2035	ssr3383	sll0982	sll1553
slr1022	slr1295	sll1633	slr1963	sll0869	smr0005	slr2132	ssr3383	sll0982	slr0638
sll1883	slr1295	slr0018	slr1963	slr1598	smr0005	slr0922	ssr3383	sll0982	slr0220
slr1133	slr1295	sll0567	slr1963	sll0868	smr0005	slr0838	ssr3383	sll0982	slr0357
slr0444	slr1295	slr0884	slr1963	slr0994	smr0005	sll1823	ssr3383	sll0982	sll1362
slr1295	slr2130	sll1342	slr1963	sll0379	smr0005	sll1056	ssr3383	sll0982	slr1550
sll1669	slr1295	slr1349	slr1963	sll1676	smr0005	slr0520	ssr3383	sll0982	sll1074
slr0585	slr1295	sll0593	slr1963	sll0927	smr0005	sll0144	ssr3383	sll0982	slr0649
slr1295	slr2075	sll0220	slr1963	sll0996	smr0005	sll0467	ssr3383	sll0495	sll0982
slr1295	slr2076	sll0899	slr1963	sll0844	smr0005	slr0711	ssr3383	sll0982	sll1425
sll0416	slr1295	slr1756	slr1963	sll1615	smr0005	sll0754	ssr3383	sll0982	slr1703
slr0156	slr1295	sll1085	slr1963	sll0202	smr0005	sll0569	ssr3383	sll0078	sll0982
slr1295	slr1641	sll1931	slr1963	slr0902	smr0005	sll1277	ssr3383	sll0982	slr0557
slr0542	slr1295	sll0083	slr1963	sll1144	smr0005	sll1110	ssr3383	sll0982	slr1884
sll0534	slr1295	slr1755	slr1963	slr0017	smr0005	sll1865	ssr3383	sll0982	slr1031
slr0165	slr1295	sll0057	slr1963	slr1424	smr0005	slr1228	ssr3383	sll0982	slr1793
sll0030	slr1295	sll0017	slr1963	slr1423	smr0005	slr0808	ssr3383	sll0982	slr0633
slr0847	slr1295	sll1185	slr1963	sll2010	smr0005	slr0082	ssr3383	sll0982	slr1278
slr0553	slr1295	slr0839	slr1963	slr0528	smr0005	slr0742	ssr3383	sll0982	slr0783
slr0812	slr1295	sll1876	slr1963	slr1351	smr0005	sll0098	ssr3383	sll0982	sll1198
slr1295	slr1347	sll0900	slr1963	sll0622	smr0005	slr1646	ssr3383	sll0204	sll0982
slr1295	slr1348	slr0608	slr1963	sll0631	smr0005	slr1130	ssr3383	sll0982	sll1750
slr1295	slr1791	slr0652	slr1963	slr0787	smr0005	slr0080	ssr3383	sll0420	sll0982
sll0594	slr1295	slr0084	slr1963	sll1852	smr0005	slr0551	ssr3383	sll0982	slr1256
slr0550	slr1295	sll1893	slr1963	slr1090	smr0005	sll0145	ssr3383	sll0982	sll1639
sll1058	slr1295	slr0500	slr1963	sll0902	smr0005	sll0708	ssr3383	sll0982	slr1899
slr1295	slr1665	slr1560	slr1963	slr0661	smr0005	slr0679	ssr3383	sll0643	sll0982
slr0536	slr1295	sll1988	slr1963	slr0526	smr0005	slr0722	ssr3383	sll0982	slr1844
slr1295	slr1874	slr1289	slr1963	sll1249	smr0005	slr0072	ssr3383	sll0459	sll0982
sll1776	slr1295	sll1556	slr1963	sll0892	smr0005	sll1547	ssr3383	sll0865	sll0982
sll0848	slr1295	slr1963	ssl3441	sll0660	smr0005	sll1234	ssr3383	sll0982	slr0733
sll0897	slr1295	slr0744	slr1963	slr1779	smr0005	sll0616	ssr3383	sll0982	sll1459
sll1933	slr1295	slr0974	slr1963	slr0394	smr0005	slr2073	ssr3383	sll0905	sll0982
slr0965	slr1295	slr1622	slr1963	slr2047	smr0005	slr1639	ssr3383	sll0047	sll0402

slr1295	slr1463	sll1196	slr1963	slr0741	smr0005	sll1108	ssr3383	sll0047	sll0728
sll1098	slr1295	sll0745	slr1963	slr1510	smr0005	sll0362	ssr3383	sll0047	sll0336
slr0434	slr1295	sll1815	slr1963	sll1043	smr0005	slr0958	ssr3383	sll0047	sll1299
sll1261	slr1295	sll1059	slr1963	slr0400	smr0005	slr1720	ssr3383	sll0047	ssl2084
sll1099	slr1295	slr1123	slr1963	sll1415	smr0005	sll0179	ssr3383	sll0047	sll2001
slr0752	slr1295	sll1760	slr1963	sll1909	smr0005	sll0454	ssr3383	sll0047	sll1430
slr0952	slr1295	sll0469	slr1963	sll0373	smr0005	sll1553	ssr3383	sll0047	sll0573
slr1295	slr1511	slr1517	slr1963	slr2035	smr0005	slr0638	ssr3383	sll0047	slr1898
sll1605	slr1295	sll0869	slr1963	slr2132	smr0005	slr0220	ssr3383	sll0047	sll0080
sll0990	slr1295	slr1598	slr1963	slr0922	smr0005	slr0357	ssr3383	sll0047	slr1022
sll1633	slr1295	sll0868	slr1963	slr0838	smr0005	sll1362	ssr3383	sll0047	sll1883
slr0018	slr1295	slr0994	slr1963	sll1823	smr0005	slr1550	ssr3383	sll0047	slr1133
sll0567	slr1295	sll0379	slr1963	sll1056	smr0005	sll1074	ssr3383	sll0047	slr0444
slr0884	slr1295	sll1676	slr1963	slr0520	smr0005	slr0649	ssr3383	sll0047	slr2130
sll1342	slr1295	sll0927	slr1963	sll0144	smr0005	sll0495	ssr3383	sll0047	sll1669
slr1295	slr1349	sll0996	slr1963	sll0467	smr0005	sll1425	ssr3383	sll0047	slr0585
sll0593	slr1295	sll0844	slr1963	slr0711	smr0005	sll0502	ssr3383	sll0047	slr2075
sll0220	slr1295	sll1615	slr1963	sll0754	smr0005	slr1703	ssr3383	sll0047	slr2076
sll0899	slr1295	sll0202	slr1963	sll0569	smr0005	sll0078	ssr3383	sll0047	sll0416
slr1295	slr1756	slr0902	slr1963	sll1277	smr0005	slr0557	ssr3383	sll0047	slr0156
sll1085	slr1295	sll1144	slr1963	sll1110	smr0005	slr1884	ssr3383	sll0047	slr1641
sll1931	slr1295	slr0017	slr1963	sll1865	smr0005	slr1031	ssr3383	sll0047	slr0542
sll0083	slr1295	slr1424	slr1963	slr1228	smr0005	slr1793	ssr3383	sll0047	sll0534
slr1295	slr1755	slr1423	slr1963	slr0808	smr0005	slr0633	ssr3383	sll0047	slr0165
sll0057	slr1295	sll2010	slr1963	slr0082	smr0005	slr1278	ssr3383	sll0030	sll0047
sll0017	slr1295	slr0528	slr1963	slr0742	smr0005	slr0783	ssr3383	sll0047	slr0847
sll1185	slr1295	slr1351	slr1963	sll0098	smr0005	sll1198	ssr3383	sll0047	slr0553
slr0839	slr1295	sll0622	slr1963	slr1646	smr0005	sll0204	ssr3383	sll0047	slr0812
sll1876	slr1295	sll0631	slr1963	slr1130	smr0005	sll1750	ssr3383	sll0047	slr1347
sll0900	slr1295	slr0787	slr1963	slr0080	smr0005	sll0420	ssr3383	sll0047	slr1348
slr0608	slr1295	sll1852	slr1963	slr0551	smr0005	slr1256	ssr3383	sll0047	slr1791
slr0652	slr1295	slr1090	slr1963	sll0145	smr0005	sll1639	ssr3383	sll0047	sll0594
slr0084	slr1295	sll0902	slr1963	sll0708	smr0005	slr1899	ssr3383	sll0047	slr0550
sll1893	slr1295	slr0661	slr1963	slr0679	smr0005	sll0643	ssr3383	sll0047	sll1058
slr0500	slr1295	slr0526	slr1963	slr0722	smr0005	slr1844	ssr3383	sll0047	slr1665
slr1295	slr1560	sll1249	slr1963	slr0072	smr0005	sll0459	ssr3383	sll0047	slr0536
sll1988	slr1295	sll0892	slr1963	sll1547	smr0005	sll0865	ssr3383	sll0047	slr1874
slr1289	slr1295	sll0660	slr1963	sll1234	smr0005	slr0733	ssr3383	sll0047	sll1776
sll1556	slr1295	slr1779	slr1963	sll0616	smr0005	sll1459	ssr3383	sll0047	sll0848
slr1295	ssl3441	slr0394	slr1963	slr2073	smr0005	sll0905	ssr3383	sll0047	sll0897
slr0744	slr1295	slr1963	slr2047	slr1639	smr0005	sll0402	sll1580	sll0047	sll1933
slr0974	slr1295	slr0741	slr1963	sll1108	smr0005	sll0728	sll1580	sll0047	slr0965
slr1295	slr1622	slr1510	slr1963	sll0362	smr0005	sll0336	sll1580	sll0047	slr1463
sll1196	slr1295	sll1043	slr1963	slr0958	smr0005	sll1299	sll1580	sll0047	sll1098
sll0745	slr1295	slr0400	slr1963	slr1720	smr0005	sll1580	ssl2084	sll0047	slr0434
sll1815	slr1295	sll1415	slr1963	sll0179	smr0005	sll1580	sll2001	sll0047	sll1261
sll1059	slr1295	sll1909	slr1963	sll0454	smr0005	sll1430	sll1580	sll0047	sll1099
slr1123	slr1295	sll0373	slr1963	sll1553	smr0005	sll0573	sll1580	sll0047	slr0752
sll1760	slr1295	slr1963	slr2035	slr0638	smr0005	sll1580	slr1898	sll0047	slr0952
sll0469	slr1295	slr1963	slr2132	slr0220	smr0005	sll0080	sll1580	sll0047	slr1511
slr1295	slr1517	slr0922	slr1963	slr0357	smr0005	sll1580	slr1022	sll0047	sll1605

sll0869	slr1295	slr0838	slr1963	sll1362	smr0005	sll1580	sll1883	sll0047	sll0990
slr1295	slr1598	sll1823	slr1963	slr1550	smr0005	sll1580	slr1133	sll0047	sll1633
sll0868	slr1295	sll1056	slr1963	sll1074	smr0005	sll1580	slr0444	sll0047	slr0018
slr0994	slr1295	slr0520	slr1963	slr0649	smr0005	sll1580	slr2130	sll0047	sll0567
sll0379	slr1295	sll0144	slr1963	sll0495	smr0005	sll1580	sll1669	sll0047	slr0884
sll1676	slr1295	sll0467	slr1963	sll1425	smr0005	sll1580	slr0585	sll0047	sll1342
sll0927	slr1295	slr0711	slr1963	sll0502	smr0005	sll1580	slr2075	sll0047	slr1349
sll0996	slr1295	sll0754	slr1963	slr1703	smr0005	sll1580	slr2076	sll0047	sll0593
sll0844	slr1295	sll0569	slr1963	sll0078	smr0005	sll0416	sll1580	sll0047	sll0220
sll1615	slr1295	sll1277	slr1963	slr0557	smr0005	sll1580	slr0156	sll0047	sll0899
sll0202	slr1295	sll1110	slr1963	slr1884	smr0005	sll1580	slr1641	sll0047	slr1756
slr0902	slr1295	sll1865	slr1963	slr1031	smr0005	sll1580	slr0542	sll0047	sll1085
sll1144	slr1295	slr1228	slr1963	slr1793	smr0005	sll0534	sll1580	sll0047	sll1931
slr0017	slr1295	slr0808	slr1963	slr0633	smr0005	sll1580	slr0165	sll0047	sll0083
slr1295	slr1424	slr0082	slr1963	slr1278	smr0005	sll0030	sll1580	sll0047	slr1755
slr1295	slr1423	slr0742	slr1963	slr0783	smr0005	sll1580	slr0847	sll0047	sll0057
sll2010	slr1295	sll0098	slr1963	sll1198	smr0005	sll1580	slr0553	sll0017	sll0047
slr0528	slr1295	slr1646	slr1963	sll0204	smr0005	sll1580	slr0812	sll0047	sll1185
slr1295	slr1351	slr1130	slr1963	sll1750	smr0005	sll1580	slr1347	sll0047	slr0839
sll0622	slr1295	slr0080	slr1963	sll0420	smr0005	sll1580	slr1348	sll0047	sll1876
sll0631	slr1295	slr0551	slr1963	slr1256	smr0005	sll1580	slr1791	sll0047	sll0900
slr0787	slr1295	sll0145	slr1963	sll1639	smr0005	sll0594	sll1580	sll0047	slr0608
sll1852	slr1295	sll0708	slr1963	slr1899	smr0005	sll1580	slr0550	sll0047	slr0652
slr1090	slr1295	slr0679	slr1963	sll0643	smr0005	sll1058	sll1580	sll0047	slr0084
sll0902	slr1295	slr0722	slr1963	slr1844	smr0005	sll1580	slr1665	sll0047	sll1893
slr0661	slr1295	slr0072	slr1963	sll0459	smr0005	sll1580	slr0536	sll0047	slr0500
slr0526	slr1295	sll1547	slr1963	sll0865	smr0005	sll1580	slr1874	sll0047	slr1560
sll1249	slr1295	sll1234	slr1963	slr0733	smr0005	sll1580	sll1776	sll0047	sll1988
sll0892	slr1295	sll0616	slr1963	sll1459	smr0005	sll0848	sll1580	sll0047	slr1289
sll0660	slr1295	slr1963	slr2073	sll0905	smr0005	sll0897	sll1580	sll0047	sll1556
slr1295	slr1779	slr1639	slr1963	sll0402	sll1398	sll1580	sll1933	sll0047	ssl3441
slr0394	slr1295	sll1108	slr1963	sll0728	sll1398	sll1580	slr0965	sll0047	slr0744
slr1295	slr2047	sll0362	slr1963	sll0336	sll1398	sll1580	slr1463	sll0047	slr0974
slr0741	slr1295	slr0958	slr1963	sll1299	sll1398	sll1098	sll1580	sll0047	slr1622
slr1295	slr1510	slr1720	slr1963	sll1398	ssl2084	sll1580	slr0434	sll0047	sll1196
sll1043	slr1295	sll0179	slr1963	sll1398	sll2001	sll1261	sll1580	sll0047	sll0745
slr0400	slr1295	sll0454	slr1963	sll1398	sll1430	sll1099	sll1580	sll0047	sll1815
sll1415	slr1295	sll1553	slr1963	sll0573	sll1398	sll1580	slr0752	sll0047	sll1059
sll1909	slr1295	slr0638	slr1963	sll1398	slr1898	sll1580	slr0952	sll0047	slr1123
sll0373	slr1295	slr0220	slr1963	sll0080	sll1398	sll1580	slr1511	sll0047	sll1760
slr1295	slr2035	slr0357	slr1963	sll1398	slr1022	sll1580	sll1605	sll0047	sll0469
slr1295	slr2132	sll1362	slr1963	sll1398	sll1883	sll0990	sll1580	sll0047	slr1517
slr0922	slr1295	slr1550	slr1963	sll1398	slr1133	sll1580	sll1633	sll0047	sll0869
slr0838	slr1295	sll1074	slr1963	sll1398	slr0444	sll1580	slr0018	sll0047	slr1598
sll1823	slr1295	slr0649	slr1963	sll1398	slr2130	sll0567	sll1580	sll0047	sll0868
sll1056	slr1295	sll0495	slr1963	sll1398	sll1669	sll1580	slr0884	sll0047	slr0994
slr0520	slr1295	sll1425	slr1963	sll1398	slr0585	sll1342	sll1580	sll0047	sll0379
sll0144	slr1295	sll0502	slr1963	sll1398	slr2075	sll1580	slr1349	sll0047	sll1676
sll0467	slr1295	slr1703	slr1963	sll1398	slr2076	sll0593	sll1580	sll0047	sll0927
slr0711	slr1295	sll0078	slr1963	sll0416	sll1398	sll0220	sll1580	sll0047	sll0996
sll0754	slr1295	slr0557	slr1963	sll1398	slr0156	sll0899	sll1580	sll0047	sll0844

sll0569	slr1295	slr1884	slr1963	sll1398	slr1641	sll1580	slr1756	sll0047	sll1615
sll1277	slr1295	slr1031	slr1963	sll1398	slr0542	sll1085	sll1580	sll0047	sll0202
sll1110	slr1295	slr1793	slr1963	sll0534	sll1398	sll1580	sll1931	sll0047	slr0902
sll1865	slr1295	slr0633	slr1963	sll1398	slr0165	sll0083	sll1580	sll0047	sll1144
slr1228	slr1295	slr1278	slr1963	sll0030	sll1398	sll1580	slr1755	sll0047	slr0017
slr0808	slr1295	slr0783	slr1963	sll1398	slr0847	sll0057	sll1580	sll0047	slr1424
slr0082	slr1295	sll1198	slr1963	sll1398	slr0553	sll0017	sll1580	sll0047	slr1423
slr0742	slr1295	sll0204	slr1963	sll1398	slr0812	sll1185	sll1580	sll0047	sll2010
sll0098	slr1295	sll1750	slr1963	sll1398	slr1347	sll1580	slr0839	sll0047	slr0528
slr1295	slr1646	sll0420	slr1963	sll1398	slr1348	sll1580	sll1876	sll0047	slr1351
slr1130	slr1295	slr1256	slr1963	sll1398	slr1791	sll0900	sll1580	sll0047	sll0622
slr0080	slr1295	sll1639	slr1963	sll0594	sll1398	sll1580	slr0608	sll0047	sll0631
slr0551	slr1295	slr1899	slr1963	sll1398	slr0550	sll1580	slr0652	sll0047	slr0787
sll0145	slr1295	sll0643	slr1963	sll1058	sll1398	sll1580	slr0084	sll0047	sll1852
sll0708	slr1295	slr1844	slr1963	sll1398	slr1665	sll1580	sll1893	sll0047	slr1090
slr0679	slr1295	sll0459	slr1963	sll1398	slr0536	sll1580	slr0500	sll0047	sll0902
slr0722	slr1295	sll0865	slr1963	sll1398	slr1874	sll1580	slr1560	sll0047	slr0661
slr0072	slr1295	slr0733	slr1963	sll1398	sll1776	sll1580	sll1988	sll0047	slr0526
sll1547	slr1295	sll1459	slr1963	sll0848	sll1398	sll1580	slr1289	sll0047	sll1249
sll1234	slr1295	sll0905	slr1963	sll0897	sll1398	sll1556	sll1580	sll0047	sll0892
sll0616	slr1295	sll0402	slr0343	sll1398	sll1933	sll1580	ssl3441	sll0047	sll0660
slr1295	slr2073	sll0728	slr0343	sll1398	slr0965	sll1580	slr0744	sll0047	slr1779
slr1295	slr1639	sll0336	slr0343	sll1398	slr1463	sll1580	slr0974	sll0047	slr0394
sll1108	slr1295	sll1299	slr0343	sll1098	sll1398	sll1580	slr1622	sll0047	slr2047
sll0362	slr1295	slr0343	ssl2084	sll1398	slr0434	sll1196	sll1580	sll0047	slr0741
slr0958	slr1295	sll2001	slr0343	sll1261	sll1398	sll0745	sll1580	sll0047	slr1510
slr1295	slr1720	sll1430	slr0343	sll1099	sll1398	sll1580	sll1815	sll0047	sll1043
sll0179	slr1295	sll0573	slr0343	sll1398	slr0752	sll1059	sll1580	sll0047	slr0400
sll0454	slr1295	slr0343	slr1898	sll1398	slr0952	sll1580	slr1123	sll0047	sll1415
sll1553	slr1295	sll0080	slr0343	sll1398	slr1511	sll1580	sll1760	sll0047	sll1909
slr0638	slr1295	slr0343	slr1022	sll1398	sll1605	sll0469	sll1580	sll0047	sll0373
slr0220	slr1295	sll1883	slr0343	sll0990	sll1398	sll1580	slr1517	sll0047	slr2035
slr0357	slr1295	slr0343	slr1133	sll1398	sll1633	sll0869	sll1580	sll0047	slr2132
sll1362	slr1295	slr0343	slr0444	sll1398	slr0018	sll1580	slr1598	sll0047	slr0922
slr1295	slr1550	slr0343	slr2130	sll0567	sll1398	sll0868	sll1580	sll0047	slr0838
sll1074	slr1295	sll1669	slr0343	sll1398	slr0884	sll1580	slr0994	sll0047	sll1823
slr0649	slr1295	slr0343	slr0585	sll1342	sll1398	sll0379	sll1580	sll0047	sll1056
sll0495	slr1295	slr0343	slr2075	sll1398	slr1349	sll1580	sll1676	sll0047	slr0520
sll1425	slr1295	slr0343	slr2076	sll0593	sll1398	sll0927	sll1580	sll0047	sll0144
sll0502	slr1295	sll0416	slr0343	sll0220	sll1398	sll0996	sll1580	sll0047	sll0467
slr1295	slr1703	slr0156	slr0343	sll0899	sll1398	sll0844	sll1580	sll0047	slr0711
sll0078	slr1295	slr0343	slr1641	sll1398	slr1756	sll1580	sll1615	sll0047	sll0754
slr0557	slr1295	slr0343	slr0542	sll1085	sll1398	sll0202	sll1580	sll0047	sll0569
slr1295	slr1884	sll0534	slr0343	sll1398	sll1931	sll1580	slr0902	sll0047	sll1277
slr1031	slr1295	slr0165	slr0343	sll0083	sll1398	sll1144	sll1580	sll0047	sll1110
slr1295	slr1793	sll0030	slr0343	sll1398	slr1755	sll1580	slr0017	sll0047	sll1865
slr0633	slr1295	slr0343	slr0847	sll0057	sll1398	sll1580	slr1424	sll0047	slr1228
slr1278	slr1295	slr0343	slr0553	sll0017	sll1398	sll1580	slr1423	sll0047	slr0808
slr0783	slr1295	slr0343	slr0812	sll1185	sll1398	sll1580	sll2010	sll0047	slr0082
sll1198	slr1295	slr0343	slr1347	sll1398	slr0839	sll1580	slr0528	sll0047	slr0742
sll0204	slr1295	slr0343	slr1348	sll1398	sll1876	sll1580	slr1351	sll0047	sll0098

sll1750	slr1295
sll0420	slr1295
slr1256	slr1295
sll1639	slr1295
slr1295	slr1899
sll0643	slr1295
slr1295	slr1844
sll0459	slr1295
sll0865	slr1295
slr0733	slr1295
sll1459	slr1295
sll0905	slr1295
sll0402	slr0513
sll0728	slr0513
sll0336	slr0513
sll1299	slr0513
slr0513	ssl2084
sll2001	slr0513
sll1430	slr0513
sll0573	slr0513
slr0513	slr1898
sll0080	slr0513
slr0513	slr1022
sll1883	slr0513
slr0513	slr1133
slr0444	slr0513
slr0513	slr2130
sll1669	slr0513
slr0513	slr0585
slr0513	slr2075
slr0513	slr2076
sll0416	slr0513
slr0156	slr0513
slr0513	slr1641
slr0513	slr0542
sll0534	slr0513
slr0165	slr0513
sll0030	slr0513
slr0513	slr0847
slr0513	slr0553
slr0513	slr0812
slr0513	slr1347
slr0513	slr1348
slr0513	slr1791
sll0594	slr0513
slr0513	slr0550
sll1058	slr0513
slr0513	slr1665
slr0513	slr0536
slr0513	slr1874
sll1776	slr0513

slr0343	slr1791
sll0594	slr0343
slr0343	slr0550
sll1058	slr0343
slr0343	slr1665
slr0343	slr0536
slr0343	slr1874
sll1776	slr0343
sll0848	slr0343
sll0897	slr0343
sll1933	slr0343
slr0343	slr0965
slr0343	slr1463
sll1098	slr0343
slr0343	slr0434
sll1261	slr0343
sll1099	slr0343
slr0343	slr0752
slr0343	slr0952
slr0343	slr1511
sll1605	slr0343
sll0990	slr0343
sll1633	slr0343
slr0018	slr0343
sll0567	slr0343
slr0343	slr0884
sll1342	slr0343
slr0343	slr1349
sll0593	slr0343
sll0220	slr0343
sll0899	slr0343
slr0343	slr1756
sll1085	slr0343
sll1931	slr0343
sll0083	slr0343
slr0343	slr1755
sll0057	slr0343
sll0017	slr0343
sll1185	slr0343
slr0343	slr0839
sll1876	slr0343
sll0900	slr0343
slr0343	slr0608
slr0343	slr0652
slr0084	slr0343
sll1893	slr0343
slr0343	slr0500
slr0343	slr1560
sll1988	slr0343
slr0343	slr1289
sll1556	slr0343

sll0900	sll1398
sll1398	slr0608
sll1398	slr0652
sll1398	slr0084
sll1398	sll1893
sll1398	slr0500
sll1398	slr1560
sll1398	sll1988
sll1398	slr1289
sll1398	sll1556
sll1398	ssl3441
sll1398	slr0744
sll1398	slr0974
sll1398	slr1622
sll1196	sll1398
sll0745	sll1398
sll1398	sll1815
sll1059	sll1398
sll1398	slr1123
sll1398	sll1760
sll0469	sll1398
sll1398	slr1517
sll0869	sll1398
sll1398	slr1598
sll0868	sll1398
sll1398	slr0994
sll0379	sll1398
sll1398	sll1676
sll0927	sll1398
sll0996	sll1398
sll0844	sll1398
sll1398	sll1615
sll0202	sll1398
sll1398	slr0902
sll1144	sll1398
sll1398	slr0017
sll1398	slr1424
sll1398	slr1423
sll1398	sll2010
sll1398	slr0528
sll1398	slr1351
sll0622	sll1398
sll0631	sll1398
sll1398	slr0787
sll1398	sll1852
sll1398	slr1090
sll0902	sll1398
sll1398	slr0661
sll1398	slr0526
sll1249	sll1398
sll0892	sll1398

sll0622	sll1580
sll0631	sll1580
sll1580	slr0787
sll1580	sll1852
sll1580	slr1090
sll0902	sll1580
sll1580	slr0661
sll1580	slr0526
sll1249	sll1580
sll0892	sll1580
sll0660	sll1580
sll1580	slr1779
sll1580	slr0394
sll1580	slr2047
sll1580	slr0741
sll1580	slr1510
sll1043	sll1580
sll1580	slr0400
sll1415	sll1580
sll1580	sll1909
sll0373	sll1580
sll1580	slr2035
sll1580	slr2132
sll1580	slr0922
sll1580	slr0838
sll1580	sll1823
sll1056	sll1580
sll1580	slr0520
sll0144	sll1580
sll0467	sll1580
sll1580	slr0711
sll0754	sll1580
sll0569	sll1580
sll1277	sll1580
sll1110	sll1580
sll1580	sll1865
sll1580	slr1228
sll1580	slr0808
sll1580	slr0082
sll1580	slr0742
sll0098	sll1580
sll1580	slr1646
sll1580	slr1130
sll1580	slr0080
sll1580	slr0551
sll0145	sll1580
sll0708	sll1580
sll1580	slr0679
sll1580	slr0722
sll1580	slr0072
sll1547	sll1580

sll0047	slr1646
sll0047	slr1130
sll0047	slr0080
sll0047	slr0551
sll0047	sll0145
sll0047	sll0708
sll0047	slr0679
sll0047	slr0722
sll0047	slr0072
sll0047	sll1547
sll0047	sll1234
sll0047	sll0616
sll0047	slr2073
sll0047	slr1639
sll0047	sll1108
sll0047	sll0362
sll0047	slr0958
sll0047	slr1720
sll0047	sll0179
sll0047	sll0454
sll0047	sll1553
sll0047	slr0638
sll0047	slr0220
sll0047	slr0357
sll0047	sll1362
sll0047	slr1550
sll0047	sll1074
sll0047	slr0649
sll0047	sll0495
sll0047	sll1425
sll0047	sll0502
sll0047	slr1703
sll0047	sll0078
sll0047	slr0557
sll0047	slr1884
sll0047	slr1031
sll0047	slr1793
sll0047	slr0633
sll0047	slr1278
sll0047	slr0783
sll0047	sll1198
sll0047	sll0204
sll0047	sll1750
sll0047	sll0420
sll0047	slr1256
sll0047	sll1639
sll0047	slr1899
sll0047	sll0643
sll0047	slr1844
sll0047	sll0459
sll0047	sll0865

sll0848	slr0513	slr0343	ssl3441	sll0660	sll1398	sll1234	sll1580	sll0047	slr0733
sll0897	slr0513	slr0343	slr0744	sll1398	slr1779	sll0616	sll1580	sll0047	sll1459
sll1933	slr0513	slr0343	slr0974	sll1398	slr0394	sll1580	slr2073	sll0047	sll0905
slr0513	slr0965	slr0343	slr1622	sll1398	slr2047	sll1580	slr1639	sll0402	slr0823
slr0513	slr1463	sll1196	slr0343	sll1398	slr0741	sll1108	sll1580	sll0728	slr0823
sll1098	slr0513	sll0745	slr0343	sll1398	slr1510	sll0362	sll1580	sll0336	slr0823
slr0434	slr0513	sll1815	slr0343	sll1043	sll1398	sll1580	slr0958	sll1299	slr0823
sll1261	slr0513	sll1059	slr0343	sll1398	slr0400	sll1580	slr1720	slr0823	ssl2084
sll1099	slr0513	slr0343	slr1123	sll1398	sll1415	sll0179	sll1580	sll2001	slr0823
slr0513	slr0752	sll1760	slr0343	sll1398	sll1909	sll0454	sll1580	sll1430	slr0823
slr0513	slr0952	sll0469	slr0343	sll0373	sll1398	sll1553	sll1580	sll0573	slr0823
sll1605	slr0513	slr0343	slr1517	sll1398	slr2035	sll1580	slr0638	slr0823	slr1898
sll0990	slr0513	sll0869	slr0343	sll1398	slr2132	sll1580	slr0220	sll0080	slr0823
sll1633	slr0513	slr0343	slr1598	sll1398	slr0922	sll1580	slr0357	slr0823	slr1022
slr0018	slr0513	sll0868	slr0343	sll1398	slr0838	sll1362	sll1580	sll1883	slr0823
sll0567	slr0513	slr0343	slr0994	sll1398	sll1823	sll1580	slr1550	slr0823	slr1133
slr0513	slr0884	sll0379	slr0343	sll1056	sll1398	sll1074	sll1580	slr0444	slr0823
sll1342	slr0513	sll1676	slr0343	sll1398	slr0520	sll1580	slr0649	slr0823	slr2130
slr0513	slr1349	sll0927	slr0343	sll0144	sll1398	sll0495	sll1580	sll1669	slr0823
sll0593	slr0513	sll0996	slr0343	sll0467	sll1398	sll1425	sll1580	slr0585	slr0823
sll0220	slr0513	sll0844	slr0343	sll1398	slr0711	sll0502	sll1580	slr0823	slr2075
sll0899	slr0513	sll1615	slr0343	sll0754	sll1398	sll1580	slr1703	slr0823	slr2076
slr0513	slr1756	sll0202	slr0343	sll0569	sll1398	sll0078	sll1580	sll0416	slr0823
sll1085	slr0513	slr0343	slr0902	sll1277	sll1398	sll1580	slr0557	slr0156	slr0823
sll1931	slr0513	sll1144	slr0343	sll1110	sll1398	sll1580	slr1884	slr0823	slr1641
sll0083	slr0513	slr0017	slr0343	sll1398	sll1865	sll1580	slr1031	slr0542	slr0823
slr0513	slr1755	slr0343	slr1424	sll1398	slr1228	sll1580	slr1793	sll0534	slr0823
sll0057	slr0513	slr0343	slr1423	sll1398	slr0808	sll1580	slr0633	slr0165	slr0823
sll0017	slr0513	sll2010	slr0343	sll1398	slr0082	sll1580	slr1278	sll0030	slr0823
sll1185	slr0513	slr0343	slr0528	sll1398	slr0742	sll1580	slr0783	slr0823	slr0847
slr0513	slr0839	slr0343	slr1351	sll0098	sll1398	sll1198	sll1580	slr0553	slr0823
sll1876	slr0513	sll0622	slr0343	sll1398	slr1646	sll0204	sll1580	slr0812	slr0823
sll0900	slr0513	sll0631	slr0343	sll1398	slr1130	sll1580	sll1750	slr0823	slr1347
slr0513	slr0608	slr0343	slr0787	sll1398	slr0080	sll0420	sll1580	slr0823	slr1348
slr0513	slr0652	sll1852	slr0343	sll1398	slr0551	sll1580	slr1256	slr0823	slr1791
slr0084	slr0513	slr0343	slr1090	sll0145	sll1398	sll1580	sll1639	sll0594	slr0823
sll1893	slr0513	sll0902	slr0343	sll0708	sll1398	sll1580	slr1899	slr0550	slr0823
slr0500	slr0513	slr0343	slr0661	sll1398	slr0679	sll0643	sll1580	sll1058	slr0823
slr0513	slr1560	slr0343	slr0526	sll1398	slr0722	sll1580	slr1844	slr0823	slr1665
sll1988	slr0513	sll1249	slr0343	sll1398	slr0072	sll0459	sll1580	slr0536	slr0823
slr0513	slr1289	sll0892	slr0343	sll1398	sll1547	sll0865	sll1580	slr0823	slr1874
sll1556	slr0513	sll0660	slr0343	sll1234	sll1398	sll1580	slr0733	sll1776	slr0823
slr0513	ssl3441	slr0343	slr1779	sll0616	sll1398	sll1459	sll1580	sll0848	slr0823
slr0513	slr0744	slr0343	slr0394	sll1398	slr2073	sll0905	sll1580	sll0897	slr0823
slr0513	slr0974	slr0343	slr2047	sll1398	slr1639	sll0402	sll1579	sll1933	slr0823
slr0513	slr1622	slr0343	slr0741	sll1108	sll1398	sll0728	sll1579	slr0823	slr0965
sll1196	slr0513	slr0343	slr1510	sll0362	sll1398	sll0336	sll1579	slr0823	slr1463
sll0745	slr0513	sll1043	slr0343	sll1398	slr0958	sll1299	sll1579	sll1098	slr0823
sll1815	slr0513	slr0343	slr0400	sll1398	slr1720	sll1579	ssl2084	slr0434	slr0823
sll1059	slr0513	sll1415	slr0343	sll0179	sll1398	sll1579	sll2001	sll1261	slr0823
slr0513	slr1123	sll1909	slr0343	sll0454	sll1398	sll1430	sll1579	sll1099	slr0823

sll1760	slr0513	sll0373	slr0343	sll1398	sll1553	sll0573	sll1579	slr0752	slr0823
sll0469	slr0513	slr0343	slr2035	sll1398	slr0638	sll1579	slr1898	slr0823	slr0952
slr0513	slr1517	slr0343	slr2132	sll1398	slr0220	sll0080	sll1579	slr0823	slr1511
sll0869	slr0513	slr0343	slr0922	sll1398	slr0357	sll1579	slr1022	sll1605	slr0823
slr0513	slr1598	slr0343	slr0838	sll1362	sll1398	sll1579	sll1883	sll0990	slr0823
sll0868	slr0513	sll1823	slr0343	sll1398	slr1550	sll1579	slr1133	sll1633	slr0823
slr0513	slr0994	sll1056	slr0343	sll1074	sll1398	sll1579	slr0444	slr0018	slr0823
sll0379	slr0513	slr0343	slr0520	sll1398	slr0649	sll1579	slr2130	sll0567	slr0823
sll1676	slr0513	sll0144	slr0343	sll0495	sll1398	sll1579	sll1669	slr0823	slr0884
sll0927	slr0513	sll0467	slr0343	sll1398	sll1425	sll1579	slr0585	sll1342	slr0823
sll0996	slr0513	slr0343	slr0711	sll0502	sll1398	sll1579	slr2075	slr0823	slr1349
sll0844	slr0513	sll0754	slr0343	sll1398	slr1703	sll1579	slr2076	sll0593	slr0823
sll1615	slr0513	sll0569	slr0343	sll0078	sll1398	sll0416	sll1579	sll0220	slr0823
sll0202	slr0513	sll1277	slr0343	sll1398	slr0557	sll1579	slr0156	sll0899	slr0823
slr0513	slr0902	sll1110	slr0343	sll1398	slr1884	sll1579	slr1641	slr0823	slr1756
sll1144	slr0513	sll1865	slr0343	sll1398	slr1031	sll1579	slr0542	sll1085	slr0823
slr0017	slr0513	slr0343	slr1228	sll1398	slr1793	sll0534	sll1579	sll1931	slr0823
slr0513	slr1424	slr0343	slr0808	sll1398	slr0633	sll1579	slr0165	sll0083	slr0823
slr0513	slr1423	slr0082	slr0343	sll1398	slr1278	sll0030	sll1579	slr0823	slr1755
sll2010	slr0513	slr0343	slr0742	sll1398	slr0783	sll1579	slr0847	sll0057	slr0823
slr0513	slr0528	sll0098	slr0343	sll1198	sll1398	sll1579	slr0553	sll0017	slr0823
slr0513	slr1351	slr0343	slr1646	sll0204	sll1398	sll1579	slr0812	sll1185	slr0823
sll0622	slr0513	slr0343	slr1130	sll1398	sll1750	sll1579	slr1347	slr0823	slr0839
sll0631	slr0513	slr0080	slr0343	sll0420	sll1398	sll1579	slr1348	sll1876	slr0823
slr0513	slr0787	slr0343	slr0551	sll1398	slr1256	sll1579	slr1791	sll0900	slr0823
sll1852	slr0513	sll0145	slr0343	sll1398	sll1639	sll0594	sll1579	slr0608	slr0823
slr0513	slr1090	sll0708	slr0343	sll1398	slr1899	sll1579	slr0550	slr0652	slr0823
sll0902	slr0513	slr0343	slr0679	sll0643	sll1398	sll1058	sll1579	slr0084	slr0823
slr0513	slr0661	slr0343	slr0722	sll1398	slr1844	sll1579	slr1665	sll1893	slr0823
slr0513	slr0526	slr0072	slr0343	sll0459	sll1398	sll1579	slr0536	slr0500	slr0823
sll1249	slr0513	sll1547	slr0343	sll0865	sll1398	sll1579	slr1874	slr0823	slr1560
sll0892	slr0513	sll1234	slr0343	sll1398	sll1459	sll1579	sll1776	sll1988	slr0823
sll0660	slr0513	sll0616	slr0343	sll0905	sll1398	sll0848	sll1579	slr0823	slr1289
slr0513	slr1779	slr0343	slr2073	sll0402	slr1181	sll0897	sll1579	sll1556	slr0823
slr0394	slr0513	slr0343	slr1639	sll0728	slr1181	sll1579	sll1933	slr0823	ssl3441
slr0513	slr2047	sll1108	slr0343	sll0336	slr1181	sll1579	slr0965	slr0744	slr0823
slr0513	slr0741	sll0362	slr0343	sll1299	slr1181	sll1579	slr1463	slr0823	slr0974
slr0513	slr1510	slr0343	slr0958	slr1181	ssl2084	sll1098	sll1579	slr0823	slr1622
sll1043	slr0513	slr0343	slr1720	sll2001	slr1181	sll1579	slr0434	sll1196	slr0823
slr0400	slr0513	sll0179	slr0343	sll1430	slr1181	sll1261	sll1579	sll0745	slr0823
sll1415	slr0513	sll0454	slr0343	sll0573	slr1181	sll1099	sll1579	sll1815	slr0823
sll1909	slr0513	sll1553	slr0343	slr1181	slr1898	sll1579	slr0752	sll1059	slr0823
sll0373	slr0513	slr0343	slr0638	sll0080	slr1181	sll1579	slr0952	slr0823	slr1123
slr0513	slr2035	slr0220	slr0343	slr1022	slr1181	sll1579	slr1511	sll1760	slr0823
slr0513	slr2132	slr0343	slr0357	sll1883	slr1181	sll1579	sll1605	sll0469	slr0823
slr0513	slr0922	sll1362	slr0343	slr1133	slr1181	sll0990	sll1579	slr0823	slr1517
slr0513	slr0838	slr0343	slr1550	slr0444	slr1181	sll1579	sll1633	sll0869	slr0823
sll1823	slr0513	sll1074	slr0343	slr1181	slr2130	sll1579	slr0018	slr0823	slr1598
sll1056	slr0513	slr0343	slr0649	sll1669	slr1181	sll0567	sll1579	sll0868	slr0823
slr0513	slr0520	sll0495	slr0343	slr0585	slr1181	sll1579	slr0884	slr0823	slr0994
sll0144	slr0513	sll1425	slr0343	slr1181	slr2075	sll1342	sll1579	sll0379	slr0823

sll0467	slr0513	sll0502	slr0343	slr1181	slr2076	sll1579	slr1349	sll1676	slr0823
slr0513	slr0711	slr0343	slr1703	sll0416	slr1181	sll0593	sll1579	sll0927	slr0823
sll0754	slr0513	sll0078	slr0343	slr0156	slr1181	sll0220	sll1579	sll0996	slr0823
sll0569	slr0513	slr0343	slr0557	slr1181	slr1641	sll0899	sll1579	sll0844	slr0823
sll1277	slr0513	slr0343	slr1884	slr0542	slr1181	sll1579	slr1756	sll1615	slr0823
sll1110	slr0513	slr0343	slr1031	sll0534	slr1181	sll1085	sll1579	sll0202	slr0823
sll1865	slr0513	slr0343	slr1793	slr0165	slr1181	sll1579	sll1931	slr0823	slr0902
slr0513	slr1228	slr0343	slr0633	sll0030	slr1181	sll0083	sll1579	sll1144	slr0823
slr0513	slr0808	slr0343	slr1278	slr0847	slr1181	sll1579	slr1755	slr0017	slr0823
slr0082	slr0513	slr0343	slr0783	slr0553	slr1181	sll0057	sll1579	slr0823	slr1424
slr0513	slr0742	sll1198	slr0343	slr0812	slr1181	sll0017	sll1579	slr0823	slr1423
sll0098	slr0513	sll0204	slr0343	slr1181	slr1347	sll1185	sll1579	sll2010	slr0823
slr0513	slr1646	sll1750	slr0343	slr1181	slr1348	sll1579	slr0839	slr0528	slr0823
slr0513	slr1130	sll0420	slr0343	slr1181	slr1791	sll1579	sll1876	slr0823	slr1351
slr0080	slr0513	slr0343	slr1256	sll0594	slr1181	sll0900	sll1579	sll0622	slr0823
slr0513	slr0551	sll1639	slr0343	slr0550	slr1181	sll1579	slr0608	sll0631	slr0823
sll0145	slr0513	slr0343	slr1899	sll1058	slr1181	sll1579	slr0652	slr0787	slr0823
sll0708	slr0513	sll0643	slr0343	slr1181	slr1665	sll1579	slr0084	sll1852	slr0823
slr0513	slr0679	slr0343	slr1844	slr0536	slr1181	sll1579	sll1893	slr0823	slr1090
slr0513	slr0722	sll0459	slr0343	slr1181	slr1874	sll1579	slr0500	sll0902	slr0823
slr0072	slr0513	sll0865	slr0343	sll1776	slr1181	sll1579	slr1560	slr0661	slr0823
sll1547	slr0513	slr0343	slr0733	sll0848	slr1181	sll1579	sll1988	slr0526	slr0823
sll1234	slr0513	sll1459	slr0343	sll0897	slr1181	sll1579	slr1289	sll1249	slr0823
sll0616	slr0513	sll0905	slr0343	sll1933	slr1181	sll1556	sll1579	sll0892	slr0823
slr0513	slr2073	sll0402	smr0010	slr0965	slr1181	sll1579	ssl3441	sll0660	slr0823
slr0513	slr1639	sll0728	smr0010	slr1181	slr1463	sll1579	slr0744	slr0823	slr1779
sll1108	slr0513	sll0336	smr0010	sll1098	slr1181	sll1579	slr0974	slr0394	slr0823
sll0362	slr0513	sll1299	smr0010	slr0434	slr1181	sll1579	slr1622	slr0823	slr2047
slr0513	slr0958	smr0010	ssl2084	sll1261	slr1181	sll1196	sll1579	slr0741	slr0823
slr0513	slr1720	sll2001	smr0010	sll1099	slr1181	sll0745	sll1579	slr0823	slr1510
sll0179	slr0513	sll1430	smr0010	slr0752	slr1181	sll1579	sll1815	sll1043	slr0823
sll0454	slr0513	sll0573	smr0010	slr0952	slr1181	sll1059	sll1579	slr0400	slr0823
sll1553	slr0513	slr1898	smr0010	slr1181	slr1511	sll1579	slr1123	sll1415	slr0823
slr0513	slr0638	sll0080	smr0010	sll1605	slr1181	sll1579	sll1760	sll1909	slr0823
slr0220	slr0513	slr1022	smr0010	sll0990	slr1181	sll0469	sll1579	sll0373	slr0823
slr0357	slr0513	sll1883	smr0010	sll1633	slr1181	sll1579	slr1517	slr0823	slr2035
sll1362	slr0513	slr1133	smr0010	slr0018	slr1181	sll0869	sll1579	slr0823	slr2132
slr0513	slr1550	slr0444	smr0010	sll0567	slr1181	sll1579	slr1598	slr0823	slr0922
sll1074	slr0513	slr2130	smr0010	slr0884	slr1181	sll0868	sll1579	slr0823	slr0838
slr0513	slr0649	sll1669	smr0010	sll1342	slr1181	sll1579	slr0994	sll1823	slr0823
sll0495	slr0513	slr0585	smr0010	slr1181	slr1349	sll0379	sll1579	sll1056	slr0823
sll1425	slr0513	slr2075	smr0010	sll0593	slr1181	sll1579	sll1676	slr0520	slr0823
sll0502	slr0513	slr2076	smr0010	sll0220	slr1181	sll0927	sll1579	sll0144	slr0823
slr0513	slr1703	sll0416	smr0010	sll0899	slr1181	sll0996	sll1579	sll0467	slr0823
sll0078	slr0513	slr0156	smr0010	slr1181	slr1756	sll0844	sll1579	slr0711	slr0823
slr0513	slr0557	slr1641	smr0010	sll1085	slr1181	sll1579	sll1615	sll0754	slr0823
slr0513	slr1884	slr0542	smr0010	sll1931	slr1181	sll0202	sll1579	sll0569	slr0823
slr0513	slr1031	sll0534	smr0010	sll0083	slr1181	sll1579	slr0902	sll1277	slr0823
slr0513	slr1793	slr0165	smr0010	slr1181	slr1755	sll1144	sll1579	sll1110	slr0823
slr0513	slr0633	sll0030	smr0010	sll0057	slr1181	sll1579	slr0017	sll1865	slr0823
slr0513	slr1278	slr0847	smr0010	sll0017	slr1181	sll1579	slr1424	slr0823	slr1228

slr0513	slr0783	slr0553	smr0010	sll1185	slr1181	sll1579	slr1423	slr0808	slr0823
sll1198	slr0513	slr0812	smr0010	slr0839	slr1181	sll1579	sll2010	slr0082	slr0823
sll0204	slr0513	slr1347	smr0010	sll1876	slr1181	sll1579	slr0528	slr0742	slr0823
sll1750	slr0513	slr1348	smr0010	sll0900	slr1181	sll1579	slr1351	sll0098	slr0823
sll0420	slr0513	slr1791	smr0010	slr0608	slr1181	sll0622	sll1579	slr0823	slr1646
slr0513	slr1256	sll0594	smr0010	slr0652	slr1181	sll0631	sll1579	slr0823	slr1130
sll1639	slr0513	slr0550	smr0010	slr0084	slr1181	sll1579	slr0787	slr0080	slr0823
slr0513	slr1899	sll1058	smr0010	sll1893	slr1181	sll1579	sll1852	slr0551	slr0823
sll0643	slr0513	slr1665	smr0010	slr0500	slr1181	sll1579	slr1090	sll0145	slr0823
slr0513	slr1844	slr0536	smr0010	slr1181	slr1560	sll0902	sll1579	sll0708	slr0823
sll0459	slr0513	slr1874	smr0010	sll1988	slr1181	sll1579	slr0661	slr0679	slr0823
sll0865	slr0513	sll1776	smr0010	slr1181	slr1289	sll1579	slr0526	slr0722	slr0823
slr0513	slr0733	sll0848	smr0010	sll1556	slr1181	sll1249	sll1579	slr0072	slr0823
sll1459	slr0513	sll0897	smr0010	slr1181	ssl3441	sll0892	sll1579	sll1547	slr0823
sll0905	slr0513	sll1933	smr0010	slr0744	slr1181	sll0660	sll1579	sll1234	slr0823
sll0247	sll0402	slr0965	smr0010	slr0974	slr1181	sll1579	slr1779	sll0616	slr0823
sll0247	sll0728	slr1463	smr0010	slr1181	slr1622	sll1579	slr0394	slr0823	slr2073
sll0247	sll0336	sll1098	smr0010	sll1196	slr1181	sll1579	slr2047	slr0823	slr1639
sll0247	sll1299	slr0434	smr0010	sll0745	slr1181	sll1579	slr0741	sll1108	slr0823
sll0247	ssl2084	sll1261	smr0010	sll1815	slr1181	sll1579	slr1510	sll0362	slr0823
sll0247	sll2001	sll1099	smr0010	sll1059	slr1181	sll1043	sll1579	slr0823	slr0958
sll0247	sll1430	slr0752	smr0010	slr1123	slr1181	sll1579	slr0400	slr0823	slr1720
sll0247	sll0573	slr0952	smr0010	sll1760	slr1181	sll1415	sll1579	sll0179	slr0823
sll0247	slr1898	slr1511	smr0010	sll0469	slr1181	sll1579	sll1909	sll0454	slr0823
sll0080	sll0247	sll1605	smr0010	slr1181	slr1517	sll0373	sll1579	sll1553	slr0823
sll0247	slr1022	sll0990	smr0010	sll0869	slr1181	sll1579	slr2035	slr0638	slr0823
sll0247	sll1883	sll1633	smr0010	slr1181	slr1598	sll1579	slr2132	slr0220	slr0823
sll0247	slr1133	slr0018	smr0010	sll0868	slr1181	sll1579	slr0922	slr0357	slr0823
sll0247	slr0444	sll0567	smr0010	slr0994	slr1181	sll1579	slr0838	sll1362	slr0823
sll0247	slr2130	slr0884	smr0010	sll0379	slr1181	sll1579	sll1823	slr0823	slr1550
sll0247	sll1669	sll1342	smr0010	sll1676	slr1181	sll1056	sll1579	sll1074	slr0823
sll0247	slr0585	slr1349	smr0010	sll0927	slr1181	sll1579	slr0520	slr0649	slr0823
sll0247	slr2075	sll0593	smr0010	sll0996	slr1181	sll0144	sll1579	sll0495	slr0823
sll0247	slr2076	sll0220	smr0010	sll0844	slr1181	sll0467	sll1579	sll1425	slr0823
sll0247	sll0416	sll0899	smr0010	sll1615	slr1181	sll1579	slr0711	sll0502	slr0823
sll0247	slr0156	slr1756	smr0010	sll0202	slr1181	sll0754	sll1579	slr0823	slr1703
sll0247	slr1641	sll1085	smr0010	slr0902	slr1181	sll0569	sll1579	sll0078	slr0823
sll0247	slr0542	sll1931	smr0010	sll1144	slr1181	sll1277	sll1579	slr0557	slr0823
sll0247	sll0534	sll0083	smr0010	slr0017	slr1181	sll1110	sll1579	slr0823	slr1884
sll0247	slr0165	slr1755	smr0010	slr1181	slr1424	sll1579	sll1865	slr0823	slr1031
sll0030	sll0247	sll0057	smr0010	slr1181	slr1423	sll1579	slr1228	slr0823	slr1793
sll0247	slr0847	sll0017	smr0010	sll2010	slr1181	sll1579	slr0808	slr0633	slr0823
sll0247	slr0553	sll1185	smr0010	slr0528	slr1181	sll1579	slr0082	slr0823	slr1278
sll0247	slr0812	slr0839	smr0010	slr1181	slr1351	sll1579	slr0742	slr0783	slr0823
sll0247	slr1347	sll1876	smr0010	sll0622	slr1181	sll0098	sll1579	sll1198	slr0823
sll0247	slr1348	sll0900	smr0010	sll0631	slr1181	sll1579	slr1646	sll0204	slr0823
sll0247	slr1791	slr0608	smr0010	slr0787	slr1181	sll1579	slr1130	sll1750	slr0823
sll0247	sll0594	slr0652	smr0010	sll1852	slr1181	sll1579	slr0080	sll0420	slr0823
sll0247	slr0550	slr0084	smr0010	slr1090	slr1181	sll1579	slr0551	slr0823	slr1256
sll0247	sll1058	sll1893	smr0010	sll0902	slr1181	sll0145	sll1579	sll1639	slr0823
sll0247	slr1665	slr0500	smr0010	slr0661	slr1181	sll0708	sll1579	slr0823	slr1899

sll0247	slr0536	slr1560	smr0010	slr0526	slr1181	sll1579	slr0679	sll0643	slr0823
sll0247	slr1874	sll1988	smr0010	sll1249	slr1181	sll1579	slr0722	slr0823	slr1844
sll0247	sll1776	slr1289	smr0010	sll0892	slr1181	sll1579	slr0072	sll0459	slr0823
sll0247	sll0848	sll1556	smr0010	sll0660	slr1181	sll1547	sll1579	sll0865	slr0823
sll0247	sll0897	smr0010	ssl3441	slr1181	slr1779	sll1234	sll1579	slr0733	slr0823
sll0247	sll1933	slr0744	smr0010	slr0394	slr1181	sll0616	sll1579	sll1459	slr0823
sll0247	slr0965	slr0974	smr0010	slr1181	slr2047	sll1579	slr2073	sll0905	slr0823
sll0247	slr1463	slr1622	smr0010	slr0741	slr1181	sll1579	slr1639	sll0226	sll0402
sll0247	sll1098	sll1196	smr0010	slr1181	slr1510	sll1108	sll1579	sll0226	sll0728
sll0247	slr0434	sll0745	smr0010	sll1043	slr1181	sll0362	sll1579	sll0226	sll0336
sll0247	sll1261	sll1815	smr0010	slr0400	slr1181	sll1579	slr0958	sll0226	sll1299
sll0247	sll1099	sll1059	smr0010	sll1415	slr1181	sll1579	slr1720	sll0226	ssl2084
sll0247	slr0752	slr1123	smr0010	sll1909	slr1181	sll0179	sll1579	sll0226	sll2001
sll0247	slr0952	sll1760	smr0010	sll0373	slr1181	sll0454	sll1579	sll0226	sll1430
sll0247	slr1511	sll0469	smr0010	slr1181	slr2035	sll1553	sll1579	sll0226	sll0573
sll0247	sll1605	slr1517	smr0010	slr1181	slr2132	sll1579	slr0638	sll0226	slr1898
sll0247	sll0990	sll0869	smr0010	slr0922	slr1181	sll1579	slr0220	sll0080	sll0226
sll0247	sll1633	slr1598	smr0010	slr0838	slr1181	sll1579	slr0357	sll0226	slr1022
sll0247	slr0018	sll0868	smr0010	sll1823	slr1181	sll1362	sll1579	sll0226	sll1883
sll0247	sll0567	slr0994	smr0010	sll1056	slr1181	sll1579	slr1550	sll0226	slr1133
sll0247	slr0884	sll0379	smr0010	slr0520	slr1181	sll1074	sll1579	sll0226	slr0444
sll0247	sll1342	sll1676	smr0010	sll0144	slr1181	sll1579	slr0649	sll0226	slr2130
sll0247	slr1349	sll0927	smr0010	sll0467	slr1181	sll0495	sll1579	sll0226	sll1669
sll0247	sll0593	sll0996	smr0010	slr0711	slr1181	sll1425	sll1579	sll0226	slr0585
sll0220	sll0247	sll0844	smr0010	sll0754	slr1181	sll0502	sll1579	sll0226	slr2075
sll0247	sll0899	sll1615	smr0010	sll0569	slr1181	sll1579	slr1703	sll0226	slr2076
sll0247	slr1756	sll0202	smr0010	sll1277	slr1181	sll0078	sll1579	sll0226	sll0416
sll0247	sll1085	slr0902	smr0010	sll1110	slr1181	sll1579	slr0557	sll0226	slr0156
sll0247	sll1931	sll1144	smr0010	sll1865	slr1181	sll1579	slr1884	sll0226	slr1641
sll0083	sll0247	slr0017	smr0010	slr1181	slr1228	sll1579	slr1031	sll0226	slr0542
sll0247	slr1755	slr1424	smr0010	slr0808	slr1181	sll1579	slr1793	sll0226	sll0534
sll0057	sll0247	slr1423	smr0010	slr0082	slr1181	sll1579	slr0633	sll0226	slr0165
sll0017	sll0247	sll2010	smr0010	slr0742	slr1181	sll1579	slr1278	sll0030	sll0226
sll0247	sll1185	slr0528	smr0010	sll0098	slr1181	sll1579	slr0783	sll0226	slr0847
sll0247	slr0839	slr1351	smr0010	slr1181	slr1646	sll1198	sll1579	sll0226	slr0553
sll0247	sll1876	sll0622	smr0010	slr1130	slr1181	sll0204	sll1579	sll0226	slr0812
sll0247	sll0900	sll0631	smr0010	slr0080	slr1181	sll1579	sll1750	sll0226	slr1347
sll0247	slr0608	slr0787	smr0010	slr0551	slr1181	sll0420	sll1579	sll0226	slr1348
sll0247	slr0652	sll1852	smr0010	sll0145	slr1181	sll1579	slr1256	sll0226	slr1791
sll0247	slr0084	slr1090	smr0010	sll0708	slr1181	sll1579	sll1639	sll0226	sll0594
sll0247	sll1893	sll0902	smr0010	slr0679	slr1181	sll1579	slr1899	sll0226	slr0550
sll0247	slr0500	slr0661	smr0010	slr0722	slr1181	sll0643	sll1579	sll0226	sll1058
sll0247	slr1560	slr0526	smr0010	slr0072	slr1181	sll1579	slr1844	sll0226	slr1665
sll0247	sll1988	sll1249	smr0010	sll1547	slr1181	sll0459	sll1579	sll0226	slr0536
sll0247	slr1289	sll0892	smr0010	sll1234	slr1181	sll0865	sll1579	sll0226	slr1874
sll0247	sll1556	sll0660	smr0010	sll0616	slr1181	sll1579	slr0733	sll0226	sll1776
sll0247	ssl3441	slr1779	smr0010	slr1181	slr2073	sll1459	sll1579	sll0226	sll0848
sll0247	slr0744	slr0394	smr0010	slr1181	slr1639	sll0905	sll1579	sll0226	sll0897
sll0247	slr0974	slr2047	smr0010	sll1108	slr1181	sll0402	ssl3093	sll0226	sll1933
sll0247	slr1622	slr0741	smr0010	sll0362	slr1181	sll0728	ssl3093	sll0226	slr0965
sll0247	sll1196	slr1510	smr0010	slr0958	slr1181	sll0336	ssl3093	sll0226	slr1463

sll0247	sll0745	sll1043	smr0010	slr1181	slr1720	sll1299	ssl3093	sll0226	sll1098
sll0247	sll1815	slr0400	smr0010	sll0179	slr1181	ssl2084	ssl3093	sll0226	slr0434
sll0247	sll1059	sll1415	smr0010	sll0454	slr1181	sll2001	ssl3093	sll0226	sll1261
sll0247	slr1123	sll1909	smr0010	sll1553	slr1181	sll1430	ssl3093	sll0226	sll1099
sll0247	sll1760	sll0373	smr0010	slr0638	slr1181	sll0573	ssl3093	sll0226	slr0752
sll0247	sll0469	slr2035	smr0010	slr0220	slr1181	slr1898	ssl3093	sll0226	slr0952
sll0247	slr1517	slr2132	smr0010	slr0357	slr1181	sll0080	ssl3093	sll0226	slr1511
sll0247	sll0869	slr0922	smr0010	sll1362	slr1181	slr1022	ssl3093	sll0226	sll1605
sll0247	slr1598	slr0838	smr0010	slr1181	slr1550	sll1883	ssl3093	sll0226	sll0990
sll0247	sll0868	sll1823	smr0010	sll1074	slr1181	slr1133	ssl3093	sll0226	sll1633
sll0247	slr0994	sll1056	smr0010	slr0649	slr1181	slr0444	ssl3093	sll0226	slr0018
sll0247	sll0379	slr0520	smr0010	sll0495	slr1181	slr2130	ssl3093	sll0226	sll0567
sll0247	sll1676	sll0144	smr0010	sll1425	slr1181	sll1669	ssl3093	sll0226	slr0884
sll0247	sll0927	sll0467	smr0010	sll0502	slr1181	slr0585	ssl3093	sll0226	sll1342
sll0247	sll0996	slr0711	smr0010	slr1181	slr1703	slr2075	ssl3093	sll0226	slr1349
sll0247	sll0844	sll0754	smr0010	sll0078	slr1181	slr2076	ssl3093	sll0226	sll0593
sll0247	sll1615	sll0569	smr0010	slr0557	slr1181	sll0416	ssl3093	sll0220	sll0226
sll0202	sll0247	sll1277	smr0010	slr1181	slr1884	slr0156	ssl3093	sll0226	sll0899
sll0247	slr0902	sll1110	smr0010	slr1031	slr1181	slr1641	ssl3093	sll0226	slr1756
sll0247	sll1144	sll1865	smr0010	slr1181	slr1793	slr0542	ssl3093	sll0226	sll1085
sll0247	slr0017	slr1228	smr0010	slr0633	slr1181	sll0534	ssl3093	sll0226	sll1931
sll0247	slr1424	slr0808	smr0010	slr1181	slr1278	slr0165	ssl3093	sll0083	sll0226
sll0247	slr1423	slr0082	smr0010	slr0783	slr1181	sll0030	ssl3093	sll0226	slr1755
sll0247	sll2010	slr0742	smr0010	sll1198	slr1181	slr0847	ssl3093	sll0057	sll0226
sll0247	slr0528	sll0098	smr0010	sll0204	slr1181	slr0553	ssl3093	sll0017	sll0226
sll0247	slr1351	slr1646	smr0010	sll1750	slr1181	slr0812	ssl3093	sll0226	sll1185
sll0247	sll0622	slr1130	smr0010	sll0420	slr1181	slr1347	ssl3093	sll0226	slr0839
sll0247	sll0631	slr0080	smr0010	slr1181	slr1256	slr1348	ssl3093	sll0226	sll1876
sll0247	slr0787	slr0551	smr0010	sll1639	slr1181	slr1791	ssl3093	sll0226	sll0900
sll0247	sll1852	sll0145	smr0010	slr1181	slr1899	sll0594	ssl3093	sll0226	slr0608
sll0247	slr1090	sll0708	smr0010	sll0643	slr1181	slr0550	ssl3093	sll0226	slr0652
sll0247	sll0902	slr0679	smr0010	slr1181	slr1844	sll1058	ssl3093	sll0226	slr0084
sll0247	slr0661	slr0722	smr0010	sll0459	slr1181	slr1665	ssl3093	sll0226	sll1893
sll0247	slr0526	slr0072	smr0010	sll0865	slr1181	slr0536	ssl3093	sll0226	slr0500
sll0247	sll1249	sll1547	smr0010	slr0733	slr1181	slr1874	ssl3093	sll0226	slr1560
sll0247	sll0892	sll1234	smr0010	sll1459	slr1181	sll1776	ssl3093	sll0226	sll1988
sll0247	sll0660	sll0616	smr0010	sll0905	slr1181	sll0848	ssl3093	sll0226	slr1289
sll0247	slr1779	slr2073	smr0010	sll0402	slr0906	sll0897	ssl3093	sll0226	sll1556
sll0247	slr0394	slr1639	smr0010	sll0728	slr0906	sll1933	ssl3093	sll0226	ssl3441
sll0247	slr2047	sll1108	smr0010	sll0336	slr0906	slr0965	ssl3093	sll0226	slr0744
sll0247	slr0741	sll0362	smr0010	sll1299	slr0906	slr1463	ssl3093	sll0226	slr0974
sll0247	slr1510	slr0958	smr0010	slr0906	ssl2084	sll1098	ssl3093	sll0226	slr1622
sll0247	sll1043	slr1720	smr0010	sll2001	slr0906	slr0434	ssl3093	sll0226	sll1196
sll0247	slr0400	sll0179	smr0010	sll1430	slr0906	sll1261	ssl3093	sll0226	sll0745
sll0247	sll1415	sll0454	smr0010	sll0573	slr0906	sll1099	ssl3093	sll0226	sll1815
sll0247	sll1909	sll1553	smr0010	slr0906	slr1898	slr0752	ssl3093	sll0226	sll1059
sll0247	sll0373	slr0638	smr0010	sll0080	slr0906	slr0952	ssl3093	sll0226	slr1123
sll0247	slr2035	slr0220	smr0010	slr0906	slr1022	slr1511	ssl3093	sll0226	sll1760
sll0247	slr2132	slr0357	smr0010	sll1883	slr0906	sll1605	ssl3093	sll0226	sll0469
sll0247	slr0922	sll1362	smr0010	slr0906	slr1133	sll0990	ssl3093	sll0226	slr1517
sll0247	slr0838	slr1550	smr0010	slr0444	slr0906	sll1633	ssl3093	sll0226	sll0869

sll0247	sll1823	sll1074	smr0010	slr0906	slr2130	slr0018	ssl3093	sll0226	slr1598
sll0247	sll1056	slr0649	smr0010	sll1669	slr0906	sll0567	ssl3093	sll0226	sll0868
sll0247	slr0520	sll0495	smr0010	slr0585	slr0906	slr0884	ssl3093	sll0226	slr0994
sll0144	sll0247	sll1425	smr0010	slr0906	slr2075	sll1342	ssl3093	sll0226	sll0379
sll0247	sll0467	sll0502	smr0010	slr0906	slr2076	slr1349	ssl3093	sll0226	sll1676
sll0247	slr0711	slr1703	smr0010	sll0416	slr0906	sll0593	ssl3093	sll0226	sll0927
sll0247	sll0754	sll0078	smr0010	slr0156	slr0906	sll0220	ssl3093	sll0226	sll0996
sll0247	sll0569	slr0557	smr0010	slr0906	slr1641	sll0899	ssl3093	sll0226	sll0844
sll0247	sll1277	slr1884	smr0010	slr0542	slr0906	slr1756	ssl3093	sll0226	sll1615
sll0247	sll1110	slr1031	smr0010	sll0534	slr0906	sll1085	ssl3093	sll0202	sll0226
sll0247	sll1865	slr1793	smr0010	slr0165	slr0906	sll1931	ssl3093	sll0226	slr0902
sll0247	slr1228	slr0633	smr0010	sll0030	slr0906	sll0083	ssl3093	sll0226	sll1144
sll0247	slr0808	slr1278	smr0010	slr0847	slr0906	slr1755	ssl3093	sll0226	slr0017
sll0247	slr0082	slr0783	smr0010	slr0553	slr0906	sll0057	ssl3093	sll0226	slr1424
sll0247	slr0742	sll1198	smr0010	slr0812	slr0906	sll0017	ssl3093	sll0226	slr1423
sll0098	sll0247	sll0204	smr0010	slr0906	slr1347	sll1185	ssl3093	sll0226	sll2010
sll0247	slr1646	sll1750	smr0010	slr0906	slr1348	slr0839	ssl3093	sll0226	slr0528
sll0247	slr1130	sll0420	smr0010	slr0906	slr1791	sll1876	ssl3093	sll0226	slr1351
sll0247	slr0080	slr1256	smr0010	sll0594	slr0906	sll0900	ssl3093	sll0226	sll0622
sll0247	slr0551	sll1639	smr0010	slr0550	slr0906	slr0608	ssl3093	sll0226	sll0631
sll0145	sll0247	slr1899	smr0010	sll1058	slr0906	slr0652	ssl3093	sll0226	slr0787
sll0247	sll0708	sll0643	smr0010	slr0906	slr1665	slr0084	ssl3093	sll0226	sll1852
sll0247	slr0679	slr1844	smr0010	slr0536	slr0906	sll1893	ssl3093	sll0226	slr1090
sll0247	slr0722	sll0459	smr0010	slr0906	slr1874	slr0500	ssl3093	sll0226	sll0902
sll0247	slr0072	sll0865	smr0010	sll1776	slr0906	slr1560	ssl3093	sll0226	slr0661
sll0247	sll1547	slr0733	smr0010	sll0848	slr0906	sll1988	ssl3093	sll0226	slr0526
sll0247	sll1234	sll1459	smr0010	sll0897	slr0906	slr1289	ssl3093	sll0226	sll1249
sll0247	sll0616	sll0905	smr0010	sll1933	slr0906	sll1556	ssl3093	sll0226	sll0892
sll0247	slr2073	sll0402	smr0003	slr0906	slr0965	ssl3093	ssl3441	sll0226	sll0660
sll0247	slr1639	sll0728	smr0003	slr0906	slr1463	slr0744	ssl3093	sll0226	slr1779
sll0247	sll1108	sll0336	smr0003	sll1098	slr0906	slr0974	ssl3093	sll0226	slr0394
sll0247	sll0362	sll1299	smr0003	slr0434	slr0906	slr1622	ssl3093	sll0226	slr2047
sll0247	slr0958	smr0003	ssl2084	sll1261	slr0906	sll1196	ssl3093	sll0226	slr0741
sll0247	slr1720	sll2001	smr0003	sll1099	slr0906	sll0745	ssl3093	sll0226	slr1510
sll0179	sll0247	sll1430	smr0003	slr0752	slr0906	sll1815	ssl3093	sll0226	sll1043
sll0247	sll0454	sll0573	smr0003	slr0906	slr0952	sll1059	ssl3093	sll0226	slr0400
sll0247	sll1553	slr1898	smr0003	slr0906	slr1511	slr1123	ssl3093	sll0226	sll1415
sll0247	slr0638	sll0080	smr0003	sll1605	slr0906	sll1760	ssl3093	sll0226	sll1909
sll0247	slr0220	slr1022	smr0003	sll0990	slr0906	sll0469	ssl3093	sll0226	sll0373
sll0247	slr0357	sll1883	smr0003	sll1633	slr0906	slr1517	ssl3093	sll0226	slr2035
sll0247	sll1362	slr1133	smr0003	slr0018	slr0906	sll0869	ssl3093	sll0226	slr2132
sll0247	slr1550	slr0444	smr0003	sll0567	slr0906	slr1598	ssl3093	sll0226	slr0922
sll0247	sll1074	slr2130	smr0003	slr0884	slr0906	sll0868	ssl3093	sll0226	slr0838
sll0247	slr0649	sll1669	smr0003	sll1342	slr0906	slr0994	ssl3093	sll0226	sll1823
sll0247	sll0495	slr0585	smr0003	slr0906	slr1349	sll0379	ssl3093	sll0226	sll1056
sll0247	sll1425	slr2075	smr0003	sll0593	slr0906	sll1676	ssl3093	sll0226	slr0520
sll0247	sll0502	slr2076	smr0003	sll0220	slr0906	sll0927	ssl3093	sll0144	sll0226
sll0247	slr1703	sll0416	smr0003	sll0899	slr0906	sll0996	ssl3093	sll0226	sll0467
sll0078	sll0247	slr0156	smr0003	slr0906	slr1756	sll0844	ssl3093	sll0226	slr0711
sll0247	slr0557	slr1641	smr0003	sll1085	slr0906	sll1615	ssl3093	sll0226	sll0754
sll0247	slr1884	slr0542	smr0003	sll1931	slr0906	sll0202	ssl3093	sll0226	sll0569

sll0247	slr1031	sll0534	smr0003	sll0083	slr0906	slr0902	ssl3093	sll0226	sll1277
sll0247	slr1793	slr0165	smr0003	slr0906	slr1755	sll1144	ssl3093	sll0226	sll1110
sll0247	slr0633	sll0030	smr0003	sll0057	slr0906	slr0017	ssl3093	sll0226	sll1865
sll0247	slr1278	slr0847	smr0003	sll0017	slr0906	slr1424	ssl3093	sll0226	slr1228
sll0247	slr0783	slr0553	smr0003	sll1185	slr0906	slr1423	ssl3093	sll0226	slr0808
sll0247	sll1198	slr0812	smr0003	slr0839	slr0906	sll2010	ssl3093	sll0226	slr0082
sll0204	sll0247	slr1347	smr0003	sll1876	slr0906	slr0528	ssl3093	sll0226	slr0742
sll0247	sll1750	slr1348	smr0003	sll0900	slr0906	slr1351	ssl3093	sll0098	sll0226
sll0247	sll0420	slr1791	smr0003	slr0608	slr0906	sll0622	ssl3093	sll0226	slr1646
sll0247	slr1256	sll0594	smr0003	slr0652	slr0906	sll0631	ssl3093	sll0226	slr1130
sll0247	sll1639	slr0550	smr0003	slr0084	slr0906	slr0787	ssl3093	sll0226	slr0080
sll0247	slr1899	sll1058	smr0003	sll1893	slr0906	sll1852	ssl3093	sll0226	slr0551
sll0247	sll0643	slr1665	smr0003	slr0500	slr0906	slr1090	ssl3093	sll0145	sll0226
sll0247	slr1844	slr0536	smr0003	slr0906	slr1560	sll0902	ssl3093	sll0226	sll0708
sll0247	sll0459	slr1874	smr0003	sll1988	slr0906	slr0661	ssl3093	sll0226	slr0679
sll0247	sll0865	sll1776	smr0003	slr0906	slr1289	slr0526	ssl3093	sll0226	slr0722
sll0247	slr0733	sll0848	smr0003	sll1556	slr0906	sll1249	ssl3093	sll0226	slr0072
sll0247	sll1459	sll0897	smr0003	slr0906	ssl3441	sll0892	ssl3093	sll0226	sll1547
sll0247	sll0905	sll1933	smr0003	slr0744	slr0906	sll0660	ssl3093	sll0226	sll1234
sll0402	slr0261	slr0965	smr0003	slr0906	slr0974	slr1779	ssl3093	sll0226	sll0616
sll0728	slr0261	slr1463	smr0003	slr0906	slr1622	slr0394	ssl3093	sll0226	slr2073
sll0336	slr0261	sll1098	smr0003	sll1196	slr0906	slr2047	ssl3093	sll0226	slr1639
sll1299	slr0261	slr0434	smr0003	sll0745	slr0906	slr0741	ssl3093	sll0226	sll1108
slr0261	ssl2084	sll1261	smr0003	sll1815	slr0906	slr1510	ssl3093	sll0226	sll0362
sll2001	slr0261	sll1099	smr0003	sll1059	slr0906	sll1043	ssl3093	sll0226	slr0958
sll1430	slr0261	slr0752	smr0003	slr0906	slr1123	slr0400	ssl3093	sll0226	slr1720
sll0573	slr0261	slr0952	smr0003	sll1760	slr0906	sll1415	ssl3093	sll0179	sll0226
slr0261	slr1898	slr1511	smr0003	sll0469	slr0906	sll1909	ssl3093	sll0226	sll0454
sll0080	slr0261	sll1605	smr0003	slr0906	slr1517	sll0373	ssl3093	sll0226	sll1553
slr0261	slr1022	sll0990	smr0003	sll0869	slr0906	slr2035	ssl3093	sll0226	slr0638
sll1883	slr0261	sll1633	smr0003	slr0906	slr1598	slr2132	ssl3093	sll0226	slr0220
slr0261	slr1133	slr0018	smr0003	sll0868	slr0906	slr0922	ssl3093	sll0226	slr0357
slr0261	slr0444	sll0567	smr0003	slr0906	slr0994	slr0838	ssl3093	sll0226	sll1362
slr0261	slr2130	slr0884	smr0003	sll0379	slr0906	sll1823	ssl3093	sll0226	slr1550
sll1669	slr0261	sll1342	smr0003	sll1676	slr0906	sll1056	ssl3093	sll0226	sll1074
slr0261	slr0585	slr1349	smr0003	sll0927	slr0906	slr0520	ssl3093	sll0226	slr0649
slr0261	slr2075	sll0593	smr0003	sll0996	slr0906	sll0144	ssl3093	sll0226	sll0495
slr0261	slr2076	sll0220	smr0003	sll0844	slr0906	sll0467	ssl3093	sll0226	sll1425
sll0416	slr0261	sll0899	smr0003	sll1615	slr0906	slr0711	ssl3093	sll0226	sll0502
slr0156	slr0261	slr1756	smr0003	sll0202	slr0906	sll0754	ssl3093	sll0226	slr1703
slr0261	slr1641	sll1085	smr0003	slr0902	slr0906	sll0569	ssl3093	sll0078	sll0226
slr0261	slr0542	sll1931	smr0003	sll1144	slr0906	sll1277	ssl3093	sll0226	slr0557
sll0534	slr0261	sll0083	smr0003	slr0017	slr0906	sll1110	ssl3093	sll0226	slr1884
slr0165	slr0261	slr1755	smr0003	slr0906	slr1424	sll1865	ssl3093	sll0226	slr1031
sll0030	slr0261	sll0057	smr0003	slr0906	slr1423	slr1228	ssl3093	sll0226	slr1793
slr0261	slr0847	sll0017	smr0003	sll2010	slr0906	slr0808	ssl3093	sll0226	slr0633
slr0261	slr0553	sll1185	smr0003	slr0528	slr0906	slr0082	ssl3093	sll0226	slr1278
slr0261	slr0812	slr0839	smr0003	slr0906	slr1351	slr0742	ssl3093	sll0226	slr0783
slr0261	slr1347	sll1876	smr0003	sll0622	slr0906	sll0098	ssl3093	sll0226	sll1198
slr0261	slr1348	sll0900	smr0003	sll0631	slr0906	slr1646	ssl3093	sll0204	sll0226
slr0261	slr1791	slr0608	smr0003	slr0787	slr0906	slr1130	ssl3093	sll0226	sll1750

sll0594	slr0261	slr0652	smr0003	sll1852	slr0906	slr0080	ssl3093	sll0226	sll0420
slr0261	slr0550	slr0084	smr0003	slr0906	slr1090	slr0551	ssl3093	sll0226	slr1256
sll1058	slr0261	sll1893	smr0003	sll0902	slr0906	sll0145	ssl3093	sll0226	sll1639
slr0261	slr1665	slr0500	smr0003	slr0661	slr0906	sll0708	ssl3093	sll0226	slr1899
slr0261	slr0536	slr1560	smr0003	slr0526	slr0906	slr0679	ssl3093	sll0226	sll0643
slr0261	slr1874	sll1988	smr0003	sll1249	slr0906	slr0722	ssl3093	sll0226	slr1844
sll1776	slr0261	slr1289	smr0003	sll0892	slr0906	slr0072	ssl3093	sll0226	sll0459
sll0848	slr0261	sll1556	smr0003	sll0660	slr0906	sll1547	ssl3093	sll0226	sll0865
sll0897	slr0261	smr0003	ssl3441	slr0906	slr1779	sll1234	ssl3093	sll0226	slr0733
sll1933	slr0261	slr0744	smr0003	slr0394	slr0906	sll0616	ssl3093	sll0226	sll1459
slr0261	slr0965	slr0974	smr0003	slr0906	slr2047	slr2073	ssl3093	sll0226	sll0905
slr0261	slr1463	slr1622	smr0003	slr0741	slr0906	slr1639	ssl3093		

Supplemental Table S3. Predicted high confident PPIs.

P1	P2	P1	P2	P1	P2	P1	P2
sll0455	sll0456	ndhK2	sll8032	sll1406	sll1407	slr0847	slr0848
slr0907	slr0909	sll1251	ssl2471	slr5021	ssr5020	sll0927	sll0928
sll7063	sll7064	sll0720	sll1552	sll1429	ssl2789	slr1249	slr1250
sll0742	sll0743	sll7043	sll7044	sll1159	sll1160	slr1042	slr1043
slr2081	slr2082	sll0898	ssl1707	slr1884	ssr3189	sll0771	sll0772
sll1002	ssl1920	slr1416	slr1417	slr1339	slr1340	slr0103	slr0104
slr1857	slr1859	sll1203	sll1204	slr0293	slr0294	slr1970	ssr3341
slr0311	slr0312	sll0789	sll0790	sll0328	sll0329	slr1248	slr1249
sll5014	ssl5015	slr0901	slr0902	sll0092	ssl0172	slr1457	slr1459
slr0945	slr0946	sll1297	ssl2559	sll5089	sll5090	sll1672	sll1673
slr0502	slr0537	sll0043	sll0044	sll1786	sll1787	slr0492	slr0493
sll1031	sll1032	sll1121	sll1123	sll0447	sll0448	sll1630	sll1631
slr0298	slr0299	slr0879	slr0880	slr5118	slr5119	sll0753	ssl1417
slr7025	slr7026	sll0896	sll0897	slr7080	slr7081	slr0179	slr0180
slr0326	slr0327	sll0401	sll0402	sll1250	ssl2471	sll1005	sll1006
sll0574	sll0575	slr0236	slr0605	slr1939	slr1940	sll0237	sll0238
slr1187	slr1188	slr1434	ssr2067	slr1318	slr1319	sll0238	sll0240
slr0947	slr0948	sll2006	sll2007	slr1659	slr1660	slr1181	sml0001
sll1009	sll1009	slr0517	slr0518	slr2122	slr2123	sll0517	ssr1480
sll0477	sll0478	sll1087	sll1089	slr0704	smr0002	slr2035	ssr3452
sll1703	sll1704	slr0252	ssr0390	sll1608	sll1609	sll1255	sll1560
sll1760	sll1761	sll0505	sll0506	slr1999	ssr1765	slr1470	slr1471
sll1580	ssl3093	slr7083	ssr7084	sll0667	sll0668	sll1495	sll1496
ssl5099	ssl5100	slr6049	slr6050	sll1306	sll1307	slr1779	slr1780
slr1626	slr1627	sll1452	sll1453	sll1654	sll1655	slr0488	slr0489
sll0928	sll0930	slr1188	slr1189	slr1678	ssr2799	slr1020	ssr1698
sll0480	sll0481	slr6006	slr6007	slr0072	slr0848	slr1419	slr1420
sll0936	ssl1792	sll1181	sll1182	sll8006	ssl8005	sll0608	sll0609
slr1545	slr1546	sll0587	sll0588	sll0431	sll0651	sll0288	sll0289
slr1109	slr1110	sll0910	sll0911	sll0173	sll0174	sll0481	sll0482
slr1657	slr1658	slr1384	slr1385	slr0108	slr0109	slr2034	ssr3451
sll1099	sll1101	sll1520	sll1521	sll5097	ssl5098	sll1340	sll1341
slr1940	ssr3300	slr0878	slr0879	slr1065	slr1066	sll0147	sll0148
slr0313	slr0314	slr1512	slr1513	sll1343	sll1344	sll1119	sll1120
slr1815	slr1816	slr1846	ssr3122	slr1383	ssr2315	sll0289	ssl0546
slr0889	ssr1499	sll0381	sll0382	sll0760	sll0761	slr5016	slr5017
slr0701	slr0702	sll1003	sll1004	sll5130	ssl5129	sll0372	sll0373
sll1160	sll1161	sll1512	sll1513	slr0364	slr0366	slr1788	slr1789
sll8001	sll8002	slr0962	ssr1604	sll8033	sll8034	slr1161	slr1162
slr1488	slr1489	slr2017	slr2018	sll0031	sll0033	sll0415	sll0416
sll1094	ssl2153	sll0148	sll0149	slr6102	slr6103	sll2005	ssl3829
slr1864	slr1865	sll1389	ssl2717	slr2102	slr2103	slr0096	slr0498
slr2123	slr2124	sll0495	sll0496	sll0327	sll0328	sll0842	sll0843
sll1163	sll1164	sll5032	sll5033	slr1176	slr1177	sll1461	sll1462
slr6004	slr6005	slr1993	slr1994	sll0297	sll0298	sll5006	ssl5007
slr1078	slr1079	ssr7035	ssr7036	slr0527	slr0528	sll1633	ssl3177
sll0797	sll0798	slr1031	slr1032	sll0265	sll0266	sll0994	sll0995
sll0191	sll0416	slr5010	ssr5011	slr1418	slr1419	sll0370	sll0371

sll1695	sll1696
sll1624	sll1624
slr5105	ssr5106
sll0577	sll0578
sll0594	sll0595
slr0817	slr0818
sll1820	sll1821
slr1742	slr1743
slr0111	slr0112
sll0488	sll0740
slr0007	slr0008
slr1228	slr1229
slr0664	slr0665
sll6036	ssl6035
slr1022	slr1023
sll1533	sll1534
sll1509	sll1510
slr6037	slr6038
sll0925	sll0926
slr0711	slr0712
slr1734	slr1735
slr2096	slr2097
slr0480	slr0482
slr1930	slr1931
sll0012	sll0666
slr0473	slr0474
sll0067	sll0068
slr1519	slr1520
sll0860	sll1317
slr1024	slr1025
sll0867	sll0868
slr1212	slr1213
sll1536	slr0821
sll1228	slr1044
sll1004	sll1005
sll0981	sll0982
slr1851	ssr3129
sll8042	sll8043
slr1063	slr1225
sll1304	sll1785
slr0210	slr1044
sll0225	sll0226
sll1182	sll1316
sll8004	ssl8003
slr0388	slr0769
sll5104	ssl5103
sll0785	sll0786
sll1803	sll1804
ssr3409	ssr3410
sll1653	sll1654
slr2010	ssr3409

slr0710	slr0711
slr1178	slr1179
sll0827	sll0828
slr1570	slr1571
slr0482	slr0483
slr1023	slr1024
slr1610	slr1611
slr1214	slr1215
slr0603	slr0604
sll0163	sll0166
slr1508	slr1509
slr1676	slr1677
slr2015	slr2016
sll0058	sll0060
slr6015	slr6016
slr1379	slr1380
slr0162	slr0163
sgl0001	ssl1045
slr1728	slr1729
sll1314	sll1315
slr1535	slr1536
sll1028	sll1029
slr0263	slr0264
slr1227	slr1228
slr0089	slr0090
slr1518	slr1519
sll1799	sll1800
slr2095	slr2096
sll1941	slr0417
slr7059	slr7060
slr1550	slr1720
slr0815	slr0816
slr1559	slr1560
sll1900	slr1348
slr1106	slr1107
sll0071	sll0072
sll1471	slr2051
slr0925	slr1034
slr0049	slr0440
sll0317	sll1474
slr7082	slr7083
slr1415	slr1416
sll0166	sll0167
slr6101	slr6102
sgl0002	sml0004
sll5028	ssl5027
slr0272	slr0273
sll0240	sll0241
sll0666	sll0667
slr5115	slr5116
sll0020	sll0021

sll0450	sll0451
sll0005	sll0006
ssr3570	ssr3571
slr2001	slr2002
slr1942	ssr3304
sll1069	sll1070
slr0795	slr0796
slr6033	ssr6032
sll1613	sll1614
slr1393	slr1394
slr1338	slr1339
sll0536	sll0537
slr0658	slr0659
sll0803	sll0804
slr6084	ssr6085
sll0296	ssl0564
sll0065	sll0066
sll0665	sll1710
sll0658	sll0659
slr1603	slr1604
slr0161	slr0162
sll0728	sll0729
slr2083	slr2084
sll0737	sll0738
slr1722	slr1723
slr5018	ssr5019
slr1485	slr1488
slr1270	slr1271
slr0913	slr0914
sll5003	sll5004
slr1050	ssr1736
sll0042	sll0043
slr7096	slr7097
sll0615	sll0616
slr0435	slr0436
slr1799	slr1800
slr0580	slr0581
sll5097	ssl5096
sll1383	sll1384
sll0984	sll0985
slr0477	slr0479
sll1322	ssl2615
sll0677	sll1710
slr1560	slr1562
slr2125	slr2126
sll0436	ssl0832
slr1683	slr1684
sll1123	sll1124
slr0142	slr0408
slr2011	ssr3410
slr0612	slr0613

sll1450	sll1634
sll0360	sll0361
slr2111	slr2112
slr6100	ssr6099
slr0230	slr0231
slr6043	slr6044
slr0989	slr1951
slr0678	slr0679
slr0059	slr0060
sll0501	sll0502
sll1677	sll1678
sll0743	sll0744
sll1390	ssl2717
slr0954	ssr1600
slr0146	slr0147
slr1575	slr1576
sll0854	sll0855
slr1471	slr1472
slr1945	ssr3307
slr1668	ssr2786
sll6052	sll6053
sll1636	sll1900
sll1231	sll1232
sll1086	sll1087
slr0236	slr0744
sll5079	sll5080
slr1537	slr1538
slr1165	slr1166
sll1499	sll1502
slr0520	slr0521
sll1092	ssl2138
sll1899	ssl3580
slr0985	slr1610
sll7077	sll7078
slr0586	slr0587
sll1937	sll1938
sll1852	sll1853
slr1919	slr1920
sll1882	sll1883
slr0271	slr0272
slr1874	slr1875
slr0927	slr0929
sll1560	sll1780
sll1388	sll1389
slr7071	ssr7072
sll0891	sll0892
sll1109	sll1110
sll1571	sll1572
sll1356	sll1358
slr1044	slr1147
slr0357	slr0358

slr0361	slr0362
slr1736	slr1737
sll6054	sll6055
slr1452	ssr2439
sll0837	sll0838
ssr6026	ssr6027
slr1072	slr1073
sll1225	sll1226
sll0751	sll0752
slr0074	slr0075
sll0819	ssr2831
slr0605	slr0605
slr0909	slr0912
sll0681	sll0682
sll0634	sll0635
slr0976	slr0977
sll1853	sll1854
slr1515	ssr2551
slr1085	slr1087
slr0769	slr0770
sll1869	sll1870
slr1095	slr1096
sll1614	sll1615
sll7002	sll7003
sll0802	sll0803
slr0890	ssr1499
sll1315	ssl2595
slr1675	slr1676
slr1136	slr1137
ssl7038	ssl7039
sll5034	sll5035
sll1605	sll1606
sll1582	sll1583
slr1168	slr1169
slr1635	slr1636
slr0207	slr0208
sll5122	sll5123
sll0645	sll0646
slr0017	slr0018
slr0559	slr0924
slr0509	slr0510
sll0268	sll0269
slr0980	slr0981
slr0738	slr0739
sll1394	sll1395
slr7073	ssr7072
slr0342	slr0343
slr1260	slr1261
sll1832	sll1833
slr1887	slr1888
slr0552	slr0553

slr5116	ssr5117
slr0076	slr0077
slr1280	slr1281
sgl0001	slr0698
sll1228	sll1229
slr1365	slr1366
slr0299	slr0300
sll1806	sll1807
slr1794	ssr2998
slr0457	slr0458
sll0448	sll0449
slr1656	smr0004
slr0703	ssr1175
sll0502	sll0503
slr1895	slr1896
slr1046	ssl2823
sll1363	ssl2667
slr7011	slr7012
slr1173	ssr1951
sll0409	sll0410
slr1814	slr1815
sll1043	sll1045
sll1035	ssl2009
sll1733	sll1734
slr1062	slr1063
slr1142	slr1143
slr1169	slr1170
slr0609	slr0610
ssl1922	ssl1923
slr1493	slr1494
sll8032	sll8033
slr0815	ssr1386
slr0374	slr0376
slr6090	slr6091
slr1204	slr1205
slr1900	slr1901
sll1823	sll1824
sll0575	sll0576
slr0359	slr0360
sll0309	sll0310
sll1240	sll1241
slr1269	slr1270
sll1611	sll1612
slr2026	slr2027
sll1735	sll1736
sll0175	ssl0312
slr1702	slr1703
sll1834	sll1835
sll1595	sll1596
slr0355	slr0356
sll1638	sll1639

sll0644	sll0645
sll0690	sll0691
slr0742	slr0743
slr0505	slr0506
slr0819	ssr1391
sll0441	sll0442
sll1282	sll1348
slr1760	slr1761
ssr6078	ssr6079
slr1591	slr1592
slr0787	slr0788
slr1719	slr1720
slr0194	slr0195
slr0697	ssr1169
sll0677	sll1716
slr2041	slr2042
slr0236	slr0237
sll0167	sll0168
slr1331	slr1332
slr0592	slr0593
slr0434	slr0435
sll0552	sll0553
sll0405	sll0406
sll1106	sll1107
ssl2922	ssl2923
sll1830	sll1831
sll1077	sll1078
sll0521	sll0522
slr7024	slr7025
sll0485	sll0486
slr0208	ssr0330
sll1400	ssl2733
slr0757	slr0758
slr1443	slr1444
ssl3436	ssl3437
slr1034	slr1035
slr1246	ssr2078
slr0373	slr0374
sll0495	slr1550
sll0670	sll0671
sll6093	ssl6092
slr1658	slr1659
slr0772	slr0773
slr2118	slr2119
slr1267	slr1269
slr1467	slr1468
slr0511	ssr0871
sll0253	sll0254
sll7069	sll7070
ssl0787	ssl0788
sll8007	ssl8008

slr0869	slr0870
sll1958	sll1959
slr0670	slr1400
slr0724	slr0725
sll1503	sll1504
sll1485	sll1486
slr1564	slr1565
sll1905	sll1906
slr1073	slr1074
sll1104	ssl2162
sll0063a	sll0064
slr0317	slr0318
sll1750	sll1751
slr1863	slr1864
sll1474	sll1780
slr0581	slr0582
slr0926	slr0927
slr1068	slr1069
slr1562	slr1563
sll0938	ssl1792
slr1644	slr1645
ssr1765	ssr1766
slr2075	slr2076
slr1929	slr1930
sll1806	ssl3437
slr1347	slr1348
slr0937	slr0938
slr1926	slr1927
slr1546	slr1547
slr0322	slr1044
slr6094	slr6095
sll0765	sll0766
sll0517	sll0518
sll0931	sll0932
slr0020	slr0021
slr7095	slr7096
slr0822	slr0823
sll1017	sll1018
slr0709	slr0710
slr0049	ssr0102
slr2110	slr2111
sll0168	sll0169
slr0067	slr0110
sll0006	sll0007
slr1259	slr1260
slr6064	slr6065
sll0564	sll0565
sll1671	sll1672
sll0776	sll0777
slr1198	slr1199
slr0895	slr0896

slr1162	slr1163
sll0183	sll0183
sll1814	sll1815
ssl7045	ssl7046
sll5061	sll5062
slr0462	ssr0817
sll1666	sll1667
slr2033	slr2034
sll0141	sll0142
slr1730	slr1731
ssr1258	ssr1765
slr0774	slr0775
slr0722	slr0723
sll1425	sll1426
slr2130	slr2131
slr0213	slr0214
sll0359	sll0360
slr5111	slr5112
slr6008	slr6009
slr0549	slr0550
slr0091	slr0092
sll1069	ssl2084
slr1600	smr0005
sll2005	sll2006
sll1220	sll1221
slr1183	slr1184
sll1256	sll1257
slr0285	slr0286
sll0296	sll0297
slr1098	slr1099
slr0645	slr0646
slr1051	slr1052
slr0184	slr0185
slr0713	smr0003
sll1530	sll1531
slr0944	slr0945
ssr6002	ssr6003
slr0264	slr0265
sll0161	ssl0296
slr6038	slr6039
slr7081	slr7082
sll0250	ssl0461
ssl5095	ssl5096
slr1413	slr1414
slr1194	slr1195
sll1041	sll1318
slr6073	slr6074
slr0392	slr0393
sll2010	sll2011
sll0938	sll0939
slr1550	ssr2611

sll1743	sll1744
sll0780	sll0781
slr0888	slr0889
slr0730	slr0731
sll1910	sll1911
sll0683	sll0684
slr0599	slr0600
slr0574	slr0575
sll1411	ssl2749
slr1199	slr1200
slr2112	slr2113
sll0926	sll0927
slr1865	slr1866
sll0534	sll0535
slr1110	slr1772
sll2013	sll2014
slr2048	slr2049
sll0280	sll0281
sll1414	sll1415
sll1541	slr1648
sll1474	sll1560
sll0860	sll0861
slr0303	slr0304
sll0855	sll0856
slr1317	slr1318
sll1639	sll1640
slr2116	slr2117
slr1239	slr1240
sll0777	sll0778
slr6072	slr6073
sll0085	sll0086
sll0783	sll0784
slr8015	slr8016
sll1298	sll1299
sll0549	sll0550
sll5048	sll5049
slr1116	slr1117
slr0065	slr0066
slr0142	slr0143
sll0396	sll0397
slr0526	slr0527
sll1477	sll1479
sll0486	sll0487
slr1209	slr1210
slr7058	slr7059
sll1765	sll1766
sll1808	sll1809
sll1484	sll1485
slr0269	slr0270
ssr6019	ssr6020
sll1462	sll1463

slr0651	slr0652
smr0006	ssr3451
sll1060	sll1061
sll0602	sll0603
sll1716	sll1717
sll1142	sll1143
slr0467	slr0468
slr0150	slr0151
sll1694	sll1695
slr1623	slr1624
slr1075	slr1076
slr5054	slr5055
sll1599	sll1600
slr5022	slr5023
sll0241	sll0242
sll0621	sll0622
slr1520	slr1521
sll0593	sll0594
sll1812	sll1813
sll5026	ssl5027
sll8004	ssl8005
slr1790	slr1791
sll0802	ssl1498
sll0068	sll0069
ssr1398	ssr1399
sll1466	ssl2823
slr0088	slr0089
slr1200	slr1201
sll1289	sll1290
sll0038	sll0039
sll1898	sll1899
sll0985	sll0986
slr0502	slr0503
sll1710	sll1716
sll1787	sll1789
sll0711	sll0712
sll0779	sll0780
sll1101	sll1102
slr1115	slr1116
sll0503	sll0504
slr6080	ssr6079
slr0638	slr0639
slr2101	slr2102
slr1628	slr1629
slr0957	slr0958
slr5093	ssr5092
sll1908	sll1909
sll1957	sll1958
sll1742	sll1743
slr1185	slr1186
sll0394	sll0395

sll1818	sll1819
slr1705	slr1706
sll0178	sll0888
sll7062	sll7063
sll1817	sll1818
sll0520	sll0521
slr0479	slr0480
sll0355	sll0356
slr0783	slr0784
sll0384	sll0385
sll7086	sll7087
sll0816	sll0817
sgl0002	sll0222
slr0452	slr0453
slr1999	ssr1258
sll1570	sll1571
sll1432	sll1433
slr0093	slr0095
slr0903	ssr1528
slr1708	ssr2857
sll0766	ssl1426
sll0170	sll0171
sll1663	sll1664
sll0039	sll0040
sll1791	sll1792
sll1534	sll1535
slr1409	slr1410
sll1427	slr1204
sml0006	ssl3441
sll8042	ssl8041
slr1901	slr1902
slr0209	ssr0330
slr1407	slr1409
slr0553	slr0554
sll1804	sll1805
sll1655	sll1656
slr1262	slr1263
sll0336	sll0337
slr1652	slr1653
slr0981	slr0982
sll1070	sll1071
slr1106	slr1768
sll0227	sll0228
slr0825	ssr1398
sll1426	sll1427
sll1358	sll1359
slr1050	slr1051
slr1667	slr1668
sll1662	sll1663
slr0150	ssl0020
sll0012	sll1716

sll1296	ssl2559
slr1273	slr1274
slr5119	ssr5120
slr2006	slr2007
sll0915	sll0916
slr0835	slr0836
sll1400	sll1401
slr1149	slr1150
sll0382	sll0383
sll1724	sll1725
sll1173	sll1174
slr1856	slr1857
sll0856	slr1204
slr0454	slr0455
sll1815	ssl3441
slr1293	ssr2142
slr1406	slr1407
slr1666	ssr2781
sll0657	ssl1255
slr0151	slr0152
slr0789	slr0790
sll1182	slr1185
sll5094	ssl5095
slr1715	slr1716
sll1304	sll1305
sll7075	ssl7074
slr0699	slr0700
sll5042	sll5043
slr1206	slr1207
slr0216	slr0217
ssl5064	ssl5065
slr2121	slr2122
sll0543	ssl1046
slr1435	slr1436
sll0853	sll0854
sll0033	sll0034
sll5033	sll5034
slr6080	slr6081
sll1860	sll1861
sll0172	sll0173
slr0318	slr0319
sll0506	sll0507
slr0925	slr0925
sll0726	sll0727
sll1476	sll1477
slr1330	slr1331
slr0350	slr0351
slr0417	slr0418
slr1101	slr1102
sll1810	sll1811
slr1043	slr1044

sll0990	sll0992
sll1096	sll1097
sll1155	sll1156
slr7010	slr7011
sll0421	sll0422
sll0100	slr1653
slr6104	slr6105
slr0168	slr0169
sll0321	sll0322
sll0752	sll0753
sll0194	sll0195
sll1916	sll1917
sll1212	sll1213
slr1384	ssr2317
sll0379	sll0380
slr0383	slr0384
sll1709	ssl3291
sll0654	sll0656
slr1084	slr1085
slr0596	slr0597
slr1368	slr1369
slr0397	slr0398
sll1676	sll1677
slr1342	slr1343
slr0659	slr0661
slr1129	slr1130
sll1581	sll1582
slr1979	slr1980
sll1428	ssl2789
sll0376	sll0377
slr0491	slr0492
sll0759	sll0760
slr1653	slr1653
slr1689	slr1690
sll0868	sll0869
sll1407	sll1408
sll1801	sll1802
slr0851	slr0852
sll1698	sll1699
slr8045	slr8046
slr0619	ssr1041
slr0533	slr1044
sll1803	ssl3432
slr2024	slr2025
slr1363	slr1364
slr8014	ssr8013
slr1842	slr1843
slr1033	ssr1720
slr1612	slr1613
slr1254	slr1255
slr1931	slr1932

sll0156	sll0157
sll0443	sll0444
sll1574	sll1575
sll1028	sll1030
sll0423	sll0424
sll7030	sll7031
slr1615	slr1616
slr1880	slr1880
slr7104	slr7105
sll1293	sll1294
sll1694	slr1456
slr1673	slr1674
slr1030	slr1031
sll1871	sll1872
sll2009	sll2010
sll1769	ssl3383
slr6031	ssr6032
sll1474	sll1475
sll1744	sll1745
slr0082	slr0083
sll0200	sll0201
slr5037	slr5038
slr1247	slr1248
slr0006	slr0007
sll0930	sll0931
sll5046	ssl5045
slr2100	slr2101
slr1390	slr1391
sll0431	sll0846
slr1329	slr1330
sll1526	sll1527
sll0369	sll0370
slr0942	slr0943
slr0877	ssr1480
slr1829	slr1830
slr1853	slr1854
sll2014	sll2015
sll0895	sll0896
sll0176	sll0177
slr7014	slr7015
slr0864	slr0865
slr1070	slr1071
slr0322	slr0323
sll7028	sll7029
sll0100	sll0100
slr0379	slr0380
sll1020	sll1021
sll0763	sll0764
sll1805	ssl3436
slr0050	ssr0102
slr1978	slr1979

sll0676	ssl1277
slr1250	slr1251
slr1774	slr1776
sll1224	ssl2420
slr0655	slr0656
sll1578	sll1579
sll0414	sll0415
sll1049	sll1051
sll1527	sll1528
sll1305	slr1751
sll1680	sll1681
slr0618	slr0619
slr1912	slr1913
slr0900	slr0901
slr0606	slr0607
slr0323	slr0324
sll1260	sll1261
slr1616	slr1617
slr2080	ssr3532
slr0156	slr0157
slr1455	slr1456
sll1262	sll1263
sll1081	sll1082
sll0083	sll0084
sll0022	sll0023
slr8046	ssr8047
sll0702	sll0703
slr0585	slr0586
slr1266	slr1267
ssr2060	ssr2061
slr0070	slr0467
slr1282	slr1283
slr1518	ssr2554
sll0665	sll1716
slr1177	slr1178
sll1408	sll1409
slr1689	ssr2831
slr1767	ssr2962
sll1323	sll1324
slr0954	slr0955
slr1196	slr1197
slr0250	slr0251
slr2007	slr2008
slr7037	ssr7036
slr0542	slr0543
slr0376	slr0377
slr1223	slr1224
slr6028	slr6029
sll0102	sll0103
sll1436	sll1437
slr1881	slr1882

sll5049	sll5050	sll1734	sll1735	slr7094	slr7095	slr0583	slr0825
slr1533	slr1534	slr1103	slr1104	sll0284	sll0286	sll1577	sll1578
sll8009	ssl8008	slr1453	slr1454	slr0964	slr0965	slr2135	slr2136
slr2009	slr2010	slr1274	slr1275	sll0254	sll1182	sll1001	ssl1918
slr0040	slr0041	slr0703	smr0002	sll0863	sll0864	slr0990	slr0992
slr1258	slr1259	slr0513	slr0514	sll1862	sll1863	slr1234	slr1235
sll5062	sll5063	slr1991	slr1992	sll1723	sll1724	sll1119	sll1119
slr1276	slr1277	sll1225	ssl2420	sll0638	sll0639	sll1036	ssl2009
sll0666	sll1710	sll0625	sll0626	slr1611	slr1612	sll1318	sll1319
sll1399	ssl2733	slr0042	slr0043	sll0749	sll0750	ssl7021	ssl7022
sll0467	sll0597	sll0317	sll1780	slr0608	slr0609	sll0317	sll1560
slr0654	slr0655	slr0114	slr0115	sll1693	sll1694	slr0328	slr0329
slr8021	slr8022	slr0969	slr0971	slr1861	slr1862	sll1451	sll1452
slr6041	slr6042	slr6047	ssr6046	sll1058	sll1059	slr6005	slr6006
slr0677	slr0678	sll7089	sll7090	slr2079	ssr3532	sll1874	sll1875
sll0263	sll0264	sll8002	ssl8003	slr0483	slr0484	slr0984	slr0985
sll0176	ssl0312	slr1674	slr1675	sll1102	sll1103	sll0553	sll0554
sll1390	sll1866	slr1302	slr1303	sll1372	sll1373	slr1638	slr1639
sll1715	sll1716	sll0786	sll0787	sll0716	slr1377	slr2046	slr2047
sll0741	sll0742	slr1585	slr1586	slr1859	slr1860	slr0519	slr0520
sll0832	sll0833	slr1044	slr2099	slr1847	slr1848	slr2037	slr2038
sll1299	sll1300	slr1261	slr1262	slr1133	slr1134	slr0607	slr0608
slr6066	slr6067	slr0809	slr0810	sll0817	ssl1520	sll1979	sll1980
sll0897	ssl1707	sll1108	sll1109	sll1380	sll1381	slr7057	slr7058
slr0902	ssr1527	sll1994	sll1995	sll0838	sll0839	slr0936	slr0937
slr1078	ssr1766	sll1555	sll1556	slr0166	ssr0256	slr0904	ssr1528
slr1599	slr1600	slr1744	slr1746	sll1229	slr1044	slr1590	slr1591
sll1326	sll1327	sll0100	sll0101	slr6105	slr6106	sll1433	sll1434
slr0870	slr0871	slr0637	slr0638	sll0201	sll0431	slr0086	slr0088
sll8034	sll8035	slr1507	slr1508	slr0946	slr0947	slr0191	slr0192
slr1305	slr1306	slr0063	slr0064	sll0861	sll0862	sll1635	sml0005
sll0685	sll1980	slr1032	slr1033	sll1811	sll1812	sll1931	sll1932
slr1906	slr1907	sll8011	sll8012	sll0493	sll0902	slr1773	slr1774
sll1786	ssl2233	sll0383	sll0384	slr1240	slr1241	slr2120	slr2121
sll2012	sll2013	slr0689	ssr1155	slr6025	ssr6024	sll0012	sll0665
slr2036	slr2037	slr1424	slr1425	sll0319	sll0320	ssl0452	ssl0453
slr8037	slr8038	slr1234	ssr2049	sll8027	ssl8028	sll0584	sll0585
slr0387	slr0388	sll0665	sll0666	sll0027	sll1733	sll0539	sll0540
sll0980	sll0981	sll0622	sll0623	slr6031	ssr6030	sll8006	sll8007
slr0598	slr0599	slr1066	slr1067	sll0544	sll0545	sll0596	sll0597
slr0695	slr1590	sll0084	sll0085	sll0545	sll0546	slr6007	slr6008
slr1044	slr1414	slr0144	slr0145	slr1436	slr1437	slr1055	slr1056
slr0148	slr0149	slr0369	slr0370	slr1664	slr1665	slr1913	slr1914
slr0929	slr0930	slr1097	slr1098	sll1154	sll1155	slr0919	slr0920
slr1113	slr1501	sll0784	sll0785	ssl5098	ssl5099	sll0658	ssl1255
sll0677	sll0678	slr1385	ssr2318	sll0699	sll0846	sll1783	sll1784
slr0333	slr0334	slr2046	ssr3467	sll0847	sll0848	sll0686	sll0687
sll0242	sll0243	slr0755	ssr1258	sll1652	sll1653	sll0762	sll0763
sll1450	sll1451	sll5026	ssl5025	sll1156	sll1157	slr1434	slr1435
slr1083	slr1084	slr1301	slr1302	slr1454	slr1455	sll1556	sll1557
slr0090	slr0091	slr0058	slr0059	sll1232	sll1233	slr1813	slr1814

slr0168	slr0338
sll0249	ssl0461
slr0455	slr0456
sll1675	sll1676
slr7097	slr7098
sll0158	sll0160
slr1839	slr1840
slr7041	ssr7040
slr1924	slr1925
sll0933	ssl1784
slr0963	slr0964
sll1679	sll1680
sll1405	sll1406
sll1479	sll1481
sll1255	sll1474
sll0666	sll1716
slr0793	slr0794
slr0115	slr0116
sll1222	sll1223
sll1864	slr0172
sll1349	sll1350
slr1456	slr1457
sll1198	ssl2384
slr5012	slr5013
sll1912	sll1913
sll1629	sll1630
sll1682	sll1683
sll0680	sll0681
slr0011	slr0012
slr1071	slr1072
sll1265	sll1267
slr0601	slr0602
slr0344	slr0345
sll1459	sll1461
slr1437	slr1438
sll1816	sml0006
sll1187	sll1188
slr1077	ssr1765
slr0739	slr0740
slr1571	slr1572
sll1659	sll1660
sll0335	sll0336
sll0431	sll0699
sll1861	sll1862
sll0254	sll1027
sll6060	ssl6061
slr0853	slr0854
sll1272	sll1273
sll5043	sll5044
slr1034	slr1034
slr0955	slr0957

sll1060	ssl2069
slr6084	ssr6083
slr0812	slr0813
sll0677	ssl1277
sll1423	ssl2781
sll1217	sll1218
slr1702	ssr2843
slr1549	ssr2611
slr0899	slr0900
sll1239	sll1240
slr1909	slr1910
sll0661	ssl1263
sll1071	sll1072
slr0468	slr0469
sll0217	sll0218
slr1211	slr1212
sll1291	sll1292
slr1185	slr1185
slr0707	slr0708
slr0211	ssr0332
slr1509	slr1510
slr0679	slr0680
slr1828	slr1829
slr2011	slr2012
slr1808	slr1809
slr0676	slr0677
sll0754	ssl1417
sll0899	sll0900
sll1598	sll1599
sll1967	sll1968
sll0445	sll0446
sll1120	sll1121
sll1752	sml0005
sll0735	sll0736
slr6004	ssr6003
sll1053	sll1054
sll0144	sll0145
slr1541	slr1542
slr5055	slr5056
sll1316	slr1185
sll0920	sll0921
sll0413	ssl0787
sll1890	sll1891
slr0232	slr0233
slr0324	slr0325
sll1202	sll1203
slr0982	slr0983
slr0327	slr0328
slr1182	slr1183
sll1960	sll1961
slr1210	slr1211

sll1166	sll1447
slr0360	slr0361
sll1932	sll1933
slr1923	slr1924
sll1023	sll1024
slr5053	slr5054
sll1001	sll1002
sll1080	sll1081
slr0786	slr0918
slr1608	ssr2711
slr1202	slr1203
slr6063	ssr6062
sll0361	sll0362
slr1257	slr1258
slr1759	slr1760
slr1990	slr1991
sll1305	slr0008
sll1714	sll1715
sll0739	sll0740
slr2115	slr2116
slr1034	ssr1720
sll0371	ssl0738
sll0808	ssl1507
sll1018	sll1019
ssr2802	ssr2803
slr0634	slr0635
sll0473	sll0474
slr0642	slr0643
sll0519	sll0520
sll0018	sll0019
sll0023	sll0024
sll0047	sll0048
sll0546	sll0547
slr1233	ssr2047
slr1138	slr1139
slr1275	slr1276
slr1229	slr1230
slr0503	slr0505
sll0271	sll0272
sll0782	ssl0564
slr0120	slr0121
sll5060	sll5061
slr1044	slr1045
slr0053	slr0054
slr1542	slr1543
slr1521	slr1522
slr1081	ssr1768
slr0240	slr0241
ssr5019	ssr5020
sll0818	ssl1520
slr7091	slr7092

sll1997	sll1998
sll1568	sll1927
sll0264	sll0265
sll1702	sll1703
sll0687	sll0688
slr0399	slr0400
slr0600	slr0601
sll1764	sll1765
sll1809	sll1810
sll0183	sll0274
sll0933	sll0934
sll1473	sll1474
slr1739	slr1740
sll0875	sll0876
slr6063	slr6064
slr1174	ssr1951
slr1491	slr1492
sll1270	sll1271
slr0495	slr0496
slr0917	slr0918
sll0661	sll0662
slr0031	slr0032
slr8036	slr8037
sll0431	sll0700
sll0183	ssl0353
sll1377	sll1378
slr0400	slr0401
slr1063	slr1443
sll0181	ssl0350
slr0728	slr0729
slr0535	slr0536
sll0281	sll0282
sll0062	sll0063
slr7012	slr7013
sll1085	sll1566
slr1139	slr1140
slr6025	ssr6026
sll1800	sll1801
slr0903	ssr1527
slr0921	slr0922
sll1998	sll1999
sll0684	sll0685
slr1041	slr1042
slr0741	slr0742
sll1263	ssl1498
sll0095	sll0096
sll7047	ssl7048
slr6012	slr6013
sll7006	ssl7007
sll1816	sll1817
sll0744	sll0745

sll1040	sll1041
slr0989	slr0990
sll0012	sll0677
sll0857	sll0858
slr1898	slr1899
slr0055	slr0056
slr1807	slr1808
slr0249	slr0250
slr1307	ssr1604
slr0487	slr0488
sll1022	sll1023
sll0646	sll0647
slr0156	slr0156
sll1813	sll1814
slr6087	slr6088
slr0195	slr0196
slr1998	slr1999
slr1233	ssr2049
slr1690	slr1691
sll0630	sll0631
slr1718	slr1719
slr0378	slr0379
sll1202	sll1409
slr1943	ssr3304
sll0504	sll0505
slr1316	slr1317
slr1279	slr1280
slr5126	slr5127
sll0418	sll0419
sll5032	ssl5031
slr1127	slr1128
ssl1920	ssl1922
slr0794	slr0795
slr1670	slr1672
sll1488	sll1489
slr0038	slr0039
sll7003	ssl7004
sll1079	sll1080
slr1852	slr1853
slr0589	slr0590
slr6045	ssr6046
slr1245	ssr2078
slr1090	ssr1789
sll1691	sll1692
slr0844	ssr1425
sll5035	sll5036
sll1528	sll1530
slr1704	ssr2848
sll0629	sll0630
sll0822	sll0823
slr6021	slr6022

sll0188	sll0189
sll0183	sll1011
slr6039	slr6040
slr0912	slr0913
sll1505	ssl2920
slr1423	slr1424
slr5073	ssr5074
sll0912	sll0913
sll8040	ssl8039
sll1504	sll1505
slr6067	slr6068
slr0251	slr0252
sll1369	sll1370
sll1305	sll1306
slr1762	slr1763
slr1928	slr1929
slr5077	slr5078
sll0651	sll0700
sll1404	sll1405
sll1472	sll1473
slr0820	ssr1391
slr0808	slr0809
slr6050	slr6051
sll1249	sll1250
sll0666	sll0677
sll1191	sll1192
ssr6085	ssr6086
sll0267	sll0268
sll5044	ssl5045
sll1180	sll1181
slr0551	slr0552
sll1900	ssl3580
sll0419	sll0420
slr1074	slr1075
sll0135	ssl0242
sll0094	sll0095
sll1443	sll1444
slr0818	slr0819
slr0935	slr0936
slr7098	slr7099
sll0020	slr0156
sll1283	sll1284
sll1939	sll1940
sll7066	sll7067
slr1414	slr1415
slr7092	ssr7093
slr0304	slr0305
slr1843	slr1844
sll1983	sll1984
slr6013	slr6014
sll1398	sll1399

slr1565	slr1566
sll1987	sll1988
slr6090	ssr6089
sll1182	sll1369
smr0006	smr0007
sll0012	sll1710
sll1528	slr1443
sll0830	ssl1552
slr8014	slr8015
sll1051	sll1052
slr0649	slr0650
slr1910	slr1911
slr1137	slr1138
ssl5113	ssl5114
slr7015	slr7016
sll0525	ssl1004
slr1915	slr1916
sll1316	sll1317
slr0525	slr0526
sll1292	sll1293
sll1382	sll1383
sll1213	sll1214
sll0007	sll0008
sll1912	ssl3615
sll1968	sll1969
sll0856	sll1427
slr6065	slr6066
sll0665	sll0677
sll1488	slr0993
sll0787	ssl1464
sll0755	sll0756
slr1195	slr1196
slr1957	slr1958
slr0112	slr0114
sll1658	sll1659
sll7050	ssl7051
sll1348	sll1349
slr0746	slr0747
slr0145	slr0146
slr0529	slr0530
slr1855	slr1856
slr1444	ssr2422
sll1255	sll1780
slr1167	slr1168
sll0262	sll0263
sll0373	sll0374
sll1770	sll1771
sll0099	sll0100
slr1235	slr1236
slr0415	slr0416
sll0174	sll0175

slr0294	slr0392
slr0013	slr0014
sll0218	sll0219
slr8044	slr8045
slr0406	slr0407
slr1917	slr1918
sll1353	sll1354
sll1562	sll1563
slr1645	slr1646
ssr2061	ssr2061
slr1885	slr1886
slr0922	slr0923
sll0220	sll0221
sll0254	sll1316
sll5046	sll5047
slr1069	slr1070
slr6047	ssr6048
sll1382	slr0150
slr2044	slr2045
sll0932	ssl1784
slr2043	slr2044
slr1492	slr1493
sll0909	sll0910
sll1802	ssl3432
sll1481	sll1482
sll1708	ssl3291
sll0252	ssl0467
sll5030	ssl5031
slr1838	slr1839
sll0223	ssl0410
slr1618	slr1619
slr1679	ssr2799
slr1540	slr1541
sll0792	sll0793
slr0983	slr0984
sll0181	sll0182
sll1579	sll1580
sll5130	sll5131
sll0272	sll0273
slr1489	slr1490
slr0075	slr0076
sll1821	sll1822
slr1617	slr1618
sll0070	sll0071
slr0186	slr0359
slr0377	slr0378
slr1841	slr1842
slr2073	slr2074
sll1483	sll1484
slr1969	ssr3341
sll0649	sll0650

slr1076	slr1077
sll1434	sll1435
sll1883	sll1884
sll0063	sll0063a
slr1377	slr1378
sll7043	ssl7042
slr1572	slr1573
sll1525	ssl2971
sll0368	sll0369
sll0377	sll0378
sll1284	sll1285
sll1444	sll1446
slr0484	slr0487
sll1709	sll1710
slr1289	slr1290
slr1045	slr1046
sll1061	sll1062
slr1272	slr1273
sll0444	sll0445
sll0862	sll0863
slr1538	slr1540
slr0164	slr0165
slr2008	slr2009
sll0849	sll0851
slr7101	slr7102
sll1119	slr1880
sll1954	ssl3692
sll1131	sll1132
sll0891	ssl1690
sll1130	ssl2245
sll1867	sml0001
sll0729	ssl1378
slr0930	ssr1558
sll1185	sll1186
slr1614	slr1615
slr1944	slr1945
sll1352	sll1353
sll0293	sll0294
sll1725	sll1726
slr0321	slr0322
slr0862	slr0863
slr0072	slr0073
slr0149	slr0150
sll1321	sll1322
slr0502	slr0609
slr0737	slr0738
slr0624	slr0625
slr6096	slr6097
slr2080	slr2081
slr1383	ssr2317
sll0237	ssl0438

sll5069	ssl5068
slr0460	slr1683
slr0756	slr0757
sll1223	sll1224
slr1751	slr1752
slr1063	slr1064
slr1114	slr1115
sll1068	ssl3451
sll0269	sll0270
slr6021	ssr6020
sll1161	sll1162
sll1124	ssl2233
sll0698	sll0699
ssl5007	ssl5008
sll1573	sll1574
slr1490	slr1491
slr1794	slr1795
sll1323	ssl2615
slr1449	slr1450
sll1984	sll1985
sll1771	sll1772
slr5021	slr5022
slr2124	slr2125
sll0993	sll0994
sll0554	sll0555
slr1201	slr1202
slr6040	slr6041
slr0940	slr0941
slr5118	ssr5117
slr0095	slr0096
slr1469	smr0011
slr0236	slr0236
slr7080	ssr7079
sll1722	sll1723
sll0337	slr1044
slr0288	slr0291
slr5087	slr5088
slr0262	slr0263
slr1287	slr1288
sll0400	sll0401
sll0487	sll0488
sll0856	sll0857
sll0716	sll1043
sll5066	ssl5065
sll5090	ssl5091
slr1484	slr1485
sll0776	slr1063
slr7013	slr7014
slr1315	slr1316
sll1276	sll1277
sll0886	sll0887

slr0856	slr0857
slr0852	slr0853
slr0156	slr1641
sll1712	sll1713
sll0478	sll0479
sll1872	sll1873
slr2119	slr2120
slr1730	ssr2912
sll0754	sll0755
slr5101	slr5102
sll1959	sll1960
sll0497	sll0498
slr2114	slr2115
slr1463	slr1464
sll5109	ssl5108
slr6100	slr6101
slr1963	slr1964
sll0145	sll0146
sll0767	ssl1426
slr2035	smr0008
slr1510	slr1511
sll1500	sll1501
slr0054	slr0055
slr2059	slr2060
sll0682	sll0683
slr1773	ssr2972
slr1681	slr1682
sll7065	sll7066
slr0147	slr0148
slr1927	slr1928
sll0258	sll0427
slr6071	slr6072
slr0807	slr0808
slr0287	ssr0482
sll1561	sll1562
slr1364	slr1365
slr1693	slr1694
sll1867	slr1311
slr1648	ssr2754
sll0873	ssl1633
sll2001	ssl2749
sll0788	sll0789
sll1926	sll1927
sll1423	sll1424
slr0041	slr0042
sll1281	sll1282
slr0380	slr0381
sll1915	sll1916
slr0966	slr0967
sll1528	slr1225
sll0815	sll0816

slr0978	slr0980
sll0183	ssl0352
sll0647	sll0648
slr0053	ssr0109
slr1811	slr1812
slr7023	slr7024
slr0965	slr0966
slr1753	slr1755
slr0833	slr0835
sll1229	sll1231
sll1464	ssl2823
slr1812	slr1813
sll0843	sll0844
slr1646	slr1647
slr1396	slr1397
sll0690	ssl1300
slr1877	slr1878
sll1151	ssl2296
ssr5120	ssr5121
slr1052	slr1053
sll0750	sll0750
sll0996	sll0997
slr1899	slr1900
slr1218	slr1219
sll0236	ssl0438
slr2098	slr2099
slr1469	slr1470
sll0810	sll0811
slr7100	slr7101
sll0258	sll1194
slr1916	slr1917
sll0412	sll0413
slr1096	slr1097
sll0699	sll0700
slr0110	slr0111
slr2002	slr2003
slr0588	slr0589
slr0080	slr0081
sll1510	sll1511
slr0239	slr0240
sll1103	sll1104
slr0456	slr0457
sll0648	sll0649
slr1936	slr1937
sll0224	sll0225
ssl3382	ssl3383
slr0210	ssr0332
sll1557	sll1558
sll1549	sll1550
sll5066	sll5067
slr0242	slr0243

sll0738	sll0739
sll0640	sll0641
sll6053	sll6054
slr2082	slr2083
slr1203	slr1204
slr0032	slr0033
sll0254	slr1254
sll1745	sll1746
slr1934	slr1935
slr0261	slr0262
sll0201	sll0700
slr2070	slr2071
slr0664	ssr1114
sll5131	sll5132
slr1442	slr1443
sll1162	sll1163
slr0770	slr0771
sll1221	sll1222
sll0685	sll0686
sll1158	sll1159
slr0737	slr1655
slr1593	slr1594
sll1186	sll1187
sll7064	sll7065
sll0410	sll0412
sll0776	sll1528
sll1822	ssl3445
sll0098	sll0099
slr0704	ssr1176
slr1783	slr1784
sll1252	sll1253
slr1672	slr1673
sll1749	sll1749a
slr1885	ssr3189
slr0743	slr0743a
sll1254	sll1255
slr6095	slr6096
sll0761	sll0762
sll1941	sll1942
slr2113	slr2114
sll0710	sll0711
slr1803	slr2144
sll2008	sll2009
slr0775	slr0776

slr1238	ssr2061
sll1342	sll1343
slr0171	slr0172
sll1763	sll1764
sll1482	sll1483
slr1081	slr1082
slr0341	slr0784
slr1224	slr1225
slr1576	slr1577
slr0799	slr0800
sll1098	sll1099
sll0201	sll0651
slr7016	ssr7017
slr0723	slr0724
slr2004	ssr3402
slr0886	slr0887
slr0386	slr0387
slr1729	ssr2912
sll1469	sll1712
sll1325	sll1326
slr1821	slr1822
sll0135	sll0136
sll1371	sll1372
sll1025	ssl1972
slr1208	slr1209
sll7033	sll7034
slr0043	slr0044
sll1078	sll1079
slr2107	slr2108
slr0331	slr1291
slr1881	ssr3184
slr6074	slr6075
sll1393	sll1394
sll1732	sll1733
sll1878	sll1879
sll0775	sll0776
sll0244	sll0245
slr0697	slr0698
sll0222	sll0223
sll0524	ssl1004
slr1453	ssr2439
sll1807	sll1808
sll0264	sll1869
sll0572	sll0573

slr1311	sml0001
slr1977	slr1978
sll1030	sll1031
sll0709	sll0710
smr0007	smr0008
slr1999	slr2000
slr6014	slr6015
slr6049	ssr6048
sll1775	sll1776
slr0949	slr0950
sll7047	ssl7046
slr0514	slr0516
slr0541	slr0542
slr0237	slr0238
slr1902	slr1903
slr0959	slr0960
slr1721	slr1722
sll0451	sll1542
sll0408	sll0409
slr1124	slr1125
sll1294	sll1296
slr0530	slr0531
slr1860	slr1861
sll1057	sll1058
slr1468	smr0011
slr0092	slr0093
sll0650	sll0651
sll0317	sll1255
slr0489	slr0491
slr0680	slr0681
sll0508	sll0509
slr0918	slr0919
sll1749	sll1750
slr1376	slr1377
slr0667	slr0668
sll0041	sll0042
slr1854	slr1855
sll1532	ssl2982
sll5047	sll5048
sll0595	sll0596
slr0951	slr0952
slr0533	slr0534
sll0901	sll0902
sll0397	sll0398

sll0446	sll0447
slr1720	slr1721
slr1601	smr0005
slr1327	ssr2201
slr1198	ssr2009
slr0746	slr1588
sll1095	sll1096
slr1544	ssr2595
sll5128	ssl5129
sll0613	sll0614
sll1737	sll1738
sll1819	sll1820
slr1082	slr1083
sll1762	sll1763
slr0196	slr0197
slr2016	slr2017
ssr0759	ssr0761
sll1021	sll1022
sll0586	sll0587
slr0105	slr0106
slr1186	slr1187
sll1324	sll1325
slr0604	slr0605
slr1392	ssr2333
sll1623	sll1624
slr0904	slr0905
slr0780	smr0001
sll1888	ssl3549
slr6042	slr6043
sll1537	sll1538
slr0804	slr0806
slr5017	slr5018
slr1986	slr2067
slr0833	ssr1407
sll1446	sll1447
sll1521	sll1522
slr6056	slr6057
slr2058	slr2059
slr5023	slr5024
sll0066	sll0067
sll5059	sll5060

Supplemental Table S4. Primers used for yeast two-hybrid experiments.

Name	Primer sequence (5'-3')
ndhA-FP	CGGAATTCATGACTTCAGGCATTGATCTTCAG
ndhA-RP	CCCCTCGAGCTAACCGCCAAAGGCCATGGGAAA
ndhB-FR	CCCCTCGAGATGGACTTTTCTAGTAACGTTGCA
ndhB-RR	CCCCTCGAGCTAGGGTAAATCATGGGAAATGGC
ndhH-FP	CCCCTCGAGATGCCGCCTTGCCTCCCCAATGGC
ndhH-RP	CCCCTCGAGCTAGCGGTCCACCGATCCCATGAT
ndhI-FP	CCCCTCGAGATGTTTAACAACATTCTCAAACAG
ndhI-RP	CCCCTCGAGCTATTCTGCTTTCACCAAATCTTC
ndhJ-FP	CGGAATTCGTGGCTGAGGAAGTGAACTCCCCC
ndhJ-RP	CCCCTCGAGCTAATAGGCATCCTGGAGTTCGTA
ndhK-FP	CGGAATTCATGAGTCCCAACCCTGCTAACCCC
ndhK-RP	CCCCTCGAGTCAGCCACGGTTTAATTGCTCCTT
ndhO-FP	CGGAATTCATGGCCGCTAAAATGAAAAAGG
ndhO-RP	CCCCTCGAGCTAAGCCAGGGCTTCGATTTGG

Supplementary Table S5 Literature verification of predicted PPIs.

Pubmed ID	Protein A	Protein B	Experiments
19843617	slr1644	slr0506	Y2H
12966069	slr1098	slr1565	Y2H
9418040	slr1777	slr1030	Y2H
19542180	sll0698	ssl3451	Y2H
17921338	sll1867	sml0001	Y2H
17921338	slr1311	sml0001	Y2H
17921338	slr1181	sml0001	Y2H
15328351	sll1867	slr1311	Y2H
12232386	slr0737	slr1655	Y2H
12232386	ssr2831	sll0819	Y2H
21642457	sll0359	sll0822	Y2H
16109709	sll0985	ssl0707	Y2H
14730074	slr0342	slr0343	Y2H
14730074	sll1327	sll1326	Y2H
14730074	sll1735	sll1734	Y2H
14730074	sll1326	sll1325	Y2H
11532008	slr0473	slr0474	Y2H
8325373	slr1280	slr1281	Y2H
15262969	slr1185	sll1316	2MUTANT
10906128	slr0331	slr1291	2MUTANT
16049785	slr2051	sll1471	2MUTANT
11562454	sll1733	sll0027	2MUTANT
9211937	sll0258	sll1194	2MUTANT
7548017	sll0258	sll0427	2MUTANT
16002996	slr1693	slr1694	Y2H

Supplementary Table S6 Functional assignment for function-unknown proteins.

slr0299	GO:0072524	<1e-4	slr0317	GO:0048522	<1e-4	slr1260	GO:0043436	<1e-4	slr1737	GO:0015291	<1e-4	ssr3341	GO:0016020	0.3576
slr1906	GO:0006793	<1e-4	slr7099	GO:0008150	<1e-4	slr0503	GO:0043436	<1e-4	slr1062	GO:0015291	<1e-4	slr1097	GO:0016020	0.3576
sll1609	GO:0051716	<1e-4	ssr5092	GO:0009142	<1e-4	slr0613	GO:0006793	<1e-4	sll5132	GO:0015267	<1e-4	slr1407	GO:0016020	0.3576
ssr2615	GO:0022411	<1e-4	sll1632	GO:0019637	<1e-4	slr6101	GO:0009893	<1e-4	ssr2962	GO:0022891	<1e-4	sll1132	GO:0016020	0.3576
sll0022	GO:0019222	<1e-4	sll0793	GO:0022607	<1e-4	ssr2962	GO:0043549	<1e-4	slr6008	GO:0015293	<1e-4	sll6054	GO:0016020	0.3576
slr0053	GO:0006793	<1e-4	ssl5064	GO:0031326	<1e-4	sll0487	GO:0009132	<1e-4	sll0749	GO:0016462	<1e-4	sll0400	GO:0016020	0.3576
slr0981	GO:0009132	<1e-4	slr1563	GO:0044282	<1e-4	sll0930	GO:0051171	<1e-4	sll0141	GO:0016741	<1e-4	slr7023	GO:0016020	0.3576
ssr5121	GO:0044260	<1e-4	slr1644	GO:0046128	<1e-4	slr7025	GO:0009126	<1e-4	slr1437	GO:0016462	<1e-4	sll1853	GO:0016020	0.3576
slr0304	GO:0080090	<1e-4	slr1169	GO:0006091	<1e-4	ssr1375	GO:0006091	<1e-4	sll1902	GO:0022838	<1e-4	sll0167	GO:0016020	0.3576
sll5030	GO:0044282	<1e-4	ssl3382	GO:0043412	<1e-4	ssl5007	GO:0052188	<1e-4	slr6103	GO:0022892	<1e-4	sll0863	GO:0016020	0.3576
slr7037	GO:0006732	<1e-4	sll1002	GO:0072524	<1e-4	sll0691	GO:0044281	<1e-4	slr1143	GO:0015291	<1e-4	slr1365	GO:0016020	0.3576
slr2105	GO:0009057	<1e-4	ssr2317	GO:0071496	<1e-4	sll0545	GO:0042180	<1e-4	sll1880	GO:0016741	<1e-4	sll0423	GO:0016020	0.3576
slr1150	GO:0006576	<1e-4	sll1608	GO:0043648	<1e-4	slr6068	GO:0071554	<1e-4	slr1778	GO:0015075	<1e-4	sll1880	GO:0016020	0.3576
slr1505	GO:0051188	<1e-4	slr7011	GO:0051188	<1e-4	ssr5121	GO:0055114	<1e-4	ssr2787	GO:0022892	<1e-4	ssl0312	GO:0016020	0.3576
ssr2009	GO:0009124	<1e-4	slr1547	GO:0009263	<1e-4	ssl0353	GO:0071496	<1e-4	sll0361	GO:0042623	<1e-4	slr1468	GO:0016020	0.3576
sll0639	GO:0006766	<1e-4	sll0888	GO:0071496	<1e-4	sll0793	GO:0019637	<1e-4	slr2048	GO:0016746	<1e-4	sll8032	GO:0016020	0.3576
ssr5019	GO:0009132	<1e-4	sll0780	GO:0010468	<1e-4	ssr3189	GO:0045184	<1e-4	sll8007	GO:0022836	<1e-4	sll5046	GO:0016020	0.3576
sll1233	GO:0006721	<1e-4	sll0995	GO:0045184	<1e-4	ssl5114	GO:0006811	<1e-4	sll1505	GO:0008171	<1e-4	sll1766	GO:0016020	0.3576
slr1053	GO:0009059	<1e-4	sll0218	GO:0009308	<1e-4	sll1995	GO:0051186	<1e-4	slr1365	GO:0016741	<1e-4	sll1414	GO:0016020	0.3576
ssl3692	GO:0080090	<1e-4	sll0444	GO:0009142	<1e-4	slr1240	GO:0001932	<1e-4	sll1853	GO:0042623	<1e-4	slr5119	GO:0016020	0.3576
slr1307	GO:0044106	<1e-4	slr0476	GO:0044283	<1e-4	sll0854	GO:0019219	<1e-4	sll2013	GO:0015405	<1e-4	slr1035	GO:0016020	0.3576
slr0192	GO:0071840	<1e-4	slr1436	GO:0009132	<1e-4	sll0670	GO:0043170	<1e-4	slr0482	GO:0043167	<1e-4	ssl2245	GO:0016020	0.3576
sll0846	GO:0010556	<1e-4	sll1267	GO:0044003	<1e-4	sll0286	GO:0051186	<1e-4	slr1206	GO:0022803	<1e-4	sll0658	GO:0016020	0.3576
slr1519	GO:0044255	<1e-4	slr0207	GO:0009987	<1e-4	slr1122	GO:0051171	<1e-4	slr0581	GO:0042623	<1e-4	sll1155	GO:0016020	0.3576
ssl7039	GO:0055114	<1e-4	slr0609	GO:0019219	<1e-4	sll0487	GO:0031323	<1e-4	sll1135	GO:0015077	<1e-4	sll1273	GO:0016020	0.3576
sll1784	GO:0006766	<1e-4	sll0765	GO:0071841	<1e-4	ssl5100	GO:0006082	<1e-4	ssl5100	GO:0005342	<1e-4	slr7015	GO:0016020	0.3576
sll1350	GO:0010556	<1e-4	sll1950	GO:0060255	<1e-4	slr0503	GO:0031323	<1e-4	slr0388	GO:0043492	<1e-4	sll0063	GO:0016020	0.3576
slr0978	GO:0006721	<1e-4	sll0614	GO:0065007	<1e-4	sll0496	GO:0006163	<1e-4	slr1303	GO:0008171	<1e-4	sll1461	GO:0016020	0.3576
slr0912	GO:0045184	<1e-4	slr1603	GO:0072522	<1e-4	ssr5106	GO:0019637	<1e-4	ssl5108	GO:0015399	<1e-4	slr1068	GO:0016020	0.3576
slr1854	GO:0034654	<1e-4	ssl5096	GO:0034660	<1e-4	sll1022	GO:0009126	<1e-4	slr1900	GO:0022890	<1e-4	sll0630	GO:0016020	0.3576
ssr2781	GO:0042180	<1e-4	sll1447	GO:0065007	<1e-4	sll1681	GO:0043436	<1e-4	slr0386	GO:0015077	<1e-4	sll0872	GO:0016020	0.3576
slr1273	GO:0006811	<1e-4	ssl2814	GO:0051716	<1e-4	slr1084	GO:0009892	<1e-4	sll1396	GO:0022838	<1e-4	slr0148	GO:0016020	0.3576
sll1925	GO:0051186	<1e-4	sll0238	GO:0071554	<1e-4	sll0272	GO:0006766	<1e-4	slr6100	GO:0003674	<1e-4	slr1161	GO:0016020	0.3576
slr1865	GO:0050896	<1e-4	slr0169	GO:0009165	<1e-4	slr6049	GO:0043170	<1e-4	ssl5068	GO:0022838	<1e-4	ssl1792	GO:0016020	0.3576
ssl3692	GO:0008150	<1e-4	slr1273	GO:0019222	<1e-4	slr0871	GO:0050794	<1e-4	sll1940	GO:0003674	<1e-4	sll0994	GO:0016020	0.3576
slr0313	GO:0019751	<1e-4	slr1726	GO:0055086	<1e-4	slr1566	GO:0046164	<1e-4	slr7094	GO:0022892	<1e-4	sll1764	GO:0016020	0.3576
slr6081	GO:0018130	<1e-4	sll0933	GO:0050794	<1e-4	sll0325	GO:0006220	<1e-4	sll0858	GO:0016746	<1e-4	slr6073	GO:0016020	0.3576
slr0848	GO:0048523	<1e-4	slr1926	GO:0072524	<1e-4	sll1068	GO:0019222	<1e-4	sll5044	GO:0016462	<1e-4	slr0388	GO:0016020	0.3576
sll1735	GO:0031326	<1e-4	slr6065	GO:0019637	<1e-4	sll0445	GO:0051246	<1e-4	sll1660	GO:0016818	<1e-4	slr6065	GO:0016020	0.3576
sll0253	GO:0044282	<1e-4	slr0876	GO:0006066	<1e-4	slr0709	GO:0009889	<1e-4	sll1289	GO:0008171	<1e-4	slr1619	GO:0016020	0.3576
sll0024	GO:0006066	<1e-4	sll1273	GO:0008610	<1e-4	slr6033	GO:0006576	<1e-4	slr1907	GO:0022838	<1e-4	slr1162	GO:0016020	0.3576
slr6068	GO:0072528	<1e-4	slr1107	GO:0009126	<1e-4	sll0168	GO:0046394	<1e-4	slr1917	GO:0015293	<1e-4	slr7083	GO:0016020	0.3576
sll0286	GO:0006082	<1e-4	slr1376	GO:0009144	<1e-4	slr1290	GO:0046483	<1e-4	sll0188	GO:0022857	<1e-4	sll1862	GO:0016020	0.3576
slr1415	GO:0071554	<1e-4	ssl1417	GO:0043412	<1e-4	sll0048	GO:0051188	<1e-4	ssl5098	GO:0003674	<1e-4	ssl2920	GO:0016020	0.3576
ssr3410	GO:0051188	<1e-4	ssr0657	GO:0071841	<1e-4	slr2119	GO:0044282	<1e-4	slr0645	GO:0016818	<1e-4	sll0710	GO:0016020	0.3576
sll0853	GO:0009308	<1e-4	sll0630	GO:0006733	<1e-4	sll2006	GO:0044260	<1e-4	sll0585	GO:0043167	<1e-4	slr1069	GO:0016020	0.3576
slr0590	GO:0016054	<1e-4	ssl0788	GO:0008150	<1e-4	slr6080	GO:0080090	<1e-4	sll0696	GO:0003674	<1e-4	slr1472	GO:0016020	0.3576
sll0847	GO:0006220	<1e-4	sll1446	GO:0010556	<1e-4	slr6008	GO:0010556	<1e-4	sll1532	GO:0022892	<1e-4	sll1004	GO:0016020	0.3576
slr0358	GO:0001932	<1e-4	ssr2009	GO:0009262	<1e-4	slr0380	GO:0006082	<1e-4	ssr5121	GO:0042623	<1e-4	slr1270	GO:0016020	0.3576
slr1241	GO:0009263	<1e-4	slr1033	GO:0052111	<1e-4	slr0476	GO:0044260	<1e-4	sll1192	GO:0016746	<1e-4	slr1363	GO:0016020	0.3576
ssl1300	GO:0044248	<1e-4	sll7067	GO:0009991	<1e-4	sll0325	GO:0046394	<1e-4	ssr2998	GO:0015077	<1e-4	sll5032	GO:0016020	0.3576
slr0148	GO:0009199	<1e-4	sll1130	GO:0044283	<1e-4	sll0424	GO:0050789	<1e-4	slr1240	GO:0016462	<1e-4	slr0393	GO:0016020	0.3576
sll0909	GO:0052111	<1e-4	slr0962	GO:0009262	<1e-4	slr0634	GO:0010468	<1e-4	slr1956	GO:0022803	<1e-4	slr1378	GO:0016020	0.3576

sll0160	GO:0018130	<1e-4	sll1797	GO:0009987	<1e-4	slr1812	GO:0051186	<1e-4	slr0586	GO:0016741	<1e-4	slr0145	GO:0016020	0.3576
ssl3829	GO:0006732	<1e-4	slr2049	GO:0072521	<1e-4	slr7095	GO:0031640	<1e-4	ssr6089	GO:0003674	<1e-4	ssr6003	GO:0016020	0.3576
slr1959	GO:0009150	<1e-4	sll1106	GO:0006811	<1e-4	slr1790	GO:0001932	<1e-4	sll5030	GO:0022891	<1e-4	sll0436	GO:0016020	0.3576
sll1186	GO:0048518	<1e-4	sll0098	GO:0006082	<1e-4	slr0408	GO:0009124	<1e-4	slr0668	GO:0015399	<1e-4	sll0815	GO:0016020	0.3576
sll1681	GO:0009132	<1e-4	sll0406	GO:0043436	<1e-4	sll1873	GO:0072521	<1e-4	ssr5092	GO:0043167	<1e-4	sll1396	GO:0016020	0.3576
sll0980	GO:0046164	<1e-4	slr1186	GO:0050794	<1e-4	slr1753	GO:0050789	<1e-4	sll1722	GO:0022836	<1e-4	slr0959	GO:0016020	0.3576
slr0980	GO:0008610	<1e-4	slr1864	GO:0009056	<1e-4	slr1441	GO:0031323	<1e-4	sll0180	GO:0008171	<1e-4	slr1484	GO:0016020	0.3576
ssr2047	GO:0005996	<1e-4	sll7065	GO:0016052	<1e-4	slr1301	GO:0044003	<1e-4	slr0516	GO:0022857	<1e-4	sll5062	GO:0016020	0.3576
slr1544	GO:0009262	<1e-4	sll0068	GO:0060255	<1e-4	sll8032	GO:0080090	<1e-4	ssr7035	GO:0043492	<1e-4	slr1116	GO:0016020	0.3576
slr1327	GO:0042455	<1e-4	sll1381	GO:0009126	<1e-4	sll0639	GO:0043170	<1e-4	slr0981	GO:0022803	<1e-4	slr1566	GO:0016020	0.3576
slr1956	GO:0044248	<1e-4	sll0174	GO:0044003	<1e-4	ssl7021	GO:0009263	<1e-4	sll0781	GO:0015267	<1e-4	slr0179	GO:0016020	0.3576
slr2011	GO:0008150	<1e-4	slr5013	GO:0009394	<1e-4	slr0476	GO:0006811	<1e-4	sll0702	GO:0015077	<1e-4	slr7095	GO:0016020	0.3576
slr1616	GO:0048518	<1e-4	ssr1155	GO:0006091	<1e-4	sll5033	GO:0044003	<1e-4	slr0147	GO:0022838	<1e-4	sll5034	GO:0016020	0.3576
sll0984	GO:0034654	<1e-4	ssl0739	GO:0044255	<1e-4	slr0317	GO:0044255	<1e-4	ssl1690	GO:0016746	<1e-4	sll1130	GO:0016020	0.3576
slr1557	GO:0042180	<1e-4	sll0023	GO:0043933	<1e-4	ssr1391	GO:0043549	<1e-4	sll0925	GO:0043492	<1e-4	ssr0332	GO:0016020	0.3576
slr7071	GO:0006766	<1e-4	sll1510	GO:0046394	<1e-4	sll1542	GO:0072527	<1e-4	ssl7007	GO:0060089	<1e-4	sll0518	GO:0016020	0.3576
slr0505	GO:0006720	<1e-4	sll1401	GO:0044271	<1e-4	ssl1918	GO:0006066	<1e-4	slr0890	GO:0016817	<1e-4	sll1166	GO:0008152	0.3466
ssr7093	GO:0051179	<1e-4	slr6081	GO:0009263	<1e-4	slr0059	GO:0019222	<1e-4	sll1036	GO:0015291	<1e-4	sll1247	GO:0008152	0.3466
sll0981	GO:0050801	<1e-4	sll1541	GO:0006811	<1e-4	ssr7093	GO:0009126	<1e-4	slr2121	GO:0022832	<1e-4	slr1533	GO:0008152	0.3466
slr0967	GO:0009309	<1e-4	sll1477	GO:0050801	<1e-4	sll0062	GO:0043648	<1e-4	sll1722	GO:0016462	<1e-4	ssr1375	GO:0008152	0.3466
slr7060	GO:0008610	<1e-4	slr0142	GO:0006066	<1e-4	slr1566	GO:0048519	<1e-4	slr0404	GO:0022857	<1e-4	slr1034	GO:0008152	0.3466
sll1461	GO:0006811	<1e-4	ssr7084	GO:0051234	<1e-4	sgl0002	GO:0009161	<1e-4	sll0995	GO:0015399	<1e-4	sll1060	GO:0008152	0.3466
slr0272	GO:0065007	<1e-4	slr0590	GO:0009144	<1e-4	sll1995	GO:0034654	<1e-4	sll8032	GO:0008171	<1e-4	slr1900	GO:0008152	0.3466
sll0590	GO:0009126	<1e-4	ssr2787	GO:0033013	<1e-4	slr0059	GO:0034660	<1e-4	slr1288	GO:0043167	<1e-4	ssr1698	GO:0008152	0.3466
slr8014	GO:0008150	<1e-4	sll7089	GO:0006720	<1e-4	sll0405	GO:0022411	<1e-4	slr0789	GO:0005342	<1e-4	sll0360	GO:0008152	0.3466
slr1074	GO:0044282	<1e-4	sll1651	GO:0044283	<1e-4	slr1116	GO:0072521	<1e-4	slr1851	GO:0016741	<1e-4	slr1107	GO:0008152	0.3466
slr1726	GO:0048518	<1e-4	slr1413	GO:0044003	<1e-4	slr0491	GO:0046907	<1e-4	ssl3829	GO:0015405	<1e-4	sll0499	GO:0008152	0.3466
slr1082	GO:0008150	<1e-4	slr0092	GO:0051179	<1e-4	slr0579	GO:0048522	<1e-4	slr0637	GO:0015267	<1e-4	sll1715	GO:0008152	0.3466
sll1049	GO:0009263	<1e-4	slr2012	GO:0051179	<1e-4	slr1676	GO:0009161	<1e-4	slr0655	GO:0022803	<1e-4	sll0096	GO:0008152	0.3466
ssr2553	GO:0009991	<1e-4	slr1437	GO:0051171	<1e-4	slr1429	GO:0060255	<1e-4	ssr3571	GO:0005342	<1e-4	ssr3532	GO:0008152	0.3466
sll0505	GO:0009150	<1e-4	ssr1391	GO:0050789	<1e-4	sll1654	GO:0009059	<1e-4	slr0592	GO:0022890	<1e-4	sll0274	GO:0008152	0.3466
slr1073	GO:0016053	<1e-4	slr1084	GO:0080090	<1e-4	slr1659	GO:0055082	<1e-4	slr0392	GO:0015267	<1e-4	slr0305	GO:0008152	0.3466
sll0552	GO:0051716	<1e-4	slr0489	GO:0044283	<1e-4	slr1071	GO:0006066	<1e-4	slr7015	GO:0003674	<1e-4	slr1083	GO:0008152	0.3466
sll1658	GO:0006811	<1e-4	sll0847	GO:0046128	<1e-4	slr6049	GO:0046128	<1e-4	slr1235	GO:0022891	<1e-4	slr1896	GO:0008152	0.3466
slr6072	GO:0065007	<1e-4	slr2120	GO:0005996	<1e-4	slr1613	GO:0009892	<1e-4	sll7034	GO:0043167	<1e-4	sll1671	GO:0008152	0.3466
slr1186	GO:0009893	<1e-4	slr1657	GO:0031640	<1e-4	ssr3402	GO:0006793	<1e-4	sll0198	GO:0022891	<1e-4	ssl0109	GO:0008152	0.3466
sll0981	GO:0009987	<1e-4	sll1123	GO:0065007	<1e-4	slr1644	GO:0044267	<1e-4	sll1252	GO:0022857	<1e-4	slr1194	GO:0008152	0.3466
slr0108	GO:0043549	<1e-4	sll0103	GO:0009987	<1e-4	slr0586	GO:0044255	<1e-4	sll5130	GO:0042623	<1e-4	slr1127	GO:0008152	0.3466
ssl5103	GO:0048878	<1e-4	slr0270	GO:0009142	<1e-4	slr6013	GO:0071554	<1e-4	sll1006	GO:0022892	<1e-4	sll0996	GO:0008152	0.3466
slr1926	GO:0009263	<1e-4	slr1670	GO:0019752	<1e-4	sll0742	GO:0006732	<1e-4	slr1472	GO:0022892	<1e-4	slr1074	GO:0008152	0.3414
ssr2998	GO:0022607	<1e-4	slr1362	GO:0006733	<1e-4	sll0157	GO:0072527	<1e-4	slr1442	GO:0015399	<1e-4	sll0281	GO:0008152	0.3414
slr0285	GO:0046907	<1e-4	slr7073	GO:0044271	<1e-4	slr1266	GO:0051347	<1e-4	slr7011	GO:0016741	<1e-4	sll0669	GO:0008152	0.3414
slr7095	GO:0009309	<1e-4	slr0111	GO:0031326	<1e-4	slr1926	GO:0019752	<1e-4	slr0209	GO:0022890	<1e-4	sll0909	GO:0008152	0.3414
ssl2065	GO:0019751	<1e-4	slr0144	GO:0044281	<1e-4	slr0291	GO:0044248	<1e-4	sll0447	GO:0022836	<1e-4	sll1659	GO:0008152	0.3414
sll0372	GO:0043436	<1e-4	sll1469	GO:0009263	<1e-4	slr0013	GO:0031640	<1e-4	sll7033	GO:0005342	<1e-4	sll1173	GO:0008152	0.3414
ssr3572	GO:0009144	<1e-4	sll8004	GO:0006091	<1e-4	slr0876	GO:0052111	<1e-4	sll0263	GO:0015399	<1e-4	sll0980	GO:0008152	0.3414
sll0192	GO:0006753	<1e-4	slr7098	GO:0019222	<1e-4	sll0405	GO:0009308	<1e-4	sll0847	GO:0042623	<1e-4	sll0156	GO:0008152	0.3414
sll0069	GO:0009165	<1e-4	slr1472	GO:0051818	<1e-4	slr6074	GO:0044106	<1e-4	sll0996	GO:0022832	<1e-4	sll0503	GO:0008152	0.3414
sll1217	GO:0008150	<1e-4	slr7059	GO:0006720	<1e-4	sll1355	GO:0044271	<1e-4	slr1547	GO:0016741	<1e-4	slr0924	GO:0008152	0.3414
slr6049	GO:0050801	<1e-4	slr0935	GO:0006576	<1e-4	sll1036	GO:0034654	<1e-4	ssl7007	GO:0022891	<1e-4	ssr1528	GO:0008152	0.3414
sll1853	GO:0090304	<1e-4	slr0304	GO:0044271	<1e-4	slr0517	GO:0006576	<1e-4	sll0314	GO:0003674	<1e-4	sll0740	GO:0008152	0.3414
sll1696	GO:0010468	<1e-4	sll0487	GO:0042455	<1e-4	sll1061	GO:0071840	<1e-4	slr1173	GO:0022892	<1e-4	slr1071	GO:0008152	0.3414
slr0731	GO:0019637	<1e-4	sll1736	GO:0065007	<1e-4	slr5077	GO:0009132	<1e-4	sll1660	GO:0060089	<1e-4	sll1634	GO:0008152	0.3414
slr1406	GO:0046164	<1e-4	slr1104	GO:0009161	<1e-4	sll0843	GO:0019222	<1e-4	ssl2009	GO:0022890	<1e-4	sml0011	GO:0008152	0.3414

slr0740	GO:0048518	<1e-4	ssl0352	GO:0009142	<1e-4	slr0294	GO:0009987	<1e-4	sll1158	GO:0015075	<1e-4	sll1399	GO:0008152	0.3414
sll0584	GO:0009394	<1e-4	sll1658	GO:0048523	<1e-4	slr0960	GO:0022607	<1e-4	slr5018	GO:0043492	<1e-4	sll0843	GO:0008152	0.3414
sll1979	GO:0072528	<1e-4	slr2060	GO:0010468	<1e-4	slr0959	GO:0009309	<1e-4	sll5028	GO:0008171	<1e-4	sll1225	GO:0008152	0.3414
sll0564	GO:0072522	<1e-4	slr1519	GO:0055114	<1e-4	ssl5095	GO:0008150	<1e-4	slr1471	GO:0008324	<1e-4	slr0789	GO:0008152	0.3414
slr1210	GO:0043412	<1e-4	slr1612	GO:0046164	<1e-4	slr0702	GO:0009987	<1e-4	sll1858	GO:0005342	<1e-4	slr1128	GO:0008152	0.3414
slr1495	GO:0006766	<1e-4	sll1095	GO:0009150	<1e-4	ssr6030	GO:0034641	<1e-4	slr2121	GO:0015405	<1e-4	sll0359	GO:0008152	0.3414
ssr2912	GO:0009199	<1e-4	sll1472	GO:0006766	<1e-4	slr5101	GO:0044281	<1e-4	ssr1951	GO:0042623	<1e-4	sll1660	GO:0008152	0.3414
sll0539	GO:0048878	<1e-4	sll1586	GO:0010556	<1e-4	slr1240	GO:0051347	<1e-4	sll0354	GO:0042623	<1e-4	sll1680	GO:0008152	0.3414
sll0765	GO:0043436	<1e-4	sll1464	GO:0050896	<1e-4	sll1608	GO:0019752	<1e-4	slr1533	GO:0022891	<1e-4	slr0318	GO:0008152	0.3414
slr0305	GO:0044260	<1e-4	slr1068	GO:0009165	<1e-4	sgl0002	GO:0009259	<1e-4	ssl5008	GO:0016741	<1e-4	sll0688	GO:0008152	0.3414
sll5006	GO:0071496	<1e-4	slr1546	GO:0009262	<1e-4	slr1260	GO:0042455	<1e-4	sll1749	GO:0016818	<1e-4	sll1372	GO:0008152	0.3414
slr1306	GO:0034641	<1e-4	sll8001	GO:0006753	<1e-4	ssr1499	GO:0019362	<1e-4	slr0581	GO:0015293	<1e-4	slr0680	GO:0008152	0.3414
ssl1498	GO:0044271	<1e-4	sll1960	GO:0065007	<1e-4	slr1547	GO:0051171	<1e-4	sll1119	GO:0022891	<1e-4	sll0911	GO:0008152	0.3414
slr6051	GO:0071841	<1e-4	slr2060	GO:0010556	<1e-4	slr0039	GO:0019222	<1e-4	slr6051	GO:0022892	<1e-4	sll1925	GO:0008152	0.3414
sll1355	GO:0022411	<1e-4	ssr2754	GO:0072521	<1e-4	slr0912	GO:0051188	<1e-4	slr0769	GO:0016746	<1e-4	ssl0739	GO:0008152	0.3414
ssl2781	GO:0071842	<1e-4	sll0160	GO:0044255	<1e-4	sll1570	GO:0008150	<1e-4	sll0552	GO:0015399	<1e-4	slr1862	GO:0008152	0.3414
sll1606	GO:0006220	<1e-4	sll0702	GO:0009309	<1e-4	slr7092	GO:0042180	<1e-4	slr0272	GO:0042623	<1e-4	slr0232	GO:0008152	0.3414
slr1603	GO:0010468	<1e-4	ssr2551	GO:0043412	<1e-4	slr7071	GO:0080090	<1e-4	ssr0335	GO:0022892	<1e-4	slr0654	GO:0008152	0.3414
slr1546	GO:0009165	<1e-4	sll0659	GO:0051188	<1e-4	sll8035	GO:0046164	<1e-4	slr2004	GO:0008171	<1e-4	slr0272	GO:0008152	0.3414
sll2007	GO:0034660	<1e-4	slr0407	GO:0009132	<1e-4	slr0366	GO:0010468	<1e-4	sll1509	GO:0022836	<1e-4	slr1613	GO:0008152	0.3414
slr0366	GO:0051347	<1e-4	slr0053	GO:0019219	<1e-4	slr0360	GO:0072527	<1e-4	sll1119	GO:0016817	<1e-4	sll1956	GO:0008152	0.3414
slr1142	GO:0044282	<1e-4	slr7081	GO:0051188	<1e-4	slr0455	GO:0005996	<1e-4	sll0167	GO:0042623	<1e-4	sll1201	GO:0008152	0.3414
ssl7042	GO:0080090	<1e-4	slr2004	GO:0009161	<1e-4	slr0356	GO:0006793	<1e-4	slr0865	GO:0022836	<1e-4	slr1259	GO:0008152	0.3414
slr1827	GO:0072528	<1e-4	ssr2912	GO:0008150	<1e-4	slr7092	GO:0048518	<1e-4	slr1425	GO:0022857	<1e-4	sll1950	GO:0008152	0.3414
slr0554	GO:0009126	<1e-4	slr7024	GO:0048518	<1e-4	sll1477	GO:0060255	<1e-4	slr0960	GO:0015293	<1e-4	slr1383	GO:0008152	0.3414
slr1196	GO:0006732	<1e-4	slr0491	GO:0009124	<1e-4	slr5013	GO:0044282	<1e-4	ssl7074	GO:0015405	<1e-4	sll1072	GO:0008152	0.3414
sll1192	GO:0034641	<1e-4	ssl0483	GO:0046164	<1e-4	slr0642	GO:0009165	<1e-4	slr1339	GO:0016741	<1e-4	slr0455	GO:0008152	0.3414
sll1632	GO:0051246	<1e-4	ssl1255	GO:0044106	<1e-4	slr1737	GO:0033013	<1e-4	slr1307	GO:0022857	<1e-4	sll0614	GO:0008152	0.3414
ssr6048	GO:0051234	<1e-4	ssl7074	GO:0051347	<1e-4	slr1612	GO:0048518	<1e-4	sll0761	GO:0022891	<1e-4	sll0737	GO:0008152	0.3414
slr1066	GO:0009889	<1e-4	slr1699	GO:0051234	<1e-4	ssr2803	GO:0044281	<1e-4	sll1040	GO:0005342	<1e-4	sll0309	GO:0008152	0.3361
slr0784	GO:0006721	<1e-4	slr0103	GO:0055114	<1e-4	sll0810	GO:0009059	<1e-4	slr0654	GO:0016817	<1e-4	ssr3122	GO:0008152	0.3361
slr1668	GO:0006732	<1e-4	slr0919	GO:0043933	<1e-4	sll7055	GO:0009059	<1e-4	sll1509	GO:0016741	<1e-4	sll1315	GO:0008152	0.3361
sll1658	GO:0055086	<1e-4	slr6071	GO:0051246	<1e-4	sll1040	GO:0048878	<1e-4	slr1827	GO:0016817	<1e-4	sll1608	GO:0008152	0.3361
ssr0657	GO:0043933	<1e-4	slr0423	GO:0051234	<1e-4	slr6005	GO:0009892	<1e-4	slr1813	GO:0022838	<1e-4	sll1530	GO:0008152	0.3361
slr0270	GO:0009056	<1e-4	slr5024	GO:0051818	<1e-4	slr0483	GO:0019222	<1e-4	ssl7042	GO:0015399	<1e-4	slr1230	GO:0008152	0.3361
sll1763	GO:0009141	<1e-4	sll1024	GO:0009117	<1e-4	ssr6003	GO:0050896	<1e-4	sll0149	GO:0022890	<1e-4	slr0006	GO:0008152	0.3361
sll1447	GO:0009141	<1e-4	ssr1391	GO:0043412	<1e-4	slr7058	GO:0009199	<1e-4	ssl5065	GO:0015399	<1e-4	ssr2962	GO:0008152	0.3361
slr0978	GO:0072521	<1e-4	sll0861	GO:0009259	<1e-4	sll0191	GO:0051171	<1e-4	sll1601	GO:0022832	<1e-4	ssr2551	GO:0008152	0.3361
sll0625	GO:0051179	<1e-4	slr1638	GO:0034641	<1e-4	ssr3300	GO:0019751	<1e-4	sll0982	GO:0015267	<1e-4	slr1638	GO:0008152	0.3361
slr1811	GO:0009259	<1e-4	slr0517	GO:0019362	<1e-4	sll0183	GO:0080090	<1e-4	slr0038	GO:0043492	<1e-4	sll0237	GO:0008152	0.3361
sll1659	GO:0019637	<1e-4	slr1033	GO:0001932	<1e-4	sll0611	GO:0072521	<1e-4	sll7028	GO:0043167	<1e-4	ssr2439	GO:0008152	0.3361
ssl0312	GO:0009889	<1e-4	slr1880	GO:0022411	<1e-4	slr0249	GO:0072527	<1e-4	sll1660	GO:0008171	<1e-4	sll0023	GO:0008152	0.3361
slr2004	GO:0060255	<1e-4	sll0625	GO:0055114	<1e-4	ssl8008	GO:0043436	<1e-4	sll1315	GO:0016818	<1e-4	sll1658	GO:0008152	0.3361
slr1865	GO:0050801	<1e-4	ssl1417	GO:0006576	<1e-4	ssr1041	GO:0009260	<1e-4	sll0513	GO:0043167	<1e-4	sll1761	GO:0008152	0.3361
slr6021	GO:0080090	<1e-4	slr1862	GO:0051818	<1e-4	slr1568	GO:0048519	<1e-4	sll7086	GO:0043167	<1e-4	slr0392	GO:0008152	0.3361
slr1394	GO:0009259	<1e-4	slr7057	GO:0043170	<1e-4	slr0453	GO:0034641	<1e-4	sll1174	GO:0005342	<1e-4	slr1262	GO:0008152	0.3361
sll1660	GO:0071554	<1e-4	sll0414	GO:0044283	<1e-4	sll0933	GO:0044275	<1e-4	ssr1951	GO:0043492	<1e-4	sll0703	GO:0008152	0.3361
slr1918	GO:0006576	<1e-4	slr0869	GO:0009132	<1e-4	ssl0352	GO:0016052	<1e-4	slr0263	GO:0016817	<1e-4	slr1073	GO:0008152	0.3361
slr2073	GO:0046164	<1e-4	slr1807	GO:0042451	<1e-4	ssr2060	GO:0009124	<1e-4	sll1640	GO:0015399	<1e-4	sll0198	GO:0008152	0.3361
sll0939	GO:0046483	<1e-4	ssl0788	GO:0009057	<1e-4	sll0615	GO:0022607	<1e-4	slr1964	GO:0015293	<1e-4	slr1648	GO:0008152	0.3361
ssr6099	GO:0051171	<1e-4	slr7082	GO:0006066	<1e-4	slr6016	GO:0044281	<1e-4	slr1570	GO:0022857	<1e-4	sll0355	GO:0008152	0.3361
sll0804	GO:0045184	<1e-4	slr0789	GO:0051171	<1e-4	slr1613	GO:0048523	<1e-4	slr1911	GO:0016818	<1e-4	slr0770	GO:0008152	0.3361
sll0156	GO:0045184	<1e-4	sll0443	GO:0009056	<1e-4	ssr6019	GO:0006811	<1e-4	sll0062	GO:0022803	<1e-4	sll1954	GO:0008152	0.3361
sll1049	GO:0051186	<1e-4	slr1570	GO:0019219	<1e-4	slr6031	GO:0071842	<1e-4	sll1763	GO:0022857	<1e-4	sll1061	GO:0008152	0.3361

ssl7007	GO:0051188	<1e-4	sll0786	GO:0044282	<1e-4	ssr6079	GO:0051171	<1e-4	sll1526	GO:0005342	<1e-4	ssl1577	GO:0008152	0.3361
slr0980	GO:0050801	<1e-4	slr7024	GO:0050896	<1e-4	slr1203	GO:0006793	<1e-4	slr1338	GO:0022838	<1e-4	slr0386	GO:0008152	0.3361
slr0655	GO:0009394	<1e-4	sll1906	GO:0080090	<1e-4	sll0888	GO:0019751	<1e-4	slr1215	GO:0022890	<1e-4	slr2144	GO:0008152	0.3361
ssr1766	GO:0009142	<1e-4	slr1033	GO:0009142	<1e-4	slr1957	GO:0006082	<1e-4	slr0923	GO:0015075	<1e-4	sll0982	GO:0008152	0.3361
sll1853	GO:0048878	<1e-4	slr7095	GO:0052111	<1e-4	slr1752	GO:0033013	<1e-4	slr0708	GO:0022832	<1e-4	sll1273	GO:0008152	0.3177
sll0785	GO:0044260	<1e-4	sll0762	GO:0008150	<1e-4	ssl1690	GO:0048519	<1e-4	slr0725	GO:0016818	<1e-4	sll5034	GO:0008152	0.3177
sll0168	GO:0042451	<1e-4	sll0253	GO:0009987	<1e-4	slr1638	GO:0009124	<1e-4	slr1385	GO:0016741	<1e-4	slr1397	GO:0008152	0.3177
sll6053	GO:0006066	<1e-4	sll1601	GO:0043436	<1e-4	slr0913	GO:0042180	<1e-4	slr1173	GO:0005342	<1e-4	slr6004	GO:0008152	0.3177
sll1348	GO:0051716	<1e-4	slr6029	GO:0051716	<1e-4	ssr6027	GO:0031640	<1e-4	ssl5114	GO:0043492	<1e-4	sgl0001	GO:0008152	0.3177
sll5062	GO:0006163	<1e-4	ssl1792	GO:0050794	<1e-4	slr0801	GO:0001932	<1e-4	slr0157	GO:0016817	<1e-4	slr1276	GO:0008152	0.3177
sll0871	GO:0022607	<1e-4	sll0858	GO:0016054	<1e-4	sll0932	GO:0034654	<1e-4	slr1186	GO:0016746	<1e-4	sll1163	GO:0008152	0.3177
slr1789	GO:0018130	<1e-4	slr0300	GO:0031640	<1e-4	sll1164	GO:0072528	<1e-4	sll0448	GO:0043167	<1e-4	slr6008	GO:0008152	0.3177
sll0424	GO:0006163	<1e-4	sll1186	GO:0019637	<1e-4	ssl2920	GO:0044281	<1e-4	sll0811	GO:0022892	<1e-4	slr1814	GO:0008152	0.3177
slr1541	GO:0034641	<1e-4	slr0082	GO:0046164	<1e-4	slr1189	GO:0044281	<1e-4	slr0907	GO:0060089	<1e-4	slr1815	GO:0008152	0.3177
slr6047	GO:0009987	<1e-4	ssr0332	GO:0065007	<1e-4	sll1797	GO:0071842	<1e-4	slr1431	GO:0022838	<1e-4	ssl0312	GO:0008152	0.3177
slr1956	GO:0001932	<1e-4	sll7090	GO:0019219	<1e-4	slr1603	GO:0009991	<1e-4	sll1714	GO:0042623	<1e-4	slr6038	GO:0008152	0.3177
sll0281	GO:0006082	<1e-4	slr1638	GO:0006066	<1e-4	sll0625	GO:0016052	<1e-4	slr1468	GO:0043492	<1e-4	sll0553	GO:0008152	0.3177
slr0211	GO:0050789	<1e-4	sll1486	GO:0052111	<1e-4	slr1519	GO:0048523	<1e-4	sll1447	GO:0043167	<1e-4	sll1162	GO:0008152	0.3177
sll0428	GO:0072522	<1e-4	sll1004	GO:0006066	<1e-4	sll1109	GO:0006811	<1e-4	slr7082	GO:0015399	<1e-4	slr6072	GO:0008152	0.3177
sll8012	GO:0051171	<1e-4	slr0453	GO:0046164	<1e-4	slr1270	GO:0043549	<1e-4	sll0496	GO:0015291	<1e-4	slr6063	GO:0008152	0.3177
ssr1375	GO:0009144	<1e-4	sll5128	GO:0044282	<1e-4	slr1260	GO:0019752	<1e-4	ssl8028	GO:0022836	<1e-4	slr6015	GO:0008152	0.3177
slr0151	GO:0009308	<1e-4	sll0815	GO:0052111	<1e-4	slr1275	GO:0006066	<1e-4	sll0539	GO:0016462	<1e-4	slr0489	GO:0008152	0.3177
sll1191	GO:0006720	<1e-4	sll1611	GO:0071496	<1e-4	slr5077	GO:0009892	<1e-4	ssr2848	GO:0015077	<1e-4	slr1576	GO:0008152	0.3177
slr7026	GO:0044003	<1e-4	slr1431	GO:0055082	<1e-4	ssl2245	GO:0043170	<1e-4	slr6073	GO:0022836	<1e-4	slr1470	GO:0008152	0.3177
slr0755	GO:0071841	<1e-4	slr0689	GO:0071841	<1e-4	sgl0002	GO:0001932	<1e-4	sll1880	GO:0015293	<1e-4	slr7025	GO:0008152	0.3177
ssr3300	GO:0009161	<1e-4	slr6073	GO:0009394	<1e-4	sll5090	GO:0009260	<1e-4	slr0742	GO:0005342	<1e-4	slr0619	GO:0008152	0.3177
sll1698	GO:0019222	<1e-4	slr5077	GO:0072528	<1e-4	ssr6062	GO:0019219	<1e-4	ssr7036	GO:0022832	<1e-4	sll0369	GO:0008152	0.3177
ssl2814	GO:0072522	<1e-4	sll1680	GO:0009132	<1e-4	slr0060	GO:0034641	<1e-4	slr0751	GO:0015293	<1e-4	sll5130	GO:0008152	0.3177
sll1411	GO:0052188	<1e-4	sll1510	GO:0009161	<1e-4	slr0554	GO:0016054	<1e-4	sll1092	GO:0022836	<1e-4	sll0406	GO:0008152	0.3177
slr1659	GO:0051179	<1e-4	sll0208	GO:0031326	<1e-4	slr1384	GO:0055082	<1e-4	ssr0759	GO:0022892	<1e-4	sll0853	GO:0008152	0.3177
ssl2069	GO:0006082	<1e-4	sll1240	GO:0008610	<1e-4	sll0843	GO:0006721	<1e-4	ssl5096	GO:0016462	<1e-4	sll1092	GO:0008152	0.3177
sll7089	GO:0009117	<1e-4	slr0400	GO:0071840	<1e-4	slr1900	GO:0090304	<1e-4	sll0518	GO:0015399	<1e-4	slr0142	GO:0008152	0.3177
sll0996	GO:0045184	<1e-4	slr0708	GO:0048878	<1e-4	slr1178	GO:0031640	<1e-4	sll1882	GO:0016818	<1e-4	ssl3379	GO:0008152	0.3177
sll1834	GO:0055082	<1e-4	sll7028	GO:0046164	<1e-4	slr7073	GO:0042180	<1e-4	sll0793	GO:0008171	<1e-4	slr0869	GO:0008152	0.3177
slr6015	GO:0043436	<1e-4	slr1699	GO:0055082	<1e-4	ssr8047	GO:0019752	<1e-4	slr1079	GO:0003674	<1e-4	slr7011	GO:0008152	0.3177
sll0482	GO:0019222	<1e-4	sll0518	GO:0046907	<1e-4	slr1970	GO:0009991	<1e-4	sll1062	GO:0043492	<1e-4	slr1363	GO:0008152	0.3177
slr1474	GO:0046907	<1e-4	slr0619	GO:0043436	<1e-4	sll1239	GO:0043412	<1e-4	sll0525	GO:0022891	<1e-4	slr0058	GO:0008152	0.3177
slr7060	GO:0019752	<1e-4	sll0498	GO:0006631	<1e-4	ssl2138	GO:0048523	<1e-4	slr7100	GO:0022892	<1e-4	sll7066	GO:0008152	0.3177
slr0689	GO:0046394	<1e-4	slr7092	GO:0009892	<1e-4	sll1717	GO:0051716	<1e-4	ssl5100	GO:0022892	<1e-4	slr0407	GO:0008152	0.3177
sll8040	GO:0001932	<1e-4	sll0932	GO:0071554	<1e-4	slr0871	GO:0050896	<1e-4	slr1235	GO:0016462	<1e-4	sll1773	GO:0008152	0.3177
sll1511	GO:0050794	<1e-4	ssl3573	GO:0010468	<1e-4	slr0453	GO:0006220	<1e-4	sll0752	GO:0015291	<1e-4	slr7014	GO:0008152	0.3177
slr2049	GO:0072528	<1e-4	sll1355	GO:0051234	<1e-4	slr1101	GO:0072528	<1e-4	ssr0335	GO:0015399	<1e-4	sll5097	GO:0008152	0.3177
ssr3532	GO:0048523	<1e-4	slr1438	GO:0010468	<1e-4	ssr3189	GO:0042180	<1e-4	sll0994	GO:0016817	<1e-4	sll1696	GO:0008152	0.3177
sll0743	GO:0022411	<1e-4	sll0072	GO:0072521	<1e-4	sll0843	GO:0044260	<1e-4	sll1293	GO:0015399	<1e-4	slr0199	GO:0008152	0.3177
sll1123	GO:0051188	<1e-4	sll0478	GO:0046128	<1e-4	slr5021	GO:0006163	<1e-4	sll1510	GO:0008171	<1e-4	slr1407	GO:0008152	0.3177
sll1526	GO:0019222	<1e-4	slr0725	GO:0051716	<1e-4	sll1609	GO:0019752	<1e-4	slr7101	GO:0022892	<1e-4	sll1155	GO:0008152	0.3177
slr7058	GO:0045184	<1e-4	ssr5121	GO:0031326	<1e-4	sll1265	GO:0009263	<1e-4	sll0309	GO:0022836	<1e-4	slr1464	GO:0008152	0.3177
sll1388	GO:0046483	<1e-4	sll0586	GO:0009260	<1e-4	slr0517	GO:0055114	<1e-4	ssl5108	GO:0016817	<1e-4	ssl1972	GO:0008152	0.3177
slr1263	GO:0044248	<1e-4	sll0445	GO:0009132	<1e-4	ssl5008	GO:0055082	<1e-4	sll1352	GO:0003674	<1e-4	sll1863	GO:0008152	0.3177
sll1632	GO:0046394	<1e-4	slr0980	GO:0009144	<1e-4	sll0272	GO:0043170	<1e-4	ssr2317	GO:0016746	<1e-4	ssr2611	GO:0008152	0.3177
ssl5064	GO:0019219	<1e-4	sll1201	GO:0016053	<1e-4	sll0499	GO:0009260	<1e-4	slr5119	GO:0016462	<1e-4	ssl7046	GO:0008152	0.3177
sll0263	GO:0019752	<1e-4	sll0875	GO:0055082	<1e-4	ssl0739	GO:0044281	<1e-4	slr0948	GO:0022890	<1e-4	sll0444	GO:0008152	0.3177
sll1022	GO:0051716	<1e-4	sll0847	GO:0080090	<1e-4	slr1852	GO:0043549	<1e-4	sll1717	GO:0022836	<1e-4	sll0761	GO:0008152	0.3177
slr1547	GO:0009987	<1e-4	ssr3570	GO:0043436	<1e-4	sll0243	GO:0042455	<1e-4	slr1927	GO:0016741	<1e-4	slr0059	GO:0008152	0.3177

slr8022	GO:0006720	<1e-4	slr5012	GO:0006631	<1e-4	sll0498	GO:0051234	<1e-4	slr0038	GO:0022838	<1e-4	sll0886	GO:0008152	0.3177
slr0442	GO:0006766	<1e-4	slr0243	GO:0009123	<1e-4	sll0994	GO:0046164	<1e-4	sll0335	GO:0022892	<1e-4	sll1797	GO:0008152	0.3177
slr0058	GO:0051246	<1e-4	slr1068	GO:0051818	<1e-4	slr1122	GO:0044260	<1e-4	ssr2317	GO:0022891	<1e-4	slr0569	GO:0008152	0.3177
slr2011	GO:0051818	<1e-4	slr0207	GO:0009126	<1e-4	slr0318	GO:0044248	<1e-4	sll7090	GO:0015267	<1e-4	slr1920	GO:0008152	0.3177
slr0765	GO:0019222	<1e-4	slr1162	GO:0009144	<1e-4	slr1998	GO:0009150	<1e-4	slr0912	GO:0016746	<1e-4	sll0293	GO:0008152	0.3177
slr0142	GO:0009117	<1e-4	sll0280	GO:0044282	<1e-4	slr1778	GO:0009893	<1e-4	ssl1300	GO:0022836	<1e-4	sll1447	GO:0008152	0.3177
sll1486	GO:0016053	<1e-4	ssr2803	GO:0051246	<1e-4	sll0630	GO:0050789	<1e-4	sll1749	GO:0022836	<1e-4	slr0505	GO:0008152	0.3177
sll0659	GO:0009142	<1e-4	slr7013	GO:0055086	<1e-4	sll0350	GO:0019751	<1e-4	sll0355	GO:0003674	<1e-4	slr1069	GO:0008152	0.3177
sll1254	GO:0065007	<1e-4	sll0932	GO:0034641	<1e-4	slr1959	GO:0034654	<1e-4	slr1958	GO:0005342	<1e-4	sll0751	GO:0008152	0.3177
slr1288	GO:0046907	<1e-4	slr6103	GO:0006733	<1e-4	sll1632	GO:0048518	<1e-4	sll7066	GO:0015077	<1e-4	sll0397	GO:0008152	0.3177
slr7023	GO:0048522	<1e-4	sll1414	GO:0034641	<1e-4	sll0272	GO:0019752	<1e-4	slr1195	GO:0022892	<1e-4	slr1657	GO:0008152	0.3177
slr0468	GO:0006733	<1e-4	ssr6003	GO:0010556	<1e-4	slr6039	GO:0006163	<1e-4	sll0183	GO:0022890	<1e-4	sll0448	GO:0008152	0.3177
slr0442	GO:0006793	<1e-4	slr0360	GO:0071840	<1e-4	slr1240	GO:0009126	<1e-4	sll1191	GO:0016462	<1e-4	ssr6085	GO:0008152	0.3177
slr1572	GO:0009126	<1e-4	slr6094	GO:0048523	<1e-4	sll1359	GO:0006766	<1e-4	sll7050	GO:0043167	<1e-4	ssl5098	GO:0008152	0.3177
sll7066	GO:0043412	<1e-4	sll0815	GO:0051716	<1e-4	sll0360	GO:0071496	<1e-4	slr0959	GO:0015291	<1e-4	sll1123	GO:0008152	0.3177
sll0218	GO:0006091	<1e-4	slr1394	GO:0072521	<1e-4	slr1189	GO:0019222	<1e-4	ssl0242	GO:0022832	<1e-4	sll1265	GO:0008152	0.3177
slr0458	GO:0043648	<1e-4	slr0049	GO:0009150	<1e-4	ssl1918	GO:0006732	<1e-4	sll1783	GO:0022838	<1e-4	sll1485	GO:0008152	0.3177
slr0981	GO:0009308	<1e-4	sll0497	GO:0046394	<1e-4	slr1493	GO:0009144	<1e-4	sll0658	GO:0016462	<1e-4	slr0065	GO:0008152	0.3177
sll0149	GO:0009987	<1e-4	sll1262	GO:0006732	<1e-4	ssr2142	GO:0051716	<1e-4	ssr3467	GO:0016741	<1e-4	sll1109	GO:0008152	0.3177
slr0575	GO:0009889	<1e-4	slr1438	GO:0009892	<1e-4	sll1934	GO:0042451	<1e-4	sll0147	GO:0016462	<1e-4	ssl5095	GO:0008152	0.3177
ssl1498	GO:0072524	<1e-4	sll1681	GO:0050794	<1e-4	slr0404	GO:0006753	<1e-4	slr0479	GO:0022891	<1e-4	ssr6079	GO:0008152	0.3177
sll0230	GO:0019752	<1e-4	slr1932	GO:0009259	<1e-4	slr6051	GO:0051246	<1e-4	slr1544	GO:0022832	<1e-4	ssl1004	GO:0008152	0.3177
sll8035	GO:0016053	<1e-4	sll1737	GO:0009150	<1e-4	sll1036	GO:0046128	<1e-4	slr1353	GO:0022857	<1e-4	slr6013	GO:0008152	0.3177
sll0098	GO:0019751	<1e-4	slr6007	GO:0006733	<1e-4	sll1765	GO:0008150	<1e-4	slr0149	GO:0060089	<1e-4	ssr6048	GO:0008152	0.3177
ssr3572	GO:0008150	<1e-4	sll0786	GO:0048518	<1e-4	slr0784	GO:0080090	<1e-4	ssr2802	GO:0043167	<1e-4	sll5047	GO:0008152	0.3177
slr1895	GO:0044275	<1e-4	slr1179	GO:0001932	<1e-4	sll1359	GO:0009056	<1e-4	ssl0294	GO:0022857	<1e-4	slr7016	GO:0008152	0.3177
sll1509	GO:0031326	<1e-4	sll1912	GO:0009199	<1e-4	ssl5103	GO:0009987	<1e-4	slr0264	GO:0016818	<1e-4	slr5116	GO:0008152	0.3177
sll0249	GO:0065007	<1e-4	ssl5100	GO:0009199	<1e-4	sll1089	GO:0009308	<1e-4	slr1471	GO:0015077	<1e-4	ssr2554	GO:0008152	0.3177
sll1265	GO:0071840	<1e-4	sll0596	GO:0006793	<1e-4	sll0436	GO:0006163	<1e-4	ssr3189	GO:0060089	<1e-4	slr7081	GO:0008152	0.3177
sll1217	GO:0009123	<1e-4	ssl2971	GO:0034654	<1e-4	slr0006	GO:0043933	<1e-4	slr1468	GO:0022890	<1e-4	slr1863	GO:0008152	0.3177
sll8004	GO:0009308	<1e-4	slr1576	GO:0055086	<1e-4	slr0582	GO:0009056	<1e-4	slr1611	GO:0008171	<1e-4	slr1378	GO:0008152	0.3177
sll0525	GO:0016054	<1e-4	slr2105	GO:0044106	<1e-4	sll5032	GO:0009161	<1e-4	slr7092	GO:0015405	<1e-4	sll0102	GO:0008152	0.3177
slr0204	GO:0048522	<1e-4	slr1189	GO:0043648	<1e-4	sll7078	GO:0051818	<1e-4	sll0321	GO:0022836	<1e-4	slr6067	GO:0008152	0.3177
sll0376	GO:0009056	<1e-4	slr1513	GO:0009161	<1e-4	sll0314	GO:0050789	<1e-4	slr0479	GO:0015291	<1e-4	sll0241	GO:0008152	0.3177
slr0179	GO:0006766	<1e-4	sll0297	GO:0051186	<1e-4	ssr7084	GO:0071842	<1e-4	slr1431	GO:0043492	<1e-4	ssr1951	GO:0008152	0.3177
slr1636	GO:0009199	<1e-4	slr1449	GO:0065007	<1e-4	ssr2554	GO:0005996	<1e-4	slr1623	GO:0022836	<1e-4	sll1880	GO:0008152	0.3177
sll0669	GO:0046128	<1e-4	ssl2064	GO:0044260	<1e-4	slr1419	GO:0044255	<1e-4	ssr3410	GO:0022836	<1e-4	slr1998	GO:0008152	0.3177
sll1162	GO:0006753	<1e-4	sll0230	GO:0034660	<1e-4	sll0487	GO:0006082	<1e-4	slr0815	GO:0022803	<1e-4	slr1306	GO:0008152	0.3177
sll0098	GO:0009161	<1e-4	ssr2711	GO:0006091	<1e-4	sll5128	GO:0050794	<1e-4	ssr1375	GO:0022836	<1e-4	ssl2148	GO:0008152	0.3177
ssr7035	GO:0072527	<1e-4	slr7025	GO:0022607	<1e-4	sll1601	GO:0044275	<1e-4	sll0325	GO:0022836	<1e-4	slr1340	GO:0008152	0.3177
slr1704	GO:0042451	<1e-4	ssl8008	GO:0006091	<1e-4	slr6015	GO:0006766	<1e-4	ssl7038	GO:0008171	<1e-4	ssl2245	GO:0008152	0.3177
slr0514	GO:0009150	<1e-4	slr1429	GO:0033013	<1e-4	ssr0759	GO:0006163	<1e-4	slr1095	GO:0022892	<1e-4	sll0400	GO:0008152	0.3177
slr0596	GO:0071554	<1e-4	sll0688	GO:0009126	<1e-4	slr1342	GO:0006066	<1e-4	sll0623	GO:0022838	<1e-4	slr1813	GO:0008152	0.3177
slr0483	GO:0006732	<1e-4	slr1847	GO:0046164	<1e-4	slr1618	GO:0044271	<1e-4	sll0858	GO:0022891	<1e-4	sll1509	GO:0008152	0.3177
slr1623	GO:0046394	<1e-4	sll1240	GO:0009262	<1e-4	slr1107	GO:0006721	<1e-4	slr6005	GO:0005342	<1e-4	slr1097	GO:0008152	0.3177
ssr5092	GO:0009056	<1e-4	sll1062	GO:0050794	<1e-4	slr1519	GO:0006753	<1e-4	sll1061	GO:0022803	<1e-4	ssl5031	GO:0008152	0.3177
sll0269	GO:0048518	<1e-4	ssl5006	GO:0044283	<1e-4	slr0937	GO:0031323	<1e-4	slr2025	GO:0022891	<1e-4	sll0481	GO:0008152	0.3177
ssl0294	GO:0043412	<1e-4	sll1162	GO:0050896	<1e-4	sll1528	GO:0019219	<1e-4	slr6015	GO:0016746	<1e-4	sll1606	GO:0008152	0.3177
slr0376	GO:0080090	<1e-4	slr1301	GO:0080090	<1e-4	sll1934	GO:0019222	<1e-4	sll1036	GO:0022891	<1e-4	sll1504	GO:0008152	0.3177
slr0589	GO:0046164	<1e-4	sll1934	GO:0009132	<1e-4	sll0068	GO:0046164	<1e-4	slr1789	GO:0022836	<1e-4	slr5119	GO:0008152	0.3177
sll1250	GO:0019362	<1e-4	sll0283	GO:0043412	<1e-4	slr1557	GO:0052111	<1e-4	slr1186	GO:0022891	<1e-4	sll1926	GO:0003824	0.2837
ssr2318	GO:0051818	<1e-4	slr1647	GO:0009117	<1e-4	ssr6086	GO:0072527	<1e-4	slr0184	GO:0042623	<1e-4	slr6088	GO:0003824	0.2837
sll1052	GO:0009144	<1e-4	ssl3573	GO:0051347	<1e-4	slr1546	GO:0009199	<1e-4	slr1591	GO:0015399	<1e-4	sll7078	GO:0003824	0.2837
sll0335	GO:0009165	<1e-4	slr1628	GO:0043549	<1e-4	slr0157	GO:0051818	<1e-4	ssr6003	GO:0005342	<1e-4	ssr2802	GO:0003824	0.2837

slr0935	GO:0009144	<1e-4	ssr5121	GO:0019222	<1e-4	slr1273	GO:0072521	<1e-4	sll0553	GO:0022892	<1e-4	slr0459	GO:0003824	0.2837
sll0162	GO:0018130	<1e-4	ssl1255	GO:0019362	<1e-4	ssr0657	GO:0048878	<1e-4	sll1123	GO:0015077	<1e-4	slr1537	GO:0003824	0.2837
slr0521	GO:0009263	<1e-4	slr1704	GO:0006082	<1e-4	slr6103	GO:0009057	<1e-4	ssr7079	GO:0022892	<1e-4	ssr0761	GO:0003824	0.2837
sll1737	GO:0009142	<1e-4	sll6053	GO:0044255	<1e-4	slr0712	GO:0006720	<1e-4	sll1378	GO:0005342	<1e-4	ssl6061	GO:0003824	0.2837
slr2084	GO:0009056	<1e-4	sll5003	GO:0022411	<1e-4	slr1614	GO:0044248	<1e-4	sll0625	GO:0015077	<1e-4	slr0184	GO:0003824	0.2837
sll0167	GO:0043549	<1e-4	slr6103	GO:0043412	<1e-4	slr0270	GO:0043170	<1e-4	ssr1528	GO:0015399	<1e-4	sll0294	GO:0003824	0.2837
slr0818	GO:0006091	<1e-4	sll1350	GO:0043933	<1e-4	slr0887	GO:0071840	<1e-4	ssr2142	GO:0003674	<1e-4	sll0691	GO:0003824	0.2837
sll0572	GO:0045184	<1e-4	sll0982	GO:0044003	<1e-4	sll0602	GO:0072528	<1e-4	ssl2420	GO:0043167	<1e-4	sll1911	GO:0003824	0.2837
sll0487	GO:0008150	<1e-4	sll1002	GO:0031326	<1e-4	ssr2755	GO:0009262	<1e-4	sll1132	GO:0022803	<1e-4	slr0967	GO:0003824	0.2837
sll1785	GO:0052111	<1e-4	slr0725	GO:0009165	<1e-4	slr5101	GO:0044275	<1e-4	sll1960	GO:0060089	<1e-4	slr7091	GO:0003824	0.2837
sll0933	GO:0042180	<1e-4	slr2120	GO:0048523	<1e-4	ssr7084	GO:0019219	<1e-4	ssr7036	GO:0043167	<1e-4	slr1173	GO:0003824	0.2837
ssr3570	GO:0019222	<1e-4	sll1691	GO:0050794	<1e-4	slr1162	GO:0010556	<1e-4	ssr2422	GO:0015077	<1e-4	ssl5025	GO:0003824	0.2837
slr1737	GO:0071554	<1e-4	ssr1407	GO:0044003	<1e-4	ssl1972	GO:0031323	<1e-4	sll0156	GO:0015291	<1e-4	slr6033	GO:0003824	0.2837
slr2052	GO:0044260	<1e-4	slr8014	GO:0080090	<1e-4	slr1519	GO:0055086	<1e-4	slr1266	GO:0043492	<1e-4	slr0588	GO:0003824	0.2837
slr1906	GO:0046483	<1e-4	ssl5100	GO:0044255	<1e-4	sll1072	GO:0051716	<1e-4	sll1252	GO:0060089	<1e-4	sll8040	GO:0003824	0.2837
sll0497	GO:0006732	<1e-4	sll1866	GO:0090304	<1e-4	ssl8005	GO:0009124	<1e-4	slr1143	GO:0003674	<1e-4	sll7069	GO:0003824	0.2837
ssr3129	GO:0009059	<1e-4	sll1306	GO:0009056	<1e-4	slr0708	GO:0008150	<1e-4	slr0147	GO:0005342	<1e-4	ssr1041	GO:0003824	0.2837
ssr1391	GO:0006811	<1e-4	slr2119	GO:0006091	<1e-4	ssr5011	GO:0016053	<1e-4	sll8011	GO:0016462	<1e-4	slr0313	GO:0003824	0.2837
sll1021	GO:0044106	<1e-4	slr5016	GO:0060255	<1e-4	ssr2318	GO:0019751	<1e-4	sll0394	GO:0016746	<1e-4	sll7031	GO:0003824	0.2837
sll1188	GO:0050896	<1e-4	sll0309	GO:0044281	<1e-4	slr1444	GO:0043648	<1e-4	sll5063	GO:0022857	<1e-4	slr8021	GO:0003824	0.2837
slr1495	GO:0009893	<1e-4	sll1722	GO:0006631	<1e-4	slr1616	GO:0019362	<1e-4	sll1388	GO:0015293	<1e-4	ssr1425	GO:0003824	0.2837
ssl7022	GO:0022411	<1e-4	ssl0352	GO:0045184	<1e-4	sll0736	GO:0006811	<1e-4	slr1470	GO:0015293	<1e-4	ssr8013	GO:0003824	0.2837
sll7055	GO:0019219	<1e-4	slr1290	GO:0010556	<1e-4	sll0167	GO:0048519	<1e-4	slr0204	GO:0005342	<1e-4	slr1101	GO:0003824	0.2837
sll0085	GO:0090304	<1e-4	sll0414	GO:0071554	<1e-4	slr1977	GO:0044003	<1e-4	sll1396	GO:0022890	<1e-4	slr0285	GO:0003824	0.2837
slr0978	GO:0009199	<1e-4	sll5028	GO:0044248	<1e-4	sll5034	GO:0006753	<1e-4	sll1954	GO:0022803	<1e-4	slr1174	GO:0003824	0.2837
slr1407	GO:0019362	<1e-4	slr0815	GO:0019222	<1e-4	sll7086	GO:0006720	<1e-4	sll6052	GO:0015291	<1e-4	ssr8047	GO:0003824	0.2837
slr7071	GO:0072521	<1e-4	slr1442	GO:0009259	<1e-4	ssl1417	GO:0009142	<1e-4	sll1318	GO:0022838	<1e-4	slr0397	GO:0003824	0.2837
slr5101	GO:0042455	<1e-4	sll1400	GO:0051716	<1e-4	slr1419	GO:0048518	<1e-4	sll1191	GO:0003674	<1e-4	sll0069	GO:0003824	0.2837
slr2105	GO:0009260	<1e-4	sll8001	GO:0006793	<1e-4	sll0930	GO:0051179	<1e-4	ssr3570	GO:0022892	<1e-4	sll0172	GO:0003824	0.2837
slr1657	GO:0042180	<1e-4	sll7062	GO:0044281	<1e-4	sll1477	GO:0046483	<1e-4	ssl5113	GO:0015075	<1e-4	sll0479	GO:0003824	0.2837
sll0751	GO:0006066	<1e-4	slr6051	GO:0033013	<1e-4	sll1611	GO:0044260	<1e-4	slr0552	GO:0016818	<1e-4	slr6009	GO:0003824	0.2837
sll0482	GO:0019362	<1e-4	slr6008	GO:0031326	<1e-4	slr2121	GO:0008150	<1e-4	slr1070	GO:0022836	<1e-4	sll1233	GO:0003824	0.2837
slr0801	GO:0009987	<1e-4	slr1363	GO:0055114	<1e-4	slr1431	GO:0006725	<1e-4	sll8040	GO:0015399	<1e-4	slr1670	GO:0003824	0.2837
sll0499	GO:0043412	<1e-4	slr1577	GO:0071496	<1e-4	slr1854	GO:0071842	<1e-4	sll1414	GO:0022832	<1e-4	sll5004	GO:0003824	0.2837
sll1119	GO:0044260	<1e-4	slr1809	GO:0044260	<1e-4	slr0913	GO:0008610	<1e-4	sll1715	GO:0043492	<1e-4	ssl0410	GO:0003824	0.2837
slr1391	GO:0048518	<1e-4	ssl1972	GO:0022411	<1e-4	sll0503	GO:0009262	<1e-4	slr1573	GO:0005342	<1e-4	ssl2064	GO:0003824	0.2837
sll0282	GO:0006811	<1e-4	sll0269	GO:0016053	<1e-4	slr2027	GO:0050801	<1e-4	slr0521	GO:0022892	<1e-4	ssr1558	GO:0003824	0.2837
slr6005	GO:0006082	<1e-4	ssl2471	GO:0051171	<1e-4	sll1505	GO:0046164	<1e-4	sll0939	GO:0042623	<1e-4	slr2070	GO:0003824	0.2837
slr1397	GO:0009161	<1e-4	slr1862	GO:0044281	<1e-4	sll0980	GO:0009394	<1e-4	sll1862	GO:0016817	<1e-4	sll1188	GO:0003824	0.2837
slr0654	GO:0055114	<1e-4	slr2144	GO:0009150	<1e-4	ssl7045	GO:0043412	<1e-4	ssl1972	GO:0016462	<1e-4	slr1886	GO:0003824	0.2837
ssl3549	GO:0046394	<1e-4	sll0241	GO:0046394	<1e-4	slr0299	GO:0051716	<1e-4	slr1864	GO:0022890	<1e-4	sll1573	GO:0003824	0.2837
ssl1263	GO:0051186	<1e-4	slr1122	GO:0048523	<1e-4	slr0498	GO:0009987	<1e-4	sll5090	GO:0005342	<1e-4	ssr5106	GO:0003824	0.2837
slr0148	GO:0031323	<1e-4	slr0668	GO:0072524	<1e-4	sll0703	GO:0051188	<1e-4	slr1851	GO:0015399	<1e-4	sll0327	GO:0003824	0.2837
slr0489	GO:0051171	<1e-4	sll0372	GO:0006631	<1e-4	sll1504	GO:0022607	<1e-4	slr0360	GO:0042623	<1e-4	slr0345	GO:0003824	0.2837
slr1495	GO:0005996	<1e-4	sll0932	GO:0001932	<1e-4	slr1923	GO:0046128	<1e-4	sll1304	GO:0016741	<1e-4	slr1935	GO:0003824	0.2837
sll8033	GO:0006811	<1e-4	slr1472	GO:0071840	<1e-4	ssl2069	GO:0060255	<1e-4	slr1493	GO:0022832	<1e-4	ssl2971	GO:0003824	0.2837
slr0482	GO:0006766	<1e-4	slr7025	GO:0008150	<1e-4	sll0310	GO:0051347	<1e-4	sll1717	GO:0016818	<1e-4	sll1024	GO:0003824	0.2837
slr6009	GO:0045184	<1e-4	sll0837	GO:0044282	<1e-4	slr1957	GO:0050801	<1e-4	slr0294	GO:0022891	<1e-4	ssl2162	GO:0003824	0.2837
slr1258	GO:0043648	<1e-4	slr1396	GO:0006091	<1e-4	sll8011	GO:0009309	<1e-4	ssr2972	GO:0042623	<1e-4	slr0106	GO:0003824	0.2837
sll0024	GO:0009059	<1e-4	slr7095	GO:0006066	<1e-4	ssr0536	GO:0051171	<1e-4	sll1510	GO:0015399	<1e-4	ssr6062	GO:0003824	0.2837
slr0670	GO:0071841	<1e-4	sll5026	GO:0009132	<1e-4	sll1095	GO:0043436	<1e-4	slr1957	GO:0005342	<1e-4	slr5101	GO:0003824	0.2837
ssr2422	GO:0009057	<1e-4	sll0282	GO:0051818	<1e-4	slr1472	GO:0043933	<1e-4	sll0939	GO:0016818	<1e-4	slr0326	GO:0003824	0.2837
slr1340	GO:0016052	<1e-4	slr1851	GO:0050801	<1e-4	slr1339	GO:0051716	<1e-4	slr1438	GO:0015293	<1e-4	slr1752	GO:0003824	0.2837
slr5111	GO:0006766	<1e-4	slr2070	GO:0009394	<1e-4	slr7057	GO:0050801	<1e-4	slr0398	GO:0015291	<1e-4	sll1979	GO:0003824	0.2837

sll0424	GO:0019222	<1e-4	ssl5100	GO:0008150	<1e-4	sll0281	GO:0072528	<1e-4	sll0237	GO:0043167	<1e-4	slr6091	GO:0003824	0.2837
sll1530	GO:0010556	<1e-4	sll0524	GO:0006576	<1e-4	ssr6020	GO:0071554	<1e-4	sll1053	GO:0015293	<1e-4	sll1285	GO:0003824	0.2837
sll2013	GO:0046483	<1e-4	slr1681	GO:0051179	<1e-4	ssl3383	GO:0050789	<1e-4	slr1306	GO:0022836	<1e-4	slr5126	GO:0003824	0.2837
slr1095	GO:0055082	<1e-4	slr1618	GO:0060255	<1e-4	ssl5027	GO:0022411	<1e-4	slr6065	GO:0015405	<1e-4	slr0516	GO:0003824	0.2837
sll1486	GO:0006811	<1e-4	slr1618	GO:0080090	<1e-4	sll7090	GO:0046128	<1e-4	ssl3451	GO:0022832	<1e-4	slr6016	GO:0003824	0.2837
slr0709	GO:0009259	<1e-4	slr1880	GO:0042455	<1e-4	sll1241	GO:0006082	<1e-4	slr1097	GO:0022836	<1e-4	ssr0759	GO:0003824	0.2837
slr1365	GO:0022607	<1e-4	slr1338	GO:0019362	<1e-4	ssl0352	GO:0019362	<1e-4	slr0476	GO:0042623	<1e-4	slr0689	GO:0003824	0.2837
slr2005	GO:0042180	<1e-4	ssr6079	GO:0019222	<1e-4	slr7091	GO:0043170	<1e-4	ssl2733	GO:0003674	<1e-4	sll5063	GO:0003824	0.2837
ssr2201	GO:0043412	<1e-4	slr7082	GO:0048519	<1e-4	sll0181	GO:0009165	<1e-4	slr0602	GO:0015405	<1e-4	ssr5092	GO:0003824	0.2837
slr1906	GO:0072524	<1e-4	sll1764	GO:0080090	<1e-4	slr0816	GO:0009150	<1e-4	ssl5091	GO:0016818	<1e-4	sll0905	GO:0003824	0.2837
sll1201	GO:0009124	<1e-4	slr1778	GO:0048523	<1e-4	slr0962	GO:0009263	<1e-4	slr0333	GO:0008171	<1e-4	slr7060	GO:0003824	0.2837
sll1390	GO:0009056	<1e-4	slr1537	GO:0044260	<1e-4	sll1132	GO:0055114	<1e-4	slr1047	GO:0022836	<1e-4	sll5028	GO:0003824	0.2837
sll0932	GO:0042180	<1e-4	ssl8005	GO:0019222	<1e-4	sll0981	GO:0019222	<1e-4	sll1652	GO:0022890	<1e-4	ssl5103	GO:0003824	0.2837
ssl0467	GO:0006082	<1e-4	sll1315	GO:0043436	<1e-4	slr1441	GO:0044260	<1e-4	slr1207	GO:0015077	<1e-4	slr0613	GO:0003824	0.2837
slr1470	GO:0019362	<1e-4	slr0637	GO:0050789	<1e-4	ssr0692	GO:0009259	<1e-4	sll0602	GO:0015291	<1e-4	ssl3549	GO:0003824	0.2837
slr6068	GO:0019637	<1e-4	slr2117	GO:0019637	<1e-4	sll0931	GO:0034654	<1e-4	ssl2065	GO:0008171	<1e-4	slr1425	GO:0003824	0.2837
ssl5025	GO:0048523	<1e-4	slr5012	GO:0048878	<1e-4	slr6091	GO:0044271	<1e-4	sll0283	GO:0043492	<1e-4	sll2011	GO:0003824	0.2837
slr1880	GO:0019222	<1e-4	slr1406	GO:0042180	<1e-4	slr1083	GO:0031323	<1e-4	sll1783	GO:0022892	<1e-4	ssl1378	GO:0003824	0.2837
sll0584	GO:0055114	<1e-4	sll1054	GO:0009126	<1e-4	ssl1690	GO:0008610	<1e-4	slr1407	GO:0015399	<1e-4	sll1254	GO:0003824	0.2837
ssr3402	GO:0043436	<1e-4	sll0760	GO:0043170	<1e-4	slr6039	GO:0065007	<1e-4	ssl5008	GO:0022857	<1e-4	slr0813	GO:0003824	0.2837
slr1940	GO:0009889	<1e-4	slr0491	GO:0009889	<1e-4	slr6044	GO:0009117	<1e-4	sll0994	GO:0022803	<1e-4	sll1151	GO:0003824	0.2837
sll7033	GO:0051171	<1e-4	slr0270	GO:0050794	<1e-4	ssr5019	GO:0022411	<1e-4	slr0442	GO:0042623	<1e-4	slr1816	GO:0003824	0.2837
sll0553	GO:0071841	<1e-4	sll1654	GO:0043933	<1e-4	ssr3570	GO:0046907	<1e-4	slr1917	GO:0015075	<1e-4	slr0755	GO:0003824	0.2837
ssl2065	GO:0006631	<1e-4	sll1965	GO:0009141	<1e-4	slr1644	GO:0071840	<1e-4	ssr0102	GO:0016741	<1e-4	ssr3467	GO:0003824	0.2837
sll0274	GO:0019752	<1e-4	slr0751	GO:0043648	<1e-4	ssl2069	GO:0006576	<1e-4	ssr1155	GO:0016817	<1e-4	sll7033	GO:0003824	0.2837
ssr0657	GO:0055082	<1e-4	slr7102	GO:0034654	<1e-4	slr2080	GO:0018130	<1e-4	slr1273	GO:0043492	<1e-4	sll0405	GO:0003824	0.2837
slr0941	GO:0006811	<1e-4	sll1956	GO:0019222	<1e-4	ssl5103	GO:0006066	<1e-4	sll1022	GO:0003674	<1e-4	slr6051	GO:0003824	0.2837
sll0406	GO:0034654	<1e-4	slr0262	GO:0006766	<1e-4	sll1541	GO:0044003	<1e-4	slr0667	GO:0022803	<1e-4	slr2060	GO:0003824	0.2837
slr1101	GO:0009263	<1e-4	ssl5007	GO:0042451	<1e-4	slr0386	GO:0009057	<1e-4	sll8012	GO:0022890	<1e-4	ssl5108	GO:0003824	0.2837
ssl2064	GO:0006753	<1e-4	sll1873	GO:0046164	<1e-4	slr0423	GO:0009117	<1e-4	slr1918	GO:0003674	<1e-4	ssl5113	GO:0003824	0.2837
ssr6085	GO:0080090	<1e-4	sll5097	GO:0060255	<1e-4	ssl2069	GO:0034641	<1e-4	ssr2201	GO:0016746	<1e-4	slr7099	GO:0003824	0.2837
slr0398	GO:0019637	<1e-4	slr1188	GO:0005996	<1e-4	slr0921	GO:0006753	<1e-4	slr0294	GO:0008171	<1e-4	slr1681	GO:0003824	0.2837
sll1022	GO:0009059	<1e-4	ssr5011	GO:0050896	<1e-4	slr6073	GO:0006793	<1e-4	slr2119	GO:0022832	<1e-4	sll0253	GO:0003824	0.2837
sll1131	GO:0050896	<1e-4	slr1547	GO:0006163	<1e-4	sll6054	GO:0009394	<1e-4	slr0217	GO:0015075	<1e-4	slr5012	GO:0003824	0.2837
sll0702	GO:0044282	<1e-4	sll1022	GO:0052111	<1e-4	sll0024	GO:0009124	<1e-4	slr2000	GO:0016817	<1e-4	slr1513	GO:0003824	0.2837
slr2110	GO:0043549	<1e-4	slr1917	GO:0051347	<1e-4	slr7096	GO:0044255	<1e-4	slr1507	GO:0022832	<1e-4	sll0062	GO:0003824	0.2837
slr1262	GO:0051179	<1e-4	sll0294	GO:0080090	<1e-4	slr0815	GO:0019751	<1e-4	ssl2064	GO:0003674	<1e-4	sll0839	GO:0003824	0.2837
sll0269	GO:0006082	<1e-4	sll1250	GO:0043549	<1e-4	sll1166	GO:0006066	<1e-4	slr1417	GO:0005342	<1e-4	slr0082	GO:0003824	0.2837
slr1407	GO:0006082	<1e-4	slr1813	GO:0051188	<1e-4	sll0545	GO:0019752	<1e-4	sll1834	GO:0060089	<1e-4	sll1049	GO:0003824	0.2837
sll0871	GO:0018130	<1e-4	sll0442	GO:0006811	<1e-4	slr0935	GO:0044282	<1e-4	sll0444	GO:0005342	<1e-4	sll0157	GO:0003824	0.2837
sll0539	GO:0009263	<1e-4	sll0837	GO:0034660	<1e-4	sll0997	GO:0051179	<1e-4	slr6068	GO:0015293	<1e-4	slr1170	GO:0003824	0.2837
ssr8013	GO:0006082	<1e-4	slr5126	GO:0071554	<1e-4	slr1774	GO:0046907	<1e-4	slr0503	GO:0015075	<1e-4	sll0670	GO:0003824	0.2837
slr0241	GO:0006733	<1e-4	sll1414	GO:0009150	<1e-4	sll0376	GO:0009057	<1e-4	ssl5096	GO:0016818	<1e-4	slr5077	GO:0003824	0.2837
slr1196	GO:0043648	<1e-4	slr0489	GO:0050794	<1e-4	sll0781	GO:0019219	<1e-4	slr1773	GO:0003674	<1e-4	sll7090	GO:0003824	0.2837
sll0930	GO:0072522	<1e-4	slr0111	GO:0019637	<1e-4	ssr3300	GO:0044271	<1e-4	sll1155	GO:0022803	<1e-4	slr7073	GO:0003824	0.2837
sll1192	GO:0042180	<1e-4	sll1414	GO:0052188	<1e-4	sll0563	GO:0034654	<1e-4	ssr2009	GO:0015075	<1e-4	sll0098	GO:0003824	0.2837
slr7081	GO:0006753	<1e-4	slr7098	GO:0046164	<1e-4	sll1698	GO:0009142	<1e-4	sll0176	GO:0015293	<1e-4	sll1321	GO:0003824	0.2837
slr1143	GO:0071496	<1e-4	sll0656	GO:0050801	<1e-4	sll1530	GO:0006163	<1e-4	slr1315	GO:0015399	<1e-4	slr2012	GO:0003824	0.2837
ssl5027	GO:0010556	<1e-4	slr1864	GO:0034660	<1e-4	sll0069	GO:0010468	<1e-4	slr1415	GO:0022832	<1e-4	ssl7007	GO:0003824	0.2837
sll1785	GO:0019219	<1e-4	slr1699	GO:0052111	<1e-4	slr0729	GO:0048518	<1e-4	slr1667	GO:0022838	<1e-4	slr6068	GO:0003824	0.2837
ssl2920	GO:0006163	<1e-4	slr0876	GO:0006733	<1e-4	ssl0242	GO:0072528	<1e-4	sll0525	GO:0022892	<1e-4	slr0590	GO:0003824	0.2837
slr1789	GO:0043412	<1e-4	ssr6020	GO:0044003	<1e-4	slr7059	GO:0046394	<1e-4	slr7016	GO:0022838	<1e-4	slr2027	GO:0003824	0.2837
slr7098	GO:0072527	<1e-4	slr0852	GO:0008150	<1e-4	slr1702	GO:0009161	<1e-4	slr6073	GO:0043167	<1e-4	sll1702	GO:0003824	0.2837
slr1895	GO:0043933	<1e-4	ssl0109	GO:0071842	<1e-4	slr1619	GO:0009892	<1e-4	ssl0483	GO:0022892	<1e-4	sll1191	GO:0003824	0.2837

sll0085	GO:0008150	<1e-4	ssr5092	GO:0051171	<1e-4	sll0301	GO:0019637	<1e-4	sll1304	GO:0022832	<1e-4	ssl7045	GO:0003824	0.2837
slr0374	GO:0060255	<1e-4	ssl3615	GO:0022411	<1e-4	sll0762	GO:0045184	<1e-4	sll1022	GO:0016462	<1e-4	slr0780	GO:0003824	0.2837
ssl0787	GO:0033013	<1e-4	sll0447	GO:0051716	<1e-4	ssr0109	GO:0006220	<1e-4	sll0854	GO:0022803	<1e-4	sll1784	GO:0003824	0.2837
sll0985	GO:0060255	<1e-4	slr0553	GO:0044003	<1e-4	ssr6099	GO:0051716	<1e-4	slr0709	GO:0016746	<1e-4	ssl7048	GO:0003824	0.2837
slr1956	GO:0006753	<1e-4	slr0784	GO:0009141	<1e-4	sll1400	GO:0080090	<1e-4	slr1376	GO:0008171	<1e-4	slr1601	GO:0003824	0.2837
sll0756	GO:0071842	<1e-4	ssl0787	GO:0048878	<1e-4	sll1240	GO:0046483	<1e-4	sll0630	GO:0008171	<1e-4	slr1449	GO:0003824	0.2837
sll0442	GO:0009123	<1e-4	slr1906	GO:0055086	<1e-4	slr0909	GO:0072521	<1e-4	sll1696	GO:0016746	<1e-4	ssl1300	GO:0003824	0.2837
sll8035	GO:0006066	<1e-4	sll1737	GO:0046164	<1e-4	sll0751	GO:0006811	<1e-4	sll5090	GO:0022890	<1e-4	sll0608	GO:0003824	0.2837
ssl3549	GO:0051179	<1e-4	sll0265	GO:0050789	<1e-4	sll1696	GO:0005996	<1e-4	slr1990	GO:0016746	<1e-4	sll7050	GO:0003824	0.2837
sll1632	GO:0050801	<1e-4	slr1464	GO:0046483	<1e-4	slr0509	GO:0050896	<1e-4	slr5023	GO:0060089	<1e-4	sll0321	GO:0003824	0.2837
slr1079	GO:0050794	<1e-4	sll0783	GO:0010468	<1e-4	sll1570	GO:0006576	<1e-4	sll1086	GO:0003674	<1e-4	sll1583	GO:0003824	0.2837
sll1630	GO:0051818	<1e-4	slr1273	GO:0055082	<1e-4	sll0763	GO:0051246	<1e-4	slr1195	GO:0022838	<1e-4	sll1319	GO:0003824	0.2837
sll8012	GO:0008150	<1e-4	slr7091	GO:0019752	<1e-4	slr1066	GO:0080090	<1e-4	slr2084	GO:0016462	<1e-4	slr1990	GO:0003824	0.2837
sll0802	GO:0044003	<1e-4	sll0810	GO:0009123	<1e-4	slr1914	GO:0009199	<1e-4	slr1977	GO:0016746	<1e-4	sll8019	GO:0003824	0.2837
sll0543	GO:0043933	<1e-4	sll1251	GO:0044260	<1e-4	slr1940	GO:0048519	<1e-4	sll1512	GO:0022892	<1e-4	slr5053	GO:0003824	0.2837
sll0499	GO:0031326	<1e-4	sll1714	GO:0048519	<1e-4	slr1378	GO:0019222	<1e-4	ssr3570	GO:0022838	<1e-4	slr6029	GO:0003824	0.2837
sll1163	GO:0031326	<1e-4	ssl0467	GO:0071841	<1e-4	sll1926	GO:0046128	<1e-4	sll5003	GO:0043167	<1e-4	sll1164	GO:0003824	0.2837
slr0852	GO:0006811	<1e-4	ssr0692	GO:0044281	<1e-4	slr0670	GO:0009259	<1e-4	slr0510	GO:0016462	<1e-4	slr5073	GO:0003824	0.2837
slr0364	GO:0065007	<1e-4	sll1254	GO:0006163	<1e-4	slr6051	GO:0044281	<1e-4	slr0496	GO:0005342	<1e-4	sll1681	GO:0003824	0.2837
ssr2787	GO:0006576	<1e-4	sll5032	GO:0009165	<1e-4	ssl1004	GO:0042455	<1e-4	slr0503	GO:0016746	<1e-4	ssl7022	GO:0003824	0.2837
ssl5099	GO:0046164	<1e-4	slr1339	GO:0051188	<1e-4	slr1913	GO:0065007	<1e-4	slr0362	GO:0003674	<1e-4	ssl5068	GO:0003824	0.2837
slr0962	GO:0051818	<1e-4	sll1240	GO:0044248	<1e-4	slr1142	GO:0006733	<1e-4	slr1533	GO:0022890	<1e-4	ssl5114	GO:0003824	0.2837
sll0751	GO:0046394	<1e-4	slr0496	GO:0044260	<1e-4	sll0442	GO:0055114	<1e-4	sll1834	GO:0022892	<1e-4	ssl2814	GO:0003824	0.2837
sll1763	GO:0045184	<1e-4	slr6008	GO:0016053	<1e-4	ssl0788	GO:0048519	<1e-4	sll1173	GO:0008171	<1e-4	sll0376	GO:0003824	0.2837
slr0656	GO:0006753	<1e-4	sll0933	GO:0019637	<1e-4	slr1956	GO:0010556	<1e-4	slr1627	GO:0015077	<1e-4	sll1898	GO:0003824	0.2837
slr0596	GO:0051171	<1e-4	sll5089	GO:0034641	<1e-4	ssr6026	GO:0065007	<1e-4	sll1925	GO:0022857	<1e-4	slr0888	GO:0003824	0.2837
sll1442	GO:0072522	<1e-4	slr0480	GO:0019219	<1e-4	sll5003	GO:0046164	<1e-4	slr1648	GO:0015293	<1e-4	sll0736	GO:0003824	0.2837
sll0156	GO:0043412	<1e-4	slr6071	GO:0009124	<1e-4	ssr1155	GO:0042451	<1e-4	slr0168	GO:0005342	<1e-4	sll1915	GO:0003824	0.2837
slr1674	GO:0034641	<1e-4	sll0996	GO:0005996	<1e-4	slr1505	GO:0009309	<1e-4	sll0376	GO:0016818	<1e-4	ssl1046	GO:0003824	0.2837
slr0169	GO:0019751	<1e-4	slr6021	GO:0006811	<1e-4	slr1911	GO:0006066	<1e-4	slr0869	GO:0022857	<1e-4	ssr5121	GO:0003824	0.2837
sll0372	GO:0009260	<1e-4	ssr2802	GO:0044271	<1e-4	sll1142	GO:0071841	<1e-4	sll7006	GO:0022803	<1e-4	sll0696	GO:0003824	0.2837
sll1752	GO:0009892	<1e-4	sll1344	GO:0043170	<1e-4	sll1738	GO:0031323	<1e-4	sll1511	GO:0022890	<1e-4	sll7086	GO:0003824	0.2837
ssr0332	GO:0042451	<1e-4	sll0886	GO:0055086	<1e-4	sll1068	GO:0009150	<1e-4	slr2027	GO:0016462	<1e-4	ssl7021	GO:0003824	0.2837
sll7031	GO:0006793	<1e-4	sll1052	GO:0019751	<1e-4	slr0637	GO:0006732	<1e-4	ssr6019	GO:0015267	<1e-4	slr1290	GO:0003824	0.2837
slr1103	GO:0006732	<1e-4	sll5026	GO:0090304	<1e-4	slr0217	GO:0072528	<1e-4	slr1259	GO:0015077	<1e-4	sll0847	GO:0003824	0.2837
sll1188	GO:0005996	<1e-4	slr1495	GO:0048523	<1e-4	slr1923	GO:0051818	<1e-4	slr0780	GO:0022890	<1e-4	ssr0657	GO:0003824	0.2837
sll1640	GO:0006082	<1e-4	sll0448	GO:0043170	<1e-4	ssr6030	GO:0051347	<1e-4	ssl3829	GO:0022803	<1e-4	slr6045	GO:0003824	0.2837
ssl5031	GO:0009126	<1e-4	ssl1792	GO:0009124	<1e-4	sll0283	GO:0009893	<1e-4	slr1260	GO:0015405	<1e-4	ssl1690	GO:0003824	0.2837
sll1192	GO:0009987	<1e-4	sll0931	GO:0051171	<1e-4	ssr3154	GO:0034654	<1e-4	sll1247	GO:0016741	<1e-4	sll7006	GO:0003824	0.2837
sll0359	GO:0009150	<1e-4	slr1449	GO:0009161	<1e-4	sll0839	GO:0009889	<1e-4	sll1912	GO:0016817	<1e-4	ssr3129	GO:0003824	0.2837
slr2025	GO:0018130	<1e-4	sll1773	GO:0034654	<1e-4	slr0634	GO:0009161	<1e-4	sll0996	GO:0008171	<1e-4	ssl5008	GO:0003824	0.2837
sll0350	GO:0006576	<1e-4	ssr3571	GO:0050794	<1e-4	sll1036	GO:0009309	<1e-4	ssl2069	GO:0022832	<1e-4	ssr6086	GO:0003824	0.2837
sll0263	GO:0009142	<1e-4	sll0443	GO:0050794	<1e-4	sll0857	GO:0046483	<1e-4	slr7059	GO:0016746	<1e-4	slr2052	GO:0003824	0.2837
sll0372	GO:0051171	<1e-4	ssl5098	GO:0009150	<1e-4	slr0924	GO:0048518	<1e-4	slr1862	GO:0022836	<1e-4	slr1206	GO:0003824	0.2837
slr1670	GO:0051818	<1e-4	sll0641	GO:0051234	<1e-4	slr0650	GO:0009124	<1e-4	sll1388	GO:0015077	<1e-4	slr1624	GO:0003824	0.2837
ssr2553	GO:0034641	<1e-4	slr0784	GO:0065007	<1e-4	slr0924	GO:0072528	<1e-4	slr0489	GO:0022892	<1e-4	sll0031	GO:0003824	0.2837
slr5118	GO:0009199	<1e-4	slr0692	GO:0048522	<1e-4	slr0780	GO:0044283	<1e-4	slr1862	GO:0016746	<1e-4	sll1068	GO:0003824	0.2837
sll1939	GO:0050789	<1e-4	slr0362	GO:0031640	<1e-4	slr0482	GO:0006091	<1e-4	sll0732	GO:0015405	<1e-4	ssr1768	GO:0003824	0.2837
slr1215	GO:0071841	<1e-4	slr1638	GO:0071496	<1e-4	sll0872	GO:0009144	<1e-4	slr0211	GO:0015075	<1e-4	ssl8028	GO:0003824	0.2837
sll0781	GO:0031323	<1e-4	slr1032	GO:0090304	<1e-4	sll0498	GO:0009260	<1e-4	slr1774	GO:0016818	<1e-4	slr0816	GO:0003824	0.2837
sll0552	GO:0051171	<1e-4	sll8007	GO:0005996	<1e-4	sll7070	GO:0055114	<1e-4	sll1606	GO:0022892	<1e-4	slr2071	GO:0003824	0.2837
sll1461	GO:0051716	<1e-4	ssr6002	GO:0009889	<1e-4	slr6103	GO:0051179	<1e-4	sll1186	GO:0022892	<1e-4	sll1495	GO:0003824	0.2837
slr1918	GO:0016052	<1e-4	slr0876	GO:0034641	<1e-4	ssr1766	GO:0071554	<1e-4	ssl2920	GO:0003674	<1e-4	ssr6030	GO:0003824	0.2837
ssl5113	GO:0022411	<1e-4	slr6075	GO:0016052	<1e-4	slr1143	GO:0048523	<1e-4	slr0476	GO:0015293	<1e-4	slr5024	GO:0003824	0.2837

ssr3570	GO:0065007	<1e-4	ssr2201	GO:0051171	<1e-4	slr0498	GO:0050794	<1e-4	slr1530	GO:0016746	<1e-4	slr0416	GO:0003824	0.2837
slr1425	GO:0005996	<1e-4	slr7102	GO:0042451	<1e-4	slr0006	GO:0009123	<1e-4	slr1177	GO:0022857	<1e-4	ssr2318	GO:0003824	0.2837
sll1062	GO:0009262	<1e-4	sll1680	GO:0001932	<1e-4	sll5030	GO:0022607	<1e-4	slr0294	GO:0015267	<1e-4	ssl3573	GO:0003824	0.2837
sll1373	GO:0019637	<1e-4	slr1161	GO:0060255	<1e-4	sll1318	GO:0080090	<1e-4	slr1628	GO:0060089	<1e-4	sll0552	GO:0003824	0.2837
sll1350	GO:0019222	<1e-4	slr0625	GO:0052111	<1e-4	slr7037	GO:0042455	<1e-4	ssl6092	GO:0016817	<1e-4	ssr3570	GO:0003824	0.2837
slr0680	GO:0048878	<1e-4	sll0280	GO:0090304	<1e-4	slr1603	GO:0009132	<1e-4	slr0924	GO:0060089	<1e-4	slr6022	GO:0003824	0.2837
slr1618	GO:0009161	<1e-4	slr1544	GO:0034654	<1e-4	slr0299	GO:0044255	<1e-4	sll1155	GO:0015293	<1e-4	slr0699	GO:0003824	0.2837
slr0337	GO:0009991	<1e-4	slr0023	GO:0022607	<1e-4	sll1372	GO:0051188	<1e-4	ssr0761	GO:0015291	<1e-4	ssr7084	GO:0003824	0.2837
sll0297	GO:0048878	<1e-4	sll0413	GO:0051179	<1e-4	slr0664	GO:0046128	<1e-4	ssl2009	GO:0022857	<1e-4	ssr3572	GO:0003824	0.2837
slr1241	GO:0008150	<1e-4	slr0366	GO:0006811	<1e-4	sll1638	GO:0072521	<1e-4	slr0049	GO:0016818	<1e-4	sll0749	GO:0003824	0.2837
sll1203	GO:0034654	<1e-4	slr6005	GO:0044271	<1e-4	sll0761	GO:0051716	<1e-4	ssl7048	GO:0003674	<1e-4	slr1911	GO:0003824	0.2837
ssr2067	GO:0051347	<1e-4	slr1807	GO:0018130	<1e-4	sll1315	GO:0034641	<1e-4	sll1095	GO:0022892	<1e-4	ssr7017	GO:0003824	0.2837
sll0802	GO:0072528	<1e-4	sll0905	GO:0055114	<1e-4	slr2119	GO:0051234	<1e-4	slr1276	GO:0015399	<1e-4	sll8027	GO:0003824	0.2837
slr1339	GO:0008150	<1e-4	slr0769	GO:0009165	<1e-4	sll0787	GO:0006753	<1e-4	ssl2245	GO:0015077	<1e-4	sll7028	GO:0003824	0.2837
slr0554	GO:0009259	<1e-4	slr1816	GO:0043412	<1e-4	slr0813	GO:0071841	<1e-4	slr1436	GO:0016746	<1e-4	slr0731	GO:0003824	0.2837
sll0183	GO:0090304	<1e-4	sll5030	GO:0044260	<1e-4	sll0160	GO:0051186	<1e-4	sll0066	GO:0022832	<1e-4	slr0812	GO:0003824	0.2837
slr1807	GO:0044106	<1e-4	slr0092	GO:0043648	<1e-4	sll0503	GO:0080090	<1e-4	slr1670	GO:0015405	<1e-4	slr2003	GO:0003824	0.2837
sll1613	GO:0006631	<1e-4	slr5102	GO:0051716	<1e-4	slr0692	GO:0034654	<1e-4	sll7064	GO:0043167	<1e-4	ssr6027	GO:0003824	0.2837
sll5090	GO:0009394	<1e-4	slr6029	GO:0051171	<1e-4	ssl7074	GO:0006721	<1e-4	slr7081	GO:0042623	<1e-4	sll1609	GO:0003824	0.2837
slr0483	GO:0051188	<1e-4	slr1913	GO:0019219	<1e-4	slr1790	GO:0034654	<1e-4	slr1441	GO:0022892	<1e-4	sll0765	GO:0003824	0.2837
slr1880	GO:0044283	<1e-4	slr1110	GO:0009987	<1e-4	slr0553	GO:0009893	<1e-4	slr0269	GO:0003674	<1e-4	slr0667	GO:0003824	0.2837
slr0303	GO:0055086	<1e-4	sll1873	GO:0046394	<1e-4	ssl5027	GO:0044248	<1e-4	ssr2049	GO:0022803	<1e-4	sll1307	GO:0003824	0.2837
slr1415	GO:0042180	<1e-4	slr0670	GO:0009893	<1e-4	ssr0332	GO:0006082	<1e-4	sll1691	GO:0022891	<1e-4	sll7034	GO:0003824	0.2837
ssl5027	GO:0080090	<1e-4	slr0619	GO:0034654	<1e-4	slr1563	GO:0019637	<1e-4	sll0913	GO:0015405	<1e-4	slr5127	GO:0003824	0.2837
slr0516	GO:0072524	<1e-4	slr2070	GO:0006066	<1e-4	sll1378	GO:0009117	<1e-4	slr1544	GO:0015075	<1e-4	slr1114	GO:0003824	0.2837
sll1142	GO:0072521	<1e-4	ssr6078	GO:0009144	<1e-4	slr5126	GO:0019637	<1e-4	slr5018	GO:0015291	<1e-4	sll1095	GO:0003824	0.2837
slr1240	GO:0009150	<1e-4	slr1071	GO:0042180	<1e-4	slr0362	GO:0006066	<1e-4	ssl1918	GO:0016741	<1e-4	ssl7074	GO:0003824	0.2837
slr6004	GO:0046164	<1e-4	slr1474	GO:0043436	<1e-4	slr1762	GO:0006091	<1e-4	sll1373	GO:0016818	<1e-4	slr1577	GO:0003824	0.2837
sll0787	GO:0043933	<1e-4	sll1509	GO:0006576	<1e-4	sll0473	GO:0009117	<1e-4	ssr1256	GO:0015293	<1e-4	slr0730	GO:0003824	0.2837
slr0670	GO:0009165	<1e-4	sgl0001	GO:0019637	<1e-4	sll7034	GO:0050794	<1e-4	ssr5106	GO:0022857	<1e-4	sll0858	GO:0003824	0.2837
slr2000	GO:0060255	<1e-4	sll0447	GO:0022411	<1e-4	slr6071	GO:0016052	<1e-4	sll0854	GO:0060089	<1e-4	slr6044	GO:0003824	0.2837
ssr8013	GO:0001932	<1e-4	sll0482	GO:0019219	<1e-4	slr7023	GO:0006732	<1e-4	sll0932	GO:0015267	<1e-4	ssl1263	GO:0003824	0.2837
sll1925	GO:0072527	<1e-4	sll0564	GO:0071841	<1e-4	slr0146	GO:0009308	<1e-4	sll0157	GO:0015293	<1e-4	slr1394	GO:0003824	0.2837
slr0366	GO:0009308	<1e-4	sll7050	GO:0071842	<1e-4	slr1513	GO:0009165	<1e-4	ssl1918	GO:0015075	<1e-4	slr0013	GO:0003824	0.2837
ssr1698	GO:0044003	<1e-4	sll0286	GO:0006631	<1e-4	sll0552	GO:0051188	<1e-4	sll7078	GO:0015267	<1e-4	sll1717	GO:0003824	0.2837
slr6051	GO:0009057	<1e-4	ssl0353	GO:0051347	<1e-4	sll0508	GO:0046164	<1e-4	sll0148	GO:0043167	<1e-4	sll8011	GO:0003824	0.2837
slr1917	GO:0009308	<1e-4	ssl1300	GO:0009259	<1e-4	slr5116	GO:0050801	<1e-4	ssr0759	GO:0015077	<1e-4	sll0101	GO:0003824	0.2837
sll0263	GO:0009889	<1e-4	sll1193	GO:0016053	<1e-4	slr1603	GO:0009309	<1e-4	sll1052	GO:0022891	<1e-4	ssl5100	GO:0003824	0.2837
slr1174	GO:0048523	<1e-4	slr7096	GO:0048523	<1e-4	ssl2717	GO:0044106	<1e-4	slr2005	GO:0015405	<1e-4	ssr2975	GO:0003824	0.2837
slr0784	GO:0031323	<1e-4	sll0847	GO:0042180	<1e-4	sll1340	GO:0043170	<1e-4	slr1530	GO:0022838	<1e-4	ssr2047	GO:0003824	0.2837
slr1353	GO:0055082	<1e-4	slr1178	GO:0006066	<1e-4	slr1816	GO:0016053	<1e-4	ssl1498	GO:0015075	<1e-4	sll0565	GO:0003824	0.2837
sll1866	GO:0051171	<1e-4	sll5004	GO:0006721	<1e-4	sll1191	GO:0034641	<1e-4	slr0109	GO:0015293	<1e-4	sll1009	GO:0008152	0.2109
sll0280	GO:0072524	<1e-4	ssl5008	GO:0033013	<1e-4	sll1735	GO:0048878	<1e-4	sll1681	GO:0015267	<1e-4	sll8027	GO:0008152	0.2109
slr0241	GO:0044283	<1e-4	slr2000	GO:0044260	<1e-4	sll0847	GO:0044248	<1e-4	slr1186	GO:0022857	<1e-4	sll1192	GO:0008152	0.2109
slr1110	GO:0005996	<1e-4	slr0217	GO:0048523	<1e-4	slr1815	GO:0051716	<1e-4	sll0149	GO:0022836	<1e-4	ssr3467	GO:0008152	0.2109
sll1411	GO:0019637	<1e-4	slr1660	GO:0009308	<1e-4	sll0176	GO:0006066	<1e-4	ssl1498	GO:0015267	<1e-4	ssl7042	GO:0008152	0.2109
slr1142	GO:0034660	<1e-4	sll1511	GO:0001932	<1e-4	sll1613	GO:0051716	<1e-4	slr0358	GO:0016818	<1e-4	sll5067	GO:0008152	0.2109
slr6103	GO:0048518	<1e-4	slr0455	GO:0045184	<1e-4	slr1436	GO:0022411	<1e-4	sll0249	GO:0015291	<1e-4	slr0670	GO:0008152	0.2109
slr1339	GO:0022607	<1e-4	sll0898	GO:0048878	<1e-4	ssl0787	GO:0006753	<1e-4	ssr0336	GO:0016741	<1e-4	sll5003	GO:0008152	0.2109
slr1406	GO:0009309	<1e-4	sll5030	GO:0019752	<1e-4	slr2032	GO:0006066	<1e-4	sll7090	GO:0022838	<1e-4	slr8021	GO:0008152	0.2109
ssl7021	GO:0051186	<1e-4	slr1431	GO:0034641	<1e-4	slr1690	GO:0009889	<1e-4	ssr0102	GO:0015077	<1e-4	sll0765	GO:0008152	0.2109
slr1413	GO:0080090	<1e-4	slr5024	GO:0045184	<1e-4	ssr0336	GO:0051234	<1e-4	sll0788	GO:0022857	<1e-4	slr5016	GO:0008152	0.2109
ssl5008	GO:0060255	<1e-4	slr1573	GO:0031640	<1e-4	slr5037	GO:0072522	<1e-4	ssl2065	GO:0022890	<1e-4	sll7067	GO:0008152	0.2109
sll1355	GO:0043549	<1e-4	slr6068	GO:0050801	<1e-4	sll0360	GO:0019219	<1e-4	ssl3382	GO:0015399	<1e-4	slr1563	GO:0008152	0.2109

sll2013	GO:0046164	<1e-4	slr6047	GO:0042451	<1e-4	ssl1690	GO:0006732	<1e-4	sll0911	GO:0016746	<1e-4	slr0699	GO:0008152	0.2109
slr0184	GO:0071841	<1e-4	ssr5106	GO:0009144	<1e-4	slr1762	GO:0042180	<1e-4	slr1406	GO:0008171	<1e-4	slr7057	GO:0008152	0.2109
ssr3570	GO:0044260	<1e-4	slr2000	GO:0009132	<1e-4	slr0392	GO:0031326	<1e-4	slr1636	GO:0015267	<1e-4	sll1188	GO:0008152	0.2109
sll1891	GO:0065007	<1e-4	sll0860	GO:0045184	<1e-4	sll0297	GO:0050794	<1e-4	sll1608	GO:0022891	<1e-4	slr7071	GO:0008152	0.2109
slr1577	GO:0008150	<1e-4	sll1749	GO:0008610	<1e-4	sll1052	GO:0006082	<1e-4	sll0532	GO:0060089	<1e-4	slr0313	GO:0008152	0.2109
slr0263	GO:0071842	<1e-4	slr0821	GO:0044106	<1e-4	sll0913	GO:0022411	<1e-4	slr0423	GO:0060089	<1e-4	slr6045	GO:0008152	0.2109
sll0659	GO:0008150	<1e-4	sll2007	GO:0022411	<1e-4	slr1875	GO:0051188	<1e-4	slr0106	GO:0022892	<1e-4	slr1789	GO:0008152	0.2109
sll0400	GO:0072528	<1e-4	slr1025	GO:0009142	<1e-4	slr5018	GO:0065007	<1e-4	ssl0294	GO:0016746	<1e-4	sll0565	GO:0008152	0.2109
slr1690	GO:0050801	<1e-4	sll0614	GO:0055082	<1e-4	ssl2471	GO:0009394	<1e-4	sll0939	GO:0016746	<1e-4	slr1438	GO:0008152	0.2109
sll0174	GO:0051234	<1e-4	slr1183	GO:0009987	<1e-4	slr1362	GO:0042455	<1e-4	slr1570	GO:0043492	<1e-4	sll0031	GO:0008152	0.2109
sll1247	GO:0051186	<1e-4	slr0569	GO:0009161	<1e-4	sll0703	GO:0016052	<1e-4	ssr6002	GO:0022836	<1e-4	ssl0788	GO:0008152	0.2109
slr0592	GO:0045184	<1e-4	sll1737	GO:0060255	<1e-4	slr1397	GO:0009132	<1e-4	slr0731	GO:0015405	<1e-4	sll0253	GO:0008152	0.2109
slr5016	GO:0052188	<1e-4	slr5116	GO:0006066	<1e-4	sll0827	GO:0043648	<1e-4	slr1778	GO:0015077	<1e-4	slr1990	GO:0008152	0.2109
slr0914	GO:0009991	<1e-4	slr1814	GO:0048522	<1e-4	ssl3549	GO:0006732	<1e-4	slr0957	GO:0016746	<1e-4	ssr0657	GO:0008152	0.2109
slr1648	GO:0071842	<1e-4	ssl7042	GO:0050801	<1e-4	ssl0353	GO:0052188	<1e-4	sll8007	GO:0015399	<1e-4	slr1599	GO:0008152	0.2109
slr2049	GO:0001932	<1e-4	ssl0750	GO:0009987	<1e-4	slr0109	GO:0009987	<1e-4	sll1192	GO:0015399	<1e-4	ssr6019	GO:0008152	0.2109
slr6028	GO:0006576	<1e-4	sll0749	GO:0071554	<1e-4	slr0769	GO:0044260	<1e-4	ssr3304	GO:0022803	<1e-4	slr0613	GO:0008152	0.2109
slr1660	GO:0006163	<1e-4	sll0085	GO:0009889	<1e-4	slr1276	GO:0043933	<1e-4	slr1098	GO:0022892	<1e-4	ssr1473	GO:0008152	0.2109
sll0354	GO:0044260	<1e-4	sll1942	GO:0009126	<1e-4	sll1340	GO:0042180	<1e-4	slr0195	GO:0022857	<1e-4	sll1698	GO:0008152	0.2109
slr0345	GO:0072527	<1e-4	sll1267	GO:0031640	<1e-4	slr1611	GO:0009892	<1e-4	slr2070	GO:0016746	<1e-4	sll8040	GO:0008152	0.2109
slr2080	GO:0009144	<1e-4	sll0702	GO:0051818	<1e-4	sll1862	GO:0072527	<1e-4	slr0609	GO:0016462	<1e-4	ssl5091	GO:0008152	0.2109
slr6104	GO:0006732	<1e-4	sll0085	GO:0072528	<1e-4	ssr0332	GO:0043933	<1e-4	slr1174	GO:0022832	<1e-4	slr0888	GO:0008152	0.2109
sll0802	GO:0046128	<1e-4	slr7102	GO:0052111	<1e-4	sll5063	GO:0034654	<1e-4	slr0521	GO:0016746	<1e-4	slr0300	GO:0008152	0.2109
ssl7045	GO:0009141	<1e-4	sll1950	GO:0018130	<1e-4	sll0645	GO:0043170	<1e-4	sll0496	GO:0016462	<1e-4	ssr3572	GO:0008152	0.2109
sll0488	GO:0046483	<1e-4	sll1601	GO:0009141	<1e-4	slr0981	GO:0009144	<1e-4	slr5012	GO:0005342	<1e-4	slr1670	GO:0008152	0.2109
ssl0109	GO:0008150	<1e-4	slr0269	GO:0046164	<1e-4	sll1186	GO:0048519	<1e-4	ssl5114	GO:0016462	<1e-4	ssl0410	GO:0008152	0.2109
slr1261	GO:0072524	<1e-4	slr1104	GO:0016052	<1e-4	sll1086	GO:0006721	<1e-4	slr1074	GO:0043167	<1e-4	sll0608	GO:0008152	0.2109
slr6049	GO:0048519	<1e-4	ssr5019	GO:0006721	<1e-4	slr0654	GO:0009056	<1e-4	slr6100	GO:0022857	<1e-4	slr0725	GO:0008152	0.2109
slr0456	GO:0006576	<1e-4	slr1977	GO:0043412	<1e-4	sll0586	GO:0009057	<1e-4	slr5013	GO:0016817	<1e-4	ssl0350	GO:0008152	0.2109
slr0924	GO:0051234	<1e-4	ssr5092	GO:0009260	<1e-4	slr0912	GO:0072522	<1e-4	slr1944	GO:0015399	<1e-4	ssr6024	GO:0008152	0.2109
sll0647	GO:0009394	<1e-4	slr1275	GO:0071842	<1e-4	ssr1768	GO:0044281	<1e-4	slr0453	GO:0005342	<1e-4	sll1717	GO:0008152	0.2109
slr1083	GO:0019637	<1e-4	sll1319	GO:0051716	<1e-4	ssl3692	GO:0052111	<1e-4	sll0062	GO:0003674	<1e-4	sll1882	GO:0008152	0.2109
slr1958	GO:0016054	<1e-4	sll5046	GO:0046128	<1e-4	sll6052	GO:0009987	<1e-4	ssl8003	GO:0015075	<1e-4	sll1640	GO:0008152	0.2109
slr0725	GO:0019219	<1e-4	sll1509	GO:0051179	<1e-4	sll0861	GO:0006721	<1e-4	slr0325	GO:0016817	<1e-4	slr1079	GO:0008152	0.2109
sll1853	GO:0006066	<1e-4	sll1068	GO:0051818	<1e-4	slr0637	GO:0009260	<1e-4	sll1251	GO:0022832	<1e-4	sll1891	GO:0008152	0.2109
slr1342	GO:0009161	<1e-4	slr1444	GO:0009991	<1e-4	slr2000	GO:0001932	<1e-4	ssr3122	GO:0022857	<1e-4	sll0670	GO:0008152	0.2109
slr7059	GO:0045184	<1e-4	sll1830	GO:0006082	<1e-4	slr1920	GO:0006066	<1e-4	slr2084	GO:0003674	<1e-4	slr2003	GO:0008152	0.2109
ssr2318	GO:0034641	<1e-4	sll1160	GO:0050794	<1e-4	sll1289	GO:0010468	<1e-4	slr0317	GO:0060089	<1e-4	slr7099	GO:0008152	0.2109
slr5073	GO:0051234	<1e-4	ssr6086	GO:0046483	<1e-4	ssr6026	GO:0009893	<1e-4	slr0909	GO:0043167	<1e-4	sll8011	GO:0008152	0.2109
ssl1046	GO:0019219	<1e-4	sll1135	GO:0051246	<1e-4	sll0691	GO:0052111	<1e-4	slr0491	GO:0022890	<1e-4	sll0162	GO:0008152	0.2109
slr5111	GO:0072524	<1e-4	sll0149	GO:0008150	<1e-4	slr0730	GO:0044003	<1e-4	slr1862	GO:0022891	<1e-4	sll6055	GO:0008152	0.2109
ssl5008	GO:0009123	<1e-4	slr0333	GO:0046907	<1e-4	slr0199	GO:0019751	<1e-4	sll0611	GO:0022838	<1e-4	sll7089	GO:0008152	0.2109
ssl1792	GO:0042455	<1e-4	ssl7045	GO:0072527	<1e-4	sll1352	GO:0072522	<1e-4	slr6044	GO:0060089	<1e-4	sll1570	GO:0008152	0.2109
slr5116	GO:0042451	<1e-4	slr1270	GO:0046164	<1e-4	slr1396	GO:0065007	<1e-4	sll1092	GO:0015267	<1e-4	slr0731	GO:0008152	0.2109
sll7030	GO:0006720	<1e-4	slr0294	GO:0009262	<1e-4	slr1071	GO:0043549	<1e-4	ssl2245	GO:0015291	<1e-4	sll1532	GO:0008152	0.2109
slr0149	GO:0009056	<1e-4	sll0691	GO:0052188	<1e-4	slr0059	GO:0044271	<1e-4	slr5024	GO:0022832	<1e-4	sll0479	GO:0008152	0.2109
slr6101	GO:0009150	<1e-4	slr0038	GO:0009161	<1e-4	sll1630	GO:0042180	<1e-4	slr1852	GO:0008171	<1e-4	slr0885	GO:0008152	0.2109
slr0271	GO:0051716	<1e-4	sll1773	GO:0043648	<1e-4	slr5118	GO:0006721	<1e-4	slr1964	GO:0022836	<1e-4	slr5126	GO:0008152	0.2109
slr1670	GO:0009260	<1e-4	sll0448	GO:0019222	<1e-4	sll0147	GO:0001932	<1e-4	ssl0739	GO:0015405	<1e-4	ssl7074	GO:0008152	0.2109
sll0867	GO:0009142	<1e-4	ssr3572	GO:0051818	<1e-4	slr2111	GO:0009263	<1e-4	slr5102	GO:0043167	<1e-4	ssr2047	GO:0008152	0.2109
sll0608	GO:0072528	<1e-4	sll1542	GO:0043436	<1e-4	sll8035	GO:0019222	<1e-4	ssr0759	GO:0022832	<1e-4	slr0270	GO:0008152	0.2109
slr0250	GO:0010556	<1e-4	sll1173	GO:0072528	<1e-4	sll1749	GO:0044282	<1e-4	ssr2802	GO:0016818	<1e-4	sll1832	GO:0008152	0.2109
ssr5011	GO:0009056	<1e-4	ssr2754	GO:0055082	<1e-4	sll1399	GO:0019222	<1e-4	ssr1155	GO:0005342	<1e-4	slr2004	GO:0008152	0.2109
sll0436	GO:0072528	<1e-4	slr0393	GO:0048523	<1e-4	slr6051	GO:0009263	<1e-4	sll0564	GO:0015077	<1e-4	ssl2069	GO:0008152	0.2109

slr2011	GO:0042451	<1e-4	ssl7046	GO:0048518	<1e-4	slr8014	GO:0009161	<1e-4	sll1239	GO:0022857	<1e-4	slr1886	GO:0008152	0.2109
sll1355	GO:0033013	<1e-4	sll0174	GO:0009117	<1e-4	slr1998	GO:0044003	<1e-4	sll1151	GO:0003674	<1e-4	slr0498	GO:0008152	0.2109
sll1658	GO:0009889	<1e-4	sll0498	GO:0042451	<1e-4	slr6090	GO:0042180	<1e-4	ssr7036	GO:0022803	<1e-4	ssr2802	GO:0008152	0.2109
slr2048	GO:0009057	<1e-4	slr1577	GO:0009161	<1e-4	slr2027	GO:0043549	<1e-4	slr0049	GO:0005342	<1e-4	sll5128	GO:0008152	0.2109
slr0285	GO:0006631	<1e-4	slr1162	GO:0009124	<1e-4	ssr3409	GO:0006733	<1e-4	slr1066	GO:0022803	<1e-4	ssl5015	GO:0008152	0.2109
ssr2554	GO:0046164	<1e-4	slr0498	GO:0051716	<1e-4	ssl3573	GO:0019751	<1e-4	sll1761	GO:0043492	<1e-4	ssl3615	GO:0008152	0.2109
slr0740	GO:0009126	<1e-4	sll0183	GO:0009987	<1e-4	ssl0750	GO:0009308	<1e-4	slr2110	GO:0016746	<1e-4	slr0590	GO:0008152	0.2109
slr0341	GO:0019637	<1e-4	ssr6089	GO:0046164	<1e-4	slr6009	GO:0060255	<1e-4	sll1736	GO:0008171	<1e-4	sll1089	GO:0008152	0.2109
slr1170	GO:0043648	<1e-4	slr1541	GO:0072528	<1e-4	slr0806	GO:0009123	<1e-4	sll0609	GO:0015267	<1e-4	slr1450	GO:0008152	0.2109
slr0976	GO:0072524	<1e-4	ssr2422	GO:0048522	<1e-4	slr0142	GO:0048519	<1e-4	sll0297	GO:0015075	<1e-4	ssl3573	GO:0008152	0.2109
sll0405	GO:0065007	<1e-4	sll0611	GO:0009263	<1e-4	sll0218	GO:0006793	<1e-4	sll1906	GO:0015077	<1e-4	slr1807	GO:0008152	0.2109
slr1397	GO:0043648	<1e-4	sll1511	GO:0009141	<1e-4	sll1552	GO:0009308	<1e-4	ssr2551	GO:0016817	<1e-4	slr1507	GO:0008152	0.2109
sll5030	GO:0031323	<1e-4	sll0590	GO:0031326	<1e-4	sll0325	GO:0051171	<1e-4	sll1086	GO:0022890	<1e-4	slr1935	GO:0008152	0.2109
sll7064	GO:0042455	<1e-4	slr2073	GO:0051179	<1e-4	sll0162	GO:0051171	<1e-4	sll0410	GO:0022891	<1e-4	sll1702	GO:0008152	0.2109
sll1188	GO:0071554	<1e-4	slr6044	GO:0010468	<1e-4	slr0912	GO:0034641	<1e-4	slr0337	GO:0042623	<1e-4	sll1049	GO:0008152	0.2109
sll0068	GO:0009260	<1e-4	sll0314	GO:0010468	<1e-4	ssl5095	GO:0010556	<1e-4	slr1590	GO:0008171	<1e-4	slr1206	GO:0008152	0.2109
ssr0692	GO:0034660	<1e-4	ssr2201	GO:0006091	<1e-4	sll0149	GO:0009150	<1e-4	slr1800	GO:0005342	<1e-4	slr1790	GO:0008152	0.2109
slr1339	GO:0048523	<1e-4	ssr0109	GO:0065007	<1e-4	ssr2711	GO:0009262	<1e-4	sgl0002	GO:0043167	<1e-4	sll1222	GO:0006810	0.2098
slr0976	GO:0044106	<1e-4	ssr3409	GO:0044281	<1e-4	sll0625	GO:0022607	<1e-4	slr0199	GO:0022832	<1e-4	sll1272	GO:0006810	0.2098
slr1660	GO:0009165	<1e-4	slr7010	GO:0048522	<1e-4	ssr2843	GO:0050896	<1e-4	sll1390	GO:0015399	<1e-4	slr0642	GO:0006810	0.2098
sll1530	GO:0006811	<1e-4	sll1495	GO:0009889	<1e-4	slr1431	GO:0043933	<1e-4	ssr1558	GO:0043492	<1e-4	sll1528	GO:0006810	0.2098
slr1074	GO:0006720	<1e-4	sll0786	GO:0009892	<1e-4	slr0453	GO:0090304	<1e-4	sll0661	GO:0042623	<1e-4	sll0149	GO:0016491	0.203
slr0060	GO:0009117	<1e-4	slr1819	GO:0009124	<1e-4	slr1025	GO:0016054	<1e-4	slr1396	GO:0015075	<1e-4	slr1702	GO:0006810	0.2005
sll0811	GO:0043436	<1e-4	slr1484	GO:0006766	<1e-4	slr0112	GO:0019637	<1e-4	ssl0352	GO:0015405	<1e-4	slr0341	GO:0006810	0.2005
slr1186	GO:0050789	<1e-4	sll8001	GO:0042455	<1e-4	sll0872	GO:0010468	<1e-4	slr8022	GO:0022892	<1e-4	slr0491	GO:0006810	0.2005
slr1533	GO:0044248	<1e-4	slr1618	GO:0046164	<1e-4	sll1250	GO:0016052	<1e-4	slr7080	GO:0022836	<1e-4	slr0810	GO:0006810	0.2005
slr1110	GO:0051186	<1e-4	slr0730	GO:0048518	<1e-4	slr1886	GO:0008150	<1e-4	ssr3304	GO:0008171	<1e-4	slr2092	GO:0006810	0.2005
sll0191	GO:0009059	<1e-4	slr6047	GO:0006766	<1e-4	slr1045	GO:0009059	<1e-4	slr7014	GO:0022832	<1e-4	slr1812	GO:0006810	0.1995
ssl3573	GO:0046164	<1e-4	sll0678	GO:0080090	<1e-4	sll0241	GO:0010468	<1e-4	slr0552	GO:0015293	<1e-4	sll0861	GO:0006810	0.1982
sll1285	GO:0006163	<1e-4	slr0374	GO:0043933	<1e-4	slr1189	GO:0006793	<1e-4	ssr3189	GO:0022836	<1e-4	ssl3382	GO:0006810	0.1977
slr0496	GO:0009987	<1e-4	sll1442	GO:0048522	<1e-4	sll1160	GO:0009262	<1e-4	sll1262	GO:0042623	<1e-4	sll0740	GO:0006810	0.1977
slr6016	GO:0090304	<1e-4	sll5089	GO:0051716	<1e-4	slr1866	GO:0071554	<1e-4	slr0730	GO:0016462	<1e-4	ssr2754	GO:0006810	0.1977
sll1511	GO:0055114	<1e-4	slr0818	GO:0046164	<1e-4	sll1009	GO:0080090	<1e-4	slr7094	GO:0005342	<1e-4	slr0272	GO:0006810	0.1977
ssl8039	GO:0031326	<1e-4	sll0611	GO:0009142	<1e-4	sll0168	GO:0052111	<1e-4	slr0172	GO:0060089	<1e-4	slr0742	GO:0006810	0.1977
ssr2848	GO:0072527	<1e-4	slr1505	GO:0009889	<1e-4	sll0867	GO:0071840	<1e-4	slr1918	GO:0005342	<1e-4	sll0147	GO:0006810	0.1977
sll7077	GO:0001932	<1e-4	sll0905	GO:0050789	<1e-4	slr0111	GO:0006753	<1e-4	sll1173	GO:0016817	<1e-4	slr0789	GO:0006810	0.1977
slr7025	GO:0051716	<1e-4	slr1773	GO:0043648	<1e-4	slr0919	GO:0055086	<1e-4	ssr6099	GO:0022836	<1e-4	slr0957	GO:0006810	0.1977
ssr7093	GO:0052188	<1e-4	sll0149	GO:0019538	<1e-4	ssr1499	GO:0009144	<1e-4	sll1225	GO:0022832	<1e-4	sll0188	GO:0006810	0.1977
sll0858	GO:0009165	<1e-4	slr1183	GO:0042451	<1e-4	slr0888	GO:0009987	<1e-4	ssr3467	GO:0022803	<1e-4	slr1895	GO:0006810	0.1977
slr0619	GO:0022411	<1e-4	slr1616	GO:0048519	<1e-4	slr1544	GO:0009892	<1e-4	sll1562	GO:0008171	<1e-4	sll1251	GO:0006810	0.1977
sll5067	GO:0006811	<1e-4	slr1339	GO:0051246	<1e-4	slr1263	GO:0043648	<1e-4	ssl5095	GO:0022857	<1e-4	sll1054	GO:0006810	0.1977
slr6103	GO:0006066	<1e-4	sll0871	GO:0009123	<1e-4	sll0266	GO:0009059	<1e-4	sll8040	GO:0016817	<1e-4	sll0225	GO:0006810	0.1977
sll0350	GO:0051347	<1e-4	slr2084	GO:0071841	<1e-4	sll1002	GO:0072521	<1e-4	sll1775	GO:0016462	<1e-4	slr0801	GO:0006810	0.1977
sll1089	GO:0051347	<1e-4	sll8012	GO:0009987	<1e-4	slr0981	GO:0051179	<1e-4	slr0552	GO:0060089	<1e-4	sll0980	GO:0006810	0.1977
slr1195	GO:0043648	<1e-4	ssr3122	GO:0043648	<1e-4	sll1717	GO:0009262	<1e-4	slr1413	GO:0022891	<1e-4	sll1399	GO:0006810	0.1977
sll0072	GO:0009117	<1e-4	sll0176	GO:0042455	<1e-4	sll1254	GO:0055086	<1e-4	sll0360	GO:0022891	<1e-4	slr0249	GO:0006810	0.1977
slr0053	GO:0043412	<1e-4	ssr2333	GO:0006721	<1e-4	slr0598	GO:0050794	<1e-4	slr1222	GO:0022832	<1e-4	slr0318	GO:0006810	0.1977
sll1835	GO:0051246	<1e-4	sll0775	GO:0072522	<1e-4	ssl0739	GO:0045184	<1e-4	slr2012	GO:0022803	<1e-4	sll1344	GO:0006810	0.1977
slr0376	GO:0045184	<1e-4	slr1907	GO:0044248	<1e-4	slr0423	GO:0006811	<1e-4	slr1895	GO:0022803	<1e-4	ssl8008	GO:0006810	0.1977
slr0207	GO:0044283	<1e-4	sll1373	GO:0052188	<1e-4	slr1659	GO:0034660	<1e-4	slr1128	GO:0015291	<1e-4	sll1390	GO:0006810	0.1977
slr0971	GO:0048518	<1e-4	sll0284	GO:0009889	<1e-4	slr5017	GO:0071840	<1e-4	ssr2755	GO:0022836	<1e-4	sll0827	GO:0006810	0.1977
slr0172	GO:0046164	<1e-4	sll1130	GO:0034660	<1e-4	sll1321	GO:0008610	<1e-4	ssr0692	GO:0016818	<1e-4	sll0911	GO:0006810	0.1977
ssl0467	GO:0031640	<1e-4	slr0112	GO:0055086	<1e-4	slr0723	GO:0009124	<1e-4	slr7073	GO:0008171	<1e-4	sll0410	GO:0006810	0.1977
sll1222	GO:0072524	<1e-4	slr6100	GO:0033013	<1e-4	sll1660	GO:0046128	<1e-4	slr1814	GO:0015293	<1e-4	sll0737	GO:0006810	0.1977

sll0397	GO:0009126	<1e-4	slr1752	GO:0022411	<1e-4	ssl1498	GO:0051171	<1e-4	sll1586	GO:0043492	<1e-4	slr1690	GO:0006810	0.1977
ssl2971	GO:0009165	<1e-4	sll0539	GO:0051347	<1e-4	ssl2971	GO:0044275	<1e-4	sll0296	GO:0015399	<1e-4	sll0752	GO:0006810	0.1977
sll1862	GO:0043436	<1e-4	slr0680	GO:0043436	<1e-4	slr0645	GO:0009987	<1e-4	slr0731	GO:0015267	<1e-4	slr1082	GO:0006810	0.1977
sll1956	GO:0046128	<1e-4	sll0670	GO:0044281	<1e-4	sll0810	GO:0009987	<1e-4	sll0335	GO:0016746	<1e-4	slr0358	GO:0006810	0.1977
sll1915	GO:0009259	<1e-4	sll5032	GO:0050789	<1e-4	sll7087	GO:0071840	<1e-4	slr1851	GO:0060089	<1e-4	slr0815	GO:0006810	0.1977
slr1429	GO:0080090	<1e-4	slr0957	GO:0051188	<1e-4	slr6049	GO:0048518	<1e-4	slr1173	GO:0003674	<1e-4	sll0981	GO:0006810	0.1977
ssl8028	GO:0044281	<1e-4	sll0702	GO:0051188	<1e-4	slr0376	GO:0019362	<1e-4	slr1917	GO:0015399	<1e-4	sll0810	GO:0016491	0.1977
sll1659	GO:0009893	<1e-4	slr0780	GO:0034660	<1e-4	slr0606	GO:0044275	<1e-4	slr0521	GO:0043167	<1e-4	sll0449	GO:0016491	0.1977
ssr5106	GO:0080090	<1e-4	slr1493	GO:0009262	<1e-4	slr1384	GO:0044106	<1e-4	sll1830	GO:0008171	<1e-4	sll0614	GO:0016491	0.1977
ssr1552	GO:0033013	<1e-4	slr2120	GO:0045184	<1e-4	slr2010	GO:0006720	<1e-4	sll1995	GO:0015291	<1e-4	slr0729	GO:0016491	0.1977
sll1698	GO:0072527	<1e-4	slr7037	GO:0034660	<1e-4	ssr1041	GO:0043170	<1e-4	slr2027	GO:0022857	<1e-4	slr0554	GO:0016491	0.1977
slr1616	GO:0046483	<1e-4	slr1593	GO:0048519	<1e-4	slr0751	GO:0009260	<1e-4	slr6039	GO:0015293	<1e-4	sll1730	GO:0016491	0.1977
slr0516	GO:0048523	<1e-4	slr0695	GO:0051171	<1e-4	ssr8013	GO:0009123	<1e-4	sll0238	GO:0022803	<1e-4	sll1240	GO:0016491	0.1977
sll0265	GO:0071842	<1e-4	slr7026	GO:0009262	<1e-4	sll1390	GO:0042455	<1e-4	slr1464	GO:0022857	<1e-4	sll0319	GO:0016491	0.1977
sll1191	GO:0006811	<1e-4	sll1225	GO:0019751	<1e-4	sll1071	GO:0016052	<1e-4	ssr1375	GO:0016462	<1e-4	slr1590	GO:0016491	0.1977
ssr1375	GO:0009165	<1e-4	ssl8003	GO:0016054	<1e-4	ssl2471	GO:0051716	<1e-4	slr5012	GO:0043492	<1e-4	slr0957	GO:0016491	0.1977
sll1086	GO:0009124	<1e-4	sll0445	GO:0005976	<1e-4	ssr1766	GO:0006733	<1e-4	slr1896	GO:0016746	<1e-4	sll1411	GO:0016491	0.1977
slr0845	GO:0009161	<1e-4	sll1858	GO:0009892	<1e-4	slr1814	GO:0010556	<1e-4	slr1052	GO:0022832	<1e-4	sll1654	GO:0016491	0.1977
sll7047	GO:0051347	<1e-4	ssr6020	GO:0009057	<1e-4	ssr1552	GO:0071554	<1e-4	slr1260	GO:0016817	<1e-4	slr0605	GO:0016491	0.1977
slr1083	GO:0019752	<1e-4	ssl0467	GO:0044260	<1e-4	sll0625	GO:0050896	<1e-4	sll1691	GO:0022803	<1e-4	sll0297	GO:0016491	0.1977
slr0695	GO:0009132	<1e-4	slr1338	GO:0034654	<1e-4	sll5047	GO:0042451	<1e-4	ssl0350	GO:0016746	<1e-4	slr1122	GO:0016491	0.1977
sll0281	GO:0006066	<1e-4	slr1864	GO:0044260	<1e-4	sll1858	GO:0008150	<1e-4	ssl7051	GO:0043492	<1e-4	sll0980	GO:0016491	0.1977
sll1068	GO:0055086	<1e-4	ssr2781	GO:0043933	<1e-4	ssl3382	GO:0052188	<1e-4	ssr3402	GO:0022892	<1e-4	slr1895	GO:0016491	0.1977
ssr2060	GO:0048518	<1e-4	sll7090	GO:0043412	<1e-4	sll1352	GO:0019222	<1e-4	sll1372	GO:0016746	<1e-4	slr1033	GO:0016491	0.1977
sll0732	GO:0006721	<1e-4	slr0362	GO:0019637	<1e-4	slr1397	GO:0044275	<1e-4	slr2118	GO:0015405	<1e-4	slr0789	GO:0016491	0.1977
slr0049	GO:0016053	<1e-4	sll1135	GO:0051818	<1e-4	sll0284	GO:0006721	<1e-4	slr1505	GO:0016741	<1e-4	sll1956	GO:0016491	0.1977
ssl0312	GO:0009056	<1e-4	ssr1552	GO:0009892	<1e-4	ssr6089	GO:0009150	<1e-4	ssl5096	GO:0043167	<1e-4	slr1415	GO:0016491	0.1977
ssr3467	GO:0006066	<1e-4	slr1315	GO:0009144	<1e-4	sll1344	GO:0005996	<1e-4	slr1396	GO:0043492	<1e-4	sll0588	GO:0016491	0.1977
slr2110	GO:0080090	<1e-4	sll1021	GO:0009987	<1e-4	ssr2201	GO:0010468	<1e-4	sll0810	GO:0015293	<1e-4	sll0410	GO:0016491	0.1977
ssr8047	GO:0043648	<1e-4	slr1896	GO:0042455	<1e-4	sll8002	GO:0006163	<1e-4	ssl1417	GO:0016817	<1e-4	sll1950	GO:0016491	0.1977
sll0406	GO:0044275	<1e-4	sll1373	GO:0006811	<1e-4	slr5016	GO:0050794	<1e-4	sll8035	GO:0005342	<1e-4	sll0995	GO:0016491	0.1977
slr7059	GO:0080090	<1e-4	slr1726	GO:0009262	<1e-4	sll0242	GO:0009126	<1e-4	slr1287	GO:0022892	<1e-4	slr0815	GO:0016491	0.1977
ssr3189	GO:0044260	<1e-4	slr0579	GO:0051234	<1e-4	slr1737	GO:0046907	<1e-4	slr1342	GO:0015075	<1e-4	slr7024	GO:0016491	0.1977
slr0852	GO:0019219	<1e-4	slr1097	GO:0044282	<1e-4	slr0930	GO:0006721	<1e-4	slr0978	GO:0043492	<1e-4	ssl0739	GO:0016491	0.1977
slr1647	GO:0072527	<1e-4	sll0062	GO:0051347	<1e-4	slr1774	GO:0009260	<1e-4	ssl7022	GO:0016818	<1e-4	slr1762	GO:0016491	0.1977
slr1590	GO:0009987	<1e-4	slr7014	GO:0071554	<1e-4	ssr0335	GO:0065007	<1e-4	ssl5114	GO:0060089	<1e-4	slr0924	GO:0016491	0.1977
slr1394	GO:0044281	<1e-4	sll0563	GO:0009144	<1e-4	slr6075	GO:0031323	<1e-4	ssl2384	GO:0042623	<1e-4	slr1259	GO:0016491	0.1977
slr0919	GO:0006163	<1e-4	slr0740	GO:0009056	<1e-4	sll1461	GO:0006753	<1e-4	slr1505	GO:0022857	<1e-4	sll0827	GO:0016491	0.1977
sll1162	GO:0006220	<1e-4	slr1811	GO:0009987	<1e-4	sll1089	GO:0046128	<1e-4	slr1726	GO:0008171	<1e-4	sll0539	GO:0016491	0.1977
sll0449	GO:0006066	<1e-4	sll0436	GO:0006732	<1e-4	ssr7084	GO:0072527	<1e-4	slr7083	GO:0016462	<1e-4	sll7065	GO:0016491	0.1977
sll0400	GO:0044248	<1e-4	slr0104	GO:0043170	<1e-4	slr1301	GO:0009123	<1e-4	sll1583	GO:0043167	<1e-4	ssl8008	GO:0016491	0.1977
slr1753	GO:0043549	<1e-4	slr1998	GO:0051171	<1e-4	sll0615	GO:0006811	<1e-4	slr1396	GO:0022832	<1e-4	slr0111	GO:0016491	0.1977
ssl8003	GO:0022607	<1e-4	slr1235	GO:0044248	<1e-4	slr0667	GO:0016053	<1e-4	ssr1391	GO:0015293	<1e-4	slr1209	GO:0016491	0.1977
slr1530	GO:0006163	<1e-4	ssr1407	GO:0044260	<1e-4	slr1611	GO:0009263	<1e-4	sll1485	GO:0016746	<1e-4	sll0156	GO:0016491	0.1977
slr1644	GO:0050896	<1e-4	sll1608	GO:0009262	<1e-4	sll0350	GO:0034654	<1e-4	slr1315	GO:0022891	<1e-4	slr1128	GO:0016491	0.1977
slr1353	GO:0019637	<1e-4	ssl2807	GO:0009309	<1e-4	slr2060	GO:0048519	<1e-4	sll1289	GO:0016817	<1e-4	slr0104	GO:0016491	0.1977
ssr3410	GO:0050801	<1e-4	sll0702	GO:0006721	<1e-4	sll1252	GO:0044275	<1e-4	slr0355	GO:0060089	<1e-4	sll1304	GO:0016491	0.1977
slr0148	GO:0072524	<1e-4	slr1419	GO:0052111	<1e-4	slr1614	GO:0009889	<1e-4	sll5006	GO:0022836	<1e-4	slr1062	GO:0016491	0.1977
slr1413	GO:0034641	<1e-4	slr0325	GO:0006733	<1e-4	slr0545	GO:0043648	<1e-4	sll1004	GO:0015075	<1e-4	sll0225	GO:0016491	0.1977
slr6081	GO:0006811	<1e-4	slr1900	GO:0034654	<1e-4	sll0281	GO:0046164	<1e-4	slr6009	GO:0022836	<1e-4	slr0172	GO:0016491	0.1977
slr0912	GO:0048878	<1e-4	ssr2848	GO:0009262	<1e-4	sll0609	GO:0006766	<1e-4	slr0514	GO:0022803	<1e-4	sll1040	GO:0016491	0.1977
ssr1375	GO:0051716	<1e-4	ssl3382	GO:0009199	<1e-4	sll1692	GO:0071842	<1e-4	sll1142	GO:0042623	<1e-4	slr1544	GO:0016491	0.1977
sll0552	GO:0019222	<1e-4	slr0092	GO:0009991	<1e-4	sll1060	GO:0009394	<1e-4	slr1541	GO:0022890	<1e-4	slr1862	GO:0016491	0.1977
sll1252	GO:0006631	<1e-4	slr1875	GO:0033013	<1e-4	ssr2803	GO:0043648	<1e-4	ssr2754	GO:0022890	<1e-4	slr0725	GO:0016020	0.1948

sll0101	GO:0009165	<1e-4	ssl3692	GO:0031640	<1e-4	sll0069	GO:0008610	<1e-4	slr1658	GO:0022836	<1e-4	sll0253	GO:0016020	0.1948
sll0198	GO:0044283	<1e-4	slr0887	GO:0051234	<1e-4	sll0405	GO:0009199	<1e-4	sll0858	GO:0015293	<1e-4	slr7094	GO:0016020	0.1948
sll8007	GO:0045184	<1e-4	sll0446	GO:0005996	<1e-4	slr7080	GO:0055086	<1e-4	sll5044	GO:0022838	<1e-4	sll1581	GO:0016020	0.1948
ssr1155	GO:0006732	<1e-4	slr0196	GO:0009308	<1e-4	sll0176	GO:0009987	<1e-4	sll0658	GO:0016818	<1e-4	slr1438	GO:0016020	0.1948
slr0456	GO:0046128	<1e-4	sll1764	GO:0046128	<1e-4	sll0553	GO:0031640	<1e-4	slr0587	GO:0015293	<1e-4	slr1819	GO:0016020	0.1948
slr0871	GO:0016052	<1e-4	slr1263	GO:0034641	<1e-4	slr1315	GO:0019751	<1e-4	ssl5103	GO:0060089	<1e-4	ssr2975	GO:0016020	0.1948
sll0355	GO:0051716	<1e-4	sll0047	GO:0006753	<1e-4	slr0184	GO:0019637	<1e-4	sll0405	GO:0022836	<1e-4	ssr2998	GO:0016020	0.1948
slr6067	GO:0044255	<1e-4	slr7099	GO:0009987	<1e-4	sll1135	GO:0052188	<1e-4	slr0519	GO:0015267	<1e-4	sll7029	GO:0016020	0.1948
sll8040	GO:0051179	<1e-4	slr1624	GO:0006753	<1e-4	sll5032	GO:0050794	<1e-4	slr1276	GO:0015293	<1e-4	slr1935	GO:0016020	0.1948
sll1509	GO:0006082	<1e-4	sll0263	GO:0009308	<1e-4	ssl7038	GO:0043170	<1e-4	sll0068	GO:0016818	<1e-4	ssr1558	GO:0016020	0.1948
sll1912	GO:0048522	<1e-4	sll1022	GO:0009987	<1e-4	slr1474	GO:0006753	<1e-4	sll0413	GO:0022838	<1e-4	slr2103	GO:0016020	0.1948
sll0641	GO:0031640	<1e-4	sll1749	GO:0019751	<1e-4	slr0291	GO:0006631	<1e-4	ssl3379	GO:0015293	<1e-4	slr6012	GO:0016020	0.1948
sll8027	GO:0006091	<1e-4	slr1177	GO:0006811	<1e-4	sll1396	GO:0009263	<1e-4	sll1426	GO:0022857	<1e-4	slr1679	GO:0016020	0.1948
sll1321	GO:0009059	<1e-4	slr7083	GO:0019362	<1e-4	ssr2060	GO:0009056	<1e-4	sll1372	GO:0008171	<1e-4	ssl3573	GO:0016020	0.1948
sll1542	GO:0006793	<1e-4	ssr1499	GO:0052188	<1e-4	ssr6024	GO:0009117	<1e-4	sll0932	GO:0016462	<1e-4	ssr8013	GO:0016020	0.1948
sll0328	GO:0072522	<1e-4	sll1757	GO:0009132	<1e-4	ssr3409	GO:0071841	<1e-4	sll0732	GO:0015399	<1e-4	ssl7074	GO:0016020	0.1948
sll5004	GO:0044003	<1e-4	slr0393	GO:0006811	<1e-4	sll1691	GO:0009259	<1e-4	sll1632	GO:0005342	<1e-4	ssl1263	GO:0016020	0.1948
sll0241	GO:0042180	<1e-4	sll0532	GO:0065007	<1e-4	slr0645	GO:0072521	<1e-4	ssr7093	GO:0022857	<1e-4	slr0064	GO:0016020	0.1948
ssr5106	GO:0072524	<1e-4	sll5069	GO:0009142	<1e-4	slr1307	GO:0006091	<1e-4	slr0625	GO:0015291	<1e-4	slr0184	GO:0016020	0.1948
slr1163	GO:0009987	<1e-4	sll7047	GO:0055086	<1e-4	slr6028	GO:0016053	<1e-4	slr8014	GO:0042623	<1e-4	slr6009	GO:0016020	0.1948
slr1117	GO:0072528	<1e-4	sll1426	GO:0051188	<1e-4	ssl7046	GO:0051186	<1e-4	slr1998	GO:0022838	<1e-4	sll1784	GO:0016020	0.1948
slr0699	GO:0048519	<1e-4	slr0300	GO:0051818	<1e-4	ssl7038	GO:0043933	<1e-4	ssr2843	GO:0042623	<1e-4	sll0839	GO:0016020	0.1948
slr0358	GO:0042180	<1e-4	ssr3154	GO:0009309	<1e-4	slr0810	GO:0016054	<1e-4	sll0297	GO:0008171	<1e-4	ssr3572	GO:0016020	0.1948
slr1737	GO:0050794	<1e-4	ssr6062	GO:0065007	<1e-4	sll0397	GO:0008150	<1e-4	ssr0332	GO:0022836	<1e-4	ssr6002	GO:0016020	0.1948
slr1535	GO:0055114	<1e-4	slr1827	GO:0019752	<1e-4	sll0394	GO:0009142	<1e-4	slr0607	GO:0022803	<1e-4	slr8022	GO:0016020	0.1948
slr1977	GO:0044282	<1e-4	slr1644	GO:0042455	<1e-4	ssr2755	GO:0060255	<1e-4	sll1656	GO:0016746	<1e-4	sll5063	GO:0016020	0.1948
slr0112	GO:0051188	<1e-4	sll6053	GO:0019362	<1e-4	slr7025	GO:0009262	<1e-4	sll0023	GO:0022803	<1e-4	slr0948	GO:0016020	0.1948
slr1215	GO:0048519	<1e-4	slr1070	GO:0051171	<1e-4	ssl5114	GO:0044271	<1e-4	ssr3467	GO:0016746	<1e-4	slr1406	GO:0016020	0.1948
sll0577	GO:0009892	<1e-4	ssl0483	GO:0046128	<1e-4	slr2048	GO:0006732	<1e-4	sll1541	GO:0016741	<1e-4	slr0816	GO:0016020	0.1948
slr0863	GO:0050801	<1e-4	slr0552	GO:0065007	<1e-4	sll1954	GO:0060255	<1e-4	slr0655	GO:0022832	<1e-4	slr0865	GO:0016020	0.1948
ssl7022	GO:0006163	<1e-4	ssr1041	GO:0009308	<1e-4	sll0871	GO:0051188	<1e-4	sll7078	GO:0008171	<1e-4	ssr1473	GO:0016020	0.1948
slr1907	GO:0045184	<1e-4	slr0345	GO:0019637	<1e-4	slr1613	GO:0043170	<1e-4	ssl1918	GO:0008171	<1e-4	ssl3829	GO:0016020	0.1948
sll8027	GO:0071842	<1e-4	slr1169	GO:0044271	<1e-4	sll0031	GO:0065007	<1e-4	slr0386	GO:0015291	<1e-4	sll7089	GO:0016020	0.1948
slr0689	GO:0034654	<1e-4	sll1737	GO:0044248	<1e-4	sll0785	GO:0031323	<1e-4	ssl5114	GO:0005342	<1e-4	sll1340	GO:0016020	0.1948
slr7097	GO:0009056	<1e-4	sll0843	GO:0009161	<1e-4	ssr1114	GO:0051171	<1e-4	sll1730	GO:0016818	<1e-4	ssr8047	GO:0016020	0.1948
slr1056	GO:0009893	<1e-4	sll1285	GO:0009262	<1e-4	slr0376	GO:0016054	<1e-4	sll0775	GO:0060089	<1e-4	ssl8039	GO:0016020	0.1948
slr1170	GO:0019219	<1e-4	sll0822	GO:0048518	<1e-4	sll0843	GO:0009991	<1e-4	slr1173	GO:0042623	<1e-4	slr1752	GO:0016020	0.1948
sll1652	GO:0034660	<1e-4	sll0183	GO:0055114	<1e-4	slr1174	GO:0046164	<1e-4	sll1390	GO:0022803	<1e-4	sll1307	GO:0016020	0.1948
slr0590	GO:0006091	<1e-4	slr0376	GO:0043549	<1e-4	sll1166	GO:0048519	<1e-4	slr1768	GO:0015291	<1e-4	sll7028	GO:0016020	0.1948
slr1177	GO:0009260	<1e-4	sll1399	GO:0006091	<1e-4	slr6044	GO:0071496	<1e-4	sll0982	GO:0016741	<1e-4	ssr5011	GO:0016020	0.1948
slr0789	GO:0016054	<1e-4	sll0781	GO:0010468	<1e-4	slr6100	GO:0051179	<1e-4	slr0725	GO:0016746	<1e-4	sll0608	GO:0016020	0.1948
slr0468	GO:0072522	<1e-4	sll0266	GO:0055114	<1e-4	slr2125	GO:0055082	<1e-4	slr0702	GO:0015405	<1e-4	ssl7051	GO:0016020	0.1948
slr1203	GO:0009123	<1e-4	slr1702	GO:0072527	<1e-4	sll1938	GO:0009892	<1e-4	slr0651	GO:0016817	<1e-4	slr5101	GO:0016020	0.1948
sll1411	GO:0044260	<1e-4	sll1086	GO:0009117	<1e-4	sll1956	GO:0009892	<1e-4	slr1070	GO:0008171	<1e-4	ssl2384	GO:0016020	0.1948
slr0303	GO:0051179	<1e-4	sll1495	GO:0006733	<1e-4	slr7015	GO:0055086	<1e-4	sll0742	GO:0022890	<1e-4	slr1670	GO:0016020	0.1948
slr0725	GO:0071841	<1e-4	sll0446	GO:0042451	<1e-4	ssr6019	GO:0031323	<1e-4	slr1045	GO:0015077	<1e-4	slr6016	GO:0016020	0.1948
slr7057	GO:0042180	<1e-4	ssl3291	GO:0051171	<1e-4	ssr1258	GO:0042180	<1e-4	slr1095	GO:0043167	<1e-4	sll1388	GO:0016020	0.1948
slr1944	GO:0019752	<1e-4	sll0863	GO:0072521	<1e-4	slr5102	GO:0034641	<1e-4	slr1956	GO:0005342	<1e-4	sll1726	GO:0016020	0.1948
slr1865	GO:0071842	<1e-4	sll0743	GO:0033013	<1e-4	ssl3829	GO:0051246	<1e-4	sll1151	GO:0016818	<1e-4	sll0670	GO:0016020	0.1948
sll6052	GO:0042451	<1e-4	sll0552	GO:0006721	<1e-4	slr1624	GO:0046164	<1e-4	sll0641	GO:0022836	<1e-4	ssl7038	GO:0016020	0.1948
sll1656	GO:0009126	<1e-4	ssr2848	GO:0055086	<1e-4	slr0169	GO:0009308	<1e-4	sll0044	GO:0015291	<1e-4	sll1702	GO:0016020	0.1948
slr0554	GO:0019751	<1e-4	slr0303	GO:0006811	<1e-4	slr6103	GO:0071840	<1e-4	slr1927	GO:0022891	<1e-4	slr1599	GO:0016020	0.1948
sll1884	GO:0009991	<1e-4	slr5018	GO:0050794	<1e-4	slr0358	GO:0048523	<1e-4	slr7080	GO:0016818	<1e-4	ssr1425	GO:0016020	0.1948
slr0241	GO:0043436	<1e-4	sll2007	GO:0009308	<1e-4	ssl5113	GO:0009309	<1e-4	slr1376	GO:0022892	<1e-4	slr1624	GO:0016020	0.1948

ssl2807	GO:0043549	<1e-4	ssl0461	GO:0044275	<1e-4	sll5028	GO:0009199	<1e-4	slr1530	GO:0043167	<1e-4	slr0313	GO:0016020	0.1948
sll0867	GO:0034654	<1e-4	sll1254	GO:0019752	<1e-4	sll1222	GO:0006766	<1e-4	sll7066	GO:0015399	<1e-4	sll0335	GO:0016020	0.1948
ssl2069	GO:0009394	<1e-4	sll0208	GO:0009308	<1e-4	sll1160	GO:0008150	<1e-4	sll0192	GO:0016741	<1e-4	sll0327	GO:0016020	0.1948
ssr5092	GO:0006811	<1e-4	ssl1378	GO:0016053	<1e-4	slr1668	GO:0051171	<1e-4	ssl7046	GO:0022803	<1e-4	slr1951	GO:0016020	0.1948
sll0785	GO:0052188	<1e-4	slr6090	GO:0080090	<1e-4	ssr1528	GO:0071554	<1e-4	slr0148	GO:0022838	<1e-4	sll0060	GO:0016020	0.1948
sll0423	GO:0019751	<1e-4	sll0444	GO:0044106	<1e-4	slr1262	GO:0033013	<1e-4	sll1526	GO:0015267	<1e-4	ssr6089	GO:0016020	0.1948
ssl0242	GO:0048523	<1e-4	slr0168	GO:0046164	<1e-4	slr0250	GO:0009144	<1e-4	sll1355	GO:0008171	<1e-4	slr0728	GO:0016020	0.1948
ssl7022	GO:0051818	<1e-4	slr1659	GO:0009124	<1e-4	sll0737	GO:0034641	<1e-4	slr1809	GO:0016741	<1e-4	ssr1155	GO:0016020	0.1948
ssl1690	GO:0006811	<1e-4	slr0392	GO:0072522	<1e-4	slr1917	GO:0071842	<1e-4	sll0412	GO:0003674	<1e-4	slr2070	GO:0016020	0.1948
sll0062	GO:0052111	<1e-4	slr1362	GO:0034660	<1e-4	slr0731	GO:0048522	<1e-4	ssr1258	GO:0022857	<1e-4	sll1476	GO:0016020	0.1948
slr1162	GO:0043933	<1e-4	sll0478	GO:0009889	<1e-4	sll0274	GO:0071496	<1e-4	ssl2064	GO:0016746	<1e-4	sll1926	GO:0016020	0.1948
slr6014	GO:0006753	<1e-4	sll0630	GO:0008150	<1e-4	sll0268	GO:0043549	<1e-4	slr7012	GO:0016741	<1e-4	slr0458	GO:0016020	0.1948
ssl7046	GO:0046128	<1e-4	slr1880	GO:0019751	<1e-4	slr1087	GO:0016053	<1e-4	slr0605	GO:0042623	<1e-4	sll0376	GO:0016020	0.1948
slr7016	GO:0071496	<1e-4	slr0909	GO:0044281	<1e-4	slr1932	GO:0009123	<1e-4	slr0456	GO:0015293	<1e-4	ssr5106	GO:0016020	0.1948
sll7063	GO:0080090	<1e-4	slr1611	GO:0006766	<1e-4	ssr6079	GO:0043170	<1e-4	sll0010	GO:0015405	<1e-4	slr0731	GO:0016020	0.1948
sll0585	GO:0006066	<1e-4	slr1535	GO:0043436	<1e-4	slr0238	GO:0031326	<1e-4	sll0932	GO:0016818	<1e-4	slr6088	GO:0016020	0.1948
slr0978	GO:0006220	<1e-4	ssl0312	GO:0055114	<1e-4	slr2038	GO:0009987	<1e-4	sll0185	GO:0043492	<1e-4	ssl7045	GO:0016020	0.1948
slr1572	GO:0009059	<1e-4	slr0592	GO:0009394	<1e-4	slr6006	GO:0051186	<1e-4	slr1338	GO:0016817	<1e-4	sll1285	GO:0016020	0.1948
slr0503	GO:0009892	<1e-4	sll0837	GO:0046907	<1e-4	slr1098	GO:0043648	<1e-4	slr6006	GO:0022857	<1e-4	slr1570	GO:0016020	0.1948
sll0443	GO:0044003	<1e-4	ssr6062	GO:0071840	<1e-4	sll1527	GO:0051716	<1e-4	sll0185	GO:0008171	<1e-4	sll1158	GO:0016020	0.1948
sll1424	GO:0009991	<1e-4	slr6022	GO:0044003	<1e-4	slr1917	GO:0046128	<1e-4	slr0151	GO:0042623	<1e-4	ssr7084	GO:0016020	0.1948
slr7011	GO:0050794	<1e-4	ssr1425	GO:0050789	<1e-4	sll0611	GO:0055114	<1e-4	ssl2064	GO:0015405	<1e-4	slr0634	GO:0016020	0.1948
sll0553	GO:0050794	<1e-4	slr6088	GO:0045184	<1e-4	sll0786	GO:0052188	<1e-4	slr1780	GO:0042623	<1e-4	sll0925	GO:0016020	0.1948
slr1970	GO:0048522	<1e-4	sll0735	GO:0001932	<1e-4	slr1363	GO:0006631	<1e-4	sll0294	GO:0042623	<1e-4	slr1627	GO:0016020	0.1948
sll1135	GO:0051716	<1e-4	sll0585	GO:0051246	<1e-4	sll1372	GO:0009150	<1e-4	ssr1473	GO:0003674	<1e-4	ssl3549	GO:0016020	0.1948
sll1761	GO:0034641	<1e-4	sll1433	GO:0019637	<1e-4	slr0957	GO:0034654	<1e-4	sll6055	GO:0005342	<1e-4	sll0044	GO:0016020	0.1948
slr2038	GO:0019219	<1e-4	slr1681	GO:0016054	<1e-4	slr1436	GO:0009893	<1e-4	slr1062	GO:0015293	<1e-4	ssr6019	GO:0016020	0.1948
sll0933	GO:0009165	<1e-4	slr0625	GO:0046483	<1e-4	slr0890	GO:0080090	<1e-4	sll1348	GO:0016741	<1e-4	sll1547	GO:0016020	0.1948
sll0846	GO:0072521	<1e-4	sll0296	GO:0044248	<1e-4	sll0577	GO:0046128	<1e-4	slr1721	GO:0022838	<1e-4	slr6087	GO:0016020	0.1948
slr0496	GO:0048522	<1e-4	slr1906	GO:0019752	<1e-4	sll1532	GO:0005996	<1e-4	slr0921	GO:0008171	<1e-4	slr0709	GO:0016020	0.1948
slr1563	GO:0044281	<1e-4	slr1557	GO:0009141	<1e-4	slr1071	GO:0048523	<1e-4	slr0960	GO:0022892	<1e-4	sll0157	GO:0016020	0.1948
slr1222	GO:0072521	<1e-4	ssl0312	GO:0042451	<1e-4	sll0487	GO:0019362	<1e-4	slr5087	GO:0015077	<1e-4	slr1241	GO:0016020	0.1948
slr1183	GO:0006163	<1e-4	slr1603	GO:0009262	<1e-4	sll1528	GO:0022411	<1e-4	ssl2148	GO:0042623	<1e-4	sll0062	GO:0016020	0.1948
slr1667	GO:0050789	<1e-4	sll1541	GO:0009056	<1e-4	slr0065	GO:0051234	<1e-4	slr5037	GO:0016741	<1e-4	sll1106	GO:0016020	0.1948
slr0815	GO:0009142	<1e-4	slr6014	GO:0043549	<1e-4	slr1959	GO:0043412	<1e-4	slr1535	GO:0015075	<1e-4	slr1623	GO:0016020	0.1948
sll1601	GO:0009263	<1e-4	slr0059	GO:0006631	<1e-4	ssr3409	GO:0009165	<1e-4	slr0668	GO:0022890	<1e-4	slr0590	GO:0016020	0.1948
ssr2802	GO:0051246	<1e-4	slr0151	GO:0051234	<1e-4	slr0784	GO:0019219	<1e-4	ssr5020	GO:0005342	<1e-4	slr1886	GO:0016020	0.1948
ssr6083	GO:0048878	<1e-4	slr2103	GO:0031323	<1e-4	ssr1473	GO:0006091	<1e-4	slr0723	GO:0022832	<1e-4	sll1254	GO:0016020	0.1948
slr6015	GO:0046128	<1e-4	slr0709	GO:0072522	<1e-4	sll1630	GO:0044260	<1e-4	sll1783	GO:0043492	<1e-4	slr8021	GO:0016020	0.1948
slr0065	GO:0072524	<1e-4	slr0423	GO:0031326	<1e-4	slr6063	GO:0009892	<1e-4	slr2012	GO:0016462	<1e-4	sll5069	GO:0016020	0.1948
slr1378	GO:0043933	<1e-4	sll0981	GO:0006721	<1e-4	slr1210	GO:0043933	<1e-4	slr1240	GO:0022890	<1e-4	ssr6086	GO:0016020	0.1948
slr0060	GO:0042455	<1e-4	ssr2802	GO:0060255	<1e-4	sll1006	GO:0051188	<1e-4	sll0314	GO:0015293	<1e-4	ssl5025	GO:0016020	0.1948
slr1964	GO:0044106	<1e-4	sll5003	GO:0009309	<1e-4	slr2111	GO:0046394	<1e-4	slr5053	GO:0016741	<1e-4	sll1289	GO:0016020	0.1948
slr1541	GO:0009059	<1e-4	ssl3692	GO:0044255	<1e-4	sll1681	GO:0009394	<1e-4	slr5037	GO:0022832	<1e-4	sll1698	GO:0016020	0.1948
sll8040	GO:0008150	<1e-4	ssr2975	GO:0043549	<1e-4	ssr3189	GO:0018130	<1e-4	slr1168	GO:0003674	<1e-4	ssl5091	GO:0016020	0.1948
slr0689	GO:0006066	<1e-4	slr7094	GO:0022411	<1e-4	sll1232	GO:0044248	<1e-4	slr0325	GO:0043167	<1e-4	slr1079	GO:0016020	0.1948
slr1956	GO:0009259	<1e-4	sll1509	GO:0055114	<1e-4	slr0650	GO:0006733	<1e-4	slr6021	GO:0042623	<1e-4	slr0771	GO:0016020	0.1948
slr0941	GO:0044281	<1e-4	sll0448	GO:0043412	<1e-4	slr5024	GO:0009262	<1e-4	sll0765	GO:0022892	<1e-4	ssr2060	GO:0016020	0.1948
sll1926	GO:0006721	<1e-4	sll1891	GO:0006091	<1e-4	sll1995	GO:0044283	<1e-4	slr0479	GO:0015075	<1e-4	sll7030	GO:0016020	0.1948
slr1576	GO:0009309	<1e-4	sll0596	GO:0044003	<1e-4	slr2118	GO:0019751	<1e-4	ssr6032	GO:0016741	<1e-4	slr5115	GO:0016020	0.1948
slr2027	GO:0001932	<1e-4	slr1484	GO:0050794	<1e-4	slr2120	GO:0043549	<1e-4	ssl5108	GO:0003674	<1e-4	slr1206	GO:0016020	0.1948
slr6104	GO:0048523	<1e-4	slr0806	GO:0072524	<1e-4	sll7047	GO:0044255	<1e-4	sll1225	GO:0015405	<1e-4	slr6071	GO:0016020	0.1948
slr5111	GO:0009126	<1e-4	sll0760	GO:0046164	<1e-4	sll1049	GO:0042451	<1e-4	ssr3189	GO:0016746	<1e-4	ssl7048	GO:0016020	0.1948
sll1925	GO:0033013	<1e-4	sll1131	GO:0001932	<1e-4	sll0645	GO:0022607	<1e-4	sll8004	GO:0043492	<1e-4	sll1583	GO:0016020	0.1948

ssl2069	GO:0034660	<1e-4	ssl2996	GO:0052111	<1e-4	sll1882	GO:0044282	<1e-4	sll1960	GO:0016818	<1e-4	sll0230	GO:0016020	0.1948
sll0249	GO:0048518	<1e-4	slr1704	GO:0051716	<1e-4	slr5111	GO:0034654	<1e-4	slr0907	GO:0015293	<1e-4	sll1495	GO:0016020	0.1948
slr1095	GO:0016053	<1e-4	slr0964	GO:0009142	<1e-4	slr7102	GO:0048518	<1e-4	sll1757	GO:0015267	<1e-4	sll1832	GO:0016020	0.1948
slr0423	GO:0006733	<1e-4	slr0207	GO:0009161	<1e-4	ssr7084	GO:0044282	<1e-4	slr2122	GO:0022890	<1e-4	slr1807	GO:0016020	0.1948
slr0692	GO:0060255	<1e-4	slr0770	GO:0072521	<1e-4	sll1348	GO:0050801	<1e-4	slr1623	GO:0015405	<1e-4	slr7060	GO:0016020	0.1948
slr5126	GO:0010468	<1e-4	sll0539	GO:0050794	<1e-4	slr0366	GO:0071840	<1e-4	slr0625	GO:0016817	<1e-4	slr1537	GO:0016020	0.1948
sll0984	GO:0009132	<1e-4	slr5024	GO:0009199	<1e-4	sll1906	GO:0009199	<1e-4	sll1203	GO:0060089	<1e-4	sll1319	GO:0016020	0.1948
sll1715	GO:0043933	<1e-4	sll0294	GO:0006631	<1e-4	slr1083	GO:0051171	<1e-4	slr0058	GO:0015293	<1e-4	sll0847	GO:0016020	0.1948
sll1573	GO:0022607	<1e-4	slr1616	GO:0044106	<1e-4	slr1084	GO:0048518	<1e-4	ssr6026	GO:0005342	<1e-4	slr1170	GO:0016020	0.1948
sll7064	GO:0046483	<1e-4	slr1150	GO:0009142	<1e-4	sll1225	GO:0019637	<1e-4	ssr6079	GO:0015293	<1e-4	slr5127	GO:0016020	0.1948
ssl2749	GO:0008610	<1e-4	slr0865	GO:0009308	<1e-4	slr1288	GO:0006631	<1e-4	slr0812	GO:0022832	<1e-4	ssl2138	GO:0016020	0.1948
slr1441	GO:0016054	<1e-4	slr1052	GO:0071554	<1e-4	slr1752	GO:0046907	<1e-4	slr1083	GO:0022836	<1e-4	ssl2595	GO:0016020	0.1948
slr7094	GO:0031323	<1e-4	slr7101	GO:0019752	<1e-4	sll0659	GO:0046483	<1e-4	sll0325	GO:0022892	<1e-4	slr0610	GO:0016020	0.1948
slr6087	GO:0043648	<1e-4	slr0554	GO:0006721	<1e-4	slr6090	GO:0009199	<1e-4	slr0569	GO:0015291	<1e-4	sll1531	GO:0016020	0.1948
slr2118	GO:0031323	<1e-4	slr2121	GO:0044282	<1e-4	slr2025	GO:0051188	<1e-4	sll0252	GO:0043492	<1e-4	slr7026	GO:0016020	0.1948
slr1450	GO:0009165	<1e-4	ssr5074	GO:0006091	<1e-4	slr0317	GO:0006811	<1e-4	slr1207	GO:0016817	<1e-4	sll1640	GO:0016020	0.1948
slr0393	GO:0009259	<1e-4	sll1486	GO:0065007	<1e-4	ssr0692	GO:0033013	<1e-4	sll0283	GO:0005342	<1e-4	sll0543	GO:0016020	0.1948
ssl8028	GO:0006631	<1e-4	sll1630	GO:0051179	<1e-4	ssr6027	GO:0009144	<1e-4	sll0102	GO:0016818	<1e-4	slr6068	GO:0016020	0.1948
sll1934	GO:0052188	<1e-4	sll1654	GO:0016052	<1e-4	slr0514	GO:0018130	<1e-4	sll1570	GO:0016818	<1e-4	slr0656	GO:0016020	0.1948
slr1690	GO:0009150	<1e-4	sgl0002	GO:0006163	<1e-4	sll0428	GO:0051186	<1e-4	slr1780	GO:0008171	<1e-4	slr2027	GO:0016020	0.1948
sll0584	GO:0072528	<1e-4	slr1235	GO:0046128	<1e-4	slr0651	GO:0044275	<1e-4	slr6064	GO:0022890	<1e-4	sll0749	GO:0016020	0.1948
ssr2615	GO:0043412	<1e-4	sll5046	GO:0071841	<1e-4	sll0410	GO:0055114	<1e-4	slr5102	GO:0022857	<1e-4	sll1714	GO:0016020	0.1948
sll1773	GO:0006766	<1e-4	sll0822	GO:0008150	<1e-4	sll1225	GO:0044281	<1e-4	slr0923	GO:0022857	<1e-4	slr1174	GO:0016020	0.1948
slr1613	GO:0046907	<1e-4	sll0563	GO:0009150	<1e-4	sll0661	GO:0055086	<1e-4	slr0145	GO:0015405	<1e-4	sll7090	GO:0016020	0.1948
slr0625	GO:0080090	<1e-4	ssr1258	GO:0071496	<1e-4	slr1788	GO:0042180	<1e-4	slr0723	GO:0022836	<1e-4	slr5053	GO:0016020	0.1948
slr1262	GO:0080090	<1e-4	sll1692	GO:0044106	<1e-4	slr1209	GO:0044003	<1e-4	sll0676	GO:0016818	<1e-4	ssl5113	GO:0016020	0.1948
sll0181	GO:0051716	<1e-4	slr1932	GO:0009309	<1e-4	ssr2317	GO:0009150	<1e-4	sll0242	GO:0022803	<1e-4	ssl3615	GO:0016020	0.1948
slr7026	GO:0009263	<1e-4	sll0172	GO:0006732	<1e-4	slr0871	GO:0009991	<1e-4	slr1384	GO:0022803	<1e-4	sll1095	GO:0016020	0.1948
slr6071	GO:0006220	<1e-4	sll0498	GO:0005996	<1e-4	slr1391	GO:0048519	<1e-4	ssr5092	GO:0022857	<1e-4	sll1151	GO:0016020	0.1948
ssl2148	GO:0016052	<1e-4	ssl2733	GO:0044260	<1e-4	slr2032	GO:0050801	<1e-4	slr1437	GO:0015405	<1e-4	sll0172	GO:0016020	0.1948
sll0608	GO:0072524	<1e-4	slr0517	GO:0009263	<1e-4	slr1614	GO:0046394	<1e-4	slr1789	GO:0015405	<1e-4	ssr7017	GO:0016020	0.1948
sll1455	GO:0034641	<1e-4	sll0272	GO:0046164	<1e-4	slr1179	GO:0009123	<1e-4	slr2025	GO:0015405	<1e-4	ssr0759	GO:0016020	0.1948
slr0670	GO:0051234	<1e-4	sll1925	GO:0044248	<1e-4	ssl3615	GO:0050896	<1e-4	sll5130	GO:0043492	<1e-4	slr1507	GO:0016020	0.1948
slr6071	GO:0009126	<1e-4	slr1384	GO:0009123	<1e-4	sll1654	GO:0072524	<1e-4	slr1657	GO:0042623	<1e-4	ssl1378	GO:0016020	0.1948
sll0269	GO:0018130	<1e-4	slr0263	GO:0006733	<1e-4	ssr2843	GO:0065007	<1e-4	slr0440	GO:0005342	<1e-4	slr0588	GO:0016020	0.1948
sll0192	GO:0006766	<1e-4	sll1640	GO:0051234	<1e-4	ssl0353	GO:0042180	<1e-4	sll0857	GO:0005342	<1e-4	ssl7021	GO:0016020	0.1948
sll7069	GO:0001932	<1e-4	sll1608	GO:0009889	<1e-4	sll1526	GO:0055082	<1e-4	slr6015	GO:0022836	<1e-4	sll1415	GO:0016020	0.1948
sll2007	GO:0046164	<1e-4	slr1342	GO:0051818	<1e-4	sll0552	GO:0043436	<1e-4	slr7082	GO:0043167	<1e-4	slr1704	GO:0016020	0.1948
sll0369	GO:0009893	<1e-4	slr0863	GO:0090304	<1e-4	slr2118	GO:0043170	<1e-4	sll0793	GO:0042623	<1e-4	slr1911	GO:0016020	0.1948
sll0188	GO:0006220	<1e-4	slr7101	GO:0048523	<1e-4	sll0905	GO:0034641	<1e-4	slr1895	GO:0015267	<1e-4	sll1164	GO:0016020	0.1948
slr0376	GO:0042451	<1e-4	ssr1558	GO:0009056	<1e-4	slr0060	GO:0008150	<1e-4	slr0455	GO:0015075	<1e-4	ssl7007	GO:0016020	0.1948
slr1807	GO:0071496	<1e-4	slr0689	GO:0034641	<1e-4	slr1210	GO:0009892	<1e-4	slr1813	GO:0015399	<1e-4	sll0552	GO:0016020	0.1948
slr0325	GO:0071840	<1e-4	ssl5098	GO:0055114	<1e-4	slr6072	GO:0016052	<1e-4	sll7034	GO:0022836	<1e-4	sll1681	GO:0016020	0.1948
sll0590	GO:0045184	<1e-4	sll1267	GO:0072524	<1e-4	slr6007	GO:0042451	<1e-4	ssl2148	GO:0016741	<1e-4	sll1464	GO:0016020	0.1948
sll1355	GO:0009893	<1e-4	slr1384	GO:0009309	<1e-4	slr0496	GO:0006811	<1e-4	sll1714	GO:0022836	<1e-4	sll0405	GO:0016020	0.1948
slr1907	GO:0009161	<1e-4	sll6054	GO:0009056	<1e-4	ssr7017	GO:0022607	<1e-4	slr1896	GO:0043167	<1e-4	ssr0109	GO:0016020	0.1948
slr0146	GO:0048878	<1e-4	slr1438	GO:0016052	<1e-4	slr0937	GO:0016054	<1e-4	sll0442	GO:0060089	<1e-4	slr6075	GO:0016020	0.1948
sll0743	GO:0045184	<1e-4	ssr6048	GO:0019362	<1e-4	slr1087	GO:0009165	<1e-4	slr1647	GO:0022803	<1e-4	sll5004	GO:0016020	0.1948
sll0102	GO:0065007	<1e-4	sll1239	GO:0043549	<1e-4	sll0328	GO:0009059	<1e-4	slr0890	GO:0015405	<1e-4	ssl7022	GO:0016020	0.1948
slr0287	GO:0006066	<1e-4	slr0416	GO:0006066	<1e-4	sll1004	GO:0044275	<1e-4	slr0039	GO:0060089	<1e-4	slr1396	GO:0016020	0.1948
sll1651	GO:0046164	<1e-4	sll0280	GO:0006733	<1e-4	slr0579	GO:0055114	<1e-4	slr6015	GO:0016817	<1e-4	slr5087	GO:0016020	0.1948
slr1886	GO:0050801	<1e-4	slr1799	GO:0046128	<1e-4	ssl1255	GO:0051818	<1e-4	ssr5121	GO:0015293	<1e-4	slr1780	GO:0016020	0.1948
slr2080	GO:0006163	<1e-4	ssr5074	GO:0006721	<1e-4	sll1173	GO:0071554	<1e-4	sll1476	GO:0016741	<1e-4	slr0303	GO:0016020	0.1948
slr1056	GO:0071554	<1e-4	slr7012	GO:0051818	<1e-4	sll1609	GO:0006811	<1e-4	slr5126	GO:0042623	<1e-4	sll1785	GO:0016020	0.1948

slr0642	GO:0009126	<1e-4	slr0407	GO:0009308	<1e-4	slr1788	GO:0071496	<1e-4	slr1570	GO:0015293	<1e-4	slr2052	GO:0016020	0.1948
sll1219	GO:0009161	<1e-4	slr1814	GO:0046483	<1e-4	slr0489	GO:0043549	<1e-4	slr1957	GO:0015291	<1e-4	slr0755	GO:0016020	0.1948
slr7082	GO:0072524	<1e-4	ssr2912	GO:0044275	<1e-4	slr1563	GO:0008610	<1e-4	ssr2318	GO:0022836	<1e-4	sll1380	GO:0016020	0.1948
sll7086	GO:0052188	<1e-4	sll0022	GO:0048518	<1e-4	slr5017	GO:0046907	<1e-4	slr0304	GO:0022838	<1e-4	slr1601	GO:0016020	0.1948
sll0586	GO:0019222	<1e-4	slr0483	GO:0051716	<1e-4	ssr0102	GO:0044271	<1e-4	sll1233	GO:0015075	<1e-4	sll0249	GO:0016020	0.1948
sll1372	GO:0044283	<1e-4	slr0334	GO:0048522	<1e-4	ssl1792	GO:0009144	<1e-4	sll1061	GO:0015077	<1e-4	sll0160	GO:0016020	0.1948
slr0870	GO:0010468	<1e-4	slr1513	GO:0072524	<1e-4	sll0996	GO:0060255	<1e-4	slr1203	GO:0008171	<1e-4	slr6033	GO:0016020	0.1948
slr0356	GO:0009117	<1e-4	sll7077	GO:0022411	<1e-4	sll0661	GO:0044248	<1e-4	ssr1375	GO:0015075	<1e-4	ssl5108	GO:0016020	0.1948
ssr2802	GO:0034641	<1e-4	sll0328	GO:0055114	<1e-4	slr0393	GO:0071841	<1e-4	sll8033	GO:0022832	<1e-4	slr6045	GO:0016020	0.1948
sll0023	GO:0022411	<1e-4	slr0581	GO:0034641	<1e-4	sll1702	GO:0055082	<1e-4	sll1832	GO:0015267	<1e-4	slr0878	GO:0016020	0.1948
slr1906	GO:0006082	<1e-4	slr7011	GO:0005996	<1e-4	slr1223	GO:0009141	<1e-4	ssr2554	GO:0022838	<1e-4	slr6029	GO:0016020	0.1948
slr0269	GO:0042180	<1e-4	slr0964	GO:0019219	<1e-4	slr0431	GO:0043549	<1e-4	slr0598	GO:0022836	<1e-4	ssl6061	GO:0016020	0.1948
ssr6030	GO:0008150	<1e-4	slr1886	GO:0051246	<1e-4	ssl3573	GO:0080090	<1e-4	sll7006	GO:0022890	<1e-4	sll1692	GO:0016020	0.1948
slr1484	GO:0006732	<1e-4	slr1034	GO:0055114	<1e-4	slr0294	GO:0051716	<1e-4	sll1783	GO:0042623	<1e-4	sll5128	GO:0016020	0.1948
ssr7017	GO:0006066	<1e-4	slr6073	GO:0034641	<1e-4	slr0650	GO:0046483	<1e-4	slr0106	GO:0016818	<1e-4	ssl2971	GO:0016020	0.1948
slr1658	GO:0051171	<1e-4	slr0416	GO:0018130	<1e-4	slr0989	GO:0042455	<1e-4	sll1162	GO:0022892	<1e-4	sll8019	GO:0016020	0.1948
slr0869	GO:0044275	<1e-4	ssl1552	GO:0051179	<1e-4	slr0575	GO:0080090	<1e-4	slr1920	GO:0015405	<1e-4	ssr3570	GO:0016020	0.1948
slr0082	GO:0043412	<1e-4	slr1636	GO:0006753	<1e-4	sll0498	GO:0006082	<1e-4	sll0252	GO:0043167	<1e-4	slr1875	GO:0016020	0.1948
sll1251	GO:0043933	<1e-4	sll0008	GO:0008150	<1e-4	ssr1766	GO:0044281	<1e-4	ssl3692	GO:0003674	<1e-4	slr0273	GO:0016020	0.1948
sll0676	GO:0034654	<1e-4	sll0609	GO:0009889	<1e-4	sll0102	GO:0051171	<1e-4	sll0615	GO:0015405	<1e-4	sll1532	GO:0016020	0.1948
slr6049	GO:0019751	<1e-4	slr1235	GO:0005996	<1e-4	slr6031	GO:0022411	<1e-4	slr5101	GO:0022892	<1e-4	slr0517	GO:0016020	0.1948
slr0937	GO:0006631	<1e-4	sll1002	GO:0006766	<1e-4	sll5069	GO:0046128	<1e-4	sll1247	GO:0015075	<1e-4	sll0525	GO:0016020	0.1948
slr1780	GO:0009059	<1e-4	sll1273	GO:0071554	<1e-4	slr1376	GO:0051818	<1e-4	slr0709	GO:0022890	<1e-4	sll7087	GO:0016020	0.1948
sll1054	GO:0009892	<1e-4	slr0468	GO:0044003	<1e-4	sll0609	GO:0008610	<1e-4	sll0630	GO:0016462	<1e-4	slr5012	GO:0016020	0.1948
slr1726	GO:0031640	<1e-4	slr1704	GO:0044283	<1e-4	ssl5100	GO:0009123	<1e-4	sll0419	GO:0022832	<1e-4	sll1891	GO:0016020	0.1948
sll1414	GO:0055086	<1e-4	sll1979	GO:0019219	<1e-4	sll1939	GO:0006811	<1e-4	slr0468	GO:0022803	<1e-4	slr0416	GO:0016020	0.1948
ssl7048	GO:0042455	<1e-4	slr0579	GO:0071841	<1e-4	slr0207	GO:0009262	<1e-4	slr1288	GO:0042623	<1e-4	slr6022	GO:0016020	0.1948
sll0031	GO:0022411	<1e-4	slr0642	GO:0071842	<1e-4	slr0637	GO:0034641	<1e-4	ssr1698	GO:0022857	<1e-4	slr7073	GO:0016020	0.1948
sll5130	GO:0009124	<1e-4	slr1223	GO:0051716	<1e-4	slr0731	GO:0043933	<1e-4	ssr2317	GO:0015291	<1e-4	sll8012	GO:0016020	0.1948
slr0148	GO:0055114	<1e-4	sll0102	GO:0071840	<1e-4	slr1778	GO:0009117	<1e-4	sll0645	GO:0015291	<1e-4	slr0325	GO:0016020	0.1948
slr0059	GO:0050801	<1e-4	sll1542	GO:0043412	<1e-4	slr1570	GO:0044271	<1e-4	ssl2814	GO:0008171	<1e-4	sll0858	GO:0016020	0.1948
slr1290	GO:0051188	<1e-4	slr0959	GO:0048522	<1e-4	sll0446	GO:0009124	<1e-4	slr1353	GO:0015399	<1e-4	slr1681	GO:0016020	0.1948
slr1614	GO:0006163	<1e-4	ssr3122	GO:0006576	<1e-4	ssr2318	GO:0043170	<1e-4	slr1194	GO:0060089	<1e-4	slr7100	GO:0016020	0.1948
sll0602	GO:0071840	<1e-4	slr1142	GO:0019219	<1e-4	slr2144	GO:0009987	<1e-4	ssl0750	GO:0022838	<1e-4	slr1114	GO:0016020	0.1948
slr1495	GO:0031323	<1e-4	slr6091	GO:0031640	<1e-4	slr1813	GO:0006733	<1e-4	slr0610	GO:0022838	<1e-4	slr1236	GO:0016020	0.1948
sll0174	GO:0006576	<1e-4	slr0023	GO:0051246	<1e-4	sll0925	GO:0046483	<1e-4	slr1122	GO:0043167	<1e-4	ssr6083	GO:0016020	0.1948
slr1034	GO:0022411	<1e-4	ssl3291	GO:0043648	<1e-4	slr7016	GO:0009124	<1e-4	slr8044	GO:0015267	<1e-4	slr1142	GO:0016020	0.1948
slr1970	GO:0048518	<1e-4	slr1940	GO:0072521	<1e-4	slr0273	GO:0005996	<1e-4	slr7098	GO:0003674	<1e-4	slr2042	GO:0016020	0.1948
sll1773	GO:0019752	<1e-4	ssl2138	GO:0044260	<1e-4	ssl2162	GO:0044106	<1e-4	sll0381	GO:0022891	<1e-4	sll8040	GO:0016020	0.1948
sll1289	GO:0019222	<1e-4	sll7064	GO:0051716	<1e-4	slr0668	GO:0048523	<1e-4	slr5116	GO:0060089	<1e-4	sll0736	GO:0016020	0.1948
slr0656	GO:0044248	<1e-4	slr1053	GO:0055114	<1e-4	sll1751	GO:0051234	<1e-4	slr0263	GO:0043492	<1e-4	slr2117	GO:0016020	0.1948
sll1433	GO:0031640	<1e-4	slr7073	GO:0044248	<1e-4	sll1950	GO:0009126	<1e-4	slr1593	GO:0016818	<1e-4	slr0587	GO:0016020	0.1948
sll7064	GO:0048519	<1e-4	sll0853	GO:0044275	<1e-4	ssr1499	GO:0006793	<1e-4	slr0273	GO:0015267	<1e-4	slr0643	GO:0016020	0.1948
sll1834	GO:0006091	<1e-4	slr0708	GO:0009987	<1e-4	sll0875	GO:0009987	<1e-4	slr0168	GO:0016817	<1e-4	slr6106	GO:0016020	0.1948
slr0065	GO:0046164	<1e-4	ssr6027	GO:0048522	<1e-4	slr1034	GO:0006793	<1e-4	slr0023	GO:0003674	<1e-4	slr0667	GO:0016020	0.1948
slr1957	GO:0006163	<1e-4	sll0785	GO:0022411	<1e-4	sll0577	GO:0051171	<1e-4	slr0053	GO:0008171	<1e-4	slr0082	GO:0016020	0.1948
slr1895	GO:0009263	<1e-4	sll1060	GO:0022607	<1e-4	ssl7042	GO:0043412	<1e-4	ssr6079	GO:0016741	<1e-4	slr6103	GO:0016020	0.1948
sll0482	GO:0042180	<1e-4	slr0374	GO:0044271	<1e-4	slr1920	GO:0009199	<1e-4	sll0481	GO:0015075	<1e-4	sll7034	GO:0016020	0.1948
slr0179	GO:0046128	<1e-4	sll0007	GO:0006163	<1e-4	slr1535	GO:0050801	<1e-4	slr1557	GO:0060089	<1e-4	sll7067	GO:0016020	0.1948
slr2027	GO:0009260	<1e-4	sll1233	GO:0046128	<1e-4	sll1866	GO:0051188	<1e-4	ssl7045	GO:0022857	<1e-4	sll1570	GO:0016020	0.1948
sll0060	GO:0046483	<1e-4	sll1510	GO:0044106	<1e-4	slr1419	GO:0051171	<1e-4	slr0923	GO:0043492	<1e-4	ssl0467	GO:0016020	0.1948
slr1788	GO:0009057	<1e-4	slr5111	GO:0050896	<1e-4	sll0588	GO:0006733	<1e-4	sll0010	GO:0022857	<1e-4	sll0101	GO:0016020	0.1948
sll0066	GO:0051186	<1e-4	slr0104	GO:0009150	<1e-4	sll1921	GO:0051171	<1e-4	slr8022	GO:0016746	<1e-4	ssl5100	GO:0016020	0.1948
ssl5091	GO:0065007	<1e-4	sll1160	GO:0005996	<1e-4	slr0924	GO:0001932	<1e-4	sll1659	GO:0005342	<1e-4	sll1089	GO:0016020	0.1948

slr1956	GO:0051818	<1e-4	slr0491	GO:0019752	<1e-4	ssl0294	GO:0055082	<1e-4	ssl7039	GO:0043492	<1e-4	slr0238	GO:0016020	0.1948
sll0563	GO:0006163	<1e-4	slr1472	GO:0051186	<1e-4	slr0142	GO:0009260	<1e-4	slr1419	GO:0015405	<1e-4	slr1087	GO:0016020	0.1948
sll0414	GO:0019752	<1e-4	ssl0294	GO:0050794	<1e-4	sll1862	GO:0048518	<1e-4	slr1577	GO:0043492	<1e-4	slr1101	GO:0016020	0.1948
slr0082	GO:0006066	<1e-4	ssl2069	GO:0009263	<1e-4	slr2121	GO:0051171	<1e-4	sll0328	GO:0043167	<1e-4	ssl6092	GO:0016020	0.1948
ssl0738	GO:0042451	<1e-4	slr1056	GO:0051186	<1e-4	sll1142	GO:0050801	<1e-4	ssr2047	GO:0015293	<1e-4	sll7078	GO:0016020	0.1948
slr0667	GO:0034641	<1e-4	slr0207	GO:0009144	<1e-4	sll0822	GO:0072528	<1e-4	sll0281	GO:0016741	<1e-4	sll8001	GO:0016020	0.1948
sll1424	GO:0046394	<1e-4	slr6087	GO:0044283	<1e-4	sll1651	GO:0019222	<1e-4	sll1340	GO:0022836	<1e-4	ssr6062	GO:0016020	0.1948
slr7101	GO:0019362	<1e-4	sll0710	GO:0071554	<1e-4	ssr7036	GO:0006733	<1e-4	sll1738	GO:0022891	<1e-4	ssl2064	GO:0016020	0.1948
ssr2803	GO:0046907	<1e-4	slr1600	GO:0046907	<1e-4	slr1263	GO:0046164	<1e-4	ssl5091	GO:0022891	<1e-4	slr0888	GO:0016020	0.1948
slr5111	GO:0071840	<1e-4	sll0413	GO:0050789	<1e-4	sll0031	GO:0031323	<1e-4	ssl2733	GO:0016462	<1e-4	sll0162	GO:0016020	0.1948
slr0058	GO:0072524	<1e-4	slr6031	GO:0065007	<1e-4	ssr2711	GO:0051347	<1e-4	ssl5008	GO:0022838	<1e-4	sll0875	GO:0016020	0.1948
ssr3341	GO:0009263	<1e-4	ssr1528	GO:0009126	<1e-4	slr1519	GO:0042451	<1e-4	sll0265	GO:0005342	<1e-4	slr1563	GO:0016020	0.1948
slr1600	GO:0031326	<1e-4	slr1261	GO:0009308	<1e-4	sll0736	GO:0072528	<1e-4	sll0282	GO:0022832	<1e-4	slr7025	GO:0006810	0.1938
sll0898	GO:0008610	<1e-4	slr2003	GO:0065007	<1e-4	slr1958	GO:0055082	<1e-4	ssr0332	GO:0022892	<1e-4	ssl3383	GO:0006810	0.1938
sll1485	GO:0006576	<1e-4	sll1469	GO:0043170	<1e-4	slr0038	GO:0051347	<1e-4	sll1002	GO:0022838	<1e-4	slr1660	GO:0006810	0.1938
sll7069	GO:0009117	<1e-4	sll1348	GO:0042180	<1e-4	sll5062	GO:0051188	<1e-4	slr1169	GO:0022832	<1e-4	slr0038	GO:0006810	0.1938
slr0780	GO:0051179	<1e-4	slr2052	GO:0048522	<1e-4	ssr2998	GO:0065007	<1e-4	sll0872	GO:0016746	<1e-4	slr0393	GO:0006810	0.1938
slr5037	GO:0006082	<1e-4	ssl1046	GO:0044271	<1e-4	slr1677	GO:0016052	<1e-4	sll8032	GO:0022892	<1e-4	sll1696	GO:0006810	0.1938
sll1921	GO:0006720	<1e-4	ssl1255	GO:0046394	<1e-4	sll0156	GO:0010556	<1e-4	slr0625	GO:0008171	<1e-4	slr2010	GO:0006810	0.1938
sll0394	GO:0009259	<1e-4	slr2084	GO:0009161	<1e-4	slr1270	GO:0009262	<1e-4	ssl2920	GO:0043167	<1e-4	slr1464	GO:0006810	0.1938
slr7037	GO:0009124	<1e-4	sll0160	GO:0009059	<1e-4	slr6033	GO:0016054	<1e-4	sll0148	GO:0005342	<1e-4	slr6021	GO:0006810	0.1938
sll1306	GO:0009308	<1e-4	slr1327	GO:0051188	<1e-4	ssr2754	GO:0009150	<1e-4	sll1858	GO:0008171	<1e-4	slr7016	GO:0006810	0.1938
slr1789	GO:0072522	<1e-4	sll2015	GO:0034654	<1e-4	sll0656	GO:0042455	<1e-4	slr0038	GO:0016741	<1e-4	ssl2920	GO:0006810	0.1938
ssl2717	GO:0042451	<1e-4	slr1275	GO:0044255	<1e-4	sll0596	GO:0009141	<1e-4	sll8012	GO:0042623	<1e-4	ssl3379	GO:0006810	0.1938
sll1166	GO:0034654	<1e-4	ssr1114	GO:0051188	<1e-4	slr1534	GO:0009142	<1e-4	sll1158	GO:0022803	<1e-4	sll1123	GO:0006810	0.1938
slr1998	GO:0052111	<1e-4	ssl3382	GO:0044260	<1e-4	slr0594	GO:0046394	<1e-4	ssr1499	GO:0016817	<1e-4	ssl5129	GO:0006810	0.1938
slr2005	GO:0048523	<1e-4	slr1811	GO:0043648	<1e-4	sll5128	GO:0033013	<1e-4	ssl2420	GO:0016746	<1e-4	sll0742	GO:0006810	0.1938
sll0031	GO:0022607	<1e-4	slr0400	GO:0050801	<1e-4	slr0816	GO:0043436	<1e-4	slr0845	GO:0008171	<1e-4	ssl5098	GO:0006810	0.1938
sll1267	GO:0051818	<1e-4	slr6063	GO:0071840	<1e-4	ssl0350	GO:0071496	<1e-4	slr7071	GO:0015293	<1e-4	sll0167	GO:0006810	0.1938
slr0821	GO:0034660	<1e-4	slr7026	GO:0009059	<1e-4	sll0669	GO:0071840	<1e-4	slr5012	GO:0003674	<1e-4	sll5032	GO:0006810	0.1938
slr0645	GO:0050896	<1e-4	ssr6078	GO:0009124	<1e-4	slr1187	GO:0006631	<1e-4	slr0742	GO:0015077	<1e-4	sll0659	GO:0006810	0.1938
slr0157	GO:0048522	<1e-4	sll1671	GO:0006082	<1e-4	sll1191	GO:0019222	<1e-4	slr0304	GO:0016818	<1e-4	slr1768	GO:0006810	0.1938
sll7047	GO:0048523	<1e-4	sll1925	GO:0046394	<1e-4	sll1289	GO:0006163	<1e-4	ssr8047	GO:0022836	<1e-4	sll0369	GO:0006810	0.1938
slr6104	GO:0052188	<1e-4	slr0975	GO:0050794	<1e-4	sll0266	GO:0051347	<1e-4	sll0031	GO:0015293	<1e-4	slr1179	GO:0006810	0.1938
slr0304	GO:0009150	<1e-4	ssr2802	GO:0048522	<1e-4	sll1455	GO:0009309	<1e-4	sll0749	GO:0042623	<1e-4	slr5018	GO:0006810	0.1938
slr1667	GO:0005996	<1e-4	sll1352	GO:0005996	<1e-4	ssl2807	GO:0009199	<1e-4	sll1832	GO:0015075	<1e-4	ssl1004	GO:0006810	0.1938
ssr1528	GO:0006766	<1e-4	slr0416	GO:0090304	<1e-4	ssr2422	GO:0006066	<1e-4	slr0482	GO:0015399	<1e-4	slr1814	GO:0006810	0.1938
slr1107	GO:0044275	<1e-4	sll1541	GO:0019751	<1e-4	slr1519	GO:0043436	<1e-4	sll1021	GO:0008171	<1e-4	slr0049	GO:0006810	0.1938
sll0980	GO:0009892	<1e-4	ssl2384	GO:0043648	<1e-4	slr0818	GO:0005996	<1e-4	sll0595	GO:0022803	<1e-4	ssr7036	GO:0006810	0.1938
sll5034	GO:0009132	<1e-4	slr1699	GO:0044283	<1e-4	sll1163	GO:0043933	<1e-4	slr0545	GO:0022891	<1e-4	sll0284	GO:0006810	0.1938
ssl6061	GO:0042451	<1e-4	ssl5091	GO:0009309	<1e-4	slr0148	GO:0006732	<1e-4	sll1737	GO:0016818	<1e-4	slr0145	GO:0006810	0.1938
slr6103	GO:0009124	<1e-4	slr0269	GO:0065007	<1e-4	slr0656	GO:0006733	<1e-4	sll1606	GO:0022857	<1e-4	slr0179	GO:0006810	0.1938
sll0827	GO:0009144	<1e-4	slr0888	GO:0044271	<1e-4	slr0935	GO:0009161	<1e-4	sll1025	GO:0015075	<1e-4	sll2013	GO:0006810	0.1938
sll1956	GO:0009123	<1e-4	slr1177	GO:0048522	<1e-4	slr1896	GO:0008150	<1e-4	sll0763	GO:0043492	<1e-4	slr1484	GO:0006810	0.1938
slr0780	GO:0034654	<1e-4	ssr2422	GO:0060255	<1e-4	sll1541	GO:0008150	<1e-4	sll1528	GO:0060089	<1e-4	sll0761	GO:0006810	0.1938
slr1174	GO:0006811	<1e-4	sll0488	GO:0006091	<1e-4	slr6087	GO:0043549	<1e-4	slr1627	GO:0015291	<1e-4	ssr6085	GO:0006810	0.1938
sll0412	GO:0008150	<1e-4	sgl0001	GO:0019751	<1e-4	sll1938	GO:0009259	<1e-4	slr0285	GO:0016817	<1e-4	ssl1520	GO:0006810	0.1938
slr1573	GO:0009263	<1e-4	ssl2749	GO:0051171	<1e-4	slr0813	GO:0048518	<1e-4	slr1612	GO:0003674	<1e-4	slr6074	GO:0006810	0.1938
sll5046	GO:0043412	<1e-4	slr0712	GO:0055082	<1e-4	slr0400	GO:0009124	<1e-4	slr1911	GO:0022890	<1e-4	ssr1766	GO:0006810	0.1938
slr1788	GO:0048523	<1e-4	sll1109	GO:0046128	<1e-4	slr0398	GO:0044282	<1e-4	slr0482	GO:0016818	<1e-4	sll1359	GO:0006810	0.1938
slr0250	GO:0001932	<1e-4	sll5046	GO:0019219	<1e-4	slr0876	GO:0042180	<1e-4	sll1634	GO:0043167	<1e-4	sll1469	GO:0006810	0.1938
sll0980	GO:0009308	<1e-4	sll1714	GO:0009124	<1e-4	slr1603	GO:0052188	<1e-4	slr0981	GO:0060089	<1e-4	sll1400	GO:0006810	0.1938
slr1513	GO:0043648	<1e-4	sll0781	GO:0009057	<1e-4	ssr0109	GO:0031323	<1e-4	slr0104	GO:0015399	<1e-4	ssl8005	GO:0006810	0.1938
ssr5019	GO:0051234	<1e-4	sll1902	GO:0006576	<1e-4	ssr1365	GO:0019222	<1e-4	sll1240	GO:0015291	<1e-4	sll5090	GO:0006810	0.1938

sll1505	GO:0051347	<1e-4	slr2144	GO:0034654	<1e-4	slr1023	GO:0044003	<1e-4	sll0710	GO:0005342	<1e-4	sll0857	GO:0006810	0.1938
slr1258	GO:0046483	<1e-4	slr0664	GO:0009889	<1e-4	slr1150	GO:0080090	<1e-4	sll0060	GO:0022838	<1e-4	sll1109	GO:0006810	0.1938
slr1339	GO:0009150	<1e-4	slr1464	GO:0048518	<1e-4	slr1674	GO:0019751	<1e-4	sll0321	GO:0022803	<1e-4	sll0174	GO:0006810	0.1938
ssl5129	GO:0016053	<1e-4	ssr2754	GO:0044275	<1e-4	slr0211	GO:0022607	<1e-4	sll5044	GO:0015405	<1e-4	slr6039	GO:0006810	0.1938
slr1079	GO:0006066	<1e-4	ssl0294	GO:0008150	<1e-4	slr1957	GO:0051234	<1e-4	sll0762	GO:0042623	<1e-4	ssl2781	GO:0006810	0.1938
sll1735	GO:0006631	<1e-4	ssl2920	GO:0006811	<1e-4	slr0172	GO:0006631	<1e-4	slr5127	GO:0016817	<1e-4	sll0760	GO:0006810	0.1938
slr0211	GO:0009150	<1e-4	slr7100	GO:0072522	<1e-4	slr1590	GO:0006811	<1e-4	slr1301	GO:0016817	<1e-4	slr1270	GO:0006810	0.1938
slr1957	GO:0008150	<1e-4	sll0930	GO:0044003	<1e-4	sll5030	GO:0009059	<1e-4	sll0216	GO:0043167	<1e-4	sll2015	GO:0006810	0.1938
sll0858	GO:0071840	<1e-4	ssl1377	GO:0010556	<1e-4	sll0751	GO:0048518	<1e-4	sll0931	GO:0042623	<1e-4	slr0870	GO:0006810	0.1938
sll0888	GO:0006066	<1e-4	ssr2333	GO:0006793	<1e-4	sll1934	GO:0034641	<1e-4	sll0175	GO:0016741	<1e-4	slr1546	GO:0006810	0.1938
sll0775	GO:0050801	<1e-4	ssr1375	GO:0009056	<1e-4	slr0192	GO:0006811	<1e-4	ssr1155	GO:0043167	<1e-4	slr1362	GO:0006810	0.1938
sll1092	GO:0009123	<1e-4	slr1266	GO:0009057	<1e-4	sll0843	GO:0019752	<1e-4	sll1022	GO:0022891	<1e-4	sll0328	GO:0006810	0.1938
slr5119	GO:0006811	<1e-4	slr6051	GO:0022411	<1e-4	ssl0350	GO:0034641	<1e-4	sll8007	GO:0022838	<1e-4	slr1927	GO:0006810	0.1938
slr1813	GO:0006082	<1e-4	slr7014	GO:0006576	<1e-4	slr0941	GO:0044275	<1e-4	slr0602	GO:0022857	<1e-4	slr0092	GO:0006810	0.1938
slr0978	GO:0080090	<1e-4	slr0729	GO:0044260	<1e-4	sll0539	GO:0009893	<1e-4	slr5111	GO:0016817	<1e-4	sll1884	GO:0006810	0.1938
slr1780	GO:0045184	<1e-4	sll0905	GO:0050794	<1e-4	sll0265	GO:0044248	<1e-4	sll8001	GO:0016746	<1e-4	ssl3142	GO:0006810	0.1938
sll1995	GO:0044281	<1e-4	slr0909	GO:0009260	<1e-4	sll0982	GO:0080090	<1e-4	sll1769	GO:0043167	<1e-4	sll1773	GO:0006810	0.1938
slr0924	GO:0044281	<1e-4	slr1557	GO:0019752	<1e-4	slr7081	GO:0042180	<1e-4	sll1304	GO:0043492	<1e-4	sll0802	GO:0006810	0.1938
sll0861	GO:0006793	<1e-4	slr0172	GO:0008150	<1e-4	ssr5121	GO:0009059	<1e-4	slr0865	GO:0008171	<1e-4	slr0959	GO:0006810	0.1938
slr0712	GO:0009117	<1e-4	slr1222	GO:0009150	<1e-4	slr0243	GO:0009141	<1e-4	sll0446	GO:0016741	<1e-4	sll5130	GO:0006810	0.1938
sll1247	GO:0009059	<1e-4	ssr0692	GO:0072522	<1e-4	slr0479	GO:0019222	<1e-4	slr1998	GO:0015399	<1e-4	slr1162	GO:0006810	0.1938
sll0524	GO:0044275	<1e-4	sll1660	GO:0048878	<1e-4	ssl1263	GO:0065007	<1e-4	sll0872	GO:0022832	<1e-4	sll0762	GO:0006810	0.1938
slr6091	GO:0051716	<1e-4	sll1250	GO:0022607	<1e-4	slr7071	GO:0009394	<1e-4	sll0319	GO:0015293	<1e-4	sll1921	GO:0006810	0.1938
sll0669	GO:0009117	<1e-4	slr0607	GO:0065007	<1e-4	slr1188	GO:0051246	<1e-4	sll2006	GO:0022892	<1e-4	slr1103	GO:0006810	0.1938
ssl5007	GO:0043412	<1e-4	slr0980	GO:0055082	<1e-4	sll0762	GO:0051186	<1e-4	ssl3573	GO:0060089	<1e-4	sll0176	GO:0006810	0.1938
sll0756	GO:0019222	<1e-4	ssr6099	GO:0043549	<1e-4	ssr2912	GO:0050896	<1e-4	sll0905	GO:0022857	<1e-4	sll1265	GO:0006810	0.1938
ssr2554	GO:0050801	<1e-4	sll0543	GO:0034654	<1e-4	ssr3129	GO:0006811	<1e-4	sll0406	GO:0022890	<1e-4	sll1203	GO:0006810	0.1938
ssr1041	GO:0052188	<1e-4	sll0863	GO:0018130	<1e-4	slr0023	GO:0043549	<1e-4	slr0887	GO:0003674	<1e-4	sll1764	GO:0006810	0.1938
slr1957	GO:0044260	<1e-4	slr1188	GO:0044283	<1e-4	sll0451	GO:0071554	<1e-4	sll5062	GO:0016818	<1e-4	slr1276	GO:0006810	0.1938
slr0981	GO:0050789	<1e-4	sll0861	GO:0048522	<1e-4	sll1201	GO:0090304	<1e-4	slr1980	GO:0016746	<1e-4	ssl2148	GO:0006810	0.1938
sll1373	GO:0006733	<1e-4	sll0661	GO:0044260	<1e-4	slr1161	GO:0044003	<1e-4	slr0168	GO:0015405	<1e-4	slr0333	GO:0006810	0.1938
ssl2717	GO:0060255	<1e-4	slr7058	GO:0044003	<1e-4	sll0676	GO:0006082	<1e-4	sll0298	GO:0022890	<1e-4	ssl1417	GO:0006810	0.1938
ssl1520	GO:0019222	<1e-4	sll0478	GO:0072524	<1e-4	sll1040	GO:0072527	<1e-4	slr2071	GO:0003674	<1e-4	slr6038	GO:0006810	0.1938
sll1396	GO:0051188	<1e-4	slr1544	GO:0051188	<1e-4	slr0184	GO:0006733	<1e-4	slr1915	GO:0016817	<1e-4	ssr5020	GO:0006810	0.1938
sll1512	GO:0044275	<1e-4	sll0183	GO:0006720	<1e-4	ssl8005	GO:0009161	<1e-4	slr0872	GO:0008171	<1e-4	slr7011	GO:0006810	0.1938
sll1738	GO:0044260	<1e-4	ssr2142	GO:0009142	<1e-4	slr0038	GO:0071554	<1e-4	sll0669	GO:0022890	<1e-4	sll1735	GO:0006810	0.1938
slr1327	GO:0044275	<1e-4	slr0887	GO:0031640	<1e-4	slr0885	GO:0072524	<1e-4	slr5119	GO:0022836	<1e-4	ssr1951	GO:0006810	0.1938
slr1287	GO:0009991	<1e-4	slr0863	GO:0051716	<1e-4	slr1056	GO:0072528	<1e-4	slr0923	GO:0015399	<1e-4	sll0478	GO:0006810	0.1938
slr1163	GO:0051716	<1e-4	sll0839	GO:0051188	<1e-4	sll1414	GO:0006732	<1e-4	slr1365	GO:0008171	<1e-4	sll1651	GO:0006810	0.1938
slr6106	GO:0051246	<1e-4	sll7070	GO:0052111	<1e-4	slr0579	GO:0071554	<1e-4	ssl5114	GO:0015075	<1e-4	sll1571	GO:0006810	0.1938
slr5119	GO:0009144	<1e-4	slr1866	GO:0051179	<1e-4	sll5046	GO:0009132	<1e-4	sll1232	GO:0003674	<1e-4	sll0406	GO:0006810	0.1938
slr1927	GO:0009309	<1e-4	slr8044	GO:0072528	<1e-4	slr1544	GO:0051347	<1e-4	slr2118	GO:0022832	<1e-4	sll0815	GO:0006810	0.1938
slr6049	GO:0009117	<1e-4	ssl0739	GO:0065007	<1e-4	ssr3410	GO:0009892	<1e-4	sll0101	GO:0042623	<1e-4	slr0146	GO:0006810	0.1938
slr2018	GO:0050896	<1e-4	ssr0536	GO:0080090	<1e-4	sll0183	GO:0048519	<1e-4	ssl0461	GO:0042623	<1e-4	slr0362	GO:0006810	0.1938
ssl8008	GO:0072521	<1e-4	sll1505	GO:0055086	<1e-4	sll1692	GO:0009259	<1e-4	slr1436	GO:0016462	<1e-4	sll1769	GO:0006810	0.1938
slr0937	GO:0042180	<1e-4	ssr1951	GO:0009144	<1e-4	slr1073	GO:0048522	<1e-4	sll1863	GO:0015405	<1e-4	sll0023	GO:0006810	0.1937
slr1568	GO:0034660	<1e-4	slr0890	GO:0006576	<1e-4	ssr0761	GO:0009309	<1e-4	slr5116	GO:0015267	<1e-4	sll0355	GO:0006810	0.1937
slr0360	GO:0048522	<1e-4	slr0816	GO:0009117	<1e-4	sll5132	GO:0048519	<1e-4	ssl0109	GO:0022803	<1e-4	sll0007	GO:0006810	0.1937
slr7099	GO:0045184	<1e-4	sll0428	GO:0006793	<1e-4	slr0551	GO:0051818	<1e-4	ssl1690	GO:0008171	<1e-4	sll1613	GO:0006810	0.1937
sll0442	GO:0019222	<1e-4	slr0964	GO:0022607	<1e-4	slr0865	GO:0009889	<1e-4	sll0596	GO:0022892	<1e-4	slr2121	GO:0006810	0.1937
sll1350	GO:0008150	<1e-4	slr0468	GO:0006732	<1e-4	sll1509	GO:0051171	<1e-4	sll8012	GO:0016817	<1e-4	ssr2439	GO:0006810	0.1937
sll0925	GO:0055086	<1e-4	slr1174	GO:0006793	<1e-4	slr0038	GO:0006220	<1e-4	sll0413	GO:0015077	<1e-4	slr2144	GO:0006810	0.1937
sll1528	GO:0006811	<1e-4	slr1258	GO:0008150	<1e-4	ssr6086	GO:0001932	<1e-4	slr1152	GO:0003674	<1e-4	slr0364	GO:0006810	0.1937
sll0647	GO:0009991	<1e-4	sll7090	GO:0072528	<1e-4	ssl1552	GO:0006766	<1e-4	slr0871	GO:0022838	<1e-4	slr2073	GO:0006810	0.1937

sll0857	GO:0044283	<1e-4	slr0386	GO:0016052	<1e-4	sll1348	GO:0009987	<1e-4	sll5044	GO:0043167	<1e-4	sll0237	GO:0006810	0.1937
sll0447	GO:0009259	<1e-4	slr0211	GO:0072521	<1e-4	ssl8008	GO:0043412	<1e-4	slr7091	GO:0042623	<1e-4	slr0192	GO:0006810	0.1937
slr0789	GO:0009893	<1e-4	sll1447	GO:0051188	<1e-4	slr1800	GO:0019362	<1e-4	slr0496	GO:0016817	<1e-4	ssr2551	GO:0006810	0.1937
slr1162	GO:0044255	<1e-4	slr1613	GO:0042451	<1e-4	slr1493	GO:0016052	<1e-4	sll0394	GO:0008171	<1e-4	sll0524	GO:0006810	0.1937
ssr6030	GO:0051188	<1e-4	sll0886	GO:0045184	<1e-4	sll0703	GO:0044281	<1e-4	sll0369	GO:0005342	<1e-4	sll1830	GO:0006810	0.1937
sll1950	GO:0009142	<1e-4	slr0294	GO:0034660	<1e-4	slr1052	GO:0048523	<1e-4	slr0625	GO:0016462	<1e-4	slr1788	GO:0006810	0.1937
sll0071	GO:0009309	<1e-4	slr0980	GO:0042180	<1e-4	slr1807	GO:0044282	<1e-4	slr1441	GO:0022891	<1e-4	slr0482	GO:0006810	0.1937
sll0752	GO:0051347	<1e-4	ssr3467	GO:0009165	<1e-4	sll1109	GO:0006163	<1e-4	slr1668	GO:0022836	<1e-4	slr1572	GO:0006810	0.1937
slr1780	GO:0046907	<1e-4	slr1628	GO:0006721	<1e-4	sll1131	GO:0016052	<1e-4	slr1807	GO:0015077	<1e-4	slr0204	GO:0006810	0.1937
sll7062	GO:0044260	<1e-4	sll0661	GO:0009117	<1e-4	ssr2802	GO:0010556	<1e-4	sll0265	GO:0015267	<1e-4	sll0756	GO:0006810	0.1937
slr0914	GO:0050801	<1e-4	sll1486	GO:0009165	<1e-4	sml0011	GO:0044271	<1e-4	slr1519	GO:0015291	<1e-4	sll0325	GO:0006810	0.1937
slr0890	GO:0009893	<1e-4	sll1775	GO:0071496	<1e-4	sll0762	GO:0009126	<1e-4	sll1562	GO:0043492	<1e-4	slr1885	GO:0006810	0.1937
sll1222	GO:0034641	<1e-4	slr1809	GO:0009308	<1e-4	slr6049	GO:0009260	<1e-4	slr0545	GO:0042623	<1e-4	slr0845	GO:0006810	0.1937
sll7034	GO:0009132	<1e-4	ssr0536	GO:0065007	<1e-4	sll0301	GO:0043549	<1e-4	sll0585	GO:0042623	<1e-4	slr0586	GO:0006810	0.1937
sll0216	GO:0009126	<1e-4	sll1797	GO:0008150	<1e-4	sll1634	GO:0051716	<1e-4	sll1715	GO:0042623	<1e-4	sll1658	GO:0006810	0.1937
sll1832	GO:0042455	<1e-4	slr1034	GO:0052188	<1e-4	slr0345	GO:0016053	<1e-4	slr0816	GO:0015077	<1e-4	sll1954	GO:0006810	0.1937
sll1173	GO:0042180	<1e-4	ssr2843	GO:0016053	<1e-4	slr0169	GO:0006766	<1e-4	slr1819	GO:0016817	<1e-4	slr1593	GO:0006810	0.1937
slr0204	GO:0051179	<1e-4	slr6106	GO:0044281	<1e-4	slr1034	GO:0009987	<1e-4	sll1835	GO:0016741	<1e-4	slr1262	GO:0006810	0.1937
ssr0536	GO:0006793	<1e-4	slr0058	GO:0009117	<1e-4	ssl3692	GO:0052188	<1e-4	sll1390	GO:0016462	<1e-4	sll1006	GO:0006810	0.1937
sll0478	GO:0009262	<1e-4	sll0875	GO:0009199	<1e-4	sll0669	GO:0019362	<1e-4	ssr1155	GO:0022892	<1e-4	slr1676	GO:0006810	0.1937
ssl2148	GO:0009056	<1e-4	slr0869	GO:0043933	<1e-4	slr5073	GO:0009150	<1e-4	ssl7045	GO:0015267	<1e-4	ssl2065	GO:0006810	0.1937
sll1613	GO:0042455	<1e-4	slr8014	GO:0006082	<1e-4	ssl7022	GO:0051716	<1e-4	ssl7048	GO:0008171	<1e-4	sll0414	GO:0006810	0.1937
slr6016	GO:0071496	<1e-4	sll0886	GO:0006066	<1e-4	slr0975	GO:0065007	<1e-4	sll8002	GO:0015075	<1e-4	sll1630	GO:0006810	0.1937
sll0175	GO:0071842	<1e-4	ssl5096	GO:0046907	<1e-4	sll0272	GO:0034654	<1e-4	sll0350	GO:0005342	<1e-4	ssr2781	GO:0006810	0.193
sll1640	GO:0010468	<1e-4	sll0008	GO:0016053	<1e-4	ssr6086	GO:0071842	<1e-4	sll0732	GO:0022803	<1e-4	slr0317	GO:0006810	0.193
slr1362	GO:0010556	<1e-4	slr5101	GO:0071842	<1e-4	sll0909	GO:0006721	<1e-4	sll1399	GO:0016818	<1e-4	slr0408	GO:0006810	0.193
sll1738	GO:0006793	<1e-4	slr6104	GO:0050794	<1e-4	sll0359	GO:0009059	<1e-4	slr1178	GO:0015075	<1e-4	sll0103	GO:0006810	0.193
ssl1046	GO:0034660	<1e-4	ssl3379	GO:0044106	<1e-4	slr0305	GO:0019222	<1e-4	sll1355	GO:0042623	<1e-4	slr2084	GO:0006810	0.193
sll0068	GO:0031640	<1e-4	slr7073	GO:0009889	<1e-4	sll0478	GO:0009987	<1e-4	sll0487	GO:0003674	<1e-4	sll0071	GO:0006810	0.193
sll1273	GO:0043436	<1e-4	sll8012	GO:0044260	<1e-4	sll0595	GO:0046394	<1e-4	sll0424	GO:0022832	<1e-4	sll1902	GO:0006810	0.193
slr6103	GO:0019362	<1e-4	sll0997	GO:0071840	<1e-4	sll0503	GO:0006163	<1e-4	slr0145	GO:0015267	<1e-4	ssr1258	GO:0006810	0.193
slr0442	GO:0051179	<1e-4	sll1306	GO:0065007	<1e-4	sll1469	GO:0071841	<1e-4	slr0509	GO:0016746	<1e-4	ssr2803	GO:0006810	0.193
slr1071	GO:0009394	<1e-4	sll1651	GO:0034641	<1e-4	sll0608	GO:0009142	<1e-4	slr2000	GO:0015077	<1e-4	slr0980	GO:0006810	0.193
slr1472	GO:0006811	<1e-4	sll1426	GO:0006721	<1e-4	slr5023	GO:0071554	<1e-4	ssr3304	GO:0060089	<1e-4	slr0848	GO:0006810	0.193
slr1241	GO:0051186	<1e-4	ssr1473	GO:0042451	<1e-4	ssl1255	GO:0009057	<1e-4	sll0781	GO:0015077	<1e-4	slr0919	GO:0006810	0.193
sll1247	GO:0031326	<1e-4	sll1173	GO:0043170	<1e-4	slr6031	GO:0016053	<1e-4	sll1132	GO:0003674	<1e-4	sll0558	GO:0006810	0.1918
sll0048	GO:0034660	<1e-4	ssl3379	GO:0033013	<1e-4	slr0169	GO:0009263	<1e-4	sll1380	GO:0015075	<1e-4	sll1751	GO:0006810	0.1918
slr0408	GO:0071842	<1e-4	sll0022	GO:0043436	<1e-4	ssl1498	GO:0006721	<1e-4	ssr0536	GO:0016462	<1e-4	slr1851	GO:0006810	0.1918
sll0497	GO:0033013	<1e-4	slr7097	GO:0046483	<1e-4	slr5127	GO:0019362	<1e-4	ssl2064	GO:0022838	<1e-4	sll0208	GO:0006810	0.1918
slr1726	GO:0044282	<1e-4	slr1174	GO:0050789	<1e-4	slr1811	GO:0009132	<1e-4	ssr3467	GO:0015291	<1e-4	sll1562	GO:0006810	0.1918
slr1398	GO:0051716	<1e-4	slr0545	GO:0019219	<1e-4	slr0670	GO:0006793	<1e-4	slr6028	GO:0016817	<1e-4	sll0783	GO:0006810	0.1918
slr1815	GO:0010468	<1e-4	ssr3570	GO:0019219	<1e-4	slr2048	GO:0006091	<1e-4	slr1647	GO:0022832	<1e-4	slr0978	GO:0006810	0.1918
slr0637	GO:0048522	<1e-4	slr6080	GO:0009150	<1e-4	sll0436	GO:0065007	<1e-4	sll1939	GO:0043167	<1e-4	slr0607	GO:0006810	0.1918
ssr3532	GO:0072521	<1e-4	sll1447	GO:0051179	<1e-4	ssr3570	GO:0046483	<1e-4	sll0676	GO:0022891	<1e-4	slr1307	GO:0006810	0.1918
slr1571	GO:0072528	<1e-4	sll0752	GO:0034654	<1e-4	slr1262	GO:0009987	<1e-4	slr2110	GO:0015399	<1e-4	slr1865	GO:0006810	0.1918
slr0816	GO:0043648	<1e-4	slr0545	GO:0048878	<1e-4	slr1103	GO:0044260	<1e-4	slr7100	GO:0022838	<1e-4	ssl0750	GO:0016491	0.1889
slr0976	GO:0009144	<1e-4	slr1098	GO:0042180	<1e-4	sll0048	GO:0006163	<1e-4	ssl8003	GO:0022836	<1e-4	sll1293	GO:0016491	0.1889
ssr3570	GO:0031326	<1e-4	slr8022	GO:0051347	<1e-4	sll1630	GO:0050896	<1e-4	sll0584	GO:0015075	<1e-4	sll1763	GO:0016491	0.1889
ssl3142	GO:0044271	<1e-4	ssl1498	GO:0071554	<1e-4	ssl0294	GO:0009199	<1e-4	slr0728	GO:0015291	<1e-4	ssl0294	GO:0016491	0.1889
sll1906	GO:0006793	<1e-4	sll0066	GO:0055086	<1e-4	sll1477	GO:0048878	<1e-4	sll0071	GO:0015291	<1e-4	sll0931	GO:0016491	0.1861
sll0310	GO:0019362	<1e-4	ssr2067	GO:0006720	<1e-4	slr0959	GO:0009892	<1e-4	slr0269	GO:0005342	<1e-4	slr1052	GO:0016491	0.1861
sll1797	GO:0016053	<1e-4	ssl2920	GO:0006753	<1e-4	sll0925	GO:0031323	<1e-4	sll0496	GO:0016817	<1e-4	sll0252	GO:0016491	0.184
slr0211	GO:0006721	<1e-4	slr0907	GO:0048518	<1e-4	sll8007	GO:0046128	<1e-4	slr0148	GO:0043167	<1e-4	slr1240	GO:0000166	0.1822
sll0488	GO:0016052	<1e-4	ssl2814	GO:0009059	<1e-4	slr1866	GO:0052111	<1e-4	slr1150	GO:0043492	<1e-4	sll1372	GO:0000166	0.1822

slr1800	GO:0009161	<1e-4	sll2011	GO:0043549	<1e-4	slr1935	GO:0048523	<1e-4	ssr0332	GO:0008171	<1e-4	ssr1528	GO:0000166	0.1822
slr1074	GO:0010468	<1e-4	slr0554	GO:0044248	<1e-4	sll0982	GO:0009124	<1e-4	slr0498	GO:0015077	<1e-4	sll1173	GO:0000166	0.1822
sll0614	GO:0019752	<1e-4	sll1123	GO:0019222	<1e-4	sll1188	GO:0044275	<1e-4	sll1961	GO:0003674	<1e-4	sll0997	GO:0000166	0.1822
slr2010	GO:0042180	<1e-4	sll0815	GO:0009150	<1e-4	slr7024	GO:0072528	<1e-4	sll1006	GO:0022890	<1e-4	slr0789	GO:0000166	0.1822
slr0505	GO:0044275	<1e-4	ssl2996	GO:0051347	<1e-4	slr1062	GO:0016053	<1e-4	sll1696	GO:0060089	<1e-4	slr0456	GO:0000166	0.1822
slr0582	GO:0051818	<1e-4	sll0783	GO:0006793	<1e-4	slr1896	GO:0019222	<1e-4	slr1142	GO:0016741	<1e-4	slr0650	GO:0000166	0.1822
sll1601	GO:0071554	<1e-4	ssr6002	GO:0006163	<1e-4	sll0157	GO:0072521	<1e-4	ssr7017	GO:0016462	<1e-4	sll1582	GO:0000166	0.1822
ssl5025	GO:0045184	<1e-4	slr1957	GO:0006721	<1e-4	slr0919	GO:0034660	<1e-4	sll0863	GO:0015267	<1e-4	sll0539	GO:0000166	0.1822
sll1691	GO:0071554	<1e-4	ssr2912	GO:0006811	<1e-4	sll1873	GO:0031323	<1e-4	sll0939	GO:0015291	<1e-4	slr2005	GO:0000166	0.1822
slr1188	GO:0009117	<1e-4	slr1568	GO:0043412	<1e-4	sll1476	GO:0046164	<1e-4	slr0250	GO:0022803	<1e-4	sll0449	GO:0000166	0.1822
sll7028	GO:0043412	<1e-4	sll0760	GO:0051246	<1e-4	sll7028	GO:0048878	<1e-4	sll1608	GO:0060089	<1e-4	slr1209	GO:0000166	0.1822
sll0237	GO:0044003	<1e-4	slr1429	GO:0051171	<1e-4	ssl0788	GO:0009259	<1e-4	slr0103	GO:0022857	<1e-4	slr0924	GO:0000166	0.1822
sll0843	GO:0016054	<1e-4	slr1863	GO:0046907	<1e-4	slr0598	GO:0051171	<1e-4	slr0582	GO:0022803	<1e-4	slr0249	GO:0000166	0.1822
sll1912	GO:0009126	<1e-4	slr0907	GO:0009123	<1e-4	sll0811	GO:0006733	<1e-4	slr0333	GO:0015077	<1e-4	slr1590	GO:0000166	0.1822
slr1122	GO:0006766	<1e-4	ssl5098	GO:0052188	<1e-4	sll1095	GO:0044260	<1e-4	slr1851	GO:0022803	<1e-4	sll0595	GO:0000166	0.1822
sll1906	GO:0051188	<1e-4	sll1763	GO:0071841	<1e-4	slr1169	GO:0072528	<1e-4	sll0888	GO:0015399	<1e-4	sll0752	GO:0000166	0.1822
sll1241	GO:0071841	<1e-4	sll1752	GO:0055082	<1e-4	sll0875	GO:0050896	<1e-4	sll8032	GO:0016818	<1e-4	slr1303	GO:0000166	0.1822
slr0863	GO:0046483	<1e-4	sll1911	GO:0046164	<1e-4	sll1652	GO:0055114	<1e-4	slr6091	GO:0015291	<1e-4	sml0011	GO:0000166	0.1822
slr1657	GO:0009126	<1e-4	slr1926	GO:0006576	<1e-4	slr5023	GO:0006811	<1e-4	slr1519	GO:0022857	<1e-4	slr1258	GO:0000166	0.1822
slr1170	GO:0044106	<1e-4	slr1601	GO:0051347	<1e-4	sll0102	GO:0042455	<1e-4	slr0613	GO:0022836	<1e-4	slr0373	GO:0000166	0.1822
sll1921	GO:0050789	<1e-4	ssl1520	GO:0009199	<1e-4	sll0608	GO:0008150	<1e-4	slr0483	GO:0022857	<1e-4	sll1352	GO:0000166	0.1822
sll1319	GO:0052111	<1e-4	sll1906	GO:0008150	<1e-4	slr0269	GO:0080090	<1e-4	sll0382	GO:0015267	<1e-4	sll0871	GO:0000166	0.1822
slr1394	GO:0009057	<1e-4	sll0410	GO:0044003	<1e-4	sll5067	GO:0051188	<1e-4	sll0319	GO:0022832	<1e-4	sll1350	GO:0000166	0.1822
slr0503	GO:0048878	<1e-4	slr0217	GO:0072527	<1e-4	ssr2755	GO:0051716	<1e-4	sll0737	GO:0015077	<1e-4	sll0359	GO:0000166	0.1822
slr0250	GO:0009259	<1e-4	sll0564	GO:0044282	<1e-4	slr1188	GO:0044275	<1e-4	slr2000	GO:0060089	<1e-4	slr0104	GO:0000166	0.1822
slr0907	GO:0034641	<1e-4	sll0350	GO:0044260	<1e-4	ssr2422	GO:0071554	<1e-4	sll7034	GO:0022890	<1e-4	sll0688	GO:0000166	0.1822
sll5006	GO:0051818	<1e-4	slr7023	GO:0071554	<1e-4	sll1634	GO:0044275	<1e-4	sll7065	GO:0043167	<1e-4	slr0664	GO:0000166	0.1822
slr5102	GO:0031640	<1e-4	sll1511	GO:0072522	<1e-4	slr5112	GO:0034654	<1e-4	slr6080	GO:0008171	<1e-4	sll1950	GO:0000166	0.1822
slr1800	GO:0048519	<1e-4	sll1009	GO:0008150	<1e-4	sll1359	GO:0043436	<1e-4	slr6088	GO:0015075	<1e-4	sll1390	GO:0000166	0.1822
sll8040	GO:0090304	<1e-4	sll0982	GO:0052188	<1e-4	sll0172	GO:0006066	<1e-4	slr0582	GO:0022838	<1e-4	slr1690	GO:0000166	0.1822
slr2080	GO:0046128	<1e-4	slr1809	GO:0009117	<1e-4	sll0641	GO:0046164	<1e-4	sll0793	GO:0022892	<1e-4	slr0702	GO:0000166	0.1822
sll1071	GO:0009150	<1e-4	sll0639	GO:0060255	<1e-4	slr7080	GO:0009059	<1e-4	slr0919	GO:0016462	<1e-4	sll1654	GO:0000166	0.1822
sll0274	GO:0042180	<1e-4	slr0870	GO:0006811	<1e-4	slr1951	GO:0006766	<1e-4	sll0886	GO:0016741	<1e-4	sll1925	GO:0000166	0.1822
sll0911	GO:0050801	<1e-4	slr1206	GO:0009892	<1e-4	sll0359	GO:0044283	<1e-4	slr0789	GO:0022803	<1e-4	slr1128	GO:0000166	0.1822
slr2052	GO:0022607	<1e-4	sll1265	GO:0009308	<1e-4	slr0708	GO:0051171	<1e-4	sll0180	GO:0043167	<1e-4	sll1956	GO:0000166	0.1822
slr1095	GO:0009165	<1e-4	slr1194	GO:0055086	<1e-4	sll1942	GO:0016054	<1e-4	sll8001	GO:0042623	<1e-4	slr0729	GO:0000166	0.1822
sll0564	GO:0051246	<1e-4	sll1654	GO:0006721	<1e-4	ssl1520	GO:0006066	<1e-4	ssr3402	GO:0015267	<1e-4	sll1512	GO:0000166	0.1822
slr1681	GO:0044281	<1e-4	sll0007	GO:0009132	<1e-4	ssr1155	GO:0019752	<1e-4	slr1033	GO:0015293	<1e-4	slr1940	GO:0000166	0.1822
slr1290	GO:0072527	<1e-4	sll0539	GO:0044260	<1e-4	slr0712	GO:0050789	<1e-4	slr6067	GO:0022836	<1e-4	sll0268	GO:0000166	0.1822
sll2015	GO:0072527	<1e-4	sll0376	GO:0006163	<1e-4	ssr2318	GO:0033013	<1e-4	sll1965	GO:0043167	<1e-4	sll1040	GO:0000166	0.1822
slr1206	GO:0006811	<1e-4	slr0232	GO:0051234	<1e-4	slr1194	GO:0034641	<1e-4	sll1340	GO:0022891	<1e-4	sll1399	GO:0000166	0.1822
slr1230	GO:0052188	<1e-4	sll1400	GO:0043933	<1e-4	sll0481	GO:0009056	<1e-4	slr1163	GO:0016817	<1e-4	sll0981	GO:0000166	0.1822
sll0780	GO:0006720	<1e-4	sll1130	GO:0042455	<1e-4	sll0846	GO:0044260	<1e-4	sll1766	GO:0008171	<1e-4	sll1272	GO:0016491	0.1821
sll1142	GO:0044003	<1e-4	slr1571	GO:0048519	<1e-4	slr0731	GO:0051171	<1e-4	slr1143	GO:0008171	<1e-4	sll0656	GO:0016491	0.1821
ssr1114	GO:0044255	<1e-4	slr0479	GO:0060255	<1e-4	sll1123	GO:0019637	<1e-4	sll5028	GO:0016817	<1e-4	slr0108	GO:0016491	0.1821
slr2025	GO:0050789	<1e-4	slr1270	GO:0009126	<1e-4	sll0309	GO:0071554	<1e-4	ssl3382	GO:0016462	<1e-4	sll1528	GO:0016491	0.1821
sll5069	GO:0006721	<1e-4	sll1381	GO:0048878	<1e-4	ssl2065	GO:0043436	<1e-4	slr1813	GO:0022803	<1e-4	sll1477	GO:0016491	0.1821
sll0496	GO:0009142	<1e-4	sll0749	GO:0009059	<1e-4	sll0982	GO:0006631	<1e-4	slr0217	GO:0022832	<1e-4	slr0871	GO:0006810	0.1819
slr0941	GO:0019219	<1e-4	sll0596	GO:0009893	<1e-4	slr1273	GO:0006721	<1e-4	sll0749	GO:0016818	<1e-4	slr1066	GO:0006810	0.1819
slr1865	GO:0019752	<1e-4	sll0925	GO:0043933	<1e-4	ssl5027	GO:0044283	<1e-4	sll0296	GO:0042623	<1e-4	sll1433	GO:0006810	0.1819
sll2015	GO:0044271	<1e-4	slr0960	GO:0019362	<1e-4	sll0997	GO:0044106	<1e-4	ssr0536	GO:0015075	<1e-4	slr1866	GO:0006810	0.1819
slr7080	GO:0051818	<1e-4	slr1534	GO:0009126	<1e-4	slr0517	GO:0043170	<1e-4	sll2006	GO:0015291	<1e-4	sll0296	GO:0006810	0.1819
slr6065	GO:0006066	<1e-4	ssr1041	GO:0005996	<1e-4	slr0112	GO:0043648	<1e-4	slr0092	GO:0015405	<1e-4	slr1896	GO:0006810	0.1819
slr1142	GO:0048522	<1e-4	slr0813	GO:0046483	<1e-4	slr0104	GO:0009987	<1e-4	ssl2971	GO:0015399	<1e-4	slr1353	GO:0006810	0.1819

sll0854	GO:0065007	<1e-4	slr0480	GO:0050794	<1e-4	slr1977	GO:0060255	<1e-4	sll0008	GO:0022890	<1e-4	sll0424	GO:0006810	0.1819
slr0551	GO:0009144	<1e-4	ssl2717	GO:0050801	<1e-4	sll1532	GO:0051179	<1e-4	sll1835	GO:0022832	<1e-4	sll1455	GO:0006810	0.1819
sll5067	GO:0042180	<1e-4	sll1797	GO:0009056	<1e-4	slr2120	GO:0009056	<1e-4	slr1864	GO:0022857	<1e-4	sll0274	GO:0006810	0.1819
sll1477	GO:0043549	<1e-4	slr8022	GO:0009124	<1e-4	slr6049	GO:0050789	<1e-4	sll1240	GO:0008171	<1e-4	slr0376	GO:0006810	0.1819
ssl1046	GO:0044260	<1e-4	slr1287	GO:0006811	<1e-4	ssl6035	GO:0050789	<1e-4	slr0264	GO:0060089	<1e-4	slr1917	GO:0006810	0.1819
slr5021	GO:0009165	<1e-4	slr7026	GO:0009892	<1e-4	sll1250	GO:0006720	<1e-4	slr0723	GO:0015075	<1e-4	slr0211	GO:0006810	0.1819
slr1813	GO:0051716	<1e-4	slr0592	GO:0009260	<1e-4	slr0654	GO:0009165	<1e-4	sll7062	GO:0022857	<1e-4	slr1391	GO:0006810	0.1819
slr0300	GO:0065007	<1e-4	slr0496	GO:0022607	<1e-4	sll0101	GO:0009263	<1e-4	ssr2551	GO:0015399	<1e-4	sll1218	GO:0006810	0.1819
slr1376	GO:0072527	<1e-4	sll1239	GO:0042455	<1e-4	ssl5099	GO:0006091	<1e-4	slr1827	GO:0005342	<1e-4	slr0243	GO:0006810	0.1819
sll1532	GO:0009987	<1e-4	sll8035	GO:0042451	<1e-4	sll1938	GO:0009144	<1e-4	slr1079	GO:0043492	<1e-4	sll1934	GO:0006810	0.1819
sll1239	GO:0005996	<1e-4	slr5102	GO:0009132	<1e-4	sll1925	GO:0006066	<1e-4	sll1191	GO:0022803	<1e-4	sll1426	GO:0006810	0.1819
sll0102	GO:0009117	<1e-4	sll1775	GO:0044282	<1e-4	sll0853	GO:0048878	<1e-4	sll0172	GO:0016462	<1e-4	sll1542	GO:0006810	0.1819
sll7050	GO:0009124	<1e-4	slr1623	GO:0051171	<1e-4	sll1773	GO:0006220	<1e-4	slr0606	GO:0042623	<1e-4	slr0876	GO:0006810	0.1819
slr7037	GO:0071842	<1e-4	slr1852	GO:0072524	<1e-4	slr0634	GO:0009394	<1e-4	slr1628	GO:0016462	<1e-4	slr1025	GO:0006810	0.1819
ssl2595	GO:0046164	<1e-4	slr0645	GO:0048519	<1e-4	slr6075	GO:0048519	<1e-4	slr1023	GO:0022890	<1e-4	sll1472	GO:0006810	0.1819
slr0456	GO:0044003	<1e-4	ssr2802	GO:0006766	<1e-4	slr0976	GO:0008150	<1e-4	slr8044	GO:0015399	<1e-4	sll0508	GO:0006810	0.1819
slr0816	GO:0009165	<1e-4	sll1613	GO:0016054	<1e-4	sll5063	GO:0006163	<1e-4	sll0022	GO:0022832	<1e-4	slr1222	GO:0006810	0.1819
slr7026	GO:0006753	<1e-4	slr0241	GO:0009308	<1e-4	ssl1792	GO:0046394	<1e-4	slr1441	GO:0016818	<1e-4	slr1612	GO:0006810	0.1819
sll8019	GO:0052111	<1e-4	ssr2803	GO:0018130	<1e-4	slr0400	GO:0010556	<1e-4	slr1142	GO:0015077	<1e-4	slr1127	GO:0006810	0.1819
sll8007	GO:0006220	<1e-4	sll0585	GO:0006766	<1e-4	slr1290	GO:0009308	<1e-4	sll1307	GO:0016746	<1e-4	slr0476	GO:0006810	0.1819
sll0909	GO:0071554	<1e-4	slr0374	GO:0006066	<1e-4	slr1619	GO:0044260	<1e-4	slr0964	GO:0016818	<1e-4	slr0480	GO:0006810	0.1819
sll1135	GO:0010556	<1e-4	slr1613	GO:0044260	<1e-4	slr0596	GO:0072522	<1e-4	slr0373	GO:0015075	<1e-4	slr1162	GO:0016491	0.1818
sll1571	GO:0019362	<1e-4	sll1757	GO:0009117	<1e-4	slr0975	GO:0009260	<1e-4	sll0577	GO:0015293	<1e-4	slr1188	GO:0016491	0.1818
slr6031	GO:0009263	<1e-4	slr1681	GO:0065007	<1e-4	sll0669	GO:0052111	<1e-4	sll1174	GO:0060089	<1e-4	sll0867	GO:0016491	0.1818
slr0431	GO:0019222	<1e-4	ssr6003	GO:0046164	<1e-4	slr1862	GO:0006576	<1e-4	slr6100	GO:0022832	<1e-4	slr7097	GO:0016491	0.1818
sll1738	GO:0009144	<1e-4	sll0263	GO:0042455	<1e-4	sll0785	GO:0044275	<1e-4	sll0068	GO:0060089	<1e-4	sll0803	GO:0016491	0.1818
slr6016	GO:0005996	<1e-4	slr1339	GO:0044003	<1e-4	slr1188	GO:0090304	<1e-4	slr1062	GO:0016746	<1e-4	sll0406	GO:0016491	0.1818
sll0147	GO:0009893	<1e-4	ssl0461	GO:0044283	<1e-4	slr0250	GO:0043412	<1e-4	sll0314	GO:0016462	<1e-4	slr7098	GO:0016491	0.1818
sll0742	GO:0044281	<1e-4	slr6021	GO:0043436	<1e-4	ssr6078	GO:0006811	<1e-4	sll7067	GO:0008171	<1e-4	slr7083	GO:0016491	0.1818
sll1411	GO:0046128	<1e-4	sll0930	GO:0048523	<1e-4	slr0468	GO:0009144	<1e-4	slr0755	GO:0016462	<1e-4	sll0293	GO:0016491	0.1818
sll1222	GO:0052188	<1e-4	ssl5129	GO:0060255	<1e-4	sll1761	GO:0022607	<1e-4	sll1906	GO:0022838	<1e-4	sll0886	GO:0016491	0.1818
sll0007	GO:0050794	<1e-4	slr5119	GO:0019637	<1e-4	sll0984	GO:0045184	<1e-4	slr1407	GO:0015405	<1e-4	ssr1407	GO:0016491	0.1818
slr7024	GO:0052111	<1e-4	slr1690	GO:0033013	<1e-4	sll1949	GO:0080090	<1e-4	sll1095	GO:0016746	<1e-4	sll0369	GO:0016491	0.1818
ssr1528	GO:0008150	<1e-4	slr5018	GO:0009132	<1e-4	slr5016	GO:0071841	<1e-4	sll1025	GO:0022838	<1e-4	slr0440	GO:0016491	0.1818
slr0065	GO:0009057	<1e-4	sll1160	GO:0051186	<1e-4	slr0848	GO:0052188	<1e-4	slr0250	GO:0015077	<1e-4	slr1363	GO:0016491	0.1818
slr1419	GO:0051186	<1e-4	sll0381	GO:0009161	<1e-4	sll1060	GO:0006733	<1e-4	slr1084	GO:0043167	<1e-4	ssl1464	GO:0016491	0.1818
sll1321	GO:0048523	<1e-4	ssl1300	GO:0046483	<1e-4	ssr1768	GO:0043549	<1e-4	ssr6020	GO:0022891	<1e-4	slr0145	GO:0016491	0.1818
sll1106	GO:0009150	<1e-4	slr0959	GO:0045184	<1e-4	sll0274	GO:0006811	<1e-4	sll5047	GO:0022836	<1e-4	sll1389	GO:0016491	0.1818
slr2012	GO:0009893	<1e-4	slr1681	GO:0033013	<1e-4	sll8019	GO:0006733	<1e-4	sll0994	GO:0022857	<1e-4	slr6008	GO:0016491	0.1818
slr1169	GO:0008150	<1e-4	ssl8028	GO:0080090	<1e-4	slr1241	GO:0009150	<1e-4	slr1814	GO:0022832	<1e-4	ssr2611	GO:0016491	0.1818
sll1884	GO:0048522	<1e-4	sll0451	GO:0031323	<1e-4	slr1603	GO:0031323	<1e-4	slr1178	GO:0016817	<1e-4	sll1939	GO:0016491	0.1818
sll0360	GO:0051716	<1e-4	sll7028	GO:0008150	<1e-4	slr2144	GO:0050801	<1e-4	sll0162	GO:0016818	<1e-4	ssl3379	GO:0016491	0.1818
sll0780	GO:0072521	<1e-4	slr1230	GO:0046128	<1e-4	sll1304	GO:0044271	<1e-4	slr2144	GO:0022891	<1e-4	sll0658	GO:0016491	0.1818
sll1315	GO:0072524	<1e-4	sll0691	GO:0010556	<1e-4	slr0967	GO:0009394	<1e-4	sll0175	GO:0015405	<1e-4	sll5032	GO:0016491	0.1818
slr1462	GO:0043933	<1e-4	slr0695	GO:0051188	<1e-4	sll1528	GO:0010556	<1e-4	slr1188	GO:0022832	<1e-4	sll1123	GO:0016491	0.1818
slr1815	GO:0050794	<1e-4	slr2060	GO:0050789	<1e-4	sll8002	GO:0009987	<1e-4	slr0416	GO:0022890	<1e-4	ssr2422	GO:0016491	0.1818
sll7055	GO:0022607	<1e-4	sll0532	GO:0019362	<1e-4	sll1552	GO:0009889	<1e-4	sll0216	GO:0022891	<1e-4	sll5061	GO:0016491	0.1818
slr0195	GO:0031323	<1e-4	sll0691	GO:0042455	<1e-4	sll1764	GO:0055082	<1e-4	sll5003	GO:0016818	<1e-4	ssr5020	GO:0016491	0.1818
sll1372	GO:0031640	<1e-4	sll0297	GO:0072527	<1e-4	slr1660	GO:0042180	<1e-4	sll1131	GO:0022857	<1e-4	sll5047	GO:0016491	0.1818
slr0192	GO:0046483	<1e-4	slr5013	GO:0019362	<1e-4	slr1188	GO:0048523	<1e-4	slr0712	GO:0015405	<1e-4	ssr5120	GO:0016491	0.1818
sll5130	GO:0009059	<1e-4	ssr1041	GO:0009150	<1e-4	slr1260	GO:0044282	<1e-4	slr1471	GO:0060089	<1e-4	sll1109	GO:0016491	0.1818
sll0394	GO:0010556	<1e-4	sll5026	GO:0010468	<1e-4	slr0305	GO:0046907	<1e-4	slr7073	GO:0022838	<1e-4	slr0890	GO:0016491	0.1818
ssr1499	GO:0044282	<1e-4	slr1394	GO:0055114	<1e-4	ssr6019	GO:0051716	<1e-4	slr5118	GO:0005342	<1e-4	sll0775	GO:0016491	0.1818
sll0981	GO:0008150	<1e-4	sll0301	GO:0009987	<1e-4	sll0691	GO:0050896	<1e-4	sll7067	GO:0022838	<1e-4	ssl5031	GO:0016491	0.1818

sll0558	GO:0034641	<1e-4	slr0695	GO:0072522	<1e-4	ssr6099	GO:0019219	<1e-4	slr6044	GO:0042623	<1e-4	sll5026	GO:0016491	0.1818
slr0207	GO:0006220	<1e-4	sll1174	GO:0019222	<1e-4	ssl1464	GO:0006066	<1e-4	sll1505	GO:0022836	<1e-4	slr0489	GO:0016491	0.1818
slr2119	GO:0048522	<1e-4	slr1168	GO:0009144	<1e-4	sll0414	GO:0072522	<1e-4	slr1301	GO:0015075	<1e-4	ssl2420	GO:0016491	0.1818
sll0487	GO:0048522	<1e-4	slr1603	GO:0050789	<1e-4	slr1513	GO:0072528	<1e-4	slr1699	GO:0015399	<1e-4	sll1306	GO:0016491	0.1818
sll1060	GO:0034660	<1e-4	slr7013	GO:0016054	<1e-4	sll0181	GO:0072524	<1e-4	ssl0787	GO:0016746	<1e-4	sll0167	GO:0016491	0.1818
ssl0467	GO:0042180	<1e-4	slr1778	GO:0046164	<1e-4	slr2110	GO:0019751	<1e-4	sll0685	GO:0016817	<1e-4	slr7082	GO:0016491	0.1818
ssr3410	GO:0019752	<1e-4	slr1275	GO:0044260	<1e-4	slr1534	GO:0072524	<1e-4	sll1752	GO:0043492	<1e-4	slr2119	GO:0016491	0.1818
sll0298	GO:0044260	<1e-4	sll1002	GO:0009309	<1e-4	slr0345	GO:0009165	<1e-4	sll7031	GO:0016818	<1e-4	slr1384	GO:0016491	0.1818
ssl7046	GO:0008150	<1e-4	slr0053	GO:0033013	<1e-4	slr1618	GO:0044106	<1e-4	ssl5103	GO:0015293	<1e-4	slr7025	GO:0016491	0.1818
slr1222	GO:0009991	<1e-4	slr0780	GO:0080090	<1e-4	sll0939	GO:0043412	<1e-4	sll7087	GO:0016818	<1e-4	sll1203	GO:0016491	0.1818
sll0350	GO:0043170	<1e-4	slr6068	GO:0072522	<1e-4	slr0845	GO:0051234	<1e-4	ssr2553	GO:0022890	<1e-4	slr0870	GO:0016491	0.1818
slr6038	GO:0009057	<1e-4	sll5026	GO:0009117	<1e-4	sll0382	GO:0018130	<1e-4	ssl2471	GO:0022892	<1e-4	ssl2148	GO:0016491	0.1818
sll0072	GO:0031323	<1e-4	sll1913	GO:0006733	<1e-4	slr1301	GO:0006811	<1e-4	slr1384	GO:0043167	<1e-4	sll1378	GO:0016491	0.1818
slr2121	GO:0009165	<1e-4	slr1462	GO:0019219	<1e-4	sll1052	GO:0044271	<1e-4	sll7034	GO:0016818	<1e-4	slr0049	GO:0016491	0.1818
sll0996	GO:0006766	<1e-4	sll1002	GO:0010468	<1e-4	ssr0336	GO:0009263	<1e-4	slr1546	GO:0043492	<1e-4	slr0065	GO:0016491	0.1818
ssl3549	GO:0031640	<1e-4	sll1262	GO:0009141	<1e-4	slr1098	GO:0044260	<1e-4	slr1896	GO:0022832	<1e-4	slr2105	GO:0016491	0.1818
sll0552	GO:0006811	<1e-4	slr1303	GO:0006066	<1e-4	slr1623	GO:0050896	<1e-4	sll2013	GO:0016818	<1e-4	sll0727	GO:0016491	0.1818
slr1913	GO:0009124	<1e-4	slr0816	GO:0043933	<1e-4	slr1235	GO:0006082	<1e-4	slr1674	GO:0022836	<1e-4	slr0146	GO:0016491	0.1818
slr2018	GO:0043412	<1e-4	sll1562	GO:0006091	<1e-4	slr1913	GO:0016054	<1e-4	slr1612	GO:0022803	<1e-4	sll0241	GO:0016491	0.1818
sll1939	GO:0009987	<1e-4	ssr7017	GO:0051716	<1e-4	sll0266	GO:0006733	<1e-4	sll1613	GO:0008171	<1e-4	slr1547	GO:0016491	0.1818
slr1865	GO:0052111	<1e-4	ssl7042	GO:0034641	<1e-4	slr0341	GO:0051186	<1e-4	ssl1417	GO:0015267	<1e-4	slr6049	GO:0016491	0.1818
slr1601	GO:0009144	<1e-4	sll0802	GO:0022411	<1e-4	slr1544	GO:0046128	<1e-4	ssl2064	GO:0015399	<1e-4	slr1566	GO:0016491	0.1818
ssr2711	GO:0006766	<1e-4	slr0104	GO:0019637	<1e-4	ssl5100	GO:0009309	<1e-4	ssl2807	GO:0022892	<1e-4	slr6067	GO:0016491	0.1818
sll0181	GO:0006753	<1e-4	slr1599	GO:0006766	<1e-4	sll1009	GO:0046164	<1e-4	sll0047	GO:0043167	<1e-4	slr7011	GO:0016491	0.1818
slr1384	GO:0033013	<1e-4	ssr6024	GO:0048519	<1e-4	slr1394	GO:0045184	<1e-4	sll1170	GO:0022890	<1e-4	sll1571	GO:0016491	0.1818
slr0815	GO:0051716	<1e-4	slr2003	GO:0051716	<1e-4	slr0937	GO:0044003	<1e-4	sll5006	GO:0022857	<1e-4	sll1252	GO:0016491	0.1818
slr0551	GO:0055082	<1e-4	slr0456	GO:0010468	<1e-4	sll0265	GO:0050896	<1e-4	sll1401	GO:0022803	<1e-4	slr1600	GO:0016491	0.1818
sll0096	GO:0071841	<1e-4	slr1261	GO:0044260	<1e-4	ssr1552	GO:0044281	<1e-4	sll5061	GO:0022836	<1e-4	sll0423	GO:0016491	0.1818
slr0146	GO:0046128	<1e-4	sll0875	GO:0044260	<1e-4	sll1730	GO:0044282	<1e-4	slr0975	GO:0016746	<1e-4	slr1104	GO:0016491	0.1818
ssr2201	GO:0072528	<1e-4	slr1450	GO:0044255	<1e-4	sll0564	GO:0055082	<1e-4	sll1109	GO:0022838	<1e-4	ssr2317	GO:0016491	0.1818
ssr5092	GO:0016053	<1e-4	sll1995	GO:0006720	<1e-4	slr1339	GO:0090304	<1e-4	ssr0109	GO:0022836	<1e-4	sll1163	GO:0016491	0.1818
slr0913	GO:0001932	<1e-4	slr6067	GO:0071842	<1e-4	ssl3383	GO:0044271	<1e-4	sll0802	GO:0015267	<1e-4	sll1884	GO:0016491	0.1818
slr0586	GO:0042180	<1e-4	slr1788	GO:0009893	<1e-4	slr1624	GO:0048878	<1e-4	slr0689	GO:0022832	<1e-4	sll1414	GO:0016491	0.1818
sll1749	GO:0051716	<1e-4	slr2070	GO:0046394	<1e-4	sll1751	GO:0055082	<1e-4	slr0619	GO:0015405	<1e-4	ssl2015	GO:0016491	0.1818
sll5004	GO:0009309	<1e-4	sll5006	GO:0080090	<1e-4	ssr1766	GO:0006766	<1e-4	slr0919	GO:0015267	<1e-4	sll1217	GO:0016491	0.1818
sll0191	GO:0072527	<1e-4	ssr5120	GO:0009260	<1e-4	sll1659	GO:0072521	<1e-4	sll0558	GO:0016741	<1e-4	slr0695	GO:0016491	0.1818
ssr5019	GO:0019637	<1e-4	sll1201	GO:0046907	<1e-4	slr1753	GO:0006066	<1e-4	slr0771	GO:0003674	<1e-4	slr1261	GO:0016491	0.1818
ssl0410	GO:0019637	<1e-4	slr2111	GO:0009056	<1e-4	sll1447	GO:0009394	<1e-4	ssr7035	GO:0022892	<1e-4	slr1956	GO:0016491	0.1818
sll0372	GO:0072528	<1e-4	slr0455	GO:0009889	<1e-4	slr1398	GO:0005996	<1e-4	ssl5007	GO:0043492	<1e-4	slr0655	GO:0016491	0.1818
slr1174	GO:0034654	<1e-4	slr0249	GO:0016052	<1e-4	sll0858	GO:0006066	<1e-4	ssl1004	GO:0015075	<1e-4	sll7062	GO:0016491	0.1818
slr1956	GO:0044283	<1e-4	sll7028	GO:0044260	<1e-4	slr7092	GO:0016052	<1e-4	sll2015	GO:0043492	<1e-4	sll0473	GO:0016491	0.1818
sll1995	GO:0043549	<1e-4	slr1196	GO:0051179	<1e-4	sll1401	GO:0019362	<1e-4	sll6054	GO:0043492	<1e-4	slr0505	GO:0016491	0.1818
sll1911	GO:0043436	<1e-4	sll0446	GO:0033013	<1e-4	sll1241	GO:0031640	<1e-4	slr0588	GO:0016746	<1e-4	sll1722	GO:0016491	0.1818
slr0313	GO:0006753	<1e-4	ssr7093	GO:0006066	<1e-4	sll5032	GO:0006066	<1e-4	slr6016	GO:0042623	<1e-4	sll1866	GO:0016491	0.1818
sll0072	GO:0051347	<1e-4	sll5026	GO:0019362	<1e-4	sll1381	GO:0044271	<1e-4	sll0293	GO:0015291	<1e-4	ssr0332	GO:0016491	0.1818
sll1528	GO:0050794	<1e-4	slr8021	GO:0048523	<1e-4	slr0232	GO:0043549	<1e-4	slr0509	GO:0042623	<1e-4	ssl2245	GO:0016491	0.1818
slr1081	GO:0006721	<1e-4	slr0092	GO:0009059	<1e-4	ssr1499	GO:0034660	<1e-4	sll7087	GO:0060089	<1e-4	slr0388	GO:0016491	0.1818
slr1591	GO:0019752	<1e-4	sll5004	GO:0046128	<1e-4	slr1327	GO:0019219	<1e-4	sll1911	GO:0015291	<1e-4	sll5044	GO:0016491	0.1818
slr1619	GO:0031326	<1e-4	ssl2996	GO:0006721	<1e-4	slr1365	GO:0034641	<1e-4	sll1318	GO:0003674	<1e-4	sll0419	GO:0016491	0.1818
slr1398	GO:0009892	<1e-4	slr1900	GO:0043412	<1e-4	slr1612	GO:0050896	<1e-4	sll0410	GO:0015075	<1e-4	slr5116	GO:0016491	0.1818
sll1880	GO:0044248	<1e-4	slr1194	GO:0031640	<1e-4	sll0071	GO:0046394	<1e-4	sll0156	GO:0008171	<1e-4	sll0443	GO:0016491	0.1818
sll0008	GO:0048519	<1e-4	sll1926	GO:0019752	<1e-4	ssl2065	GO:0006733	<1e-4	slr0408	GO:0022836	<1e-4	slr1273	GO:0016491	0.1818
slr1259	GO:0071496	<1e-4	slr6008	GO:0072527	<1e-4	sll0761	GO:0050801	<1e-4	slr5018	GO:0022803	<1e-4	slr7101	GO:0016491	0.1818
slr1116	GO:0072527	<1e-4	sll0208	GO:0042455	<1e-4	sll0780	GO:0006066	<1e-4	sll1638	GO:0042623	<1e-4	slr1864	GO:0016491	0.1818

slr0656	GO:0051188	<1e-4	slr6016	GO:0055086	<1e-4	ssr1258	GO:0006066	<1e-4	slr0104	GO:0022838	<1e-4	slr1306	GO:0016491	0.1818
sll0272	GO:0016054	<1e-4	slr6106	GO:0050801	<1e-4	sll0775	GO:0090304	<1e-4	sll1160	GO:0016741	<1e-4	slr7015	GO:0016491	0.1818
ssl7048	GO:0071496	<1e-4	slr5127	GO:0051246	<1e-4	slr0479	GO:0065007	<1e-4	sll0994	GO:0022832	<1e-4	slr6080	GO:0016491	0.1818
sll0253	GO:0071554	<1e-4	slr6063	GO:0008150	<1e-4	slr0815	GO:0052188	<1e-4	slr1915	GO:0015267	<1e-4	slr1195	GO:0016491	0.1818
slr0404	GO:0072528	<1e-4	sll1613	GO:0044255	<1e-4	ssr6099	GO:0052111	<1e-4	sll2006	GO:0015399	<1e-4	slr0569	GO:0016491	0.1818
slr1023	GO:0048523	<1e-4	sll0007	GO:0009308	<1e-4	sll0412	GO:0044260	<1e-4	ssl2971	GO:0003674	<1e-4	sll0985	GO:0006810	0.1791
slr2101	GO:0006732	<1e-4	slr0108	GO:0045184	<1e-4	ssl1004	GO:0051716	<1e-4	sll1921	GO:0016818	<1e-4	slr1964	GO:0016491	0.1783
ssl0832	GO:0072522	<1e-4	sll5026	GO:0009124	<1e-4	slr0479	GO:0071841	<1e-4	sml0011	GO:0060089	<1e-4	slr2011	GO:0016491	0.1783
sll0981	GO:0044260	<1e-4	ssr6085	GO:0051171	<1e-4	ssl8039	GO:0009893	<1e-4	slr2012	GO:0022836	<1e-4	slr1437	GO:0016491	0.1783
sll0355	GO:0019751	<1e-4	slr6072	GO:0051234	<1e-4	ssr5020	GO:0010468	<1e-4	slr0642	GO:0015405	<1e-4	slr0023	GO:0016491	0.1783
sll0590	GO:0009144	<1e-4	slr7082	GO:0022607	<1e-4	sll0175	GO:0060255	<1e-4	slr0708	GO:0015293	<1e-4	slr1854	GO:0016491	0.1783
slr7082	GO:0080090	<1e-4	sll0994	GO:0009144	<1e-4	ssr6027	GO:0072527	<1e-4	sll5047	GO:0015399	<1e-4	sll1656	GO:0016491	0.1783
ssr0332	GO:0009123	<1e-4	slr2119	GO:0044281	<1e-4	ssr7036	GO:0050801	<1e-4	slr0709	GO:0015293	<1e-4	sll1835	GO:0016491	0.1783
slr1951	GO:0065007	<1e-4	slr0270	GO:0009132	<1e-4	sll1272	GO:0009150	<1e-4	sll1411	GO:0015267	<1e-4	ssr2755	GO:0016491	0.1783
slr6013	GO:0065007	<1e-4	slr0871	GO:0051347	<1e-4	slr1767	GO:0009124	<1e-4	slr0551	GO:0043492	<1e-4	slr0392	GO:0016491	0.1783
sll0355	GO:0060255	<1e-4	ssl0461	GO:0006082	<1e-4	slr0318	GO:0080090	<1e-4	slr1875	GO:0016817	<1e-4	sll0314	GO:0016491	0.1783
slr1082	GO:0050789	<1e-4	sll0350	GO:0009126	<1e-4	slr1116	GO:0009309	<1e-4	sll0875	GO:0015077	<1e-4	sll0185	GO:0016491	0.1783
sll0547	GO:0050789	<1e-4	slr1658	GO:0043933	<1e-4	slr1767	GO:0009394	<1e-4	ssr3571	GO:0043167	<1e-4	sll0780	GO:0016491	0.1783
ssr7036	GO:0006220	<1e-4	sll1531	GO:0031640	<1e-4	sll0268	GO:0010468	<1e-4	sll1934	GO:0015291	<1e-4	slr0913	GO:0016491	0.1783
slr1600	GO:0044106	<1e-4	slr0514	GO:0048518	<1e-4	slr1827	GO:0051246	<1e-4	sll7062	GO:0022890	<1e-4	ssr3122	GO:0016491	0.1783
sll7050	GO:0051179	<1e-4	slr0651	GO:0050789	<1e-4	sll1834	GO:0022607	<1e-4	sll0413	GO:0022891	<1e-4	slr1573	GO:0016491	0.1783
sll0863	GO:0009150	<1e-4	sll0498	GO:0009308	<1e-4	sll0263	GO:0008150	<1e-4	slr1534	GO:0022890	<1e-4	slr1593	GO:0016491	0.1783
sll0732	GO:0046164	<1e-4	slr0852	GO:0043933	<1e-4	slr1752	GO:0044275	<1e-4	slr0397	GO:0022803	<1e-4	sll1315	GO:0016491	0.1783
sll5063	GO:0034641	<1e-4	slr1644	GO:0051716	<1e-4	sll0783	GO:0090304	<1e-4	sll0827	GO:0016817	<1e-4	slr0519	GO:0016491	0.1783
slr1152	GO:0051171	<1e-4	slr0482	GO:0009260	<1e-4	sll0630	GO:0072521	<1e-4	slr0243	GO:0015075	<1e-4	sll1949	GO:0016491	0.1783
sll2011	GO:0051716	<1e-4	slr7012	GO:0065007	<1e-4	sll1939	GO:0065007	<1e-4	slr1384	GO:0015075	<1e-4	sll0007	GO:0016491	0.1783
sll8033	GO:0071842	<1e-4	slr1847	GO:0044106	<1e-4	slr0670	GO:0044003	<1e-4	sll1355	GO:0005342	<1e-4	sll0524	GO:0016491	0.1783
slr0740	GO:0006091	<1e-4	sll7067	GO:0006811	<1e-4	sll0802	GO:0044283	<1e-4	slr0890	GO:0060089	<1e-4	sll1608	GO:0016491	0.1783
slr1353	GO:0009987	<1e-4	sll0066	GO:0006220	<1e-4	sll0283	GO:0072524	<1e-4	ssl1520	GO:0005342	<1e-4	sll0497	GO:0016491	0.1783
slr1263	GO:0051234	<1e-4	ssr2754	GO:0016054	<1e-4	slr1194	GO:0055082	<1e-4	slr6106	GO:0015293	<1e-4	ssr2439	GO:0016491	0.1783
slr2125	GO:0006811	<1e-4	sll0451	GO:0060255	<1e-4	slr0590	GO:0071496	<1e-4	sll0996	GO:0022890	<1e-4	slr0921	GO:0016491	0.1783
slr0692	GO:0051171	<1e-4	slr0392	GO:0071841	<1e-4	sll0293	GO:0009262	<1e-4	sll1472	GO:0015291	<1e-4	ssl0352	GO:0016491	0.1783
sll0478	GO:0051188	<1e-4	slr1415	GO:0006091	<1e-4	sll1054	GO:0009132	<1e-4	slr0509	GO:0043167	<1e-4	sll1006	GO:0016491	0.1783
slr1209	GO:0072527	<1e-4	slr1186	GO:0009987	<1e-4	sll0630	GO:0009199	<1e-4	slr1104	GO:0015291	<1e-4	slr1778	GO:0016491	0.1783
sll1233	GO:0046394	<1e-4	slr0271	GO:0050801	<1e-4	sll0269	GO:0072528	<1e-4	ssr1558	GO:0016817	<1e-4	sll0237	GO:0016491	0.1783
slr1773	GO:0051179	<1e-4	slr2038	GO:0044248	<1e-4	sll0085	GO:0009309	<1e-4	slr0712	GO:0015399	<1e-4	slr0941	GO:0016491	0.1783
slr5024	GO:0006576	<1e-4	slr1383	GO:0010468	<1e-4	ssl1552	GO:0055114	<1e-4	sll0249	GO:0016741	<1e-4	ssr2551	GO:0016491	0.1783
ssl2814	GO:0006721	<1e-4	slr6033	GO:0042451	<1e-4	slr1557	GO:0009144	<1e-4	slr0241	GO:0015267	<1e-4	ssl2807	GO:0016491	0.1783
slr1209	GO:0022411	<1e-4	sll0102	GO:0050794	<1e-4	slr0065	GO:0044281	<1e-4	slr6033	GO:0016818	<1e-4	sll1563	GO:0016491	0.1783
sll0930	GO:0006720	<1e-4	slr0392	GO:0080090	<1e-4	sll5034	GO:0008150	<1e-4	slr1218	GO:0016462	<1e-4	slr0482	GO:0016491	0.1783
slr6015	GO:0019752	<1e-4	slr1128	GO:0009141	<1e-4	slr0262	GO:0016053	<1e-4	slr0285	GO:0042623	<1e-4	slr0872	GO:0016491	0.1783
slr1618	GO:0008610	<1e-4	slr0318	GO:0033013	<1e-4	sll1262	GO:0048522	<1e-4	sll1052	GO:0022803	<1e-4	sll0606	GO:0016491	0.1774
ssr2998	GO:0031640	<1e-4	sll0565	GO:0044282	<1e-4	sll1400	GO:0006793	<1e-4	slr1773	GO:0016746	<1e-4	ssr1256	GO:0016491	0.1774
slr1932	GO:0071554	<1e-4	slr0184	GO:0009199	<1e-4	sll1025	GO:0080090	<1e-4	slr1441	GO:0003674	<1e-4	sll1912	GO:0016491	0.1774
sll1906	GO:0006732	<1e-4	ssl3382	GO:0009117	<1e-4	sll5109	GO:0031326	<1e-4	slr0980	GO:0022857	<1e-4	ssr2201	GO:0016491	0.1774
ssl6061	GO:0008150	<1e-4	slr7102	GO:0046128	<1e-4	slr8022	GO:0042180	<1e-4	sll0670	GO:0022803	<1e-4	slr2038	GO:0016491	0.1774
slr1593	GO:0050801	<1e-4	slr1398	GO:0071842	<1e-4	sll7062	GO:0009263	<1e-4	slr6022	GO:0043167	<1e-4	slr0980	GO:0016491	0.1774
ssr2843	GO:0009056	<1e-4	sll0898	GO:0044003	<1e-4	ssl0788	GO:0051246	<1e-4	sll8011	GO:0043167	<1e-4	ssr2781	GO:0016491	0.1774
slr0362	GO:0046164	<1e-4	ssr3300	GO:0045184	<1e-4	sll7047	GO:0048878	<1e-4	sll1131	GO:0016462	<1e-4	slr0317	GO:0016491	0.1774
slr7015	GO:0006811	<1e-4	ssl2920	GO:0060255	<1e-4	sll0175	GO:0044281	<1e-4	sll0479	GO:0042623	<1e-4	sll0071	GO:0016491	0.1774
sll1426	GO:0009161	<1e-4	ssr1765	GO:0009987	<1e-4	sll1024	GO:0071841	<1e-4	slr1998	GO:0060089	<1e-4	slr1495	GO:0016491	0.1774
sll0188	GO:0016054	<1e-4	sll1583	GO:0006753	<1e-4	sll0355	GO:0071840	<1e-4	slr0590	GO:0015405	<1e-4	sll0763	GO:0016491	0.1774
sll0553	GO:0019752	<1e-4	slr0645	GO:0001932	<1e-4	sll0436	GO:0009893	<1e-4	slr1287	GO:0015075	<1e-4	sll1186	GO:0016491	0.1773
slr0300	GO:0050896	<1e-4	sll0585	GO:0006082	<1e-4	slr0725	GO:0044003	<1e-4	slr6004	GO:0022836	<1e-4	slr0250	GO:0016491	0.1773

slr1507	GO:0008150	<1e-4	sll0547	GO:0009165	<1e-4	ssl3379	GO:0031326	<1e-4	sll1252	GO:0042623	<1e-4	slr1533	GO:0016491	0.1773
sll1304	GO:0051347	<1e-4	sll1401	GO:0009059	<1e-4	sll0863	GO:0050896	<1e-4	ssr2912	GO:0043167	<1e-4	sll1542	GO:0016491	0.1773
slr0989	GO:0019752	<1e-4	slr7098	GO:0072524	<1e-4	slr0168	GO:0071496	<1e-4	slr2049	GO:0005342	<1e-4	sll1601	GO:0016491	0.1773
sll1151	GO:0009056	<1e-4	sll0274	GO:0006631	<1e-4	slr1591	GO:0043549	<1e-4	slr0579	GO:0016818	<1e-4	slr1194	GO:0016491	0.1773
slr1799	GO:0009309	<1e-4	slr6100	GO:0031640	<1e-4	sll1526	GO:0034654	<1e-4	sll5097	GO:0016817	<1e-4	sll0864	GO:0016491	0.1773
slr0650	GO:0009987	<1e-4	sll0296	GO:0071840	<1e-4	sll0508	GO:0071496	<1e-4	sll1262	GO:0016746	<1e-4	slr0545	GO:0016491	0.1773
slr1566	GO:0001932	<1e-4	sll1036	GO:0016053	<1e-4	sll0327	GO:0009987	<1e-4	ssl1464	GO:0022838	<1e-4	slr1674	GO:0016491	0.1773
slr1572	GO:0009308	<1e-4	slr0453	GO:0043436	<1e-4	ssr2611	GO:0042451	<1e-4	slr1170	GO:0008171	<1e-4	sll0499	GO:0016491	0.1773
sll0860	GO:0019362	<1e-4	slr1875	GO:0043549	<1e-4	slr2110	GO:0055114	<1e-4	sll0933	GO:0043169	<1e-4	ssr0536	GO:0016491	0.1773
sll1400	GO:0008150	<1e-4	slr5016	GO:0005996	<1e-4	ssl0738	GO:0022607	<1e-4	ssl0467	GO:0005342	<1e-4	slr0876	GO:0016491	0.1773
slr0728	GO:0019222	<1e-4	slr1600	GO:0009132	<1e-4	sll0547	GO:0072524	<1e-4	sll0608	GO:0015291	<1e-4	sll1247	GO:0016491	0.1773
slr7011	GO:0009141	<1e-4	slr0948	GO:0055114	<1e-4	sll1939	GO:0008610	<1e-4	slr6021	GO:0015293	<1e-4	slr0937	GO:0016491	0.1773
slr0112	GO:0051347	<1e-4	slr1152	GO:0044106	<1e-4	slr1150	GO:0031640	<1e-4	slr7013	GO:0022803	<1e-4	sll0274	GO:0016491	0.1773
ssl5064	GO:0044248	<1e-4	slr1780	GO:0019751	<1e-4	slr1170	GO:0019222	<1e-4	sll0162	GO:0022892	<1e-4	slr0423	GO:0016491	0.1773
slr1429	GO:0042455	<1e-4	sll0925	GO:0044248	<1e-4	sll7070	GO:0022607	<1e-4	sll1797	GO:0015291	<1e-4	slr1023	GO:0016491	0.1773
slr1273	GO:0010468	<1e-4	sll0933	GO:0006811	<1e-4	slr0023	GO:0051347	<1e-4	ssl8039	GO:0016817	<1e-4	slr1376	GO:0016491	0.1773
ssl0750	GO:0033013	<1e-4	slr0888	GO:0006732	<1e-4	ssl5129	GO:0016052	<1e-4	ssl1378	GO:0022890	<1e-4	slr1107	GO:0016491	0.1773
sll1173	GO:0043549	<1e-4	slr5037	GO:0034641	<1e-4	sll0266	GO:0034654	<1e-4	sll1239	GO:0022803	<1e-4	slr1025	GO:0016491	0.1773
slr1188	GO:0042180	<1e-4	slr1025	GO:0008150	<1e-4	slr6031	GO:0043412	<1e-4	ssr2843	GO:0016817	<1e-4	sll1455	GO:0016491	0.1773
sll1765	GO:0046907	<1e-4	sll1714	GO:0046394	<1e-4	ssr5074	GO:0042451	<1e-4	sll5006	GO:0022832	<1e-4	sll0296	GO:0016491	0.1773
sll0756	GO:0001932	<1e-4	slr7099	GO:0009057	<1e-4	sll1469	GO:0044281	<1e-4	sll1002	GO:0015075	<1e-4	slr1353	GO:0016491	0.1773
sll1132	GO:0009263	<1e-4	slr1441	GO:0050789	<1e-4	slr5013	GO:0044106	<1e-4	slr1537	GO:0003674	<1e-4	slr0157	GO:0016491	0.1773
sll1040	GO:0043648	<1e-4	sll1071	GO:0072524	<1e-4	sll7050	GO:0031323	<1e-4	sll1830	GO:0022890	<1e-4	slr1917	GO:0016491	0.1773
ssl2064	GO:0072524	<1e-4	slr0582	GO:0016054	<1e-4	slr1363	GO:0009260	<1e-4	slr5073	GO:0016818	<1e-4	slr1557	GO:0016491	0.1773
slr1122	GO:0071496	<1e-4	ssr2781	GO:0006720	<1e-4	slr1098	GO:0090304	<1e-4	slr0013	GO:0005342	<1e-4	sll0854	GO:0016491	0.1773
slr6075	GO:0051171	<1e-4	slr0975	GO:0009059	<1e-4	slr2049	GO:0050794	<1e-4	slr0810	GO:0043167	<1e-4	sll1218	GO:0016491	0.1773
slr1547	GO:0019752	<1e-4	slr2105	GO:0006091	<1e-4	slr1415	GO:0009123	<1e-4	slr6021	GO:0016817	<1e-4	ssl0109	GO:0016491	0.1773
sll1950	GO:0008150	<1e-4	ssl5099	GO:0006732	<1e-4	ssr1258	GO:0009309	<1e-4	slr7026	GO:0015291	<1e-4	slr0503	GO:0016491	0.1773
ssr2551	GO:0042451	<1e-4	ssr5020	GO:0006631	<1e-4	slr0606	GO:0006811	<1e-4	sll0444	GO:0022803	<1e-4	slr1519	GO:0016491	0.1773
ssl3383	GO:0042455	<1e-4	slr0890	GO:0044283	<1e-4	sll1832	GO:0065007	<1e-4	ssr7035	GO:0043167	<1e-4	ssr3532	GO:0016491	0.1773
sll0803	GO:0009123	<1e-4	sll1965	GO:0006220	<1e-4	sll0761	GO:0051171	<1e-4	sll1063	GO:0015077	<1e-4	slr0076	GO:0016491	0.1773
sll1715	GO:0043170	<1e-4	slr6074	GO:0051234	<1e-4	sll1062	GO:0052188	<1e-4	slr1600	GO:0016818	<1e-4	sll0263	GO:0016491	0.1773
sll1570	GO:0046128	<1e-4	ssl5027	GO:0052111	<1e-4	slr1657	GO:0031326	<1e-4	sll1714	GO:0060089	<1e-4	ssr2843	GO:0016491	0.1762
sll1632	GO:0006732	<1e-4	sll1660	GO:0009309	<1e-4	sll1630	GO:0006793	<1e-4	slr0241	GO:0022838	<1e-4	sll1858	GO:0016491	0.1762
sll0737	GO:0005996	<1e-4	ssr0109	GO:0006091	<1e-4	sll1766	GO:0048523	<1e-4	sll1630	GO:0016817	<1e-4	slr2092	GO:0016491	0.1762
slr0397	GO:0006082	<1e-4	sll0803	GO:0019752	<1e-4	slr1275	GO:0006631	<1e-4	slr1052	GO:0015075	<1e-4	slr1603	GO:0016491	0.1762
ssr2972	GO:0043412	<1e-4	slr0907	GO:0005996	<1e-4	ssl3383	GO:0009124	<1e-4	slr1082	GO:0022857	<1e-4	sll1737	GO:0016491	0.1762
sll0456	GO:0043648	<1e-4	slr1436	GO:0019362	<1e-4	slr1413	GO:0090304	<1e-4	ssl1378	GO:0015399	<1e-4	slr0341	GO:0016491	0.1762
sll0456	GO:0072522	<1e-4	slr0217	GO:0006631	<1e-4	slr7100	GO:0009165	<1e-4	sll5032	GO:0005342	<1e-4	ssl0353	GO:0016491	0.1756
ssr6048	GO:0051716	<1e-4	slr1066	GO:0072528	<1e-4	sll2011	GO:0009161	<1e-4	sll7070	GO:0022832	<1e-4	slr1851	GO:0016491	0.1756
slr0376	GO:0006091	<1e-4	sll1835	GO:0031640	<1e-4	sll1022	GO:0071496	<1e-4	sll0645	GO:0016741	<1e-4	slr1767	GO:0016491	0.1756
sll7067	GO:0042180	<1e-4	sll1736	GO:0071496	<1e-4	sll0503	GO:0044271	<1e-4	ssl5096	GO:0022838	<1e-4	sll0822	GO:0016491	0.1756
ssr2998	GO:0072521	<1e-4	slr0810	GO:0009142	<1e-4	ssr5106	GO:0031640	<1e-4	slr0264	GO:0022836	<1e-4	sll1526	GO:0016491	0.1756
ssr5074	GO:0043436	<1e-4	slr1188	GO:0031640	<1e-4	slr1196	GO:0031640	<1e-4	sll0864	GO:0016741	<1e-4	slr1726	GO:0016491	0.1756
slr1470	GO:0072527	<1e-4	slr7083	GO:0065007	<1e-4	sll0156	GO:0034660	<1e-4	sll0301	GO:0043492	<1e-4	sll0910	GO:0016491	0.1756
sll0639	GO:0009057	<1e-4	slr1591	GO:0050794	<1e-4	ssr2439	GO:0051716	<1e-4	sll1400	GO:0022857	<1e-4	slr0594	GO:0016491	0.1756
ssl2245	GO:0046164	<1e-4	sll0022	GO:0043549	<1e-4	slr0514	GO:0006631	<1e-4	sll5006	GO:0015267	<1e-4	sll0022	GO:0016491	0.1756
sll1691	GO:0065007	<1e-4	slr1927	GO:0019222	<1e-4	slr0482	GO:0048522	<1e-4	slr0503	GO:0022857	<1e-4	slr0589	GO:0016491	0.1756
sll0156	GO:0031326	<1e-4	slr1484	GO:0006082	<1e-4	sll1218	GO:0080090	<1e-4	slr7098	GO:0043167	<1e-4	ssl2996	GO:0016491	0.1756
slr7012	GO:0009987	<1e-4	slr0731	GO:0051347	<1e-4	sll1906	GO:0044283	<1e-4	slr2092	GO:0015405	<1e-4	slr0263	GO:0016491	0.1756
ssl5015	GO:0019637	<1e-4	slr0241	GO:0022607	<1e-4	slr1906	GO:0006631	<1e-4	slr6063	GO:0022892	<1e-4	sll0788	GO:0016491	0.1756
slr0272	GO:0016054	<1e-4	slr0431	GO:0006720	<1e-4	slr2048	GO:0034654	<1e-4	sll1186	GO:0060089	<1e-4	slr0740	GO:0016491	0.1756
sll1318	GO:0044260	<1e-4	slr1101	GO:0006811	<1e-4	slr6012	GO:0072527	<1e-4	sll1486	GO:0005342	<1e-4	sll1562	GO:0016491	0.1756
slr6029	GO:0006733	<1e-4	ssr1528	GO:0044255	<1e-4	sll0611	GO:0044255	<1e-4	sll1509	GO:0015291	<1e-4	sll0325	GO:0000166	0.1715

ssr7072	GO:0051171	<1e-4	ssl0242	GO:0009987	<1e-4	ssl2069	GO:0019362	<1e-4	slr1913	GO:0016818	<1e-4	slr0386	GO:0000166	0.1715
slr0199	GO:0009144	<1e-4	sll1160	GO:0080090	<1e-4	sll0478	GO:0019222	<1e-4	slr0885	GO:0015267	<1e-4	slr0431	GO:0000166	0.1715
sll1225	GO:0008150	<1e-4	slr0581	GO:0009124	<1e-4	sll1761	GO:0009889	<1e-4	sll0412	GO:0015293	<1e-4	sll0198	GO:0000166	0.1715
slr6021	GO:0072528	<1e-4	sll1464	GO:0010556	<1e-4	slr0670	GO:0009142	<1e-4	slr0151	GO:0022857	<1e-4	slr0364	GO:0000166	0.1715
slr0169	GO:0044283	<1e-4	slr5126	GO:0055082	<1e-4	sll1763	GO:0031326	<1e-4	sll0103	GO:0022838	<1e-4	slr0392	GO:0000166	0.1715
sll0242	GO:0009150	<1e-4	slr5018	GO:0071841	<1e-4	sll0167	GO:0009259	<1e-4	slr1851	GO:0003674	<1e-4	slr2122	GO:0000166	0.1715
ssr6024	GO:0016053	<1e-4	slr0317	GO:0006163	<1e-4	slr1603	GO:0043436	<1e-4	sll1830	GO:0016746	<1e-4	slr1964	GO:0000166	0.1715
slr1612	GO:0071840	<1e-4	ssr5019	GO:0048522	<1e-4	slr0287	GO:0042451	<1e-4	sll8004	GO:0015291	<1e-4	sll0010	GO:0000166	0.1715
ssr3570	GO:0051246	<1e-4	ssr2711	GO:0009126	<1e-4	slr0013	GO:0006163	<1e-4	ssl1046	GO:0016817	<1e-4	slr0941	GO:0000166	0.1715
ssr2615	GO:0006066	<1e-4	sll1390	GO:0009059	<1e-4	sll0871	GO:0009893	<1e-4	slr1122	GO:0022803	<1e-4	slr0845	GO:0000166	0.1715
slr1507	GO:0072521	<1e-4	slr0613	GO:0043412	<1e-4	slr6074	GO:0008150	<1e-4	ssl1263	GO:0043492	<1e-4	slr1084	GO:0000166	0.1715
sll1166	GO:0009150	<1e-4	sll1359	GO:0019752	<1e-4	sll7062	GO:0072521	<1e-4	sll0252	GO:0022890	<1e-4	slr1210	GO:0000166	0.1715
slr1907	GO:0019219	<1e-4	sll1601	GO:0008610	<1e-4	sll1348	GO:0044275	<1e-4	slr6039	GO:0022857	<1e-4	ssl0352	GO:0000166	0.1715
slr1259	GO:0043412	<1e-4	slr1053	GO:0043933	<1e-4	slr0609	GO:0051188	<1e-4	slr1081	GO:0022892	<1e-4	slr1593	GO:0000166	0.1715
sll0822	GO:0006753	<1e-4	slr2105	GO:0072528	<1e-4	slr1087	GO:0046164	<1e-4	slr1547	GO:0016462	<1e-4	sll0456	GO:0000166	0.1715
sll0827	GO:0071842	<1e-4	sll1192	GO:0050801	<1e-4	sll1289	GO:0019751	<1e-4	slr1533	GO:0015291	<1e-4	slr1932	GO:0000166	0.1715
ssl0467	GO:0009262	<1e-4	slr1852	GO:0051171	<1e-4	slr1611	GO:0033013	<1e-4	sll1651	GO:0022892	<1e-4	sll1658	GO:0000166	0.1715
slr0013	GO:0046483	<1e-4	ssl2245	GO:0051716	<1e-4	slr0313	GO:0006732	<1e-4	sll1912	GO:0060089	<1e-4	slr0872	GO:0000166	0.1715
slr1927	GO:0006091	<1e-4	sll0804	GO:0009056	<1e-4	slr1862	GO:0031323	<1e-4	sll0181	GO:0003674	<1e-4	sll0007	GO:0000166	0.1715
sll1464	GO:0009124	<1e-4	slr0730	GO:0051171	<1e-4	ssl7048	GO:0009259	<1e-4	sll0497	GO:0008171	<1e-4	slr1437	GO:0000166	0.1715
sll1021	GO:0046483	<1e-4	slr2012	GO:0006091	<1e-4	slr6088	GO:0044260	<1e-4	slr0333	GO:0003674	<1e-4	slr0144	GO:0000166	0.1715
sll0539	GO:0009059	<1e-4	sll0925	GO:0009150	<1e-4	ssr5074	GO:0006732	<1e-4	sll0837	GO:0015075	<1e-4	slr0519	GO:0000166	0.1715
slr0959	GO:0022411	<1e-4	slr0313	GO:0009987	<1e-4	ssr5011	GO:0009132	<1e-4	slr1619	GO:0043492	<1e-4	sll1061	GO:0000166	0.1715
sll0335	GO:0051186	<1e-4	ssr0759	GO:0009059	<1e-4	slr1906	GO:0006720	<1e-4	slr5023	GO:0022832	<1e-4	slr1571	GO:0000166	0.1715
ssr2848	GO:0044003	<1e-4	slr1788	GO:0016052	<1e-4	ssl1300	GO:0019752	<1e-4	sll1119	GO:0016746	<1e-4	slr1073	GO:0000166	0.1715
slr7081	GO:0052111	<1e-4	slr0941	GO:0090304	<1e-4	slr1940	GO:0072528	<1e-4	sll0335	GO:0043167	<1e-4	sll0381	GO:0000166	0.1715
slr0816	GO:0016054	<1e-4	ssl2384	GO:0051246	<1e-4	slr1659	GO:0051171	<1e-4	sll0101	GO:0022832	<1e-4	sll0355	GO:0000166	0.1715
slr1863	GO:0044248	<1e-4	slr0039	GO:0071841	<1e-4	slr1998	GO:0048522	<1e-4	ssr2049	GO:0022891	<1e-4	slr1572	GO:0000166	0.1715
sll0508	GO:0055086	<1e-4	slr0769	GO:0042451	<1e-4	sll1193	GO:0009309	<1e-4	slr1752	GO:0015267	<1e-4	slr0360	GO:0000166	0.1715
slr0364	GO:0010556	<1e-4	sll7087	GO:0009144	<1e-4	slr1566	GO:0043933	<1e-4	sll1389	GO:0016741	<1e-4	slr2073	GO:0000166	0.1715
slr6075	GO:0065007	<1e-4	sll0069	GO:0044260	<1e-4	slr1790	GO:0019752	<1e-4	sll1400	GO:0015399	<1e-4	slr0023	GO:0000166	0.1715
ssr1425	GO:0006793	<1e-4	sll1068	GO:0072521	<1e-4	sll0513	GO:0009991	<1e-4	slr1601	GO:0016746	<1e-4	slr1618	GO:0000166	0.1715
ssl5031	GO:0009144	<1e-4	sll8007	GO:0042180	<1e-4	slr1648	GO:0043436	<1e-4	sll1511	GO:0015267	<1e-4	slr1644	GO:0016491	0.1695
ssl7046	GO:0006066	<1e-4	slr0712	GO:0046483	<1e-4	slr0145	GO:0009165	<1e-4	sll0623	GO:0022857	<1e-4	ssr2142	GO:0016491	0.1695
slr0729	GO:0046164	<1e-4	slr6051	GO:0051186	<1e-4	ssl8005	GO:0019751	<1e-4	sll1911	GO:0022838	<1e-4	slr1431	GO:0016491	0.1695
slr7025	GO:0046907	<1e-4	sll1241	GO:0008610	<1e-4	slr0645	GO:0009059	<1e-4	slr0318	GO:0015399	<1e-4	sll0933	GO:0006810	0.1692
sll1401	GO:0006163	<1e-4	ssr6027	GO:0006631	<1e-4	sll0661	GO:0005996	<1e-4	sll1608	GO:0015399	<1e-4	sll0445	GO:0016491	0.1685
slr1576	GO:0006091	<1e-4	slr5112	GO:0034641	<1e-4	sll0839	GO:0043436	<1e-4	slr1023	GO:0016462	<1e-4	sll0242	GO:0016491	0.164
ssl8039	GO:0016054	<1e-4	slr0238	GO:0009165	<1e-4	slr0341	GO:0006082	<1e-4	sll5097	GO:0016741	<1e-4	sll0933	GO:0016491	0.164
sll8012	GO:0048519	<1e-4	slr0845	GO:0009987	<1e-4	sll5028	GO:0052188	<1e-4	ssl5007	GO:0005342	<1e-4	slr1918	GO:0000166	0.1598
ssr6026	GO:0010468	<1e-4	ssr3572	GO:0010468	<1e-4	sll1586	GO:0045184	<1e-4	sll0103	GO:0016817	<1e-4	sll1426	GO:0000166	0.1598
sll1289	GO:0072528	<1e-4	slr0810	GO:0009987	<1e-4	slr2032	GO:0072527	<1e-4	ssr0336	GO:0043492	<1e-4	slr1533	GO:0000166	0.1598
sll0243	GO:0043436	<1e-4	sll2006	GO:0046164	<1e-4	slr1168	GO:0043549	<1e-4	slr1438	GO:0060089	<1e-4	slr2025	GO:0000166	0.1598
sll1142	GO:0006066	<1e-4	sll6053	GO:0071842	<1e-4	sll1272	GO:0071554	<1e-4	ssr3300	GO:0022892	<1e-4	slr1659	GO:0000166	0.1598
slr0505	GO:0051234	<1e-4	slr0845	GO:0006576	<1e-4	ssl7038	GO:0006733	<1e-4	slr0914	GO:0022857	<1e-4	sll0283	GO:0000166	0.1598
sll1252	GO:0009142	<1e-4	sll0496	GO:0042451	<1e-4	slr1342	GO:0042180	<1e-4	slr1762	GO:0008171	<1e-4	sll0615	GO:0000166	0.1598
slr1053	GO:0090304	<1e-4	ssl0242	GO:0044282	<1e-4	ssr2972	GO:0006793	<1e-4	slr1210	GO:0008171	<1e-4	sll0301	GO:0000166	0.1598
sll0284	GO:0034654	<1e-4	sll2013	GO:0050801	<1e-4	sll1832	GO:0006793	<1e-4	slr0872	GO:0022890	<1e-4	sll0499	GO:0000166	0.1598
slr1114	GO:0009893	<1e-4	slr1023	GO:0044260	<1e-4	slr0654	GO:0071496	<1e-4	slr0596	GO:0022857	<1e-4	slr1896	GO:0000166	0.1598
sll1251	GO:0009056	<1e-4	sll1884	GO:0042455	<1e-4	slr0287	GO:0080090	<1e-4	slr0146	GO:0016741	<1e-4	sll0508	GO:0000166	0.1598
sll1730	GO:0046907	<1e-4	sll1433	GO:0009987	<1e-4	sll1749	GO:0009150	<1e-4	sll1390	GO:0015077	<1e-4	slr0551	GO:0000166	0.1598
slr1468	GO:0009142	<1e-4	slr1681	GO:0072528	<1e-4	slr1611	GO:0034641	<1e-4	sll0802	GO:0015293	<1e-4	slr0852	GO:0000166	0.1598
ssl3382	GO:0055086	<1e-4	sll0783	GO:0019752	<1e-4	sll0423	GO:0071554	<1e-4	slr1122	GO:0016818	<1e-4	sll0148	GO:0000166	0.1598
ssl2138	GO:0046907	<1e-4	ssl7045	GO:0034654	<1e-4	slr0496	GO:0051234	<1e-4	ssr2711	GO:0015291	<1e-4	sll0274	GO:0000166	0.1598

ssr2755	GO:0043436	<1e-4	sll0861	GO:0009987	<1e-4	sll1476	GO:0072521	<1e-4	ssr1041	GO:0022832	<1e-4	ssr1375	GO:0000166	0.1598
ssr3154	GO:0071496	<1e-4	sll1025	GO:0009141	<1e-4	slr0912	GO:0048523	<1e-4	slr0169	GO:0016462	<1e-4	sll1318	GO:0000166	0.1598
slr0948	GO:0034654	<1e-4	sll0068	GO:0009987	<1e-4	sll7034	GO:0006631	<1e-4	slr0232	GO:0015267	<1e-4	slr1612	GO:0000166	0.1598
slr0980	GO:0006766	<1e-4	slr0605	GO:0048522	<1e-4	slr0912	GO:0009144	<1e-4	slr0976	GO:0022832	<1e-4	sll1433	GO:0000166	0.1598
slr1260	GO:0006811	<1e-4	sll5132	GO:0051171	<1e-4	ssr6085	GO:0044283	<1e-4	sll0656	GO:0022836	<1e-4	ssr3532	GO:0000166	0.1598
slr0575	GO:0006066	<1e-4	slr1258	GO:0044106	<1e-4	ssl5025	GO:0031323	<1e-4	slr2119	GO:0016741	<1e-4	slr1107	GO:0000166	0.1598
sll0024	GO:0009117	<1e-4	slr1668	GO:0044271	<1e-4	slr6033	GO:0050794	<1e-4	slr1767	GO:0060089	<1e-4	slr1196	GO:0000166	0.1598
sll1611	GO:0009150	<1e-4	sll0314	GO:0034654	<1e-4	sll1858	GO:0006766	<1e-4	slr0172	GO:0022838	<1e-4	slr1023	GO:0000166	0.1598
ssr7035	GO:0016053	<1e-4	slr6009	GO:0055086	<1e-4	sll1680	GO:0065007	<1e-4	sll0863	GO:0043492	<1e-4	slr1066	GO:0000166	0.1598
ssr7036	GO:0048518	<1e-4	slr1262	GO:0034641	<1e-4	sll7050	GO:0055082	<1e-4	slr1599	GO:0022832	<1e-4	sll1219	GO:0000166	0.1598
slr0821	GO:0009889	<1e-4	slr1339	GO:0016053	<1e-4	sll1939	GO:0009144	<1e-4	slr0318	GO:0022890	<1e-4	slr1799	GO:0000166	0.1598
sll7050	GO:0009893	<1e-4	sll0068	GO:0048523	<1e-4	slr5077	GO:0072522	<1e-4	slr1365	GO:0022803	<1e-4	slr1222	GO:0000166	0.1598
sll1630	GO:0052111	<1e-4	slr1275	GO:0050896	<1e-4	slr1864	GO:0044282	<1e-4	slr0059	GO:0016741	<1e-4	sll1455	GO:0000166	0.1598
slr0695	GO:0044271	<1e-4	sll0274	GO:0031323	<1e-4	slr1911	GO:0009259	<1e-4	slr2032	GO:0022836	<1e-4	slr1471	GO:0016491	0.1575
slr0272	GO:0009262	<1e-4	sll0216	GO:0006066	<1e-4	slr0273	GO:0010468	<1e-4	sll0319	GO:0022891	<1e-4	slr6031	GO:0000166	0.157
ssr2998	GO:0009059	<1e-4	sll0296	GO:0006720	<1e-4	sll8019	GO:0080090	<1e-4	ssr3572	GO:0022891	<1e-4	sll7062	GO:0000166	0.157
sll0867	GO:0046907	<1e-4	sll1155	GO:0072521	<1e-4	slr7100	GO:0060255	<1e-4	sll0875	GO:0015267	<1e-4	slr1484	GO:0000166	0.157
slr1070	GO:0008150	<1e-4	slr1407	GO:0045184	<1e-4	slr0366	GO:0051171	<1e-4	slr1699	GO:0022836	<1e-4	slr1187	GO:0000166	0.157
slr0887	GO:0009308	<1e-4	ssr0332	GO:0006631	<1e-4	slr2010	GO:0060255	<1e-4	sll0811	GO:0043492	<1e-4	sll0369	GO:0000166	0.157
slr1173	GO:0044283	<1e-4	slr1999	GO:0009991	<1e-4	slr0318	GO:0052111	<1e-4	sll0216	GO:0022838	<1e-4	sll7043	GO:0000166	0.157
slr1470	GO:0051818	<1e-4	sll0448	GO:0009150	<1e-4	sll1163	GO:0048522	<1e-4	ssl2420	GO:0022857	<1e-4	sll5033	GO:0000166	0.157
sll0997	GO:0019222	<1e-4	sll1024	GO:0009165	<1e-4	ssr6030	GO:0009123	<1e-4	sll0742	GO:0022803	<1e-4	sll2015	GO:0000166	0.157
slr7094	GO:0009260	<1e-4	slr0408	GO:0044260	<1e-4	slr6012	GO:0044248	<1e-4	slr1471	GO:0022832	<1e-4	sll1401	GO:0000166	0.157
sll0496	GO:0006811	<1e-4	sll0788	GO:0031326	<1e-4	sll1979	GO:0009991	<1e-4	sll1022	GO:0016746	<1e-4	slr0059	GO:0000166	0.157
slr0168	GO:0051171	<1e-4	sll1915	GO:0044283	<1e-4	sll1166	GO:0019362	<1e-4	slr1210	GO:0015075	<1e-4	sll1906	GO:0000166	0.157
slr2092	GO:0046907	<1e-4	slr0300	GO:0044255	<1e-4	slr6074	GO:0009259	<1e-4	slr1474	GO:0015399	<1e-4	slr7059	GO:0000166	0.157
sll1736	GO:0016053	<1e-4	slr1812	GO:0033013	<1e-4	slr6100	GO:0044106	<1e-4	sll0048	GO:0015293	<1e-4	slr1116	GO:0000166	0.157
sll1915	GO:0009165	<1e-4	sll0577	GO:0019219	<1e-4	sll0102	GO:0072527	<1e-4	slr1600	GO:0060089	<1e-4	ssl5098	GO:0000166	0.157
slr1676	GO:0009150	<1e-4	slr0386	GO:0072524	<1e-4	slr1034	GO:0051171	<1e-4	sll1218	GO:0022836	<1e-4	slr1378	GO:0000166	0.157
sll1726	GO:0009889	<1e-4	slr1152	GO:0060255	<1e-4	sll0162	GO:0009987	<1e-4	slr1951	GO:0015293	<1e-4	ssl3383	GO:0000166	0.157
slr1774	GO:0034654	<1e-4	slr1601	GO:0001932	<1e-4	slr0692	GO:0009260	<1e-4	sll1106	GO:0016818	<1e-4	sll1389	GO:0000166	0.157
ssl1464	GO:0060255	<1e-4	ssl5091	GO:0043436	<1e-4	slr1073	GO:0043412	<1e-4	slr1425	GO:0016741	<1e-4	sll1961	GO:0000166	0.157
slr1087	GO:0043412	<1e-4	sll0743	GO:0031640	<1e-4	slr1990	GO:0009161	<1e-4	sll1170	GO:0016818	<1e-4	slr6064	GO:0000166	0.157
sll0932	GO:0050896	<1e-4	sll1086	GO:0031326	<1e-4	sll1252	GO:0016053	<1e-4	ssr2787	GO:0022891	<1e-4	slr1619	GO:0000166	0.157
sll1715	GO:0006576	<1e-4	slr1110	GO:0051188	<1e-4	slr7100	GO:0016053	<1e-4	sll1433	GO:0015291	<1e-4	ssl5007	GO:0000166	0.157
slr1484	GO:0006721	<1e-4	sll0188	GO:0050794	<1e-4	sll0314	GO:0006811	<1e-4	slr0645	GO:0022803	<1e-4	sll1063	GO:0000166	0.157
ssr3341	GO:0009309	<1e-4	slr1342	GO:0006733	<1e-4	sll0577	GO:0050789	<1e-4	ssl5129	GO:0008171	<1e-4	slr1097	GO:0000166	0.157
slr1914	GO:0048518	<1e-4	sll0980	GO:0048519	<1e-4	ssl0353	GO:0051186	<1e-4	slr0111	GO:0022832	<1e-4	sll0406	GO:0000166	0.157
sll0249	GO:0046164	<1e-4	slr1847	GO:0006811	<1e-4	slr1258	GO:0009057	<1e-4	slr1913	GO:0022892	<1e-4	ssl0312	GO:0000166	0.157
sll0183	GO:0009124	<1e-4	slr0305	GO:0006220	<1e-4	sll0982	GO:0046128	<1e-4	slr6091	GO:0015267	<1e-4	sll0328	GO:0000166	0.157
slr1998	GO:0072528	<1e-4	ssr3571	GO:0006766	<1e-4	ssr2781	GO:0005996	<1e-4	sll0253	GO:0022803	<1e-4	slr1863	GO:0000166	0.157
sll1722	GO:0009309	<1e-4	sll1702	GO:0006793	<1e-4	sll1542	GO:0009892	<1e-4	sll0749	GO:0015291	<1e-4	ssr1499	GO:0000166	0.157
sll0910	GO:0071841	<1e-4	sll1381	GO:0044275	<1e-4	slr6066	GO:0006766	<1e-4	sll1285	GO:0003674	<1e-4	slr7016	GO:0000166	0.157
slr2070	GO:0065007	<1e-4	sll0615	GO:0009260	<1e-4	slr6064	GO:0019362	<1e-4	sll1381	GO:0022857	<1e-4	slr0971	GO:0000166	0.157
sll0898	GO:0009892	<1e-4	slr5017	GO:0042455	<1e-4	slr0195	GO:0008610	<1e-4	slr1601	GO:0015077	<1e-4	slr1365	GO:0000166	0.157
sll0742	GO:0051186	<1e-4	sll1089	GO:0019222	<1e-4	sll0410	GO:0008150	<1e-4	sll0216	GO:0022832	<1e-4	slr1923	GO:0000166	0.157
ssr2803	GO:0048522	<1e-4	sll1630	GO:0044248	<1e-4	slr5119	GO:0050794	<1e-4	slr1081	GO:0043492	<1e-4	slr1957	GO:0000166	0.157
slr1413	GO:0009260	<1e-4	sll0394	GO:0051246	<1e-4	ssl5008	GO:0006220	<1e-4	sll0175	GO:0060089	<1e-4	sll1062	GO:0000166	0.157
slr7037	GO:0009987	<1e-4	slr0431	GO:0046164	<1e-4	ssl0738	GO:0046907	<1e-4	sll1512	GO:0003674	<1e-4	slr0337	GO:0000166	0.157
slr5024	GO:0009126	<1e-4	slr6066	GO:0051347	<1e-4	ssl8005	GO:0052111	<1e-4	slr0273	GO:0022857	<1e-4	ssl0461	GO:0000166	0.157
ssr6019	GO:0072528	<1e-4	sll8040	GO:0071840	<1e-4	slr6015	GO:0048523	<1e-4	sll1925	GO:0016817	<1e-4	sll2013	GO:0000166	0.157
slr6091	GO:0048878	<1e-4	sll1830	GO:0019219	<1e-4	sll0294	GO:0031640	<1e-4	ssr6089	GO:0022832	<1e-4	slr1920	GO:0000166	0.157
slr1365	GO:0009889	<1e-4	sll0062	GO:0031326	<1e-4	slr1863	GO:0044281	<1e-4	slr1177	GO:0042623	<1e-4	sll1651	GO:0000166	0.157
slr6106	GO:0071496	<1e-4	sll1891	GO:0009308	<1e-4	slr6033	GO:0043648	<1e-4	sll0752	GO:0015077	<1e-4	ssl1464	GO:0000166	0.157

ssl3829	GO:0009059	<1e-4	slr0249	GO:0072528	<1e-4	sll0645	GO:0016054	<1e-4	slr1762	GO:0022892	<1e-4	sll1400	GO:0000166	0.157
sll0242	GO:0051171	<1e-4	sll0325	GO:0006066	<1e-4	ssl7048	GO:0050794	<1e-4	ssl2781	GO:0022892	<1e-4	slr7098	GO:0000166	0.157
ssr3129	GO:0019637	<1e-4	slr0606	GO:0071842	<1e-4	sll0149	GO:0044003	<1e-4	sll1832	GO:0022857	<1e-4	ssl1923	GO:0000166	0.157
sll0068	GO:0051179	<1e-4	slr1209	GO:0019219	<1e-4	sll0181	GO:0022607	<1e-4	sll0815	GO:0016818	<1e-4	sll1863	GO:0000166	0.157
slr0914	GO:0044283	<1e-4	sll1773	GO:0065007	<1e-4	ssl2069	GO:0019222	<1e-4	slr1726	GO:0015405	<1e-4	sll1866	GO:0000166	0.157
sll0911	GO:0009141	<1e-4	slr1056	GO:0071841	<1e-4	slr1535	GO:0006766	<1e-4	slr1266	GO:0022803	<1e-4	ssl8003	GO:0000166	0.157
ssl1972	GO:0044275	<1e-4	slr1415	GO:0031323	<1e-4	sll0586	GO:0042455	<1e-4	sll0022	GO:0016818	<1e-4	slr0592	GO:0000166	0.157
ssl7039	GO:0071840	<1e-4	slr7102	GO:0008150	<1e-4	sll1853	GO:0009123	<1e-4	slr0151	GO:0015293	<1e-4	sll0436	GO:0000166	0.157
ssr1375	GO:0009199	<1e-4	slr6065	GO:0016052	<1e-4	slr1070	GO:0065007	<1e-4	sgl0001	GO:0016462	<1e-4	sll5061	GO:0000166	0.157
slr1900	GO:0001932	<1e-4	sll0786	GO:0042451	<1e-4	slr1464	GO:0009987	<1e-4	sll7070	GO:0016462	<1e-4	sll0008	GO:0000166	0.157
slr7080	GO:0046483	<1e-4	sll1785	GO:0009123	<1e-4	slr0654	GO:0009892	<1e-4	ssl0352	GO:0016818	<1e-4	sll7064	GO:0000166	0.157
slr1918	GO:0048878	<1e-4	slr1537	GO:0065007	<1e-4	sll0756	GO:0016053	<1e-4	slr0199	GO:0015075	<1e-4	slr7014	GO:0000166	0.157
sll0007	GO:0052188	<1e-4	slr1768	GO:0051716	<1e-4	slr1353	GO:0009199	<1e-4	ssr6083	GO:0022836	<1e-4	sll1696	GO:0000166	0.157
ssl5100	GO:0050794	<1e-4	slr0146	GO:0044255	<1e-4	sll0024	GO:0009165	<1e-4	sll0354	GO:0060089	<1e-4	slr0262	GO:0000166	0.157
slr0291	GO:0043933	<1e-4	ssr3467	GO:0052111	<1e-4	sll0518	GO:0044106	<1e-4	slr1079	GO:0060089	<1e-4	ssl5096	GO:0000166	0.157
slr1866	GO:0044003	<1e-4	slr0151	GO:0019752	<1e-4	slr1513	GO:0051246	<1e-4	sll1472	GO:0015399	<1e-4	slr1168	GO:0000166	0.157
slr1082	GO:0009165	<1e-4	ssl5015	GO:0051716	<1e-4	ssr2754	GO:0009142	<1e-4	sll1570	GO:0015075	<1e-4	slr0038	GO:0000166	0.157
ssl1520	GO:0022411	<1e-4	ssr1407	GO:0001932	<1e-4	sll0372	GO:0044248	<1e-4	slr0148	GO:0016746	<1e-4	sll1757	GO:0000166	0.157
slr1097	GO:0071496	<1e-4	ssl7021	GO:0043648	<1e-4	slr1914	GO:0055082	<1e-4	ssl3382	GO:0015075	<1e-4	sll0423	GO:0000166	0.157
sll0811	GO:0016054	<1e-4	sll1956	GO:0048519	<1e-4	slr2144	GO:0065007	<1e-4	ssl1552	GO:0016462	<1e-4	slr6021	GO:0000166	0.157
sll0749	GO:0009262	<1e-4	ssl0739	GO:0006220	<1e-4	slr1619	GO:0005996	<1e-4	slr1438	GO:0043492	<1e-4	slr1260	GO:0000166	0.157
sll1174	GO:0022411	<1e-4	slr1069	GO:0009150	<1e-4	ssl2245	GO:0009144	<1e-4	ssr0536	GO:0060089	<1e-4	sll0218	GO:0000166	0.157
slr0588	GO:0052188	<1e-4	slr1174	GO:0009126	<1e-4	slr1097	GO:0044281	<1e-4	sll0180	GO:0060089	<1e-4	slr0959	GO:0000166	0.157
slr5101	GO:0009165	<1e-4	slr0263	GO:0042455	<1e-4	slr1303	GO:0033013	<1e-4	slr0147	GO:0016462	<1e-4	sll1769	GO:0000166	0.157
slr0642	GO:0016052	<1e-4	sll0102	GO:0016052	<1e-4	slr0975	GO:0048522	<1e-4	slr0241	GO:0016818	<1e-4	sll1853	GO:0000166	0.157
sll1164	GO:0019362	<1e-4	sll5132	GO:0006733	<1e-4	slr6028	GO:0055114	<1e-4	sll1373	GO:0008171	<1e-4	slr6073	GO:0000166	0.157
sll0995	GO:0006631	<1e-4	slr7081	GO:0051171	<1e-4	slr0645	GO:0034641	<1e-4	slr7091	GO:0005342	<1e-4	sll5090	GO:0000166	0.157
ssl2996	GO:0031326	<1e-4	slr0816	GO:0006733	<1e-4	slr0196	GO:0009199	<1e-4	slr1378	GO:0022891	<1e-4	ssr1766	GO:0000166	0.157
slr0912	GO:0006720	<1e-4	slr6014	GO:0016052	<1e-4	slr1084	GO:0016052	<1e-4	sll1155	GO:0042623	<1e-4	slr2101	GO:0000166	0.157
sll1174	GO:0042180	<1e-4	slr1681	GO:0050789	<1e-4	sll1232	GO:0046483	<1e-4	slr1194	GO:0022836	<1e-4	sll1217	GO:0000166	0.157
sll0760	GO:0072527	<1e-4	ssr3341	GO:0072522	<1e-4	slr0482	GO:0016052	<1e-4	ssl5096	GO:0022891	<1e-4	slr1546	GO:0000166	0.157
sll1486	GO:0055082	<1e-4	slr0217	GO:0009056	<1e-4	ssl5103	GO:0006220	<1e-4	sll1692	GO:0022890	<1e-4	sll1722	GO:0000166	0.157
sll0669	GO:0050801	<1e-4	slr6013	GO:0046483	<1e-4	slr0038	GO:0046128	<1e-4	slr2111	GO:0043492	<1e-4	sll0282	GO:0000166	0.157
sll0413	GO:0046128	<1e-4	slr5126	GO:0009987	<1e-4	sll7055	GO:0051347	<1e-4	sll1737	GO:0015075	<1e-4	ssr6079	GO:0000166	0.157
ssr1951	GO:0048522	<1e-4	slr2071	GO:0009123	<1e-4	ssl1255	GO:0051188	<1e-4	slr1920	GO:0022857	<1e-4	sll1735	GO:0000166	0.157
sll1764	GO:0044255	<1e-4	sll0359	GO:0006793	<1e-4	sll0787	GO:0046128	<1e-4	slr1338	GO:0022803	<1e-4	sll5006	GO:0000166	0.157
slr6063	GO:0043648	<1e-4	sll0872	GO:0072521	<1e-4	sll0309	GO:0009263	<1e-4	ssr2317	GO:0005342	<1e-4	slr1721	GO:0000166	0.157
slr1762	GO:0034654	<1e-4	slr6066	GO:0010468	<1e-4	ssr7035	GO:0052111	<1e-4	slr0272	GO:0015293	<1e-4	slr0784	GO:0000166	0.157
slr2011	GO:0034641	<1e-4	slr6014	GO:0048523	<1e-4	sll0274	GO:0008150	<1e-4	sll1442	GO:0022857	<1e-4	ssl2009	GO:0000166	0.157
ssl5100	GO:0009991	<1e-4	sll1862	GO:0052188	<1e-4	sll7065	GO:0006733	<1e-4	sll1348	GO:0003674	<1e-4	sll5034	GO:0000166	0.157
sll0428	GO:0009991	<1e-4	slr1276	GO:0009987	<1e-4	slr6106	GO:0034654	<1e-4	sll5033	GO:0022890	<1e-4	ssl2781	GO:0000166	0.157
ssl0750	GO:0050789	<1e-4	slr6008	GO:0009889	<1e-4	slr0480	GO:0019752	<1e-4	sll1304	GO:0042623	<1e-4	sll0168	GO:0000166	0.157
slr2120	GO:0006631	<1e-4	ssl0467	GO:0018130	<1e-4	sll1130	GO:0055086	<1e-4	slr0948	GO:0060089	<1e-4	ssr5020	GO:0000166	0.157
sll1737	GO:0006576	<1e-4	slr1261	GO:0010556	<1e-4	slr1944	GO:0009892	<1e-4	slr0769	GO:0022832	<1e-4	slr0388	GO:0000166	0.157
sll0518	GO:0051186	<1e-4	slr1927	GO:0072527	<1e-4	ssr1391	GO:0046483	<1e-4	slr0232	GO:0022803	<1e-4	ssr6020	GO:0000166	0.157
ssr7093	GO:0009889	<1e-4	sll0997	GO:0044281	<1e-4	sll1675	GO:0050801	<1e-4	sll1472	GO:0022891	<1e-4	sll0293	GO:0000166	0.157
slr0179	GO:0051188	<1e-4	slr0262	GO:0009262	<1e-4	slr0871	GO:0019751	<1e-4	ssl2471	GO:0043167	<1e-4	slr1768	GO:0000166	0.157
slr1437	GO:0034654	<1e-4	slr0374	GO:0090304	<1e-4	slr1169	GO:0050794	<1e-4	sll0647	GO:0016818	<1e-4	ssr7072	GO:0000166	0.157
slr8014	GO:0018130	<1e-4	slr1143	GO:0072524	<1e-4	sll0479	GO:0006732	<1e-4	sll1461	GO:0022803	<1e-4	slr0356	GO:0000166	0.157
sll1764	GO:0044248	<1e-4	slr7012	GO:0022411	<1e-4	slr1474	GO:0006793	<1e-4	sll5004	GO:0022836	<1e-4	slr7015	GO:0000166	0.157
ssr1499	GO:0009117	<1e-4	slr0725	GO:0042180	<1e-4	slr1362	GO:0006721	<1e-4	sll5128	GO:0015075	<1e-4	sll0181	GO:0000166	0.157
sll6055	GO:0060255	<1e-4	ssl2420	GO:0009308	<1e-4	slr1342	GO:0065007	<1e-4	sll0688	GO:0043167	<1e-4	ssl5095	GO:0000166	0.157
sll0496	GO:0071554	<1e-4	sll0359	GO:0043170	<1e-4	sll0785	GO:0072522	<1e-4	sll0982	GO:0043492	<1e-4	slr7080	GO:0000166	0.157
ssr0109	GO:0034660	<1e-4	slr6090	GO:0051818	<1e-4	slr6047	GO:0009259	<1e-4	ssr2611	GO:0003674	<1e-4	sll1265	GO:0000166	0.157

slr1613	GO:0005996	<1e-4	sll1040	GO:0009165	<1e-4	slr0552	GO:0019222	<1e-4	slr1773	GO:0022832	<1e-4	sll0241	GO:0000166	0.157
sll1388	GO:0072527	<1e-4	sll1512	GO:0009124	<1e-4	slr1178	GO:0051188	<1e-4	slr2122	GO:0015291	<1e-4	ssl1004	GO:0000166	0.157
sll1446	GO:0071554	<1e-4	sll0837	GO:0072527	<1e-4	sll0688	GO:0080090	<1e-4	sll1995	GO:0016462	<1e-4	sll1053	GO:0000166	0.157
slr0489	GO:0051818	<1e-4	slr1768	GO:0044283	<1e-4	ssl2162	GO:0072524	<1e-4	slr1406	GO:0022832	<1e-4	sll1232	GO:0000166	0.157
sll1675	GO:0006163	<1e-4	ssl0738	GO:0044281	<1e-4	sll5067	GO:0009987	<1e-4	ssl5103	GO:0022832	<1e-4	sll1511	GO:0000166	0.157
slr7091	GO:0009199	<1e-4	ssl5064	GO:0006811	<1e-4	sll7070	GO:0051716	<1e-4	ssl6061	GO:0015399	<1e-4	sll0444	GO:0000166	0.157
sll0596	GO:0006220	<1e-4	sll1163	GO:0044003	<1e-4	sll1262	GO:0051716	<1e-4	slr0912	GO:0016817	<1e-4	sll8032	GO:0000166	0.157
sll1359	GO:0008150	<1e-4	ssl8028	GO:0006576	<1e-4	slr0871	GO:0044260	<1e-4	slr1223	GO:0008171	<1e-4	slr0479	GO:0000166	0.1568
ssl5008	GO:0055086	<1e-4	slr0751	GO:0009141	<1e-4	slr0848	GO:0051234	<1e-4	sll1062	GO:0022838	<1e-4	sll0183	GO:0000166	0.1568
sll7090	GO:0044271	<1e-4	slr2125	GO:0045184	<1e-4	sll0272	GO:0050794	<1e-4	slr1472	GO:0016818	<1e-4	sll0022	GO:0000166	0.1568
sll5132	GO:0051188	<1e-4	ssr5011	GO:0072521	<1e-4	ssr8047	GO:0043933	<1e-4	sll5130	GO:0016462	<1e-4	slr1307	GO:0000166	0.1568
slr5111	GO:0051716	<1e-4	ssl0352	GO:0006721	<1e-4	slr6012	GO:0055086	<1e-4	ssr6099	GO:0015267	<1e-4	sll1526	GO:0000166	0.1568
slr0496	GO:0050789	<1e-4	slr1306	GO:0006766	<1e-4	sll1906	GO:0009126	<1e-4	slr6049	GO:0016746	<1e-4	slr1047	GO:0000166	0.1568
sll0630	GO:0065007	<1e-4	slr1236	GO:0009059	<1e-4	sll7047	GO:0042451	<1e-4	slr1442	GO:0043167	<1e-4	sll0822	GO:0000166	0.1568
sll1915	GO:0042455	<1e-4	sll1675	GO:0009056	<1e-4	slr0491	GO:0009132	<1e-4	sll1021	GO:0003674	<1e-4	ssr1391	GO:0000166	0.1568
slr1215	GO:0042455	<1e-4	sll0272	GO:0009987	<1e-4	slr6038	GO:0072522	<1e-4	sll1638	GO:0043167	<1e-4	slr1444	GO:0000166	0.1568
slr0453	GO:0010556	<1e-4	sll0508	GO:0080090	<1e-4	slr5077	GO:0019752	<1e-4	slr0770	GO:0022832	<1e-4	slr1847	GO:0000166	0.1568
slr0587	GO:0009142	<1e-4	sll5109	GO:0051818	<1e-4	slr5037	GO:0043648	<1e-4	slr2032	GO:0016462	<1e-4	sll0732	GO:0000166	0.1568
sll1380	GO:0019637	<1e-4	sll1132	GO:0044275	<1e-4	sll1158	GO:0009987	<1e-4	slr0006	GO:0043492	<1e-4	sll1344	GO:0005488	0.1563
sll1735	GO:0052188	<1e-4	sll0394	GO:0050794	<1e-4	slr1052	GO:0046394	<1e-4	ssl2471	GO:0016741	<1e-4	sll1054	GO:0005488	0.1563
slr1110	GO:0044283	<1e-4	sll0286	GO:0009141	<1e-4	sll1399	GO:0052111	<1e-4	sll5132	GO:0015291	<1e-4	slr0483	GO:0005488	0.1563
slr1450	GO:0065007	<1e-4	slr1340	GO:0009059	<1e-4	sll0982	GO:0008610	<1e-4	ssl3615	GO:0015267	<1e-4	sll1730	GO:0005488	0.1563
ssl7022	GO:0044248	<1e-4	sll1608	GO:0009142	<1e-4	slr1397	GO:0019222	<1e-4	slr1365	GO:0022857	<1e-4	slr0742	GO:0005488	0.1563
slr1342	GO:0072521	<1e-4	ssr1765	GO:0051171	<1e-4	ssr2998	GO:0034654	<1e-4	slr5018	GO:0022838	<1e-4	slr0373	GO:0005488	0.1563
sll0281	GO:0048518	<1e-4	sll0372	GO:0034641	<1e-4	ssl3692	GO:0055114	<1e-4	slr5037	GO:0008171	<1e-4	sll0688	GO:0005488	0.1563
ssr2962	GO:0016053	<1e-4	slr1809	GO:0046483	<1e-4	sll1675	GO:0043170	<1e-4	slr6031	GO:0022892	<1e-4	slr1033	GO:0005488	0.1563
slr0613	GO:0043549	<1e-4	sll0752	GO:0019637	<1e-4	sll1784	GO:0019362	<1e-4	slr0468	GO:0005342	<1e-4	slr0989	GO:0005488	0.1563
sll0872	GO:0045184	<1e-4	slr1189	GO:0006811	<1e-4	sll1232	GO:0019637	<1e-4	sll1541	GO:0015077	<1e-4	slr1209	GO:0005488	0.1563
sll1132	GO:0006220	<1e-4	slr1070	GO:0044106	<1e-4	sll1979	GO:0051716	<1e-4	sll1307	GO:0022836	<1e-4	slr0510	GO:0005488	0.1563
sll1950	GO:0009056	<1e-4	sll1446	GO:0044271	<1e-4	sll0424	GO:0016052	<1e-4	slr1471	GO:0042623	<1e-4	slr0172	GO:0005488	0.1563
sll1726	GO:0048522	<1e-4	slr0725	GO:0009056	<1e-4	sll0860	GO:0018130	<1e-4	slr0964	GO:0015399	<1e-4	sll0595	GO:0005488	0.1563
ssl1464	GO:0044260	<1e-4	slr2110	GO:0009056	<1e-4	ssl2138	GO:0006066	<1e-4	slr1591	GO:0016741	<1e-4	sll1352	GO:0005488	0.1563
ssr2755	GO:0009263	<1e-4	slr2025	GO:0044281	<1e-4	slr0294	GO:0009126	<1e-4	sll0611	GO:0016746	<1e-4	slr0249	GO:0005488	0.1563
sll1052	GO:0042180	<1e-4	sll1632	GO:0009308	<1e-4	slr1920	GO:0048523	<1e-4	sll1352	GO:0042623	<1e-4	sll0412	GO:0005488	0.1563
slr5018	GO:0010556	<1e-4	ssl7074	GO:0009259	<1e-4	sll0149	GO:0048522	<1e-4	slr5024	GO:0060089	<1e-4	sll0623	GO:0005488	0.1563
sll0810	GO:0044260	<1e-4	slr1187	GO:0052111	<1e-4	slr2049	GO:0044106	<1e-4	sll1241	GO:0016818	<1e-4	slr1415	GO:0005488	0.1563
slr0619	GO:0009117	<1e-4	slr0217	GO:0009161	<1e-4	ssr2787	GO:0019637	<1e-4	sll0696	GO:0022857	<1e-4	slr1895	GO:0005488	0.1563
slr0702	GO:0018130	<1e-4	sll5063	GO:0051347	<1e-4	slr0356	GO:0044282	<1e-4	sll1352	GO:0022857	<1e-4	slr1613	GO:0005488	0.1563
slr1674	GO:0009893	<1e-4	slr8014	GO:0044248	<1e-4	ssl5007	GO:0016052	<1e-4	slr1788	GO:0015405	<1e-4	ssr1552	GO:0005488	0.1563
sll0405	GO:0051716	<1e-4	sll1119	GO:0043412	<1e-4	slr5116	GO:0009889	<1e-4	sll0630	GO:0016746	<1e-4	slr1977	GO:0005488	0.1563
ssr3532	GO:0005996	<1e-4	ssr2848	GO:0042455	<1e-4	ssl5114	GO:0009892	<1e-4	slr1895	GO:0016818	<1e-4	sll0156	GO:0005488	0.1563
ssr2142	GO:0016051	<1e-4	slr1699	GO:0051179	<1e-4	slr1530	GO:0065007	<1e-4	ssl8008	GO:0016818	<1e-4	sll1660	GO:0005488	0.1563
sll1921	GO:0043933	<1e-4	slr1470	GO:0044248	<1e-4	ssr6099	GO:0043648	<1e-4	slr1880	GO:0022891	<1e-4	slr1809	GO:0005488	0.1563
slr1177	GO:0045184	<1e-4	slr0264	GO:0009057	<1e-4	slr0625	GO:0042180	<1e-4	slr1047	GO:0015075	<1e-4	sll0997	GO:0005488	0.1563
sll0237	GO:0034654	<1e-4	slr1273	GO:0060255	<1e-4	slr0184	GO:0009144	<1e-4	sll0044	GO:0016746	<1e-4	sll0787	GO:0005488	0.1563
sll0710	GO:0016053	<1e-4	slr1383	GO:0006811	<1e-4	sll0216	GO:0006753	<1e-4	slr6075	GO:0022836	<1e-4	sll0147	GO:0005488	0.1563
slr1339	GO:0050801	<1e-4	slr1570	GO:0055086	<1e-4	ssr0657	GO:0044271	<1e-4	sll1002	GO:0015399	<1e-4	sll0740	GO:0005488	0.1563
ssl0352	GO:0072528	<1e-4	ssl0787	GO:0071496	<1e-4	slr0147	GO:0019752	<1e-4	slr1811	GO:0022832	<1e-4	slr0468	GO:0005488	0.1563
slr0521	GO:0071840	<1e-4	sll0822	GO:0009117	<1e-4	ssr2781	GO:0008150	<1e-4	slr0708	GO:0016746	<1e-4	sll1634	GO:0005488	0.1563
sll0160	GO:0051179	<1e-4	slr1110	GO:0022411	<1e-4	sll7067	GO:0006766	<1e-4	slr1647	GO:0022838	<1e-4	slr0596	GO:0005488	0.1563
slr0924	GO:0009260	<1e-4	slr1353	GO:0031640	<1e-4	ssr0335	GO:0019752	<1e-4	ssr6026	GO:0022838	<1e-4	slr0104	GO:0005488	0.1563
slr1203	GO:0051171	<1e-4	sll1938	GO:0009165	<1e-4	ssr1041	GO:0051716	<1e-4	sll0253	GO:0005342	<1e-4	slr0962	GO:0005488	0.1563
sll1348	GO:0005996	<1e-4	slr1667	GO:0051188	<1e-4	sll8011	GO:0046164	<1e-4	slr1236	GO:0015399	<1e-4	slr0815	GO:0005488	0.1563
sll1052	GO:0044248	<1e-4	slr6091	GO:0006163	<1e-4	sll8033	GO:0080090	<1e-4	slr1102	GO:0043492	<1e-4	sll1925	GO:0005488	0.1563

sll1656	GO:0048519	<1e-4	sll1630	GO:0009142	<1e-4	slr0637	GO:0072521	<1e-4	ssr1256	GO:0022857	<1e-4	slr1258	GO:0005488	0.1563
sll0296	GO:0044271	<1e-4	sll1866	GO:0009144	<1e-4	slr1222	GO:0001932	<1e-4	slr0730	GO:0003674	<1e-4	slr0554	GO:0005488	0.1563
sll1611	GO:0043436	<1e-4	sll1613	GO:0052111	<1e-4	sll1089	GO:0072524	<1e-4	slr1095	GO:0022803	<1e-4	slr1287	GO:0005488	0.1563
sll1222	GO:0019637	<1e-4	sll0488	GO:0031640	<1e-4	sll1315	GO:0009056	<1e-4	ssl0738	GO:0015291	<1e-4	slr0053	GO:0005488	0.1563
sll0189	GO:0009124	<1e-4	slr0299	GO:0042455	<1e-4	sll0301	GO:0052111	<1e-4	slr1576	GO:0022890	<1e-4	sll1201	GO:0005488	0.1563
slr0582	GO:0046394	<1e-4	slr1260	GO:0009144	<1e-4	sll0447	GO:0009165	<1e-4	slr6013	GO:0022832	<1e-4	slr1152	GO:0005488	0.1563
slr1999	GO:0051818	<1e-4	slr1920	GO:0044281	<1e-4	slr1612	GO:0022607	<1e-4	slr0545	GO:0022857	<1e-4	slr1762	GO:0005488	0.1563
sll7067	GO:0051188	<1e-4	sll0444	GO:0072522	<1e-4	slr1087	GO:0009308	<1e-4	slr0941	GO:0022891	<1e-4	slr0111	GO:0005488	0.1563
sll8033	GO:0009260	<1e-4	sll1414	GO:0051188	<1e-4	sll1464	GO:0019362	<1e-4	slr0388	GO:0022857	<1e-4	slr1338	GO:0005488	0.1563
slr1122	GO:0051186	<1e-4	sll0783	GO:0009991	<1e-4	ssr1258	GO:0044260	<1e-4	ssr1258	GO:0022892	<1e-4	slr1668	GO:0005488	0.1563
slr2038	GO:0019751	<1e-4	sll0447	GO:0071554	<1e-4	slr1163	GO:0016053	<1e-4	ssl0467	GO:0016462	<1e-4	slr1128	GO:0005488	0.1563
sll1340	GO:0006163	<1e-4	sll1530	GO:0043412	<1e-4	slr2049	GO:0052188	<1e-4	sll7089	GO:0015405	<1e-4	slr0112	GO:0005488	0.1563
slr1951	GO:0006220	<1e-4	sll1652	GO:0031640	<1e-4	slr1376	GO:0043436	<1e-4	slr1450	GO:0016746	<1e-4	sll7065	GO:0005488	0.1563
sll0024	GO:0055086	<1e-4	sll0294	GO:0005996	<1e-4	sll0639	GO:0009394	<1e-4	slr6068	GO:0016818	<1e-4	slr2038	GO:0000166	0.1545
sll0735	GO:0006091	<1e-4	slr0601	GO:0065007	<1e-4	sll1163	GO:0006082	<1e-4	sgl0001	GO:0005342	<1e-4	sll0238	GO:0000166	0.1545
ssr1425	GO:0044281	<1e-4	sll1608	GO:0050789	<1e-4	sll0710	GO:0090304	<1e-4	sll0857	GO:0060089	<1e-4	slr0408	GO:0000166	0.1545
sll0781	GO:0042451	<1e-4	sll1530	GO:0031326	<1e-4	sll0198	GO:0005996	<1e-4	sll0564	GO:0016746	<1e-4	slr0208	GO:0000166	0.1545
sll1155	GO:0055082	<1e-4	ssl7042	GO:0006066	<1e-4	sll1130	GO:0046128	<1e-4	slr7015	GO:0060089	<1e-4	sll1775	GO:0000166	0.1545
sll0861	GO:0065007	<1e-4	slr2105	GO:0009056	<1e-4	ssr1425	GO:0016053	<1e-4	ssl2009	GO:0015405	<1e-4	slr0579	GO:0000166	0.1545
sll8040	GO:0031640	<1e-4	sll0361	GO:0008150	<1e-4	sll1355	GO:0019752	<1e-4	ssr2009	GO:0016817	<1e-4	slr0442	GO:0000166	0.1545
ssr0109	GO:0043549	<1e-4	sll1510	GO:0022607	<1e-4	slr0299	GO:0046907	<1e-4	sll0487	GO:0008171	<1e-4	slr0980	GO:0000166	0.1545
sll0898	GO:0006091	<1e-4	sll1510	GO:0031323	<1e-4	slr0581	GO:0031326	<1e-4	slr1209	GO:0016817	<1e-4	ssr2803	GO:0000166	0.1545
slr0606	GO:0080090	<1e-4	sll6054	GO:0051716	<1e-4	ssl3549	GO:0006220	<1e-4	ssr3572	GO:0008171	<1e-4	ssr1258	GO:0000166	0.1545
slr0431	GO:0046483	<1e-4	ssl5098	GO:0018130	<1e-4	slr2121	GO:0033013	<1e-4	sll0588	GO:0015075	<1e-4	sll0272	GO:0000166	0.1545
ssr6030	GO:0006631	<1e-4	sll1769	GO:0009165	<1e-4	slr1222	GO:0046483	<1e-4	sll0327	GO:0060089	<1e-4	slr0848	GO:0000166	0.1545
slr7099	GO:0071840	<1e-4	slr1519	GO:0051171	<1e-4	sll0735	GO:0048518	<1e-4	sll1956	GO:0022891	<1e-4	ssl3291	GO:0000166	0.1545
sll0630	GO:0042180	<1e-4	ssl5015	GO:0005996	<1e-4	sll1163	GO:0006733	<1e-4	sll1389	GO:0022892	<1e-4	slr1721	GO:0005488	0.1509
sll0325	GO:0009308	<1e-4	sll1240	GO:0042451	<1e-4	slr0634	GO:0019751	<1e-4	slr6051	GO:0015293	<1e-4	slr0598	GO:0005488	0.1509
sll1218	GO:0046128	<1e-4	sll0157	GO:0009161	<1e-4	sll1352	GO:0009987	<1e-4	ssl8003	GO:0016462	<1e-4	slr2105	GO:0005488	0.1509
sll1142	GO:0071554	<1e-4	sll0071	GO:0051347	<1e-4	ssr1499	GO:0009150	<1e-4	sll1068	GO:0016462	<1e-4	sll1265	GO:0005488	0.1509
slr7024	GO:0008150	<1e-4	slr0789	GO:0006066	<1e-4	sll1570	GO:0048518	<1e-4	slr6033	GO:0005342	<1e-4	slr0619	GO:0005488	0.1509
sll7069	GO:0009165	<1e-4	sll7050	GO:0072524	<1e-4	slr1083	GO:0055114	<1e-4	sll0911	GO:0015293	<1e-4	sll0176	GO:0005488	0.1509
slr1647	GO:0051188	<1e-4	sll0691	GO:0065007	<1e-4	sll1880	GO:0031640	<1e-4	slr1073	GO:0015291	<1e-4	slr1958	GO:0005488	0.1509
ssl2471	GO:0044106	<1e-4	sll0423	GO:0065007	<1e-4	slr6012	GO:0042180	<1e-4	slr0142	GO:0042623	<1e-4	slr0049	GO:0005488	0.1509
sll1380	GO:0071842	<1e-4	ssl3692	GO:0009057	<1e-4	sll1340	GO:0022607	<1e-4	slr1097	GO:0015293	<1e-4	sll0265	GO:0005488	0.1509
ssr5106	GO:0010556	<1e-4	slr0605	GO:0071840	<1e-4	slr7073	GO:0080090	<1e-4	slr0619	GO:0016746	<1e-4	sll0775	GO:0005488	0.1509
slr0863	GO:0016053	<1e-4	slr0285	GO:0009057	<1e-4	slr0975	GO:0034641	<1e-4	sll1217	GO:0060089	<1e-4	ssl1417	GO:0005488	0.1509
slr5073	GO:0006811	<1e-4	slr7025	GO:0006220	<1e-4	slr0262	GO:0008610	<1e-4	sll0238	GO:0022892	<1e-4	sll1509	GO:0005488	0.1509
sll1061	GO:0042451	<1e-4	sll0147	GO:0060255	<1e-4	sll0804	GO:0006082	<1e-4	slr1702	GO:0015267	<1e-4	slr6015	GO:0005488	0.1509
ssr6002	GO:0090304	<1e-4	sll1359	GO:0031323	<1e-4	sll0284	GO:0009126	<1e-4	sll0793	GO:0015291	<1e-4	slr1263	GO:0005488	0.1509
sll1424	GO:0009124	<1e-4	sll1396	GO:0022607	<1e-4	slr1702	GO:0006733	<1e-4	sll1773	GO:0015077	<1e-4	slr6039	GO:0005488	0.1509
sll0752	GO:0051171	<1e-4	slr0637	GO:0009117	<1e-4	sll1173	GO:0009262	<1e-4	sll0910	GO:0015291	<1e-4	slr2119	GO:0005488	0.1509
sll0623	GO:0052188	<1e-4	slr0865	GO:0009199	<1e-4	slr0962	GO:0019219	<1e-4	sll1960	GO:0015291	<1e-4	slr0601	GO:0005488	0.1509
sll1119	GO:0046128	<1e-4	sll1086	GO:0010468	<1e-4	slr0692	GO:0043648	<1e-4	slr8021	GO:0016746	<1e-4	ssl0312	GO:0005488	0.1509
sll1265	GO:0009259	<1e-4	slr7026	GO:0071840	<1e-4	sll0167	GO:0034654	<1e-4	slr1721	GO:0005342	<1e-4	slr1168	GO:0005488	0.1509
slr2025	GO:0006091	<1e-4	ssr2551	GO:0071842	<1e-4	sll0174	GO:0055114	<1e-4	sll1089	GO:0022803	<1e-4	sll1004	GO:0005488	0.1509
ssl0353	GO:0044248	<1e-4	sll1233	GO:0071841	<1e-4	sll1442	GO:0009308	<1e-4	slr0801	GO:0016818	<1e-4	sll1764	GO:0005488	0.1509
ssl2069	GO:0009059	<1e-4	slr0863	GO:0009142	<1e-4	sll1583	GO:0050794	<1e-4	slr1398	GO:0015077	<1e-4	slr0151	GO:0005488	0.1509
sll1285	GO:0009991	<1e-4	sll0068	GO:0072524	<1e-4	slr1923	GO:0016054	<1e-4	sll0547	GO:0003674	<1e-4	slr1273	GO:0005488	0.1509
slr7092	GO:0009991	<1e-4	slr0598	GO:0051179	<1e-4	ssr5011	GO:0006793	<1e-4	sll1396	GO:0016741	<1e-4	sll7062	GO:0005488	0.1509
sll8035	GO:0019219	<1e-4	slr1507	GO:0022411	<1e-4	slr0642	GO:0044003	<1e-4	sll0994	GO:0016741	<1e-4	sll0168	GO:0005488	0.1509
slr1384	GO:0009150	<1e-4	slr1169	GO:0031323	<1e-4	ssl1918	GO:0009262	<1e-4	sll0225	GO:0022857	<1e-4	sll0406	GO:0005488	0.1509
sll0498	GO:0010556	<1e-4	slr1819	GO:0019222	<1e-4	slr0491	GO:0009892	<1e-4	slr0810	GO:0042623	<1e-4	sll0727	GO:0005488	0.1509
slr7095	GO:0044283	<1e-4	slr0392	GO:0006721	<1e-4	slr6091	GO:0043436	<1e-4	slr0334	GO:0015077	<1e-4	sll0930	GO:0005488	0.1509

sll1447	GO:0045184	<1e-4	sll1634	GO:0051818	<1e-4	slr0039	GO:0042180	<1e-4	sll0886	GO:0016746	<1e-4	sll1025	GO:0005488	0.1509
ssl1792	GO:0008610	<1e-4	ssr0335	GO:0051186	<1e-4	ssr0759	GO:0010468	<1e-4	slr0376	GO:0015291	<1e-4	sll0815	GO:0005488	0.1509
sll7070	GO:0006733	<1e-4	slr0038	GO:0044260	<1e-4	slr1116	GO:0042455	<1e-4	sll1068	GO:0008171	<1e-4	ssl5045	GO:0005488	0.1509
sll1062	GO:0043436	<1e-4	sll1652	GO:0065007	<1e-4	slr1070	GO:0052111	<1e-4	sll1222	GO:0022832	<1e-4	slr6064	GO:0005488	0.1509
sll1061	GO:0006733	<1e-4	sll5132	GO:0031640	<1e-4	sll1766	GO:0050896	<1e-4	sll0189	GO:0042623	<1e-4	sll5047	GO:0005488	0.1509
ssl0312	GO:0009126	<1e-4	slr7099	GO:0051716	<1e-4	sll1251	GO:0051179	<1e-4	slr5126	GO:0015291	<1e-4	slr1619	GO:0005488	0.1509
ssr3129	GO:0006091	<1e-4	ssr3341	GO:0050794	<1e-4	sll1461	GO:0051347	<1e-4	slr0957	GO:0022832	<1e-4	slr0960	GO:0005488	0.1509
sll1061	GO:0071554	<1e-4	slr2011	GO:0044260	<1e-4	sll0147	GO:0006576	<1e-4	slr1681	GO:0003674	<1e-4	slr7101	GO:0005488	0.1509
sll5030	GO:0042455	<1e-4	slr1721	GO:0072527	<1e-4	ssr2611	GO:0065007	<1e-4	slr1613	GO:0022838	<1e-4	sll1651	GO:0005488	0.1509
ssr2439	GO:0031326	<1e-4	slr1117	GO:0006082	<1e-4	slr1203	GO:0071554	<1e-4	sll0010	GO:0016746	<1e-4	ssr6079	GO:0005488	0.1509
sll1400	GO:0006811	<1e-4	slr0848	GO:0009893	<1e-4	slr0695	GO:0046128	<1e-4	sll6054	GO:0016741	<1e-4	ssr6085	GO:0005488	0.1509
slr0888	GO:0052188	<1e-4	sll0898	GO:0019752	<1e-4	slr1107	GO:0034641	<1e-4	sll7087	GO:0015077	<1e-4	slr0606	GO:0005488	0.1509
slr1179	GO:0055114	<1e-4	ssl3142	GO:0051171	<1e-4	sll0785	GO:0051179	<1e-4	ssr3129	GO:0022857	<1e-4	ssr6026	GO:0005488	0.1509
sll1251	GO:0034641	<1e-4	slr1098	GO:0010556	<1e-4	slr1557	GO:0006721	<1e-4	slr0053	GO:0042623	<1e-4	slr1116	GO:0005488	0.1509
ssr2318	GO:0009165	<1e-4	sll5026	GO:0072521	<1e-4	slr0667	GO:0051246	<1e-4	ssr0692	GO:0022836	<1e-4	slr6013	GO:0005488	0.1509
ssr3154	GO:0051818	<1e-4	slr0400	GO:0031323	<1e-4	slr0208	GO:0019637	<1e-4	ssr2009	GO:0008171	<1e-4	slr1611	GO:0005488	0.1509
slr0169	GO:0006720	<1e-4	sll1906	GO:0042455	<1e-4	slr1505	GO:0072527	<1e-4	slr0291	GO:0016818	<1e-4	sll0008	GO:0005488	0.1509
sll0188	GO:0044275	<1e-4	sll0858	GO:0019752	<1e-4	slr1179	GO:0065007	<1e-4	slr2005	GO:0022832	<1e-4	slr1864	GO:0005488	0.1509
sll0103	GO:0044255	<1e-4	sll0743	GO:0006631	<1e-4	sll1925	GO:0046128	<1e-4	slr0634	GO:0015405	<1e-4	sll0553	GO:0005488	0.1509
sll1563	GO:0034654	<1e-4	ssl1046	GO:0051171	<1e-4	sll1675	GO:0009260	<1e-4	sll5067	GO:0008171	<1e-4	slr0964	GO:0005488	0.1509
sll7050	GO:0006163	<1e-4	sll7062	GO:0046394	<1e-4	sll1004	GO:0009123	<1e-4	sll0913	GO:0008171	<1e-4	sll1862	GO:0005488	0.1509
sll0930	GO:0006066	<1e-4	slr1162	GO:0051347	<1e-4	sll0069	GO:0016052	<1e-4	sll6055	GO:0015293	<1e-4	slr0195	GO:0005488	0.1509
slr8044	GO:0048518	<1e-4	slr1541	GO:0051246	<1e-4	slr1865	GO:0005996	<1e-4	slr2003	GO:0043167	<1e-4	sll1273	GO:0005488	0.1509
ssr3189	GO:0034641	<1e-4	sll0449	GO:0019219	<1e-4	sll1912	GO:0071840	<1e-4	sll1319	GO:0043492	<1e-4	slr1464	GO:0005488	0.1509
ssr6086	GO:0071840	<1e-4	slr1438	GO:0042451	<1e-4	slr0151	GO:0009123	<1e-4	slr1143	GO:0022838	<1e-4	sll1884	GO:0005488	0.1509
sll0071	GO:0006793	<1e-4	sll2006	GO:0006631	<1e-4	slr2032	GO:0046164	<1e-4	slr0680	GO:0022803	<1e-4	ssl2781	GO:0005488	0.1509
sll0216	GO:0009889	<1e-4	sll1191	GO:0006576	<1e-4	sll7031	GO:0006753	<1e-4	sll1164	GO:0005342	<1e-4	sll0328	GO:0005488	0.1509
sll1225	GO:0043549	<1e-4	sll0176	GO:0051179	<1e-4	slr1270	GO:0022607	<1e-4	ssl7045	GO:0043167	<1e-4	ssr1499	GO:0005488	0.1509
sll1606	GO:0051347	<1e-4	sll0861	GO:0055086	<1e-4	sll0149	GO:0006732	<1e-4	ssl5045	GO:0008171	<1e-4	slr1568	GO:0005488	0.1509
slr1437	GO:0072527	<1e-4	slr0740	GO:0022411	<1e-4	sll1906	GO:0050801	<1e-4	slr2122	GO:0022838	<1e-4	slr6080	GO:0005488	0.1509
ssl1552	GO:0051716	<1e-4	ssr3189	GO:0071842	<1e-4	slr6033	GO:0019222	<1e-4	ssl2162	GO:0022832	<1e-4	slr1768	GO:0005488	0.1509
slr1571	GO:0019751	<1e-4	slr0393	GO:0009987	<1e-4	sll1884	GO:0051234	<1e-4	slr0065	GO:0022857	<1e-4	sll1961	GO:0005488	0.1509
slr0273	GO:0052111	<1e-4	slr0976	GO:0071840	<1e-4	ssr2843	GO:0046483	<1e-4	sll0524	GO:0022836	<1e-4	slr1260	GO:0005488	0.1509
ssr1256	GO:0006793	<1e-4	sll1476	GO:0065007	<1e-4	slr0723	GO:0009123	<1e-4	slr1101	GO:0016462	<1e-4	slr0264	GO:0005488	0.1509
sll0360	GO:0043436	<1e-4	ssr3467	GO:0046483	<1e-4	ssl8039	GO:0005996	<1e-4	sll5128	GO:0015291	<1e-4	slr1547	GO:0005488	0.1509
ssl1300	GO:0009126	<1e-4	sll0449	GO:0051234	<1e-4	sll1832	GO:0046483	<1e-4	sll1252	GO:0015399	<1e-4	ssl2920	GO:0005488	0.1509
sll0661	GO:0006733	<1e-4	sll7066	GO:0050801	<1e-4	ssr7035	GO:0006163	<1e-4	slr1778	GO:0043492	<1e-4	sll1160	GO:0005488	0.1509
slr7082	GO:0046907	<1e-4	ssr7079	GO:0042451	<1e-4	sll0047	GO:0009991	<1e-4	slr0602	GO:0016817	<1e-4	sll0293	GO:0005488	0.1509
ssl2420	GO:0009057	<1e-4	slr0586	GO:0006631	<1e-4	ssl2384	GO:0051347	<1e-4	sll1921	GO:0022857	<1e-4	slr2118	GO:0005488	0.1509
slr1636	GO:0080090	<1e-4	sll0497	GO:0044106	<1e-4	slr1062	GO:0044281	<1e-4	ssr2781	GO:0015405	<1e-4	sll0742	GO:0005488	0.1509
slr1753	GO:0006576	<1e-4	slr6008	GO:0072524	<1e-4	slr0613	GO:0051171	<1e-4	sll1960	GO:0022803	<1e-4	sll1510	GO:0005488	0.1509
ssl5098	GO:0051246	<1e-4	slr1107	GO:0034654	<1e-4	ssl5113	GO:0034654	<1e-4	sll1698	GO:0043492	<1e-4	ssl2009	GO:0005488	0.1509
sll0552	GO:0006733	<1e-4	sll1109	GO:0034654	<1e-4	slr0480	GO:0044281	<1e-4	sll1233	GO:0015399	<1e-4	slr0146	GO:0005488	0.1509
ssr2972	GO:0031640	<1e-4	slr0645	GO:0071840	<1e-4	sll5090	GO:0006576	<1e-4	sll1511	GO:0043492	<1e-4	sll0473	GO:0005488	0.1509
sll0996	GO:0044283	<1e-4	sll0414	GO:0055082	<1e-4	sll0749	GO:0009141	<1e-4	sll0280	GO:0015267	<1e-4	ssr7036	GO:0005488	0.1509
sll0905	GO:0016053	<1e-4	sll1289	GO:0042180	<1e-4	slr1519	GO:0044260	<1e-4	slr1187	GO:0016818	<1e-4	sll0448	GO:0005488	0.1509
ssl7007	GO:0009124	<1e-4	sll0298	GO:0010468	<1e-4	ssl0242	GO:0034641	<1e-4	ssl3549	GO:0015077	<1e-4	sll8004	GO:0005488	0.1509
sll0609	GO:0006163	<1e-4	slr1449	GO:0009165	<1e-4	ssl5091	GO:0009123	<1e-4	sll0060	GO:0015077	<1e-4	sll1400	GO:0005488	0.1509
ssr3154	GO:0052188	<1e-4	sll0863	GO:0072524	<1e-4	ssl1918	GO:0043170	<1e-4	sll1532	GO:0016746	<1e-4	slr1097	GO:0005488	0.1509
sll1061	GO:0019752	<1e-4	slr0341	GO:0006733	<1e-4	slr6101	GO:0044255	<1e-4	sll0933	GO:0015405	<1e-4	ssr5020	GO:0005488	0.1509
slr7083	GO:0031326	<1e-4	sll0678	GO:0009142	<1e-4	sll1702	GO:0009892	<1e-4	slr2103	GO:0015291	<1e-4	sll1203	GO:0005488	0.1509
slr1636	GO:0006576	<1e-4	slr5023	GO:0043648	<1e-4	sll1267	GO:0006733	<1e-4	slr1864	GO:0022836	<1e-4	slr7082	GO:0005488	0.1509
ssr1698	GO:0019752	<1e-4	sll1476	GO:0019222	<1e-4	ssl5031	GO:0009263	<1e-4	sll8033	GO:0016746	<1e-4	ssr3341	GO:0005488	0.1509
sll1036	GO:0065007	<1e-4	slr0517	GO:0009893	<1e-4	slr0818	GO:0072522	<1e-4	sll8002	GO:0022838	<1e-4	sll0609	GO:0005488	0.1509

sll0505	GO:0009262	<1e-4	slr1546	GO:0006721	<1e-4	ssl5064	GO:0006066	<1e-4	sll1630	GO:0015077	<1e-4	slr1261	GO:0005488	0.1509
slr1959	GO:0006091	<1e-4	sll1761	GO:0019752	<1e-4	sll1656	GO:0072522	<1e-4	slr0554	GO:0022891	<1e-4	sll1217	GO:0005488	0.1509
slr0092	GO:0006732	<1e-4	sll0933	GO:0072521	<1e-4	ssl8003	GO:0019222	<1e-4	slr5023	GO:0016746	<1e-4	slr1566	GO:0005488	0.1509
slr0730	GO:0009141	<1e-4	sll0424	GO:0051234	<1e-4	slr2032	GO:0044271	<1e-4	sll0860	GO:0015291	<1e-4	sll0751	GO:0005488	0.1509
slr0960	GO:0019752	<1e-4	slr0625	GO:0051818	<1e-4	sll0488	GO:0048519	<1e-4	slr0388	GO:0022803	<1e-4	slr7095	GO:0005488	0.1509
sll0939	GO:0048523	<1e-4	slr0442	GO:0043412	<1e-4	sll0382	GO:0009150	<1e-4	slr1923	GO:0005342	<1e-4	slr1207	GO:0005488	0.1509
sll5047	GO:0043436	<1e-4	sll0436	GO:0080090	<1e-4	slr1223	GO:0005996	<1e-4	sll1512	GO:0022836	<1e-4	slr5118	GO:0005488	0.1509
sll1832	GO:0042180	<1e-4	slr1127	GO:0022411	<1e-4	slr0103	GO:0009161	<1e-4	slr1886	GO:0022891	<1e-4	ssr2554	GO:0005488	0.1509
sll0860	GO:0051246	<1e-4	slr0514	GO:0009117	<1e-4	slr1774	GO:0031640	<1e-4	sll1873	GO:0022836	<1e-4	ssr5120	GO:0005488	0.1509
slr0109	GO:0006091	<1e-4	sll0424	GO:0072527	<1e-4	sll1671	GO:0031326	<1e-4	slr0249	GO:0022832	<1e-4	sll0811	GO:0005488	0.1509
ssl0483	GO:0009394	<1e-4	slr0341	GO:0006793	<1e-4	slr1397	GO:0043436	<1e-4	slr6100	GO:0022836	<1e-4	slr1927	GO:0005488	0.1509
slr1570	GO:0019751	<1e-4	sll0436	GO:0009056	<1e-4	slr1619	GO:0010468	<1e-4	ssr2554	GO:0005342	<1e-4	sll1722	GO:0005488	0.1509
sll1784	GO:0044271	<1e-4	sll5132	GO:0019752	<1e-4	ssl5064	GO:0051171	<1e-4	slr0337	GO:0022857	<1e-4	slr1535	GO:0005488	0.1509
slr1618	GO:0009059	<1e-4	sll0319	GO:0046164	<1e-4	sll1086	GO:0043648	<1e-4	sll0068	GO:0022832	<1e-4	slr1472	GO:0005488	0.1509
ssl5007	GO:0072521	<1e-4	slr1218	GO:0055086	<1e-4	slr0358	GO:0051716	<1e-4	slr0065	GO:0015405	<1e-4	sll1765	GO:0005488	0.1509
sll0736	GO:0044282	<1e-4	slr1907	GO:0006720	<1e-4	slr0848	GO:0044281	<1e-4	sll1691	GO:0015399	<1e-4	slr1677	GO:0005488	0.1509
sll0837	GO:0050789	<1e-4	sll5089	GO:0044271	<1e-4	sll1658	GO:0044275	<1e-4	ssl7022	GO:0016741	<1e-4	sll0478	GO:0005488	0.1509
sll1254	GO:0009262	<1e-4	slr1203	GO:0006082	<1e-4	slr0271	GO:0065007	<1e-4	sll0188	GO:0015291	<1e-4	ssl1498	GO:0005488	0.1509
slr1142	GO:0080090	<1e-4	slr1864	GO:0055082	<1e-4	slr1852	GO:0016054	<1e-4	slr1056	GO:0016817	<1e-4	slr0148	GO:0005488	0.1509
slr5021	GO:0044275	<1e-4	ssl1300	GO:0006793	<1e-4	sll2006	GO:0022607	<1e-4	sll0008	GO:0016746	<1e-4	sll0761	GO:0005488	0.1509
ssl1378	GO:0009165	<1e-4	sll0382	GO:0006066	<1e-4	slr7095	GO:0009124	<1e-4	sll1132	GO:0022892	<1e-4	slr0505	GO:0005488	0.1509
slr0645	GO:0006811	<1e-4	slr1174	GO:0048522	<1e-4	slr1753	GO:0044275	<1e-4	sll0565	GO:0015293	<1e-4	sll8032	GO:0005488	0.1509
sgl0002	GO:0043412	<1e-4	slr0238	GO:0006091	<1e-4	slr1266	GO:0048523	<1e-4	slr0076	GO:0015267	<1e-4	sll5033	GO:0005488	0.1509
slr1507	GO:0043648	<1e-4	slr7098	GO:0008150	<1e-4	slr0392	GO:0043412	<1e-4	sll0765	GO:0022838	<1e-4	ssl5129	GO:0005488	0.1509
sll0294	GO:0044106	<1e-4	slr1101	GO:0009123	<1e-4	slr1222	GO:0018130	<1e-4	ssl2814	GO:0016462	<1e-4	sll5044	GO:0005488	0.1509
slr7010	GO:0009991	<1e-4	sll1835	GO:0060255	<1e-4	slr1935	GO:0055086	<1e-4	sll0172	GO:0060089	<1e-4	sll1571	GO:0005488	0.1509
sll0189	GO:0046394	<1e-4	slr1573	GO:0009144	<1e-4	ssl2749	GO:0044271	<1e-4	sll1638	GO:0015399	<1e-4	slr7015	GO:0005488	0.1509
sll5003	GO:0051171	<1e-4	slr7037	GO:0051716	<1e-4	sll0985	GO:0009059	<1e-4	sll0997	GO:0042623	<1e-4	ssl2245	GO:0005488	0.1509
slr1152	GO:0009126	<1e-4	sll0174	GO:0060255	<1e-4	sll1527	GO:0006631	<1e-4	sll0727	GO:0015077	<1e-4	slr0380	GO:0005488	0.1509
slr1648	GO:0034654	<1e-4	sll1692	GO:0050789	<1e-4	ssr5120	GO:0006576	<1e-4	sll1764	GO:0043167	<1e-4	ssr6048	GO:0005488	0.1509
sll0863	GO:0048522	<1e-4	sll0369	GO:0009126	<1e-4	sll0763	GO:0060255	<1e-4	slr1385	GO:0016817	<1e-4	slr0695	GO:0005488	0.1509
ssr2848	GO:0009117	<1e-4	sll1979	GO:0031323	<1e-4	ssl7021	GO:0016054	<1e-4	ssr3410	GO:0015291	<1e-4	slr1103	GO:0005488	0.1509
slr6008	GO:0043170	<1e-4	ssr6019	GO:0090304	<1e-4	ssr2975	GO:0046907	<1e-4	sll0678	GO:0015267	<1e-4	ssr3410	GO:0005488	0.1509
slr1066	GO:0042451	<1e-4	slr0386	GO:0050896	<1e-4	sll0328	GO:0018130	<1e-4	slr1505	GO:0043167	<1e-4	slr1407	GO:0005488	0.1509
slr0204	GO:0055086	<1e-4	slr0374	GO:0006793	<1e-4	sll2013	GO:0072522	<1e-4	slr1394	GO:0022832	<1e-4	sll0659	GO:0005488	0.1509
ssr6024	GO:0006220	<1e-4	sll1511	GO:0006220	<1e-4	sll0272	GO:0009892	<1e-4	sll0656	GO:0022891	<1e-4	slr1315	GO:0005488	0.1509
slr0142	GO:0060255	<1e-4	slr1505	GO:0019751	<1e-4	sll0875	GO:0034641	<1e-4	slr1116	GO:0022803	<1e-4	slr0769	GO:0005488	0.1509
slr5127	GO:0046128	<1e-4	sll7089	GO:0006793	<1e-4	slr0333	GO:0018130	<1e-4	slr6090	GO:0015291	<1e-4	slr1378	GO:0005488	0.1509
slr0039	GO:0005996	<1e-4	sll1241	GO:0043412	<1e-4	sll1552	GO:0051347	<1e-4	slr1413	GO:0005342	<1e-4	slr0398	GO:0005488	0.1509
slr1601	GO:0044248	<1e-4	sll0314	GO:0009889	<1e-4	sll1659	GO:0009142	<1e-4	sll1570	GO:0016462	<1e-4	sll2013	GO:0005488	0.1509
slr7101	GO:0048878	<1e-4	slr1537	GO:0072524	<1e-4	ssr5019	GO:0072521	<1e-4	slr0957	GO:0060089	<1e-4	slr0065	GO:0005488	0.1509
slr1045	GO:0051171	<1e-4	sll1530	GO:0042455	<1e-4	slr1240	GO:0009394	<1e-4	sll1979	GO:0016462	<1e-4	slr6031	GO:0005488	0.1509
slr1470	GO:0060255	<1e-4	sll0286	GO:0009126	<1e-4	slr0142	GO:0009150	<1e-4	ssl8039	GO:0015075	<1e-4	slr1614	GO:0000166	0.149
slr1383	GO:0010556	<1e-4	slr1537	GO:0051171	<1e-4	slr2025	GO:0055086	<1e-4	slr1917	GO:0015405	<1e-4	ssl0294	GO:0000166	0.149
sll0369	GO:0009991	<1e-4	slr0291	GO:0044255	<1e-4	sll2015	GO:0048519	<1e-4	sll0846	GO:0022838	<1e-4	ssl0750	GO:0000166	0.149
sll0822	GO:0006766	<1e-4	sll0524	GO:0044260	<1e-4	sll1060	GO:0051186	<1e-4	sll5004	GO:0016817	<1e-4	slr0818	GO:0000166	0.1458
sgl0002	GO:0051234	<1e-4	sll0785	GO:0019219	<1e-4	sll5090	GO:0006066	<1e-4	ssr6024	GO:0015077	<1e-4	sll1528	GO:0000166	0.1458
sll1995	GO:0042180	<1e-4	slr1150	GO:0006811	<1e-4	sll0815	GO:0022607	<1e-4	slr0815	GO:0008171	<1e-4	slr1591	GO:0000166	0.1458
slr0505	GO:0010468	<1e-4	slr7100	GO:0006720	<1e-4	slr1362	GO:0006576	<1e-4	slr2011	GO:0016741	<1e-4	sll0446	GO:0000166	0.1458
sll1054	GO:0016053	<1e-4	slr0770	GO:0006766	<1e-4	slr1493	GO:0009141	<1e-4	sll1736	GO:0043492	<1e-4	slr1699	GO:0000166	0.1429
slr6028	GO:0051171	<1e-4	slr1535	GO:0050896	<1e-4	slr0981	GO:0071496	<1e-4	slr6021	GO:0015077	<1e-4	sll0413	GO:0000166	0.1429
slr0813	GO:0051246	<1e-4	sll0319	GO:0016052	<1e-4	ssl8005	GO:0006766	<1e-4	slr1223	GO:0022890	<1e-4	slr1052	GO:0000166	0.1429
ssr2009	GO:0044106	<1e-4	slr7095	GO:0019752	<1e-4	sll0096	GO:0009142	<1e-4	sll1372	GO:0015405	<1e-4	slr0642	GO:0005488	0.1403
sll0703	GO:0019751	<1e-4	sll0066	GO:0046907	<1e-4	sll1773	GO:0071496	<1e-4	ssl7038	GO:0016462	<1e-4	sll0446	GO:0005488	0.1403

sll0451	GO:0052188	<1e-4	slr2084	GO:0009059	<1e-4	slr5126	GO:0009199	<1e-4	slr0092	GO:0016817	<1e-4	sll0656	GO:0005488	0.1403
sll0804	GO:0080090	<1e-4	slr1767	GO:0044255	<1e-4	slr0813	GO:0046164	<1e-4	slr0769	GO:0015077	<1e-4	sll1995	GO:0005488	0.1403
sll1396	GO:0046394	<1e-4	sll0274	GO:0009308	<1e-4	slr0345	GO:0006576	<1e-4	ssr5074	GO:0015293	<1e-4	sll1222	GO:0005488	0.1403
sll5047	GO:0019637	<1e-4	sll7067	GO:0051179	<1e-4	slr1442	GO:0009309	<1e-4	slr0545	GO:0016462	<1e-4	sll1446	GO:0005488	0.1403
ssr1256	GO:0051179	<1e-4	ssl2420	GO:0044255	<1e-4	ssl7038	GO:0009142	<1e-4	ssl3451	GO:0022836	<1e-4	slr1591	GO:0005488	0.1403
sll1130	GO:0048523	<1e-4	sll1372	GO:0044003	<1e-4	slr1186	GO:0009126	<1e-4	slr7082	GO:0022857	<1e-4	ssl2420	GO:0005215	0.1398
slr1241	GO:0006766	<1e-4	slr0602	GO:0048523	<1e-4	sll6053	GO:0016052	<1e-4	slr2144	GO:0022803	<1e-4	ssr3304	GO:0005215	0.1398
sll0982	GO:0009059	<1e-4	slr0334	GO:0043648	<1e-4	slr8021	GO:0009165	<1e-4	sll0980	GO:0043167	<1e-4	slr7097	GO:0005215	0.1398
ssl3142	GO:0019362	<1e-4	ssr0759	GO:0009124	<1e-4	slr0442	GO:0009117	<1e-4	sll0815	GO:0015075	<1e-4	slr1315	GO:0005215	0.1398
slr7094	GO:0051186	<1e-4	slr0408	GO:0043549	<1e-4	sll1315	GO:0019222	<1e-4	sll1601	GO:0016818	<1e-4	sll0266	GO:0005215	0.1398
slr6009	GO:0052188	<1e-4	slr0913	GO:0050794	<1e-4	sll5109	GO:0034654	<1e-4	sll0853	GO:0022838	<1e-4	slr1365	GO:0005215	0.1398
slr1752	GO:0010556	<1e-4	sll1130	GO:0044271	<1e-4	ssl2069	GO:0051171	<1e-4	sll1785	GO:0060089	<1e-4	slr7013	GO:0005215	0.1398
ssr6030	GO:0031323	<1e-4	slr0264	GO:0042180	<1e-4	sll1319	GO:0009394	<1e-4	sll1142	GO:0022832	<1e-4	slr1195	GO:0005215	0.1398
slr1472	GO:0009309	<1e-4	sll0266	GO:0051188	<1e-4	slr0006	GO:0052111	<1e-4	sll0847	GO:0022838	<1e-4	ssr2912	GO:0005215	0.1398
sll0577	GO:0009057	<1e-4	ssl7007	GO:0009987	<1e-4	ssl1923	GO:0019637	<1e-4	sll8011	GO:0016817	<1e-4	slr0049	GO:0005215	0.1398
sll1911	GO:0009263	<1e-4	ssr7079	GO:0006811	<1e-4	slr1851	GO:0034660	<1e-4	slr0510	GO:0022892	<1e-4	ssl3451	GO:0005215	0.1398
slr1570	GO:0046128	<1e-4	ssl1255	GO:0009161	<1e-4	ssl7039	GO:0043933	<1e-4	slr1862	GO:0022857	<1e-4	slr1547	GO:0005215	0.1398
sll1273	GO:0022411	<1e-4	sll1163	GO:0022607	<1e-4	sll0376	GO:0048518	<1e-4	slr1577	GO:0016817	<1e-4	slr7080	GO:0005215	0.1398
sll0752	GO:0009141	<1e-4	sll1632	GO:0048523	<1e-4	sll0047	GO:0043436	<1e-4	ssr6024	GO:0015267	<1e-4	slr6039	GO:0005215	0.1398
ssl0353	GO:0044275	<1e-4	slr1444	GO:0044248	<1e-4	slr1690	GO:0060255	<1e-4	ssr1528	GO:0022891	<1e-4	sll0860	GO:0005215	0.1398
slr6106	GO:0019222	<1e-4	slr5127	GO:0019222	<1e-4	sll1372	GO:0006631	<1e-4	slr5126	GO:0003674	<1e-4	slr6074	GO:0005215	0.1398
ssl0788	GO:0044106	<1e-4	slr1288	GO:0050896	<1e-4	sll1307	GO:0051171	<1e-4	ssl7038	GO:0015405	<1e-4	sll1092	GO:0005215	0.1398
slr1396	GO:0009132	<1e-4	slr1737	GO:0009987	<1e-4	sll1381	GO:0006066	<1e-4	ssr1473	GO:0022857	<1e-4	slr6073	GO:0005215	0.1398
ssl1972	GO:0033013	<1e-4	slr1914	GO:0044282	<1e-4	slr1493	GO:0072524	<1e-4	ssl1464	GO:0022832	<1e-4	ssl2471	GO:0005215	0.1398
slr5111	GO:0006733	<1e-4	slr0780	GO:0034641	<1e-4	sll1089	GO:0071840	<1e-4	sll1372	GO:0016741	<1e-4	slr0598	GO:0005215	0.1398
sll1318	GO:0016052	<1e-4	slr1327	GO:0044283	<1e-4	sll0497	GO:0019219	<1e-4	sll1400	GO:0060089	<1e-4	ssl5098	GO:0005215	0.1398
sll0216	GO:0043549	<1e-4	slr5017	GO:0031640	<1e-4	slr1944	GO:0001932	<1e-4	slr0006	GO:0022891	<1e-4	slr2118	GO:0005215	0.1398
sll1348	GO:0072528	<1e-4	ssl8028	GO:0043549	<1e-4	slr1074	GO:0071842	<1e-4	slr0060	GO:0016741	<1e-4	slr0142	GO:0005215	0.1398
sll8002	GO:0048522	<1e-4	slr0702	GO:0071841	<1e-4	sll0354	GO:0044283	<1e-4	ssl0242	GO:0022892	<1e-4	sll0174	GO:0005215	0.1398
sll1193	GO:0016054	<1e-4	ssl0461	GO:0044282	<1e-4	sll1095	GO:0055086	<1e-4	sll1715	GO:0015405	<1e-4	sll1414	GO:0005215	0.1398
slr0601	GO:0009056	<1e-4	slr0483	GO:0009056	<1e-4	sll0296	GO:0044260	<1e-4	slr0619	GO:0016818	<1e-4	sll1921	GO:0005215	0.1398
slr1069	GO:0034641	<1e-4	sll0172	GO:0009123	<1e-4	slr0476	GO:0048523	<1e-4	slr0498	GO:0005342	<1e-4	slr0362	GO:0005215	0.1398
slr1470	GO:0044106	<1e-4	sll2015	GO:0071840	<1e-4	ssl0788	GO:0009142	<1e-4	slr5073	GO:0015075	<1e-4	sll1509	GO:0005215	0.1398
ssl5103	GO:0051171	<1e-4	sll8033	GO:0044003	<1e-4	sll0499	GO:0006631	<1e-4	sll1735	GO:0043167	<1e-4	slr1660	GO:0005215	0.1398
sll0372	GO:0009309	<1e-4	ssr6089	GO:0044275	<1e-4	slr1557	GO:0048878	<1e-4	slr1627	GO:0005342	<1e-4	slr1223	GO:0005215	0.1398
sll0072	GO:0018130	<1e-4	ssl5129	GO:0022607	<1e-4	slr1338	GO:0055086	<1e-4	sll1505	GO:0043167	<1e-4	ssr7072	GO:0005215	0.1398
ssl3451	GO:0019637	<1e-4	slr1262	GO:0090304	<1e-4	sll0048	GO:0009987	<1e-4	slr7071	GO:0016462	<1e-4	ssr2422	GO:0005215	0.1398
sll1651	GO:0055082	<1e-4	sll1863	GO:0060255	<1e-4	slr0553	GO:0042451	<1e-4	sll0198	GO:0022890	<1e-4	slr0569	GO:0005215	0.1398
slr7026	GO:0006811	<1e-4	slr0689	GO:0042180	<1e-4	sll1225	GO:0009991	<1e-4	sll0047	GO:0008171	<1e-4	sll1757	GO:0005215	0.1398
ssr2201	GO:0042180	<1e-4	slr0049	GO:0006576	<1e-4	sgl0001	GO:0048519	<1e-4	sll1531	GO:0008171	<1e-4	ssl3142	GO:0005215	0.1398
sll1862	GO:0071554	<1e-4	slr1081	GO:0001932	<1e-4	slr1900	GO:0044255	<1e-4	sll1447	GO:0022891	<1e-4	sgl0002	GO:0005215	0.1398
sll1775	GO:0009165	<1e-4	slr0909	GO:0006091	<1e-4	sll1942	GO:0048522	<1e-4	ssr7084	GO:0022891	<1e-4	slr0552	GO:0005215	0.1398
slr2048	GO:0050789	<1e-4	slr1194	GO:0019752	<1e-4	slr1047	GO:0071840	<1e-4	slr1816	GO:0022857	<1e-4	sll1722	GO:0005215	0.1398
sgl0001	GO:0034660	<1e-4	sll1509	GO:0006811	<1e-4	sll1318	GO:0009987	<1e-4	ssr0102	GO:0016817	<1e-4	slr1068	GO:0005215	0.1398
slr1535	GO:0006163	<1e-4	sll0751	GO:0009892	<1e-4	slr0285	GO:0009987	<1e-4	slr7081	GO:0022836	<1e-4	slr1535	GO:0005215	0.1398
slr7092	GO:0043933	<1e-4	sll1378	GO:0051188	<1e-4	slr0770	GO:0051716	<1e-4	sll0508	GO:0015267	<1e-4	sll7066	GO:0005215	0.1398
slr1169	GO:0051246	<1e-4	sll1461	GO:0042180	<1e-4	slr0613	GO:0044248	<1e-4	ssr6086	GO:0022890	<1e-4	slr1600	GO:0005215	0.1398
sll0147	GO:0048878	<1e-4	sll1675	GO:0043436	<1e-4	slr0146	GO:0055082	<1e-4	ssl3692	GO:0016817	<1e-4	slr6072	GO:0005215	0.1398
slr1660	GO:0050794	<1e-4	sll0760	GO:0051186	<1e-4	sll0443	GO:0071554	<1e-4	sll0174	GO:0015399	<1e-4	sll5061	GO:0005215	0.1398
slr0962	GO:0051186	<1e-4	slr1591	GO:0050801	<1e-4	slr0845	GO:0009059	<1e-4	sll1834	GO:0022803	<1e-4	slr1103	GO:0005215	0.1398
slr0941	GO:0009987	<1e-4	sll1613	GO:0043648	<1e-4	sll1738	GO:0051234	<1e-4	slr1568	GO:0016741	<1e-4	sll1160	GO:0005215	0.1398
sll1613	GO:0043933	<1e-4	slr0144	GO:0051716	<1e-4	sll0788	GO:0006091	<1e-4	ssr2067	GO:0008171	<1e-4	slr0092	GO:0005215	0.1398
slr0196	GO:0051818	<1e-4	slr0104	GO:0031323	<1e-4	sll0565	GO:0052188	<1e-4	slr0232	GO:0003674	<1e-4	sll0727	GO:0005215	0.1398
slr0442	GO:0051716	<1e-4	slr1263	GO:0044003	<1e-4	slr1097	GO:0006766	<1e-4	sll0183	GO:0016746	<1e-4	sll0872	GO:0005215	0.1398

ssl0739	GO:0034660	<1e-4	ssl5129	GO:0042455	<1e-4	sll1512	GO:0006811	<1e-4	sll0488	GO:0015405	<1e-4	slr1468	GO:0005215	0.1398
slr1083	GO:0042451	<1e-4	sll0024	GO:0055082	<1e-4	sll1902	GO:0051347	<1e-4	slr8022	GO:0015075	<1e-4	sll0444	GO:0005215	0.1398
slr1362	GO:0060255	<1e-4	ssl5015	GO:0048878	<1e-4	sll1004	GO:0016052	<1e-4	slr1923	GO:0022892	<1e-4	slr1261	GO:0005215	0.1398
sll6054	GO:0019637	<1e-4	slr0650	GO:0006793	<1e-4	sll7065	GO:0045184	<1e-4	sll0274	GO:0016817	<1e-4	slr0971	GO:0005215	0.1398
slr0334	GO:0072521	<1e-4	sll8035	GO:0045184	<1e-4	slr6065	GO:0009144	<1e-4	sll0359	GO:0043492	<1e-4	slr1619	GO:0005215	0.1398
slr1081	GO:0010468	<1e-4	sll1611	GO:0009892	<1e-4	slr1413	GO:0022411	<1e-4	slr1681	GO:0016817	<1e-4	slr7012	GO:0005215	0.1398
slr1541	GO:0009259	<1e-4	slr6044	GO:0043436	<1e-4	sll1355	GO:0034641	<1e-4	sll0933	GO:0022832	<1e-4	slr1753	GO:0005215	0.1398
slr1104	GO:0019637	<1e-4	slr5111	GO:0033013	<1e-4	ssl0750	GO:0006163	<1e-4	slr1940	GO:0015267	<1e-4	slr0151	GO:0005215	0.1398
ssr2554	GO:0006220	<1e-4	sll8019	GO:0010556	<1e-4	sll0400	GO:0009260	<1e-4	slr7011	GO:0022890	<1e-4	slr1464	GO:0005215	0.1398
sll0293	GO:0048522	<1e-4	slr1104	GO:0051347	<1e-4	ssr6002	GO:0044255	<1e-4	slr5112	GO:0003674	<1e-4	sll0518	GO:0005215	0.1398
slr6087	GO:0042455	<1e-4	slr0151	GO:0019222	<1e-4	ssr3467	GO:0043933	<1e-4	slr0668	GO:0016741	<1e-4	sll1461	GO:0005215	0.1398
sll1304	GO:0009150	<1e-4	sll2013	GO:0009056	<1e-4	sll0786	GO:0009987	<1e-4	sll1086	GO:0015399	<1e-4	slr7081	GO:0005215	0.1398
slr0366	GO:0009142	<1e-4	slr0241	GO:0071496	<1e-4	sll0047	GO:0019222	<1e-4	sll1388	GO:0016746	<1e-4	slr1116	GO:0005215	0.1398
sll0451	GO:0048518	<1e-4	ssr2711	GO:0051818	<1e-4	slr1618	GO:0033013	<1e-4	slr0082	GO:0022803	<1e-4	slr1339	GO:0005215	0.1398
ssl1918	GO:0042451	<1e-4	slr0914	GO:0051716	<1e-4	sll0445	GO:0045184	<1e-4	slr0812	GO:0022838	<1e-4	sll0406	GO:0005215	0.1398
slr0262	GO:0051186	<1e-4	sgl0002	GO:0006811	<1e-4	slr0981	GO:0019751	<1e-4	ssr2912	GO:0022892	<1e-4	slr1566	GO:0005215	0.1398
slr1800	GO:0008150	<1e-4	slr0195	GO:0043170	<1e-4	sll1573	GO:0009059	<1e-4	slr6091	GO:0005342	<1e-4	sll0930	GO:0005215	0.1398
sll1002	GO:0009117	<1e-4	sll0446	GO:0009308	<1e-4	sll0736	GO:0048522	<1e-4	sll1532	GO:0022836	<1e-4	slr0196	GO:0005215	0.1398
sll0499	GO:0009987	<1e-4	ssl0352	GO:0019637	<1e-4	ssr2060	GO:0009141	<1e-4	slr1263	GO:0016746	<1e-4	sll1735	GO:0005215	0.1398
sll0268	GO:0019752	<1e-4	slr1576	GO:0019362	<1e-4	slr1415	GO:0009987	<1e-4	slr1177	GO:0015075	<1e-4	sll0218	GO:0005215	0.1398
sll1201	GO:0006733	<1e-4	sll0905	GO:0043549	<1e-4	slr1195	GO:0009889	<1e-4	ssr3572	GO:0060089	<1e-4	sll5034	GO:0005215	0.1398
slr0976	GO:0009892	<1e-4	slr1150	GO:0052111	<1e-4	sll0174	GO:0046128	<1e-4	slr1150	GO:0005342	<1e-4	sll1691	GO:0005215	0.1398
slr1150	GO:0006721	<1e-4	slr1667	GO:0006576	<1e-4	slr0709	GO:0019752	<1e-4	slr1998	GO:0022891	<1e-4	sll1123	GO:0005215	0.1398
sll5004	GO:0042180	<1e-4	slr1195	GO:0006721	<1e-4	slr0380	GO:0050794	<1e-4	slr6006	GO:0016817	<1e-4	sll1132	GO:0005215	0.1398
slr0148	GO:0009141	<1e-4	sll7062	GO:0008150	<1e-4	ssl2595	GO:0048518	<1e-4	sll0982	GO:0005342	<1e-4	sll7043	GO:0005215	0.1398
sll0394	GO:0044282	<1e-4	slr5073	GO:0006091	<1e-4	slr1142	GO:0043549	<1e-4	sll1119	GO:0060089	<1e-4	sll1960	GO:0005215	0.1398
sll0487	GO:0009124	<1e-4	slr6006	GO:0051234	<1e-4	sll1691	GO:0071496	<1e-4	ssl2420	GO:0022892	<1e-4	ssr1951	GO:0005215	0.1398
ssr5019	GO:0055114	<1e-4	sll5028	GO:0010556	<1e-4	slr1353	GO:0045184	<1e-4	sll1640	GO:0022838	<1e-4	slr1957	GO:0005215	0.1398
sll1979	GO:0051171	<1e-4	sll1934	GO:0006163	<1e-4	slr1142	GO:0051347	<1e-4	slr1990	GO:0015291	<1e-4	sll0857	GO:0005215	0.1398
sll0787	GO:0034641	<1e-4	slr1362	GO:0034641	<1e-4	sll1717	GO:0048518	<1e-4	sll1659	GO:0043167	<1e-4	slr6100	GO:0005215	0.1398
sll0676	GO:0051179	<1e-4	sll1131	GO:0009893	<1e-4	slr1854	GO:0006720	<1e-4	sll0837	GO:0005342	<1e-4	ssr6079	GO:0005215	0.1398
slr1104	GO:0044260	<1e-4	slr7060	GO:0006091	<1e-4	sll1222	GO:0042180	<1e-4	slr5101	GO:0015405	<1e-4	sll1053	GO:0005215	0.1398
sll0543	GO:0009123	<1e-4	slr1854	GO:0009260	<1e-4	slr1811	GO:0051234	<1e-4	slr5012	GO:0016818	<1e-4	sll1485	GO:0005215	0.1398
sll0085	GO:0044248	<1e-4	ssr2142	GO:0009123	<1e-4	slr1437	GO:0072524	<1e-4	sll0405	GO:0016817	<1e-4	slr0592	GO:0005215	0.1398
slr0179	GO:0009161	<1e-4	slr0742	GO:0080090	<1e-4	slr6094	GO:0009144	<1e-4	slr0784	GO:0022803	<1e-4	slr0655	GO:0005215	0.1398
ssl8003	GO:0044255	<1e-4	ssr2754	GO:0072522	<1e-4	slr0801	GO:0034654	<1e-4	ssr1391	GO:0022832	<1e-4	slr1419	GO:0005215	0.1398
slr1429	GO:0046164	<1e-4	ssr6026	GO:0009987	<1e-4	sll1476	GO:0051246	<1e-4	slr5053	GO:0022832	<1e-4	slr7098	GO:0005215	0.1398
sll5109	GO:0051716	<1e-4	ssl5008	GO:0009132	<1e-4	sll1024	GO:0050801	<1e-4	slr1417	GO:0015399	<1e-4	sll0448	GO:0005215	0.1398
sll1717	GO:0019362	<1e-4	sll1289	GO:0009123	<1e-4	slr0610	GO:0006091	<1e-4	sll0532	GO:0016462	<1e-4	sll0369	GO:0005215	0.1398
slr1636	GO:0048522	<1e-4	slr1507	GO:0071842	<1e-4	slr2110	GO:0034654	<1e-4	ssl2065	GO:0016817	<1e-4	sll0710	GO:0005215	0.1398
slr0589	GO:0043436	<1e-4	ssl7045	GO:0031640	<1e-4	slr1906	GO:0006753	<1e-4	slr1236	GO:0016817	<1e-4	ssr2611	GO:0005215	0.1398
slr1816	GO:0046394	<1e-4	sll0301	GO:0006793	<1e-4	slr0184	GO:0071842	<1e-4	ssl2245	GO:0043167	<1e-4	slr1470	GO:0005215	0.1398
sll1913	GO:0006631	<1e-4	slr6065	GO:0072524	<1e-4	slr1353	GO:0009132	<1e-4	sll7006	GO:0015075	<1e-4	sll1062	GO:0005215	0.1398
sll1068	GO:0034654	<1e-4	slr0609	GO:0044260	<1e-4	slr0400	GO:0046394	<1e-4	sll0007	GO:0022892	<1e-4	slr1069	GO:0005215	0.1398
slr1780	GO:0043549	<1e-4	sll1714	GO:0071841	<1e-4	sll0280	GO:0006793	<1e-4	sll0436	GO:0043167	<1e-4	slr0146	GO:0005215	0.1398
slr0787	GO:0009893	<1e-4	slr1614	GO:0009991	<1e-4	sll0412	GO:0051347	<1e-4	slr5087	GO:0008171	<1e-4	slr0890	GO:0005215	0.1398
sll7033	GO:0009892	<1e-4	sll0216	GO:0044281	<1e-4	ssr1425	GO:0009144	<1e-4	ssl3383	GO:0022803	<1e-4	sll1004	GO:0005215	0.1398
slr7025	GO:0051186	<1e-4	slr1276	GO:0009893	<1e-4	sll1531	GO:0042180	<1e-4	slr0605	GO:0015291	<1e-4	slr1303	GO:0005215	0.1393
ssr0692	GO:0045184	<1e-4	ssr7036	GO:0010556	<1e-4	slr1472	GO:0033013	<1e-4	slr2060	GO:0043492	<1e-4	slr1415	GO:0005215	0.1393
sll0168	GO:0051171	<1e-4	slr0144	GO:0009987	<1e-4	sll0595	GO:0051234	<1e-4	slr5127	GO:0022892	<1e-4	slr0249	GO:0005215	0.1393
slr0483	GO:0009893	<1e-4	ssr3300	GO:0048522	<1e-4	sll0208	GO:0051818	<1e-4	sll7050	GO:0016741	<1e-4	slr1053	GO:0005215	0.1393
sll0412	GO:0051179	<1e-4	slr7099	GO:0019752	<1e-4	sll0749	GO:0008150	<1e-4	slr1600	GO:0022803	<1e-4	slr1070	GO:0005215	0.1393
sll1736	GO:0009893	<1e-4	sll1132	GO:0052111	<1e-4	ssl2148	GO:0065007	<1e-4	sll0670	GO:0022892	<1e-4	sll0564	GO:0005215	0.1393
sll1158	GO:0065007	<1e-4	sll1934	GO:0071842	<1e-4	slr2018	GO:0009124	<1e-4	sll0185	GO:0022832	<1e-4	slr1062	GO:0005215	0.1393

ssr5019	GO:0072524	<1e-4	slr1998	GO:0071841	<1e-4	slr0770	GO:0009308	<1e-4	slr1944	GO:0015267	<1e-4	slr0169	GO:0005215	0.1393
slr0702	GO:0060255	<1e-4	sll0172	GO:0080090	<1e-4	slr1391	GO:0044248	<1e-4	slr0195	GO:0043492	<1e-4	slr0664	GO:0005215	0.1393
sll1761	GO:0009141	<1e-4	slr0480	GO:0050801	<1e-4	sll0508	GO:0001932	<1e-4	ssl2971	GO:0022832	<1e-4	slr1977	GO:0005215	0.1393
slr1636	GO:0009116	<1e-4	slr0740	GO:0006576	<1e-4	ssl2595	GO:0080090	<1e-4	sll1511	GO:0022832	<1e-4	slr0729	GO:0005215	0.1393
sll1912	GO:0018130	<1e-4	slr0294	GO:0051234	<1e-4	slr7016	GO:0042451	<1e-4	sll0448	GO:0003674	<1e-4	slr0456	GO:0005215	0.1393
slr0816	GO:0046164	<1e-4	sll7055	GO:0009165	<1e-4	slr0602	GO:0044283	<1e-4	slr6072	GO:0008171	<1e-4	sll0585	GO:0005215	0.1393
sll8033	GO:0009117	<1e-4	slr1441	GO:0009057	<1e-4	ssl0738	GO:0048523	<1e-4	sll1188	GO:0043167	<1e-4	slr0172	GO:0005215	0.1393
sll1949	GO:0009161	<1e-4	slr1066	GO:0051716	<1e-4	sll1251	GO:0043412	<1e-4	sll1289	GO:0022836	<1e-4	ssr1552	GO:0005215	0.1393
sll0410	GO:0071554	<1e-4	slr1534	GO:0048522	<1e-4	slr2111	GO:0042455	<1e-4	sll7067	GO:0015291	<1e-4	slr0060	GO:0005215	0.1393
slr6028	GO:0060255	<1e-4	slr0172	GO:0042451	<1e-4	slr0092	GO:0006721	<1e-4	sll1411	GO:0005342	<1e-4	slr1590	GO:0005215	0.1393
slr1033	GO:0042180	<1e-4	sll1173	GO:0048522	<1e-4	sll0263	GO:0034641	<1e-4	slr0815	GO:0022890	<1e-4	ssl0483	GO:0005215	0.1393
slr6103	GO:0051234	<1e-4	slr1956	GO:0009199	<1e-4	sll0157	GO:0043933	<1e-4	sll1940	GO:0016817	<1e-4	sll0685	GO:0005215	0.1393
ssr2009	GO:0006066	<1e-4	slr1230	GO:0031323	<1e-4	slr1900	GO:0016052	<1e-4	slr0082	GO:0022891	<1e-4	slr7024	GO:0005215	0.1393
sll0577	GO:0009199	<1e-4	ssl3142	GO:0048518	<1e-4	slr1544	GO:0044003	<1e-4	slr2101	GO:0042623	<1e-4	ssr2754	GO:0005215	0.1393
slr0476	GO:0071840	<1e-4	slr0285	GO:0006753	<1e-4	slr1789	GO:0043933	<1e-4	sll0063	GO:0022890	<1e-4	sll1873	GO:0005215	0.1393
sll1273	GO:0044282	<1e-4	slr0400	GO:0034654	<1e-4	sll0659	GO:0072522	<1e-4	slr1612	GO:0005342	<1e-4	slr1259	GO:0005215	0.1393
slr0592	GO:0031323	<1e-4	slr1209	GO:0009394	<1e-4	ssr6048	GO:0008150	<1e-4	ssr1765	GO:0060089	<1e-4	slr2125	GO:0005215	0.1393
slr1187	GO:0072528	<1e-4	sll0448	GO:0044282	<1e-4	slr1116	GO:0051234	<1e-4	ssl8008	GO:0016462	<1e-4	sll1021	GO:0005215	0.1393
sll1068	GO:0051246	<1e-4	ssl7038	GO:0009987	<1e-4	ssl1255	GO:0009165	<1e-4	sll1692	GO:0016818	<1e-4	sll0614	GO:0005215	0.1393
sll1512	GO:0046164	<1e-4	slr0655	GO:0019752	<1e-4	sll1681	GO:0072528	<1e-4	sll1164	GO:0043167	<1e-4	slr1288	GO:0005215	0.1393
sll7050	GO:0048523	<1e-4	slr0708	GO:0006082	<1e-4	sll0602	GO:0009056	<1e-4	slr0634	GO:0016817	<1e-4	slr1169	GO:0005215	0.1393
sll1671	GO:0048518	<1e-4	ssl7022	GO:0019222	<1e-4	sll0623	GO:0048878	<1e-4	slr1593	GO:0043492	<1e-4	slr0789	GO:0005215	0.1393
slr6064	GO:0019219	<1e-4	slr1778	GO:0072522	<1e-4	slr0516	GO:0043170	<1e-4	sll1563	GO:0016746	<1e-4	sll0281	GO:0005215	0.1393
sll5062	GO:0072524	<1e-4	sll1054	GO:0051179	<1e-4	slr0196	GO:0019752	<1e-4	sll1123	GO:0043492	<1e-4	sll1938	GO:0005215	0.1393
sll0996	GO:0042180	<1e-4	sll1979	GO:0022607	<1e-4	slr0699	GO:0009889	<1e-4	ssr2755	GO:0016817	<1e-4	slr1690	GO:0005215	0.1393
slr0082	GO:0046128	<1e-4	sll1262	GO:0006220	<1e-4	ssr1528	GO:0009987	<1e-4	slr0872	GO:0015267	<1e-4	slr0887	GO:0005215	0.1393
sll1461	GO:0043549	<1e-4	slr0689	GO:0044106	<1e-4	slr1568	GO:0006811	<1e-4	slr0962	GO:0022803	<1e-4	sll0787	GO:0005215	0.1393
slr1533	GO:0051716	<1e-4	slr6028	GO:0065007	<1e-4	sll0982	GO:0006066	<1e-4	sll0319	GO:0016817	<1e-4	slr1074	GO:0005215	0.1393
slr0453	GO:0043170	<1e-4	slr0668	GO:0033013	<1e-4	sll0596	GO:0016054	<1e-4	slr1398	GO:0016741	<1e-4	sll0297	GO:0005215	0.1393
slr0065	GO:0009987	<1e-4	slr0262	GO:0043412	<1e-4	slr6103	GO:0050789	<1e-4	slr0596	GO:0022892	<1e-4	ssr1114	GO:0005215	0.1393
slr1442	GO:0009056	<1e-4	slr0728	GO:0022607	<1e-4	slr7010	GO:0009309	<1e-4	ssr6027	GO:0043167	<1e-4	sll0911	GO:0005215	0.1393
ssl3379	GO:0019222	<1e-4	slr5127	GO:0044255	<1e-4	slr1307	GO:0071554	<1e-4	sll1348	GO:0015291	<1e-4	sll0861	GO:0005488	0.1366
slr1505	GO:0006732	<1e-4	sll1446	GO:0022411	<1e-4	ssr6019	GO:0052188	<1e-4	sll0369	GO:0060089	<1e-4	sll0602	GO:0016787	0.1361
sll0354	GO:0046164	<1e-4	slr1668	GO:0009394	<1e-4	sll1109	GO:0019751	<1e-4	sll0069	GO:0015405	<1e-4	slr0935	GO:0016787	0.1361
slr1307	GO:0071840	<1e-4	slr1762	GO:0006732	<1e-4	sll0413	GO:0071554	<1e-4	sll0641	GO:0008171	<1e-4	sll1241	GO:0016787	0.1361
sll1866	GO:0044275	<1e-4	slr0554	GO:0034654	<1e-4	slr0380	GO:0009263	<1e-4	slr1110	GO:0015405	<1e-4	slr1541	GO:0016787	0.1361
sll0788	GO:0071842	<1e-4	ssr2318	GO:0050789	<1e-4	slr7058	GO:0009991	<1e-4	sll1832	GO:0043492	<1e-4	slr1413	GO:0016787	0.1361
sll1527	GO:0009262	<1e-4	sll1526	GO:0006720	<1e-4	sll0381	GO:0006811	<1e-4	sll1024	GO:0022836	<1e-4	slr0172	GO:0016787	0.1361
slr0770	GO:0019362	<1e-4	ssl5031	GO:0006733	<1e-4	slr2000	GO:0071840	<1e-4	sll0735	GO:0016741	<1e-4	sll1659	GO:0016787	0.1361
sll0098	GO:0048523	<1e-4	slr0393	GO:0009893	<1e-4	slr1799	GO:0051179	<1e-4	sll1766	GO:0016462	<1e-4	slr2115	GO:0016787	0.1361
ssr0692	GO:0044260	<1e-4	slr1875	GO:0034660	<1e-4	slr2027	GO:0071840	<1e-4	slr2052	GO:0022832	<1e-4	sll1873	GO:0016787	0.1361
slr0588	GO:0019222	<1e-4	sll8002	GO:0006766	<1e-4	ssl2471	GO:0048878	<1e-4	sll1350	GO:0022803	<1e-4	sll1240	GO:0016787	0.1361
sll5063	GO:0019752	<1e-4	sll1476	GO:0048518	<1e-4	slr1122	GO:0044255	<1e-4	sll1390	GO:0022838	<1e-4	ssr1114	GO:0016787	0.1361
slr0249	GO:0050896	<1e-4	slr0816	GO:0046483	<1e-4	sll1390	GO:0050789	<1e-4	slr0978	GO:0022857	<1e-4	sll0319	GO:0016787	0.1361
sll0499	GO:0071842	<1e-4	slr1290	GO:0009141	<1e-4	sll1250	GO:0071841	<1e-4	slr5053	GO:0015075	<1e-4	sll0281	GO:0016787	0.1361
slr1935	GO:0046907	<1e-4	ssl5095	GO:0042180	<1e-4	sll5062	GO:0043170	<1e-4	slr6028	GO:0015267	<1e-4	sll1352	GO:0016787	0.1361
sll5026	GO:0009142	<1e-4	sll1761	GO:0043170	<1e-4	sll5062	GO:0006066	<1e-4	sll0756	GO:0016746	<1e-4	slr0249	GO:0016787	0.1361
sll1608	GO:0016054	<1e-4	slr0592	GO:0051246	<1e-4	slr0907	GO:0009144	<1e-4	sll1898	GO:0022803	<1e-4	slr0232	GO:0016787	0.1361
sll1769	GO:0031640	<1e-4	ssl3291	GO:0071842	<1e-4	sll1979	GO:0044260	<1e-4	slr1690	GO:0022838	<1e-4	slr1690	GO:0016787	0.1361
ssl2245	GO:0001932	<1e-4	slr6004	GO:0034654	<1e-4	sll0263	GO:0050789	<1e-4	sll0216	GO:0022857	<1e-4	slr1152	GO:0016787	0.1361
ssl0467	GO:0044255	<1e-4	slr1819	GO:0022607	<1e-4	slr1209	GO:0009259	<1e-4	ssr3410	GO:0022832	<1e-4	sll1730	GO:0016787	0.1361
slr1073	GO:0044275	<1e-4	slr0689	GO:0009144	<1e-4	sll1938	GO:0019637	<1e-4	slr6088	GO:0005342	<1e-4	sll0590	GO:0016787	0.1361
sll0867	GO:0009893	<1e-4	ssl0294	GO:0018130	<1e-4	slr5118	GO:0006793	<1e-4	slr7057	GO:0022838	<1e-4	slr0924	GO:0016787	0.1361
sll1954	GO:0008150	<1e-4	slr1143	GO:0009262	<1e-4	sll0811	GO:0006766	<1e-4	slr1704	GO:0060089	<1e-4	slr1852	GO:0016787	0.1361

ssr6019	GO:0008150	<1e-4	sll1913	GO:0044260	<1e-4	slr2011	GO:0055114	<1e-4	sll0518	GO:0060089	<1e-4	ssl3382	GO:0016787	0.1361
slr0249	GO:0048518	<1e-4	ssr2962	GO:0071554	<1e-4	sll0577	GO:0034641	<1e-4	slr5087	GO:0022836	<1e-4	slr0654	GO:0016787	0.1361
sll0702	GO:0044275	<1e-4	sll0238	GO:0071841	<1e-4	ssr2962	GO:0016054	<1e-4	sll0751	GO:0016818	<1e-4	ssl8008	GO:0016787	0.1361
slr7015	GO:0019752	<1e-4	ssl2138	GO:0051716	<1e-4	sll1352	GO:0009059	<1e-4	slr0456	GO:0008171	<1e-4	sll1251	GO:0016787	0.1361
slr1915	GO:0052111	<1e-4	sll1251	GO:0044281	<1e-4	slr0598	GO:0009141	<1e-4	slr1103	GO:0022857	<1e-4	slr1287	GO:0016787	0.1361
ssl2471	GO:0008150	<1e-4	slr6094	GO:0072527	<1e-4	ssr2611	GO:0055082	<1e-4	slr0740	GO:0022832	<1e-4	slr0104	GO:0016787	0.1361
ssr6089	GO:0043549	<1e-4	slr1773	GO:0052111	<1e-4	slr1880	GO:0046164	<1e-4	ssl5113	GO:0022832	<1e-4	sll1170	GO:0016787	0.1361
slr1363	GO:0009150	<1e-4	ssr8047	GO:0031323	<1e-4	slr0397	GO:0043436	<1e-4	slr1196	GO:0015077	<1e-4	ssr1552	GO:0016787	0.1361
sll1950	GO:0042455	<1e-4	ssr3409	GO:0006163	<1e-4	sll5047	GO:0006091	<1e-4	sll0565	GO:0016818	<1e-4	slr0650	GO:0016787	0.1361
ssr6086	GO:0022607	<1e-4	slr1601	GO:0050896	<1e-4	sll7063	GO:0019362	<1e-4	ssl2920	GO:0042623	<1e-4	sll0911	GO:0016787	0.1361
sll1155	GO:0034654	<1e-4	ssr3570	GO:0009165	<1e-4	slr0313	GO:0044248	<1e-4	slr0909	GO:0016818	<1e-4	sll0268	GO:0016787	0.1361
slr0168	GO:0006631	<1e-4	sll0410	GO:0044248	<1e-4	slr0144	GO:0009260	<1e-4	slr2038	GO:0022803	<1e-4	slr1895	GO:0016787	0.1361
sll7078	GO:0009142	<1e-4	sll0615	GO:0009309	<1e-4	sll0487	GO:0050794	<1e-4	slr7096	GO:0022838	<1e-4	slr1590	GO:0016787	0.1361
slr1394	GO:0072527	<1e-4	sll1926	GO:0055086	<1e-4	slr0610	GO:0009263	<1e-4	slr0360	GO:0016818	<1e-4	slr0742	GO:0016787	0.1361
sll1979	GO:0009892	<1e-4	sll1092	GO:0009893	<1e-4	slr1800	GO:0044271	<1e-4	slr0645	GO:0043492	<1e-4	sll0740	GO:0016787	0.1361
sll0149	GO:0009141	<1e-4	sll0309	GO:0009199	<1e-4	slr1215	GO:0009259	<1e-4	sll0994	GO:0015291	<1e-4	sll1304	GO:0016787	0.1361
sll1025	GO:0009142	<1e-4	sll2007	GO:0046128	<1e-4	sll1765	GO:0006720	<1e-4	sll0419	GO:0022890	<1e-4	slr0169	GO:0016787	0.1361
sll0189	GO:0009260	<1e-4	sll1979	GO:0010556	<1e-4	ssl2595	GO:0052111	<1e-4	sll0405	GO:0022832	<1e-4	sll0449	GO:0016787	0.1361
sll5132	GO:0009059	<1e-4	ssr2333	GO:0006753	<1e-4	sll0265	GO:0044003	<1e-4	slr2018	GO:0042623	<1e-4	slr1070	GO:0016787	0.1361
sll0282	GO:0009991	<1e-4	slr7024	GO:0006220	<1e-4	slr0594	GO:0009124	<1e-4	sll1036	GO:0060089	<1e-4	ssr2754	GO:0016787	0.1361
sll7030	GO:0051186	<1e-4	sll1884	GO:0072527	<1e-4	sll1267	GO:0044260	<1e-4	sll0436	GO:0015399	<1e-4	sll0539	GO:0016787	0.1361
ssr2201	GO:0044003	<1e-4	ssr1375	GO:0051171	<1e-4	ssr3570	GO:0051171	<1e-4	sll1072	GO:0015399	<1e-4	sll1541	GO:0016787	0.1361
ssr2047	GO:0033013	<1e-4	sll1188	GO:0071840	<1e-4	slr0468	GO:0080090	<1e-4	slr1207	GO:0016741	<1e-4	sll0688	GO:0016787	0.1361
slr0723	GO:0046394	<1e-4	sll7077	GO:0006091	<1e-4	slr0689	GO:0051171	<1e-4	slr1095	GO:0016818	<1e-4	slr0112	GO:0016787	0.1361
sll1071	GO:0009117	<1e-4	ssr6083	GO:0072521	<1e-4	slr6100	GO:0009144	<1e-4	slr1699	GO:0060089	<1e-4	slr1647	GO:0016787	0.1361
slr1875	GO:0006733	<1e-4	sll7062	GO:0019222	<1e-4	slr0962	GO:0044003	<1e-4	slr5087	GO:0022857	<1e-4	sll0141	GO:0016787	0.1361
ssl5015	GO:0006811	<1e-4	sll1307	GO:0009124	<1e-4	ssr0332	GO:0001932	<1e-4	slr1611	GO:0022857	<1e-4	sll1054	GO:0016787	0.1361
slr1959	GO:0018130	<1e-4	ssl3829	GO:0009123	<1e-4	slr0517	GO:0034641	<1e-4	sll5044	GO:0016818	<1e-4	sll1505	GO:0016787	0.1361
sll0096	GO:0072521	<1e-4	slr0039	GO:0072521	<1e-4	slr1307	GO:0010468	<1e-4	slr0103	GO:0022836	<1e-4	slr1074	GO:0016787	0.1361
sll1469	GO:0006091	<1e-4	ssr0657	GO:0009056	<1e-4	slr2038	GO:0019222	<1e-4	sll0225	GO:0022836	<1e-4	sll0752	GO:0016787	0.1361
sll1504	GO:0031640	<1e-4	slr1161	GO:0009132	<1e-4	sll1446	GO:0034654	<1e-4	slr1576	GO:0022803	<1e-4	slr0053	GO:0016787	0.1361
slr1600	GO:0048523	<1e-4	slr7092	GO:0046128	<1e-4	slr1442	GO:0009987	<1e-4	sll0913	GO:0015267	<1e-4	slr1809	GO:0016787	0.1361
sll5062	GO:0016052	<1e-4	slr6065	GO:0031640	<1e-4	slr1183	GO:0031640	<1e-4	slr0605	GO:0022832	<1e-4	slr0483	GO:0016787	0.1361
slr0587	GO:0006766	<1e-4	sll0230	GO:0072528	<1e-4	slr2060	GO:0072524	<1e-4	sll0861	GO:0016741	<1e-4	slr1338	GO:0016787	0.1361
sll0688	GO:0006721	<1e-4	sll1834	GO:0046907	<1e-4	slr1788	GO:0046907	<1e-4	ssl2733	GO:0015399	<1e-4	slr0981	GO:0016787	0.1361
sll1173	GO:0065007	<1e-4	slr0112	GO:0019222	<1e-4	slr1143	GO:0006811	<1e-4	sll1735	GO:0016817	<1e-4	slr0887	GO:0016787	0.1361
slr1270	GO:0072521	<1e-4	sll0376	GO:0044106	<1e-4	slr1032	GO:0034641	<1e-4	slr0609	GO:0016817	<1e-4	sll1267	GO:0016787	0.1361
sll0615	GO:0051246	<1e-4	ssr5020	GO:0009987	<1e-4	slr0818	GO:0052111	<1e-4	slr1391	GO:0015291	<1e-4	sll0871	GO:0016787	0.1361
slr0971	GO:0071840	<1e-4	ssl5113	GO:0009117	<1e-4	sll1130	GO:0046907	<1e-4	slr1236	GO:0005342	<1e-4	sll1411	GO:0016787	0.1361
slr0821	GO:0048522	<1e-4	sll7089	GO:0071496	<1e-4	slr0742	GO:0031326	<1e-4	slr1614	GO:0016462	<1e-4	slr0510	GO:0016787	0.1361
sll1036	GO:0019222	<1e-4	sll1797	GO:0019637	<1e-4	slr1023	GO:0022607	<1e-4	sll1702	GO:0003674	<1e-4	sll1201	GO:0016787	0.1361
slr0337	GO:0016053	<1e-4	slr0609	GO:0009991	<1e-4	sll1411	GO:0006066	<1e-4	ssl3451	GO:0043492	<1e-4	slr7024	GO:0016787	0.1361
sll0008	GO:0006720	<1e-4	slr0579	GO:0051347	<1e-4	slr1541	GO:0065007	<1e-4	sll0162	GO:0022832	<1e-4	sll1071	GO:0016787	0.1361
slr0941	GO:0042455	<1e-4	sll1131	GO:0009991	<1e-4	sll8001	GO:0051188	<1e-4	slr0888	GO:0015267	<1e-4	slr0111	GO:0016787	0.1361
slr1052	GO:0016054	<1e-4	sll1267	GO:0016053	<1e-4	sll0614	GO:0009059	<1e-4	slr0392	GO:0022890	<1e-4	slr1259	GO:0016787	0.1361
sll1119	GO:0006576	<1e-4	slr1143	GO:0043170	<1e-4	slr0821	GO:0071841	<1e-4	sll7047	GO:0043167	<1e-4	sll7065	GO:0016787	0.1361
slr1990	GO:0051716	<1e-4	sll0732	GO:0006082	<1e-4	slr1438	GO:0080090	<1e-4	sll1040	GO:0015077	<1e-4	sll0781	GO:0016787	0.1361
ssl0294	GO:0009144	<1e-4	ssl1972	GO:0006732	<1e-4	sll1630	GO:0051234	<1e-4	slr0885	GO:0022891	<1e-4	sll0888	GO:0016787	0.1361
ssl1577	GO:0016053	<1e-4	slr0553	GO:0006732	<1e-4	ssr1552	GO:0006576	<1e-4	slr0609	GO:0022857	<1e-4	sll0980	GO:0016787	0.1361
ssr6086	GO:0034641	<1e-4	slr1070	GO:0044260	<1e-4	slr0937	GO:0051716	<1e-4	sll1915	GO:0008171	<1e-4	slr0149	GO:0016787	0.1361
slr1097	GO:0006066	<1e-4	sll5003	GO:0042180	<1e-4	sll1040	GO:0009117	<1e-4	sll0252	GO:0016788	<1e-4	slr0680	GO:0016787	0.1361
sll1638	GO:0009260	<1e-4	slr2110	GO:0034641	<1e-4	sll7078	GO:0072528	<1e-4	slr5111	GO:0022890	<1e-4	slr0702	GO:0016787	0.1361
ssr0692	GO:0006766	<1e-4	slr0207	GO:0033013	<1e-4	sll0827	GO:0048522	<1e-4	sll1640	GO:0016462	<1e-4	sll0189	GO:0016787	0.1361
sll0788	GO:0044255	<1e-4	slr1926	GO:0055086	<1e-4	slr1864	GO:0006082	<1e-4	slr1218	GO:0008171	<1e-4	sll1390	GO:0016787	0.1361

sll1344	GO:0065007	<1e-4	slr0145	GO:0009308	<1e-4	sll1233	GO:0045184	<1e-4	sll1485	GO:0005342	<1e-4	slr1240	GO:0016787	0.1361
slr1270	GO:0044260	<1e-4	sll0381	GO:0044281	<1e-4	slr1081	GO:0008610	<1e-4	sll1130	GO:0005342	<1e-4	slr0815	GO:0016787	0.1361
slr1116	GO:0044248	<1e-4	slr0848	GO:0065007	<1e-4	ssr1258	GO:0006793	<1e-4	slr1470	GO:0022838	<1e-4	slr1033	GO:0016787	0.1361
slr1258	GO:0016054	<1e-4	sll2011	GO:0065007	<1e-4	slr0552	GO:0006066	<1e-4	sll0696	GO:0015291	<1e-4	slr0456	GO:0016787	0.1361
slr6038	GO:0051171	<1e-4	sll0518	GO:0048523	<1e-4	sll0611	GO:0046394	<1e-4	sll0925	GO:0022857	<1e-4	sll1660	GO:0016787	0.1361
ssr5074	GO:0045184	<1e-4	slr1563	GO:0019222	<1e-4	sll0585	GO:0009056	<1e-4	ssr5020	GO:0015405	<1e-4	ssl0483	GO:0016787	0.1361
slr5021	GO:0034641	<1e-4	sll1399	GO:0008150	<1e-4	sll0547	GO:0044281	<1e-4	ssr2962	GO:0016818	<1e-4	sll0412	GO:0016787	0.1361
slr0211	GO:0072527	<1e-4	slr1143	GO:0009394	<1e-4	sll1979	GO:0060255	<1e-4	slr1338	GO:0022832	<1e-4	slr1415	GO:0016787	0.1361
ssl8005	GO:0006082	<1e-4	sll0661	GO:0051716	<1e-4	ssl0467	GO:0051186	<1e-4	slr1327	GO:0016817	<1e-4	slr2125	GO:0016787	0.1361
slr0765	GO:0080090	<1e-4	sll1511	GO:0071840	<1e-4	slr1668	GO:0042455	<1e-4	slr0509	GO:0043492	<1e-4	slr1062	GO:0016787	0.1361
sll1378	GO:0001932	<1e-4	slr0272	GO:0044283	<1e-4	sll0564	GO:0009991	<1e-4	slr0038	GO:0022857	<1e-4	slr1658	GO:0016787	0.1361
slr0552	GO:0043412	<1e-4	ssr1114	GO:0044106	<1e-4	sll0310	GO:0009142	<1e-4	slr1301	GO:0022803	<1e-4	slr0468	GO:0016787	0.1361
sll0732	GO:0009142	<1e-4	sll1135	GO:0043549	<1e-4	slr7037	GO:0043648	<1e-4	ssr0759	GO:0016741	<1e-4	ssl0739	GO:0016787	0.1361
ssl2595	GO:0033013	<1e-4	slr7014	GO:0010556	<1e-4	slr1173	GO:0048519	<1e-4	sll1942	GO:0015405	<1e-4	slr1613	GO:0016787	0.1361
slr0300	GO:0010468	<1e-4	sll1671	GO:0046394	<1e-4	sll7087	GO:0006066	<1e-4	sll0263	GO:0015293	<1e-4	sll1372	GO:0016787	0.1361
ssr2975	GO:0019222	<1e-4	slr6074	GO:0051716	<1e-4	sll0996	GO:0071842	<1e-4	slr0625	GO:0022891	<1e-4	sll0359	GO:0016787	0.1361
sll0857	GO:0006220	<1e-4	sll5130	GO:0043170	<1e-4	ssr2201	GO:0009141	<1e-4	sll0861	GO:0016818	<1e-4	ssr2009	GO:0016787	0.1361
sll1219	GO:0052111	<1e-4	slr0723	GO:0044248	<1e-4	sll7063	GO:0016054	<1e-4	slr0610	GO:0022890	<1e-4	sll0180	GO:0016787	0.1361
slr1117	GO:0071554	<1e-4	slr0551	GO:0072524	<1e-4	ssl5091	GO:0071554	<1e-4	ssr2848	GO:0015293	<1e-4	sll0188	GO:0016787	0.1361
sll1717	GO:0042455	<1e-4	sll5069	GO:0042180	<1e-4	sll1132	GO:0080090	<1e-4	sll6054	GO:0003674	<1e-4	slr0348	GO:0005488	0.1355
sll1095	GO:0019751	<1e-4	sll1380	GO:0009991	<1e-4	slr0364	GO:0044283	<1e-4	sll0066	GO:0022803	<1e-4	slr1437	GO:0005488	0.1355
slr0863	GO:0042180	<1e-4	ssl0739	GO:0006733	<1e-4	sll0762	GO:0044003	<1e-4	ssr2972	GO:0015399	<1e-4	sll0414	GO:0005488	0.1355
sll0188	GO:0009394	<1e-4	slr0147	GO:0009260	<1e-4	sll1024	GO:0050794	<1e-4	sll1186	GO:0022891	<1e-4	slr0192	GO:0005488	0.1355
slr1789	GO:0043648	<1e-4	slr0569	GO:0051716	<1e-4	slr0816	GO:0009142	<1e-4	sll0102	GO:0015291	<1e-4	sll0023	GO:0005488	0.1355
slr2032	GO:0050794	<1e-4	slr0655	GO:0018130	<1e-4	sll0803	GO:0006631	<1e-4	slr7073	GO:0015293	<1e-4	ssr2962	GO:0005488	0.1355
slr1168	GO:0006766	<1e-4	sll1036	GO:0050801	<1e-4	slr0602	GO:0080090	<1e-4	slr1670	GO:0015075	<1e-4	slr1530	GO:0005488	0.1355
ssr2787	GO:0065007	<1e-4	slr1290	GO:0031640	<1e-4	slr0869	GO:0043648	<1e-4	slr0948	GO:0016818	<1e-4	sll0756	GO:0005488	0.1355
sll8035	GO:0006793	<1e-4	slr0655	GO:0080090	<1e-4	sll0572	GO:0044283	<1e-4	sll0406	GO:0015405	<1e-4	slr0404	GO:0005488	0.1355
sll1267	GO:0034654	<1e-4	slr1378	GO:0042455	<1e-4	slr7013	GO:0044282	<1e-4	sll7067	GO:0022890	<1e-4	sll1086	GO:0005488	0.1355
slr1799	GO:0043170	<1e-4	sll1464	GO:0018130	<1e-4	ssr0536	GO:0052188	<1e-4	ssl5129	GO:0022832	<1e-4	slr1275	GO:0005488	0.1355
slr1827	GO:0046164	<1e-4	sll0793	GO:0009893	<1e-4	slr1363	GO:0009259	<1e-4	slr0869	GO:0016818	<1e-4	ssl0352	GO:0005488	0.1355
slr0505	GO:0006811	<1e-4	sll0688	GO:0051818	<1e-4	sll0442	GO:0009893	<1e-4	sll0839	GO:0016818	<1e-4	slr0941	GO:0005488	0.1355
sll0147	GO:0019222	<1e-4	slr1087	GO:0044106	<1e-4	slr1116	GO:0051186	<1e-4	slr0243	GO:0042623	<1e-4	slr0360	GO:0005488	0.1355
sll1433	GO:0009259	<1e-4	ssl2996	GO:0009892	<1e-4	ssr2803	GO:0009150	<1e-4	ssl0750	GO:0015405	<1e-4	sll1656	GO:0005488	0.1355
sll1350	GO:0043648	<1e-4	slr0975	GO:0042455	<1e-4	sll1040	GO:0006631	<1e-4	slr1470	GO:0003674	<1e-4	ssr3571	GO:0005488	0.1355
sll0284	GO:0022411	<1e-4	sll1219	GO:0071554	<1e-4	sll1372	GO:0080090	<1e-4	sll1132	GO:0022838	<1e-4	slr1932	GO:0005488	0.1355
slr6080	GO:0019752	<1e-4	slr0645	GO:0071554	<1e-4	ssl2162	GO:0065007	<1e-4	sll1388	GO:0042623	<1e-4	ssl0242	GO:0005488	0.1355
slr0208	GO:0006732	<1e-4	slr0337	GO:0001932	<1e-4	slr0053	GO:0043549	<1e-4	slr6072	GO:0043167	<1e-4	slr1964	GO:0005488	0.1355
sll5130	GO:0008150	<1e-4	sll1359	GO:0034654	<1e-4	ssl2814	GO:0048522	<1e-4	slr0890	GO:0043492	<1e-4	slr0294	GO:0005488	0.1355
slr0670	GO:0072528	<1e-4	sll0381	GO:0006082	<1e-4	sll1652	GO:0009987	<1e-4	slr1809	GO:0016746	<1e-4	sll0007	GO:0005488	0.1355
slr1847	GO:0009165	<1e-4	sll5089	GO:0006163	<1e-4	ssl7021	GO:0055114	<1e-4	slr0318	GO:0015293	<1e-4	sll1830	GO:0005488	0.1355
slr0263	GO:0043549	<1e-4	slr1547	GO:0051186	<1e-4	sll1858	GO:0009123	<1e-4	slr1173	GO:0022832	<1e-4	ssl2065	GO:0005488	0.1355
ssl3692	GO:0009263	<1e-4	sll0765	GO:0022607	<1e-4	slr0765	GO:0009059	<1e-4	sll1389	GO:0005342	<1e-4	slr1084	GO:0005488	0.1355
ssl1300	GO:0006721	<1e-4	ssl0461	GO:0009987	<1e-4	sll0225	GO:0044281	<1e-4	sll0481	GO:0043492	<1e-4	slr1177	GO:0005488	0.1355
sll1273	GO:0009309	<1e-4	sll0740	GO:0006066	<1e-4	sll1680	GO:0009309	<1e-4	sll0837	GO:0016746	<1e-4	sll0177	GO:0005488	0.1355
ssl0788	GO:0031323	<1e-4	ssr6026	GO:0009394	<1e-4	sll0864	GO:0072524	<1e-4	slr1209	GO:0015405	<1e-4	slr2122	GO:0005488	0.1355
sll0372	GO:0044271	<1e-4	slr1425	GO:0045184	<1e-4	sll0742	GO:0031326	<1e-4	slr1573	GO:0043167	<1e-4	slr1880	GO:0005488	0.1355
slr5111	GO:0019637	<1e-4	sll0584	GO:0046164	<1e-4	sll5063	GO:0051818	<1e-4	sll0872	GO:0016818	<1e-4	sll1658	GO:0005488	0.1355
sll1092	GO:0009142	<1e-4	slr2000	GO:0006576	<1e-4	slr6022	GO:0009889	<1e-4	ssr2843	GO:0022891	<1e-4	sll1002	GO:0005488	0.1355
sll1340	GO:0006793	<1e-4	slr1591	GO:0009150	<1e-4	sll0609	GO:0090304	<1e-4	ssl7021	GO:0022838	<1e-4	sll1630	GO:0005488	0.1355
sll0639	GO:0006733	<1e-4	sll0669	GO:0016054	<1e-4	sll7087	GO:0046907	<1e-4	slr0345	GO:0022857	<1e-4	slr0723	GO:0005488	0.1355
sll0761	GO:0009893	<1e-4	sll0925	GO:0051716	<1e-4	slr1568	GO:0044271	<1e-4	sll0176	GO:0043492	<1e-4	sll0085	GO:0005488	0.1355
slr1668	GO:0006811	<1e-4	sll7047	GO:0019219	<1e-4	slr1413	GO:0001932	<1e-4	slr1880	GO:0016462	<1e-4	ssr2787	GO:0005488	0.1355
sll0238	GO:0006163	<1e-4	ssr2802	GO:0019219	<1e-4	sll0293	GO:0006066	<1e-4	slr1907	GO:0022892	<1e-4	slr0872	GO:0005488	0.1355

sll1530	GO:0009059	<1e-4	sll1004	GO:0031326	<1e-4	slr7037	GO:0009991	<1e-4	slr7098	GO:0043492	<1e-4	slr1854	GO:0005488	0.1355
sll1925	GO:0009161	<1e-4	sll0572	GO:0051186	<1e-4	sll0496	GO:0065007	<1e-4	slr1152	GO:0022892	<1e-4	sll1613	GO:0005488	0.1355
slr0545	GO:0008150	<1e-4	sll8027	GO:0051179	<1e-4	slr0142	GO:0044248	<1e-4	slr1068	GO:0022832	<1e-4	slr1999	GO:0005488	0.1355
slr1593	GO:0008150	<1e-4	slr1815	GO:0044106	<1e-4	slr0712	GO:0019751	<1e-4	slr1577	GO:0022892	<1e-4	slr1210	GO:0005488	0.1355
ssl7021	GO:0010556	<1e-4	slr7083	GO:0055082	<1e-4	sll8019	GO:0009059	<1e-4	slr1568	GO:0016746	<1e-4	sll1736	GO:0005488	0.1355
slr1437	GO:0009161	<1e-4	sll1241	GO:0006220	<1e-4	slr1721	GO:0071554	<1e-4	slr5013	GO:0022891	<1e-4	sll1632	GO:0005215	0.1352
slr0144	GO:0005996	<1e-4	sll1510	GO:0071554	<1e-4	sll0732	GO:0006733	<1e-4	slr6106	GO:0008171	<1e-4	slr0348	GO:0005215	0.1352
slr0770	GO:0034654	<1e-4	slr1563	GO:0009987	<1e-4	slr6094	GO:0044275	<1e-4	slr2005	GO:0016818	<1e-4	sll0676	GO:0005215	0.1352
slr1240	GO:0009117	<1e-4	slr0049	GO:0006766	<1e-4	sll0319	GO:0046128	<1e-4	slr6075	GO:0015399	<1e-4	slr0941	GO:0005215	0.1352
slr6039	GO:0044271	<1e-4	ssl7046	GO:0046394	<1e-4	ssl5100	GO:0052188	<1e-4	slr1636	GO:0022803	<1e-4	sll1761	GO:0005215	0.1352
sll1939	GO:0044260	<1e-4	slr0607	GO:0031326	<1e-4	ssl1417	GO:0051246	<1e-4	sll0499	GO:0015291	<1e-4	sll0524	GO:0005215	0.1352
slr5017	GO:0006766	<1e-4	sll1049	GO:0050789	<1e-4	slr1162	GO:0031326	<1e-4	slr1702	GO:0022891	<1e-4	slr0404	GO:0005215	0.1352
slr1827	GO:0006766	<1e-4	slr6008	GO:0051186	<1e-4	slr1690	GO:0044281	<1e-4	sll1717	GO:0022891	<1e-4	slr1230	GO:0005215	0.1352
ssr7017	GO:0006721	<1e-4	sll1241	GO:0009132	<1e-4	sll1880	GO:0044255	<1e-4	slr0416	GO:0003674	<1e-4	slr0294	GO:0005215	0.1352
sll8040	GO:0043170	<1e-4	slr2120	GO:0010556	<1e-4	sll1541	GO:0006082	<1e-4	sll1381	GO:0042623	<1e-4	ssl2065	GO:0005215	0.1352
ssr2317	GO:0009987	<1e-4	sll1562	GO:0044281	<1e-4	sll1764	GO:0044283	<1e-4	sll0669	GO:0003674	<1e-4	slr0204	GO:0005215	0.1352
slr1062	GO:0009117	<1e-4	slr1396	GO:0044271	<1e-4	slr0489	GO:0006066	<1e-4	slr1920	GO:0022892	<1e-4	slr1913	GO:0005215	0.1352
sll0780	GO:0045184	<1e-4	slr1811	GO:0009308	<1e-4	slr7015	GO:0052111	<1e-4	ssl5007	GO:0022832	<1e-4	sll0414	GO:0005215	0.1352
slr1436	GO:0009987	<1e-4	slr1568	GO:0051234	<1e-4	slr0642	GO:0046164	<1e-4	sll0696	GO:0060089	<1e-4	slr2011	GO:0005215	0.1352
ssl1792	GO:0019219	<1e-4	sll1658	GO:0072528	<1e-4	sll1562	GO:0019362	<1e-4	slr1222	GO:0022836	<1e-4	sll1658	GO:0005215	0.1352
slr0304	GO:0009394	<1e-4	slr7091	GO:0043412	<1e-4	sll1381	GO:0048523	<1e-4	slr5127	GO:0022838	<1e-4	sll1061	GO:0005215	0.1352
sll0524	GO:0050801	<1e-4	sll0860	GO:0050794	<1e-4	slr1704	GO:0090304	<1e-4	sll1949	GO:0022838	<1e-4	slr2144	GO:0005215	0.1352
sll8033	GO:0048522	<1e-4	ssr1473	GO:0051171	<1e-4	slr6006	GO:0034641	<1e-4	sll1769	GO:0016818	<1e-4	ssl0242	GO:0005215	0.1352
ssl2971	GO:0043549	<1e-4	sll1390	GO:0071842	<1e-4	slr0108	GO:0072528	<1e-4	sll0860	GO:0022836	<1e-4	sll0085	GO:0005215	0.1352
sll0854	GO:0071842	<1e-4	sll1797	GO:0022411	<1e-4	sll0297	GO:0072524	<1e-4	slr0348	GO:0022857	<1e-4	slr1667	GO:0005215	0.1352
slr0765	GO:0043170	<1e-4	ssr0657	GO:0006066	<1e-4	slr1566	GO:0033013	<1e-4	slr0607	GO:0060089	<1e-4	slr0586	GO:0005215	0.1352
slr2038	GO:0009394	<1e-4	sll1472	GO:0034641	<1e-4	sll0615	GO:0050794	<1e-4	sll0857	GO:0016817	<1e-4	slr2121	GO:0005215	0.1352
sll0325	GO:0071496	<1e-4	sll0563	GO:0044283	<1e-4	sll0445	GO:0019222	<1e-4	slr1944	GO:0042623	<1e-4	sll0237	GO:0005215	0.1352
sll0413	GO:0019362	<1e-4	sll0413	GO:0044281	<1e-4	slr2012	GO:0009056	<1e-4	ssr6099	GO:0016818	<1e-4	slr1638	GO:0005215	0.1352
ssl3382	GO:0008150	<1e-4	sll0243	GO:0044255	<1e-4	slr1183	GO:0051188	<1e-4	sll1601	GO:0008171	<1e-4	sll0243	GO:0005215	0.1352
slr1170	GO:0043170	<1e-4	slr1101	GO:0006733	<1e-4	sll0802	GO:0009892	<1e-4	sll0761	GO:0015291	<1e-4	slr1436	GO:0005215	0.1352
sll5034	GO:0008610	<1e-4	sll0062	GO:0055114	<1e-4	slr0967	GO:0006811	<1e-4	slr1074	GO:0022803	<1e-4	slr1999	GO:0005215	0.1352
sll0863	GO:0010468	<1e-4	slr0907	GO:0044255	<1e-4	sll1318	GO:0071840	<1e-4	sll1433	GO:0022857	<1e-4	slr1530	GO:0005215	0.1352
slr1576	GO:0009150	<1e-4	slr1737	GO:0006793	<1e-4	slr1563	GO:0071840	<1e-4	sll0853	GO:0015293	<1e-4	sll1530	GO:0005215	0.1352
sll0328	GO:0043170	<1e-4	sll0564	GO:0065007	<1e-4	slr2038	GO:0019752	<1e-4	slr0935	GO:0015075	<1e-4	sll0068	GO:0000166	0.1345
ssr8013	GO:0043170	<1e-4	ssl7022	GO:0009141	<1e-4	slr1441	GO:0009124	<1e-4	slr0416	GO:0016462	<1e-4	ssl1255	GO:0005488	0.1337
sll0071	GO:0006163	<1e-4	sll0749	GO:0055082	<1e-4	slr7095	GO:0009987	<1e-4	sll0980	GO:0022892	<1e-4	slr1385	GO:0005488	0.1337
sll1022	GO:0071554	<1e-4	slr0294	GO:0046394	<1e-4	slr0303	GO:0051347	<1e-4	sll1036	GO:0008171	<1e-4	slr0491	GO:0005488	0.1337
slr6072	GO:0006220	<1e-4	slr1222	GO:0006811	<1e-4	sll0756	GO:0019752	<1e-4	sll0735	GO:0022838	<1e-4	ssr2843	GO:0005488	0.1337
sll0539	GO:0019222	<1e-4	slr1600	GO:0009161	<1e-4	sll7030	GO:0009144	<1e-4	sll1002	GO:0022892	<1e-4	sll0788	GO:0005488	0.1325
ssr6085	GO:0044282	<1e-4	ssl0242	GO:0006733	<1e-4	ssl1300	GO:0009123	<1e-4	slr0039	GO:0015293	<1e-4	slr0514	GO:0005488	0.1325
sll7087	GO:0044283	<1e-4	slr1799	GO:0022411	<1e-4	slr1800	GO:0019751	<1e-4	slr0232	GO:0043492	<1e-4	sll0175	GO:0005488	0.1325
ssl1255	GO:0043549	<1e-4	slr1648	GO:0071841	<1e-4	sll1510	GO:0043412	<1e-4	sll1938	GO:0015291	<1e-4	sll0192	GO:0005488	0.1325
sll1163	GO:0044271	<1e-4	slr1287	GO:0006753	<1e-4	sll1006	GO:0048518	<1e-4	slr1033	GO:0043167	<1e-4	sll0022	GO:0005488	0.1325
slr0398	GO:0051347	<1e-4	sll7069	GO:0044271	<1e-4	slr1103	GO:0009059	<1e-4	slr0023	GO:0022890	<1e-4	slr1726	GO:0005488	0.1325
sll0995	GO:0019362	<1e-4	sll1906	GO:0044275	<1e-4	sll1401	GO:0051186	<1e-4	sll0558	GO:0016746	<1e-4	slr1047	GO:0005488	0.1325
slr0509	GO:0009165	<1e-4	slr0337	GO:0006732	<1e-4	sll8002	GO:0009165	<1e-4	sll1763	GO:0022838	<1e-4	ssl1377	GO:0005488	0.1325
sll0614	GO:0009987	<1e-4	slr0730	GO:0072522	<1e-4	sll1340	GO:0009144	<1e-4	sll1531	GO:0016462	<1e-4	sll1562	GO:0005488	0.1325
sll1002	GO:0046128	<1e-4	slr1533	GO:0022607	<1e-4	slr1590	GO:0072522	<1e-4	sll1036	GO:0022892	<1e-4	slr1767	GO:0005488	0.1325
ssr3402	GO:0009260	<1e-4	sll0405	GO:0019752	<1e-4	sll0736	GO:0018130	<1e-4	ssr6062	GO:0043167	<1e-4	sll0558	GO:0005488	0.1325
sll0590	GO:0044283	<1e-4	slr1023	GO:0016052	<1e-4	slr0217	GO:0019751	<1e-4	slr0725	GO:0016741	<1e-4	sll0505	GO:0005488	0.1325
sll5097	GO:0051188	<1e-4	slr1306	GO:0016052	<1e-4	slr1644	GO:0042451	<1e-4	ssl5045	GO:0016741	<1e-4	sll0932	GO:0005488	0.1309
slr1999	GO:0008150	<1e-4	ssr5020	GO:0006766	<1e-4	sll0827	GO:0055082	<1e-4	slr0058	GO:0022803	<1e-4	ssr2781	GO:0005488	0.1309
slr7092	GO:0051186	<1e-4	sll1714	GO:0034660	<1e-4	ssr6024	GO:0046164	<1e-4	slr0728	GO:0022892	<1e-4	ssr1256	GO:0005488	0.1309

ssl3573	GO:0016052	<1e-4	sll2011	GO:0019752	<1e-4	sll1775	GO:0034641	<1e-4	slr1449	GO:0015267	<1e-4	sll0272	GO:0005488	0.1309
slr1546	GO:0009124	<1e-4	slr0142	GO:0006721	<1e-4	sll1752	GO:0009394	<1e-4	slr0914	GO:0016462	<1e-4	ssr2803	GO:0005488	0.1309
sll0584	GO:0019362	<1e-4	slr1790	GO:0072522	<1e-4	sll7033	GO:0009150	<1e-4	slr0967	GO:0022803	<1e-4	sll1119	GO:0005488	0.1309
sll0048	GO:0009150	<1e-4	slr1618	GO:0034660	<1e-4	sll0763	GO:0043170	<1e-4	sll0588	GO:0016462	<1e-4	sll1250	GO:0005488	0.1309
slr5087	GO:0042180	<1e-4	slr1547	GO:0071496	<1e-4	slr5018	GO:0009123	<1e-4	sll7031	GO:0003674	<1e-4	sll0238	GO:0005488	0.1309
slr0967	GO:0009123	<1e-4	ssl5103	GO:0060255	<1e-4	ssr0692	GO:0009142	<1e-4	slr1047	GO:0005342	<1e-4	sll0606	GO:0005488	0.1309
slr0416	GO:0044106	<1e-4	sll0372	GO:0009199	<1e-4	ssr1288	GO:0046483	<1e-4	sll0436	GO:0022832	<1e-4	sll0647	GO:0005488	0.1309
slr0192	GO:0034654	<1e-4	sll1024	GO:0006753	<1e-4	slr5023	GO:0010468	<1e-4	sll1164	GO:0042623	<1e-4	slr1519	GO:0005488	0.13
ssl5098	GO:0009991	<1e-4	slr6009	GO:0019222	<1e-4	slr1114	GO:0006576	<1e-4	sll0008	GO:0016818	<1e-4	slr1398	GO:0005488	0.13
sll7069	GO:0019752	<1e-4	sll0609	GO:0010468	<1e-4	slr6012	GO:0046164	<1e-4	sll0098	GO:0022891	<1e-4	slr0852	GO:0005488	0.13
slr1084	GO:0031326	<1e-4	slr1513	GO:0051347	<1e-4	slr1546	GO:0051716	<1e-4	slr0971	GO:0003674	<1e-4	sll0996	GO:0005488	0.13
ssl0350	GO:0046394	<1e-4	sll1926	GO:0044260	<1e-4	slr6039	GO:0051716	<1e-4	slr0590	GO:0015077	<1e-4	sll1218	GO:0005488	0.13
sll0788	GO:0019362	<1e-4	sll0327	GO:0006766	<1e-4	slr0262	GO:0009142	<1e-4	sll1307	GO:0022892	<1e-4	sll0354	GO:0005488	0.13
slr8021	GO:0008610	<1e-4	slr1628	GO:0031323	<1e-4	slr7080	GO:0045184	<1e-4	sll0577	GO:0022890	<1e-4	slr1376	GO:0005488	0.13
slr1407	GO:0009987	<1e-4	ssl5098	GO:0044260	<1e-4	slr0337	GO:0071841	<1e-4	sll1979	GO:0016818	<1e-4	slr1533	GO:0005488	0.13
ssl5096	GO:0009144	<1e-4	slr1535	GO:0072522	<1e-4	sll1378	GO:0052188	<1e-4	sll1461	GO:0016817	<1e-4	slr0376	GO:0005488	0.13
slr0815	GO:0044003	<1e-4	sll0985	GO:0006766	<1e-4	sll0413	GO:0006631	<1e-4	sll0783	GO:0060089	<1e-4	ssr3532	GO:0005488	0.13
sll0266	GO:0019752	<1e-4	slr1935	GO:0006576	<1e-4	slr0813	GO:0042451	<1e-4	slr1052	GO:0016741	<1e-4	sll1542	GO:0005488	0.13
sll0496	GO:0071496	<1e-4	ssr5121	GO:0080090	<1e-4	slr0589	GO:0006576	<1e-4	slr0510	GO:0043492	<1e-4	slr1441	GO:0005488	0.13
sll0761	GO:0043549	<1e-4	slr0241	GO:0019637	<1e-4	sll5033	GO:0009161	<1e-4	ssl5065	GO:0043492	<1e-4	sll1601	GO:0005488	0.13
slr0104	GO:0019222	<1e-4	sll1247	GO:0042180	<1e-4	slr2111	GO:0008150	<1e-4	slr0771	GO:0015077	<1e-4	slr0209	GO:0005488	0.13
sll1359	GO:0019637	<1e-4	sll0198	GO:0019752	<1e-4	sll1531	GO:0072522	<1e-4	ssr3402	GO:0022836	<1e-4	sll1318	GO:0005488	0.13
sll0293	GO:0009260	<1e-4	slr0731	GO:0010556	<1e-4	slr0060	GO:0022411	<1e-4	sll1049	GO:0022891	<1e-4	slr0876	GO:0005488	0.13
slr7073	GO:0019751	<1e-4	sll0008	GO:0033013	<1e-4	sll5130	GO:0072522	<1e-4	sll1401	GO:0022836	<1e-4	ssr0536	GO:0005488	0.13
slr6022	GO:0022607	<1e-4	sll0447	GO:0022607	<1e-4	sll0997	GO:0065007	<1e-4	ssl7007	GO:0008171	<1e-4	sll0424	GO:0005488	0.13
slr0398	GO:0009991	<1e-4	slr1104	GO:0019222	<1e-4	sll0372	GO:0006811	<1e-4	slr1927	GO:0060089	<1e-4	slr1917	GO:0005488	0.13
slr0364	GO:0009142	<1e-4	slr1186	GO:0046394	<1e-4	ssr2047	GO:0009144	<1e-4	sll1486	GO:0015293	<1e-4	ssr1375	GO:0005488	0.13
sll0412	GO:0048522	<1e-4	sll0931	GO:0006793	<1e-4	sll0208	GO:0044282	<1e-4	slr2084	GO:0043167	<1e-4	slr0305	GO:0005488	0.13
sll0553	GO:0071496	<1e-4	slr0060	GO:0009132	<1e-4	sll0160	GO:0006720	<1e-4	sll1757	GO:0042623	<1e-4	slr1799	GO:0005488	0.13
slr1397	GO:0043933	<1e-4	slr6005	GO:0006720	<1e-4	sll0047	GO:0042180	<1e-4	slr1470	GO:0015399	<1e-4	slr0076	GO:0005488	0.13
slr0243	GO:0043412	<1e-4	sll0008	GO:0006766	<1e-4	sll0281	GO:0019751	<1e-4	sll0101	GO:0016817	<1e-4	sll0360	GO:0005488	0.13
ssl3382	GO:0009123	<1e-4	slr1474	GO:0009889	<1e-4	sll8033	GO:0042180	<1e-4	ssl2064	GO:0043492	<1e-4	slr0304	GO:0005488	0.13
sll1542	GO:0052111	<1e-4	slr0416	GO:0005996	<1e-4	sll1950	GO:0071554	<1e-4	slr2038	GO:0005342	<1e-4	sll1715	GO:0005488	0.13
slr1599	GO:0006091	<1e-4	slr1866	GO:0050789	<1e-4	sll1527	GO:0019752	<1e-4	slr6067	GO:0015077	<1e-4	sll1527	GO:0005488	0.1294
slr1809	GO:0090304	<1e-4	slr2073	GO:0009142	<1e-4	slr1880	GO:0080090	<1e-4	ssr6048	GO:0016746	<1e-4	sll1293	GO:0005488	0.1294
ssr1765	GO:0009150	<1e-4	slr1398	GO:0010556	<1e-4	slr2038	GO:0044106	<1e-4	ssl6061	GO:0003674	<1e-4	slr0605	GO:0017111	0.1261
sll0488	GO:0050896	<1e-4	ssr2711	GO:0009117	<1e-4	slr1122	GO:0051179	<1e-4	sll1938	GO:0022892	<1e-4	sll1240	GO:0017111	0.1261
sll0788	GO:0009259	<1e-4	sll1570	GO:0034654	<1e-4	slr0519	GO:0016054	<1e-4	sll0405	GO:0015075	<1e-4	sll0740	GO:0017111	0.1261
ssr2047	GO:0034641	<1e-4	slr0148	GO:0051716	<1e-4	sll1352	GO:0043933	<1e-4	ssl2069	GO:0015399	<1e-4	slr2005	GO:0017111	0.1261
slr1209	GO:0009141	<1e-4	slr1301	GO:0045184	<1e-4	sll0328	GO:0006163	<1e-4	sll0423	GO:0016817	<1e-4	sll0909	GO:0017111	0.1261
slr6022	GO:0009117	<1e-4	slr0476	GO:0065007	<1e-4	slr0609	GO:0051716	<1e-4	ssl5031	GO:0008171	<1e-4	sll1541	GO:0017111	0.1261
slr1069	GO:0065007	<1e-4	ssr1375	GO:0022607	<1e-4	sll1321	GO:0031640	<1e-4	slr1951	GO:0005342	<1e-4	ssr2009	GO:0017111	0.1261
sll1912	GO:0019362	<1e-4	ssl5008	GO:0009263	<1e-4	sll1562	GO:0006720	<1e-4	sll0354	GO:0022892	<1e-4	sll1267	GO:0017111	0.1261
slr1084	GO:0043549	<1e-4	ssl0750	GO:0044255	<1e-4	slr0243	GO:0043436	<1e-4	slr0613	GO:0015291	<1e-4	slr1493	GO:0017111	0.1261
slr0431	GO:0046394	<1e-4	slr2012	GO:0043412	<1e-4	sll1426	GO:0042451	<1e-4	slr1546	GO:0015405	<1e-4	sll0787	GO:0017111	0.1261
ssl2781	GO:0045184	<1e-4	ssl5031	GO:0043436	<1e-4	sll5062	GO:0044281	<1e-4	ssr2009	GO:0015405	<1e-4	slr1809	GO:0017111	0.1261
sll0147	GO:0051188	<1e-4	sll7086	GO:0009892	<1e-4	sll1571	GO:0046164	<1e-4	slr1603	GO:0015291	<1e-4	sll1399	GO:0017111	0.1261
sll1902	GO:0043412	<1e-4	sll0615	GO:0006066	<1e-4	slr1699	GO:0019637	<1e-4	slr0887	GO:0016741	<1e-4	sll0188	GO:0017111	0.1261
slr0271	GO:0046164	<1e-4	sll1681	GO:0006091	<1e-4	slr0913	GO:0071496	<1e-4	sll0871	GO:0016741	<1e-4	slr0358	GO:0017111	0.1261
ssr3402	GO:0080090	<1e-4	sll0997	GO:0006163	<1e-4	sll0804	GO:0005996	<1e-4	slr1619	GO:0043167	<1e-4	slr0924	GO:0017111	0.1261
sll1702	GO:0009059	<1e-4	sll1350	GO:0006082	<1e-4	sll0428	GO:0060255	<1e-4	sll0157	GO:0016746	<1e-4	sll1938	GO:0017111	0.1261
ssr6003	GO:0048523	<1e-4	slr0971	GO:0009263	<1e-4	sll1921	GO:0009123	<1e-4	slr0863	GO:0022891	<1e-4	sll1390	GO:0017111	0.1261
slr0957	GO:0009124	<1e-4	slr1944	GO:0044271	<1e-4	sll2011	GO:0034660	<1e-4	slr1032	GO:0015399	<1e-4	sll0297	GO:0017111	0.1261
sll1751	GO:0051347	<1e-4	slr7037	GO:0043170	<1e-4	slr1391	GO:0051171	<1e-4	sll0253	GO:0060089	<1e-4	sll0837	GO:0017111	0.1261

sll0449	GO:0046128	<1e-4	slr1628	GO:0050789	<1e-4	slr6063	GO:0043436	<1e-4	slr5116	GO:0015075	<1e-4	slr0935	GO:0017111	0.1261
slr1273	GO:0009259	<1e-4	sll0658	GO:0005996	<1e-4	sll1321	GO:0048522	<1e-4	slr1170	GO:0015405	<1e-4	sll0737	GO:0017111	0.1261
slr0913	GO:0006066	<1e-4	slr1623	GO:0009262	<1e-4	ssr3122	GO:0051171	<1e-4	sll5089	GO:0015291	<1e-4	sll0590	GO:0017111	0.1261
sll1954	GO:0046164	<1e-4	sll0802	GO:0019751	<1e-4	sll0497	GO:0009259	<1e-4	slr1122	GO:0022891	<1e-4	slr0815	GO:0017111	0.1261
slr0345	GO:0044283	<1e-4	slr1169	GO:0019219	<1e-4	sll0488	GO:0009126	<1e-4	slr0586	GO:0022838	<1e-4	slr1258	GO:0017111	0.1261
slr5116	GO:0044255	<1e-4	slr1895	GO:0048523	<1e-4	slr1070	GO:0009059	<1e-4	slr7097	GO:0015291	<1e-4	sll1659	GO:0017111	0.1261
slr5016	GO:0009142	<1e-4	sll7034	GO:0006753	<1e-4	slr1762	GO:0006220	<1e-4	slr1851	GO:0015291	<1e-4	sll1201	GO:0017111	0.1261
sll0243	GO:0009161	<1e-4	sll0156	GO:0051171	<1e-4	slr0588	GO:0006811	<1e-4	sll0192	GO:0022891	<1e-4	slr0053	GO:0017111	0.1261
slr1074	GO:0051186	<1e-4	ssr0536	GO:0006811	<1e-4	ssr5120	GO:0006066	<1e-4	sll0793	GO:0005342	<1e-4	ssl0739	GO:0017111	0.1261
sll0737	GO:0048519	<1e-4	slr0082	GO:0071554	<1e-4	slr0404	GO:0044248	<1e-4	sll0985	GO:0022890	<1e-4	sll1512	GO:0017111	0.1261
slr1495	GO:0006091	<1e-4	sll1675	GO:0072522	<1e-4	sll1640	GO:0031326	<1e-4	sll1092	GO:0022891	<1e-4	sll0911	GO:0017111	0.1261
slr0145	GO:0031326	<1e-4	slr1362	GO:0009308	<1e-4	sll0933	GO:0009117	<1e-4	sll0443	GO:0016746	<1e-4	sll1350	GO:0017111	0.1261
slr1935	GO:0072521	<1e-4	sll7031	GO:0009987	<1e-4	slr1959	GO:0006721	<1e-4	sll7090	GO:0003674	<1e-4	slr0172	GO:0017111	0.1261
sll0543	GO:0034641	<1e-4	slr1087	GO:0009150	<1e-4	sll1562	GO:0043412	<1e-4	sll8019	GO:0022891	<1e-4	sll1344	GO:0017111	0.1261
ssr2781	GO:0052111	<1e-4	ssl1255	GO:0009892	<1e-4	slr1168	GO:0009150	<1e-4	sgl0001	GO:0043167	<1e-4	sll1071	GO:0017111	0.1261
sll0310	GO:0009141	<1e-4	slr0959	GO:0010556	<1e-4	slr2105	GO:0042180	<1e-4	slr2060	GO:0022832	<1e-4	slr2125	GO:0017111	0.1261
sll6054	GO:0043436	<1e-4	slr1068	GO:0043170	<1e-4	sll7028	GO:0055086	<1e-4	sll0022	GO:0015077	<1e-4	sll0688	GO:0017111	0.1261
sll0623	GO:0009309	<1e-4	sll1763	GO:0046394	<1e-4	slr0356	GO:0046907	<1e-4	sll1950	GO:0043167	<1e-4	sll0449	GO:0017111	0.1261
slr0151	GO:0008150	<1e-4	slr1854	GO:0050801	<1e-4	sll1965	GO:0065007	<1e-4	sll0872	GO:0022836	<1e-4	slr0702	GO:0017111	0.1261
ssl7074	GO:0048518	<1e-4	slr2005	GO:0055082	<1e-4	slr0456	GO:0072521	<1e-4	slr1534	GO:0022891	<1e-4	slr0149	GO:0017111	0.1261
sll1921	GO:0051246	<1e-4	slr1236	GO:0009987	<1e-4	sll0872	GO:0043648	<1e-4	ssr2787	GO:0022832	<1e-4	sll1660	GO:0017111	0.1261
slr2025	GO:0051179	<1e-4	slr0149	GO:0009057	<1e-4	slr5126	GO:0080090	<1e-4	slr0699	GO:0015291	<1e-4	slr1259	GO:0017111	0.1261
sll0756	GO:0051188	<1e-4	ssr2047	GO:0008150	<1e-4	sll1192	GO:0046907	<1e-4	sll1123	GO:0022857	<1e-4	ssr2754	GO:0017111	0.1261
slr0581	GO:0052188	<1e-4	slr1977	GO:0042180	<1e-4	slr1168	GO:0051171	<1e-4	sll1542	GO:0022836	<1e-4	sll1352	GO:0017111	0.1261
slr0211	GO:0065007	<1e-4	sll1939	GO:0009263	<1e-4	slr1419	GO:0009987	<1e-4	slr0376	GO:0043167	<1e-4	slr1288	GO:0017111	0.1261
sll1956	GO:0044281	<1e-4	ssr7093	GO:0031323	<1e-4	sll0625	GO:0019637	<1e-4	slr0642	GO:0016462	<1e-4	ssl3382	GO:0017111	0.1261
slr0148	GO:0044260	<1e-4	sll0301	GO:0006811	<1e-4	sll0645	GO:0031640	<1e-4	sll7034	GO:0008171	<1e-4	slr1287	GO:0017111	0.1261
sll0225	GO:0042455	<1e-4	slr1053	GO:0071842	<1e-4	sll0532	GO:0043170	<1e-4	slr0423	GO:0015267	<1e-4	sll1411	GO:0017111	0.1261
sll1372	GO:0044275	<1e-4	sll1006	GO:0034660	<1e-4	slr5016	GO:0009117	<1e-4	sll0827	GO:0015405	<1e-4	sll1956	GO:0017111	0.1261
sll1884	GO:0080090	<1e-4	slr5126	GO:0044248	<1e-4	sll0066	GO:0008610	<1e-4	slr1519	GO:0022832	<1e-4	sll1021	GO:0017111	0.1261
sll1132	GO:0022607	<1e-4	sll0189	GO:0006220	<1e-4	slr1767	GO:0006163	<1e-4	ssl1520	GO:0008171	<1e-4	slr1074	GO:0017111	0.1261
sll0147	GO:0009199	<1e-4	slr0468	GO:0044260	<1e-4	slr0103	GO:0009165	<1e-4	sll1660	GO:0015291	<1e-4	slr0981	GO:0017111	0.1261
sll7050	GO:0051246	<1e-4	sll0802	GO:0072521	<1e-4	sll1761	GO:0044106	<1e-4	ssl0483	GO:0015077	<1e-4	slr1082	GO:0017111	0.1261
slr5073	GO:0071496	<1e-4	sll1381	GO:0034654	<1e-4	sll0007	GO:0051818	<1e-4	slr0813	GO:0016746	<1e-4	sll0141	GO:0017111	0.1261
sll0394	GO:0006793	<1e-4	slr1107	GO:0051347	<1e-4	sll0103	GO:0051171	<1e-4	sll1586	GO:0008171	<1e-4	slr0962	GO:0017111	0.1261
sll0350	GO:0034641	<1e-4	ssr6079	GO:0051716	<1e-4	slr0013	GO:0046907	<1e-4	slr1070	GO:0016462	<1e-4	slr0169	GO:0017111	0.1261
sll1006	GO:0042451	<1e-4	slr2101	GO:0090304	<1e-4	sll0448	GO:0048518	<1e-4	sll1715	GO:0015291	<1e-4	slr1128	GO:0017111	0.1261
ssr2754	GO:0044248	<1e-4	sll7087	GO:0022607	<1e-4	ssl7007	GO:0006721	<1e-4	slr0060	GO:0016462	<1e-4	sll1950	GO:0017111	0.1261
slr0364	GO:0050801	<1e-4	sll0909	GO:0051818	<1e-4	slr0431	GO:0051234	<1e-4	slr0145	GO:0015293	<1e-4	sll1225	GO:0017111	0.1261
slr0876	GO:0009893	<1e-4	sll1563	GO:0071842	<1e-4	ssr6089	GO:0009123	<1e-4	slr5116	GO:0022857	<1e-4	slr1033	GO:0017111	0.1261
sll0253	GO:0065007	<1e-4	slr0038	GO:0071841	<1e-4	slr1437	GO:0019222	<1e-4	ssl0350	GO:0016817	<1e-4	slr7024	GO:0017111	0.1261
sll1219	GO:0080090	<1e-4	slr0521	GO:0046483	<1e-4	sll1193	GO:0009057	<1e-4	sll0837	GO:0042623	<1e-4	slr0729	GO:0017111	0.1261
sll8019	GO:0006732	<1e-4	slr0784	GO:0051188	<1e-4	sll1304	GO:0042180	<1e-4	slr1927	GO:0042623	<1e-4	slr1070	GO:0017111	0.1261
sll0168	GO:0043933	<1e-4	sll6052	GO:0072528	<1e-4	slr6063	GO:0044271	<1e-4	slr1544	GO:0022857	<1e-4	sll0827	GO:0017111	0.1261
sll7067	GO:0006733	<1e-4	sll1201	GO:0045184	<1e-4	slr0667	GO:0044260	<1e-4	sll1455	GO:0003674	<1e-4	slr1940	GO:0017111	0.1261
slr0509	GO:0006066	<1e-4	slr2060	GO:0051188	<1e-4	slr0579	GO:0009987	<1e-4	sll0811	GO:0022832	<1e-4	slr0483	GO:0017111	0.1261
sll0614	GO:0006066	<1e-4	ssl3615	GO:0044283	<1e-4	slr1128	GO:0009132	<1e-4	ssr5120	GO:0043492	<1e-4	ssr1552	GO:0017111	0.1261
slr0887	GO:0019637	<1e-4	sgl0001	GO:0071840	<1e-4	sll1956	GO:0043412	<1e-4	slr0364	GO:0060089	<1e-4	ssl0483	GO:0017111	0.1261
ssr1766	GO:0009057	<1e-4	sll1119	GO:0010468	<1e-4	sll1763	GO:0006163	<1e-4	slr5127	GO:0015399	<1e-4	sll1054	GO:0017111	0.1261
sll1396	GO:0072528	<1e-4	slr0885	GO:0046483	<1e-4	sll8012	GO:0051186	<1e-4	slr2073	GO:0022836	<1e-4	sll0595	GO:0017111	0.1261
sll0871	GO:0050794	<1e-4	sll1526	GO:0080090	<1e-4	slr1493	GO:0019362	<1e-4	slr1507	GO:0016818	<1e-4	slr1762	GO:0017111	0.1261
slr2125	GO:0051234	<1e-4	slr5017	GO:0065007	<1e-4	sll0268	GO:0034660	<1e-4	slr0650	GO:0016462	<1e-4	sll1251	GO:0017111	0.1261
sll1738	GO:0043170	<1e-4	slr0619	GO:0019752	<1e-4	slr1572	GO:0001932	<1e-4	sll0552	GO:0022838	<1e-4	slr0104	GO:0017111	0.1261
slr0366	GO:0006732	<1e-4	sll0861	GO:0060255	<1e-4	sll1378	GO:0008150	<1e-4	ssl5045	GO:0015399	<1e-4	slr0111	GO:0017111	0.1261

sll5047	GO:0044282	<1e-4	slr1186	GO:0016054	<1e-4	slr1261	GO:0006082	<1e-4	slr5101	GO:0043492	<1e-4	slr0887	GO:0017111	0.1261
slr1230	GO:0022607	<1e-4	ssl3142	GO:0046128	<1e-4	slr1169	GO:0046483	<1e-4	sll0328	GO:0005342	<1e-4	sll0147	GO:0017111	0.1261
slr0479	GO:0022411	<1e-4	ssr1552	GO:0009144	<1e-4	slr0876	GO:0006220	<1e-4	sll5004	GO:0022857	<1e-4	sll0180	GO:0017111	0.1261
sll1934	GO:0051347	<1e-4	slr5037	GO:0048878	<1e-4	sll1380	GO:0072528	<1e-4	ssl2138	GO:0005342	<1e-4	slr2115	GO:0017111	0.1261
sll2011	GO:0009987	<1e-4	slr0613	GO:0009987	<1e-4	sll0775	GO:0009309	<1e-4	slr1541	GO:0016741	<1e-4	slr0957	GO:0017111	0.1261
slr0407	GO:0044275	<1e-4	sll0496	GO:0052188	<1e-4	slr0423	GO:0006220	<1e-4	sll0314	GO:0042623	<1e-4	slr1303	GO:0017111	0.1261
ssr6002	GO:0009150	<1e-4	slr6022	GO:0043648	<1e-4	sll1954	GO:0022607	<1e-4	sll0543	GO:0016741	<1e-4	sll1040	GO:0017111	0.1261
slr0967	GO:0080090	<1e-4	sll0783	GO:0080090	<1e-4	ssr7017	GO:0016052	<1e-4	sll1241	GO:0022891	<1e-4	slr0060	GO:0017111	0.1261
slr0108	GO:0071841	<1e-4	sll0749	GO:0009124	<1e-4	sll0068	GO:0044260	<1e-4	slr2018	GO:0043167	<1e-4	ssl8008	GO:0017111	0.1261
slr1774	GO:0048523	<1e-4	sll1158	GO:0046907	<1e-4	slr0207	GO:0072522	<1e-4	slr7060	GO:0016746	<1e-4	slr0596	GO:0017111	0.1261
slr2025	GO:0034654	<1e-4	slr6091	GO:0051171	<1e-4	sll0359	GO:0071841	<1e-4	slr1768	GO:0015267	<1e-4	slr1169	GO:0017111	0.1261
sll0355	GO:0055086	<1e-4	sll0242	GO:0009199	<1e-4	slr6021	GO:0072522	<1e-4	ssr2972	GO:0060089	<1e-4	slr1852	GO:0017111	0.1261
sll1304	GO:0006631	<1e-4	slr0489	GO:0016052	<1e-4	sll0647	GO:0072524	<1e-4	sll0939	GO:0015267	<1e-4	slr2048	GO:0017111	0.1261
sll1749	GO:0006631	<1e-4	slr1068	GO:0006720	<1e-4	ssl7039	GO:0043170	<1e-4	ssl3451	GO:0022890	<1e-4	slr0455	GO:0017111	0.1261
ssr3402	GO:0009117	<1e-4	slr6038	GO:0072527	<1e-4	sll1272	GO:0043648	<1e-4	ssl3451	GO:0015399	<1e-4	sll0225	GO:0017111	0.1261
ssl1972	GO:0006766	<1e-4	slr0610	GO:0044260	<1e-4	slr1956	GO:0006732	<1e-4	ssr2848	GO:0022857	<1e-4	sll0623	GO:0017111	0.1261
slr1619	GO:0009057	<1e-4	slr0404	GO:0006220	<1e-4	slr1944	GO:0009124	<1e-4	sll1940	GO:0016746	<1e-4	sll0564	GO:0017111	0.1261
sll0505	GO:0006721	<1e-4	slr0601	GO:0006721	<1e-4	ssr3409	GO:0033013	<1e-4	sll0647	GO:0016746	<1e-4	slr1862	GO:0017111	0.1261
ssr1552	GO:0006631	<1e-4	slr1307	GO:0034654	<1e-4	slr1773	GO:0006766	<1e-4	ssr3572	GO:0022803	<1e-4	slr1209	GO:0017111	0.1261
slr7058	GO:0009144	<1e-4	slr0712	GO:0006576	<1e-4	sll1380	GO:0051186	<1e-4	sll0524	GO:0005342	<1e-4	slr1895	GO:0017111	0.1261
slr1183	GO:0090304	<1e-4	slr0887	GO:0006720	<1e-4	slr1660	GO:0034660	<1e-4	slr2115	GO:0022857	<1e-4	sll0359	GO:0017111	0.1261
slr1618	GO:0048522	<1e-4	ssl2471	GO:0080090	<1e-4	slr1472	GO:0051171	<1e-4	sll5047	GO:0060089	<1e-4	slr0605	GO:0003676	0.1243
slr1342	GO:0016053	<1e-4	slr0204	GO:0090304	<1e-4	slr1168	GO:0044255	<1e-4	slr1442	GO:0015077	<1e-4	sll1938	GO:0003676	0.1243
slr0169	GO:0050794	<1e-4	sll1285	GO:0071842	<1e-4	sll0449	GO:0006720	<1e-4	ssl5025	GO:0016462	<1e-4	slr0650	GO:0003676	0.1243
sll1675	GO:0006793	<1e-4	slr0059	GO:0044260	<1e-4	slr1906	GO:0051188	<1e-4	slr1577	GO:0016746	<1e-4	slr1053	GO:0003676	0.1243
slr7096	GO:0044003	<1e-4	slr1616	GO:0042451	<1e-4	sll1203	GO:0050801	<1e-4	slr1062	GO:0022891	<1e-4	ssl0739	GO:0003676	0.1243
slr1566	GO:0009165	<1e-4	slr0232	GO:0006753	<1e-4	ssl3573	GO:0009309	<1e-4	slr1419	GO:0042623	<1e-4	sll0359	GO:0003676	0.1243
slr1753	GO:0043170	<1e-4	sll8035	GO:0051171	<1e-4	slr0147	GO:0072521	<1e-4	slr0196	GO:0022890	<1e-4	slr2125	GO:0003676	0.1243
slr1168	GO:0044275	<1e-4	sll1203	GO:0060255	<1e-4	sll5132	GO:0051818	<1e-4	slr0145	GO:0022857	<1e-4	slr1852	GO:0003676	0.1243
sll1696	GO:0009117	<1e-4	slr1074	GO:0022411	<1e-4	slr2049	GO:0050801	<1e-4	sll1659	GO:0022803	<1e-4	sll0141	GO:0003676	0.1243
sll0803	GO:0016052	<1e-4	slr5017	GO:0043549	<1e-4	sll1004	GO:0009259	<1e-4	slr0960	GO:0008171	<1e-4	sll0737	GO:0003676	0.1243
ssl3451	GO:0006753	<1e-4	sll0098	GO:0019362	<1e-4	ssl0788	GO:0071840	<1e-4	sll0762	GO:0016462	<1e-4	slr1541	GO:0003676	0.1243
sll0858	GO:0009260	<1e-4	slr1493	GO:0009987	<1e-4	sll2015	GO:0042180	<1e-4	sll7077	GO:0005342	<1e-4	slr0104	GO:0003676	0.1243
slr0271	GO:0009123	<1e-4	slr0806	GO:0080090	<1e-4	slr1638	GO:0046907	<1e-4	slr1676	GO:0022838	<1e-4	ssr1114	GO:0003676	0.1243
slr0299	GO:0044260	<1e-4	slr0505	GO:0009165	<1e-4	sll1638	GO:0050789	<1e-4	sll1060	GO:0043492	<1e-4	slr0169	GO:0003676	0.1243
slr0586	GO:0031326	<1e-4	ssr5020	GO:0006066	<1e-4	ssl0483	GO:0016052	<1e-4	sll5061	GO:0043492	<1e-4	sll1399	GO:0003676	0.1243
slr0810	GO:0009123	<1e-4	slr1790	GO:0006721	<1e-4	slr1674	GO:0006082	<1e-4	sll0449	GO:0016817	<1e-4	sll0995	GO:0003676	0.1243
ssr6062	GO:0048519	<1e-4	sll1834	GO:0065007	<1e-4	slr0913	GO:0009394	<1e-4	slr1600	GO:0022891	<1e-4	sll0837	GO:0003676	0.1243
slr1082	GO:0046164	<1e-4	sll1477	GO:0046128	<1e-4	sll0424	GO:0009123	<1e-4	ssr2047	GO:0022890	<1e-4	slr0957	GO:0003676	0.1243
slr1170	GO:0050801	<1e-4	slr1376	GO:0042455	<1e-4	ssl1520	GO:0019752	<1e-4	ssl0832	GO:0016817	<1e-4	slr0654	GO:0003676	0.1243
sll0871	GO:0043648	<1e-4	sll0048	GO:0055086	<1e-4	slr6100	GO:0044248	<1e-4	sll1775	GO:0015405	<1e-4	sll1241	GO:0003676	0.1243
slr1025	GO:0043933	<1e-4	ssl1552	GO:0034654	<1e-4	sll2011	GO:0034654	<1e-4	slr1565	GO:0005342	<1e-4	slr0232	GO:0003676	0.1243
slr2038	GO:0006753	<1e-4	sll1447	GO:0009059	<1e-4	sll1446	GO:0071841	<1e-4	slr8044	GO:0015405	<1e-4	slr1762	GO:0003676	0.1243
ssl2064	GO:0009892	<1e-4	slr5111	GO:0006732	<1e-4	slr6045	GO:0018130	<1e-4	slr1098	GO:0022857	<1e-4	sll7065	GO:0003676	0.1243
ssr3570	GO:0042180	<1e-4	slr1362	GO:0031640	<1e-4	sll1942	GO:0019362	<1e-4	sll0301	GO:0022891	<1e-4	slr1895	GO:0003676	0.1243
sll0355	GO:0009260	<1e-4	ssr3300	GO:0044282	<1e-4	slr0637	GO:0016054	<1e-4	slr6005	GO:0003674	<1e-4	sll0888	GO:0003676	0.1243
sll0710	GO:0006163	<1e-4	sll1632	GO:0009150	<1e-4	slr0514	GO:0009263	<1e-4	ssr3122	GO:0022892	<1e-4	sll0188	GO:0003676	0.1243
slr1811	GO:0006733	<1e-4	ssr5106	GO:0048518	<1e-4	slr0680	GO:0006220	<1e-4	sll0788	GO:0015267	<1e-4	sll0156	GO:0003676	0.1243
sll1119	GO:0033013	<1e-4	sll1715	GO:0050789	<1e-4	sll0253	GO:0006793	<1e-4	slr6080	GO:0015291	<1e-4	sll0268	GO:0003676	0.1243
sll0781	GO:0048522	<1e-4	ssr6024	GO:0055086	<1e-4	slr1068	GO:0008610	<1e-4	ssl7048	GO:0015293	<1e-4	slr0053	GO:0003676	0.1243
sll1863	GO:0006721	<1e-4	sll0688	GO:0034641	<1e-4	sll1722	GO:0044260	<1e-4	ssr6027	GO:0060089	<1e-4	slr1413	GO:0003676	0.1243
sll0071	GO:0033013	<1e-4	sll0659	GO:0080090	<1e-4	slr1168	GO:0055114	<1e-4	slr1417	GO:0008171	<1e-4	slr1288	GO:0003676	0.1243
slr6094	GO:0001932	<1e-4	slr1187	GO:0006793	<1e-4	sll5034	GO:0009308	<1e-4	sll0641	GO:0015293	<1e-4	slr1240	GO:0003676	0.1243
sll0328	GO:0043412	<1e-4	sll1163	GO:0009144	<1e-4	ssl1520	GO:0009260	<1e-4	ssl2996	GO:0016818	<1e-4	sll0688	GO:0003676	0.1243

sll1272	GO:0055086	<1e-4	sll1995	GO:0051179	<1e-4	slr0366	GO:0050896	<1e-4	sll0309	GO:0016462	<1e-4	slr2005	GO:0003676	0.1243
ssl5098	GO:0048878	<1e-4	slr7011	GO:0044282	<1e-4	slr2111	GO:0044260	<1e-4	slr2018	GO:0022832	<1e-4	sll1752	GO:0003676	0.1243
sll1541	GO:0046128	<1e-4	sll0781	GO:0006163	<1e-4	slr0728	GO:0043412	<1e-4	ssl7039	GO:0015077	<1e-4	sll1411	GO:0003676	0.1243
slr0957	GO:0080090	<1e-4	sll0168	GO:0046128	<1e-4	slr1425	GO:0008150	<1e-4	ssr2803	GO:0022891	<1e-4	slr1128	GO:0003676	0.1243
ssr5019	GO:0080090	<1e-4	slr0575	GO:0043648	<1e-4	sll1233	GO:0006733	<1e-4	slr0765	GO:0060089	<1e-4	sll1950	GO:0003676	0.1243
ssr5011	GO:0006732	<1e-4	slr1033	GO:0019751	<1e-4	sll1477	GO:0051186	<1e-4	ssl1690	GO:0042623	<1e-4	sll0180	GO:0003676	0.1243
sll0479	GO:0019751	<1e-4	slr1261	GO:0008150	<1e-4	slr0821	GO:0072524	<1e-4	slr0109	GO:0003674	<1e-4	sll0281	GO:0003676	0.1243
sll1583	GO:0046128	<1e-4	sll0749	GO:0005996	<1e-4	sll1054	GO:0009059	<1e-4	sll0751	GO:0022838	<1e-4	sll1925	GO:0003676	0.1243
sll1913	GO:0016054	<1e-4	ssr5011	GO:0016052	<1e-4	slr1464	GO:0052111	<1e-4	slr7071	GO:0015399	<1e-4	sll1251	GO:0003676	0.1243
slr1203	GO:0009165	<1e-4	sll0149	GO:0044248	<1e-4	sll1233	GO:0009059	<1e-4	ssr5011	GO:0016462	<1e-4	sll1240	GO:0003676	0.1243
slr6016	GO:0033013	<1e-4	slr1951	GO:0006721	<1e-4	slr1648	GO:0046128	<1e-4	slr0978	GO:0042623	<1e-4	sll0588	GO:0003676	0.1243
sll0286	GO:0019222	<1e-4	slr0609	GO:0006721	<1e-4	sll2011	GO:0052188	<1e-4	sll0198	GO:0016462	<1e-4	slr1303	GO:0003676	0.1243
slr1275	GO:0048519	<1e-4	ssr6019	GO:0005996	<1e-4	sll0984	GO:0080090	<1e-4	slr1658	GO:0015075	<1e-4	sll1582	GO:0003676	0.1243
sll0602	GO:0051716	<1e-4	sll1163	GO:0019219	<1e-4	slr1342	GO:0001932	<1e-4	slr1530	GO:0003674	<1e-4	slr0664	GO:0003676	0.1243
slr2110	GO:0018130	<1e-4	sll0788	GO:0009161	<1e-4	slr1970	GO:0072522	<1e-4	sll1426	GO:0003674	<1e-4	slr1999	GO:0016787	0.1232
slr6038	GO:0019752	<1e-4	slr5073	GO:0006066	<1e-4	slr1624	GO:0044282	<1e-4	sll0443	GO:0043167	<1e-4	sll0185	GO:0016787	0.1232
sll0298	GO:0071842	<1e-4	slr0149	GO:0006732	<1e-4	ssr6079	GO:0044003	<1e-4	sll0198	GO:0022838	<1e-4	ssl0352	GO:0016787	0.1232
slr5073	GO:0006753	<1e-4	ssl7047	GO:0050794	<1e-4	slr0146	GO:0009057	<1e-4	slr0104	GO:0060089	<1e-4	slr0006	GO:0016787	0.1232
slr1593	GO:0048522	<1e-4	ssr2060	GO:0006631	<1e-4	sll0007	GO:0034641	<1e-4	sll0763	GO:0015267	<1e-4	slr1788	GO:0016787	0.1232
slr7010	GO:0052188	<1e-4	slr0913	GO:0045184	<1e-4	sll0508	GO:0022411	<1e-4	slr6080	GO:0043167	<1e-4	slr1210	GO:0016787	0.1232
ssr3129	GO:0009117	<1e-4	sll1485	GO:0043933	<1e-4	slr1179	GO:0009132	<1e-4	sll0822	GO:0043492	<1e-4	slr0845	GO:0016787	0.1232
slr2027	GO:0080090	<1e-4	sll1247	GO:0072524	<1e-4	slr0664	GO:0022411	<1e-4	slr1365	GO:0022832	<1e-4	ssr2787	GO:0016787	0.1232
sll1638	GO:0043648	<1e-4	sll0508	GO:0071554	<1e-4	slr1854	GO:0043648	<1e-4	ssr2998	GO:0016746	<1e-4	sll0785	GO:0016787	0.1232
sll0577	GO:0009991	<1e-4	sll0761	GO:0046164	<1e-4	sll1737	GO:0051186	<1e-4	ssr1499	GO:0016741	<1e-4	ssr3571	GO:0016787	0.1232
sll1681	GO:0022607	<1e-4	slr1699	GO:0006766	<1e-4	sll0225	GO:0009308	<1e-4	slr0144	GO:0008171	<1e-4	sll0355	GO:0016787	0.1232
slr7014	GO:0042180	<1e-4	slr0269	GO:0009141	<1e-4	slr1127	GO:0006721	<1e-4	sll1785	GO:0015291	<1e-4	ssr3122	GO:0016787	0.1232
slr0887	GO:0051171	<1e-4	slr0852	GO:0042180	<1e-4	sll0192	GO:0009161	<1e-4	sll0436	GO:0022838	<1e-4	slr1885	GO:0016787	0.1232
sll2006	GO:0042455	<1e-4	sll5026	GO:0009394	<1e-4	sll0286	GO:0009132	<1e-4	slr1940	GO:0043492	<1e-4	slr0668	GO:0016787	0.1232
sll0586	GO:0051246	<1e-4	slr5101	GO:0006066	<1e-4	slr1790	GO:0009199	<1e-4	slr1915	GO:0016818	<1e-4	ssr2439	GO:0016787	0.1232
slr1530	GO:0043412	<1e-4	slr1363	GO:0009165	<1e-4	sll0327	GO:0006811	<1e-4	slr1425	GO:0003674	<1e-4	slr1203	GO:0016787	0.1232
slr0147	GO:0022607	<1e-4	sll1606	GO:0055086	<1e-4	slr0728	GO:0019751	<1e-4	sll1736	GO:0015399	<1e-4	slr1593	GO:0016787	0.1232
sll1350	GO:0019362	<1e-4	sll0997	GO:0019752	<1e-4	ssr6083	GO:0031326	<1e-4	slr1628	GO:0016817	<1e-4	sll0780	GO:0016787	0.1232
ssl7021	GO:0006066	<1e-4	sll8012	GO:0071554	<1e-4	sll0355	GO:0042180	<1e-4	slr1397	GO:0015405	<1e-4	sll1830	GO:0016787	0.1232
slr0890	GO:0006753	<1e-4	slr0271	GO:0006811	<1e-4	ssr6062	GO:0048522	<1e-4	slr2101	GO:0016746	<1e-4	slr0144	GO:0016787	0.1232
slr1339	GO:0051347	<1e-4	sll0661	GO:0009165	<1e-4	slr5112	GO:0048519	<1e-4	sll8033	GO:0003674	<1e-4	sll1761	GO:0016787	0.1232
slr1577	GO:0051818	<1e-4	ssl1417	GO:0006220	<1e-4	slr1778	GO:0048519	<1e-4	sll1751	GO:0015405	<1e-4	sll0524	GO:0016787	0.1232
ssr7035	GO:0006766	<1e-4	sll0188	GO:0051188	<1e-4	slr0208	GO:0048878	<1e-4	slr1565	GO:0043167	<1e-4	slr0192	GO:0016787	0.1232
ssr2553	GO:0042451	<1e-4	sll0863	GO:0044271	<1e-4	slr0489	GO:0031640	<1e-4	slr1704	GO:0043167	<1e-4	slr0294	GO:0016787	0.1232
sll5067	GO:0071841	<1e-4	ssl8039	GO:0009059	<1e-4	sll0843	GO:0043436	<1e-4	sll0763	GO:0060089	<1e-4	slr1081	GO:0016787	0.1232
sll1552	GO:0043412	<1e-4	ssl7046	GO:0045184	<1e-4	slr1307	GO:0043412	<1e-4	ssr2047	GO:0016746	<1e-4	sll0982	GO:0016787	0.1232
sll5130	GO:0060255	<1e-4	sll0218	GO:0042180	<1e-4	slr1619	GO:0006163	<1e-4	slr0981	GO:0015293	<1e-4	slr1177	GO:0016787	0.1232
ssr1558	GO:0048519	<1e-4	sll7069	GO:0048522	<1e-4	sll1571	GO:0043933	<1e-4	sll1447	GO:0016817	<1e-4	sll1965	GO:0016787	0.1232
sll0160	GO:0019637	<1e-4	sll5097	GO:0006720	<1e-4	slr1790	GO:0006576	<1e-4	slr0392	GO:0015077	<1e-4	sll1954	GO:0016787	0.1232
sll1025	GO:0051246	<1e-4	slr6088	GO:0006721	<1e-4	ssl1300	GO:0045184	<1e-4	slr0594	GO:0022803	<1e-4	sll0066	GO:0016787	0.1232
slr0179	GO:0019222	<1e-4	sll0911	GO:0051179	<1e-4	sll1882	GO:0034654	<1e-4	ssr2962	GO:0022892	<1e-4	ssr2551	GO:0016787	0.1232
ssl5064	GO:0009259	<1e-4	slr0334	GO:0043170	<1e-4	slr6049	GO:0048522	<1e-4	slr1045	GO:0015405	<1e-4	slr0872	GO:0016787	0.1232
sll1730	GO:0009309	<1e-4	slr8014	GO:0009263	<1e-4	slr1071	GO:0009165	<1e-4	ssl5008	GO:0022891	<1e-4	slr1667	GO:0016787	0.1232
slr0921	GO:0050789	<1e-4	sll1092	GO:0048519	<1e-4	sll5028	GO:0044281	<1e-4	slr1862	GO:0016817	<1e-4	slr1275	GO:0016787	0.1232
slr1886	GO:0051716	<1e-4	slr1384	GO:0019362	<1e-4	slr1999	GO:0009144	<1e-4	slr6039	GO:0015075	<1e-4	slr1572	GO:0016787	0.1232
ssr2554	GO:0080090	<1e-4	sll1692	GO:0009161	<1e-4	slr0039	GO:0034654	<1e-4	sll0985	GO:0022857	<1e-4	slr1778	GO:0016787	0.1232
slr0111	GO:0009260	<1e-4	sll0788	GO:0042180	<1e-4	slr1940	GO:0080090	<1e-4	sll1477	GO:0015405	<1e-4	slr0482	GO:0016787	0.1232
slr1573	GO:0009892	<1e-4	sll1775	GO:0090304	<1e-4	sll7070	GO:0050801	<1e-4	ssl1792	GO:0015267	<1e-4	slr1437	GO:0016787	0.1232
sll1698	GO:0006753	<1e-4	sll1447	GO:0051716	<1e-4	ssl0787	GO:0006811	<1e-4	sll1477	GO:0008171	<1e-4	sll0085	GO:0016787	0.1232
ssl8028	GO:0071554	<1e-4	sll1784	GO:0046483	<1e-4	sll1265	GO:0009142	<1e-4	slr1385	GO:0016746	<1e-4	slr1266	GO:0016787	0.1232

sll0328	GO:0009263	<1e-4	slr1513	GO:0034654	<1e-4	ssl1046	GO:0009991	<1e-4	sll1658	GO:0022832	<1e-4	sll0243	GO:0016787	0.1232
slr1774	GO:0051188	<1e-4	sll1925	GO:0080090	<1e-4	sll1606	GO:0006793	<1e-4	slr1222	GO:0015291	<1e-4	sll0532	GO:0016787	0.1232
slr5112	GO:0019219	<1e-4	sll0174	GO:0016053	<1e-4	sll1162	GO:0006721	<1e-4	sll1060	GO:0003674	<1e-4	sll0309	GO:0016787	0.1232
ssr2843	GO:0043412	<1e-4	slr0971	GO:0006732	<1e-4	slr5017	GO:0009141	<1e-4	slr1047	GO:0022892	<1e-4	slr2121	GO:0016787	0.1232
slr0806	GO:0009263	<1e-4	slr1143	GO:0006091	<1e-4	slr1616	GO:0006733	<1e-4	slr0730	GO:0043167	<1e-4	slr0103	GO:0016787	0.1232
ssr6020	GO:0090304	<1e-4	sll0072	GO:0046128	<1e-4	sll0572	GO:0050801	<1e-4	slr0076	GO:0022832	<1e-4	slr0364	GO:0016787	0.1232
slr1616	GO:0042180	<1e-4	ssr7072	GO:0071841	<1e-4	sll1240	GO:0009394	<1e-4	slr0871	GO:0015267	<1e-4	sll1613	GO:0016787	0.1232
slr0232	GO:0006720	<1e-4	slr1142	GO:0006732	<1e-4	sll1272	GO:0065007	<1e-4	slr0184	GO:0022892	<1e-4	slr0723	GO:0016787	0.1232
ssr3341	GO:0043412	<1e-4	slr0654	GO:0008150	<1e-4	slr0914	GO:0042180	<1e-4	sll1765	GO:0022803	<1e-4	sll0198	GO:0016787	0.1232
sll0909	GO:0050789	<1e-4	slr0264	GO:0044271	<1e-4	slr0169	GO:0072524	<1e-4	sll1119	GO:0022890	<1e-4	sll1315	GO:0016787	0.1232
slr0667	GO:0044271	<1e-4	slr1851	GO:0009144	<1e-4	slr0108	GO:0043933	<1e-4	slr1258	GO:0003674	<1e-4	slr2122	GO:0016787	0.1232
sll1652	GO:0071496	<1e-4	slr1612	GO:0009889	<1e-4	slr1660	GO:0001932	<1e-4	sll1926	GO:0022832	<1e-4	sll1563	GO:0016787	0.1232
slr1593	GO:0009987	<1e-4	slr6094	GO:0051179	<1e-4	ssr6086	GO:0071496	<1e-4	sll0282	GO:0016818	<1e-4	sll0456	GO:0016787	0.1232
slr0598	GO:0044275	<1e-4	sll7090	GO:0055082	<1e-4	slr0039	GO:0009141	<1e-4	ssr2439	GO:0016818	<1e-4	slr2073	GO:0016787	0.1232
slr1056	GO:0044248	<1e-4	sll0863	GO:0008150	<1e-4	slr0408	GO:0051179	<1e-4	slr0317	GO:0016817	<1e-4	slr2144	GO:0016787	0.1232
sll5046	GO:0006720	<1e-4	sll1186	GO:0010468	<1e-4	sll0513	GO:0019222	<1e-4	sll0022	GO:0015399	<1e-4	sll1736	GO:0016787	0.1232
sll0198	GO:0051234	<1e-4	slr1241	GO:0006631	<1e-4	slr0852	GO:0090304	<1e-4	sgl0001	GO:0016818	<1e-4	sll1835	GO:0016787	0.1232
sll1306	GO:0019219	<1e-4	ssr6002	GO:0044281	<1e-4	slr1547	GO:0022607	<1e-4	slr1114	GO:0005342	<1e-4	slr1648	GO:0016787	0.1232
sll0062	GO:0006720	<1e-4	ssl1552	GO:0048878	<1e-4	sll8027	GO:0046128	<1e-4	sll0456	GO:0015405	<1e-4	slr0770	GO:0016787	0.1232
slr1572	GO:0044255	<1e-4	ssr5092	GO:0050794	<1e-4	slr1956	GO:0033013	<1e-4	sll1926	GO:0016462	<1e-4	ssr2755	GO:0016787	0.1232
sll0787	GO:0009889	<1e-4	sll0647	GO:0071496	<1e-4	sll5004	GO:0034641	<1e-4	slr1507	GO:0015291	<1e-4	slr1913	GO:0016787	0.1232
sll1660	GO:0006066	<1e-4	slr1276	GO:0072521	<1e-4	slr0148	GO:0044255	<1e-4	slr1438	GO:0015075	<1e-4	ssl2065	GO:0016787	0.1232
sll0609	GO:0051716	<1e-4	ssl1577	GO:0009260	<1e-4	slr1363	GO:0019219	<1e-4	slr2070	GO:0022838	<1e-4	slr1638	GO:0016787	0.1232
ssr2848	GO:0051171	<1e-4	slr0645	GO:0010468	<1e-4	slr7098	GO:0009262	<1e-4	sll1239	GO:0005342	<1e-4	slr0348	GO:0016787	0.1232
slr0364	GO:0009132	<1e-4	sll0191	GO:0006631	<1e-4	ssr0109	GO:0031640	<1e-4	slr6049	GO:0016462	<1e-4	sll0237	GO:0016787	0.1232
sll1834	GO:0072522	<1e-4	slr6012	GO:0051246	<1e-4	slr1780	GO:0050789	<1e-4	sll5046	GO:0022891	<1e-4	sll0756	GO:0016787	0.1232
sll1726	GO:0043412	<1e-4	slr0914	GO:0019751	<1e-4	slr0325	GO:0051179	<1e-4	sll0861	GO:0015291	<1e-4	sll0280	GO:0016787	0.1232
slr0199	GO:0005996	<1e-4	slr1462	GO:0010468	<1e-4	sll1380	GO:0044255	<1e-4	ssr8013	GO:0003674	<1e-4	sll0445	GO:0000166	0.1214
slr1623	GO:0044271	<1e-4	ssr2201	GO:0006066	<1e-4	slr1163	GO:0019751	<1e-4	sll1052	GO:0015077	<1e-4	sll1318	GO:0005215	0.1213
slr7011	GO:0009260	<1e-4	slr1071	GO:0044106	<1e-4	sll1495	GO:0044275	<1e-4	sll1218	GO:0022803	<1e-4	slr1918	GO:0005215	0.1213
ssl3142	GO:0034641	<1e-4	slr1537	GO:0046164	<1e-4	slr1668	GO:0019751	<1e-4	sll1247	GO:0016462	<1e-4	sll1601	GO:0005215	0.1213
ssl5114	GO:0034654	<1e-4	sll1527	GO:0046164	<1e-4	sll1659	GO:0048878	<1e-4	sll8002	GO:0015405	<1e-4	slr0250	GO:0005215	0.1213
slr1918	GO:0051234	<1e-4	slr6039	GO:0009124	<1e-4	sll6054	GO:0051186	<1e-4	sll1858	GO:0022857	<1e-4	sll1219	GO:0005215	0.1213
sll1730	GO:0009259	<1e-4	sll0933	GO:0052111	<1e-4	sll1352	GO:0044106	<1e-4	slr1915	GO:0016746	<1e-4	sll2006	GO:0005215	0.1213
ssl1417	GO:0016054	<1e-4	sll1638	GO:0006163	<1e-4	sll1411	GO:0048522	<1e-4	sll0543	GO:0043167	<1e-4	slr0243	GO:0005215	0.1213
ssl5100	GO:0010468	<1e-4	slr6073	GO:0006082	<1e-4	ssl1417	GO:0009394	<1e-4	slr5053	GO:0016462	<1e-4	slr0476	GO:0005215	0.1213
sll0156	GO:0044248	<1e-4	slr0144	GO:0009991	<1e-4	sll1086	GO:0009991	<1e-4	ssl1004	GO:0016462	<1e-4	slr0871	GO:0005215	0.1213
ssr2781	GO:0016053	<1e-4	slr0702	GO:0006576	<1e-4	ssl0109	GO:0009892	<1e-4	slr1866	GO:0022803	<1e-4	ssr1698	GO:0005215	0.1213
sll1307	GO:0048519	<1e-4	slr0545	GO:0034641	<1e-4	slr6104	GO:0045184	<1e-4	ssr6079	GO:0060089	<1e-4	slr0211	GO:0005215	0.1213
slr1546	GO:0010468	<1e-4	slr6075	GO:0072521	<1e-4	slr1576	GO:0034641	<1e-4	sll1608	GO:0022803	<1e-4	slr1917	GO:0005215	0.1213
ssl5095	GO:0042451	<1e-4	sll1373	GO:0006576	<1e-4	ssr1114	GO:0006811	<1e-4	slr1612	GO:0015399	<1e-4	slr0503	GO:0005215	0.1213
ssr0657	GO:0043170	<1e-4	ssl0483	GO:0043170	<1e-4	slr6104	GO:0072521	<1e-4	slr1417	GO:0016818	<1e-4	sll1715	GO:0005215	0.1213
sll0703	GO:0009132	<1e-4	slr1530	GO:0008610	<1e-4	slr6104	GO:0006721	<1e-4	sll0269	GO:0016741	<1e-4	sll0743	GO:0005215	0.1213
sll0577	GO:0006220	<1e-4	slr1101	GO:0050801	<1e-4	ssl7074	GO:0009309	<1e-4	ssr2962	GO:0015293	<1e-4	sll0296	GO:0005215	0.1213
sll0449	GO:0009142	<1e-4	slr1958	GO:0046907	<1e-4	sll0265	GO:0051171	<1e-4	slr1187	GO:0005342	<1e-4	slr0423	GO:0005215	0.1213
slr0885	GO:0044260	<1e-4	sll0071	GO:0006082	<1e-4	sll0853	GO:0044283	<1e-4	sll0909	GO:0015291	<1e-4	sll1942	GO:0005215	0.1213
slr0341	GO:0010556	<1e-4	slr1235	GO:0043648	<1e-4	ssr2843	GO:0022411	<1e-4	slr0404	GO:0015077	<1e-4	ssr1499	GO:0016787	0.1203
ssl1263	GO:0018130	<1e-4	sll1442	GO:0071840	<1e-4	sll1193	GO:0071496	<1e-4	ssr0109	GO:0022892	<1e-4	sll1757	GO:0016787	0.1203
slr1082	GO:0019222	<1e-4	slr0516	GO:0006082	<1e-4	slr6080	GO:0043933	<1e-4	slr6031	GO:0022832	<1e-4	slr1468	GO:0016787	0.1203
slr0456	GO:0031326	<1e-4	sll0703	GO:0072521	<1e-4	slr6063	GO:0051716	<1e-4	slr6031	GO:0005342	<1e-4	ssr2912	GO:0016787	0.1203
ssr2615	GO:0019222	<1e-4	slr0362	GO:0008150	<1e-4	slr0318	GO:0006631	<1e-4	slr1066	GO:0022838	<1e-4	slr1178	GO:0016787	0.1203
ssr6062	GO:0046907	<1e-4	slr0509	GO:0031323	<1e-4	sll1671	GO:0016054	<1e-4	ssr1698	GO:0022890	<1e-4	slr0148	GO:0016787	0.1203
ssl7021	GO:0009394	<1e-4	sll0443	GO:0009308	<1e-4	sll1938	GO:0009987	<1e-4	sll0787	GO:0015075	<1e-4	slr6004	GO:0016787	0.1203
slr1674	GO:0044106	<1e-4	sll0381	GO:0043648	<1e-4	sll0181	GO:0006732	<1e-4	sll1691	GO:0042623	<1e-4	slr7013	GO:0016787	0.1203

ssl2814	GO:0009144	<1e-4	slr1493	GO:0031326	<1e-4	ssr6085	GO:0044275	<1e-4	ssl5031	GO:0022836	<1e-4	sll1396	GO:0016787	0.1203
sll1749	GO:0010556	<1e-4	sll1651	GO:0044282	<1e-4	slr0607	GO:0044271	<1e-4	slr0348	GO:0016817	<1e-4	ssl5095	GO:0016787	0.1203
slr1177	GO:0071554	<1e-4	sll0584	GO:0046483	<1e-4	slr1315	GO:0048518	<1e-4	slr6008	GO:0015291	<1e-4	sll0751	GO:0016787	0.1203
ssr0102	GO:0048878	<1e-4	sll1049	GO:0009987	<1e-4	sll1863	GO:0006793	<1e-4	sll0982	GO:0022803	<1e-4	slr1863	GO:0016787	0.1203
slr1173	GO:0009056	<1e-4	slr8044	GO:0043412	<1e-4	slr0545	GO:0043170	<1e-4	sll0448	GO:0022891	<1e-4	sll1025	GO:0016787	0.1203
slr0619	GO:0044106	<1e-4	sll0802	GO:0051179	<1e-4	sll0780	GO:0001932	<1e-4	sll7065	GO:0022891	<1e-4	sll0860	GO:0016787	0.1203
slr0392	GO:0050794	<1e-4	ssl7022	GO:0050794	<1e-4	slr0876	GO:0009165	<1e-4	sll1186	GO:0015399	<1e-4	sll0775	GO:0016787	0.1203
slr0888	GO:0009259	<1e-4	slr1315	GO:0006732	<1e-4	slr1033	GO:0048523	<1e-4	slr1196	GO:0043492	<1e-4	slr7025	GO:0016787	0.1203
sll1232	GO:0072521	<1e-4	slr0887	GO:0010556	<1e-4	sll1722	GO:0009394	<1e-4	sll1455	GO:0022890	<1e-4	slr0960	GO:0016787	0.1203
slr0262	GO:0008150	<1e-4	sll0156	GO:0006733	<1e-4	slr0058	GO:0009260	<1e-4	sll0007	GO:0015267	<1e-4	slr0092	GO:0016787	0.1203
slr5037	GO:0031323	<1e-4	slr1235	GO:0006732	<1e-4	slr1062	GO:0009165	<1e-4	slr6014	GO:0060089	<1e-4	sll0174	GO:0016787	0.1203
ssr1407	GO:0009308	<1e-4	sll5033	GO:0009141	<1e-4	sll1979	GO:0019752	<1e-4	sll0498	GO:0022891	<1e-4	ssr2317	GO:0016787	0.1203
slr0516	GO:0042451	<1e-4	sll0412	GO:0042455	<1e-4	ssl0350	GO:0006082	<1e-4	slr0209	GO:0015291	<1e-4	sll0423	GO:0016787	0.1203
ssr6024	GO:0009199	<1e-4	sll0444	GO:0044282	<1e-4	ssr3410	GO:0010468	<1e-4	slr7024	GO:0015405	<1e-4	slr0362	GO:0016787	0.1203
sll1151	GO:0009987	<1e-4	ssl3291	GO:0048523	<1e-4	slr0356	GO:0046164	<1e-4	slr1069	GO:0015291	<1e-4	slr6014	GO:0016787	0.1203
sll0241	GO:0006766	<1e-4	slr0303	GO:0034654	<1e-4	slr2003	GO:0006576	<1e-4	sll0518	GO:0003674	<1e-4	sll0518	GO:0016787	0.1203
ssr2962	GO:0044106	<1e-4	slr1419	GO:0006576	<1e-4	slr0964	GO:0006811	<1e-4	slr1571	GO:0016462	<1e-4	slr0065	GO:0016787	0.1203
sll0888	GO:0016052	<1e-4	slr0496	GO:0051171	<1e-4	sll0319	GO:0006631	<1e-4	slr0771	GO:0060089	<1e-4	slr1273	GO:0016787	0.1203
slr0362	GO:0019752	<1e-4	sll0810	GO:0044275	<1e-4	sll1135	GO:0065007	<1e-4	sll0023	GO:0015077	<1e-4	sgl0001	GO:0016787	0.1203
sll0984	GO:0031323	<1e-4	slr0769	GO:0006732	<1e-4	ssr2615	GO:0034641	<1e-4	slr1603	GO:0022890	<1e-4	sll1773	GO:0016787	0.1203
slr1218	GO:0009892	<1e-4	slr2103	GO:0009893	<1e-4	sll0585	GO:0046483	<1e-4	slr2025	GO:0022890	<1e-4	slr6031	GO:0016787	0.1203
sll0406	GO:0009991	<1e-4	slr6075	GO:0008150	<1e-4	ssl1255	GO:0018130	<1e-4	sll0763	GO:0022803	<1e-4	sll5062	GO:0016787	0.1203
slr1107	GO:0031640	<1e-4	sll1611	GO:0009991	<1e-4	ssl0410	GO:0006811	<1e-4	slr1866	GO:0042623	<1e-4	slr0870	GO:0016787	0.1203
ssr6003	GO:0044271	<1e-4	slr0147	GO:0019219	<1e-4	sll1049	GO:0055086	<1e-4	slr1541	GO:0022832	<1e-4	slr0168	GO:0016787	0.1203
sll1247	GO:0009259	<1e-4	slr1178	GO:0008150	<1e-4	slr1799	GO:0050801	<1e-4	slr0579	GO:0022892	<1e-4	slr1576	GO:0016787	0.1203
slr1095	GO:0009394	<1e-4	slr7015	GO:0019751	<1e-4	sll5046	GO:0071554	<1e-4	slr2101	GO:0022832	<1e-4	slr1207	GO:0016787	0.1203
slr1862	GO:0044248	<1e-4	sll0296	GO:0006811	<1e-4	slr1819	GO:0043436	<1e-4	slr0509	GO:0016462	<1e-4	sll1866	GO:0016787	0.1203
slr0082	GO:0051716	<1e-4	sll0448	GO:0006732	<1e-4	slr0006	GO:0065007	<1e-4	ssr5117	GO:0022832	<1e-4	slr1958	GO:0016787	0.1203
sll0068	GO:0006766	<1e-4	ssr5074	GO:0043549	<1e-4	slr6007	GO:0009260	<1e-4	slr7095	GO:0015267	<1e-4	ssr2422	GO:0016787	0.1203
sll0740	GO:0016052	<1e-4	sll1960	GO:0044281	<1e-4	slr2120	GO:0044003	<1e-4	sll1158	GO:0005342	<1e-4	slr0619	GO:0016787	0.1203
sll1835	GO:0048523	<1e-4	sll0482	GO:0009165	<1e-4	sll1880	GO:0033013	<1e-4	ssr1558	GO:0015075	<1e-4	sll5006	GO:0016787	0.1203
sll0198	GO:0043933	<1e-4	sll1304	GO:0001932	<1e-4	slr1935	GO:0052188	<1e-4	ssr2201	GO:0015075	<1e-4	slr7096	GO:0016787	0.1203
slr1163	GO:0071496	<1e-4	slr0505	GO:0009161	<1e-4	slr1419	GO:0043549	<1e-4	slr1203	GO:0015293	<1e-4	ssl2245	GO:0016787	0.1203
sll0350	GO:0051171	<1e-4	slr5021	GO:0009987	<1e-4	slr1450	GO:0050801	<1e-4	slr1170	GO:0016462	<1e-4	slr2010	GO:0016787	0.1203
sll5090	GO:0051246	<1e-4	slr6090	GO:0050896	<1e-4	slr6103	GO:0042451	<1e-4	ssr6085	GO:0015267	<1e-4	slr0569	GO:0016787	0.1203
sll1714	GO:0009141	<1e-4	slr1083	GO:0006811	<1e-4	ssr2611	GO:0006732	<1e-4	sll0614	GO:0022892	<1e-4	ssr5120	GO:0016787	0.1203
slr0053	GO:0043436	<1e-4	sll1956	GO:0033013	<1e-4	slr1449	GO:0006811	<1e-4	slr0423	GO:0015077	<1e-4	sgl0002	GO:0016787	0.1203
slr5118	GO:0052111	<1e-4	slr1593	GO:0048878	<1e-4	slr0313	GO:0016054	<1e-4	sll0382	GO:0015291	<1e-4	sll1378	GO:0016787	0.1203
sll1272	GO:0042180	<1e-4	slr1273	GO:0080090	<1e-4	slr0065	GO:0072527	<1e-4	sll1151	GO:0015075	<1e-4	ssl0312	GO:0016787	0.1203
slr1263	GO:0065007	<1e-4	slr2125	GO:0006220	<1e-4	slr0519	GO:0065007	<1e-4	slr1827	GO:0016746	<1e-4	slr0598	GO:0016787	0.1203
sll0752	GO:0042180	<1e-4	slr0712	GO:0051246	<1e-4	sll0456	GO:0006732	<1e-4	slr1162	GO:0022891	<1e-4	ssl2717	GO:0016787	0.1203
slr1866	GO:0006091	<1e-4	sll1254	GO:0006721	<1e-4	slr0751	GO:0008150	<1e-4	slr0145	GO:0060089	<1e-4	sll1355	GO:0016787	0.1203
sll5047	GO:0071840	<1e-4	sll0863	GO:0009394	<1e-4	slr0771	GO:0009056	<1e-4	slr1122	GO:0022838	<1e-4	ssl2420	GO:0016787	0.1203
slr1474	GO:0009057	<1e-4	sll0997	GO:0072527	<1e-4	slr1338	GO:0046164	<1e-4	sll0309	GO:0022857	<1e-4	slr0146	GO:0016787	0.1203
ssr2962	GO:0009123	<1e-4	sll1218	GO:0006793	<1e-4	slr0729	GO:0022411	<1e-4	slr1812	GO:0016462	<1e-4	slr1315	GO:0016787	0.1203
sll0428	GO:0009056	<1e-4	slr1773	GO:0046394	<1e-4	slr1519	GO:0071841	<1e-4	slr0109	GO:0016741	<1e-4	slr0784	GO:0016787	0.1203
sll1380	GO:0044260	<1e-4	sll1254	GO:0019751	<1e-4	sll1254	GO:0016054	<1e-4	ssr6020	GO:0043492	<1e-4	ssl1498	GO:0016787	0.1203
sll1158	GO:0009165	<1e-4	sll0062	GO:0044271	<1e-4	sll1155	GO:0046394	<1e-4	slr7092	GO:0043167	<1e-4	ssr6048	GO:0016787	0.1203
sll1880	GO:0071840	<1e-4	slr0654	GO:0031326	<1e-4	ssr6099	GO:0043412	<1e-4	ssr2422	GO:0060089	<1e-4	sll0168	GO:0016787	0.1203
sll0875	GO:0009263	<1e-4	slr1189	GO:0016054	<1e-4	slr1865	GO:0009144	<1e-4	slr0670	GO:0060089	<1e-4	slr7101	GO:0016787	0.1203
slr6094	GO:0080090	<1e-4	slr0871	GO:0051171	<1e-4	slr0606	GO:0009263	<1e-4	ssl3451	GO:0015405	<1e-4	slr0333	GO:0016787	0.1203
sll1738	GO:0010468	<1e-4	slr1911	GO:0051171	<1e-4	sll1132	GO:0044255	<1e-4	slr1644	GO:0004872	<1e-4	slr1957	GO:0016787	0.1203
sll1835	GO:0006720	<1e-4	slr7026	GO:0072528	<1e-4	slr0300	GO:0009123	<1e-4	sll1061	GO:0022832	<1e-4	slr1168	GO:0016787	0.1203
slr5037	GO:0044260	<1e-4	sll0763	GO:0071842	<1e-4	sll0552	GO:0009889	<1e-4	slr0305	GO:0005342	<1e-4	sll0853	GO:0016787	0.1203

ssl3692	GO:0051171	<1e-4	sll1319	GO:0051186	<1e-4	slr1210	GO:0006766	<1e-4	slr1847	GO:0042623	<1e-4	slr6039	GO:0016787	0.1203
slr1143	GO:0060255	<1e-4	sml0011	GO:0044282	<1e-4	ssr3189	GO:0031640	<1e-4	ssr3300	GO:0005342	<1e-4	sll0478	GO:0016787	0.1203
slr1813	GO:0044106	<1e-4	slr0273	GO:0019222	<1e-4	sll1068	GO:0045184	<1e-4	sll0803	GO:0022890	<1e-4	slr0489	GO:0016787	0.1203
slr5037	GO:0009117	<1e-4	slr5116	GO:0009117	<1e-4	slr0476	GO:0009059	<1e-4	sll0354	GO:0015293	<1e-4	slr7083	GO:0016787	0.1203
slr0610	GO:0034654	<1e-4	sll1401	GO:0072528	<1e-4	slr8021	GO:0046907	<1e-4	ssl1552	GO:0015291	<1e-4	sll5130	GO:0016787	0.1203
sll0736	GO:0033013	<1e-4	sll1174	GO:0031640	<1e-4	ssl0410	GO:0044248	<1e-4	slr5112	GO:0043167	<1e-4	slr7081	GO:0016787	0.1203
slr1419	GO:0009262	<1e-4	sll0157	GO:0044275	<1e-4	slr0304	GO:0045184	<1e-4	ssl0461	GO:0015075	<1e-4	slr1218	GO:0016787	0.1203
slr0157	GO:0009262	<1e-4	slr1081	GO:0042180	<1e-4	ssr6027	GO:0090304	<1e-4	slr1865	GO:0043167	<1e-4	sll1960	GO:0016787	0.1203
slr0806	GO:0055086	<1e-4	slr0852	GO:0009150	<1e-4	slr1045	GO:0009150	<1e-4	sll2006	GO:0015075	<1e-4	ssr0332	GO:0016787	0.1203
slr7091	GO:0010556	<1e-4	slr6103	GO:0080090	<1e-4	sll1352	GO:0022411	<1e-4	slr2084	GO:0015267	<1e-4	sll0369	GO:0016787	0.1203
ssl5015	GO:0051818	<1e-4	ssl3451	GO:0009263	<1e-4	slr0885	GO:0001932	<1e-4	sll0147	GO:0003674	<1e-4	sll1163	GO:0016787	0.1203
slr1603	GO:0022411	<1e-4	sll1396	GO:0034641	<1e-4	sll0552	GO:0006082	<1e-4	sll1401	GO:0043167	<1e-4	slr6063	GO:0016787	0.1203
sll0572	GO:0009308	<1e-4	slr1815	GO:0048522	<1e-4	slr1699	GO:0044275	<1e-4	slr1547	GO:0043167	<1e-4	ssr7036	GO:0016787	0.1203
sll1542	GO:0019222	<1e-4	slr1911	GO:0018130	<1e-4	slr1623	GO:0043412	<1e-4	slr6090	GO:0022838	<1e-4	slr7012	GO:0016787	0.1203
slr1819	GO:0051716	<1e-4	slr1210	GO:0006720	<1e-4	sll0857	GO:0009309	<1e-4	slr1083	GO:0016746	<1e-4	sll0282	GO:0016787	0.1203
sll1381	GO:0051188	<1e-4	slr1915	GO:0009117	<1e-4	slr0596	GO:0008150	<1e-4	slr1773	GO:0022836	<1e-4	slr0355	GO:0016787	0.1203
ssl0467	GO:0005996	<1e-4	sll1765	GO:0009059	<1e-4	ssr2551	GO:0009142	<1e-4	slr1407	GO:0015075	<1e-4	slr0058	GO:0016787	0.1203
slr8022	GO:0009117	<1e-4	slr0821	GO:0046164	<1e-4	slr0304	GO:0006733	<1e-4	sll0606	GO:0016746	<1e-4	sll1217	GO:0016787	0.1203
slr6021	GO:0009892	<1e-4	slr1601	GO:0018130	<1e-4	slr1780	GO:0006220	<1e-4	sll0641	GO:0016818	<1e-4	slr1814	GO:0016787	0.1203
sll0996	GO:0051347	<1e-4	slr1726	GO:0072521	<1e-4	sll1764	GO:0055086	<1e-4	sll0321	GO:0015267	<1e-4	slr0890	GO:0016787	0.1203
slr0670	GO:0051347	<1e-4	slr0876	GO:0033013	<1e-4	slr1365	GO:0048523	<1e-4	slr6029	GO:0016817	<1e-4	sll1203	GO:0016787	0.1203
sll1960	GO:0048518	<1e-4	sll1239	GO:0009165	<1e-4	slr1702	GO:0044271	<1e-4	slr0498	GO:0022857	<1e-4	sll0498	GO:0016787	0.1203
slr0374	GO:0043436	<1e-4	slr0082	GO:0034654	<1e-4	slr0712	GO:0044255	<1e-4	ssr6003	GO:0022892	<1e-4	sll0761	GO:0016787	0.1203
slr2105	GO:0044282	<1e-4	slr0334	GO:0009124	<1e-4	slr1932	GO:0033013	<1e-4	sll1995	GO:0015399	<1e-4	slr7016	GO:0016787	0.1203
sll1925	GO:0048518	<1e-4	slr6047	GO:0008150	<1e-4	sll0611	GO:0018130	<1e-4	sll1960	GO:0015399	<1e-4	sll1155	GO:0016787	0.1203
sll0609	GO:0006733	<1e-4	sll1252	GO:0008610	<1e-4	slr0393	GO:0005996	<1e-4	slr1206	GO:0015291	<1e-4	sll7063	GO:0016787	0.1203
sll0359	GO:0051818	<1e-4	ssr5011	GO:0043933	<1e-4	slr2025	GO:0048878	<1e-4	sll1658	GO:0022838	<1e-4	slr1535	GO:0016787	0.1203
slr1811	GO:0006220	<1e-4	ssl0738	GO:0009987	<1e-4	sll0737	GO:0016052	<1e-4	sll0069	GO:0022857	<1e-4	ssl3451	GO:0016787	0.1203
ssr8047	GO:0009987	<1e-4	ssr1258	GO:0051347	<1e-4	ssl1498	GO:0031326	<1e-4	slr0742	GO:0015267	<1e-4	sll1606	GO:0016787	0.1203
ssl7022	GO:0010556	<1e-4	sll1352	GO:0046394	<1e-4	slr0269	GO:0051246	<1e-4	sll1021	GO:0022803	<1e-4	slr1276	GO:0016787	0.1203
sll1109	GO:0016052	<1e-4	ssl5100	GO:0006733	<1e-4	ssr7079	GO:0009161	<1e-4	slr7096	GO:0015399	<1e-4	slr1534	GO:0016787	0.1203
sll1036	GO:0006811	<1e-4	slr1670	GO:0019637	<1e-4	ssr0082	GO:0043933	<1e-4	sll1162	GO:0042623	<1e-4	sll1765	GO:0016787	0.1203
sll0508	GO:0034654	<1e-4	ssl3382	GO:0016054	<1e-4	sll0482	GO:0006220	<1e-4	sll0752	GO:0015267	<1e-4	ssr0335	GO:0016787	0.1203
slr6047	GO:0034641	<1e-4	sll1319	GO:0072527	<1e-4	slr1851	GO:0019751	<1e-4	slr1568	GO:0022890	<1e-4	ssl5129	GO:0016787	0.1203
slr0870	GO:0042180	<1e-4	ssl5008	GO:0043436	<1e-4	slr2004	GO:0006721	<1e-4	sll0558	GO:0015291	<1e-4	slr0049	GO:0016787	0.1203
slr0740	GO:0050801	<1e-4	slr5112	GO:0006766	<1e-4	sll0456	GO:0046164	<1e-4	slr1363	GO:0015267	<1e-4	ssl1923	GO:0016787	0.1203
slr1657	GO:0009123	<1e-4	sll1853	GO:0043170	<1e-4	sll0659	GO:0009889	<1e-4	slr0579	GO:0022832	<1e-4	sll5047	GO:0016787	0.1203
sll0608	GO:0009117	<1e-4	slr0586	GO:0071554	<1e-4	sll1381	GO:0005996	<1e-4	slr0498	GO:0016462	<1e-4	slr0552	GO:0016787	0.1203
slr1203	GO:0009987	<1e-4	sll1880	GO:0071842	<1e-4	slr0872	GO:0046483	<1e-4	slr0453	GO:0015399	<1e-4	sll0762	GO:0016787	0.1203
slr1658	GO:0008150	<1e-4	slr1913	GO:0046907	<1e-4	sll7070	GO:0008150	<1e-4	slr0249	GO:0022836	<1e-4	slr7023	GO:0016787	0.1203
sll1036	GO:0009161	<1e-4	ssl0738	GO:0034654	<1e-4	sll0198	GO:0080090	<1e-4	sll7064	GO:0022838	<1e-4	sll1880	GO:0016787	0.1203
sll1832	GO:0052111	<1e-4	sll5069	GO:0009165	<1e-4	slr6033	GO:0055082	<1e-4	slr0273	GO:0022803	<1e-4	sll0293	GO:0016787	0.1203
sll0837	GO:0045184	<1e-4	slr0989	GO:0050896	<1e-4	sll1388	GO:0006163	<1e-4	slr0440	GO:0043492	<1e-4	slr1102	GO:0016787	0.1203
sll0676	GO:0001932	<1e-4	slr1507	GO:0031640	<1e-4	slr6104	GO:0044248	<1e-4	ssr6046	GO:0042623	<1e-4	sll1921	GO:0016787	0.1203
sll1926	GO:0005996	<1e-4	slr1648	GO:0001932	<1e-4	sll1164	GO:0048878	<1e-4	slr1628	GO:0016741	<1e-4	sll0473	GO:0016787	0.1203
slr1194	GO:0072522	<1e-4	sll0837	GO:0019751	<1e-4	slr0581	GO:0009199	<1e-4	sll1769	GO:0016462	<1e-4	slr6006	GO:0016787	0.1203
slr6044	GO:0006753	<1e-4	slr6021	GO:0009260	<1e-4	slr2000	GO:0050896	<1e-4	slr1339	GO:0015291	<1e-4	slr1660	GO:0016787	0.1203
slr0729	GO:0031640	<1e-4	sll5069	GO:0044271	<1e-4	sll1785	GO:0009132	<1e-4	ssr3570	GO:0016746	<1e-4	sll1162	GO:0016787	0.1203
slr0217	GO:0009059	<1e-4	slr1301	GO:0019222	<1e-4	slr0049	GO:0006793	<1e-4	slr0503	GO:0016462	<1e-4	slr1195	GO:0016787	0.1203
slr0498	GO:0034641	<1e-4	sll1348	GO:0048519	<1e-4	slr1659	GO:0046128	<1e-4	slr7026	GO:0022891	<1e-4	slr1568	GO:0016787	0.1203
sll0242	GO:0009144	<1e-4	ssr8047	GO:0048523	<1e-4	slr1378	GO:0051347	<1e-4	slr1025	GO:0043167	<1e-4	ssl7046	GO:0016787	0.1203
sll0281	GO:0022607	<1e-4	ssr2317	GO:0071840	<1e-4	slr0509	GO:0044260	<1e-4	sll1873	GO:0022803	<1e-4	slr6072	GO:0016787	0.1203
slr0552	GO:0072522	<1e-4	sll0482	GO:0006091	<1e-4	slr0607	GO:0043170	<1e-4	sll1834	GO:0015405	<1e-4	slr1365	GO:0016787	0.1203
slr0398	GO:0009394	<1e-4	slr1413	GO:0009144	<1e-4	slr2012	GO:0019637	<1e-4	slr6005	GO:0015077	<1e-4	sll1696	GO:0016787	0.1203

ssr1558	GO:0009059	<1e-4	sll0098	GO:0006721	<1e-4	sll1285	GO:0006066	<1e-4	slr0725	GO:0022890	<1e-4	slr2118	GO:0016787	0.1203
slr1462	GO:0010556	<1e-4	slr1069	GO:0055114	<1e-4	sll5109	GO:0071840	<1e-4	ssl8028	GO:0015291	<1e-4	sll0553	GO:0016787	0.1203
ssr3189	GO:0009394	<1e-4	ssr2551	GO:0010468	<1e-4	slr0930	GO:0009126	<1e-4	sll0811	GO:0016817	<1e-4	slr1721	GO:0016787	0.1203
sll1757	GO:0055082	<1e-4	sll0218	GO:0001932	<1e-4	sll1702	GO:0006091	<1e-4	ssl0483	GO:0022857	<1e-4	slr2032	GO:0016787	0.1203
slr0442	GO:0034660	<1e-4	slr0651	GO:0006220	<1e-4	ssr6062	GO:0018130	<1e-4	slr1053	GO:0016741	<1e-4	slr0196	GO:0016787	0.1203
sll1532	GO:0051716	<1e-4	slr0692	GO:0008150	<1e-4	slr1599	GO:0006733	<1e-4	slr2003	GO:0022857	<1e-4	sll0218	GO:0016787	0.1203
sll1542	GO:0042451	<1e-4	ssl7022	GO:0005996	<1e-4	slr0082	GO:0006732	<1e-4	slr0249	GO:0043167	<1e-4	slr0695	GO:0016787	0.1203
ssr6086	GO:0019752	<1e-4	slr0723	GO:0050801	<1e-4	slr0876	GO:0044106	<1e-4	sll1696	GO:0015077	<1e-4	sll1853	GO:0016787	0.1203
ssr2802	GO:0050801	<1e-4	slr0581	GO:0019751	<1e-4	sll0623	GO:0043648	<1e-4	ssl2245	GO:0022857	<1e-4	sll0863	GO:0016787	0.1203
slr1187	GO:0048878	<1e-4	ssl5091	GO:0006631	<1e-4	slr0907	GO:0006721	<1e-4	slr1114	GO:0008171	<1e-4	slr7015	GO:0016787	0.1203
slr0145	GO:0051347	<1e-4	slr0596	GO:0060255	<1e-4	sll1495	GO:0071842	<1e-4	slr1935	GO:0022803	<1e-4	ssl5065	GO:0016787	0.1203
sll1656	GO:0019219	<1e-4	slr1161	GO:0034641	<1e-4	slr1636	GO:0072522	<1e-4	ssr6027	GO:0022892	<1e-4	slr6015	GO:0016787	0.1203
ssr2962	GO:0071840	<1e-4	slr0913	GO:0006091	<1e-4	slr0397	GO:0010556	<1e-4	slr1218	GO:0022832	<1e-4	sll1348	GO:0016787	0.1203
slr1814	GO:0022411	<1e-4	sll0066	GO:0071496	<1e-4	slr1886	GO:0034654	<1e-4	slr0148	GO:0022890	<1e-4	sll1252	GO:0016787	0.1203
slr6106	GO:0009124	<1e-4	sll0443	GO:0008610	<1e-4	slr7013	GO:0051179	<1e-4	sll0272	GO:0015077	<1e-4	sll8007	GO:0016787	0.1203
ssl2807	GO:0044255	<1e-4	sll0168	GO:0009262	<1e-4	slr2027	GO:0090304	<1e-4	ssl5015	GO:0022832	<1e-4	sll0930	GO:0016787	0.1203
slr0404	GO:0050801	<1e-4	ssl7042	GO:0071840	<1e-4	ssr5092	GO:0046164	<1e-4	sll1289	GO:0015291	<1e-4	sll5032	GO:0016787	0.1203
slr0728	GO:0009144	<1e-4	ssl3829	GO:0044275	<1e-4	ssl5091	GO:0009987	<1e-4	sll1586	GO:0022838	<1e-4	slr1677	GO:0016787	0.1203
sll0283	GO:0052188	<1e-4	ssl3383	GO:0019362	<1e-4	slr1681	GO:0050801	<1e-4	slr0362	GO:0015075	<1e-4	sll0102	GO:0016787	0.1203
ssr3571	GO:0006793	<1e-4	slr1557	GO:0072528	<1e-4	slr7013	GO:0006733	<1e-4	slr0345	GO:0016746	<1e-4	sll8002	GO:0016787	0.1203
slr7094	GO:0042180	<1e-4	sll0301	GO:0009309	<1e-4	slr7059	GO:0072521	<1e-4	sll0382	GO:0015075	<1e-4	slr1484	GO:0016787	0.1203
slr0607	GO:0071842	<1e-4	ssl2971	GO:0051246	<1e-4	ssr5120	GO:0072522	<1e-4	ssr6079	GO:0022836	<1e-4	slr6073	GO:0016787	0.1203
slr0192	GO:0009141	<1e-4	slr1290	GO:0051818	<1e-4	slr0250	GO:0055114	<1e-4	slr1260	GO:0060089	<1e-4	sll0443	GO:0016787	0.1203
sll1239	GO:0019219	<1e-4	slr0919	GO:0050896	<1e-4	slr0642	GO:0019752	<1e-4	sll1652	GO:0015291	<1e-4	sll5033	GO:0016787	0.1203
ssr0102	GO:0009117	<1e-4	ssl1498	GO:0009889	<1e-4	sll0843	GO:0006082	<1e-4	slr1102	GO:0022836	<1e-4	ssl5045	GO:0016787	0.1203
slr1338	GO:0009987	<1e-4	slr7098	GO:0009161	<1e-4	slr0204	GO:0043436	<1e-4	ssl1972	GO:0015291	<1e-4	sll1092	GO:0016787	0.1203
ssr2439	GO:0018130	<1e-4	sll1002	GO:0019219	<1e-4	ssl2807	GO:0006811	<1e-4	slr0023	GO:0022838	<1e-4	slr0751	GO:0016787	0.1203
sll8019	GO:0009124	<1e-4	slr0263	GO:0031640	<1e-4	slr7094	GO:0051716	<1e-4	sll0147	GO:0015293	<1e-4	slr1914	GO:0016787	0.1203
sll0611	GO:0009141	<1e-4	sll1726	GO:0048523	<1e-4	slr1998	GO:0009893	<1e-4	sll0066	GO:0016462	<1e-4	sll0846	GO:0016787	0.1203
sll0162	GO:0009132	<1e-4	slr1288	GO:0006721	<1e-4	slr6091	GO:0051234	<1e-4	slr1073	GO:0022891	<1e-4	sll0008	GO:0016787	0.1203
sll0488	GO:0033013	<1e-4	slr0869	GO:0009199	<1e-4	slr0503	GO:0071841	<1e-4	sll0249	GO:0022838	<1e-4	slr2101	GO:0016787	0.1203
slr1660	GO:0009889	<1e-4	sll1563	GO:0009126	<1e-4	slr1576	GO:0043549	<1e-4	sll7078	GO:0022832	<1e-4	sll7064	GO:0016787	0.1203
ssl5027	GO:0046164	<1e-4	slr0204	GO:0042451	<1e-4	sll0442	GO:0009987	<1e-4	slr1376	GO:0015077	<1e-4	slr0912	GO:0016787	0.1203
sll1193	GO:0008150	<1e-4	sll0623	GO:0009260	<1e-4	sll1738	GO:0010556	<1e-4	slr0592	GO:0022857	<1e-4	slr1547	GO:0016787	0.1203
sll1040	GO:0009123	<1e-4	slr0303	GO:0008610	<1e-4	slr0151	GO:0031323	<1e-4	sll0608	GO:0022892	<1e-4	sll1004	GO:0016787	0.1203
slr2121	GO:0010556	<1e-4	sll1307	GO:0046164	<1e-4	slr2125	GO:0048518	<1e-4	slr2073	GO:0005342	<1e-4	slr0199	GO:0016787	0.1203
slr1431	GO:0044283	<1e-4	ssl3383	GO:0051171	<1e-4	ssl2749	GO:0060255	<1e-4	slr0341	GO:0015291	<1e-4	slr1472	GO:0016787	0.1203
sll1542	GO:0046128	<1e-4	sll0596	GO:0080090	<1e-4	sll0761	GO:0009308	<1e-4	slr0388	GO:0015077	<1e-4	ssr5117	GO:0016787	0.1203
slr6065	GO:0044248	<1e-4	sll1158	GO:0050896	<1e-4	ssl7039	GO:0009892	<1e-4	sll0847	GO:0022857	<1e-4	sll2015	GO:0016787	0.1203
slr6100	GO:0006766	<1e-4	ssr2975	GO:0071841	<1e-4	sll0658	GO:0033013	<1e-4	slr0869	GO:0022838	<1e-4	slr1095	GO:0016787	0.1203
sll1373	GO:0071842	<1e-4	slr1241	GO:0009141	<1e-4	sll0864	GO:0044248	<1e-4	slr1565	GO:0015075	<1e-4	slr1927	GO:0016787	0.1203
slr0392	GO:0048522	<1e-4	ssr2802	GO:0072524	<1e-4	sll0793	GO:0044260	<1e-4	sll7006	GO:0016817	<1e-4	sll0063	GO:0016787	0.1203
sll0242	GO:0019752	<1e-4	sll0188	GO:0006091	<1e-4	slr1301	GO:0006631	<1e-4	ssr2615	GO:0015399	<1e-4	ssr3410	GO:0016787	0.1203
slr1530	GO:0006576	<1e-4	slr0806	GO:0050794	<1e-4	slr0545	GO:0045184	<1e-4	sll0781	GO:0022892	<1e-4	slr1470	GO:0016787	0.1203
sll0658	GO:0009123	<1e-4	slr0392	GO:0051186	<1e-4	slr0872	GO:0019222	<1e-4	sll1858	GO:0015267	<1e-4	sll7047	GO:0016787	0.1203
ssl1464	GO:0006753	<1e-4	ssl2069	GO:0046394	<1e-4	slr0613	GO:0044255	<1e-4	slr1069	GO:0022891	<1e-4	ssl8003	GO:0016787	0.1203
sll0218	GO:0006811	<1e-4	ssr3570	GO:0009059	<1e-4	slr1261	GO:0046483	<1e-4	slr0890	GO:0015399	<1e-4	slr1069	GO:0016787	0.1203
slr6045	GO:0071841	<1e-4	sll0710	GO:0009394	<1e-4	sll1573	GO:0033013	<1e-4	sll1306	GO:0005342	<1e-4	slr6065	GO:0016787	0.1203
slr6039	GO:0019222	<1e-4	slr1365	GO:0051188	<1e-4	slr0742	GO:0051716	<1e-4	slr0108	GO:0016741	<1e-4	slr1261	GO:0016787	0.1203
sll0268	GO:0018130	<1e-4	ssr7084	GO:0051246	<1e-4	slr7098	GO:0044281	<1e-4	slr1907	GO:0015399	<1e-4	ssr6085	GO:0016787	0.1203
ssr3532	GO:0006163	<1e-4	sll1769	GO:0009117	<1e-4	slr7094	GO:0034654	<1e-4	sll0066	GO:0022836	<1e-4	sll8004	GO:0016787	0.1203
slr1188	GO:0051188	<1e-4	slr0521	GO:0050801	<1e-4	slr0596	GO:0006721	<1e-4	slr0264	GO:0016462	<1e-4	sll1638	GO:0016787	0.1203
slr0668	GO:0042180	<1e-4	slr1290	GO:0006631	<1e-4	slr1657	GO:0006753	<1e-4	slr0271	GO:0016817	<1e-4	slr6013	GO:0016787	0.1203
slr0157	GO:0051246	<1e-4	ssr2972	GO:0009199	<1e-4	slr0650	GO:0044106	<1e-4	ssr0332	GO:0015405	<1e-4	sll0742	GO:0016787	0.1203

sll0847	GO:0071554	<1e-4	slr2052	GO:0042455	<1e-4	sll0294	GO:0050801	<1e-4	sll0871	GO:0015399	<1e-4	sll0397	GO:0016787	0.1203
slr0959	GO:0051171	<1e-4	ssr1425	GO:0009308	<1e-4	slr6073	GO:0046128	<1e-4	slr1774	GO:0015399	<1e-4	slr1097	GO:0016787	0.1203
sll0085	GO:0006576	<1e-4	slr1025	GO:0065007	<1e-4	sll5128	GO:0048519	<1e-4	slr1262	GO:0008171	<1e-4	slr1179	GO:0016787	0.1203
sll1251	GO:0006720	<1e-4	sll0382	GO:0016052	<1e-4	sll0498	GO:0016053	<1e-4	sll1130	GO:0016462	<1e-4	sll7066	GO:0016787	0.1203
sll8035	GO:0044271	<1e-4	slr1977	GO:0009262	<1e-4	sll1715	GO:0051234	<1e-4	sll1071	GO:0022803	<1e-4	slr7097	GO:0016787	0.1203
sll0565	GO:0006066	<1e-4	ssl2920	GO:0048523	<1e-4	slr2119	GO:0009308	<1e-4	ssr1951	GO:0005342	<1e-4	sll0658	GO:0016787	0.1203
sll0192	GO:0042451	<1e-4	sll1563	GO:0006220	<1e-4	sll0272	GO:0031323	<1e-4	slr0588	GO:0015075	<1e-4	slr1068	GO:0016787	0.1203
sll0443	GO:0072521	<1e-4	slr1628	GO:0009309	<1e-4	slr2012	GO:0050801	<1e-4	slr0291	GO:0008171	<1e-4	slr1956	GO:0016787	0.1203
sll1505	GO:0048523	<1e-4	sll0487	GO:0022411	<1e-4	slr0712	GO:0006091	<1e-4	slr0111	GO:0022892	<1e-4	sll1273	GO:0016787	0.1203
slr0964	GO:0051186	<1e-4	slr6075	GO:0016054	<1e-4	sll0085	GO:0051246	<1e-4	sll5090	GO:0015291	<1e-4	sll0597	GO:0016787	0.1203
sll6055	GO:0006091	<1e-4	slr0589	GO:0006733	<1e-4	slr1169	GO:0009117	<1e-4	sll0656	GO:0022832	<1e-4	slr0151	GO:0016787	0.1203
slr0333	GO:0051347	<1e-4	slr0689	GO:0044281	<1e-4	slr1394	GO:0006793	<1e-4	slr1816	GO:0015405	<1e-4	slr1223	GO:0016787	0.1203
ssr2711	GO:0009263	<1e-4	slr1260	GO:0016052	<1e-4	slr0144	GO:0043436	<1e-4	slr1391	GO:0016462	<1e-4	slr0581	GO:0016787	0.1203
slr0207	GO:0072524	<1e-4	sll1504	GO:0044248	<1e-4	sll1071	GO:0009260	<1e-4	sll8019	GO:0015267	<1e-4	ssr7072	GO:0016787	0.1203
sll0815	GO:0072527	<1e-4	slr0890	GO:0051818	<1e-4	sll1784	GO:0044106	<1e-4	slr0482	GO:0042623	<1e-4	slr1768	GO:0016787	0.1203
slr1240	GO:0046164	<1e-4	sll1608	GO:0034641	<1e-4	slr2048	GO:0043170	<1e-4	sll0606	GO:0003674	<1e-4	slr0356	GO:0016787	0.1203
ssl0312	GO:0009309	<1e-4	sll1251	GO:0052111	<1e-4	slr0456	GO:0016053	<1e-4	sll1563	GO:0022838	<1e-4	sll1691	GO:0016787	0.1203
ssr2142	GO:0010556	<1e-4	ssr6020	GO:0006811	<1e-4	sll0505	GO:0071496	<1e-4	slr6087	GO:0016818	<1e-4	sll1306	GO:0016787	0.1203
sll0872	GO:0016052	<1e-4	sll0602	GO:0009987	<1e-4	sll0160	GO:0044106	<1e-4	slr6091	GO:0016462	<1e-4	sll2013	GO:0016787	0.1203
sll1926	GO:0051171	<1e-4	slr0730	GO:0044260	<1e-4	slr1628	GO:0046394	<1e-4	sll0815	GO:0022890	<1e-4	ssr3304	GO:0016787	0.1203
sll1372	GO:0009199	<1e-4	sll1378	GO:0009056	<1e-4	sll0702	GO:0050896	<1e-4	sll0282	GO:0022891	<1e-4	sll0609	GO:0016787	0.1203
slr1033	GO:0051188	<1e-4	ssl2162	GO:0055114	<1e-4	slr0404	GO:0009132	<1e-4	sll1191	GO:0022892	<1e-4	sll0586	GO:0005215	0.1202
sll0984	GO:0055082	<1e-4	sll0702	GO:0009889	<1e-4	slr0592	GO:0044260	<1e-4	sll0436	GO:0060089	<1e-4	slr0978	GO:0005215	0.1202
sll1131	GO:0009165	<1e-4	slr1117	GO:0009123	<1e-4	sll1163	GO:0033013	<1e-4	ssl2384	GO:0016462	<1e-4	slr0594	GO:0005215	0.1202
slr1161	GO:0018130	<1e-4	slr7011	GO:0008610	<1e-4	sll2015	GO:0031326	<1e-4	sll0740	GO:0003674	<1e-4	ssl0353	GO:0005215	0.1202
sll1461	GO:0009144	<1e-4	ssl0242	GO:0052111	<1e-4	slr0971	GO:0044281	<1e-4	ssl0739	GO:0022836	<1e-4	slr0923	GO:0005215	0.1202
slr0655	GO:0009893	<1e-4	ssr0657	GO:0009893	<1e-4	slr1236	GO:0071496	<1e-4	slr6104	GO:0015075	<1e-4	sll0558	GO:0005215	0.1202
sll5130	GO:0071840	<1e-4	slr1436	GO:0009259	<1e-4	sll5109	GO:0009309	<1e-4	ssr3304	GO:0015405	<1e-4	slr0589	GO:0005215	0.1202
sll1068	GO:0043436	<1e-4	sll0864	GO:0009059	<1e-4	slr0872	GO:0071554	<1e-4	ssr2049	GO:0015267	<1e-4	slr1047	GO:0005215	0.1202
ssl0352	GO:0050794	<1e-4	ssr0692	GO:0009991	<1e-4	ssr3409	GO:0016054	<1e-4	slr0589	GO:0016462	<1e-4	sll0822	GO:0005215	0.1202
ssr3129	GO:0051186	<1e-4	slr0962	GO:0009059	<1e-4	sll1834	GO:0009057	<1e-4	slr6045	GO:0016462	<1e-4	slr1098	GO:0005215	0.1202
sll0183	GO:0018130	<1e-4	ssr2972	GO:0071841	<1e-4	sll0623	GO:0019222	<1e-4	slr1362	GO:0016818	<1e-4	slr0609	GO:0005215	0.1202
ssl7048	GO:0034654	<1e-4	sll1306	GO:0006082	<1e-4	sll0443	GO:0072527	<1e-4	sll0588	GO:0022832	<1e-4	sll0022	GO:0005215	0.1202
ssr1256	GO:0046483	<1e-4	slr0151	GO:0006732	<1e-4	slr1601	GO:0034641	<1e-4	slr0318	GO:0015075	<1e-4	sll0783	GO:0005215	0.1202
slr1519	GO:0006721	<1e-4	slr1668	GO:0009308	<1e-4	ssl5114	GO:0043648	<1e-4	slr0082	GO:0015291	<1e-4	slr0740	GO:0005215	0.1202
slr0976	GO:0006811	<1e-4	sll1511	GO:0022607	<1e-4	sll1469	GO:0009126	<1e-4	ssl0467	GO:0016741	<1e-4	sll1751	GO:0005215	0.1202
sll0446	GO:0043170	<1e-4	sll7031	GO:0044248	<1e-4	sll0843	GO:0006091	<1e-4	ssl5095	GO:0005342	<1e-4	sll0505	GO:0005215	0.1202
sll0524	GO:0072521	<1e-4	sll7050	GO:0009889	<1e-4	slr7102	GO:0009123	<1e-4	sll0564	GO:0015293	<1e-4	ssl1377	GO:0005215	0.1202
sll0456	GO:0009260	<1e-4	sll1764	GO:0019752	<1e-4	sll0925	GO:0044283	<1e-4	slr1474	GO:0042623	<1e-4	sll1858	GO:0005215	0.1195
sll1902	GO:0006082	<1e-4	slr0325	GO:0034641	<1e-4	sll8033	GO:0019752	<1e-4	sll0446	GO:0015399	<1e-4	sll1737	GO:0005215	0.1195
ssl2595	GO:0051347	<1e-4	slr0848	GO:0051347	<1e-4	slr0651	GO:0042180	<1e-4	ssl0353	GO:0022832	<1e-4	slr1702	GO:0005215	0.1195
sll1866	GO:0044255	<1e-4	sll0524	GO:0048523	<1e-4	ssr6020	GO:0065007	<1e-4	ssl2717	GO:0015293	<1e-4	slr1603	GO:0005215	0.1195
slr6014	GO:0009262	<1e-4	slr2071	GO:0072524	<1e-4	slr1537	GO:0019752	<1e-4	ssr2611	GO:0005342	<1e-4	slr1385	GO:0005215	0.1195
ssr0336	GO:0051188	<1e-4	slr1827	GO:0006720	<1e-4	slr0397	GO:0006163	<1e-4	sll1251	GO:0043492	<1e-4	slr0586	GO:0003676	0.117
slr0913	GO:0044260	<1e-4	sll0103	GO:0055086	<1e-4	sll1186	GO:0042451	<1e-4	slr1210	GO:0022803	<1e-4	sll1006	GO:0003676	0.117
slr0058	GO:0043436	<1e-4	sll1797	GO:0009259	<1e-4	slr6067	GO:0071496	<1e-4	slr2144	GO:0043167	<1e-4	slr1230	GO:0003676	0.117
sll8019	GO:0044282	<1e-4	sll1142	GO:0009987	<1e-4	sll0688	GO:0008150	<1e-4	sll1380	GO:0015399	<1e-4	sll1002	GO:0003676	0.117
sll0547	GO:0046128	<1e-4	slr1907	GO:0055082	<1e-4	slr1505	GO:0048878	<1e-4	slr0060	GO:0015077	<1e-4	sll1835	GO:0003676	0.117
slr0458	GO:0051186	<1e-4	slr1541	GO:0043170	<1e-4	ssl5091	GO:0009260	<1e-4	slr1624	GO:0005342	<1e-4	ssr2439	GO:0003676	0.117
sll0162	GO:0009991	<1e-4	sll0676	GO:0034660	<1e-4	slr6049	GO:0052188	<1e-4	slr0195	GO:0016462	<1e-4	slr1573	GO:0003676	0.117
ssr0536	GO:0009165	<1e-4	sll0294	GO:0043436	<1e-4	sll7078	GO:0009262	<1e-4	slr1660	GO:0022838	<1e-4	sll0456	GO:0003676	0.117
slr1827	GO:0009150	<1e-4	sll1378	GO:0051171	<1e-4	sll0198	GO:0019219	<1e-4	sll1163	GO:0015405	<1e-4	sll0314	GO:0003676	0.117
sll0647	GO:0031323	<1e-4	slr0272	GO:0022607	<1e-4	slr0619	GO:0006793	<1e-4	ssl0739	GO:0015077	<1e-4	slr1073	GO:0003676	0.117
slr0458	GO:0019637	<1e-4	sll1400	GO:0009150	<1e-4	sll1240	GO:0071840	<1e-4	sll0678	GO:0022803	<1e-4	slr0404	GO:0003676	0.117

sll1611	GO:0009056	<1e-4	sll1164	GO:0031640	<1e-4	ssr1375	GO:0019637	<1e-4	sll0658	GO:0043167	<1e-4	slr0872	GO:0003676	0.117
slr1258	GO:0044282	<1e-4	sll1608	GO:0050794	<1e-4	sll1433	GO:0019222	<1e-4	sll0419	GO:0022857	<1e-4	sll0785	GO:0003676	0.117
slr0232	GO:0080090	<1e-4	slr5023	GO:0044282	<1e-4	slr0514	GO:0051246	<1e-4	sll1658	GO:0016817	<1e-4	slr0023	GO:0003676	0.117
slr1591	GO:0048518	<1e-4	slr7025	GO:0044281	<1e-4	slr0285	GO:0034654	<1e-4	sll0327	GO:0015075	<1e-4	slr1964	GO:0003676	0.117
sll0103	GO:0034641	<1e-4	sll0931	GO:0019752	<1e-4	sll1265	GO:0006721	<1e-4	slr7059	GO:0022892	<1e-4	slr0770	GO:0003676	0.117
slr1566	GO:0072527	<1e-4	slr0634	GO:0051179	<1e-4	ssr6019	GO:0006721	<1e-4	slr1396	GO:0016746	<1e-4	slr1210	GO:0003676	0.117
slr0092	GO:0009161	<1e-4	sll0174	GO:0072528	<1e-4	ssl2814	GO:0044260	<1e-4	ssr2551	GO:0015291	<1e-4	sll0414	GO:0003676	0.117
slr0865	GO:0046164	<1e-4	sll1541	GO:0044283	<1e-4	slr0723	GO:0009263	<1e-4	ssl0483	GO:0016818	<1e-4	slr1593	GO:0003676	0.117
slr1303	GO:0006163	<1e-4	sll1675	GO:0009991	<1e-4	ssr1499	GO:0006066	<1e-4	ssr3189	GO:0005342	<1e-4	slr0144	GO:0003676	0.117
ssr0336	GO:0016053	<1e-4	slr1177	GO:0019362	<1e-4	slr1658	GO:0031323	<1e-4	slr0148	GO:0016462	<1e-4	ssl0352	GO:0003676	0.117
sll1702	GO:0045184	<1e-4	slr5077	GO:0009309	<1e-4	sll1784	GO:0009161	<1e-4	sll0478	GO:0022891	<1e-4	slr0723	GO:0003676	0.117
slr2084	GO:0048518	<1e-4	sll1634	GO:0080090	<1e-4	sll0762	GO:0090304	<1e-4	slr1342	GO:0015405	<1e-4	ssl0242	GO:0003676	0.117
slr2103	GO:0046907	<1e-4	slr0207	GO:0080090	<1e-4	ssr2317	GO:0044255	<1e-4	ssr3467	GO:0015267	<1e-4	sll1262	GO:0003676	0.117
slr1287	GO:0071554	<1e-4	slr1814	GO:0009262	<1e-4	slr0519	GO:0006631	<1e-4	slr1778	GO:0042623	<1e-4	sll0756	GO:0003676	0.117
slr1614	GO:0006811	<1e-4	ssl2384	GO:0006721	<1e-4	slr0491	GO:0009059	<1e-4	sll0444	GO:0016462	<1e-4	sll1613	GO:0003676	0.117
slr1990	GO:0008150	<1e-4	slr0109	GO:0009892	<1e-4	slr7080	GO:0009056	<1e-4	sll0419	GO:0015077	<1e-4	slr1676	GO:0003676	0.117
slr0725	GO:0051818	<1e-4	slr0053	GO:0044283	<1e-4	slr7098	GO:0009259	<1e-4	sll0414	GO:0015291	<1e-4	slr1437	GO:0003676	0.117
sll1060	GO:0050789	<1e-4	slr0770	GO:0051818	<1e-4	slr1047	GO:0043648	<1e-4	slr1383	GO:0022803	<1e-4	slr1084	GO:0003676	0.117
sll0595	GO:0009057	<1e-4	slr6028	GO:0009893	<1e-4	sll5090	GO:0044281	<1e-4	slr0619	GO:0015075	<1e-4	ssr2787	GO:0003676	0.117
sll1396	GO:0046164	<1e-4	slr1557	GO:0010468	<1e-4	slr5112	GO:0009991	<1e-4	slr0919	GO:0015293	<1e-4	sll0645	GO:0003676	0.117
slr1045	GO:0006766	<1e-4	sll1380	GO:0046128	<1e-4	slr7012	GO:0006220	<1e-4	sll1201	GO:0016746	<1e-4	slr0482	GO:0003676	0.117
sll0414	GO:0019751	<1e-4	slr0890	GO:0009308	<1e-4	slr2071	GO:0019751	<1e-4	slr1363	GO:0008171	<1e-4	slr1638	GO:0003676	0.117
slr1726	GO:0050801	<1e-4	sll1222	GO:0006163	<1e-4	slr1915	GO:0071554	<1e-4	sll1289	GO:0015075	<1e-4	slr0408	GO:0005215	0.1167
slr1612	GO:0009987	<1e-4	slr0455	GO:0044260	<1e-4	ssr6026	GO:0009056	<1e-4	sll0532	GO:0015291	<1e-4	sll1250	GO:0005215	0.1167
sml0011	GO:0009892	<1e-4	slr1767	GO:0009309	<1e-4	slr6013	GO:0022607	<1e-4	slr0404	GO:0022832	<1e-4	ssr1258	GO:0005215	0.1167
sll0102	GO:0009150	<1e-4	sll1132	GO:0044283	<1e-4	slr1851	GO:0006220	<1e-4	sll1166	GO:0042623	<1e-4	slr1565	GO:0005215	0.1167
slr2073	GO:0045184	<1e-4	sll0905	GO:0019219	<1e-4	slr0863	GO:0072528	<1e-4	ssl1690	GO:0022891	<1e-4	sll1912	GO:0005215	0.1167
ssr3572	GO:0006066	<1e-4	ssr0536	GO:0044255	<1e-4	sll1775	GO:0044275	<1e-4	slr2038	GO:0022891	<1e-4	slr0919	GO:0005215	0.1167
slr0598	GO:0071841	<1e-4	slr1103	GO:0048523	<1e-4	sll7067	GO:0019222	<1e-4	sll5006	GO:0043167	<1e-4	sll0272	GO:0005215	0.1167
sll0871	GO:0009987	<1e-4	sll1785	GO:0050794	<1e-4	slr1438	GO:0051716	<1e-4	sll0980	GO:0016817	<1e-4	slr1774	GO:0005215	0.1163
sll1461	GO:0019222	<1e-4	slr0299	GO:0031640	<1e-4	sll0069	GO:0009059	<1e-4	slr0489	GO:0008171	<1e-4	sll1528	GO:0005215	0.1163
slr0482	GO:0046128	<1e-4	sll1186	GO:0044281	<1e-4	slr5013	GO:0042180	<1e-4	ssr7093	GO:0008171	<1e-4	sll1222	GO:0005215	0.1163
sll1155	GO:0031323	<1e-4	slr0689	GO:0052188	<1e-4	slr6081	GO:0046483	<1e-4	ssl6061	GO:0022838	<1e-4	slr0147	GO:0005215	0.1163
slr1789	GO:0043436	<1e-4	slr1431	GO:0051171	<1e-4	slr0393	GO:0009308	<1e-4	sll0447	GO:0022803	<1e-4	slr0642	GO:0005215	0.1163
sll1926	GO:0044281	<1e-4	slr0869	GO:0072521	<1e-4	sll7062	GO:0034641	<1e-4	slr1773	GO:0015291	<1e-4	slr0818	GO:0005215	0.1163
slr6015	GO:0009141	<1e-4	slr1342	GO:0044283	<1e-4	slr0708	GO:0006811	<1e-4	sll1882	GO:0015405	<1e-4	sll1995	GO:0005215	0.1163
slr1376	GO:0051347	<1e-4	slr1495	GO:0016054	<1e-4	ssl3451	GO:0009991	<1e-4	slr6063	GO:0043167	<1e-4	sll1272	GO:0005215	0.1163
ssr2912	GO:0009117	<1e-4	slr0519	GO:0009123	<1e-4	slr8014	GO:0009394	<1e-4	sll0843	GO:0015405	<1e-4	slr0765	GO:0005215	0.1163
slr0480	GO:0009142	<1e-4	sll0639	GO:0080090	<1e-4	slr6045	GO:0034660	<1e-4	ssr5092	GO:0022836	<1e-4	ssr0692	GO:0005215	0.1163
slr0250	GO:0071841	<1e-4	slr1863	GO:0009259	<1e-4	slr1398	GO:0071496	<1e-4	slr1450	GO:0043492	<1e-4	slr5126	GO:0006810	0.1161
sll0249	GO:0006091	<1e-4	slr0249	GO:0071840	<1e-4	slr0192	GO:0044248	<1e-4	ssr2201	GO:0022803	<1e-4	ssr3572	GO:0006810	0.1161
sll1089	GO:0034641	<1e-4	ssr5121	GO:0006631	<1e-4	ssl2733	GO:0051186	<1e-4	slr0742	GO:0016817	<1e-4	sll6055	GO:0006810	0.1161
sll0405	GO:0009059	<1e-4	ssl0410	GO:0048523	<1e-4	slr0921	GO:0072524	<1e-4	sll0499	GO:0003674	<1e-4	ssr6019	GO:0006810	0.1161
sll0740	GO:0050896	<1e-4	sll0787	GO:0006220	<1e-4	sll1532	GO:0009126	<1e-4	slr1623	GO:0022832	<1e-4	slr1752	GO:0006810	0.1161
sll1949	GO:0051186	<1e-4	slr6049	GO:0009056	<1e-4	sll1925	GO:0050789	<1e-4	slr0876	GO:0016741	<1e-4	ssl6061	GO:0006810	0.1161
slr1813	GO:0022411	<1e-4	sll5003	GO:0006766	<1e-4	sll1318	GO:0046164	<1e-4	sll1040	GO:0043167	<1e-4	slr5073	GO:0006810	0.1161
sll5006	GO:0042455	<1e-4	sll1164	GO:0048518	<1e-4	slr0980	GO:0055114	<1e-4	sll0168	GO:0016741	<1e-4	sll1164	GO:0006810	0.1161
sll0473	GO:0072528	<1e-4	slr1117	GO:0016054	<1e-4	slr1726	GO:0060255	<1e-4	slr1896	GO:0016818	<1e-4	slr0967	GO:0006810	0.1161
sll0762	GO:0006066	<1e-4	slr2000	GO:0090304	<1e-4	slr2118	GO:0071840	<1e-4	sll1321	GO:0043167	<1e-4	sll1891	GO:0006810	0.1161
slr2111	GO:0019362	<1e-4	ssr0761	GO:0044282	<1e-4	sll1217	GO:0022411	<1e-4	sll0424	GO:0015405	<1e-4	slr2070	GO:0006810	0.1161
ssr3341	GO:0050789	<1e-4	sll1659	GO:0050794	<1e-4	ssl2420	GO:0022607	<1e-4	slr1940	GO:0015399	<1e-4	ssr5074	GO:0006810	0.1161
sll7089	GO:0009987	<1e-4	sll1934	GO:0044271	<1e-4	sll0630	GO:0006753	<1e-4	sll1532	GO:0043492	<1e-4	sll0372	GO:0006810	0.1161
slr0496	GO:0009889	<1e-4	sll0350	GO:0019222	<1e-4	slr1383	GO:0019219	<1e-4	slr7102	GO:0016462	<1e-4	ssl7039	GO:0006810	0.1161
sll0811	GO:0009144	<1e-4	ssl8028	GO:0031326	<1e-4	ssl5031	GO:0009991	<1e-4	slr0243	GO:0022838	<1e-4	slr0238	GO:0006810	0.1161

ssl1577	GO:0055082	<1e-4	slr0516	GO:0044106	<1e-4	sll1340	GO:0006066	<1e-4	sll0293	GO:0022803	<1e-4	slr0948	GO:0006810	0.1161
sll1272	GO:0008610	<1e-4	sll0446	GO:0044271	<1e-4	slr1045	GO:0006811	<1e-4	slr0888	GO:0005342	<1e-4	sll7033	GO:0006810	0.1161
sll1942	GO:0009056	<1e-4	ssr3154	GO:0048878	<1e-4	ssl7039	GO:0071496	<1e-4	slr0656	GO:0022836	<1e-4	sll1583	GO:0006810	0.1161
slr2111	GO:0048523	<1e-4	slr0871	GO:0019752	<1e-4	sll0980	GO:0071841	<1e-4	sll1526	GO:0022891	<1e-4	sll0608	GO:0006810	0.1161
sll1766	GO:0051246	<1e-4	slr1097	GO:0006733	<1e-4	sll1853	GO:0009126	<1e-4	slr0699	GO:0005342	<1e-4	sll1036	GO:0006810	0.1161
sll0482	GO:0009308	<1e-4	slr2012	GO:0009144	<1e-4	sll7067	GO:0060255	<1e-4	sll0488	GO:0022891	<1e-4	sll0905	GO:0006810	0.1161
slr0569	GO:0009199	<1e-4	slr0294	GO:0006576	<1e-4	slr0407	GO:0009987	<1e-4	slr1767	GO:0043167	<1e-4	sll0479	GO:0006810	0.1161
sll0513	GO:0019637	<1e-4	sll0647	GO:0009124	<1e-4	slr0232	GO:0006721	<1e-4	ssl5098	GO:0022890	<1e-4	sll0298	GO:0006810	0.1161
sll1925	GO:0009141	<1e-4	sll1024	GO:0006082	<1e-4	sll1396	GO:0046907	<1e-4	sll1025	GO:0003674	<1e-4	sll1158	GO:0006810	0.1161
sll1652	GO:0006721	<1e-4	sll0301	GO:0051716	<1e-4	slr0680	GO:0006732	<1e-4	sll1527	GO:0022891	<1e-4	slr1577	GO:0006810	0.1161
ssl1046	GO:0006811	<1e-4	sll1863	GO:0008150	<1e-4	slr0709	GO:0009150	<1e-4	ssr2781	GO:0043167	<1e-4	sll1495	GO:0006810	0.1161
slr6063	GO:0006220	<1e-4	slr0545	GO:0019222	<1e-4	sll0558	GO:0009141	<1e-4	ssl5095	GO:0022890	<1e-4	slr6106	GO:0006810	0.1161
slr7012	GO:0009056	<1e-4	slr7083	GO:0009308	<1e-4	slr0038	GO:0052188	<1e-4	slr1676	GO:0022890	<1e-4	sll0062	GO:0006810	0.1161
slr1814	GO:0006733	<1e-4	slr0491	GO:0071842	<1e-4	ssr6019	GO:0055114	<1e-4	slr0509	GO:0022803	<1e-4	sll1785	GO:0006810	0.1161
sll5097	GO:0090304	<1e-4	slr0863	GO:0006576	<1e-4	ssr0335	GO:0046483	<1e-4	slr0579	GO:0015405	<1e-4	slr1951	GO:0006810	0.1161
ssl1417	GO:0010556	<1e-4	slr0962	GO:0042451	<1e-4	slr1087	GO:0019219	<1e-4	ssr6062	GO:0015077	<1e-4	slr0575	GO:0006810	0.1161
sll1573	GO:0005996	<1e-4	sll1530	GO:0009394	<1e-4	sll1832	GO:0043436	<1e-4	slr6021	GO:0022857	<1e-4	ssl1300	GO:0006810	0.1161
sll1495	GO:0051246	<1e-4	slr1964	GO:0019219	<1e-4	slr0579	GO:0060255	<1e-4	slr1082	GO:0043492	<1e-4	slr1394	GO:0006810	0.1161
ssr1766	GO:0045184	<1e-4	slr2125	GO:0019222	<1e-4	slr7100	GO:0044281	<1e-4	sll1906	GO:0022857	<1e-4	ssl5100	GO:0006810	0.1161
slr0380	GO:0046128	<1e-4	sll1396	GO:0006066	<1e-4	slr1406	GO:0046128	<1e-4	slr1074	GO:0015077	<1e-4	slr0708	GO:0006810	0.1161
sll1925	GO:0009132	<1e-4	slr1437	GO:0006576	<1e-4	sll1934	GO:0006811	<1e-4	ssr7017	GO:0016817	<1e-4	sll0543	GO:0006810	0.1161
sll0997	GO:0034641	<1e-4	sll7086	GO:0006753	<1e-4	slr8044	GO:0016053	<1e-4	sll1022	GO:0022892	<1e-4	slr1513	GO:0006810	0.1161
sll1939	GO:0080090	<1e-4	slr0818	GO:0080090	<1e-4	slr7016	GO:0052188	<1e-4	slr0300	GO:0043492	<1e-4	sll5089	GO:0006810	0.1161
slr1468	GO:0006091	<1e-4	ssl1464	GO:0009141	<1e-4	slr1391	GO:0031640	<1e-4	slr1866	GO:0060089	<1e-4	ssr6083	GO:0006810	0.1161
slr6064	GO:0080090	<1e-4	ssl0242	GO:0009308	<1e-4	sll1702	GO:0055086	<1e-4	slr0358	GO:0005342	<1e-4	ssr2553	GO:0006810	0.1161
slr0601	GO:0005996	<1e-4	sll0310	GO:0009132	<1e-4	slr1396	GO:0072524	<1e-4	slr7080	GO:0022890	<1e-4	sll7087	GO:0006810	0.1161
sll1656	GO:0051188	<1e-4	slr0978	GO:0006811	<1e-4	slr0545	GO:0043436	<1e-4	slr1852	GO:0022836	<1e-4	sll5030	GO:0006810	0.1161
ssr8047	GO:0046128	<1e-4	sll7067	GO:0009987	<1e-4	sll0710	GO:0050801	<1e-4	slr0440	GO:0022890	<1e-4	ssr7079	GO:0006810	0.1161
sll0558	GO:0006733	<1e-4	sll0756	GO:0043648	<1e-4	sll1321	GO:0065007	<1e-4	sll1307	GO:0015291	<1e-4	slr0184	GO:0006810	0.1161
slr1095	GO:0071840	<1e-4	sll1656	GO:0034641	<1e-4	slr6031	GO:0006811	<1e-4	slr6016	GO:0016462	<1e-4	slr6088	GO:0006810	0.1161
sll0586	GO:0051716	<1e-4	slr1999	GO:0034641	<1e-4	sll1736	GO:0009308	<1e-4	slr1339	GO:0022890	<1e-4	sll1052	GO:0006810	0.1161
sll0414	GO:0001932	<1e-4	sll1640	GO:0043933	<1e-4	sll1942	GO:0072524	<1e-4	slr0848	GO:0015075	<1e-4	slr1816	GO:0006810	0.1161
sll1763	GO:0019752	<1e-4	sll7033	GO:0009124	<1e-4	slr0725	GO:0051179	<1e-4	slr0712	GO:0015075	<1e-4	slr1915	GO:0006810	0.1161
slr0147	GO:0033013	<1e-4	sll1071	GO:0044106	<1e-4	slr1799	GO:0046394	<1e-4	slr7080	GO:0022803	<1e-4	ssr2060	GO:0006810	0.1161
sll1640	GO:0052111	<1e-4	slr1315	GO:0009059	<1e-4	slr0740	GO:0009262	<1e-4	sll0174	GO:0016817	<1e-4	slr0914	GO:0006810	0.1161
slr5118	GO:0050789	<1e-4	slr0625	GO:0051716	<1e-4	sll1882	GO:0016053	<1e-4	sll0611	GO:0022892	<1e-4	slr7099	GO:0006810	0.1161
slr0619	GO:0050896	<1e-4	sll1934	GO:0006091	<1e-4	ssl2733	GO:0034660	<1e-4	sll5003	GO:0022890	<1e-4	ssl0410	GO:0006810	0.1161
slr6100	GO:0051347	<1e-4	slr0935	GO:0006220	<1e-4	sll1873	GO:0072528	<1e-4	sll1273	GO:0005342	<1e-4	ssr8047	GO:0006810	0.1161
slr0582	GO:0009199	<1e-4	slr7059	GO:0009059	<1e-4	ssl2138	GO:0042180	<1e-4	ssl5103	GO:0015267	<1e-4	sll1191	GO:0006810	0.1161
slr6065	GO:0044275	<1e-4	sll1830	GO:0051234	<1e-4	slr0168	GO:0006720	<1e-4	slr7013	GO:0022890	<1e-4	slr0313	GO:0006810	0.1161
slr0914	GO:0055114	<1e-4	slr1667	GO:0010556	<1e-4	slr1045	GO:0046907	<1e-4	slr0039	GO:0005342	<1e-4	sll0691	GO:0006810	0.1161
sll0022	GO:0071842	<1e-4	slr1568	GO:0043170	<1e-4	slr7080	GO:0009263	<1e-4	sll1442	GO:0005342	<1e-4	sll0552	GO:0006810	0.1161
slr0516	GO:0043933	<1e-4	slr1670	GO:0009144	<1e-4	ssr6020	GO:0009263	<1e-4	slr5023	GO:0015405	<1e-4	slr0013	GO:0006810	0.1161
sll1542	GO:0051188	<1e-4	slr0594	GO:0072528	<1e-4	sll1675	GO:0031640	<1e-4	slr1699	GO:0022892	<1e-4	ssr1425	GO:0006810	0.1161
ssl3829	GO:0044271	<1e-4	slr2000	GO:0031323	<1e-4	slr1142	GO:0034654	<1e-4	slr7013	GO:0022891	<1e-4	sll1652	GO:0006810	0.1161
sll5026	GO:0050801	<1e-4	slr1648	GO:0006793	<1e-4	sll1736	GO:0022411	<1e-4	slr6015	GO:0015405	<1e-4	slr1442	GO:0006810	0.1161
slr1095	GO:0009056	<1e-4	slr0780	GO:0048878	<1e-4	sll1656	GO:0052111	<1e-4	sll1162	GO:0015405	<1e-4	sll8019	GO:0006810	0.1161
slr0845	GO:0051179	<1e-4	slr5077	GO:0044260	<1e-4	slr5118	GO:0051171	<1e-4	slr0489	GO:0043492	<1e-4	slr0590	GO:0006810	0.1161
sll0761	GO:0051179	<1e-4	sll0553	GO:0006720	<1e-4	slr7010	GO:0006793	<1e-4	sll5033	GO:0015399	<1e-4	slr0667	GO:0006810	0.1161
sll0577	GO:0043933	<1e-4	slr2120	GO:0009308	<1e-4	slr1053	GO:0010556	<1e-4	sll1036	GO:0015267	<1e-4	slr1170	GO:0006810	0.1161
slr1599	GO:0006811	<1e-4	sll0670	GO:0046164	<1e-4	sll1201	GO:0009263	<1e-4	sll0216	GO:0022836	<1e-4	slr8022	GO:0006810	0.1161
sll0405	GO:0009056	<1e-4	ssl0739	GO:0051171	<1e-4	ssl1300	GO:0006720	<1e-4	slr1601	GO:0015291	<1e-4	slr5102	GO:0006810	0.1161
slr6033	GO:0065007	<1e-4	slr0579	GO:0034654	<1e-4	slr0581	GO:0043933	<1e-4	ssr3304	GO:0003674	<1e-4	ssr3467	GO:0006810	0.1161
sll1388	GO:0052188	<1e-4	sll0793	GO:0044271	<1e-4	sll7033	GO:0046164	<1e-4	sll0419	GO:0016746	<1e-4	sll1233	GO:0006810	0.1161

sll1222	GO:0051347	<1e-4	slr0680	GO:0044248	<1e-4	slr0263	GO:0046907	<1e-4	slr0801	GO:0016817	<1e-4	slr5013	GO:0006810	0.1161
slr1895	GO:0019752	<1e-4	slr1462	GO:0006733	<1e-4	sll1675	GO:0009141	<1e-4	slr0345	GO:0043492	<1e-4	sll1106	GO:0006810	0.1161
slr1083	GO:0009142	<1e-4	slr0453	GO:0006733	<1e-4	slr1657	GO:0022411	<1e-4	slr6080	GO:0015405	<1e-4	ssr3570	GO:0006810	0.1161
slr1195	GO:0072524	<1e-4	slr1599	GO:0048523	<1e-4	ssl1046	GO:0072527	<1e-4	slr0960	GO:0016817	<1e-4	ssl5114	GO:0006810	0.1161
sll1068	GO:0071841	<1e-4	sll1455	GO:0043648	<1e-4	slr1406	GO:0031640	<1e-4	sll0068	GO:0015291	<1e-4	sll0060	GO:0006810	0.1161
slr0876	GO:0071554	<1e-4	ssr3122	GO:0019751	<1e-4	slr1546	GO:0006091	<1e-4	slr7071	GO:0005342	<1e-4	sll1609	GO:0006810	0.1161
sll1163	GO:0045184	<1e-4	sll0188	GO:0019751	<1e-4	sll1433	GO:0006811	<1e-4	slr1353	GO:0016817	<1e-4	sll7089	GO:0006810	0.1161
sll1052	GO:0009059	<1e-4	sll1504	GO:0065007	<1e-4	slr1032	GO:0050789	<1e-4	sll0443	GO:0043492	<1e-4	slr1507	GO:0006810	0.1161
slr6090	GO:0072521	<1e-4	ssr7035	GO:0006732	<1e-4	slr1624	GO:0009141	<1e-4	slr1885	GO:0042623	<1e-4	slr0458	GO:0006810	0.1161
ssl7039	GO:0044282	<1e-4	ssr3341	GO:0016054	<1e-4	ssr5106	GO:0009260	<1e-4	sll0872	GO:0022891	<1e-4	slr0755	GO:0006810	0.1161
slr1780	GO:0055114	<1e-4	sll0175	GO:0051186	<1e-4	sll1155	GO:0051234	<1e-4	ssl7042	GO:0022857	<1e-4	slr1537	GO:0006810	0.1161
ssr2755	GO:0009150	<1e-4	sll1511	GO:0048522	<1e-4	slr0431	GO:0010556	<1e-4	sll1785	GO:0022832	<1e-4	ssl5103	GO:0006810	0.1161
slr6087	GO:0006091	<1e-4	sll0024	GO:0006793	<1e-4	sll0060	GO:0009059	<1e-4	sll1089	GO:0022892	<1e-4	ssl1918	GO:0006810	0.1161
ssr3122	GO:0009260	<1e-4	sll0428	GO:0006091	<1e-4	slr1425	GO:0042451	<1e-4	sll0980	GO:0008171	<1e-4	ssl5025	GO:0006810	0.1161
ssr6089	GO:0050789	<1e-4	sll0176	GO:0009144	<1e-4	slr0407	GO:0006720	<1e-4	ssl7045	GO:0003674	<1e-4	sll1702	GO:0006810	0.1161
slr0326	GO:0009142	<1e-4	slr1659	GO:0006631	<1e-4	slr1307	GO:0048518	<1e-4	sll1219	GO:0015399	<1e-4	ssl2138	GO:0006810	0.1161
slr1603	GO:0019219	<1e-4	ssr1407	GO:0051716	<1e-4	slr7058	GO:0009123	<1e-4	sll1638	GO:0015075	<1e-4	slr0217	GO:0006810	0.1161
ssr6086	GO:0048522	<1e-4	ssl7039	GO:0006766	<1e-4	slr0845	GO:0044003	<1e-4	slr1895	GO:0016741	<1e-4	slr7100	GO:0006810	0.1161
sll7077	GO:0046483	<1e-4	ssl2781	GO:0034660	<1e-4	slr0498	GO:0044275	<1e-4	slr1628	GO:0015399	<1e-4	slr0517	GO:0006810	0.1161
slr0023	GO:0050896	<1e-4	sll0436	GO:0055086	<1e-4	slr6066	GO:0043170	<1e-4	slr1263	GO:0022836	<1e-4	slr1163	GO:0006810	0.1161
sll0786	GO:0048523	<1e-4	sll1950	GO:0006732	<1e-4	sll0319	GO:0065007	<1e-4	sll8032	GO:0015405	<1e-4	sll7055	GO:0006810	0.1161
slr1780	GO:0016052	<1e-4	slr0204	GO:0034654	<1e-4	slr0587	GO:0051347	<1e-4	sll1239	GO:0042623	<1e-4	slr5112	GO:0006810	0.1161
slr0656	GO:0043648	<1e-4	sll0564	GO:0019752	<1e-4	sll1447	GO:0009262	<1e-4	slr1431	GO:0016829	<1e-4	sll1049	GO:0006810	0.1161
ssr0102	GO:0071554	<1e-4	slr7015	GO:0072522	<1e-4	slr1917	GO:0009056	<1e-4	ssl7021	GO:0042623	<1e-4	sll1319	GO:0006810	0.1161
slr1261	GO:0019222	<1e-4	sll0749	GO:0006220	<1e-4	slr0967	GO:0019362	<1e-4	slr0742	GO:0022891	<1e-4	slr2103	GO:0006810	0.1161
sll1380	GO:0009056	<1e-4	ssr1558	GO:0048523	<1e-4	sll0676	GO:0019222	<1e-4	sll1289	GO:0016462	<1e-4	ssr1041	GO:0006810	0.1161
slr7096	GO:0031640	<1e-4	slr0708	GO:0006732	<1e-4	sll0775	GO:0019637	<1e-4	sll0793	GO:0016741	<1e-4	ssl0350	GO:0006810	0.1161
sll1372	GO:0051234	<1e-4	ssl2471	GO:0043648	<1e-4	ssr5074	GO:0009889	<1e-4	slr7057	GO:0015399	<1e-4	slr0670	GO:0006810	0.1161
sll0047	GO:0046128	<1e-4	ssl1577	GO:0051186	<1e-4	sll0524	GO:0043648	<1e-4	sll0854	GO:0022838	<1e-4	slr5102	GO:0016491	0.116
ssr6030	GO:0005996	<1e-4	slr0489	GO:0009165	<1e-4	slr7011	GO:0050789	<1e-4	slr1624	GO:0022803	<1e-4	slr1790	GO:0016491	0.116
sll5030	GO:0034641	<1e-4	slr0144	GO:0031323	<1e-4	slr2144	GO:0072528	<1e-4	slr1025	GO:0003674	<1e-4	sll1254	GO:0016491	0.116
slr1533	GO:0006066	<1e-4	sll1873	GO:0022411	<1e-4	slr1444	GO:0006811	<1e-4	slr1895	GO:0022832	<1e-4	sll0749	GO:0016491	0.116
slr1612	GO:0009260	<1e-4	slr0238	GO:0071841	<1e-4	ssl5031	GO:0006811	<1e-4	sll0007	GO:0016462	<1e-4	ssr1558	GO:0016491	0.116
slr6028	GO:0071554	<1e-4	ssr7035	GO:0009056	<1e-4	slr0594	GO:0050794	<1e-4	sll0297	GO:0022892	<1e-4	ssl7051	GO:0016491	0.116
slr2070	GO:0046128	<1e-4	slr2121	GO:0042180	<1e-4	sll1442	GO:0009165	<1e-4	sll0518	GO:0005575	<1e-4	slr1449	GO:0016491	0.116
sll0022	GO:0051716	<1e-4	ssl8003	GO:0010468	<1e-4	sll0443	GO:0043648	<1e-4	ssl7045	GO:0044446	<1e-4	slr5087	GO:0016491	0.116
sll0238	GO:0044271	<1e-4	slr0959	GO:0046164	<1e-4	sll1726	GO:0006091	<1e-4	sll0944	GO:0044464	<1e-4	sll1380	GO:0016491	0.116
sll5089	GO:0071841	<1e-4	slr1907	GO:0006732	<1e-4	sll1262	GO:0042451	<1e-4	slr1362	GO:0044444	<1e-4	sll0162	GO:0016491	0.116
slr0169	GO:0071554	<1e-4	slr0491	GO:0031640	<1e-4	sll0639	GO:0009259	<1e-4	sll5090	GO:0044425	<1e-4	slr8021	GO:0016491	0.116
sll1640	GO:0072522	<1e-4	slr6068	GO:0006066	<1e-4	slr0269	GO:0051188	<1e-4	slr1664	GO:0005575	<1e-4	slr0039	GO:0016491	0.116
slr0392	GO:0009260	<1e-4	ssl1300	GO:0009056	<1e-4	slr2018	GO:0051171	<1e-4	sll0265	GO:0005575	<1e-4	slr5126	GO:0016491	0.116
ssl3829	GO:0050896	<1e-4	sll0503	GO:0008150	<1e-4	slr0935	GO:0046483	<1e-4	sll0359	GO:0043226	<1e-4	slr0345	GO:0016491	0.116
sll0656	GO:0051246	<1e-4	slr5012	GO:0009057	<1e-4	slr0211	GO:0034660	<1e-4	slr0374	GO:0044446	<1e-4	sll0047	GO:0016491	0.116
slr0601	GO:0045184	<1e-4	slr1436	GO:0071840	<1e-4	ssr6085	GO:0009199	<1e-4	slr1301	GO:0044425	<1e-4	slr1628	GO:0016491	0.116
ssr6024	GO:0019222	<1e-4	ssl2148	GO:0043170	<1e-4	slr0680	GO:0050789	<1e-4	sll0263	GO:0044446	<1e-4	sll0875	GO:0016491	0.116
slr7011	GO:0046164	<1e-4	slr0376	GO:0051818	<1e-4	sll1563	GO:0072528	<1e-4	sll1934	GO:0005575	<1e-4	sll0098	GO:0016491	0.116
slr1170	GO:0009262	<1e-4	slr1614	GO:0046164	<1e-4	sll1656	GO:0051716	<1e-4	sll1737	GO:0005575	<1e-4	slr0708	GO:0016491	0.116
slr0476	GO:0006082	<1e-4	sll1344	GO:0006811	<1e-4	sll1926	GO:0009987	<1e-4	slr1998	GO:0044446	<1e-4	slr1944	GO:0016491	0.116
ssr2551	GO:0044260	<1e-4	sll5090	GO:0051818	<1e-4	slr1178	GO:0042180	<1e-4	slr0023	GO:0044422	<1e-4	sll7006	GO:0016491	0.116
slr2018	GO:0016053	<1e-4	ssr1258	GO:0034641	<1e-4	slr1990	GO:0009150	<1e-4	slr0664	GO:0044446	<1e-4	slr0885	GO:0016491	0.116
slr0579	GO:0072522	<1e-4	slr0285	GO:0051171	<1e-4	slr1083	GO:0009991	<1e-4	slr1263	GO:0043226	<1e-4	ssl1046	GO:0016491	0.116
sll1766	GO:0044003	<1e-4	slr7100	GO:0006220	<1e-4	slr6100	GO:0048522	<1e-4	sll0547	GO:0044422	<1e-4	slr6009	GO:0016491	0.116
sll1306	GO:0019222	<1e-4	ssl0787	GO:0071842	<1e-4	slr1619	GO:0044255	<1e-4	sll0063	GO:0044424	<1e-4	slr1970	GO:0016491	0.116
sll0405	GO:0009161	<1e-4	slr1603	GO:0080090	<1e-4	sll0328	GO:0052188	<1e-4	slr1152	GO:0005575	<1e-4	slr1438	GO:0016491	0.116

ssl5099	GO:0016054	<1e-4	slr0168	GO:0005996	<1e-4	sll0810	GO:0055086	<1e-4	slr1638	GO:0044422	<1e-4	sll1652	GO:0016491	0.116
sll0577	GO:0048522	<1e-4	ssr2439	GO:0009892	<1e-4	slr1896	GO:0016052	<1e-4	slr0383	GO:0044424	<1e-4	ssr2998	GO:0016491	0.116
slr1444	GO:0006733	<1e-4	sll5069	GO:0019752	<1e-4	slr1436	GO:0072528	<1e-4	slr6091	GO:0005575	<1e-4	ssr5106	GO:0016491	0.116
slr0637	GO:0006576	<1e-4	sll0925	GO:0042451	<1e-4	ssl3382	GO:0019637	<1e-4	slr0709	GO:0043226	<1e-4	slr2052	GO:0016491	0.116
slr0924	GO:0044260	<1e-4	slr0195	GO:0090304	<1e-4	ssr7079	GO:0042455	<1e-4	sll1586	GO:0044446	<1e-4	ssl2069	GO:0016491	0.116
sll1188	GO:0009141	<1e-4	slr0151	GO:0071840	<1e-4	slr1927	GO:0006733	<1e-4	slr6014	GO:0044446	<1e-4	sll0765	GO:0016491	0.116
slr6090	GO:0051186	<1e-4	slr2052	GO:0034641	<1e-4	sll2011	GO:0072521	<1e-4	ssr6003	GO:0043226	<1e-4	slr0914	GO:0016491	0.116
sll0369	GO:0006733	<1e-4	slr1812	GO:0009123	<1e-4	ssr2201	GO:0060255	<1e-4	sll0072	GO:0044424	<1e-4	sll1233	GO:0016491	0.116
slr1079	GO:0051818	<1e-4	slr1363	GO:0006753	<1e-4	slr0270	GO:0045184	<1e-4	slr1799	GO:0044424	<1e-4	slr1396	GO:0016491	0.116
ssl2717	GO:0048523	<1e-4	slr1951	GO:0072521	<1e-4	slr1062	GO:0009263	<1e-4	sll0645	GO:0005575	<1e-4	ssr5121	GO:0016491	0.116
slr7059	GO:0006811	<1e-4	slr0058	GO:0048523	<1e-4	slr1378	GO:0006732	<1e-4	sll1388	GO:0044425	<1e-4	sll5063	GO:0016491	0.116
slr1704	GO:0019362	<1e-4	slr7012	GO:0009260	<1e-4	slr0172	GO:0009308	<1e-4	sll0710	GO:0044422	<1e-4	sll8019	GO:0016491	0.116
sll0183	GO:0006066	<1e-4	slr0751	GO:0051716	<1e-4	sll1219	GO:0019222	<1e-4	slr6073	GO:0044425	<1e-4	ssl3829	GO:0016491	0.116
ssr7017	GO:0048519	<1e-4	slr0740	GO:0051716	<1e-4	slr0729	GO:0090304	<1e-4	sll0237	GO:0005575	<1e-4	slr1142	GO:0016491	0.116
sll1352	GO:0048523	<1e-4	sll7069	GO:0046483	<1e-4	sll0760	GO:0006753	<1e-4	slr1034	GO:0044425	<1e-4	ssr1041	GO:0016491	0.116
slr0869	GO:0019637	<1e-4	slr1178	GO:0051716	<1e-4	ssl1378	GO:0009117	<1e-4	sll0702	GO:0044444	<1e-4	slr1577	GO:0016491	0.116
sll0913	GO:0006733	<1e-4	sll0803	GO:0071842	<1e-4	slr1259	GO:0006066	<1e-4	slr0111	GO:0043226	<1e-4	sll1486	GO:0016491	0.116
slr7097	GO:0043648	<1e-4	slr1303	GO:0006082	<1e-4	slr1799	GO:0006721	<1e-4	sll0645	GO:0044446	<1e-4	sll5003	GO:0016491	0.116
slr1667	GO:0048518	<1e-4	sll1285	GO:0044255	<1e-4	slr0625	GO:0009142	<1e-4	sll5032	GO:0044424	<1e-4	slr0575	GO:0016491	0.116
slr0625	GO:0009165	<1e-4	sll1378	GO:0044255	<1e-4	slr0217	GO:0051818	<1e-4	sll1950	GO:0044422	<1e-4	sll5132	GO:0016491	0.116
sll0298	GO:0009893	<1e-4	slr1263	GO:0044271	<1e-4	slr1537	GO:0006766	<1e-4	sll1965	GO:0005575	<1e-4	sll1532	GO:0016491	0.116
sll0168	GO:0072528	<1e-4	sll1528	GO:0051716	<1e-4	sll0703	GO:0080090	<1e-4	ssr2962	GO:0043226	<1e-4	sll0563	GO:0016491	0.116
ssl1263	GO:0009893	<1e-4	slr2060	GO:0006766	<1e-4	sll0301	GO:0072522	<1e-4	slr0388	GO:0043226	<1e-4	slr1101	GO:0016491	0.116
slr2118	GO:0006721	<1e-4	ssl5098	GO:0009059	<1e-4	sll0611	GO:0019637	<1e-4	slr1679	GO:0044446	<1e-4	sll8012	GO:0016491	0.116
slr0642	GO:0009308	<1e-4	sll1321	GO:0009262	<1e-4	ssr0336	GO:0052188	<1e-4	sll1240	GO:0044446	<1e-4	sll1192	GO:0016491	0.116
slr0456	GO:0044106	<1e-4	sll0775	GO:0022607	<1e-4	sll7031	GO:0008150	<1e-4	slr1263	GO:0044464	<1e-4	ssr7093	GO:0016491	0.116
ssr1766	GO:0051171	<1e-4	sll0397	GO:0019637	<1e-4	ssr6020	GO:0006576	<1e-4	slr2025	GO:0044464	<1e-4	slr0816	GO:0016491	0.116
slr0989	GO:0008150	<1e-4	ssr0335	GO:0006721	<1e-4	ssr1552	GO:0043648	<1e-4	sll8002	GO:0005575	<1e-4	sll0060	GO:0016491	0.116
slr1025	GO:0009892	<1e-4	slr0885	GO:0009132	<1e-4	sll0678	GO:0006163	<1e-4	sll1350	GO:0044444	<1e-4	slr6075	GO:0016491	0.116
slr0458	GO:0044281	<1e-4	sll2006	GO:0016052	<1e-4	slr0588	GO:0009889	<1e-4	slr1851	GO:0044446	<1e-4	sll1381	GO:0016491	0.116
ssl5113	GO:0033013	<1e-4	sll8011	GO:0072521	<1e-4	sll0872	GO:0009893	<1e-4	sll1495	GO:0044424	<1e-4	slr0516	GO:0016491	0.116
ssl0788	GO:0048518	<1e-4	sll0503	GO:0009893	<1e-4	slr0589	GO:0009161	<1e-4	sll1267	GO:0005575	<1e-4	slr1056	GO:0016491	0.116
ssl0787	GO:0009889	<1e-4	sll1757	GO:0043170	<1e-4	slr1667	GO:0046128	<1e-4	slr0590	GO:0043226	<1e-4	slr2071	GO:0016491	0.116
sll0756	GO:0055086	<1e-4	slr1752	GO:0006082	<1e-4	sll1757	GO:0072524	<1e-4	ssr3122	GO:0044446	<1e-4	sll0253	GO:0016491	0.116
slr0364	GO:0009165	<1e-4	ssr3154	GO:0031640	<1e-4	slr1194	GO:0072521	<1e-4	slr1194	GO:0044422	<1e-4	slr0725	GO:0016491	0.116
sll7063	GO:0005996	<1e-4	ssr3122	GO:0045184	<1e-4	slr0770	GO:0042180	<1e-4	slr0013	GO:0043226	<1e-4	ssr0336	GO:0016491	0.116
slr0049	GO:0048523	<1e-4	sll1715	GO:0048523	<1e-4	sll1232	GO:0072522	<1e-4	sll0871	GO:0044425	<1e-4	slr1951	GO:0016491	0.116
slr1513	GO:0055082	<1e-4	ssr3154	GO:0009262	<1e-4	sll1390	GO:0072521	<1e-4	slr0326	GO:0044425	<1e-4	slr1507	GO:0016491	0.116
ssr5074	GO:0034654	<1e-4	slr1069	GO:0048518	<1e-4	sll0875	GO:0046483	<1e-4	sll0355	GO:0044464	<1e-4	sll1164	GO:0016491	0.116
sll0183	GO:0048523	<1e-4	slr1183	GO:0022607	<1e-4	sll0394	GO:0043933	<1e-4	ssl7046	GO:0005575	<1e-4	slr1737	GO:0016491	0.116
slr0645	GO:0006066	<1e-4	sll0602	GO:0006733	<1e-4	sll0282	GO:0019219	<1e-4	slr1306	GO:0044422	<1e-4	slr0645	GO:0016491	0.116
slr1638	GO:0051179	<1e-4	sll0623	GO:0090304	<1e-4	slr1614	GO:0045184	<1e-4	sll5006	GO:0005575	<1e-4	ssl0410	GO:0016491	0.116
slr0941	GO:0010556	<1e-4	sll0024	GO:0050801	<1e-4	slr7011	GO:0009144	<1e-4	slr0217	GO:0044444	<1e-4	sll0847	GO:0016491	0.116
slr1236	GO:0051347	<1e-4	sll1960	GO:0048878	<1e-4	ssr1552	GO:0009123	<1e-4	sll0436	GO:0005575	<1e-4	sll7089	GO:0016491	0.116
sll1882	GO:0051179	<1e-4	slr1187	GO:0044255	<1e-4	sll1608	GO:0042455	<1e-4	slr6064	GO:0044425	<1e-4	sll0101	GO:0016491	0.116
sll1378	GO:0080090	<1e-4	sll5132	GO:0048522	<1e-4	slr0642	GO:0006732	<1e-4	sll1541	GO:0044422	<1e-4	sll0031	GO:0016491	0.116
sll0710	GO:0006091	<1e-4	slr1875	GO:0006631	<1e-4	slr0885	GO:0052188	<1e-4	slr2111	GO:0044444	<1e-4	ssl0788	GO:0016491	0.116
sll0563	GO:0044282	<1e-4	sll7078	GO:0019637	<1e-4	ssl1263	GO:0031640	<1e-4	slr7097	GO:0005575	<1e-4	ssl2138	GO:0016491	0.116
sll7086	GO:0048518	<1e-4	sll0997	GO:0009161	<1e-4	sll1233	GO:0022607	<1e-4	slr1614	GO:0044446	<1e-4	slr0241	GO:0016491	0.116
sll1586	GO:0019751	<1e-4	sll1613	GO:0010468	<1e-4	slr1441	GO:0006733	<1e-4	sll1071	GO:0005575	<1e-4	sll5004	GO:0016491	0.116
sll0518	GO:0051171	<1e-4	sll1232	GO:0042180	<1e-4	sll1426	GO:0001932	<1e-4	ssr3467	GO:0005575	<1e-4	ssl7038	GO:0016491	0.116
sll6054	GO:0055114	<1e-4	ssr6003	GO:0009057	<1e-4	ssl6061	GO:0010468	<1e-4	ssr1528	GO:0043226	<1e-4	ssr3467	GO:0016491	0.116
slr0287	GO:0009260	<1e-4	sll7031	GO:0044283	<1e-4	sll0615	GO:0006720	<1e-4	sll1542	GO:0005575	<1e-4	slr0300	GO:0016491	0.116
slr1052	GO:0071496	<1e-4	sll0867	GO:0019222	<1e-4	slr0431	GO:0016054	<1e-4	sll7030	GO:0005575	<1e-4	slr5127	GO:0016491	0.116

slr0356	GO:0009059	<1e-4	sll0939	GO:0044003	<1e-4	sll1835	GO:0072527	<1e-4	slr0586	GO:0044424	<1e-4	sll0736	GO:0016491	0.116
slr0356	GO:0009991	<1e-4	slr0914	GO:0010468	<1e-4	sll0595	GO:0043648	<1e-4	ssr0657	GO:0044464	<1e-4	slr2012	GO:0016491	0.116
ssl2245	GO:0051179	<1e-4	sll0098	GO:0071842	<1e-4	slr2111	GO:0006732	<1e-4	ssr1552	GO:0044464	<1e-4	slr0325	GO:0016491	0.116
slr1647	GO:0051234	<1e-4	slr1484	GO:0006163	<1e-4	slr0196	GO:0044271	<1e-4	slr5024	GO:0043226	<1e-4	slr0610	GO:0016491	0.116
sll0670	GO:0072522	<1e-4	slr0184	GO:0048519	<1e-4	slr1098	GO:0031323	<1e-4	ssr2422	GO:0044425	<1e-4	slr1915	GO:0016491	0.116
sll0871	GO:0006631	<1e-4	sll1960	GO:0043170	<1e-4	sll1240	GO:0080090	<1e-4	sll1884	GO:0005575	<1e-4	ssr1425	GO:0016491	0.116
sll0861	GO:0009126	<1e-4	ssl1918	GO:0048878	<1e-4	sll1571	GO:0044283	<1e-4	slr2120	GO:0005575	<1e-4	slr1394	GO:0016491	0.116
slr1505	GO:0048522	<1e-4	sll1352	GO:0009144	<1e-4	sll0811	GO:0009161	<1e-4	sll1503	GO:0044444	<1e-4	sll0525	GO:0016491	0.116
sll0659	GO:0006732	<1e-4	slr0728	GO:0046128	<1e-4	slr1179	GO:0008150	<1e-4	ssr6048	GO:0005575	<1e-4	ssl1300	GO:0016491	0.116
slr1288	GO:0033013	<1e-4	ssl1464	GO:0009263	<1e-4	slr1814	GO:0009259	<1e-4	ssl3615	GO:0044425	<1e-4	slr1327	GO:0016491	0.116
slr1677	GO:0050794	<1e-4	sll1389	GO:0010556	<1e-4	ssl5095	GO:0052188	<1e-4	slr0049	GO:0044444	<1e-4	slr1819	GO:0016491	0.116
ssl3451	GO:0009132	<1e-4	slr8021	GO:0022411	<1e-4	slr0588	GO:0022411	<1e-4	slr1935	GO:0044424	<1e-4	ssr6083	GO:0016491	0.116
sll5047	GO:0044275	<1e-4	slr0770	GO:0046394	<1e-4	slr5023	GO:0009165	<1e-4	sll0447	GO:0005575	<1e-4	sll0172	GO:0016491	0.116
slr1658	GO:0080090	<1e-4	sll0447	GO:0031326	<1e-4	slr0456	GO:0072528	<1e-4	slr6080	GO:0044425	<1e-4	sll0335	GO:0016491	0.116
ssr8013	GO:0042455	<1e-4	slr0416	GO:0071496	<1e-4	ssr6024	GO:0019219	<1e-4	slr1353	GO:0005575	<1e-4	sll1609	GO:0016491	0.116
ssl5031	GO:0042180	<1e-4	slr1173	GO:0022411	<1e-4	slr1429	GO:0009308	<1e-4	sll1640	GO:0044464	<1e-4	slr0270	GO:0016491	0.116
sll0060	GO:0072524	<1e-4	slr1593	GO:0051347	<1e-4	sll1315	GO:0051186	<1e-4	slr0059	GO:0044464	<1e-4	sll1052	GO:0016491	0.116
sll1222	GO:0009141	<1e-4	slr1957	GO:0006733	<1e-4	ssr3571	GO:0051716	<1e-4	slr1789	GO:0044444	<1e-4	ssr6030	GO:0016491	0.116
slr1306	GO:0006163	<1e-4	sll5034	GO:0009991	<1e-4	sll1162	GO:0046394	<1e-4	sll7029	GO:0044425	<1e-4	sll1714	GO:0016491	0.116
sll0024	GO:0055114	<1e-4	slr0709	GO:0006811	<1e-4	slr1047	GO:0048518	<1e-4	sll1396	GO:0044444	<1e-4	slr0334	GO:0016491	0.116
sll0423	GO:0051246	<1e-4	sll0980	GO:0006721	<1e-4	slr0519	GO:0048878	<1e-4	slr7102	GO:0044446	<1e-4	ssr1155	GO:0016491	0.116
ssr2711	GO:0044275	<1e-4	sll1446	GO:0009144	<1e-4	slr0362	GO:0009987	<1e-4	slr1863	GO:0043226	<1e-4	slr0582	GO:0016491	0.116
sll1307	GO:0019752	<1e-4	slr1391	GO:0055082	<1e-4	slr0695	GO:0009165	<1e-4	sll1155	GO:0044444	<1e-4	slr0184	GO:0016491	0.116
slr0065	GO:0009056	<1e-4	slr6045	GO:0009889	<1e-4	slr0751	GO:0072522	<1e-4	sll5003	GO:0044444	<1e-4	slr0238	GO:0016491	0.116
sll1527	GO:0048878	<1e-4	sll0360	GO:0009132	<1e-4	sll5006	GO:0072528	<1e-4	sll0595	GO:0044446	<1e-4	slr7094	GO:0016491	0.116
ssl3573	GO:0009144	<1e-4	sll0749	GO:0006732	<1e-4	sll1315	GO:0051171	<1e-4	ssl5015	GO:0044422	<1e-4	slr1235	GO:0005215	0.114
sll1634	GO:0009893	<1e-4	slr0423	GO:0009892	<1e-4	ssr2912	GO:0050794	<1e-4	sll1265	GO:0044444	<1e-4	ssl0294	GO:0005215	0.114
ssr6083	GO:0050896	<1e-4	slr1886	GO:0031326	<1e-4	slr1079	GO:0080090	<1e-4	ssr0692	GO:0044424	<1e-4	sll1527	GO:0005215	0.114
sll1476	GO:0051188	<1e-4	slr1886	GO:0009165	<1e-4	ssl8039	GO:0043170	<1e-4	ssr2047	GO:0043226	<1e-4	slr1266	GO:0017111	0.1123
slr1307	GO:0009892	<1e-4	slr0145	GO:0009132	<1e-4	slr1999	GO:0009056	<1e-4	ssl1552	GO:0044464	<1e-4	sll0198	GO:0017111	0.1123
sll0762	GO:0019751	<1e-4	ssl3615	GO:0019222	<1e-4	sll1092	GO:0034641	<1e-4	sll0479	GO:0044425	<1e-4	slr1437	GO:0017111	0.1123
slr1062	GO:0006091	<1e-4	ssl1004	GO:0043412	<1e-4	slr0937	GO:0071496	<1e-4	slr5037	GO:0044425	<1e-4	slr1593	GO:0017111	0.1123
sll0742	GO:0065007	<1e-4	sll0793	GO:0009199	<1e-4	slr0272	GO:0050789	<1e-4	ssr1258	GO:0005575	<1e-4	sll0381	GO:0017111	0.1123
slr0092	GO:0050896	<1e-4	slr1926	GO:0050801	<1e-4	slr0962	GO:0051347	<1e-4	slr0271	GO:0044446	<1e-4	slr1999	GO:0017111	0.1123
ssl5114	GO:0080090	<1e-4	sll1884	GO:0052188	<1e-4	sll1160	GO:0009165	<1e-4	slr1240	GO:0044446	<1e-4	slr1778	GO:0017111	0.1123
sll0803	GO:0018130	<1e-4	sll0585	GO:0006576	<1e-4	sll1191	GO:0071841	<1e-4	sll1735	GO:0044425	<1e-4	sll0185	GO:0017111	0.1123
slr6106	GO:0052111	<1e-4	sll0630	GO:0071841	<1e-4	slr6075	GO:0019752	<1e-4	sll1873	GO:0044464	<1e-4	sll1613	GO:0017111	0.1123
ssl3451	GO:0043549	<1e-4	slr0887	GO:0046483	<1e-4	ssr1768	GO:0052111	<1e-4	sll0944	GO:0043226	<1e-4	slr1571	GO:0017111	0.1123
slr0588	GO:0009126	<1e-4	ssl7039	GO:0009893	<1e-4	slr1767	GO:0044003	<1e-4	slr1396	GO:0005575	<1e-4	slr2122	GO:0017111	0.1123
sll0496	GO:0006220	<1e-4	slr1570	GO:0051186	<1e-4	sll1378	GO:0050794	<1e-4	slr0273	GO:0044424	<1e-4	sll1630	GO:0017111	0.1123
slr0059	GO:0016054	<1e-4	sll1769	GO:0071554	<1e-4	slr0360	GO:0009991	<1e-4	sll0060	GO:0043226	<1e-4	slr0103	GO:0017111	0.1123
slr2000	GO:0016053	<1e-4	slr6007	GO:0022607	<1e-4	sll1089	GO:0090304	<1e-4	sll1656	GO:0044446	<1e-4	slr1081	GO:0017111	0.1123
slr0407	GO:0008150	<1e-4	slr1614	GO:0009123	<1e-4	ssr6079	GO:0016053	<1e-4	slr0586	GO:0043226	<1e-4	sll0243	GO:0017111	0.1123
slr0305	GO:0009126	<1e-4	sll0428	GO:0042451	<1e-4	slr0725	GO:0006631	<1e-4	ssl1707	GO:0005575	<1e-4	slr0348	GO:0017111	0.1123
slr7101	GO:0009132	<1e-4	slr1590	GO:0046483	<1e-4	slr0318	GO:0009893	<1e-4	sll1835	GO:0043226	<1e-4	slr0913	GO:0017111	0.1123
slr1780	GO:0008150	<1e-4	slr7010	GO:0048878	<1e-4	sll0810	GO:0009260	<1e-4	sll0441	GO:0044425	<1e-4	sll0066	GO:0017111	0.1123
slr0607	GO:0048519	<1e-4	sll0656	GO:0072521	<1e-4	sll0294	GO:0019752	<1e-4	slr2071	GO:0044464	<1e-4	ssl2065	GO:0017111	0.1123
slr1571	GO:0034654	<1e-4	sll0985	GO:0016070	<1e-4	sll1658	GO:0006720	<1e-4	slr2070	GO:0044464	<1e-4	sll0944	GO:0017111	0.1123
sll0071	GO:0009117	<1e-4	ssr7084	GO:0044283	<1e-4	slr1101	GO:0006082	<1e-4	sll1874	GO:0043226	<1e-4	ssr2439	GO:0017111	0.1123
slr0199	GO:0044275	<1e-4	slr0654	GO:0019751	<1e-4	slr1951	GO:0050801	<1e-4	ssl3549	GO:0044425	<1e-4	slr1885	GO:0017111	0.1123
slr1964	GO:0009991	<1e-4	sll1318	GO:0051171	<1e-4	slr0337	GO:0008150	<1e-4	sll0301	GO:0044425	<1e-4	slr1573	GO:0017111	0.1123
slr0333	GO:0009161	<1e-4	sll0596	GO:0006066	<1e-4	slr0489	GO:0048519	<1e-4	slr1773	GO:0044422	<1e-4	slr1932	GO:0017111	0.1123
sll1884	GO:0009987	<1e-4	slr6068	GO:0071841	<1e-4	sll0860	GO:0043436	<1e-4	slr5112	GO:0044446	<1e-4	slr0770	GO:0017111	0.1123
slr7015	GO:0009132	<1e-4	slr7016	GO:0042180	<1e-4	slr1800	GO:0034641	<1e-4	slr1437	GO:0044424	<1e-4	ssr1765	GO:0017111	0.1123

sll1164	GO:0008610	<1e-4	sll7065	GO:0006721	<1e-4	slr6015	GO:0006066	<1e-4	slr0609	GO:0044425	<1e-4	slr1964	GO:0017111	0.1123
slr0587	GO:0051234	<1e-4	ssl2471	GO:0046164	<1e-4	sll1785	GO:0044255	<1e-4	sll0283	GO:0044425	<1e-4	sll0524	GO:0017111	0.1123
slr6072	GO:0055114	<1e-4	slr1599	GO:0071841	<1e-4	sll1250	GO:0019751	<1e-4	slr1926	GO:0005575	<1e-4	sll0314	GO:0017111	0.1123
sll0867	GO:0046394	<1e-4	slr1444	GO:0043412	<1e-4	sll5032	GO:0051179	<1e-4	ssl0242	GO:0044464	<1e-4	slr0006	GO:0017111	0.1123
sll0639	GO:0009199	<1e-4	slr0655	GO:0006066	<1e-4	slr0852	GO:0072524	<1e-4	slr6057	GO:0044422	<1e-4	sll0325	GO:0017111	0.1123
sll0588	GO:0072521	<1e-4	ssr0335	GO:0005996	<1e-4	sll8001	GO:0019752	<1e-4	slr8044	GO:0044424	<1e-4	ssr3571	GO:0017111	0.1123
sll1307	GO:0006082	<1e-4	ssl1918	GO:0006733	<1e-4	sll1049	GO:0019637	<1e-4	slr7057	GO:0005575	<1e-4	slr0192	GO:0017111	0.1123
slr7071	GO:0009892	<1e-4	sll1726	GO:0019751	<1e-4	sll1071	GO:0009126	<1e-4	sll1921	GO:0005575	<1e-4	slr1854	GO:0017111	0.1123
sll1273	GO:0006220	<1e-4	slr1927	GO:0042451	<1e-4	slr1813	GO:0009893	<1e-4	slr0053	GO:0005575	<1e-4	slr1084	GO:0017111	0.1123
sll1722	GO:0008610	<1e-4	ssr1407	GO:0019219	<1e-4	sll1040	GO:0009142	<1e-4	ssl1377	GO:0044464	<1e-4	sll0216	GO:0017111	0.1123
sll1054	GO:0019222	<1e-4	sll0524	GO:0031323	<1e-4	sll0007	GO:0051171	<1e-4	ssl7042	GO:0043226	<1e-4	slr1648	GO:0017111	0.1123
sll1123	GO:0051818	<1e-4	slr1970	GO:0072524	<1e-4	sll5026	GO:0051818	<1e-4	ssl3379	GO:0044422	<1e-4	slr0872	GO:0017111	0.1123
slr1812	GO:0072522	<1e-4	ssr7036	GO:0044283	<1e-4	slr1069	GO:0006811	<1e-4	sll0253	GO:0044444	<1e-4	sll0756	GO:0017111	0.1123
sll1158	GO:0006163	<1e-4	slr0816	GO:0043549	<1e-4	ssr6019	GO:0048878	<1e-4	ssr2711	GO:0005575	<1e-4	slr1788	GO:0017111	0.1123
slr0326	GO:0042451	<1e-4	sll0735	GO:0051186	<1e-4	sll0445	GO:0072528	<1e-4	ssr2962	GO:0005575	<1e-4	sll1442	GO:0017111	0.1123
slr0249	GO:0071554	<1e-4	slr1970	GO:0006811	<1e-4	ssl0788	GO:0006793	<1e-4	sll1541	GO:0044446	<1e-4	slr0023	GO:0017111	0.1123
slr1450	GO:0055114	<1e-4	slr1215	GO:0009262	<1e-4	sll1832	GO:0022411	<1e-4	slr0304	GO:0044424	<1e-4	slr0392	GO:0017111	0.1123
sll1201	GO:0044248	<1e-4	ssr1407	GO:0006811	<1e-4	slr6103	GO:0009117	<1e-4	sll1372	GO:0044444	<1e-4	slr1177	GO:0017111	0.1123
slr5024	GO:0072528	<1e-4	sll1021	GO:0043412	<1e-4	slr7015	GO:0010468	<1e-4	slr1189	GO:0044424	<1e-4	sll1563	GO:0017111	0.1123
slr0730	GO:0009123	<1e-4	sll1401	GO:0051188	<1e-4	slr1616	GO:0009117	<1e-4	sll0095	GO:0044424	<1e-4	sll0280	GO:0017111	0.1123
slr6028	GO:0052188	<1e-4	sll1531	GO:0006811	<1e-4	slr1047	GO:0050789	<1e-4	slr1050	GO:0044422	<1e-4	slr1880	GO:0017111	0.1123
slr0645	GO:0009056	<1e-4	sll0765	GO:0071840	<1e-4	ssr3402	GO:0048522	<1e-4	slr0907	GO:0044464	<1e-4	sll1315	GO:0017111	0.1123
slr0751	GO:0031640	<1e-4	slr0049	GO:0046128	<1e-4	slr0725	GO:0006720	<1e-4	sll1505	GO:0044444	<1e-4	ssr3122	GO:0017111	0.1123
sll1174	GO:0055114	<1e-4	slr1116	GO:0006766	<1e-4	sll1273	GO:0071841	<1e-4	sll0225	GO:0044424	<1e-4	slr1638	GO:0017111	0.1123
ssr2201	GO:0009262	<1e-4	sll0615	GO:0006766	<1e-4	slr1023	GO:0009056	<1e-4	sll7075	GO:0044424	<1e-4	sll0532	GO:0017111	0.1123
slr2092	GO:0006793	<1e-4	sll1702	GO:0001932	<1e-4	ssl5099	GO:0008150	<1e-4	sll1380	GO:0044425	<1e-4	slr0431	GO:0017111	0.1123
sll0749	GO:0043549	<1e-4	ssr3189	GO:0072521	<1e-4	slr1276	GO:0044283	<1e-4	slr5017	GO:0005575	<1e-4	ssr2755	GO:0017111	0.1123
sll0060	GO:0008610	<1e-4	slr0453	GO:0009126	<1e-4	ssl3573	GO:0009161	<1e-4	ssr0335	GO:0043226	<1e-4	sll1002	GO:0017111	0.1123
slr0937	GO:0048522	<1e-4	slr2120	GO:0072521	<1e-4	sll0449	GO:0001932	<1e-4	sll5089	GO:0044464	<1e-4	sll0456	GO:0017111	0.1123
ssr6086	GO:0060255	<1e-4	sll8040	GO:0044248	<1e-4	sll0298	GO:0051186	<1e-4	slr1915	GO:0005575	<1e-4	slr1275	GO:0017111	0.1123
sll1186	GO:0009124	<1e-4	ssl3291	GO:0042180	<1e-4	sll7055	GO:0006631	<1e-4	sll5062	GO:0044444	<1e-4	slr2121	GO:0017111	0.1123
slr0333	GO:0044248	<1e-4	sll1735	GO:0050794	<1e-4	slr1670	GO:0072528	<1e-4	sll0630	GO:0044446	<1e-4	slr1676	GO:0017111	0.1123
sll0775	GO:0042180	<1e-4	slr1391	GO:0051188	<1e-4	slr1194	GO:0051186	<1e-4	slr6049	GO:0044425	<1e-4	sll1262	GO:0017111	0.1123
slr0680	GO:0044106	<1e-4	ssl0350	GO:0071841	<1e-4	slr2000	GO:0019222	<1e-4	sll1562	GO:0044444	<1e-4	slr0519	GO:0017111	0.1123
sll0282	GO:0051347	<1e-4	sll0265	GO:0016054	<1e-4	slr6073	GO:0072521	<1e-4	slr1470	GO:0005575	<1e-4	slr0404	GO:0017111	0.1123
sll0072	GO:0006720	<1e-4	slr2005	GO:0046394	<1e-4	slr1240	GO:0006721	<1e-4	sll0623	GO:0005575	<1e-4	ssr2615	GO:0017111	0.1123
slr1557	GO:0009263	<1e-4	ssr7084	GO:0051347	<1e-4	sll0857	GO:0043549	<1e-4	sll1378	GO:0005575	<1e-4	sll0355	GO:0017111	0.1123
ssr1768	GO:0006766	<1e-4	slr1773	GO:0008150	<1e-4	sll1151	GO:0065007	<1e-4	slr0976	GO:0044422	<1e-4	sll0085	GO:0017111	0.1123
sll0436	GO:0009259	<1e-4	slr1240	GO:0022411	<1e-4	slr2103	GO:0080090	<1e-4	slr2144	GO:0044444	<1e-4	slr1262	GO:0017111	0.1123
sll5033	GO:0009394	<1e-4	sll1054	GO:0009260	<1e-4	slr2038	GO:0006576	<1e-4	sll0779	GO:0044425	<1e-4	sll0414	GO:0017111	0.1123
slr0291	GO:0046164	<1e-4	sll1696	GO:0050794	<1e-4	slr5016	GO:0043648	<1e-4	sll1752	GO:0005575	<1e-4	slr7023	GO:0003676	0.112
sll0793	GO:0080090	<1e-4	slr0498	GO:0048878	<1e-4	sll1504	GO:0051716	<1e-4	sll0298	GO:0005575	<1e-4	sll0611	GO:0003676	0.112
sll1254	GO:0006793	<1e-4	sll0503	GO:0043436	<1e-4	slr0514	GO:0046907	<1e-4	slr1913	GO:0005575	<1e-4	sll1414	GO:0003676	0.112
slr0147	GO:0018130	<1e-4	sll1552	GO:0052111	<1e-4	sll1715	GO:0065007	<1e-4	slr6051	GO:0043226	<1e-4	sll1773	GO:0003676	0.112
slr0863	GO:0071496	<1e-4	slr1168	GO:0009991	<1e-4	slr6008	GO:0009059	<1e-4	slr2004	GO:0044464	<1e-4	slr6005	GO:0003676	0.112
slr1885	GO:0009057	<1e-4	ssl0750	GO:0046394	<1e-4	slr0551	GO:0044248	<1e-4	slr1970	GO:0005575	<1e-4	sll1401	GO:0003676	0.112
sll1476	GO:0071841	<1e-4	ssr6020	GO:0072528	<1e-4	slr6071	GO:0051347	<1e-4	slr1117	GO:0043226	<1e-4	sll0498	GO:0003676	0.112
sll8032	GO:0019222	<1e-4	slr1288	GO:0016052	<1e-4	ssr3129	GO:0050801	<1e-4	slr1519	GO:0044424	<1e-4	sll0761	GO:0003676	0.112
sll7077	GO:0042451	<1e-4	sll0446	GO:0006793	<1e-4	ssr1375	GO:0044003	<1e-4	slr1376	GO:0043226	<1e-4	ssr1766	GO:0003676	0.112
slr0521	GO:0072524	<1e-4	sll1714	GO:0072524	<1e-4	sll0149	GO:0051171	<1e-4	slr1276	GO:0005575	<1e-4	slr1600	GO:0003676	0.112
slr5012	GO:0048523	<1e-4	slr1573	GO:0010468	<1e-4	slr7081	GO:0044255	<1e-4	sll0479	GO:0005575	<1e-4	slr1472	GO:0003676	0.112
slr0108	GO:0019219	<1e-4	sll1399	GO:0022411	<1e-4	slr6029	GO:0005996	<1e-4	ssr2333	GO:0044424	<1e-4	slr7081	GO:0003676	0.112
sll0743	GO:0009126	<1e-4	sll0639	GO:0034641	<1e-4	slr1613	GO:0050896	<1e-4	ssl3829	GO:0043226	<1e-4	slr1956	GO:0003676	0.112
slr0243	GO:0019637	<1e-4	slr0291	GO:0051179	<1e-4	sll0996	GO:0050896	<1e-4	slr1599	GO:0005575	<1e-4	sll5130	GO:0003676	0.112

sll0743	GO:0034654	<1e-4	ssr3129	GO:0006721	<1e-4	sll1272	GO:0051234	<1e-4	slr1847	GO:0044422	<1e-4	ssr6046	GO:0003676	0.112
slr1896	GO:0006091	<1e-4	slr1127	GO:0009263	<1e-4	sll0577	GO:0009141	<1e-4	sll0909	GO:0044424	<1e-4	sll0218	GO:0003676	0.112
slr1376	GO:0019637	<1e-4	sll0588	GO:0019637	<1e-4	slr1590	GO:0042180	<1e-4	slr6074	GO:0044422	<1e-4	ssr6032	GO:0003676	0.112
sll0350	GO:0019752	<1e-4	slr1900	GO:0042180	<1e-4	sll5130	GO:0009141	<1e-4	ssl8003	GO:0044446	<1e-4	slr1187	GO:0003676	0.112
sll1528	GO:0071840	<1e-4	sll0249	GO:0060255	<1e-4	sll1541	GO:0044248	<1e-4	slr8021	GO:0044422	<1e-4	slr7101	GO:0003676	0.112
sll1775	GO:0010556	<1e-4	ssl8028	GO:0009165	<1e-4	slr1572	GO:0043648	<1e-4	slr0812	GO:0044444	<1e-4	sll1961	GO:0003676	0.112
sll5062	GO:0043648	<1e-4	slr1384	GO:0006066	<1e-4	slr6039	GO:0046164	<1e-4	slr1378	GO:0043226	<1e-4	sll1004	GO:0003676	0.112
ssr2439	GO:0006811	<1e-4	slr0876	GO:0009987	<1e-4	slr1179	GO:0006066	<1e-4	sll1250	GO:0044444	<1e-4	slr0195	GO:0003676	0.112
slr7026	GO:0009117	<1e-4	sll1773	GO:0044003	<1e-4	sll1863	GO:0071496	<1e-4	sll1504	GO:0005575	<1e-4	slr5023	GO:0003676	0.112
slr1690	GO:0019752	<1e-4	sll0335	GO:0034654	<1e-4	slr2049	GO:0051179	<1e-4	slr1789	GO:0005575	<1e-4	sll1766	GO:0003676	0.112
slr5073	GO:0090304	<1e-4	ssr5092	GO:0046907	<1e-4	sll0175	GO:0072522	<1e-4	sll0355	GO:0044446	<1e-4	slr0769	GO:0003676	0.112
slr0039	GO:0009987	<1e-4	slr0765	GO:0009262	<1e-4	slr0924	GO:0009263	<1e-4	sll1601	GO:0005575	<1e-4	sll1757	GO:0003676	0.112
sll0588	GO:0006091	<1e-4	slr0241	GO:0009117	<1e-4	sll0863	GO:0009144	<1e-4	slr1507	GO:0044422	<1e-4	sll0872	GO:0003676	0.112
slr1768	GO:0006811	<1e-4	slr6090	GO:0051246	<1e-4	sll1942	GO:0051188	<1e-4	ssr1552	GO:0005575	<1e-4	sll7063	GO:0003676	0.112
slr0742	GO:0008150	<1e-4	slr1896	GO:0009199	<1e-4	ssr3129	GO:0044271	<1e-4	sll1764	GO:0044446	<1e-4	slr0869	GO:0003676	0.112
slr1852	GO:0046128	<1e-4	slr1999	GO:0051186	<1e-4	sll1447	GO:0016054	<1e-4	slr1807	GO:0044425	<1e-4	sll0913	GO:0003676	0.112
slr0815	GO:0006732	<1e-4	sll1882	GO:0006766	<1e-4	sll0558	GO:0042455	<1e-4	slr7091	GO:0044446	<1e-4	ssl1972	GO:0003676	0.112
sll0886	GO:0009141	<1e-4	sll0325	GO:0043412	<1e-4	sll1217	GO:0006732	<1e-4	sll1949	GO:0044464	<1e-4	sll1348	GO:0003676	0.112
slr1813	GO:0052111	<1e-4	sll1858	GO:0044282	<1e-4	slr1768	GO:0034654	<1e-4	ssr3572	GO:0005575	<1e-4	slr6090	GO:0003676	0.112
sll5004	GO:0031323	<1e-4	sll0691	GO:0044248	<1e-4	sll0676	GO:0052111	<1e-4	sll1121	GO:0043226	<1e-4	slr0146	GO:0003676	0.112
slr1638	GO:0006811	<1e-4	sll0072	GO:0006766	<1e-4	slr0325	GO:0051716	<1e-4	slr6087	GO:0044422	<1e-4	slr6014	GO:0003676	0.112
slr0407	GO:0006811	<1e-4	ssl8028	GO:0072524	<1e-4	slr1178	GO:0006733	<1e-4	sll5090	GO:0044464	<1e-4	ssl2781	GO:0003676	0.112
sll1173	GO:0051171	<1e-4	slr0545	GO:0016052	<1e-4	slr0740	GO:0019752	<1e-4	slr1261	GO:0044425	<1e-4	sll5062	GO:0003676	0.112
slr1670	GO:0009259	<1e-4	slr0554	GO:0044281	<1e-4	slr5021	GO:0051171	<1e-4	slr1273	GO:0043226	<1e-4	sll2013	GO:0003676	0.112
sll0749	GO:0044281	<1e-4	slr1117	GO:0009309	<1e-4	slr0398	GO:0051716	<1e-4	sll0044	GO:0044424	<1e-4	sll0811	GO:0003676	0.112
sll1252	GO:0048518	<1e-4	sll1766	GO:0051188	<1e-4	sll1411	GO:0072522	<1e-4	slr1914	GO:0005575	<1e-4	sll1906	GO:0003676	0.112
sll0482	GO:0016053	<1e-4	slr0919	GO:0046483	<1e-4	slr2103	GO:0072522	<1e-4	slr1188	GO:0044444	<1e-4	slr1261	GO:0003676	0.112
slr7102	GO:0019637	<1e-4	ssl2807	GO:0019222	<1e-4	slr1911	GO:0048519	<1e-4	ssr1114	GO:0044464	<1e-4	slr0333	GO:0003676	0.112
slr1437	GO:0044282	<1e-4	ssr1114	GO:0016053	<1e-4	slr1262	GO:0006091	<1e-4	sll0703	GO:0044464	<1e-4	slr6074	GO:0003676	0.112
ssr1765	GO:0051246	<1e-4	slr1188	GO:0006631	<1e-4	slr6090	GO:0044271	<1e-4	sll1505	GO:0005575	<1e-4	sll0282	GO:0003676	0.112
slr0456	GO:0009259	<1e-4	slr5111	GO:0009259	<1e-4	slr5012	GO:0051234	<1e-4	slr1914	GO:0044422	<1e-4	slr1547	GO:0003676	0.112
slr0959	GO:0006163	<1e-4	sll0488	GO:0080090	<1e-4	slr0948	GO:0006753	<1e-4	slr1866	GO:0044446	<1e-4	slr0751	GO:0003676	0.112
sll0688	GO:0006811	<1e-4	slr1670	GO:0006721	<1e-4	sml0011	GO:0016052	<1e-4	slr1998	GO:0043226	<1e-4	ssl0312	GO:0003676	0.112
slr0596	GO:0033013	<1e-4	sll1915	GO:0016054	<1e-4	slr0249	GO:0044255	<1e-4	sll1698	GO:0044446	<1e-4	slr1218	GO:0003676	0.112
sll1203	GO:0009124	<1e-4	slr0680	GO:0019222	<1e-4	slr0852	GO:0080090	<1e-4	sll7067	GO:0043226	<1e-4	sll0048	GO:0003676	0.112
sll0047	GO:0006163	<1e-4	sll0325	GO:0034641	<1e-4	sll0428	GO:0009889	<1e-4	slr1069	GO:0044464	<1e-4	sll5061	GO:0003676	0.112
slr2070	GO:0048522	<1e-4	sll1954	GO:0044282	<1e-4	slr1636	GO:0044248	<1e-4	ssl3829	GO:0005575	<1e-4	sll1921	GO:0003676	0.112
sll0263	GO:0044255	<1e-4	ssr1528	GO:0009059	<1e-4	slr0305	GO:0009123	<1e-4	slr6072	GO:0044444	<1e-4	sll1764	GO:0003676	0.112
sll1021	GO:0034654	<1e-4	slr1183	GO:0034654	<1e-4	sll1757	GO:0031640	<1e-4	ssr5121	GO:0043226	<1e-4	slr0058	GO:0003676	0.112
sll0319	GO:0009117	<1e-4	slr2038	GO:0009309	<1e-4	slr1415	GO:0009126	<1e-4	sll0444	GO:0044444	<1e-4	ssl5129	GO:0003676	0.112
ssl0294	GO:0009123	<1e-4	sll0263	GO:0071496	<1e-4	slr1767	GO:0042455	<1e-4	sll1272	GO:0005575	<1e-4	sll8033	GO:0003676	0.112
sll0445	GO:0071842	<1e-4	slr0941	GO:0009991	<1e-4	ssl2471	GO:0009141	<1e-4	sll0301	GO:0043226	<1e-4	sll1939	GO:0003676	0.112
sll1671	GO:0043436	<1e-4	sll1109	GO:0072521	<1e-4	slr0989	GO:0051716	<1e-4	ssr3572	GO:0044424	<1e-4	sll1696	GO:0003676	0.112
slr0964	GO:0006576	<1e-4	sll0172	GO:0009056	<1e-4	sll1469	GO:0009057	<1e-4	sll1006	GO:0044424	<1e-4	slr5018	GO:0003676	0.112
sll1472	GO:0072522	<1e-4	slr1052	GO:0080090	<1e-4	slr1814	GO:0006720	<1e-4	sll7063	GO:0044446	<1e-4	slr1095	GO:0003676	0.112
slr0613	GO:0051818	<1e-4	slr0784	GO:0048878	<1e-4	sll0068	GO:0009309	<1e-4	slr1507	GO:0005575	<1e-4	slr0362	GO:0003676	0.112
slr0818	GO:0034660	<1e-4	ssr6030	GO:0016053	<1e-4	slr7013	GO:0006082	<1e-4	sll0382	GO:0043226	<1e-4	ssr2611	GO:0003676	0.112
sll7033	GO:0042451	<1e-4	slr0104	GO:0006631	<1e-4	slr1648	GO:0042180	<1e-4	slr0482	GO:0043226	<1e-4	slr0971	GO:0003676	0.112
slr1353	GO:0009893	<1e-4	sll1318	GO:0051186	<1e-4	sll0281	GO:0046483	<1e-4	slr0181	GO:0044444	<1e-4	slr1907	GO:0003676	0.112
slr1128	GO:0010556	<1e-4	sll0857	GO:0055086	<1e-4	sll0103	GO:0072524	<1e-4	sll0597	GO:0005575	<1e-4	slr6072	GO:0003676	0.112
slr1081	GO:0009893	<1e-4	slr5101	GO:0006082	<1e-4	slr0169	GO:0009262	<1e-4	sll0590	GO:0044464	<1e-4	ssl3383	GO:0003676	0.112
sll0382	GO:0071841	<1e-4	sll1049	GO:0006753	<1e-4	slr2048	GO:0071842	<1e-4	ssr2554	GO:0044425	<1e-4	ssl1004	GO:0003676	0.112
slr5118	GO:0044248	<1e-4	slr0810	GO:0051246	<1e-4	slr1702	GO:0009142	<1e-4	slr0146	GO:0044422	<1e-4	slr1102	GO:0003676	0.112
slr0609	GO:0009141	<1e-4	sll1495	GO:0009124	<1e-4	slr1464	GO:0009165	<1e-4	slr0581	GO:0044464	<1e-4	sll5044	GO:0003676	0.112

ssl2749	GO:0044003	<1e-4	slr0145	GO:0009991	<1e-4	slr6065	GO:0006721	<1e-4	sll0397	GO:0043226	<1e-4	slr6008	GO:0003676	0.112
sll0563	GO:0045184	<1e-4	ssl2148	GO:0051234	<1e-4	slr1062	GO:0043648	<1e-4	slr1647	GO:0044444	<1e-4	slr6006	GO:0003676	0.112
sll0910	GO:0043170	<1e-4	slr0589	GO:0009262	<1e-4	sll1570	GO:0009991	<1e-4	slr6051	GO:0044446	<1e-4	ssl2009	GO:0003676	0.112
slr0554	GO:0050896	<1e-4	slr0602	GO:0009987	<1e-4	slr1644	GO:0080090	<1e-4	ssl7042	GO:0044424	<1e-4	ssr0335	GO:0003676	0.112
sll0577	GO:0043412	<1e-4	slr1384	GO:0006811	<1e-4	slr0587	GO:0051246	<1e-4	slr1429	GO:0044422	<1e-4	ssl1417	GO:0003676	0.112
sll0839	GO:0001932	<1e-4	slr0456	GO:0006091	<1e-4	ssr2318	GO:0051234	<1e-4	sll0839	GO:0005575	<1e-4	slr6013	GO:0003676	0.112
slr0065	GO:0006733	<1e-4	sll1054	GO:0034654	<1e-4	slr0907	GO:0008610	<1e-4	slr0299	GO:0044444	<1e-4	slr0695	GO:0003676	0.112
slr0980	GO:0006793	<1e-4	sll0863	GO:0046394	<1e-4	slr0272	GO:0006811	<1e-4	ssl5100	GO:0005575	<1e-4	slr6021	GO:0003676	0.112
slr1752	GO:0051171	<1e-4	slr8014	GO:0009056	<1e-4	slr1069	GO:0048878	<1e-4	sll0060	GO:0044446	<1e-4	sll5090	GO:0003676	0.112
ssr1258	GO:0043933	<1e-4	slr1178	GO:0044282	<1e-4	sll0630	GO:0008610	<1e-4	ssl7045	GO:0005575	<1e-4	sll6054	GO:0003676	0.112
ssr6002	GO:0051818	<1e-4	slr0341	GO:0019362	<1e-4	slr1790	GO:0050801	<1e-4	slr1885	GO:0044424	<1e-4	ssl2148	GO:0003676	0.112
sll1613	GO:0009117	<1e-4	sll1119	GO:0031323	<1e-4	slr1599	GO:0008150	<1e-4	slr2038	GO:0044424	<1e-4	slr1179	GO:0003676	0.112
slr0263	GO:0043933	<1e-4	slr2018	GO:0009161	<1e-4	sll0355	GO:0044271	<1e-4	sll0010	GO:0044425	<1e-4	slr0959	GO:0003676	0.112
slr6072	GO:0009161	<1e-4	sll0361	GO:0044283	<1e-4	slr7060	GO:0022411	<1e-4	sll1461	GO:0044425	<1e-4	sll0630	GO:0003676	0.112
slr1547	GO:0046128	<1e-4	slr2144	GO:0052188	<1e-4	sll1702	GO:0009144	<1e-4	sll0010	GO:0043226	<1e-4	sll0867	GO:0003676	0.112
slr6007	GO:0006576	<1e-4	slr0521	GO:0009056	<1e-4	slr1098	GO:0051818	<1e-4	sll1681	GO:0044425	<1e-4	slr1378	GO:0003676	0.112
slr1383	GO:0031326	<1e-4	ssr3571	GO:0009394	<1e-4	ssr2611	GO:0022411	<1e-4	slr0060	GO:0044444	<1e-4	slr0355	GO:0003676	0.112
ssl0832	GO:0080090	<1e-4	sll0444	GO:0009892	<1e-4	sll0925	GO:0043648	<1e-4	sll1218	GO:0044425	<1e-4	slr1273	GO:0003676	0.112
ssl5113	GO:0042455	<1e-4	slr1788	GO:0019751	<1e-4	slr0423	GO:0071496	<1e-4	sll0175	GO:0044424	<1e-4	sll1092	GO:0003676	0.112
sll1736	GO:0071842	<1e-4	sll1272	GO:0044255	<1e-4	sll1241	GO:0009057	<1e-4	slr0711	GO:0044424	<1e-4	ssl8003	GO:0003676	0.112
slr0870	GO:0046483	<1e-4	slr1052	GO:0009123	<1e-4	sll1531	GO:0043412	<1e-4	sll0216	GO:0044425	<1e-4	ssr3304	GO:0003676	0.112
sll0372	GO:0080090	<1e-4	sll0397	GO:0065007	<1e-4	sll1106	GO:0065007	<1e-4	slr0575	GO:0005575	<1e-4	slr1464	GO:0003676	0.112
slr0634	GO:0090304	<1e-4	sll1495	GO:0009142	<1e-4	slr0821	GO:0033013	<1e-4	slr1188	GO:0043226	<1e-4	slr7011	GO:0003676	0.112
ssl5099	GO:0043933	<1e-4	slr2120	GO:0055082	<1e-4	ssr3571	GO:0019219	<1e-4	slr0575	GO:0044425	<1e-4	sll1797	GO:0003676	0.112
slr1169	GO:0043933	<1e-4	sll1446	GO:0006163	<1e-4	sll1086	GO:0006811	<1e-4	sll1106	GO:0044446	<1e-4	slr0380	GO:0003676	0.112
sll1186	GO:0016052	<1e-4	sll0103	GO:0048519	<1e-4	ssl2420	GO:0048522	<1e-4	slr1470	GO:0044422	<1e-4	slr0151	GO:0003676	0.112
slr1603	GO:0045184	<1e-4	slr1699	GO:0016054	<1e-4	slr0103	GO:0009056	<1e-4	sll1252	GO:0005575	<1e-4	sll8002	GO:0003676	0.112
ssr6020	GO:0019362	<1e-4	slr6066	GO:0009124	<1e-4	ssl8005	GO:0050794	<1e-4	slr2060	GO:0044444	<1e-4	slr7096	GO:0003676	0.112
slr6015	GO:0009056	<1e-4	slr1107	GO:0055082	<1e-4	slr0913	GO:0034641	<1e-4	sll0847	GO:0044444	<1e-4	sll1769	GO:0003676	0.112
sll0858	GO:0009308	<1e-4	ssl7021	GO:0051716	<1e-4	sll1654	GO:0043648	<1e-4	ssl5095	GO:0044446	<1e-4	sll1783	GO:0003676	0.112
ssr6019	GO:0019637	<1e-4	slr0935	GO:0009150	<1e-4	ssr1041	GO:0046483	<1e-4	sll0189	GO:0005575	<1e-4	sll1510	GO:0003676	0.112
slr1541	GO:0031323	<1e-4	sll0736	GO:0009987	<1e-4	slr6031	GO:0006732	<1e-4	sll0939	GO:0043226	<1e-4	slr1660	GO:0003676	0.112
sll0162	GO:0006082	<1e-4	slr1623	GO:0072521	<1e-4	slr1628	GO:0006753	<1e-4	sll1315	GO:0044446	<1e-4	slr1116	GO:0003676	0.112
sll1219	GO:0051716	<1e-4	slr0104	GO:0046394	<1e-4	sll0283	GO:0006732	<1e-4	sll0005	GO:0044422	<1e-4	slr0619	GO:0003676	0.112
sll0630	GO:0043549	<1e-4	slr0957	GO:0019222	<1e-4	sll1380	GO:0034654	<1e-4	slr0863	GO:0044446	<1e-4	slr6063	GO:0003676	0.112
sll0783	GO:0034654	<1e-4	slr0980	GO:0006753	<1e-4	sll1424	GO:0018130	<1e-4	sll0172	GO:0005575	<1e-4	slr1611	GO:0003676	0.112
sll1714	GO:0046128	<1e-4	slr5021	GO:0006082	<1e-4	sll1272	GO:0009124	<1e-4	sll1769	GO:0044446	<1e-4	slr0305	GO:0016787	0.1112
sll7063	GO:0051186	<1e-4	sll0449	GO:0044106	<1e-4	sll6052	GO:0034641	<1e-4	slr1565	GO:0005575	<1e-4	slr0157	GO:0016787	0.1112
sll1866	GO:0009056	<1e-4	sll0639	GO:0009150	<1e-4	slr1618	GO:0019362	<1e-4	sll0785	GO:0044444	<1e-4	sll1426	GO:0016787	0.1112
slr1327	GO:0005996	<1e-4	ssl5113	GO:0009142	<1e-4	slr2004	GO:0055114	<1e-4	ssl3142	GO:0044425	<1e-4	slr1918	GO:0016787	0.1112
slr1591	GO:0090304	<1e-4	slr0195	GO:0009142	<1e-4	slr7091	GO:0009124	<1e-4	sll5132	GO:0044446	<1e-4	sll1186	GO:0016787	0.1112
slr0318	GO:0009142	<1e-4	ssl5091	GO:0009889	<1e-4	slr6088	GO:0009262	<1e-4	slr0852	GO:0005575	<1e-4	sll0596	GO:0016787	0.1112
slr6066	GO:0006220	<1e-4	slr1275	GO:0009309	<1e-4	slr6094	GO:0009893	<1e-4	sll1547	GO:0044464	<1e-4	slr0551	GO:0016787	0.1112
sll2007	GO:0006576	<1e-4	slr5073	GO:0009124	<1e-4	slr0699	GO:0009263	<1e-4	slr0038	GO:0044424	<1e-4	sll0447	GO:0016787	0.1112
sll1240	GO:0009144	<1e-4	slr6100	GO:0019752	<1e-4	sll0503	GO:0042451	<1e-4	slr0780	GO:0044422	<1e-4	sll1934	GO:0016787	0.1112
sll1306	GO:0006720	<1e-4	ssr1473	GO:0033013	<1e-4	slr5102	GO:0006753	<1e-4	slr1032	GO:0044425	<1e-4	sll0283	GO:0016787	0.1112
sll2013	GO:0034654	<1e-4	slr2012	GO:0006163	<1e-4	sll1251	GO:0055086	<1e-4	sll0319	GO:0044446	<1e-4	sll1247	GO:0016787	0.1112
slr1464	GO:0044260	<1e-4	sll0479	GO:0031323	<1e-4	sll1681	GO:0009987	<1e-4	sgl0002	GO:0044425	<1e-4	sll0296	GO:0016787	0.1112
slr7096	GO:0044260	<1e-4	slr0728	GO:0006811	<1e-4	slr1799	GO:0009394	<1e-4	ssl2749	GO:0044446	<1e-4	ssr0536	GO:0016787	0.1112
slr1599	GO:0009262	<1e-4	slr6045	GO:0052111	<1e-4	sll0572	GO:0001932	<1e-4	slr1573	GO:0044464	<1e-4	slr1900	GO:0016787	0.1112
ssr2754	GO:0042451	<1e-4	sll1775	GO:0006631	<1e-4	sll1764	GO:0009893	<1e-4	sll0176	GO:0005575	<1e-4	slr1391	GO:0016787	0.1112
slr1449	GO:0043549	<1e-4	slr1789	GO:0009059	<1e-4	slr0957	GO:0048519	<1e-4	slr0147	GO:0044425	<1e-4	slr1612	GO:0016787	0.1112
slr0806	GO:0006220	<1e-4	slr1101	GO:0072527	<1e-4	sll1911	GO:0006163	<1e-4	sll0857	GO:0044464	<1e-4	sll1218	GO:0016787	0.1112
slr6104	GO:0043412	<1e-4	sll7087	GO:0018130	<1e-4	slr1442	GO:0071842	<1e-4	slr5024	GO:0005575	<1e-4	slr0287	GO:0016787	0.1112

sll5128	GO:0051188	<1e-4	sll1715	GO:0051188	<1e-4	sll0602	GO:0009165	<1e-4	ssl5025	GO:0005575	<1e-4	slr1066	GO:0016787	0.1112
slr0590	GO:0010556	<1e-4	sll1052	GO:0031326	<1e-4	slr1397	GO:0009126	<1e-4	slr6038	GO:0044422	<1e-4	slr1045	GO:0016787	0.1112
sll5033	GO:0034654	<1e-4	sll1784	GO:0051347	<1e-4	ssl1464	GO:0016054	<1e-4	slr1236	GO:0043226	<1e-4	slr1866	GO:0016787	0.1112
sll0854	GO:0005996	<1e-4	slr0924	GO:0006091	<1e-4	sll0296	GO:0031323	<1e-4	slr0416	GO:0044464	<1e-4	slr1896	GO:0016787	0.1112
sll8027	GO:0022411	<1e-4	sll0670	GO:0072528	<1e-4	slr1505	GO:0009126	<1e-4	sll0811	GO:0043226	<1e-4	slr0376	GO:0016787	0.1112
sll0532	GO:0009165	<1e-4	slr0935	GO:0009991	<1e-4	slr1183	GO:0006811	<1e-4	slr6028	GO:0005575	<1e-4	slr1917	GO:0016787	0.1112
slr0287	GO:0072528	<1e-4	slr0479	GO:0051716	<1e-4	slr1544	GO:0006066	<1e-4	slr0635	GO:0005575	<1e-4	slr1196	GO:0016787	0.1112
sll1763	GO:0051171	<1e-4	sll1651	GO:0071554	<1e-4	ssl3692	GO:0006066	<1e-4	sll0102	GO:0044424	<1e-4	sll0148	GO:0016787	0.1112
slr0937	GO:0009987	<1e-4	slr0146	GO:0033013	<1e-4	sll0446	GO:0048878	<1e-4	sll1960	GO:0043226	<1e-4	slr0503	GO:0016787	0.1112
sll0423	GO:0044106	<1e-4	sll0545	GO:0009308	<1e-4	ssr1951	GO:0034654	<1e-4	ssr2755	GO:0005575	<1e-4	slr1222	GO:0016787	0.1112
slr6006	GO:0050896	<1e-4	slr0491	GO:0080090	<1e-4	sll0980	GO:0009144	<1e-4	sll0448	GO:0044444	<1e-4	slr0852	GO:0016787	0.1112
slr0374	GO:0009124	<1e-4	slr0848	GO:0009144	<1e-4	slr1613	GO:0006721	<1e-4	ssl2781	GO:0043226	<1e-4	slr1353	GO:0016787	0.1112
sll0181	GO:0090304	<1e-4	slr2073	GO:0051818	<1e-4	slr0179	GO:0051347	<1e-4	ssl5113	GO:0005575	<1e-4	sll0360	GO:0016787	0.1112
sll2011	GO:0080090	<1e-4	ssl0352	GO:0052188	<1e-4	slr0589	GO:0006811	<1e-4	sll0518	GO:0044446	<1e-4	slr0423	GO:0016787	0.1112
slr1398	GO:0080090	<1e-4	slr0554	GO:0042455	<1e-4	sll1495	GO:0046907	<1e-4	sll1969	GO:0044444	<1e-4	sll1942	GO:0016787	0.1112
slr6029	GO:0042451	<1e-4	slr0147	GO:0051716	<1e-4	sll0898	GO:0055086	<1e-4	slr1444	GO:0043226	<1e-4	slr1398	GO:0016787	0.1112
sll0933	GO:0090304	<1e-4	sll0263	GO:0055086	<1e-4	slr0888	GO:0009144	<1e-4	slr1648	GO:0044464	<1e-4	sll0274	GO:0016787	0.1112
sll1632	GO:0065007	<1e-4	sll0847	GO:0072528	<1e-4	slr7025	GO:0006811	<1e-4	sll1272	GO:0044424	<1e-4	slr1194	GO:0016787	0.1112
slr1816	GO:0009141	<1e-4	slr1990	GO:0009057	<1e-4	sll1766	GO:0019637	<1e-4	sll0068	GO:0044422	<1e-4	ssr1375	GO:0016787	0.1112
slr2111	GO:0022411	<1e-4	ssr5011	GO:0052188	<1e-4	sll0188	GO:0060255	<1e-4	ssl2971	GO:0044444	<1e-4	sll1542	GO:0016787	0.1112
sll7065	GO:0009263	<1e-4	ssr2551	GO:0042180	<1e-4	slr0581	GO:0009262	<1e-4	sll1265	GO:0044446	<1e-4	slr0476	GO:0016787	0.1112
ssl2065	GO:0016054	<1e-4	sll1388	GO:0048519	<1e-4	sll1902	GO:0044271	<1e-4	sll0191	GO:0044424	<1e-4	slr0209	GO:0016787	0.1112
slr2027	GO:0009309	<1e-4	slr0871	GO:0051716	<1e-4	slr2105	GO:0031640	<1e-4	slr1230	GO:0044464	<1e-4	slr1025	GO:0016787	0.1112
slr0609	GO:0050896	<1e-4	sll1225	GO:0046128	<1e-4	slr1799	GO:0051186	<1e-4	slr0964	GO:0044422	<1e-4	slr0304	GO:0016787	0.1112
ssr1155	GO:0019751	<1e-4	sll0608	GO:0006091	<1e-4	slr0699	GO:0009262	<1e-4	slr1811	GO:0043226	<1e-4	slr1505	GO:0016787	0.1112
sll0827	GO:0009165	<1e-4	slr6101	GO:0009308	<1e-4	slr6080	GO:0051171	<1e-4	ssr3189	GO:0044425	<1e-4	sll0864	GO:0016787	0.1112
sll1738	GO:0009123	<1e-4	sll1638	GO:0009161	<1e-4	ssr2551	GO:0043933	<1e-4	sll0875	GO:0043226	<1e-4	sll0354	GO:0016787	0.1112
slr0496	GO:0051186	<1e-4	slr1668	GO:0009987	<1e-4	slr1053	GO:0044106	<1e-4	slr0725	GO:0044424	<1e-4	slr0876	GO:0016787	0.1112
sll0710	GO:0009262	<1e-4	slr1240	GO:0006793	<1e-4	slr5037	GO:0071496	<1e-4	slr1413	GO:0044424	<1e-4	sll0743	GO:0016787	0.1112
sll0068	GO:0019752	<1e-4	ssl5096	GO:0008610	<1e-4	sll1884	GO:0009132	<1e-4	sll0265	GO:0044444	<1e-4	slr1659	GO:0016787	0.1112
sll1192	GO:0044248	<1e-4	slr0476	GO:0090304	<1e-4	slr1519	GO:0009141	<1e-4	sll0488	GO:0044424	<1e-4	ssr3532	GO:0016787	0.1112
sll0839	GO:0072521	<1e-4	slr0108	GO:0080090	<1e-4	slr0551	GO:0009165	<1e-4	slr0356	GO:0044424	<1e-4	sll0985	GO:0005488	0.1103
slr0146	GO:0046164	<1e-4	sll1601	GO:0006753	<1e-4	slr0784	GO:0009308	<1e-4	slr1363	GO:0044422	<1e-4	sll0283	GO:0017111	0.1098
sll0410	GO:0046907	<1e-4	sll1531	GO:0044260	<1e-4	sll0293	GO:0019752	<1e-4	slr6081	GO:0044422	<1e-4	slr0157	GO:0017111	0.1098
sll7066	GO:0006811	<1e-4	slr0168	GO:0090304	<1e-4	sll0710	GO:0019362	<1e-4	sll7078	GO:0044444	<1e-4	slr1918	GO:0017111	0.1098
slr1932	GO:0009263	<1e-4	sll1352	GO:0019751	<1e-4	sll8001	GO:0005996	<1e-4	slr0168	GO:0044464	<1e-4	slr1196	GO:0017111	0.1098
slr6022	GO:0051818	<1e-4	slr0921	GO:0065007	<1e-4	slr5023	GO:0051716	<1e-4	slr0053	GO:0044464	<1e-4	ssl3692	GO:0017111	0.1098
ssl2733	GO:0006793	<1e-4	sll0846	GO:0001932	<1e-4	slr6091	GO:0050801	<1e-4	sll0558	GO:0044464	<1e-4	sll0615	GO:0017111	0.1098
sll1068	GO:0019751	<1e-4	slr0482	GO:0071496	<1e-4	sll7086	GO:0009150	<1e-4	slr1919	GO:0044464	<1e-4	slr0250	GO:0017111	0.1098
slr1236	GO:0050896	<1e-4	slr1122	GO:0019752	<1e-4	sll0376	GO:0065007	<1e-4	sll1352	GO:0005575	<1e-4	sll0447	GO:0017111	0.1098
ssl1520	GO:0044282	<1e-4	slr1547	GO:0009059	<1e-4	slr1186	GO:0034654	<1e-4	sll0473	GO:0043226	<1e-4	sll1542	GO:0017111	0.1098
sll1880	GO:0051171	<1e-4	sll0785	GO:0050896	<1e-4	sll1913	GO:0009308	<1e-4	sll1262	GO:0044446	<1e-4	slr1194	GO:0017111	0.1098
sll1583	GO:0051246	<1e-4	sll0661	GO:0043549	<1e-4	slr5118	GO:0009132	<1e-4	slr0356	GO:0005575	<1e-4	sll1455	GO:0017111	0.1098
sll0060	GO:0055086	<1e-4	slr7098	GO:0019752	<1e-4	slr1537	GO:0051716	<1e-4	ssr3570	GO:0043226	<1e-4	slr1127	GO:0017111	0.1098
slr6045	GO:0044255	<1e-4	slr1415	GO:0065007	<1e-4	slr5119	GO:0051716	<1e-4	sll0297	GO:0043226	<1e-4	slr0076	GO:0017111	0.1098
slr1880	GO:0008150	<1e-4	slr6066	GO:0072528	<1e-4	ssl2781	GO:0051234	<1e-4	slr0634	GO:0044446	<1e-4	sll1318	GO:0017111	0.1098
slr0667	GO:0072524	<1e-4	sll1240	GO:0050794	<1e-4	sll7086	GO:0071496	<1e-4	sll0488	GO:0044444	<1e-4	sll0354	GO:0017111	0.1098
slr0981	GO:0043412	<1e-4	slr0654	GO:0051186	<1e-4	slr1070	GO:0006576	<1e-4	slr2027	GO:0044444	<1e-4	sll1060	GO:0017111	0.1098
ssr6062	GO:0071842	<1e-4	ssr3467	GO:0042180	<1e-4	sll0188	GO:0042180	<1e-4	slr0610	GO:0044425	<1e-4	slr1023	GO:0017111	0.1098
slr1964	GO:0009893	<1e-4	slr0341	GO:0019752	<1e-4	slr0613	GO:0019751	<1e-4	sll1692	GO:0044422	<1e-4	slr1441	GO:0017111	0.1098
sll0596	GO:0009199	<1e-4	slr7100	GO:0046907	<1e-4	sll5046	GO:0009262	<1e-4	sll7062	GO:0043226	<1e-4	sll0296	GO:0017111	0.1098
ssr6089	GO:0044283	<1e-4	sll0611	GO:0050789	<1e-4	ssr6048	GO:0009142	<1e-4	ssr2754	GO:0044424	<1e-4	slr0551	GO:0017111	0.1098
ssr2615	GO:0022607	<1e-4	sll0376	GO:0006066	<1e-4	slr0468	GO:0042180	<1e-4	slr7010	GO:0044446	<1e-4	sll1433	GO:0017111	0.1098
slr0505	GO:0031326	<1e-4	slr1462	GO:0048518	<1e-4	slr1577	GO:0009117	<1e-4	slr0111	GO:0005575	<1e-4	slr1376	GO:0017111	0.1098

ssl1004	GO:0051171	<1e-4	slr0751	GO:0006732	<1e-4	ssl1690	GO:0022838	<1e-4	ssr2201	GO:0005575	<1e-4	ssr1698	GO:0017111	0.1098
sll0525	GO:0055086	<1e-4	slr0271	GO:0051171	<1e-4	ssr2060	GO:0022857	<1e-4	sll1956	GO:0044444	<1e-4	slr0243	GO:0017111	0.1098
sll1476	GO:0006576	<1e-4	slr1068	GO:0051246	<1e-4	sll8004	GO:0008171	<1e-4	sll1304	GO:0044446	<1e-4	sll0301	GO:0017111	0.1098
sll1691	GO:0031323	<1e-4	sll8040	GO:0006732	<1e-4	slr5116	GO:0016818	<1e-4	ssl6092	GO:0005575	<1e-4	ssl0109	GO:0017111	0.1098
slr8014	GO:0050789	<1e-4	slr0784	GO:0048519	<1e-4	slr1603	GO:0015075	<1e-4	slr1862	GO:0044446	<1e-4	sll0864	GO:0017111	0.1098
slr1590	GO:0009150	<1e-4	slr1470	GO:0044255	<1e-4	ssl1300	GO:0015291	<1e-4	slr0250	GO:0044446	<1e-4	sll0513	GO:0017111	0.1098
slr1033	GO:0008150	<1e-4	slr1307	GO:0018130	<1e-4	slr0039	GO:0015405	<1e-4	sll1505	GO:0044464	<1e-4	slr1917	GO:0017111	0.1098
slr1270	GO:0009892	<1e-4	sll0188	GO:0019637	<1e-4	sll1573	GO:0022857	<1e-4	ssr2067	GO:0044425	<1e-4	slr0287	GO:0017111	0.1098
slr2004	GO:0044282	<1e-4	ssl3451	GO:0006766	<1e-4	slr2105	GO:0022890	<1e-4	ssr1114	GO:0043226	<1e-4	sll0148	GO:0017111	0.1098
sll0413	GO:0031640	<1e-4	sll1307	GO:0006091	<1e-4	sll1166	GO:0022838	<1e-4	slr6051	GO:0005575	<1e-4	slr1045	GO:0017111	0.1098
ssl5008	GO:0019752	<1e-4	slr2110	GO:0051171	<1e-4	sll0752	GO:0022832	<1e-4	slr0192	GO:0044446	<1e-4	slr1398	GO:0017111	0.1098
slr7098	GO:0071554	<1e-4	ssr2843	GO:0009150	<1e-4	slr2070	GO:0015291	<1e-4	slr0971	GO:0044422	<1e-4	sll1135	GO:0017111	0.1098
sll1858	GO:0009987	<1e-4	sll1583	GO:0051716	<1e-4	sll1446	GO:0008171	<1e-4	sll1757	GO:0043226	<1e-4	slr0476	GO:0017111	0.1098
slr5021	GO:0045184	<1e-4	slr7012	GO:0044281	<1e-4	ssr5106	GO:0015405	<1e-4	slr0730	GO:0044425	<1e-4	sll1472	GO:0017111	0.1098
slr2118	GO:0055086	<1e-4	ssl2065	GO:0050789	<1e-4	sll1736	GO:0022832	<1e-4	sll0482	GO:0043226	<1e-4	slr0305	GO:0017111	0.1098
slr1425	GO:0006811	<1e-4	slr0013	GO:0051347	<1e-4	slr1168	GO:0016741	<1e-4	ssr1155	GO:0044444	<1e-4	sll0382	GO:0017111	0.1098
ssr0335	GO:0034641	<1e-4	slr1032	GO:0008610	<1e-4	slr1647	GO:0015293	<1e-4	sll0372	GO:0005575	<1e-4	sll0508	GO:0017111	0.1098
ssr3532	GO:0009056	<1e-4	slr1070	GO:0009263	<1e-4	slr1623	GO:0015291	<1e-4	slr1179	GO:0044446	<1e-4	sll1942	GO:0017111	0.1098
slr5037	GO:0051716	<1e-4	slr1444	GO:0072524	<1e-4	slr0871	GO:0008171	<1e-4	sll0503	GO:0005575	<1e-4	ssr3532	GO:0017111	0.1098
sll0670	GO:0009199	<1e-4	sll0564	GO:0052111	<1e-4	sll5003	GO:0015291	<1e-4	sll0638	GO:0044422	<1e-4	ssr1375	GO:0017111	0.1098
slr0211	GO:0051171	<1e-4	sll1552	GO:0009056	<1e-4	sll0661	GO:0015405	<1e-4	slr1565	GO:0044425	<1e-4	slr0876	GO:0017111	0.1098
sll0625	GO:0009263	<1e-4	sll1902	GO:0051186	<1e-4	sll1191	GO:0015291	<1e-4	slr1025	GO:0044444	<1e-4	sll1586	GO:0017111	0.1098
slr1376	GO:0044282	<1e-4	sll0372	GO:0055082	<1e-4	slr0924	GO:0015267	<1e-4	sll1002	GO:0044446	<1e-4	slr1866	GO:0017111	0.1098
sll0309	GO:0044271	<1e-4	ssr7072	GO:0009124	<1e-4	sll1797	GO:0060089	<1e-4	slr7060	GO:0044422	<1e-4	slr1896	GO:0017111	0.1098
slr0053	GO:0006811	<1e-4	ssr2333	GO:0016052	<1e-4	slr6080	GO:0016746	<1e-4	ssl3549	GO:0005575	<1e-4	ssr2848	GO:0017111	0.1098
slr1142	GO:0042180	<1e-4	sll0735	GO:0009141	<1e-4	slr1980	GO:0015291	<1e-4	slr1565	GO:0044444	<1e-4	slr0545	GO:0017111	0.1098
ssl3383	GO:0022607	<1e-4	ssr2553	GO:0055114	<1e-4	slr1915	GO:0015293	<1e-4	slr2048	GO:0044446	<1e-4	sll0996	GO:0017111	0.1098
sll0101	GO:0031323	<1e-4	sll1942	GO:0046483	<1e-4	slr0285	GO:0015293	<1e-4	sll0931	GO:0005575	<1e-4	slr0871	GO:0017111	0.1098
sll1652	GO:0009161	<1e-4	slr0771	GO:0060255	<1e-4	ssr0335	GO:0016818	<1e-4	slr0698	GO:0044446	<1e-4	sll1934	GO:0017111	0.1098
slr0552	GO:0009165	<1e-4	sll0545	GO:0001932	<1e-4	sll0847	GO:0015291	<1e-4	sll1447	GO:0044422	<1e-4	sll1601	GO:0017111	0.1098
sll0513	GO:0006082	<1e-4	slr7073	GO:0022607	<1e-4	slr0241	GO:0043492	<1e-4	sll1583	GO:0044425	<1e-4	sll1174	GO:0017111	0.1098
slr6072	GO:0043170	<1e-4	slr1056	GO:0043549	<1e-4	slr1263	GO:0016741	<1e-4	slr1342	GO:0043226	<1e-4	slr1315	GO:0017111	0.1094
slr5087	GO:0009132	<1e-4	sll1304	GO:0009161	<1e-4	sll0047	GO:0015405	<1e-4	ssr3571	GO:0044424	<1e-4	slr0695	GO:0017111	0.1094
slr5087	GO:0016053	<1e-4	ssr5120	GO:0048878	<1e-4	slr1168	GO:0022890	<1e-4	sll1866	GO:0043226	<1e-4	sll0284	GO:0017111	0.1094
slr0172	GO:0009057	<1e-4	sll8002	GO:0008150	<1e-4	ssl5108	GO:0043167	<1e-4	slr0876	GO:0044464	<1e-4	sll1638	GO:0017111	0.1094
slr0360	GO:0006220	<1e-4	sll0588	GO:0009893	<1e-4	sll1373	GO:0022890	<1e-4	slr0725	GO:0044464	<1e-4	slr1187	GO:0017111	0.1094
ssl1690	GO:0044003	<1e-4	slr1863	GO:0051347	<1e-4	slr1886	GO:0016817	<1e-4	sll0072	GO:0044444	<1e-4	sll0803	GO:0017111	0.1094
ssl3549	GO:0072522	<1e-4	sll0586	GO:0009889	<1e-4	sll7034	GO:0015291	<1e-4	sll1163	GO:0044422	<1e-4	sll7063	GO:0017111	0.1094
slr6028	GO:0006721	<1e-4	ssl1464	GO:0019362	<1e-4	sll1949	GO:0008171	<1e-4	sll1265	GO:0044424	<1e-4	slr7101	GO:0017111	0.1094
sll7033	GO:0009987	<1e-4	slr1827	GO:0050789	<1e-4	slr0789	GO:0022892	<1e-4	sll1009	GO:0044464	<1e-4	ssl2920	GO:0017111	0.1094
slr1062	GO:0009056	<1e-4	sll6052	GO:0009893	<1e-4	sll0563	GO:0003674	<1e-4	sll0803	GO:0044422	<1e-4	slr0601	GO:0017111	0.1094
sll1606	GO:0008150	<1e-4	sll0577	GO:0001932	<1e-4	slr0489	GO:0022832	<1e-4	sll1913	GO:0044464	<1e-4	sll0846	GO:0017111	0.1094
ssr0536	GO:0010468	<1e-4	slr2005	GO:0043412	<1e-4	slr0699	GO:0043492	<1e-4	ssr7036	GO:0044444	<1e-4	ssl7046	GO:0017111	0.1094
sll1424	GO:0006066	<1e-4	slr1702	GO:0001932	<1e-4	ssl8008	GO:0015267	<1e-4	ssl2471	GO:0005575	<1e-4	slr1365	GO:0017111	0.1094
ssl2245	GO:0009987	<1e-4	ssr3129	GO:0009394	<1e-4	sll1862	GO:0022832	<1e-4	sll0503	GO:0044446	<1e-4	slr1534	GO:0017111	0.1094
sll1640	GO:0006721	<1e-4	slr1450	GO:0052188	<1e-4	sll1634	GO:0005342	<1e-4	slr0586	GO:0044464	<1e-4	sll5033	GO:0017111	0.1094
slr1342	GO:0009309	<1e-4	sll1306	GO:0031640	<1e-4	slr1083	GO:0043167	<1e-4	sll1021	GO:0005575	<1e-4	ssl2420	GO:0017111	0.1094
sll7070	GO:0072521	<1e-4	sll1173	GO:0044271	<1e-4	slr0821	GO:0016746	<1e-4	ssl1707	GO:0044444	<1e-4	ssr5120	GO:0017111	0.1094
sll7033	GO:0009141	<1e-4	sll1095	GO:0042180	<1e-4	slr1235	GO:0015077	<1e-4	slr0609	GO:0044424	<1e-4	slr1150	GO:0017111	0.1094
sll0156	GO:0009260	<1e-4	sll0888	GO:0009057	<1e-4	ssr7035	GO:0016741	<1e-4	sll0066	GO:0044422	<1e-4	slr1186	GO:0017111	0.1094
sll2013	GO:0051234	<1e-4	slr1544	GO:0009309	<1e-4	ssr7100	GO:0043167	<1e-4	slr1431	GO:0044424	<1e-4	sll0659	GO:0017111	0.1094
ssl0353	GO:0060255	<1e-4	slr0655	GO:0042451	<1e-4	sll0442	GO:0003674	<1e-4	sll0525	GO:0005575	<1e-4	slr7083	GO:0017111	0.1094
slr0670	GO:0044282	<1e-4	ssl0832	GO:0031323	<1e-4	sll0218	GO:0022892	<1e-4	sll0023	GO:0043226	<1e-4	sll1866	GO:0017111	0.1094
slr1914	GO:0006163	<1e-4	ssl5100	GO:0050896	<1e-4	sll8032	GO:0022838	<1e-4	sll0623	GO:0044446	<1e-4	sll0597	GO:0017111	0.1094

slr1624	GO:0071841	<1e-4	slr2025	GO:0031326	<1e-4	sll1319	GO:0008171	<1e-4	slr0351	GO:0005575	<1e-4	slr7081	GO:0017111	0.1094
slr1611	GO:0006066	<1e-4	slr0285	GO:0019222	<1e-4	slr0921	GO:0022892	<1e-4	sll7006	GO:0044425	<1e-4	slr0264	GO:0017111	0.1094
ssl0739	GO:0006721	<1e-4	ssr2802	GO:0044282	<1e-4	ssr2912	GO:0015267	<1e-4	sll0263	GO:0044444	<1e-4	slr6067	GO:0017111	0.1094
slr2110	GO:0050789	<1e-4	sll0350	GO:0009141	<1e-4	slr1258	GO:0016462	<1e-4	ssl5064	GO:0044444	<1e-4	slr0655	GO:0017111	0.1094
sll0096	GO:0034641	<1e-4	sll1372	GO:0046907	<1e-4	slr0006	GO:0022836	<1e-4	slr1816	GO:0044424	<1e-4	sll0857	GO:0017111	0.1094
slr0273	GO:0019751	<1e-4	slr1441	GO:0009309	<1e-4	slr0505	GO:0043167	<1e-4	slr0692	GO:0005575	<1e-4	ssr2554	GO:0017111	0.1094
slr1660	GO:0046394	<1e-4	slr0605	GO:0051171	<1e-4	slr1260	GO:0022890	<1e-4	ssl1046	GO:0044422	<1e-4	sll5062	GO:0017111	0.1094
slr1613	GO:0072527	<1e-4	sll1495	GO:0043648	<1e-4	slr0598	GO:0022857	<1e-4	slr6038	GO:0005575	<1e-4	slr7098	GO:0017111	0.1094
sll1511	GO:0019751	<1e-4	sll0614	GO:0006220	<1e-4	sll0141	GO:0022892	<1e-4	slr1128	GO:0044444	<1e-4	ssl5065	GO:0017111	0.1094
slr1812	GO:0044260	<1e-4	sll1696	GO:0042180	<1e-4	slr1385	GO:0022838	<1e-4	slr0971	GO:0044424	<1e-4	sll1063	GO:0017111	0.1094
slr0601	GO:0006631	<1e-4	sll5028	GO:0009165	<1e-4	sll0085	GO:0022803	<1e-4	slr7083	GO:0044422	<1e-4	sll1510	GO:0017111	0.1094
sll1388	GO:0009394	<1e-4	ssl3692	GO:0009132	<1e-4	sll0596	GO:0022891	<1e-4	sll1939	GO:0043226	<1e-4	slr1195	GO:0017111	0.1094
sll1464	GO:0008610	<1e-4	sll0354	GO:0042455	<1e-4	slr1917	GO:0060089	<1e-4	sll0253	GO:0005575	<1e-4	sll0436	GO:0017111	0.1094
slr0869	GO:0051246	<1e-4	slr1209	GO:0051234	<1e-4	slr0976	GO:0043167	<1e-4	slr0291	GO:0044422	<1e-4	slr1178	GO:0017111	0.1094
sll1318	GO:0006733	<1e-4	ssl3829	GO:0009165	<1e-4	ssl2162	GO:0016741	<1e-4	sll1151	GO:0044444	<1e-4	ssr3341	GO:0017111	0.1094
slr6080	GO:0046394	<1e-4	sll0584	GO:0031323	<1e-4	slr0006	GO:0016818	<1e-4	sll0691	GO:0044464	<1e-4	slr1470	GO:0017111	0.1094
ssl8005	GO:0009260	<1e-4	sll0586	GO:0006811	<1e-4	ssl7074	GO:0022892	<1e-4	sll1373	GO:0005575	<1e-4	ssl5098	GO:0017111	0.1094
sll7065	GO:0006576	<1e-4	slr1413	GO:0044271	<1e-4	slr1170	GO:0005342	<1e-4	slr0769	GO:0044464	<1e-4	sll1461	GO:0017111	0.1094
sll1092	GO:0008610	<1e-4	ssl2384	GO:0009150	<1e-4	sll1939	GO:0016818	<1e-4	slr0294	GO:0044444	<1e-4	ssr0332	GO:0017111	0.1094
ssr5011	GO:0051234	<1e-4	ssr0102	GO:0048523	<1e-4	slr6049	GO:0043492	<1e-4	ssr8047	GO:0044446	<1e-4	slr0552	GO:0017111	0.1094
ssl2814	GO:0009165	<1e-4	slr1186	GO:0072528	<1e-4	sll0861	GO:0046873	<1e-4	sll0688	GO:0043226	<1e-4	sll1939	GO:0017111	0.1094
sll0735	GO:0009991	<1e-4	slr0869	GO:0048523	<1e-4	sll1400	GO:0022890	<1e-4	sll0175	GO:0044444	<1e-4	slr0598	GO:0017111	0.1094
slr0852	GO:0009260	<1e-4	sll0788	GO:0009144	<1e-4	ssl1255	GO:0005342	<1e-4	sll0981	GO:0043226	<1e-4	ssl5045	GO:0017111	0.1094
slr0360	GO:0009059	<1e-4	slr1082	GO:0046128	<1e-4	sll0867	GO:0022832	<1e-4	slr1677	GO:0044422	<1e-4	sll0811	GO:0017111	0.1094
sll0905	GO:0034654	<1e-4	sll1472	GO:0009123	<1e-4	sll0525	GO:0043167	<1e-4	sll1121	GO:0044446	<1e-4	sll0282	GO:0017111	0.1094
sll5003	GO:0071840	<1e-4	slr0989	GO:0043412	<1e-4	slr1788	GO:0022836	<1e-4	slr1827	GO:0044444	<1e-4	slr0971	GO:0017111	0.1094
sll0804	GO:0034641	<1e-4	slr1376	GO:0055114	<1e-4	sll1902	GO:0015291	<1e-4	slr6072	GO:0044425	<1e-4	slr1097	GO:0017111	0.1094
slr1600	GO:0051186	<1e-4	sll0253	GO:0006082	<1e-4	sll0268	GO:0015077	<1e-4	slr0204	GO:0005575	<1e-4	ssr1499	GO:0017111	0.1094
slr1084	GO:0006766	<1e-4	sll1675	GO:0009124	<1e-4	slr7081	GO:0016462	<1e-4	slr0728	GO:0005575	<1e-4	slr7082	GO:0017111	0.1094
sll0405	GO:0019222	<1e-4	slr0049	GO:0009893	<1e-4	ssl2471	GO:0022891	<1e-4	slr0482	GO:0044424	<1e-4	ssr7072	GO:0017111	0.1094
sll0656	GO:0009262	<1e-4	ssl7038	GO:0046394	<1e-4	slr2110	GO:0060089	<1e-4	sll0488	GO:0044464	<1e-4	slr1306	GO:0017111	0.1094
slr1800	GO:0065007	<1e-4	slr1468	GO:0009150	<1e-4	slr0157	GO:0043167	<1e-4	slr7091	GO:0005575	<1e-4	sll0802	GO:0017111	0.1094
slr1143	GO:0031640	<1e-4	slr7094	GO:0009057	<1e-4	ssr2843	GO:0022832	<1e-4	slr1900	GO:0044425	<1e-4	sll0419	GO:0017111	0.1094
slr0787	GO:0044255	<1e-4	slr1338	GO:0044106	<1e-4	slr2048	GO:0016817	<1e-4	sll1634	GO:0044424	<1e-4	slr0355	GO:0017111	0.1094
slr1210	GO:0010468	<1e-4	slr1918	GO:0044255	<1e-4	sll0995	GO:0015405	<1e-4	slr0514	GO:0044446	<1e-4	slr2101	GO:0017111	0.1094
slr0919	GO:0051716	<1e-4	sll1061	GO:0009308	<1e-4	sll1730	GO:0022832	<1e-4	slr1721	GO:0044464	<1e-4	ssl1923	GO:0017111	0.1094
slr5013	GO:0009199	<1e-4	slr0870	GO:0080090	<1e-4	sll1714	GO:0043167	<1e-4	slr1073	GO:0005575	<1e-4	sll0886	GO:0017111	0.1094
ssl5114	GO:0044283	<1e-4	sll1891	GO:0044282	<1e-4	ssr2848	GO:0060089	<1e-4	sll0864	GO:0044424	<1e-4	sll1884	GO:0017111	0.1094
ssr2998	GO:0072527	<1e-4	sll8002	GO:0044260	<1e-4	sll7047	GO:0022803	<1e-4	sll0160	GO:0043226	<1e-4	slr1923	GO:0017111	0.1094
sll1938	GO:0072524	<1e-4	sll0218	GO:0009057	<1e-4	slr1670	GO:0008171	<1e-4	slr1896	GO:0044444	<1e-4	slr1261	GO:0017111	0.1094
slr2073	GO:0043549	<1e-4	slr0605	GO:0009141	<1e-4	sll5028	GO:0016462	<1e-4	slr1169	GO:0044425	<1e-4	ssl2245	GO:0017111	0.1094
sll0525	GO:0065007	<1e-4	slr7101	GO:0048518	<1e-4	ssl2920	GO:0016818	<1e-4	sll0445	GO:0043226	<1e-4	slr1362	GO:0017111	0.1094
sll0939	GO:0046164	<1e-4	ssl1046	GO:0031640	<1e-4	slr7101	GO:0016746	<1e-4	sll5109	GO:0044446	<1e-4	sll0994	GO:0017111	0.1094
slr0023	GO:0009123	<1e-4	slr1083	GO:0034660	<1e-4	ssl1972	GO:0015399	<1e-4	slr0658	GO:0005575	<1e-4	slr0751	GO:0017111	0.1094
slr1638	GO:0031326	<1e-4	ssl3615	GO:0045184	<1e-4	slr1082	GO:0022832	<1e-4	slr0967	GO:0044444	<1e-4	slr2032	GO:0017111	0.1094
sll1784	GO:0050794	<1e-4	sll0863	GO:0009308	<1e-4	sll0102	GO:0015077	<1e-4	slr1906	GO:0044464	<1e-4	slr1677	GO:0017111	0.1094
ssl2162	GO:0031323	<1e-4	sll0732	GO:0016052	<1e-4	sll0147	GO:0022836	<1e-4	slr1647	GO:0044424	<1e-4	slr5023	GO:0017111	0.1094
slr1752	GO:0043648	<1e-4	sll1766	GO:0009123	<1e-4	slr0872	GO:0043492	<1e-4	sll1151	GO:0005575	<1e-4	sll0176	GO:0017111	0.1094
slr1169	GO:0019751	<1e-4	slr1557	GO:0009142	<1e-4	sll0156	GO:0022832	<1e-4	sll0905	GO:0044446	<1e-4	ssl3383	GO:0017111	0.1094
slr0742	GO:0019362	<1e-4	sll0602	GO:0006766	<1e-4	sll0547	GO:0015267	<1e-4	sll1693	GO:0005575	<1e-4	ssr3410	GO:0017111	0.1094
sll7050	GO:0009142	<1e-4	sll0804	GO:0046164	<1e-4	ssr2611	GO:0060089	<1e-4	slr1468	GO:0005575	<1e-4	ssr6026	GO:0017111	0.1094
ssr0102	GO:0072527	<1e-4	slr0519	GO:0042451	<1e-4	slr1977	GO:0060089	<1e-4	sll1608	GO:0044425	<1e-4	ssr2611	GO:0017111	0.1094
sll1696	GO:0034641	<1e-4	sll0265	GO:0009124	<1e-4	sll1698	GO:0003674	<1e-4	slr0959	GO:0044446	<1e-4	sll1348	GO:0017111	0.1094
slr0848	GO:0009056	<1e-4	ssl7021	GO:0009150	<1e-4	sll0688	GO:0015077	<1e-4	slr0771	GO:0044424	<1e-4	sll0473	GO:0017111	0.1094

sll0735	GO:0051716	<1e-4	slr0590	GO:0009262	<1e-4	slr1753	GO:0005342	<1e-4	slr0326	GO:0005575	<1e-4	slr6013	GO:0017111	0.1094
ssl1263	GO:0072522	<1e-4	slr5037	GO:0006631	<1e-4	slr0765	GO:0015267	<1e-4	ssl1707	GO:0044422	<1e-4	ssr1407	GO:0017111	0.1094
sll0175	GO:0071841	<1e-4	slr1301	GO:0044255	<1e-4	ssl1417	GO:0022832	<1e-4	slr6012	GO:0044425	<1e-4	sll8032	GO:0017111	0.1094
slr2032	GO:0008150	<1e-4	sll1373	GO:0048878	<1e-4	slr6081	GO:0016462	<1e-4	slr7097	GO:0044424	<1e-4	slr0337	GO:0017111	0.1094
slr0962	GO:0006066	<1e-4	sll7047	GO:0009141	<1e-4	ssl3379	GO:0022891	<1e-4	ssl8039	GO:0044424	<1e-4	sgl0001	GO:0017111	0.1094
slr1599	GO:0019219	<1e-4	sll0641	GO:0042180	<1e-4	sll5061	GO:0022803	<1e-4	sll0487	GO:0005575	<1e-4	slr1223	GO:0017111	0.1094
ssl5114	GO:0008150	<1e-4	slr1670	GO:0051716	<1e-4	sll1995	GO:0015405	<1e-4	slr0619	GO:0044446	<1e-4	slr1566	GO:0017111	0.1094
slr1505	GO:0034654	<1e-4	slr6072	GO:0022411	<1e-4	sll0742	GO:0016462	<1e-4	slr1183	GO:0044425	<1e-4	slr0356	GO:0017111	0.1094
sll0499	GO:0055086	<1e-4	ssr3300	GO:0008150	<1e-4	slr1940	GO:0015077	<1e-4	sll1902	GO:0044444	<1e-4	sll0008	GO:0017111	0.1094
ssl3379	GO:0019362	<1e-4	sll0787	GO:0046907	<1e-4	sll0565	GO:0015267	<1e-4	sll0266	GO:0044444	<1e-4	ssl5095	GO:0017111	0.1094
slr6006	GO:0009150	<1e-4	slr5087	GO:0006753	<1e-4	sll5046	GO:0005342	<1e-4	sll0898	GO:0044424	<1e-4	slr0262	GO:0017111	0.1094
slr0852	GO:0009394	<1e-4	slr1127	GO:0006720	<1e-4	slr0670	GO:0016817	<1e-4	slr0708	GO:0044425	<1e-4	sll1414	GO:0017111	0.1094
sll1052	GO:0050896	<1e-4	sll1285	GO:0044281	<1e-4	sll1735	GO:0015075	<1e-4	slr1627	GO:0044446	<1e-4	ssl1417	GO:0017111	0.1094
sll1068	GO:0001932	<1e-4	sll1541	GO:0009309	<1e-4	slr0476	GO:0016818	<1e-4	sll0762	GO:0043226	<1e-4	sll1155	GO:0017111	0.1094
slr6013	GO:0044275	<1e-4	slr7010	GO:0080090	<1e-4	slr0345	GO:0022891	<1e-4	slr1668	GO:0044422	<1e-4	slr1260	GO:0017111	0.1094
slr1397	GO:0072527	<1e-4	ssl1464	GO:0006811	<1e-4	slr1236	GO:0015293	<1e-4	slr6016	GO:0044422	<1e-4	sll0860	GO:0017111	0.1094
slr0695	GO:0072521	<1e-4	slr1915	GO:0006220	<1e-4	slr1287	GO:0015405	<1e-4	slr1194	GO:0043226	<1e-4	slr1914	GO:0017111	0.1094
ssr6003	GO:0009059	<1e-4	ssl5091	GO:0048523	<1e-4	slr1207	GO:0022892	<1e-4	sll1285	GO:0005575	<1e-4	sll0293	GO:0017111	0.1094
slr0325	GO:0009144	<1e-4	slr0770	GO:0016054	<1e-4	sll0062	GO:0015075	<1e-4	sll0096	GO:0044464	<1e-4	slr1207	GO:0017111	0.1094
sll1092	GO:0071841	<1e-4	ssr1698	GO:0009056	<1e-4	slr7059	GO:0042623	<1e-4	slr5115	GO:0005575	<1e-4	slr1339	GO:0017111	0.1094
sll1542	GO:0006631	<1e-4	slr7013	GO:0009987	<1e-4	ssr6089	GO:0008171	<1e-4	ssl2717	GO:0043226	<1e-4	slr6014	GO:0017111	0.1094
ssl2245	GO:0034654	<1e-4	slr1187	GO:0051246	<1e-4	slr1107	GO:0022803	<1e-4	slr0065	GO:0044446	<1e-4	slr6021	GO:0017111	0.1094
sll0871	GO:0044248	<1e-4	sll1130	GO:0009199	<1e-4	slr1854	GO:0022892	<1e-4	sll0925	GO:0043226	<1e-4	sll1961	GO:0017111	0.1094
ssl0787	GO:0009991	<1e-4	slr2010	GO:0022411	<1e-4	slr0360	GO:0022891	<1e-4	slr1970	GO:0044422	<1e-4	slr7097	GO:0017111	0.1094
ssr3410	GO:0009889	<1e-4	slr1944	GO:0009991	<1e-4	sll1785	GO:0022803	<1e-4	sll1835	GO:0044444	<1e-4	sll7062	GO:0017111	0.1094
slr0872	GO:0090304	<1e-4	slr0496	GO:0009165	<1e-4	slr0870	GO:0015405	<1e-4	slr0870	GO:0044446	<1e-4	sll1232	GO:0017111	0.1094
sll0168	GO:0009991	<1e-4	slr1235	GO:0051179	<1e-4	slr1444	GO:0015405	<1e-4	sll1053	GO:0044446	<1e-4	sll1355	GO:0017111	0.1094
slr1047	GO:0051179	<1e-4	ssr5019	GO:0010556	<1e-4	slr0551	GO:0016741	<1e-4	sll1388	GO:0005575	<1e-4	sll1053	GO:0017111	0.1094
ssr2802	GO:0009309	<1e-4	sll1267	GO:0019752	<1e-4	sll1250	GO:0022803	<1e-4	slr7058	GO:0044424	<1e-4	sgl0002	GO:0017111	0.1094
slr6029	GO:0034660	<1e-4	slr5021	GO:0009309	<1e-4	sll5130	GO:0016818	<1e-4	slr0596	GO:0044422	<1e-4	sll0913	GO:0017111	0.1094
slr1306	GO:0016053	<1e-4	ssr6062	GO:0006066	<1e-4	slr1210	GO:0016741	<1e-4	slr0602	GO:0044464	<1e-4	slr2118	GO:0017111	0.1094
sll5109	GO:0008610	<1e-4	ssl0410	GO:0043436	<1e-4	slr0479	GO:0022836	<1e-4	slr0151	GO:0044424	<1e-4	ssl1004	GO:0017111	0.1094
slr1114	GO:0006811	<1e-4	ssl2996	GO:0031323	<1e-4	sll0606	GO:0022832	<1e-4	slr2010	GO:0044444	<1e-4	sll1265	GO:0017111	0.1094
slr0602	GO:0045184	<1e-4	slr0039	GO:0043436	<1e-4	slr1376	GO:0016741	<1e-4	sll1359	GO:0044425	<1e-4	slr1956	GO:0017111	0.1094
sll0023	GO:0006811	<1e-4	sll1447	GO:0006793	<1e-4	sll1240	GO:0043167	<1e-4	slr1778	GO:0043226	<1e-4	slr0151	GO:0017111	0.1094
ssl2065	GO:0009144	<1e-4	ssl2148	GO:0051818	<1e-4	sll0688	GO:0005342	<1e-4	slr2119	GO:0044425	<1e-4	slr7013	GO:0017111	0.1094
slr0238	GO:0009056	<1e-4	ssl5096	GO:0009126	<1e-4	sll1509	GO:0015399	<1e-4	sll0925	GO:0005575	<1e-4	slr0505	GO:0017111	0.1094
ssr5121	GO:0051246	<1e-4	sll1344	GO:0055114	<1e-4	slr1218	GO:0016746	<1e-4	slr0810	GO:0005575	<1e-4	sll0443	GO:0017111	0.1094
ssr6078	GO:0071496	<1e-4	slr0887	GO:0051716	<1e-4	slr1363	GO:0016741	<1e-4	slr1101	GO:0044444	<1e-4	sll1025	GO:0017111	0.1094
slr1570	GO:0071496	<1e-4	slr1616	GO:0022411	<1e-4	slr1215	GO:0015267	<1e-4	slr0358	GO:0005575	<1e-4	sll1109	GO:0017111	0.1094
sll7090	GO:0048518	<1e-4	slr1315	GO:0044248	<1e-4	slr7023	GO:0022890	<1e-4	slr1827	GO:0044425	<1e-4	slr0380	GO:0017111	0.1094
slr1362	GO:0016054	<1e-4	slr1462	GO:0034660	<1e-4	slr0333	GO:0060089	<1e-4	ssr2802	GO:0005575	<1e-4	slr5118	GO:0017111	0.1094
sll0905	GO:0048523	<1e-4	ssr6002	GO:0016052	<1e-4	slr1258	GO:0022832	<1e-4	sll0419	GO:0044464	<1e-4	slr1660	GO:0017111	0.1094
ssr3122	GO:0048522	<1e-4	slr1206	GO:0009123	<1e-4	ssr5011	GO:0015399	<1e-4	slr6014	GO:0044425	<1e-4	sll0863	GO:0017111	0.1094
sll0910	GO:0009132	<1e-4	sll0811	GO:0009059	<1e-4	sll1424	GO:0022836	<1e-4	slr1070	GO:0005575	<1e-4	ssr0335	GO:0017111	0.1094
slr1069	GO:0044283	<1e-4	sll0837	GO:0051179	<1e-4	slr1101	GO:0022832	<1e-4	slr0643	GO:0044422	<1e-4	sll0102	GO:0017111	0.1094
sll0736	GO:0008150	<1e-4	sll0327	GO:0031326	<1e-4	sll0284	GO:0016746	<1e-4	ssr1766	GO:0044424	<1e-4	slr0606	GO:0017111	0.1094
sll0857	GO:0006163	<1e-4	sll1640	GO:0065007	<1e-4	sll1399	GO:0016462	<1e-4	sll1570	GO:0044444	<1e-4	ssl2009	GO:0017111	0.1094
sll0781	GO:0044271	<1e-4	sll5030	GO:0001932	<1e-4	ssl1552	GO:0016817	<1e-4	sll1252	GO:0044446	<1e-4	sll2015	GO:0017111	0.1094
ssr3402	GO:0071554	<1e-4	slr0709	GO:0050801	<1e-4	slr1107	GO:0015399	<1e-4	slr1940	GO:0044424	<1e-4	slr1263	GO:0017111	0.1094
sll0269	GO:0043549	<1e-4	slr1593	GO:0072524	<1e-4	slr1814	GO:0015267	<1e-4	slr0519	GO:0043226	<1e-4	sll0742	GO:0017111	0.1094
sll1486	GO:0009893	<1e-4	slr0103	GO:0043549	<1e-4	sll1352	GO:0015267	<1e-4	ssl2009	GO:0005575	<1e-4	sll5061	GO:0017111	0.1094
sll1424	GO:0072522	<1e-4	slr1069	GO:0006733	<1e-4	slr1568	GO:0022836	<1e-4	slr0689	GO:0005575	<1e-4	sll1217	GO:0017111	0.1094
sll0584	GO:0048519	<1e-4	sll0157	GO:0006793	<1e-4	sll0793	GO:0015399	<1e-4	slr5116	GO:0043226	<1e-4	slr0581	GO:0017111	0.1094

slr0479	GO:0019752	<1e-4	slr0505	GO:0009199	<1e-4	sll1692	GO:0043167	<1e-4	slr0270	GO:0044422	<1e-4	slr6015	GO:0017111	0.1094
slr6028	GO:0009991	<1e-4	sll0265	GO:0051179	<1e-4	slr0607	GO:0005342	<1e-4	ssl0410	GO:0044444	<1e-4	sll7043	GO:0017111	0.1094
sll5034	GO:0051347	<1e-4	slr0888	GO:0050789	<1e-4	ssl1417	GO:0015077	<1e-4	slr1241	GO:0044425	<1e-4	sll0397	GO:0017111	0.1094
slr5126	GO:0031326	<1e-4	sll0786	GO:0090304	<1e-4	sml0011	GO:0015291	<1e-4	slr2004	GO:0044446	<1e-4	sll0168	GO:0017111	0.1094
slr1315	GO:0051179	<1e-4	slr1187	GO:0019752	<1e-4	sll0751	GO:0015293	<1e-4	sll0608	GO:0005575	<1e-4	slr1342	GO:0017111	0.1094
slr1235	GO:0006066	<1e-4	slr1914	GO:0048519	<1e-4	slr0959	GO:0022890	<1e-4	sll1477	GO:0005575	<1e-4	sll1203	GO:0017111	0.1094
slr1442	GO:0045184	<1e-4	sll0191	GO:0016052	<1e-4	slr1790	GO:0043492	<1e-4	ssl1300	GO:0044444	<1e-4	ssr1951	GO:0017111	0.1094
slr0317	GO:0019637	<1e-4	sll1396	GO:0071496	<1e-4	sll0590	GO:0016741	<1e-4	slr0498	GO:0044424	<1e-4	slr1270	GO:0017111	0.1094
ssr6083	GO:0044282	<1e-4	sll7031	GO:0045184	<1e-4	sll1573	GO:0015267	<1e-4	slr0695	GO:0044444	<1e-4	sll1396	GO:0017111	0.1094
sll0752	GO:0006811	<1e-4	slr1266	GO:0052188	<1e-4	slr0270	GO:0043167	<1e-4	slr1107	GO:0044444	<1e-4	slr7096	GO:0017111	0.1094
sll0047	GO:0019362	<1e-4	slr1866	GO:0006720	<1e-4	slr1240	GO:0042623	<1e-4	sll5109	GO:0043226	<1e-4	ssr6046	GO:0017111	0.1094
sll1504	GO:0008150	<1e-4	sll0314	GO:0044255	<1e-4	sll1656	GO:0016818	<1e-4	ssl1045	GO:0005575	<1e-4	sll0218	GO:0017111	0.1094
slr1161	GO:0019222	<1e-4	slr1173	GO:0050794	<1e-4	slr7012	GO:0015077	<1e-4	slr0291	GO:0044446	<1e-4	slr1276	GO:0017111	0.1094
sll0405	GO:0009892	<1e-4	ssr3409	GO:0009394	<1e-4	slr1413	GO:0016462	<1e-4	sll1123	GO:0044464	<1e-4	ssr2317	GO:0017111	0.1094
sll1239	GO:0051234	<1e-4	ssl5129	GO:0006721	<1e-4	sll0266	GO:0022890	<1e-4	ssr6079	GO:0044464	<1e-4	slr1863	GO:0017111	0.1094
slr1964	GO:0006733	<1e-4	sll5004	GO:0009991	<1e-4	slr1315	GO:0022890	<1e-4	slr0351	GO:0043226	<1e-4	slr6008	GO:0017111	0.1094
sll1640	GO:0009161	<1e-4	slr1194	GO:0051171	<1e-4	sll0328	GO:0015293	<1e-4	sll1251	GO:0044446	<1e-4	sll0853	GO:0017111	0.1094
slr0770	GO:0043170	<1e-4	ssr2843	GO:0034654	<1e-4	slr0408	GO:0043492	<1e-4	sll0647	GO:0005575	<1e-4	slr0870	GO:0017111	0.1094
sll0811	GO:0009991	<1e-4	slr2073	GO:0006753	<1e-4	slr1534	GO:0016462	<1e-4	sll1455	GO:0044425	<1e-4	slr7095	GO:0017111	0.1094
slr2105	GO:0051347	<1e-4	slr0111	GO:0046394	<1e-4	slr1207	GO:0060089	<1e-4	slr1623	GO:0044446	<1e-4	slr6006	GO:0017111	0.1094
sll1552	GO:0043549	<1e-4	ssr0657	GO:0055086	<1e-4	slr1450	GO:0022892	<1e-4	slr1900	GO:0005575	<1e-4	sll8033	GO:0017111	0.1094
sll1071	GO:0022607	<1e-4	slr0971	GO:0006811	<1e-4	sll0696	GO:0016817	<1e-4	slr1437	GO:0043226	<1e-4	slr0168	GO:0017111	0.1094
ssr2142	GO:0009056	<1e-4	sll8027	GO:0022607	<1e-4	ssl0109	GO:0043167	<1e-4	sll1552	GO:0044444	<1e-4	slr0142	GO:0017111	0.1094
sll0547	GO:0009262	<1e-4	sll0572	GO:0044255	<1e-4	ssr2972	GO:0015077	<1e-4	sll0804	GO:0005575	<1e-4	sll1960	GO:0017111	0.1094
sll1654	GO:0006091	<1e-4	sll1052	GO:0006721	<1e-4	ssr7017	GO:0016818	<1e-4	sll1608	GO:0044422	<1e-4	slr1472	GO:0017111	0.1094
sll1570	GO:0044248	<1e-4	slr1261	GO:0048518	<1e-4	sll5026	GO:0016817	<1e-4	slr0812	GO:0044446	<1e-4	slr7092	GO:0017111	0.1094
sll1389	GO:0055082	<1e-4	slr2144	GO:0006811	<1e-4	sll1025	GO:0043167	<1e-4	slr6100	GO:0044424	<1e-4	slr1813	GO:0017111	0.1094
slr0731	GO:0071554	<1e-4	sll0448	GO:0060255	<1e-4	slr0386	GO:0022890	<1e-4	sll1638	GO:0044446	<1e-4	slr1768	GO:0017111	0.1094
slr6021	GO:0051171	<1e-4	sll0284	GO:0052111	<1e-4	slr6101	GO:0015293	<1e-4	slr0082	GO:0044424	<1e-4	ssr6079	GO:0017111	0.1094
slr1183	GO:0044275	<1e-4	sll0735	GO:0072524	<1e-4	slr7081	GO:0016741	<1e-4	slr1261	GO:0043226	<1e-4	slr6004	GO:0017111	0.1094
sll0225	GO:0006163	<1e-4	slr5119	GO:0034641	<1e-4	sll1095	GO:0015405	<1e-4	ssl7007	GO:0005575	<1e-4	slr0049	GO:0017111	0.1094
ssl5129	GO:0051171	<1e-4	slr0269	GO:0009987	<1e-4	slr0516	GO:0022892	<1e-4	sll1547	GO:0044424	<1e-4	slr7014	GO:0017111	0.1094
ssr6048	GO:0006766	<1e-4	sll1455	GO:0006631	<1e-4	sll1784	GO:0043167	<1e-4	ssl1498	GO:0044446	<1e-4	slr1273	GO:0017111	0.1094
sll8007	GO:0006163	<1e-4	sll7077	GO:0006720	<1e-4	sll0175	GO:0015291	<1e-4	ssr7072	GO:0044464	<1e-4	slr7025	GO:0017111	0.1094
slr0144	GO:0009124	<1e-4	sll1399	GO:0050794	<1e-4	sll0810	GO:0022892	<1e-4	slr0610	GO:0044446	<1e-4	sll0658	GO:0017111	0.1094
slr5112	GO:0009262	<1e-4	slr1187	GO:0009262	<1e-4	sll0905	GO:0008171	<1e-4	sll7066	GO:0044446	<1e-4	sll7066	GO:0017111	0.1094
slr0269	GO:0052188	<1e-4	ssl5007	GO:0050794	<1e-4	slr0771	GO:0022892	<1e-4	slr6067	GO:0044422	<1e-4	sll8007	GO:0017111	0.1094
slr0728	GO:0051347	<1e-4	sll0359	GO:0071842	<1e-4	ssl0461	GO:0016462	<1e-4	sll0327	GO:0044422	<1e-4	sll0423	GO:0017111	0.1094
slr0207	GO:0019222	<1e-4	slr1790	GO:0031640	<1e-4	slr0668	GO:0015405	<1e-4	sll0505	GO:0044446	<1e-4	slr6064	GO:0017111	0.1094
slr2032	GO:0044260	<1e-4	slr1600	GO:0043170	<1e-4	slr0356	GO:0016817	<1e-4	ssl5008	GO:0044444	<1e-4	sll0406	GO:0017111	0.1094
sll5063	GO:0050896	<1e-4	slr0157	GO:0050794	<1e-4	sll0296	GO:0016818	<1e-4	slr0386	GO:0043226	<1e-4	slr6065	GO:0017111	0.1094
slr0937	GO:0006732	<1e-4	slr1073	GO:0009309	<1e-4	ssr6083	GO:0003674	<1e-4	slr1690	GO:0005575	<1e-4	slr0199	GO:0017111	0.1094
slr2110	GO:0044275	<1e-4	sll1925	GO:0009117	<1e-4	slr0552	GO:0015405	<1e-4	sll0168	GO:0044422	<1e-4	ssr6085	GO:0017111	0.1094
sll1186	GO:0009991	<1e-4	slr0065	GO:0006220	<1e-4	slr6008	GO:0022857	<1e-4	sll0309	GO:0044422	<1e-4	slr1397	GO:0017111	0.1094
slr1437	GO:0072521	<1e-4	sll1247	GO:0006793	<1e-4	sll0756	GO:0016817	<1e-4	slr1814	GO:0044444	<1e-4	slr0145	GO:0017111	0.1094
sll8007	GO:0006082	<1e-4	slr1315	GO:0046128	<1e-4	slr1290	GO:0022832	<1e-4	slr0702	GO:0044422	<1e-4	sll5046	GO:0017111	0.1094
slr2117	GO:0006732	<1e-4	slr1940	GO:0072527	<1e-4	slr0058	GO:0008171	<1e-4	sll8011	GO:0044446	<1e-4	ssl1792	GO:0017111	0.1094
ssl0294	GO:0019752	<1e-4	ssl3379	GO:0080090	<1e-4	sll0615	GO:0022838	<1e-4	slr1050	GO:0005575	<1e-4	slr5018	GO:0017111	0.1094
slr1570	GO:0009057	<1e-4	sll0499	GO:0048878	<1e-4	slr5118	GO:0015291	<1e-4	slr1628	GO:0044425	<1e-4	slr0960	GO:0017111	0.1094
slr0921	GO:0009892	<1e-4	sll1239	GO:0090304	<1e-4	sll0156	GO:0005342	<1e-4	slr2042	GO:0005575	<1e-4	slr1721	GO:0017111	0.1094
slr0712	GO:0048878	<1e-4	slr1047	GO:0006576	<1e-4	slr1177	GO:0008171	<1e-4	slr6009	GO:0044446	<1e-4	slr1907	GO:0017111	0.1094
sll1447	GO:0051186	<1e-4	slr2032	GO:0034641	<1e-4	sll1106	GO:0015291	<1e-4	slr0458	GO:0044444	<1e-4	ssl2471	GO:0017111	0.1094
slr2032	GO:0009892	<1e-4	slr0914	GO:0009141	<1e-4	slr1307	GO:0016462	<1e-4	ssr1552	GO:0044446	<1e-4	slr0784	GO:0017111	0.1094
sll0413	GO:0045184	<1e-4	ssr3572	GO:0031323	<1e-4	ssl3615	GO:0015291	<1e-4	slr1376	GO:0005575	<1e-4	sll0265	GO:0017111	0.1094

sll1891	GO:0045184	<1e-4	slr0285	GO:0009117	<1e-4	sll1174	GO:0022857	<1e-4	slr5116	GO:0044444	<1e-4	ssr6032	GO:0017111	0.1094
slr2092	GO:0009199	<1e-4	slr0607	GO:0009987	<1e-4	slr1083	GO:0003674	<1e-4	sll1738	GO:0005575	<1e-4	sll0762	GO:0017111	0.1094
slr1799	GO:0009199	<1e-4	slr0400	GO:0006720	<1e-4	ssr0332	GO:0016462	<1e-4	slr6015	GO:0044425	<1e-4	sll8002	GO:0017111	0.1094
slr0521	GO:0050896	<1e-4	slr0505	GO:0009126	<1e-4	slr0865	GO:0015293	<1e-4	slr0181	GO:0044422	<1e-4	slr1179	GO:0017111	0.1094
slr0852	GO:0072522	<1e-4	slr6080	GO:0045184	<1e-4	slr1896	GO:0015399	<1e-4	ssl1577	GO:0044424	<1e-4	sll1880	GO:0017111	0.1094
slr0272	GO:0048518	<1e-4	slr1431	GO:0010556	<1e-4	sll0198	GO:0015075	<1e-4	sll1373	GO:0043226	<1e-4	ssl5129	GO:0017111	0.1094
sll0857	GO:0052111	<1e-4	slr1959	GO:0044106	<1e-4	slr0103	GO:0022832	<1e-4	sll1874	GO:0044424	<1e-4	slr0146	GO:0017111	0.1094
sll0980	GO:0009262	<1e-4	slr0238	GO:0044003	<1e-4	ssl5096	GO:0015405	<1e-4	slr1667	GO:0044464	<1e-4	sll1252	GO:0017111	0.1094
slr1178	GO:0006766	<1e-4	slr1956	GO:0006220	<1e-4	ssr2972	GO:0016462	<1e-4	sll1130	GO:0005575	<1e-4	sll1766	GO:0017111	0.1094
slr0667	GO:0034660	<1e-4	sll1388	GO:0006811	<1e-4	sll0499	GO:0015267	<1e-4	slr0594	GO:0005575	<1e-4	sll0609	GO:0017111	0.1094
sll1355	GO:0009161	<1e-4	sll0553	GO:0008150	<1e-4	ssl5113	GO:0015267	<1e-4	sll8019	GO:0044444	<1e-4	slr7080	GO:0017111	0.1094
slr8022	GO:0044275	<1e-4	slr1670	GO:0043436	<1e-4	slr2118	GO:0016818	<1e-4	sll5044	GO:0044425	<1e-4	sll1389	GO:0017111	0.1094
ssr2554	GO:0009132	<1e-4	slr7100	GO:0090304	<1e-4	sll7087	GO:0043167	<1e-4	slr0479	GO:0044422	<1e-4	ssl0312	GO:0017111	0.1094
ssl7038	GO:0071842	<1e-4	sll1318	GO:0006766	<1e-4	slr1854	GO:0022836	<1e-4	sll7070	GO:0044424	<1e-4	sll7064	GO:0017111	0.1094
slr1415	GO:0005996	<1e-4	sll8019	GO:0019751	<1e-4	slr5037	GO:0022838	<1e-4	slr0935	GO:0044446	<1e-4	slr1419	GO:0017111	0.1094
slr2119	GO:0006721	<1e-4	sml0011	GO:0042180	<1e-4	ssl0352	GO:0016741	<1e-4	ssr2009	GO:0044425	<1e-4	sll1511	GO:0017111	0.1094
slr1530	GO:0016052	<1e-4	slr0325	GO:0034660	<1e-4	slr0397	GO:0022838	<1e-4	slr5077	GO:0044446	<1e-4	sll0810	GO:0009058	0.1071
slr6016	GO:0006733	<1e-4	sll1390	GO:0031640	<1e-4	ssl7039	GO:0016746	<1e-4	sll5128	GO:0044422	<1e-4	sll0449	GO:0009058	0.1071
slr1681	GO:0009165	<1e-4	sll1659	GO:0009144	<1e-4	sll0854	GO:0008171	<1e-4	slr0271	GO:0044424	<1e-4	slr0801	GO:0009058	0.1071
slr1339	GO:0009123	<1e-4	slr0058	GO:0046394	<1e-4	ssl0832	GO:0043167	<1e-4	ssr2611	GO:0044425	<1e-4	slr1122	GO:0009058	0.1071
slr1419	GO:0051234	<1e-4	slr2080	GO:0033013	<1e-4	sll1763	GO:0022892	<1e-4	sll0688	GO:0005575	<1e-4	slr1809	GO:0009058	0.1071
slr6066	GO:0043436	<1e-4	slr0145	GO:0006721	<1e-4	sll0525	GO:0022803	<1e-4	ssr0657	GO:0044422	<1e-4	sll1730	GO:0009058	0.1071
slr6051	GO:0031640	<1e-4	slr2038	GO:0055082	<1e-4	slr0695	GO:0015405	<1e-4	sll0148	GO:0044424	<1e-4	slr1940	GO:0009058	0.1071
slr0654	GO:0005996	<1e-4	slr6068	GO:0051246	<1e-4	sll0740	GO:0008171	<1e-4	sll0888	GO:0044424	<1e-4	slr1413	GO:0009058	0.1071
ssl0109	GO:0006811	<1e-4	slr6009	GO:0072527	<1e-4	ssr0536	GO:0043492	<1e-4	slr0157	GO:0044422	<1e-4	sll1956	GO:0009058	0.1071
slr6101	GO:0051818	<1e-4	slr0149	GO:0006766	<1e-4	slr1207	GO:0022803	<1e-4	slr1601	GO:0044446	<1e-4	sll0503	GO:0009058	0.1071
sll0397	GO:0009893	<1e-4	slr1721	GO:0051186	<1e-4	slr6004	GO:0016818	<1e-4	sll0242	GO:0044444	<1e-4	slr0249	GO:0009058	0.1071
slr0598	GO:0019752	<1e-4	ssl2996	GO:0019362	<1e-4	slr1262	GO:0060089	<1e-4	slr0876	GO:0044444	<1e-4	sll0268	GO:0009058	0.1071
sll1472	GO:0042455	<1e-4	slr5087	GO:0006733	<1e-4	slr1923	GO:0016741	<1e-4	slr0913	GO:0044422	<1e-4	sll0688	GO:0009058	0.1071
sll0242	GO:0044275	<1e-4	sll0910	GO:0071842	<1e-4	sll0147	GO:0016741	<1e-4	sll0236	GO:0044446	<1e-4	ssl8008	GO:0009058	0.1071
slr0787	GO:0019637	<1e-4	slr1034	GO:0018130	<1e-4	sll0508	GO:0015291	<1e-4	sll7050	GO:0005575	<1e-4	slr0650	GO:0009058	0.1071
ssl2717	GO:0034654	<1e-4	slr7098	GO:0034660	<1e-4	slr0082	GO:0016818	<1e-4	sll1424	GO:0044422	<1e-4	slr1053	GO:0009058	0.1071
slr0551	GO:0051171	<1e-4	ssl0353	GO:0051188	<1e-4	slr2032	GO:0008171	<1e-4	sll1135	GO:0044424	<1e-4	slr1977	GO:0009058	0.1071
sll0647	GO:0051186	<1e-4	sll7090	GO:0051716	<1e-4	ssr1114	GO:0022857	<1e-4	ssr7084	GO:0044444	<1e-4	slr1852	GO:0009058	0.1071
slr5023	GO:0006066	<1e-4	slr1340	GO:0072528	<1e-4	slr1235	GO:0060089	<1e-4	sll1691	GO:0044446	<1e-4	ssr3300	GO:0009058	0.1071
sll0525	GO:0022411	<1e-4	ssl5015	GO:0043933	<1e-4	slr6100	GO:0022803	<1e-4	sll1318	GO:0044464	<1e-4	slr2005	GO:0009058	0.1071
slr0708	GO:0005996	<1e-4	slr2117	GO:0051171	<1e-4	sll0727	GO:0043492	<1e-4	sll5033	GO:0044422	<1e-4	slr0962	GO:0009058	0.1071
sll0265	GO:0009117	<1e-4	ssr6078	GO:0019222	<1e-4	slr1628	GO:0022857	<1e-4	sll0981	GO:0044464	<1e-4	sll1873	GO:0009058	0.1071
slr0211	GO:0006066	<1e-4	slr1187	GO:0060255	<1e-4	slr0742	GO:0022892	<1e-4	ssl5068	GO:0005575	<1e-4	slr1827	GO:0009058	0.1071
slr0865	GO:0006793	<1e-4	slr0670	GO:0009132	<1e-4	slr1811	GO:0016462	<1e-4	sll0335	GO:0044424	<1e-4	sll1640	GO:0005488	0.1066
slr1819	GO:0016052	<1e-4	slr1896	GO:0080090	<1e-4	sll1109	GO:0015399	<1e-4	sll1433	GO:0044464	<1e-4	sll0335	GO:0005488	0.1066
slr1699	GO:0048519	<1e-4	slr1790	GO:0080090	<1e-4	sll1862	GO:0043167	<1e-4	slr1307	GO:0044444	<1e-4	sll6055	GO:0005488	0.1066
ssl0739	GO:0042180	<1e-4	slr0976	GO:0046128	<1e-4	sll1068	GO:0043167	<1e-4	sll1424	GO:0044444	<1e-4	ssl7051	GO:0005488	0.1066
slr1505	GO:0034660	<1e-4	slr1071	GO:0009308	<1e-4	sll0658	GO:0015293	<1e-4	slr0386	GO:0044425	<1e-4	slr1875	GO:0005488	0.1066
sll1186	GO:0009123	<1e-4	slr0935	GO:0071840	<1e-4	ssl2009	GO:0015267	<1e-4	slr1505	GO:0044425	<1e-4	sll0047	GO:0005488	0.1066
sll7070	GO:0043412	<1e-4	slr0695	GO:0005996	<1e-4	sll1254	GO:0015267	<1e-4	slr0050	GO:0044446	<1e-4	sll1926	GO:0005488	0.1066
sll0661	GO:0044003	<1e-4	slr0060	GO:0042451	<1e-4	sll0763	GO:0022838	<1e-4	sll0688	GO:0044444	<1e-4	sll0858	GO:0005488	0.1066
slr1084	GO:0008150	<1e-4	slr0865	GO:0006066	<1e-4	slr1307	GO:0022836	<1e-4	sll0473	GO:0044446	<1e-4	sll0543	GO:0005488	0.1066
sll1913	GO:0042180	<1e-4	slr5111	GO:0006082	<1e-4	slr0552	GO:0016746	<1e-4	slr0812	GO:0044464	<1e-4	ssr2318	GO:0005488	0.1066
sll0479	GO:0019752	<1e-4	slr0192	GO:0044283	<1e-4	ssl7042	GO:0015293	<1e-4	slr1944	GO:0044464	<1e-4	slr7073	GO:0005488	0.1066
slr1301	GO:0006220	<1e-4	sll0565	GO:0046128	<1e-4	slr1101	GO:0022892	<1e-4	slr1577	GO:0044446	<1e-4	ssl5091	GO:0005488	0.1066
sll0931	GO:0052111	<1e-4	sll0473	GO:0046164	<1e-4	sll0909	GO:0016817	<1e-4	ssl0109	GO:0005575	<1e-4	ssl2064	GO:0005488	0.1066
ssr6062	GO:0009308	<1e-4	slr2005	GO:0006066	<1e-4	slr0810	GO:0022892	<1e-4	slr2032	GO:0005575	<1e-4	sll1142	GO:0005488	0.1066
slr0598	GO:0034641	<1e-4	sll1965	GO:0043436	<1e-4	slr7011	GO:0016818	<1e-4	sll5004	GO:0044446	<1e-4	ssr0761	GO:0005488	0.1066

slr0376	GO:0019751	<1e-4	sll0447	GO:0019362	<1e-4	ssr2803	GO:0015293	<1e-4	sll1429	GO:0044422	<1e-4	sll7031	GO:0005488	0.1066
slr7080	GO:0009987	<1e-4	slr0912	GO:0009117	<1e-4	slr0598	GO:0005342	<1e-4	ssl5129	GO:0044444	<1e-4	slr5037	GO:0005488	0.1066
ssr7084	GO:0042180	<1e-4	slr0613	GO:0019362	<1e-4	sll0478	GO:0022890	<1e-4	slr2110	GO:0044422	<1e-4	sll0372	GO:0005488	0.1066
slr1484	GO:0051246	<1e-4	sll1571	GO:0051246	<1e-4	ssl1004	GO:0015405	<1e-4	sll1761	GO:0044425	<1e-4	slr5111	GO:0005488	0.1066
sll1315	GO:0009123	<1e-4	sll1218	GO:0008610	<1e-4	ssl7007	GO:0043167	<1e-4	slr0211	GO:0044444	<1e-4	slr5013	GO:0005488	0.1066
slr0325	GO:0050896	<1e-4	slr1767	GO:0006576	<1e-4	slr7073	GO:0043492	<1e-4	ssl0738	GO:0043226	<1e-4	ssr0109	GO:0005488	0.1066
slr1025	GO:0009199	<1e-4	slr1865	GO:0055082	<1e-4	sll1252	GO:0015077	<1e-4	sll1858	GO:0044444	<1e-4	slr1807	GO:0005488	0.1066
slr1601	GO:0050789	<1e-4	sll0479	GO:0009987	<1e-4	slr1398	GO:0042623	<1e-4	slr0845	GO:0005575	<1e-4	sll1373	GO:0005488	0.1066
slr6100	GO:0006082	<1e-4	slr1363	GO:0046907	<1e-4	ssl1520	GO:0022836	<1e-4	sll1130	GO:0043226	<1e-4	slr1704	GO:0005488	0.1066
sll0167	GO:0009126	<1e-4	sll1247	GO:0009991	<1e-4	sll0608	GO:0022832	<1e-4	slr1194	GO:0044425	<1e-4	slr0712	GO:0005488	0.1066
sll0071	GO:0022607	<1e-4	ssr2615	GO:0009057	<1e-4	sll1424	GO:0015077	<1e-4	sll1192	GO:0044444	<1e-4	slr5112	GO:0005488	0.1066
slr1914	GO:0009132	<1e-4	slr1699	GO:0055114	<1e-4	slr1495	GO:0042623	<1e-4	ssl1004	GO:0044446	<1e-4	ssr7093	GO:0005488	0.1066
sll1531	GO:0044003	<1e-4	slr1429	GO:0006576	<1e-4	slr0887	GO:0015293	<1e-4	sll0176	GO:0044446	<1e-4	sll7087	GO:0005488	0.1066
sll1476	GO:0051716	<1e-4	sll0563	GO:0006733	<1e-4	ssl7074	GO:0022832	<1e-4	sll1505	GO:0044424	<1e-4	sll1321	GO:0005488	0.1066
sll0007	GO:0022607	<1e-4	slr1222	GO:0043549	<1e-4	ssl0738	GO:0016817	<1e-4	ssl3573	GO:0044424	<1e-4	sll1717	GO:0005488	0.1066
ssl0294	GO:0009263	<1e-4	sll0702	GO:0034654	<1e-4	sll0284	GO:0015405	<1e-4	slr7082	GO:0044425	<1e-4	sll0062	GO:0005488	0.1066
sll1630	GO:0006631	<1e-4	sll1765	GO:0034660	<1e-4	slr1627	GO:0016817	<1e-4	ssl7022	GO:0005575	<1e-4	sll1882	GO:0005488	0.1066
slr6065	GO:0071842	<1e-4	ssl2384	GO:0043549	<1e-4	sll1380	GO:0022836	<1e-4	ssl0738	GO:0044422	<1e-4	ssl1690	GO:0005488	0.1066
ssr1499	GO:0071496	<1e-4	ssl0461	GO:0019222	<1e-4	slr6100	GO:0015267	<1e-4	ssl5114	GO:0044464	<1e-4	ssl7038	GO:0005488	0.1066
ssl1255	GO:0048523	<1e-4	slr1270	GO:0051716	<1e-4	slr0262	GO:0015077	<1e-4	sll7086	GO:0044464	<1e-4	ssl7042	GO:0005488	0.1066
sll1873	GO:0008150	<1e-4	sll1389	GO:0044255	<1e-4	ssl8008	GO:0016741	<1e-4	slr1674	GO:0044446	<1e-4	sll1573	GO:0005488	0.1066
sll0479	GO:0055086	<1e-4	slr2110	GO:0009144	<1e-4	sll0449	GO:0022890	<1e-4	sll0743	GO:0044464	<1e-4	slr0651	GO:0005488	0.1066
slr0619	GO:0034641	<1e-4	slr0514	GO:0006066	<1e-4	slr1275	GO:0015293	<1e-4	ssr6079	GO:0005575	<1e-4	slr0588	GO:0005488	0.1066
ssl1918	GO:0033013	<1e-4	sll1289	GO:0019752	<1e-4	ssr2047	GO:0042623	<1e-4	slr1638	GO:0005575	<1e-4	slr0270	GO:0005488	0.1066
slr0263	GO:0006811	<1e-4	ssl8028	GO:0016053	<1e-4	slr1814	GO:0015077	<1e-4	ssl0353	GO:0043226	<1e-4	sll0031	GO:0005488	0.1066
ssr2975	GO:0071840	<1e-4	sll0354	GO:0006811	<1e-4	ssl3549	GO:0016462	<1e-4	sll0478	GO:0044425	<1e-4	slr1951	GO:0005488	0.1066
sll0558	GO:0080090	<1e-4	slr0708	GO:0051234	<1e-4	sll6055	GO:0043167	<1e-4	slr0960	GO:0044424	<1e-4	ssl2069	GO:0005488	0.1066
slr0103	GO:0006721	<1e-4	slr0870	GO:0050794	<1e-4	slr1107	GO:0016741	<1e-4	ssr0657	GO:0044425	<1e-4	slr1570	GO:0005488	0.1066
ssl5008	GO:0022607	<1e-4	sll1511	GO:0008610	<1e-4	sll1527	GO:0005342	<1e-4	slr1117	GO:0044446	<1e-4	ssr1558	GO:0005488	0.1066
slr1935	GO:0008150	<1e-4	slr1790	GO:0009142	<1e-4	slr5013	GO:0016818	<1e-4	slr7014	GO:0044444	<1e-4	slr1425	GO:0005488	0.1066
slr1900	GO:0071840	<1e-4	slr1082	GO:0022411	<1e-4	sll0614	GO:0015399	<1e-4	slr0404	GO:0044446	<1e-4	slr1737	GO:0005488	0.1066
ssr6030	GO:0006732	<1e-4	sll1530	GO:0019637	<1e-4	slr6074	GO:0022836	<1e-4	slr0723	GO:0044425	<1e-4	slr7100	GO:0005488	0.1066
slr7080	GO:0042455	<1e-4	slr1862	GO:0016054	<1e-4	slr1767	GO:0043492	<1e-4	slr1073	GO:0044422	<1e-4	ssl7022	GO:0005488	0.1066
sll0167	GO:0042455	<1e-4	sll1049	GO:0050801	<1e-4	sll5089	GO:0008171	<1e-4	slr1047	GO:0044446	<1e-4	slr0181	GO:0005488	0.1066
slr1885	GO:0006066	<1e-4	sll1583	GO:0009991	<1e-4	slr7092	GO:0022857	<1e-4	slr1847	GO:0005575	<1e-4	sll1911	GO:0005488	0.1066
ssr2754	GO:0019751	<1e-4	slr0505	GO:0009309	<1e-4	ssr2201	GO:0015267	<1e-4	slr7024	GO:0044444	<1e-4	slr0976	GO:0005488	0.1066
sll5063	GO:0042180	<1e-4	sll0815	GO:0050794	<1e-4	slr1107	GO:0015267	<1e-4	sll1692	GO:0005575	<1e-4	sll0736	GO:0005488	0.1066
sll1321	GO:0009124	<1e-4	slr1819	GO:0071842	<1e-4	sll8011	GO:0016741	<1e-4	slr1927	GO:0043226	<1e-4	sll1233	GO:0005488	0.1066
slr1721	GO:0044255	<1e-4	ssl1552	GO:0019222	<1e-4	slr1261	GO:0016462	<1e-4	slr6033	GO:0044444	<1e-4	slr0345	GO:0005488	0.1066
sll1106	GO:0046164	<1e-4	ssl0312	GO:0072521	<1e-4	sll0265	GO:0016817	<1e-4	sll0760	GO:0044425	<1e-4	ssl5100	GO:0005488	0.1066
slr6005	GO:0072528	<1e-4	slr0740	GO:0018130	<1e-4	slr0431	GO:0015077	<1e-4	ssr1407	GO:0044464	<1e-4	ssl5008	GO:0005488	0.1066
sll5034	GO:0044255	<1e-4	sll1162	GO:0009260	<1e-4	sll5089	GO:0022803	<1e-4	sll1530	GO:0044444	<1e-4	ssr6099	GO:0005488	0.1066
slr2071	GO:0006066	<1e-4	slr0770	GO:0009141	<1e-4	slr1097	GO:0022892	<1e-4	sll7063	GO:0005575	<1e-4	ssr6086	GO:0005488	0.1066
slr0656	GO:0051818	<1e-4	sll1399	GO:0009260	<1e-4	sll0827	GO:0022836	<1e-4	slr1327	GO:0044425	<1e-4	sll1158	GO:0005488	0.1066
sll1239	GO:0051171	<1e-4	slr1767	GO:0048518	<1e-4	sll0505	GO:0043492	<1e-4	sll0355	GO:0044425	<1e-4	sll1702	GO:0005488	0.1066
sll1681	GO:0046394	<1e-4	slr0602	GO:0031326	<1e-4	ssr2553	GO:0016817	<1e-4	sll1319	GO:0044425	<1e-4	slr0967	GO:0005488	0.1066
sll1219	GO:0019637	<1e-4	sll0930	GO:0009889	<1e-4	ssr2009	GO:0022857	<1e-4	ssl1918	GO:0044444	<1e-4	sll8040	GO:0005488	0.1066
sll1832	GO:0009132	<1e-4	slr0489	GO:0009259	<1e-4	slr1342	GO:0016817	<1e-4	slr0208	GO:0005575	<1e-4	slr1990	GO:0005488	0.1066
ssr1258	GO:0019362	<1e-4	ssl1417	GO:0065007	<1e-4	slr1726	GO:0016746	<1e-4	slr1236	GO:0044424	<1e-4	slr0238	GO:0005488	0.1066
sll0325	GO:0042451	<1e-4	slr0869	GO:0051188	<1e-4	ssr6002	GO:0022803	<1e-4	slr2101	GO:0005575	<1e-4	ssr6089	GO:0005488	0.1066
slr0211	GO:0009259	<1e-4	sll0168	GO:0046483	<1e-4	sll1321	GO:0015077	<1e-4	sll1761	GO:0044464	<1e-4	ssr2975	GO:0005488	0.1066
slr6008	GO:0044106	<1e-4	sll0933	GO:0009262	<1e-4	ssr3571	GO:0008171	<1e-4	slr1668	GO:0044446	<1e-4	slr0755	GO:0005488	0.1066
ssl5113	GO:0050789	<1e-4	slr0400	GO:0042451	<1e-4	ssl3573	GO:0022838	<1e-4	sll1925	GO:0005575	<1e-4	sll1068	GO:0005488	0.1066
sll1002	GO:0009263	<1e-4	sll1272	GO:0006721	<1e-4	slr0569	GO:0003674	<1e-4	slr1438	GO:0044422	<1e-4	slr1886	GO:0005488	0.1066

ssr3300	GO:0005996	<1e-4	slr1726	GO:0048523	<1e-4	slr6074	GO:0016746	<1e-4	slr6057	GO:0005575	<1e-4	slr1577	GO:0005488	0.1066
slr0364	GO:0043549	<1e-4	ssl2069	GO:0022607	<1e-4	slr1814	GO:0042623	<1e-4	sll1267	GO:0044425	<1e-4	ssr2802	GO:0005488	0.1066
sll0751	GO:0006091	<1e-4	slr6073	GO:0044003	<1e-4	sll0781	GO:0016462	<1e-4	sll0743	GO:0044422	<1e-4	sll0625	GO:0005488	0.1066
slr2084	GO:0042455	<1e-4	sll1853	GO:0019362	<1e-4	sll0496	GO:0015293	<1e-4	slr1813	GO:0005575	<1e-4	slr1670	GO:0005488	0.1066
slr1657	GO:0043412	<1e-4	slr1303	GO:0048522	<1e-4	ssl0788	GO:0008171	<1e-4	slr1718	GO:0044424	<1e-4	slr5127	GO:0005488	0.1066
ssr0336	GO:0044283	<1e-4	sll1054	GO:0043170	<1e-4	sll1447	GO:0015405	<1e-4	sll5069	GO:0044446	<1e-4	sll1036	GO:0005488	0.1066
ssr1425	GO:0009991	<1e-4	sll7055	GO:0048519	<1e-4	ssr2554	GO:0016817	<1e-4	slr2025	GO:0005575	<1e-4	ssr6030	GO:0005488	0.1066
ssl0483	GO:0044248	<1e-4	sll0397	GO:0051188	<1e-4	ssl7022	GO:0022832	<1e-4	sll1500	GO:0044424	<1e-4	slr0291	GO:0005488	0.1066
sll1714	GO:0009132	<1e-4	sll1660	GO:0072528	<1e-4	sll0103	GO:0022803	<1e-4	slr1913	GO:0044422	<1e-4	sll0565	GO:0005488	0.1066
slr0149	GO:0052188	<1e-4	sll5004	GO:0071842	<1e-4	slr0168	GO:0022836	<1e-4	slr0885	GO:0005575	<1e-4	sll0552	GO:0005488	0.1066
slr0291	GO:0051716	<1e-4	slr2144	GO:0019752	<1e-4	sll1219	GO:0003674	<1e-4	sll0156	GO:0044425	<1e-4	ssl1918	GO:0005488	0.1066
slr8044	GO:0034641	<1e-4	slr0810	GO:0008610	<1e-4	ssl5103	GO:0022890	<1e-4	sll1862	GO:0044444	<1e-4	slr0326	GO:0005488	0.1066
sll1021	GO:0019222	<1e-4	slr1474	GO:0055082	<1e-4	slr1073	GO:0043492	<1e-4	sll0857	GO:0044444	<1e-4	slr1819	GO:0005488	0.1066
slr1033	GO:0009057	<1e-4	slr1816	GO:0008150	<1e-4	sll5132	GO:0005342	<1e-4	slr1070	GO:0044425	<1e-4	sll1388	GO:0005488	0.1066
ssl2749	GO:0051716	<1e-4	sll1201	GO:0034660	<1e-4	sll1570	GO:0016741	<1e-4	slr1964	GO:0044422	<1e-4	slr2103	GO:0005488	0.1066
sll5097	GO:0006793	<1e-4	slr0273	GO:0008150	<1e-4	ssr8013	GO:0015399	<1e-4	slr1577	GO:0043226	<1e-4	ssl8039	GO:0005488	0.1066
sll1660	GO:0009059	<1e-4	slr0596	GO:0050789	<1e-4	slr1648	GO:0022892	<1e-4	sll1476	GO:0044464	<1e-4	slr0948	GO:0005488	0.1066
sll0837	GO:0044271	<1e-4	slr0582	GO:0043436	<1e-4	sll1891	GO:0015075	<1e-4	slr0458	GO:0044424	<1e-4	slr1438	GO:0005488	0.1066
slr6087	GO:0044281	<1e-4	ssl2065	GO:0009161	<1e-4	sll0446	GO:0022892	<1e-4	sll0737	GO:0044446	<1e-4	slr0885	GO:0005488	0.1066
sll1880	GO:0006066	<1e-4	sll1659	GO:0009309	<1e-4	sll1634	GO:0015075	<1e-4	slr1084	GO:0044444	<1e-4	sll0496	GO:0005488	0.1066
sll0478	GO:0046394	<1e-4	sll0068	GO:0006811	<1e-4	sll0328	GO:0016462	<1e-4	ssr7072	GO:0005575	<1e-4	sll1834	GO:0005488	0.1066
slr0771	GO:0031323	<1e-4	sll1630	GO:0009132	<1e-4	slr1470	GO:0016462	<1e-4	slr0509	GO:0044424	<1e-4	slr2000	GO:0005488	0.1066
ssr6078	GO:0046164	<1e-4	slr1927	GO:0008610	<1e-4	slr0818	GO:0003674	<1e-4	ssl2138	GO:0044422	<1e-4	slr6087	GO:0005488	0.1066
slr1384	GO:0048519	<1e-4	slr5077	GO:0006066	<1e-4	sll5003	GO:0022838	<1e-4	slr1812	GO:0043226	<1e-4	slr0397	GO:0005488	0.1066
slr0921	GO:0044260	<1e-4	slr1183	GO:0009142	<1e-4	slr1863	GO:0015293	<1e-4	slr6033	GO:0044464	<1e-4	slr6033	GO:0005488	0.1066
ssr7036	GO:0034654	<1e-4	sll1389	GO:0072522	<1e-4	slr1704	GO:0022890	<1e-4	slr0962	GO:0044424	<1e-4	slr0863	GO:0005488	0.1066
sll6052	GO:0009117	<1e-4	slr6065	GO:0009987	<1e-4	slr0519	GO:0015291	<1e-4	slr1376	GO:0044444	<1e-4	sll1979	GO:0005488	0.1066
sll1464	GO:0009117	<1e-4	slr0871	GO:0071496	<1e-4	sll0098	GO:0016741	<1e-4	sll1766	GO:0043226	<1e-4	slr0453	GO:0005488	0.1066
sll0661	GO:0071554	<1e-4	sll1464	GO:0072521	<1e-4	slr1546	GO:0022838	<1e-4	slr1343	GO:0044425	<1e-4	sll1583	GO:0005488	0.1066
slr1935	GO:0009262	<1e-4	sll1025	GO:0009308	<1e-4	slr0398	GO:0016746	<1e-4	slr1186	GO:0044424	<1e-4	slr2071	GO:0005488	0.1066
sll1531	GO:0009263	<1e-4	sll0071	GO:0043170	<1e-4	slr1851	GO:0022857	<1e-4	ssr8013	GO:0005575	<1e-4	sll0160	GO:0005488	0.1066
slr0468	GO:0008150	<1e-4	slr1438	GO:0048518	<1e-4	slr7091	GO:0003674	<1e-4	slr0503	GO:0044425	<1e-4	ssr5106	GO:0005488	0.1066
sll1068	GO:0051179	<1e-4	ssl1004	GO:0006082	<1e-4	slr0765	GO:0022890	<1e-4	sll0837	GO:0044446	<1e-4	slr0516	GO:0005488	0.1066
slr1150	GO:0008150	<1e-4	sll2015	GO:0055114	<1e-4	sll1912	GO:0016818	<1e-4	sll1722	GO:0044464	<1e-4	sll0172	GO:0005488	0.1066
slr0503	GO:0006220	<1e-4	ssl1464	GO:0072524	<1e-4	sll0031	GO:0022803	<1e-4	sll1267	GO:0044444	<1e-4	sll7050	GO:0005488	0.1066
slr1774	GO:0045184	<1e-4	sll1608	GO:0050896	<1e-4	ssl3451	GO:0016817	<1e-4	ssr7084	GO:0005575	<1e-4	slr1056	GO:0005488	0.1066
slr7094	GO:0044281	<1e-4	slr6090	GO:0055114	<1e-4	sll1692	GO:0022803	<1e-4	sll0149	GO:0044425	<1e-4	ssl7048	GO:0005488	0.1066
sll1265	GO:0019637	<1e-4	slr1394	GO:0009142	<1e-4	slr1142	GO:0022838	<1e-4	slr1718	GO:0005575	<1e-4	ssr6019	GO:0005488	0.1066
ssl2781	GO:0009260	<1e-4	slr8044	GO:0051246	<1e-4	sll0230	GO:0003674	<1e-4	slr0909	GO:0043226	<1e-4	ssl0350	GO:0005488	0.1066
sll1934	GO:0009150	<1e-4	sll1240	GO:0034641	<1e-4	slr1114	GO:0015293	<1e-4	ssr0657	GO:0005575	<1e-4	ssl1300	GO:0005488	0.1066
slr0610	GO:0009987	<1e-4	sll5128	GO:0006733	<1e-4	sll0931	GO:0016817	<1e-4	sll1660	GO:0044464	<1e-4	ssr2067	GO:0005488	0.1066
sll0872	GO:0046164	<1e-4	sll1995	GO:0009057	<1e-4	sll1373	GO:0043167	<1e-4	ssr6002	GO:0043226	<1e-4	ssr2047	GO:0005488	0.1066
ssr3467	GO:0009259	<1e-4	sll7066	GO:0006066	<1e-4	slr0049	GO:0015405	<1e-4	slr1840	GO:0005575	<1e-4	sll0875	GO:0005488	0.1066
sll0703	GO:0072528	<1e-4	slr0801	GO:0009144	<1e-4	sll0413	GO:0015293	<1e-4	sll1504	GO:0044444	<1e-4	sll1049	GO:0005488	0.1066
ssl2384	GO:0046483	<1e-4	slr1619	GO:0022607	<1e-4	sll0590	GO:0008171	<1e-4	slr1840	GO:0044464	<1e-4	slr1442	GO:0005488	0.1066
ssr6026	GO:0006733	<1e-4	slr7010	GO:0055114	<1e-4	ssr6003	GO:0015291	<1e-4	sll1170	GO:0044425	<1e-4	slr1623	GO:0005488	0.1066
sll0787	GO:0016054	<1e-4	sll1735	GO:0046394	<1e-4	ssr5121	GO:0022803	<1e-4	slr0695	GO:0044425	<1e-4	slr1236	GO:0005488	0.1066
ssr2912	GO:0006163	<1e-4	slr0039	GO:0009991	<1e-4	sll1906	GO:0043492	<1e-4	sll8012	GO:0044425	<1e-4	slr1601	GO:0005488	0.1066
slr1287	GO:0006082	<1e-4	sll0369	GO:0044281	<1e-4	sll0283	GO:0022891	<1e-4	slr0208	GO:0044424	<1e-4	ssl5114	GO:0005488	0.1066
slr1900	GO:0046164	<1e-4	sll0230	GO:0044281	<1e-4	slr6039	GO:0042623	<1e-4	ssr1766	GO:0044446	<1e-4	ssl2595	GO:0005488	0.1066
slr0981	GO:0055082	<1e-4	slr1737	GO:0006766	<1e-4	slr1195	GO:0016462	<1e-4	slr1571	GO:0044422	<1e-4	slr0217	GO:0005488	0.1066
ssr3129	GO:0045184	<1e-4	sll7087	GO:0009124	<1e-4	sll1866	GO:0005342	<1e-4	slr1944	GO:0044425	<1e-4	slr1970	GO:0005488	0.1066
slr1702	GO:0009059	<1e-4	slr1896	GO:0052188	<1e-4	sll0678	GO:0022836	<1e-4	sll7066	GO:0043226	<1e-4	sll0670	GO:0005488	0.1066
slr0393	GO:0071840	<1e-4	slr1753	GO:0044106	<1e-4	sll7006	GO:0005342	<1e-4	slr1624	GO:0005575	<1e-4	slr6028	GO:0005488	0.1066

sll1926	GO:0042451	<1e-4	sll0286	GO:0050789	<1e-4	sll1225	GO:0015267	<1e-4	slr6049	GO:0044444	<1e-4	ssl5015	GO:0005488	0.1066
slr0151	GO:0006091	<1e-4	sll1217	GO:0080090	<1e-4	ssr2049	GO:0005342	<1e-4	slr2032	GO:0044425	<1e-4	slr0325	GO:0005488	0.1066
slr0789	GO:0019752	<1e-4	ssl7048	GO:0009260	<1e-4	slr0238	GO:0022803	<1e-4	sll1486	GO:0044446	<1e-4	slr1847	GO:0016787	0.1065
sll1766	GO:0060255	<1e-4	sll0783	GO:0065007	<1e-4	sll0857	GO:0043492	<1e-4	sll0189	GO:0044424	<1e-4	sll0910	GO:0016787	0.1065
ssl7045	GO:0018130	<1e-4	slr5053	GO:0043436	<1e-4	sll0803	GO:0016817	<1e-4	sll0994	GO:0044446	<1e-4	ssl1377	GO:0016787	0.1065
ssr2333	GO:0006163	<1e-4	sll1950	GO:0072524	<1e-4	sll0980	GO:0015077	<1e-4	slr7095	GO:0044424	<1e-4	sll0577	GO:0016787	0.1065
sll1866	GO:0044260	<1e-4	ssr5120	GO:0060255	<1e-4	slr0586	GO:0022892	<1e-4	sll0810	GO:0005575	<1e-4	slr1307	GO:0016787	0.1065
slr1261	GO:0031323	<1e-4	sll0397	GO:0006066	<1e-4	sll1702	GO:0022836	<1e-4	ssr2711	GO:0044444	<1e-4	slr1444	GO:0016787	0.1065
ssr0332	GO:0071840	<1e-4	slr0692	GO:0046907	<1e-4	slr0453	GO:0060089	<1e-4	sll1736	GO:0044425	<1e-4	sll0022	GO:0016787	0.1065
sll1640	GO:0009132	<1e-4	slr0551	GO:0043648	<1e-4	slr0634	GO:0015075	<1e-4	slr6094	GO:0044424	<1e-4	ssl2733	GO:0016787	0.1065
sll1106	GO:0009144	<1e-4	ssl7031	GO:0010468	<1e-4	ssr7072	GO:0022891	<1e-4	ssl2733	GO:0044446	<1e-4	ssr1391	GO:0016787	0.1065
sll0008	GO:0050794	<1e-4	slr1530	GO:0006732	<1e-4	sll0149	GO:0042623	<1e-4	slr0455	GO:0044446	<1e-4	sll0586	GO:0016787	0.1065
slr1152	GO:0006082	<1e-4	sll0939	GO:0009117	<1e-4	sll0419	GO:0015405	<1e-4	sll0803	GO:0043226	<1e-4	slr1098	GO:0016787	0.1065
slr7014	GO:0006082	<1e-4	slr1566	GO:0006732	<1e-4	slr8022	GO:0022836	<1e-4	sll0253	GO:0044422	<1e-4	slr0740	GO:0016787	0.1065
ssr2975	GO:0006631	<1e-4	slr7010	GO:0046483	<1e-4	slr0172	GO:0022832	<1e-4	slr1895	GO:0044425	<1e-4	slr0263	GO:0016787	0.1065
sll0837	GO:0033013	<1e-4	sll1021	GO:0009132	<1e-4	slr0192	GO:0043492	<1e-4	sll0577	GO:0005575	<1e-4	slr0978	GO:0016787	0.1065
sll0803	GO:0019219	<1e-4	ssr0109	GO:0009142	<1e-4	slr1240	GO:0015075	<1e-4	sll5003	GO:0044464	<1e-4	slr0514	GO:0016787	0.1065
slr1338	GO:0052111	<1e-4	sll0505	GO:0044271	<1e-4	slr0667	GO:0005342	<1e-4	slr0269	GO:0044425	<1e-4	slr1047	GO:0016787	0.1065
slr1914	GO:0016054	<1e-4	slr1788	GO:0009262	<1e-4	slr1270	GO:0016818	<1e-4	slr1240	GO:0043226	<1e-4	sll0175	GO:0016787	0.1065
slr6022	GO:0009309	<1e-4	sll0175	GO:0072521	<1e-4	ssl2781	GO:0016462	<1e-4	ssl8028	GO:0044424	<1e-4	slr1865	GO:0016787	0.1065
sll1241	GO:0044003	<1e-4	sll1530	GO:0046164	<1e-4	slr8014	GO:0016817	<1e-4	slr1056	GO:0044424	<1e-4	slr1471	GO:0000166	0.1065
sll1192	GO:0090304	<1e-4	ssr2972	GO:0071554	<1e-4	sll5046	GO:0015399	<1e-4	slr6031	GO:0044424	<1e-4	sll0361	GO:0016787	0.1065
sll0931	GO:0031323	<1e-4	sll1233	GO:0006066	<1e-4	slr6045	GO:0043167	<1e-4	slr0360	GO:0044425	<1e-4	slr0607	GO:0016787	0.1065
sll0793	GO:0050794	<1e-4	sll0688	GO:0031640	<1e-4	sll0572	GO:0016818	<1e-4	slr0609	GO:0044446	<1e-4	sll0192	GO:0016787	0.1065
slr0423	GO:0048523	<1e-4	sll0447	GO:0046483	<1e-4	sll1659	GO:0016817	<1e-4	ssl0787	GO:0005575	<1e-4	slr1726	GO:0016787	0.1065
ssr0102	GO:0009126	<1e-4	slr0059	GO:0043170	<1e-4	slr1699	GO:0022890	<1e-4	slr0157	GO:0043226	<1e-4	sll0183	GO:0016787	0.1065
slr0199	GO:0050794	<1e-4	slr1203	GO:0051716	<1e-4	sll1915	GO:0016817	<1e-4	ssr3572	GO:0044446	<1e-4	slr1767	GO:0016787	0.1065
sll0602	GO:0052188	<1e-4	sll1254	GO:0051716	<1e-4	sll0933	GO:0022836	<1e-4	sll1472	GO:0044464	<1e-4	sll0488	GO:0005488	0.1049
slr0613	GO:0009142	<1e-4	sll1036	GO:0009123	<1e-4	sll7070	GO:0022803	<1e-4	sll0355	GO:0044444	<1e-4	ssr1258	GO:0016787	0.1048
sll0810	GO:0046164	<1e-4	ssr1155	GO:0010468	<1e-4	sll0062	GO:0022838	<1e-4	slr6038	GO:0044446	<1e-4	ssl3291	GO:0016787	0.1048
ssl0483	GO:0044255	<1e-4	sll0864	GO:0044275	<1e-4	sll2015	GO:0022832	<1e-4	slr1565	GO:0044464	<1e-4	sll0269	GO:0016787	0.1048
sll1527	GO:0010468	<1e-4	slr1206	GO:0009263	<1e-4	slr1667	GO:0022857	<1e-4	sll7034	GO:0005575	<1e-4	sll0272	GO:0016787	0.1048
sll7067	GO:0051171	<1e-4	slr0065	GO:0043412	<1e-4	sll0301	GO:0015077	<1e-4	sll0847	GO:0044446	<1e-4	sll1119	GO:0016787	0.1048
sll1658	GO:0043412	<1e-4	ssl5025	GO:0051716	<1e-4	slr0092	GO:0022892	<1e-4	ssr3304	GO:0044444	<1e-4	slr0208	GO:0016787	0.1048
slr1674	GO:0031640	<1e-4	sll1106	GO:0080090	<1e-4	slr1900	GO:0022803	<1e-4	sll5046	GO:0005575	<1e-4	slr1565	GO:0016787	0.1048
ssr6099	GO:0044003	<1e-4	sll0810	GO:0019752	<1e-4	slr1223	GO:0015405	<1e-4	slr1417	GO:0044464	<1e-4	sll0103	GO:0016787	0.1048
slr1964	GO:0006220	<1e-4	sll0765	GO:0046164	<1e-4	slr2105	GO:0015405	<1e-4	sll0785	GO:0044446	<1e-4	slr0919	GO:0016787	0.1048
sll0822	GO:0044275	<1e-4	slr0689	GO:0048518	<1e-4	slr1599	GO:0015291	<1e-4	slr0404	GO:0005575	<1e-4	sll0071	GO:0016787	0.1048
slr1450	GO:0019222	<1e-4	sll0354	GO:0019752	<1e-4	ssl0410	GO:0022836	<1e-4	sll1086	GO:0005575	<1e-4	slr1032	GO:0016787	0.1048
sll0647	GO:0006082	<1e-4	ssr6078	GO:0006082	<1e-4	slr2101	GO:0015267	<1e-4	sll2007	GO:0044464	<1e-4	slr0848	GO:0016787	0.1048
sll1304	GO:0046164	<1e-4	ssl0483	GO:0006066	<1e-4	slr1852	GO:0015293	<1e-4	ssr2802	GO:0044425	<1e-4	sll1250	GO:0016787	0.1048
sll0497	GO:0019752	<1e-4	ssr1407	GO:0019752	<1e-4	slr0287	GO:0016746	<1e-4	slr1365	GO:0044425	<1e-4	ssl0832	GO:0016787	0.1048
slr1819	GO:0044281	<1e-4	sll1573	GO:0009987	<1e-4	sll6052	GO:0043167	<1e-4	slr7037	GO:0005575	<1e-4	slr2038	GO:0016787	0.1048
ssl8028	GO:0046394	<1e-4	sll1784	GO:0080090	<1e-4	sll0242	GO:0043169	<1e-4	slr2060	GO:0044446	<1e-4	slr0980	GO:0016787	0.1048
sll0497	GO:0010556	<1e-4	slr0326	GO:0009260	<1e-4	sll0071	GO:0022836	<1e-4	sll1396	GO:0044424	<1e-4	slr1052	GO:0005215	0.1042
sll5006	GO:0042451	<1e-4	slr0006	GO:0044283	<1e-4	sll0189	GO:0015399	<1e-4	slr1657	GO:0044446	<1e-4	slr1812	GO:0005215	0.1042
ssr8047	GO:0031326	<1e-4	ssr6019	GO:0043933	<1e-4	slr1062	GO:0015405	<1e-4	sll1219	GO:0044444	<1e-4	slr0810	GO:0016787	0.1032
slr5037	GO:0019362	<1e-4	slr0619	GO:0009263	<1e-4	slr0596	GO:0016741	<1e-4	slr0821	GO:0044424	<1e-4	slr1603	GO:0016787	0.1032
sll0518	GO:0006811	<1e-4	sll1389	GO:0022607	<1e-4	slr0184	GO:0016818	<1e-4	slr7023	GO:0044422	<1e-4	slr2092	GO:0016787	0.1032
slr1774	GO:0009308	<1e-4	slr0751	GO:0001932	<1e-4	slr1613	GO:0015405	<1e-4	slr0285	GO:0005575	<1e-4	sll1858	GO:0016787	0.1032
sll0837	GO:0043436	<1e-4	sll1652	GO:0006066	<1e-4	ssl0353	GO:0016462	<1e-4	ssr0102	GO:0044424	<1e-4	slr0341	GO:0016787	0.1032
slr0871	GO:0051188	<1e-4	slr1263	GO:0051716	<1e-4	sll1902	GO:0016746	<1e-4	ssr6027	GO:0005575	<1e-4	sll1737	GO:0016787	0.1032
slr1591	GO:0016052	<1e-4	sll1233	GO:0009165	<1e-4	sll1898	GO:0015077	<1e-4	slr1162	GO:0044464	<1e-4	slr1098	GO:0017111	0.1027
sll1611	GO:0051347	<1e-4	ssl5129	GO:0009987	<1e-4	sll0513	GO:0015405	<1e-4	sll0284	GO:0044464	<1e-4	sll0558	GO:0017111	0.1027

sll0149	GO:0019637	<1e-4	sll1411	GO:0006220	<1e-4	slr0852	GO:0022838	<1e-4	slr1222	GO:0044444	<1e-4	slr0909	GO:0017111	0.1027
slr1896	GO:0051179	<1e-4	ssr5019	GO:0006811	<1e-4	sll1306	GO:0003674	<1e-4	slr2060	GO:0044464	<1e-4	sll0577	GO:0017111	0.1027
slr0742	GO:0009991	<1e-4	sll1611	GO:0052111	<1e-4	sll1608	GO:0015291	<1e-4	sll0740	GO:0043226	<1e-4	ssl2733	GO:0017111	0.1027
slr0872	GO:0005996	<1e-4	ssl2384	GO:0051234	<1e-4	ssr5106	GO:0015291	<1e-4	slr1066	GO:0005575	<1e-4	sll0183	GO:0017111	0.1027
slr0848	GO:0006066	<1e-4	slr1101	GO:0009141	<1e-4	sll1092	GO:0043492	<1e-4	slr0209	GO:0043226	<1e-4	slr1800	GO:0017111	0.1027
sll0400	GO:0006576	<1e-4	sll1640	GO:0090304	<1e-4	sll0414	GO:0022892	<1e-4	slr0264	GO:0044444	<1e-4	ssl1377	GO:0017111	0.1027
slr1071	GO:0006733	<1e-4	sll1024	GO:0044271	<1e-4	sll1757	GO:0003674	<1e-4	slr6044	GO:0044444	<1e-4	slr0923	GO:0017111	0.1027
sll0328	GO:0044275	<1e-4	sll0639	GO:0042180	<1e-4	sll1340	GO:0016818	<1e-4	slr0907	GO:0005575	<1e-4	slr1301	GO:0017111	0.1027
sll1979	GO:0034654	<1e-4	sll1272	GO:0042451	<1e-4	slr0569	GO:0022832	<1e-4	slr0885	GO:0044422	<1e-4	slr0978	GO:0017111	0.1027
slr2052	GO:0051179	<1e-4	sll0192	GO:0042180	<1e-4	slr0521	GO:0022891	<1e-4	slr0317	GO:0044444	<1e-4	sll0732	GO:0017111	0.1027
slr1977	GO:0048518	<1e-4	slr6051	GO:0009987	<1e-4	ssr7017	GO:0015399	<1e-4	sll0238	GO:0043226	<1e-4	slr1726	GO:0017111	0.1027
slr0980	GO:0051171	<1e-4	sll0788	GO:0009394	<1e-4	sll0280	GO:0008171	<1e-4	sll1632	GO:0044424	<1e-4	slr1307	GO:0017111	0.1027
sll0843	GO:0009892	<1e-4	sll0552	GO:0033013	<1e-4	sll5089	GO:0016746	<1e-4	ssl5008	GO:0005575	<1e-4	slr0607	GO:0017111	0.1027
slr0771	GO:0006066	<1e-4	slr0552	GO:0034660	<1e-4	slr0770	GO:0015405	<1e-4	sll0449	GO:0044425	<1e-4	slr1851	GO:0017111	0.1027
ssl1577	GO:0009141	<1e-4	sll1173	GO:0009309	<1e-4	sll0742	GO:0016817	<1e-4	sll0068	GO:0044424	<1e-4	sll0910	GO:0017111	0.1027
slr1306	GO:0071842	<1e-4	slr1339	GO:0043933	<1e-4	slr1591	GO:0022892	<1e-4	sll0597	GO:0044444	<1e-4	sll0586	GO:0017111	0.1027
slr7096	GO:0009117	<1e-4	sll0532	GO:0005996	<1e-4	slr0751	GO:0016741	<1e-4	ssl5096	GO:0044424	<1e-4	slr0479	GO:0017111	0.1027
sll1174	GO:0048519	<1e-4	sll1757	GO:0051171	<1e-4	ssl1046	GO:0015293	<1e-4	slr0770	GO:0044422	<1e-4	slr1767	GO:0017111	0.1027
ssr3532	GO:0051171	<1e-4	ssr3409	GO:0051171	<1e-4	slr0668	GO:0015293	<1e-4	slr5021	GO:0044446	<1e-4	sll0022	GO:0017111	0.1027
slr0146	GO:0006811	<1e-4	slr0930	GO:0090304	<1e-4	slr0392	GO:0016746	<1e-4	sll1825	GO:0043226	<1e-4	slr1847	GO:0017111	0.1027
sll1765	GO:0006066	<1e-4	sll1193	GO:0005996	<1e-4	sll8040	GO:0015291	<1e-4	slr2080	GO:0044425	<1e-4	slr1444	GO:0017111	0.1027
slr1425	GO:0016053	<1e-4	ssl0242	GO:0033013	<1e-4	ssl7042	GO:0015075	<1e-4	slr1397	GO:0044444	<1e-4	slr0263	GO:0017111	0.1027
sll1061	GO:0006082	<1e-4	slr1896	GO:0051716	<1e-4	ssl3615	GO:0022890	<1e-4	ssr1256	GO:0044424	<1e-4	slr0496	GO:0017111	0.1027
sll1352	GO:0008150	<1e-4	slr1218	GO:0071841	<1e-4	sll1939	GO:0022890	<1e-4	slr2073	GO:0005575	<1e-4	slr1811	GO:0017111	0.1027
slr6007	GO:0043549	<1e-4	ssl2384	GO:0009142	<1e-4	sll1173	GO:0022857	<1e-4	sll1158	GO:0044446	<1e-4	ssr1391	GO:0017111	0.1027
sll1378	GO:0051347	<1e-4	sll1060	GO:0008610	<1e-4	slr0184	GO:0016746	<1e-4	sll1775	GO:0044424	<1e-4	sll1446	GO:0016787	0.1007
sll0225	GO:0034654	<1e-4	sll0023	GO:0042451	<1e-4	sll0863	GO:0003674	<1e-4	sll0676	GO:0044425	<1e-4	sll0446	GO:0016787	0.1007
sll1359	GO:0010556	<1e-4	ssr5011	GO:0044260	<1e-4	slr1087	GO:0022891	<1e-4	ssr3410	GO:0044464	<1e-4	slr0818	GO:0016787	0.1007
slr1647	GO:0006811	<1e-4	sll0496	GO:0051818	<1e-4	slr0870	GO:0015075	<1e-4	sll7047	GO:0005575	<1e-4	sll1995	GO:0016787	0.1007
ssr2962	GO:0009309	<1e-4	slr6005	GO:0006163	<1e-4	ssr3122	GO:0016746	<1e-4	slr1913	GO:0044425	<1e-4	slr1773	GO:0016787	0.1007
slr1940	GO:0055114	<1e-4	slr7026	GO:0009123	<1e-4	slr1262	GO:0016818	<1e-4	slr0442	GO:0044422	<1e-4	slr0642	GO:0016787	0.1007
sll0760	GO:0052111	<1e-4	sll0456	GO:0044271	<1e-4	sll1072	GO:0043167	<1e-4	slr1721	GO:0005575	<1e-4	slr1591	GO:0016787	0.1007
ssl5064	GO:0043933	<1e-4	sgl0001	GO:0016052	<1e-4	sll0298	GO:0015077	<1e-4	slr1472	GO:0043226	<1e-4	slr1774	GO:0016787	0.1007
slr0393	GO:0034641	<1e-4	slr1913	GO:0009150	<1e-4	sll1132	GO:0016817	<1e-4	sll0669	GO:0005575	<1e-4	slr0765	GO:0016787	0.1007
slr0978	GO:0009263	<1e-4	sll0997	GO:0044255	<1e-4	slr2084	GO:0022803	<1e-4	sll0871	GO:0005575	<1e-4	ssr3154	GO:0016787	0.1007
ssl2717	GO:0080090	<1e-4	ssl3692	GO:0044282	<1e-4	slr8021	GO:0015077	<1e-4	slr6047	GO:0044444	<1e-4	sll0735	GO:0016787	0.1007
sll6054	GO:0046164	<1e-4	sll1528	GO:0006753	<1e-4	slr0404	GO:0043167	<1e-4	slr0553	GO:0005575	<1e-4	sll0584	GO:0016787	0.0973
ssl1552	GO:0072522	<1e-4	ssr6083	GO:0009260	<1e-4	ssr1552	GO:0016818	<1e-4	sll1388	GO:0044424	<1e-4	sll1293	GO:0016787	0.0973
slr1809	GO:0009987	<1e-4	slr0263	GO:0006753	<1e-4	sll0905	GO:0015077	<1e-4	ssl0750	GO:0044464	<1e-4	ssl0294	GO:0016787	0.0973
sll0498	GO:0006066	<1e-4	sll0913	GO:0006766	<1e-4	sll0007	GO:0015075	<1e-4	sll0400	GO:0043226	<1e-4	ssl0750	GO:0016787	0.0973
sll1512	GO:0072521	<1e-4	sll1965	GO:0009059	<1e-4	slr5119	GO:0022832	<1e-4	slr2144	GO:0044425	<1e-4	sll1763	GO:0016787	0.0973
slr2110	GO:0006220	<1e-4	slr6045	GO:0009123	<1e-4	ssr2554	GO:0022832	<1e-4	slr0637	GO:0044424	<1e-4	sll1527	GO:0016787	0.0973
slr0109	GO:0046164	<1e-4	slr1624	GO:0060255	<1e-4	slr6009	GO:0016817	<1e-4	ssr6085	GO:0044424	<1e-4	sll0350	GO:0016787	0.0973
slr1847	GO:0019752	<1e-4	ssl1552	GO:0019362	<1e-4	ssl2384	GO:0022832	<1e-4	slr2110	GO:0044464	<1e-4	ssl0787	GO:0003676	0.0971
ssl8039	GO:0009987	<1e-4	slr0238	GO:0016054	<1e-4	ssl5007	GO:0016462	<1e-4	slr0613	GO:0044444	<1e-4	sll0096	GO:0003676	0.0971
ssl6061	GO:0031323	<1e-4	slr0960	GO:0050794	<1e-4	ssr6048	GO:0043492	<1e-4	sll0811	GO:0044425	<1e-4	sll2006	GO:0003676	0.0971
ssl3291	GO:0071496	<1e-4	sll1252	GO:0072521	<1e-4	sll0436	GO:0016818	<1e-4	sll0095	GO:0044422	<1e-4	sll0854	GO:0003676	0.0971
sll0762	GO:0034641	<1e-4	slr0442	GO:0080090	<1e-4	sll0525	GO:0015291	<1e-4	sll0930	GO:0044425	<1e-4	slr1866	GO:0003676	0.0971
sll0981	GO:0052111	<1e-4	slr1544	GO:0019751	<1e-4	ssl1520	GO:0016741	<1e-4	slr0483	GO:0044425	<1e-4	ssr3532	GO:0003676	0.0971
sll0615	GO:0044003	<1e-4	slr0111	GO:0046907	<1e-4	ssl3451	GO:0015291	<1e-4	ssr1768	GO:0043226	<1e-4	ssr0536	GO:0003676	0.0971
ssl7007	GO:0009144	<1e-4	ssr2802	GO:0008150	<1e-4	slr1951	GO:0022857	<1e-4	ssl5003	GO:0044446	<1e-4	slr0937	GO:0003676	0.0971
sll1193	GO:0016051	<1e-4	slr1847	GO:0009123	<1e-4	slr1513	GO:0015291	<1e-4	slr0935	GO:0044424	<1e-4	slr1127	GO:0003676	0.0971
sll0984	GO:0052111	<1e-4	sll0069	GO:0051716	<1e-4	ssr3129	GO:0043492	<1e-4	sll1965	GO:0044464	<1e-4	slr1896	GO:0003676	0.0971
slr2121	GO:0044106	<1e-4	sll0007	GO:0006091	<1e-4	sll0787	GO:0022857	<1e-4	slr0863	GO:0043226	<1e-4	sll0382	GO:0003676	0.0971

slr0058	GO:0009057	<1e-4	slr1534	GO:0019752	<1e-4	slr1721	GO:0015399	<1e-4	slr0400	GO:0044446	<1e-4	sll0263	GO:0003676	0.0971
sll7028	GO:0051716	<1e-4	slr5102	GO:0071496	<1e-4	sll1285	GO:0016741	<1e-4	sll0558	GO:0044425	<1e-4	sll0447	GO:0003676	0.0971
slr5024	GO:0010556	<1e-4	sll0658	GO:0044281	<1e-4	ssl0787	GO:0043492	<1e-4	sll0996	GO:0044446	<1e-4	sll0743	GO:0003676	0.0971
sll1372	GO:0008610	<1e-4	slr7016	GO:0051246	<1e-4	ssl3382	GO:0022891	<1e-4	sll0685	GO:0044444	<1e-4	slr0476	GO:0003676	0.0971
sll1960	GO:0009123	<1e-4	sll0149	GO:0072527	<1e-4	sll1735	GO:0015399	<1e-4	sll0252	GO:0044446	<1e-4	slr0076	GO:0003676	0.0971
sll7062	GO:0006811	<1e-4	slr1677	GO:0009057	<1e-4	slr1397	GO:0008171	<1e-4	sll0268	GO:0044464	<1e-4	slr0852	GO:0003676	0.0971
slr1513	GO:0009987	<1e-4	slr0303	GO:0090304	<1e-4	sll0237	GO:0016746	<1e-4	slr1079	GO:0044425	<1e-4	slr1674	GO:0003676	0.0971
sll0218	GO:0006066	<1e-4	sll1942	GO:0044260	<1e-4	sll0854	GO:0003674	<1e-4	sll0208	GO:0044464	<1e-4	slr1557	GO:0003676	0.0971
slr6065	GO:0046164	<1e-4	slr1847	GO:0051188	<1e-4	ssl1918	GO:0022890	<1e-4	sll0775	GO:0005575	<1e-4	slr1196	GO:0003676	0.0971
sll0761	GO:0042180	<1e-4	ssr7093	GO:0043933	<1e-4	slr1659	GO:0022890	<1e-4	slr2071	GO:0043226	<1e-4	sll0274	GO:0003676	0.0971
ssr6048	GO:0009987	<1e-4	slr1927	GO:0044106	<1e-4	slr0181	GO:0015405	<1e-4	ssl1263	GO:0043226	<1e-4	sll1934	GO:0003676	0.0971
slr6106	GO:0044283	<1e-4	sll1925	GO:0044003	<1e-4	slr1623	GO:0022891	<1e-4	sll0162	GO:0005575	<1e-4	slr0287	GO:0003676	0.0971
sll0444	GO:0043436	<1e-4	sll0871	GO:0044106	<1e-4	sll0280	GO:0015399	<1e-4	sll7030	GO:0043226	<1e-4	sll0933	GO:0005488	0.0964
ssr6086	GO:0044275	<1e-4	sll0702	GO:0046164	<1e-4	ssr2067	GO:0060089	<1e-4	sll1832	GO:0044424	<1e-4	ssr2781	GO:0017111	0.0955
slr7016	GO:0009259	<1e-4	sll1528	GO:0045184	<1e-4	slr1087	GO:0043167	<1e-4	sll7006	GO:0044422	<1e-4	slr0848	GO:0017111	0.0955
ssr0109	GO:0044275	<1e-4	slr0458	GO:0009144	<1e-4	slr1438	GO:0022803	<1e-4	slr0400	GO:0043226	<1e-4	slr0980	GO:0017111	0.0955
ssl8028	GO:0044260	<1e-4	sll1040	GO:0044003	<1e-4	sll0871	GO:0042623	<1e-4	sll0424	GO:0044425	<1e-4	slr2018	GO:0017111	0.0955
slr1450	GO:0090304	<1e-4	slr6091	GO:0009123	<1e-4	sll1472	GO:0016746	<1e-4	sll0518	GO:0044464	<1e-4	sll0269	GO:0017111	0.0955
sll0609	GO:0009892	<1e-4	sll1201	GO:0019222	<1e-4	slr0208	GO:0022891	<1e-4	ssl0350	GO:0044444	<1e-4	sll0071	GO:0017111	0.0955
slr1657	GO:0046164	<1e-4	slr1468	GO:0071842	<1e-4	slr0271	GO:0015267	<1e-4	sll1166	GO:0044464	<1e-4	slr0317	GO:0017111	0.0955
sll8019	GO:0051716	<1e-4	sll0702	GO:0055086	<1e-4	slr0921	GO:0022838	<1e-4	ssr5019	GO:0044424	<1e-4	ssl0832	GO:0017111	0.0955
sll1949	GO:0046128	<1e-4	sll0241	GO:0006721	<1e-4	sll6055	GO:0015399	<1e-4	sll0749	GO:0005575	<1e-4	slr0442	GO:0017111	0.0955
sll0595	GO:0044271	<1e-4	sll0498	GO:0060255	<1e-4	sll1834	GO:0022891	<1e-4	sll7075	GO:0005575	<1e-4	sll0702	GO:0017111	0.0955
sll0381	GO:0080090	<1e-4	slr0948	GO:0018130	<1e-4	slr0870	GO:0016741	<1e-4	ssr2049	GO:0044464	<1e-4	slr1032	GO:0017111	0.0955
slr0104	GO:0051179	<1e-4	slr1464	GO:0008610	<1e-4	ssl7045	GO:0016818	<1e-4	sll0802	GO:0044444	<1e-4	sll0647	GO:0017111	0.0955
slr1907	GO:0009117	<1e-4	slr1927	GO:0019752	<1e-4	sll1344	GO:0015405	<1e-4	slr1964	GO:0044444	<1e-4	slr2084	GO:0017111	0.0955
ssl1255	GO:0016052	<1e-4	slr1114	GO:0006066	<1e-4	ssl1263	GO:0060089	<1e-4	sll1049	GO:0044424	<1e-4	ssl3291	GO:0017111	0.0955
sll0661	GO:0044283	<1e-4	sll1390	GO:0051171	<1e-4	slr5118	GO:0022803	<1e-4	slr5018	GO:0044444	<1e-4	sll1424	GO:0017111	0.0955
sll1832	GO:0001932	<1e-4	ssr2975	GO:0044106	<1e-4	sll0623	GO:0003674	<1e-4	slr1082	GO:0005575	<1e-4	ssr1256	GO:0017111	0.0955
slr1081	GO:0055114	<1e-4	slr0654	GO:0045184	<1e-4	sll0167	GO:0015291	<1e-4	slr8014	GO:0044425	<1e-4	sll1775	GO:0017111	0.0955
ssl5098	GO:0022607	<1e-4	ssl7042	GO:0022411	<1e-4	slr2117	GO:0022836	<1e-4	sll0168	GO:0005575	<1e-4	slr0919	GO:0017111	0.0955
slr1544	GO:0051234	<1e-4	ssl3291	GO:0019637	<1e-4	slr0238	GO:0016741	<1e-4	slr1618	GO:0044464	<1e-4	sll1749	GO:0017111	0.0955
ssr3572	GO:0034654	<1e-4	sll1131	GO:0072528	<1e-4	slr0208	GO:0015399	<1e-4	sll5130	GO:0005575	<1e-4	sll0103	GO:0017111	0.0955
slr0345	GO:0043436	<1e-4	sll1163	GO:0009123	<1e-4	slr0362	GO:0015405	<1e-4	slr2125	GO:0005575	<1e-4	sll0932	GO:0017111	0.0955
ssl7038	GO:0051818	<1e-4	slr0241	GO:0065007	<1e-4	sll0168	GO:0022892	<1e-4	slr0989	GO:0005575	<1e-4	ssr2803	GO:0017111	0.0955
slr1799	GO:0046907	<1e-4	slr0765	GO:0006220	<1e-4	slr0496	GO:0015291	<1e-4	sll1119	GO:0043226	<1e-4	slr0579	GO:0017111	0.0955
slr0960	GO:0043170	<1e-4	sll0284	GO:0046128	<1e-4	sll1542	GO:0005342	<1e-4	slr0065	GO:0043226	<1e-4	slr0408	GO:0017111	0.0955
sll0586	GO:0034641	<1e-4	sll7050	GO:0044248	<1e-4	ssr2553	GO:0015399	<1e-4	slr1441	GO:0044422	<1e-4	slr1636	GO:0016787	0.0952
sll1378	GO:0065007	<1e-4	slr0602	GO:0006082	<1e-4	slr0468	GO:0015077	<1e-4	sll1511	GO:0044464	<1e-4	sll0931	GO:0016787	0.0952
slr1571	GO:0050789	<1e-4	slr7024	GO:0019222	<1e-4	slr0602	GO:0022832	<1e-4	slr1895	GO:0005575	<1e-4	sll0413	GO:0016787	0.0952
sll1455	GO:0009150	<1e-4	sll0443	GO:0018130	<1e-4	sll1250	GO:0016817	<1e-4	ssl2920	GO:0005575	<1e-4	slr1052	GO:0016787	0.0952
slr1397	GO:0009259	<1e-4	slr6028	GO:0042180	<1e-4	slr0975	GO:0043167	<1e-4	slr0863	GO:0044444	<1e-4	slr1117	GO:0009058	0.0945
slr0108	GO:0006220	<1e-4	slr1851	GO:0009141	<1e-4	sll0301	GO:0042623	<1e-4	sll0985	GO:0005575	<1e-4	slr1932	GO:0009058	0.0945
slr1127	GO:0031326	<1e-4	slr0294	GO:0051171	<1e-4	sll1702	GO:0043492	<1e-4	slr0655	GO:0005575	<1e-4	slr0482	GO:0009058	0.0945
slr1676	GO:0043648	<1e-4	ssl1004	GO:0008150	<1e-4	sll1162	GO:0022838	<1e-4	sll1541	GO:0044424	<1e-4	sll0785	GO:0009058	0.0945
slr6039	GO:0050801	<1e-4	ssr3341	GO:0006091	<1e-4	slr0606	GO:0022890	<1e-4	slr1103	GO:0005575	<1e-4	slr0431	GO:0009058	0.0945
slr1815	GO:0006732	<1e-4	slr0013	GO:0044275	<1e-4	sll0185	GO:0016817	<1e-4	slr1799	GO:0044422	<1e-4	ssl2065	GO:0009058	0.0945
slr1809	GO:0034660	<1e-4	sll0294	GO:0072521	<1e-4	sll0456	GO:0042623	<1e-4	sll0638	GO:0044425	<1e-4	sll0780	GO:0009058	0.0945
slr1431	GO:0009056	<1e-4	slr8021	GO:0009126	<1e-4	ssr6002	GO:0016817	<1e-4	slr6057	GO:0044424	<1e-4	slr1571	GO:0009058	0.0945
slr0013	GO:0050789	<1e-4	slr6045	GO:0019751	<1e-4	sll1527	GO:0003674	<1e-4	ssl0109	GO:0044446	<1e-4	sll0645	GO:0009058	0.0945
ssr5106	GO:0043549	<1e-4	sll1095	GO:0071841	<1e-4	slr8014	GO:0022891	<1e-4	slr0545	GO:0005575	<1e-4	slr1437	GO:0009058	0.0945
sll1071	GO:0010468	<1e-4	sll1348	GO:0044003	<1e-4	slr1196	GO:0005342	<1e-4	slr0579	GO:0044444	<1e-4	sll0237	GO:0009058	0.0945
sll7047	GO:0009150	<1e-4	sll1504	GO:0019752	<1e-4	ssr6026	GO:0022803	<1e-4	slr1215	GO:0043234	<1e-4	slr0294	GO:0009058	0.0945
slr0582	GO:0006091	<1e-4	slr0364	GO:0001932	<1e-4	sll1563	GO:0022857	<1e-4	sll0702	GO:0044446	<1e-4	slr1436	GO:0009058	0.0945

slr1098	GO:0046483	<1e-4	slr0971	GO:0044003	<1e-4	slr0106	GO:0022891	<1e-4	slr1290	GO:0044444	<1e-4	slr1573	GO:0009058	0.0945
ssl1923	GO:0052188	<1e-4	slr0238	GO:0090304	<1e-4	ssl3291	GO:0015077	<1e-4	slr0273	GO:0044444	<1e-4	sll0243	GO:0009058	0.0945
slr0142	GO:0001932	<1e-4	slr0082	GO:0055114	<1e-4	ssr6003	GO:0016462	<1e-4	slr2011	GO:0044422	<1e-4	slr0913	GO:0009058	0.0945
ssl2420	GO:0006220	<1e-4	sll0625	GO:0009394	<1e-4	sll1702	GO:0022803	<1e-4	ssr2553	GO:0044444	<1e-4	sll0103	GO:0003676	0.094
sll0310	GO:0090304	<1e-4	slr1378	GO:0031323	<1e-4	ssl0350	GO:0016818	<1e-4	sll1940	GO:0044446	<1e-4	sll0238	GO:0003676	0.094
slr2119	GO:0009117	<1e-4	sll1652	GO:0048519	<1e-4	sll0995	GO:0015293	<1e-4	slr0121	GO:0005575	<1e-4	ssr2201	GO:0003676	0.094
sll0909	GO:0006631	<1e-4	slr0607	GO:0019362	<1e-4	ssr3304	GO:0022890	<1e-4	slr1652	GO:0044446	<1e-4	slr1565	GO:0003676	0.094
sll1355	GO:0019751	<1e-4	sll1239	GO:0016053	<1e-4	slr1590	GO:0016741	<1e-4	sll1751	GO:0005575	<1e-4	sll0763	GO:0003676	0.094
sll1173	GO:0048523	<1e-4	slr1676	GO:0044260	<1e-4	sll1570	GO:0022857	<1e-4	ssr2551	GO:0044444	<1e-4	slr0579	GO:0003676	0.094
sll1785	GO:0051818	<1e-4	sll0847	GO:0043648	<1e-4	slr0581	GO:0008171	<1e-4	ssr1552	GO:0043226	<1e-4	sll1775	GO:0003676	0.094
sll0496	GO:0051234	<1e-4	sll1130	GO:0034654	<1e-4	ssl5095	GO:0015291	<1e-4	sll0872	GO:0044444	<1e-4	slr2038	GO:0003676	0.094
slr1081	GO:0034654	<1e-4	sll1773	GO:0044260	<1e-4	slr1365	GO:0016746	<1e-4	slr0619	GO:0044422	<1e-4	slr0980	GO:0003676	0.094
sll2006	GO:0019222	<1e-4	sll1442	GO:0052188	<1e-4	slr0144	GO:0016462	<1e-4	sll0614	GO:0005575	<1e-4	sll0558	GO:0003676	0.0934
ssr6085	GO:0006066	<1e-4	slr1959	GO:0031640	<1e-4	slr1676	GO:0016746	<1e-4	ssr1425	GO:0044424	<1e-4	slr1800	GO:0003676	0.0934
slr1599	GO:0051188	<1e-4	slr1571	GO:0072522	<1e-4	slr0596	GO:0022803	<1e-4	sll1834	GO:0044422	<1e-4	ssl2996	GO:0003676	0.0934
slr0408	GO:0046483	<1e-4	slr0273	GO:0060255	<1e-4	slr0610	GO:0043167	<1e-4	slr0209	GO:0044464	<1e-4	slr1143	GO:0003676	0.0934
sll0803	GO:0009262	<1e-4	ssl2595	GO:0006163	<1e-4	ssr2551	GO:0008171	<1e-4	sll0410	GO:0005575	<1e-4	slr0589	GO:0003676	0.0934
slr1429	GO:0050794	<1e-4	slr1365	GO:0019752	<1e-4	ssr2787	GO:0022890	<1e-4	sll5044	GO:0044444	<1e-4	sll0732	GO:0003676	0.0934
ssr2439	GO:0065007	<1e-4	sll1307	GO:0009141	<1e-4	sll1751	GO:0015267	<1e-4	ssr3409	GO:0005575	<1e-4	sll0586	GO:0003676	0.0934
slr0813	GO:0009056	<1e-4	sll0545	GO:0072521	<1e-4	sll0412	GO:0042623	<1e-4	slr0060	GO:0005575	<1e-4	slr1847	GO:0003676	0.0934
sll1891	GO:0016054	<1e-4	slr7083	GO:0044281	<1e-4	sll1485	GO:0015077	<1e-4	sll1380	GO:0005575	<1e-4	slr0594	GO:0003676	0.0934
slr1660	GO:0031323	<1e-4	sll1132	GO:0009124	<1e-4	ssl1792	GO:0005342	<1e-4	sll0371	GO:0044422	<1e-4	sll0788	GO:0003676	0.0934
sll1573	GO:0006732	<1e-4	slr0551	GO:0050801	<1e-4	slr0888	GO:0043167	<1e-4	sll7086	GO:0005575	<1e-4	slr1865	GO:0003676	0.0934
ssr1256	GO:0042455	<1e-4	ssl1520	GO:0034641	<1e-4	sll1477	GO:0016741	<1e-4	slr6106	GO:0044444	<1e-4	slr1811	GO:0003676	0.0934
sll1751	GO:0006631	<1e-4	sll0293	GO:0006091	<1e-4	sll5030	GO:0015405	<1e-4	slr1613	GO:0044444	<1e-4	slr1385	GO:0003676	0.0925
sll0008	GO:0090304	<1e-4	sll0804	GO:0044260	<1e-4	slr0355	GO:0022838	<1e-4	sll0188	GO:0005575	<1e-4	slr2092	GO:0003676	0.0925
slr6072	GO:0009309	<1e-4	ssl0312	GO:0046483	<1e-4	slr6022	GO:0022803	<1e-4	slr6006	GO:0044444	<1e-4	slr0491	GO:0003676	0.0925
ssr2787	GO:0016052	<1e-4	sll1186	GO:0048878	<1e-4	sll0298	GO:0022891	<1e-4	sll1925	GO:0044422	<1e-4	slr1702	GO:0003676	0.0925
slr1959	GO:0051188	<1e-4	sll0269	GO:0051347	<1e-4	sll0478	GO:0016817	<1e-4	sll6052	GO:0044424	<1e-4	slr1215	GO:0016787	0.0911
sll7031	GO:0052111	<1e-4	ssl0738	GO:0048518	<1e-4	ssr6046	GO:0016462	<1e-4	slr0551	GO:0044446	<1e-4	sll0068	GO:0016787	0.0911
ssl3382	GO:0050801	<1e-4	ssr6099	GO:0051234	<1e-4	slr1753	GO:0016741	<1e-4	slr0144	GO:0044424	<1e-4	slr1603	GO:0017111	0.0908
sll0781	GO:0052188	<1e-4	slr1866	GO:0009259	<1e-4	ssr5117	GO:0016817	<1e-4	slr1419	GO:0044425	<1e-4	slr0810	GO:0017111	0.0908
slr1183	GO:0055114	<1e-4	sll0160	GO:0009150	<1e-4	slr1721	GO:0015291	<1e-4	sll7078	GO:0044446	<1e-4	sll0661	GO:0017111	0.0908
sll1203	GO:0009199	<1e-4	sll0189	GO:0009394	<1e-4	slr1474	GO:0015405	<1e-4	slr1363	GO:0044444	<1e-4	ssr2049	GO:0017111	0.0908
slr6080	GO:0034641	<1e-4	slr2052	GO:0006721	<1e-4	sll0513	GO:0015291	<1e-4	slr1287	GO:0005575	<1e-4	slr1702	GO:0017111	0.0908
ssr2422	GO:0044271	<1e-4	slr0592	GO:0019222	<1e-4	ssl5015	GO:0043492	<1e-4	sll5128	GO:0005575	<1e-4	ssr2843	GO:0017111	0.0908
ssr0761	GO:0065007	<1e-4	slr1753	GO:0019222	<1e-4	slr7073	GO:0016746	<1e-4	sll1131	GO:0044424	<1e-4	slr2092	GO:0017111	0.0908
sll0446	GO:0009889	<1e-4	slr6065	GO:0065007	<1e-4	sll1764	GO:0015291	<1e-4	slr0337	GO:0044446	<1e-4	slr0491	GO:0017111	0.0908
slr0907	GO:0048878	<1e-4	ssr0759	GO:0044271	<1e-4	sll1764	GO:0016746	<1e-4	slr6101	GO:0044464	<1e-4	ssr2142	GO:0005215	0.0897
slr0637	GO:0090304	<1e-4	sll1640	GO:0044106	<1e-4	sll1586	GO:0015293	<1e-4	sll1528	GO:0005575	<1e-4	slr1644	GO:0005215	0.0897
ssl1577	GO:0009165	<1e-4	ssl2069	GO:0008150	<1e-4	sll0944	GO:0043167	<1e-4	slr1600	GO:0044446	<1e-4	slr0380	GO:0009058	0.0895
sll0272	GO:0009165	<1e-4	slr0801	GO:0009260	<1e-4	sll1388	GO:0015399	<1e-4	ssr2554	GO:0043226	<1e-4	sll0406	GO:0009058	0.0895
ssr2998	GO:0016053	<1e-4	slr1863	GO:0071496	<1e-4	sll1040	GO:0008171	<1e-4	sll0804	GO:0044422	<1e-4	slr0262	GO:0009058	0.0895
sll1203	GO:0044260	<1e-4	slr1169	GO:0009987	<1e-4	slr0149	GO:0015075	<1e-4	slr1474	GO:0044424	<1e-4	slr0964	GO:0009058	0.0895
slr0770	GO:0019637	<1e-4	ssl2420	GO:0019219	<1e-4	slr1464	GO:0043167	<1e-4	slr5112	GO:0044425	<1e-4	ssl5095	GO:0009058	0.0895
slr0250	GO:0046164	<1e-4	sll1131	GO:0051818	<1e-4	slr0695	GO:0022890	<1e-4	slr0398	GO:0005575	<1e-4	slr1069	GO:0009058	0.0895
slr0039	GO:0009161	<1e-4	sll1830	GO:0008150	<1e-4	sll7043	GO:0016741	<1e-4	slr7059	GO:0044424	<1e-4	slr0407	GO:0009058	0.0895
slr1544	GO:0052188	<1e-4	ssr2142	GO:0016070	<1e-4	sll0837	GO:0043167	<1e-4	slr0722	GO:0044446	<1e-4	sll0886	GO:0009058	0.0895
sll0803	GO:0044281	<1e-4	sll0572	GO:0006163	<1e-4	slr5126	GO:0022857	<1e-4	sll0488	GO:0044446	<1e-4	slr1535	GO:0009058	0.0895
slr0423	GO:0071554	<1e-4	slr2070	GO:0009893	<1e-4	sgl0002	GO:0022838	<1e-4	sll0412	GO:0043226	<1e-4	sll0775	GO:0009058	0.0895
slr0810	GO:0009260	<1e-4	slr1114	GO:0019752	<1e-4	ssr1258	GO:0016817	<1e-4	slr1851	GO:0044444	<1e-4	sll1863	GO:0009058	0.0895
slr6045	GO:0009165	<1e-4	slr1778	GO:0048522	<1e-4	slr1023	GO:0016818	<1e-4	ssr0692	GO:0044422	<1e-4	ssl8003	GO:0009058	0.0895
ssr6099	GO:0018130	<1e-4	slr1104	GO:0050801	<1e-4	sll1142	GO:0016818	<1e-4	slr1266	GO:0043226	<1e-4	ssl2920	GO:0009058	0.0895
slr1365	GO:0006766	<1e-4	sll0272	GO:0051716	<1e-4	sll5032	GO:0022890	<1e-4	sll1086	GO:0044464	<1e-4	slr1095	GO:0009058	0.0895

ssr7017	GO:0034641	<1e-4	sll1714	GO:0008610	<1e-4	sll0008	GO:0022836	<1e-4	sll0242	GO:0044424	<1e-4	slr1162	GO:0009058	0.0895
sll1995	GO:0009123	<1e-4	slr0476	GO:0006793	<1e-4	slr1444	GO:0016741	<1e-4	slr1998	GO:0044444	<1e-4	sll0742	GO:0009058	0.0895
slr2004	GO:0044003	<1e-4	slr1790	GO:0008610	<1e-4	sll0098	GO:0016746	<1e-4	slr0667	GO:0044464	<1e-4	sll1921	GO:0009058	0.0895
slr7094	GO:0080090	<1e-4	sll0688	GO:0051171	<1e-4	slr6081	GO:0015267	<1e-4	sll0371	GO:0005575	<1e-4	ssl3383	GO:0009058	0.0895
slr0976	GO:0043436	<1e-4	sll0481	GO:0052188	<1e-4	slr0192	GO:0015267	<1e-4	slr0813	GO:0044425	<1e-4	slr1534	GO:0009058	0.0895
sll0854	GO:0009308	<1e-4	slr6064	GO:0009199	<1e-4	slr8044	GO:0042623	<1e-4	slr1275	GO:0044424	<1e-4	slr0151	GO:0009058	0.0895
sll0023	GO:0044106	<1e-4	slr0059	GO:0050794	<1e-4	slr7071	GO:0022832	<1e-4	slr0355	GO:0044446	<1e-4	sll0846	GO:0009058	0.0895
slr0784	GO:0019752	<1e-4	sll0361	GO:0051347	<1e-4	slr0482	GO:0015077	<1e-4	slr7101	GO:0005575	<1e-4	sll1062	GO:0009058	0.0895
slr1807	GO:0009199	<1e-4	ssr5011	GO:0008150	<1e-4	ssr1766	GO:0016817	<1e-4	sll7033	GO:0043226	<1e-4	sll0168	GO:0009058	0.0895
slr1681	GO:0019751	<1e-4	sll1344	GO:0046164	<1e-4	sll0756	GO:0015291	<1e-4	slr1774	GO:0005575	<1e-4	sll0266	GO:0009058	0.0895
sgl0002	GO:0006066	<1e-4	slr1977	GO:0043933	<1e-4	sll1251	GO:0003674	<1e-4	slr0889	GO:0043226	<1e-4	sll0658	GO:0009058	0.0895
sll1447	GO:0018130	<1e-4	sll1186	GO:0051171	<1e-4	ssl3829	GO:0043167	<1e-4	slr1276	GO:0044424	<1e-4	slr1768	GO:0009058	0.0895
sll0189	GO:0034660	<1e-4	sll5063	GO:0043549	<1e-4	slr1052	GO:0022803	<1e-4	slr1533	GO:0005575	<1e-4	ssr3410	GO:0009058	0.0895
ssl2384	GO:0009889	<1e-4	sll0031	GO:0051171	<1e-4	sll0996	GO:0015077	<1e-4	sll0710	GO:0044424	<1e-4	ssr2554	GO:0009058	0.0895
sll7034	GO:0051179	<1e-4	slr1571	GO:0048878	<1e-4	sll1285	GO:0016462	<1e-4	slr6100	GO:0043226	<1e-4	slr7058	GO:0009058	0.0895
slr0813	GO:0044003	<1e-4	slr1964	GO:0009123	<1e-4	slr2092	GO:0015075	<1e-4	slr1450	GO:0044424	<1e-4	slr1907	GO:0009058	0.0895
sll1938	GO:0034641	<1e-4	sll0688	GO:0033013	<1e-4	slr0654	GO:0005342	<1e-4	slr0784	GO:0044446	<1e-4	slr6015	GO:0009058	0.0895
sll1054	GO:0009056	<1e-4	ssl5129	GO:0006082	<1e-4	ssr3341	GO:0015267	<1e-4	sll1693	GO:0044464	<1e-4	ssl3451	GO:0009058	0.0895
slr1187	GO:0044281	<1e-4	slr0318	GO:0051186	<1e-4	ssl1923	GO:0022832	<1e-4	slr0376	GO:0044425	<1e-4	ssl5129	GO:0009058	0.0895
ssr5120	GO:0034641	<1e-4	slr0655	GO:0052111	<1e-4	sll0397	GO:0005342	<1e-4	slr1959	GO:0044464	<1e-4	sll1906	GO:0009058	0.0895
sll1464	GO:0019752	<1e-4	sll1510	GO:0043170	<1e-4	sll1222	GO:0016817	<1e-4	slr1726	GO:0043226	<1e-4	slr0333	GO:0009058	0.0895
sll0085	GO:0009199	<1e-4	slr0398	GO:0046907	<1e-4	ssr5074	GO:0015291	<1e-4	slr0586	GO:0005575	<1e-4	ssr0102	GO:0009058	0.0895
slr0780	GO:0048519	<1e-4	sll0710	GO:0071496	<1e-4	sll0586	GO:0043492	<1e-4	sll0095	GO:0044446	<1e-4	slr0552	GO:0009058	0.0895
slr0765	GO:0045184	<1e-4	sll0242	GO:0009260	<1e-4	ssr6083	GO:0016462	<1e-4	sll1680	GO:0005575	<1e-4	sll0284	GO:0009058	0.0895
slr0654	GO:0051234	<1e-4	slr1087	GO:0009124	<1e-4	slr6015	GO:0042623	<1e-4	ssl1046	GO:0044425	<1e-4	slr0590	GO:0005215	0.0846
ssl0483	GO:0006811	<1e-4	sll1601	GO:0048878	<1e-4	sll1447	GO:0060089	<1e-4	sll1832	GO:0044444	<1e-4	sll0160	GO:0005215	0.0846
slr0588	GO:0044106	<1e-4	slr6091	GO:0034654	<1e-4	slr2027	GO:0015267	<1e-4	ssl0352	GO:0044444	<1e-4	slr0813	GO:0005215	0.0846
slr0407	GO:0043549	<1e-4	slr0514	GO:0051171	<1e-4	sll0888	GO:0016741	<1e-4	slr7081	GO:0044464	<1e-4	slr7071	GO:0005215	0.0846
sll0735	GO:0043549	<1e-4	sll1528	GO:0051246	<1e-4	ssr6085	GO:0003674	<1e-4	sll0898	GO:0044422	<1e-4	sll7090	GO:0005215	0.0846
sll8004	GO:0042455	<1e-4	ssr0102	GO:0009056	<1e-4	slr6029	GO:0022857	<1e-4	ssl5100	GO:0044444	<1e-4	slr2071	GO:0005215	0.0846
sll0508	GO:0071841	<1e-4	slr0948	GO:0006733	<1e-4	slr1103	GO:0015267	<1e-4	slr1104	GO:0044425	<1e-4	slr1507	GO:0005215	0.0846
slr1342	GO:0009262	<1e-4	slr0695	GO:0051716	<1e-4	slr0049	GO:0022892	<1e-4	ssr3154	GO:0043226	<1e-4	ssl5068	GO:0005215	0.0846
sll0853	GO:0043648	<1e-4	slr1619	GO:0044106	<1e-4	slr0821	GO:0003674	<1e-4	sll8032	GO:0044422	<1e-4	sll5067	GO:0005215	0.0846
sll0858	GO:0009394	<1e-4	sll6054	GO:0048878	<1e-4	sll1940	GO:0015405	<1e-4	sll1773	GO:0005575	<1e-4	sll0765	GO:0005215	0.0846
ssl0832	GO:0006721	<1e-4	ssr2755	GO:0071842	<1e-4	slr1864	GO:0015267	<1e-4	slr1025	GO:0005575	<1e-4	slr1628	GO:0005215	0.0846
slr1927	GO:0009117	<1e-4	ssl0787	GO:0051246	<1e-4	sll0861	GO:0015077	<1e-4	slr0238	GO:0044464	<1e-4	slr6087	GO:0005215	0.0846
sll0147	GO:0044248	<1e-4	slr7012	GO:0005996	<1e-4	sll1131	GO:0015399	<1e-4	slr1209	GO:0005575	<1e-4	ssl0467	GO:0005215	0.0846
slr0957	GO:0050789	<1e-4	sll7047	GO:0090304	<1e-4	sll0479	GO:0016818	<1e-4	sll1109	GO:0044464	<1e-4	sll1784	GO:0005215	0.0846
slr1450	GO:0043436	<1e-4	slr0013	GO:0009199	<1e-4	slr0356	GO:0015075	<1e-4	slr6101	GO:0044422	<1e-4	ssr7084	GO:0005215	0.0846
slr1342	GO:0009126	<1e-4	sll1956	GO:0016054	<1e-4	sll1262	GO:0015293	<1e-4	ssl0353	GO:0005575	<1e-4	sll7028	GO:0005215	0.0846
ssr5074	GO:0044260	<1e-4	slr6006	GO:0051818	<1e-4	ssr6019	GO:0022838	<1e-4	slr1186	GO:0043226	<1e-4	sll1340	GO:0005215	0.0846
slr7099	GO:0031640	<1e-4	sll1151	GO:0046483	<1e-4	sll0980	GO:0022832	<1e-4	slr0168	GO:0044446	<1e-4	slr6033	GO:0005215	0.0846
ssl5025	GO:0072524	<1e-4	ssr6083	GO:0071496	<1e-4	slr0404	GO:0043492	<1e-4	slr1977	GO:0005575	<1e-4	slr5053	GO:0005215	0.0846
slr1394	GO:0065007	<1e-4	sll0376	GO:0031640	<1e-4	sll0925	GO:0015293	<1e-4	ssr1041	GO:0005575	<1e-4	slr0241	GO:0005215	0.0846
slr1816	GO:0009126	<1e-4	slr2084	GO:0006766	<1e-4	slr2119	GO:0005342	<1e-4	slr0569	GO:0044424	<1e-4	sll0496	GO:0005215	0.0846
sll0761	GO:0046394	<1e-4	slr1287	GO:0080090	<1e-4	sll0888	GO:0022838	<1e-4	ssl2384	GO:0044446	<1e-4	ssl2384	GO:0005215	0.0846
slr1419	GO:0006721	<1e-4	slr0602	GO:0071554	<1e-4	slr1544	GO:0015399	<1e-4	sll1142	GO:0044422	<1e-4	ssr2553	GO:0005215	0.0846
sll0230	GO:0009124	<1e-4	slr1699	GO:0080090	<1e-4	slr0587	GO:0042623	<1e-4	slr1576	GO:0005575	<1e-4	slr1394	GO:0005215	0.0846
sll8004	GO:0072527	<1e-4	sll1024	GO:0031640	<1e-4	sll1571	GO:0015267	<1e-4	slr0755	GO:0043226	<1e-4	sll0376	GO:0005215	0.0846
slr1413	GO:0031326	<1e-4	ssr0657	GO:0052188	<1e-4	sll7050	GO:0015291	<1e-4	slr0670	GO:0044425	<1e-4	ssl8028	GO:0005215	0.0846
slr1152	GO:0048523	<1e-4	sll1586	GO:0043933	<1e-4	sll1350	GO:0022890	<1e-4	slr1222	GO:0005575	<1e-4	sll8035	GO:0005215	0.0846
slr1107	GO:0043549	<1e-4	slr0345	GO:0051188	<1e-4	slr0092	GO:0015293	<1e-4	sll1527	GO:0044446	<1e-4	slr0273	GO:0005215	0.0846
ssl1520	GO:0019362	<1e-4	slr1127	GO:0048522	<1e-4	slr1721	GO:0022892	<1e-4	sll0044	GO:0043226	<1e-4	ssl5113	GO:0005215	0.0846
slr2049	GO:0009126	<1e-4	sll1509	GO:0051188	<1e-4	ssl3692	GO:0043492	<1e-4	sll1736	GO:0005575	<1e-4	slr0728	GO:0005215	0.0846

slr7100	GO:0009259	<1e-4	slr1676	GO:0044275	<1e-4	sll0266	GO:0015399	<1e-4	slr2121	GO:0005575	<1e-4	slr2004	GO:0005215	0.0846
slr6022	GO:0033013	<1e-4	slr1690	GO:0048523	<1e-4	slr1207	GO:0015291	<1e-4	slr0455	GO:0044464	<1e-4	ssl7038	GO:0005215	0.0846
slr0337	GO:0006220	<1e-4	sll1021	GO:0016053	<1e-4	ssr2843	GO:0060089	<1e-4	sll0007	GO:0044425	<1e-4	sll0044	GO:0005215	0.0846
sll7090	GO:0072522	<1e-4	slr0981	GO:0044106	<1e-4	slr0144	GO:0022838	<1e-4	slr7101	GO:0044422	<1e-4	sll7033	GO:0005215	0.0846
slr1223	GO:0042455	<1e-4	sll1062	GO:0072524	<1e-4	ssr6020	GO:0016818	<1e-4	slr6022	GO:0044446	<1e-4	ssr1425	GO:0005215	0.0846
slr1376	GO:0043412	<1e-4	slr0404	GO:0006082	<1e-4	sll0595	GO:0015399	<1e-4	slr7080	GO:0044422	<1e-4	slr5102	GO:0005215	0.0846
sll1265	GO:0009309	<1e-4	sll1062	GO:0016053	<1e-4	ssr5092	GO:0022891	<1e-4	slr7012	GO:0044422	<1e-4	sll0162	GO:0005215	0.0846
slr0360	GO:0072521	<1e-4	slr1935	GO:0010556	<1e-4	ssr5117	GO:0022857	<1e-4	sll0785	GO:0044464	<1e-4	slr1450	GO:0005215	0.0846
sll0846	GO:0006631	<1e-4	sll0931	GO:0034660	<1e-4	sll1307	GO:0016817	<1e-4	ssr1552	GO:0044425	<1e-4	slr7099	GO:0005215	0.0846
slr1862	GO:0051347	<1e-4	slr0146	GO:0034654	<1e-4	sll0888	GO:0022857	<1e-4	ssr1473	GO:0043226	<1e-4	ssr6027	GO:0005215	0.0846
sll1262	GO:0034641	<1e-4	slr6067	GO:0009141	<1e-4	slr1053	GO:0043167	<1e-4	slr7059	GO:0005575	<1e-4	sll1068	GO:0005215	0.0846
slr0689	GO:0044282	<1e-4	sll1476	GO:0009889	<1e-4	slr0325	GO:0022857	<1e-4	slr1391	GO:0005575	<1e-4	slr0771	GO:0005215	0.0846
sll0096	GO:0016054	<1e-4	sll5090	GO:0055086	<1e-4	slr0496	GO:0015267	<1e-4	slr5087	GO:0043226	<1e-4	slr1206	GO:0005215	0.0846
sll0827	GO:0006091	<1e-4	slr1023	GO:0048878	<1e-4	slr1577	GO:0015077	<1e-4	slr7100	GO:0043226	<1e-4	slr2070	GO:0005215	0.0846
sll1769	GO:0006732	<1e-4	slr2049	GO:0009262	<1e-4	sll1400	GO:0022836	<1e-4	slr2011	GO:0044446	<1e-4	slr5112	GO:0005215	0.0846
sll0424	GO:0009889	<1e-4	slr2010	GO:0046907	<1e-4	slr1470	GO:0022890	<1e-4	slr1436	GO:0044446	<1e-4	sll0939	GO:0005215	0.0846
slr6005	GO:0090304	<1e-4	slr0699	GO:0071496	<1e-4	ssl0750	GO:0016746	<1e-4	sll1542	GO:0044464	<1e-4	slr1290	GO:0005215	0.0846
ssl3382	GO:0050794	<1e-4	sll1570	GO:0009309	<1e-4	slr1957	GO:0022803	<1e-4	ssl7022	GO:0044444	<1e-4	slr7100	GO:0005215	0.0846
sll1938	GO:0009126	<1e-4	sll1252	GO:0006082	<1e-4	slr0364	GO:0022890	<1e-4	slr0545	GO:0044424	<1e-4	slr1114	GO:0005215	0.0846
sll1942	GO:0051171	<1e-4	slr2105	GO:0051716	<1e-4	sll1542	GO:0015291	<1e-4	slr1773	GO:0005575	<1e-4	ssr6078	GO:0005215	0.0846
ssl5007	GO:0046164	<1e-4	sll1024	GO:0080090	<1e-4	slr0442	GO:0015293	<1e-4	slr1363	GO:0044464	<1e-4	sll1049	GO:0005215	0.0846
ssr2754	GO:0022607	<1e-4	slr0731	GO:0006066	<1e-4	sll0602	GO:0016462	<1e-4	sll1132	GO:0044425	<1e-4	ssr1041	GO:0005215	0.0846
slr5126	GO:0042455	<1e-4	ssr3189	GO:0046907	<1e-4	slr0729	GO:0003674	<1e-4	sll1911	GO:0005575	<1e-4	sll1640	GO:0005215	0.0846
slr5021	GO:0009123	<1e-4	sll0710	GO:0009987	<1e-4	slr0376	GO:0022832	<1e-4	slr0104	GO:0044446	<1e-4	sll7069	GO:0005215	0.0846
ssl5027	GO:0050896	<1e-4	slr0919	GO:0009263	<1e-4	ssl2148	GO:0015405	<1e-4	slr1676	GO:0005575	<1e-4	ssl1263	GO:0005215	0.0846
slr7059	GO:0034660	<1e-4	sll1086	GO:0072522	<1e-4	slr0575	GO:0015399	<1e-4	sll7034	GO:0044464	<1e-4	slr1627	GO:0005215	0.0846
sll0863	GO:0046164	<1e-4	slr5111	GO:0031323	<1e-4	slr0440	GO:0003674	<1e-4	sll0005	GO:0005575	<1e-4	slr2049	GO:0005215	0.0846
slr0810	GO:0044282	<1e-4	sll1541	GO:0031326	<1e-4	slr5101	GO:0015293	<1e-4	slr1535	GO:0044422	<1e-4	sll1164	GO:0005215	0.0846
slr6094	GO:0019219	<1e-4	sll8001	GO:0006066	<1e-4	sll1411	GO:0042623	<1e-4	slr1143	GO:0044464	<1e-4	slr1780	GO:0005215	0.0846
sll1060	GO:0006793	<1e-4	sll0274	GO:0050789	<1e-4	sll1222	GO:0022803	<1e-4	slr0199	GO:0005575	<1e-4	ssl0410	GO:0005215	0.0846
sll0191	GO:0034654	<1e-4	slr1391	GO:0042451	<1e-4	slr1464	GO:0015405	<1e-4	sll8040	GO:0044425	<1e-4	slr6081	GO:0005215	0.0846
sll1040	GO:0055082	<1e-4	slr7096	GO:0006082	<1e-4	slr1513	GO:0022857	<1e-4	sll1956	GO:0044446	<1e-4	sll0847	GO:0005215	0.0846
ssr2962	GO:0008610	<1e-4	slr0976	GO:0009260	<1e-4	sll7070	GO:0022838	<1e-4	sll7087	GO:0044425	<1e-4	slr0521	GO:0005215	0.0846
sll0230	GO:0072521	<1e-4	ssl2138	GO:0050801	<1e-4	sll8004	GO:0022890	<1e-4	sll0157	GO:0044425	<1e-4	slr0416	GO:0005215	0.0846
slr7058	GO:0071840	<1e-4	slr0299	GO:0010556	<1e-4	sll1884	GO:0005342	<1e-4	sll1485	GO:0043226	<1e-4	ssr0336	GO:0005215	0.0846
ssr3189	GO:0051188	<1e-4	slr1104	GO:0042451	<1e-4	ssr3129	GO:0015267	<1e-4	slr0765	GO:0005575	<1e-4	ssl3549	GO:0005215	0.0846
slr1563	GO:0009263	<1e-4	ssl1792	GO:0042451	<1e-4	slr1752	GO:0015291	<1e-4	slr0195	GO:0044424	<1e-4	sll5030	GO:0005215	0.0846
slr2052	GO:0009263	<1e-4	sll8007	GO:0044271	<1e-4	slr1721	GO:0022832	<1e-4	ssr3304	GO:0044424	<1e-4	slr1951	GO:0005215	0.0846
slr6080	GO:0050896	<1e-4	slr1266	GO:0045184	<1e-4	slr1557	GO:0016741	<1e-4	slr7102	GO:0005575	<1e-4	slr0709	GO:0005215	0.0846
sll0985	GO:0051716	<1e-4	slr0023	GO:0009987	<1e-4	ssl3291	GO:0060089	<1e-4	sll0167	GO:0005575	<1e-4	slr0730	GO:0005215	0.0846
sll0284	GO:0009117	<1e-4	slr0333	GO:0009309	<1e-4	slr0059	GO:0043167	<1e-4	sll0355	GO:0044422	<1e-4	slr0325	GO:0005215	0.0846
slr1127	GO:0051716	<1e-4	sll0678	GO:0009309	<1e-4	slr0919	GO:0022892	<1e-4	slr7100	GO:0044424	<1e-4	sll1834	GO:0005215	0.0846
slr0609	GO:0008150	<1e-4	slr1568	GO:0006766	<1e-4	slr1484	GO:0016462	<1e-4	slr0668	GO:0044422	<1e-4	ssl5103	GO:0005215	0.0846
ssl2717	GO:0051818	<1e-4	slr1917	GO:0031326	<1e-4	slr0702	GO:0015399	<1e-4	sll0752	GO:0005575	<1e-4	slr6103	GO:0005215	0.0846
ssl5096	GO:0043436	<1e-4	slr0907	GO:0031323	<1e-4	sll0854	GO:0022891	<1e-4	slr0064	GO:0044425	<1e-4	slr0634	GO:0005215	0.0846
slr0594	GO:0050801	<1e-4	sll1166	GO:0043170	<1e-4	slr1847	GO:0022832	<1e-4	slr1107	GO:0005575	<1e-4	sll8011	GO:0005215	0.0846
ssr2333	GO:0043933	<1e-4	slr5112	GO:0009117	<1e-4	sll1240	GO:0022836	<1e-4	sll0752	GO:0044424	<1e-4	sll0098	GO:0005215	0.0846
slr6071	GO:0009259	<1e-4	sll8004	GO:0009263	<1e-4	slr0249	GO:0016741	<1e-4	slr1068	GO:0044422	<1e-4	slr7094	GO:0005215	0.0846
slr1470	GO:0031323	<1e-4	slr0650	GO:0043412	<1e-4	slr0483	GO:0022838	<1e-4	sll0811	GO:0044444	<1e-4	ssl2069	GO:0005215	0.0846
slr1915	GO:0048523	<1e-4	slr0818	GO:0009987	<1e-4	slr0326	GO:0015291	<1e-4	sll0639	GO:0044425	<1e-4	ssl5108	GO:0005215	0.0846
sll5033	GO:0065007	<1e-4	sll7055	GO:0071554	<1e-4	ssl1377	GO:0016818	<1e-4	slr1865	GO:0044422	<1e-4	slr1624	GO:0005215	0.0846
slr7101	GO:0071840	<1e-4	sll0861	GO:0048518	<1e-4	sll0479	GO:0003674	<1e-4	sll1581	GO:0044446	<1e-4	slr1681	GO:0005215	0.0846
sll1738	GO:0072524	<1e-4	sll0980	GO:0009987	<1e-4	slr1911	GO:0015293	<1e-4	ssr5074	GO:0044446	<1e-4	slr1087	GO:0005215	0.0846
sll0787	GO:0009056	<1e-4	slr1862	GO:0031640	<1e-4	slr0109	GO:0022857	<1e-4	slr0729	GO:0043226	<1e-4	slr1816	GO:0005215	0.0846

slr1611	GO:0005996	<1e-4	slr0871	GO:0044282	<1e-4	sll1949	GO:0015291	<1e-4	sll0269	GO:0044425	<1e-4	sll1158	GO:0005215	0.0846
slr1177	GO:0006631	<1e-4	slr1900	GO:0044260	<1e-4	slr1362	GO:0015405	<1e-4	ssr0332	GO:0005575	<1e-4	slr1174	GO:0005215	0.0846
slr0483	GO:0033013	<1e-4	sll0609	GO:0009059	<1e-4	sll1021	GO:0042623	<1e-4	ssr3402	GO:0044424	<1e-4	slr0667	GO:0005215	0.0846
slr2073	GO:0044271	<1e-4	sll0167	GO:0009141	<1e-4	slr0725	GO:0022838	<1e-4	slr0552	GO:0044425	<1e-4	sll0031	GO:0005215	0.0846
sll1240	GO:0009142	<1e-4	sll1926	GO:0043933	<1e-4	slr1362	GO:0005342	<1e-4	sll8002	GO:0044446	<1e-4	sll1570	GO:0005215	0.0846
slr1128	GO:0034641	<1e-4	slr0731	GO:0006811	<1e-4	sll1902	GO:0005342	<1e-4	ssr0536	GO:0005575	<1e-4	sll7077	GO:0005215	0.0846
sll0641	GO:0009059	<1e-4	slr1513	GO:0010556	<1e-4	sll0148	GO:0022803	<1e-4	sll1632	GO:0005575	<1e-4	slr0039	GO:0005215	0.0846
slr0959	GO:0019222	<1e-4	sll0008	GO:0009309	<1e-4	slr0358	GO:0016817	<1e-4	slr1915	GO:0044446	<1e-4	ssl7074	GO:0005215	0.0846
slr0468	GO:0044271	<1e-4	ssl5103	GO:0043170	<1e-4	slr0313	GO:0015267	<1e-4	slr2052	GO:0044425	<1e-4	slr0699	GO:0005215	0.0846
sll1433	GO:0006732	<1e-4	slr5017	GO:0009124	<1e-4	sll5034	GO:0015293	<1e-4	slr0232	GO:0044425	<1e-4	slr5127	GO:0005215	0.0846
ssr0109	GO:0050794	<1e-4	sll1532	GO:0009124	<1e-4	sll0749	GO:0015075	<1e-4	ssr2975	GO:0044425	<1e-4	sll1464	GO:0005215	0.0846
sll0505	GO:0042180	<1e-4	sll0446	GO:0009260	<1e-4	sll0266	GO:0022857	<1e-4	slr1406	GO:0044444	<1e-4	ssl1918	GO:0005215	0.0846
sll1658	GO:0009260	<1e-4	sll0939	GO:0019222	<1e-4	slr0211	GO:0015293	<1e-4	slr1053	GO:0044424	<1e-4	ssr7093	GO:0005215	0.0846
sll5067	GO:0055086	<1e-4	sll0149	GO:0071842	<1e-4	slr6101	GO:0022838	<1e-4	slr0519	GO:0044424	<1e-4	ssr2972	GO:0005215	0.0846
slr1415	GO:0018130	<1e-4	sll0505	GO:0048522	<1e-4	sll1714	GO:0022890	<1e-4	sll0647	GO:0044446	<1e-4	ssr5011	GO:0005215	0.0846
slr1415	GO:0006576	<1e-4	sll1485	GO:0001932	<1e-4	sll0488	GO:0043492	<1e-4	sll1882	GO:0005575	<1e-4	ssl1552	GO:0005215	0.0846
slr6013	GO:0006066	<1e-4	slr1809	GO:0051188	<1e-4	sll0414	GO:0015077	<1e-4	slr1721	GO:0044424	<1e-4	sll1289	GO:0005215	0.0846
slr6074	GO:0010468	<1e-4	slr1383	GO:0019751	<1e-4	sll0996	GO:0022836	<1e-4	sll0354	GO:0044422	<1e-4	slr6068	GO:0005215	0.0846
ssr2439	GO:0009308	<1e-4	slr0325	GO:0034654	<1e-4	slr5077	GO:0022832	<1e-4	ssr5120	GO:0005575	<1e-4	sll0678	GO:0005215	0.0846
slr0789	GO:0071496	<1e-4	sll0539	GO:0006811	<1e-4	sll7087	GO:0022803	<1e-4	sll0508	GO:0044424	<1e-4	slr0181	GO:0005215	0.0846
slr2011	GO:0009123	<1e-4	ssl0294	GO:0044003	<1e-4	slr1186	GO:0043492	<1e-4	slr0865	GO:0043226	<1e-4	slr1944	GO:0005215	0.0846
sll1131	GO:0051188	<1e-4	sll0609	GO:0009142	<1e-4	slr0606	GO:0022857	<1e-4	slr1438	GO:0005575	<1e-4	sll8027	GO:0005215	0.0846
slr0634	GO:0006793	<1e-4	sll1921	GO:0065007	<1e-4	sll1344	GO:0042623	<1e-4	ssr2553	GO:0044425	<1e-4	ssl7022	GO:0005215	0.0846
slr1162	GO:0006220	<1e-4	slr0503	GO:0009132	<1e-4	ssl2996	GO:0016746	<1e-4	slr1667	GO:0044424	<1e-4	sll1373	GO:0005215	0.0846
ssr5121	GO:0016054	<1e-4	ssl7038	GO:0034641	<1e-4	slr1681	GO:0022891	<1e-4	slr0058	GO:0044424	<1e-4	slr1394	GO:0000166	0.0842
sll1071	GO:0019752	<1e-4	slr1263	GO:0071496	<1e-4	slr1809	GO:0022892	<1e-4	slr2032	GO:0044444	<1e-4	slr0725	GO:0000166	0.0842
sll7078	GO:0009150	<1e-4	sll1191	GO:0051179	<1e-4	slr6049	GO:0060089	<1e-4	sll0487	GO:0044425	<1e-4	slr0273	GO:0000166	0.0842
slr1660	GO:0009259	<1e-4	slr1875	GO:0006082	<1e-4	slr0592	GO:0016817	<1e-4	slr6038	GO:0044425	<1e-4	sll5030	GO:0000166	0.0842
slr6065	GO:0046483	<1e-4	sll0702	GO:0009150	<1e-4	slr0060	GO:0022836	<1e-4	slr1025	GO:0044425	<1e-4	sll0157	GO:0000166	0.0842
sll1680	GO:0044260	<1e-4	sll0513	GO:0006732	<1e-4	slr6004	GO:0015405	<1e-4	ssr0761	GO:0005575	<1e-4	ssl5103	GO:0000166	0.0842
slr0821	GO:0055082	<1e-4	ssr2047	GO:0009893	<1e-4	slr0964	GO:0022832	<1e-4	slr2084	GO:0043226	<1e-4	sll1714	GO:0000166	0.0842
slr1535	GO:0051818	<1e-4	slr0208	GO:0019362	<1e-4	slr1407	GO:0022832	<1e-4	sll1250	GO:0044446	<1e-4	sll1609	GO:0000166	0.0842
slr7015	GO:0009260	<1e-4	sll1095	GO:0006091	<1e-4	slr0818	GO:0043167	<1e-4	sll0898	GO:0043226	<1e-4	sll0372	GO:0000166	0.0842
sll1995	GO:0051171	<1e-4	sll1949	GO:0071841	<1e-4	slr1812	GO:0005342	<1e-4	ssl5091	GO:0044444	<1e-4	ssr5074	GO:0000166	0.0842
ssl3692	GO:0019222	<1e-4	sll1960	GO:0052111	<1e-4	slr5024	GO:0016818	<1e-4	slr6009	GO:0044422	<1e-4	sll5128	GO:0000166	0.0842
sll0098	GO:0010468	<1e-4	sll0864	GO:0044003	<1e-4	sll1775	GO:0022890	<1e-4	sll1289	GO:0005575	<1e-4	sll1717	GO:0000166	0.0842
sll0085	GO:0055086	<1e-4	slr7014	GO:0009259	<1e-4	slr1032	GO:0015267	<1e-4	slr1215	GO:0044464	<1e-4	sll5067	GO:0000166	0.0842
slr1215	GO:0071840	<1e-4	slr1083	GO:0009308	<1e-4	sll1072	GO:0015405	<1e-4	ssl5025	GO:0044425	<1e-4	slr7060	GO:0000166	0.0842
ssr3571	GO:0005996	<1e-4	sll0658	GO:0006793	<1e-4	sll7089	GO:0003674	<1e-4	sll1352	GO:0044464	<1e-4	sll1495	GO:0000166	0.0842
slr1956	GO:0052188	<1e-4	ssl1520	GO:0046164	<1e-4	sll1995	GO:0015077	<1e-4	sll1164	GO:0005575	<1e-4	sll0793	GO:0000166	0.0842
slr1670	GO:0006720	<1e-4	sll0096	GO:0009991	<1e-4	slr0596	GO:0016746	<1e-4	ssl0109	GO:0044422	<1e-4	sll8011	GO:0000166	0.0842
slr0168	GO:0009394	<1e-4	slr5017	GO:0043648	<1e-4	slr0241	GO:0042623	<1e-4	slr1415	GO:0005575	<1e-4	ssr3572	GO:0000166	0.0842
slr0272	GO:0090304	<1e-4	sll0932	GO:0009161	<1e-4	slr0670	GO:0015075	<1e-4	sll0216	GO:0005575	<1e-4	sll1698	GO:0000166	0.0842
slr1951	GO:0009144	<1e-4	sll5067	GO:0044281	<1e-4	slr0398	GO:0016462	<1e-4	slr6088	GO:0044425	<1e-4	ssr6019	GO:0000166	0.0842
sll1761	GO:0009057	<1e-4	slr1186	GO:0009141	<1e-4	ssr1552	GO:0005342	<1e-4	sll1658	GO:0044422	<1e-4	ssl7045	GO:0000166	0.0842
slr1170	GO:0009987	<1e-4	ssr6030	GO:0046907	<1e-4	sll0274	GO:0043492	<1e-4	slr1530	GO:0043226	<1e-4	ssl7048	GO:0000166	0.0842
slr0634	GO:0009309	<1e-4	slr6033	GO:0046164	<1e-4	slr0204	GO:0003674	<1e-4	sll0586	GO:0044424	<1e-4	slr1327	GO:0000166	0.0842
sll1542	GO:0046907	<1e-4	slr2071	GO:0019752	<1e-4	slr1789	GO:0022890	<1e-4	slr0262	GO:0043226	<1e-4	ssr6078	GO:0000166	0.0842
sll1315	GO:0071496	<1e-4	sll0361	GO:0006631	<1e-4	ssr0657	GO:0015075	<1e-4	ssr5120	GO:0044425	<1e-4	ssl1378	GO:0000166	0.0842
sll0444	GO:0034641	<1e-4	sll0595	GO:0006163	<1e-4	sll0436	GO:0008171	<1e-4	sll7062	GO:0044446	<1e-4	slr0271	GO:0000166	0.0842
ssr1425	GO:0051716	<1e-4	sll1232	GO:0018130	<1e-4	sll1424	GO:0015293	<1e-4	ssr6089	GO:0044464	<1e-4	sll1915	GO:0000166	0.0842
slr1033	GO:0009259	<1e-4	sll7028	GO:0072522	<1e-4	slr0262	GO:0042623	<1e-4	ssl0461	GO:0044425	<1e-4	ssr5092	GO:0000166	0.0842
sll0839	GO:0006631	<1e-4	slr1572	GO:0080090	<1e-4	slr6087	GO:0022803	<1e-4	slr0482	GO:0044444	<1e-4	sll5132	GO:0000166	0.0842
slr0483	GO:0034654	<1e-4	sll5130	GO:0051716	<1e-4	slr0145	GO:0016817	<1e-4	slr1657	GO:0005575	<1e-4	sll1532	GO:0000166	0.0842

ssr2802	GO:0043648	<1e-4	sll5132	GO:0051246	<1e-4	sll0872	GO:0015291	<1e-4	ssl5031	GO:0044444	<1e-4	sll0608	GO:0000166	0.0842
sll1321	GO:0044003	<1e-4	sll1979	GO:0046164	<1e-4	sll5067	GO:0015405	<1e-4	ssl7038	GO:0044446	<1e-4	sll1151	GO:0000166	0.0842
sll1722	GO:0009165	<1e-4	slr1530	GO:0046483	<1e-4	slr0398	GO:0015077	<1e-4	sll0857	GO:0044424	<1e-4	ssr7079	GO:0000166	0.0842
sll1304	GO:0006753	<1e-4	slr0869	GO:0044260	<1e-4	sll1022	GO:0060089	<1e-4	slr7060	GO:0044424	<1e-4	sll1142	GO:0000166	0.0842
sll0691	GO:0019222	<1e-4	sll0930	GO:0009117	<1e-4	ssr6019	GO:0022832	<1e-4	sll1061	GO:0043226	<1e-4	ssr2711	GO:0000166	0.0842
slr1173	GO:0055086	<1e-4	sll7030	GO:0044281	<1e-4	slr1667	GO:0015399	<1e-4	sll0325	GO:0043226	<1e-4	ssl2069	GO:0000166	0.0842
sll0071	GO:0009056	<1e-4	sll1201	GO:0010556	<1e-4	sll0867	GO:0016817	<1e-4	sll1761	GO:0005575	<1e-4	sll1570	GO:0000166	0.0842
ssr1499	GO:0009987	<1e-4	sll0905	GO:0009117	<1e-4	ssl2814	GO:0022836	<1e-4	sll0180	GO:0044422	<1e-4	ssr6030	GO:0000166	0.0842
slr2117	GO:0001932	<1e-4	sll0350	GO:0006066	<1e-4	slr1083	GO:0015291	<1e-4	slr1340	GO:0044464	<1e-4	sll8040	GO:0000166	0.0842
sll0335	GO:0031323	<1e-4	sll1061	GO:0080090	<1e-4	sll1321	GO:0005342	<1e-4	sll0436	GO:0043226	<1e-4	slr5101	GO:0000166	0.0842
sll1863	GO:0071841	<1e-4	ssl5113	GO:0006793	<1e-4	sml0011	GO:0022832	<1e-4	slr0145	GO:0043226	<1e-4	slr1450	GO:0000166	0.0842
ssl3142	GO:0060255	<1e-4	slr1563	GO:0051179	<1e-4	sll1380	GO:0016741	<1e-4	ssr8047	GO:0044444	<1e-4	slr7102	GO:0000166	0.0842
sll1906	GO:0019752	<1e-4	sll0269	GO:0048878	<1e-4	ssr2802	GO:0015077	<1e-4	slr1819	GO:0044464	<1e-4	sll0321	GO:0000166	0.0842
sll5090	GO:0009144	<1e-4	slr0821	GO:0042455	<1e-4	slr1659	GO:0043492	<1e-4	ssl0832	GO:0005575	<1e-4	slr0238	GO:0000166	0.0842
sll1797	GO:0043170	<1e-4	ssl7021	GO:0006163	<1e-4	sll0815	GO:0003674	<1e-4	slr1259	GO:0005575	<1e-4	sll0496	GO:0000166	0.0842
slr1814	GO:0016054	<1e-4	sll0413	GO:0060255	<1e-4	slr1647	GO:0015405	<1e-4	slr6100	GO:0005575	<1e-4	slr2012	GO:0000166	0.0842
slr1178	GO:0051234	<1e-4	sll1979	GO:0034641	<1e-4	sll1757	GO:0008171	<1e-4	slr1926	GO:0044446	<1e-4	slr1670	GO:0000166	0.0842
sll1388	GO:0005996	<1e-4	sll1832	GO:0051818	<1e-4	sll0268	GO:0016741	<1e-4	ssl1004	GO:0044464	<1e-4	sll8035	GO:0000166	0.0842
slr0476	GO:0019637	<1e-4	sll0925	GO:0046907	<1e-4	sll0497	GO:0022892	<1e-4	ssr3532	GO:0005575	<1e-4	slr0813	GO:0000166	0.0842
slr1101	GO:0009260	<1e-4	sll1155	GO:0052111	<1e-4	sll0572	GO:0060089	<1e-4	slr1023	GO:0005575	<1e-4	ssl2814	GO:0000166	0.0842
sll1092	GO:0006811	<1e-4	ssl0832	GO:0043436	<1e-4	slr0709	GO:0015405	<1e-4	slr1911	GO:0043226	<1e-4	sll0062	GO:0000166	0.0842
slr0006	GO:0043648	<1e-4	slr7096	GO:0042455	<1e-4	slr7071	GO:0043167	<1e-4	sll0793	GO:0044424	<1e-4	ssr0657	GO:0000166	0.0842
sll1380	GO:0019362	<1e-4	sll0361	GO:0009259	<1e-4	sll0925	GO:0016462	<1e-4	sll1049	GO:0044444	<1e-4	slr0109	GO:0000166	0.0842
ssl5015	GO:0034641	<1e-4	ssl2807	GO:0019751	<1e-4	sll0925	GO:0015267	<1e-4	slr0959	GO:0043226	<1e-4	ssr7084	GO:0000166	0.0842
slr0157	GO:0009394	<1e-4	sll0524	GO:0033013	<1e-4	ssl7039	GO:0022838	<1e-4	sll1563	GO:0044464	<1e-4	slr0588	GO:0000166	0.0842
slr1702	GO:0072528	<1e-4	slr5018	GO:0006793	<1e-4	ssr2615	GO:0060089	<1e-4	slr0863	GO:0044425	<1e-4	ssr2975	GO:0000166	0.0842
slr6074	GO:0009126	<1e-4	slr1927	GO:0046483	<1e-4	slr0168	GO:0022832	<1e-4	slr0179	GO:0044422	<1e-4	sll7078	GO:0000166	0.0842
sll0072	GO:0043412	<1e-4	slr1114	GO:0043648	<1e-4	slr1203	GO:0016741	<1e-4	sll0783	GO:0005575	<1e-4	slr1789	GO:0000166	0.0842
sll1736	GO:0009132	<1e-4	sll1359	GO:0009394	<1e-4	slr1600	GO:0043167	<1e-4	sll0505	GO:0044425	<1e-4	slr0888	GO:0000166	0.0842
slr1241	GO:0006066	<1e-4	slr1790	GO:0009260	<1e-4	sll0423	GO:0015293	<1e-4	slr1670	GO:0044464	<1e-4	slr1915	GO:0000166	0.0842
slr0941	GO:0055114	<1e-4	sll0024	GO:0071554	<1e-4	slr7094	GO:0022803	<1e-4	slr6066	GO:0044464	<1e-4	sll0253	GO:0000166	0.0842
slr0498	GO:0034660	<1e-4	ssr5020	GO:0019219	<1e-4	slr0960	GO:0016462	<1e-4	ssl7042	GO:0044422	<1e-4	slr6028	GO:0000166	0.0842
slr1790	GO:0044255	<1e-4	sll0645	GO:0009161	<1e-4	sll0488	GO:0015077	<1e-4	slr1790	GO:0044422	<1e-4	sll1940	GO:0000166	0.0842
sll0783	GO:0048522	<1e-4	slr2071	GO:0072521	<1e-4	sll1486	GO:0015267	<1e-4	sll0590	GO:0005575	<1e-4	slr6033	GO:0000166	0.0842
sll1022	GO:0033013	<1e-4	ssl1972	GO:0048523	<1e-4	slr0784	GO:0015077	<1e-4	sll1916	GO:0044424	<1e-4	slr2117	GO:0000166	0.0842
ssl6061	GO:0051188	<1e-4	ssl5031	GO:0055082	<1e-4	slr5119	GO:0022890	<1e-4	slr0702	GO:0043226	<1e-4	slr0948	GO:0000166	0.0842
slr1287	GO:0008610	<1e-4	slr5012	GO:0009126	<1e-4	slr7026	GO:0022836	<1e-4	ssl5103	GO:0044464	<1e-4	slr7094	GO:0000166	0.0842
slr0709	GO:0043436	<1e-4	slr0553	GO:0009889	<1e-4	sll1784	GO:0015291	<1e-4	ssr0332	GO:0044424	<1e-4	ssl3829	GO:0000166	0.0842
sll0382	GO:0009124	<1e-4	slr0179	GO:0043549	<1e-4	slr0770	GO:0015291	<1e-4	slr1142	GO:0005575	<1e-4	sll0925	GO:0000166	0.0842
ssr2554	GO:0006811	<1e-4	slr1704	GO:0031640	<1e-4	slr6045	GO:0015077	<1e-4	slr1590	GO:0005575	<1e-4	slr2060	GO:0000166	0.0842
sll0525	GO:0048523	<1e-4	slr2144	GO:0009263	<1e-4	sll0381	GO:0008171	<1e-4	slr0978	GO:0044464	<1e-4	slr5087	GO:0000166	0.0842
slr7094	GO:0006811	<1e-4	slr6008	GO:0055114	<1e-4	sll5130	GO:0022891	<1e-4	slr1288	GO:0005575	<1e-4	sll1131	GO:0000166	0.0842
ssl0483	GO:0046907	<1e-4	slr0271	GO:0006066	<1e-4	sll0867	GO:0060089	<1e-4	slr1462	GO:0005575	<1e-4	ssr1425	GO:0000166	0.0842
sll0335	GO:0006220	<1e-4	sll0359	GO:0051234	<1e-4	slr1168	GO:0005342	<1e-4	slr1385	GO:0044446	<1e-4	slr0241	GO:0000166	0.0842
slr1762	GO:0065007	<1e-4	sll0423	GO:0009126	<1e-4	slr0455	GO:0015399	<1e-4	slr0607	GO:0044422	<1e-4	ssl0467	GO:0000166	0.0842
slr0989	GO:0009308	<1e-4	sll0875	GO:0009059	<1e-4	sll0727	GO:0022838	<1e-4	sll0413	GO:0044464	<1e-4	slr0689	GO:0000166	0.0842
sll0280	GO:0048519	<1e-4	sll0479	GO:0009892	<1e-4	sll0096	GO:0060089	<1e-4	sll0022	GO:0005575	<1e-4	sll1089	GO:0000166	0.0842
slr1789	GO:0050801	<1e-4	slr0358	GO:0009893	<1e-4	ssl0788	GO:0015405	<1e-4	slr6008	GO:0044422	<1e-4	slr5024	GO:0000166	0.0842
slr0108	GO:0072521	<1e-4	ssr2009	GO:0046128	<1e-4	slr7059	GO:0005342	<1e-4	sll5030	GO:0044422	<1e-4	ssr1473	GO:0000166	0.0842
sll0301	GO:0048523	<1e-4	slr6104	GO:0019637	<1e-4	ssl0461	GO:0022838	<1e-4	ssr6099	GO:0044422	<1e-4	ssl1690	GO:0000166	0.0842
sll1002	GO:0016054	<1e-4	slr1047	GO:0009260	<1e-4	sgl0002	GO:0022803	<1e-4	ssl5065	GO:0044424	<1e-4	ssl8039	GO:0000166	0.0842
sll0623	GO:0006082	<1e-4	slr1398	GO:0009057	<1e-4	ssr0109	GO:0015267	<1e-4	slr2004	GO:0044422	<1e-4	ssr1768	GO:0000166	0.0842
slr2111	GO:0051186	<1e-4	ssl3549	GO:0022411	<1e-4	sll1135	GO:0022832	<1e-4	slr1977	GO:0044425	<1e-4	slr0313	GO:0000166	0.0842
sll0499	GO:0052188	<1e-4	sll1061	GO:0008150	<1e-4	ssr6062	GO:0015399	<1e-4	slr0184	GO:0044464	<1e-4	ssr2318	GO:0000166	0.0842

sll1634	GO:0072524	<1e-4	slr7057	GO:0009889	<1e-4	slr1951	GO:0022803	<1e-4	sll5003	GO:0005575	<1e-4	sll1188	GO:0000166	0.0842
slr1084	GO:0045184	<1e-4	ssr2060	GO:0052188	<1e-4	ssl7042	GO:0043492	<1e-4	sll1503	GO:0044422	<1e-4	ssl6092	GO:0000166	0.0842
sll0479	GO:0043933	<1e-4	ssr3129	GO:0042455	<1e-4	slr0702	GO:0015291	<1e-4	slr0635	GO:0044425	<1e-4	slr0642	GO:0017111	0.0833
slr1699	GO:0046394	<1e-4	ssr7079	GO:0043170	<1e-4	ssr5020	GO:0022803	<1e-4	slr1702	GO:0044424	<1e-4	ssr3154	GO:0017111	0.0833
ssr0336	GO:0046128	<1e-4	sll1252	GO:0051188	<1e-4	sll0944	GO:0003674	<1e-4	slr7080	GO:0043226	<1e-4	slr0147	GO:0017111	0.0833
slr0362	GO:0048523	<1e-4	sll1902	GO:0045184	<1e-4	slr0291	GO:0015293	<1e-4	sll0822	GO:0044464	<1e-4	sll1477	GO:0017111	0.0833
sll0752	GO:0016052	<1e-4	sll0172	GO:0031640	<1e-4	ssl2971	GO:0043167	<1e-4	sll1735	GO:0044464	<1e-4	slr0765	GO:0017111	0.0833
sll1532	GO:0044282	<1e-4	slr1188	GO:0046394	<1e-4	sll1040	GO:0003674	<1e-4	sll0218	GO:0044444	<1e-4	sll1995	GO:0017111	0.0833
sll0103	GO:0009309	<1e-4	sll0564	GO:0009892	<1e-4	sll0645	GO:0022891	<1e-4	slr1664	GO:0044425	<1e-4	sll0735	GO:0017111	0.0833
sll0647	GO:0046128	<1e-4	sll0314	GO:0080090	<1e-4	ssr0536	GO:0022892	<1e-4	slr2092	GO:0044425	<1e-4	sll1222	GO:0017111	0.0833
ssl5100	GO:0034641	<1e-4	ssl1378	GO:0006753	<1e-4	slr0147	GO:0043167	<1e-4	slr1168	GO:0044425	<1e-4	slr0108	GO:0017111	0.0833
sll1862	GO:0055086	<1e-4	ssr5092	GO:0009161	<1e-4	ssr2848	GO:0008171	<1e-4	slr0818	GO:0043226	<1e-4	sll1528	GO:0017111	0.0833
slr0374	GO:0050801	<1e-4	sll1306	GO:0043933	<1e-4	slr7094	GO:0015075	<1e-4	slr6091	GO:0044446	<1e-4	sll1446	GO:0017111	0.0833
sll1158	GO:0009260	<1e-4	sll0488	GO:0018130	<1e-4	slr1505	GO:0015293	<1e-4	ssl2595	GO:0005575	<1e-4	sll1272	GO:0017111	0.0833
slr7025	GO:0051188	<1e-4	slr1150	GO:0050896	<1e-4	sll1926	GO:0015267	<1e-4	sll1173	GO:0005575	<1e-4	ssr2142	GO:0016787	0.0829
slr1186	GO:0071840	<1e-4	slr1052	GO:0033013	<1e-4	slr1813	GO:0003674	<1e-4	slr5017	GO:0044444	<1e-4	slr1644	GO:0016787	0.0829
slr1852	GO:0046907	<1e-4	slr1407	GO:0052188	<1e-4	sll1863	GO:0015077	<1e-4	slr0870	GO:0044444	<1e-4	sll0445	GO:0005215	0.0825
slr7095	GO:0051716	<1e-4	slr0184	GO:0072521	<1e-4	slr0695	GO:0015293	<1e-4	ssr2047	GO:0044425	<1e-4	slr1917	GO:0009058	0.0813
slr1170	GO:0034660	<1e-4	slr1917	GO:0009117	<1e-4	slr5024	GO:0015399	<1e-4	slr0613	GO:0044425	<1e-4	sll1186	GO:0009058	0.0813
ssl8003	GO:0009144	<1e-4	sll1586	GO:0046164	<1e-4	sll0763	GO:0022891	<1e-4	sll0585	GO:0043226	<1e-4	slr1866	GO:0009058	0.0813
sll0157	GO:0006733	<1e-4	slr1288	GO:0019752	<1e-4	slr0606	GO:0015075	<1e-4	sll1570	GO:0044446	<1e-4	slr1441	GO:0009058	0.0813
slr6081	GO:0048518	<1e-4	sll0314	GO:0016053	<1e-4	slr1557	GO:0043167	<1e-4	ssl7048	GO:0044425	<1e-4	slr1353	GO:0009058	0.0813
sll1192	GO:0044275	<1e-4	sll1307	GO:0006721	<1e-4	ssl3142	GO:0022890	<1e-4	slr0964	GO:0043226	<1e-4	sll0547	GO:0009058	0.0813
sll1381	GO:0048522	<1e-4	slr0208	GO:0051188	<1e-4	sll0198	GO:0008171	<1e-4	slr6064	GO:0044464	<1e-4	slr1398	GO:0009058	0.0813
ssr3402	GO:0009150	<1e-4	slr7059	GO:0051171	<1e-4	sll1378	GO:0022857	<1e-4	ssr6003	GO:0044425	<1e-4	sll1218	GO:0009058	0.0813
sll0069	GO:0055086	<1e-4	slr0204	GO:0071496	<1e-4	slr7080	GO:0016817	<1e-4	sll0563	GO:0044424	<1e-4	sll1174	GO:0009058	0.0813
sll0775	GO:0009308	<1e-4	ssr3572	GO:0006163	<1e-4	sll0775	GO:0015267	<1e-4	ssr0536	GO:0044446	<1e-4	slr0305	GO:0009058	0.0813
slr0366	GO:0043549	<1e-4	ssr0336	GO:0072527	<1e-4	ssl1377	GO:0015077	<1e-4	sll6053	GO:0044446	<1e-4	slr2025	GO:0009058	0.0813
slr7091	GO:0045184	<1e-4	slr0919	GO:0044271	<1e-4	ssr2422	GO:0016741	<1e-4	slr0981	GO:0043226	<1e-4	sll0513	GO:0009058	0.0813
slr0598	GO:0033013	<1e-4	slr0787	GO:0001932	<1e-4	slr0038	GO:0015075	<1e-4	sll0926	GO:0044444	<1e-4	sll1472	GO:0009058	0.0813
sll0448	GO:0042455	<1e-4	sll1765	GO:0051347	<1e-4	slr0780	GO:0015399	<1e-4	ssr3570	GO:0044446	<1e-4	sll0301	GO:0009058	0.0813
sll1135	GO:0050794	<1e-4	slr1670	GO:0044248	<1e-4	sll0563	GO:0022857	<1e-4	slr6071	GO:0044464	<1e-4	sll0596	GO:0009058	0.0813
ssr3341	GO:0008150	<1e-4	slr1530	GO:0009991	<1e-4	slr0355	GO:0015077	<1e-4	sll1348	GO:0044422	<1e-4	sll0584	GO:0017111	0.08
sll6052	GO:0009124	<1e-4	ssl6035	GO:0006576	<1e-4	slr1143	GO:0022857	<1e-4	sll0156	GO:0044444	<1e-4	sll1763	GO:0017111	0.08
slr1563	GO:0080090	<1e-4	sll0497	GO:0006733	<1e-4	slr0554	GO:0042623	<1e-4	sll1912	GO:0043226	<1e-4	ssl0750	GO:0017111	0.08
sll2011	GO:0046394	<1e-4	sll0586	GO:0016054	<1e-4	slr0872	GO:0043167	<1e-4	slr0082	GO:0044422	<1e-4	sll1527	GO:0017111	0.08
sll1834	GO:0009056	<1e-4	slr0356	GO:0072521	<1e-4	sll0319	GO:0005342	<1e-4	slr1444	GO:0044424	<1e-4	sll1293	GO:0017111	0.08
slr1737	GO:0009141	<1e-4	slr0606	GO:0006220	<1e-4	ssr6048	GO:0022857	<1e-4	sll1946	GO:0044424	<1e-4	ssl0294	GO:0017111	0.08
slr6074	GO:0050896	<1e-4	ssl7048	GO:0008150	<1e-4	sll1447	GO:0016746	<1e-4	ssr1375	GO:0043226	<1e-4	slr1235	GO:0017111	0.08
sll5089	GO:0043549	<1e-4	ssr1765	GO:0019222	<1e-4	slr6064	GO:0003674	<1e-4	slr0358	GO:0044446	<1e-4	sll0350	GO:0017111	0.08
ssl8008	GO:0006732	<1e-4	slr1275	GO:0045184	<1e-4	sll0397	GO:0022832	<1e-4	slr0287	GO:0044422	<1e-4	slr0579	GO:0009058	0.0799
slr1611	GO:0008150	<1e-4	slr0326	GO:0034660	<1e-4	ssr1765	GO:0022892	<1e-4	sll1942	GO:0043226	<1e-4	ssr2201	GO:0009058	0.0799
slr0313	GO:0009889	<1e-4	sll0780	GO:0006733	<1e-4	slr1023	GO:0022838	<1e-4	ssr2201	GO:0043226	<1e-4	slr2018	GO:0009058	0.0799
ssl8003	GO:0051716	<1e-4	sll0181	GO:0006766	<1e-4	sll0981	GO:0022838	<1e-4	slr1875	GO:0044444	<1e-4	slr0317	GO:0009058	0.0799
slr0885	GO:0051246	<1e-4	slr2005	GO:0006733	<1e-4	slr1767	GO:0022890	<1e-4	slr6094	GO:0005575	<1e-4	sll1424	GO:0009058	0.0799
slr1935	GO:0090304	<1e-4	slr2092	GO:0006220	<1e-4	sll0984	GO:0022838	<1e-4	slr0912	GO:0044464	<1e-4	slr1303	GO:0003723	0.0788
sml0011	GO:0006811	<1e-4	slr0053	GO:0009893	<1e-4	sll1531	GO:0022891	<1e-4	slr0581	GO:0044424	<1e-4	sll1938	GO:0003723	0.0788
slr1721	GO:0048519	<1e-4	sll0702	GO:0045184	<1e-4	slr6022	GO:0022890	<1e-4	sll1573	GO:0044464	<1e-4	slr1413	GO:0003723	0.0788
sll0189	GO:0006721	<1e-4	slr0589	GO:0072528	<1e-4	sll1606	GO:0005342	<1e-4	ssr2318	GO:0044464	<1e-4	slr1762	GO:0003723	0.0788
sll1201	GO:0022607	<1e-4	slr1819	GO:0033013	<1e-4	sll7086	GO:0015399	<1e-4	sll0382	GO:0044446	<1e-4	sll1634	GO:0003723	0.0788
slr6073	GO:0006066	<1e-4	sll1738	GO:0006220	<1e-4	slr1865	GO:0015405	<1e-4	slr2038	GO:0005575	<1e-4	slr0112	GO:0003723	0.0788
sll0611	GO:0009893	<1e-4	slr1396	GO:0034654	<1e-4	slr6033	GO:0043167	<1e-4	sll0843	GO:0044425	<1e-4	ssr1552	GO:0003723	0.0788
ssr3129	GO:0006082	<1e-4	sll1381	GO:0051347	<1e-4	slr0111	GO:0060089	<1e-4	slr1179	GO:0044422	<1e-4	slr1383	GO:0003723	0.0788
slr1657	GO:0043436	<1e-4	ssr3300	GO:0009123	<1e-4	sll1004	GO:0008171	<1e-4	slr0957	GO:0005575	<1e-4	sll1372	GO:0003723	0.0788

sll1715	GO:0009259	<1e-4	sll5130	GO:0009161	<1e-4	sll0448	GO:0015405	<1e-4	slr0980	GO:0044444	<1e-4	sll1512	GO:0003723	0.0788
slr0058	GO:0055082	<1e-4	ssr6078	GO:0043549	<1e-4	sll0252	GO:0022803	<1e-4	sll1965	GO:0044425	<1e-4	sll1267	GO:0003723	0.0788
sll0269	GO:0006720	<1e-4	slr6094	GO:0006082	<1e-4	sll0141	GO:0022836	<1e-4	slr2012	GO:0005575	<1e-4	slr1169	GO:0003723	0.0788
sll0350	GO:0080090	<1e-4	sll1757	GO:0009165	<1e-4	sll1757	GO:0022832	<1e-4	ssl7022	GO:0044464	<1e-4	sll0740	GO:0003723	0.0788
sll2007	GO:0006811	<1e-4	slr1659	GO:0050794	<1e-4	slr1025	GO:0022857	<1e-4	sll1254	GO:0044444	<1e-4	sll1241	GO:0003723	0.0788
sll7087	GO:0044260	<1e-4	sll1222	GO:0006753	<1e-4	sll1106	GO:0008171	<1e-4	slr0121	GO:0044425	<1e-4	slr1074	GO:0003723	0.0788
slr1636	GO:0043648	<1e-4	sll1671	GO:0019637	<1e-4	slr0937	GO:0016746	<1e-4	ssl1918	GO:0044425	<1e-4	slr0596	GO:0003723	0.0788
slr0729	GO:0019637	<1e-4	sll0191	GO:0055114	<1e-4	sll0553	GO:0015077	<1e-4	sll0997	GO:0043226	<1e-4	sll1170	GO:0003723	0.0788
sll0230	GO:0051347	<1e-4	ssl0294	GO:0055114	<1e-4	sll5046	GO:0022857	<1e-4	slr6103	GO:0044444	<1e-4	sll0188	GO:0003723	0.0788
slr0689	GO:0022411	<1e-4	sll0846	GO:0051186	<1e-4	sll0272	GO:0015267	<1e-4	sll1652	GO:0044425	<1e-4	sll0585	GO:0003723	0.0788
sll2013	GO:0080090	<1e-4	slr7092	GO:0009057	<1e-4	sll7050	GO:0060089	<1e-4	sll1262	GO:0005575	<1e-4	slr1259	GO:0003723	0.0788
slr2011	GO:0010556	<1e-4	slr1215	GO:0046128	<1e-4	sll0994	GO:0015399	<1e-4	sll0832	GO:0005575	<1e-4	sll0981	GO:0003723	0.0788
sll0293	GO:0009991	<1e-4	slr2119	GO:0009161	<1e-4	ssl3383	GO:0003674	<1e-4	slr2046	GO:0044464	<1e-4	slr0554	GO:0003723	0.0788
ssr3129	GO:0046128	<1e-4	sll1692	GO:0072522	<1e-4	slr1576	GO:0015075	<1e-4	slr1852	GO:0044425	<1e-4	sll1738	GO:0003723	0.0788
ssl6035	GO:0044260	<1e-4	sll0283	GO:0050794	<1e-4	ssl0832	GO:0016462	<1e-4	sll1834	GO:0044464	<1e-4	slr1647	GO:0003723	0.0788
sll1163	GO:0050794	<1e-4	sll0241	GO:0009059	<1e-4	ssr7084	GO:0015405	<1e-4	slr0305	GO:0043226	<1e-4	slr0455	GO:0003723	0.0788
sll1942	GO:0043412	<1e-4	slr6039	GO:0019219	<1e-4	slr0605	GO:0022838	<1e-4	slr0157	GO:0044444	<1e-4	slr1690	GO:0003723	0.0788
ssl1577	GO:0009132	<1e-4	sll0518	GO:0005996	<1e-4	slr1900	GO:0015267	<1e-4	sll1784	GO:0005575	<1e-4	ssl3382	GO:0003723	0.0788
ssl0832	GO:0009309	<1e-4	sll1717	GO:0022607	<1e-4	slr0645	GO:0015399	<1e-4	slr6014	GO:0044444	<1e-4	slr1287	GO:0003723	0.0788
sll0656	GO:0051179	<1e-4	sll0810	GO:0071554	<1e-4	sll0327	GO:0015077	<1e-4	slr0065	GO:0044444	<1e-4	ssl0739	GO:0003723	0.0788
ssl0353	GO:0006220	<1e-4	sll1472	GO:0042180	<1e-4	slr1056	GO:0060089	<1e-4	sll5030	GO:0044446	<1e-4	slr0887	GO:0003723	0.0788
slr1163	GO:0043412	<1e-4	slr0103	GO:0009259	<1e-4	slr0645	GO:0022891	<1e-4	sll0756	GO:0044446	<1e-4	slr1827	GO:0003723	0.0788
slr0058	GO:0055114	<1e-4	slr1431	GO:0009892	<1e-4	sll1505	GO:0016741	<1e-4	sll6054	GO:0044464	<1e-4	slr7024	GO:0003723	0.0788
ssr1499	GO:0051347	<1e-4	sll1698	GO:0051188	<1e-4	sll1571	GO:0022803	<1e-4	ssl5099	GO:0044464	<1e-4	sll1411	GO:0003723	0.0788
slr2080	GO:0006220	<1e-4	ssl3142	GO:0046907	<1e-4	slr1215	GO:0015077	<1e-4	sll7055	GO:0043226	<1e-4	sll0888	GO:0003723	0.0788
slr1638	GO:0043933	<1e-4	slr0909	GO:0080090	<1e-4	slr1886	GO:0015405	<1e-4	slr1568	GO:0043226	<1e-4	slr1809	GO:0003723	0.0788
slr1034	GO:0034641	<1e-4	ssl2064	GO:0055114	<1e-4	slr6081	GO:0043167	<1e-4	slr0845	GO:0044446	<1e-4	sll0156	GO:0003723	0.0788
sll1424	GO:0022411	<1e-4	sll5097	GO:0046907	<1e-4	ssl7007	GO:0022832	<1e-4	slr1753	GO:0043226	<1e-4	sll0669	GO:0003723	0.0788
slr0634	GO:0009165	<1e-4	slr0082	GO:0009260	<1e-4	slr0355	GO:0016817	<1e-4	slr0978	GO:0044446	<1e-4	sll0564	GO:0003723	0.0788
slr6101	GO:0072527	<1e-4	slr6081	GO:0008610	<1e-4	slr2119	GO:0016817	<1e-4	slr0106	GO:0044424	<1e-4	sll0752	GO:0003723	0.0788
sll0611	GO:0009144	<1e-4	sll7077	GO:0051234	<1e-4	slr1958	GO:0022892	<1e-4	slr1260	GO:0044446	<1e-4	slr0373	GO:0003723	0.0788
sll0793	GO:0046394	<1e-4	slr1056	GO:0009987	<1e-4	sll0595	GO:0003674	<1e-4	sll1912	GO:0044464	<1e-4	slr0789	GO:0003723	0.0788
sll0518	GO:0071841	<1e-4	slr0243	GO:0009991	<1e-4	ssl5045	GO:0016817	<1e-4	slr0498	GO:0005575	<1e-4	sll1541	GO:0003723	0.0788
sll5004	GO:0006811	<1e-4	sll1265	GO:0006732	<1e-4	sll5063	GO:0015405	<1e-4	slr1095	GO:0043226	<1e-4	ssr2754	GO:0003723	0.0788
sll1025	GO:0043549	<1e-4	slr2092	GO:0009893	<1e-4	sll0539	GO:0015267	<1e-4	sll1485	GO:0044422	<1e-4	sll1173	GO:0003723	0.0788
sll1913	GO:0051179	<1e-4	sll0022	GO:0055114	<1e-4	sll0788	GO:0022832	<1e-4	ssl5113	GO:0044424	<1e-4	sll1399	GO:0003723	0.0788
slr0318	GO:0044282	<1e-4	sll0861	GO:0009991	<1e-4	ssr3570	GO:0022832	<1e-4	sll0293	GO:0044444	<1e-4	slr2048	GO:0003723	0.0788
sll0611	GO:0060255	<1e-4	sll0301	GO:0034654	<1e-4	slr0852	GO:0015291	<1e-4	slr7099	GO:0044444	<1e-4	slr1338	GO:0003723	0.0788
slr1767	GO:0008150	<1e-4	sll1562	GO:0019752	<1e-4	sll0481	GO:0003674	<1e-4	slr0596	GO:0044424	<1e-4	sll0871	GO:0003723	0.0788
sll0176	GO:0050794	<1e-4	slr1977	GO:0019752	<1e-4	sll0141	GO:0015075	<1e-4	slr1571	GO:0044464	<1e-4	slr0318	GO:0003723	0.0788
sll1054	GO:0055082	<1e-4	slr7091	GO:0006811	<1e-4	sll1613	GO:0015267	<1e-4	slr5102	GO:0044464	<1e-4	sll1659	GO:0003723	0.0788
sll1658	GO:0044260	<1e-4	slr0751	GO:0051234	<1e-4	slr0755	GO:0015405	<1e-4	sll1130	GO:0044422	<1e-4	slr1122	GO:0003723	0.0788
slr1047	GO:0046483	<1e-4	slr1999	GO:0019752	<1e-4	ssr2439	GO:0022838	<1e-4	slr7057	GO:0044446	<1e-4	slr2115	GO:0003723	0.0788
sll0981	GO:0065007	<1e-4	slr1864	GO:0010556	<1e-4	ssr1951	GO:0022832	<1e-4	sll0552	GO:0005575	<1e-4	slr0801	GO:0003723	0.0788
ssr5074	GO:0006720	<1e-4	slr1053	GO:0006733	<1e-4	slr0459	GO:0016746	<1e-4	slr0607	GO:0005575	<1e-4	slr0358	GO:0003723	0.0788
sll1714	GO:0051246	<1e-4	ssl2814	GO:0051186	<1e-4	sll1583	GO:0022857	<1e-4	ssr2201	GO:0044464	<1e-4	slr1812	GO:0003676	0.0782
slr2121	GO:0009889	<1e-4	sll1024	GO:0034641	<1e-4	slr1998	GO:0043492	<1e-4	ssl0312	GO:0044425	<1e-4	sll0413	GO:0003676	0.0782
slr1753	GO:0043436	<1e-4	slr0516	GO:0009262	<1e-4	sll1737	GO:0022838	<1e-4	sll0595	GO:0044425	<1e-4	slr0108	GO:0003676	0.0781
sll1372	GO:0009991	<1e-4	slr1704	GO:0051246	<1e-4	sll1882	GO:0016741	<1e-4	sll5030	GO:0044424	<1e-4	sll1528	GO:0003676	0.0781
slr1033	GO:0072524	<1e-4	sll0314	GO:0009132	<1e-4	sll0274	GO:0022836	<1e-4	slr1658	GO:0044424	<1e-4	slr1591	GO:0003676	0.0781
ssr7079	GO:0051246	<1e-4	sll1151	GO:0046164	<1e-4	slr1980	GO:0022836	<1e-4	slr0581	GO:0044446	<1e-4	slr0818	GO:0003676	0.0781
slr0725	GO:0016052	<1e-4	sll0911	GO:0006732	<1e-4	slr1780	GO:0060089	<1e-4	sll1866	GO:0044424	<1e-4	ssr3154	GO:0003676	0.0781
slr0815	GO:0022411	<1e-4	sll1552	GO:0034660	<1e-4	slr0273	GO:0015405	<1e-4	sll0160	GO:0044422	<1e-4	slr0642	GO:0003676	0.0781
slr2121	GO:0009150	<1e-4	ssl1263	GO:0044281	<1e-4	slr1142	GO:0060089	<1e-4	sll0382	GO:0044425	<1e-4	sll0735	GO:0003676	0.0781

sll1911	GO:0048518	<1e-4	sll2013	GO:0006721	<1e-4	slr7099	GO:0022836	<1e-4	ssr6030	GO:0044444	<1e-4	slr1235	GO:0003676	0.0777
sll7086	GO:0046164	<1e-4	sll1659	GO:0051716	<1e-4	slr1956	GO:0015399	<1e-4	sll1960	GO:0044446	<1e-4	sll1527	GO:0003676	0.0777
slr1813	GO:0051171	<1e-4	sll0274	GO:0071840	<1e-4	slr0325	GO:0022890	<1e-4	slr2005	GO:0043226	<1e-4	slr1800	GO:0009058	0.0771
slr2000	GO:0006066	<1e-4	sll0743	GO:0006811	<1e-4	sll0444	GO:0042623	<1e-4	sll0446	GO:0044446	<1e-4	ssl2996	GO:0009058	0.0771
sll2007	GO:0009161	<1e-4	slr1800	GO:0072522	<1e-4	ssl6035	GO:0042623	<1e-4	slr0664	GO:0044424	<1e-4	slr1444	GO:0009058	0.0771
ssl8008	GO:0071554	<1e-4	slr1611	GO:0019222	<1e-4	ssr2615	GO:0015405	<1e-4	slr1546	GO:0044424	<1e-4	sll0558	GO:0009058	0.0771
sll0749	GO:0072522	<1e-4	ssl1792	GO:0006811	<1e-4	slr2025	GO:0022838	<1e-4	ssr3532	GO:0043226	<1e-4	sll1526	GO:0009058	0.0771
ssl3451	GO:0048878	<1e-4	sll0584	GO:0048518	<1e-4	slr1472	GO:0016817	<1e-4	sll0382	GO:0044464	<1e-4	ssr1391	GO:0009058	0.0771
slr1895	GO:0006793	<1e-4	sll0785	GO:0050794	<1e-4	ssl5031	GO:0015293	<1e-4	slr0816	GO:0044444	<1e-4	slr0594	GO:0009058	0.0771
sll8011	GO:0006220	<1e-4	slr1470	GO:0071554	<1e-4	slr1163	GO:0022892	<1e-4	slr0249	GO:0044422	<1e-4	sll0931	GO:0017111	0.0768
slr0588	GO:0043549	<1e-4	ssl1690	GO:0006163	<1e-4	sll1414	GO:0016462	<1e-4	sll1979	GO:0044425	<1e-4	slr1636	GO:0017111	0.0768
sll1219	GO:0008150	<1e-4	sll1660	GO:0072524	<1e-4	sll0274	GO:0042623	<1e-4	sll1089	GO:0044444	<1e-4	slr1812	GO:0017111	0.0768
ssl7045	GO:0009161	<1e-4	sll1715	GO:0044255	<1e-4	sll1265	GO:0022890	<1e-4	sll0926	GO:0043226	<1e-4	sll0149	GO:0000166	0.0735
ssl2471	GO:0048523	<1e-4	sll0071	GO:0050801	<1e-4	slr6090	GO:0022832	<1e-4	slr0217	GO:0044464	<1e-4	sll0242	GO:0016787	0.0723
sll1880	GO:0044003	<1e-4	sll1068	GO:0048522	<1e-4	slr0863	GO:0015075	<1e-4	slr0423	GO:0044444	<1e-4	slr1915	GO:0016787	0.0719
ssl8005	GO:0008150	<1e-4	sll1476	GO:0006066	<1e-4	slr1807	GO:0022832	<1e-4	slr8021	GO:0044424	<1e-4	ssl2971	GO:0016787	0.0719
sll0282	GO:0046128	<1e-4	slr1951	GO:0006066	<1e-4	sll8032	GO:0016746	<1e-4	sll1348	GO:0005575	<1e-4	sll0563	GO:0016787	0.0719
ssr2317	GO:0072528	<1e-4	slr1163	GO:0008610	<1e-4	sll0361	GO:0022891	<1e-4	sll0503	GO:0044444	<1e-4	sll5067	GO:0016787	0.0719
slr0664	GO:0055086	<1e-4	ssr0692	GO:0006576	<1e-4	slr0872	GO:0042623	<1e-4	sll0230	GO:0044424	<1e-4	ssr0657	GO:0016787	0.0719
sll7033	GO:0051234	<1e-4	slr1900	GO:0009117	<1e-4	sll5044	GO:0022857	<1e-4	ssr1765	GO:0044464	<1e-4	slr1087	GO:0016787	0.0719
slr0238	GO:0009059	<1e-4	sll1495	GO:0072527	<1e-4	slr1917	GO:0005342	<1e-4	slr0975	GO:0044424	<1e-4	ssl5025	GO:0016787	0.0719
sll0189	GO:0009126	<1e-4	slr1917	GO:0042451	<1e-4	sll1784	GO:0022891	<1e-4	slr2122	GO:0005575	<1e-4	ssl3573	GO:0016787	0.0719
slr0667	GO:0019637	<1e-4	sll7034	GO:0072521	<1e-4	slr0514	GO:0022892	<1e-4	slr1599	GO:0043226	<1e-4	slr6045	GO:0016787	0.0719
slr0404	GO:0008150	<1e-4	slr0262	GO:0019637	<1e-4	ssr8047	GO:0015291	<1e-4	sll1160	GO:0044424	<1e-4	sll1717	GO:0016787	0.0719
ssr6048	GO:0009889	<1e-4	slr0195	GO:0006220	<1e-4	slr1203	GO:0022857	<1e-4	ssr1114	GO:0044424	<1e-4	slr1101	GO:0016787	0.0719
sll0925	GO:0055082	<1e-4	sll1142	GO:0051186	<1e-4	slr1534	GO:0005342	<1e-4	slr1571	GO:0044424	<1e-4	slr0521	GO:0016787	0.0719
slr1442	GO:0006066	<1e-4	slr1174	GO:0009987	<1e-4	slr1068	GO:0015291	<1e-4	ssl0787	GO:0043226	<1e-4	ssr3570	GO:0016787	0.0719
sll1469	GO:0065007	<1e-4	slr1071	GO:0009991	<1e-4	slr0924	GO:0015293	<1e-4	sll1643	GO:0044446	<1e-4	slr0865	GO:0016787	0.0719
ssr7079	GO:0022607	<1e-4	ssr2318	GO:0009132	<1e-4	slr1918	GO:0015077	<1e-4	slr0957	GO:0043226	<1e-4	slr0863	GO:0016787	0.0719
slr8022	GO:0009059	<1e-4	slr0325	GO:0006631	<1e-4	slr0957	GO:0015293	<1e-4	slr2000	GO:0044425	<1e-4	slr0888	GO:0016787	0.0719
ssr6048	GO:0065007	<1e-4	sll1072	GO:0051188	<1e-4	slr0392	GO:0022857	<1e-4	sll1426	GO:0044446	<1e-4	ssr0109	GO:0016787	0.0719
sll5097	GO:0031326	<1e-4	slr1074	GO:0019752	<1e-4	sll0047	GO:0015077	<1e-4	slr0392	GO:0044446	<1e-4	sll1254	GO:0016787	0.0719
slr1187	GO:0051347	<1e-4	sll1089	GO:0006720	<1e-4	slr1383	GO:0008171	<1e-4	sll0192	GO:0005575	<1e-4	ssr7079	GO:0016787	0.0719
ssr6003	GO:0044283	<1e-4	sll1411	GO:0046164	<1e-4	slr2010	GO:0016462	<1e-4	slr1812	GO:0005575	<1e-4	sll0736	GO:0016787	0.0719
sll0066	GO:0031323	<1e-4	ssl7022	GO:0051171	<1e-4	slr1095	GO:0016746	<1e-4	slr0642	GO:0044425	<1e-4	slr0285	GO:0016787	0.0719
sll1446	GO:0045184	<1e-4	ssl0832	GO:0051186	<1e-4	slr1920	GO:0016462	<1e-4	slr0589	GO:0044444	<1e-4	slr2000	GO:0016787	0.0719
slr0656	GO:0050789	<1e-4	sll0656	GO:0090304	<1e-4	slr1169	GO:0042623	<1e-4	slr0588	GO:0044425	<1e-4	slr0269	GO:0016787	0.0719
slr0006	GO:0090304	<1e-4	sll1348	GO:0071842	<1e-4	sll1512	GO:0043492	<1e-4	sll0886	GO:0044464	<1e-4	ssl3549	GO:0016787	0.0719
ssl7007	GO:0046907	<1e-4	slr1774	GO:0022411	<1e-4	slr1150	GO:0015075	<1e-4	slr1644	GO:0043226	<1e-4	sll1192	GO:0016787	0.0719
sll0293	GO:0019222	<1e-4	sll1757	GO:0009893	<1e-4	ssr5074	GO:0005342	<1e-4	sll0638	GO:0005575	<1e-4	sll1036	GO:0016787	0.0719
sll1797	GO:0009161	<1e-4	slr1920	GO:0072521	<1e-4	slr1468	GO:0060089	<1e-4	ssl2471	GO:0044446	<1e-4	sll1068	GO:0016787	0.0719
sll1233	GO:0031326	<1e-4	slr8044	GO:0044275	<1e-4	slr7095	GO:0022857	<1e-4	slr1895	GO:0044446	<1e-4	slr0645	GO:0016787	0.0719
slr1563	GO:0051234	<1e-4	sll0198	GO:0016053	<1e-4	slr0149	GO:0016741	<1e-4	ssl3382	GO:0044425	<1e-4	sll1726	GO:0016787	0.0719
slr1970	GO:0033013	<1e-4	sll0284	GO:0044282	<1e-4	sll0761	GO:0016818	<1e-4	slr2117	GO:0005575	<1e-4	ssl2595	GO:0016787	0.0719
sll0181	GO:0044275	<1e-4	ssr8013	GO:0010468	<1e-4	slr0285	GO:0015405	<1e-4	slr1462	GO:0044444	<1e-4	slr0397	GO:0016787	0.0719
ssl5015	GO:0050801	<1e-4	slr1068	GO:0048522	<1e-4	sll0611	GO:0060089	<1e-4	slr1384	GO:0005575	<1e-4	slr0755	GO:0016787	0.0719
slr1261	GO:0050801	<1e-4	slr1441	GO:0009199	<1e-4	ssr2439	GO:0022836	<1e-4	sll1170	GO:0044422	<1e-4	sll1698	GO:0016787	0.0719
sll8019	GO:0016052	<1e-4	slr6038	GO:0006220	<1e-4	sll0939	GO:0022838	<1e-4	slr1923	GO:0044422	<1e-4	slr6087	GO:0016787	0.0719
ssr6002	GO:0008610	<1e-4	slr6094	GO:0009260	<1e-4	sll0272	GO:0015399	<1e-4	sll1429	GO:0044446	<1e-4	sll1583	GO:0016787	0.0719
sll8012	GO:0051818	<1e-4	sll5003	GO:0008610	<1e-4	sll1233	GO:0022891	<1e-4	slr1827	GO:0044446	<1e-4	sll8040	GO:0016787	0.0719
slr1623	GO:0009141	<1e-4	ssr8047	GO:0051171	<1e-4	slr0264	GO:0022892	<1e-4	slr5073	GO:0043226	<1e-4	ssr2972	GO:0016787	0.0719
slr1261	GO:0009141	<1e-4	sll1573	GO:0009117	<1e-4	slr1852	GO:0016817	<1e-4	sll0325	GO:0044425	<1e-4	slr1681	GO:0016787	0.0719
slr0169	GO:0006732	<1e-4	slr1276	GO:0009165	<1e-4	slr0272	GO:0060089	<1e-4	sll0249	GO:0044425	<1e-4	slr1442	GO:0016787	0.0719
slr1301	GO:0009056	<1e-4	ssr0109	GO:0008150	<1e-4	slr0408	GO:0015293	<1e-4	slr1809	GO:0005575	<1e-4	ssr6089	GO:0016787	0.0719

slr1464	GO:0009262	<1e-4	ssr6048	GO:0044283	<1e-4	sll1582	GO:0008171	<1e-4	sll0381	GO:0005575	<1e-4	slr0667	GO:0016787	0.0719
sll0071	GO:0034654	<1e-4	slr0709	GO:0043412	<1e-4	sll0614	GO:0015267	<1e-4	slr0108	GO:0044446	<1e-4	slr0731	GO:0016787	0.0719
sll6055	GO:0050794	<1e-4	sll1233	GO:0072524	<1e-4	ssr5020	GO:0022857	<1e-4	sll1714	GO:0044422	<1e-4	sll0044	GO:0016787	0.0719
slr0273	GO:0044106	<1e-4	slr1815	GO:0019637	<1e-4	sll0149	GO:0016746	<1e-4	slr0909	GO:0044422	<1e-4	slr7091	GO:0016787	0.0719
slr2010	GO:0016053	<1e-4	ssl3383	GO:0044260	<1e-4	sll6052	GO:0005342	<1e-4	ssr2843	GO:0044446	<1e-4	ssr3467	GO:0016787	0.0719
ssl5027	GO:0048523	<1e-4	slr1101	GO:0016053	<1e-4	slr0594	GO:0015267	<1e-4	slr0592	GO:0044446	<1e-4	slr6016	GO:0016787	0.0719
slr7101	GO:0009892	<1e-4	slr0199	GO:0009309	<1e-4	sll0410	GO:0022838	<1e-4	sll0372	GO:0044464	<1e-4	slr6029	GO:0016787	0.0719
slr1913	GO:0009260	<1e-4	sll1738	GO:0009165	<1e-4	sll1414	GO:0060089	<1e-4	slr0285	GO:0044446	<1e-4	slr2070	GO:0016787	0.0719
sll7087	GO:0051716	<1e-4	sll1359	GO:0048519	<1e-4	slr1230	GO:0016462	<1e-4	sll5044	GO:0043226	<1e-4	slr0634	GO:0016787	0.0719
slr1998	GO:0052188	<1e-4	sll0586	GO:0042451	<1e-4	sll1388	GO:0043167	<1e-4	slr0699	GO:0043226	<1e-4	sll0060	GO:0016787	0.0719
slr0935	GO:0072521	<1e-4	slr6075	GO:0044255	<1e-4	slr0975	GO:0022838	<1e-4	sll0742	GO:0044425	<1e-4	sll7067	GO:0016787	0.0719
ssl1417	GO:0009144	<1e-4	sll0410	GO:0046483	<1e-4	slr0006	GO:0015399	<1e-4	slr0243	GO:0005575	<1e-4	sll0670	GO:0016787	0.0719
sll1400	GO:0006631	<1e-4	sll1240	GO:0019362	<1e-4	ssr2848	GO:0022890	<1e-4	sll0176	GO:0043226	<1e-4	sll0321	GO:0016787	0.0719
slr1188	GO:0009126	<1e-4	slr1600	GO:0019752	<1e-4	slr6064	GO:0022891	<1e-4	sll0676	GO:0044446	<1e-4	sll7006	GO:0016787	0.0719
sll1902	GO:0010468	<1e-4	sll7069	GO:0050801	<1e-4	sll1692	GO:0015291	<1e-4	ssr3122	GO:0043226	<1e-4	ssr2711	GO:0016787	0.0719
sll0283	GO:0033013	<1e-4	slr1530	GO:0019637	<1e-4	slr2027	GO:0043167	<1e-4	slr1886	GO:0005575	<1e-4	slr1670	GO:0016787	0.0719
slr1235	GO:0009161	<1e-4	slr0769	GO:0008150	<1e-4	slr0334	GO:0022832	<1e-4	slr1939	GO:0005575	<1e-4	ssr6062	GO:0016787	0.0719
ssl0461	GO:0010468	<1e-4	slr0456	GO:0071840	<1e-4	slr1262	GO:0022857	<1e-4	slr1184	GO:0044446	<1e-4	ssl2814	GO:0016787	0.0719
slr1398	GO:0051246	<1e-4	slr0109	GO:0071841	<1e-4	sll1396	GO:0060089	<1e-4	sll1273	GO:0043226	<1e-4	slr0976	GO:0016787	0.0719
slr1601	GO:0055114	<1e-4	sll0702	GO:0016052	<1e-4	ssr1041	GO:0015075	<1e-4	sll0933	GO:0044444	<1e-4	slr6044	GO:0016787	0.0719
ssr2802	GO:0071842	<1e-4	sll1273	GO:0009260	<1e-4	slr0243	GO:0022891	<1e-4	slr1814	GO:0044425	<1e-4	slr2060	GO:0016787	0.0719
slr1959	GO:0009889	<1e-4	slr0147	GO:0019362	<1e-4	slr1276	GO:0015077	<1e-4	sll1874	GO:0044444	<1e-4	slr0271	GO:0016787	0.0719
ssl2971	GO:0065007	<1e-4	sll2007	GO:0048519	<1e-4	slr1799	GO:0015077	<1e-4	slr0404	GO:0044464	<1e-4	slr1886	GO:0016787	0.0719
slr1342	GO:0019637	<1e-4	slr0184	GO:0043412	<1e-4	sll0625	GO:0022836	<1e-4	sll1784	GO:0044422	<1e-4	sll0905	GO:0016787	0.0719
slr0217	GO:0006066	<1e-4	slr1913	GO:0050896	<1e-4	slr1210	GO:0016818	<1e-4	ssr0335	GO:0044446	<1e-4	ssr6078	GO:0016787	0.0719
sll0172	GO:0009117	<1e-4	slr0575	GO:0044271	<1e-4	sll5062	GO:0022857	<1e-4	ssl8039	GO:0044422	<1e-4	slr6081	GO:0016787	0.0719
sll0909	GO:0034660	<1e-4	sll1442	GO:0071496	<1e-4	slr2071	GO:0022891	<1e-4	slr1647	GO:0044422	<1e-4	slr0300	GO:0016787	0.0719
sll0442	GO:0006066	<1e-4	slr6004	GO:0009123	<1e-4	slr1571	GO:0022832	<1e-4	ssl1498	GO:0044422	<1e-4	sll1784	GO:0016787	0.0719
sll1527	GO:0009126	<1e-4	ssl0242	GO:0071554	<1e-4	sll0997	GO:0008171	<1e-4	ssr3304	GO:0044422	<1e-4	sll5004	GO:0016787	0.0719
slr1398	GO:0051186	<1e-4	sll0847	GO:0006082	<1e-4	slr1069	GO:0043167	<1e-4	sll0225	GO:0044446	<1e-4	ssr6027	GO:0016787	0.0719
slr1964	GO:0009987	<1e-4	ssl7022	GO:0051347	<1e-4	slr0304	GO:0016741	<1e-4	ssl2065	GO:0044425	<1e-4	sll1340	GO:0016787	0.0719
slr1340	GO:0043933	<1e-4	ssr2615	GO:0055086	<1e-4	ssr5117	GO:0015405	<1e-4	slr2118	GO:0005575	<1e-4	slr7099	GO:0016787	0.0719
sll0069	GO:0022607	<1e-4	slr0601	GO:0009394	<1e-4	sll0837	GO:0022892	<1e-4	slr0050	GO:0044444	<1e-4	sll0696	GO:0016787	0.0719
sll8012	GO:0009308	<1e-4	sll1651	GO:0006793	<1e-4	ssr2318	GO:0003674	<1e-4	sll1426	GO:0044425	<1e-4	ssr0336	GO:0016787	0.0719
slr1117	GO:0009889	<1e-4	sll0072	GO:0051186	<1e-4	slr1577	GO:0003674	<1e-4	sll5090	GO:0005575	<1e-4	ssr6024	GO:0016787	0.0719
slr0964	GO:0006732	<1e-4	sll1262	GO:0009117	<1e-4	slr1307	GO:0042623	<1e-4	sll0192	GO:0044444	<1e-4	slr5111	GO:0016787	0.0719
sll1262	GO:0006720	<1e-4	sll7077	GO:0044271	<1e-4	slr0211	GO:0022891	<1e-4	sll0905	GO:0044424	<1e-4	ssl1046	GO:0016787	0.0719
slr6064	GO:0034641	<1e-4	slr0360	GO:0009263	<1e-4	sll5004	GO:0022803	<1e-4	slr1809	GO:0044444	<1e-4	slr6091	GO:0016787	0.0719
slr0656	GO:0071554	<1e-4	slr0144	GO:0034654	<1e-4	slr1303	GO:0015399	<1e-4	sll1950	GO:0005575	<1e-4	slr1628	GO:0016787	0.0719
sll1004	GO:0072522	<1e-4	slr7016	GO:0050896	<1e-4	sll0505	GO:0022892	<1e-4	slr1600	GO:0005575	<1e-4	slr1394	GO:0016787	0.0719
ssr6048	GO:0046164	<1e-4	sll0788	GO:0046164	<1e-4	sll0238	GO:0015293	<1e-4	slr0249	GO:0044446	<1e-4	ssl7048	GO:0016787	0.0719
sll1477	GO:0031323	<1e-4	sll1715	GO:0043436	<1e-4	sml0011	GO:0022892	<1e-4	ssl2384	GO:0044464	<1e-4	ssr6083	GO:0016787	0.0719
sll8032	GO:0009141	<1e-4	slr0262	GO:0044283	<1e-4	slr2105	GO:0015077	<1e-4	slr0869	GO:0043226	<1e-4	ssl2064	GO:0016787	0.0719
slr1847	GO:0008150	<1e-4	slr0416	GO:0046394	<1e-4	ssl2781	GO:0015293	<1e-4	sll0735	GO:0044425	<1e-4	slr5102	GO:0016787	0.0719
slr1413	GO:0006766	<1e-4	slr1667	GO:0034641	<1e-4	ssr2912	GO:0005342	<1e-4	sll1946	GO:0043226	<1e-4	slr6104	GO:0016787	0.0719
sll0048	GO:0006811	<1e-4	slr1218	GO:0050801	<1e-4	sll0282	GO:0022857	<1e-4	slr1236	GO:0044422	<1e-4	slr5024	GO:0016787	0.0719
ssl2138	GO:0009144	<1e-4	ssr7093	GO:0018130	<1e-4	ssl0738	GO:0005342	<1e-4	slr1394	GO:0044422	<1e-4	slr0291	GO:0016787	0.0719
sll0565	GO:0031323	<1e-4	slr2027	GO:0051347	<1e-4	slr5116	GO:0043492	<1e-4	slr1097	GO:0044425	<1e-4	slr0699	GO:0016787	0.0719
slr0482	GO:0043933	<1e-4	slr1674	GO:0048518	<1e-4	sll1188	GO:0015077	<1e-4	sll5026	GO:0044422	<1e-4	sll0405	GO:0016787	0.0719
sll0451	GO:0072528	<1e-4	slr0780	GO:0072522	<1e-4	slr1753	GO:0022838	<1e-4	slr1718	GO:0043226	<1e-4	slr7073	GO:0016787	0.0719
ssr2787	GO:0006766	<1e-4	sll0238	GO:0043170	<1e-4	sll1318	GO:0022836	<1e-4	slr2042	GO:0044425	<1e-4	ssl7038	GO:0016787	0.0719
slr1690	GO:0034641	<1e-4	sll0266	GO:0009141	<1e-4	slr0334	GO:0060089	<1e-4	slr6051	GO:0044425	<1e-4	slr5112	GO:0016787	0.0719
sll1254	GO:0010468	<1e-4	slr0397	GO:0019751	<1e-4	ssl1918	GO:0022857	<1e-4	ssr7017	GO:0044424	<1e-4	sll0062	GO:0016787	0.0719
slr6106	GO:0009893	<1e-4	slr8021	GO:0006720	<1e-4	ssr6089	GO:0022836	<1e-4	sll5109	GO:0044425	<1e-4	slr7094	GO:0016787	0.0719

slr1721	GO:0052188	<1e-4	slr0503	GO:0050789	<1e-4	slr6031	GO:0043167	<1e-4	sll7067	GO:0044446	<1e-4	sll1095	GO:0016787	0.0719
ssl5114	GO:0010468	<1e-4	slr1339	GO:0043549	<1e-4	slr0667	GO:0022857	<1e-4	slr0924	GO:0044446	<1e-4	sll0678	GO:0016787	0.0719
slr1062	GO:0006793	<1e-4	sll1442	GO:0006753	<1e-4	sll0702	GO:0016741	<1e-4	slr0789	GO:0005575	<1e-4	slr6103	GO:0016787	0.0719
sll0167	GO:0044283	<1e-4	slr1998	GO:0006721	<1e-4	sll1186	GO:0016462	<1e-4	sll0286	GO:0044425	<1e-4	sll1158	GO:0016787	0.0719
sll0639	GO:0090304	<1e-4	slr0784	GO:0055086	<1e-4	ssr2975	GO:0015075	<1e-4	slr7099	GO:0044464	<1e-4	sll5030	GO:0016787	0.0719
slr1098	GO:0051171	<1e-4	slr0569	GO:0006066	<1e-4	sll0505	GO:0016818	<1e-4	slr1534	GO:0044464	<1e-4	sll7033	GO:0016787	0.0719
ssl0109	GO:0071840	<1e-4	sll7078	GO:0051234	<1e-4	sll0325	GO:0022803	<1e-4	slr1436	GO:0044425	<1e-4	sll1692	GO:0016787	0.0719
slr0596	GO:0009123	<1e-4	sll0376	GO:0009260	<1e-4	slr6072	GO:0016462	<1e-4	ssr2781	GO:0005575	<1e-4	sll1233	GO:0016787	0.0719
ssr3300	GO:0022411	<1e-4	slr0872	GO:0065007	<1e-4	sll1411	GO:0043167	<1e-4	slr1726	GO:0005575	<1e-4	slr1450	GO:0016787	0.0719
sll1926	GO:0034654	<1e-4	slr0238	GO:0034660	<1e-4	slr0243	GO:0016462	<1e-4	sll1830	GO:0044422	<1e-4	sll0552	GO:0016787	0.0719
slr1084	GO:0050789	<1e-4	slr1880	GO:0006753	<1e-4	slr1880	GO:0060089	<1e-4	sll7087	GO:0044464	<1e-4	sll1531	GO:0016787	0.0719
slr0318	GO:0034654	<1e-4	ssl3383	GO:0016054	<1e-4	slr1073	GO:0022890	<1e-4	sll0185	GO:0044425	<1e-4	slr5053	GO:0016787	0.0719
slr0364	GO:0006066	<1e-4	sll1656	GO:0050801	<1e-4	slr0601	GO:0015077	<1e-4	slr1306	GO:0044424	<1e-4	slr1980	GO:0016787	0.0719
sll0703	GO:0042180	<1e-4	slr1726	GO:0009057	<1e-4	slr0204	GO:0015291	<1e-4	sll1396	GO:0044464	<1e-4	slr0948	GO:0016787	0.0719
slr0967	GO:0010468	<1e-4	sll0505	GO:0051186	<1e-4	slr0482	GO:0015267	<1e-4	ssl1464	GO:0044424	<1e-4	sll1911	GO:0016787	0.0719
sll0846	GO:0046128	<1e-4	sll0024	GO:0009132	<1e-4	slr0476	GO:0015405	<1e-4	slr5101	GO:0044446	<1e-4	ssr5092	GO:0016787	0.0719
sll0101	GO:0060255	<1e-4	ssl0483	GO:0043549	<1e-4	ssr3571	GO:0022838	<1e-4	sll0236	GO:0043226	<1e-4	sll0157	GO:0016787	0.0719
slr0509	GO:0043549	<1e-4	sll1965	GO:0051186	<1e-4	sll7077	GO:0015077	<1e-4	slr0337	GO:0044444	<1e-4	slr1114	GO:0016787	0.0719
slr0521	GO:0019637	<1e-4	slr2010	GO:0044003	<1e-4	slr5111	GO:0008171	<1e-4	slr0670	GO:0043226	<1e-4	slr0345	GO:0016787	0.0719
sll0822	GO:0052188	<1e-4	ssr5106	GO:0050794	<1e-4	slr1353	GO:0015293	<1e-4	slr0818	GO:0005575	<1e-4	ssl7074	GO:0016787	0.0719
slr1493	GO:0009142	<1e-4	slr0453	GO:0009132	<1e-4	sll0702	GO:0015399	<1e-4	sll1217	GO:0044424	<1e-4	slr0498	GO:0016787	0.0719
sll0284	GO:0006082	<1e-4	slr0023	GO:0006721	<1e-4	sll8001	GO:0015291	<1e-4	slr1068	GO:0044424	<1e-4	slr0771	GO:0016787	0.0719
ssr6020	GO:0006091	<1e-4	slr0872	GO:0009161	<1e-4	slr0680	GO:0042623	<1e-4	ssl2971	GO:0005575	<1e-4	slr8044	GO:0016787	0.0719
ssl8005	GO:0043648	<1e-4	slr0431	GO:0006721	<1e-4	slr7013	GO:0022838	<1e-4	slr0670	GO:0044464	<1e-4	slr0670	GO:0016787	0.0719
sll0031	GO:0009124	<1e-4	slr0729	GO:0006733	<1e-4	sll0188	GO:0043492	<1e-4	sll1071	GO:0044446	<1e-4	ssr8047	GO:0016787	0.0719
sll1318	GO:0019222	<1e-4	ssr1768	GO:0072527	<1e-4	ssl1377	GO:0016817	<1e-4	sll0424	GO:0044446	<1e-4	slr2110	GO:0016787	0.0719
sll1749	GO:0006163	<1e-4	ssl8005	GO:0055114	<1e-4	slr7099	GO:0022803	<1e-4	sll1609	GO:0005575	<1e-4	slr0708	GO:0016787	0.0719
slr0770	GO:0072527	<1e-4	sll1378	GO:0019637	<1e-4	slr1474	GO:0005342	<1e-4	sll0524	GO:0044446	<1e-4	sll7034	GO:0016787	0.0719
slr2049	GO:0019752	<1e-4	slr0729	GO:0009123	<1e-4	ssr5074	GO:0016746	<1e-4	slr1676	GO:0044446	<1e-4	sll0298	GO:0016787	0.0719
sll0760	GO:0051234	<1e-4	sll0804	GO:0051234	<1e-4	sll1752	GO:0022891	<1e-4	slr1218	GO:0044424	<1e-4	sll0372	GO:0016787	0.0719
ssl2971	GO:0072522	<1e-4	slr1533	GO:0019222	<1e-4	slr0812	GO:0015405	<1e-4	slr2092	GO:0044422	<1e-4	slr7060	GO:0016787	0.0719
sll1054	GO:0006163	<1e-4	sll0101	GO:0071840	<1e-4	slr7080	GO:0008171	<1e-4	slr0294	GO:0044446	<1e-4	sll0925	GO:0016787	0.0719
sll1006	GO:0060255	<1e-4	sll1632	GO:0043436	<1e-4	sll2011	GO:0060089	<1e-4	slr0400	GO:0005575	<1e-4	sll0875	GO:0016787	0.0719
sll6055	GO:0033013	<1e-4	slr0356	GO:0050794	<1e-4	sll0272	GO:0022832	<1e-4	sll0268	GO:0005575	<1e-4	slr5037	GO:0016787	0.0719
sll7064	GO:0055086	<1e-4	slr0909	GO:0006082	<1e-4	sll0647	GO:0022890	<1e-4	sll0360	GO:0044424	<1e-4	ssr6002	GO:0016787	0.0719
slr1098	GO:0006066	<1e-4	slr1259	GO:0034660	<1e-4	ssr2803	GO:0003674	<1e-4	slr0619	GO:0005575	<1e-4	sll1940	GO:0016787	0.0719
slr1957	GO:0043170	<1e-4	ssr2009	GO:0009161	<1e-4	slr1101	GO:0008171	<1e-4	ssr6024	GO:0044446	<1e-4	ssr2067	GO:0016787	0.0719
slr1611	GO:0042180	<1e-4	sll0595	GO:0090304	<1e-4	sll0309	GO:0015077	<1e-4	ssl3383	GO:0044422	<1e-4	slr0590	GO:0016787	0.0719
sll0503	GO:0009132	<1e-4	slr0586	GO:0005996	<1e-4	ssr1155	GO:0043492	<1e-4	sll1882	GO:0044425	<1e-4	slr1570	GO:0016787	0.0719
ssl7042	GO:0060255	<1e-4	slr1071	GO:0071554	<1e-4	slr0667	GO:0015293	<1e-4	slr7101	GO:0043226	<1e-4	sll1373	GO:0016787	0.0719
slr7100	GO:0019752	<1e-4	slr0919	GO:0019219	<1e-4	slr1704	GO:0022832	<1e-4	slr1342	GO:0005575	<1e-4	slr1170	GO:0016787	0.0719
sll1247	GO:0051246	<1e-4	ssl5027	GO:0042180	<1e-4	slr2111	GO:0043167	<1e-4	slr0937	GO:0005575	<1e-4	slr1474	GO:0016787	0.0719
sll0863	GO:0048523	<1e-4	sll0658	GO:0043436	<1e-4	slr0937	GO:0022836	<1e-4	sll1186	GO:0044422	<1e-4	slr0325	GO:0016787	0.0719
slr1383	GO:0065007	<1e-4	sll0678	GO:0009132	<1e-4	sll0861	GO:0060089	<1e-4	ssr2553	GO:0043226	<1e-4	slr2103	GO:0016787	0.0719
sll0283	GO:0019637	<1e-4	sll5128	GO:0046164	<1e-4	slr1657	GO:0022891	<1e-4	slr0609	GO:0044422	<1e-4	sll1151	GO:0016787	0.0719
slr5012	GO:0009165	<1e-4	slr2121	GO:0051716	<1e-4	slr7011	GO:0015075	<1e-4	sll1934	GO:0044446	<1e-4	ssr7093	GO:0016787	0.0719
sll0564	GO:0034641	<1e-4	slr0613	GO:0051347	<1e-4	slr1789	GO:0008171	<1e-4	sll8011	GO:0044425	<1e-4	sll1464	GO:0016787	0.0719
slr0818	GO:0018130	<1e-4	sll0413	GO:0009117	<1e-4	slr2038	GO:0022838	<1e-4	sll1835	GO:0005575	<1e-4	ssl5103	GO:0016787	0.0719
sll0543	GO:0044282	<1e-4	ssr0336	GO:0019752	<1e-4	slr1668	GO:0015399	<1e-4	ssl1923	GO:0044422	<1e-4	ssr1155	GO:0016787	0.0719
slr1681	GO:0042180	<1e-4	slr1034	GO:0050789	<1e-4	sll0630	GO:0015267	<1e-4	sll1250	GO:0044424	<1e-4	ssl5113	GO:0016787	0.0719
sll1512	GO:0051818	<1e-4	slr5024	GO:0034641	<1e-4	slr1658	GO:0008171	<1e-4	slr0607	GO:0044464	<1e-4	sll1898	GO:0016787	0.0719
sll0381	GO:0009126	<1e-4	sll0595	GO:0009889	<1e-4	slr0195	GO:0022832	<1e-4	slr1425	GO:0005575	<1e-4	ssr1041	GO:0016787	0.0719
slr1407	GO:0009141	<1e-4	slr6021	GO:0072524	<1e-4	slr2004	GO:0022803	<1e-4	ssl1004	GO:0005575	<1e-4	sll1285	GO:0016787	0.0719
sll1651	GO:0072527	<1e-4	slr2048	GO:0009124	<1e-4	slr0751	GO:0022857	<1e-4	slr1919	GO:0044444	<1e-4	ssr1473	GO:0016787	0.0719

slr0813	GO:0050789	<1e-4	sll1675	GO:0006576	<1e-4	slr1431	GO:0015291	<1e-4	slr8044	GO:0044422	<1e-4	slr0575	GO:0016787	0.0719
sll0473	GO:0072527	<1e-4	sll0451	GO:0009150	<1e-4	ssl7051	GO:0015405	<1e-4	slr1648	GO:0044425	<1e-4	ssr5121	GO:0016787	0.0719
ssl3573	GO:0006220	<1e-4	slr0642	GO:0008150	<1e-4	sll1163	GO:0015267	<1e-4	slr1573	GO:0043226	<1e-4	sll0839	GO:0016787	0.0719
sll7062	GO:0006766	<1e-4	sll1915	GO:0043170	<1e-4	slr1865	GO:0005342	<1e-4	slr1505	GO:0044446	<1e-4	ssl1263	GO:0016787	0.0719
slr0211	GO:0044255	<1e-4	slr2111	GO:0009260	<1e-4	sll0103	GO:0022890	<1e-4	sll0586	GO:0044464	<1e-4	sll1188	GO:0016787	0.0719
sll0192	GO:0043648	<1e-4	ssl0738	GO:0072524	<1e-4	slr1179	GO:0003674	<1e-4	slr0272	GO:0043226	<1e-4	sll6055	GO:0016787	0.0719
ssl2595	GO:0009308	<1e-4	sll1400	GO:0055114	<1e-4	sll1726	GO:0015075	<1e-4	sll0497	GO:0044446	<1e-4	slr0967	GO:0016787	0.0719
slr1790	GO:0009262	<1e-4	slr1396	GO:0080090	<1e-4	slr0271	GO:0016741	<1e-4	ssl2920	GO:0044446	<1e-4	ssl0788	GO:0016787	0.0719
sll0596	GO:0010556	<1e-4	slr0423	GO:0046164	<1e-4	sll0595	GO:0016462	<1e-4	slr1600	GO:0044444	<1e-4	slr1935	GO:0016787	0.0719
ssr3410	GO:0050896	<1e-4	slr1915	GO:0019222	<1e-4	slr0810	GO:0022891	<1e-4	slr5115	GO:0043226	<1e-4	ssl7042	GO:0016787	0.0719
ssr6002	GO:0055114	<1e-4	slr0787	GO:0071496	<1e-4	slr0398	GO:0016817	<1e-4	slr0930	GO:0005575	<1e-4	slr1780	GO:0016787	0.0719
sll0160	GO:0009893	<1e-4	sll1433	GO:0050794	<1e-4	sll1898	GO:0016462	<1e-4	sll1054	GO:0005575	<1e-4	slr8022	GO:0016787	0.0719
sll1654	GO:0055086	<1e-4	sll1950	GO:0019222	<1e-4	slr0845	GO:0022836	<1e-4	sll0141	GO:0044444	<1e-4	slr1142	GO:0016787	0.0719
ssr3467	GO:0052188	<1e-4	ssr0536	GO:0001932	<1e-4	slr0769	GO:0008171	<1e-4	slr1230	GO:0005575	<1e-4	sll1319	GO:0016787	0.0719
sll0810	GO:0031640	<1e-4	slr0060	GO:0009161	<1e-4	ssl0461	GO:0022803	<1e-4	ssr1768	GO:0044444	<1e-4	ssr2318	GO:0016787	0.0719
sll0552	GO:0050896	<1e-4	sll1530	GO:0050789	<1e-4	sll8012	GO:0043167	<1e-4	slr0023	GO:0043226	<1e-4	slr1970	GO:0016787	0.0719
ssr2912	GO:0055114	<1e-4	slr1618	GO:0055086	<1e-4	slr0318	GO:0016741	<1e-4	ssr5011	GO:0044444	<1e-4	sll0847	GO:0016787	0.0719
sll0656	GO:0051716	<1e-4	ssl3692	GO:0009124	<1e-4	ssr2781	GO:0003674	<1e-4	slr2105	GO:0044424	<1e-4	ssr2802	GO:0016787	0.0719
slr0453	GO:0009144	<1e-4	ssr6089	GO:0010468	<1e-4	ssl0461	GO:0016741	<1e-4	sll1532	GO:0044422	<1e-4	slr0651	GO:0016787	0.0719
sll1049	GO:0009057	<1e-4	slr0482	GO:0009141	<1e-4	slr2027	GO:0043492	<1e-4	slr1944	GO:0044444	<1e-4	sll1785	GO:0016787	0.0719
sll1426	GO:0044281	<1e-4	slr0039	GO:0006091	<1e-4	sll1095	GO:0015075	<1e-4	sll8004	GO:0043226	<1e-4	slr1163	GO:0016787	0.0719
sll0297	GO:0001932	<1e-4	ssr0332	GO:0046164	<1e-4	ssl2148	GO:0022890	<1e-4	slr1690	GO:0044446	<1e-4	slr1173	GO:0016787	0.0719
sll1442	GO:0009394	<1e-4	sll0283	GO:0044282	<1e-4	slr0801	GO:0016746	<1e-4	sll0451	GO:0043226	<1e-4	slr1577	GO:0016787	0.0719
slr1895	GO:0009893	<1e-4	sll0192	GO:0009150	<1e-4	slr0364	GO:0016818	<1e-4	sll0737	GO:0005575	<1e-4	sll8011	GO:0016787	0.0719
sll6054	GO:0009144	<1e-4	sll1866	GO:0009199	<1e-4	sll0023	GO:0015399	<1e-4	sll0513	GO:0044425	<1e-4	ssl2162	GO:0016787	0.0719
ssr8013	GO:0060255	<1e-4	sll0761	GO:0044283	<1e-4	slr0168	GO:0015075	<1e-4	sll0167	GO:0044424	<1e-4	ssl5091	GO:0016787	0.0719
slr0592	GO:0042180	<1e-4	ssr2422	GO:0009132	<1e-4	slr7026	GO:0016817	<1e-4	sll0267	GO:0044464	<1e-4	slr0587	GO:0016787	0.0719
sll1652	GO:0044282	<1e-4	sll1318	GO:0034654	<1e-4	slr0980	GO:0043167	<1e-4	slr1290	GO:0044424	<1e-4	sll1573	GO:0016787	0.0719
slr1911	GO:0042455	<1e-4	ssr7017	GO:0010468	<1e-4	ssr2754	GO:0022836	<1e-4	sll0414	GO:0044425	<1e-4	sll0565	GO:0016787	0.0719
slr0398	GO:0019751	<1e-4	sll5006	GO:0009199	<1e-4	slr0356	GO:0022832	<1e-4	sll1511	GO:0005575	<1e-4	sll7028	GO:0016787	0.0719
slr0514	GO:0010556	<1e-4	slr1210	GO:0009262	<1e-4	sll1512	GO:0016818	<1e-4	sll1726	GO:0044425	<1e-4	slr0613	GO:0016787	0.0719
sll7047	GO:0043648	<1e-4	slr1875	GO:0034654	<1e-4	sll0359	GO:0022803	<1e-4	sll1053	GO:0044444	<1e-4	slr0813	GO:0016787	0.0719
slr1101	GO:0051716	<1e-4	sll1469	GO:0072524	<1e-4	ssr2975	GO:0043167	<1e-4	slr0818	GO:0044425	<1e-4	sll1476	GO:0016787	0.0719
slr6101	GO:0009260	<1e-4	sll0786	GO:0009165	<1e-4	ssr1768	GO:0015077	<1e-4	sll5006	GO:0044464	<1e-4	sll1131	GO:0016787	0.0719
slr0325	GO:0005996	<1e-4	ssl2069	GO:0009262	<1e-4	slr0023	GO:0016741	<1e-4	slr1429	GO:0044425	<1e-4	sll1495	GO:0016787	0.0719
slr0740	GO:0031326	<1e-4	slr1918	GO:0009161	<1e-4	ssl2069	GO:0015267	<1e-4	sll1433	GO:0005575	<1e-4	sll0249	GO:0016787	0.0719
sll1671	GO:0009132	<1e-4	slr6094	GO:0048518	<1e-4	sll1106	GO:0043492	<1e-4	sll1447	GO:0044424	<1e-4	slr5101	GO:0016787	0.0719
slr6075	GO:0055082	<1e-4	ssl2420	GO:0019752	<1e-4	sll0508	GO:0042623	<1e-4	slr1863	GO:0044425	<1e-4	slr1752	GO:0016787	0.0719
slr0885	GO:0006720	<1e-4	sll1414	GO:0044003	<1e-4	ssr0102	GO:0022832	<1e-4	slr1799	GO:0044446	<1e-4	sll7050	GO:0016787	0.0719
slr1045	GO:0034641	<1e-4	slr5101	GO:0050896	<1e-4	slr0453	GO:0022892	<1e-4	slr1840	GO:0044446	<1e-4	ssr2047	GO:0016787	0.0719
slr1614	GO:0019752	<1e-4	sll0359	GO:0009117	<1e-4	sll0645	GO:0022838	<1e-4	sll0732	GO:0043226	<1e-4	slr1236	GO:0016787	0.0719
sll1675	GO:0019222	<1e-4	ssl1046	GO:0006220	<1e-4	sll0023	GO:0022836	<1e-4	sll1726	GO:0043226	<1e-4	slr0709	GO:0016787	0.0719
slr7095	GO:0009132	<1e-4	ssl2749	GO:0046128	<1e-4	sll1830	GO:0043167	<1e-4	slr1704	GO:0043226	<1e-4	sll0479	GO:0016787	0.0719
slr0303	GO:0008150	<1e-4	sll1273	GO:0009126	<1e-4	slr0348	GO:0015075	<1e-4	slr6004	GO:0044424	<1e-4	ssl0410	GO:0016787	0.0719
slr1071	GO:0009262	<1e-4	slr0545	GO:0048522	<1e-4	slr0784	GO:0022892	<1e-4	sll1691	GO:0044444	<1e-4	sll7087	GO:0016787	0.0719
sll1715	GO:0009263	<1e-4	sll1072	GO:0009056	<1e-4	sll1219	GO:0015291	<1e-4	slr0872	GO:0043226	<1e-4	slr0689	GO:0016787	0.0719
slr1920	GO:0072524	<1e-4	sll0062	GO:0044283	<1e-4	sll1170	GO:0022836	<1e-4	slr0468	GO:0044446	<1e-4	ssl1378	GO:0016787	0.0719
sll0274	GO:0009059	<1e-4	slr1097	GO:0006631	<1e-4	slr0848	GO:0043167	<1e-4	sll1898	GO:0005575	<1e-4	slr0013	GO:0016787	0.0719
slr1790	GO:0044260	<1e-4	ssr2754	GO:0009199	<1e-4	slr0243	GO:0015405	<1e-4	sll0103	GO:0044425	<1e-4	slr0106	GO:0016787	0.0719
ssr2142	GO:0018130	<1e-4	ssl1792	GO:0052111	<1e-4	ssl1972	GO:0015405	<1e-4	slr0453	GO:0043226	<1e-4	ssr3402	GO:0016787	0.0719
slr6031	GO:0048522	<1e-4	ssl1417	GO:0055086	<1e-4	sll0661	GO:0043167	<1e-4	slr0459	GO:0044444	<1e-4	slr0453	GO:0016787	0.0719
slr1437	GO:0009056	<1e-4	ssl1263	GO:0005996	<1e-4	sll1348	GO:0016818	<1e-4	slr1767	GO:0044444	<1e-4	sll7089	GO:0016787	0.0719
ssr7035	GO:0019751	<1e-4	slr0667	GO:0006720	<1e-4	sll5089	GO:0022857	<1e-4	slr5112	GO:0005575	<1e-4	ssr1768	GO:0016787	0.0719
ssl2749	GO:0008150	<1e-4	slr0909	GO:0072524	<1e-4	sll1934	GO:0022891	<1e-4	slr2038	GO:0044422	<1e-4	slr2004	GO:0016787	0.0719

sll7050	GO:0071840	<1e-4	slr0362	GO:0044271	<1e-4	sll0661	GO:0015267	<1e-4	slr0654	GO:0044425	<1e-4	slr1449	GO:0016787	0.0719
slr0771	GO:0006811	<1e-4	slr1541	GO:0046164	<1e-4	slr1864	GO:0015075	<1e-4	slr1210	GO:0044422	<1e-4	slr0812	GO:0016787	0.0719
ssl0410	GO:0009150	<1e-4	slr0208	GO:0090304	<1e-4	slr1240	GO:0022857	<1e-4	ssl7048	GO:0044424	<1e-4	sll0608	GO:0016787	0.0719
slr1648	GO:0009150	<1e-4	slr0656	GO:0044260	<1e-4	slr1178	GO:0022891	<1e-4	sll0355	GO:0005575	<1e-4	slr2012	GO:0016787	0.0719
slr0689	GO:0009394	<1e-4	slr0553	GO:0006766	<1e-4	slr7023	GO:0022803	<1e-4	slr1557	GO:0044424	<1e-4	sll1321	GO:0016787	0.0719
slr0199	GO:0031323	<1e-4	ssr0761	GO:0046907	<1e-4	ssl2009	GO:0016462	<1e-4	sll1797	GO:0044446	<1e-4	slr1406	GO:0016787	0.0719
sll1714	GO:0022607	<1e-4	sll0294	GO:0009263	<1e-4	sll0487	GO:0022832	<1e-4	slr1259	GO:0044424	<1e-4	ssl5008	GO:0016787	0.0719
slr0885	GO:0043170	<1e-4	slr1339	GO:0009893	<1e-4	sll5062	GO:0022836	<1e-4	ssr3409	GO:0044446	<1e-4	slr7100	GO:0016787	0.0719
sll0659	GO:0009309	<1e-4	slr1084	GO:0006631	<1e-4	slr0581	GO:0016741	<1e-4	ssr1425	GO:0005575	<1e-4	slr0184	GO:0016787	0.0719
slr0959	GO:0009165	<1e-4	slr0937	GO:0009991	<1e-4	sll5044	GO:0043492	<1e-4	sll0241	GO:0044444	<1e-4	sll2007	GO:0009058	0.0711
slr0232	GO:0051246	<1e-4	slr1363	GO:0043933	<1e-4	ssr0109	GO:0022832	<1e-4	slr1415	GO:0044422	<1e-4	sll0661	GO:0009058	0.0711
slr7098	GO:0009987	<1e-4	ssr6027	GO:0006811	<1e-4	slr0263	GO:0022803	<1e-4	slr1178	GO:0043226	<1e-4	slr0810	GO:0009058	0.0711
sll1009	GO:0048519	<1e-4	sll1608	GO:0052111	<1e-4	slr1572	GO:0022891	<1e-4	ssr5117	GO:0044464	<1e-4	sll0898	GO:0009058	0.0695
sll7064	GO:0072522	<1e-4	sll1251	GO:0046128	<1e-4	ssr6078	GO:0015077	<1e-4	ssr2318	GO:0005575	<1e-4	slr1773	GO:0009058	0.0695
sll1630	GO:0033013	<1e-4	sll1979	GO:0051186	<1e-4	sll0659	GO:0022803	<1e-4	slr1102	GO:0005575	<1e-4	slr0108	GO:0009058	0.0695
slr0169	GO:0051234	<1e-4	ssl0787	GO:0050896	<1e-4	sll0577	GO:0016746	<1e-4	slr0111	GO:0044422	<1e-4	sll0815	GO:0003723	0.0672
sll0742	GO:0009165	<1e-4	ssl2814	GO:0046164	<1e-4	slr0771	GO:0015075	<1e-4	ssr1951	GO:0005575	<1e-4	sll0775	GO:0003723	0.0672
sll0282	GO:0046907	<1e-4	sll0350	GO:0009308	<1e-4	slr6072	GO:0015399	<1e-4	slr0013	GO:0044425	<1e-4	sll0863	GO:0003723	0.0672
slr1052	GO:0052111	<1e-4	slr1195	GO:0008150	<1e-4	sll0539	GO:0022803	<1e-4	ssr3129	GO:0044446	<1e-4	sll0369	GO:0003723	0.0672
slr0645	GO:0043933	<1e-4	ssr5074	GO:0009124	<1e-4	slr5013	GO:0016746	<1e-4	slr0863	GO:0044464	<1e-4	slr6015	GO:0003723	0.0672
sll1656	GO:0044271	<1e-4	slr1885	GO:0044248	<1e-4	sll0615	GO:0043167	<1e-4	sll1873	GO:0044425	<1e-4	ssr3341	GO:0003723	0.0672
ssr1552	GO:0071496	<1e-4	ssl2138	GO:0009262	<1e-4	sll0780	GO:0042623	<1e-4	sll0405	GO:0044464	<1e-4	slr1179	GO:0003723	0.0672
slr1800	GO:0044003	<1e-4	slr0398	GO:0051171	<1e-4	sll5046	GO:0043492	<1e-4	sll0499	GO:0005575	<1e-4	sll1638	GO:0003723	0.0672
sll0837	GO:0019752	<1e-4	sll1109	GO:0009150	<1e-4	slr0751	GO:0016817	<1e-4	ssl2069	GO:0005575	<1e-4	slr1657	GO:0003723	0.0672
slr6022	GO:0016054	<1e-4	slr0575	GO:0050789	<1e-4	slr0489	GO:0022803	<1e-4	sll1737	GO:0044422	<1e-4	sll0218	GO:0003723	0.0672
sll1225	GO:0042180	<1e-4	ssr6083	GO:0006721	<1e-4	sll1563	GO:0022891	<1e-4	slr1362	GO:0005575	<1e-4	slr0489	GO:0003723	0.0672
slr0291	GO:0009150	<1e-4	sll1174	GO:0031326	<1e-4	sll0553	GO:0003674	<1e-4	slr2110	GO:0044424	<1e-4	slr7023	GO:0003723	0.0672
sll0780	GO:0048518	<1e-4	sll1273	GO:0044271	<1e-4	slr1956	GO:0022892	<1e-4	sll1942	GO:0044425	<1e-4	sll1396	GO:0003723	0.0672
sll7031	GO:0009259	<1e-4	sll1634	GO:0044003	<1e-4	sll0172	GO:0008171	<1e-4	sll0596	GO:0005575	<1e-4	ssl2245	GO:0003723	0.0672
sll0872	GO:0044003	<1e-4	slr0065	GO:0019222	<1e-4	slr6073	GO:0015293	<1e-4	ssl0788	GO:0044424	<1e-4	slr6064	GO:0003723	0.0672
slr2070	GO:0008610	<1e-4	slr0151	GO:0051716	<1e-4	slr0816	GO:0015291	<1e-4	ssl1378	GO:0044424	<1e-4	sll1691	GO:0003723	0.0672
sll1119	GO:0009150	<1e-4	sll0023	GO:0006766	<1e-4	ssr6020	GO:0015075	<1e-4	ssr5117	GO:0005575	<1e-4	slr1363	GO:0003723	0.0672
slr2111	GO:0071841	<1e-4	sll0875	GO:0019752	<1e-4	slr0725	GO:0003674	<1e-4	ssr1407	GO:0044424	<1e-4	slr1753	GO:0003723	0.0672
slr7060	GO:0044248	<1e-4	sll0376	GO:0008610	<1e-4	slr1614	GO:0043167	<1e-4	sll0269	GO:0044422	<1e-4	sll1510	GO:0003723	0.0672
slr0818	GO:0009161	<1e-4	slr0326	GO:0048522	<1e-4	ssl2138	GO:0022891	<1e-4	slr1288	GO:0043226	<1e-4	sll0176	GO:0003723	0.0672
slr0553	GO:0050789	<1e-4	slr1648	GO:0006066	<1e-4	sll0447	GO:0016818	<1e-4	sll0585	GO:0044464	<1e-4	sll1252	GO:0003723	0.0672
slr1103	GO:0046907	<1e-4	slr1376	GO:0009259	<1e-4	slr1188	GO:0022803	<1e-4	ssr2551	GO:0043226	<1e-4	sll8033	GO:0003723	0.0672
slr1935	GO:0009309	<1e-4	sll1654	GO:0009987	<1e-4	slr0848	GO:0015291	<1e-4	sll1583	GO:0005575	<1e-4	slr6005	GO:0003723	0.0672
slr0650	GO:0006811	<1e-4	sll0585	GO:0042451	<1e-4	slr7091	GO:0022832	<1e-4	ssr8013	GO:0044422	<1e-4	sll1401	GO:0003723	0.0672
sll0188	GO:0071842	<1e-4	sll8004	GO:0055114	<1e-4	sll0543	GO:0043492	<1e-4	slr1391	GO:0044424	<1e-4	slr0058	GO:0003723	0.0672
sll0703	GO:0016054	<1e-4	slr1340	GO:0019637	<1e-4	slr0588	GO:0042623	<1e-4	ssl1004	GO:0044444	<1e-4	slr0552	GO:0003723	0.0672
slr1173	GO:0048878	<1e-4	slr1977	GO:0046907	<1e-4	slr0709	GO:0022838	<1e-4	sll5028	GO:0044425	<1e-4	slr0264	GO:0003723	0.0672
slr0294	GO:0006721	<1e-4	slr1612	GO:0006720	<1e-4	sll0069	GO:0022803	<1e-4	sll1582	GO:0043226	<1e-4	sll0611	GO:0003723	0.0672
sll1217	GO:0034641	<1e-4	ssr6085	GO:0051246	<1e-4	slr0148	GO:0043492	<1e-4	sll1265	GO:0044464	<1e-4	sll5046	GO:0003723	0.0672
slr0594	GO:0044106	<1e-4	slr1768	GO:0051188	<1e-4	slr1470	GO:0022803	<1e-4	slr1177	GO:0044446	<1e-4	slr6074	GO:0003723	0.0672
sll0803	GO:0051246	<1e-4	sll1162	GO:0044003	<1e-4	ssl1464	GO:0003674	<1e-4	sll1193	GO:0043226	<1e-4	sll0063	GO:0003723	0.0672
sll0630	GO:0046164	<1e-4	sll0192	GO:0043170	<1e-4	sll0875	GO:0003674	<1e-4	sll6055	GO:0044424	<1e-4	ssr5020	GO:0003723	0.0672
slr1195	GO:0019751	<1e-4	sll0588	GO:0051818	<1e-4	ssl3573	GO:0003674	<1e-4	ssr7035	GO:0044444	<1e-4	slr0388	GO:0003723	0.0672
sll1526	GO:0009259	<1e-4	slr1056	GO:0044281	<1e-4	slr1644	GO:0003674	<1e-4	slr0479	GO:0005575	<1e-4	sll0630	GO:0003723	0.0672
ssr6089	GO:0048519	<1e-4	sll0274	GO:0043549	<1e-4	slr1568	GO:0060089	<1e-4	slr6071	GO:0044422	<1e-4	ssr6032	GO:0003723	0.0672
sll0167	GO:0006220	<1e-4	slr0670	GO:0050794	<1e-4	slr2070	GO:0022892	<1e-4	slr1230	GO:0044446	<1e-4	sll1414	GO:0003723	0.0672
slr1209	GO:0044255	<1e-4	slr0731	GO:0016052	<1e-4	sll1763	GO:0015399	<1e-4	slr0262	GO:0044425	<1e-4	sll1389	GO:0003723	0.0672
sll1769	GO:0052188	<1e-4	slr8044	GO:0009889	<1e-4	sll0447	GO:0005342	<1e-4	slr0689	GO:0044424	<1e-4	sll0930	GO:0003723	0.0672
slr2005	GO:0051179	<1e-4	sll5063	GO:0044281	<1e-4	slr0416	GO:0022891	<1e-4	slr0890	GO:0044422	<1e-4	sll0994	GO:0003723	0.0672

slr1429	GO:0043412	<1e-4	ssl3451	GO:0043170	<1e-4	slr0358	GO:0015267	<1e-4	slr7024	GO:0044446	<1e-4	sll1109	GO:0003723	0.0672
slr0476	GO:0046164	<1e-4	slr0575	GO:0055082	<1e-4	slr0103	GO:0015291	<1e-4	sll0071	GO:0044464	<1e-4	slr7097	GO:0003723	0.0672
slr1082	GO:0009394	<1e-4	slr0852	GO:0051186	<1e-4	slr0913	GO:0043167	<1e-4	sll7006	GO:0044464	<1e-4	slr1576	GO:0003723	0.0672
sll0524	GO:0009126	<1e-4	sll1265	GO:0009057	<1e-4	sll0740	GO:0015399	<1e-4	ssl1577	GO:0044422	<1e-4	ssr6003	GO:0003723	0.0672
slr0590	GO:0042180	<1e-4	slr0602	GO:0046128	<1e-4	sll0172	GO:0022891	<1e-4	sll5004	GO:0044444	<1e-4	slr1187	GO:0003723	0.0672
sll1934	GO:0046907	<1e-4	sll7066	GO:0044248	<1e-4	sll1348	GO:0022803	<1e-4	slr5077	GO:0044444	<1e-4	slr5018	GO:0003723	0.0672
sll0609	GO:0018130	<1e-4	sll0162	GO:0071496	<1e-4	ssr1041	GO:0042623	<1e-4	sll8032	GO:0044424	<1e-4	sll0406	GO:0003723	0.0672
slr2144	GO:0006576	<1e-4	sll0743	GO:0008150	<1e-4	sll1531	GO:0015291	<1e-4	slr0668	GO:0044444	<1e-4	sll1773	GO:0003723	0.0672
sll0162	GO:0034660	<1e-4	sll0069	GO:0055114	<1e-4	slr6005	GO:0022838	<1e-4	sll0832	GO:0044444	<1e-4	sll1651	GO:0003723	0.0672
sll0710	GO:0055082	<1e-4	sll0487	GO:0006066	<1e-4	sll0563	GO:0042623	<1e-4	slr7071	GO:0043226	<1e-4	sll6054	GO:0003723	0.0672
slr0262	GO:0009309	<1e-4	slr0852	GO:0071842	<1e-4	slr0483	GO:0015293	<1e-4	slr1568	GO:0044424	<1e-4	slr1365	GO:0003723	0.0672
sll1455	GO:0009161	<1e-4	slr0730	GO:0034654	<1e-4	ssr2142	GO:0015291	<1e-4	slr0723	GO:0044422	<1e-4	slr0146	GO:0003723	0.0672
sll0737	GO:0043412	<1e-4	slr1677	GO:0006732	<1e-4	slr1624	GO:0015291	<1e-4	sll0412	GO:0044424	<1e-4	slr6049	GO:0003723	0.0672
sll1022	GO:0042455	<1e-4	ssl2420	GO:0006066	<1e-4	sll0861	GO:0016788	<1e-4	slr0613	GO:0044446	<1e-4	sll1273	GO:0003723	0.0672
slr1907	GO:0009165	<1e-4	sll8012	GO:0033013	<1e-4	slr1572	GO:0043167	<1e-4	ssl3573	GO:0044422	<1e-4	slr5119	GO:0003723	0.0672
ssr3122	GO:0006163	<1e-4	slr1173	GO:0016054	<1e-4	slr1513	GO:0016746	<1e-4	slr1699	GO:0044446	<1e-4	ssr1407	GO:0003723	0.0672
slr0269	GO:0043170	<1e-4	slr0318	GO:0006082	<1e-4	slr1338	GO:0022891	<1e-4	slr1571	GO:0043226	<1e-4	sll1757	GO:0003723	0.0672
sll1763	GO:0051234	<1e-4	sll1995	GO:0065007	<1e-4	slr0914	GO:0022892	<1e-4	slr0680	GO:0005575	<1e-4	slr0337	GO:0003723	0.0672
sll1531	GO:0065007	<1e-4	slr1098	GO:0019752	<1e-4	slr0505	GO:0015291	<1e-4	slr0937	GO:0044424	<1e-4	slr1270	GO:0003723	0.0672
sll1671	GO:0033013	<1e-4	sll0867	GO:0006220	<1e-4	slr7059	GO:0015293	<1e-4	slr5126	GO:0044464	<1e-4	slr1306	GO:0003723	0.0672
slr1407	GO:0042451	<1e-4	sll2011	GO:0031640	<1e-4	sll7090	GO:0022891	<1e-4	sll1512	GO:0005575	<1e-4	slr0196	GO:0003723	0.0672
sll1939	GO:0009124	<1e-4	ssr8013	GO:0071841	<1e-4	slr7098	GO:0015267	<1e-4	slr1880	GO:0005575	<1e-4	slr7012	GO:0003723	0.0672
sll0847	GO:0016053	<1e-4	ssl7048	GO:0022607	<1e-4	slr0489	GO:0016817	<1e-4	sll1201	GO:0044425	<1e-4	sll1797	GO:0003723	0.0672
slr1913	GO:0043648	<1e-4	ssr7072	GO:0031323	<1e-4	sll1956	GO:0015293	<1e-4	slr0871	GO:0005575	<1e-4	sll0423	GO:0003723	0.0672
slr0730	GO:0051234	<1e-4	sll8035	GO:0022607	<1e-4	sll1736	GO:0022836	<1e-4	slr1177	GO:0005575	<1e-4	sll1509	GO:0003723	0.0672
slr1676	GO:0042180	<1e-4	sll1095	GO:0048878	<1e-4	sll7062	GO:0003674	<1e-4	sll0141	GO:0005575	<1e-4	ssl2781	GO:0003723	0.0672
ssl6061	GO:0072528	<1e-4	slr1885	GO:0042180	<1e-4	sll0925	GO:0015405	<1e-4	sll0008	GO:0005575	<1e-4	sll0481	GO:0003723	0.0672
ssl2471	GO:0065007	<1e-4	sll0296	GO:0009394	<1e-4	slr2005	GO:0022838	<1e-4	slr2003	GO:0005575	<1e-4	sll1123	GO:0003723	0.0672
ssr7093	GO:0009141	<1e-4	slr1940	GO:0009259	<1e-4	ssl1520	GO:0043492	<1e-4	slr0108	GO:0043226	<1e-4	sll1485	GO:0003723	0.0672
slr2073	GO:0043436	<1e-4	slr1338	GO:0019222	<1e-4	sll0802	GO:0016746	<1e-4	sll1289	GO:0044446	<1e-4	slr0870	GO:0003723	0.0672
ssr2787	GO:0044260	<1e-4	ssr0761	GO:0018130	<1e-4	sml0011	GO:0015267	<1e-4	sll7070	GO:0044444	<1e-4	slr0964	GO:0003723	0.0672
sll0360	GO:0071842	<1e-4	slr0263	GO:0031326	<1e-4	slr1875	GO:0015293	<1e-4	sll1188	GO:0044464	<1e-4	slr1920	GO:0003723	0.0672
ssl1792	GO:0044003	<1e-4	sll1735	GO:0009991	<1e-4	slr5037	GO:0043492	<1e-4	slr0392	GO:0005575	<1e-4	sll1696	GO:0003723	0.0672
ssl3451	GO:0019222	<1e-4	slr1196	GO:0034660	<1e-4	slr0065	GO:0016462	<1e-4	slr0416	GO:0005575	<1e-4	sll0008	GO:0003723	0.0672
slr1519	GO:0048519	<1e-4	sll1570	GO:0006066	<1e-4	ssr1499	GO:0022838	<1e-4	ssr3300	GO:0044422	<1e-4	sll0048	GO:0003723	0.0672
slr0243	GO:0044260	<1e-4	slr5018	GO:0072527	<1e-4	sll0044	GO:0015399	<1e-4	slr1419	GO:0044446	<1e-4	sll0886	GO:0003723	0.0672
slr1170	GO:0008610	<1e-4	sll0857	GO:0051186	<1e-4	sll7064	GO:0015267	<1e-4	sll1675	GO:0044422	<1e-4	sll7047	GO:0003723	0.0672
slr1342	GO:0009259	<1e-4	slr0801	GO:0016052	<1e-4	slr2121	GO:0015293	<1e-4	slr1339	GO:0044444	<1e-4	sll0284	GO:0003723	0.0672
sll0269	GO:0044275	<1e-4	sll8035	GO:0009308	<1e-4	slr0725	GO:0022891	<1e-4	sll1477	GO:0044446	<1e-4	sll1004	GO:0003723	0.0672
sll1950	GO:0009308	<1e-4	sll1092	GO:0022411	<1e-4	sll1388	GO:0060089	<1e-4	slr7092	GO:0005575	<1e-4	sll0293	GO:0003723	0.0672
sll0980	GO:0006720	<1e-4	sll0645	GO:0042451	<1e-4	sll1862	GO:0016462	<1e-4	slr2032	GO:0044446	<1e-4	slr1188	GO:0003723	0.0672
sll1106	GO:0006220	<1e-4	sll1162	GO:0034641	<1e-4	slr1174	GO:0022857	<1e-4	sll0932	GO:0044424	<1e-4	slr0601	GO:0003723	0.0672
sll0327	GO:0019637	<1e-4	slr6063	GO:0052111	<1e-4	sll1606	GO:0003674	<1e-4	ssl7074	GO:0044464	<1e-4	sll1022	GO:0003723	0.0672
slr1223	GO:0006811	<1e-4	sll1109	GO:0044255	<1e-4	ssl0739	GO:0003674	<1e-4	sll0659	GO:0044464	<1e-4	ssr3189	GO:0003723	0.0672
ssr6079	GO:0046164	<1e-4	sll1738	GO:0006091	<1e-4	slr0341	GO:0016817	<1e-4	slr1365	GO:0044464	<1e-4	sll1606	GO:0003723	0.0672
sll1401	GO:0019222	<1e-4	sll0910	GO:0006766	<1e-4	sll1954	GO:0016746	<1e-4	sll0793	GO:0044422	<1e-4	ssr1766	GO:0003723	0.0672
sll1764	GO:0071841	<1e-4	sll1632	GO:0080090	<1e-4	sll1390	GO:0022832	<1e-4	slr7100	GO:0044422	<1e-4	sll1921	GO:0003723	0.0672
slr1327	GO:0006733	<1e-4	sll0156	GO:0065007	<1e-4	ssl3142	GO:0022803	<1e-4	sll1570	GO:0044464	<1e-4	slr7016	GO:0003723	0.0672
ssl8039	GO:0009260	<1e-4	slr1557	GO:0009893	<1e-4	slr6067	GO:0005342	<1e-4	sll5004	GO:0043226	<1e-4	slr6006	GO:0003723	0.0672
sll1123	GO:0009124	<1e-4	slr0358	GO:0043648	<1e-4	ssr6089	GO:0015293	<1e-4	sll0787	GO:0044424	<1e-4	ssl0461	GO:0003723	0.0672
slr5013	GO:0009124	<1e-4	sll1109	GO:0009259	<1e-4	sll0827	GO:0022857	<1e-4	sll0282	GO:0044464	<1e-4	ssl2717	GO:0003723	0.0672
sll1773	GO:0006720	<1e-4	slr1603	GO:0043549	<1e-4	slr2052	GO:0015293	<1e-4	sll1512	GO:0044444	<1e-4	slr0971	GO:0003723	0.0672
slr0479	GO:0009161	<1e-4	slr1964	GO:0071840	<1e-4	sll1054	GO:0015293	<1e-4	sll5026	GO:0043226	<1e-4	slr1535	GO:0003723	0.0672
slr1875	GO:0052188	<1e-4	slr1069	GO:0050801	<1e-4	sll1348	GO:0022892	<1e-4	slr1273	GO:0044424	<1e-4	slr6080	GO:0003723	0.0672

sll1552	GO:0065007	<1e-4	sll0449	GO:0043436	<1e-4	slr0588	GO:0016462	<1e-4	sll7090	GO:0043226	<1e-4	slr1927	GO:0003723	0.0672
slr0888	GO:0019637	<1e-4	sll1698	GO:0016052	<1e-4	slr0699	GO:0016462	<1e-4	sll0252	GO:0044424	<1e-4	sll1217	GO:0003723	0.0672
sll0532	GO:0034654	<1e-4	slr0169	GO:0009199	<1e-4	slr1273	GO:0022838	<1e-4	slr0199	GO:0044446	<1e-4	slr0059	GO:0003723	0.0672
sll1173	GO:0019637	<1e-4	slr0664	GO:0009308	<1e-4	sll1562	GO:0022890	<1e-4	slr0689	GO:0044446	<1e-4	sll0068	GO:0017111	0.0665
slr6014	GO:0001932	<1e-4	sll0319	GO:0009124	<1e-4	slr1571	GO:0008171	<1e-4	slr1534	GO:0043226	<1e-4	slr1215	GO:0017111	0.0665
sll1960	GO:0051818	<1e-4	slr1788	GO:0065007	<1e-4	slr1568	GO:0022832	<1e-4	sll1009	GO:0044444	<1e-4	sll0023	GO:0003723	0.0657
slr2118	GO:0010468	<1e-4	slr7057	GO:0019751	<1e-4	slr0816	GO:0022890	<1e-4	sll0441	GO:0044446	<1e-4	sll0198	GO:0003723	0.0657
sll1511	GO:0016052	<1e-4	slr2119	GO:0044260	<1e-4	slr0313	GO:0015075	<1e-4	slr6068	GO:0044425	<1e-4	sll0982	GO:0003723	0.0657
slr0169	GO:0044248	<1e-4	sll1400	GO:0019222	<1e-4	slr1259	GO:0022892	<1e-4	slr0816	GO:0044422	<1e-4	sll0780	GO:0003723	0.0657
sll0237	GO:0051171	<1e-4	slr0217	GO:0018130	<1e-4	sll1485	GO:0003674	<1e-4	ssl0352	GO:0044425	<1e-4	sll1006	GO:0003723	0.0657
ssl1378	GO:0072527	<1e-4	sll0238	GO:0051246	<1e-4	sll0737	GO:0016818	<1e-4	slr1866	GO:0005575	<1e-4	sll0066	GO:0003723	0.0657
sll1654	GO:0051347	<1e-4	sll1036	GO:0052111	<1e-4	slr1875	GO:0022803	<1e-4	sll0072	GO:0005575	<1e-4	slr2011	GO:0003723	0.0657
slr1493	GO:0071496	<1e-4	ssl8039	GO:0009259	<1e-4	sll1898	GO:0016817	<1e-4	sll1006	GO:0044464	<1e-4	slr1676	GO:0003723	0.0657
ssl0109	GO:0010556	<1e-4	sll1737	GO:0043436	<1e-4	slr7097	GO:0015405	<1e-4	slr1813	GO:0044422	<1e-4	slr2121	GO:0003723	0.0657
slr1658	GO:0071496	<1e-4	sll1289	GO:0050789	<1e-4	sll1203	GO:0016746	<1e-4	slr1378	GO:0005575	<1e-4	slr1203	GO:0003723	0.0657
slr1576	GO:0006720	<1e-4	slr1875	GO:0051171	<1e-4	sll1659	GO:0015267	<1e-4	slr0148	GO:0044425	<1e-4	slr0941	GO:0003723	0.0657
sll1717	GO:0043436	<1e-4	sll1965	GO:0043549	<1e-4	slr0978	GO:0016462	<1e-4	sll1858	GO:0005575	<1e-4	sll1736	GO:0003723	0.0657
sll1219	GO:0009394	<1e-4	ssl8003	GO:0051171	<1e-4	slr0664	GO:0022892	<1e-4	slr1052	GO:0005575	<1e-4	slr1618	GO:0003723	0.0657
slr1142	GO:0006082	<1e-4	sll1021	GO:0006163	<1e-4	slr7012	GO:0043492	<1e-4	ssr2317	GO:0043226	<1e-4	slr1913	GO:0003723	0.0657
ssl1464	GO:0051179	<1e-4	sll0327	GO:0051818	<1e-4	slr0592	GO:0016462	<1e-4	ssr6024	GO:0044422	<1e-4	sll1002	GO:0003723	0.0657
slr7023	GO:0009142	<1e-4	slr0204	GO:0048518	<1e-4	slr2048	GO:0016818	<1e-4	sll0069	GO:0044424	<1e-4	sll1835	GO:0003723	0.0657
slr8014	GO:0051246	<1e-4	slr1923	GO:0051186	<1e-4	slr1194	GO:0015291	<1e-4	ssl2065	GO:0005575	<1e-4	slr1210	GO:0003723	0.0657
slr1900	GO:0009394	<1e-4	sll1954	GO:0009893	<1e-4	ssl0353	GO:0022838	<1e-4	sll0676	GO:0044424	<1e-4	slr1593	GO:0003723	0.0657
slr1816	GO:0006720	<1e-4	slr0509	GO:0051188	<1e-4	slr0709	GO:0042623	<1e-4	slr0971	GO:0043226	<1e-4	slr1571	GO:0003723	0.0657
ssr5106	GO:0008610	<1e-4	sll0444	GO:0050801	<1e-4	slr1342	GO:0022892	<1e-4	ssl7051	GO:0005575	<1e-4	ssr2551	GO:0003723	0.0657
slr5087	GO:0031326	<1e-4	slr2032	GO:0009165	<1e-4	slr2032	GO:0005342	<1e-4	slr7060	GO:0044425	<1e-4	slr0103	GO:0003723	0.0657
slr7057	GO:0009141	<1e-4	ssl5031	GO:0052111	<1e-4	sll1267	GO:0016818	<1e-4	sll0168	GO:0044446	<1e-4	slr1573	GO:0003723	0.0657
slr0655	GO:0051234	<1e-4	sll0803	GO:0043933	<1e-4	slr2000	GO:0043167	<1e-4	ssr1765	GO:0044425	<1e-4	slr0294	GO:0003723	0.0657
sll0405	GO:0051234	<1e-4	ssl2749	GO:0045184	<1e-4	sll1068	GO:0022890	<1e-4	slr5118	GO:0005575	<1e-4	sll1954	GO:0003723	0.0657
ssl1300	GO:0043436	<1e-4	sll0602	GO:0072524	<1e-4	sll1912	GO:0022891	<1e-4	slr5118	GO:0044464	<1e-4	sll0314	GO:0003723	0.0657
ssl1004	GO:0005996	<1e-4	slr1081	GO:0009991	<1e-4	sll0811	GO:0016741	<1e-4	ssr6086	GO:0044424	<1e-4	sll1830	GO:0003723	0.0657
slr1944	GO:0031323	<1e-4	sll1381	GO:0051246	<1e-4	slr1471	GO:0022803	<1e-4	sll0236	GO:0005575	<1e-4	slr0921	GO:0003723	0.0657
sll1289	GO:0006082	<1e-4	slr0654	GO:0006066	<1e-4	ssr2067	GO:0016818	<1e-4	slr0195	GO:0044446	<1e-4	slr1275	GO:0003723	0.0657
sll8032	GO:0048518	<1e-4	sll1201	GO:0055082	<1e-4	ssl2420	GO:0022890	<1e-4	sll1004	GO:0044444	<1e-4	ssr2755	GO:0003723	0.0657
sll1390	GO:0051246	<1e-4	sll0381	GO:0051347	<1e-4	ssr2998	GO:0016462	<1e-4	slr5018	GO:0044422	<1e-4	sll1262	GO:0003723	0.0657
sll1250	GO:0043648	<1e-4	slr7059	GO:0009056	<1e-4	slr1599	GO:0016746	<1e-4	ssl1972	GO:0044444	<1e-4	sll0010	GO:0003723	0.0657
sll0994	GO:0051171	<1e-4	sll0230	GO:0019751	<1e-4	slr1266	GO:0015267	<1e-4	slr0872	GO:0044425	<1e-4	slr1266	GO:0003723	0.0657
slr0780	GO:0050794	<1e-4	sll0691	GO:0048523	<1e-4	ssl8039	GO:0022892	<1e-4	sll0760	GO:0044464	<1e-4	slr0192	GO:0003723	0.0657
slr1624	GO:0006082	<1e-4	slr0146	GO:0009165	<1e-4	ssr6032	GO:0005342	<1e-4	slr1173	GO:0043226	<1e-4	sll1530	GO:0003723	0.0657
slr0374	GO:0065007	<1e-4	slr0980	GO:0046483	<1e-4	sll7078	GO:0022838	<1e-4	ssl5031	GO:0005575	<1e-4	slr1230	GO:0003723	0.0657
slr1413	GO:0009059	<1e-4	slr1657	GO:0033013	<1e-4	slr0587	GO:0043492	<1e-4	slr1815	GO:0005575	<1e-4	sll0669	GO:0004871	0.0644
sll1071	GO:0016054	<1e-4	slr2004	GO:0044248	<1e-4	slr1895	GO:0022838	<1e-4	sll0658	GO:0044425	<1e-4	sll0810	GO:0004871	0.0644
slr6029	GO:0009987	<1e-4	sll0793	GO:0009132	<1e-4	slr6075	GO:0008171	<1e-4	slr1827	GO:0043226	<1e-4	slr0053	GO:0004871	0.0644
slr1926	GO:0010468	<1e-4	slr0157	GO:0006766	<1e-4	sll0062	GO:0008171	<1e-4	slr6031	GO:0005575	<1e-4	slr0975	GO:0004871	0.0644
sll8007	GO:0009117	<1e-4	sll1979	GO:0043412	<1e-4	slr0208	GO:0003674	<1e-4	sll5047	GO:0044425	<1e-4	sll1654	GO:0004871	0.0644
slr1397	GO:0051186	<1e-4	slr1670	GO:0042451	<1e-4	sll0864	GO:0015405	<1e-4	slr0294	GO:0005575	<1e-4	sll1240	GO:0004871	0.0644
sll1289	GO:0016054	<1e-4	slr6022	GO:0006732	<1e-4	sll5006	GO:0042623	<1e-4	slr0519	GO:0044425	<1e-4	sll0740	GO:0004871	0.0644
sml0011	GO:0071841	<1e-4	sll0875	GO:0006066	<1e-4	slr1875	GO:0022838	<1e-4	slr0588	GO:0044464	<1e-4	ssr2754	GO:0004871	0.0644
sll0933	GO:0009132	<1e-4	slr1767	GO:0019751	<1e-4	sll1510	GO:0015291	<1e-4	ssr5074	GO:0005575	<1e-4	sll1350	GO:0004871	0.0644
sll1913	GO:0034660	<1e-4	slr0519	GO:0055114	<1e-4	sll1583	GO:0015405	<1e-4	slr1674	GO:0044464	<1e-4	slr0801	GO:0004871	0.0644
sll6053	GO:0009309	<1e-4	slr1773	GO:0048878	<1e-4	slr1900	GO:0022857	<1e-4	sll0854	GO:0044444	<1e-4	slr1590	GO:0004871	0.0644
sll0854	GO:0072528	<1e-4	ssr6083	GO:0008150	<1e-4	ssl2996	GO:0022890	<1e-4	sll1061	GO:0044422	<1e-4	ssl3382	GO:0004871	0.0644
sll0280	GO:0080090	<1e-4	slr0592	GO:0009132	<1e-4	slr1944	GO:0015291	<1e-4	sll0545	GO:0005575	<1e-4	sll0909	GO:0004871	0.0644
sll0183	GO:0009394	<1e-4	sll1315	GO:0090304	<1e-4	sll1531	GO:0005342	<1e-4	slr0358	GO:0044424	<1e-4	slr1895	GO:0004871	0.0644

slr1301	GO:0006163	<1e-4	sll1853	GO:0009263	<1e-4	slr2003	GO:0042623	<1e-4	slr0380	GO:0044464	<1e-4	slr1303	GO:0004871	0.0644
sll1218	GO:0051186	<1e-4	ssr5120	GO:0048518	<1e-4	slr0914	GO:0008171	<1e-4	sll0933	GO:0044446	<1e-4	slr2048	GO:0004871	0.0644
slr1624	GO:0009987	<1e-4	slr1383	GO:0009263	<1e-4	ssl0312	GO:0016462	<1e-4	ssl8005	GO:0005575	<1e-4	sll0981	GO:0004871	0.0644
sll0242	GO:0009124	<1e-4	slr0625	GO:0005996	<1e-4	slr1103	GO:0015075	<1e-4	sll0756	GO:0044464	<1e-4	slr1827	GO:0004871	0.0644
slr1107	GO:0065007	<1e-4	sll0448	GO:0006811	<1e-4	sll1232	GO:0015291	<1e-4	sll7078	GO:0044422	<1e-4	sll0412	GO:0004871	0.0644
sll0911	GO:0051347	<1e-4	slr1240	GO:0009161	<1e-4	sll0553	GO:0060089	<1e-4	sll1752	GO:0044444	<1e-4	sll0141	GO:0004871	0.0644
sll0871	GO:0071840	<1e-4	sll0751	GO:0055082	<1e-4	slr1681	GO:0042623	<1e-4	slr1570	GO:0044422	<1e-4	slr1082	GO:0004871	0.0644
slr1599	GO:0009144	<1e-4	sll1240	GO:0006811	<1e-4	slr0082	GO:0022836	<1e-4	slr6021	GO:0043226	<1e-4	sll1873	GO:0004871	0.0644
sll0263	GO:0006066	<1e-4	slr1906	GO:0019637	<1e-4	slr7060	GO:0022857	<1e-4	slr0476	GO:0044464	<1e-4	sll1372	GO:0004871	0.0644
slr0232	GO:0009893	<1e-4	sll1009	GO:0006720	<1e-4	sll1632	GO:0016817	<1e-4	slr1907	GO:0044424	<1e-4	slr0455	GO:0004871	0.0644
slr0453	GO:0009123	<1e-4	ssl7038	GO:0080090	<1e-4	sll1570	GO:0022836	<1e-4	sll0827	GO:0044444	<1e-4	sll1634	GO:0004871	0.0644
slr1624	GO:0046907	<1e-4	sll0487	GO:0055114	<1e-4	sll0172	GO:0022803	<1e-4	slr1702	GO:0044444	<1e-4	slr1338	GO:0004871	0.0644
sll0410	GO:0051818	<1e-4	sll0939	GO:0018130	<1e-4	slr0730	GO:0022803	<1e-4	sll0930	GO:0043226	<1e-4	sll0837	GO:0004871	0.0644
ssr1256	GO:0052111	<1e-4	slr6090	GO:0009263	<1e-4	sll1040	GO:0015075	<1e-4	slr1338	GO:0044425	<1e-4	sll0843	GO:0004871	0.0644
slr0345	GO:0051818	<1e-4	ssl8028	GO:0048878	<1e-4	slr1980	GO:0022803	<1e-4	sll5004	GO:0044425	<1e-4	slr0554	GO:0004871	0.0644
sll1350	GO:0051716	<1e-4	slr2084	GO:0046128	<1e-4	slr1956	GO:0043167	<1e-4	slr0366	GO:0005575	<1e-4	sll1071	GO:0004871	0.0644
slr7096	GO:0042451	<1e-4	slr7094	GO:0065007	<1e-4	slr0924	GO:0016462	<1e-4	sll1239	GO:0044422	<1e-4	sll1173	GO:0004871	0.0644
slr0103	GO:0065007	<1e-4	slr0179	GO:0031640	<1e-4	sll0174	GO:0015405	<1e-4	slr1470	GO:0044444	<1e-4	slr1668	GO:0004871	0.0644
sll1528	GO:0044283	<1e-4	slr1415	GO:0006733	<1e-4	slr0601	GO:0060089	<1e-4	sll0565	GO:0043226	<1e-4	sll1352	GO:0004871	0.0644
slr0907	GO:0008150	<1e-4	ssl7048	GO:0044275	<1e-4	slr2071	GO:0015075	<1e-4	sll6052	GO:0005575	<1e-4	sll0614	GO:0004871	0.0644
sll0085	GO:0019751	<1e-4	sll0933	GO:0051186	<1e-4	slr0870	GO:0022892	<1e-4	sll7062	GO:0044444	<1e-4	slr1122	GO:0004871	0.0644
sll1318	GO:0006720	<1e-4	sll1193	GO:0044260	<1e-4	sll1486	GO:0022890	<1e-4	slr0971	GO:0044425	<1e-4	slr1383	GO:0004871	0.0644
slr0668	GO:0006576	<1e-4	sll0565	GO:0034654	<1e-4	slr0594	GO:0042623	<1e-4	sll5067	GO:0044422	<1e-4	sll0888	GO:0004871	0.0644
ssr2787	GO:0006811	<1e-4	slr0109	GO:0006082	<1e-4	sll8011	GO:0043492	<1e-4	sll0419	GO:0005575	<1e-4	sll0189	GO:0004871	0.0644
slr1493	GO:0042180	<1e-4	sll0160	GO:0006721	<1e-4	sll1681	GO:0022892	<1e-4	sll7090	GO:0044446	<1e-4	sll1541	GO:0004871	0.0644
slr1677	GO:0009144	<1e-4	slr0771	GO:0009987	<1e-4	ssr2551	GO:0016746	<1e-4	sll1552	GO:0044425	<1e-4	slr1762	GO:0004871	0.0644
slr1196	GO:0009144	<1e-4	slr1571	GO:0044281	<1e-4	slr0914	GO:0015075	<1e-4	sll0101	GO:0005575	<1e-4	slr2125	GO:0004871	0.0644
ssl1046	GO:0042451	<1e-4	slr1534	GO:0009132	<1e-4	slr2038	GO:0016818	<1e-4	ssr3129	GO:0043226	<1e-4	slr0654	GO:0004871	0.0644
sll1863	GO:0009889	<1e-4	slr0517	GO:0046164	<1e-4	slr0810	GO:0016741	<1e-4	sll1965	GO:0044424	<1e-4	slr0935	GO:0004871	0.0644
sll0243	GO:0044283	<1e-4	sll0405	GO:0046394	<1e-4	slr0852	GO:0003674	<1e-4	sll1882	GO:0044464	<1e-4	slr0962	GO:0004871	0.0644
sll0545	GO:0009263	<1e-4	slr1415	GO:0031640	<1e-4	slr0013	GO:0008171	<1e-4	sll0588	GO:0005575	<1e-4	slr0468	GO:0004871	0.0644
ssl2807	GO:0051818	<1e-4	slr1032	GO:0006732	<1e-4	slr0655	GO:0022838	<1e-4	sll1902	GO:0044424	<1e-4	sll1399	GO:0004871	0.0644
sll1797	GO:0048518	<1e-4	slr2119	GO:0006766	<1e-4	slr5087	GO:0015291	<1e-4	sll1552	GO:0005575	<1e-4	sll0473	GO:0004871	0.0634
sll1726	GO:0043436	<1e-4	ssl0410	GO:0034660	<1e-4	ssr3300	GO:0022832	<1e-4	slr0269	GO:0044464	<1e-4	sll0553	GO:0004871	0.0634
ssr2781	GO:0019752	<1e-4	ssr5074	GO:0009059	<1e-4	ssr7036	GO:0022836	<1e-4	slr1886	GO:0044446	<1e-4	slr0960	GO:0004871	0.0634
sll0553	GO:0009893	<1e-4	slr0863	GO:0055114	<1e-4	slr1339	GO:0015077	<1e-4	sll0280	GO:0044424	<1e-4	sll2013	GO:0004871	0.0634
sll1542	GO:0050801	<1e-4	slr1774	GO:0046128	<1e-4	sll0608	GO:0022891	<1e-4	ssr5121	GO:0044444	<1e-4	sll1203	GO:0004871	0.0634
sll0888	GO:0006733	<1e-4	slr1484	GO:0065007	<1e-4	sll1132	GO:0042623	<1e-4	slr0865	GO:0044444	<1e-4	sll1921	GO:0004871	0.0634
slr1183	GO:0071841	<1e-4	slr0975	GO:0006733	<1e-4	sll0985	GO:0016741	<1e-4	sll2013	GO:0044424	<1e-4	sll1853	GO:0004871	0.0634
slr0872	GO:0009262	<1e-4	slr1628	GO:0044248	<1e-4	sll0405	GO:0016746	<1e-4	slr0941	GO:0044424	<1e-4	slr7092	GO:0004871	0.0634
sll0218	GO:0009144	<1e-4	slr0554	GO:0048878	<1e-4	slr1270	GO:0005342	<1e-4	sll0736	GO:0005575	<1e-4	ssl5129	GO:0004871	0.0634
slr1647	GO:0009124	<1e-4	sll0314	GO:0045184	<1e-4	slr1571	GO:0016746	<1e-4	slr0076	GO:0044422	<1e-4	sll0241	GO:0004871	0.0634
ssl5098	GO:0006163	<1e-4	slr0751	GO:0052188	<1e-4	slr0476	GO:0016746	<1e-4	slr7100	GO:0005575	<1e-4	ssl2781	GO:0004871	0.0634
slr2018	GO:0072527	<1e-4	slr1174	GO:0048518	<1e-4	sll1399	GO:0015077	<1e-4	sll0208	GO:0044425	<1e-4	slr1907	GO:0004871	0.0634
slr1114	GO:0006721	<1e-4	sll0069	GO:0051234	<1e-4	sll0743	GO:0015405	<1e-4	slr1338	GO:0044444	<1e-4	sll8002	GO:0004871	0.0634
slr1690	GO:0010468	<1e-4	slr1383	GO:0046907	<1e-4	slr1668	GO:0022838	<1e-4	ssr7035	GO:0005575	<1e-4	slr1813	GO:0004871	0.0634
sll1109	GO:0044275	<1e-4	sll5028	GO:0071842	<1e-4	slr1932	GO:0005342	<1e-4	slr1767	GO:0005575	<1e-4	sll0609	GO:0004871	0.0634
sll1086	GO:0008150	<1e-4	sll5067	GO:0009144	<1e-4	sll0933	GO:0022857	<1e-4	ssl7046	GO:0044424	<1e-4	sll0710	GO:0004871	0.0634
sll1531	GO:0043648	<1e-4	slr0243	GO:0016054	<1e-4	slr6073	GO:0022891	<1e-4	slr5037	GO:0005575	<1e-4	slr0440	GO:0004871	0.0634
sll1106	GO:0009308	<1e-4	slr6021	GO:0019362	<1e-4	slr7015	GO:0005342	<1e-4	slr7081	GO:0044422	<1e-4	slr7082	GO:0004871	0.0634
sll8035	GO:0016054	<1e-4	ssl3382	GO:0006732	<1e-4	sll1495	GO:0016818	<1e-4	slr1419	GO:0005575	<1e-4	slr7025	GO:0004871	0.0634
slr7011	GO:0055086	<1e-4	slr6100	GO:0006753	<1e-4	slr1530	GO:0022892	<1e-4	sll0685	GO:0005575	<1e-4	ssr5117	GO:0004871	0.0634
slr6090	GO:0051234	<1e-4	sll0756	GO:0006811	<1e-4	sll0266	GO:0015291	<1e-4	sll0525	GO:0044425	<1e-4	sll5034	GO:0004871	0.0634
sll1714	GO:0071842	<1e-4	slr0380	GO:0009260	<1e-4	slr0594	GO:0022891	<1e-4	sll1500	GO:0005575	<1e-4	slr1657	GO:0004871	0.0634

slr6104	GO:0044260	<1e-4	slr1103	GO:0044255	<1e-4	slr0871	GO:0042623	<1e-4	slr1840	GO:0043226	<1e-4	slr0552	GO:0004871	0.0634
slr6051	GO:0044003	<1e-4	sll0647	GO:0043412	<1e-4	sll0372	GO:0016462	<1e-4	sll1350	GO:0005575	<1e-4	slr0146	GO:0004871	0.0634
ssr2317	GO:0071554	<1e-4	slr1045	GO:0009308	<1e-4	slr2060	GO:0015291	<1e-4	slr0725	GO:0044425	<1e-4	sll0811	GO:0004871	0.0634
sll1461	GO:0052188	<1e-4	slr1591	GO:0009893	<1e-4	ssr1951	GO:0016818	<1e-4	slr1852	GO:0005575	<1e-4	slr1677	GO:0004871	0.0634
slr0699	GO:0006721	<1e-4	slr0217	GO:0006721	<1e-4	ssr2201	GO:0022890	<1e-4	slr1303	GO:0043226	<1e-4	sll8007	GO:0004871	0.0634
slr1944	GO:0009259	<1e-4	ssr0761	GO:0005996	<1e-4	slr1210	GO:0022838	<1e-4	slr6106	GO:0044424	<1e-4	slr1568	GO:0004871	0.0634
sll0788	GO:0050896	<1e-4	slr7026	GO:0042455	<1e-4	sll5004	GO:0015291	<1e-4	slr6072	GO:0044422	<1e-4	sll1163	GO:0004871	0.0634
slr1935	GO:0033013	<1e-4	sll0910	GO:0043648	<1e-4	sll0063	GO:0022832	<1e-4	slr1187	GO:0044422	<1e-4	sll0867	GO:0004871	0.0634
slr1087	GO:0006793	<1e-4	slr1862	GO:0044282	<1e-4	slr2117	GO:0005342	<1e-4	slr0516	GO:0044422	<1e-4	ssl0312	GO:0004871	0.0634
slr1290	GO:0034660	<1e-4	slr0596	GO:0044106	<1e-4	slr1636	GO:0022832	<1e-4	slr7102	GO:0044444	<1e-4	ssl1464	GO:0004871	0.0634
ssl7074	GO:0044283	<1e-4	slr1913	GO:0051246	<1e-4	sll1063	GO:0043492	<1e-4	sll1401	GO:0044444	<1e-4	ssr6079	GO:0004871	0.0634
sll1960	GO:0006091	<1e-4	ssr1698	GO:0009260	<1e-4	slr1474	GO:0015075	<1e-4	slr0373	GO:0044444	<1e-4	ssl1972	GO:0004871	0.0634
sll0847	GO:0006576	<1e-4	slr0503	GO:0046164	<1e-4	sll0069	GO:0022832	<1e-4	slr1035	GO:0005575	<1e-4	slr6074	GO:0004871	0.0634
sll1485	GO:0010468	<1e-4	slr0195	GO:0006091	<1e-4	sll0532	GO:0022890	<1e-4	sll0898	GO:0044446	<1e-4	slr1306	GO:0004871	0.0634
slr2060	GO:0055086	<1e-4	sll1321	GO:0046907	<1e-4	ssl0352	GO:0016817	<1e-4	ssl0483	GO:0044444	<1e-4	ssl5095	GO:0004871	0.0634
slr6022	GO:0052188	<1e-4	ssr2553	GO:0009117	<1e-4	slr0217	GO:0060089	<1e-4	ssl5091	GO:0044446	<1e-4	ssl3383	GO:0004871	0.0634
slr1647	GO:0055086	<1e-4	sll5028	GO:0019219	<1e-4	ssr3304	GO:0005342	<1e-4	ssr2912	GO:0044424	<1e-4	slr1365	GO:0004871	0.0634
sll0198	GO:0031326	<1e-4	sll0630	GO:0006732	<1e-4	sll0670	GO:0022838	<1e-4	sll0661	GO:0044444	<1e-4	slr0362	GO:0004871	0.0634
sll0069	GO:0031640	<1e-4	slr1275	GO:0009308	<1e-4	slr1287	GO:0022803	<1e-4	ssr6003	GO:0044446	<1e-4	slr1263	GO:0004871	0.0634
sll1956	GO:0009117	<1e-4	ssl1520	GO:0072522	<1e-4	sll1528	GO:0008171	<1e-4	ssr6062	GO:0044422	<1e-4	ssr5020	GO:0004871	0.0634
slr0887	GO:0009141	<1e-4	slr0948	GO:0046483	<1e-4	ssl0242	GO:0060089	<1e-4	slr1999	GO:0044422	<1e-4	sll1757	GO:0004871	0.0634
sll0281	GO:0006811	<1e-4	sll1505	GO:0009991	<1e-4	sll0839	GO:0016817	<1e-4	ssr3122	GO:0044444	<1e-4	ssr2317	GO:0004871	0.0634
slr7058	GO:0043412	<1e-4	sll1785	GO:0009987	<1e-4	slr7073	GO:0022892	<1e-4	sll1640	GO:0043226	<1e-4	slr0168	GO:0004871	0.0634
sll0496	GO:0009123	<1e-4	slr0924	GO:0006811	<1e-4	slr6039	GO:0043492	<1e-4	slr1773	GO:0043226	<1e-4	slr6006	GO:0004871	0.0634
sll0860	GO:0050789	<1e-4	sll0661	GO:0006091	<1e-4	sll1063	GO:0022890	<1e-4	sll1321	GO:0044424	<1e-4	ssr2611	GO:0004871	0.0634
sll0765	GO:0043170	<1e-4	sll7086	GO:0019752	<1e-4	slr6100	GO:0043492	<1e-4	sll0031	GO:0044446	<1e-4	slr0890	GO:0004871	0.0634
sll1912	GO:0009142	<1e-4	sll5034	GO:0055086	<1e-4	sll0293	GO:0022892	<1e-4	sll1526	GO:0044424	<1e-4	slr0145	GO:0004871	0.0634
sll0047	GO:0072524	<1e-4	slr0650	GO:0046164	<1e-4	slr1627	GO:0016462	<1e-4	slr1413	GO:0044464	<1e-4	sll2015	GO:0004871	0.0634
sll5034	GO:0051171	<1e-4	slr1788	GO:0048518	<1e-4	sll0149	GO:0046873	<1e-4	sll0376	GO:0005575	<1e-4	slr0196	GO:0004871	0.0634
sll2011	GO:0008150	<1e-4	slr0442	GO:0072524	<1e-4	ssr6002	GO:0015077	<1e-4	sll1632	GO:0044422	<1e-4	sll1123	GO:0004871	0.0634
slr1215	GO:0009263	<1e-4	sll1166	GO:0046164	<1e-4	sll7077	GO:0016462	<1e-4	sll0611	GO:0043226	<1e-4	ssl1498	GO:0004871	0.0634
sll0822	GO:0052111	<1e-4	sll0149	GO:0034654	<1e-4	sll0577	GO:0043492	<1e-4	slr0031	GO:0043226	<1e-4	sll0063	GO:0004871	0.0634
slr0865	GO:0051188	<1e-4	sll0265	GO:0009893	<1e-4	sll1613	GO:0015399	<1e-4	sll0572	GO:0044424	<1e-4	slr1721	GO:0004871	0.0634
slr0006	GO:0042451	<1e-4	sll1950	GO:0051716	<1e-4	sll1192	GO:0043492	<1e-4	sll1132	GO:0043226	<1e-4	ssr1407	GO:0004871	0.0634
sll1399	GO:0006631	<1e-4	slr0656	GO:0031323	<1e-4	sll1109	GO:0042623	<1e-4	slr0356	GO:0044446	<1e-4	sgl0002	GO:0004871	0.0634
slr1338	GO:0009263	<1e-4	sll1265	GO:0016053	<1e-4	sll0861	GO:0016462	<1e-4	slr0964	GO:0044464	<1e-4	slr7080	GO:0004871	0.0634
sll0360	GO:0009309	<1e-4	slr1616	GO:0009123	<1e-4	sll0910	GO:0022857	<1e-4	sll0162	GO:0044464	<1e-4	slr1363	GO:0004871	0.0634
slr1241	GO:0009259	<1e-4	ssl0467	GO:0031323	<1e-4	sll1201	GO:0022857	<1e-4	sll1884	GO:0044425	<1e-4	sll0397	GO:0004871	0.0634
slr0907	GO:0009126	<1e-4	sll0160	GO:0044283	<1e-4	ssl3573	GO:0015267	<1e-4	slr0921	GO:0044446	<1e-4	slr0655	GO:0004871	0.0634
ssl1300	GO:0046164	<1e-4	slr1563	GO:0052188	<1e-4	sll8033	GO:0022803	<1e-4	slr1071	GO:0044446	<1e-4	slr1547	GO:0004871	0.0634
sll1773	GO:0051179	<1e-4	slr0192	GO:0008150	<1e-4	slr0496	GO:0015077	<1e-4	slr1168	GO:0044424	<1e-4	slr2010	GO:0004871	0.0634
sll2011	GO:0046164	<1e-4	slr0148	GO:0006811	<1e-4	sll0980	GO:0005342	<1e-4	sll0272	GO:0044444	<1e-4	ssl2420	GO:0004871	0.0634
slr1699	GO:0006732	<1e-4	slr1911	GO:0019362	<1e-4	ssr5092	GO:0042623	<1e-4	slr0204	GO:0044425	<1e-4	sll5006	GO:0004871	0.0634
slr0049	GO:0044271	<1e-4	sll1306	GO:0044248	<1e-4	ssl7048	GO:0043492	<1e-4	sll0602	GO:0044444	<1e-4	ssl2920	GO:0004871	0.0634
sll8027	GO:0071841	<1e-4	sll0547	GO:0018130	<1e-4	sll1656	GO:0022857	<1e-4	ssr2333	GO:0005575	<1e-4	slr0489	GO:0004871	0.0634
sll1954	GO:0008610	<1e-4	sll1853	GO:0071842	<1e-4	sll1921	GO:0042623	<1e-4	sll7066	GO:0005575	<1e-4	slr0751	GO:0004871	0.0634
sll1218	GO:0010556	<1e-4	slr1944	GO:0055086	<1e-4	slr0380	GO:0043492	<1e-4	sll1241	GO:0044446	<1e-4	sll7062	GO:0004871	0.0634
slr1495	GO:0034641	<1e-4	slr1737	GO:0018130	<1e-4	ssr0761	GO:0022891	<1e-4	slr0145	GO:0005575	<1e-4	sll7063	GO:0004871	0.0634
slr1599	GO:0033013	<1e-4	ssr6085	GO:0018130	<1e-4	slr0263	GO:0042623	<1e-4	sll1638	GO:0044424	<1e-4	slr1260	GO:0004871	0.0634
ssl0353	GO:0019219	<1e-4	sll1217	GO:0009141	<1e-4	ssl3383	GO:0015405	<1e-4	sll0611	GO:0044425	<1e-4	sll0423	GO:0004871	0.0634
sll0543	GO:0033013	<1e-4	ssr1768	GO:0019219	<1e-4	slr0326	GO:0042623	<1e-4	sll5061	GO:0043226	<1e-4	slr0092	GO:0004871	0.0634
ssl5015	GO:0051171	<1e-4	ssr7072	GO:0044282	<1e-4	sll1006	GO:0060089	<1e-4	slr0013	GO:0044446	<1e-4	slr1069	GO:0004871	0.0634
slr1397	GO:0009308	<1e-4	ssr2554	GO:0065007	<1e-4	slr0655	GO:0015077	<1e-4	sll5089	GO:0044425	<1e-4	ssl5045	GO:0004871	0.0634
ssr0335	GO:0072522	<1e-4	slr0959	GO:0046483	<1e-4	slr0957	GO:0016817	<1e-4	slr7101	GO:0044425	<1e-4	ssl2009	GO:0004871	0.0634

slr0941	GO:0009161	<1e-4	slr6072	GO:0071841	<1e-4	sll0198	GO:0015267	<1e-4	slr1152	GO:0044446	<1e-4	sll0048	GO:0004871	0.0634
sll0505	GO:0009308	<1e-4	slr2071	GO:0043933	<1e-4	slr5111	GO:0022891	<1e-4	sll1472	GO:0005575	<1e-4	sll1053	GO:0004871	0.0634
slr1276	GO:0043436	<1e-4	slr1866	GO:0022607	<1e-4	sll0867	GO:0016746	<1e-4	ssl2245	GO:0044446	<1e-4	sll1939	GO:0004871	0.0634
slr0144	GO:0071842	<1e-4	sll8004	GO:0008150	<1e-4	sll1036	GO:0015293	<1e-4	slr0545	GO:0044464	<1e-4	sll0102	GO:0004871	0.0634
slr2010	GO:0019222	<1e-4	slr1073	GO:0072522	<1e-4	sll0192	GO:0016746	<1e-4	sll1586	GO:0044424	<1e-4	ssr7072	GO:0004871	0.0634
ssr7093	GO:0044275	<1e-4	slr0456	GO:0048878	<1e-4	slr7023	GO:0022857	<1e-4	sll7066	GO:0044425	<1e-4	sll1773	GO:0004871	0.0634
sll0710	GO:0006753	<1e-4	ssr6002	GO:0006733	<1e-4	slr0199	GO:0003674	<1e-4	slr2120	GO:0043226	<1e-4	slr0784	GO:0004871	0.0634
slr1116	GO:0046907	<1e-4	sll1766	GO:0046164	<1e-4	ssl0787	GO:0015075	<1e-4	ssl2065	GO:0044446	<1e-4	slr0398	GO:0004871	0.0634
ssr3467	GO:0009150	<1e-4	sll1562	GO:0055086	<1e-4	sll1250	GO:0022891	<1e-4	slr1918	GO:0044464	<1e-4	sll1765	GO:0004871	0.0634
sll0372	GO:0044283	<1e-4	slr1612	GO:0008150	<1e-4	slr1110	GO:0016746	<1e-4	ssl0483	GO:0044424	<1e-4	slr1566	GO:0004871	0.0634
slr1885	GO:0009199	<1e-4	slr1576	GO:0019752	<1e-4	sll0444	GO:0022891	<1e-4	ssl5065	GO:0044464	<1e-4	sll1401	GO:0004871	0.0634
ssl7022	GO:0090304	<1e-4	sll0218	GO:0009132	<1e-4	sll0997	GO:0005342	<1e-4	slr0112	GO:0044464	<1e-4	sll0930	GO:0004871	0.0634
slr1847	GO:0065007	<1e-4	sll1424	GO:0009308	<1e-4	slr1923	GO:0043167	<1e-4	sll1562	GO:0044425	<1e-4	sll1025	GO:0004871	0.0634
sll0225	GO:0048519	<1e-4	slr1056	GO:0009309	<1e-4	slr1152	GO:0015293	<1e-4	slr0157	GO:0044464	<1e-4	sll0448	GO:0004871	0.0634
sll0596	GO:0006721	<1e-4	sll1773	GO:0006163	<1e-4	ssr3122	GO:0016817	<1e-4	sll0309	GO:0005575	<1e-4	ssr2422	GO:0004871	0.0634
sll5090	GO:0051171	<1e-4	slr0967	GO:0009056	<1e-4	sll0263	GO:0016462	<1e-4	slr0554	GO:0044424	<1e-4	slr0619	GO:0004871	0.0634
slr1768	GO:0072522	<1e-4	ssl5027	GO:0019637	<1e-4	slr1032	GO:0015293	<1e-4	sll0985	GO:0044424	<1e-4	sll0658	GO:0004871	0.0634
sll0447	GO:0043549	<1e-4	slr5021	GO:0051716	<1e-4	slr1767	GO:0003674	<1e-4	slr0667	GO:0043226	<1e-4	slr6072	GO:0004871	0.0634
sll7089	GO:0016053	<1e-4	sll0564	GO:0018130	<1e-4	sll0658	GO:0022803	<1e-4	ssr6099	GO:0044424	<1e-4	slr1218	GO:0004871	0.0634
slr0404	GO:0044282	<1e-4	sll0886	GO:0043170	<1e-4	slr1053	GO:0008171	<1e-4	slr1383	GO:0044446	<1e-4	sll1232	GO:0004871	0.0634
slr0287	GO:0009263	<1e-4	sll7069	GO:0009309	<1e-4	sll2011	GO:0016741	<1e-4	slr6081	GO:0044425	<1e-4	sll1571	GO:0004871	0.0634
sll0853	GO:0009260	<1e-4	ssl1577	GO:0051347	<1e-4	slr0169	GO:0043492	<1e-4	ssl5031	GO:0044446	<1e-4	slr1384	GO:0004871	0.0634
slr1505	GO:0072522	<1e-4	sll0428	GO:0034654	<1e-4	sll0909	GO:0015075	<1e-4	sll0822	GO:0005575	<1e-4	ssr1499	GO:0004871	0.0634
sll5006	GO:0009308	<1e-4	slr7083	GO:0009123	<1e-4	ssr0109	GO:0016817	<1e-4	sll0670	GO:0005575	<1e-4	ssr0332	GO:0004871	0.0634
slr7071	GO:0009263	<1e-4	slr1047	GO:0019751	<1e-4	sll1240	GO:0022891	<1e-4	slr0325	GO:0044446	<1e-4	slr1362	GO:0004871	0.0634
slr6101	GO:0060255	<1e-4	sll1764	GO:0042180	<1e-4	ssr1375	GO:0022890	<1e-4	slr5127	GO:0044444	<1e-4	slr1270	GO:0004871	0.0634
ssr2998	GO:0006576	<1e-4	sll1340	GO:0090304	<1e-4	slr0655	GO:0022890	<1e-4	slr0300	GO:0044446	<1e-4	sll5046	GO:0004871	0.0634
sll1570	GO:0048523	<1e-4	sll0985	GO:0009123	<1e-4	slr1814	GO:0022890	<1e-4	sll1675	GO:0044444	<1e-4	slr1636	GO:0009058	0.0628
slr6039	GO:0019752	<1e-4	slr1507	GO:0009150	<1e-4	slr0709	GO:0022892	<1e-4	sll7065	GO:0005575	<1e-4	sll0413	GO:0009058	0.0628
sll1285	GO:0046164	<1e-4	ssl3615	GO:0051347	<1e-4	sll1495	GO:0022803	<1e-4	slr0192	GO:0043226	<1e-4	slr1471	GO:0005215	0.0626
slr1287	GO:0043170	<1e-4	slr0728	GO:0080090	<1e-4	slr0728	GO:0016818	<1e-4	slr0642	GO:0005575	<1e-4	ssr2553	GO:0003676	0.0623
slr1415	GO:0009893	<1e-4	sll0864	GO:0009893	<1e-4	sll1414	GO:0022836	<1e-4	sll0296	GO:0044424	<1e-4	sll0376	GO:0003676	0.0623
sll0157	GO:0071496	<1e-4	slr0863	GO:0006720	<1e-4	sll0216	GO:0060089	<1e-4	sll1173	GO:0044424	<1e-4	sll0565	GO:0003676	0.0623
sll0445	GO:0051179	<1e-4	sll2015	GO:0044282	<1e-4	slr0962	GO:0060089	<1e-4	slr0751	GO:0005575	<1e-4	slr0967	GO:0003676	0.0623
ssr3467	GO:0044271	<1e-4	sll1939	GO:0042455	<1e-4	sll1247	GO:0043492	<1e-4	slr1183	GO:0043226	<1e-4	slr1970	GO:0003676	0.0623
sll0096	GO:0009126	<1e-4	sll0456	GO:0009141	<1e-4	ssl0832	GO:0060089	<1e-4	slr2115	GO:0044444	<1e-4	sll1052	GO:0003676	0.0623
slr0179	GO:0009892	<1e-4	sll1072	GO:0044283	<1e-4	slr0358	GO:0022838	<1e-4	sll1898	GO:0044425	<1e-4	sll8019	GO:0003676	0.0623
sll1954	GO:0009394	<1e-4	sll0611	GO:0072524	<1e-4	sll1783	GO:0016817	<1e-4	slr0341	GO:0005575	<1e-4	slr0667	GO:0003676	0.0623
slr0269	GO:0044003	<1e-4	slr1900	GO:0009123	<1e-4	slr0217	GO:0022803	<1e-4	slr1863	GO:0044464	<1e-4	slr6087	GO:0003676	0.0623
sll0615	GO:0050896	<1e-4	sll0301	GO:0009893	<1e-4	slr6009	GO:0022892	<1e-4	slr0006	GO:0044422	<1e-4	sll1583	GO:0003676	0.0623
ssr7072	GO:0043648	<1e-4	ssr3129	GO:0009892	<1e-4	sll0243	GO:0016741	<1e-4	slr0813	GO:0005575	<1e-4	ssr6086	GO:0003676	0.0623
slr1885	GO:0019637	<1e-4	slr0442	GO:0072528	<1e-4	ssl1004	GO:0043492	<1e-4	slr5119	GO:0043226	<1e-4	ssl3573	GO:0003676	0.0623
slr1570	GO:0055114	<1e-4	sll0532	GO:0050789	<1e-4	ssr1558	GO:0022857	<1e-4	sll1004	GO:0005575	<1e-4	slr2103	GO:0003676	0.0623
slr6103	GO:0016052	<1e-4	slr5053	GO:0043170	<1e-4	slr1103	GO:0015399	<1e-4	slr2027	GO:0005575	<1e-4	slr6106	GO:0003676	0.0623
sll0787	GO:0055114	<1e-4	slr2012	GO:0065007	<1e-4	slr0208	GO:0016741	<1e-4	slr1178	GO:0044464	<1e-4	slr1449	GO:0003676	0.0623
sll7089	GO:0080090	<1e-4	sll1583	GO:0042451	<1e-4	slr0869	GO:0060089	<1e-4	sll0737	GO:0044422	<1e-4	slr0780	GO:0003676	0.0623
slr2080	GO:0044260	<1e-4	ssr2422	GO:0006631	<1e-4	slr0712	GO:0003674	<1e-4	slr0509	GO:0044422	<1e-4	slr1951	GO:0003676	0.0623
slr7015	GO:0019222	<1e-4	sll1267	GO:0008150	<1e-4	slr0360	GO:0043167	<1e-4	slr1152	GO:0044422	<1e-4	sll1307	GO:0003676	0.0623
slr1721	GO:0006631	<1e-4	ssl0832	GO:0051716	<1e-4	slr0249	GO:0042623	<1e-4	sll1606	GO:0005575	<1e-4	ssr1041	GO:0003676	0.0623
slr7098	GO:0019751	<1e-4	ssl5064	GO:0048523	<1e-4	ssr3341	GO:0015399	<1e-4	slr0341	GO:0044446	<1e-4	ssr3129	GO:0003676	0.0623
slr1073	GO:0009308	<1e-4	sll0595	GO:0019752	<1e-4	slr1809	GO:0022836	<1e-4	slr1189	GO:0005575	<1e-4	sll1095	GO:0003676	0.0623
ssl0350	GO:0048523	<1e-4	ssl0832	GO:0016053	<1e-4	sll0839	GO:0016741	<1e-4	sll1381	GO:0043226	<1e-4	slr5102	GO:0003676	0.0623
sll8011	GO:0048518	<1e-4	sll1660	GO:0009141	<1e-4	slr1789	GO:0005342	<1e-4	sll0872	GO:0044425	<1e-4	sll7069	GO:0003676	0.0623
slr1699	GO:0008610	<1e-4	sll1106	GO:0009889	<1e-4	slr0341	GO:0022836	<1e-4	sll0394	GO:0005575	<1e-4	ssr3572	GO:0003676	0.0623

ssl5113	GO:0009893	<1e-4	sll0157	GO:0044248	<1e-4	sll1726	GO:0022836	<1e-4	slr6016	GO:0044446	<1e-4	sll1486	GO:0003676	0.0623
slr0723	GO:0019751	<1e-4	sll1730	GO:0043549	<1e-4	ssr6089	GO:0022890	<1e-4	slr0765	GO:0043226	<1e-4	slr1628	GO:0003676	0.0623
sgl0002	GO:0050789	<1e-4	sll0737	GO:0009161	<1e-4	sll0185	GO:0015293	<1e-4	sll0615	GO:0044425	<1e-4	slr1780	GO:0003676	0.0623
slr6103	GO:0010468	<1e-4	sll1469	GO:0031326	<1e-4	slr1571	GO:0022836	<1e-4	sll1053	GO:0044425	<1e-4	ssl5015	GO:0003676	0.0623
slr2027	GO:0009150	<1e-4	sll1692	GO:0009142	<1e-4	slr1188	GO:0015075	<1e-4	slr2037	GO:0044464	<1e-4	slr5012	GO:0003676	0.0623
slr1951	GO:0008150	<1e-4	slr5127	GO:0006082	<1e-4	ssr7035	GO:0016462	<1e-4	sll1340	GO:0043226	<1e-4	ssr6078	GO:0003676	0.0623
slr0909	GO:0065007	<1e-4	slr1071	GO:0019222	<1e-4	slr0305	GO:0060089	<1e-4	sll1940	GO:0044444	<1e-4	sll1652	GO:0003676	0.0623
sll1638	GO:0048878	<1e-4	sll1241	GO:0006720	<1e-4	sll1155	GO:0022890	<1e-4	slr1854	GO:0005575	<1e-4	slr0109	GO:0003676	0.0623
slr5077	GO:0009394	<1e-4	sll0913	GO:0044275	<1e-4	ssr3571	GO:0022892	<1e-4	slr0592	GO:0005575	<1e-4	sll1926	GO:0003676	0.0623
slr0108	GO:0009308	<1e-4	sll1173	GO:0019222	<1e-4	slr1648	GO:0043492	<1e-4	sll0933	GO:0005575	<1e-4	sll7070	GO:0003676	0.0623
sll0981	GO:0022411	<1e-4	slr0482	GO:0050801	<1e-4	sll5046	GO:0015293	<1e-4	sll0732	GO:0005575	<1e-4	slr0610	GO:0003676	0.0623
slr0456	GO:0009126	<1e-4	sll0553	GO:0051716	<1e-4	sll1203	GO:0022857	<1e-4	slr1441	GO:0044424	<1e-4	slr0914	GO:0003676	0.0623
sll1504	GO:0042451	<1e-4	slr1534	GO:0044260	<1e-4	ssl0109	GO:0016817	<1e-4	slr1918	GO:0044424	<1e-4	ssl8028	GO:0003676	0.0623
slr0304	GO:0051186	<1e-4	slr6015	GO:0009142	<1e-4	slr0971	GO:0015291	<1e-4	slr1855	GO:0044446	<1e-4	slr1807	GO:0003676	0.0623
slr1168	GO:0046483	<1e-4	slr1442	GO:0006091	<1e-4	ssr3572	GO:0015405	<1e-4	slr0169	GO:0044425	<1e-4	sll1882	GO:0003676	0.0623
ssr1473	GO:0034654	<1e-4	sll1726	GO:0010556	<1e-4	slr1721	GO:0015405	<1e-4	slr1778	GO:0044425	<1e-4	sll7031	GO:0003676	0.0623
slr1915	GO:0006576	<1e-4	slr0610	GO:0031323	<1e-4	sll0372	GO:0060089	<1e-4	slr1507	GO:0043226	<1e-4	ssr1558	GO:0003676	0.0623
slr0871	GO:0008610	<1e-4	sll0481	GO:0046483	<1e-4	slr1070	GO:0015405	<1e-4	slr2125	GO:0044446	<1e-4	ssr0109	GO:0003676	0.0623
slr0270	GO:0072522	<1e-4	ssr1391	GO:0043436	<1e-4	slr1363	GO:0015077	<1e-4	slr1737	GO:0044464	<1e-4	slr0816	GO:0003676	0.0623
slr0380	GO:0009259	<1e-4	slr2103	GO:0042455	<1e-4	slr6031	GO:0022803	<1e-4	sll1912	GO:0005575	<1e-4	slr0670	GO:0003676	0.0623
slr6006	GO:0009892	<1e-4	sll0933	GO:0006732	<1e-4	slr0545	GO:0022836	<1e-4	sll1671	GO:0044424	<1e-4	ssr5074	GO:0003676	0.0623
sll0532	GO:0009059	<1e-4	sll0241	GO:0051179	<1e-4	slr0845	GO:0043167	<1e-4	slr0023	GO:0044446	<1e-4	slr1624	GO:0003676	0.0623
sgl0002	GO:0034654	<1e-4	slr0179	GO:0071841	<1e-4	slr0360	GO:0016462	<1e-4	slr0765	GO:0044464	<1e-4	sll0160	GO:0003676	0.0623
slr6021	GO:0072527	<1e-4	ssl8008	GO:0009141	<1e-4	sll1509	GO:0022857	<1e-4	ssr6086	GO:0044422	<1e-4	sll1692	GO:0003676	0.0623
slr0598	GO:0045184	<1e-4	ssl0788	GO:0019222	<1e-4	sll0249	GO:0043167	<1e-4	slr2046	GO:0044424	<1e-4	slr2117	GO:0003676	0.0623
ssr1499	GO:0034654	<1e-4	slr0149	GO:0044255	<1e-4	slr0398	GO:0015267	<1e-4	slr0039	GO:0044444	<1e-4	ssr2047	GO:0003676	0.0623
slr1223	GO:0009308	<1e-4	slr1495	GO:0006576	<1e-4	sll0710	GO:0003674	<1e-4	sll0590	GO:0044444	<1e-4	sll0062	GO:0003676	0.0623
slr0334	GO:0009893	<1e-4	slr1162	GO:0006733	<1e-4	sll0702	GO:0016818	<1e-4	slr6008	GO:0043226	<1e-4	sll0939	GO:0003676	0.0623
ssl1263	GO:0071554	<1e-4	slr2092	GO:0080090	<1e-4	ssr5020	GO:0015267	<1e-4	sll1315	GO:0044464	<1e-4	slr0516	GO:0003676	0.0623
ssl3291	GO:0009394	<1e-4	sll6055	GO:0006811	<1e-4	slr1852	GO:0022892	<1e-4	ssr0692	GO:0005575	<1e-4	sll1254	GO:0003676	0.0623
ssr6003	GO:0019362	<1e-4	slr1568	GO:0009260	<1e-4	ssl2595	GO:0015075	<1e-4	sll1511	GO:0044444	<1e-4	slr1623	GO:0003676	0.0623
slr0263	GO:0006721	<1e-4	slr5023	GO:0009309	<1e-4	ssr1256	GO:0005342	<1e-4	sll0702	GO:0005575	<1e-4	sll8040	GO:0003676	0.0623
sll0871	GO:0048518	<1e-4	slr0575	GO:0019222	<1e-4	sll1304	GO:0015077	<1e-4	sll0473	GO:0044425	<1e-4	sll1717	GO:0003676	0.0623
slr1143	GO:0006163	<1e-4	slr5119	GO:0008150	<1e-4	slr0642	GO:0016817	<1e-4	sll0553	GO:0005575	<1e-4	ssr5011	GO:0003676	0.0623
slr7083	GO:0044282	<1e-4	slr0818	GO:0006082	<1e-4	sll1586	GO:0016462	<1e-4	slr0579	GO:0044446	<1e-4	slr1206	GO:0003676	0.0623
sll1401	GO:0006721	<1e-4	sll0985	GO:0009132	<1e-4	sll0487	GO:0022891	<1e-4	sll0162	GO:0043226	<1e-4	slr0039	GO:0003676	0.0623
slr6016	GO:0072522	<1e-4	sll1512	GO:0033013	<1e-4	sll1696	GO:0022838	<1e-4	sll1735	GO:0044446	<1e-4	sll0031	GO:0003676	0.0623
sll0543	GO:0009161	<1e-4	sll0827	GO:0006066	<1e-4	slr0801	GO:0008171	<1e-4	sll0858	GO:0044444	<1e-4	sll1531	GO:0003676	0.0623
slr1628	GO:0048878	<1e-4	sll1485	GO:0009117	<1e-4	slr1083	GO:0015075	<1e-4	slr0975	GO:0044444	<1e-4	slr8022	GO:0003676	0.0623
slr1940	GO:0009260	<1e-4	slr8021	GO:0080090	<1e-4	slr1659	GO:0015405	<1e-4	slr0181	GO:0044425	<1e-4	slr0217	GO:0003676	0.0623
sll7070	GO:0019751	<1e-4	slr1704	GO:0080090	<1e-4	slr0989	GO:0043167	<1e-4	sll0174	GO:0043226	<1e-4	sll0496	GO:0003676	0.0623
sll1873	GO:0042180	<1e-4	ssr6062	GO:0009117	<1e-4	sll0085	GO:0015405	<1e-4	ssr3122	GO:0044464	<1e-4	slr0587	GO:0003676	0.0623
slr1917	GO:0019637	<1e-4	slr0356	GO:0006631	<1e-4	slr0376	GO:0043492	<1e-4	slr1275	GO:0044422	<1e-4	sll5132	GO:0003676	0.0623
sll0827	GO:0050794	<1e-4	slr1927	GO:0001932	<1e-4	sll1355	GO:0015267	<1e-4	slr1187	GO:0044464	<1e-4	ssr2711	GO:0003676	0.0623
slr1568	GO:0071841	<1e-4	slr0149	GO:0018130	<1e-4	slr1914	GO:0015291	<1e-4	slr1343	GO:0044444	<1e-4	ssr5121	GO:0003676	0.0623
sll1504	GO:0006793	<1e-4	slr1677	GO:0060255	<1e-4	ssr6024	GO:0015405	<1e-4	slr1790	GO:0044424	<1e-4	slr0521	GO:0003676	0.0623
slr1327	GO:0051716	<1e-4	ssr2554	GO:0043412	<1e-4	sll1002	GO:0022832	<1e-4	slr0516	GO:0005575	<1e-4	slr0709	GO:0003676	0.0623
slr1189	GO:0046394	<1e-4	slr1505	GO:0018130	<1e-4	ssl8039	GO:0043167	<1e-4	sll1965	GO:0044446	<1e-4	ssr6027	GO:0003676	0.0623
sll1749	GO:0046907	<1e-4	slr1301	GO:0009132	<1e-4	sll1135	GO:0022892	<1e-4	sll1531	GO:0044444	<1e-4	sll0625	GO:0003676	0.0623
slr0300	GO:0044260	<1e-4	slr2018	GO:0071496	<1e-4	ssr2787	GO:0042623	<1e-4	ssl0410	GO:0043226	<1e-4	slr2110	GO:0003676	0.0623
slr6080	GO:0046164	<1e-4	slr1865	GO:0009199	<1e-4	sll0355	GO:0015293	<1e-4	slr1122	GO:0044425	<1e-4	sll0069	GO:0003676	0.0623
ssl3383	GO:0033013	<1e-4	sll0183	GO:0016053	<1e-4	sll0360	GO:0015075	<1e-4	slr0179	GO:0044446	<1e-4	slr7100	GO:0003676	0.0623
ssl5100	GO:0042451	<1e-4	sll8033	GO:0009199	<1e-4	ssl7046	GO:0022891	<1e-4	sll1495	GO:0044464	<1e-4	ssr0761	GO:0003676	0.0623
slr1262	GO:0043549	<1e-4	slr0806	GO:0042455	<1e-4	ssl8039	GO:0015405	<1e-4	sll7090	GO:0044425	<1e-4	slr7099	GO:0003676	0.0623

sll1062	GO:0072527	<1e-4	slr7023	GO:0043412	<1e-4	sll0157	GO:0015075	<1e-4	slr0609	GO:0044464	<1e-4	ssl1046	GO:0003676	0.0623
sll1426	GO:0051186	<1e-4	ssr2975	GO:0044271	<1e-4	slr1660	GO:0015291	<1e-4	sll7087	GO:0044424	<1e-4	slr0238	GO:0003676	0.0623
sll1396	GO:0044271	<1e-4	slr0270	GO:0044106	<1e-4	ssr1425	GO:0015293	<1e-4	slr1541	GO:0044444	<1e-4	slr0813	GO:0003676	0.0623
slr1223	GO:0051818	<1e-4	sll6052	GO:0080090	<1e-4	ssr0941	GO:0005342	<1e-4	slr0243	GO:0043226	<1e-4	slr2004	GO:0003676	0.0623
slr1183	GO:0018130	<1e-4	sll0910	GO:0050801	<1e-4	slr1572	GO:0022836	<1e-4	slr6104	GO:0044444	<1e-4	sll0696	GO:0003676	0.0623
sll1469	GO:0072527	<1e-4	slr0607	GO:0050801	<1e-4	ssr6027	GO:0015293	<1e-4	sll0269	GO:0044464	<1e-4	sll1233	GO:0003676	0.0623
sll1344	GO:0034654	<1e-4	slr1362	GO:0009263	<1e-4	sll1306	GO:0022891	<1e-4	sll1446	GO:0044425	<1e-4	slr0865	GO:0003676	0.0623
sll1285	GO:0009893	<1e-4	sll0442	GO:0055082	<1e-4	sll1049	GO:0005342	<1e-4	slr1397	GO:0044425	<1e-4	slr6104	GO:0003676	0.0623
slr1951	GO:0010556	<1e-4	slr1378	GO:0072528	<1e-4	sll1634	GO:0022892	<1e-4	slr1450	GO:0044446	<1e-4	sll1726	GO:0003676	0.0623
ssr1258	GO:0065007	<1e-4	ssl7074	GO:0050801	<1e-4	sll0280	GO:0060089	<1e-4	sll1613	GO:0005575	<1e-4	slr1406	GO:0003676	0.0623
slr8044	GO:0060255	<1e-4	ssl3382	GO:0043436	<1e-4	slr1474	GO:0060089	<1e-4	sll1399	GO:0044425	<1e-4	slr2003	GO:0003676	0.0623
slr0333	GO:0006732	<1e-4	sll1455	GO:0034654	<1e-4	ssr5120	GO:0005342	<1e-4	slr1816	GO:0043226	<1e-4	slr0637	GO:0003676	0.0623
slr0179	GO:0043436	<1e-4	slr0305	GO:0010468	<1e-4	sll1049	GO:0015405	<1e-4	slr1188	GO:0005575	<1e-4	slr0689	GO:0003676	0.0623
slr7025	GO:0006721	<1e-4	slr1068	GO:0009141	<1e-4	sll0584	GO:0005342	<1e-4	slr1394	GO:0005575	<1e-4	sll0060	GO:0003676	0.0623
sll6055	GO:0051818	<1e-4	slr1571	GO:0046907	<1e-4	sll1783	GO:0016741	<1e-4	slr1660	GO:0044424	<1e-4	slr1737	GO:0003676	0.0623
ssr6030	GO:0019362	<1e-4	sll0839	GO:0009262	<1e-4	sll1765	GO:0015291	<1e-4	slr2012	GO:0044446	<1e-4	ssr5106	GO:0003676	0.0623
sll1775	GO:0009117	<1e-4	slr1128	GO:0006766	<1e-4	sll0740	GO:0015405	<1e-4	ssr2787	GO:0005575	<1e-4	slr5073	GO:0003676	0.0623
sll1319	GO:0010468	<1e-4	slr1977	GO:0055082	<1e-4	slr6049	GO:0022857	<1e-4	ssl5007	GO:0044444	<1e-4	slr0184	GO:0003676	0.0623
sll0822	GO:0006066	<1e-4	slr1533	GO:0048878	<1e-4	sll1773	GO:0043167	<1e-4	slr2080	GO:0044422	<1e-4	slr0181	GO:0003676	0.0623
sll5046	GO:0043648	<1e-4	slr0702	GO:0072528	<1e-4	slr0978	GO:0015077	<1e-4	slr0355	GO:0044464	<1e-4	sll0253	GO:0003676	0.0623
slr0082	GO:0055086	<1e-4	sll0661	GO:0052111	<1e-4	slr1474	GO:0022891	<1e-4	sll1874	GO:0044446	<1e-4	sll1089	GO:0003676	0.0623
sll1735	GO:0090304	<1e-4	slr1812	GO:0016054	<1e-4	ssr2755	GO:0022803	<1e-4	sll1511	GO:0043226	<1e-4	sll5030	GO:0003676	0.0623
slr1032	GO:0051179	<1e-4	sll1911	GO:0019637	<1e-4	sll1378	GO:0060089	<1e-4	sll0905	GO:0044464	<1e-4	slr6068	GO:0003676	0.0623
slr1956	GO:0044282	<1e-4	slr1614	GO:0044260	<1e-4	slr1070	GO:0016817	<1e-4	sll7033	GO:0044425	<1e-4	ssr2060	GO:0003676	0.0623
slr6008	GO:0006811	<1e-4	slr1778	GO:0009394	<1e-4	ssr6078	GO:0022892	<1e-4	slr1505	GO:0005575	<1e-4	sll8001	GO:0003676	0.0623
slr1721	GO:0009892	<1e-4	ssr1407	GO:0034654	<1e-4	slr0728	GO:0005342	<1e-4	ssr7084	GO:0044446	<1e-4	slr1980	GO:0003676	0.0623
slr1270	GO:0009260	<1e-4	slr7094	GO:0046907	<1e-4	sll5004	GO:0015405	<1e-4	sll1273	GO:0005575	<1e-4	sll1285	GO:0003676	0.0623
slr0605	GO:0060255	<1e-4	slr1593	GO:0046483	<1e-4	slr1865	GO:0022836	<1e-4	sll1164	GO:0044464	<1e-4	sll1609	GO:0003676	0.0623
sll8035	GO:0019752	<1e-4	ssr2422	GO:0065007	<1e-4	sll5006	GO:0016741	<1e-4	sll5030	GO:0005575	<1e-4	slr1507	GO:0003676	0.0623
slr1340	GO:0034641	<1e-4	slr0456	GO:0051234	<1e-4	sll1247	GO:0042623	<1e-4	slr6081	GO:0043226	<1e-4	slr8021	GO:0003676	0.0623
slr0148	GO:0034654	<1e-4	slr0270	GO:0044248	<1e-4	sll7047	GO:0060089	<1e-4	slr1767	GO:0044425	<1e-4	sll0905	GO:0003676	0.0623
sll0985	GO:0034660	<1e-4	sll0518	GO:0009117	<1e-4	ssr6030	GO:0003674	<1e-4	slr5023	GO:0044424	<1e-4	ssl5114	GO:0003676	0.0623
sll0208	GO:0055114	<1e-4	slr0888	GO:0046907	<1e-4	sll0176	GO:0015077	<1e-4	sll0606	GO:0005575	<1e-4	slr0602	GO:0016787	0.0614
slr1807	GO:0034641	<1e-4	slr0590	GO:0034641	<1e-4	ssl2148	GO:0060089	<1e-4	slr1593	GO:0044422	<1e-4	slr0771	GO:0017111	0.0589
sll0781	GO:0050801	<1e-4	sll8004	GO:0051179	<1e-4	sll0871	GO:0043167	<1e-4	slr1406	GO:0044464	<1e-4	slr0731	GO:0017111	0.0589
ssl0467	GO:0055114	<1e-4	slr0554	GO:0019752	<1e-4	sll1726	GO:0042623	<1e-4	sll0478	GO:0005575	<1e-4	ssr0761	GO:0017111	0.0589
sll0803	GO:0044106	<1e-4	sll0381	GO:0006220	<1e-4	slr1613	GO:0015399	<1e-4	slr0582	GO:0044425	<1e-4	sll0565	GO:0017111	0.0589
sll0543	GO:0044275	<1e-4	sll0265	GO:0071841	<1e-4	ssr2787	GO:0022838	<1e-4	slr1117	GO:0044425	<1e-4	sll0376	GO:0017111	0.0589
slr1590	GO:0009199	<1e-4	slr5102	GO:0009308	<1e-4	slr0569	GO:0042623	<1e-4	sll0147	GO:0044464	<1e-4	slr5012	GO:0017111	0.0589
sll1340	GO:0048523	<1e-4	sll0787	GO:0006811	<1e-4	slr1636	GO:0043492	<1e-4	sll1025	GO:0044446	<1e-4	slr1142	GO:0017111	0.0589
slr1900	GO:0009059	<1e-4	sll1835	GO:0051186	<1e-4	sll0678	GO:0022838	<1e-4	slr0376	GO:0044446	<1e-4	sll8040	GO:0017111	0.0589
slr1261	GO:0042455	<1e-4	slr7080	GO:0019751	<1e-4	slr0863	GO:0015267	<1e-4	slr1315	GO:0005575	<1e-4	slr0813	GO:0017111	0.0589
slr1462	GO:0051171	<1e-4	slr1110	GO:0006066	<1e-4	slr1970	GO:0005342	<1e-4	sll0249	GO:0044444	<1e-4	slr1290	GO:0017111	0.0589
ssl1923	GO:0043648	<1e-4	sll1586	GO:0065007	<1e-4	sll0630	GO:0042623	<1e-4	slr1667	GO:0044444	<1e-4	sll0905	GO:0017111	0.0589
ssl7022	GO:0009308	<1e-4	slr1557	GO:0055082	<1e-4	sll0763	GO:0016746	<1e-4	slr1690	GO:0044424	<1e-4	slr0816	GO:0017111	0.0589
sll0565	GO:0006732	<1e-4	slr1573	GO:0009262	<1e-4	slr0613	GO:0043167	<1e-4	slr1644	GO:0005575	<1e-4	slr1704	GO:0017111	0.0589
ssl0410	GO:0051188	<1e-4	sll1915	GO:0045184	<1e-4	ssl5100	GO:0022890	<1e-4	ssl5015	GO:0044425	<1e-4	slr1627	GO:0017111	0.0589
slr7101	GO:0009987	<1e-4	slr0250	GO:0006753	<1e-4	sll1241	GO:0022838	<1e-4	slr0121	GO:0044422	<1e-4	slr1623	GO:0017111	0.0589
slr0147	GO:0006631	<1e-4	ssl8005	GO:0010556	<1e-4	slr0184	GO:0015077	<1e-4	slr1990	GO:0044422	<1e-4	slr6045	GO:0017111	0.0589
sll1611	GO:0006576	<1e-4	slr1875	GO:0006766	<1e-4	sll2006	GO:0022890	<1e-4	sll7065	GO:0043226	<1e-4	ssl5108	GO:0017111	0.0589
sll1730	GO:0043436	<1e-4	slr1230	GO:0090304	<1e-4	slr7011	GO:0008171	<1e-4	ssl7039	GO:0044464	<1e-4	ssr6086	GO:0017111	0.0589
ssl2245	GO:0006811	<1e-4	slr7037	GO:0071496	<1e-4	sll1835	GO:0043492	<1e-4	sll0198	GO:0005575	<1e-4	ssl7048	GO:0017111	0.0589
ssl3829	GO:0060255	<1e-4	slr1179	GO:0042455	<1e-4	sll0372	GO:0016817	<1e-4	slr1206	GO:0005575	<1e-4	sll7086	GO:0017111	0.0589
slr1854	GO:0055114	<1e-4	sll0513	GO:0046907	<1e-4	slr1917	GO:0015291	<1e-4	sll1071	GO:0044424	<1e-4	slr5127	GO:0017111	0.0589

ssl1004	GO:0006631	<1e-4	slr1660	GO:0006811	<1e-4	slr1438	GO:0016741	<1e-4	slr0712	GO:0044464	<1e-4	sll0172	GO:0017111	0.0589
sll1285	GO:0001932	<1e-4	sll1186	GO:0006732	<1e-4	sll1726	GO:0043492	<1e-4	slr1590	GO:0044425	<1e-4	sll1319	GO:0017111	0.0589
sll1089	GO:0044282	<1e-4	ssl0352	GO:0042180	<1e-4	ssl2807	GO:0022838	<1e-4	slr1189	GO:0044446	<1e-4	slr0976	GO:0017111	0.0589
slr0751	GO:0051188	<1e-4	slr0519	GO:0019222	<1e-4	slr0989	GO:0042623	<1e-4	sll0237	GO:0044422	<1e-4	slr7091	GO:0017111	0.0589
slr0326	GO:0009987	<1e-4	slr1773	GO:0044283	<1e-4	slr7026	GO:0016741	<1e-4	slr0742	GO:0044425	<1e-4	slr5111	GO:0017111	0.0589
sll1040	GO:0006066	<1e-4	slr1816	GO:0006066	<1e-4	sll7077	GO:0015405	<1e-4	sll0661	GO:0044446	<1e-4	sll8011	GO:0017111	0.0589
sll1995	GO:0009889	<1e-4	slr6044	GO:0046483	<1e-4	sll0572	GO:0015405	<1e-4	slr0456	GO:0005575	<1e-4	ssl1378	GO:0017111	0.0589
slr0708	GO:0009165	<1e-4	slr0609	GO:0009263	<1e-4	ssl1378	GO:0022803	<1e-4	slr0954	GO:0044444	<1e-4	ssr8047	GO:0017111	0.0589
sll1396	GO:0009132	<1e-4	slr1533	GO:0042180	<1e-4	sll0230	GO:0016741	<1e-4	sll7034	GO:0043226	<1e-4	slr0656	GO:0017111	0.0589
sll0802	GO:0051347	<1e-4	ssr7036	GO:0043933	<1e-4	sll1340	GO:0016741	<1e-4	slr1114	GO:0005575	<1e-4	slr0106	GO:0017111	0.0589
sll0586	GO:0043549	<1e-4	slr0921	GO:0046128	<1e-4	sll1232	GO:0015405	<1e-4	slr1343	GO:0005575	<1e-4	sll5003	GO:0017111	0.0589
sll0751	GO:0008150	<1e-4	slr1681	GO:0045184	<1e-4	slr2000	GO:0043492	<1e-4	slr0981	GO:0005575	<1e-4	ssr2318	GO:0017111	0.0589
sll1461	GO:0008610	<1e-4	sll1411	GO:0072524	<1e-4	slr2005	GO:0016746	<1e-4	sll1858	GO:0044424	<1e-4	sll1692	GO:0017111	0.0589
sll0547	GO:0009893	<1e-4	ssr2781	GO:0065007	<1e-4	slr6101	GO:0008171	<1e-4	sll1219	GO:0044424	<1e-4	sll1340	GO:0017111	0.0589
slr1644	GO:0043436	<1e-4	slr1114	GO:0009308	<1e-4	slr1396	GO:0042623	<1e-4	sll0780	GO:0044422	<1e-4	slr1438	GO:0017111	0.0589
sll0802	GO:0009117	<1e-4	sll0910	GO:0016052	<1e-4	slr1045	GO:0022890	<1e-4	sll0614	GO:0044446	<1e-4	sll7089	GO:0017111	0.0589
slr0374	GO:0009057	<1e-4	slr1378	GO:0044271	<1e-4	sll1068	GO:0022838	<1e-4	sll1764	GO:0005575	<1e-4	ssl7022	GO:0017111	0.0589
ssl2595	GO:0072521	<1e-4	sll0839	GO:0048518	<1e-4	sll1783	GO:0022857	<1e-4	slr0038	GO:0044444	<1e-4	sll8035	GO:0017111	0.0589
sll1882	GO:0072524	<1e-4	ssl5098	GO:0019751	<1e-4	slr2027	GO:0016818	<1e-4	sll1950	GO:0044464	<1e-4	sll1495	GO:0017111	0.0589
slr0740	GO:0034654	<1e-4	slr1915	GO:0042180	<1e-4	ssl1417	GO:0015405	<1e-4	slr1170	GO:0044425	<1e-4	sll1926	GO:0017111	0.0589
ssr1407	GO:0008150	<1e-4	slr2111	GO:0044255	<1e-4	slr0589	GO:0015405	<1e-4	sll0558	GO:0044444	<1e-4	slr0610	GO:0017111	0.0589
sll1834	GO:0042180	<1e-4	sll1355	GO:0055086	<1e-4	sll5026	GO:0015075	<1e-4	sll1373	GO:0044446	<1e-4	sll5128	GO:0017111	0.0589
sll5047	GO:0006732	<1e-4	slr0765	GO:0044275	<1e-4	slr1170	GO:0015293	<1e-4	slr6103	GO:0044446	<1e-4	slr1819	GO:0017111	0.0589
sll1461	GO:0043412	<1e-4	sll0482	GO:0009144	<1e-4	slr0554	GO:0022803	<1e-4	sll0068	GO:0044425	<1e-4	sll0294	GO:0017111	0.0589
sll1188	GO:0051716	<1e-4	slr0006	GO:0006163	<1e-4	slr1657	GO:0022892	<1e-4	sll0048	GO:0043226	<1e-4	slr6022	GO:0017111	0.0589
slr1864	GO:0072528	<1e-4	sll0802	GO:0008150	<1e-4	sll0909	GO:0060089	<1e-4	slr1513	GO:0005575	<1e-4	slr1087	GO:0017111	0.0589
ssl2162	GO:0071554	<1e-4	ssr3341	GO:0009889	<1e-4	slr0038	GO:0008171	<1e-4	slr1702	GO:0005575	<1e-4	ssr7035	GO:0017111	0.0589
ssl1378	GO:0006733	<1e-4	ssl3451	GO:0051347	<1e-4	ssr2998	GO:0022838	<1e-4	slr0302	GO:0044425	<1e-4	slr1079	GO:0017111	0.0589
sll0827	GO:0009142	<1e-4	slr0149	GO:0019752	<1e-4	sll0445	GO:0015291	<1e-4	sll0994	GO:0044424	<1e-4	ssl3829	GO:0017111	0.0589
sll1766	GO:0019752	<1e-4	slr5024	GO:0046128	<1e-4	ssl7074	GO:0060089	<1e-4	slr1034	GO:0044422	<1e-4	sll0101	GO:0017111	0.0589
sll1938	GO:0006732	<1e-4	slr0148	GO:0043412	<1e-4	slr1854	GO:0042623	<1e-4	slr1591	GO:0005575	<1e-4	sll6055	GO:0017111	0.0589
sll0447	GO:0071496	<1e-4	slr0651	GO:0051716	<1e-4	ssr5020	GO:0015399	<1e-4	slr1301	GO:0005575	<1e-4	sll1192	GO:0017111	0.0589
sll0997	GO:0071496	<1e-4	slr1667	GO:0044106	<1e-4	sll0282	GO:0022836	<1e-4	slr1577	GO:0044424	<1e-4	sll1158	GO:0017111	0.0589
ssr2711	GO:0065007	<1e-4	slr1951	GO:0044275	<1e-4	slr6106	GO:0016817	<1e-4	slr1363	GO:0044446	<1e-4	slr0300	GO:0017111	0.0589
slr1098	GO:0009142	<1e-4	slr1169	GO:0072521	<1e-4	sll1530	GO:0016817	<1e-4	sll5130	GO:0044425	<1e-4	ssl1046	GO:0017111	0.0589
sll0539	GO:0042455	<1e-4	slr1413	GO:0009987	<1e-4	sll1634	GO:0015293	<1e-4	slr0755	GO:0044424	<1e-4	sll0552	GO:0017111	0.0589
slr1353	GO:0055086	<1e-4	sll0286	GO:0009263	<1e-4	sll0763	GO:0015075	<1e-4	sll0101	GO:0044444	<1e-4	slr7073	GO:0017111	0.0589
slr0303	GO:0044248	<1e-4	sll1319	GO:0071842	<1e-4	slr1084	GO:0022803	<1e-4	slr1025	GO:0043226	<1e-4	sll5067	GO:0017111	0.0589
slr0742	GO:0019752	<1e-4	sll0473	GO:0051716	<1e-4	sll0160	GO:0060089	<1e-4	sll0198	GO:0044425	<1e-4	slr1114	GO:0017111	0.0589
slr6038	GO:0006732	<1e-4	slr1977	GO:0044281	<1e-4	ssl2920	GO:0015293	<1e-4	sll0524	GO:0044422	<1e-4	sll1024	GO:0017111	0.0589
slr6044	GO:0006793	<1e-4	ssr1765	GO:0044271	<1e-4	slr0505	GO:0022836	<1e-4	ssl1520	GO:0044446	<1e-4	ssr7079	GO:0017111	0.0589
sll1285	GO:0060255	<1e-4	slr1179	GO:0009263	<1e-4	sll1378	GO:0016746	<1e-4	slr0146	GO:0044446	<1e-4	slr1449	GO:0017111	0.0589
sll1352	GO:0051716	<1e-4	sll1373	GO:0034660	<1e-4	sll0381	GO:0022832	<1e-4	slr0209	GO:0005575	<1e-4	ssr7093	GO:0017111	0.0589
sll1388	GO:0006066	<1e-4	sll7063	GO:0009308	<1e-4	sll0321	GO:0015405	<1e-4	slr0456	GO:0044464	<1e-4	slr0780	GO:0017111	0.0589
ssl2717	GO:0046128	<1e-4	slr1767	GO:0050789	<1e-4	slr0059	GO:0015075	<1e-4	ssr6083	GO:0044422	<1e-4	sll1698	GO:0017111	0.0589
sll6052	GO:0044275	<1e-4	slr2005	GO:0009262	<1e-4	sll1002	GO:0016741	<1e-4	sll1372	GO:0044424	<1e-4	sll7033	GO:0017111	0.0589
ssr0332	GO:0090304	<1e-4	ssr2615	GO:0006721	<1e-4	slr0863	GO:0042623	<1e-4	slr5021	GO:0044444	<1e-4	slr0217	GO:0017111	0.0589
sll1254	GO:0006766	<1e-4	sll0008	GO:0019637	<1e-4	sll0811	GO:0015405	<1e-4	slr1084	GO:0044425	<1e-4	sll1151	GO:0017111	0.0589
ssl8028	GO:0010468	<1e-4	sll0584	GO:0022607	<1e-4	sll1691	GO:0022832	<1e-4	slr7015	GO:0043226	<1e-4	ssr7017	GO:0017111	0.0589
slr1032	GO:0034654	<1e-4	slr1114	GO:0009165	<1e-4	slr1394	GO:0042623	<1e-4	sll0804	GO:0044444	<1e-4	slr0634	GO:0017111	0.0589
sll1606	GO:0006082	<1e-4	sll1352	GO:0018130	<1e-4	ssl0787	GO:0016462	<1e-4	sll0547	GO:0044424	<1e-4	slr1816	GO:0017111	0.0589
slr0912	GO:0006220	<1e-4	slr1378	GO:0009126	<1e-4	sll0177	GO:0005342	<1e-4	ssl0738	GO:0044444	<1e-4	slr1599	GO:0017111	0.0589
sll8004	GO:0031323	<1e-4	ssl5114	GO:0009142	<1e-4	sll1174	GO:0015293	<1e-4	ssr6089	GO:0005575	<1e-4	sll1188	GO:0017111	0.0589
sll0524	GO:0009987	<1e-4	ssr6083	GO:0044275	<1e-4	slr1980	GO:0005342	<1e-4	sll0735	GO:0044422	<1e-4	slr0013	GO:0017111	0.0589

ssr8047	GO:0019222	<1e-4	sll1025	GO:0050789	<1e-4	slr0112	GO:0016741	<1e-4	ssr3409	GO:0043226	<1e-4	sll1882	GO:0017111	0.0589
ssl2148	GO:0009141	<1e-4	ssr0657	GO:0043412	<1e-4	slr1900	GO:0016818	<1e-4	slr1753	GO:0044425	<1e-4	sll1164	GO:0017111	0.0589
sll0811	GO:0051818	<1e-4	slr0269	GO:0055086	<1e-4	sll0602	GO:0005342	<1e-4	ssl1972	GO:0005575	<1e-4	sll0847	GO:0017111	0.0589
sll5062	GO:0009126	<1e-4	ssl2996	GO:0006091	<1e-4	slr0872	GO:0022832	<1e-4	slr1737	GO:0005575	<1e-4	sll0608	GO:0017111	0.0589
slr2003	GO:0034660	<1e-4	sll1289	GO:0051246	<1e-4	slr1362	GO:0043492	<1e-4	slr2101	GO:0043226	<1e-4	sll1979	GO:0017111	0.0589
ssr1256	GO:0044255	<1e-4	slr2049	GO:0022411	<1e-4	ssr2754	GO:0016818	<1e-4	slr1895	GO:0044422	<1e-4	sll1583	GO:0017111	0.0589
sll1089	GO:0050794	<1e-4	sll1906	GO:0016054	<1e-4	slr1880	GO:0022832	<1e-4	slr0729	GO:0044424	<1e-4	slr1513	GO:0017111	0.0589
sll5032	GO:0019222	<1e-4	sll0023	GO:0055114	<1e-4	ssl1300	GO:0016746	<1e-4	slr1074	GO:0044446	<1e-4	slr2000	GO:0017111	0.0589
slr7060	GO:0050794	<1e-4	slr0271	GO:0031323	<1e-4	sll0498	GO:0005342	<1e-4	sll0691	GO:0044446	<1e-4	slr0184	GO:0017111	0.0589
sll1142	GO:0001932	<1e-4	slr1636	GO:0009892	<1e-4	sll1530	GO:0022832	<1e-4	slr1184	GO:0005575	<1e-4	slr0273	GO:0017111	0.0589
sll0424	GO:0043170	<1e-4	slr0978	GO:0010468	<1e-4	slr0712	GO:0015267	<1e-4	ssl1918	GO:0044464	<1e-4	sll7090	GO:0017111	0.0589
sll1658	GO:0065007	<1e-4	slr1152	GO:0071496	<1e-4	slr1303	GO:0015405	<1e-4	sll1123	GO:0044444	<1e-4	slr1570	GO:0017111	0.0589
slr1875	GO:0018130	<1e-4	slr0408	GO:0051716	<1e-4	slr1230	GO:0022838	<1e-4	sll0253	GO:0044425	<1e-4	ssr1155	GO:0017111	0.0589
sll0584	GO:0006163	<1e-4	slr1073	GO:0033013	<1e-4	sll0499	GO:0015399	<1e-4	slr1535	GO:0044446	<1e-4	slr6075	GO:0017111	0.0589
sll1654	GO:0008610	<1e-4	sll0661	GO:0080090	<1e-4	sll1863	GO:0005342	<1e-4	slr2042	GO:0044422	<1e-4	slr0575	GO:0017111	0.0589
ssl5129	GO:0072521	<1e-4	slr1462	GO:0051716	<1e-4	slr5127	GO:0015075	<1e-4	ssr2611	GO:0043226	<1e-4	sll1486	GO:0017111	0.0589
sll0547	GO:0009056	<1e-4	sll0539	GO:0060255	<1e-4	slr1648	GO:0022857	<1e-4	slr1464	GO:0044422	<1e-4	sll1191	GO:0017111	0.0589
slr0919	GO:0072528	<1e-4	ssr7036	GO:0006576	<1e-4	ssr5092	GO:0015405	<1e-4	sll1071	GO:0044422	<1e-4	sll1784	GO:0017111	0.0589
ssr1375	GO:0051246	<1e-4	sll1092	GO:0042180	<1e-4	slr0981	GO:0015405	<1e-4	slr0919	GO:0044425	<1e-4	sll0793	GO:0017111	0.0589
slr5013	GO:0022411	<1e-4	slr6005	GO:0005996	<1e-4	slr1812	GO:0022891	<1e-4	sll1130	GO:0044444	<1e-4	slr0285	GO:0017111	0.0589
slr2060	GO:0022607	<1e-4	slr6064	GO:0009987	<1e-4	slr1169	GO:0060089	<1e-4	ssr0761	GO:0044422	<1e-4	sll0044	GO:0017111	0.0589
slr7097	GO:0016053	<1e-4	sll1640	GO:0019222	<1e-4	slr1230	GO:0015291	<1e-4	sll7034	GO:0044422	<1e-4	sll0479	GO:0017111	0.0589
slr0787	GO:0072521	<1e-4	sll0639	GO:0051179	<1e-4	slr1576	GO:0060089	<1e-4	ssl5099	GO:0005575	<1e-4	ssr6019	GO:0017111	0.0589
slr1419	GO:0060255	<1e-4	ssl3379	GO:0009308	<1e-4	sll1399	GO:0015405	<1e-4	slr0374	GO:0043226	<1e-4	sll7034	GO:0017111	0.0589
slr1875	GO:0008150	<1e-4	slr1116	GO:0044281	<1e-4	slr2117	GO:0015399	<1e-4	slr2060	GO:0043226	<1e-4	sll5063	GO:0017111	0.0589
slr1161	GO:0043549	<1e-4	ssl3549	GO:0046128	<1e-4	slr0468	GO:0015267	<1e-4	slr0773	GO:0044424	<1e-4	sll1891	GO:0017111	0.0589
sll1262	GO:0009132	<1e-4	slr1623	GO:0009309	<1e-4	slr0250	GO:0022838	<1e-4	sll0410	GO:0044444	<1e-4	ssl1300	GO:0017111	0.0589
sll1652	GO:0044248	<1e-4	sll1715	GO:0006721	<1e-4	slr1907	GO:0022803	<1e-4	sll1119	GO:0044446	<1e-4	ssr2060	GO:0017111	0.0589
slr5012	GO:0044260	<1e-4	slr1438	GO:0006720	<1e-4	slr0423	GO:0043167	<1e-4	ssl1004	GO:0044425	<1e-4	ssr2711	GO:0017111	0.0589
ssr0692	GO:0001932	<1e-4	slr1533	GO:0008150	<1e-4	slr0637	GO:0022857	<1e-4	sll1095	GO:0044446	<1e-4	ssl5114	GO:0017111	0.0589
sll0008	GO:0009308	<1e-4	slr1230	GO:0042455	<1e-4	slr1074	GO:0043492	<1e-4	sll1289	GO:0044422	<1e-4	ssl7007	GO:0017111	0.0589
slr2120	GO:0033013	<1e-4	slr0509	GO:0044003	<1e-4	sll0547	GO:0022891	<1e-4	sll0369	GO:0005575	<1e-4	slr0238	GO:0017111	0.0589
ssr1256	GO:0065007	<1e-4	sll7090	GO:0050801	<1e-4	sll1240	GO:0022857	<1e-4	sll0609	GO:0044446	<1e-4	ssr2047	GO:0017111	0.0589
slr0108	GO:0022607	<1e-4	slr0654	GO:0033013	<1e-4	sll0669	GO:0008171	<1e-4	ssl5045	GO:0044422	<1e-4	ssr5106	GO:0017111	0.0589
ssr6026	GO:0006720	<1e-4	sll0871	GO:0019637	<1e-4	slr5053	GO:0016818	<1e-4	slr1647	GO:0005575	<1e-4	sll1307	GO:0017111	0.0589
ssl0788	GO:0009117	<1e-4	ssr2711	GO:0052188	<1e-4	sll0984	GO:0003674	<1e-4	slr1563	GO:0044444	<1e-4	sll5028	GO:0017111	0.0589
sll0857	GO:0022607	<1e-4	slr0957	GO:0008610	<1e-4	slr0397	GO:0008171	<1e-4	sll1024	GO:0044424	<1e-4	slr2012	GO:0017111	0.0589
sll0572	GO:0009117	<1e-4	sll0743	GO:0016054	<1e-4	slr0912	GO:0015077	<1e-4	sll1512	GO:0043226	<1e-4	slr0590	GO:0017111	0.0589
slr1194	GO:0046164	<1e-4	slr0801	GO:0019362	<1e-4	sll1158	GO:0022891	<1e-4	ssr6062	GO:0005575	<1e-4	ssr6024	GO:0017111	0.0589
slr0262	GO:0010556	<1e-4	slr0169	GO:0019222	<1e-4	sll1225	GO:0022892	<1e-4	slr1657	GO:0044425	<1e-4	slr0651	GO:0017111	0.0589
sll0815	GO:0009991	<1e-4	sll7031	GO:0016053	<1e-4	sll0614	GO:0022891	<1e-4	slr6090	GO:0044424	<1e-4	ssr5092	GO:0017111	0.0589
slr0787	GO:0051347	<1e-4	sll0243	GO:0051347	<1e-4	sll0410	GO:0060089	<1e-4	sll0602	GO:0044422	<1e-4	slr0345	GO:0017111	0.0589
slr1847	GO:0009991	<1e-4	sll1426	GO:0006811	<1e-4	sll0539	GO:0022857	<1e-4	sll1389	GO:0044422	<1e-4	sll0939	GO:0017111	0.0589
ssr2711	GO:0009057	<1e-4	slr0232	GO:0019219	<1e-4	sll1542	GO:0016818	<1e-4	sll0846	GO:0044444	<1e-4	slr0613	GO:0017111	0.0589
ssl6035	GO:0009123	<1e-4	slr1814	GO:0009117	<1e-4	sll0361	GO:0015293	<1e-4	sll5047	GO:0044422	<1e-4	ssr3570	GO:0017111	0.0589
sll0518	GO:0009893	<1e-4	slr1753	GO:0006082	<1e-4	sll1380	GO:0015293	<1e-4	ssl5114	GO:0044425	<1e-4	ssl5025	GO:0017111	0.0589
sll1340	GO:0019752	<1e-4	sll0984	GO:0055114	<1e-4	sll1761	GO:0016818	<1e-4	sll0775	GO:0044422	<1e-4	slr0730	GO:0017111	0.0589
ssr6085	GO:0009309	<1e-4	slr1546	GO:0006066	<1e-4	sll7078	GO:0015405	<1e-4	slr6066	GO:0044425	<1e-4	slr2004	GO:0017111	0.0589
slr1203	GO:0044260	<1e-4	slr1721	GO:0005996	<1e-4	sll0811	GO:0022857	<1e-4	slr1644	GO:0044464	<1e-4	sll0060	GO:0017111	0.0589
sll0552	GO:0009165	<1e-4	slr0731	GO:0080090	<1e-4	slr0192	GO:0016817	<1e-4	slr0521	GO:0044422	<1e-4	ssr6062	GO:0017111	0.0589
sll7087	GO:0071554	<1e-4	sll1192	GO:0009150	<1e-4	slr2117	GO:0016818	<1e-4	sll0162	GO:0044444	<1e-4	slr0689	GO:0017111	0.0589
slr1052	GO:0006753	<1e-4	sll1752	GO:0019637	<1e-4	slr1648	GO:0016818	<1e-4	slr0359	GO:0005575	<1e-4	sll1388	GO:0017111	0.0589
slr0914	GO:0010556	<1e-4	slr1900	GO:0009165	<1e-4	slr7013	GO:0008171	<1e-4	ssr7093	GO:0044422	<1e-4	slr1970	GO:0017111	0.0589
slr1472	GO:0050789	<1e-4	sll0451	GO:0043648	<1e-4	sll1352	GO:0015293	<1e-4	sll0702	GO:0044425	<1e-4	sll1834	GO:0017111	0.0589

slr1570	GO:0072522	<1e-4	ssr0109	GO:0046394	<1e-4	slr1290	GO:0015293	<1e-4	slr1103	GO:0044424	<1e-4	sll1898	GO:0017111	0.0589
slr0108	GO:0043412	<1e-4	sll0281	GO:0034654	<1e-4	sll0096	GO:0008171	<1e-4	ssl3615	GO:0044444	<1e-4	sll0298	GO:0017111	0.0589
sll1359	GO:0071496	<1e-4	ssr0536	GO:0051246	<1e-4	slr0509	GO:0022857	<1e-4	ssl2069	GO:0043226	<1e-4	slr1681	GO:0017111	0.0589
slr0341	GO:0018130	<1e-4	ssl5031	GO:0051234	<1e-4	sll0242	GO:0016829	<1e-4	ssl2807	GO:0044424	<1e-4	sll0563	GO:0017111	0.0589
slr1970	GO:0019637	<1e-4	sll1863	GO:0050794	<1e-4	sll1765	GO:0042623	<1e-4	ssr6083	GO:0044444	<1e-4	ssl2384	GO:0017111	0.0589
sll0743	GO:0018130	<1e-4	slr1813	GO:0055082	<1e-4	ssr6078	GO:0016462	<1e-4	slr1752	GO:0044464	<1e-4	sll0858	GO:0017111	0.0589
sll0436	GO:0009394	<1e-4	slr5013	GO:0009059	<1e-4	sll1773	GO:0015293	<1e-4	slr0872	GO:0044446	<1e-4	ssl6092	GO:0017111	0.0589
slr1667	GO:0019222	<1e-4	slr1206	GO:0008150	<1e-4	sll1054	GO:0022857	<1e-4	slr1652	GO:0044424	<1e-4	ssr2972	GO:0017111	0.0589
sll1749	GO:0042451	<1e-4	slr2025	GO:0006753	<1e-4	ssl8028	GO:0022838	<1e-4	sll1737	GO:0044425	<1e-4	ssl7021	GO:0017111	0.0589
sml0011	GO:0046907	<1e-4	sll1006	GO:0006082	<1e-4	slr1301	GO:0015405	<1e-4	sll1203	GO:0044464	<1e-4	slr1915	GO:0017111	0.0589
sll0847	GO:0048878	<1e-4	sll1509	GO:0072522	<1e-4	slr6067	GO:0016462	<1e-4	slr1150	GO:0005575	<1e-4	slr0498	GO:0017111	0.0589
sll1095	GO:0034641	<1e-4	slr1668	GO:0072528	<1e-4	sll0071	GO:0016818	<1e-4	ssl2065	GO:0044422	<1e-4	slr0948	GO:0017111	0.0589
slr0145	GO:0043412	<1e-4	sll0888	GO:0009117	<1e-4	sll0585	GO:0016462	<1e-4	ssl2971	GO:0044424	<1e-4	sll0160	GO:0017111	0.0589
slr0374	GO:0045184	<1e-4	ssr1768	GO:0022607	<1e-4	sll5061	GO:0003674	<1e-4	slr6090	GO:0005575	<1e-4	slr0269	GO:0017111	0.0589
sll5069	GO:0016054	<1e-4	sll1131	GO:0010556	<1e-4	ssr2554	GO:0015291	<1e-4	sll0101	GO:0044446	<1e-4	ssr1425	GO:0017111	0.0589
slr1056	GO:0048523	<1e-4	slr0458	GO:0072524	<1e-4	slr0981	GO:0008171	<1e-4	sll0939	GO:0044446	<1e-4	slr0181	GO:0017111	0.0589
ssr1114	GO:0009123	<1e-4	ssl5103	GO:0048523	<1e-4	sll0678	GO:0022890	<1e-4	ssl5091	GO:0043226	<1e-4	ssr3402	GO:0017111	0.0589
slr0092	GO:0006576	<1e-4	sll0314	GO:0043436	<1e-4	ssl3549	GO:0022892	<1e-4	slr1189	GO:0044444	<1e-4	sll0394	GO:0017111	0.0589
slr5111	GO:0019222	<1e-4	slr0147	GO:0051234	<1e-4	slr0243	GO:0015077	<1e-4	slr0981	GO:0044464	<1e-4	slr6028	GO:0017111	0.0589
sll1218	GO:0008150	<1e-4	ssl1923	GO:0051234	<1e-4	sll1954	GO:0016462	<1e-4	sll0406	GO:0044444	<1e-4	slr1670	GO:0017111	0.0589
slr6038	GO:0055086	<1e-4	slr1812	GO:0034660	<1e-4	slr1177	GO:0043167	<1e-4	sll1315	GO:0044422	<1e-4	sll5089	GO:0017111	0.0589
slr7015	GO:0034641	<1e-4	sll1061	GO:0043549	<1e-4	ssr2553	GO:0015405	<1e-4	sll1738	GO:0044424	<1e-4	ssl1263	GO:0017111	0.0589
ssl5114	GO:0046907	<1e-4	slr0325	GO:0006720	<1e-4	slr1927	GO:0016818	<1e-4	slr1353	GO:0043226	<1e-4	ssr0657	GO:0017111	0.0589
slr1918	GO:0009987	<1e-4	sll1949	GO:0050789	<1e-4	slr1816	GO:0022803	<1e-4	sll0602	GO:0043226	<1e-4	sll1940	GO:0017111	0.0589
ssl1046	GO:0071554	<1e-4	slr0605	GO:0006721	<1e-4	slr2018	GO:0022838	<1e-4	slr0596	GO:0005575	<1e-4	slr0521	GO:0017111	0.0589
slr0689	GO:0051716	<1e-4	slr1195	GO:0044106	<1e-4	sll1757	GO:0022857	<1e-4	ssr1765	GO:0005575	<1e-4	sll0691	GO:0017111	0.0589
slr0169	GO:0044003	<1e-4	slr0199	GO:0006732	<1e-4	sll1722	GO:0015405	<1e-4	sll0103	GO:0044422	<1e-4	slr6088	GO:0017111	0.0589
slr1177	GO:0043170	<1e-4	slr7010	GO:0051171	<1e-4	sll0283	GO:0015267	<1e-4	sll5062	GO:0044422	<1e-4	sll1640	GO:0017111	0.0589
sll7034	GO:0005996	<1e-4	sll7063	GO:0009893	<1e-4	slr1142	GO:0022836	<1e-4	sll0513	GO:0044464	<1e-4	ssr5011	GO:0017111	0.0589
ssl3142	GO:0046164	<1e-4	slr2101	GO:0043549	<1e-4	sll5033	GO:0016462	<1e-4	slr1069	GO:0044422	<1e-4	ssr5074	GO:0017111	0.0589
sll0369	GO:0034660	<1e-4	sll0931	GO:0009987	<1e-4	slr0313	GO:0015399	<1e-4	slr1778	GO:0005575	<1e-4	sll1681	GO:0017111	0.0589
sll5034	GO:0065007	<1e-4	slr1704	GO:0046907	<1e-4	slr1659	GO:0015399	<1e-4	slr0397	GO:0044446	<1e-4	slr1601	GO:0017111	0.0589
slr1047	GO:0050801	<1e-4	sll0647	GO:0010468	<1e-4	sll0328	GO:0022832	<1e-4	sll1063	GO:0044444	<1e-4	sll1285	GO:0017111	0.0589
slr1674	GO:0006811	<1e-4	slr0392	GO:0052111	<1e-4	slr1970	GO:0016741	<1e-4	slr0751	GO:0044422	<1e-4	sll1785	GO:0017111	0.0589
sll7033	GO:0009165	<1e-4	sll0925	GO:0001932	<1e-4	sll1251	GO:0022892	<1e-4	slr0980	GO:0043226	<1e-4	sll0031	GO:0017111	0.0589
ssl1378	GO:0009260	<1e-4	slr6006	GO:0009199	<1e-4	ssl2807	GO:0022857	<1e-4	slr0978	GO:0005575	<1e-4	slr1170	GO:0017111	0.0589
slr1196	GO:0051716	<1e-4	ssr2067	GO:0048518	<1e-4	sll0586	GO:0022891	<1e-4	slr0208	GO:0044444	<1e-4	slr0888	GO:0017111	0.0589
slr0291	GO:0042455	<1e-4	sll1086	GO:0009161	<1e-4	slr7083	GO:0015077	<1e-4	ssl0242	GO:0044446	<1e-4	sll0839	GO:0017111	0.0589
sll1414	GO:0051171	<1e-4	sll1396	GO:0044248	<1e-4	sll0309	GO:0022838	<1e-4	slr6044	GO:0044425	<1e-4	slr5024	GO:0017111	0.0589
slr6014	GO:0031323	<1e-4	ssr1391	GO:0009199	<1e-4	slr6016	GO:0022838	<1e-4	slr0605	GO:0005575	<1e-4	slr0967	GO:0017111	0.0589
slr0468	GO:0042455	<1e-4	slr6104	GO:0048518	<1e-4	ssl7007	GO:0022803	<1e-4	sll1160	GO:0044444	<1e-4	slr0637	GO:0017111	0.0589
slr0601	GO:0008610	<1e-4	sll7064	GO:0051246	<1e-4	ssr7084	GO:0022890	<1e-4	sll7065	GO:0044444	<1e-4	sll7069	GO:0017111	0.0589
sll0898	GO:0009987	<1e-4	ssr0109	GO:0043648	<1e-4	sll0783	GO:0016818	<1e-4	ssr2843	GO:0005575	<1e-4	sll1106	GO:0017111	0.0589
slr1122	GO:0001932	<1e-4	sll5046	GO:0044282	<1e-4	ssr1528	GO:0022890	<1e-4	sll8032	GO:0044425	<1e-4	sll0069	GO:0017111	0.0589
slr1601	GO:0009263	<1e-4	slr2004	GO:0019751	<1e-4	slr0907	GO:0022803	<1e-4	slr0232	GO:0044424	<1e-4	slr0670	GO:0017111	0.0589
sll0564	GO:0010468	<1e-4	ssr1558	GO:0044281	<1e-4	sll1151	GO:0016746	<1e-4	ssl1498	GO:0044424	<1e-4	slr5112	GO:0017111	0.0589
ssr6099	GO:0043170	<1e-4	slr0634	GO:0051716	<1e-4	slr1935	GO:0016462	<1e-4	slr0145	GO:0044444	<1e-4	ssl5068	GO:0017111	0.0589
slr7081	GO:0010468	<1e-4	slr1611	GO:0051188	<1e-4	slr1162	GO:0022890	<1e-4	sll1915	GO:0044444	<1e-4	sll0678	GO:0017111	0.0589
slr1045	GO:0065007	<1e-4	slr0610	GO:0010556	<1e-4	sll0645	GO:0043492	<1e-4	slr1385	GO:0044425	<1e-4	ssl2971	GO:0017111	0.0589
slr1807	GO:0048518	<1e-4	slr1173	GO:0051234	<1e-4	ssr1391	GO:0016817	<1e-4	slr0957	GO:0044464	<1e-4	slr0728	GO:0017111	0.0589
sll0162	GO:0019752	<1e-4	sll7064	GO:0071496	<1e-4	sll1272	GO:0015291	<1e-4	sll1247	GO:0005575	<1e-4	slr0516	GO:0017111	0.0589
sll1769	GO:0033013	<1e-4	sll1913	GO:0044106	<1e-4	ssr1407	GO:0016741	<1e-4	slr0592	GO:0044424	<1e-4	sll1570	GO:0017111	0.0589
slr1885	GO:0065007	<1e-4	sll1698	GO:0051186	<1e-4	slr0519	GO:0060089	<1e-4	slr1676	GO:0044424	<1e-4	ssl6061	GO:0017111	0.0589
slr0109	GO:0005996	<1e-4	slr1568	GO:0009142	<1e-4	sll7086	GO:0016741	<1e-4	sll1571	GO:0044424	<1e-4	ssr0336	GO:0017111	0.0589

sll0590	GO:0019752	<1e-4	sll1906	GO:0006631	<1e-4	sll1832	GO:0016746	<1e-4	sll6054	GO:0005575	<1e-4	ssl5091	GO:0017111	0.0589
slr1935	GO:0006066	<1e-4	slr1577	GO:0071840	<1e-4	ssr2553	GO:0022832	<1e-4	sll0691	GO:0005575	<1e-4	slr0709	GO:0017111	0.0589
ssl5096	GO:0042180	<1e-4	sll0479	GO:0031326	<1e-4	sll0623	GO:0016746	<1e-4	sll1608	GO:0044444	<1e-4	slr7071	GO:0017111	0.0589
sll0804	GO:0009124	<1e-4	slr0333	GO:0051179	<1e-4	sll1446	GO:0022891	<1e-4	slr1618	GO:0044446	<1e-4	ssr6089	GO:0017111	0.0589
sll1785	GO:0042180	<1e-4	slr1188	GO:0051179	<1e-4	slr6067	GO:0016741	<1e-4	sll1068	GO:0044424	<1e-4	sll1095	GO:0017111	0.0589
slr0870	GO:0048522	<1e-4	sll0188	GO:0009117	<1e-4	ssl0410	GO:0015399	<1e-4	slr1815	GO:0044425	<1e-4	sll1476	GO:0017111	0.0589
slr1809	GO:0048522	<1e-4	sll1632	GO:0009124	<1e-4	sll7031	GO:0015293	<1e-4	sll1455	GO:0044446	<1e-4	slr6091	GO:0017111	0.0589
sll7055	GO:0060255	<1e-4	sll0047	GO:0043648	<1e-4	slr0742	GO:0015293	<1e-4	sll1938	GO:0044422	<1e-4	slr0453	GO:0017111	0.0589
sll1882	GO:0009263	<1e-4	slr1648	GO:0050794	<1e-4	sll0982	GO:0043167	<1e-4	slr0692	GO:0044422	<1e-4	ssl7051	GO:0017111	0.0589
slr0392	GO:0042180	<1e-4	sll1267	GO:0008610	<1e-4	sll5089	GO:0015075	<1e-4	ssr0761	GO:0044464	<1e-4	slr0397	GO:0017111	0.0589
slr6049	GO:0019752	<1e-4	ssl0242	GO:0010556	<1e-4	slr0169	GO:0016817	<1e-4	sll5063	GO:0044425	<1e-4	sll7067	GO:0017111	0.0589
sll0266	GO:0009308	<1e-4	slr6106	GO:0043648	<1e-4	ssl5091	GO:0008171	<1e-4	sll0508	GO:0043226	<1e-4	sll7078	GO:0017111	0.0589
sll1162	GO:0009117	<1e-4	slr1128	GO:0009889	<1e-4	slr0818	GO:0015267	<1e-4	ssr6020	GO:0044424	<1e-4	ssl2162	GO:0017111	0.0589
sll1858	GO:0048518	<1e-4	sll0361	GO:0042451	<1e-4	sll0414	GO:0008171	<1e-4	slr0742	GO:0044446	<1e-4	slr0645	GO:0017111	0.0589
slr7058	GO:0009308	<1e-4	slr0784	GO:0071554	<1e-4	slr6005	GO:0015267	<1e-4	slr1704	GO:0005575	<1e-4	slr2003	GO:0017111	0.0589
slr7081	GO:0060255	<1e-4	slr6016	GO:0009057	<1e-4	slr6090	GO:0015399	<1e-4	slr1677	GO:0044464	<1e-4	sll0670	GO:0017111	0.0589
slr1353	GO:0065007	<1e-4	ssl6035	GO:0046164	<1e-4	sll0252	GO:0022836	<1e-4	sll6053	GO:0044444	<1e-4	slr5101	GO:0017111	0.0589
sll0174	GO:0051347	<1e-4	ssr5106	GO:0043933	<1e-4	slr0305	GO:0015399	<1e-4	slr0388	GO:0044425	<1e-4	slr0313	GO:0017111	0.0589
slr1415	GO:0046128	<1e-4	sll1071	GO:0051818	<1e-4	sll0780	GO:0022890	<1e-4	sll8007	GO:0044446	<1e-4	sll1131	GO:0017111	0.0589
sll0283	GO:0051716	<1e-4	slr6071	GO:0009991	<1e-4	sll0811	GO:0015291	<1e-4	sll0359	GO:0044464	<1e-4	sll1321	GO:0017111	0.0589
slr0103	GO:0019219	<1e-4	slr6049	GO:0051179	<1e-4	sll1442	GO:0003674	<1e-4	sll0488	GO:0043226	<1e-4	sll1254	GO:0017111	0.0589
sll0023	GO:0006721	<1e-4	slr0602	GO:0019751	<1e-4	slr1484	GO:0022832	<1e-4	ssl2148	GO:0005575	<1e-4	ssr3129	GO:0017111	0.0589
sll1965	GO:0006066	<1e-4	slr7011	GO:0046483	<1e-4	slr1613	GO:0060089	<1e-4	slr0780	GO:0044444	<1e-4	slr1474	GO:0017111	0.0589
slr1053	GO:0072528	<1e-4	sll1267	GO:0006793	<1e-4	sll1751	GO:0016818	<1e-4	sll1763	GO:0005575	<1e-4	slr5126	GO:0017111	0.0589
slr0049	GO:0072524	<1e-4	sll0066	GO:0006631	<1e-4	ssr1407	GO:0016746	<1e-4	ssl1972	GO:0044425	<1e-4	sll0372	GO:0017111	0.0589
ssr6062	GO:0043933	<1e-4	slr0250	GO:0052111	<1e-4	ssl5031	GO:0015077	<1e-4	slr1999	GO:0044425	<1e-4	sll0765	GO:0017111	0.0589
sll0871	GO:0043933	<1e-4	sll0930	GO:0006811	<1e-4	sll1089	GO:0008171	<1e-4	sll1696	GO:0005575	<1e-4	sll0230	GO:0017111	0.0589
sll1060	GO:0044003	<1e-4	sll1738	GO:0009117	<1e-4	sll1752	GO:0016818	<1e-4	sll0103	GO:0005575	<1e-4	slr0039	GO:0017111	0.0589
slr1998	GO:0009308	<1e-4	sll1388	GO:0034660	<1e-4	sll0446	GO:0060089	<1e-4	ssr0102	GO:0005575	<1e-4	ssr6002	GO:0017111	0.0589
slr1778	GO:0010556	<1e-4	slr1864	GO:0009260	<1e-4	sll0614	GO:0043492	<1e-4	slr1162	GO:0044446	<1e-4	slr1327	GO:0017111	0.0589
ssl5103	GO:0072524	<1e-4	slr0169	GO:0045184	<1e-4	slr1566	GO:0042623	<1e-4	slr7082	GO:0005575	<1e-4	slr8022	GO:0017111	0.0589
slr1519	GO:0060255	<1e-4	sll1217	GO:0051234	<1e-4	slr1276	GO:0042623	<1e-4	sll1500	GO:0044425	<1e-4	slr5073	GO:0017111	0.0589
sgl0002	GO:0042180	<1e-4	slr0480	GO:0006576	<1e-4	sll1352	GO:0060089	<1e-4	sll1510	GO:0044444	<1e-4	slr1875	GO:0017111	0.0589
ssl2814	GO:0048519	<1e-4	sll5030	GO:0044275	<1e-4	slr1235	GO:0043167	<1e-4	slr6064	GO:0005575	<1e-4	slr0270	GO:0017111	0.0589
sll1307	GO:0009126	<1e-4	ssr2848	GO:0009141	<1e-4	slr0610	GO:0008171	<1e-4	slr1533	GO:0043226	<1e-4	slr1807	GO:0017111	0.0589
sll0048	GO:0006733	<1e-4	slr7059	GO:0016054	<1e-4	sll1004	GO:0015399	<1e-4	slr0592	GO:0044422	<1e-4	slr1866	GO:0003723	0.0584
slr1290	GO:0034654	<1e-4	sll5034	GO:0006793	<1e-4	ssl1918	GO:0016818	<1e-4	slr0076	GO:0043226	<1e-4	sll0148	GO:0003723	0.0584
sll1563	GO:0009889	<1e-4	sll7089	GO:0060255	<1e-4	ssr3300	GO:0008171	<1e-4	sll1024	GO:0005575	<1e-4	sll0274	GO:0003723	0.0584
sll7050	GO:0050789	<1e-4	sll0585	GO:0044255	<1e-4	slr0092	GO:0016818	<1e-4	slr1591	GO:0044444	<1e-4	slr1066	GO:0003723	0.0584
ssr2551	GO:0009987	<1e-4	slr5017	GO:0072527	<1e-4	sll0443	GO:0022836	<1e-4	ssl7021	GO:0044424	<1e-4	sll0096	GO:0003723	0.0584
ssr3154	GO:0051188	<1e-4	slr0692	GO:0034641	<1e-4	slr0440	GO:0015399	<1e-4	slr1097	GO:0005575	<1e-4	slr1519	GO:0003723	0.0584
slr6075	GO:0044260	<1e-4	sll0381	GO:0006091	<1e-4	slr2012	GO:0022832	<1e-4	slr5101	GO:0044444	<1e-4	sll1426	GO:0003723	0.0584
slr1507	GO:0006766	<1e-4	slr1045	GO:0048519	<1e-4	sll5130	GO:0015291	<1e-4	slr0050	GO:0044424	<1e-4	sll0547	GO:0003723	0.0584
slr1762	GO:0072524	<1e-4	sll5128	GO:0044248	<1e-4	ssl3142	GO:0016746	<1e-4	slr0712	GO:0043226	<1e-4	slr1900	GO:0003723	0.0584
slr0196	GO:0009889	<1e-4	sll0656	GO:0042180	<1e-4	ssl1377	GO:0022838	<1e-4	sll1424	GO:0044425	<1e-4	slr0209	GO:0003723	0.0584
ssr2975	GO:0010556	<1e-4	sll5097	GO:0044260	<1e-4	sll1380	GO:0003674	<1e-4	ssr0759	GO:0044444	<1e-4	sll0499	GO:0003723	0.0584
slr0442	GO:0022411	<1e-4	sll1784	GO:0042455	<1e-4	sll7043	GO:0022891	<1e-4	slr1150	GO:0044444	<1e-4	slr1918	GO:0003723	0.0584
ssl5064	GO:0022411	<1e-4	sll0783	GO:0051188	<1e-4	sll1696	GO:0016462	<1e-4	sll0060	GO:0044464	<1e-4	sll0854	GO:0003723	0.0584
sll0863	GO:0044106	<1e-4	slr6007	GO:0046164	<1e-4	slr1600	GO:0015077	<1e-4	slr0196	GO:0005575	<1e-4	sll2006	GO:0003723	0.0584
slr0039	GO:0043648	<1e-4	sll0309	GO:0009057	<1e-4	slr2111	GO:0015077	<1e-4	slr6038	GO:0044444	<1e-4	sll1455	GO:0003723	0.0584
slr1541	GO:0019637	<1e-4	slr0476	GO:0009057	<1e-4	slr1611	GO:0016817	<1e-4	ssr6085	GO:0044444	<1e-4	slr1025	GO:0003723	0.0584
slr1415	GO:0019637	<1e-4	slr2025	GO:0010468	<1e-4	slr0304	GO:0015267	<1e-4	ssl2595	GO:0044425	<1e-4	sll0360	GO:0003723	0.0584
slr1161	GO:0009394	<1e-4	slr1774	GO:0009199	<1e-4	slr0592	GO:0015293	<1e-4	slr1493	GO:0005575	<1e-4	ssr1375	GO:0003723	0.0584
slr0667	GO:0034654	<1e-4	sll5130	GO:0051188	<1e-4	slr0708	GO:0015077	<1e-4	slr0038	GO:0044446	<1e-4	sll1942	GO:0003723	0.0584

slr1624	GO:0051246	<1e-4	sll1068	GO:0009259	<1e-4	ssl2733	GO:0022857	<1e-4	slr8044	GO:0005575	<1e-4	ssl0109	GO:0003723	0.0584
slr1222	GO:0009126	<1e-4	slr0935	GO:0009262	<1e-4	slr1025	GO:0015291	<1e-4	sll0872	GO:0005575	<1e-4	slr1612	GO:0003723	0.0584
slr0147	GO:0009150	<1e-4	sll1089	GO:0009260	<1e-4	sll5128	GO:0016741	<1e-4	slr6103	GO:0005575	<1e-4	sll1586	GO:0003723	0.0584
ssr5011	GO:0044281	<1e-4	slr1681	GO:0048878	<1e-4	ssr1558	GO:0015405	<1e-4	slr1241	GO:0005575	<1e-4	slr1083	GO:0003723	0.0584
sll1722	GO:0046128	<1e-4	sll0552	GO:0006066	<1e-4	sll0447	GO:0015293	<1e-4	slr1168	GO:0044444	<1e-4	sll1219	GO:0003723	0.0584
slr1363	GO:0042451	<1e-4	sll0676	GO:0008610	<1e-4	sll1509	GO:0022838	<1e-4	slr1417	GO:0043226	<1e-4	slr1557	GO:0003723	0.0584
sll1350	GO:0051818	<1e-4	slr1266	GO:0046128	<1e-4	slr0821	GO:0060089	<1e-4	sll1469	GO:0005575	<1e-4	sll1934	GO:0003723	0.0584
ssl3615	GO:0019751	<1e-4	slr1809	GO:0046394	<1e-4	sll1722	GO:0005342	<1e-4	slr1162	GO:0044425	<1e-4	slr1391	GO:0003723	0.0584
sll1142	GO:0006720	<1e-4	sll0740	GO:0046164	<1e-4	slr5102	GO:0016746	<1e-4	sll0447	GO:0044422	<1e-4	slr1084	GO:0004871	0.0582
sll0397	GO:0034660	<1e-4	sll0788	GO:0045184	<1e-4	slr0680	GO:0015399	<1e-4	sll0281	GO:0044422	<1e-4	sll0381	GO:0004871	0.0582
slr1674	GO:0006631	<1e-4	sll0176	GO:0051171	<1e-4	slr1032	GO:0022890	<1e-4	sll1939	GO:0044464	<1e-4	sll0982	GO:0004871	0.0582
slr1276	GO:0044255	<1e-4	slr5023	GO:0048523	<1e-4	sll1151	GO:0022857	<1e-4	ssr1951	GO:0044464	<1e-4	slr2011	GO:0004871	0.0582
sll0590	GO:0009141	<1e-4	slr2103	GO:0034641	<1e-4	sll0803	GO:0060089	<1e-4	sll1214	GO:0044424	<1e-4	sll1965	GO:0004871	0.0582
slr2101	GO:0072528	<1e-4	sll8011	GO:0006811	<1e-4	sgl0001	GO:0008171	<1e-4	slr0954	GO:0043226	<1e-4	slr1913	GO:0004871	0.0582
slr7092	GO:0048523	<1e-4	sll1151	GO:0009260	<1e-4	sll1873	GO:0016817	<1e-4	sll1660	GO:0044422	<1e-4	slr0482	GO:0004871	0.0582
slr0264	GO:0022607	<1e-4	sll1021	GO:0031326	<1e-4	sll1495	GO:0022890	<1e-4	sll0811	GO:0005575	<1e-4	slr2122	GO:0004871	0.0582
ssl7046	GO:0022607	<1e-4	sll0985	GO:0044281	<1e-4	sll0676	GO:0022836	<1e-4	sll1770	GO:0044444	<1e-4	slr1203	GO:0004871	0.0582
ssl2138	GO:0046394	<1e-4	slr1819	GO:0046164	<1e-4	slr0356	GO:0043492	<1e-4	slr1035	GO:0044464	<1e-4	sll1954	GO:0004871	0.0582
slr1116	GO:0006721	<1e-4	slr6090	GO:0046164	<1e-4	slr1599	GO:0015075	<1e-4	sll0847	GO:0044422	<1e-4	slr1073	GO:0004871	0.0582
ssl1923	GO:0072524	<1e-4	sll0481	GO:0006631	<1e-4	slr5023	GO:0015291	<1e-4	sll0740	GO:0044422	<1e-4	slr1177	GO:0004871	0.0582
sll1132	GO:0048519	<1e-4	slr1117	GO:0042180	<1e-4	sll0445	GO:0004872	<1e-4	slr1084	GO:0005575	<1e-4	ssr3122	GO:0004871	0.0582
ssl6061	GO:0042455	<1e-4	ssr2803	GO:0043412	<1e-4	sll1925	GO:0015291	<1e-4	slr1788	GO:0044425	<1e-4	sll1315	GO:0004871	0.0582
slr0299	GO:0018130	<1e-4	sll1757	GO:0080090	<1e-4	ssr2553	GO:0022891	<1e-4	sll1769	GO:0005575	<1e-4	slr0913	GO:0004871	0.0582
sll1882	GO:0018130	<1e-4	sll1396	GO:0052111	<1e-4	sll0552	GO:0016741	<1e-4	sll0743	GO:0005575	<1e-4	slr0845	GO:0004871	0.0582
slr6073	GO:0009059	<1e-4	slr0709	GO:0009059	<1e-4	sll7089	GO:0022890	<1e-4	ssr1698	GO:0005575	<1e-4	slr0723	GO:0004871	0.0582
ssr5121	GO:0042180	<1e-4	slr1442	GO:0009892	<1e-4	sll1217	GO:0016741	<1e-4	sll1532	GO:0044424	<1e-4	slr1638	GO:0004871	0.0582
sll0931	GO:0006631	<1e-4	sll0382	GO:0006631	<1e-4	ssr1473	GO:0015267	<1e-4	sll0372	GO:0043226	<1e-4	sll0676	GO:0004871	0.0582
slr1866	GO:0051716	<1e-4	slr7025	GO:0009259	<1e-4	sll8033	GO:0016741	<1e-4	slr1668	GO:0044425	<1e-4	slr1618	GO:0004871	0.0582
sll8007	GO:0044106	<1e-4	slr0962	GO:0034641	<1e-4	ssr2317	GO:0015077	<1e-4	sll8004	GO:0044444	<1e-4	sll0237	GO:0004871	0.0582
slr0581	GO:0071842	<1e-4	sll6052	GO:0005996	<1e-4	slr1753	GO:0015293	<1e-4	slr0689	GO:0043226	<1e-4	slr1964	GO:0004871	0.0582
sll5062	GO:0042451	<1e-4	ssr7035	GO:0006576	<1e-4	ssl8008	GO:0043492	<1e-4	sll0022	GO:0044444	<1e-4	ssr2439	GO:0004871	0.0582
slr1866	GO:0016052	<1e-4	sll0172	GO:0048878	<1e-4	slr1315	GO:0015075	<1e-4	slr1495	GO:0044424	<1e-4	sll0944	GO:0004871	0.0582
slr1813	GO:0048518	<1e-4	sll0405	GO:0044106	<1e-4	slr0695	GO:0016817	<1e-4	ssl7007	GO:0044422	<1e-4	slr1854	GO:0004871	0.0582
sll0703	GO:0046164	<1e-4	sll0160	GO:0051234	<1e-4	sll0577	GO:0060089	<1e-4	sll1191	GO:0044464	<1e-4	slr1572	GO:0004871	0.0582
ssl8028	GO:0009123	<1e-4	sll1939	GO:0046128	<1e-4	slr5087	GO:0003674	<1e-4	sll1214	GO:0044446	<1e-4	slr0192	GO:0004871	0.0582
ssl5015	GO:0034654	<1e-4	slr0729	GO:0009056	<1e-4	sll0044	GO:0043167	<1e-4	sll0545	GO:0044424	<1e-4	sll0355	GO:0004871	0.0582
sll7062	GO:0009394	<1e-4	slr0157	GO:0044248	<1e-4	slr1070	GO:0005342	<1e-4	sll1095	GO:0005575	<1e-4	slr0586	GO:0004871	0.0582
slr2092	GO:0006720	<1e-4	slr5017	GO:0042180	<1e-4	sll0325	GO:0022891	<1e-4	sll5067	GO:0005575	<1e-4	slr0386	GO:0004871	0.0582
slr0579	GO:0006733	<1e-4	slr6091	GO:0046164	<1e-4	slr0431	GO:0005342	<1e-4	slr0702	GO:0044425	<1e-4	sll0532	GO:0004871	0.0582
sll1722	GO:0050794	<1e-4	slr1612	GO:0071842	<1e-4	slr1619	GO:0022892	<1e-4	sll1834	GO:0044446	<1e-4	ssr3571	GO:0004871	0.0582
ssl7042	GO:0001932	<1e-4	sll1476	GO:0031323	<1e-4	sll0249	GO:0022891	<1e-4	ssr6032	GO:0044464	<1e-4	sll0325	GO:0004871	0.0582
sll7064	GO:0016052	<1e-4	slr0482	GO:0044271	<1e-4	sll0284	GO:0022892	<1e-4	slr0496	GO:0044464	<1e-4	sll1658	GO:0004871	0.0582
sll1060	GO:0044271	<1e-4	sll1956	GO:0009141	<1e-4	sll5030	GO:0015293	<1e-4	ssr2067	GO:0044464	<1e-4	slr1230	GO:0004871	0.0582
sll0062	GO:0050896	<1e-4	slr5023	GO:0044260	<1e-4	slr0588	GO:0022891	<1e-4	sll0853	GO:0044444	<1e-4	slr1726	GO:0003723	0.0568
slr0582	GO:0044281	<1e-4	sll1135	GO:0048878	<1e-4	sll7065	GO:0016818	<1e-4	slr0848	GO:0044446	<1e-4	slr0514	GO:0003723	0.0568
ssl2064	GO:0043436	<1e-4	slr1187	GO:0034641	<1e-4	sll1573	GO:0042623	<1e-4	sll1532	GO:0043226	<1e-4	slr0496	GO:0003723	0.0568
sll6053	GO:0010556	<1e-4	slr6049	GO:0019637	<1e-4	sll5090	GO:0003674	<1e-4	ssl3451	GO:0005575	<1e-4	sll0192	GO:0003723	0.0568
slr0272	GO:0010468	<1e-4	sll0488	GO:0009150	<1e-4	slr1875	GO:0022892	<1e-4	slr1681	GO:0044444	<1e-4	sll0822	GO:0003723	0.0568
slr1600	GO:0051246	<1e-4	sll0595	GO:0009142	<1e-4	sll7086	GO:0015267	<1e-4	slr0885	GO:0044464	<1e-4	slr0740	GO:0003723	0.0568
sll0518	GO:0050896	<1e-4	slr0111	GO:0006732	<1e-4	slr0334	GO:0022838	<1e-4	slr0407	GO:0044424	<1e-4	slr0263	GO:0003723	0.0568
slr0551	GO:0071840	<1e-4	sll0909	GO:0009308	<1e-4	slr2144	GO:0016817	<1e-4	sll0858	GO:0044464	<1e-4	slr1098	GO:0003723	0.0568
sll0678	GO:0009165	<1e-4	sll8004	GO:0055086	<1e-4	slr0594	GO:0015077	<1e-4	sll1911	GO:0044444	<1e-4	sll0577	GO:0003723	0.0568
slr2103	GO:0006631	<1e-4	slr1047	GO:0046164	<1e-4	sll1773	GO:0022891	<1e-4	sll0176	GO:0044464	<1e-4	slr0589	GO:0003723	0.0568
slr7092	GO:0009262	<1e-4	sll0249	GO:0051179	<1e-4	ssr1558	GO:0015399	<1e-4	sll0853	GO:0044446	<1e-4	sll0175	GO:0003723	0.0568

sll0066	GO:0009893	<1e-4	slr7037	GO:0046164	<1e-4	sll1464	GO:0003674	<1e-4	sll0443	GO:0044444	<1e-4	slr1811	GO:0003723	0.0568
sll1939	GO:0048878	<1e-4	sll0595	GO:0009892	<1e-4	sll7028	GO:0022803	<1e-4	sll1862	GO:0044424	<1e-4	slr1143	GO:0003723	0.0568
slr0380	GO:0009991	<1e-4	sll1191	GO:0009987	<1e-4	slr1809	GO:0016818	<1e-4	sll1373	GO:0044422	<1e-4	slr1215	GO:0009058	0.0562
slr0358	GO:0006082	<1e-4	slr1261	GO:0050794	<1e-4	slr1896	GO:0015075	<1e-4	ssl3692	GO:0044425	<1e-4	sll0252	GO:0016787	0.0562
sll1832	GO:0006811	<1e-4	slr0496	GO:0065007	<1e-4	slr1546	GO:0022832	<1e-4	ssl1520	GO:0044422	<1e-4	slr1644	GO:0017111	0.0554
sll5033	GO:0006732	<1e-4	slr0313	GO:0050896	<1e-4	sll0372	GO:0015293	<1e-4	sll6053	GO:0005575	<1e-4	slr1431	GO:0017111	0.0554
sll0473	GO:0009142	<1e-4	sll1265	GO:0048522	<1e-4	ssl1377	GO:0015291	<1e-4	ssl1923	GO:0044424	<1e-4	ssr2142	GO:0017111	0.0554
slr1406	GO:0022411	<1e-4	sll1486	GO:0006766	<1e-4	sll0369	GO:0015399	<1e-4	slr2037	GO:0005575	<1e-4	sll0933	GO:0003676	0.0553
slr2103	GO:0051347	<1e-4	slr1474	GO:0051188	<1e-4	slr6029	GO:0016818	<1e-4	sll0810	GO:0044464	<1e-4	slr0588	GO:0009058	0.0547
sll0314	GO:0031323	<1e-4	sll0241	GO:0042451	<1e-4	sll0984	GO:0015077	<1e-4	ssl2162	GO:0005575	<1e-4	slr0285	GO:0009058	0.0547
slr6051	GO:0051347	<1e-4	slr1472	GO:0019637	<1e-4	sll1049	GO:0015291	<1e-4	sll8035	GO:0044444	<1e-4	sll0736	GO:0009058	0.0547
slr0172	GO:0019752	<1e-4	slr5118	GO:0065007	<1e-4	sll0743	GO:0015399	<1e-4	slr1815	GO:0044464	<1e-4	sll0372	GO:0009058	0.0547
sll5030	GO:0050801	<1e-4	slr1449	GO:0009141	<1e-4	slr1025	GO:0015267	<1e-4	sll1225	GO:0044464	<1e-4	ssr0109	GO:0009058	0.0547
slr1236	GO:0018130	<1e-4	slr0607	GO:0046128	<1e-4	slr0172	GO:0022891	<1e-4	slr1183	GO:0005575	<1e-4	sll1926	GO:0009058	0.0547
sll1372	GO:0006766	<1e-4	ssr2843	GO:0034641	<1e-4	ssr2067	GO:0005342	<1e-4	slr5013	GO:0044444	<1e-4	slr0269	GO:0009058	0.0547
sll0545	GO:0033013	<1e-4	slr0930	GO:0051246	<1e-4	slr2115	GO:0016462	<1e-4	sll5044	GO:0044446	<1e-4	sll0608	GO:0009058	0.0547
slr1495	GO:0042180	<1e-4	slr1819	GO:0051188	<1e-4	slr1470	GO:0015267	<1e-4	sll0266	GO:0044424	<1e-4	sll0905	GO:0009058	0.0547
sll1511	GO:0009893	<1e-4	sll1344	GO:0055082	<1e-4	sll0980	GO:0022891	<1e-4	slr1789	GO:0044424	<1e-4	sll0858	GO:0009058	0.0547
slr1923	GO:0051716	<1e-4	sll0192	GO:0044106	<1e-4	sll0702	GO:0060089	<1e-4	sll1160	GO:0043226	<1e-4	slr1438	GO:0009058	0.0547
sll6054	GO:0009165	<1e-4	sll0265	GO:0019222	<1e-4	ssl5007	GO:0016746	<1e-4	sll1495	GO:0005575	<1e-4	slr1935	GO:0009058	0.0547
ssr2142	GO:0005976	<1e-4	slr0594	GO:0043549	<1e-4	sll0867	GO:0005342	<1e-4	slr6068	GO:0043226	<1e-4	slr7091	GO:0009058	0.0547
slr0149	GO:0009263	<1e-4	slr1613	GO:0006576	<1e-4	slr1505	GO:0042623	<1e-4	slr0634	GO:0005575	<1e-4	sll0563	GO:0009058	0.0547
sll0023	GO:0009132	<1e-4	slr1053	GO:0048878	<1e-4	slr0667	GO:0043492	<1e-4	ssl1552	GO:0005575	<1e-4	sll0625	GO:0009058	0.0547
sll1262	GO:0018130	<1e-4	slr1572	GO:0044003	<1e-4	slr0291	GO:0022838	<1e-4	slr1816	GO:0044446	<1e-4	slr0725	GO:0009058	0.0547
ssl0353	GO:0006720	<1e-4	ssr5120	GO:0009126	<1e-4	ssr2060	GO:0015293	<1e-4	sll1884	GO:0043226	<1e-4	sll0069	GO:0009058	0.0547
slr7060	GO:0006793	<1e-4	sll1586	GO:0009057	<1e-4	slr0976	GO:0016741	<1e-4	ssl1263	GO:0005575	<1e-4	sll1692	GO:0009058	0.0547
slr0345	GO:0048523	<1e-4	slr0871	GO:0008150	<1e-4	sll0141	GO:0022832	<1e-4	slr0582	GO:0044444	<1e-4	sll0482	GO:0009058	0.0547
sll0804	GO:0051716	<1e-4	sll1135	GO:0009260	<1e-4	slr1534	GO:0022832	<1e-4	sll7031	GO:0005575	<1e-4	sll1570	GO:0009058	0.0547
slr0912	GO:0009142	<1e-4	ssr6086	GO:0009056	<1e-4	ssl1972	GO:0022838	<1e-4	slr7081	GO:0044424	<1e-4	slr1624	GO:0009058	0.0547
sll0762	GO:0009124	<1e-4	slr5024	GO:0016054	<1e-4	sll1372	GO:0060089	<1e-4	slr0702	GO:0044464	<1e-4	slr0013	GO:0009058	0.0547
sll1389	GO:0006793	<1e-4	slr1752	GO:0006811	<1e-4	slr0545	GO:0016746	<1e-4	slr2052	GO:0044464	<1e-4	sll1698	GO:0009058	0.0547
sll1004	GO:0006811	<1e-4	sll7034	GO:0006082	<1e-4	slr0199	GO:0042623	<1e-4	slr0250	GO:0005575	<1e-4	ssl2384	GO:0009058	0.0547
slr1570	GO:0031326	<1e-4	slr1790	GO:0031323	<1e-4	sll1715	GO:0005342	<1e-4	slr1052	GO:0044446	<1e-4	sll8035	GO:0009058	0.0547
slr0971	GO:0051818	<1e-4	sll1632	GO:0042180	<1e-4	sll1344	GO:0022838	<1e-4	sll1530	GO:0005575	<1e-4	ssl5091	GO:0009058	0.0547
slr1415	GO:0046394	<1e-4	sll0479	GO:0019219	<1e-4	sll0909	GO:0015405	<1e-4	slr1599	GO:0044424	<1e-4	slr0334	GO:0009058	0.0547
slr2084	GO:0044275	<1e-4	ssl1552	GO:0043933	<1e-4	sll0412	GO:0015405	<1e-4	sll1415	GO:0044424	<1e-4	ssr1558	GO:0009058	0.0547
sll0645	GO:0046483	<1e-4	slr0243	GO:0016052	<1e-4	slr6067	GO:0022832	<1e-4	sll5026	GO:0044424	<1e-4	sll0678	GO:0009058	0.0547
sll1530	GO:0009987	<1e-4	sll1232	GO:0009199	<1e-4	slr1468	GO:0015405	<1e-4	slr1270	GO:0043226	<1e-4	ssl1378	GO:0009058	0.0547
sll0913	GO:0072521	<1e-4	sll1068	GO:0044255	<1e-4	sll0269	GO:0022891	<1e-4	ssl5098	GO:0044425	<1e-4	slr1807	GO:0009058	0.0547
slr1436	GO:0072524	<1e-4	slr8044	GO:0044281	<1e-4	slr1544	GO:0022892	<1e-4	slr6013	GO:0043226	<1e-4	sll7086	GO:0009058	0.0547
slr1644	GO:0031323	<1e-4	slr0196	GO:0009150	<1e-4	slr1790	GO:0022890	<1e-4	ssl0832	GO:0044424	<1e-4	slr1056	GO:0009058	0.0547
slr1900	GO:0009991	<1e-4	ssr2975	GO:0006220	<1e-4	sll0449	GO:0043492	<1e-4	ssl0483	GO:0005575	<1e-4	ssl1918	GO:0009058	0.0547
slr0554	GO:0044255	<1e-4	sll1761	GO:0090304	<1e-4	sll0237	GO:0022832	<1e-4	slr1915	GO:0043226	<1e-4	ssr6099	GO:0009058	0.0547
slr0380	GO:0018130	<1e-4	slr1726	GO:0009142	<1e-4	sll1571	GO:0043167	<1e-4	slr0668	GO:0005575	<1e-4	sll0062	GO:0009058	0.0547
slr0243	GO:0019362	<1e-4	sll1495	GO:0071840	<1e-4	sll0565	GO:0043492	<1e-4	slr0031	GO:0044444	<1e-4	slr0582	GO:0009058	0.0547
slr1068	GO:0006766	<1e-4	sll1505	GO:0019751	<1e-4	sll0044	GO:0043492	<1e-4	slr2048	GO:0044444	<1e-4	slr0863	GO:0009058	0.0547
slr1196	GO:0042455	<1e-4	sll0980	GO:0016053	<1e-4	sll1691	GO:0043167	<1e-4	sll0436	GO:0044425	<1e-4	slr1789	GO:0009058	0.0547
sll1446	GO:0044282	<1e-4	sll0586	GO:0009142	<1e-4	slr1951	GO:0016462	<1e-4	ssr1114	GO:0005575	<1e-4	slr0948	GO:0009058	0.0547
slr0712	GO:0009141	<1e-4	slr0709	GO:0008610	<1e-4	slr0709	GO:0022857	<1e-4	sll1267	GO:0044446	<1e-4	sll1321	GO:0009058	0.0547
slr0930	GO:0008150	<1e-4	slr1800	GO:0019752	<1e-4	slr0919	GO:0022832	<1e-4	sll0309	GO:0044425	<1e-4	slr0397	GO:0009058	0.0547
ssl2920	GO:0009124	<1e-4	sll1586	GO:0051171	<1e-4	sll0839	GO:0043167	<1e-4	slr1152	GO:0044444	<1e-4	sll7031	GO:0009058	0.0547
sll1671	GO:0034654	<1e-4	sll7066	GO:0046128	<1e-4	sll0376	GO:0016462	<1e-4	slr1174	GO:0043226	<1e-4	slr0416	GO:0009058	0.0547
sll5030	GO:0051186	<1e-4	slr6039	GO:0046128	<1e-4	sll1912	GO:0015267	<1e-4	ssr6078	GO:0044464	<1e-4	sll0839	GO:0009058	0.0547
ssr3154	GO:0044260	<1e-4	sll0590	GO:0009892	<1e-4	slr1702	GO:0043167	<1e-4	ssr3304	GO:0044425	<1e-4	slr1537	GO:0009058	0.0547

sll1219	GO:0042180	<1e-4	slr6038	GO:0009987	<1e-4	slr1425	GO:0022892	<1e-4	ssr6026	GO:0005575	<1e-4	sll0252	GO:0005215	0.053
slr1970	GO:0009144	<1e-4	sll1692	GO:0042451	<1e-4	sll1411	GO:0015399	<1e-4	slr0625	GO:0005575	<1e-4	ssr1375	GO:0004871	0.0529
ssr1425	GO:0006576	<1e-4	slr0664	GO:0072521	<1e-4	sll1681	GO:0022803	<1e-4	slr0658	GO:0044424	<1e-4	sll1586	GO:0004871	0.0529
sll0444	GO:0009057	<1e-4	sll1573	GO:0009141	<1e-4	slr2000	GO:0016462	<1e-4	ssl2749	GO:0044422	<1e-4	slr0876	GO:0004871	0.0529
slr1535	GO:0009150	<1e-4	slr1854	GO:0006631	<1e-4	ssl7051	GO:0016741	<1e-4	sll7075	GO:0044422	<1e-4	slr1127	GO:0004871	0.0529
sll0765	GO:0005996	<1e-4	slr7083	GO:0009987	<1e-4	slr1236	GO:0016741	<1e-4	slr0249	GO:0044425	<1e-4	slr0503	GO:0004871	0.0529
slr1189	GO:0051186	<1e-4	sll1307	GO:0071841	<1e-4	sll0525	GO:0043492	<1e-4	sll0419	GO:0044422	<1e-4	ssr1698	GO:0004871	0.0529
slr0455	GO:0006720	<1e-4	sll5090	GO:0010556	<1e-4	ssl0787	GO:0022890	<1e-4	ssr0109	GO:0043226	<1e-4	sll2006	GO:0004871	0.0529
sll1252	GO:0043549	<1e-4	slr0668	GO:0009309	<1e-4	slr7016	GO:0016741	<1e-4	slr0442	GO:0043226	<1e-4	sll1218	GO:0004871	0.0529
slr1032	GO:0072528	<1e-4	sll1359	GO:0044255	<1e-4	slr1385	GO:0043167	<1e-4	slr0598	GO:0044422	<1e-4	sll0301	GO:0004871	0.0529
sll0678	GO:0051246	<1e-4	slr1788	GO:0009056	<1e-4	ssl3692	GO:0022838	<1e-4	slr6067	GO:0044446	<1e-4	slr1519	GO:0004871	0.0529
slr1670	GO:0018130	<1e-4	slr0147	GO:0048522	<1e-4	slr6008	GO:0022832	<1e-4	ssr2787	GO:0044424	<1e-4	sll0508	GO:0004871	0.0529
sll1504	GO:0022411	<1e-4	slr0195	GO:0009987	<1e-4	slr1807	GO:0022803	<1e-4	slr1780	GO:0005575	<1e-4	sll1433	GO:0004871	0.0529
sll1979	GO:0006721	<1e-4	sll0328	GO:0042451	<1e-4	sll1735	GO:0016746	<1e-4	slr1840	GO:0044444	<1e-4	slr2025	GO:0004871	0.0529
sll0198	GO:0072522	<1e-4	sll1884	GO:0009123	<1e-4	slr1102	GO:0005342	<1e-4	slr0887	GO:0044422	<1e-4	slr1353	GO:0004871	0.0529
slr1863	GO:0060255	<1e-4	ssl2717	GO:0009308	<1e-4	slr0317	GO:0008171	<1e-4	slr7099	GO:0044425	<1e-4	slr1107	GO:0004871	0.0529
slr1173	GO:0009262	<1e-4	slr7097	GO:0051347	<1e-4	slr2110	GO:0016817	<1e-4	sll1273	GO:0044464	<1e-4	sll0743	GO:0004871	0.0529
sll5109	GO:0006631	<1e-4	sll5109	GO:0071842	<1e-4	ssl7046	GO:0015405	<1e-4	sll1119	GO:0044464	<1e-4	sll0096	GO:0004871	0.0529
sll1272	GO:0009263	<1e-4	slr1657	GO:0044275	<1e-4	sll0933	GO:0016817	<1e-4	slr0637	GO:0005575	<1e-4	slr1045	GO:0004871	0.0529
slr0195	GO:0044281	<1e-4	slr1611	GO:0050896	<1e-4	slr2110	GO:0022838	<1e-4	sll0735	GO:0005575	<1e-4	sll0447	GO:0004871	0.0529
slr1070	GO:0009142	<1e-4	sll1036	GO:0009132	<1e-4	slr1104	GO:0016818	<1e-4	slr1618	GO:0005575	<1e-4	slr1025	GO:0004871	0.0529
slr0300	GO:0055082	<1e-4	slr6071	GO:0019222	<1e-4	sll0294	GO:0043492	<1e-4	sll8001	GO:0044444	<1e-4	slr1557	GO:0004871	0.0529
sll0691	GO:0048518	<1e-4	ssr1425	GO:0010468	<1e-4	slr1702	GO:0022838	<1e-4	ssl2064	GO:0044464	<1e-4	slr1194	GO:0004871	0.0529
slr0610	GO:0045184	<1e-4	slr0423	GO:0046907	<1e-4	slr1102	GO:0015291	<1e-4	slr1045	GO:0044425	<1e-4	slr1612	GO:0004871	0.0529
sll0096	GO:0001932	<1e-4	sll0423	GO:0044282	<1e-4	ssr2067	GO:0015399	<1e-4	slr0207	GO:0044446	<1e-4	sll0445	GO:0017111	0.0527
slr0264	GO:0044255	<1e-4	slr1944	GO:0009889	<1e-4	sll1071	GO:0015293	<1e-4	sll0413	GO:0043226	<1e-4	sll0641	GO:0017111	0.0527
slr0980	GO:0009259	<1e-4	ssl2733	GO:0044275	<1e-4	slr0108	GO:0015293	<1e-4	slr0287	GO:0044464	<1e-4	sll0985	GO:0016787	0.0526
ssr0332	GO:0009165	<1e-4	ssl3829	GO:0042180	<1e-4	slr0941	GO:0008171	<1e-4	ssr5120	GO:0044446	<1e-4	sll0269	GO:0003723	0.0508
sll1389	GO:0034641	<1e-4	sll1241	GO:0019752	<1e-4	sll1272	GO:0043167	<1e-4	slr1667	GO:0043226	<1e-4	sll0702	GO:0003723	0.0508
ssr1768	GO:0044271	<1e-4	slr1104	GO:0022607	<1e-4	slr1174	GO:0015293	<1e-4	sll1891	GO:0005575	<1e-4	sll0647	GO:0003723	0.0508
ssr2848	GO:0006631	<1e-4	sll1304	GO:0006720	<1e-4	slr1338	GO:0022857	<1e-4	ssr3570	GO:0005575	<1e-4	slr0317	GO:0003723	0.0508
slr1398	GO:0006793	<1e-4	ssl2807	GO:0080090	<1e-4	slr0108	GO:0022832	<1e-4	sll1232	GO:0005575	<1e-4	sll0272	GO:0003723	0.0508
sll0072	GO:0009889	<1e-4	slr0455	GO:0006811	<1e-4	slr0300	GO:0016741	<1e-4	slr6088	GO:0044446	<1e-4	sll0103	GO:0003723	0.0508
sll1873	GO:0006631	<1e-4	sll1601	GO:0019752	<1e-4	ssl7051	GO:0015267	<1e-4	ssr1425	GO:0044425	<1e-4	sll1912	GO:0003723	0.0508
sll1659	GO:0050789	<1e-4	slr0489	GO:0043933	<1e-4	ssr6046	GO:0022890	<1e-4	sll8012	GO:0044422	<1e-4	slr0919	GO:0003723	0.0508
sll0241	GO:0019752	<1e-4	sll7033	GO:0031323	<1e-4	ssl3615	GO:0015405	<1e-4	ssr6026	GO:0044444	<1e-4	sll0606	GO:0003723	0.0508
ssl7022	GO:0065007	<1e-4	ssr3467	GO:0048523	<1e-4	sll1531	GO:0015077	<1e-4	ssl5008	GO:0044425	<1e-4	sll0861	GO:0016787	0.05
slr1644	GO:0009263	<1e-4	sll1571	GO:0031326	<1e-4	sll0369	GO:0022832	<1e-4	slr5087	GO:0005575	<1e-4	slr1658	GO:0005515	0.0493
sll1061	GO:0009259	<1e-4	sll1652	GO:0072522	<1e-4	sll1763	GO:0022832	<1e-4	slr0708	GO:0043226	<1e-4	ssr3300	GO:0005515	0.0493
ssr2711	GO:0044260	<1e-4	sll8002	GO:0009123	<1e-4	sll0847	GO:0022832	<1e-4	sll0596	GO:0044446	<1e-4	slr0815	GO:0005515	0.0493
slr2052	GO:0050789	<1e-4	slr1534	GO:0034654	<1e-4	sll0327	GO:0005342	<1e-4	sll1166	GO:0044424	<1e-4	sll1390	GO:0005515	0.0493
slr1563	GO:0009893	<1e-4	slr0771	GO:0005996	<1e-4	sll7047	GO:0022832	<1e-4	sll1675	GO:0044424	<1e-4	sll0297	GO:0005515	0.0493
ssr2781	GO:0071841	<1e-4	sll0641	GO:0019362	<1e-4	slr1258	GO:0015075	<1e-4	slr1577	GO:0044425	<1e-4	slr1169	GO:0005515	0.0493
sll1769	GO:0010468	<1e-4	ssl0350	GO:0009991	<1e-4	sll1863	GO:0015267	<1e-4	slr0482	GO:0044422	<1e-4	slr0468	GO:0005515	0.0493
sll0659	GO:0071840	<1e-4	slr0172	GO:0009991	<1e-4	sll0442	GO:0015291	<1e-4	sll1573	GO:0043226	<1e-4	slr0112	GO:0005515	0.0493
ssl0242	GO:0006576	<1e-4	slr0480	GO:0006720	<1e-4	slr0250	GO:0060089	<1e-4	slr1774	GO:0044425	<1e-4	slr0962	GO:0005515	0.0493
slr0728	GO:0009057	<1e-4	slr1674	GO:0031326	<1e-4	slr2118	GO:0022891	<1e-4	ssl2138	GO:0044444	<1e-4	slr0935	GO:0005515	0.0493
sll8007	GO:0006811	<1e-4	slr1957	GO:0071554	<1e-4	slr0345	GO:0008171	<1e-4	slr0442	GO:0005575	<1e-4	sll0997	GO:0005515	0.0493
sll1696	GO:0009124	<1e-4	sll2013	GO:0009199	<1e-4	slr5037	GO:0016818	<1e-4	ssr3300	GO:0005575	<1e-4	slr0272	GO:0005515	0.0493
sll1834	GO:0009991	<1e-4	slr6005	GO:0009165	<1e-4	ssl7074	GO:0022836	<1e-4	sll0325	GO:0044424	<1e-4	slr0483	GO:0005515	0.0493
slr1071	GO:0044260	<1e-4	slr0168	GO:0009263	<1e-4	slr0505	GO:0022857	<1e-4	ssr8047	GO:0044422	<1e-4	slr2005	GO:0005515	0.0493
sll0763	GO:0080090	<1e-4	sll0584	GO:0009161	<1e-4	slr1397	GO:0015291	<1e-4	slr0106	GO:0044422	<1e-4	slr1074	GO:0005515	0.0493
sll0412	GO:0009144	<1e-4	slr1958	GO:0072528	<1e-4	sgl0001	GO:0015405	<1e-4	slr0517	GO:0044425	<1e-4	slr0924	GO:0005515	0.0493
ssl1577	GO:0072522	<1e-4	ssr6003	GO:0043933	<1e-4	sll1954	GO:0016817	<1e-4	ssr5121	GO:0044422	<1e-4	sll1659	GO:0005515	0.0493

slr1110	GO:0019752	<1e-4	sll1601	GO:0060255	<1e-4	ssr6019	GO:0060089	<1e-4	slr6090	GO:0043226	<1e-4	slr1613	GO:0005515	0.0493
sll1024	GO:0051179	<1e-4	sll0543	GO:0006721	<1e-4	sll0732	GO:0022832	<1e-4	sll0910	GO:0044422	<1e-4	slr0510	GO:0005515	0.0493
sll1532	GO:0071840	<1e-4	sll0310	GO:0044260	<1e-4	slr1194	GO:0005342	<1e-4	slr7026	GO:0005575	<1e-4	slr1827	GO:0005515	0.0493
slr1261	GO:0006091	<1e-4	sll1304	GO:0051186	<1e-4	slr0503	GO:0015293	<1e-4	sll1874	GO:0005575	<1e-4	slr0111	GO:0005515	0.0493
ssl0294	GO:0080090	<1e-4	slr0869	GO:0006631	<1e-4	sll5097	GO:0022857	<1e-4	sll0268	GO:0043226	<1e-4	sll0740	GO:0005515	0.0493
sll1072	GO:0009150	<1e-4	sll0192	GO:0055082	<1e-4	slr0610	GO:0015291	<1e-4	sll0572	GO:0044444	<1e-4	sll0752	GO:0005515	0.0493
sll1239	GO:0034641	<1e-4	slr1442	GO:0051716	<1e-4	sll0376	GO:0043167	<1e-4	sll0010	GO:0044464	<1e-4	sll1173	GO:0005515	0.0493
slr6006	GO:0051179	<1e-4	slr1340	GO:0006811	<1e-4	slr1572	GO:0015075	<1e-4	slr0989	GO:0044444	<1e-4	sll1040	GO:0005515	0.0493
sll0614	GO:0010468	<1e-4	sll0853	GO:0050794	<1e-4	sll0861	GO:0042626	<1e-4	ssl7021	GO:0005575	<1e-4	sll0572	GO:0005515	0.0493
sll0447	GO:0043170	<1e-4	sll7090	GO:0044281	<1e-4	sll0266	GO:0015405	<1e-4	ssl7007	GO:0044424	<1e-4	sll1241	GO:0005515	0.0493
slr1726	GO:0009991	<1e-4	slr0049	GO:0034641	<1e-4	sll5003	GO:0015399	<1e-4	sll0156	GO:0044446	<1e-4	sll1730	GO:0005515	0.0493
ssr2787	GO:0009987	<1e-4	ssr6003	GO:0043412	<1e-4	sll0875	GO:0022832	<1e-4	slr1601	GO:0005575	<1e-4	slr0456	GO:0005515	0.0493
sll0553	GO:0022607	<1e-4	slr0610	GO:0008610	<1e-4	slr1599	GO:0022857	<1e-4	slr0272	GO:0005575	<1e-4	sll0180	GO:0005515	0.0493
slr1668	GO:0046164	<1e-4	slr1851	GO:0006721	<1e-4	sll1025	GO:0043492	<1e-4	slr0031	GO:0044424	<1e-4	sll1512	GO:0005515	0.0493
ssl0312	GO:0072522	<1e-4	slr1752	GO:0046128	<1e-4	slr5119	GO:0015293	<1e-4	sll1532	GO:0044444	<1e-4	slr0172	GO:0005515	0.0493
sll1174	GO:0009893	<1e-4	sll0327	GO:0071842	<1e-4	slr1442	GO:0016462	<1e-4	sll1634	GO:0044425	<1e-4	sll0281	GO:0005515	0.0493
slr0408	GO:0006766	<1e-4	slr1619	GO:0051188	<1e-4	slr1259	GO:0005342	<1e-4	slr1768	GO:0043226	<1e-4	sll0410	GO:0005515	0.0493
slr0456	GO:0046483	<1e-4	ssr6083	GO:0033013	<1e-4	ssr1114	GO:0022891	<1e-4	slr0092	GO:0044444	<1e-4	sll0781	GO:0005515	0.0493
slr1568	GO:0090304	<1e-4	sll1232	GO:0019751	<1e-4	sll1853	GO:0016818	<1e-4	slr6029	GO:0044425	<1e-4	slr0232	GO:0005515	0.0493
slr0589	GO:0042180	<1e-4	ssl5099	GO:0019752	<1e-4	sll1942	GO:0016817	<1e-4	sll0638	GO:0044446	<1e-4	sll1054	GO:0005515	0.0493
ssr2047	GO:0009260	<1e-4	slr1530	GO:0050794	<1e-4	slr1998	GO:0022836	<1e-4	ssl7007	GO:0044446	<1e-4	ssl8008	GO:0005515	0.0493
sll2013	GO:0051818	<1e-4	slr0887	GO:0006793	<1e-4	slr2084	GO:0015293	<1e-4	slr1117	GO:0044464	<1e-4	slr1258	GO:0005515	0.0493
slr0545	GO:0006753	<1e-4	slr1097	GO:0006721	<1e-4	slr1956	GO:0060089	<1e-4	ssl3291	GO:0043226	<1e-4	sll1072	GO:0005515	0.0493
ssl7074	GO:0009199	<1e-4	sll0590	GO:0010556	<1e-4	slr0815	GO:0022891	<1e-4	slr1900	GO:0044444	<1e-4	slr0789	GO:0005515	0.0493
ssr2615	GO:0009987	<1e-4	sll1390	GO:0019752	<1e-4	sll1965	GO:0016741	<1e-4	sll7078	GO:0044424	<1e-4	slr0060	GO:0005515	0.0493
sll1938	GO:0042180	<1e-4	slr1444	GO:0009141	<1e-4	sll1749	GO:0015405	<1e-4	ssl6061	GO:0044464	<1e-4	sll1170	GO:0005515	0.0493
slr6045	GO:0046164	<1e-4	slr0885	GO:0009394	<1e-4	sll0564	GO:0016741	<1e-4	slr1812	GO:0044446	<1e-4	sll1738	GO:0005515	0.0493
sll0590	GO:0019637	<1e-4	slr0006	GO:0006091	<1e-4	slr6063	GO:0043492	<1e-4	slr0243	GO:0044422	<1e-4	sll0827	GO:0005515	0.0493
ssr2047	GO:0043436	<1e-4	sll8001	GO:0051234	<1e-4	ssr6048	GO:0043167	<1e-4	sll1447	GO:0044425	<1e-4	slr0923	GO:0004871	0.0487
sll1009	GO:0009987	<1e-4	sll7065	GO:0016054	<1e-4	slr0730	GO:0022838	<1e-4	slr2118	GO:0044424	<1e-4	sll0577	GO:0004871	0.0487
slr6064	GO:0019637	<1e-4	slr0453	GO:0051188	<1e-4	slr1122	GO:0016741	<1e-4	slr1391	GO:0043226	<1e-4	sll0192	GO:0004871	0.0487
sll5006	GO:0045184	<1e-4	ssl2065	GO:0009056	<1e-4	slr0613	GO:0022857	<1e-4	slr0596	GO:0043226	<1e-4	slr0978	GO:0004871	0.0487
sll0068	GO:0072528	<1e-4	slr0702	GO:0050789	<1e-4	slr1863	GO:0003674	<1e-4	slr1081	GO:0043226	<1e-4	slr0607	GO:0004871	0.0487
ssl3291	GO:0031323	<1e-4	ssr1155	GO:0005996	<1e-4	sll1512	GO:0043167	<1e-4	slr1636	GO:0005575	<1e-4	ssl0353	GO:0004871	0.0487
slr0407	GO:0072528	<1e-4	sll0785	GO:0019362	<1e-4	slr1800	GO:0043167	<1e-4	sll0931	GO:0044425	<1e-4	sll0910	GO:0004871	0.0487
slr1704	GO:0072521	<1e-4	sll0775	GO:0044271	<1e-4	ssl2996	GO:0015405	<1e-4	slr1846	GO:0044446	<1e-4	sll1562	GO:0004871	0.0487
sll5109	GO:0009263	<1e-4	sll0069	GO:0009987	<1e-4	sll0442	GO:0015075	<1e-4	sll0063	GO:0043226	<1e-4	slr1865	GO:0004871	0.0487
sll8035	GO:0005996	<1e-4	sll0456	GO:0046483	<1e-4	slr0876	GO:0015293	<1e-4	sll1455	GO:0005575	<1e-4	slr1811	GO:0004871	0.0487
slr0780	GO:0051716	<1e-4	ssr2554	GO:0009394	<1e-4	slr0989	GO:0022890	<1e-4	slr0865	GO:0005575	<1e-4	sll1526	GO:0004871	0.0487
slr6012	GO:0051818	<1e-4	slr1066	GO:0051171	<1e-4	sll0397	GO:0015293	<1e-4	sll0597	GO:0044446	<1e-4	slr1301	GO:0004871	0.0487
sll1702	GO:0009142	<1e-4	sll0447	GO:0009892	<1e-4	sll0802	GO:0005342	<1e-4	slr1188	GO:0044424	<1e-4	sll0558	GO:0004871	0.0487
slr1863	GO:0006811	<1e-4	slr1535	GO:0045184	<1e-4	slr1179	GO:0043167	<1e-4	sll1530	GO:0044446	<1e-4	slr0594	GO:0004871	0.0487
slr0400	GO:0055086	<1e-4	slr1659	GO:0034641	<1e-4	slr1179	GO:0008171	<1e-4	slr0865	GO:0044425	<1e-4	slr0962	GO:0016740	0.0478
slr0935	GO:0042180	<1e-4	slr1223	GO:0046164	<1e-4	sll1915	GO:0043492	<1e-4	slr0656	GO:0044425	<1e-4	sll1304	GO:0016740	0.0478
slr5118	GO:0010556	<1e-4	slr0872	GO:0006733	<1e-4	slr5126	GO:0015075	<1e-4	sll1241	GO:0005575	<1e-4	sll1411	GO:0016740	0.0478
sll0189	GO:0006766	<1e-4	slr0341	GO:0050789	<1e-4	slr0149	GO:0015291	<1e-4	sll0296	GO:0005575	<1e-4	slr0112	GO:0016740	0.0478
slr1194	GO:0071496	<1e-4	ssr7035	GO:0009059	<1e-4	slr1944	GO:0016818	<1e-4	sll0072	GO:0043226	<1e-4	slr0742	GO:0016740	0.0478
slr1073	GO:0046164	<1e-4	sll5032	GO:0051716	<1e-4	slr0360	GO:0015405	<1e-4	ssr6032	GO:0005575	<1e-4	sll1730	GO:0016740	0.0478
slr0619	GO:0009123	<1e-4	sll1726	GO:0080090	<1e-4	ssl3692	GO:0022832	<1e-4	sll0565	GO:0005575	<1e-4	slr0981	GO:0016740	0.0478
slr1444	GO:0046164	<1e-4	slr0654	GO:0046164	<1e-4	slr0243	GO:0015291	<1e-4	slr0489	GO:0005575	<1e-4	slr0596	GO:0016740	0.0478
ssr0759	GO:0006091	<1e-4	sll0995	GO:0050801	<1e-4	sll1476	GO:0042623	<1e-4	slr0852	GO:0044464	<1e-4	sll1021	GO:0016740	0.0478
sll0743	GO:0006082	<1e-4	sll1306	GO:0042180	<1e-4	slr1095	GO:0016817	<1e-4	sll1783	GO:0044464	<1e-4	slr1827	GO:0016740	0.0478
slr0374	GO:0006220	<1e-4	sll1995	GO:0006811	<1e-4	slr0742	GO:0022803	<1e-4	ssl0467	GO:0044446	<1e-4	sll1239	GO:0016740	0.0478
slr0423	GO:0045184	<1e-4	sgl0001	GO:0071554	<1e-4	ssl0350	GO:0022832	<1e-4	slr1572	GO:0043226	<1e-4	sll1390	GO:0016740	0.0478

slr0806	GO:0055082	<1e-4	sll0735	GO:0071554	<1e-4	sll7034	GO:0022832	<1e-4	sll1340	GO:0005575	<1e-4	sll0180	GO:0016740	0.0478
slr1101	GO:0043933	<1e-4	slr0516	GO:0019222	<1e-4	slr1114	GO:0015399	<1e-4	ssl3142	GO:0044464	<1e-4	sll0319	GO:0016740	0.0478
ssr3532	GO:0044282	<1e-4	sll0543	GO:0051234	<1e-4	slr0975	GO:0016741	<1e-4	slr2119	GO:0044422	<1e-4	slr0169	GO:0016740	0.0478
sll0497	GO:0006811	<1e-4	sll0837	GO:0055114	<1e-4	slr2092	GO:0043167	<1e-4	ssr5011	GO:0044464	<1e-4	slr2048	GO:0016740	0.0478
sll5026	GO:0009126	<1e-4	slr0400	GO:0046164	<1e-4	sll0237	GO:0016818	<1e-4	sll1400	GO:0044422	<1e-4	slr0680	GO:0016740	0.0478
sll1911	GO:0044275	<1e-4	slr1590	GO:0051188	<1e-4	sll0376	GO:0022857	<1e-4	slr1944	GO:0044446	<1e-4	ssr1552	GO:0016740	0.0478
slr1768	GO:0043549	<1e-4	sll1262	GO:0006766	<1e-4	sll0185	GO:0022836	<1e-4	ssr8047	GO:0005575	<1e-4	sll1659	GO:0016740	0.0478
slr0514	GO:0050794	<1e-4	slr5087	GO:0050789	<1e-4	slr1773	GO:0016818	<1e-4	sll0597	GO:0044425	<1e-4	slr1977	GO:0016740	0.0478
sll0588	GO:0044255	<1e-4	sll5130	GO:0043436	<1e-4	sll0997	GO:0015077	<1e-4	sll0549	GO:0044464	<1e-4	slr0373	GO:0016740	0.0478
slr6065	GO:0031323	<1e-4	ssl8003	GO:0051818	<1e-4	sll0736	GO:0042623	<1e-4	slr2110	GO:0005575	<1e-4	slr2125	GO:0016740	0.0478
ssr5019	GO:0009262	<1e-4	sll1306	GO:0009161	<1e-4	slr0232	GO:0015399	<1e-4	slr6050	GO:0044446	<1e-4	sll1241	GO:0016740	0.0478
sll0871	GO:0044282	<1e-4	sll0443	GO:0009117	<1e-4	sll0414	GO:0022857	<1e-4	slr1462	GO:0044464	<1e-4	sll0585	GO:0016740	0.0478
sll0749	GO:0031640	<1e-4	slr0813	GO:0009259	<1e-4	slr5102	GO:0016817	<1e-4	sll0216	GO:0044422	<1e-4	sll0911	GO:0016740	0.0478
sll0444	GO:0072524	<1e-4	slr1956	GO:0009987	<1e-4	sll1024	GO:0015077	<1e-4	sll0335	GO:0044446	<1e-4	sll1582	GO:0016740	0.0478
slr0503	GO:0022411	<1e-4	slr0821	GO:0006733	<1e-4	sll5061	GO:0016746	<1e-4	sll0296	GO:0044444	<1e-4	ssr3300	GO:0016740	0.0478
ssr3402	GO:0051347	<1e-4	sll0625	GO:0044248	<1e-4	ssr7036	GO:0015399	<1e-4	slr1959	GO:0044444	<1e-4	sll1738	GO:0016740	0.0478
slr1398	GO:0034660	<1e-4	sll1174	GO:0065007	<1e-4	slr1612	GO:0015293	<1e-4	sll1880	GO:0005575	<1e-4	slr1053	GO:0016740	0.0478
ssl5091	GO:0044275	<1e-4	sll1446	GO:0046128	<1e-4	ssr1698	GO:0043167	<1e-4	sll1651	GO:0043226	<1e-4	sll1350	GO:0016740	0.0478
sll0449	GO:0009262	<1e-4	ssr7017	GO:0031323	<1e-4	sll1461	GO:0016818	<1e-4	sll1764	GO:0044424	<1e-4	slr2115	GO:0016740	0.0478
sll0994	GO:0009260	<1e-4	slr0909	GO:0006631	<1e-4	slr2027	GO:0022803	<1e-4	slr1752	GO:0044425	<1e-4	slr1762	GO:0016740	0.0478
slr2049	GO:0043412	<1e-4	sll1761	GO:0043648	<1e-4	slr0870	GO:0016818	<1e-4	ssl8005	GO:0044422	<1e-4	slr1541	GO:0016740	0.0478
sll0811	GO:0009889	<1e-4	sll0294	GO:0034660	<1e-4	sll7070	GO:0022891	<1e-4	sll0424	GO:0043226	<1e-4	slr1288	GO:0016740	0.0478
slr0870	GO:0019222	<1e-4	ssr1114	GO:0010468	<1e-4	ssr6024	GO:0015293	<1e-4	sll0639	GO:0005575	<1e-4	slr1383	GO:0016740	0.0478
sll1891	GO:0009161	<1e-4	slr1699	GO:0043170	<1e-4	sll1934	GO:0042623	<1e-4	sll0284	GO:0044446	<1e-4	slr0801	GO:0016740	0.0478
sll1531	GO:0009889	<1e-4	slr0552	GO:0006793	<1e-4	sll1472	GO:0016818	<1e-4	sll0296	GO:0044464	<1e-4	sll1654	GO:0016740	0.0478
sll1464	GO:0052111	<1e-4	slr0664	GO:0009141	<1e-4	slr1102	GO:0015075	<1e-4	slr1315	GO:0044425	<1e-4	sll0888	GO:0016740	0.0478
sll1247	GO:0031323	<1e-4	ssl1972	GO:0008150	<1e-4	slr1079	GO:0022891	<1e-4	sll1415	GO:0005575	<1e-4	ssl3382	GO:0016740	0.0478
sll1853	GO:0048523	<1e-4	slr0784	GO:0046907	<1e-4	slr0060	GO:0022832	<1e-4	slr6044	GO:0044464	<1e-4	sll1956	GO:0016740	0.0478
slr1270	GO:0034641	<1e-4	ssr1768	GO:0008610	<1e-4	slr0489	GO:0043167	<1e-4	sll0066	GO:0005575	<1e-4	sll1505	GO:0016740	0.0478
sll1902	GO:0065007	<1e-4	slr1566	GO:0051171	<1e-4	slr1676	GO:0022832	<1e-4	slr1920	GO:0044422	<1e-4	slr1287	GO:0016740	0.0478
slr1964	GO:0031640	<1e-4	slr1926	GO:0046483	<1e-4	slr2027	GO:0016741	<1e-4	slr0109	GO:0005575	<1e-4	sll0141	GO:0016740	0.0478
sll1304	GO:0009263	<1e-4	sll0783	GO:0060255	<1e-4	sll0615	GO:0015077	<1e-4	slr7057	GO:0044424	<1e-4	slr1062	GO:0016740	0.0478
ssl3383	GO:0009126	<1e-4	sll1681	GO:0006733	<1e-4	sll0406	GO:0043167	<1e-4	slr1923	GO:0044424	<1e-4	sll0981	GO:0016740	0.0478
slr0326	GO:0019752	<1e-4	slr1999	GO:0016054	<1e-4	ssr2843	GO:0016741	<1e-4	sll0786	GO:0044425	<1e-4	slr0249	GO:0016740	0.0478
slr1117	GO:0048522	<1e-4	sll0060	GO:0006732	<1e-4	slr1236	GO:0015267	<1e-4	ssr2317	GO:0044446	<1e-4	slr1647	GO:0016740	0.0478
slr1659	GO:0044281	<1e-4	slr2005	GO:0006766	<1e-4	slr0291	GO:0016746	<1e-4	ssl2781	GO:0044425	<1e-4	slr1128	GO:0016740	0.0478
ssr7084	GO:0009117	<1e-4	slr1907	GO:0010468	<1e-4	sll0218	GO:0016817	<1e-4	slr0217	GO:0005575	<1e-4	sll0539	GO:0016740	0.0478
ssl3291	GO:0072522	<1e-4	sll1455	GO:0065007	<1e-4	slr0607	GO:0016746	<1e-4	slr1533	GO:0044444	<1e-4	sll0156	GO:0016740	0.0478
sll0355	GO:0044248	<1e-4	slr1900	GO:0045184	<1e-4	sll1938	GO:0022890	<1e-4	sll1193	GO:0044424	<1e-4	slr1169	GO:0016740	0.0478
sll1193	GO:0051246	<1e-4	sll1797	GO:0009126	<1e-4	sll0645	GO:0042623	<1e-4	slr1896	GO:0005575	<1e-4	slr1544	GO:0016740	0.0478
slr0656	GO:0051716	<1e-4	slr0489	GO:0050896	<1e-4	slr0921	GO:0016817	<1e-4	ssr2972	GO:0044444	<1e-4	sll0980	GO:0016740	0.0478
sll0676	GO:0009142	<1e-4	sll1442	GO:0045184	<1e-4	sll5032	GO:0003674	<1e-4	slr6100	GO:0044425	<1e-4	slr1437	GO:0016740	0.0467
slr6045	GO:0001932	<1e-4	sll0742	GO:0009987	<1e-4	slr1535	GO:0003674	<1e-4	slr0990	GO:0044444	<1e-4	sll0309	GO:0016740	0.0467
sll0066	GO:0042180	<1e-4	ssl3142	GO:0009126	<1e-4	slr0887	GO:0042623	<1e-4	slr1363	GO:0044424	<1e-4	slr1932	GO:0016740	0.0467
sll1606	GO:0071841	<1e-4	sll1174	GO:0072522	<1e-4	sll0160	GO:0022832	<1e-4	ssr5020	GO:0044422	<1e-4	slr1230	GO:0016740	0.0467
sll0047	GO:0005996	<1e-4	sll5033	GO:0006066	<1e-4	sll1797	GO:0015399	<1e-4	slr5013	GO:0005575	<1e-4	sll1632	GO:0016740	0.0467
slr5023	GO:0009142	<1e-4	sll0101	GO:0044260	<1e-4	sll8035	GO:0015405	<1e-4	slr1338	GO:0044446	<1e-4	slr2111	GO:0016740	0.0467
ssr8013	GO:0006163	<1e-4	ssr0335	GO:0009144	<1e-4	sll0325	GO:0022890	<1e-4	sll1461	GO:0043226	<1e-4	sll0023	GO:0016740	0.0467
slr1303	GO:0042180	<1e-4	slr1864	GO:0072522	<1e-4	sll1527	GO:0015291	<1e-4	slr6005	GO:0044464	<1e-4	sll0497	GO:0016740	0.0467
ssl2807	GO:0006631	<1e-4	slr1885	GO:0006732	<1e-4	ssl3382	GO:0005342	<1e-4	ssl6061	GO:0043226	<1e-4	ssr2551	GO:0016740	0.0467
slr1034	GO:0072528	<1e-4	slr1365	GO:0031326	<1e-4	slr1270	GO:0008171	<1e-4	ssr2201	GO:0044446	<1e-4	sll0216	GO:0016740	0.0467
slr1082	GO:0043170	<1e-4	sll0269	GO:0046164	<1e-4	sll1306	GO:0015293	<1e-4	sll0448	GO:0044446	<1e-4	slr0431	GO:0016740	0.0467
sll1775	GO:0065007	<1e-4	sll8040	GO:0006576	<1e-4	slr1187	GO:0015077	<1e-4	slr1530	GO:0044464	<1e-4	ssr2962	GO:0016740	0.0467
sll1442	GO:0044283	<1e-4	slr2060	GO:0051179	<1e-4	slr7097	GO:0042623	<1e-4	sll1531	GO:0044446	<1e-4	slr1084	GO:0016740	0.0467

sll0910	GO:0009117	<1e-4	ssr0761	GO:0009057	<1e-4	slr7083	GO:0005342	<1e-4	slr0643	GO:0005575	<1e-4	ssr2615	GO:0016740	0.0467
slr5012	GO:0031640	<1e-4	slr1907	GO:0043412	<1e-4	slr0006	GO:0022890	<1e-4	slr1222	GO:0044422	<1e-4	ssl2807	GO:0016740	0.0467
slr1179	GO:0034654	<1e-4	sll1902	GO:0090304	<1e-4	ssl1377	GO:0008171	<1e-4	slr6005	GO:0044444	<1e-4	slr1788	GO:0016740	0.0467
sll0639	GO:0071496	<1e-4	sll1061	GO:0051171	<1e-4	sll0505	GO:0015077	<1e-4	ssl8003	GO:0044422	<1e-4	slr0392	GO:0016740	0.0467
slr1391	GO:0009165	<1e-4	sll0297	GO:0006766	<1e-4	slr1789	GO:0043167	<1e-4	slr2070	GO:0044446	<1e-4	slr0006	GO:0016740	0.0467
slr1103	GO:0033013	<1e-4	sll0661	GO:0052188	<1e-4	ssr1425	GO:0043492	<1e-4	slr1917	GO:0044424	<1e-4	slr1571	GO:0016740	0.0467
sll1135	GO:0006721	<1e-4	slr2120	GO:0009117	<1e-4	ssl2807	GO:0022803	<1e-4	ssr6002	GO:0005575	<1e-4	sll1658	GO:0016740	0.0467
slr8021	GO:0051818	<1e-4	slr5077	GO:0044282	<1e-4	slr0625	GO:0022892	<1e-4	sll5132	GO:0044422	<1e-4	slr1667	GO:0016740	0.0467
ssl8028	GO:0009892	<1e-4	sll1060	GO:0071554	<1e-4	ssl2814	GO:0022891	<1e-4	ssl7048	GO:0044464	<1e-4	slr0668	GO:0016740	0.0467
sll0423	GO:0001932	<1e-4	sll0216	GO:0050794	<1e-4	sll0423	GO:0022838	<1e-4	sll0854	GO:0044464	<1e-4	slr0941	GO:0016740	0.0467
slr6033	GO:0009132	<1e-4	slr0211	GO:0009309	<1e-4	sll0743	GO:0016462	<1e-4	slr1809	GO:0043226	<1e-4	slr0455	GO:0008168	0.0454
sll7063	GO:0009124	<1e-4	sll1640	GO:0031323	<1e-4	sll1186	GO:0022890	<1e-4	slr0582	GO:0043226	<1e-4	slr1762	GO:0008168	0.0454
sll0614	GO:0009262	<1e-4	slr2011	GO:0072522	<1e-4	sll0846	GO:0016746	<1e-4	sll1218	GO:0043226	<1e-4	ssr2754	GO:0008168	0.0454
slr0374	GO:0080090	<1e-4	slr0909	GO:0050896	<1e-4	slr1114	GO:0016741	<1e-4	slr0869	GO:0005575	<1e-4	slr1413	GO:0008168	0.0454
sll1049	GO:0019751	<1e-4	slr6100	GO:0048878	<1e-4	sll6052	GO:0022803	<1e-4	ssr2009	GO:0005575	<1e-4	sll1950	GO:0008168	0.0454
ssl8003	GO:0019362	<1e-4	sll1696	GO:0034654	<1e-4	slr6101	GO:0015075	<1e-4	sll0481	GO:0044425	<1e-4	slr2048	GO:0008168	0.0454
slr0709	GO:0034641	<1e-4	sll5089	GO:0009161	<1e-4	slr2025	GO:0015399	<1e-4	sll1232	GO:0044424	<1e-4	slr1658	GO:0008168	0.0454
sll0301	GO:0031640	<1e-4	sll0508	GO:0043648	<1e-4	sll0010	GO:0016818	<1e-4	slr1926	GO:0044425	<1e-4	sll0156	GO:0008168	0.0454
sll0505	GO:0044260	<1e-4	slr1799	GO:0010556	<1e-4	sll0446	GO:0022838	<1e-4	sll1969	GO:0043226	<1e-4	sll0740	GO:0008168	0.0454
sll0488	GO:0009141	<1e-4	sll1024	GO:0048878	<1e-4	slr6103	GO:0022803	<1e-4	slr0914	GO:0044424	<1e-4	sll0810	GO:0008168	0.0454
slr0172	GO:0051171	<1e-4	slr6049	GO:0060255	<1e-4	sll1273	GO:0015291	<1e-4	sll5046	GO:0044446	<1e-4	sll1021	GO:0008168	0.0454
sll1119	GO:0009308	<1e-4	slr0960	GO:0072528	<1e-4	ssr1114	GO:0022838	<1e-4	sll7047	GO:0044424	<1e-4	sll0141	GO:0008168	0.0454
slr5126	GO:0009161	<1e-4	slr1303	GO:0048518	<1e-4	sll1285	GO:0022838	<1e-4	sll1173	GO:0044444	<1e-4	slr1259	GO:0008168	0.0454
slr1812	GO:0050896	<1e-4	slr0731	GO:0043170	<1e-4	sll1583	GO:0015291	<1e-4	slr0483	GO:0043226	<1e-4	slr1613	GO:0008168	0.0454
sll1736	GO:0042455	<1e-4	slr1262	GO:0034654	<1e-4	ssr1552	GO:0016746	<1e-4	sll1381	GO:0044444	<1e-4	slr0887	GO:0008168	0.0454
slr5024	GO:0071841	<1e-4	slr1676	GO:0010556	<1e-4	slr7016	GO:0016817	<1e-4	slr0771	GO:0044425	<1e-4	slr0729	GO:0008168	0.0454
ssl5096	GO:0051347	<1e-4	slr0625	GO:0071554	<1e-4	sll0861	GO:0022838	<1e-4	sll0381	GO:0044422	<1e-4	slr1209	GO:0008168	0.0454
slr2111	GO:0009126	<1e-4	slr1814	GO:0044260	<1e-4	slr0586	GO:0015293	<1e-4	ssr3122	GO:0005575	<1e-4	slr1053	GO:0008168	0.0454
slr0516	GO:0009892	<1e-4	sll1939	GO:0071554	<1e-4	sll1730	GO:0043167	<1e-4	slr0575	GO:0044422	<1e-4	slr0664	GO:0008168	0.0454
ssr1425	GO:0006066	<1e-4	sll0563	GO:0019219	<1e-4	slr0730	GO:0022891	<1e-4	sll1586	GO:0005575	<1e-4	slr0111	GO:0008168	0.0454
ssl2920	GO:0071840	<1e-4	slr0656	GO:0071842	<1e-4	slr7023	GO:0016741	<1e-4	ssr0102	GO:0043226	<1e-4	sll1752	GO:0008168	0.0454
sll1938	GO:0065007	<1e-4	sll7062	GO:0016054	<1e-4	sll0149	GO:0005216	<1e-4	slr0241	GO:0005575	<1e-4	sll0588	GO:0008168	0.0454
slr1907	GO:0006793	<1e-4	ssr3532	GO:0006793	<1e-4	slr1110	GO:0022838	<1e-4	slr1917	GO:0043226	<1e-4	slr1977	GO:0008168	0.0454
ssr0102	GO:0019219	<1e-4	sll0854	GO:0051818	<1e-4	slr0510	GO:0043167	<1e-4	slr7083	GO:0005575	<1e-4	ssl8008	GO:0008168	0.0454
slr5012	GO:0072524	<1e-4	ssl3142	GO:0051716	<1e-4	sll1321	GO:0015399	<1e-4	sll0691	GO:0044444	<1e-4	sll0669	GO:0008168	0.0454
sll1873	GO:0044003	<1e-4	slr1056	GO:0006631	<1e-4	slr0217	GO:0016817	<1e-4	ssl8008	GO:0044422	<1e-4	sll0997	GO:0008168	0.0454
slr1275	GO:0009987	<1e-4	sll0328	GO:0006753	<1e-4	slr1571	GO:0022892	<1e-4	slr1436	GO:0044464	<1e-4	slr2005	GO:0008168	0.0454
slr1721	GO:0050794	<1e-4	sll0588	GO:0031326	<1e-4	slr1774	GO:0015293	<1e-4	sll1898	GO:0044464	<1e-4	sll0147	GO:0008168	0.0454
sll2006	GO:0019751	<1e-4	slr1079	GO:0048878	<1e-4	sll0230	GO:0022832	<1e-4	slr1066	GO:0044464	<1e-4	slr1122	GO:0008168	0.0454
sll1241	GO:0045184	<1e-4	ssr2711	GO:0042180	<1e-4	ssl2148	GO:0016817	<1e-4	sll1571	GO:0005575	<1e-4	sll1002	GO:0005975	0.0448
slr2070	GO:0071496	<1e-4	sll1752	GO:0022607	<1e-4	ssl0739	GO:0016741	<1e-4	slr1436	GO:0005575	<1e-4	ssr2551	GO:0005975	0.0448
slr1287	GO:0042451	<1e-4	ssl2595	GO:0046394	<1e-4	slr1263	GO:0005342	<1e-4	slr1033	GO:0005575	<1e-4	slr1573	GO:0005975	0.0448
ssr0332	GO:0044282	<1e-4	slr5112	GO:0009309	<1e-4	sll0860	GO:0022857	<1e-4	slr2122	GO:0044444	<1e-4	sll0703	GO:0005975	0.0448
sll0265	GO:0009126	<1e-4	ssl1520	GO:0046907	<1e-4	sll0157	GO:0015405	<1e-4	slr0959	GO:0044425	<1e-4	sll0237	GO:0005975	0.0448
slr1964	GO:0019751	<1e-4	slr1599	GO:0055086	<1e-4	slr1977	GO:0043492	<1e-4	sll5006	GO:0044425	<1e-4	ssr2439	GO:0005975	0.0448
ssr7035	GO:0072521	<1e-4	slr1032	GO:0072524	<1e-4	slr1394	GO:0022890	<1e-4	ssl2245	GO:0044444	<1e-4	sll0309	GO:0005975	0.0448
ssl3615	GO:0016054	<1e-4	slr0590	GO:0006753	<1e-4	slr1073	GO:0022857	<1e-4	slr2121	GO:0044446	<1e-4	slr1230	GO:0005975	0.0448
slr1396	GO:0009161	<1e-4	sll0102	GO:0034660	<1e-4	slr1150	GO:0016746	<1e-4	sll2006	GO:0005575	<1e-4	sll0243	GO:0005975	0.0448
sll0249	GO:0006732	<1e-4	slr6045	GO:0009199	<1e-4	ssl0350	GO:0022890	<1e-4	sll0985	GO:0044422	<1e-4	sll0785	GO:0005975	0.0448
sll0167	GO:0043648	<1e-4	slr0272	GO:0009987	<1e-4	sll1380	GO:0015405	<1e-4	slr1071	GO:0044444	<1e-4	ssr2787	GO:0005975	0.0448
slr2103	GO:0046164	<1e-4	slr1484	GO:0006091	<1e-4	ssl5108	GO:0005342	<1e-4	sll1979	GO:0005575	<1e-4	slr1436	GO:0005975	0.0448
ssr2142	GO:0046483	<1e-4	slr1636	GO:0043549	<1e-4	slr0304	GO:0015293	<1e-4	sll1726	GO:0005575	<1e-4	slr2144	GO:0005975	0.0448
slr0821	GO:0006066	<1e-4	slr1230	GO:0019222	<1e-4	sll0860	GO:0015075	<1e-4	sll0063	GO:0044464	<1e-4	slr1081	GO:0005975	0.0448
ssr6027	GO:0072522	<1e-4	ssr2553	GO:0034654	<1e-4	ssl6035	GO:0022857	<1e-4	slr0698	GO:0005575	<1e-4	slr1885	GO:0005975	0.0448

ssr2803	GO:0048878	<1e-4	slr2003	GO:0009123	<1e-4	sll0252	GO:0004872	<1e-4	sll1285	GO:0044444	<1e-4	slr0913	GO:0005975	0.0448
slr6049	GO:0006732	<1e-4	slr1677	GO:0052188	<1e-4	sll5090	GO:0022832	<1e-4	ssr6027	GO:0044424	<1e-4	ssl2807	GO:0005975	0.0448
slr1376	GO:0034654	<1e-4	slr0605	GO:0052188	<1e-4	ssr2553	GO:0042623	<1e-4	ssl2162	GO:0043226	<1e-4	slr1117	GO:0005975	0.0448
slr1340	GO:0065007	<1e-4	ssr6099	GO:0022411	<1e-4	slr1074	GO:0015293	<1e-4	slr1419	GO:0044422	<1e-4	slr1638	GO:0005975	0.0448
slr0039	GO:0050896	<1e-4	sll5032	GO:0009987	<1e-4	sll1730	GO:0022892	<1e-4	slr1570	GO:0044425	<1e-4	slr0569	GO:0005515	0.0447
slr1117	GO:0009126	<1e-4	sll0024	GO:0031640	<1e-4	slr1819	GO:0043167	<1e-4	sll7029	GO:0005575	<1e-4	ssl5095	GO:0005515	0.0447
sll1218	GO:0009260	<1e-4	ssr5074	GO:0009057	<1e-4	sll1638	GO:0015267	<1e-4	slr0751	GO:0044425	<1e-4	sll1571	GO:0005515	0.0447
slr1209	GO:0009260	<1e-4	ssl5100	GO:0090304	<1e-4	slr7057	GO:0016817	<1e-4	slr7059	GO:0044446	<1e-4	slr1397	GO:0005515	0.0447
slr0423	GO:0044003	<1e-4	ssr1558	GO:0009150	<1e-4	slr2084	GO:0005342	<1e-4	slr0360	GO:0044464	<1e-4	ssl0312	GO:0005515	0.0447
sll0760	GO:0044260	<1e-4	ssr2754	GO:0051179	<1e-4	sll1528	GO:0022857	<1e-4	ssr1528	GO:0044464	<1e-4	slr0262	GO:0005515	0.0447
sll1552	GO:0006631	<1e-4	slr6065	GO:0043412	<1e-4	slr6015	GO:0015399	<1e-4	sll0783	GO:0044464	<1e-4	slr6090	GO:0005515	0.0447
slr1814	GO:0019222	<1e-4	ssl1923	GO:0031326	<1e-4	sll0596	GO:0015405	<1e-4	sll1388	GO:0044444	<1e-4	slr6101	GO:0005515	0.0447
slr1544	GO:0048523	<1e-4	sll1455	GO:0005996	<1e-4	slr1045	GO:0005342	<1e-4	slr1547	GO:0005575	<1e-4	slr5118	GO:0005515	0.0447
ssr2439	GO:0006631	<1e-4	slr1287	GO:0046164	<1e-4	slr1814	GO:0016818	<1e-4	slr6074	GO:0043226	<1e-4	slr0337	GO:0005515	0.0447
slr1032	GO:0006066	<1e-4	sll0412	GO:0006732	<1e-4	sll0781	GO:0015399	<1e-4	slr6004	GO:0044464	<1e-4	sll8004	GO:0005515	0.0447
ssl5095	GO:0072524	<1e-4	slr1668	GO:0019752	<1e-4	sll0478	GO:0022836	<1e-4	sll1155	GO:0044425	<1e-4	sll1866	GO:0005515	0.0447
slr1196	GO:0046128	<1e-4	slr0109	GO:0008150	<1e-4	slr0392	GO:0016462	<1e-4	slr1398	GO:0044446	<1e-4	slr0959	GO:0005515	0.0447
ssl2814	GO:0065007	<1e-4	sll0269	GO:0010556	<1e-4	sml0011	GO:0022836	<1e-4	ssl3177	GO:0005575	<1e-4	sll1735	GO:0005515	0.0447
ssl1498	GO:0006720	<1e-4	slr1315	GO:0034660	<1e-4	slr0780	GO:0015291	<1e-4	ssr3129	GO:0005575	<1e-4	ssr6048	GO:0005515	0.0447
slr0870	GO:0044260	<1e-4	slr2073	GO:0042455	<1e-4	slr1768	GO:0022832	<1e-4	slr0889	GO:0044446	<1e-4	ssr2611	GO:0005515	0.0447
ssr0332	GO:0043549	<1e-4	sll0875	GO:0006220	<1e-4	slr1384	GO:0003674	<1e-4	slr1083	GO:0044446	<1e-4	slr7095	GO:0005515	0.0447
slr1911	GO:0009308	<1e-4	slr0937	GO:0009141	<1e-4	ssr6085	GO:0042623	<1e-4	sll1254	GO:0043226	<1e-4	sll1773	GO:0005515	0.0447
sll1025	GO:0009987	<1e-4	ssr7084	GO:0034641	<1e-4	ssr2711	GO:0016462	<1e-4	slr0810	GO:0044424	<1e-4	slr1168	GO:0005515	0.0447
sll1509	GO:0006732	<1e-4	slr1628	GO:0034660	<1e-4	sll1765	GO:0015405	<1e-4	sll1797	GO:0005575	<1e-4	ssr3189	GO:0005515	0.0447
sll0676	GO:0071496	<1e-4	slr1203	GO:0008150	<1e-4	slr0416	GO:0008171	<1e-4	sll5046	GO:0044424	<1e-4	slr1097	GO:0005515	0.0447
ssr6002	GO:0050801	<1e-4	slr2005	GO:0019637	<1e-4	ssl6092	GO:0022832	<1e-4	ssl5095	GO:0043226	<1e-4	slr5116	GO:0005515	0.0447
slr0545	GO:0065007	<1e-4	sll0414	GO:0031640	<1e-4	slr0780	GO:0022857	<1e-4	slr1104	GO:0044444	<1e-4	slr0398	GO:0005515	0.0447
slr0112	GO:0009260	<1e-4	sll1681	GO:0009892	<1e-4	slr1593	GO:0015405	<1e-4	sll1503	GO:0044425	<1e-4	ssl5129	GO:0005515	0.0447
ssl2781	GO:0046164	<1e-4	sll1321	GO:0022411	<1e-4	ssr2998	GO:0022832	<1e-4	slr6094	GO:0044446	<1e-4	slr2032	GO:0005515	0.0447
slr0270	GO:0051246	<1e-4	ssr6099	GO:0072521	<1e-4	ssr1114	GO:0015405	<1e-4	slr0878	GO:0044464	<1e-4	sll1355	GO:0005515	0.0447
slr1880	GO:0052188	<1e-4	slr6067	GO:0048878	<1e-4	sll1586	GO:0016741	<1e-4	ssl0350	GO:0044422	<1e-4	sll1691	GO:0005515	0.0447
sll1526	GO:0005996	<1e-4	slr1276	GO:0071841	<1e-4	sll1250	GO:0015267	<1e-4	ssr1558	GO:0044464	<1e-4	ssl2420	GO:0005515	0.0447
sll7050	GO:0046128	<1e-4	ssr6062	GO:0051716	<1e-4	slr0104	GO:0043167	<1e-4	sll0786	GO:0043226	<1e-4	slr7083	GO:0005515	0.0447
slr5037	GO:0046128	<1e-4	sll1485	GO:0009165	<1e-4	slr7094	GO:0022836	<1e-4	slr1128	GO:0044422	<1e-4	slr1863	GO:0005515	0.0447
ssl1464	GO:0071840	<1e-4	slr1940	GO:0009142	<1e-4	sll1606	GO:0022832	<1e-4	ssr1766	GO:0044464	<1e-4	ssl5007	GO:0005515	0.0447
sll1832	GO:0044248	<1e-4	ssr2333	GO:0019222	<1e-4	ssr1155	GO:0022890	<1e-4	sll0328	GO:0005575	<1e-4	slr6067	GO:0005515	0.0447
ssr1425	GO:0071496	<1e-4	slr0787	GO:0006091	<1e-4	sll0069	GO:0015077	<1e-4	slr0013	GO:0005575	<1e-4	ssl5098	GO:0005515	0.0447
slr0755	GO:0045184	<1e-4	sll0503	GO:0050801	<1e-4	slr7099	GO:0022857	<1e-4	slr1087	GO:0044425	<1e-4	sll1510	GO:0005515	0.0447
slr1053	GO:0019362	<1e-4	sll0995	GO:0009059	<1e-4	sll0448	GO:0022838	<1e-4	sll1214	GO:0044464	<1e-4	slr0196	GO:0005515	0.0447
slr7102	GO:0006721	<1e-4	slr1287	GO:0006720	<1e-4	sll1573	GO:0022836	<1e-4	sll0185	GO:0005575	<1e-4	sll1797	GO:0005515	0.0447
slr0397	GO:0071840	<1e-4	slr0270	GO:0006631	<1e-4	slr0373	GO:0042623	<1e-4	slr1240	GO:0044424	<1e-4	slr1957	GO:0005515	0.0447
slr1301	GO:0009987	<1e-4	slr1173	GO:0001932	<1e-4	sll1954	GO:0022836	<1e-4	sll1306	GO:0044446	<1e-4	ssl3142	GO:0005515	0.0447
slr0294	GO:0006733	<1e-4	slr0503	GO:0046483	<1e-4	sll0280	GO:0022836	<1e-4	ssr0759	GO:0043226	<1e-4	sll0762	GO:0005515	0.0447
slr0731	GO:0009165	<1e-4	ssl5064	GO:0009892	<1e-4	slr2110	GO:0042623	<1e-4	slr1383	GO:0005575	<1e-4	sll1766	GO:0005515	0.0447
ssl7046	GO:0046907	<1e-4	slr1668	GO:0065007	<1e-4	sll1040	GO:0022803	<1e-4	sll0596	GO:0043226	<1e-4	ssl2245	GO:0005515	0.0447
slr1886	GO:0043412	<1e-4	slr0145	GO:0019637	<1e-4	slr0981	GO:0005342	<1e-4	sll0931	GO:0044464	<1e-4	slr0751	GO:0005515	0.0447
slr1800	GO:0051716	<1e-4	sll0241	GO:0043549	<1e-4	sll1201	GO:0043167	<1e-4	slr1353	GO:0044446	<1e-4	sll1884	GO:0005515	0.0447
sll0168	GO:0031326	<1e-4	slr0058	GO:0016054	<1e-4	slr0912	GO:0005342	<1e-4	slr1210	GO:0044464	<1e-4	sll1921	GO:0005515	0.0447
slr0146	GO:0071842	<1e-4	sll0761	GO:0052188	<1e-4	slr1415	GO:0015399	<1e-4	slr0708	GO:0044464	<1e-4	sll1123	GO:0005515	0.0447
ssl0467	GO:0072524	<1e-4	ssr0761	GO:0051246	<1e-4	sll7066	GO:0015075	<1e-4	sll0886	GO:0043226	<1e-4	ssl2471	GO:0005515	0.0447
sll7065	GO:0043933	<1e-4	slr0554	GO:0051818	<1e-4	slr0195	GO:0060089	<1e-4	sll0584	GO:0044425	<1e-4	slr6064	GO:0005515	0.0447
sll0656	GO:0051186	<1e-4	sll1722	GO:0019752	<1e-4	sll1135	GO:0042623	<1e-4	ssl1464	GO:0044422	<1e-4	ssr3410	GO:0005515	0.0447
sll5132	GO:0009889	<1e-4	slr1472	GO:0016054	<1e-4	ssr2009	GO:0016818	<1e-4	slr5012	GO:0044444	<1e-4	sll0293	GO:0005515	0.0447
ssl2920	GO:0065007	<1e-4	sll0350	GO:0044248	<1e-4	slr1674	GO:0042623	<1e-4	slr0341	GO:0044444	<1e-4	slr1814	GO:0005515	0.0447

sll0350	GO:0009309	<1e-4	sll1573	GO:0055082	<1e-4	ssr6019	GO:0005342	<1e-4	slr1880	GO:0044444	<1e-4	sll0802	GO:0005515	0.0447
slr0871	GO:0006082	<1e-4	slr0613	GO:0018130	<1e-4	ssr3402	GO:0016746	<1e-4	sll5097	GO:0044464	<1e-4	sll1863	GO:0005515	0.0447
slr1815	GO:0050896	<1e-4	slr1340	GO:0042180	<1e-4	ssr1391	GO:0022857	<1e-4	slr0602	GO:0044425	<1e-4	slr7080	GO:0005515	0.0447
slr7060	GO:0006720	<1e-4	slr1152	GO:0009132	<1e-4	sll1571	GO:0022890	<1e-4	sll0428	GO:0044425	<1e-4	ssl1004	GO:0005515	0.0447
sll1680	GO:0031326	<1e-4	slr1103	GO:0009987	<1e-4	sll1609	GO:0022836	<1e-4	slr2000	GO:0043226	<1e-4	slr0264	GO:0005515	0.0447
slr0195	GO:0065007	<1e-4	slr0364	GO:0034654	<1e-4	slr1896	GO:0015267	<1e-4	sll1680	GO:0044464	<1e-4	slr1484	GO:0005515	0.0447
slr1862	GO:0051716	<1e-4	sll0172	GO:0001932	<1e-4	sll1532	GO:0005342	<1e-4	slr0359	GO:0044464	<1e-4	slr0355	GO:0005515	0.0447
ssr0102	GO:0051171	<1e-4	sll1527	GO:0019222	<1e-4	slr0848	GO:0060089	<1e-4	slr0728	GO:0043226	<1e-4	sll1606	GO:0005515	0.0447
slr0517	GO:0072522	<1e-4	sll8007	GO:0034641	<1e-4	sll1265	GO:0060089	<1e-4	ssr2802	GO:0044464	<1e-4	sll0406	GO:0005515	0.0447
sll0284	GO:0044271	<1e-4	sll0861	GO:0072521	<1e-4	sll5063	GO:0042623	<1e-4	slr0148	GO:0044422	<1e-4	ssl5031	GO:0005515	0.0447
sll0085	GO:0043933	<1e-4	slr0304	GO:0019752	<1e-4	slr0271	GO:0022803	<1e-4	sll1318	GO:0044425	<1e-4	slr1907	GO:0005515	0.0447
sll1004	GO:0022411	<1e-4	slr1660	GO:0043933	<1e-4	ssr1766	GO:0022838	<1e-4	sll1509	GO:0005575	<1e-4	sll0241	GO:0005515	0.0447
slr0144	GO:0008150	<1e-4	ssr1528	GO:0034654	<1e-4	sll0149	GO:0043492	<1e-4	sll0588	GO:0044446	<1e-4	slr1721	GO:0005515	0.0447
sll0218	GO:0018130	<1e-4	sll0022	GO:0006082	<1e-4	ssr1552	GO:0015293	<1e-4	sll0157	GO:0044444	<1e-4	sll1163	GO:0005515	0.0447
sll1002	GO:0009132	<1e-4	slr7013	GO:0051171	<1e-4	slr0232	GO:0005342	<1e-4	slr1413	GO:0044422	<1e-4	sll1306	GO:0005515	0.0447
slr2032	GO:0034660	<1e-4	slr0442	GO:0046483	<1e-4	ssl5091	GO:0016817	<1e-4	sll0783	GO:0043226	<1e-4	sll0487	GO:0005515	0.0447
sll1942	GO:0043436	<1e-4	sll0265	GO:0048523	<1e-4	sll1340	GO:0016817	<1e-4	sll0980	GO:0044446	<1e-4	ssr2317	GO:0005515	0.0447
sll0482	GO:0006066	<1e-4	sll0678	GO:0043933	<1e-4	slr0887	GO:0015267	<1e-4	ssr6099	GO:0005575	<1e-4	sll7066	GO:0005515	0.0447
slr1958	GO:0006766	<1e-4	sll1738	GO:0060255	<1e-4	sll7089	GO:0042623	<1e-4	sll0325	GO:0005575	<1e-4	sll0742	GO:0005515	0.0447
slr0729	GO:0072528	<1e-4	sll0911	GO:0019637	<1e-4	sll1092	GO:0015291	<1e-4	slr1290	GO:0044464	<1e-4	sll1022	GO:0005515	0.0447
slr0305	GO:0006811	<1e-4	slr1628	GO:0090304	<1e-4	sll2015	GO:0016746	<1e-4	slr5127	GO:0044425	<1e-4	sll1414	GO:0005515	0.0447
slr1616	GO:0006082	<1e-4	ssr5106	GO:0046164	<1e-4	ssl5096	GO:0015075	<1e-4	slr1464	GO:0005575	<1e-4	slr7013	GO:0005515	0.0447
slr1276	GO:0051171	<1e-4	sll0062	GO:0009892	<1e-4	slr7096	GO:0022890	<1e-4	sll1109	GO:0044444	<1e-4	sll1862	GO:0005515	0.0447
sll1495	GO:0009260	<1e-4	slr0742	GO:0009123	<1e-4	sll0905	GO:0005342	<1e-4	slr0554	GO:0005575	<1e-4	sll1217	GO:0005515	0.0447
slr8021	GO:0009889	<1e-4	ssr7036	GO:0009142	<1e-4	slr1047	GO:0022838	<1e-4	slr0957	GO:0044425	<1e-4	slr0890	GO:0005515	0.0447
ssr0759	GO:0051179	<1e-4	ssl3573	GO:0009987	<1e-4	slr0060	GO:0022891	<1e-4	slr0360	GO:0043226	<1e-4	slr0505	GO:0005515	0.0447
sll6055	GO:0031326	<1e-4	slr1658	GO:0031640	<1e-4	slr1568	GO:0016818	<1e-4	slr2092	GO:0005575	<1e-4	slr1998	GO:0005515	0.0447
slr1258	GO:0005996	<1e-4	sll0481	GO:0050794	<1e-4	sll1477	GO:0043167	<1e-4	slr0789	GO:0044424	<1e-4	sll1638	GO:0005515	0.0447
sll0641	GO:0006766	<1e-4	slr7073	GO:0009260	<1e-4	sll0803	GO:0022836	<1e-4	sll0310	GO:0044425	<1e-4	slr0058	GO:0005515	0.0447
slr1917	GO:0009141	<1e-4	sll5063	GO:0042455	<1e-4	sll0505	GO:0043167	<1e-4	slr1535	GO:0043226	<1e-4	slr0971	GO:0005515	0.0447
slr6049	GO:0001932	<1e-4	sll0102	GO:0044248	<1e-4	slr1081	GO:0016818	<1e-4	sll0658	GO:0044422	<1e-4	slr0333	GO:0005515	0.0447
sll2015	GO:0034641	<1e-4	slr0505	GO:0009987	<1e-4	slr0431	GO:0042623	<1e-4	sll1004	GO:0044446	<1e-4	slr1103	GO:0005515	0.0447
sll0410	GO:0006163	<1e-4	ssr1499	GO:0042180	<1e-4	ssl7021	GO:0060089	<1e-4	slr1914	GO:0043226	<1e-4	slr7096	GO:0005515	0.0447
ssl0461	GO:0019751	<1e-4	slr0872	GO:0055114	<1e-4	sll1160	GO:0016746	<1e-4	slr0334	GO:0044425	<1e-4	slr0151	GO:0005515	0.0447
sll1925	GO:0072524	<1e-4	slr6088	GO:0019637	<1e-4	slr1301	GO:0022891	<1e-4	ssl0410	GO:0044422	<1e-4	slr1378	GO:0005515	0.0447
slr6074	GO:0006631	<1e-4	slr1178	GO:0009165	<1e-4	slr0243	GO:0060089	<1e-4	sll0858	GO:0005575	<1e-4	ssl7046	GO:0005515	0.0447
sll5067	GO:0044255	<1e-4	sll0062	GO:0044255	<1e-4	sll1765	GO:0022891	<1e-4	sll1862	GO:0044464	<1e-4	sll0498	GO:0005515	0.0447
slr6015	GO:0031326	<1e-4	slr0169	GO:0009126	<1e-4	ssl5025	GO:0015077	<1e-4	slr1275	GO:0043226	<1e-4	slr6065	GO:0005515	0.0447
sll0742	GO:0042455	<1e-4	sll1132	GO:0050789	<1e-4	slr0876	GO:0003674	<1e-4	sll0102	GO:0005575	<1e-4	slr1178	GO:0005515	0.0447
slr6071	GO:0050801	<1e-4	slr0888	GO:0044106	<1e-4	slr1327	GO:0015293	<1e-4	slr1266	GO:0005575	<1e-4	sll0930	GO:0005515	0.0447
ssl0832	GO:0019752	<1e-4	slr0810	GO:0016053	<1e-4	slr0885	GO:0008171	<1e-4	slr6007	GO:0044425	<1e-4	sll1252	GO:0005515	0.0447
slr0919	GO:0044260	<1e-4	slr1116	GO:0046164	<1e-4	slr1886	GO:0015399	<1e-4	sll1960	GO:0044422	<1e-4	slr1342	GO:0005515	0.0447
slr0913	GO:0016053	<1e-4	sll1348	GO:0006793	<1e-4	slr0554	GO:0022890	<1e-4	slr0496	GO:0044424	<1e-4	slr6072	GO:0005515	0.0447
sll0497	GO:0034654	<1e-4	sll1396	GO:0071841	<1e-4	slr6049	GO:0016818	<1e-4	slr0442	GO:0044425	<1e-4	slr1069	GO:0005515	0.0447
slr0408	GO:0022411	<1e-4	sll1960	GO:0044248	<1e-4	slr0262	GO:0003674	<1e-4	sll0249	GO:0044446	<1e-4	sll0008	GO:0005515	0.0447
sll0742	GO:0009059	<1e-4	sll0325	GO:0043648	<1e-4	slr1196	GO:0016746	<1e-4	sll0503	GO:0043226	<1e-4	slr0380	GO:0005515	0.0447
sll0238	GO:0006066	<1e-4	ssl5113	GO:0019752	<1e-4	ssl5025	GO:0043167	<1e-4	slr0601	GO:0005575	<1e-4	sll0168	GO:0005515	0.0447
sll0939	GO:0008610	<1e-4	sll1675	GO:0044106	<1e-4	sll0265	GO:0015077	<1e-4	slr0195	GO:0043226	<1e-4	sll2013	GO:0005515	0.0447
slr1773	GO:0090304	<1e-4	slr1398	GO:0050789	<1e-4	slr1918	GO:0043167	<1e-4	ssr3300	GO:0044446	<1e-4	sll1764	GO:0005515	0.0447
sll0410	GO:0050896	<1e-4	slr0852	GO:0043436	<1e-4	ssr3341	GO:0022836	<1e-4	sll0696	GO:0005575	<1e-4	slr7015	GO:0005515	0.0447
sll0479	GO:0009165	<1e-4	slr0634	GO:0045184	<1e-4	slr6072	GO:0015405	<1e-4	sll0498	GO:0005575	<1e-4	slr5023	GO:0005515	0.0447
sll1401	GO:0019752	<1e-4	sll1880	GO:0009308	<1e-4	ssl5091	GO:0015293	<1e-4	slr0333	GO:0005575	<1e-4	slr1660	GO:0005515	0.0447
slr1082	GO:0009142	<1e-4	slr1544	GO:0065007	<1e-4	slr1263	GO:0008171	<1e-4	slr0815	GO:0044425	<1e-4	slr0695	GO:0005515	0.0447
slr0890	GO:0044248	<1e-4	sll1613	GO:0044003	<1e-4	sll1155	GO:0022857	<1e-4	sll5128	GO:0043226	<1e-4	sll1485	GO:0005515	0.0447

sll1915	GO:0009150	<1e-4	sll0995	GO:0019637	<1e-4	sll0225	GO:0022803	<1e-4	slr1919	GO:0005575	<1e-4	slr1273	GO:0005515	0.0447
slr1152	GO:0006793	<1e-4	slr2073	GO:0009889	<1e-4	sll0007	GO:0043492	<1e-4	slr6080	GO:0005575	<1e-4	slr0362	GO:0005515	0.0447
slr0609	GO:0051234	<1e-4	slr1391	GO:0090304	<1e-4	sll0189	GO:0005342	<1e-4	ssl7074	GO:0044425	<1e-4	slr7011	GO:0005515	0.0447
sll0513	GO:0010556	<1e-4	slr1957	GO:0044283	<1e-4	sll1166	GO:0005342	<1e-4	sll7069	GO:0044422	<1e-4	sll0775	GO:0005515	0.0447
slr0667	GO:0009165	<1e-4	ssr3300	GO:0019362	<1e-4	sll1344	GO:0015399	<1e-4	slr1195	GO:0044422	<1e-4	sll1461	GO:0005515	0.0447
slr0989	GO:0072527	<1e-4	sll0763	GO:0009161	<1e-4	slr1690	GO:0043492	<1e-4	sll0839	GO:0044422	<1e-4	slr1600	GO:0005515	0.0447
slr0407	GO:0009150	<1e-4	sll0423	GO:0044260	<1e-4	slr0209	GO:0022857	<1e-4	slr1914	GO:0044424	<1e-4	slr1864	GO:0005515	0.0447
slr0755	GO:0051347	<1e-4	slr7014	GO:0009987	<1e-4	slr5111	GO:0015267	<1e-4	slr0731	GO:0043226	<1e-4	ssr2615	GO:0005515	0.0446
slr1464	GO:0090304	<1e-4	sll0007	GO:0006811	<1e-4	sll0162	GO:0015267	<1e-4	slr1215	GO:0043226	<1e-4	slr1572	GO:0005515	0.0446
sll0293	GO:0009132	<1e-4	slr0108	GO:0019222	<1e-4	sll0676	GO:0043167	<1e-4	slr0503	GO:0044446	<1e-4	sll1761	GO:0005515	0.0446
sll5033	GO:0060255	<1e-4	slr2144	GO:0046394	<1e-4	sll0996	GO:0005342	<1e-4	ssr2439	GO:0044422	<1e-4	sll0237	GO:0005515	0.0446
slr1812	GO:0001932	<1e-4	slr6013	GO:0006732	<1e-4	sll5047	GO:0022838	<1e-4	ssl0312	GO:0044464	<1e-4	slr2144	GO:0005515	0.0446
slr5013	GO:0065007	<1e-4	slr7010	GO:0006091	<1e-4	sll1632	GO:0015075	<1e-4	slr7012	GO:0005575	<1e-4	slr1964	GO:0005515	0.0446
ssr1473	GO:0019222	<1e-4	ssr1041	GO:0072528	<1e-4	sll0101	GO:0015267	<1e-4	ssl1972	GO:0044446	<1e-4	ssr3571	GO:0005515	0.0446
sll0678	GO:0042180	<1e-4	sll0268	GO:0045184	<1e-4	sll0513	GO:0016462	<1e-4	sll1411	GO:0044425	<1e-4	slr2121	GO:0005515	0.0446
sll0325	GO:0009123	<1e-4	sll0702	GO:0009126	<1e-4	slr1442	GO:0043492	<1e-4	ssl1004	GO:0044422	<1e-4	slr0294	GO:0005515	0.0446
sll1495	GO:0051234	<1e-4	sll1241	GO:0005996	<1e-4	slr1612	GO:0016462	<1e-4	ssr2975	GO:0005575	<1e-4	slr1436	GO:0005515	0.0446
ssr6085	GO:0043170	<1e-4	ssr2047	GO:0009056	<1e-4	slr1546	GO:0022803	<1e-4	sll0822	GO:0044444	<1e-4	slr0386	GO:0005515	0.0446
slr1544	GO:0055086	<1e-4	sll0376	GO:0006720	<1e-4	sll0355	GO:0016741	<1e-4	ssr1558	GO:0044422	<1e-4	sll0007	GO:0005515	0.0446
ssr1698	GO:0009057	<1e-4	slr0740	GO:0008150	<1e-4	sll1784	GO:0043492	<1e-4	slr0919	GO:0005575	<1e-4	slr0348	GO:0005515	0.0446
slr0695	GO:0055114	<1e-4	ssr1951	GO:0043933	<1e-4	slr1768	GO:0022890	<1e-4	slr0489	GO:0044424	<1e-4	sll1315	GO:0005515	0.0446
sll0069	GO:0051347	<1e-4	sll0661	GO:0048522	<1e-4	sll1265	GO:0015077	<1e-4	sll0314	GO:0005575	<1e-4	slr0204	GO:0005515	0.0446
sll1717	GO:0048878	<1e-4	sll0525	GO:0055114	<1e-4	slr0594	GO:0015405	<1e-4	ssr2439	GO:0005575	<1e-4	slr1885	GO:0005515	0.0446
slr1290	GO:0019752	<1e-4	sll0354	GO:0009165	<1e-4	sll1304	GO:0022836	<1e-4	slr1773	GO:0044424	<1e-4	sll1613	GO:0005515	0.0446
sll7063	GO:0042451	<1e-4	ssr6099	GO:0009987	<1e-4	slr1150	GO:0060089	<1e-4	sll1241	GO:0043226	<1e-4	sll0177	GO:0005515	0.0446
sll1495	GO:0051186	<1e-4	slr7082	GO:0009260	<1e-4	sll1681	GO:0042623	<1e-4	slr1050	GO:0044425	<1e-4	slr1667	GO:0005515	0.0446
slr2111	GO:0044248	<1e-4	sll0543	GO:0010556	<1e-4	ssr0335	GO:0060089	<1e-4	sll0861	GO:0043234	<1e-4	slr0006	GO:0005515	0.0446
slr1261	GO:0071840	<1e-4	slr1259	GO:0071840	<1e-4	slr0144	GO:0022890	<1e-4	slr0195	GO:0044444	<1e-4	sll0645	GO:0005515	0.0446
sll1049	GO:0009394	<1e-4	sll7069	GO:0016054	<1e-4	sll1285	GO:0015399	<1e-4	slr0519	GO:0005575	<1e-4	ssr2755	GO:0005515	0.0446
ssr6086	GO:0090304	<1e-4	sll1891	GO:0006631	<1e-4	sll1373	GO:0042623	<1e-4	slr1407	GO:0044444	<1e-4	slr2073	GO:0005515	0.0446
sll1247	GO:0033013	<1e-4	slr6101	GO:0019751	<1e-4	slr1438	GO:0015291	<1e-4	slr1851	GO:0005575	<1e-4	slr1230	GO:0005515	0.0446
slr0755	GO:0050801	<1e-4	slr1970	GO:0046907	<1e-4	ssr6083	GO:0043167	<1e-4	slr1287	GO:0044464	<1e-4	sll0497	GO:0005515	0.0446
ssl0738	GO:0044283	<1e-4	sll0189	GO:0042180	<1e-4	slr0651	GO:0016746	<1e-4	sll0837	GO:0043226	<1e-4	sll0381	GO:0005515	0.0446
sll0545	GO:0009987	<1e-4	slr0960	GO:0006732	<1e-4	slr1449	GO:0016462	<1e-4	slr5073	GO:0044425	<1e-4	sll1002	GO:0005515	0.0446
slr0287	GO:0046483	<1e-4	slr1084	GO:0051716	<1e-4	sll1272	GO:0022891	<1e-4	slr0483	GO:0044422	<1e-4	ssr1765	GO:0005515	0.0446
slr5023	GO:0006793	<1e-4	slr0272	GO:0052111	<1e-4	sll1834	GO:0043492	<1e-4	slr0517	GO:0044446	<1e-4	sll0216	GO:0005515	0.0446
slr0770	GO:0009199	<1e-4	ssl7038	GO:0009144	<1e-4	slr0505	GO:0015075	<1e-4	ssl1792	GO:0044422	<1e-4	sll0325	GO:0005515	0.0446
slr1572	GO:0051186	<1e-4	ssl7042	GO:0008610	<1e-4	ssr1256	GO:0015267	<1e-4	sll1581	GO:0044422	<1e-4	sll0085	GO:0005515	0.0446
sll1769	GO:0044003	<1e-4	sll0737	GO:0048522	<1e-4	slr0650	GO:0015077	<1e-4	sll5132	GO:0043226	<1e-4	slr1210	GO:0005515	0.0446
sll0647	GO:0055114	<1e-4	slr0865	GO:0050794	<1e-4	slr0204	GO:0022892	<1e-4	slr1261	GO:0044422	<1e-4	sll0243	GO:0005515	0.0446
sll0283	GO:0042451	<1e-4	slr2004	GO:0050896	<1e-4	slr0888	GO:0060089	<1e-4	ssl7021	GO:0043226	<1e-4	sll1775	GO:0004871	0.0445
slr6005	GO:0019751	<1e-4	sll0286	GO:0031326	<1e-4	sll0925	GO:0022803	<1e-4	slr0941	GO:0044444	<1e-4	sll1424	GO:0004871	0.0445
slr1394	GO:0043933	<1e-4	sll1891	GO:0034654	<1e-4	slr0650	GO:0015291	<1e-4	sll0284	GO:0044424	<1e-4	ssl0832	GO:0004871	0.0445
sll0639	GO:0044248	<1e-4	slr0870	GO:0006576	<1e-4	slr0976	GO:0016462	<1e-4	sll1630	GO:0044444	<1e-4	sll0103	GO:0004871	0.0445
ssr1552	GO:0055114	<1e-4	sll0268	GO:0042455	<1e-4	slr0771	GO:0016462	<1e-4	slr0806	GO:0044464	<1e-4	sll1902	GO:0004871	0.0445
sll0310	GO:0019751	<1e-4	sll0588	GO:0009056	<1e-4	slr0362	GO:0015291	<1e-4	sll1390	GO:0044424	<1e-4	sll0238	GO:0004871	0.0445
slr0082	GO:0008610	<1e-4	slr6074	GO:0044248	<1e-4	sll1926	GO:0015291	<1e-4	sll1995	GO:0044425	<1e-4	slr0208	GO:0004871	0.0445
slr7083	GO:0009124	<1e-4	sll1218	GO:0051347	<1e-4	slr1800	GO:0022891	<1e-4	slr1098	GO:0044464	<1e-4	sll1250	GO:0004871	0.0445
slr1484	GO:0006220	<1e-4	sll1388	GO:0006732	<1e-4	slr2018	GO:0015267	<1e-4	sll0780	GO:0044424	<1e-4	sll0702	GO:0004871	0.0445
slr0519	GO:0046394	<1e-4	sll7078	GO:0044248	<1e-4	sll0913	GO:0043167	<1e-4	sll1691	GO:0005575	<1e-4	sll0269	GO:0004871	0.0445
slr0869	GO:0071842	<1e-4	sll0442	GO:0022607	<1e-4	sll0930	GO:0015077	<1e-4	ssl2009	GO:0044425	<1e-4	sll0606	GO:0004871	0.0445
slr0272	GO:0009991	<1e-4	slr1062	GO:0019222	<1e-4	ssr3189	GO:0043492	<1e-4	sll1950	GO:0043226	<1e-4	sll0071	GO:0004871	0.0445
slr6075	GO:0006793	<1e-4	sll0451	GO:0043933	<1e-4	sll1763	GO:0008171	<1e-4	slr5112	GO:0043226	<1e-4	slr0356	GO:0008168	0.0442
sll1495	GO:0072528	<1e-4	slr0787	GO:0019222	<1e-4	sll1485	GO:0022891	<1e-4	sll0384	GO:0043226	<1e-4	sll0102	GO:0008168	0.0442

slr5126	GO:0009057	<1e-4	ssl2807	GO:0051179	<1e-4	slr0238	GO:0015405	<1e-4	sll0297	GO:0044464	<1e-4	sll8004	GO:0008168	0.0442
sll1532	GO:0019222	<1e-4	slr6004	GO:0044003	<1e-4	slr0655	GO:0016817	<1e-4	sll1203	GO:0044446	<1e-4	sll5047	GO:0008168	0.0442
sll0905	GO:0019751	<1e-4	ssr2781	GO:0009260	<1e-4	slr1547	GO:0022836	<1e-4	sll0846	GO:0044424	<1e-4	ssl2245	GO:0008168	0.0442
slr6101	GO:0046483	<1e-4	ssr2551	GO:0031326	<1e-4	sll1764	GO:0015077	<1e-4	sll5006	GO:0044446	<1e-4	sll0857	GO:0008168	0.0442
slr5024	GO:0019751	<1e-4	sll1319	GO:0006811	<1e-4	slr0863	GO:0043492	<1e-4	ssr5117	GO:0043226	<1e-4	slr0784	GO:0008168	0.0442
slr0172	GO:0016054	<1e-4	sll0282	GO:0043170	<1e-4	sll1563	GO:0015291	<1e-4	sll7087	GO:0005575	<1e-4	sll1853	GO:0008168	0.0442
slr0551	GO:0006631	<1e-4	slr2071	GO:0034641	<1e-4	slr7060	GO:0042623	<1e-4	sll7055	GO:0044444	<1e-4	slr1095	GO:0008168	0.0442
ssr3129	GO:0019222	<1e-4	slr0249	GO:0009161	<1e-4	sll5097	GO:0015291	<1e-4	sll0451	GO:0044446	<1e-4	sll1396	GO:0008168	0.0442
slr0692	GO:0006091	<1e-4	slr1230	GO:0051716	<1e-4	ssr3410	GO:0016746	<1e-4	sll0678	GO:0005575	<1e-4	slr1273	GO:0008168	0.0442
sll5004	GO:0080090	<1e-4	sll0031	GO:0009892	<1e-4	ssr1256	GO:0015077	<1e-4	sll1239	GO:0044446	<1e-4	slr0337	GO:0008168	0.0442
sll0930	GO:0051186	<1e-4	sll0609	GO:0031326	<1e-4	ssl1690	GO:0022892	<1e-4	slr0878	GO:0044422	<1e-4	sll1766	GO:0008168	0.0442
sll1273	GO:0065007	<1e-4	ssl8005	GO:0044283	<1e-4	sll0710	GO:0022832	<1e-4	slr2117	GO:0044424	<1e-4	slr1261	GO:0008168	0.0442
ssl1577	GO:0042455	<1e-4	sll8001	GO:0055086	<1e-4	slr0300	GO:0022832	<1e-4	slr7100	GO:0044425	<1e-4	sll1863	GO:0008168	0.0442
ssr6083	GO:0009892	<1e-4	sll1158	GO:0071841	<1e-4	sll0933	GO:0015077	<1e-4	slr0890	GO:0005575	<1e-4	ssl8003	GO:0008168	0.0442
sll5128	GO:0080090	<1e-4	slr1999	GO:0071842	<1e-4	ssr1407	GO:0043492	<1e-4	sll1469	GO:0044425	<1e-4	sll5044	GO:0008168	0.0442
sll1950	GO:0006066	<1e-4	sll0525	GO:0008610	<1e-4	sll1834	GO:0016462	<1e-4	sll0843	GO:0005575	<1e-4	ssl5045	GO:0008168	0.0442
slr0728	GO:0034654	<1e-4	sll0249	GO:0009263	<1e-4	sll0414	GO:0043167	<1e-4	slr1847	GO:0044444	<1e-4	ssl5095	GO:0008168	0.0442
sll1609	GO:0034654	<1e-4	slr0959	GO:0042180	<1e-4	sll0995	GO:0022857	<1e-4	sll0101	GO:0043226	<1e-4	slr0505	GO:0008168	0.0442
ssl5103	GO:0005996	<1e-4	sll1583	GO:0034654	<1e-4	sll0172	GO:0016817	<1e-4	slr0976	GO:0044446	<1e-4	ssl1004	GO:0008168	0.0442
sll5026	GO:0009889	<1e-4	sll7034	GO:0043436	<1e-4	sll0981	GO:0003674	<1e-4	sll0756	GO:0005575	<1e-4	sll1109	GO:0008168	0.0442
slr1194	GO:0044260	<1e-4	ssl0750	GO:0044248	<1e-4	slr7057	GO:0015405	<1e-4	slr1547	GO:0044444	<1e-4	sll1378	GO:0008168	0.0442
sll6054	GO:0009141	<1e-4	slr0196	GO:0006733	<1e-4	slr1570	GO:0015399	<1e-4	ssl1707	GO:0044446	<1e-4	slr0598	GO:0008168	0.0442
ssl7045	GO:0006091	<1e-4	sll0996	GO:0019222	<1e-4	slr2060	GO:0022892	<1e-4	ssr2060	GO:0044446	<1e-4	ssr2611	GO:0008168	0.0442
sll1123	GO:0034641	<1e-4	slr1203	GO:0019752	<1e-4	ssl3549	GO:0042623	<1e-4	sll1132	GO:0005575	<1e-4	sll1906	GO:0008168	0.0442
ssl2420	GO:0043933	<1e-4	slr0887	GO:0009892	<1e-4	ssr5074	GO:0022892	<1e-4	sll7065	GO:0044422	<1e-4	slr1600	GO:0008168	0.0442
slr0157	GO:0046164	<1e-4	sll0424	GO:0043412	<1e-4	sll1528	GO:0043167	<1e-4	slr1484	GO:0044422	<1e-4	sll1401	GO:0008168	0.0442
sll0325	GO:0046164	<1e-4	ssl8008	GO:0006576	<1e-4	slr1799	GO:0016818	<1e-4	sll0793	GO:0043226	<1e-4	sll0930	GO:0008168	0.0442
slr0272	GO:0034654	<1e-4	sll0066	GO:0019752	<1e-4	slr0172	GO:0015077	<1e-4	ssl5045	GO:0044464	<1e-4	sll0762	GO:0008168	0.0442
ssr5092	GO:0043412	<1e-4	sll1766	GO:0008150	<1e-4	ssl6092	GO:0016741	<1e-4	slr0459	GO:0044424	<1e-4	slr0959	GO:0008168	0.0442
slr0789	GO:0009199	<1e-4	sll1902	GO:0071496	<1e-4	slr0392	GO:0015075	<1e-4	sll8033	GO:0044425	<1e-4	sll8033	GO:0008168	0.0442
sll0740	GO:0006082	<1e-4	sll0298	GO:0051716	<1e-4	slr1397	GO:0042623	<1e-4	sll0553	GO:0044425	<1e-4	ssl0312	GO:0008168	0.0442
sll0736	GO:0046164	<1e-4	slr6038	GO:0006766	<1e-4	ssl5015	GO:0022890	<1e-4	slr0458	GO:0044446	<1e-4	sll0181	GO:0008168	0.0442
slr1546	GO:0016053	<1e-4	slr1752	GO:0009144	<1e-4	sll1853	GO:0060089	<1e-4	ssl0353	GO:0044444	<1e-4	sll7047	GO:0008168	0.0442
sll1089	GO:0009144	<1e-4	slr1378	GO:0022607	<1e-4	slr1495	GO:0016741	<1e-4	slr7071	GO:0044424	<1e-4	ssl5007	GO:0008168	0.0442
sll0269	GO:0090304	<1e-4	sll0284	GO:0009056	<1e-4	sll1289	GO:0015267	<1e-4	slr0065	GO:0005575	<1e-4	sll5062	GO:0008168	0.0442
slr0821	GO:0055114	<1e-4	sll0294	GO:0009987	<1e-4	sll2015	GO:0016818	<1e-4	slr0587	GO:0043226	<1e-4	slr0262	GO:0008168	0.0442
ssr3409	GO:0009308	<1e-4	sll1906	GO:0045184	<1e-4	sll1433	GO:0003674	<1e-4	slr0263	GO:0005575	<1e-4	sll0658	GO:0008168	0.0442
ssr5120	GO:0009892	<1e-4	slr1468	GO:0046394	<1e-4	sll8007	GO:0016818	<1e-4	ssl1417	GO:0005575	<1e-4	sll1265	GO:0008168	0.0442
ssr5121	GO:0019752	<1e-4	slr0613	GO:0009057	<1e-4	sll0552	GO:0022803	<1e-4	slr1591	GO:0044446	<1e-4	sll0473	GO:0008168	0.0442
slr0345	GO:0048878	<1e-4	sll0678	GO:0044106	<1e-4	slr1385	GO:0060089	<1e-4	slr2120	GO:0044425	<1e-4	sll1773	GO:0008168	0.0442
slr0569	GO:0031640	<1e-4	ssr2422	GO:0006793	<1e-4	sll0606	GO:0022891	<1e-4	sll1119	GO:0044444	<1e-4	ssl1464	GO:0008168	0.0442
ssl7074	GO:0019362	<1e-4	slr0498	GO:0006631	<1e-4	sll1318	GO:0022890	<1e-4	slr1196	GO:0044425	<1e-4	sll0008	GO:0008168	0.0442
slr1544	GO:0009056	<1e-4	slr0092	GO:0034654	<1e-4	ssl2009	GO:0015291	<1e-4	sll1054	GO:0044464	<1e-4	ssl1792	GO:0008168	0.0442
sll0442	GO:0051171	<1e-4	sll5109	GO:0051347	<1e-4	sll0294	GO:0022890	<1e-4	slr5023	GO:0044422	<1e-4	slr1207	GO:0008168	0.0442
slr6049	GO:0044248	<1e-4	slr0269	GO:0045184	<1e-4	sll1486	GO:0022838	<1e-4	slr1128	GO:0005575	<1e-4	slr1907	GO:0008168	0.0442
slr1068	GO:0034641	<1e-4	ssl2920	GO:0044275	<1e-4	sll7090	GO:0022857	<1e-4	slr0109	GO:0044464	<1e-4	sll1691	GO:0008168	0.0442
slr0607	GO:0019752	<1e-4	slr5037	GO:0042180	<1e-4	slr6021	GO:0022803	<1e-4	slr7095	GO:0044446	<1e-4	slr1923	GO:0008168	0.0442
sll1380	GO:0043412	<1e-4	sll0752	GO:0043549	<1e-4	ssl1255	GO:0016741	<1e-4	slr1627	GO:0044424	<1e-4	slr0196	GO:0008168	0.0442
sll1726	GO:0071554	<1e-4	ssl0467	GO:0048878	<1e-4	sll1609	GO:0016746	<1e-4	slr1442	GO:0043226	<1e-4	sll5097	GO:0008168	0.0442
sll0189	GO:0071554	<1e-4	slr1935	GO:0009132	<1e-4	slr0989	GO:0022832	<1e-4	sll0763	GO:0044464	<1e-4	slr0907	GO:0008168	0.0442
ssr2333	GO:0080090	<1e-4	slr0751	GO:0051171	<1e-4	sll1054	GO:0016462	<1e-4	slr1215	GO:0044424	<1e-4	ssr6020	GO:0008168	0.0442
sll0645	GO:0009150	<1e-4	sll1086	GO:0046164	<1e-4	sll1022	GO:0016741	<1e-4	slr5077	GO:0005575	<1e-4	ssr2422	GO:0008168	0.0442
slr6009	GO:0051234	<1e-4	slr0360	GO:0034654	<1e-4	ssl3829	GO:0022892	<1e-4	ssl1377	GO:0043226	<1e-4	sll0406	GO:0008168	0.0442
slr2004	GO:0048522	<1e-4	sll0047	GO:0051188	<1e-4	sll1866	GO:0008171	<1e-4	sll1609	GO:0044425	<1e-4	slr1998	GO:0008168	0.0442

ssr2803	GO:0046394	<1e-4	sll1495	GO:0055114	<1e-4	ssr3402	GO:0015399	<1e-4	sll1021	GO:0044425	<1e-4	slr0619	GO:0008168	0.0442
slr0356	GO:0080090	<1e-4	slr6067	GO:0071840	<1e-4	slr0935	GO:0022832	<1e-4	sll1715	GO:0044425	<1e-4	sll0803	GO:0008168	0.0442
ssl3142	GO:0044255	<1e-4	slr1544	GO:0050896	<1e-4	slr0587	GO:0016462	<1e-4	slr1384	GO:0044422	<1e-4	sll0710	GO:0008168	0.0442
slr1544	GO:0048518	<1e-4	slr1614	GO:0009141	<1e-4	ssl1417	GO:0022857	<1e-4	slr0613	GO:0044422	<1e-4	sll1638	GO:0008168	0.0442
slr1875	GO:0072524	<1e-4	sll0827	GO:0001932	<1e-4	ssl5113	GO:0015291	<1e-4	sll1063	GO:0043226	<1e-4	slr0552	GO:0008168	0.0442
slr0360	GO:0022607	<1e-4	ssr2554	GO:0043933	<1e-4	slr0468	GO:0043167	<1e-4	slr6067	GO:0044424	<1e-4	ssl2781	GO:0008168	0.0442
slr0489	GO:0022607	<1e-4	slr1052	GO:0005996	<1e-4	sll1188	GO:0043492	<1e-4	sll7062	GO:0044422	<1e-4	ssl1520	GO:0008168	0.0442
ssr1473	GO:0001932	<1e-4	sll2013	GO:0051179	<1e-4	slr6072	GO:0022891	<1e-4	slr6006	GO:0044446	<1e-4	sll1217	GO:0008168	0.0442
sll0382	GO:0009892	<1e-4	slr1383	GO:0044003	<1e-4	sll1926	GO:0022857	<1e-4	slr1812	GO:0044424	<1e-4	slr2105	GO:0008168	0.0442
sll0981	GO:0006082	<1e-4	slr1449	GO:0043436	<1e-4	sll0177	GO:0015075	<1e-4	slr1097	GO:0044424	<1e-4	sll7043	GO:0008168	0.0442
sll0732	GO:0050794	<1e-4	slr1964	GO:0051246	<1e-4	slr5119	GO:0015399	<1e-4	slr6081	GO:0005575	<1e-4	sgl0001	GO:0008168	0.0442
slr1612	GO:0006721	<1e-4	slr1704	GO:0060255	<1e-4	sll0085	GO:0015291	<1e-4	slr6009	GO:0043226	<1e-4	sll0727	GO:0008168	0.0442
slr1863	GO:0009056	<1e-4	slr1915	GO:0050896	<1e-4	slr1911	GO:0022832	<1e-4	sll1611	GO:0044464	<1e-4	slr7082	GO:0008168	0.0442
sll0843	GO:0010468	<1e-4	ssl1377	GO:0048518	<1e-4	ssr0102	GO:0022890	<1e-4	slr0269	GO:0044446	<1e-4	slr1576	GO:0008168	0.0442
slr0789	GO:0046394	<1e-4	ssr0332	GO:0019222	<1e-4	sll0623	GO:0015267	<1e-4	slr0195	GO:0044464	<1e-4	sll1511	GO:0008168	0.0442
sll6054	GO:0034660	<1e-4	slr0645	GO:0080090	<1e-4	slr2071	GO:0022892	<1e-4	sml0011	GO:0044425	<1e-4	slr6073	GO:0008168	0.0442
slr1376	GO:0022411	<1e-4	slr0483	GO:0044248	<1e-4	sll7065	GO:0022803	<1e-4	ssr0759	GO:0044464	<1e-4	sll0518	GO:0008168	0.0442
slr0871	GO:0009144	<1e-4	slr0601	GO:0031640	<1e-4	sll1201	GO:0022803	<1e-4	slr2052	GO:0044422	<1e-4	slr2101	GO:0008168	0.0442
sll0539	GO:0006220	<1e-4	slr0914	GO:0046164	<1e-4	sll1582	GO:0022838	<1e-4	slr0023	GO:0044444	<1e-4	slr0960	GO:0008168	0.0442
slr0416	GO:0051171	<1e-4	sll0786	GO:0043933	<1e-4	sll0685	GO:0015075	<1e-4	slr0397	GO:0044464	<1e-4	slr7096	GO:0008168	0.0442
sll1049	GO:0018130	<1e-4	ssr3129	GO:0009123	<1e-4	slr1398	GO:0015405	<1e-4	sll0863	GO:0005575	<1e-4	ssr6048	GO:0008168	0.0442
ssr1528	GO:0051818	<1e-4	slr0579	GO:0044106	<1e-4	sll0524	GO:0015075	<1e-4	sll1469	GO:0044444	<1e-4	ssl2717	GO:0008168	0.0442
slr1215	GO:0006725	<1e-4	slr1721	GO:0031326	<1e-4	slr0723	GO:0015293	<1e-4	ssr0109	GO:0005575	<1e-4	sll0913	GO:0008168	0.0442
sll1119	GO:0051246	<1e-4	sll0552	GO:0090304	<1e-4	slr8044	GO:0016746	<1e-4	slr0725	GO:0005575	<1e-4	sll7062	GO:0008168	0.0442
ssr0335	GO:0009987	<1e-4	slr0579	GO:0009309	<1e-4	sll1654	GO:0015075	<1e-4	ssr5011	GO:0044422	<1e-4	sll2013	GO:0008168	0.0442
sll1735	GO:0006091	<1e-4	slr0498	GO:0042180	<1e-4	sll0602	GO:0015077	<1e-4	slr0552	GO:0005575	<1e-4	slr1677	GO:0008168	0.0442
ssl3573	GO:0042180	<1e-4	slr1472	GO:0071496	<1e-4	slr0699	GO:0043167	<1e-4	slr1568	GO:0044425	<1e-4	slr0821	GO:0008168	0.0442
slr0305	GO:0006720	<1e-4	sll1775	GO:0048523	<1e-4	slr1170	GO:0016817	<1e-4	ssl5091	GO:0005575	<1e-4	ssl2148	GO:0008168	0.0442
sll0216	GO:0044275	<1e-4	slr1215	GO:0009161	<1e-4	ssr2142	GO:0022892	<1e-4	ssl5114	GO:0005575	<1e-4	slr7059	GO:0008168	0.0442
slr0888	GO:0050896	<1e-4	slr0815	GO:0080090	<1e-4	slr1365	GO:0015267	<1e-4	slr5017	GO:0044422	<1e-4	ssr2912	GO:0008168	0.0442
sll0482	GO:0055086	<1e-4	sll1359	GO:0034641	<1e-4	slr1288	GO:0022857	<1e-4	slr0345	GO:0044424	<1e-4	sll5061	GO:0008168	0.0442
sll0793	GO:0044275	<1e-4	slr5053	GO:0006220	<1e-4	slr1618	GO:0016746	<1e-4	sll0183	GO:0044446	<1e-4	slr1863	GO:0008168	0.0442
slr7096	GO:0009259	<1e-4	slr5053	GO:0071840	<1e-4	sll1304	GO:0060089	<1e-4	sll0997	GO:0005575	<1e-4	sll5046	GO:0008168	0.0442
slr7023	GO:0055086	<1e-4	slr2110	GO:0009199	<1e-4	sll1530	GO:0015405	<1e-4	slr1537	GO:0044424	<1e-4	sll1862	GO:0008168	0.0442
slr6013	GO:0051188	<1e-4	sll0545	GO:0051716	<1e-4	sll0737	GO:0005342	<1e-4	sll1201	GO:0044422	<1e-4	slr5119	GO:0008168	0.0442
ssl5015	GO:0006733	<1e-4	ssl2420	GO:0008150	<1e-4	sll1995	GO:0022803	<1e-4	slr0637	GO:0044444	<1e-4	slr6101	GO:0008168	0.0442
sll1054	GO:0009262	<1e-4	ssl3383	GO:0010556	<1e-4	slr1306	GO:0016818	<1e-4	slr0789	GO:0044464	<1e-4	ssr3189	GO:0008168	0.0442
slr1530	GO:0019751	<1e-4	sll0886	GO:0046394	<1e-4	ssr5011	GO:0015267	<1e-4	slr0569	GO:0005575	<1e-4	sll1757	GO:0008168	0.0442
ssr2787	GO:0006091	<1e-4	ssr6019	GO:0006793	<1e-4	sll5034	GO:0003674	<1e-4	ssl5008	GO:0044422	<1e-4	slr0388	GO:0008168	0.0442
sll1891	GO:0022607	<1e-4	sll1942	GO:0006576	<1e-4	slr6033	GO:0022857	<1e-4	slr0711	GO:0005575	<1e-4	slr1927	GO:0008168	0.0442
ssr6099	GO:0019222	<1e-4	sll0584	GO:0050896	<1e-4	sll0448	GO:0022890	<1e-4	sll0062	GO:0005575	<1e-4	ssl2471	GO:0008168	0.0442
sll1912	GO:0045184	<1e-4	slr1790	GO:0044248	<1e-4	slr1143	GO:0043492	<1e-4	slr0360	GO:0005575	<1e-4	sll0498	GO:0008168	0.0442
sll0608	GO:0019751	<1e-4	sll1304	GO:0034660	<1e-4	ssr2551	GO:0015075	<1e-4	ssl1046	GO:0005575	<1e-4	sll0815	GO:0008168	0.0442
slr1493	GO:0044271	<1e-4	sll0857	GO:0008150	<1e-4	sll0803	GO:0016818	<1e-4	sll0853	GO:0044424	<1e-4	sll0811	GO:0008168	0.0442
sll0272	GO:0046128	<1e-4	sll0301	GO:0006163	<1e-4	ssr1558	GO:0022838	<1e-4	slr0913	GO:0043226	<1e-4	sll1884	GO:0008168	0.0442
sll5006	GO:0006766	<1e-4	slr1127	GO:0042455	<1e-4	slr0157	GO:0022890	<1e-4	sll0623	GO:0044444	<1e-4	sll5090	GO:0008168	0.0442
slr1827	GO:0006811	<1e-4	sll0008	GO:0055086	<1e-4	slr0642	GO:0015293	<1e-4	slr1235	GO:0044464	<1e-4	sll7063	GO:0008168	0.0442
sll1469	GO:0031323	<1e-4	slr6087	GO:0090304	<1e-4	slr1230	GO:0022891	<1e-4	ssl2064	GO:0005575	<1e-4	slr7011	GO:0008168	0.0442
sll0031	GO:0050896	<1e-4	sll0736	GO:0071840	<1e-4	ssl5015	GO:0016462	<1e-4	slr1143	GO:0043226	<1e-4	ssl0738	GO:0008168	0.0442
slr7023	GO:0022607	<1e-4	sll0545	GO:0006091	<1e-4	ssr1473	GO:0015077	<1e-4	slr0907	GO:0044422	<1e-4	slr7080	GO:0008168	0.0442
sll0007	GO:0016053	<1e-4	sll0319	GO:0052111	<1e-4	sll7043	GO:0022857	<1e-4	slr1343	GO:0044464	<1e-4	slr7012	GO:0008168	0.0442
slr1210	GO:0043170	<1e-4	ssr7035	GO:0009117	<1e-4	sgl0001	GO:0015293	<1e-4	sll1696	GO:0043226	<1e-4	ssr2049	GO:0003723	0.0435
slr1270	GO:0065007	<1e-4	sll0647	GO:0019751	<1e-4	slr0172	GO:0022857	<1e-4	ssr1951	GO:0044424	<1e-4	slr0491	GO:0003723	0.0435
slr6081	GO:0072527	<1e-4	ssr3300	GO:0043412	<1e-4	slr1378	GO:0022832	<1e-4	slr1152	GO:0043226	<1e-4	slr1385	GO:0003723	0.0435

ssl7039	GO:0009056	<1e-4	ssr6027	GO:0060255	<1e-4	sll0843	GO:0016817	<1e-4	slr0731	GO:0044464	<1e-4	ssl1255	GO:0003723	0.0435
ssl5015	GO:0044248	<1e-4	sll0176	GO:0010556	<1e-4	sll1757	GO:0005342	<1e-4	slr0587	GO:0044425	<1e-4	sll0661	GO:0003723	0.0435
sll0688	GO:0009132	<1e-4	sll1222	GO:0022411	<1e-4	sll0423	GO:0042623	<1e-4	ssr3154	GO:0044422	<1e-4	slr1603	GO:0003723	0.0435
slr0552	GO:0016054	<1e-4	ssr1041	GO:0051188	<1e-4	slr0729	GO:0022891	<1e-4	sll0505	GO:0005575	<1e-4	sll0242	GO:0017111	0.0432
sll0931	GO:0051188	<1e-4	sll5026	GO:0045184	<1e-4	sll0606	GO:0022857	<1e-4	ssl2009	GO:0044446	<1e-4	sll0688	GO:0005975	0.0429
slr0148	GO:0006082	<1e-4	slr1513	GO:0044003	<1e-4	slr1394	GO:0008171	<1e-4	slr0784	GO:0044422	<1e-4	slr0207	GO:0005975	0.0429
slr0755	GO:0016053	<1e-4	sll0181	GO:0009199	<1e-4	slr1690	GO:0016746	<1e-4	ssl0294	GO:0044464	<1e-4	sll0319	GO:0005975	0.0429
slr0787	GO:0008150	<1e-4	slr1886	GO:0019222	<1e-4	slr1907	GO:0016817	<1e-4	sll0788	GO:0044425	<1e-4	slr0957	GO:0005975	0.0429
sll0160	GO:0044282	<1e-4	slr0964	GO:0072521	<1e-4	slr0172	GO:0015267	<1e-4	slr1107	GO:0044464	<1e-4	sll0572	GO:0005975	0.0429
slr0299	GO:0051347	<1e-4	slr1110	GO:0046164	<1e-4	ssr6032	GO:0003674	<1e-4	slr1495	GO:0005575	<1e-4	sll1659	GO:0005975	0.0429
sll1638	GO:0006732	<1e-4	slr1977	GO:0071842	<1e-4	ssr6003	GO:0043492	<1e-4	slr1851	GO:0043226	<1e-4	ssr2009	GO:0005975	0.0429
slr0058	GO:0044275	<1e-4	sll1203	GO:0071840	<1e-4	slr7060	GO:0022838	<1e-4	ssr3571	GO:0005575	<1e-4	slr0975	GO:0005975	0.0429
sll5034	GO:0001932	<1e-4	ssr7084	GO:0090304	<1e-4	slr0398	GO:0016741	<1e-4	sll0024	GO:0005575	<1e-4	slr0801	GO:0005975	0.0429
slr1956	GO:0019637	<1e-4	slr0179	GO:0006753	<1e-4	sll0609	GO:0022857	<1e-4	slr1679	GO:0044444	<1e-4	slr0169	GO:0005975	0.0429
sll0283	GO:0046164	<1e-4	slr0291	GO:0006066	<1e-4	sll0185	GO:0022857	<1e-4	sll1680	GO:0043226	<1e-4	slr1338	GO:0005975	0.0429
sll5132	GO:0080090	<1e-4	sll0803	GO:0050794	<1e-4	slr0285	GO:0016746	<1e-4	sll0871	GO:0043226	<1e-4	sll0281	GO:0005975	0.0429
sll0272	GO:0090304	<1e-4	sll0160	GO:0009141	<1e-4	slr1493	GO:0022836	<1e-4	slr0480	GO:0005575	<1e-4	sll1411	GO:0005975	0.0429
sll0208	GO:0016053	<1e-4	sll1155	GO:0022607	<1e-4	sll0243	GO:0022832	<1e-4	slr1660	GO:0005575	<1e-4	sll1267	GO:0005975	0.0429
sll5032	GO:0006091	<1e-4	ssr3189	GO:0043436	<1e-4	slr0545	GO:0015293	<1e-4	sll0982	GO:0005575	<1e-4	slr1070	GO:0005975	0.0429
ssr2060	GO:0042180	<1e-4	sll1640	GO:0009308	<1e-4	sll0863	GO:0015075	<1e-4	ssl0352	GO:0005575	<1e-4	slr1809	GO:0005975	0.0429
sll0552	GO:0009987	<1e-4	slr0619	GO:0072528	<1e-4	sll1272	GO:0016817	<1e-4	slr1189	GO:0044464	<1e-4	slr0605	GO:0005975	0.0429
slr0869	GO:0009056	<1e-4	slr1362	GO:0009056	<1e-4	sll1939	GO:0016462	<1e-4	slr2012	GO:0044444	<1e-4	slr0924	GO:0005975	0.0429
slr2018	GO:0050789	<1e-4	slr0491	GO:0006066	<1e-4	slr7102	GO:0022891	<1e-4	sll1381	GO:0044446	<1e-4	sll0980	GO:0005975	0.0429
sll0325	GO:0060255	<1e-4	sll1766	GO:0044283	<1e-4	slr0971	GO:0042623	<1e-4	slr1681	GO:0044422	<1e-4	slr0149	GO:0005975	0.0429
ssr5011	GO:0006091	<1e-4	sll0314	GO:0071840	<1e-4	slr7024	GO:0015291	<1e-4	slr0780	GO:0005575	<1e-4	slr7024	GO:0005975	0.0429
sll0478	GO:0009150	<1e-4	ssr2972	GO:0034641	<1e-4	ssr5092	GO:0015075	<1e-4	slr0181	GO:0044446	<1e-4	slr1258	GO:0005975	0.0429
sll7064	GO:0010468	<1e-4	sll0710	GO:0042455	<1e-4	sll1218	GO:0022857	<1e-4	slr0699	GO:0005575	<1e-4	sll1654	GO:0005975	0.0429
sll0238	GO:0050789	<1e-4	slr7083	GO:0071496	<1e-4	slr6028	GO:0005342	<1e-4	ssr2553	GO:0044464	<1e-4	sll0810	GO:0005975	0.0429
sll1004	GO:0071842	<1e-4	slr1847	GO:0046128	<1e-4	slr0416	GO:0022832	<1e-4	sll1757	GO:0044446	<1e-4	slr0654	GO:0005975	0.0429
ssl2015	GO:0009394	<1e-4	sll1611	GO:0055086	<1e-4	slr1958	GO:0008171	<1e-4	slr1470	GO:0044424	<1e-4	sll0188	GO:0005975	0.0429
sll5033	GO:0001932	<1e-4	slr1406	GO:0043170	<1e-4	slr0613	GO:0015077	<1e-4	ssr2047	GO:0005575	<1e-4	ssr1528	GO:0005975	0.0429
sll5097	GO:0010556	<1e-4	slr7071	GO:0009260	<1e-4	slr1913	GO:0022803	<1e-4	slr7098	GO:0005575	<1e-4	sll1021	GO:0005975	0.0429
ssl3142	GO:0006721	<1e-4	sll0071	GO:0050789	<1e-4	sll1400	GO:0003674	<1e-4	slr0204	GO:0044464	<1e-4	slr0989	GO:0005975	0.0429
slr1571	GO:0009263	<1e-4	slr0930	GO:0019637	<1e-4	slr2103	GO:0022891	<1e-4	ssr1765	GO:0043226	<1e-4	sll0189	GO:0005975	0.0429
slr6039	GO:0048522	<1e-4	slr1074	GO:0006082	<1e-4	slr0914	GO:0005342	<1e-4	ssr1041	GO:0044425	<1e-4	sll0911	GO:0005975	0.0429
slr2003	GO:0043170	<1e-4	ssl2064	GO:0051234	<1e-4	slr1178	GO:0008171	<1e-4	sll1442	GO:0044425	<1e-4	slr0468	GO:0005975	0.0429
sll7066	GO:0009056	<1e-4	slr0108	GO:0016052	<1e-4	ssr2551	GO:0022857	<1e-4	slr6068	GO:0044446	<1e-4	slr0060	GO:0005975	0.0429
sll0376	GO:0055082	<1e-4	slr0204	GO:0009987	<1e-4	sll1219	GO:0022832	<1e-4	sll1656	GO:0005575	<1e-4	sll0297	GO:0005975	0.0429
sll0532	GO:0006066	<1e-4	slr2071	GO:0045184	<1e-4	sll1063	GO:0060089	<1e-4	slr6063	GO:0044446	<1e-4	slr1658	GO:0005975	0.0429
slr6081	GO:0065007	<1e-4	slr1270	GO:0044106	<1e-4	slr6073	GO:0022803	<1e-4	slr1603	GO:0044424	<1e-4	slr1977	GO:0005975	0.0429
sll0410	GO:0019751	<1e-4	sll1252	GO:0042451	<1e-4	sll1608	GO:0003674	<1e-4	ssr2422	GO:0044444	<1e-4	sll1730	GO:0005975	0.0429
slr0192	GO:0051179	<1e-4	sll1509	GO:0031323	<1e-4	slr6044	GO:0016817	<1e-4	slr0303	GO:0044424	<1e-4	slr1303	GO:0005975	0.0429
ssr3122	GO:0031323	<1e-4	ssr3572	GO:0052188	<1e-4	slr1920	GO:0042623	<1e-4	slr5111	GO:0044425	<1e-4	slr0789	GO:0005975	0.0429
ssl2733	GO:0009260	<1e-4	slr0845	GO:0006793	<1e-4	slr7015	GO:0016818	<1e-4	ssr7072	GO:0044424	<1e-4	sll0658	GO:0016740	0.0428
slr1062	GO:0072528	<1e-4	slr6014	GO:0019752	<1e-4	slr1864	GO:0015405	<1e-4	slr0487	GO:0044464	<1e-4	slr1195	GO:0016740	0.0428
slr0509	GO:0009059	<1e-4	sll1512	GO:0090304	<1e-4	sll1765	GO:0015075	<1e-4	sll1040	GO:0005575	<1e-4	sll1132	GO:0016740	0.0428
slr1429	GO:0042180	<1e-4	slr1677	GO:0010556	<1e-4	sll0811	GO:0022838	<1e-4	sll0414	GO:0044422	<1e-4	slr1660	GO:0016740	0.0428
slr1658	GO:0048519	<1e-4	ssr2553	GO:0043933	<1e-4	slr2101	GO:0022857	<1e-4	sll0751	GO:0044425	<1e-4	sll1764	GO:0016740	0.0428
slr6091	GO:0019222	<1e-4	sll0448	GO:0044255	<1e-4	sll1511	GO:0016817	<1e-4	ssl2920	GO:0044424	<1e-4	slr2105	GO:0016740	0.0428
slr1593	GO:0046164	<1e-4	ssr1766	GO:0051234	<1e-4	sll1942	GO:0043492	<1e-4	sll0545	GO:0044422	<1e-4	sll1062	GO:0016740	0.0428
slr1082	GO:0072524	<1e-4	slr6008	GO:0009165	<1e-4	sll7063	GO:0015405	<1e-4	sll1241	GO:0044464	<1e-4	slr1534	GO:0016740	0.0428
ssl0350	GO:0022607	<1e-4	slr0885	GO:0009308	<1e-4	slr0326	GO:0022890	<1e-4	sll0615	GO:0044464	<1e-4	slr1068	GO:0016740	0.0428
slr0975	GO:0071840	<1e-4	slr1541	GO:0051179	<1e-4	sll1652	GO:0015405	<1e-4	slr7011	GO:0044425	<1e-4	ssl2420	GO:0016740	0.0428
slr1110	GO:0051234	<1e-4	ssr8047	GO:0044255	<1e-4	sll1563	GO:0060089	<1e-4	sll1510	GO:0044446	<1e-4	sll0048	GO:0016740	0.0428

sll1858	GO:0031326	<1e-4	slr1425	GO:0009124	<1e-4	ssl1263	GO:0015075	<1e-4	slr0006	GO:0044464	<1e-4	sll0241	GO:0016740	0.0428
slr2038	GO:0046907	<1e-4	slr1519	GO:0016053	<1e-4	ssl7074	GO:0015267	<1e-4	slr0362	GO:0044444	<1e-4	slr1600	GO:0016740	0.0428
slr7026	GO:0080090	<1e-4	sll1510	GO:0051716	<1e-4	slr0667	GO:0016817	<1e-4	sll0325	GO:0044422	<1e-4	slr1923	GO:0016740	0.0428
sll0565	GO:0016052	<1e-4	sll0328	GO:0006066	<1e-4	slr1668	GO:0022892	<1e-4	sll1763	GO:0044422	<1e-4	slr1207	GO:0016740	0.0428
ssl2069	GO:0010468	<1e-4	slr0362	GO:0006766	<1e-4	sll1062	GO:0015267	<1e-4	slr6045	GO:0044422	<1e-4	slr0264	GO:0016740	0.0428
sll1218	GO:0006811	<1e-4	slr0109	GO:0009165	<1e-4	sll1832	GO:0015405	<1e-4	slr0348	GO:0005575	<1e-4	sll0473	GO:0016740	0.0428
sll1040	GO:0019222	<1e-4	ssr7079	GO:0048519	<1e-4	slr1104	GO:0022892	<1e-4	sll0780	GO:0044446	<1e-4	slr6100	GO:0016740	0.0428
sll1511	GO:0019219	<1e-4	slr7026	GO:0019219	<1e-4	sll0023	GO:0016741	<1e-4	sll2013	GO:0005575	<1e-4	slr1813	GO:0016740	0.0428
slr1114	GO:0009199	<1e-4	sll0803	GO:0043412	<1e-4	ssl2595	GO:0016817	<1e-4	slr0364	GO:0044422	<1e-4	slr1365	GO:0016740	0.0428
sll0382	GO:0034654	<1e-4	ssr1499	GO:0009199	<1e-4	slr6033	GO:0015293	<1e-4	ssr6085	GO:0043226	<1e-4	slr6074	GO:0016740	0.0428
slr1186	GO:0009124	<1e-4	sll7031	GO:0019751	<1e-4	sll1218	GO:0022832	<1e-4	slr1613	GO:0044425	<1e-4	slr5018	GO:0016740	0.0428
slr1079	GO:0019362	<1e-4	slr1812	GO:0055114	<1e-4	slr1668	GO:0022832	<1e-4	slr0941	GO:0043226	<1e-4	slr1261	GO:0016740	0.0428
sll1512	GO:0019752	<1e-4	sll1052	GO:0042451	<1e-4	sll0539	GO:0022890	<1e-4	slr1573	GO:0044425	<1e-4	slr0907	GO:0016740	0.0428
slr6063	GO:0006732	<1e-4	sll1763	GO:0008610	<1e-4	slr5053	GO:0043167	<1e-4	sll1995	GO:0044444	<1e-4	slr0960	GO:0016740	0.0428
sll1201	GO:0051234	<1e-4	ssr3129	GO:0048518	<1e-4	slr1128	GO:0043167	<1e-4	slr0272	GO:0044444	<1e-4	ssl2781	GO:0016740	0.0428
slr0285	GO:0009126	<1e-4	sll0780	GO:0009199	<1e-4	slr6006	GO:0015077	<1e-4	sll2013	GO:0043226	<1e-4	slr1263	GO:0016740	0.0428
slr1122	GO:0090304	<1e-4	sll1433	GO:0009056	<1e-4	ssl0750	GO:0022890	<1e-4	sll1939	GO:0005575	<1e-4	sll1961	GO:0016740	0.0428
sll0497	GO:0048519	<1e-4	slr1964	GO:0042180	<1e-4	sll1769	GO:0015075	<1e-4	slr0770	GO:0005575	<1e-4	slr1566	GO:0016740	0.0428
slr7097	GO:0009260	<1e-4	sll1714	GO:0006811	<1e-4	sll1106	GO:0043167	<1e-4	slr0064	GO:0044464	<1e-4	sll0176	GO:0016740	0.0428
slr0404	GO:0072522	<1e-4	sml0011	GO:0034660	<1e-4	sll7077	GO:0015075	<1e-4	ssr3467	GO:0044464	<1e-4	slr7023	GO:0016740	0.0428
slr1415	GO:0008610	<1e-4	slr2117	GO:0048518	<1e-4	ssl0483	GO:0022838	<1e-4	ssl5065	GO:0043226	<1e-4	slr0142	GO:0016740	0.0428
slr8022	GO:0052111	<1e-4	sll1601	GO:0051234	<1e-4	sll0281	GO:0005342	<1e-4	sll1293	GO:0005575	<1e-4	slr7011	GO:0016740	0.0428
sll0354	GO:0019362	<1e-4	slr1194	GO:0006811	<1e-4	sll1863	GO:0043167	<1e-4	sll1651	GO:0005575	<1e-4	sll1696	GO:0016740	0.0428
sll0524	GO:0048878	<1e-4	slr6038	GO:0009308	<1e-4	slr1900	GO:0043167	<1e-4	sll1969	GO:0044446	<1e-4	sll1378	GO:0016740	0.0428
slr1406	GO:0009262	<1e-4	slr1449	GO:0051716	<1e-4	slr0112	GO:0043492	<1e-4	sll1273	GO:0044425	<1e-4	ssl5098	GO:0016740	0.0428
slr0082	GO:0044283	<1e-4	slr0360	GO:0001932	<1e-4	ssr6099	GO:0015293	<1e-4	slr6072	GO:0005575	<1e-4	sll1348	GO:0016740	0.0428
sll0982	GO:0016052	<1e-4	ssr6085	GO:0006720	<1e-4	sll1797	GO:0022803	<1e-4	sll7067	GO:0044424	<1e-4	slr6021	GO:0016740	0.0428
sll0296	GO:0006793	<1e-4	slr1084	GO:0009144	<1e-4	sll0596	GO:0005342	<1e-4	ssl7051	GO:0044422	<1e-4	sll0518	GO:0016740	0.0428
slr1704	GO:0009150	<1e-4	sll0410	GO:0019637	<1e-4	slr0869	GO:0003674	<1e-4	sll8007	GO:0044444	<1e-4	slr1998	GO:0016740	0.0428
slr1681	GO:0009141	<1e-4	sll0319	GO:0031323	<1e-4	sll1609	GO:0003674	<1e-4	sll1541	GO:0005575	<1e-4	sll0742	GO:0016740	0.0428
sll0062	GO:0071842	<1e-4	sll1192	GO:0048519	<1e-4	slr1932	GO:0016817	<1e-4	sll1654	GO:0005575	<1e-4	sll1389	GO:0016740	0.0428
sll1233	GO:0033013	<1e-4	ssr1499	GO:0043933	<1e-4	sll0740	GO:0022803	<1e-4	sll1192	GO:0044464	<1e-4	sll1160	GO:0016740	0.0428
sll0558	GO:0009150	<1e-4	ssr2781	GO:0044260	<1e-4	sll7050	GO:0016818	<1e-4	slr1365	GO:0044444	<1e-4	sll5034	GO:0016740	0.0428
sll0737	GO:0009117	<1e-4	slr1568	GO:0022607	<1e-4	slr0082	GO:0043167	<1e-4	slr1611	GO:0044464	<1e-4	slr8014	GO:0016740	0.0428
slr0651	GO:0006066	<1e-4	ssl5091	GO:0043170	<1e-4	slr1660	GO:0022892	<1e-4	slr1127	GO:0043226	<1e-4	slr1384	GO:0016740	0.0428
ssr2848	GO:0009124	<1e-4	slr0553	GO:0009141	<1e-4	slr1917	GO:0016462	<1e-4	sll0780	GO:0044444	<1e-4	sll1162	GO:0016740	0.0428
sll1735	GO:0051179	<1e-4	sll7050	GO:0006576	<1e-4	sll0944	GO:0016817	<1e-4	slr7016	GO:0044424	<1e-4	sll5097	GO:0016740	0.0428
slr0380	GO:0052111	<1e-4	slr0364	GO:0046164	<1e-4	slr0810	GO:0015291	<1e-4	slr5119	GO:0044422	<1e-4	slr6008	GO:0016740	0.0428
ssr3402	GO:0031326	<1e-4	ssr3122	GO:0009309	<1e-4	sll0595	GO:0022857	<1e-4	slr1419	GO:0043226	<1e-4	ssr2912	GO:0016740	0.0428
slr1644	GO:0009056	<1e-4	sll1344	GO:0009199	<1e-4	sll1652	GO:0022832	<1e-4	ssr2317	GO:0044424	<1e-4	slr5116	GO:0016740	0.0428
sll0175	GO:0019637	<1e-4	slr0914	GO:0009892	<1e-4	slr2038	GO:0042623	<1e-4	slr0356	GO:0044425	<1e-4	sll5044	GO:0016740	0.0428
sll0910	GO:0006753	<1e-4	slr1218	GO:0043648	<1e-4	ssr8047	GO:0043167	<1e-4	slr7060	GO:0044464	<1e-4	sll0659	GO:0016740	0.0428
slr1394	GO:0022607	<1e-4	sll1025	GO:0009056	<1e-4	ssr5120	GO:0022890	<1e-4	sll0984	GO:0044424	<1e-4	slr6065	GO:0016740	0.0428
ssr3572	GO:0044248	<1e-4	sll0243	GO:0090304	<1e-4	slr0060	GO:0060089	<1e-4	ssr2848	GO:0005575	<1e-4	slr0964	GO:0016740	0.0428
sll0319	GO:0006721	<1e-4	sll0481	GO:0050801	<1e-4	ssr6020	GO:0022832	<1e-4	slr2048	GO:0005575	<1e-4	slr1484	GO:0016740	0.0428
sll7089	GO:0051234	<1e-4	sll0268	GO:0044281	<1e-4	sll0994	GO:0043492	<1e-4	slr5111	GO:0005575	<1e-4	sll8007	GO:0016740	0.0428
sll0160	GO:0009124	<1e-4	slr0552	GO:0009263	<1e-4	slr0112	GO:0022836	<1e-4	slr0092	GO:0005575	<1e-4	slr6073	GO:0016740	0.0428
slr1572	GO:0051246	<1e-4	sll0678	GO:0071842	<1e-4	slr5077	GO:0015267	<1e-4	sll1319	GO:0005575	<1e-4	ssr6048	GO:0016740	0.0428
slr1537	GO:0009126	<1e-4	slr1266	GO:0043648	<1e-4	ssr0692	GO:0022857	<1e-4	ssl2733	GO:0044444	<1e-4	slr0148	GO:0016740	0.0428
slr0596	GO:0045184	<1e-4	slr0645	GO:0016052	<1e-4	ssl3142	GO:0015405	<1e-4	sll1915	GO:0044424	<1e-4	slr7082	GO:0016740	0.0428
sll1509	GO:0043412	<1e-4	sll1726	GO:0006721	<1e-4	slr1437	GO:0015267	<1e-4	sll0763	GO:0044424	<1e-4	slr0049	GO:0016740	0.0428
sll0503	GO:0048878	<1e-4	slr1813	GO:0051186	<1e-4	sll1961	GO:0016818	<1e-4	sll0325	GO:0044444	<1e-4	slr1103	GO:0016740	0.0428
slr0981	GO:0006811	<1e-4	sll7089	GO:0043436	<1e-4	ssr0759	GO:0022890	<1e-4	ssr1698	GO:0044444	<1e-4	ssl3383	GO:0016740	0.0428
slr1926	GO:0050789	<1e-4	sll0230	GO:0006811	<1e-4	slr0157	GO:0016462	<1e-4	sll1825	GO:0005575	<1e-4	sll1638	GO:0016740	0.0428

ssl3383	GO:0009889	<1e-4	sll5003	GO:0044003	<1e-4	slr2121	GO:0015077	<1e-4	sll0532	GO:0044425	<1e-4	sll0727	GO:0016740	0.0428
slr0651	GO:0031326	<1e-4	sll1414	GO:0044260	<1e-4	ssr6002	GO:0016818	<1e-4	sll0319	GO:0005575	<1e-4	sll7063	GO:0016740	0.0428
slr1103	GO:0055086	<1e-4	sll0645	GO:0051246	<1e-4	ssr6085	GO:0043167	<1e-4	sll0060	GO:0005575	<1e-4	slr6072	GO:0016740	0.0428
slr0967	GO:0043933	<1e-4	sll1052	GO:0034641	<1e-4	ssl7039	GO:0022832	<1e-4	sll1916	GO:0005575	<1e-4	slr0489	GO:0016740	0.0428
slr1927	GO:0006721	<1e-4	ssr1425	GO:0043412	<1e-4	slr0980	GO:0016818	<1e-4	sll0608	GO:0044425	<1e-4	slr0145	GO:0016740	0.0428
slr1441	GO:0051716	<1e-4	slr0065	GO:0044248	<1e-4	sll0563	GO:0015267	<1e-4	sll0443	GO:0044446	<1e-4	slr1363	GO:0016740	0.0428
slr0053	GO:0010468	<1e-4	ssr6003	GO:0051246	<1e-4	sll1656	GO:0016817	<1e-4	slr0313	GO:0044444	<1e-4	slr0058	GO:0016740	0.0428
slr0496	GO:0055086	<1e-4	sll1062	GO:0006766	<1e-4	ssr7079	GO:0015075	<1e-4	slr0376	GO:0043226	<1e-4	sll0265	GO:0016740	0.0428
ssl8003	GO:0009262	<1e-4	slr1470	GO:0009263	<1e-4	ssl0750	GO:0015291	<1e-4	slr2105	GO:0044444	<1e-4	slr1927	GO:0016740	0.0428
sll1476	GO:0042180	<1e-4	slr8022	GO:0034660	<1e-4	sll1025	GO:0022832	<1e-4	slr0801	GO:0044425	<1e-4	slr7083	GO:0016740	0.0428
sll1692	GO:0080090	<1e-4	sll0905	GO:0019222	<1e-4	ssl5091	GO:0005342	<1e-4	sll1426	GO:0005575	<1e-4	slr1260	GO:0016740	0.0428
slr0458	GO:0050794	<1e-4	slr1187	GO:0043648	<1e-4	sll0177	GO:0042623	<1e-4	ssr1258	GO:0044446	<1e-4	sll1921	GO:0016740	0.0428
sll7055	GO:0044255	<1e-4	sll1004	GO:0042451	<1e-4	sll1068	GO:0005342	<1e-4	sll1061	GO:0005575	<1e-4	slr0356	GO:0016740	0.0428
ssl3829	GO:0072527	<1e-4	ssl5129	GO:0055082	<1e-4	sll0168	GO:0003674	<1e-4	ssr7079	GO:0044422	<1e-4	sll0397	GO:0016740	0.0428
sll0641	GO:0006730	<1e-4	slr7037	GO:0046907	<1e-4	slr1396	GO:0016817	<1e-4	sll0740	GO:0044424	<1e-4	slr0337	GO:0005975	0.0418
slr1306	GO:0046907	<1e-4	sll0360	GO:0006066	<1e-4	slr0076	GO:0060089	<1e-4	sll0406	GO:0044424	<1e-4	slr0505	GO:0005975	0.0418
slr0423	GO:0048519	<1e-4	sll7077	GO:0010468	<1e-4	sll0280	GO:0022891	<1e-4	slr1546	GO:0044444	<1e-4	slr0907	GO:0005975	0.0418
sll7086	GO:0051716	<1e-4	sll5034	GO:0006811	<1e-4	slr0989	GO:0043492	<1e-4	slr1210	GO:0044444	<1e-4	ssl2781	GO:0005975	0.0418
sll1062	GO:0034654	<1e-4	slr0479	GO:0008150	<1e-4	ssr2998	GO:0022890	<1e-4	ssr6024	GO:0043226	<1e-4	sll1022	GO:0005975	0.0418
slr7013	GO:0050789	<1e-4	slr7091	GO:0051171	<1e-4	sll7047	GO:0022857	<1e-4	slr6051	GO:0044464	<1e-4	sll0174	GO:0005975	0.0418
slr7015	GO:0008150	<1e-4	slr1262	GO:0006733	<1e-4	sll0272	GO:0022803	<1e-4	slr8022	GO:0005575	<1e-4	slr0362	GO:0005975	0.0418
slr1623	GO:0051186	<1e-4	slr1530	GO:0071554	<1e-4	slr0742	GO:0016818	<1e-4	slr6044	GO:0044446	<1e-4	ssr6026	GO:0005975	0.0418
sll1934	GO:0009144	<1e-4	slr0607	GO:0044248	<1e-4	slr1413	GO:0008171	<1e-4	sll0162	GO:0044446	<1e-4	sll7047	GO:0005975	0.0418
sll1052	GO:0008150	<1e-4	slr0789	GO:0051188	<1e-4	ssl1792	GO:0042623	<1e-4	sll1173	GO:0044425	<1e-4	sll0406	GO:0005975	0.0418
sll1915	GO:0009056	<1e-4	slr1240	GO:0043170	<1e-4	ssl5108	GO:0022832	<1e-4	sll7043	GO:0044444	<1e-4	slr1260	GO:0005975	0.0418
sll1252	GO:0051171	<1e-4	sll7066	GO:0042451	<1e-4	sll1632	GO:0015267	<1e-4	ssr2848	GO:0044425	<1e-4	slr0569	GO:0005975	0.0418
sll0611	GO:0050801	<1e-4	sll0225	GO:0044248	<1e-4	sll0585	GO:0015405	<1e-4	slr2125	GO:0044464	<1e-4	sll0760	GO:0005975	0.0418
slr1619	GO:0065007	<1e-4	slr7102	GO:0009165	<1e-4	slr0106	GO:0008171	<1e-4	sll1068	GO:0005575	<1e-4	slr1429	GO:0005975	0.0418
sll0400	GO:0045184	<1e-4	sll0497	GO:0009889	<1e-4	sll1400	GO:0043167	<1e-4	ssr3570	GO:0044422	<1e-4	sll1884	GO:0005975	0.0418
slr1546	GO:0055114	<1e-4	slr1143	GO:0065007	<1e-4	sll1061	GO:0022838	<1e-4	ssl3829	GO:0044444	<1e-4	ssl0312	GO:0005975	0.0418
sll1162	GO:0043648	<1e-4	sll5032	GO:0051186	<1e-4	slr0108	GO:0022857	<1e-4	sll1696	GO:0044422	<1e-4	sll8032	GO:0005975	0.0418
sll7033	GO:0009126	<1e-4	slr1576	GO:0006811	<1e-4	sll0909	GO:0022891	<1e-4	sll0939	GO:0044444	<1e-4	slr1187	GO:0005975	0.0418
slr1107	GO:0006091	<1e-4	sll0981	GO:0044282	<1e-4	sll0047	GO:0042623	<1e-4	slr1977	GO:0044422	<1e-4	sll1414	GO:0005975	0.0418
slr0168	GO:0019362	<1e-4	sll0743	GO:0048522	<1e-4	slr1188	GO:0016817	<1e-4	slr0810	GO:0044446	<1e-4	slr0065	GO:0005975	0.0418
ssr5011	GO:0051347	<1e-4	slr6065	GO:0043648	<1e-4	sll0216	GO:0042623	<1e-4	sll0371	GO:0043226	<1e-4	sll1921	GO:0005975	0.0418
ssr1558	GO:0008150	<1e-4	sll1036	GO:0009124	<1e-4	sll7031	GO:0022803	<1e-4	ssr7072	GO:0044444	<1e-4	slr2105	GO:0005975	0.0418
slr1150	GO:0060255	<1e-4	sll0822	GO:0048878	<1e-4	ssr0102	GO:0022891	<1e-4	slr0241	GO:0044424	<1e-4	sll5047	GO:0005975	0.0418
sll0931	GO:0052188	<1e-4	slr7025	GO:0071554	<1e-4	sll1411	GO:0016741	<1e-4	ssl0353	GO:0044446	<1e-4	sll1123	GO:0005975	0.0418
ssr1391	GO:0009165	<1e-4	slr1958	GO:0044255	<1e-4	slr5024	GO:0015293	<1e-4	slr0848	GO:0005575	<1e-4	slr5119	GO:0005975	0.0418
slr0709	GO:0048518	<1e-4	slr2118	GO:0044271	<1e-4	ssl3382	GO:0022832	<1e-4	slr0980	GO:0005575	<1e-4	sll1509	GO:0005975	0.0418
slr1396	GO:0051716	<1e-4	ssl1690	GO:0042180	<1e-4	sll0444	GO:0008171	<1e-4	ssl1263	GO:0044424	<1e-4	sll1109	GO:0005975	0.0418
slr1306	GO:0051188	<1e-4	ssr2554	GO:0046128	<1e-4	slr8022	GO:0022832	<1e-4	sgl0002	GO:0005575	<1e-4	slr0592	GO:0005975	0.0418
sll1761	GO:0050801	<1e-4	slr0179	GO:0051818	<1e-4	slr1659	GO:0022891	<1e-4	slr1398	GO:0044464	<1e-4	sll0786	GO:0005975	0.0418
slr7059	GO:0050789	<1e-4	slr2111	GO:0046483	<1e-4	sll1151	GO:0060089	<1e-4	sll0732	GO:0044444	<1e-4	slr0655	GO:0005975	0.0418
slr1495	GO:0009117	<1e-4	ssr2333	GO:0043436	<1e-4	ssl0294	GO:0016818	<1e-4	slr0453	GO:0044464	<1e-4	slr5023	GO:0005975	0.0418
slr0605	GO:0051716	<1e-4	ssl2733	GO:0044281	<1e-4	sll0361	GO:0022892	<1e-4	slr0082	GO:0044464	<1e-4	slr1186	GO:0005975	0.0418
slr0590	GO:0071841	<1e-4	slr1827	GO:0019222	<1e-4	slr1658	GO:0016818	<1e-4	sll8012	GO:0043226	<1e-4	slr0196	GO:0005975	0.0418
slr0960	GO:0051818	<1e-4	slr0845	GO:0031323	<1e-4	sll0843	GO:0015399	<1e-4	slr1957	GO:0044422	<1e-4	slr2101	GO:0005975	0.0418
slr5119	GO:0055082	<1e-4	slr0060	GO:0009892	<1e-4	sll1002	GO:0016746	<1e-4	sll0325	GO:0044446	<1e-4	slr0059	GO:0005975	0.0418
sll0102	GO:0072528	<1e-4	ssr7084	GO:0009889	<1e-4	slr0404	GO:0022891	<1e-4	slr0105	GO:0044425	<1e-4	sll5026	GO:0005975	0.0418
ssr0536	GO:0019362	<1e-4	sll0413	GO:0016052	<1e-4	sll1785	GO:0043167	<1e-4	slr0299	GO:0005575	<1e-4	sll0218	GO:0005975	0.0418
slr0356	GO:0009987	<1e-4	slr1240	GO:0080090	<1e-4	sll7066	GO:0016462	<1e-4	ssr1499	GO:0005575	<1e-4	sll0751	GO:0005975	0.0418
ssr1499	GO:0009142	<1e-4	sll0181	GO:0045184	<1e-4	ssr8047	GO:0015405	<1e-4	slr0890	GO:0044424	<1e-4	slr0168	GO:0005975	0.0418
slr0989	GO:0051234	<1e-4	slr1923	GO:0009308	<1e-4	sll0225	GO:0016741	<1e-4	sll0756	GO:0044424	<1e-4	ssr0102	GO:0005975	0.0418

slr1103	GO:0043549	<1e-4	slr0975	GO:0042180	<1e-4	sll1477	GO:0042623	<1e-4	slr1920	GO:0043226	<1e-4	sll8033	GO:0005975	0.0418
slr1668	GO:0001932	<1e-4	slr0341	GO:0060255	<1e-4	sll0265	GO:0016741	<1e-4	sll1749	GO:0044464	<1e-4	slr0058	GO:0005975	0.0418
slr0455	GO:0044283	<1e-4	slr1790	GO:0009165	<1e-4	slr1918	GO:0015075	<1e-4	slr0337	GO:0043226	<1e-4	slr0552	GO:0005975	0.0418
slr6022	GO:0006576	<1e-4	ssr1768	GO:0009987	<1e-4	slr2111	GO:0022857	<1e-4	slr0848	GO:0044424	<1e-4	slr1462	GO:0005975	0.0418
slr1406	GO:0044260	<1e-4	slr0569	GO:0009057	<1e-4	sll0785	GO:0022832	<1e-4	slr1266	GO:0044464	<1e-4	slr6047	GO:0005975	0.0418
sll0444	GO:0044275	<1e-4	ssr7017	GO:0006793	<1e-4	sll0508	GO:0022838	<1e-4	sll1447	GO:0005575	<1e-4	sll0994	GO:0005975	0.0418
sll1130	GO:0043648	<1e-4	slr6066	GO:0009991	<1e-4	sll0298	GO:0022836	<1e-4	sll1062	GO:0044424	<1e-4	slr6015	GO:0005975	0.0418
slr0921	GO:0009150	<1e-4	sll5130	GO:0051234	<1e-4	sll0335	GO:0022891	<1e-4	sll0175	GO:0005575	<1e-4	ssl1464	GO:0005975	0.0418
sll0565	GO:0006766	<1e-4	sll1752	GO:0051179	<1e-4	slr1442	GO:0015075	<1e-4	slr0148	GO:0005575	<1e-4	slr1907	GO:0005975	0.0418
sll0350	GO:0051179	<1e-4	slr0082	GO:0009262	<1e-4	ssl0788	GO:0022803	<1e-4	ssr0102	GO:0044464	<1e-4	slr6090	GO:0005975	0.0418
slr1704	GO:0071554	<1e-4	sll0225	GO:0009987	<1e-4	slr2144	GO:0015077	<1e-4	sll0572	GO:0044464	<1e-4	sll0266	GO:0005975	0.0418
ssr2848	GO:0033013	<1e-4	sll0266	GO:0043412	<1e-4	slr0863	GO:0016817	<1e-4	slr1600	GO:0043226	<1e-4	slr1068	GO:0005975	0.0418
sll5097	GO:0043170	<1e-4	slr0594	GO:0019219	<1e-4	sll7063	GO:0003674	<1e-4	sll0096	GO:0044446	<1e-4	sll0008	GO:0005975	0.0418
slr1816	GO:0050794	<1e-4	sll1304	GO:0009987	<1e-4	sll0265	GO:0022838	<1e-4	slr1611	GO:0005575	<1e-4	slr6039	GO:0005975	0.0418
ssr2781	GO:0009144	<1e-4	slr0317	GO:0006631	<1e-4	sll0743	GO:0022892	<1e-4	sll1192	GO:0005575	<1e-4	slr6063	GO:0005975	0.0418
slr1391	GO:0055086	<1e-4	ssl6061	GO:0048522	<1e-4	sll0156	GO:0016462	<1e-4	slr6104	GO:0043226	<1e-4	slr1677	GO:0005975	0.0418
slr1920	GO:0072522	<1e-4	slr0294	GO:0048523	<1e-4	sll0446	GO:0022803	<1e-4	sll1380	GO:0044464	<1e-4	slr1814	GO:0005975	0.0418
slr1926	GO:0006082	<1e-4	slr1811	GO:0018130	<1e-4	slr2049	GO:0015267	<1e-4	slr0654	GO:0043226	<1e-4	slr1195	GO:0005975	0.0418
slr1266	GO:0008610	<1e-4	ssl5031	GO:0006793	<1e-4	slr0810	GO:0015267	<1e-4	ssr6083	GO:0005575	<1e-4	slr0195	GO:0005975	0.0418
sll0181	GO:0044255	<1e-4	sll0641	GO:0055082	<1e-4	ssl0738	GO:0015293	<1e-4	slr1342	GO:0044446	<1e-4	slr1273	GO:0005975	0.0418
ssr6078	GO:0048522	<1e-4	slr5016	GO:0050801	<1e-4	slr1032	GO:0016741	<1e-4	slr6094	GO:0044444	<1e-4	slr1095	GO:0005975	0.0418
sll0756	GO:0009144	<1e-4	sll0310	GO:0009144	<1e-4	ssr3129	GO:0016817	<1e-4	ssl1792	GO:0043226	<1e-4	slr1397	GO:0005975	0.0418
sll0939	GO:0051234	<1e-4	slr0769	GO:0006082	<1e-4	ssr3129	GO:0022890	<1e-4	ssl7039	GO:0044422	<1e-4	slr1276	GO:0005975	0.0418
slr6045	GO:0009057	<1e-4	sll1040	GO:0052188	<1e-4	ssr2317	GO:0015293	<1e-4	slr1886	GO:0044464	<1e-4	ssr0332	GO:0005975	0.0418
sll5028	GO:0044283	<1e-4	sll0085	GO:0051188	<1e-4	slr2119	GO:0015405	<1e-4	sll0609	GO:0044444	<1e-4	slr1484	GO:0005975	0.0418
ssl5129	GO:0055114	<1e-4	ssr6024	GO:0044260	<1e-4	sll0198	GO:0015291	<1e-4	sll1926	GO:0043226	<1e-4	sll1092	GO:0005975	0.0418
sll0445	GO:0044282	<1e-4	slr1647	GO:0034654	<1e-4	slr1644	GO:0022838	<1e-4	slr5016	GO:0044444	<1e-4	slr6065	GO:0005975	0.0418
sll1399	GO:0008610	<1e-4	sll0735	GO:0010468	<1e-4	slr1275	GO:0015399	<1e-4	slr1505	GO:0044422	<1e-4	ssr5120	GO:0005975	0.0418
sll7055	GO:0090304	<1e-4	sll7028	GO:0072521	<1e-4	ssr6003	GO:0016746	<1e-4	sll1307	GO:0044446	<1e-4	slr2032	GO:0005975	0.0418
sll1942	GO:0009889	<1e-4	ssl5015	GO:0071554	<1e-4	ssl1552	GO:0016818	<1e-4	slr0723	GO:0044424	<1e-4	ssl3379	GO:0005975	0.0418
sll8002	GO:0009259	<1e-4	sll7089	GO:0044275	<1e-4	sll1542	GO:0022892	<1e-4	ssl3291	GO:0044444	<1e-4	sll1764	GO:0005975	0.0418
sll0760	GO:0006811	<1e-4	slr0650	GO:0034654	<1e-4	slr0642	GO:0022838	<1e-4	slr7037	GO:0044464	<1e-4	slr0751	GO:0005975	0.0418
sll0985	GO:0019752	<1e-4	slr1918	GO:0019752	<1e-4	sll0183	GO:0015405	<1e-4	sll1135	GO:0044422	<1e-4	ssr3409	GO:0005975	0.0418
sll1505	GO:0048518	<1e-4	slr1601	GO:0019751	<1e-4	sll1853	GO:0043167	<1e-4	slr1288	GO:0044444	<1e-4	slr1957	GO:0005975	0.0418
sll0382	GO:0043412	<1e-4	slr1070	GO:0009132	<1e-4	ssr3122	GO:0022890	<1e-4	slr1880	GO:0043226	<1e-4	ssr3189	GO:0005975	0.0418
sll1359	GO:0051716	<1e-4	sll1658	GO:0006066	<1e-4	sll0793	GO:0022890	<1e-4	slr0852	GO:0044425	<1e-4	slr0145	GO:0005975	0.0418
slr0398	GO:0006753	<1e-4	sll0980	GO:0005996	<1e-4	sll2015	GO:0043167	<1e-4	slr1114	GO:0044424	<1e-4	sll0811	GO:0005975	0.0418
slr0516	GO:0031640	<1e-4	slr1658	GO:0044271	<1e-4	ssl2471	GO:0060089	<1e-4	slr1773	GO:0044464	<1e-4	ssl5007	GO:0005975	0.0418
sll0996	GO:0048518	<1e-4	slr7096	GO:0051347	<1e-4	sll1464	GO:0015267	<1e-4	slr1073	GO:0044446	<1e-4	slr0870	GO:0005975	0.0418
slr1062	GO:0048522	<1e-4	slr2005	GO:0018130	<1e-4	sll1902	GO:0022890	<1e-4	ssl5027	GO:0005575	<1e-4	slr1270	GO:0005975	0.0418
sll1509	GO:0019752	<1e-4	sll1239	GO:0052111	<1e-4	slr0606	GO:0022838	<1e-4	slr1959	GO:0005575	<1e-4	sll0481	GO:0005975	0.0418
slr0207	GO:0009263	<1e-4	slr1863	GO:0019752	<1e-4	slr1572	GO:0015291	<1e-4	slr0145	GO:0044446	<1e-4	ssl1520	GO:0005975	0.0418
slr0149	GO:0044271	<1e-4	sll1995	GO:0008610	<1e-4	slr0813	GO:0042623	<1e-4	sll1825	GO:0044464	<1e-4	slr0959	GO:0005975	0.0418
slr6038	GO:0051818	<1e-4	slr2027	GO:0019752	<1e-4	ssl1520	GO:0015075	<1e-4	slr5017	GO:0044424	<1e-4	sll0846	GO:0005975	0.0418
sll1250	GO:0051716	<1e-4	ssr5121	GO:0044281	<1e-4	ssl7045	GO:0016817	<1e-4	slr0503	GO:0005575	<1e-4	slr1600	GO:0005975	0.0418
sll0068	GO:0044271	<1e-4	sll0413	GO:0048519	<1e-4	ssr3532	GO:0022803	<1e-4	slr1484	GO:0044424	<1e-4	slr1464	GO:0005975	0.0418
ssl5027	GO:0071841	<1e-4	sll0518	GO:0072528	<1e-4	sll0981	GO:0022836	<1e-4	sll0751	GO:0044444	<1e-4	slr1923	GO:0005975	0.0418
slr1535	GO:0006720	<1e-4	sll0283	GO:0009260	<1e-4	sll1119	GO:0015405	<1e-4	sll1178	GO:0005575	<1e-4	sll0301	GO:0005515	0.0414
slr1990	GO:0043170	<1e-4	slr5116	GO:0005996	<1e-4	ssl1263	GO:0022891	<1e-4	sll1401	GO:0044424	<1e-4	slr1505	GO:0005515	0.0414
slr2048	GO:0006733	<1e-4	sll7087	GO:0006733	<1e-4	sll2015	GO:0022857	<1e-4	sll0310	GO:0044422	<1e-4	sll0864	GO:0005515	0.0414
slr0651	GO:0019751	<1e-4	ssl2420	GO:0055082	<1e-4	slr0144	GO:0015399	<1e-4	sll1021	GO:0044446	<1e-4	sll0274	GO:0005515	0.0414
sll0888	GO:0019752	<1e-4	sll0888	GO:0044271	<1e-4	sll0735	GO:0005342	<1e-4	sll0180	GO:0005575	<1e-4	ssr1375	GO:0005515	0.0414
sll1751	GO:0071841	<1e-4	sll0930	GO:0008150	<1e-4	sll0553	GO:0022836	<1e-4	sll1714	GO:0005575	<1e-4	slr0937	GO:0005515	0.0414
sgl0002	GO:0042455	<1e-4	slr0270	GO:0050801	<1e-4	slr0510	GO:0015405	<1e-4	sll1769	GO:0044425	<1e-4	sll1942	GO:0005515	0.0414

slr0863	GO:0006163	<1e-4	slr1315	GO:0043170	<1e-4	sll1863	GO:0022890	<1e-4	slr0355	GO:0044422	<1e-4	sll1186	GO:0005515	0.0414
slr6028	GO:0044283	<1e-4	slr1384	GO:0009893	<1e-4	sll0237	GO:0022838	<1e-4	ssl2138	GO:0044446	<1e-4	slr1417	GO:0005515	0.0414
slr1168	GO:0072522	<1e-4	sll0783	GO:0044260	<1e-4	slr6039	GO:0022836	<1e-4	ssr5074	GO:0044444	<1e-4	sll1219	GO:0005515	0.0414
sll1123	GO:0051716	<1e-4	slr1780	GO:0042180	<1e-4	sll0007	GO:0022803	<1e-4	sll0493	GO:0005575	<1e-4	slr1866	GO:0005515	0.0414
sll5089	GO:0019222	<1e-4	sll0157	GO:0006066	<1e-4	slr0318	GO:0016817	<1e-4	slr1068	GO:0005575	<1e-4	ssr2843	GO:0004871	0.0414
slr1699	GO:0009260	<1e-4	slr1533	GO:0072524	<1e-4	ssr3571	GO:0015077	<1e-4	slr1964	GO:0044424	<1e-4	sll1737	GO:0004871	0.0414
slr1419	GO:0051716	<1e-4	ssr1473	GO:0006811	<1e-4	slr1768	GO:0016746	<1e-4	slr1258	GO:0005575	<1e-4	sll0615	GO:0005515	0.0414
slr1186	GO:0044255	<1e-4	slr0374	GO:0019219	<1e-4	sll1142	GO:0022892	<1e-4	slr1957	GO:0005575	<1e-4	sll0996	GO:0005515	0.0414
slr1240	GO:0052188	<1e-4	ssr1425	GO:0046164	<1e-4	ssr7079	GO:0003674	<1e-4	slr6094	GO:0043226	<1e-4	ssl3692	GO:0005515	0.0414
sll1658	GO:0043549	<1e-4	sll5047	GO:0034641	<1e-4	slr1128	GO:0022838	<1e-4	slr0453	GO:0044446	<1e-4	sll0447	GO:0005515	0.0414
sll1832	GO:0060255	<1e-4	sll1912	GO:0006766	<1e-4	sll0359	GO:0042623	<1e-4	slr1045	GO:0005575	<1e-4	slr0209	GO:0005515	0.0414
sll0242	GO:0006163	<1e-4	slr6067	GO:0051347	<1e-4	sll5047	GO:0016746	<1e-4	sll0410	GO:0044422	<1e-4	slr1441	GO:0005515	0.0414
sll0623	GO:0042180	<1e-4	slr0770	GO:0009057	<1e-4	sll1131	GO:0015405	<1e-4	slr0059	GO:0044446	<1e-4	sll0547	GO:0005515	0.0414
slr1081	GO:0006811	<1e-4	ssr2439	GO:0019222	<1e-4	ssr2615	GO:0042623	<1e-4	slr0287	GO:0044444	<1e-4	sll1166	GO:0005515	0.0414
sll1160	GO:0048523	<1e-4	sll1527	GO:0044281	<1e-4	slr0545	GO:0043167	<1e-4	ssr3402	GO:0043226	<1e-4	ssr1698	GO:0005515	0.0414
slr5073	GO:0042451	<1e-4	slr1306	GO:0006811	<1e-4	sll1939	GO:0022857	<1e-4	sll1563	GO:0043226	<1e-4	slr1045	GO:0005515	0.0414
sll1938	GO:0006091	<1e-4	ssl7046	GO:0051716	<1e-4	sll1583	GO:0022836	<1e-4	sll0638	GO:0044444	<1e-4	slr2092	GO:0004871	0.0414
sll1060	GO:0006766	<1e-4	slr1612	GO:0044283	<1e-4	slr7057	GO:0015267	<1e-4	sll0552	GO:0043226	<1e-4	sll0066	GO:0008168	0.0398
slr0023	GO:0009262	<1e-4	slr1073	GO:0016054	<1e-4	slr2011	GO:0015075	<1e-4	slr0658	GO:0043226	<1e-4	sll0756	GO:0008168	0.0398
slr1918	GO:0048518	<1e-4	ssr3402	GO:0071496	<1e-4	slr0211	GO:0003674	<1e-4	sll0827	GO:0044446	<1e-4	sll0676	GO:0008168	0.0398
slr0516	GO:0006732	<1e-4	sll8033	GO:0009123	<1e-4	sll0394	GO:0022803	<1e-4	sll1940	GO:0043226	<1e-4	sll1086	GO:0008168	0.0398
sll5069	GO:0009144	<1e-4	slr1568	GO:0051716	<1e-4	slr7097	GO:0015293	<1e-4	slr5118	GO:0044444	<1e-4	slr1084	GO:0008168	0.0398
sll1355	GO:0009123	<1e-4	slr5126	GO:0071842	<1e-4	slr1472	GO:0022832	<1e-4	ssr2998	GO:0044425	<1e-4	slr1999	GO:0008168	0.0398
slr1911	GO:0009394	<1e-4	sll0735	GO:0006163	<1e-4	slr0516	GO:0016741	<1e-4	sll7043	GO:0044425	<1e-4	ssr1765	GO:0008168	0.0398
slr1613	GO:0033013	<1e-4	slr0360	GO:0051179	<1e-4	sll0552	GO:0022857	<1e-4	slr1116	GO:0044425	<1e-4	sll0085	GO:0008168	0.0398
slr0723	GO:0044260	<1e-4	sll1884	GO:0034660	<1e-4	slr1468	GO:0015075	<1e-4	ssr7036	GO:0044425	<1e-4	slr1530	GO:0008168	0.0398
sll1166	GO:0006766	<1e-4	slr1429	GO:0048522	<1e-4	sll1315	GO:0022891	<1e-4	slr1647	GO:0043226	<1e-4	slr0192	GO:0008168	0.0398
slr1534	GO:0044283	<1e-4	sll5047	GO:0044271	<1e-4	sll1606	GO:0015077	<1e-4	ssr3410	GO:0044424	<1e-4	sll1442	GO:0008168	0.0398
sll1726	GO:0009117	<1e-4	slr0755	GO:0009161	<1e-4	sll1528	GO:0042623	<1e-4	sll0172	GO:0044422	<1e-4	slr1073	GO:0008168	0.0398
slr0637	GO:0044271	<1e-4	slr2011	GO:0009308	<1e-4	slr1789	GO:0016817	<1e-4	sll0283	GO:0043226	<1e-4	ssl0352	GO:0008168	0.0398
slr0667	GO:0019362	<1e-4	slr0579	GO:0055082	<1e-4	sll1273	GO:0022891	<1e-4	slr1619	GO:0005575	<1e-4	slr1618	GO:0008168	0.0398
slr7080	GO:0042451	<1e-4	slr1638	GO:0022607	<1e-4	sll1142	GO:0022803	<1e-4	slr0184	GO:0005575	<1e-4	slr1788	GO:0008168	0.0398
sll1052	GO:0072524	<1e-4	slr0930	GO:0005996	<1e-4	ssl5113	GO:0022892	<1e-4	slr5118	GO:0044424	<1e-4	slr1885	GO:0008168	0.0398
slr0341	GO:0051234	<1e-4	sll1424	GO:0043648	<1e-4	sll1006	GO:0016741	<1e-4	slr6080	GO:0044464	<1e-4	slr1676	GO:0008168	0.0398
sll1348	GO:0009199	<1e-4	slr0596	GO:0071840	<1e-4	slr1970	GO:0015291	<1e-4	sll1218	GO:0005575	<1e-4	slr1573	GO:0008168	0.0398
sll0381	GO:0051716	<1e-4	sll0488	GO:0050794	<1e-4	slr2110	GO:0022836	<1e-4	slr0651	GO:0044444	<1e-4	sll1632	GO:0008168	0.0398
sll7031	GO:0006091	<1e-4	sll5030	GO:0051347	<1e-4	sll1352	GO:0015405	<1e-4	ssr2060	GO:0044424	<1e-4	sll0010	GO:0008168	0.0398
slr1383	GO:0044282	<1e-4	sll1233	GO:0072521	<1e-4	sll0335	GO:0003674	<1e-4	slr1150	GO:0044446	<1e-4	slr0294	GO:0008168	0.0398
slr0294	GO:0034654	<1e-4	slr1566	GO:0009150	<1e-4	ssr1951	GO:0015291	<1e-4	slr0299	GO:0044422	<1e-4	sll0381	GO:0008168	0.0398
slr7025	GO:0033013	<1e-4	slr1074	GO:0051347	<1e-4	slr0552	GO:0022838	<1e-4	sll0606	GO:0044424	<1e-4	ssl2807	GO:0008168	0.0398
ssr1698	GO:0051716	<1e-4	sll0752	GO:0048519	<1e-4	sll0424	GO:0022838	<1e-4	sll0444	GO:0044425	<1e-4	slr1203	GO:0008168	0.0398
sll1321	GO:0009987	<1e-4	sll0752	GO:0050896	<1e-4	sll5067	GO:0016746	<1e-4	sll5044	GO:0005575	<1e-4	slr0941	GO:0008168	0.0398
ssr2962	GO:0072524	<1e-4	sll1247	GO:0071841	<1e-4	slr1557	GO:0016817	<1e-4	slr1068	GO:0044444	<1e-4	ssr2962	GO:0008168	0.0398
sll0414	GO:0051347	<1e-4	sll1040	GO:0051171	<1e-4	ssr2047	GO:0022892	<1e-4	slr1612	GO:0044424	<1e-4	slr0386	GO:0008168	0.0398
sll0609	GO:0050896	<1e-4	ssl3573	GO:0006066	<1e-4	sll0732	GO:0015293	<1e-4	sll1651	GO:0044425	<1e-4	sll1835	GO:0008168	0.0398
sll8032	GO:0051716	<1e-4	slr6064	GO:0055082	<1e-4	slr6029	GO:0008171	<1e-4	slr1278	GO:0044425	<1e-4	sll1630	GO:0008168	0.0398
slr1815	GO:0046483	<1e-4	slr1194	GO:0006732	<1e-4	slr0948	GO:0016741	<1e-4	slr1970	GO:0044424	<1e-4	slr0921	GO:0008168	0.0398
sll0596	GO:0043549	<1e-4	slr5126	GO:0044255	<1e-4	slr0318	GO:0022892	<1e-4	slr1303	GO:0044464	<1e-4	ssl2065	GO:0008168	0.0398
slr0586	GO:0019752	<1e-4	sll1880	GO:0006721	<1e-4	slr6022	GO:0008171	<1e-4	sll0010	GO:0005575	<1e-4	sll1608	GO:0008168	0.0398
slr1177	GO:0006721	<1e-4	ssr5011	GO:0043170	<1e-4	slr0594	GO:0015291	<1e-4	sll0846	GO:0005575	<1e-4	sll0497	GO:0008168	0.0398
slr1636	GO:0072528	<1e-4	slr1563	GO:0009123	<1e-4	ssr0536	GO:0003674	<1e-4	sll1835	GO:0044446	<1e-4	slr1519	GO:0016740	0.0375
slr1383	GO:0044271	<1e-4	slr5021	GO:0019752	<1e-4	slr1273	GO:0022803	<1e-4	sll0282	GO:0043226	<1e-4	slr0876	GO:0016740	0.0375
ssl1046	GO:0071842	<1e-4	sll1830	GO:0052111	<1e-4	slr1186	GO:0015293	<1e-4	slr0151	GO:0044422	<1e-4	sll1586	GO:0016740	0.0375
slr1819	GO:0008150	<1e-4	sll1611	GO:0048519	<1e-4	slr0581	GO:0015405	<1e-4	sll0185	GO:0044422	<1e-4	slr0287	GO:0016740	0.0375

sll1373	GO:0009199	<1e-4	sll0735	GO:0019637	<1e-4	ssl7042	GO:0022838	<1e-4	sll0611	GO:0044446	<1e-4	sll1222	GO:0003723	0.0375
ssl0461	GO:0009123	<1e-4	slr0238	GO:0051179	<1e-4	slr2103	GO:0060089	<1e-4	slr0730	GO:0005575	<1e-4	ssr1698	GO:0016740	0.0375
sll0857	GO:0044106	<1e-4	slr0930	GO:0046128	<1e-4	ssl1377	GO:0022803	<1e-4	slr2105	GO:0044422	<1e-4	slr0818	GO:0003723	0.0375
slr0416	GO:0048522	<1e-4	sll0218	GO:0009991	<1e-4	sll0623	GO:0016817	<1e-4	slr2144	GO:0005575	<1e-4	slr1900	GO:0016740	0.0375
slr1363	GO:0046164	<1e-4	sll0827	GO:0052111	<1e-4	slr0887	GO:0022838	<1e-4	slr1287	GO:0044444	<1e-4	sll1477	GO:0003723	0.0375
sll0854	GO:0046483	<1e-4	sll1464	GO:0048878	<1e-4	sll0268	GO:0015267	<1e-4	sll1009	GO:0005575	<1e-4	slr1918	GO:0016740	0.0375
sll1835	GO:0042180	<1e-4	ssl2971	GO:0016054	<1e-4	ssl2148	GO:0016462	<1e-4	slr6006	GO:0005575	<1e-4	slr0243	GO:0016740	0.0375
ssl2807	GO:0009144	<1e-4	sll1203	GO:0006811	<1e-4	sll1162	GO:0060089	<1e-4	sll0802	GO:0043226	<1e-4	slr0211	GO:0016740	0.0375
sll0590	GO:0006733	<1e-4	slr0416	GO:0006220	<1e-4	sll1250	GO:0005342	<1e-4	sll0513	GO:0043226	<1e-4	slr1391	GO:0016740	0.0375
slr1495	GO:0009123	<1e-4	sll1715	GO:0006220	<1e-4	slr2103	GO:0016817	<1e-4	sll0148	GO:0005575	<1e-4	slr0157	GO:0016740	0.0375
slr0184	GO:0009123	<1e-4	slr8044	GO:0006082	<1e-4	slr7094	GO:0015399	<1e-4	slr1101	GO:0044446	<1e-4	ssl0787	GO:0016740	0.0375
ssl0738	GO:0016054	<1e-4	slr6064	GO:0044003	<1e-4	slr1101	GO:0042623	<1e-4	sll1109	GO:0044422	<1e-4	slr0108	GO:0003723	0.0375
sll1319	GO:0046164	<1e-4	slr1590	GO:0009394	<1e-4	sll5090	GO:0043167	<1e-4	slr1307	GO:0044424	<1e-4	sll1166	GO:0016740	0.0375
sll1166	GO:0044281	<1e-4	sll1086	GO:0055086	<1e-4	slr5077	GO:0060089	<1e-4	sll1304	GO:0005575	<1e-4	sll1247	GO:0016740	0.0375
ssl6035	GO:0034641	<1e-4	sll0294	GO:0046483	<1e-4	sll0647	GO:0022857	<1e-4	slr1195	GO:0005575	<1e-4	sll0735	GO:0003723	0.0375
sll1378	GO:0044106	<1e-4	sll0886	GO:0009263	<1e-4	sll0543	GO:0016818	<1e-4	sll0066	GO:0043226	<1e-4	slr1441	GO:0016740	0.0375
sll1921	GO:0051179	<1e-4	sll1095	GO:0050794	<1e-4	sll0448	GO:0016462	<1e-4	ssr1528	GO:0044424	<1e-4	sll0148	GO:0016740	0.0375
ssl5095	GO:0046164	<1e-4	slr2003	GO:0043436	<1e-4	sll1053	GO:0043492	<1e-4	slr0656	GO:0005575	<1e-4	slr0147	GO:0003723	0.0375
sll0787	GO:0006066	<1e-4	slr1023	GO:0044106	<1e-4	slr1999	GO:0015291	<1e-4	ssl0410	GO:0044446	<1e-4	slr0937	GO:0016740	0.0375
ssl0467	GO:0006576	<1e-4	ssl5113	GO:0046128	<1e-4	sll0763	GO:0015293	<1e-4	slr5102	GO:0044422	<1e-4	slr1917	GO:0016740	0.0375
slr0813	GO:0090304	<1e-4	sll0525	GO:0051347	<1e-4	ssr1256	GO:0022890	<1e-4	sll0147	GO:0005575	<1e-4	slr1866	GO:0016740	0.0375
ssr1256	GO:0018130	<1e-4	slr0692	GO:0048878	<1e-4	sll0586	GO:0016817	<1e-4	sll0779	GO:0005575	<1e-4	slr0376	GO:0016740	0.0375
slr6068	GO:0045184	<1e-4	slr0345	GO:0009057	<1e-4	slr1407	GO:0015267	<1e-4	ssr1155	GO:0043226	<1e-4	slr0304	GO:0016740	0.0375
sll1414	GO:0005996	<1e-4	slr1927	GO:0034654	<1e-4	sll7089	GO:0022892	<1e-4	sll7089	GO:0005575	<1e-4	sll1472	GO:0016740	0.0375
slr0392	GO:0022607	<1e-4	sll1797	GO:0051171	<1e-4	slr0269	GO:0022803	<1e-4	slr6045	GO:0005575	<1e-4	ssl0109	GO:0016740	0.0375
ssl5129	GO:0009889	<1e-4	sll1510	GO:0009117	<1e-4	sll7078	GO:0016746	<1e-4	sll1722	GO:0044446	<1e-4	slr1083	GO:0016740	0.0375
slr0607	GO:0009117	<1e-4	slr1095	GO:0044003	<1e-4	sll1751	GO:0016462	<1e-4	slr1472	GO:0044464	<1e-4	sll0424	GO:0016740	0.0375
slr0195	GO:0051818	<1e-4	slr0765	GO:0043648	<1e-4	slr6016	GO:0016746	<1e-4	slr0380	GO:0044422	<1e-4	slr0909	GO:0005515	0.0369
slr0521	GO:0009309	<1e-4	slr1862	GO:0009991	<1e-4	sll0172	GO:0022892	<1e-4	sll1063	GO:0044446	<1e-4	ssl0353	GO:0005515	0.0369
ssl2733	GO:0042455	<1e-4	ssl7007	GO:0019752	<1e-4	slr5077	GO:0015291	<1e-4	sll0360	GO:0044422	<1e-4	sll1562	GO:0005515	0.0369
slr2118	GO:0019362	<1e-4	sll0498	GO:0022411	<1e-4	slr1338	GO:0022892	<1e-4	sll1583	GO:0044422	<1e-4	sll0505	GO:0005515	0.0369
sll1834	GO:0009893	<1e-4	slr1577	GO:0051179	<1e-4	slr1534	GO:0015405	<1e-4	slr7012	GO:0043226	<1e-4	slr1767	GO:0005515	0.0369
sll1004	GO:0042455	<1e-4	sll1352	GO:0052188	<1e-4	sll1021	GO:0043167	<1e-4	sll1643	GO:0044422	<1e-4	slr0607	GO:0005515	0.0369
slr1866	GO:0009144	<1e-4	sll0428	GO:0006721	<1e-4	sll5063	GO:0022891	<1e-4	sll0735	GO:0044446	<1e-4	sll0788	GO:0005515	0.0369
sll0503	GO:0050789	<1e-4	ssl7022	GO:0009126	<1e-4	slr7092	GO:0015077	<1e-4	sll0296	GO:0044446	<1e-4	slr0514	GO:0005515	0.0369
slr6068	GO:0044283	<1e-4	sll0263	GO:0019751	<1e-4	sll1891	GO:0016817	<1e-4	slr5101	GO:0044422	<1e-4	slr0479	GO:0005515	0.0369
sll1072	GO:0010556	<1e-4	ssl0410	GO:0065007	<1e-4	sll1965	GO:0042623	<1e-4	sll5028	GO:0005575	<1e-4	ssl2733	GO:0005515	0.0369
slr1657	GO:0034641	<1e-4	sll1469	GO:0006720	<1e-4	ssr1258	GO:0043492	<1e-4	slr0476	GO:0044424	<1e-4	ssl0294	GO:0003723	0.0364
sll1389	GO:0009263	<1e-4	sll6054	GO:0044003	<1e-4	sll0280	GO:0016817	<1e-4	ssr1552	GO:0044444	<1e-4	sll1293	GO:0003723	0.0364
sll0060	GO:0018130	<1e-4	slr7057	GO:0009991	<1e-4	sll0839	GO:0015399	<1e-4	sll1262	GO:0044424	<1e-4	sll0350	GO:0003723	0.0364
sll0565	GO:0051716	<1e-4	slr1568	GO:0050896	<1e-4	ssl2807	GO:0015399	<1e-4	slr0397	GO:0044425	<1e-4	sll1763	GO:0003723	0.0364
sll1155	GO:0009263	<1e-4	sll0488	GO:0016053	<1e-4	sll0405	GO:0016741	<1e-4	slr1079	GO:0005575	<1e-4	slr0355	GO:0016772	0.0358
sll1132	GO:0031326	<1e-4	sll1773	GO:0009309	<1e-4	sll1832	GO:0022838	<1e-4	slr0519	GO:0044464	<1e-4	slr6101	GO:0016772	0.0358
slr1827	GO:0051234	<1e-4	sll1225	GO:0044248	<1e-4	sll1884	GO:0016746	<1e-4	slr1900	GO:0044464	<1e-4	slr0264	GO:0016772	0.0358
slr1170	GO:0048518	<1e-4	sll6053	GO:0072524	<1e-4	sll0319	GO:0015291	<1e-4	slr6087	GO:0044446	<1e-4	sll1485	GO:0016772	0.0358
ssl0739	GO:0048522	<1e-4	sll0442	GO:0009142	<1e-4	ssr6027	GO:0022857	<1e-4	sll1552	GO:0044446	<1e-4	slr1660	GO:0016772	0.0358
sll0068	GO:0034641	<1e-4	slr7083	GO:0072527	<1e-4	slr0980	GO:0022832	<1e-4	sll0786	GO:0044446	<1e-4	slr1104	GO:0016772	0.0358
sll1640	GO:0046128	<1e-4	slr1052	GO:0009260	<1e-4	sll0761	GO:0015405	<1e-4	ssl5031	GO:0044425	<1e-4	ssr2422	GO:0016772	0.0358
slr2071	GO:0071841	<1e-4	slr1034	GO:0006082	<1e-4	sll1254	GO:0022836	<1e-4	sll1630	GO:0044446	<1e-4	ssl3142	GO:0016772	0.0358
sll7078	GO:0016053	<1e-4	slr7011	GO:0019362	<1e-4	ssr2142	GO:0043167	<1e-4	slr0813	GO:0044446	<1e-4	slr2119	GO:0016772	0.0358
sll1477	GO:0043170	<1e-4	ssl3692	GO:0009056	<1e-4	sll5097	GO:0022890	<1e-4	ssl6035	GO:0044444	<1e-4	sll2013	GO:0016772	0.0358
slr0769	GO:0071496	<1e-4	sll5003	GO:0045184	<1e-4	slr0262	GO:0022836	<1e-4	sll1372	GO:0043226	<1e-4	slr1472	GO:0016772	0.0358
sll0691	GO:0006163	<1e-4	slr6007	GO:0044260	<1e-4	sll1053	GO:0016818	<1e-4	slr1186	GO:0044422	<1e-4	ssr2912	GO:0016772	0.0358
sll0048	GO:0006793	<1e-4	sll1654	GO:0006766	<1e-4	sll0410	GO:0005342	<1e-4	sll7064	GO:0044464	<1e-4	slr6013	GO:0016772	0.0358

slr0890	GO:0006811	<1e-4	ssl7042	GO:0046164	<1e-4	ssl3382	GO:0022890	<1e-4	slr6104	GO:0044424	<1e-4	ssl8003	GO:0016772	0.0358
ssl3573	GO:0019222	<1e-4	ssl0461	GO:0042451	<1e-4	slr5037	GO:0022857	<1e-4	slr0554	GO:0044422	<1e-4	slr0196	GO:0016772	0.0358
ssr2067	GO:0034641	<1e-4	ssr6020	GO:0050794	<1e-4	slr0195	GO:0008171	<1e-4	sll0867	GO:0005575	<1e-4	slr1306	GO:0016772	0.0358
slr2052	GO:0006811	<1e-4	sll0355	GO:0051818	<1e-4	ssr7084	GO:0022857	<1e-4	slr0912	GO:0005575	<1e-4	sll7047	GO:0016772	0.0358
slr1493	GO:0051234	<1e-4	sll1752	GO:0046164	<1e-4	slr1178	GO:0015077	<1e-4	slr0341	GO:0044464	<1e-4	sll0406	GO:0016772	0.0358
sll1469	GO:0031640	<1e-4	ssl1972	GO:0022607	<1e-4	sll1063	GO:0008171	<1e-4	sll1527	GO:0044424	<1e-4	sll0048	GO:0016772	0.0358
sll1359	GO:0048522	<1e-4	sll1757	GO:0009144	<1e-4	slr1365	GO:0015293	<1e-4	slr1819	GO:0044446	<1e-4	ssr0102	GO:0016772	0.0358
slr1178	GO:0009262	<1e-4	sll1542	GO:0045184	<1e-4	sll8004	GO:0005342	<1e-4	slr0650	GO:0044425	<1e-4	slr1721	GO:0016772	0.0358
slr0751	GO:0022411	<1e-4	slr1104	GO:0043933	<1e-4	sll0442	GO:0022890	<1e-4	slr7023	GO:0044464	<1e-4	slr1150	GO:0016772	0.0358
slr0941	GO:0009309	<1e-4	slr0605	GO:0016053	<1e-4	slr7092	GO:0016818	<1e-4	slr1168	GO:0005575	<1e-4	sll1696	GO:0016772	0.0358
slr5101	GO:0043170	<1e-4	ssr2711	GO:0009260	<1e-4	slr1363	GO:0022890	<1e-4	sll1219	GO:0005575	<1e-4	sll0444	GO:0016772	0.0358
slr1827	GO:0043933	<1e-4	sll0410	GO:0016054	<1e-4	sll0293	GO:0043167	<1e-4	sll0775	GO:0044464	<1e-4	slr6064	GO:0016772	0.0358
sll1562	GO:0009889	<1e-4	slr1052	GO:0051246	<1e-4	sll1586	GO:0022891	<1e-4	sll0005	GO:0044444	<1e-4	slr0440	GO:0016772	0.0358
sll0189	GO:0009057	<1e-4	slr0207	GO:0009165	<1e-4	sll0572	GO:0015293	<1e-4	sll1252	GO:0044422	<1e-4	ssr1407	GO:0016772	0.0358
sll5069	GO:0044275	<1e-4	slr0602	GO:0065007	<1e-4	sll1135	GO:0016746	<1e-4	slr0383	GO:0005575	<1e-4	slr1464	GO:0016772	0.0358
sll1002	GO:0022607	<1e-4	slr1218	GO:0060255	<1e-4	sll0473	GO:0015075	<1e-4	sll1142	GO:0005575	<1e-4	ssr6046	GO:0016772	0.0358
slr2105	GO:0009889	<1e-4	sll0149	GO:0009117	<1e-4	ssr1375	GO:0015291	<1e-4	sll0564	GO:0043226	<1e-4	sll2015	GO:0016772	0.0358
sll8040	GO:0009309	<1e-4	slr6044	GO:0006732	<1e-4	slr1918	GO:0015293	<1e-4	sll0400	GO:0044444	<1e-4	ssl2920	GO:0016772	0.0358
sll0191	GO:0009394	<1e-4	sll1764	GO:0019222	<1e-4	slr2103	GO:0022857	<1e-4	sll1119	GO:0005575	<1e-4	sll1766	GO:0016772	0.0358
slr0112	GO:0046394	<1e-4	slr1767	GO:0001932	<1e-4	slr1097	GO:0022891	<1e-4	sll1751	GO:0044444	<1e-4	ssl1923	GO:0016772	0.0358
slr1773	GO:0051347	<1e-4	sll0498	GO:0006720	<1e-4	slr1788	GO:0016818	<1e-4	sll1399	GO:0044424	<1e-4	slr2010	GO:0016772	0.0358
slr0360	GO:0009987	<1e-4	sll0283	GO:0055082	<1e-4	slr0076	GO:0015077	<1e-4	slr5126	GO:0005575	<1e-4	sll1063	GO:0016772	0.0358
sll1862	GO:0072522	<1e-4	slr1917	GO:0051188	<1e-4	slr0364	GO:0016817	<1e-4	sgl0002	GO:0043226	<1e-4	slr7059	GO:0016772	0.0358
sll0479	GO:0048519	<1e-4	slr0291	GO:0019752	<1e-4	slr0723	GO:0022803	<1e-4	slr1958	GO:0044424	<1e-4	slr1270	GO:0016772	0.0358
ssl1520	GO:0050896	<1e-4	ssl3829	GO:0005996	<1e-4	slr1752	GO:0005342	<1e-4	sll1882	GO:0044422	<1e-4	slr1484	GO:0016772	0.0358
slr1721	GO:0044275	<1e-4	sll0298	GO:0006811	<1e-4	ssl7042	GO:0003674	<1e-4	sll1225	GO:0044424	<1e-4	slr0148	GO:0016772	0.0358
slr0725	GO:0019751	<1e-4	slr1128	GO:0071842	<1e-4	slr1704	GO:0022803	<1e-4	sll1232	GO:0044464	<1e-4	slr1260	GO:0016772	0.0358
slr1068	GO:0009132	<1e-4	slr0479	GO:0042455	<1e-4	slr1315	GO:0043167	<1e-4	sll0280	GO:0044422	<1e-4	ssl5007	GO:0016772	0.0358
ssl1552	GO:0009260	<1e-4	slr0771	GO:0051246	<1e-4	slr1468	GO:0015399	<1e-4	slr6015	GO:0043226	<1e-4	sll0008	GO:0016772	0.0358
slr1998	GO:0009259	<1e-4	ssl2420	GO:0071840	<1e-4	sll1378	GO:0043167	<1e-4	ssr6079	GO:0043226	<1e-4	sll0397	GO:0016772	0.0358
slr2121	GO:0051347	<1e-4	slr1087	GO:0009056	<1e-4	slr8021	GO:0015399	<1e-4	slr1513	GO:0044444	<1e-4	slr6015	GO:0016772	0.0358
ssl5095	GO:0022607	<1e-4	sll1252	GO:0009259	<1e-4	sll0710	GO:0042623	<1e-4	slr0199	GO:0044424	<1e-4	sll0857	GO:0016772	0.0358
sll0886	GO:0072527	<1e-4	sll1873	GO:0009059	<1e-4	slr5119	GO:0016746	<1e-4	sll1773	GO:0044444	<1e-4	ssr1766	GO:0016772	0.0358
ssr2755	GO:0071496	<1e-4	sll0996	GO:0006066	<1e-4	ssl5068	GO:0015405	<1e-4	sll1306	GO:0005575	<1e-4	ssr6020	GO:0016772	0.0358
ssl5129	GO:0044255	<1e-4	slr1376	GO:0050794	<1e-4	sll1714	GO:0016817	<1e-4	slr1959	GO:0044446	<1e-4	sll5097	GO:0016772	0.0358
slr0770	GO:0043648	<1e-4	ssr2755	GO:0048878	<1e-4	slr0869	GO:0016741	<1e-4	sll1486	GO:0005575	<1e-4	slr1546	GO:0016772	0.0358
slr1162	GO:0043412	<1e-4	sll1949	GO:0034641	<1e-4	slr0651	GO:0008171	<1e-4	slr0810	GO:0043226	<1e-4	slr1178	GO:0016772	0.0358
slr0708	GO:0055086	<1e-4	sll2013	GO:0071841	<1e-4	slr1788	GO:0022838	<1e-4	slr1045	GO:0044444	<1e-4	sll1880	GO:0016772	0.0358
ssl7038	GO:0072528	<1e-4	slr0569	GO:0005996	<1e-4	ssl3829	GO:0022838	<1e-4	slr0092	GO:0044446	<1e-4	ssl0738	GO:0016772	0.0358
ssl0832	GO:0046483	<1e-4	slr6081	GO:0043648	<1e-4	ssl1918	GO:0022838	<1e-4	slr0708	GO:0044424	<1e-4	slr7101	GO:0016772	0.0358
sll1942	GO:0071554	<1e-4	sll7065	GO:0065007	<1e-4	sll1378	GO:0015399	<1e-4	sll0293	GO:0044425	<1e-4	slr6090	GO:0016772	0.0358
slr2060	GO:0006066	<1e-4	sll1858	GO:0051716	<1e-4	slr2101	GO:0016818	<1e-4	slr0801	GO:0044422	<1e-4	sll1447	GO:0016772	0.0358
slr1618	GO:0019637	<1e-4	slr6031	GO:0048518	<1e-4	slr5018	GO:0005342	<1e-4	sll0071	GO:0044424	<1e-4	sll0174	GO:0016772	0.0358
ssl0750	GO:0009262	<1e-4	sll1401	GO:0060255	<1e-4	slr0709	GO:0003674	<1e-4	sll0803	GO:0005575	<1e-4	sll0168	GO:0016772	0.0358
slr1087	GO:0044248	<1e-4	sll1563	GO:0034641	<1e-4	slr8021	GO:0008171	<1e-4	ssr0536	GO:0044464	<1e-4	slr0151	GO:0016772	0.0358
slr0299	GO:0044271	<1e-4	sll8012	GO:0072528	<1e-4	slr1417	GO:0016741	<1e-4	slr0658	GO:0044422	<1e-4	slr0356	GO:0016772	0.0358
ssr0102	GO:0071840	<1e-4	sll1765	GO:0006721	<1e-4	slr0109	GO:0043492	<1e-4	sll1254	GO:0044464	<1e-4	ssl1520	GO:0016772	0.0358
ssr7079	GO:0009262	<1e-4	sll1698	GO:0072524	<1e-4	slr0913	GO:0022832	<1e-4	slr5101	GO:0044464	<1e-4	sll1571	GO:0016772	0.0358
ssr3341	GO:0009142	<1e-4	slr0172	GO:0009892	<1e-4	slr0408	GO:0016817	<1e-4	sll0241	GO:0044446	<1e-4	sll5026	GO:0016772	0.0358
sll7050	GO:0051186	<1e-4	sll1273	GO:0009123	<1e-4	sll1400	GO:0016818	<1e-4	ssr7072	GO:0044446	<1e-4	slr1863	GO:0016772	0.0358
slr1917	GO:0055114	<1e-4	slr1618	GO:0048519	<1e-4	ssr0335	GO:0043492	<1e-4	slr1107	GO:0044446	<1e-4	slr1566	GO:0016772	0.0358
slr0888	GO:0006721	<1e-4	sll1832	GO:0052188	<1e-4	sll1528	GO:0022838	<1e-4	sll0563	GO:0005575	<1e-4	sll1769	GO:0016772	0.0358
sll0931	GO:0006720	<1e-4	sll1921	GO:0048878	<1e-4	sll0188	GO:0022803	<1e-4	slr0921	GO:0044422	<1e-4	sll1092	GO:0016772	0.0358
sll1764	GO:0009161	<1e-4	sll0310	GO:0009165	<1e-4	slr1417	GO:0015267	<1e-4	slr1944	GO:0044424	<1e-4	slr1534	GO:0016772	0.0358

slr1809	GO:0009260	<1e-4	slr7010	GO:0009394	<1e-4	sll5033	GO:0016818	<1e-4	sll1262	GO:0044425	<1e-4	sll0518	GO:0016772	0.0358
slr1753	GO:0034641	<1e-4	slr0356	GO:0044283	<1e-4	slr2003	GO:0015293	<1e-4	slr0878	GO:0005575	<1e-4	slr1095	GO:0016772	0.0358
slr1472	GO:0044106	<1e-4	sll0871	GO:0019219	<1e-4	sll0676	GO:0008171	<1e-4	sll1072	GO:0044425	<1e-4	sll5033	GO:0016772	0.0358
slr1083	GO:0043412	<1e-4	sll0098	GO:0051234	<1e-4	slr1288	GO:0022803	<1e-4	slr1978	GO:0044446	<1e-4	sll1306	GO:0016772	0.0358
sll1527	GO:0009142	<1e-4	slr2027	GO:0009165	<1e-4	slr0341	GO:0060089	<1e-4	sll1765	GO:0044422	<1e-4	ssl1792	GO:0016772	0.0358
sll0839	GO:0043933	<1e-4	slr0921	GO:0006066	<1e-4	slr1541	GO:0015077	<1e-4	slr1576	GO:0043226	<1e-4	sll1400	GO:0016772	0.0358
slr1809	GO:0072528	<1e-4	slr1081	GO:0044283	<1e-4	sll0488	GO:0016829	<1e-4	slr0480	GO:0044464	<1e-4	slr1864	GO:0016772	0.0358
sll0860	GO:0009892	<1e-4	sll0294	GO:0019362	<1e-4	slr1800	GO:0022838	<1e-4	sll1239	GO:0044424	<1e-4	sll0167	GO:0016772	0.0358
sll1912	GO:0006732	<1e-4	ssr6024	GO:0009150	<1e-4	sll5089	GO:0016817	<1e-4	slr0169	GO:0044446	<1e-4	slr0199	GO:0016772	0.0358
slr7024	GO:0044260	<1e-4	sll0585	GO:0019752	<1e-4	sll0394	GO:0016818	<1e-4	slr0598	GO:0044424	<1e-4	sll7062	GO:0016772	0.0358
sll0670	GO:0005996	<1e-4	sll1698	GO:0009987	<1e-4	slr0262	GO:0022891	<1e-4	sll1472	GO:0044422	<1e-4	ssr3189	GO:0016772	0.0358
ssr1499	GO:0019219	<1e-4	ssr6086	GO:0019362	<1e-4	ssr2318	GO:0022803	<1e-4	slr0264	GO:0043226	<1e-4	slr0821	GO:0016772	0.0358
sll0658	GO:0031640	<1e-4	sll0525	GO:0006732	<1e-4	sll0854	GO:0016462	<1e-4	ssl1255	GO:0005575	<1e-4	slr0870	GO:0016772	0.0358
sll0543	GO:0080090	<1e-4	slr7024	GO:0019751	<1e-4	sll1961	GO:0015075	<1e-4	slr2118	GO:0044422	<1e-4	sll1160	GO:0016772	0.0358
slr0552	GO:0052188	<1e-4	sll1902	GO:0051716	<1e-4	slr1944	GO:0022836	<1e-4	sll1680	GO:0044444	<1e-4	slr1753	GO:0016772	0.0358
sll1142	GO:0071842	<1e-4	sll0608	GO:0044275	<1e-4	sll0710	GO:0015405	<1e-4	sll1503	GO:0044446	<1e-4	sll8032	GO:0016772	0.0358
slr0914	GO:0009394	<1e-4	ssl5007	GO:0005996	<1e-4	sll1738	GO:0015267	<1e-4	slr0967	GO:0044424	<1e-4	slr2032	GO:0016772	0.0358
slr1677	GO:0009199	<1e-4	slr0142	GO:0044283	<1e-4	slr1565	GO:0015291	<1e-4	ssr6002	GO:0044464	<1e-4	slr0606	GO:0016772	0.0358
sll1717	GO:0006091	<1e-4	sll5069	GO:0019362	<1e-4	slr0416	GO:0022803	<1e-4	slr1104	GO:0044446	<1e-4	slr1920	GO:0016772	0.0358
slr7014	GO:0009308	<1e-4	sll0456	GO:0016054	<1e-4	sll1063	GO:0022891	<1e-4	sll0381	GO:0044446	<1e-4	slr0912	GO:0016772	0.0358
slr0199	GO:0043648	<1e-4	slr2070	GO:0055082	<1e-4	ssr6062	GO:0015075	<1e-4	slr1599	GO:0044446	<1e-4	sll1509	GO:0016772	0.0358
sll0995	GO:0046394	<1e-4	sll8011	GO:0044282	<1e-4	slr1767	GO:0022803	<1e-4	slr1676	GO:0044422	<1e-4	slr2105	GO:0016772	0.0358
sll0381	GO:0009991	<1e-4	ssr1951	GO:0050896	<1e-4	sll0442	GO:0016818	<1e-4	slr0337	GO:0005575	<1e-4	slr6031	GO:0016772	0.0358
sll0072	GO:0016053	<1e-4	slr1195	GO:0046907	<1e-4	sll1373	GO:0016746	<1e-4	sll0822	GO:0044424	<1e-4	slr6008	GO:0016772	0.0358
sll0854	GO:0055082	<1e-4	sll2011	GO:0072527	<1e-4	ssl2971	GO:0005342	<1e-4	slr0514	GO:0044444	<1e-4	sll1961	GO:0016772	0.0358
sll7070	GO:0072527	<1e-4	slr5118	GO:0019222	<1e-4	slr1186	GO:0022838	<1e-4	ssl3379	GO:0005575	<1e-4	slr0398	GO:0016772	0.0358
sll1036	GO:0019752	<1e-4	slr0172	GO:0018130	<1e-4	slr1923	GO:0015077	<1e-4	slr1576	GO:0044424	<1e-4	slr7016	GO:0016772	0.0358
sll0775	GO:0046164	<1e-4	sll0854	GO:0048518	<1e-4	slr1990	GO:0022836	<1e-4	slr0960	GO:0005575	<1e-4	sll1783	GO:0016772	0.0358
ssl5091	GO:0010468	<1e-4	slr0975	GO:0051347	<1e-4	sll1132	GO:0043167	<1e-4	ssl8003	GO:0005575	<1e-4	slr1914	GO:0016772	0.0358
slr1303	GO:0071554	<1e-4	sll7077	GO:0009059	<1e-4	ssr1114	GO:0022892	<1e-4	sll1095	GO:0044424	<1e-4	sll1022	GO:0016772	0.0358
slr0326	GO:0042180	<1e-4	slr1365	GO:0006576	<1e-4	sll0189	GO:0015291	<1e-4	sll1469	GO:0044464	<1e-4	ssl3379	GO:0016772	0.0358
sll1442	GO:0006793	<1e-4	sll0994	GO:0080090	<1e-4	slr1025	GO:0015077	<1e-4	sll1173	GO:0043226	<1e-4	slr6006	GO:0016772	0.0358
sll0553	GO:0009132	<1e-4	sll1021	GO:0009150	<1e-4	slr0689	GO:0043492	<1e-4	sll0623	GO:0044464	<1e-4	ssr0335	GO:0044237	0.0357
sll1381	GO:0034641	<1e-4	slr1290	GO:0046128	<1e-4	slr2121	GO:0016818	<1e-4	slr1544	GO:0044425	<1e-4	sll1232	GO:0044237	0.0357
sll0101	GO:0009892	<1e-4	sll0994	GO:0006082	<1e-4	slr0270	GO:0060089	<1e-4	slr0302	GO:0005575	<1e-4	sll1638	GO:0044237	0.0357
sll0007	GO:0044248	<1e-4	slr0211	GO:0001932	<1e-4	slr0888	GO:0015405	<1e-4	sll1433	GO:0044444	<1e-4	sll0181	GO:0044237	0.0357
slr0317	GO:0044271	<1e-4	sll1751	GO:0060255	<1e-4	sll0996	GO:0015399	<1e-4	slr1363	GO:0005575	<1e-4	sll0994	GO:0044237	0.0357
sll6054	GO:0008610	<1e-4	slr1097	GO:0009260	<1e-4	sll1530	GO:0022857	<1e-4	sll1949	GO:0044424	<1e-4	ssl2920	GO:0044237	0.0357
slr6073	GO:0065007	<1e-4	sll0503	GO:0044255	<1e-4	sll1921	GO:0016746	<1e-4	slr1273	GO:0044464	<1e-4	slr1546	GO:0044237	0.0357
slr0592	GO:0060255	<1e-4	slr1507	GO:0009893	<1e-4	slr0456	GO:0016741	<1e-4	sll0931	GO:0044446	<1e-4	sll1400	GO:0044237	0.0357
sll0436	GO:0009059	<1e-4	slr0313	GO:0071842	<1e-4	sll1542	GO:0043492	<1e-4	sll0147	GO:0044446	<1e-4	sll0102	GO:0044237	0.0357
sll0022	GO:0090304	<1e-4	slr1926	GO:0009308	<1e-4	slr0192	GO:0022890	<1e-4	slr0960	GO:0044444	<1e-4	sll1414	GO:0044237	0.0357
ssr7084	GO:0009056	<1e-4	ssr0332	GO:0009132	<1e-4	slr1493	GO:0016817	<1e-4	slr1162	GO:0044422	<1e-4	slr7092	GO:0044237	0.0357
slr0712	GO:0009893	<1e-4	slr0912	GO:0072524	<1e-4	sll0888	GO:0022836	<1e-4	slr0303	GO:0005575	<1e-4	ssr7036	GO:0044237	0.0357
slr1273	GO:0045184	<1e-4	slr1847	GO:0034641	<1e-4	sll1472	GO:0022857	<1e-4	ssr2848	GO:0043226	<1e-4	slr6013	GO:0044237	0.0357
sll8002	GO:0042180	<1e-4	ssr2333	GO:0022607	<1e-4	sll0508	GO:0016818	<1e-4	sll0763	GO:0005575	<1e-4	slr7011	GO:0044237	0.0357
sll0602	GO:0031326	<1e-4	sll1052	GO:0071496	<1e-4	slr0076	GO:0022891	<1e-4	sll0177	GO:0043226	<1e-4	slr6015	GO:0044237	0.0357
sll1510	GO:0009889	<1e-4	sll1965	GO:0046164	<1e-4	sll0863	GO:0022890	<1e-4	slr1762	GO:0044422	<1e-4	slr7023	GO:0044237	0.0357
sll1658	GO:0008150	<1e-4	ssr2787	GO:0042180	<1e-4	sll1882	GO:0016462	<1e-4	slr1789	GO:0044422	<1e-4	slr7097	GO:0044237	0.0357
ssr7036	GO:0009161	<1e-4	slr1495	GO:0009987	<1e-4	sll1135	GO:0060089	<1e-4	slr1103	GO:0044422	<1e-4	slr6047	GO:0044237	0.0357
slr6091	GO:0019362	<1e-4	sll1835	GO:0009394	<1e-4	slr1150	GO:0015405	<1e-4	sll0446	GO:0044424	<1e-4	sll0872	GO:0044237	0.0357
ssr6026	GO:0046128	<1e-4	sll0860	GO:0006631	<1e-4	sll0096	GO:0042623	<1e-4	sml0011	GO:0044464	<1e-4	sll1062	GO:0044237	0.0357
slr0345	GO:0065007	<1e-4	sll7063	GO:0006576	<1e-4	sll0788	GO:0022892	<1e-4	sll1344	GO:0044446	<1e-4	sll0174	GO:0044237	0.0357
slr1034	GO:0031323	<1e-4	sll1950	GO:0052111	<1e-4	sll0751	GO:0016462	<1e-4	sll1192	GO:0043226	<1e-4	sll1217	GO:0044237	0.0357

sll5006	GO:0009132	<1e-4	sll1965	GO:0009889	<1e-4	sll1942	GO:0015399	<1e-4	sll0736	GO:0044425	<1e-4	sll1880	GO:0044237	0.0357
slr1470	GO:0048523	<1e-4	sll0786	GO:0019222	<1e-4	ssl5100	GO:0022838	<1e-4	slr2125	GO:0044424	<1e-4	slr7016	GO:0044237	0.0357
sll0249	GO:0043412	<1e-4	sll1730	GO:0050794	<1e-4	slr1726	GO:0043167	<1e-4	slr1900	GO:0044424	<1e-4	slr1150	GO:0044237	0.0357
slr5018	GO:0006753	<1e-4	slr0269	GO:0009142	<1e-4	ssl7039	GO:0015405	<1e-4	sll0473	GO:0044422	<1e-4	slr5017	GO:0044237	0.0357
sll0361	GO:0019362	<1e-4	slr0813	GO:0044271	<1e-4	slr6005	GO:0016462	<1e-4	slr1978	GO:0005575	<1e-4	slr1365	GO:0044237	0.0357
slr1415	GO:0019219	<1e-4	slr6031	GO:0034641	<1e-4	sll1251	GO:0008171	<1e-4	sll1267	GO:0044422	<1e-4	slr1462	GO:0044237	0.0357
slr1391	GO:0009117	<1e-4	sll0294	GO:0048522	<1e-4	sll0688	GO:0015405	<1e-4	sll0369	GO:0044446	<1e-4	sll0369	GO:0044237	0.0357
sll0854	GO:0006720	<1e-4	slr1376	GO:0008150	<1e-4	sll0857	GO:0022857	<1e-4	slr6039	GO:0043226	<1e-4	sll5034	GO:0044237	0.0357
slr1638	GO:0010468	<1e-4	sll2011	GO:0009123	<1e-4	sll7050	GO:0015399	<1e-4	sll0008	GO:0044446	<1e-4	slr0058	GO:0044237	0.0357
sll0406	GO:0050801	<1e-4	slr6029	GO:0006732	<1e-4	sll7078	GO:0015399	<1e-4	ssr2439	GO:0044464	<1e-4	ssr2912	GO:0044237	0.0357
slr1920	GO:0072527	<1e-4	slr5102	GO:0051171	<1e-4	slr1142	GO:0015075	<1e-4	slr0482	GO:0044425	<1e-4	ssr5120	GO:0044237	0.0357
sll0858	GO:0019219	<1e-4	sll0242	GO:0009263	<1e-4	slr5111	GO:0042623	<1e-4	slr0521	GO:0044446	<1e-4	slr0148	GO:0044237	0.0357
sll5030	GO:0051179	<1e-4	sll7050	GO:0022411	<1e-4	ssl5096	GO:0015291	<1e-4	ssr1407	GO:0044444	<1e-4	sll1401	GO:0044237	0.0357
slr1768	GO:0034641	<1e-4	slr1573	GO:0046483	<1e-4	slr5013	GO:0003674	<1e-4	slr1590	GO:0044464	<1e-4	sll5032	GO:0044237	0.0357
sll0913	GO:0050789	<1e-4	ssr0335	GO:0060255	<1e-4	sll0225	GO:0060089	<1e-4	slr1484	GO:0044425	<1e-4	ssl5007	GO:0044237	0.0357
slr1495	GO:0052111	<1e-4	sll1201	GO:0008150	<1e-4	slr1438	GO:0003674	<1e-4	slr0954	GO:0044424	<1e-4	sll0760	GO:0044237	0.0357
sll0060	GO:0048522	<1e-4	ssr2047	GO:0051171	<1e-4	sll1396	GO:0015077	<1e-4	ssr6078	GO:0044422	<1e-4	ssr6048	GO:0044237	0.0357
sll1757	GO:0009991	<1e-4	ssr2998	GO:0009892	<1e-4	slr1546	GO:0015293	<1e-4	slr2010	GO:0044424	<1e-4	slr0362	GO:0044237	0.0357
ssr1768	GO:0031640	<1e-4	slr0709	GO:0001932	<1e-4	ssl3379	GO:0022892	<1e-4	sll0230	GO:0005575	<1e-4	slr1600	GO:0044237	0.0357
sll0335	GO:0008150	<1e-4	slr8022	GO:0019222	<1e-4	ssl5045	GO:0022891	<1e-4	slr0667	GO:0044446	<1e-4	sll1769	GO:0044237	0.0357
slr0818	GO:0016054	<1e-4	slr1886	GO:0006753	<1e-4	slr0325	GO:0060089	<1e-4	slr0468	GO:0044424	<1e-4	sll1651	GO:0044237	0.0357
sll1267	GO:0009056	<1e-4	ssr1256	GO:0048522	<1e-4	sll0147	GO:0015075	<1e-4	slr0172	GO:0005575	<1e-4	slr5018	GO:0044237	0.0357
sll0676	GO:0008150	<1e-4	slr1083	GO:0051188	<1e-4	ssl0467	GO:0042623	<1e-4	slr0082	GO:0044446	<1e-4	sll0406	GO:0044237	0.0357
slr7102	GO:0006732	<1e-4	slr0423	GO:0055114	<1e-4	ssr1155	GO:0022836	<1e-4	slr0106	GO:0005575	<1e-4	sll7047	GO:0044237	0.0357
ssr3154	GO:0080090	<1e-4	sll0400	GO:0044283	<1e-4	slr1923	GO:0016746	<1e-4	sll1714	GO:0044464	<1e-4	sgl0001	GO:0044237	0.0357
sll0085	GO:0034660	<1e-4	sll1486	GO:0051716	<1e-4	sll1763	GO:0015075	<1e-4	sll0274	GO:0044425	<1e-4	slr6007	GO:0044237	0.0357
sll1203	GO:0009142	<1e-4	slr0376	GO:0072528	<1e-4	slr0865	GO:0043167	<1e-4	slr0106	GO:0044425	<1e-4	sll0423	GO:0044237	0.0357
ssr2843	GO:0009142	<1e-4	sll0238	GO:0009165	<1e-4	slr1566	GO:0016741	<1e-4	slr1768	GO:0044422	<1e-4	sll0176	GO:0044237	0.0357
slr0976	GO:0044281	<1e-4	slr0885	GO:0019752	<1e-4	slr0250	GO:0015075	<1e-4	slr0924	GO:0043226	<1e-4	slr6031	GO:0044237	0.0357
slr1095	GO:0042180	<1e-4	sll0327	GO:0022607	<1e-4	ssl7045	GO:0043492	<1e-4	slr0975	GO:0005575	<1e-4	slr7081	GO:0044237	0.0357
sll1571	GO:0009141	<1e-4	slr0695	GO:0071496	<1e-4	ssr6083	GO:0060089	<1e-4	slr1170	GO:0043226	<1e-4	sll5062	GO:0044237	0.0357
sll0448	GO:0044106	<1e-4	slr1097	GO:0052111	<1e-4	slr0334	GO:0022803	<1e-4	slr0023	GO:0005575	<1e-4	slr1611	GO:0044237	0.0357
sll1765	GO:0055086	<1e-4	sll1736	GO:0051818	<1e-4	slr0602	GO:0043492	<1e-4	sll0742	GO:0044424	<1e-4	sll7064	GO:0044237	0.0357
sll5130	GO:0044260	<1e-4	ssr3122	GO:0042455	<1e-4	ssr2317	GO:0016462	<1e-4	ssl1046	GO:0043226	<1e-4	sll0786	GO:0044237	0.0357
sll1424	GO:0051234	<1e-4	slr6022	GO:0009059	<1e-4	sll0994	GO:0016462	<1e-4	sll0875	GO:0044446	<1e-4	sll1510	GO:0044237	0.0357
slr1087	GO:0072521	<1e-4	sll1512	GO:0006631	<1e-4	slr0554	GO:0015405	<1e-4	sll0069	GO:0005575	<1e-4	slr1923	GO:0044237	0.0357
ssl1004	GO:0009124	<1e-4	slr1084	GO:0009987	<1e-4	slr1303	GO:0015293	<1e-4	ssr2553	GO:0044446	<1e-4	slr0890	GO:0044237	0.0357
sll0737	GO:0031323	<1e-4	sll1319	GO:0009161	<1e-4	ssr5011	GO:0016817	<1e-4	sll1262	GO:0043226	<1e-4	sll0265	GO:0044237	0.0357
slr1799	GO:0072522	<1e-4	slr1915	GO:0072521	<1e-4	sll8033	GO:0016817	<1e-4	sll1547	GO:0044446	<1e-4	ssl5096	GO:0044237	0.0357
slr7071	GO:0034641	<1e-4	sll1902	GO:0019751	<1e-4	sll1123	GO:0022890	<1e-4	slr2060	GO:0044422	<1e-4	slr0870	GO:0044237	0.0357
sll1025	GO:0009132	<1e-4	slr6029	GO:0065007	<1e-4	slr1865	GO:0015399	<1e-4	ssr2317	GO:0005575	<1e-4	ssl7046	GO:0044237	0.0357
ssr2781	GO:0050896	<1e-4	sll5033	GO:0055114	<1e-4	slr1866	GO:0015075	<1e-4	sll1542	GO:0043226	<1e-4	sll1884	GO:0044237	0.0357
slr1288	GO:0043412	<1e-4	sll0864	GO:0043648	<1e-4	slr0848	GO:0016817	<1e-4	sll1106	GO:0044424	<1e-4	slr6021	GO:0044237	0.0357
slr0587	GO:0031640	<1e-4	sll1853	GO:0009309	<1e-4	slr0108	GO:0043167	<1e-4	sll1131	GO:0044422	<1e-4	sll0218	GO:0044237	0.0357
ssl3692	GO:0009126	<1e-4	ssr3402	GO:0019637	<1e-4	slr0592	GO:0022836	<1e-4	slr2032	GO:0044424	<1e-4	slr0199	GO:0044237	0.0357
slr5126	GO:0018130	<1e-4	ssr5074	GO:0009987	<1e-4	ssr2611	GO:0015291	<1e-4	sll0243	GO:0044422	<1e-4	ssl1004	GO:0044237	0.0357
ssr5120	GO:0006721	<1e-4	slr1807	GO:0050896	<1e-4	sgl0002	GO:0015293	<1e-4	slr0670	GO:0044446	<1e-4	slr1195	GO:0044237	0.0357
slr1603	GO:0043933	<1e-4	sll1307	GO:0044106	<1e-4	slr0586	GO:0022803	<1e-4	sll5097	GO:0005575	<1e-4	slr0356	GO:0044237	0.0357
sll0577	GO:0009144	<1e-4	slr6073	GO:0006811	<1e-4	slr0937	GO:0015267	<1e-4	slr7101	GO:0044446	<1e-4	ssl0738	GO:0044237	0.0357
sll1476	GO:0048522	<1e-4	slr2004	GO:0033013	<1e-4	slr5102	GO:0022891	<1e-4	sll0328	GO:0043226	<1e-4	slr1957	GO:0044237	0.0357
ssr1766	GO:0046128	<1e-4	sll7047	GO:0044281	<1e-4	sll1254	GO:0042623	<1e-4	sll1738	GO:0044444	<1e-4	slr0049	GO:0044237	0.0357
sll1380	GO:0031326	<1e-4	slr0664	GO:0009309	<1e-4	slr1819	GO:0022857	<1e-4	ssr6083	GO:0043226	<1e-4	ssr5020	GO:0044237	0.0357
slr5021	GO:0009141	<1e-4	slr1071	GO:0010556	<1e-4	sll0397	GO:0015399	<1e-4	slr6050	GO:0005575	<1e-4	sll5006	GO:0044237	0.0357
sll1089	GO:0050801	<1e-4	slr1391	GO:0071554	<1e-4	slr1537	GO:0015267	<1e-4	sll0071	GO:0044425	<1e-4	slr1864	GO:0044237	0.0357

sll1552	GO:0045184	<1e-4	ssr0761	GO:0009260	<1e-4	ssl1552	GO:0005342	<1e-4	sll1960	GO:0044424	<1e-4	slr7025	GO:0044237	0.0357
sll0584	GO:0009893	<1e-4	slr1082	GO:0051716	<1e-4	sll0230	GO:0022892	<1e-4	sll0451	GO:0044422	<1e-4	slr0959	GO:0044237	0.0357
slr0285	GO:0009259	<1e-4	sll1691	GO:0009987	<1e-4	sll1797	GO:0016817	<1e-4	sll0269	GO:0005575	<1e-4	ssr6026	GO:0044237	0.0357
sll5033	GO:0006082	<1e-4	slr1222	GO:0050801	<1e-4	sll1722	GO:0015267	<1e-4	sll1155	GO:0005575	<1e-4	sll0630	GO:0044237	0.0357
slr0930	GO:0051716	<1e-4	sll2007	GO:0009259	<1e-4	slr1169	GO:0016741	<1e-4	slr5023	GO:0044464	<1e-4	slr0806	GO:0044237	0.0357
sll1166	GO:0009991	<1e-4	ssr7036	GO:0009987	<1e-4	ssr7072	GO:0016746	<1e-4	slr0989	GO:0044464	<1e-4	slr0569	GO:0044237	0.0357
sll0072	GO:0072528	<1e-4	slr0294	GO:0006066	<1e-4	slr1799	GO:0022890	<1e-4	slr1398	GO:0005575	<1e-4	sll0867	GO:0044237	0.0357
sll0310	GO:0009126	<1e-4	slr1816	GO:0044283	<1e-4	slr2012	GO:0016746	<1e-4	sll1400	GO:0044424	<1e-4	ssr1499	GO:0044237	0.0357
slr0023	GO:0044248	<1e-4	sll0355	GO:0009161	<1e-4	slr0442	GO:0060089	<1e-4	sll1142	GO:0044425	<1e-4	sll6053	GO:0044237	0.0357
slr1505	GO:0043933	<1e-4	sll0310	GO:0060255	<1e-4	slr1169	GO:0008171	<1e-4	sll0359	GO:0005575	<1e-4	slr0398	GO:0044237	0.0357
slr1259	GO:0044248	<1e-4	slr1116	GO:0006793	<1e-4	slr2144	GO:0022892	<1e-4	ssr1499	GO:0044446	<1e-4	sll0328	GO:0044237	0.0357
ssr2972	GO:0009123	<1e-4	slr0111	GO:0033013	<1e-4	sml0011	GO:0005342	<1e-4	slr1117	GO:0044422	<1e-4	slr1464	GO:0044237	0.0357
slr0208	GO:0052188	<1e-4	sll0443	GO:0050896	<1e-4	slr0575	GO:0022891	<1e-4	sll0944	GO:0005575	<1e-4	slr0601	GO:0044237	0.0357
sll1390	GO:0016054	<1e-4	slr0157	GO:0009059	<1e-4	slr0142	GO:0015075	<1e-4	slr0625	GO:0044425	<1e-4	sll1477	GO:0004871	0.0357
sll1613	GO:0071840	<1e-4	slr1648	GO:0051188	<1e-4	sll1714	GO:0015291	<1e-4	ssr1951	GO:0044422	<1e-4	sll0446	GO:0004871	0.0357
ssr1768	GO:0019751	<1e-4	slr1117	GO:0006766	<1e-4	slr1568	GO:0022857	<1e-4	slr0930	GO:0044425	<1e-4	slr0642	GO:0004871	0.0357
sll1632	GO:0006163	<1e-4	sll1455	GO:0051347	<1e-4	sll0888	GO:0043167	<1e-4	slr0273	GO:0005575	<1e-4	sll1528	GO:0004871	0.0357
sll1735	GO:0016053	<1e-4	sll1586	GO:0022411	<1e-4	ssl6061	GO:0016818	<1e-4	sll0096	GO:0005575	<1e-4	ssr0692	GO:0004871	0.0357
slr0588	GO:0009991	<1e-4	slr1127	GO:0009132	<1e-4	slr0355	GO:0015291	<1e-4	ssr0692	GO:0044444	<1e-4	slr0147	GO:0004871	0.0357
sll1606	GO:0031326	<1e-4	sll1390	GO:0046128	<1e-4	ssr3129	GO:0008171	<1e-4	slr0773	GO:0005575	<1e-4	sll1913	GO:0005975	0.034
ssr6078	GO:0009165	<1e-4	slr1913	GO:0048878	<1e-4	sll1658	GO:0015405	<1e-4	sll0564	GO:0044424	<1e-4	slr0476	GO:0005975	0.034
ssl1046	GO:0016054	<1e-4	sll1388	GO:0050801	<1e-4	sll1925	GO:0022803	<1e-4	slr6028	GO:0044444	<1e-4	sll1671	GO:0005975	0.034
sll0168	GO:0006082	<1e-4	slr0821	GO:0048523	<1e-4	sll1651	GO:0015291	<1e-4	slr1407	GO:0044446	<1e-4	sll1426	GO:0005975	0.034
slr0670	GO:0072522	<1e-4	slr1062	GO:0046394	<1e-4	slr1807	GO:0022890	<1e-4	slr0305	GO:0044424	<1e-4	sll0864	GO:0005975	0.034
slr0806	GO:0016053	<1e-4	slr0581	GO:0009141	<1e-4	slr0552	GO:0015075	<1e-4	slr1814	GO:0044424	<1e-4	slr0852	GO:0005975	0.034
sll1611	GO:0071840	<1e-4	slr1484	GO:0019751	<1e-4	slr0702	GO:0015077	<1e-4	slr0104	GO:0005575	<1e-4	sll1219	GO:0005975	0.034
slr0455	GO:0008610	<1e-4	ssl2065	GO:0009117	<1e-4	sll1378	GO:0042623	<1e-4	slr2071	GO:0044446	<1e-4	slr0304	GO:0005975	0.034
sll0524	GO:0046483	<1e-4	ssr6003	GO:0048522	<1e-4	sll1191	GO:0016741	<1e-4	sll1086	GO:0044444	<1e-4	slr1391	GO:0005975	0.034
slr0208	GO:0008150	<1e-4	sll0661	GO:0008150	<1e-4	slr1084	GO:0015399	<1e-4	sll0854	GO:0044424	<1e-4	slr1612	GO:0005975	0.034
ssr1114	GO:0055114	<1e-4	slr2010	GO:0045184	<1e-4	slr6049	GO:0022832	<1e-4	sll7069	GO:0044446	<1e-4	slr1025	GO:0005975	0.034
sll1934	GO:0034660	<1e-4	sll0047	GO:0009123	<1e-4	sll1201	GO:0060089	<1e-4	sll1634	GO:0044422	<1e-4	slr0871	GO:0005975	0.034
slr6049	GO:0009126	<1e-4	sll0661	GO:0009199	<1e-4	sll0615	GO:0022892	<1e-4	slr1571	GO:0044425	<1e-4	sll0296	GO:0005975	0.034
slr1611	GO:0034654	<1e-4	ssr5019	GO:0055082	<1e-4	sll1766	GO:0022832	<1e-4	slr1762	GO:0044425	<1e-4	sll1455	GO:0005975	0.034
slr1657	GO:0048518	<1e-4	slr6088	GO:0043436	<1e-4	slr2010	GO:0016818	<1e-4	sll1938	GO:0043226	<1e-4	sll0096	GO:0005975	0.034
ssr2201	GO:0019219	<1e-4	slr1098	GO:0019751	<1e-4	sll0553	GO:0015291	<1e-4	slr1425	GO:0044422	<1e-4	slr0211	GO:0005975	0.034
slr2010	GO:0006066	<1e-4	sll1372	GO:0045184	<1e-4	slr0587	GO:0016746	<1e-4	slr1339	GO:0005575	<1e-4	sll0283	GO:0005975	0.034
sll0360	GO:0051818	<1e-4	sll5046	GO:0009142	<1e-4	slr0967	GO:0042623	<1e-4	sll0832	GO:0044424	<1e-4	slr1674	GO:0005975	0.034
slr2004	GO:0006163	<1e-4	sll1611	GO:0019222	<1e-4	sll0997	GO:0015291	<1e-4	slr1056	GO:0044422	<1e-4	sll1586	GO:0005975	0.034
slr1110	GO:0009117	<1e-4	ssr0761	GO:0071842	<1e-4	ssl7038	GO:0022890	<1e-4	ssl5027	GO:0043226	<1e-4	sll0854	GO:0005975	0.034
slr1600	GO:0005996	<1e-4	slr1047	GO:0019219	<1e-4	sll1251	GO:0022857	<1e-4	slr1270	GO:0044422	<1e-4	sll0508	GO:0005975	0.034
slr1819	GO:0031326	<1e-4	slr7059	GO:0044248	<1e-4	slr0199	GO:0043167	<1e-4	slr6068	GO:0005575	<1e-4	sll0360	GO:0005975	0.034
slr1923	GO:0071554	<1e-4	ssl2717	GO:0008150	<1e-4	sll0237	GO:0022803	<1e-4	slr1762	GO:0005575	<1e-4	slr1533	GO:0005975	0.034
ssr6089	GO:0008150	<1e-4	sll1504	GO:0046164	<1e-4	ssr2201	GO:0005342	<1e-4	ssl5098	GO:0044464	<1e-4	slr1519	GO:0005975	0.034
sll1315	GO:0009893	<1e-4	slr1066	GO:0009308	<1e-4	ssr1375	GO:0022838	<1e-4	ssr6089	GO:0044425	<1e-4	slr1752	GO:0003723	0.0339
slr0702	GO:0044255	<1e-4	sll0160	GO:0072522	<1e-4	slr2110	GO:0022857	<1e-4	sll0008	GO:0044464	<1e-4	slr0865	GO:0003723	0.0339
sll0871	GO:0051171	<1e-4	sll1658	GO:0048878	<1e-4	slr1557	GO:0022803	<1e-4	sll1640	GO:0044425	<1e-4	slr7091	GO:0003723	0.0339
sll1252	GO:0005996	<1e-4	ssr5092	GO:0065007	<1e-4	sll0775	GO:0022832	<1e-4	sll0749	GO:0043226	<1e-4	sll0249	GO:0003723	0.0339
slr0509	GO:0044255	<1e-4	slr1327	GO:0046128	<1e-4	sll1092	GO:0016462	<1e-4	slr1886	GO:0044424	<1e-4	ssl7022	GO:0003723	0.0339
ssr2422	GO:0009893	<1e-4	slr1196	GO:0009165	<1e-4	slr1812	GO:0015077	<1e-4	slr0181	GO:0005575	<1e-4	sll1373	GO:0003723	0.0339
sll0335	GO:0046164	<1e-4	sll0854	GO:0052111	<1e-4	ssl5065	GO:0015405	<1e-4	sll1049	GO:0044464	<1e-4	ssr0336	GO:0003723	0.0339
slr1951	GO:0051179	<1e-4	ssl5103	GO:0009057	<1e-4	sll0539	GO:0005342	<1e-4	ssl0410	GO:0044464	<1e-4	slr0241	GO:0003723	0.0339
sll1068	GO:0055114	<1e-4	sll7062	GO:0072527	<1e-4	ssr2754	GO:0022803	<1e-4	sgl0001	GO:0044444	<1e-4	sll5030	GO:0003723	0.0339
sll1106	GO:0051246	<1e-4	slr1276	GO:0055082	<1e-4	slr0967	GO:0060089	<1e-4	slr7098	GO:0044422	<1e-4	ssl7039	GO:0003723	0.0339
slr5127	GO:0010556	<1e-4	sll0553	GO:0009889	<1e-4	sll0350	GO:0015405	<1e-4	slr0373	GO:0005575	<1e-4	slr1290	GO:0003723	0.0339

slr5087	GO:0009260	<1e-4	sll0595	GO:0019751	<1e-4	sll1163	GO:0016462	<1e-4	sll0384	GO:0044446	<1e-4	slr2027	GO:0003723	0.0339		
sll1240	GO:0071496	<1e-4	slr1917	GO:0034641	<1e-4	ssr1766	GO:0043167	<1e-4	sll0487	GO:0043226	<1e-4	sll1131	GO:0003723	0.0339		
sll0096	GO:0048519	<1e-4	slr0801	GO:0071840	<1e-4	sll1006	GO:0022836	<1e-4	slr6033	GO:0044425	<1e-4	sll8019	GO:0003723	0.0339		
slr0262	GO:0009132	<1e-4	sll0444	GO:0008150	<1e-4	slr0269	GO:0022836	<1e-4	ssl0350	GO:0005575	<1e-4	sll0858	GO:0003723	0.0339		
sll0176	GO:0009059	<1e-4	sll0804	GO:0046394	<1e-4	slr5024	GO:0043492	<1e-4	sll0249	GO:0044424	<1e-4	sll7033	GO:0003723	0.0339		
slr6067	GO:0048522	<1e-4	sll0160	GO:0009057	<1e-4	slr0876	GO:0015267	<1e-4	sll0237	GO:0044444	<1e-4	ssr3402	GO:0003723	0.0339		
slr1288	GO:0006766	<1e-4	sll0181	GO:0051179	<1e-4	slr1619	GO:0022838	<1e-4	sll1265	GO:0005575	<1e-4	sll0625	GO:0003723	0.0339		
sll0102	GO:0046128	<1e-4	sll1698	GO:0071554	<1e-4	sll5090	GO:0015293	<1e-4	slr0909	GO:0044464	<1e-4	slr5073	GO:0003723	0.0339		
sll0162	GO:0046394	<1e-4	ssl3549	GO:0044003	<1e-4	sll0160	GO:0003674	<1e-4	slr0169	GO:0044422	<1e-4	slr1681	GO:0003723	0.0339		
ssr3154	GO:0009132	<1e-4	sll7047	GO:0006732	<1e-4	sll0995	GO:0015077	<1e-4	slr0082	GO:0044425	<1e-4	slr6091	GO:0003723	0.0339		
sll0473	GO:0009987	<1e-4	slr1537	GO:0048522	<1e-4	slr1702	GO:0015405	<1e-4	slr5116	GO:0044422	<1e-4	sll0691	GO:0003723	0.0339		
sll1751	GO:0034654	<1e-4	slr1958	GO:0009142	<1e-4	sll0242	GO:0004872	<1e-4	sll1913	GO:0044424	<1e-4	sll1052	GO:0003723	0.0339		
slr1886	GO:0006091	<1e-4	slr0863	GO:0044106	<1e-4	sll1630	GO:0016462	<1e-4	slr0787	GO:0005575	<1e-4	sll0696	GO:0003723	0.0339		
ssr6083	GO:0016053	<1e-4	slr1570	GO:0009892	<1e-4	sll0925	GO:0060089	<1e-4	slr0613	GO:0005575	<1e-4	sll0172	GO:0003723	0.0339		
slr1618	GO:0009124	<1e-4	slr1614	GO:0051186	<1e-4	slr0937	GO:0016462	<1e-4	ssl7038	GO:0005575	<1e-4	sll1289	GO:0003723	0.0339		
sll1571	GO:0080090	<1e-4	slr1074	GO:0051716	<1e-4	sll1380	GO:0022891	<1e-4	slr0487	GO:0005575	<1e-4	slr0300	GO:0003723	0.0339		
slr1667	GO:0009132	<1e-4	sll0238	GO:0090304	<1e-4	slr0516	GO:0016817	<1e-4	slr0692	GO:0044425	<1e-4	slr1627	GO:0003723	0.0339		
slr1178	GO:0009260	<1e-4	slr1721	GO:0022607	<1e-4	slr1288	GO:0015291	<1e-4	slr2049	GO:0044424	<1e-4	slr6088	GO:0003723	0.0339		
slr0581	GO:0031323	<1e-4	slr1188	GO:0010556	<1e-4	sll1192	GO:0022857	<1e-4	slr1613	GO:0044464	<1e-4	slr0755	GO:0003723	0.0339		
ssr3570	GO:0034641	<1e-4	ssl0787	GO:0006220	<1e-4	sll0085	GO:0015075	<1e-4	ssr2333	GO:0044425	<1e-4	sll1476	GO:0003723	0.0339		
slr0708	GO:0034641	<1e-4	sll1766	GO:0006720	<1e-4	sll1735	GO:0008171	<1e-4	slr0740	GO:0044422	<1e-4	sll8011	GO:0003723	0.0339		
slr2000	GO:0009057	<1e-4	slr0111	GO:0051234	<1e-4	sll0327	GO:0022836	<1e-4	ssl2807	GO:0044444	<1e-4	ssr1155	GO:0003723	0.0339		
ssr3532	GO:0045184	<1e-4	slr0888	GO:0016053	<1e-4	slr2052	GO:0060089	<1e-4	slr1614	GO:0044464	<1e-4	slr2004	GO:0003723	0.0339		
sll1390	GO:0043933	<1e-4	slr1398	GO:0046394	<1e-4	sll1262	GO:0016462	<1e-4	slr0596	GO:0044425	<1e-4	ssl5114	GO:0003723	0.0339		
ssr5106	GO:0009165	<1e-4	slr0364	GO:0006091	<1e-4	sll0867	GO:0015399	<1e-4	sll1527	GO:0043226	<1e-4	slr7060	GO:0003723	0.0339		
sll0069	GO:0022411	<1e-4	ssl2065	GO:0022607	<1e-4	slr5126	GO:0016818	<1e-4	sll1693	GO:0044422	<1e-4	slr6106	GO:0003723	0.0339		
sll1938	GO:0006066	<1e-4	sll7033	GO:0010468	<1e-4	sll0756	GO:0043167	<1e-4	sll1006	GO:0044422	<1e-4	ssl7045	GO:0003723	0.0339		
sll0048	GO:0019751	<1e-4	slr0489	GO:0046394	<1e-4	slr0184	GO:0003674	<1e-4	slr1183	GO:0044424	<1e-4	slr7099	GO:0003723	0.0339		
slr1619	GO:0044283	<1e-4	sll1913	GO:0080090	<1e-4	slr0498	GO:0022832	<1e-4	sll1696	GO:0044446	<1e-4	sll8027	GO:0003723	0.0339		
slr0935	GO:0018130	<1e-4	slr0941	GO:0043436	<1e-4	slr1230	GO:0003674	<1e-4	sll5033	GO:0044446	<1e-4	slr2070	GO:0003723	0.0339		
slr1196	GO:0071841	<1e-4	sll0847	GO:0008610	<1e-4	sll0010	GO:0022803	<1e-4	sll1915	GO:0005575	<1e-4	slr0656	GO:0003723	0.0339		
slr1053	GO:0051716	<1e-4	slr0852	GO:0044271	<1e-4	sll1863	GO:0015399	<1e-4	slr1203	GO:0044444	<1e-4	slr1406	GO:0003723	0.0339		
slr0196	GO:0009309	<1e-4	slr8014	GO:0001932	<1e-4	ssl3451	GO:0016746	<1e-4	slr1537	GO:0044444	<1e-4	sll1832	GO:0003723	0.0339		
sll0861	GO:0051246	<1e-4	sll0553	GO:0072528	<1e-4	slr1365	GO:0016818	<1e-4	sll0508	GO:0005575	<1e-4	slr2110	GO:0003723	0.0339		
sll0354	GO:0016052	<1e-4	sll1563	GO:0044271	<1e-4	sll0216	GO:0015405	<1e-4	sll0048	GO:0044444	<1e-4	slr6028	GO:0003723	0.0339		
ssr2711	GO:0019752	<1e-4	sll6052	GO:0019752	<1e-4	sll0189	GO:0022892	<1e-4	sll1348	GO:0044425	<1e-4	ssr2802	GO:0003723	0.0339		
slr1907	GO:0071496	<1e-4	slr7094	GO:0009124	<1e-4	sll0177	GO:0016741	<1e-4	ssr3570	GO:0044464	<1e-4	sll0405	GO:0003723	0.0339		
slr1413	GO:0071842	<1e-4	sll1939	GO:0019362	<1e-4	sll1250	GO:0022838	<1e-4	slr6075	GO:0005575	<1e-4	slr0217	GO:0003723	0.0339		
slr0625	GO:0006720	<1e-4	sll1025	GO:0009124	<1e-4	slr1809	GO:0005342	<1e-4	sll1722	GO:0043226	<1e-4	slr0976	GO:0003723	0.0339		
slr0655	GO:0008150	<1e-4	sll1751	GO:0019751	<1e-4	ssl5113	GO:0015077	<1e-4	sll1062	GO:0044464	<1e-4	ssl7038	GO:0003723	0.0339		
slr1235	GO:0042455	<1e-4	sll0293	GO:0009117	<1e-4	sll1949	GO:0016746	<1e-4	slr1397	GO:0005575	<1e-4	sll7050	GO:0003723	0.0339		
ssr5020	GO:0009893	<1e-4	ssr6003	GO:0051818	<1e-4	slr1576	GO:0022892	<1e-4	slr0519	GO:0044422	<1e-4	slr0013	GO:0003723	0.0339		
sll5132	GO:0016053	<1e-4	ssr1391	GO:0044248	<1e-4	sll0787	GO:0022836	<1e-4	sll0381	GO:0043226	<1e-4	sll1380	GO:0003723	0.0339		
slr1110	GO:0071496	<1e-4	sll0982	GO:0006793	<1e-4	sll1638	GO:0016746	<1e-4	slr1150	GO:0044464	<1e-4	ssl7007	GO:0003723	0.0339		
sll0382	GO:0050789	<1e-4	sll1131	GO:0043549	<1e-4	slr0376	GO:0022803	<1e-4	slr0594	GO:0044446	<1e-4	sll6052	GO:0003723	0.0339		
sll1469	GO:0043933	<1e-4	sll0563	GO:0019637	<1e-4	ssr1768	GO:0022892	<1e-4	slr0317	GO:0044424	<1e-4	slr1079	GO:0003723	0.0339		
ssr6048	GO:0044260	<1e-4	ssr7084	GO:0006066	<1e-4	sll1063	GO:0016746	<1e-4	slr7013	GO:0044444	<1e-4	slr0313	GO:0003723	0.0339		
slr1103	GO:0042451	<1e-4	slr0810	GO:0006811	<1e-4	ssl8003	GO:0022892	<1e-4	slr0208	GO:0044422	<1e-4	sll0552	GO:0003723	0.0339		
sll0360	GO:0051171	<1e-4	ssr8047	GO:0006576	<1e-4	sll0525	GO:0015399	<1e-4	slr1648	GO:0044422	<1e-4	slr0914	GO:0003723	0.0339		
sll0071	GO:0009394	<1e-4	ssl1417	GO:0009892	<1e-4	slr1863	GO:0022890	<1e-4	sll1247	GO:0044422	<1e-4	slr5024	GO:0003723	0.0339		
sll8035	GO:0022411	<1e-4	sll1634	GO:0019222	<1e-4	slr0263	GO:0015075	<1e-4	sll5034	GO:0044422	<1e-4	slr1737	GO:0003723	0.0339		
ssr2962	GO:0009117	<1e-4	ssl2420	GO:0006793	<1e-4	sll0802	GO:0016818	<1e-4	sll1583	GO:0044424	<1e-4	sll8040	GO:0003723	0.0339		
sll1785	GO:0009892	<1e-4	slr1572	GO:0072521	<1e-4	ssr1258	GO:0022803	<1e-4	slr1223	GO:0044444	<1e-4	slr6051	GO:0003723	0.0339		
sll0872	GO:0010556	<1e-4	slr1624	GO:0009059	<1e-4	slr6074	GO:0016462	<1e-4	slr0393	GO:0044424	<1e-4	sll0565	GO:0003723	0.0339		

ssl8003	GO:0050794	<1e-4	slr6074	GO:0009142	<1e-4	slr1437	GO:0043167	<1e-4	slr0168	GO:0044422	<1e-4	sll1652	GO:0003723	0.0339
slr1052	GO:0009889	<1e-4	sll1201	GO:0006811	<1e-4	slr6075	GO:0015293	<1e-4	slr2103	GO:0044446	<1e-4	slr2060	GO:0003723	0.0339
slr1290	GO:0042180	<1e-4	ssl1377	GO:0051171	<1e-4	sll0597	GO:0003674	<1e-4	sll0923	GO:0044424	<1e-4	slr0106	GO:0003723	0.0339
ssl3383	GO:0006091	<1e-4	slr1128	GO:0044003	<1e-4	slr1923	GO:0015291	<1e-4	slr0590	GO:0044464	<1e-4	slr1875	GO:0003723	0.0339
slr0871	GO:0031640	<1e-4	slr0476	GO:0019219	<1e-4	slr1927	GO:0015399	<1e-4	sll0786	GO:0005575	<1e-4	ssl7074	GO:0003723	0.0339
slr1505	GO:0043170	<1e-4	slr0976	GO:0031326	<1e-4	slr1203	GO:0022803	<1e-4	sll1461	GO:0044424	<1e-4	slr5053	GO:0003723	0.0339
slr1384	GO:0006163	<1e-4	slr1047	GO:0006721	<1e-4	ssr1768	GO:0060089	<1e-4	slr0144	GO:0044444	<1e-4	slr6087	GO:0003723	0.0339
sll0062	GO:0034660	<1e-4	ssl3549	GO:0009262	<1e-4	slr0397	GO:0022891	<1e-4	ssr6046	GO:0044422	<1e-4	ssl1263	GO:0003723	0.0339
slr1104	GO:0048522	<1e-4	slr6021	GO:0009142	<1e-4	slr0326	GO:0015399	<1e-4	slr0514	GO:0044464	<1e-4	slr0863	GO:0003723	0.0339
slr7026	GO:0042180	<1e-4	ssl8033	GO:0048523	<1e-4	slr0725	GO:0022892	<1e-4	slr0142	GO:0044425	<1e-4	slr0575	GO:0003723	0.0339
sll1164	GO:0033013	<1e-4	slr0058	GO:0009142	<1e-4	slr1436	GO:0022890	<1e-4	slr1417	GO:0044446	<1e-4	slr1163	GO:0003723	0.0339
sll0397	GO:0001932	<1e-4	sll0641	GO:0016054	<1e-4	sll0268	GO:0022832	<1e-4	sll1469	GO:0044446	<1e-4	slr1101	GO:0003723	0.0339
slr0519	GO:0060255	<1e-4	sll0189	GO:0034641	<1e-4	sll0994	GO:0015293	<1e-4	sll5030	GO:0044425	<1e-4	slr7102	GO:0003723	0.0339
sll0103	GO:0048522	<1e-4	sll0397	GO:0051171	<1e-4	ssl1972	GO:0015293	<1e-4	sll0910	GO:0044444	<1e-4	slr6022	GO:0003723	0.0339
sll1265	GO:0080090	<1e-4	ssr7079	GO:0009144	<1e-4	slr0364	GO:0043167	<1e-4	ssl7039	GO:0044446	<1e-4	sll0736	GO:0003723	0.0339
slr0552	GO:0043436	<1e-4	slr0517	GO:0009144	<1e-4	sll1191	GO:0005342	<1e-4	slr0581	GO:0005575	<1e-4	ssr2972	GO:0003723	0.0339
slr1407	GO:0009991	<1e-4	sll1835	GO:0016053	<1e-4	sll0060	GO:0043167	<1e-4	ssl7042	GO:0044425	<1e-4	ssr7084	GO:0003723	0.0339
slr0273	GO:0009141	<1e-4	sll1586	GO:0009987	<1e-4	slr0784	GO:0008171	<1e-4	ssr1375	GO:0005575	<1e-4	ssr6024	GO:0003723	0.0339
slr1495	GO:0009056	<1e-4	ssl7007	GO:0006793	<1e-4	sll0419	GO:0015075	<1e-4	ssl1377	GO:0044446	<1e-4	sll1882	GO:0003723	0.0339
sll0361	GO:0009262	<1e-4	ssr2962	GO:0019751	<1e-4	ssr1499	GO:0016462	<1e-4	sll0022	GO:0044422	<1e-4	sll0442	GO:0003723	0.0339
sll1965	GO:0019222	<1e-4	sll0183	GO:0009132	<1e-4	sll0096	GO:0015075	<1e-4	sll1946	GO:0044425	<1e-4	slr0730	GO:0003723	0.0339
sll1389	GO:0009987	<1e-4	slr1956	GO:0005996	<1e-4	sll1414	GO:0022890	<1e-4	ssl2781	GO:0044444	<1e-4	slr0291	GO:0003723	0.0339
sll1863	GO:0045184	<1e-4	slr1441	GO:0034660	<1e-4	sll0360	GO:0015267	<1e-4	slr0146	GO:0044464	<1e-4	ssr6083	GO:0003723	0.0339
sll1737	GO:0034654	<1e-4	sll1573	GO:0048522	<1e-4	sll0071	GO:0008171	<1e-4	sll1940	GO:0005575	<1e-4	slr1513	GO:0003723	0.0339
sll0614	GO:0072528	<1e-4	slr0912	GO:0016054	<1e-4	ssr5092	GO:0022838	<1e-4	slr7025	GO:0005575	<1e-4	slr6068	GO:0003723	0.0339
slr0479	GO:0009259	<1e-4	slr0145	GO:0019222	<1e-4	slr1187	GO:0015293	<1e-4	sll0985	GO:0044464	<1e-4	slr0670	GO:0003723	0.0339
slr1880	GO:0009141	<1e-4	sll1191	GO:0044248	<1e-4	slr1053	GO:0016746	<1e-4	ssl6092	GO:0044425	<1e-4	sll0298	GO:0003723	0.0339
ssl1464	GO:0044283	<1e-4	sll2013	GO:0009165	<1e-4	slr1052	GO:0022857	<1e-4	slr0388	GO:0044464	<1e-4	slr1507	GO:0003723	0.0339
sll0630	GO:0009260	<1e-4	sll1717	GO:0044255	<1e-4	slr0554	GO:0015077	<1e-4	slr0366	GO:0044444	<1e-4	sll0905	GO:0003723	0.0339
slr6047	GO:0009144	<1e-4	sll1912	GO:0006811	<1e-4	sll2013	GO:0043492	<1e-4	ssr6003	GO:0044424	<1e-4	slr0885	GO:0003723	0.0339
sll0479	GO:0043436	<1e-4	sll1652	GO:0006220	<1e-4	ssl7021	GO:0015405	<1e-4	sll1160	GO:0044425	<1e-4	slr1780	GO:0003723	0.0339
slr7016	GO:0071842	<1e-4	sll0710	GO:0008610	<1e-4	slr1450	GO:0022836	<1e-4	slr0731	GO:0005575	<1e-4	sll1036	GO:0003723	0.0339
sll1166	GO:0044275	<1e-4	slr1168	GO:0051716	<1e-4	ssl6061	GO:0042623	<1e-4	sll1902	GO:0044464	<1e-4	sll7087	GO:0003723	0.0339
slr0981	GO:0051716	<1e-4	sll8004	GO:0046128	<1e-4	sll1526	GO:0022832	<1e-4	slr1241	GO:0044424	<1e-4	ssl6061	GO:0003723	0.0339
ssr2998	GO:0043549	<1e-4	slr0670	GO:0090304	<1e-4	sll0678	GO:0043492	<1e-4	slr1940	GO:0044425	<1e-4	slr1438	GO:0003723	0.0339
slr1571	GO:0055114	<1e-4	slr1513	GO:0031326	<1e-4	slr1266	GO:0015399	<1e-4	ssr2049	GO:0044422	<1e-4	slr0728	GO:0003723	0.0339
slr1241	GO:0009987	<1e-4	slr6015	GO:0072522	<1e-4	slr1847	GO:0022890	<1e-4	ssr6048	GO:0044446	<1e-4	sll0765	GO:0003723	0.0339
slr1866	GO:0009308	<1e-4	slr1056	GO:0050801	<1e-4	slr6006	GO:0022838	<1e-4	slr1900	GO:0044446	<1e-4	sll0327	GO:0003723	0.0339
sll0449	GO:0051818	<1e-4	sll0301	GO:0010556	<1e-4	sll0810	GO:0005342	<1e-4	slr0588	GO:0044424	<1e-4	sll7006	GO:0003723	0.0339
slr1188	GO:0046907	<1e-4	slr0168	GO:0009141	<1e-4	slr0869	GO:0042623	<1e-4	sll0328	GO:0044422	<1e-4	ssr5011	GO:0003723	0.0339
sll5032	GO:0006733	<1e-4	slr1568	GO:0034654	<1e-4	sll1634	GO:0015405	<1e-4	slr0780	GO:0044446	<1e-4	sll0157	GO:0003723	0.0339
sll6055	GO:0006576	<1e-4	slr0729	GO:0044283	<1e-4	slr0664	GO:0022836	<1e-4	slr1566	GO:0044422	<1e-4	slr5013	GO:0003723	0.0339
slr0581	GO:0009056	<1e-4	ssl1377	GO:0071496	<1e-4	slr1362	GO:0015075	<1e-4	slr1023	GO:0044444	<1e-4	slr7057	GO:0003723	0.0339
slr0362	GO:0016054	<1e-4	sll1062	GO:0044106	<1e-4	sll0069	GO:0015075	<1e-4	slr7095	GO:0044444	<1e-4	slr6044	GO:0003723	0.0339
slr0482	GO:0042455	<1e-4	sll1757	GO:0043549	<1e-4	slr0821	GO:0015077	<1e-4	slr0151	GO:0044444	<1e-4	ssr5106	GO:0003723	0.0339
slr0625	GO:0050789	<1e-4	sll1609	GO:0016054	<1e-4	sll1192	GO:0016462	<1e-4	sll1634	GO:0043226	<1e-4	slr0326	GO:0003723	0.0339
slr0514	GO:0019752	<1e-4	sll1530	GO:0006766	<1e-4	slr0482	GO:0016746	<1e-4	sll1784	GO:0044446	<1e-4	slr1396	GO:0003723	0.0339
sll7089	GO:0009892	<1e-4	sgl0001	GO:0050789	<1e-4	sll1132	GO:0015077	<1e-4	sll1934	GO:0044422	<1e-4	sll7086	GO:0003723	0.0339
slr7091	GO:0010468	<1e-4	slr1066	GO:0006811	<1e-4	ssr1473	GO:0022892	<1e-4	sll1319	GO:0043226	<1e-4	slr2071	GO:0003723	0.0339
sll0547	GO:0006631	<1e-4	sll0488	GO:0044271	<1e-4	slr0038	GO:0042623	<1e-4	slr0392	GO:0044424	<1e-4	slr0269	GO:0003723	0.0339
sll1606	GO:0072522	<1e-4	slr0909	GO:0051818	<1e-4	sll1219	GO:0043492	<1e-4	ssr5092	GO:0044425	<1e-4	ssl1378	GO:0003723	0.0339
ssr2067	GO:0044106	<1e-4	sll7066	GO:0019222	<1e-4	sll1582	GO:0022890	<1e-4	slr1813	GO:0044446	<1e-4	slr0651	GO:0003723	0.0339
slr5013	GO:0022607	<1e-4	ssr6089	GO:0009309	<1e-4	ssr6032	GO:0016462	<1e-4	sll7050	GO:0044422	<1e-4	sll1424	GO:0005515	0.0334
slr0553	GO:0050896	<1e-4	slr1209	GO:0052111	<1e-4	slr0654	GO:0043167	<1e-4	sll0268	GO:0044422	<1e-4	slr1032	GO:0005515	0.0334

ssl1792	GO:0005996	<1e-4	sll0742	GO:0055082	<1e-4	sll1225	GO:0003674	<1e-4	slr1071	GO:0005575	<1e-4	slr2084	GO:0005515	0.0334
slr1188	GO:0006720	<1e-4	ssr7017	GO:0034654	<1e-4	sll0252	GO:0022892	<1e-4	slr0453	GO:0044444	<1e-4	ssr2781	GO:0005515	0.0334
sll1373	GO:0019752	<1e-4	slr1196	GO:0071496	<1e-4	sll1455	GO:0005342	<1e-4	sll0085	GO:0044424	<1e-4	sll1775	GO:0005515	0.0334
slr6033	GO:0044282	<1e-4	sll1726	GO:0009199	<1e-4	sll1089	GO:0016462	<1e-4	slr6073	GO:0043226	<1e-4	ssl3291	GO:0005515	0.0334
slr0872	GO:0051188	<1e-4	sll0274	GO:0048518	<1e-4	sll0266	GO:0015077	<1e-4	sll1251	GO:0005575	<1e-4	sll0647	GO:0005515	0.0334
slr1276	GO:0008150	<1e-4	sll1884	GO:0051716	<1e-4	sll1834	GO:0016741	<1e-4	sll1652	GO:0044422	<1e-4	ssr2754	GO:0044237	0.0333
ssl1498	GO:0009132	<1e-4	sll1730	GO:0006766	<1e-4	slr0157	GO:0015405	<1e-4	sll1911	GO:0043226	<1e-4	sll1925	GO:0044237	0.0333
sll1304	GO:0050801	<1e-4	slr1896	GO:0009142	<1e-4	ssr6085	GO:0060089	<1e-4	slr1263	GO:0044422	<1e-4	slr0149	GO:0044237	0.0333
slr0769	GO:0016052	<1e-4	slr1472	GO:0009059	<1e-4	slr1636	GO:0022891	<1e-4	slr1999	GO:0005575	<1e-4	slr0801	GO:0044237	0.0333
sll0007	GO:0051347	<1e-4	slr1468	GO:0044283	<1e-4	slr1081	GO:0022890	<1e-4	slr0458	GO:0044425	<1e-4	sll1411	GO:0044237	0.0333
slr0569	GO:0009132	<1e-4	sll0639	GO:0034660	<1e-4	slr1944	GO:0022857	<1e-4	slr1880	GO:0044446	<1e-4	slr1862	GO:0044237	0.0333
sll0496	GO:0051716	<1e-4	sll0446	GO:0046907	<1e-4	slr0914	GO:0015291	<1e-4	slr0909	GO:0005575	<1e-4	slr1977	GO:0044237	0.0333
ssl0483	GO:0009142	<1e-4	sll1715	GO:0055082	<1e-4	sll0397	GO:0022892	<1e-4	sll5006	GO:0044422	<1e-4	slr1413	GO:0044237	0.0333
sll0281	GO:0006163	<1e-4	slr1906	GO:0008150	<1e-4	slr0380	GO:0022857	<1e-4	ssl6092	GO:0043226	<1e-4	slr1338	GO:0044237	0.0333
sll0577	GO:0005996	<1e-4	slr1127	GO:0034641	<1e-4	sll0406	GO:0022838	<1e-4	slr1338	GO:0043226	<1e-4	sll1390	GO:0044237	0.0333
slr1530	GO:0031640	<1e-4	slr1362	GO:0006732	<1e-4	slr1259	GO:0015075	<1e-4	slr6075	GO:0044444	<1e-4	sll1659	GO:0044237	0.0333
ssl0352	GO:0051188	<1e-4	slr1702	GO:0090304	<1e-4	sll0765	GO:0022803	<1e-4	ssr7093	GO:0005575	<1e-4	slr0483	GO:0044237	0.0333
sll1472	GO:0051347	<1e-4	sll1250	GO:0055086	<1e-4	sll0048	GO:0022838	<1e-4	slr6057	GO:0044464	<1e-4	slr1122	GO:0044237	0.0333
slr2118	GO:0048522	<1e-4	sll0909	GO:0010556	<1e-4	ssl2814	GO:0042623	<1e-4	sll1355	GO:0044424	<1e-4	sll0740	GO:0044237	0.0333
sll0253	GO:0009263	<1e-4	ssr3467	GO:0006631	<1e-4	slr1677	GO:0022892	<1e-4	slr1263	GO:0005575	<1e-4	sll0888	GO:0044237	0.0333
slr6091	GO:0031323	<1e-4	sll0810	GO:0009262	<1e-4	slr7024	GO:0015399	<1e-4	slr1505	GO:0044424	<1e-4	sll1350	GO:0044237	0.0333
sll0765	GO:0044255	<1e-4	sll1414	GO:0016052	<1e-4	sll0630	GO:0060089	<1e-4	sll0590	GO:0044446	<1e-4	slr1415	GO:0044237	0.0333
ssr2333	GO:0071842	<1e-4	slr1920	GO:0050794	<1e-4	ssl3142	GO:0060089	<1e-4	slr1162	GO:0005575	<1e-4	ssl3382	GO:0044237	0.0333
slr2125	GO:0071496	<1e-4	slr0345	GO:0042451	<1e-4	sll0185	GO:0015405	<1e-4	sll1880	GO:0044444	<1e-4	slr1613	GO:0044237	0.0333
sll0645	GO:0052111	<1e-4	slr1638	GO:0006082	<1e-4	sll1344	GO:0015291	<1e-4	slr2018	GO:0044444	<1e-4	slr1616	GO:0044237	0.0333
slr6039	GO:0006732	<1e-4	slr5087	GO:0019752	<1e-4	sll0875	GO:0015293	<1e-4	sll0449	GO:0044444	<1e-4	sll0911	GO:0044237	0.0333
ssl3382	GO:0051171	<1e-4	sll0811	GO:0034654	<1e-4	sll0397	GO:0022857	<1e-4	sll1873	GO:0044422	<1e-4	slr1287	GO:0044237	0.0333
sll1388	GO:0043436	<1e-4	sll1240	GO:0072524	<1e-4	sll5032	GO:0022838	<1e-4	slr7057	GO:0044422	<1e-4	sll0451	GO:0044237	0.0333
ssr3341	GO:0006811	<1e-4	sll1155	GO:0006066	<1e-4	sll1613	GO:0042623	<1e-4	slr7011	GO:0044444	<1e-4	sll0359	GO:0044237	0.0333
sll1123	GO:0043412	<1e-4	slr1819	GO:0051171	<1e-4	slr1222	GO:0015077	<1e-4	sll0614	GO:0044425	<1e-4	sll1654	GO:0044237	0.0333
sll0309	GO:0071842	<1e-4	slr1918	GO:0009260	<1e-4	slr0650	GO:0005342	<1e-4	slr0479	GO:0044444	<1e-4	slr1082	GO:0044237	0.0333
ssl0739	GO:0009059	<1e-4	ssl3291	GO:0043412	<1e-4	sll0597	GO:0015077	<1e-4	sll1424	GO:0044464	<1e-4	slr2080	GO:0044237	0.0333
sll1219	GO:0042455	<1e-4	sll8001	GO:0046483	<1e-4	sll0785	GO:0022838	<1e-4	slr1940	GO:0005575	<1e-4	sll0981	GO:0044237	0.0333
sll1611	GO:0043648	<1e-4	sll1217	GO:0051716	<1e-4	ssl0353	GO:0016818	<1e-4	slr2121	GO:0044444	<1e-4	slr0815	GO:0044237	0.0333
ssl2749	GO:0006066	<1e-4	ssl1972	GO:0043170	<1e-4	slr1195	GO:0016817	<1e-4	slr1394	GO:0044444	<1e-4	sll1267	GO:0044237	0.0333
ssr2047	GO:0072527	<1e-4	slr1570	GO:0071840	<1e-4	ssr3532	GO:0042623	<1e-4	sll1252	GO:0043226	<1e-4	slr0930	GO:0044237	0.0333
slr1464	GO:0043170	<1e-4	slr0941	GO:0016054	<1e-4	sll1130	GO:0022890	<1e-4	slr7096	GO:0044444	<1e-4	slr0468	GO:0044237	0.0333
slr0509	GO:0080090	<1e-4	ssl2971	GO:0044248	<1e-4	slr1624	GO:0022891	<1e-4	sll1158	GO:0005575	<1e-4	slr1288	GO:0044237	0.0333
sll0609	GO:0050794	<1e-4	slr0730	GO:0019222	<1e-4	slr0453	GO:0043167	<1e-4	sll1542	GO:0044446	<1e-4	ssr1528	GO:0044237	0.0333
sll1949	GO:0065007	<1e-4	sll0319	GO:0006753	<1e-4	slr0887	GO:0060089	<1e-4	slr1816	GO:0005575	<1e-4	slr0680	GO:0044237	0.0333
slr2038	GO:0046164	<1e-4	slr0103	GO:0010468	<1e-4	sll0282	GO:0043167	<1e-4	sll1123	GO:0043226	<1e-4	sll1021	GO:0044237	0.0333
slr7098	GO:0050794	<1e-4	sll1217	GO:0043170	<1e-4	slr1276	GO:0016746	<1e-4	sll1671	GO:0005575	<1e-4	slr1152	GO:0044237	0.0333
sll0436	GO:0009150	<1e-4	slr1668	GO:0048522	<1e-4	slr1932	GO:0043492	<1e-4	slr0360	GO:0044424	<1e-4	slr0887	GO:0044237	0.0333
slr1203	GO:0044275	<1e-4	sll0614	GO:0009141	<1e-4	sll0321	GO:0016746	<1e-4	slr7026	GO:0044444	<1e-4	sll1251	GO:0044237	0.0333
ssl1520	GO:0044281	<1e-4	sll5030	GO:0048522	<1e-4	slr1623	GO:0022892	<1e-4	sll1062	GO:0044446	<1e-4	sll0297	GO:0044237	0.0333
sll0060	GO:0051234	<1e-4	sll1863	GO:0006720	<1e-4	slr1998	GO:0003674	<1e-4	sll0063	GO:0044422	<1e-4	slr1544	GO:0044237	0.0333
slr0483	GO:0071554	<1e-4	slr0317	GO:0034654	<1e-4	slr0957	GO:0016462	<1e-4	sll0361	GO:0044444	<1e-4	slr1827	GO:0044237	0.0333
sll0761	GO:0071842	<1e-4	slr0376	GO:0052188	<1e-4	sll1272	GO:0015293	<1e-4	slr0053	GO:0044444	<1e-4	sll0296	GO:0008168	0.0331
slr0397	GO:0009394	<1e-4	slr0458	GO:0006811	<1e-4	ssl3573	GO:0043492	<1e-4	slr1290	GO:0044425	<1e-4	slr1441	GO:0008168	0.0331
sll0853	GO:0034654	<1e-4	slr0442	GO:0009142	<1e-4	sll0355	GO:0015399	<1e-4	ssl1577	GO:0044464	<1e-4	slr0211	GO:0008168	0.0331
sll0925	GO:0044003	<1e-4	slr0168	GO:0060255	<1e-4	sll0939	GO:0015405	<1e-4	sll0525	GO:0044422	<1e-4	ssr2848	GO:0008168	0.0331
slr0333	GO:0043648	<1e-4	sll7067	GO:0072521	<1e-4	ssl3549	GO:0015075	<1e-4	slr1450	GO:0005575	<1e-4	slr1866	GO:0008168	0.0331
sll1188	GO:0031326	<1e-4	slr1811	GO:0050801	<1e-4	sll0595	GO:0015405	<1e-4	slr0407	GO:0044464	<1e-4	sll1186	GO:0008168	0.0331
slr1071	GO:0009892	<1e-4	ssr2551	GO:0008610	<1e-4	ssr3154	GO:0022857	<1e-4	sll1186	GO:0005575	<1e-4	ssr3532	GO:0008168	0.0331

sll1163	GO:0060255	<1e-4	sll1251	GO:0042180	<1e-4	sll1660	GO:0005342	<1e-4	sll0488	GO:0005575	<1e-4	sll1433	GO:0008168	0.0331
sll1262	GO:0019222	<1e-4	sll1193	GO:0072521	<1e-4	sll1763	GO:0016462	<1e-4	sll1477	GO:0043226	<1e-4	sll0864	GO:0008168	0.0331
sll1863	GO:0009132	<1e-4	slr0941	GO:0009124	<1e-4	sll7086	GO:0022890	<1e-4	slr6073	GO:0044424	<1e-4	slr1417	GO:0008168	0.0331
slr1628	GO:0009889	<1e-4	sll1344	GO:0008150	<1e-4	sll0230	GO:0005342	<1e-4	sll0062	GO:0043226	<1e-4	sll1166	GO:0008168	0.0331
slr1079	GO:0009059	<1e-4	sll1853	GO:0071496	<1e-4	slr1438	GO:0005342	<1e-4	ssr6019	GO:0043226	<1e-4	sll0508	GO:0008168	0.0331
slr0458	GO:0009308	<1e-4	slr0765	GO:0052111	<1e-4	slr1207	GO:0043492	<1e-4	sll0781	GO:0044444	<1e-4	slr1398	GO:0008168	0.0331
sll0238	GO:0006576	<1e-4	ssr2755	GO:0046128	<1e-4	sll0615	GO:0016462	<1e-4	sll0442	GO:0044425	<1e-4	slr1222	GO:0008168	0.0331
sll1304	GO:0051171	<1e-4	sll0451	GO:0044106	<1e-4	slr7024	GO:0016746	<1e-4	sll8001	GO:0005575	<1e-4	sll0263	GO:0008168	0.0331
sll0499	GO:0019752	<1e-4	slr1362	GO:0046164	<1e-4	slr0456	GO:0015075	<1e-4	sll0981	GO:0044422	<1e-4	slr1519	GO:0008168	0.0331
sll1071	GO:0031640	<1e-4	sll0274	GO:0060255	<1e-4	sll0661	GO:0008171	<1e-4	slr5013	GO:0043226	<1e-4	slr0551	GO:0008168	0.0331
sll1446	GO:0046164	<1e-4	sll0172	GO:0046483	<1e-4	slr1152	GO:0022836	<1e-4	sll0350	GO:0044446	<1e-4	slr1083	GO:0008168	0.0331
ssl3382	GO:0051818	<1e-4	slr0610	GO:0071496	<1e-4	slr0885	GO:0060089	<1e-4	slr1429	GO:0044424	<1e-4	slr0871	GO:0008168	0.0331
sll7047	GO:0051171	<1e-4	sll0162	GO:0019219	<1e-4	sll0930	GO:0016817	<1e-4	slr1990	GO:0005575	<1e-4	sll0382	GO:0008168	0.0331
sll1995	GO:0009144	<1e-4	slr0480	GO:0019751	<1e-4	sll7078	GO:0043167	<1e-4	sll1307	GO:0005575	<1e-4	slr0503	GO:0008168	0.0331
slr1163	GO:0009199	<1e-4	slr1917	GO:0006732	<1e-4	sll0102	GO:0022832	<1e-4	ssr2553	GO:0005575	<1e-4	sll1715	GO:0008168	0.0331
sll0296	GO:0043933	<1e-4	sll0982	GO:0044260	<1e-4	slr0712	GO:0043492	<1e-4	slr0269	GO:0043226	<1e-4	ssr1698	GO:0008168	0.0331
ssl0242	GO:0043549	<1e-4	slr6091	GO:0071842	<1e-4	slr1143	GO:0016462	<1e-4	slr0305	GO:0044444	<1e-4	slr0476	GO:0008168	0.0331
slr6103	GO:0033013	<1e-4	slr7098	GO:0034654	<1e-4	ssr2803	GO:0016817	<1e-4	sll1240	GO:0005575	<1e-4	sll0447	GO:0008168	0.0331
slr0514	GO:0046394	<1e-4	sll0781	GO:0050794	<1e-4	slr1110	GO:0015267	<1e-4	sll0444	GO:0005575	<1e-4	slr1353	GO:0008168	0.0331
ssl5007	GO:0044106	<1e-4	ssl0352	GO:0009141	<1e-4	ssr6099	GO:0016817	<1e-4	sll8032	GO:0044464	<1e-4	slr1659	GO:0008168	0.0331
sll0931	GO:0072521	<1e-4	slr1920	GO:0043412	<1e-4	slr5073	GO:0015077	<1e-4	slr6014	GO:0044464	<1e-4	sll0547	GO:0008168	0.0331
sll0756	GO:0006066	<1e-4	ssl8008	GO:0009991	<1e-4	sll1882	GO:0016746	<1e-4	slr0383	GO:0044444	<1e-4	slr0589	GO:0016740	0.0329
sll0925	GO:0071842	<1e-4	slr6016	GO:0050896	<1e-4	slr6101	GO:0005342	<1e-4	ssl7039	GO:0044424	<1e-4	ssr1391	GO:0016740	0.0329
slr0211	GO:0008150	<1e-4	slr1259	GO:0051171	<1e-4	slr1406	GO:0016818	<1e-4	slr0103	GO:0005575	<1e-4	sll0788	GO:0016740	0.0329
sll7063	GO:0034641	<1e-4	slr1885	GO:0009123	<1e-4	ssr6002	GO:0003674	<1e-4	slr0208	GO:0044425	<1e-4	sll0361	GO:0016740	0.0329
sll0804	GO:0051347	<1e-4	sll0860	GO:0009308	<1e-4	slr1419	GO:0043492	<1e-4	slr1442	GO:0044464	<1e-4	ssl0353	GO:0016740	0.0329
slr0006	GO:0019752	<1e-4	sll0670	GO:0019222	<1e-4	sll0048	GO:0016817	<1e-4	sll1500	GO:0044446	<1e-4	slr0607	GO:0016740	0.0329
sll1586	GO:0022607	<1e-4	sll0703	GO:0019219	<1e-4	ssr0332	GO:0060089	<1e-4	slr0355	GO:0005575	<1e-4	sll0577	GO:0016740	0.0329
slr1142	GO:0043412	<1e-4	ssr1765	GO:0050896	<1e-4	sll1464	GO:0016462	<1e-4	slr0784	GO:0005575	<1e-4	ssl2996	GO:0016740	0.0329
slr0848	GO:0016052	<1e-4	ssl0788	GO:0006091	<1e-4	sll0781	GO:0022838	<1e-4	slr0789	GO:0044444	<1e-4	slr1143	GO:0016740	0.0329
slr6007	GO:0033013	<1e-4	slr0609	GO:0009124	<1e-4	slr1095	GO:0015267	<1e-4	slr2000	GO:0005575	<1e-4	slr0923	GO:0016740	0.0329
ssl5103	GO:0044282	<1e-4	slr7073	GO:0042451	<1e-4	sll1340	GO:0043492	<1e-4	slr0380	GO:0005575	<1e-4	slr0607	GO:0005975	0.0326
sll0280	GO:0051716	<1e-4	slr1195	GO:0019219	<1e-4	ssr6083	GO:0016818	<1e-4	ssr2754	GO:0044464	<1e-4	slr1847	GO:0005975	0.0326
slr0092	GO:0046483	<1e-4	sll0497	GO:0044003	<1e-4	sll5030	GO:0015291	<1e-4	slr1865	GO:0005575	<1e-4	ssr1391	GO:0005975	0.0326
ssl2996	GO:0034654	<1e-4	slr1541	GO:0006066	<1e-4	sll0602	GO:0015293	<1e-4	slr7013	GO:0043226	<1e-4	sll1611	GO:0005975	0.0326
slr0148	GO:0051188	<1e-4	slr1066	GO:0034660	<1e-4	ssl7048	GO:0015405	<1e-4	ssr2611	GO:0044464	<1e-4	slr0740	GO:0005975	0.0326
sll1355	GO:0071496	<1e-4	ssr6048	GO:0009892	<1e-4	sll0327	GO:0022832	<1e-4	ssl1464	GO:0044464	<1e-4	slr1811	GO:0005975	0.0326
slr0304	GO:0009262	<1e-4	slr0702	GO:0048519	<1e-4	slr1789	GO:0015267	<1e-4	ssr3410	GO:0005575	<1e-4	slr0479	GO:0005975	0.0326
ssr8013	GO:0031323	<1e-4	slr0393	GO:0034654	<1e-4	slr2121	GO:0015291	<1e-4	slr0144	GO:0044425	<1e-4	sll0910	GO:0005975	0.0326
ssl0832	GO:0051246	<1e-4	slr0935	GO:0050801	<1e-4	ssl0312	GO:0015291	<1e-4	slr1866	GO:0044444	<1e-4	sll0505	GO:0005975	0.0326
slr1566	GO:0019222	<1e-4	slr1117	GO:0051186	<1e-4	sll0414	GO:0015405	<1e-4	slr1815	GO:0044444	<1e-4	slr1926	GO:0005975	0.0326
ssr2975	GO:0019637	<1e-4	slr0270	GO:0006753	<1e-4	slr1958	GO:0003674	<1e-4	slr2018	GO:0044424	<1e-4	slr1865	GO:0005975	0.0326
slr7059	GO:0006721	<1e-4	slr1260	GO:0006066	<1e-4	sll0843	GO:0016818	<1e-4	ssr1473	GO:0044424	<1e-4	sll0175	GO:0005975	0.0326
slr0667	GO:0016054	<1e-4	sll8002	GO:0044248	<1e-4	sll1273	GO:0015077	<1e-4	slr0271	GO:0044425	<1e-4	slr0978	GO:0005975	0.0326
ssl1377	GO:0048523	<1e-4	sll1396	GO:0009893	<1e-4	slr0270	GO:0043492	<1e-4	sll1464	GO:0005575	<1e-4	sll0737	GO:0016772	0.0325
ssl2814	GO:0071496	<1e-4	sll1130	GO:0072524	<1e-4	slr0601	GO:0022892	<1e-4	ssl3383	GO:0044446	<1e-4	slr1940	GO:0016772	0.0325
slr1177	GO:0048519	<1e-4	sll1757	GO:0042180	<1e-4	slr8021	GO:0005342	<1e-4	slr0192	GO:0044464	<1e-4	sll0297	GO:0016772	0.0325
sll0481	GO:0044275	<1e-4	slr0816	GO:0044283	<1e-4	slr1907	GO:0043167	<1e-4	ssr2615	GO:0044425	<1e-4	slr0318	GO:0016772	0.0325
slr0386	GO:0006732	<1e-4	sll5046	GO:0006721	<1e-4	sll1400	GO:0005342	<1e-4	ssr6002	GO:0044424	<1e-4	sll0268	GO:0016772	0.0325
slr1918	GO:0080090	<1e-4	ssl2384	GO:0006163	<1e-4	sll0444	GO:0015405	<1e-4	sll1340	GO:0044464	<1e-4	sll1040	GO:0016772	0.0325
sll7055	GO:0009394	<1e-4	slr0273	GO:0071840	<1e-4	slr1613	GO:0022836	<1e-4	sll0847	GO:0044425	<1e-4	slr1613	GO:0016772	0.0325
slr1462	GO:0019222	<1e-4	sll0508	GO:0022607	<1e-4	sll1250	GO:0022892	<1e-4	sll1447	GO:0044446	<1e-4	slr0935	GO:0016772	0.0325
sll0499	GO:0009161	<1e-4	sll0023	GO:0043648	<1e-4	ssl1300	GO:0043492	<1e-4	sll1485	GO:0005575	<1e-4	sll0997	GO:0016772	0.0325
slr0491	GO:0034641	<1e-4	sll0286	GO:0009057	<1e-4	sll0022	GO:0003674	<1e-4	sll1285	GO:0044424	<1e-4	slr0924	GO:0016772	0.0325

sll0656	GO:0019362	<1e-4	slr0476	GO:0001932	<1e-4	sll0864	GO:0015075	<1e-4	slr2010	GO:0043226	<1e-4	slr0729	GO:0016772	0.0325
sll1531	GO:0055082	<1e-4	slr1628	GO:0031326	<1e-4	sll0301	GO:0015075	<1e-4	sll0543	GO:0005575	<1e-4	sll0585	GO:0016772	0.0325
sll5097	GO:0019222	<1e-4	slr6009	GO:0046394	<1e-4	slr7015	GO:0015405	<1e-4	slr1611	GO:0044446	<1e-4	slr0358	GO:0016772	0.0325
slr6029	GO:0009892	<1e-4	slr0092	GO:0048518	<1e-4	ssl2138	GO:0015267	<1e-4	sll1400	GO:0043226	<1e-4	slr0060	GO:0016772	0.0325
slr7015	GO:0009394	<1e-4	sll0815	GO:0031326	<1e-4	slr1970	GO:0022803	<1e-4	slr0262	GO:0044424	<1e-4	sll1582	GO:0016772	0.0325
ssr0336	GO:0072521	<1e-4	sll1640	GO:0051246	<1e-4	sll0853	GO:0022803	<1e-4	sll0071	GO:0005575	<1e-4	slr0962	GO:0016772	0.0325
ssr7093	GO:0009394	<1e-4	slr1162	GO:0065007	<1e-4	sll1866	GO:0015399	<1e-4	slr1101	GO:0044425	<1e-4	sll1738	GO:0016772	0.0325
slr1070	GO:0050896	<1e-4	slr0606	GO:0009893	<1e-4	slr1647	GO:0015075	<1e-4	sll1956	GO:0005575	<1e-4	ssl3382	GO:0016772	0.0325
slr1082	GO:0009991	<1e-4	sll1570	GO:0043933	<1e-4	slr0957	GO:0022803	<1e-4	slr2118	GO:0044446	<1e-4	slr1541	GO:0016772	0.0325
sll1072	GO:0052111	<1e-4	ssr0336	GO:0050896	<1e-4	sll1063	GO:0015075	<1e-4	sll1915	GO:0044446	<1e-4	sll1399	GO:0016772	0.0325
slr1951	GO:0009124	<1e-4	slr0634	GO:0009132	<1e-4	sll0532	GO:0022857	<1e-4	ssr2009	GO:0043226	<1e-4	ssl0739	GO:0016772	0.0325
ssr3532	GO:0009892	<1e-4	slr6038	GO:0019751	<1e-4	sll0785	GO:0015291	<1e-4	slr1062	GO:0044425	<1e-4	slr1128	GO:0016772	0.0325
slr1571	GO:0033013	<1e-4	slr0146	GO:0009987	<1e-4	slr1168	GO:0022838	<1e-4	slr0269	GO:0044424	<1e-4	sll0981	GO:0016772	0.0325
slr6008	GO:0006163	<1e-4	slr1811	GO:0050789	<1e-4	ssr1155	GO:0022803	<1e-4	ssl0242	GO:0005575	<1e-4	slr0989	GO:0016772	0.0325
slr0590	GO:0033013	<1e-4	slr0168	GO:0044281	<1e-4	slr0270	GO:0016818	<1e-4	ssl3573	GO:0005575	<1e-4	slr1690	GO:0016772	0.0325
slr0337	GO:0006066	<1e-4	ssr0692	GO:0080090	<1e-4	slr1363	GO:0015293	<1e-4	slr1468	GO:0044422	<1e-4	sll0781	GO:0016772	0.0325
sll1751	GO:0009889	<1e-4	slr1235	GO:0034654	<1e-4	slr7011	GO:0022891	<1e-4	slr1474	GO:0005575	<1e-4	slr1288	GO:0016772	0.0325
slr2117	GO:0051347	<1e-4	sll0024	GO:0006721	<1e-4	slr1863	GO:0016741	<1e-4	slr0801	GO:0043226	<1e-4	sll1173	GO:0016772	0.0325
slr6014	GO:0055082	<1e-4	slr6008	GO:0044275	<1e-4	slr2117	GO:0015267	<1e-4	sll1025	GO:0005575	<1e-4	sll1240	GO:0016772	0.0325
slr1537	GO:0009260	<1e-4	sll0595	GO:0042455	<1e-4	slr1066	GO:0022832	<1e-4	slr1999	GO:0044446	<1e-4	sll0588	GO:0016772	0.0325
ssl2814	GO:0006733	<1e-4	sll0732	GO:0019219	<1e-4	slr1847	GO:0015399	<1e-4	slr0609	GO:0044444	<1e-4	slr0149	GO:0016772	0.0325
sll0864	GO:0001932	<1e-4	sll1526	GO:0048518	<1e-4	slr2125	GO:0060089	<1e-4	sll0428	GO:0044444	<1e-4	slr0112	GO:0016772	0.0325
ssl8008	GO:0006766	<1e-4	sll1304	GO:0009309	<1e-4	sll7006	GO:0043492	<1e-4	sll0752	GO:0043226	<1e-4	slr0975	GO:0016772	0.0325
ssl5025	GO:0006220	<1e-4	ssr0336	GO:0006811	<1e-4	slr1809	GO:0042623	<1e-4	sll1715	GO:0044444	<1e-4	sll7065	GO:0016772	0.0325
ssr2754	GO:0008150	<1e-4	sll1160	GO:0042180	<1e-4	slr7025	GO:0015267	<1e-4	ssr2047	GO:0044464	<1e-4	slr0650	GO:0016772	0.0325
slr1188	GO:0009394	<1e-4	sll1542	GO:0016052	<1e-4	slr7097	GO:0016462	<1e-4	slr1612	GO:0005575	<1e-4	slr0483	GO:0016772	0.0325
sll1355	GO:0009259	<1e-4	sll0503	GO:0044281	<1e-4	slr1572	GO:0015405	<1e-4	sll1691	GO:0043226	<1e-4	slr1862	GO:0016772	0.0325
slr0590	GO:0006066	<1e-4	slr1178	GO:0044283	<1e-4	ssl2814	GO:0060089	<1e-4	ssl5096	GO:0044446	<1e-4	sml0011	GO:0016772	0.0325
sll0503	GO:0051171	<1e-4	slr0082	GO:0072522	<1e-4	slr1162	GO:0015405	<1e-4	slr0579	GO:0044425	<1e-4	slr0373	GO:0016772	0.0325
slr6012	GO:0009142	<1e-4	ssr6099	GO:0009059	<1e-4	slr1470	GO:0008171	<1e-4	sll0267	GO:0044446	<1e-4	sll0281	GO:0016772	0.0325
sll1882	GO:0044106	<1e-4	sll0354	GO:0052111	<1e-4	slr0554	GO:0043492	<1e-4	slr2073	GO:0044425	<1e-4	slr0605	GO:0016772	0.0325
slr0888	GO:0044260	<1e-4	slr6064	GO:0045184	<1e-4	sll0268	GO:0015293	<1e-4	slr6039	GO:0005575	<1e-4	sll0564	GO:0016772	0.0325
ssr6030	GO:0043170	<1e-4	slr1660	GO:0009199	<1e-4	sll1940	GO:0015267	<1e-4	slr6104	GO:0044422	<1e-4	sll1956	GO:0016772	0.0325
sgl0001	GO:0009308	<1e-4	ssl8003	GO:0071496	<1e-4	sll7087	GO:0015293	<1e-4	ssl2996	GO:0044422	<1e-4	slr1827	GO:0016772	0.0325
slr1150	GO:0051246	<1e-4	slr1964	GO:0006721	<1e-4	slr0049	GO:0015399	<1e-4	slr6028	GO:0044425	<1e-4	sll1390	GO:0016772	0.0325
ssr6083	GO:0034660	<1e-4	sll0394	GO:0009394	<1e-4	sll1063	GO:0015405	<1e-4	slr1464	GO:0044424	<1e-4	sll1071	GO:0016772	0.0325
slr0458	GO:0009165	<1e-4	sll1306	GO:0055114	<1e-4	slr0196	GO:0042623	<1e-4	slr0581	GO:0044425	<1e-4	slr0455	GO:0016772	0.0325
slr1816	GO:0072524	<1e-4	sll0023	GO:0009991	<1e-4	sll5006	GO:0015293	<1e-4	sll5046	GO:0044422	<1e-4	sll0911	GO:0016772	0.0325
sll0498	GO:0008150	<1e-4	sll0761	GO:0009124	<1e-4	slr6013	GO:0003674	<1e-4	sll0024	GO:0044444	<1e-4	slr0169	GO:0016772	0.0325
slr5024	GO:0080090	<1e-4	slr0890	GO:0009117	<1e-4	sll0864	GO:0022891	<1e-4	ssl7048	GO:0005575	<1e-4	sll0572	GO:0016772	0.0325
sll0024	GO:0043933	<1e-4	ssl2064	GO:0044248	<1e-4	sll0478	GO:0008171	<1e-4	sll0639	GO:0044464	<1e-4	slr2005	GO:0016772	0.0325
ssr5106	GO:0009987	<1e-4	slr1032	GO:0071554	<1e-4	sll0350	GO:0015293	<1e-4	sll0524	GO:0043226	<1e-4	sll0149	GO:0016787	0.0316
sll1381	GO:0051186	<1e-4	slr6015	GO:0006732	<1e-4	sll0785	GO:0022891	<1e-4	sll0242	GO:0043234	<1e-4	ssl0750	GO:0004871	0.0311
slr2092	GO:0071496	<1e-4	slr1774	GO:0031323	<1e-4	sll0243	GO:0003674	<1e-4	slr1053	GO:0044422	<1e-4	sll0350	GO:0004871	0.0311
slr7071	GO:0042180	<1e-4	slr1053	GO:0009309	<1e-4	ssl8001	GO:0043167	<1e-4	slr1070	GO:0044424	<1e-4	slr1614	GO:0004871	0.0311
slr0980	GO:0009309	<1e-4	sll5069	GO:0009309	<1e-4	sll1702	GO:0015405	<1e-4	slr7071	GO:0005575	<1e-4	sll1424	GO:0016740	0.0308
slr7024	GO:0034641	<1e-4	sll1106	GO:0010468	<1e-4	slr1152	GO:0016817	<1e-4	sll0282	GO:0005575	<1e-4	slr2018	GO:0016740	0.0308
slr7073	GO:0009199	<1e-4	sll1446	GO:0006732	<1e-4	slr1940	GO:0022890	<1e-4	sll1891	GO:0044464	<1e-4	ssr1258	GO:0016740	0.0308
slr7013	GO:0006631	<1e-4	sll1232	GO:0009991	<1e-4	slr0981	GO:0016746	<1e-4	sll1830	GO:0005575	<1e-4	slr0408	GO:0016740	0.0308
slr1815	GO:0046164	<1e-4	sll1956	GO:0009308	<1e-4	slr6005	GO:0015075	<1e-4	slr1343	GO:0044422	<1e-4	slr0980	GO:0016740	0.0308
sll7034	GO:0006793	<1e-4	slr0482	GO:0009263	<1e-4	sll0843	GO:0015075	<1e-4	slr0038	GO:0044422	<1e-4	sll0702	GO:0016740	0.0308
sll1630	GO:0006811	<1e-4	sll1464	GO:0009132	<1e-4	slr1570	GO:0016746	<1e-4	slr7099	GO:0005575	<1e-4	sll1119	GO:0016740	0.0308
slr0634	GO:0022411	<1e-4	slr1419	GO:0033013	<1e-4	slr6104	GO:0043492	<1e-4	slr0594	GO:0043226	<1e-4	slr1110	GO:0016740	0.0308
sll1273	GO:0009124	<1e-4	ssl5027	GO:0016053	<1e-4	slr1383	GO:0016746	<1e-4	slr0722	GO:0005575	<1e-4	sll0763	GO:0016740	0.0308

slr0489	GO:0051347	<1e-4	ssr1114	GO:0048522	<1e-4	sll0678	GO:0022892	<1e-4	slr0211	GO:0044422	<1e-4	slr1413	GO:0016820	0.0306
slr0092	GO:0048523	<1e-4	sll0787	GO:0071554	<1e-4	ssl0788	GO:0016818	<1e-4	slr1911	GO:0044422	<1e-4	sll1752	GO:0016820	0.0306
slr0482	GO:0080090	<1e-4	sll0563	GO:0048519	<1e-4	slr0317	GO:0022890	<1e-4	slr0770	GO:0044425	<1e-4	sll7065	GO:0016820	0.0306
slr8044	GO:0072522	<1e-4	ssr2611	GO:0071554	<1e-4	sll0253	GO:0016818	<1e-4	slr0888	GO:0043226	<1e-4	ssr1528	GO:0016820	0.0306
slr0890	GO:0019751	<1e-4	slr0458	GO:0010468	<1e-4	slr0287	GO:0003674	<1e-4	sll0192	GO:0044422	<1e-4	slr0742	GO:0016820	0.0306
slr1023	GO:0009117	<1e-4	ssl2148	GO:0072524	<1e-4	sll0405	GO:0016462	<1e-4	sll1906	GO:0044425	<1e-4	sll0980	GO:0016820	0.0306
sll0198	GO:0019222	<1e-4	sll0898	GO:0009165	<1e-4	slr6033	GO:0003674	<1e-4	sll1714	GO:0044444	<1e-4	sll0787	GO:0016820	0.0306
slr0305	GO:0034641	<1e-4	slr1081	GO:0050789	<1e-4	slr1613	GO:0016746	<1e-4	sll0744	GO:0044422	<1e-4	slr1636	GO:0003723	0.0306
sll1472	GO:0009260	<1e-4	sll1464	GO:0019219	<1e-4	ssl5065	GO:0016817	<1e-4	sll5063	GO:0043226	<1e-4	sll0888	GO:0016820	0.0306
sll0585	GO:0006721	<1e-4	slr1034	GO:0031640	<1e-4	sll1155	GO:0043167	<1e-4	sll0499	GO:0044424	<1e-4	slr7024	GO:0016820	0.0306
ssr3571	GO:0009142	<1e-4	slr1800	GO:0046128	<1e-4	slr0789	GO:0015267	<1e-4	slr1209	GO:0044464	<1e-4	slr1052	GO:0003723	0.0306
slr1449	GO:0009150	<1e-4	slr2118	GO:0044260	<1e-4	slr0172	GO:0022803	<1e-4	slr0978	GO:0044422	<1e-4	sll0595	GO:0016820	0.0306
sll0031	GO:0048522	<1e-4	sll5089	GO:0046164	<1e-4	slr0821	GO:0015293	<1e-4	sll1306	GO:0044444	<1e-4	slr1303	GO:0016820	0.0306
slr7102	GO:0009394	<1e-4	sll0253	GO:0016052	<1e-4	slr5077	GO:0015399	<1e-4	sll8012	GO:0005575	<1e-4	sll0781	GO:0016820	0.0306
sll1613	GO:0009161	<1e-4	slr1577	GO:0006091	<1e-4	ssr0102	GO:0043167	<1e-4	slr0740	GO:0044444	<1e-4	slr1862	GO:0016820	0.0306
slr1563	GO:0044283	<1e-4	sll0513	GO:0080090	<1e-4	slr1436	GO:0015291	<1e-4	slr0645	GO:0043226	<1e-4	sll1251	GO:0016820	0.0306
slr6063	GO:0009893	<1e-4	slr1413	GO:0048523	<1e-4	slr0111	GO:0015291	<1e-4	sll0735	GO:0044424	<1e-4	sll0572	GO:0016820	0.0306
slr1209	GO:0072522	<1e-4	sll1372	GO:0019219	<1e-4	sll1071	GO:0005342	<1e-4	sll1757	GO:0044425	<1e-4	sll1170	GO:0016820	0.0306
sll1254	GO:0009165	<1e-4	sll0994	GO:0051186	<1e-4	sll7062	GO:0015399	<1e-4	sll0867	GO:0044444	<1e-4	slr1658	GO:0016820	0.0306
ssr6089	GO:0051716	<1e-4	sll1608	GO:0031326	<1e-4	ssr1528	GO:0015293	<1e-4	sll1193	GO:0005575	<1e-4	sll1654	GO:0016820	0.0306
slr6100	GO:0072527	<1e-4	sll0473	GO:0034654	<1e-4	slr1394	GO:0022891	<1e-4	slr1116	GO:0044446	<1e-4	sll0281	GO:0016820	0.0306
slr1215	GO:0042180	<1e-4	slr7012	GO:0009150	<1e-4	slr7023	GO:0022832	<1e-4	sll0293	GO:0005575	<1e-4	sll0156	GO:0016820	0.0306
sll0872	GO:0009057	<1e-4	sll1882	GO:0009123	<1e-4	sll0413	GO:0016817	<1e-4	slr0606	GO:0005575	<1e-4	sll0449	GO:0016820	0.0306
ssr1528	GO:0044248	<1e-4	slr1066	GO:0019219	<1e-4	slr0459	GO:0060089	<1e-4	sll1586	GO:0044464	<1e-4	slr1062	GO:0016820	0.0306
slr6049	GO:0044283	<1e-4	slr6100	GO:0010556	<1e-4	sll1660	GO:0022857	<1e-4	slr7015	GO:0044446	<1e-4	slr0149	GO:0016820	0.0306
slr1287	GO:0044255	<1e-4	slr1774	GO:0019752	<1e-4	slr6013	GO:0022838	<1e-4	slr0110	GO:0044446	<1e-4	slr0729	GO:0016820	0.0306
slr0863	GO:0050794	<1e-4	sll1472	GO:0051188	<1e-4	slr1674	GO:0043167	<1e-4	sll1751	GO:0044424	<1e-4	slr1383	GO:0016820	0.0306
sll0243	GO:0034641	<1e-4	slr1767	GO:0009161	<1e-4	slr1699	GO:0016818	<1e-4	ssr5092	GO:0043226	<1e-4	sll0837	GO:0016820	0.0306
sll1866	GO:0006793	<1e-4	slr0364	GO:0072522	<1e-4	slr6045	GO:0008171	<1e-4	slr0388	GO:0005575	<1e-4	slr1033	GO:0016820	0.0306
slr1122	GO:0009262	<1e-4	slr0111	GO:0051179	<1e-4	sll1906	GO:0015291	<1e-4	sll0335	GO:0005575	<1e-4	sll1054	GO:0016820	0.0306
sll1784	GO:0009987	<1e-4	slr0431	GO:0009124	<1e-4	sll1389	GO:0022890	<1e-4	slr1811	GO:0044446	<1e-4	sll1239	GO:0016820	0.0306
slr1990	GO:0009394	<1e-4	slr1999	GO:0048523	<1e-4	slr2003	GO:0015405	<1e-4	sll1503	GO:0005575	<1e-4	slr0468	GO:0016820	0.0306
slr0208	GO:0031326	<1e-4	sll1541	GO:0048519	<1e-4	sll0752	GO:0003674	<1e-4	slr0521	GO:0044464	<1e-4	sll1267	GO:0016820	0.0306
slr0731	GO:0048523	<1e-4	sll0670	GO:0065007	<1e-4	sll1834	GO:0015291	<1e-4	ssr7079	GO:0043226	<1e-4	sll0737	GO:0016820	0.0306
sll1006	GO:0006066	<1e-4	slr1913	GO:0055086	<1e-4	sll1455	GO:0022836	<1e-4	slr1658	GO:0005575	<1e-4	slr1940	GO:0016820	0.0306
slr5102	GO:0050794	<1e-4	slr0709	GO:0009161	<1e-4	slr0668	GO:0022803	<1e-4	slr1069	GO:0044444	<1e-4	sll0685	GO:0016820	0.0306
sll1132	GO:0009144	<1e-4	slr1025	GO:0006082	<1e-4	sll0749	GO:0022803	<1e-4	slr1778	GO:0044424	<1e-4	slr0654	GO:0016820	0.0306
ssl1690	GO:0019637	<1e-4	slr1303	GO:0006766	<1e-4	sll1954	GO:0015291	<1e-4	slr1591	GO:0044424	<1e-4	slr0373	GO:0016820	0.0306
sll1656	GO:0046483	<1e-4	ssr2317	GO:0044282	<1e-4	sll1863	GO:0016817	<1e-4	sll5069	GO:0005575	<1e-4	slr1668	GO:0016820	0.0306
slr1023	GO:0009259	<1e-4	ssl5025	GO:0009132	<1e-4	sll6052	GO:0022857	<1e-4	sll0659	GO:0005575	<1e-4	sll1738	GO:0016820	0.0306
slr0192	GO:0019752	<1e-4	sll1174	GO:0019752	<1e-4	sll1956	GO:0015077	<1e-4	sll0048	GO:0044425	<1e-4	sll0359	GO:0016820	0.0306
sll1749	GO:0034641	<1e-4	sll0543	GO:0009991	<1e-4	ssl7038	GO:0022857	<1e-4	sll1949	GO:0005575	<1e-4	sll1241	GO:0016820	0.0306
sll0216	GO:0009123	<1e-4	sll0263	GO:0044248	<1e-4	slr6016	GO:0015075	<1e-4	sll1170	GO:0044444	<1e-4	slr0060	GO:0016820	0.0306
slr5111	GO:0006753	<1e-4	sll0602	GO:0048518	<1e-4	sll0293	GO:0022891	<1e-4	sll0400	GO:0044424	<1e-4	slr1082	GO:0016820	0.0306
sll5026	GO:0019752	<1e-4	ssl1378	GO:0022607	<1e-4	sll0442	GO:0022836	<1e-4	ssr6032	GO:0044444	<1e-4	slr2048	GO:0016820	0.0306
ssr5019	GO:0050896	<1e-4	slr1163	GO:0009124	<1e-4	sll1062	GO:0022836	<1e-4	slr1050	GO:0044424	<1e-4	slr1493	GO:0016820	0.0306
slr5023	GO:0009394	<1e-4	slr5037	GO:0009141	<1e-4	slr6073	GO:0022832	<1e-4	sll1961	GO:0044446	<1e-4	slr1762	GO:0016820	0.0306
slr1935	GO:0031326	<1e-4	slr6014	GO:0009144	<1e-4	sll1306	GO:0016818	<1e-4	slr0728	GO:0044422	<1e-4	sll1225	GO:0016820	0.0306
slr1215	GO:0022411	<1e-4	slr0935	GO:0019752	<1e-4	slr0145	GO:0005342	<1e-4	ssl5015	GO:0005575	<1e-4	slr0962	GO:0016820	0.0306
slr1045	GO:0010556	<1e-4	slr0431	GO:0009199	<1e-4	sll5033	GO:0022832	<1e-4	sll0369	GO:0044424	<1e-4	slr0358	GO:0016820	0.0306
ssl2781	GO:0009987	<1e-4	slr0172	GO:0009126	<1e-4	sll0857	GO:0042623	<1e-4	sll1736	GO:0044424	<1e-4	slr0249	GO:0016820	0.0306
sll1022	GO:0071841	<1e-4	slr0408	GO:0009987	<1e-4	slr1851	GO:0016817	<1e-4	sll0742	GO:0044446	<1e-4	slr2005	GO:0016820	0.0306
slr0157	GO:0009126	<1e-4	slr1206	GO:0044255	<1e-4	ssl5113	GO:0015405	<1e-4	sll1254	GO:0005575	<1e-4	slr0232	GO:0016820	0.0306
slr1505	GO:0005996	<1e-4	sll0314	GO:0016052	<1e-4	sll0162	GO:0015077	<1e-4	sll0069	GO:0044444	<1e-4	slr0554	GO:0016820	0.0306

slr0468	GO:0006091	<1e-4	slr1752	GO:0072524	<1e-4	sll0498	GO:0015405	<1e-4	slr0642	GO:0044422	<1e-4	sll0585	GO:0016820	0.0306
slr0554	GO:0009059	<1e-4	slr0184	GO:0072522	<1e-4	slr5127	GO:0005342	<1e-4	ssl0461	GO:0044424	<1e-4	sll0911	GO:0016820	0.0306
slr1266	GO:0009124	<1e-4	sll0188	GO:0052188	<1e-4	sll0253	GO:0015293	<1e-4	slr1906	GO:0005575	<1e-4	slr0510	GO:0016820	0.0306
sll0608	GO:0065007	<1e-4	ssr0692	GO:0051179	<1e-4	sll0168	GO:0022891	<1e-4	slr1541	GO:0044424	<1e-4	sll0505	GO:0008168	0.0297
slr0601	GO:0008150	<1e-4	slr0172	GO:0009260	<1e-4	slr0264	GO:0015075	<1e-4	slr1278	GO:0043226	<1e-4	ssr1391	GO:0008168	0.0297
slr0049	GO:0034654	<1e-4	sll1691	GO:0033013	<1e-4	slr1203	GO:0005342	<1e-4	sll1735	GO:0044444	<1e-4	sll0192	GO:0008168	0.0297
slr0172	GO:0031326	<1e-4	slr0587	GO:0006163	<1e-4	sll1426	GO:0015405	<1e-4	sll0860	GO:0043226	<1e-4	slr1800	GO:0008168	0.0297
slr2105	GO:0043648	<1e-4	slr1533	GO:0043933	<1e-4	slr1624	GO:0022838	<1e-4	sll1164	GO:0044446	<1e-4	sll1751	GO:0008168	0.0297
slr1566	GO:0048518	<1e-4	ssl5129	GO:0048522	<1e-4	slr1056	GO:0003674	<1e-4	slr1053	GO:0044446	<1e-4	slr1307	GO:0008168	0.0297
ssl8008	GO:0044271	<1e-4	ssr2962	GO:0044003	<1e-4	ssr1768	GO:0015293	<1e-4	sll0443	GO:0044425	<1e-4	sll0361	GO:0008168	0.0297
sll5132	GO:0052188	<1e-4	sll1722	GO:0051246	<1e-4	slr1812	GO:0022832	<1e-4	sll0297	GO:0005575	<1e-4	slr0607	GO:0008168	0.0297
slr6007	GO:0043170	<1e-4	slr1885	GO:0051716	<1e-4	slr0398	GO:0022832	<1e-4	sll1611	GO:0043226	<1e-4	slr0496	GO:0008168	0.0297
slr7082	GO:0046164	<1e-4	ssl0832	GO:0060255	<1e-4	sll7028	GO:0008171	<1e-4	slr0667	GO:0005575	<1e-4	slr0978	GO:0008168	0.0297
ssr2439	GO:0072527	<1e-4	slr0913	GO:0071841	<1e-4	slr0978	GO:0005342	<1e-4	ssr2422	GO:0043226	<1e-4	slr1726	GO:0008168	0.0297
slr1068	GO:0044248	<1e-4	ssr3409	GO:0008610	<1e-4	slr5127	GO:0022832	<1e-4	slr1603	GO:0044464	<1e-4	sll1562	GO:0008168	0.0297
slr1173	GO:0022607	<1e-4	sll0174	GO:0031326	<1e-4	slr0269	GO:0015291	<1e-4	sll0424	GO:0044424	<1e-4	slr1098	GO:0008168	0.0297
slr2025	GO:0050794	<1e-4	sll0585	GO:0051716	<1e-4	slr1045	GO:0015293	<1e-4	slr0489	GO:0044444	<1e-4	slr0514	GO:0008168	0.0297
slr0642	GO:0009987	<1e-4	sll0804	GO:0009117	<1e-4	sll1262	GO:0015077	<1e-4	slr1951	GO:0044422	<1e-4	sll0783	GO:0008168	0.0297
sll1942	GO:0019222	<1e-4	slr0112	GO:0008150	<1e-4	sll1769	GO:0016741	<1e-4	slr1627	GO:0044422	<1e-4	sll1526	GO:0008168	0.0297
ssr3402	GO:0034660	<1e-4	sll1089	GO:0043549	<1e-4	slr0049	GO:0043492	<1e-4	slr1187	GO:0044444	<1e-4	slr0909	GO:0008168	0.0297
slr1114	GO:0006631	<1e-4	sll1306	GO:0055082	<1e-4	slr0957	GO:0003674	<1e-4	ssl5108	GO:0005575	<1e-4	sll1573	GO:0004871	0.0295
sll0910	GO:0006082	<1e-4	slr1047	GO:0051171	<1e-4	ssr0102	GO:0022838	<1e-4	slr1468	GO:0044446	<1e-4	slr0106	GO:0004871	0.0295
slr1920	GO:0001932	<1e-4	slr0601	GO:0046128	<1e-4	slr1261	GO:0022838	<1e-4	slr1557	GO:0005575	<1e-4	ssr6099	GO:0004871	0.0295
sll0854	GO:0016052	<1e-4	sll1192	GO:0009259	<1e-4	ssr2998	GO:0015267	<1e-4	ssl2807	GO:0044464	<1e-4	slr1142	GO:0004871	0.0295
slr2073	GO:0009126	<1e-4	slr8021	GO:0052188	<1e-4	ssr1407	GO:0022890	<1e-4	sll0802	GO:0044446	<1e-4	slr0498	GO:0004871	0.0295
sll0175	GO:0044260	<1e-4	slr0269	GO:0071496	<1e-4	slr0217	GO:0022891	<1e-4	sll1752	GO:0044446	<1e-4	sll1882	GO:0004871	0.0295
slr0924	GO:0006733	<1e-4	sll1654	GO:0009263	<1e-4	ssr1258	GO:0015075	<1e-4	slr1406	GO:0043226	<1e-4	slr6016	GO:0004871	0.0295
sll7033	GO:0071496	<1e-4	ssr6099	GO:0044260	<1e-4	sll0062	GO:0016746	<1e-4	sll1692	GO:0044424	<1e-4	slr1951	GO:0004871	0.0295
slr1659	GO:0071842	<1e-4	ssl1255	GO:0051716	<1e-4	sll0478	GO:0022892	<1e-4	sll0630	GO:0043226	<1e-4	ssl0467	GO:0004871	0.0295
sll1766	GO:0034654	<1e-4	ssl2064	GO:0046164	<1e-4	sll1304	GO:0005342	<1e-4	sll0272	GO:0044425	<1e-4	ssl1300	GO:0004871	0.0295
ssl5025	GO:0044248	<1e-4	sll1835	GO:0009893	<1e-4	slr1142	GO:0022803	<1e-4	slr6015	GO:0044444	<1e-4	slr2071	GO:0004871	0.0295
sll0101	GO:0006576	<1e-4	slr0376	GO:0048519	<1e-4	ssr3154	GO:0016462	<1e-4	sll0241	GO:0005575	<1e-4	sll1158	GO:0004871	0.0295
sll0822	GO:0006732	<1e-4	ssr0536	GO:0044283	<1e-4	slr1235	GO:0022838	<1e-4	sll1640	GO:0005575	<1e-4	slr1290	GO:0004871	0.0295
slr1702	GO:0050801	<1e-4	slr0092	GO:0010556	<1e-4	slr2125	GO:0042623	<1e-4	sll1681	GO:0005575	<1e-4	sll1321	GO:0004871	0.0295
slr1258	GO:0071496	<1e-4	sll1250	GO:0008150	<1e-4	slr0168	GO:0003674	<1e-4	sll0449	GO:0044422	<1e-4	slr5112	GO:0004871	0.0295
slr1603	GO:0034641	<1e-4	ssr1155	GO:0009132	<1e-4	ssr5117	GO:0016818	<1e-4	sll0327	GO:0005575	<1e-4	sll1726	GO:0004871	0.0295
slr1307	GO:0006721	<1e-4	slr0505	GO:0009144	<1e-4	slr0651	GO:0022832	<1e-4	ssl2069	GO:0044446	<1e-4	sll1373	GO:0004871	0.0295
slr1462	GO:0006631	<1e-4	slr0006	GO:0051246	<1e-4	ssr2439	GO:0042623	<1e-4	sll0141	GO:0044424	<1e-4	slr1816	GO:0004871	0.0295
sll1315	GO:0009124	<1e-4	slr5017	GO:0019222	<1e-4	slr1690	GO:0043167	<1e-4	sll1581	GO:0044464	<1e-4	sll7078	GO:0004871	0.0295
slr0291	GO:0055086	<1e-4	sll1135	GO:0048523	<1e-4	ssr1391	GO:0016746	<1e-4	sll0360	GO:0043226	<1e-4	ssl8028	GO:0004871	0.0295
sll1949	GO:0009260	<1e-4	sll1765	GO:0044282	<1e-4	slr0142	GO:0022832	<1e-4	sll1355	GO:0005575	<1e-4	slr6075	GO:0004871	0.0295
slr1209	GO:0009144	<1e-4	sll1950	GO:0006163	<1e-4	slr6068	GO:0015291	<1e-4	slr0039	GO:0044424	<1e-4	ssr7017	GO:0004871	0.0295
sll1241	GO:0009262	<1e-4	slr7102	GO:0080090	<1e-4	sll0905	GO:0043167	<1e-4	slr0348	GO:0044424	<1e-4	sll1652	GO:0004871	0.0295
slr0168	GO:0016052	<1e-4	sll1158	GO:0050789	<1e-4	slr1944	GO:0022832	<1e-4	slr1095	GO:0044446	<1e-4	slr6103	GO:0004871	0.0295
slr0650	GO:0008150	<1e-4	slr0038	GO:0042451	<1e-4	sll0749	GO:0015293	<1e-4	slr0887	GO:0044464	<1e-4	ssr6089	GO:0004871	0.0295
slr1206	GO:0080090	<1e-4	ssl2138	GO:0009309	<1e-4	sll0147	GO:0005342	<1e-4	slr7026	GO:0044422	<1e-4	slr0459	GO:0004871	0.0295
slr1674	GO:0009987	<1e-4	sll7090	GO:0043549	<1e-4	slr0712	GO:0042623	<1e-4	ssr1558	GO:0044424	<1e-4	sll0625	GO:0004871	0.0295
slr0787	GO:0019752	<1e-4	slr1670	GO:0010556	<1e-4	ssr8047	GO:0060089	<1e-4	slr8044	GO:0044446	<1e-4	slr1970	GO:0004871	0.0295
sll5032	GO:0055114	<1e-4	sll1461	GO:0043436	<1e-4	slr6104	GO:0022892	<1e-4	sll1193	GO:0044464	<1e-4	sll1380	GO:0004871	0.0295
ssr1552	GO:0051179	<1e-4	slr1495	GO:0033013	<1e-4	slr0059	GO:0005342	<1e-4	slr0655	GO:0044425	<1e-4	slr0637	GO:0004871	0.0295
slr0305	GO:0071496	<1e-4	sll0381	GO:0016053	<1e-4	slr1681	GO:0015291	<1e-4	ssr2422	GO:0044446	<1e-4	slr6033	GO:0004871	0.0295
slr1187	GO:0009144	<1e-4	sll0085	GO:0051716	<1e-4	slr1885	GO:0043492	<1e-4	slr1670	GO:0044425	<1e-4	ssr7035	GO:0004871	0.0295
slr1862	GO:0009893	<1e-4	slr1571	GO:0051186	<1e-4	sll5026	GO:0043492	<1e-4	slr0869	GO:0044446	<1e-4	ssr1041	GO:0004871	0.0295
sll1583	GO:0034641	<1e-4	sll0335	GO:0042180	<1e-4	ssr5011	GO:0016746	<1e-4	sll0238	GO:0044422	<1e-4	sll0563	GO:0004871	0.0295

sll0577	GO:0031323	<1e-4	ssl0738	GO:0009144	<1e-4	sll0678	GO:0043167	<1e-4	slr1417	GO:0044422	<1e-4	ssl1046	GO:0004871	0.0295
ssl5027	GO:0006793	<1e-4	sll0314	GO:0006066	<1e-4	sll0762	GO:0043167	<1e-4	ssl2749	GO:0044464	<1e-4	slr1406	GO:0004871	0.0295
sll1378	GO:0046164	<1e-4	sll0553	GO:0065007	<1e-4	sll1192	GO:0022836	<1e-4	sll7077	GO:0044446	<1e-4	slr1537	GO:0004871	0.0295
sll0008	GO:0071840	<1e-4	slr7012	GO:0009161	<1e-4	slr1262	GO:0016817	<1e-4	sll0913	GO:0044422	<1e-4	slr2052	GO:0004871	0.0295
slr7011	GO:0022607	<1e-4	slr7010	GO:0006576	<1e-4	sll0497	GO:0016817	<1e-4	slr6013	GO:0044444	<1e-4	sll7031	GO:0004871	0.0295
slr1958	GO:0009123	<1e-4	slr1990	GO:0044003	<1e-4	sll0749	GO:0016741	<1e-4	ssl3379	GO:0044444	<1e-4	slr7094	GO:0004871	0.0295
sll0280	GO:0010556	<1e-4	ssr0759	GO:0071841	<1e-4	sll1086	GO:0016741	<1e-4	slr0723	GO:0044464	<1e-4	sll1151	GO:0004871	0.0295
sll1273	GO:0051716	<1e-4	sll0930	GO:0019219	<1e-4	ssr5074	GO:0015077	<1e-4	slr0589	GO:0005575	<1e-4	ssl7051	GO:0004871	0.0295
slr1187	GO:0055114	<1e-4	sll0242	GO:0043933	<1e-4	slr1262	GO:0016746	<1e-4	ssl2471	GO:0044422	<1e-4	slr0300	GO:0004871	0.0295
slr6090	GO:0019219	<1e-4	ssr2975	GO:0055086	<1e-4	slr6103	GO:0022857	<1e-4	sll1582	GO:0005575	<1e-4	ssl3615	GO:0004871	0.0295
slr1084	GO:0052188	<1e-4	slr0818	GO:0072528	<1e-4	ssr6030	GO:0022832	<1e-4	sll0360	GO:0044444	<1e-4	sll0552	GO:0004871	0.0295
sll0443	GO:0048523	<1e-4	slr0509	GO:0033013	<1e-4	slr0598	GO:0016746	<1e-4	slr1978	GO:0043226	<1e-4	slr7026	GO:0004871	0.0295
ssr3410	GO:0019637	<1e-4	sll0048	GO:0052188	<1e-4	sll0736	GO:0022857	<1e-4	sll0301	GO:0044424	<1e-4	slr0948	GO:0004871	0.0295
sll1285	GO:0009141	<1e-4	sll7066	GO:0009117	<1e-4	slr0948	GO:0016817	<1e-4	sll0281	GO:0044446	<1e-4	slr0516	GO:0004871	0.0295
sll1671	GO:0051188	<1e-4	slr1441	GO:0006753	<1e-4	slr1847	GO:0016462	<1e-4	slr1173	GO:0044422	<1e-4	slr0590	GO:0004871	0.0295
slr1082	GO:0008610	<1e-4	ssr3122	GO:0009057	<1e-4	sll1873	GO:0015405	<1e-4	sll0532	GO:0044446	<1e-4	sll1476	GO:0004871	0.0295
sll0294	GO:0006811	<1e-4	slr1161	GO:0019752	<1e-4	ssr3410	GO:0043492	<1e-4	sll0175	GO:0044422	<1e-4	ssl5008	GO:0004871	0.0295
sll0265	GO:0046394	<1e-4	sll0436	GO:0051171	<1e-4	sll1252	GO:0022832	<1e-4	sll1783	GO:0044446	<1e-4	slr0271	GO:0004871	0.0295
slr7071	GO:0005996	<1e-4	slr2010	GO:0006811	<1e-4	sll0272	GO:0015291	<1e-4	sll0994	GO:0005575	<1e-4	sll0062	GO:0004871	0.0295
slr0147	GO:0034660	<1e-4	slr1142	GO:0048878	<1e-4	ssr2318	GO:0015291	<1e-4	sll1652	GO:0044464	<1e-4	slr5087	GO:0004871	0.0295
slr0964	GO:0043170	<1e-4	sll0216	GO:0009150	<1e-4	sll0590	GO:0005342	<1e-4	ssr3341	GO:0005575	<1e-4	sll1911	GO:0004871	0.0295
sll1318	GO:0046483	<1e-4	sll1692	GO:0006163	<1e-4	sll1835	GO:0022836	<1e-4	slr1659	GO:0043226	<1e-4	sll0230	GO:0004871	0.0295
slr1462	GO:0009126	<1e-4	sll0175	GO:0009161	<1e-4	sll1262	GO:0022891	<1e-4	slr0702	GO:0044446	<1e-4	slr6088	GO:0004871	0.0295
slr1128	GO:0080090	<1e-4	sll1954	GO:0045184	<1e-4	ssr8013	GO:0015075	<1e-4	ssr0761	GO:0044446	<1e-4	sll1926	GO:0004871	0.0295
ssl2814	GO:0006082	<1e-4	ssr2998	GO:0048519	<1e-4	slr0300	GO:0022891	<1e-4	slr0967	GO:0005575	<1e-4	sll0162	GO:0004871	0.0295
slr7099	GO:0080090	<1e-4	sll0860	GO:0009150	<1e-4	slr2052	GO:0022891	<1e-4	sll8004	GO:0005575	<1e-4	ssl5103	GO:0004871	0.0295
slr1932	GO:0071840	<1e-4	ssr2962	GO:0044260	<1e-4	sll0623	GO:0022890	<1e-4	slr1391	GO:0044444	<1e-4	slr1886	GO:0004871	0.0295
sll0266	GO:0034660	<1e-4	ssl2717	GO:0009161	<1e-4	sll1714	GO:0015267	<1e-4	sll5062	GO:0044464	<1e-4	sll1068	GO:0004871	0.0295
sll0913	GO:0009161	<1e-4	slr0765	GO:0044248	<1e-4	slr0408	GO:0022832	<1e-4	slr0582	GO:0044422	<1e-4	sll1049	GO:0004871	0.0295
sll5097	GO:0043436	<1e-4	ssl1552	GO:0009308	<1e-4	slr0780	GO:0022892	<1e-4	slr1062	GO:0044422	<1e-4	sll1640	GO:0004871	0.0295
slr0815	GO:0071841	<1e-4	sll7065	GO:0006082	<1e-4	sll1174	GO:0022891	<1e-4	slr1240	GO:0005575	<1e-4	slr6104	GO:0004871	0.0295
sll0176	GO:0019637	<1e-4	sll5069	GO:0009987	<1e-4	slr1789	GO:0022838	<1e-4	ssl0312	GO:0043226	<1e-4	slr5077	GO:0004871	0.0295
sll1225	GO:0006066	<1e-4	slr1917	GO:0043412	<1e-4	slr1391	GO:0016746	<1e-4	sll5089	GO:0044422	<1e-4	sll5089	GO:0004871	0.0295
slr1493	GO:0006220	<1e-4	sll0280	GO:0009987	<1e-4	sll2015	GO:0016741	<1e-4	slr1627	GO:0005575	<1e-4	slr5101	GO:0004871	0.0295
sll0735	GO:0009893	<1e-4	slr0637	GO:0051171	<1e-4	sll1086	GO:0016462	<1e-4	slr0976	GO:0043226	<1e-4	slr0885	GO:0004871	0.0295
slr1142	GO:0044255	<1e-4	slr1752	GO:0051246	<1e-4	slr0670	GO:0008171	<1e-4	slr1573	GO:0044446	<1e-4	sll1495	GO:0004871	0.0295
slr5127	GO:0090304	<1e-4	slr1301	GO:0034660	<1e-4	sll0068	GO:0022891	<1e-4	slr1087	GO:0044446	<1e-4	sll1340	GO:0004871	0.0295
slr0613	GO:0009165	<1e-4	sll0048	GO:0051347	<1e-4	sll0296	GO:0015267	<1e-4	ssl0738	GO:0044446	<1e-4	ssl1552	GO:0004871	0.0295
slr0196	GO:0044248	<1e-4	sll1151	GO:0046907	<1e-4	slr1799	GO:0003674	<1e-4	sll0609	GO:0044425	<1e-4	sll0249	GO:0004871	0.0295
ssr1765	GO:0050789	<1e-4	slr0935	GO:0009056	<1e-4	sll0860	GO:0015293	<1e-4	ssl1255	GO:0044446	<1e-4	sll7089	GO:0004871	0.0295
slr1636	GO:0043933	<1e-4	slr1127	GO:0010468	<1e-4	slr0848	GO:0022857	<1e-4	slr1875	GO:0044424	<1e-4	ssl8039	GO:0004871	0.0295
slr0393	GO:0055114	<1e-4	sll0584	GO:0071842	<1e-4	sll1954	GO:0022891	<1e-4	sll0985	GO:0044444	<1e-4	slr1911	GO:0004871	0.0295
slr1613	GO:0006732	<1e-4	slr0270	GO:0016053	<1e-4	slr0730	GO:0060089	<1e-4	sll0787	GO:0044444	<1e-4	slr0625	GO:0004871	0.0295
sll0997	GO:0006576	<1e-4	slr1406	GO:0043436	<1e-4	ssr3341	GO:0016818	<1e-4	slr0112	GO:0005575	<1e-4	slr2103	GO:0004871	0.0295
sll5128	GO:0009161	<1e-4	sll0482	GO:0043412	<1e-4	sll0552	GO:0008171	<1e-4	slr0341	GO:0043226	<1e-4	slr1875	GO:0004871	0.0295
slr2000	GO:0043933	<1e-4	slr0924	GO:0048878	<1e-4	slr1767	GO:0022836	<1e-4	slr5116	GO:0005575	<1e-4	slr1577	GO:0004871	0.0295
slr0586	GO:0043436	<1e-4	slr1636	GO:0006811	<1e-4	slr1533	GO:0043167	<1e-4	slr1958	GO:0044464	<1e-4	ssl1378	GO:0004871	0.0295
slr0885	GO:0019751	<1e-4	sll0293	GO:0052188	<1e-4	slr0503	GO:0003674	<1e-4	sll5062	GO:0044424	<1e-4	slr1327	GO:0004871	0.0295
ssr2843	GO:0046164	<1e-4	sll0858	GO:0009126	<1e-4	sll1225	GO:0015075	<1e-4	sll1832	GO:0044425	<1e-4	ssl5091	GO:0004871	0.0295
sll7063	GO:0051171	<1e-4	sll0167	GO:0050794	<1e-4	slr1648	GO:0015291	<1e-4	ssr1155	GO:0005575	<1e-4	ssl7039	GO:0004871	0.0295
sll1040	GO:0034654	<1e-4	sll1186	GO:0033013	<1e-4	slr5127	GO:0022857	<1e-4	sgl0001	GO:0044464	<1e-4	ssl5100	GO:0004871	0.0295
slr1590	GO:0050789	<1e-4	ssr6020	GO:0010468	<1e-4	slr0455	GO:0016741	<1e-4	slr1547	GO:0044464	<1e-4	slr5102	GO:0004871	0.0295
sll0508	GO:0051347	<1e-4	slr1056	GO:0043412	<1e-4	ssr1258	GO:0022891	<1e-4	ssr6019	GO:0044464	<1e-4	slr2070	GO:0004871	0.0295
sll0369	GO:0009144	<1e-4	slr0610	GO:0042180	<1e-4	slr1079	GO:0015293	<1e-4	sll0394	GO:0044446	<1e-4	ssr7084	GO:0004871	0.0295

sll0913	GO:0019222	<1e-4	sll1158	GO:0080090	<1e-4	sll1350	GO:0043167	<1e-4	ssr2972	GO:0005575	<1e-4	slr5073	GO:0004871	0.0295
slr0386	GO:0009150	<1e-4	slr1183	GO:0016054	<1e-4	sll1217	GO:0003674	<1e-4	ssr1425	GO:0044422	<1e-4	ssr3402	GO:0004871	0.0295
sll1552	GO:0051716	<1e-4	ssr2333	GO:0046128	<1e-4	ssr5121	GO:0043492	<1e-4	ssr5120	GO:0043226	<1e-4	slr7057	GO:0004871	0.0295
ssr3570	GO:0009132	<1e-4	sll1022	GO:0060255	<1e-4	sll1002	GO:0022890	<1e-4	sll0539	GO:0044425	<1e-4	slr0587	GO:0004871	0.0295
slr5017	GO:0060255	<1e-4	slr1223	GO:0042451	<1e-4	sll1891	GO:0015077	<1e-4	sll0242	GO:0005575	<1e-4	slr6045	GO:0004871	0.0295
sll1160	GO:0043170	<1e-4	slr0341	GO:0048518	<1e-4	slr1258	GO:0005342	<1e-4	sll7047	GO:0043226	<1e-4	sll0984	GO:0004871	0.0295
sll0218	GO:0043549	<1e-4	sll1265	GO:0046907	<1e-4	sll1201	GO:0022838	<1e-4	slr0333	GO:0044425	<1e-4	ssr0657	GO:0004871	0.0295
sll0442	GO:0048522	<1e-4	sll1691	GO:0050789	<1e-4	slr0751	GO:0022891	<1e-4	slr7081	GO:0044444	<1e-4	sll0875	GO:0004871	0.0295
sll1652	GO:0042180	<1e-4	slr1025	GO:0009308	<1e-4	slr1896	GO:0022891	<1e-4	sll0157	GO:0044422	<1e-4	slr1599	GO:0004871	0.0295
sll6055	GO:0048523	<1e-4	slr2118	GO:0048878	<1e-4	slr0356	GO:0022892	<1e-4	slr1790	GO:0043226	<1e-4	slr1163	GO:0004871	0.0295
sll1541	GO:0050801	<1e-4	slr0586	GO:0033013	<1e-4	sll0498	GO:0016462	<1e-4	slr5077	GO:0044464	<1e-4	slr0241	GO:0004871	0.0295
ssr6083	GO:0046128	<1e-4	slr0709	GO:0006220	<1e-4	sll1089	GO:0015267	<1e-4	slr7057	GO:0043226	<1e-4	slr8022	GO:0004871	0.0295
ssr1528	GO:0044260	<1e-4	ssr0102	GO:0042455	<1e-4	sll1632	GO:0016462	<1e-4	slr1437	GO:0044446	<1e-4	ssr2047	GO:0004871	0.0295
slr0575	GO:0071841	<1e-4	sll0413	GO:0022607	<1e-4	slr6101	GO:0022832	<1e-4	slr0207	GO:0005575	<1e-4	sll0101	GO:0004871	0.0295
sll0763	GO:0046483	<1e-4	sll1950	GO:0016053	<1e-4	ssr3467	GO:0022892	<1e-4	ssr6020	GO:0044444	<1e-4	sll1898	GO:0004871	0.0295
ssr6027	GO:0043412	<1e-4	ssr1155	GO:0051171	<1e-4	sll0981	GO:0043167	<1e-4	ssl8003	GO:0044464	<1e-4	slr5012	GO:0004871	0.0295
sll1389	GO:0019222	<1e-4	sll1239	GO:0001932	<1e-4	slr0496	GO:0008171	<1e-4	sll8019	GO:0044422	<1e-4	ssr2975	GO:0004871	0.0295
sll1573	GO:0009142	<1e-4	slr7024	GO:0046128	<1e-4	sll0481	GO:0022836	<1e-4	slr0954	GO:0044464	<1e-4	slr0865	GO:0004871	0.0295
slr1436	GO:0006220	<1e-4	slr1045	GO:0019222	<1e-4	slr0423	GO:0016817	<1e-4	slr0521	GO:0005575	<1e-4	ssr1425	GO:0004871	0.0295
sll5130	GO:0046164	<1e-4	ssl5114	GO:0051716	<1e-4	sll1979	GO:0022892	<1e-4	slr1307	GO:0005575	<1e-4	slr0634	GO:0004871	0.0295
slr6101	GO:0051188	<1e-4	slr6013	GO:0009394	<1e-4	ssr3402	GO:0022890	<1e-4	sll0218	GO:0043226	<1e-4	slr6029	GO:0004871	0.0295
sll1784	GO:0009117	<1e-4	slr0364	GO:0009117	<1e-4	slr1911	GO:0015291	<1e-4	ssl5096	GO:0044425	<1e-4	sll5004	GO:0004871	0.0295
slr0392	GO:0009394	<1e-4	slr1915	GO:0050801	<1e-4	slr1627	GO:0016818	<1e-4	slr2012	GO:0043226	<1e-4	sll8011	GO:0004871	0.0295
slr1128	GO:0008150	<1e-4	sll1061	GO:0016054	<1e-4	slr1222	GO:0005342	<1e-4	ssr3304	GO:0044446	<1e-4	sll1784	GO:0004871	0.0295
sll1162	GO:0008150	<1e-4	sll1611	GO:0019637	<1e-4	slr2060	GO:0015077	<1e-4	sll0007	GO:0043226	<1e-4	sll1089	GO:0004871	0.0295
sll8011	GO:0051234	<1e-4	slr1923	GO:0009161	<1e-4	slr1084	GO:0022838	<1e-4	sll0783	GO:0044444	<1e-4	slr1624	GO:0004871	0.0295
sll0189	GO:0019637	<1e-4	slr0104	GO:0044106	<1e-4	ssr2711	GO:0015399	<1e-4	sll0446	GO:0005575	<1e-4	sll7086	GO:0004871	0.0295
slr1541	GO:0080090	<1e-4	ssr2047	GO:0046907	<1e-4	sll1004	GO:0015293	<1e-4	ssr6046	GO:0043226	<1e-4	sll7028	GO:0004871	0.0295
ssr6003	GO:0006811	<1e-4	sll8035	GO:0009141	<1e-4	sll1906	GO:0003674	<1e-4	slr7092	GO:0044424	<1e-4	slr5037	GO:0004871	0.0295
ssr3532	GO:0016053	<1e-4	sll0639	GO:0051818	<1e-4	sll1250	GO:0003674	<1e-4	slr1170	GO:0044444	<1e-4	slr7100	GO:0004871	0.0295
ssr6026	GO:0019219	<1e-4	slr1484	GO:0048519	<1e-4	slr1970	GO:0022892	<1e-4	slr1122	GO:0044424	<1e-4	slr6081	GO:0004871	0.0295
slr7012	GO:0051171	<1e-4	sll0096	GO:0072524	<1e-4	ssl5008	GO:0015267	<1e-4	slr6064	GO:0044422	<1e-4	sll0293	GO:0016820	0.0291
slr0602	GO:0006631	<1e-4	slr6044	GO:0009394	<1e-4	slr1990	GO:0042623	<1e-4	ssr6062	GO:0044424	<1e-4	sll0863	GO:0016820	0.0291
slr0453	GO:0050801	<1e-4	slr0890	GO:0006091	<1e-4	slr2038	GO:0015399	<1e-4	sll0923	GO:0043226	<1e-4	sll0846	GO:0016820	0.0291
ssr0692	GO:0009987	<1e-4	slr6103	GO:0019637	<1e-4	sll1542	GO:0042623	<1e-4	slr1102	GO:0044446	<1e-4	ssr0332	GO:0016820	0.0291
slr5013	GO:0019222	<1e-4	ssr3571	GO:0009057	<1e-4	slr1098	GO:0005342	<1e-4	sll1024	GO:0043226	<1e-4	slr1095	GO:0016820	0.0291
slr0060	GO:0065007	<1e-4	sll0780	GO:0044260	<1e-4	ssr8013	GO:0043492	<1e-4	ssl3549	GO:0044424	<1e-4	sll8032	GO:0016820	0.0291
slr0909	GO:0044106	<1e-4	sll1262	GO:0008150	<1e-4	slr6080	GO:0015077	<1e-4	sll7029	GO:0044424	<1e-4	ssr5120	GO:0016820	0.0291
slr0709	GO:0033013	<1e-4	slr6106	GO:0009161	<1e-4	slr7014	GO:0022838	<1e-4	slr1215	GO:0044425	<1e-4	sll0518	GO:0016820	0.0291
ssl3291	GO:0009308	<1e-4	slr0755	GO:0052188	<1e-4	slr0109	GO:0015405	<1e-4	sll0412	GO:0044444	<1e-4	sll1155	GO:0016820	0.0291
slr2111	GO:0009123	<1e-4	slr0453	GO:0019362	<1e-4	sll1272	GO:0016746	<1e-4	slr1570	GO:0044446	<1e-4	sll0860	GO:0016820	0.0291
slr1568	GO:0042455	<1e-4	sll0424	GO:0006721	<1e-4	slr8021	GO:0022803	<1e-4	sll1942	GO:0044444	<1e-4	sll7043	GO:0016820	0.0291
sll0481	GO:0009124	<1e-4	sll1461	GO:0006793	<1e-4	slr2049	GO:0022803	<1e-4	sll1455	GO:0044424	<1e-4	slr0262	GO:0016820	0.0291
sll1954	GO:0019222	<1e-4	slr1999	GO:0042180	<1e-4	ssl3549	GO:0016818	<1e-4	slr1210	GO:0044424	<1e-4	ssl2717	GO:0016820	0.0291
sll0762	GO:0042180	<1e-4	slr2125	GO:0006720	<1e-4	slr1813	GO:0015405	<1e-4	slr6004	GO:0005575	<1e-4	ssl3451	GO:0016820	0.0291
sll1542	GO:0055114	<1e-4	ssr7093	GO:0051234	<1e-4	slr2011	GO:0060089	<1e-4	slr8022	GO:0044422	<1e-4	ssl0312	GO:0016820	0.0291
ssr6026	GO:0080090	<1e-4	slr1203	GO:0019222	<1e-4	sll0102	GO:0015293	<1e-4	slr0299	GO:0043226	<1e-4	slr1546	GO:0016820	0.0291
sgl0001	GO:0019219	<1e-4	slr1773	GO:0001932	<1e-4	sll1512	GO:0016746	<1e-4	sll1399	GO:0005575	<1e-4	ssr1951	GO:0016820	0.0291
slr1657	GO:0050794	<1e-4	sll1273	GO:0044281	<1e-4	ssr3402	GO:0022838	<1e-4	sll0982	GO:0043226	<1e-4	slr1813	GO:0016820	0.0291
slr1800	GO:0009987	<1e-4	slr1920	GO:0009893	<1e-4	sll1884	GO:0022857	<1e-4	ssr5020	GO:0005575	<1e-4	slr0038	GO:0016820	0.0291
ssl3451	GO:0009259	<1e-4	sll1191	GO:0043412	<1e-4	slr1053	GO:0015291	<1e-4	sll5032	GO:0005575	<1e-4	slr0569	GO:0016820	0.0291
slr6016	GO:0043648	<1e-4	ssr6085	GO:0006220	<1e-4	slr1613	GO:0043167	<1e-4	slr6087	GO:0044464	<1e-4	slr2105	GO:0016820	0.0291
slr2038	GO:0051171	<1e-4	ssr7084	GO:0044003	<1e-4	sll0168	GO:0022836	<1e-4	ssr7084	GO:0044464	<1e-4	slr2119	GO:0016820	0.0291
slr1396	GO:0048523	<1e-4	slr1306	GO:0044106	<1e-4	slr1406	GO:0015291	<1e-4	sll0218	GO:0044422	<1e-4	slr1068	GO:0016820	0.0291

slr1864	GO:0009893	<1e-4	sll5130	GO:0006793	<1e-4	slr0337	GO:0043492	<1e-4	sll0670	GO:0044464	<1e-4	slr2118	GO:0016820	0.0291
sll0350	GO:0042180	<1e-4	sll0888	GO:0010468	<1e-4	slr1025	GO:0015399	<1e-4	slr0981	GO:0044424	<1e-4	sll6054	GO:0016820	0.0291
slr1276	GO:0005996	<1e-4	slr1263	GO:0052188	<1e-4	sll0242	GO:0016462	<1e-4	slr1419	GO:0044424	<1e-4	sll0803	GO:0016820	0.0291
sll1542	GO:0034641	<1e-4	sll1262	GO:0009165	<1e-4	sll1307	GO:0022857	<1e-4	slr6045	GO:0044444	<1e-4	slr0168	GO:0016820	0.0291
slr1384	GO:0043549	<1e-4	slr0334	GO:0046394	<1e-4	sll1174	GO:0022890	<1e-4	sll5128	GO:0044444	<1e-4	slr1263	GO:0016820	0.0291
sll1191	GO:0019752	<1e-4	slr2048	GO:0051347	<1e-4	slr1636	GO:0022836	<1e-4	sll0243	GO:0005575	<1e-4	sll0102	GO:0016820	0.0291
sll8012	GO:0043412	<1e-4	sll0815	GO:0010468	<1e-4	slr2115	GO:0015399	<1e-4	ssl2384	GO:0043226	<1e-4	slr0388	GO:0016820	0.0291
ssl2162	GO:0019752	<1e-4	sll7069	GO:0043648	<1e-4	slr1442	GO:0016741	<1e-4	ssl2814	GO:0043226	<1e-4	slr1104	GO:0016820	0.0291
slr6106	GO:0022411	<1e-4	sll1751	GO:0051179	<1e-4	slr1495	GO:0015291	<1e-4	sll0149	GO:0044444	<1e-4	slr1484	GO:0016820	0.0291
sll1752	GO:0050794	<1e-4	sll1830	GO:0031326	<1e-4	slr1194	GO:0042623	<1e-4	slr6049	GO:0044446	<1e-4	sll1769	GO:0016820	0.0291
slr0581	GO:0048878	<1e-4	slr7060	GO:0051234	<1e-4	ssl2807	GO:0060089	<1e-4	sll0779	GO:0044464	<1e-4	ssr2422	GO:0016820	0.0291
sll1372	GO:0009123	<1e-4	ssl0483	GO:0006766	<1e-4	slr2011	GO:0022803	<1e-4	sll1174	GO:0043226	<1e-4	slr1864	GO:0016820	0.0291
ssl3549	GO:0006733	<1e-4	sll8019	GO:0022411	<1e-4	slr0907	GO:0022838	<1e-4	sll0858	GO:0044422	<1e-4	slr0380	GO:0016820	0.0291
ssl2064	GO:0051347	<1e-4	sll1155	GO:0009259	<1e-4	slr1644	GO:0015291	<1e-4	slr1885	GO:0043226	<1e-4	slr1576	GO:0016820	0.0291
sll1006	GO:0009263	<1e-4	ssl5091	GO:0051716	<1e-4	sll5061	GO:0015077	<1e-4	slr0232	GO:0044422	<1e-4	ssl1004	GO:0016820	0.0291
sll0436	GO:0009987	<1e-4	ssl7042	GO:0044260	<1e-4	slr1052	GO:0060089	<1e-4	slr2011	GO:0005575	<1e-4	sll0611	GO:0016820	0.0291
slr2084	GO:0044003	<1e-4	sll0669	GO:0051179	<1e-4	sll0298	GO:0043492	<1e-4	slr0305	GO:0044446	<1e-4	sll0886	GO:0016820	0.0291
slr0919	GO:0005996	<1e-4	slr0872	GO:0009309	<1e-4	ssl1378	GO:0016462	<1e-4	sll1692	GO:0044464	<1e-4	slr5116	GO:0016820	0.0291
sll0096	GO:0048878	<1e-4	slr1196	GO:0019752	<1e-4	sll5062	GO:0005342	<1e-4	slr0505	GO:0044464	<1e-4	ssl5096	GO:0016820	0.0291
sll1049	GO:0006066	<1e-4	slr0592	GO:0051818	<1e-4	slr1648	GO:0022836	<1e-4	sll1581	GO:0043226	<1e-4	sll5032	GO:0016820	0.0291
slr1023	GO:0071496	<1e-4	sll5003	GO:0022607	<1e-4	sll1304	GO:0022857	<1e-4	slr1913	GO:0043226	<1e-4	sll0710	GO:0016820	0.0291
ssr3402	GO:0044271	<1e-4	slr7057	GO:0009199	<1e-4	slr1951	GO:0015405	<1e-4	slr1590	GO:0044422	<1e-4	slr6090	GO:0016820	0.0291
sll8012	GO:0043436	<1e-4	slr2025	GO:0009889	<1e-4	slr0509	GO:0015293	<1e-4	ssl7051	GO:0043226	<1e-4	ssl7046	GO:0016820	0.0291
sll1866	GO:0006753	<1e-4	sll5062	GO:0051818	<1e-4	slr1847	GO:0003674	<1e-4	sll1613	GO:0044446	<1e-4	slr1619	GO:0016820	0.0291
sll1318	GO:0009117	<1e-4	sll1262	GO:0009893	<1e-4	sll0858	GO:0043492	<1e-4	slr0914	GO:0044444	<1e-4	sll1853	GO:0016820	0.0291
slr1847	GO:0006091	<1e-4	ssr5092	GO:0072521	<1e-4	ssl0483	GO:0022836	<1e-4	sll0272	GO:0044422	<1e-4	sll0872	GO:0016820	0.0291
sll1174	GO:0006733	<1e-4	sll0905	GO:0043648	<1e-4	slr5023	GO:0022857	<1e-4	ssl5096	GO:0043226	<1e-4	sll0762	GO:0016820	0.0291
ssr6089	GO:0046394	<1e-4	sll1979	GO:0043648	<1e-4	ssr8013	GO:0008171	<1e-4	sll1698	GO:0044425	<1e-4	ssr6032	GO:0016820	0.0291
sll0670	GO:0048518	<1e-4	slr0610	GO:0044281	<1e-4	slr0975	GO:0060089	<1e-4	sll0298	GO:0043226	<1e-4	sll5090	GO:0016820	0.0291
sll1797	GO:0009199	<1e-4	slr2027	GO:0043412	<1e-4	slr6006	GO:0016462	<1e-4	slr0211	GO:0044464	<1e-4	ssr6020	GO:0016820	0.0291
slr0364	GO:0006811	<1e-4	sll1151	GO:0052188	<1e-4	sll5062	GO:0015405	<1e-4	sll0044	GO:0005575	<1e-4	sll0167	GO:0016820	0.0291
slr1103	GO:0009144	<1e-4	sll1359	GO:0006793	<1e-4	sll1541	GO:0022803	<1e-4	slr1917	GO:0044425	<1e-4	sll1906	GO:0016820	0.0291
slr0650	GO:0051818	<1e-4	slr0755	GO:0044003	<1e-4	slr1911	GO:0022892	<1e-4	sll0862	GO:0005575	<1e-4	sll1109	GO:0016820	0.0291
slr0962	GO:0031323	<1e-4	sll6055	GO:0046164	<1e-4	slr2038	GO:0003674	<1e-4	ssl1378	GO:0043226	<1e-4	slr0337	GO:0016820	0.0291
slr1287	GO:0055114	<1e-4	slr0092	GO:0071496	<1e-4	slr7095	GO:0016817	<1e-4	sll1352	GO:0044422	<1e-4	slr1103	GO:0016820	0.0291
sll0910	GO:0009199	<1e-4	slr1977	GO:0046394	<1e-4	sll0456	GO:0016741	<1e-4	slr1593	GO:0005575	<1e-4	slr0059	GO:0016820	0.0291
slr0602	GO:0043648	<1e-4	slr0243	GO:0009199	<1e-4	sll1761	GO:0043167	<1e-4	ssr2803	GO:0005575	<1e-4	sll2013	GO:0016820	0.0291
slr1431	GO:0006631	<1e-4	slr1438	GO:0033013	<1e-4	sll1654	GO:0060089	<1e-4	sll5034	GO:0005575	<1e-4	slr1168	GO:0016820	0.0291
slr1875	GO:0010468	<1e-4	slr7058	GO:0019222	<1e-4	slr1236	GO:0015075	<1e-4	slr0204	GO:0044446	<1e-4	ssr3410	GO:0016820	0.0291
sll1921	GO:0001932	<1e-4	slr1915	GO:0090304	<1e-4	sll1389	GO:0042623	<1e-4	sll1730	GO:0044446	<1e-4	sll0176	GO:0016820	0.0291
sll1265	GO:0044003	<1e-4	slr0192	GO:0006082	<1e-4	sll1634	GO:0060089	<1e-4	sll1166	GO:0044446	<1e-4	slr7025	GO:0016820	0.0291
sll0804	GO:0010556	<1e-4	sll0218	GO:0008610	<1e-4	slr1397	GO:0016817	<1e-4	slr0959	GO:0044422	<1e-4	sll1273	GO:0016820	0.0291
sll1601	GO:0019362	<1e-4	sll0822	GO:0044248	<1e-4	sll1307	GO:0016818	<1e-4	slr1260	GO:0005575	<1e-4	sll0630	GO:0016820	0.0291
slr6074	GO:0019222	<1e-4	sll0839	GO:0009309	<1e-4	ssl1923	GO:0042623	<1e-4	slr1852	GO:0044422	<1e-4	sll1160	GO:0016820	0.0291
slr1287	GO:0009262	<1e-4	sll0656	GO:0009056	<1e-4	sll7069	GO:0015075	<1e-4	slr6007	GO:0044422	<1e-4	slr1956	GO:0016820	0.0291
sll1411	GO:0009141	<1e-4	sll0294	GO:0065007	<1e-4	slr0667	GO:0043167	<1e-4	slr0575	GO:0043226	<1e-4	sll5026	GO:0016820	0.0291
ssl1378	GO:0048518	<1e-4	slr0692	GO:0019222	<1e-4	slr1990	GO:0015293	<1e-4	slr1944	GO:0005575	<1e-4	ssl3379	GO:0016820	0.0291
sll1071	GO:0060255	<1e-4	ssr2754	GO:0046907	<1e-4	slr1353	GO:0022836	<1e-4	slr1998	GO:0044425	<1e-4	sll7047	GO:0016820	0.0291
ssl2749	GO:0009987	<1e-4	slr1790	GO:0016053	<1e-4	slr6088	GO:0015399	<1e-4	slr0480	GO:0044422	<1e-4	sll1766	GO:0016820	0.0291
slr1188	GO:0019751	<1e-4	slr0318	GO:0046164	<1e-4	ssl0350	GO:0015077	<1e-4	slr1079	GO:0044444	<1e-4	slr0552	GO:0016820	0.0291
slr0059	GO:0072522	<1e-4	slr0702	GO:0071842	<1e-4	sll8035	GO:0022803	<1e-4	sll1830	GO:0044444	<1e-4	sll1961	GO:0016820	0.0291
slr1570	GO:0019637	<1e-4	slr2048	GO:0009165	<1e-4	sll1155	GO:0016818	<1e-4	sll1350	GO:0044446	<1e-4	ssr3341	GO:0016820	0.0291
slr0924	GO:0051818	<1e-4	slr1263	GO:0009124	<1e-4	slr1187	GO:0022857	<1e-4	sll8027	GO:0044444	<1e-4	slr7096	GO:0016820	0.0291
slr0656	GO:0051246	<1e-4	ssl8005	GO:0043549	<1e-4	ssl0410	GO:0016818	<1e-4	sll1488	GO:0044422	<1e-4	sll1355	GO:0016820	0.0291

ssr1155	GO:0009124	<1e-4	ssr2975	GO:0008150	<1e-4	ssl2971	GO:0022838	<1e-4	slr0914	GO:0005575	<1e-4	sll1773	GO:0016820	0.0291
slr0801	GO:0019752	<1e-4	ssr5019	GO:0071840	<1e-4	slr0587	GO:0015075	<1e-4	sll8012	GO:0044446	<1e-4	slr0195	GO:0016820	0.0291
sll7077	GO:0009262	<1e-4	sll1424	GO:0006811	<1e-4	ssl2595	GO:0060089	<1e-4	slr6050	GO:0044424	<1e-4	sll1884	GO:0016820	0.0291
sll1656	GO:0019751	<1e-4	slr1230	GO:0016053	<1e-4	ssl0483	GO:0015291	<1e-4	ssr2067	GO:0044424	<1e-4	sll5006	GO:0016820	0.0291
slr0848	GO:0072522	<1e-4	sll0913	GO:0044260	<1e-4	sll1531	GO:0042623	<1e-4	sll0909	GO:0044425	<1e-4	sll5034	GO:0016820	0.0291
sll1510	GO:0051186	<1e-4	slr0551	GO:0009262	<1e-4	sll1060	GO:0015291	<1e-4	slr1127	GO:0044444	<1e-4	sll0328	GO:0016820	0.0291
ssr6002	GO:0009394	<1e-4	slr0553	GO:0009059	<1e-4	sll1321	GO:0016741	<1e-4	sll1247	GO:0044464	<1e-4	slr6013	GO:0016820	0.0291
sll0394	GO:0031323	<1e-4	ssr6078	GO:0044248	<1e-4	ssr1528	GO:0016818	<1e-4	sll0658	GO:0044424	<1e-4	slr1407	GO:0016820	0.0291
sll1912	GO:0051179	<1e-4	sll1222	GO:0009161	<1e-4	sll0913	GO:0016462	<1e-4	slr1107	GO:0044422	<1e-4	sll1764	GO:0016820	0.0291
sll0280	GO:0044248	<1e-4	ssr5121	GO:0009117	<1e-4	slr0263	GO:0015291	<1e-4	sll1542	GO:0044422	<1e-4	sll8007	GO:0016820	0.0291
sll0031	GO:0052188	<1e-4	slr1163	GO:0009263	<1e-4	sll0564	GO:0015075	<1e-4	slr1611	GO:0043226	<1e-4	sll1960	GO:0016820	0.0291
slr0907	GO:0044260	<1e-4	sll0062	GO:0019637	<1e-4	sll1442	GO:0015267	<1e-4	slr1235	GO:0044444	<1e-4	sll1606	GO:0016820	0.0291
slr1152	GO:0055082	<1e-4	ssl5113	GO:0048878	<1e-4	sll1306	GO:0022832	<1e-4	sll2007	GO:0044424	<1e-4	slr1315	GO:0016820	0.0291
slr1809	GO:0019362	<1e-4	ssr6002	GO:0009124	<1e-4	ssr7036	GO:0022891	<1e-4	sll0756	GO:0044422	<1e-4	sll0266	GO:0016820	0.0291
sll1380	GO:0071554	<1e-4	slr0692	GO:0072524	<1e-4	sll1411	GO:0015291	<1e-4	slr1623	GO:0044424	<1e-4	sll0218	GO:0016820	0.0291
slr1807	GO:0051188	<1e-4	sll0839	GO:0022411	<1e-4	ssl0461	GO:0022857	<1e-4	sll0361	GO:0044422	<1e-4	slr1468	GO:0016820	0.0291
ssr1765	GO:0072524	<1e-4	slr0729	GO:0034641	<1e-4	slr0601	GO:0015405	<1e-4	sll0565	GO:0044424	<1e-4	sll1511	GO:0016820	0.0291
slr2092	GO:0008150	<1e-4	slr1383	GO:0043549	<1e-4	sll1601	GO:0022838	<1e-4	sll1651	GO:0044464	<1e-4	sll1722	GO:0016820	0.0291
slr0919	GO:0046128	<1e-4	slr0317	GO:0051171	<1e-4	sll5004	GO:0022892	<1e-4	slr1081	GO:0005575	<1e-4	slr1753	GO:0016820	0.0291
ssl1690	GO:0051186	<1e-4	ssl5129	GO:0016054	<1e-4	slr0151	GO:0043167	<1e-4	ssr1375	GO:0044422	<1e-4	ssr2803	GO:0005975	0.0285
slr1880	GO:0043933	<1e-4	slr6016	GO:0019222	<1e-4	slr1927	GO:0015293	<1e-4	slr1570	GO:0005575	<1e-4	slr2018	GO:0005975	0.0285
slr1161	GO:0006753	<1e-4	sll1509	GO:0022607	<1e-4	sll0982	GO:0008171	<1e-4	slr1852	GO:0044446	<1e-4	slr2084	GO:0005975	0.0285
slr1406	GO:0009123	<1e-4	slr5126	GO:0050896	<1e-4	slr0250	GO:0003674	<1e-4	sll7077	GO:0043226	<1e-4	sll0272	GO:0005975	0.0285
sll0068	GO:0034660	<1e-4	sll1763	GO:0044106	<1e-4	ssr1768	GO:0016818	<1e-4	slr6033	GO:0043226	<1e-4	sll1002	GO:0016772	0.0285
sll1611	GO:0044275	<1e-4	slr1752	GO:0006720	<1e-4	slr1998	GO:0022857	<1e-4	slr0812	GO:0043226	<1e-4	slr0586	GO:0016772	0.0285
sll1873	GO:0050896	<1e-4	slr1209	GO:0048878	<1e-4	slr1546	GO:0016817	<1e-4	sll8002	GO:0044425	<1e-4	sll0381	GO:0016772	0.0285
sll0071	GO:0009991	<1e-4	slr1464	GO:0051186	<1e-4	ssl2138	GO:0042623	<1e-4	slr0699	GO:0044424	<1e-4	sll1006	GO:0016772	0.0285
slr1624	GO:0009199	<1e-4	slr1959	GO:0009141	<1e-4	sll0925	GO:0022892	<1e-4	sll6052	GO:0043226	<1e-4	sll0414	GO:0016772	0.0285
ssl3829	GO:0009892	<1e-4	sll1527	GO:0072521	<1e-4	sll1511	GO:0008171	<1e-4	slr0711	GO:0044425	<1e-4	slr1437	GO:0016772	0.0285
ssr7072	GO:0044271	<1e-4	sll1193	GO:0044281	<1e-4	sll0727	GO:0022836	<1e-4	sll0685	GO:0044425	<1e-4	sll0314	GO:0016772	0.0285
sll1340	GO:0033013	<1e-4	sll0478	GO:0044248	<1e-4	slr1599	GO:0016817	<1e-4	sll0577	GO:0044464	<1e-4	slr0921	GO:0016772	0.0285
slr0109	GO:0051186	<1e-4	slr1721	GO:0055086	<1e-4	slr1577	GO:0016818	<1e-4	slr1203	GO:0005575	<1e-4	sll1830	GO:0016772	0.0285
slr1677	GO:0009892	<1e-4	slr1895	GO:0052111	<1e-4	sll1505	GO:0015405	<1e-4	sll1162	GO:0005575	<1e-4	sll0780	GO:0016772	0.0285
sll1979	GO:0006720	<1e-4	slr2025	GO:0072522	<1e-4	slr1187	GO:0022836	<1e-4	sll0177	GO:0005575	<1e-4	sll0066	GO:0016772	0.0285
ssl7038	GO:0044271	<1e-4	slr1827	GO:0043170	<1e-4	sll1654	GO:0016818	<1e-4	slr2092	GO:0044446	<1e-4	slr2144	GO:0016772	0.0285
slr0582	GO:0080090	<1e-4	slr0199	GO:0016052	<1e-4	sll0031	GO:0016462	<1e-4	slr1677	GO:0043226	<1e-4	sll0010	GO:0016772	0.0285
sll1071	GO:0051347	<1e-4	slr5017	GO:0019751	<1e-4	sll0499	GO:0016817	<1e-4	sll0611	GO:0044422	<1e-4	sll0524	GO:0016772	0.0285
sll0436	GO:0006811	<1e-4	slr7060	GO:0009987	<1e-4	sll1651	GO:0022803	<1e-4	sll1995	GO:0005575	<1e-4	sll0309	GO:0016772	0.0285
ssl3451	GO:0080090	<1e-4	sll7065	GO:0001932	<1e-4	slr1417	GO:0022892	<1e-4	slr1915	GO:0044424	<1e-4	sll1656	GO:0016772	0.0285
slr5077	GO:0006811	<1e-4	sll1528	GO:0006732	<1e-4	sll1352	GO:0016462	<1e-4	sll1442	GO:0044464	<1e-4	slr0103	GO:0016772	0.0285
ssr6079	GO:0010556	<1e-4	sll1510	GO:0009991	<1e-4	ssl2064	GO:0015267	<1e-4	sll1858	GO:0043226	<1e-4	slr0668	GO:0016772	0.0285
sll0909	GO:0031323	<1e-4	sll0024	GO:0022607	<1e-4	slr2111	GO:0015267	<1e-4	slr0196	GO:0043226	<1e-4	sll1949	GO:0016772	0.0285
ssl8003	GO:0009309	<1e-4	slr1907	GO:0044260	<1e-4	sll0585	GO:0016817	<1e-4	sll0815	GO:0044422	<1e-4	slr2011	GO:0016772	0.0285
slr0151	GO:0001932	<1e-4	sll0611	GO:0009161	<1e-4	sll0063	GO:0008171	<1e-4	sll0843	GO:0043226	<1e-4	slr0192	GO:0016772	0.0285
sll8007	GO:0051188	<1e-4	slr1513	GO:0072522	<1e-4	ssr3154	GO:0022832	<1e-4	slr1073	GO:0044444	<1e-4	slr1572	GO:0016772	0.0285
sll1162	GO:0006091	<1e-4	sll1979	GO:0009893	<1e-4	ssl6035	GO:0016818	<1e-4	slr2048	GO:0044425	<1e-4	slr1203	GO:0016772	0.0285
slr1025	GO:0009165	<1e-4	ssl1417	GO:0009059	<1e-4	sll0982	GO:0015075	<1e-4	sgl0001	GO:0044446	<1e-4	slr1676	GO:0016772	0.0285
sll1835	GO:0050896	<1e-4	slr8021	GO:0009262	<1e-4	slr0645	GO:0022890	<1e-4	slr2011	GO:0043226	<1e-4	slr1571	GO:0016772	0.0285
slr0976	GO:0043170	<1e-4	sll1979	GO:0042180	<1e-4	slr1101	GO:0016818	<1e-4	sll0930	GO:0044424	<1e-4	slr0845	GO:0016772	0.0285
slr1533	GO:0043436	<1e-4	sll1766	GO:0006066	<1e-4	ssl0353	GO:0015293	<1e-4	slr1116	GO:0044464	<1e-4	sll1736	GO:0016772	0.0285
sll1512	GO:0006732	<1e-4	slr1600	GO:0034654	<1e-4	sll0608	GO:0008171	<1e-4	slr1235	GO:0005575	<1e-4	sll0243	GO:0016772	0.0285
sll0802	GO:0044106	<1e-4	ssl7046	GO:0033013	<1e-4	sll1640	GO:0060089	<1e-4	slr0654	GO:0005575	<1e-4	slr2073	GO:0016772	0.0285
sll0985	GO:0009893	<1e-4	slr0668	GO:0009117	<1e-4	slr1544	GO:0043167	<1e-4	slr0416	GO:0044446	<1e-4	ssl2065	GO:0016772	0.0285
sll0024	GO:0044003	<1e-4	sll0268	GO:0071842	<1e-4	slr1378	GO:0003674	<1e-4	slr0730	GO:0044424	<1e-4	slr0723	GO:0016772	0.0285

sll1934	GO:0044282	<1e-4	sll1378	GO:0052111	<1e-4	sll1853	GO:0022832	<1e-4	sll1770	GO:0043226	<1e-4	slr1262	GO:0016772	0.0285
ssr2803	GO:0019751	<1e-4	sll0614	GO:0071554	<1e-4	slr1854	GO:0015075	<1e-4	slr0651	GO:0005575	<1e-4	sll1613	GO:0016772	0.0285
slr1923	GO:0072524	<1e-4	sll1348	GO:0051171	<1e-4	slr0907	GO:0015291	<1e-4	sll8001	GO:0044425	<1e-4	slr1230	GO:0016772	0.0285
sll0181	GO:0006066	<1e-4	slr0656	GO:0043933	<1e-4	slr2073	GO:0016817	<1e-4	sll1906	GO:0044444	<1e-4	ssl0352	GO:0016772	0.0285
slr1875	GO:0009124	<1e-4	sll6055	GO:0042455	<1e-4	sll1656	GO:0016462	<1e-4	slr1084	GO:0043226	<1e-4	slr2111	GO:0016772	0.0285
slr0948	GO:0051234	<1e-4	slr0602	GO:0019219	<1e-4	sll0243	GO:0005342	<1e-4	sll1433	GO:0044446	<1e-4	slr0006	GO:0016772	0.0285
slr2049	GO:0044275	<1e-4	sll1911	GO:0042180	<1e-4	sll0577	GO:0022832	<1e-4	sll0611	GO:0005575	<1e-4	slr1618	GO:0016772	0.0285
slr0294	GO:0045184	<1e-4	slr1851	GO:0044255	<1e-4	ssl2996	GO:0022857	<1e-4	slr0184	GO:0044422	<1e-4	sll0532	GO:0016772	0.0285
slr0519	GO:0006753	<1e-4	sll1570	GO:0009059	<1e-4	sll0156	GO:0015405	<1e-4	ssl1707	GO:0044425	<1e-4	sll1086	GO:0016772	0.0285
ssr3532	GO:0009259	<1e-4	slr1384	GO:0050794	<1e-4	sll0543	GO:0003674	<1e-4	sll0761	GO:0044424	<1e-4	ssl1255	GO:0005515	0.0281
slr1056	GO:0006163	<1e-4	slr6028	GO:0006733	<1e-4	sll1853	GO:0022857	<1e-4	slr1918	GO:0044444	<1e-4	slr2092	GO:0005515	0.0281
slr1619	GO:0046394	<1e-4	sll1022	GO:0046128	<1e-4	sll1511	GO:0016818	<1e-4	slr0967	GO:0044422	<1e-4	sll1737	GO:0005515	0.0281
slr1068	GO:0044271	<1e-4	sll7067	GO:0009057	<1e-4	slr1918	GO:0042623	<1e-4	sll2015	GO:0005575	<1e-4	sll1749	GO:0008168	0.0276
slr1025	GO:0009123	<1e-4	slr2117	GO:0046164	<1e-4	slr0589	GO:0015267	<1e-4	slr6088	GO:0005575	<1e-4	sll0647	GO:0008168	0.0276
slr1601	GO:0009059	<1e-4	ssl1300	GO:0009308	<1e-4	ssl3379	GO:0016741	<1e-4	ssl1690	GO:0044446	<1e-4	ssr2803	GO:0008168	0.0276
slr0890	GO:0090304	<1e-4	slr1101	GO:0009126	<1e-4	slr0269	GO:0043167	<1e-4	sll0659	GO:0044422	<1e-4	ssr2201	GO:0008168	0.0276
sll0381	GO:0048522	<1e-4	sll0732	GO:0009123	<1e-4	ssr2049	GO:0022836	<1e-4	sll0564	GO:0044444	<1e-4	sll1902	GO:0008168	0.0276
ssr6002	GO:0006631	<1e-4	sll0811	GO:0016052	<1e-4	sll7090	GO:0022890	<1e-4	slr1636	GO:0044425	<1e-4	slr2084	GO:0008168	0.0276
sll0803	GO:0001932	<1e-4	ssl2245	GO:0051347	<1e-4	slr1275	GO:0003674	<1e-4	sll0984	GO:0044464	<1e-4	slr0208	GO:0008168	0.0276
slr0299	GO:0051179	<1e-4	sll0939	GO:0046907	<1e-4	sll1715	GO:0016462	<1e-4	sll1359	GO:0005575	<1e-4	sll0071	GO:0008168	0.0276
slr0815	GO:0046164	<1e-4	sll0481	GO:0090304	<1e-4	slr0348	GO:0022838	<1e-4	slr7037	GO:0044446	<1e-4	sll8035	GO:0005975	0.0275
sll0981	GO:0019637	<1e-4	sll7064	GO:0051347	<1e-4	sll1532	GO:0022838	<1e-4	slr1664	GO:0044422	<1e-4	sll1531	GO:0005975	0.0275
ssl1464	GO:0055086	<1e-4	sll0787	GO:0034654	<1e-4	sll6054	GO:0016462	<1e-4	ssr2843	GO:0044464	<1e-4	slr2027	GO:0005975	0.0275
slr1535	GO:0051246	<1e-4	sll0702	GO:0046128	<1e-4	ssl5098	GO:0015267	<1e-4	slr0064	GO:0044422	<1e-4	slr6091	GO:0005975	0.0275
slr6090	GO:0008610	<1e-4	ssr7084	GO:0009150	<1e-4	sll0436	GO:0016746	<1e-4	ssl5007	GO:0005575	<1e-4	slr1163	GO:0005975	0.0275
sll1162	GO:0006811	<1e-4	sll0930	GO:0034641	<1e-4	sll0274	GO:0022891	<1e-4	ssr8013	GO:0044425	<1e-4	slr8044	GO:0005975	0.0275
ssr1698	GO:0043549	<1e-4	slr7025	GO:0016052	<1e-4	slr0909	GO:0060089	<1e-4	sll0096	GO:0044424	<1e-4	ssr1155	GO:0005975	0.0275
sll0372	GO:0065007	<1e-4	sll2007	GO:0071841	<1e-4	slr0865	GO:0015405	<1e-4	sll0857	GO:0005575	<1e-4	sll1681	GO:0005975	0.0275
slr0816	GO:0052188	<1e-4	slr0870	GO:0031326	<1e-4	slr0489	GO:0015293	<1e-4	sgl0001	GO:0044424	<1e-4	sll1882	GO:0005975	0.0275
sll0737	GO:0048878	<1e-4	sll0192	GO:0048519	<1e-4	slr1591	GO:0060089	<1e-4	slr0635	GO:0044464	<1e-4	slr5087	GO:0005975	0.0275
slr0337	GO:0072527	<1e-4	sll0691	GO:0072521	<1e-4	sll8002	GO:0022832	<1e-4	ssr2912	GO:0044425	<1e-4	ssl1263	GO:0005975	0.0275
slr0489	GO:0006793	<1e-4	slr1847	GO:0046483	<1e-4	sll0564	GO:0022836	<1e-4	slr0431	GO:0043226	<1e-4	slr0582	GO:0005975	0.0275
slr1623	GO:0045184	<1e-4	slr1464	GO:0006811	<1e-4	slr7057	GO:0022832	<1e-4	sll1400	GO:0005575	<1e-4	slr0725	GO:0005975	0.0275
sml0011	GO:0052188	<1e-4	sll0298	GO:0009141	<1e-4	sll1160	GO:0043167	<1e-4	sll0072	GO:0044425	<1e-4	ssr3129	GO:0005975	0.0275
slr0337	GO:0016054	<1e-4	sll1866	GO:0050896	<1e-4	sll0553	GO:0016746	<1e-4	sll0096	GO:0043226	<1e-4	slr1886	GO:0005975	0.0275
sll0412	GO:0006811	<1e-4	slr0730	GO:0019637	<1e-4	slr0144	GO:0015075	<1e-4	sll0910	GO:0005575	<1e-4	slr1241	GO:0005975	0.0275
ssr0332	GO:0072522	<1e-4	sll0310	GO:0009260	<1e-4	sll0253	GO:0015077	<1e-4	sll0010	GO:0044424	<1e-4	sll8012	GO:0005975	0.0275
sll0751	GO:0009260	<1e-4	sll0241	GO:0006091	<1e-4	ssl2065	GO:0043492	<1e-4	ssl0461	GO:0005575	<1e-4	sll5003	GO:0005975	0.0275
ssl2996	GO:0051716	<1e-4	slr0243	GO:0055086	<1e-4	slr5119	GO:0022857	<1e-4	ssl5064	GO:0043226	<1e-4	ssl5100	GO:0005975	0.0275
slr7097	GO:0019637	<1e-4	slr1179	GO:0019222	<1e-4	slr1541	GO:0015075	<1e-4	sll7065	GO:0044464	<1e-4	slr5111	GO:0005975	0.0275
sll1306	GO:0044281	<1e-4	slr1815	GO:0009144	<1e-4	slr5101	GO:0015291	<1e-4	ssl5068	GO:0043226	<1e-4	ssl7042	GO:0005975	0.0275
sll1912	GO:0051234	<1e-4	sll0765	GO:0001932	<1e-4	sll7065	GO:0016462	<1e-4	sll1162	GO:0044444	<1e-4	sll1036	GO:0005975	0.0275
ssl2814	GO:0042180	<1e-4	ssr5092	GO:0060255	<1e-4	sll1060	GO:0015267	<1e-4	slr1128	GO:0044425	<1e-4	slr0590	GO:0005975	0.0275
ssl5103	GO:0008150	<1e-4	slr1923	GO:0051171	<1e-4	ssr1766	GO:0022803	<1e-4	sll0412	GO:0005575	<1e-4	sll0678	GO:0005975	0.0275
sll0274	GO:0009141	<1e-4	ssl2162	GO:0046394	<1e-4	sll0225	GO:0015077	<1e-4	slr1493	GO:0044444	<1e-4	slr1079	GO:0005975	0.0275
sll1380	GO:0046394	<1e-4	slr0787	GO:0009263	<1e-4	slr0142	GO:0003674	<1e-4	sll0024	GO:0044446	<1e-4	slr7057	GO:0005975	0.0275
slr0146	GO:0006082	<1e-4	ssr1155	GO:0071840	<1e-4	slr1980	GO:0015399	<1e-4	slr1196	GO:0044464	<1e-4	slr7073	GO:0005975	0.0275
sll0101	GO:0044281	<1e-4	sll7069	GO:0080090	<1e-4	sll1783	GO:0022890	<1e-4	slr1273	GO:0044422	<1e-4	slr5037	GO:0005975	0.0275
sll0751	GO:0019219	<1e-4	slr2119	GO:0009144	<1e-4	slr1920	GO:0043167	<1e-4	sll1702	GO:0044444	<1e-4	sll0736	GO:0005975	0.0275
slr0238	GO:0043412	<1e-4	slr0845	GO:0009892	<1e-4	slr0890	GO:0022832	<1e-4	slr6004	GO:0044444	<1e-4	ssr6099	GO:0005975	0.0275
sll1911	GO:0051179	<1e-4	ssr6079	GO:0072528	<1e-4	sll0614	GO:0022857	<1e-4	sll1775	GO:0005575	<1e-4	slr2000	GO:0005975	0.0275
ssl3142	GO:0043549	<1e-4	slr7099	GO:0009165	<1e-4	slr7026	GO:0022838	<1e-4	slr0588	GO:0044444	<1e-4	sll7070	GO:0005975	0.0275
slr0049	GO:0046164	<1e-4	sll0909	GO:0048518	<1e-4	slr1618	GO:0022838	<1e-4	slr0651	GO:0044425	<1e-4	slr0699	GO:0005975	0.0275
slr1303	GO:0006811	<1e-4	slr1493	GO:0046128	<1e-4	slr0271	GO:0022892	<1e-4	sll0545	GO:0044464	<1e-4	slr6044	GO:0005975	0.0275

sll1884	GO:0043436	<1e-4	sll0372	GO:0001932	<1e-4	slr1789	GO:0022857	<1e-4	sll0498	GO:0044422	<1e-4	slr0730	GO:0005975	0.0275
sll1123	GO:0018130	<1e-4	sll8035	GO:0046128	<1e-4	slr0250	GO:0043492	<1e-4	slr1939	GO:0044464	<1e-4	sll1381	GO:0005975	0.0275
sll0572	GO:0010468	<1e-4	slr1474	GO:0043648	<1e-4	slr1307	GO:0015077	<1e-4	ssl8039	GO:0005575	<1e-4	slr1290	GO:0005975	0.0275
sll0867	GO:0006811	<1e-4	slr0358	GO:0065007	<1e-4	slr6005	GO:0022892	<1e-4	slr1343	GO:0044446	<1e-4	sll1340	GO:0005975	0.0275
slr0870	GO:0050896	<1e-4	sll1151	GO:0043933	<1e-4	ssr2067	GO:0043492	<1e-4	sll0085	GO:0044444	<1e-4	slr7071	GO:0005975	0.0275
slr8014	GO:0009132	<1e-4	sll0263	GO:0010468	<1e-4	slr1290	GO:0043492	<1e-4	ssr2781	GO:0044446	<1e-4	slr0588	GO:0005975	0.0275
slr1396	GO:0009056	<1e-4	slr0270	GO:0022411	<1e-4	slr2110	GO:0022832	<1e-4	sll0547	GO:0044446	<1e-4	slr0651	GO:0005975	0.0275
ssl5008	GO:0009309	<1e-4	ssr3572	GO:0043648	<1e-4	ssl1377	GO:0015399	<1e-4	sll0160	GO:0044425	<1e-4	sll0298	GO:0005975	0.0275
slr0111	GO:0006721	<1e-4	sll1765	GO:0019222	<1e-4	ssl0483	GO:0015075	<1e-4	sll7090	GO:0005575	<1e-4	slr0976	GO:0005975	0.0275
sll0811	GO:0051171	<1e-4	sll1512	GO:0022411	<1e-4	slr1627	GO:0022836	<1e-4	slr0211	GO:0005575	<1e-4	slr0613	GO:0005975	0.0275
slr0729	GO:0009260	<1e-4	sll1749	GO:0055086	<1e-4	sll0740	GO:0015267	<1e-4	sll6053	GO:0044424	<1e-4	ssr2318	GO:0005975	0.0275
slr1896	GO:0009165	<1e-4	slr6068	GO:0031326	<1e-4	sll1036	GO:0005342	<1e-4	sll1359	GO:0044464	<1e-4	sll1095	GO:0005975	0.0275
ssr8047	GO:0034641	<1e-4	sll8019	GO:0044106	<1e-4	sll1071	GO:0015291	<1e-4	sll0445	GO:0005575	<1e-4	sll5004	GO:0005975	0.0275
sll0886	GO:0006811	<1e-4	ssl0410	GO:0006066	<1e-4	sll0815	GO:0016817	<1e-4	sll1609	GO:0043226	<1e-4	slr0865	GO:0005975	0.0275
sll0428	GO:0031323	<1e-4	slr1396	GO:0009308	<1e-4	sll1240	GO:0016746	<1e-4	slr2071	GO:0005575	<1e-4	sll7028	GO:0005975	0.0275
sll1411	GO:0019219	<1e-4	sll0656	GO:0048518	<1e-4	slr1956	GO:0043492	<1e-4	sll0608	GO:0043226	<1e-4	sll0442	GO:0005975	0.0275
sll1552	GO:0009263	<1e-4	sll5062	GO:0050896	<1e-4	slr1047	GO:0015077	<1e-4	slr6065	GO:0005575	<1e-4	slr0816	GO:0005975	0.0275
sll0898	GO:0009199	<1e-4	slr5073	GO:0051716	<1e-4	slr1209	GO:0016818	<1e-4	ssl2996	GO:0044424	<1e-4	slr0458	GO:0005975	0.0275
ssr6030	GO:0034654	<1e-4	ssr5092	GO:0031326	<1e-4	slr0142	GO:0022857	<1e-4	sll7086	GO:0043226	<1e-4	slr1142	GO:0005975	0.0275
slr0145	GO:0009123	<1e-4	ssl1417	GO:0009161	<1e-4	slr6031	GO:0015405	<1e-4	slr0818	GO:0044446	<1e-4	sll0172	GO:0005975	0.0275
sll0763	GO:0065007	<1e-4	ssr2554	GO:0044260	<1e-4	sll0369	GO:0022836	<1e-4	slr0006	GO:0005575	<1e-4	slr5013	GO:0005975	0.0275
slr1340	GO:0042451	<1e-4	ssr1528	GO:0043170	<1e-4	slr7071	GO:0022803	<1e-4	ssl5098	GO:0005575	<1e-4	sll0162	GO:0005975	0.0275
slr1699	GO:0019219	<1e-4	sll1192	GO:0006082	<1e-4	sll1340	GO:0060089	<1e-4	sll0658	GO:0044444	<1e-4	slr0692	GO:0005975	0.0275
sll0350	GO:0010556	<1e-4	sll7030	GO:0009309	<1e-4	ssl0410	GO:0015293	<1e-4	sll1956	GO:0043226	<1e-4	ssr6062	GO:0005975	0.0275
ssl7021	GO:0005996	<1e-4	slr1259	GO:0044106	<1e-4	sll1399	GO:0022832	<1e-4	slr0976	GO:0044425	<1e-4	sll1609	GO:0005975	0.0275
ssr7084	GO:0055086	<1e-4	sll0216	GO:0045184	<1e-4	slr1799	GO:0005342	<1e-4	sll0595	GO:0044424	<1e-4	slr1537	GO:0005975	0.0275
slr1306	GO:0050801	<1e-4	slr0360	GO:0043436	<1e-4	slr0731	GO:0015293	<1e-4	slr0670	GO:0044422	<1e-4	slr1174	GO:0005975	0.0275
sll0609	GO:0006220	<1e-4	slr1681	GO:0051818	<1e-4	slr1807	GO:0016818	<1e-4	sll1765	GO:0044424	<1e-4	ssr6019	GO:0005975	0.0275
ssr2317	GO:0055114	<1e-4	slr2117	GO:0045184	<1e-4	sll1002	GO:0043492	<1e-4	sll0584	GO:0044446	<1e-4	slr1450	GO:0005975	0.0275
sll0361	GO:0048518	<1e-4	ssl2384	GO:0006720	<1e-4	sll0044	GO:0015293	<1e-4	slr1616	GO:0044422	<1e-4	sll0372	GO:0005975	0.0275
slr0317	GO:0046394	<1e-4	slr0142	GO:0072521	<1e-4	sll0980	GO:0016462	<1e-4	slr1025	GO:0044424	<1e-4	slr6029	GO:0005975	0.0275
sll0564	GO:0033013	<1e-4	sll0282	GO:0006721	<1e-4	sll0424	GO:0015267	<1e-4	ssl2162	GO:0044422	<1e-4	slr2117	GO:0005975	0.0275
sll0298	GO:0019362	<1e-4	sll0268	GO:0072528	<1e-4	ssl0461	GO:0060089	<1e-4	sll1995	GO:0044464	<1e-4	slr0509	GO:0005975	0.0275
sll0031	GO:0042455	<1e-4	slr1940	GO:0051347	<1e-4	ssr5117	GO:0016741	<1e-4	sll1173	GO:0044446	<1e-4	slr5012	GO:0005975	0.0275
sll1797	GO:0060255	<1e-4	slr0948	GO:0008150	<1e-4	ssr1753	GO:0016818	<1e-4	slr0459	GO:0044446	<1e-4	slr6071	GO:0005975	0.0275
sll0810	GO:0006766	<1e-4	sll1106	GO:0072524	<1e-4	slr2071	GO:0043492	<1e-4	slr1999	GO:0044464	<1e-4	slr2004	GO:0005975	0.0275
slr2012	GO:0010556	<1e-4	sll1726	GO:0072522	<1e-4	slr0801	GO:0022836	<1e-4	slr5111	GO:0043226	<1e-4	sll1911	GO:0005975	0.0275
sll0443	GO:0060255	<1e-4	sll0863	GO:0051818	<1e-4	sll1318	GO:0005342	<1e-4	sll0191	GO:0044425	<1e-4	slr0587	GO:0005975	0.0275
sll0670	GO:0009126	<1e-4	sll1414	GO:0006721	<1e-4	sll1380	GO:0016817	<1e-4	slr0496	GO:0005575	<1e-4	ssr6078	GO:0005975	0.0275
slr1053	GO:0034660	<1e-4	sll1352	GO:0044003	<1e-4	sll1464	GO:0022832	<1e-4	slr1679	GO:0044464	<1e-4	sll7067	GO:0005975	0.0275
sll6053	GO:0046164	<1e-4	slr2080	GO:0065007	<1e-4	slr1767	GO:0015293	<1e-4	sll0487	GO:0044424	<1e-4	ssr2553	GO:0005975	0.0275
sll0172	GO:0009892	<1e-4	slr1071	GO:0048522	<1e-4	slr1342	GO:0008171	<1e-4	slr0407	GO:0044444	<1e-4	ssr8047	GO:0005975	0.0275
slr2052	GO:0009987	<1e-4	sll1735	GO:0051188	<1e-4	sll0939	GO:0015399	<1e-4	slr1970	GO:0043226	<1e-4	slr0326	GO:0005975	0.0275
slr1195	GO:0065007	<1e-4	slr1648	GO:0009117	<1e-4	slr0272	GO:0022891	<1e-4	slr1978	GO:0044425	<1e-4	sll1717	GO:0005975	0.0275
sll1659	GO:0046164	<1e-4	sll1696	GO:0009892	<1e-4	slr1674	GO:0003674	<1e-4	slr2000	GO:0044422	<1e-4	ssr3570	GO:0005975	0.0275
sll1906	GO:0044282	<1e-4	slr1847	GO:0016053	<1e-4	slr1270	GO:0022891	<1e-4	ssr5074	GO:0043226	<1e-4	ssl1046	GO:0005975	0.0275
slr0317	GO:0042455	<1e-4	ssl3379	GO:0019752	<1e-4	sll5089	GO:0022890	<1e-4	sll0944	GO:0044446	<1e-4	slr1474	GO:0005975	0.0275
sll0863	GO:0071840	<1e-4	slr5102	GO:0009987	<1e-4	sll1511	GO:0015075	<1e-4	slr0579	GO:0005575	<1e-4	slr1327	GO:0005975	0.0275
sll1630	GO:0009259	<1e-4	slr1081	GO:0009123	<1e-4	slr6087	GO:0042623	<1e-4	ssl8028	GO:0044446	<1e-4	slr0269	GO:0005975	0.0275
sll0424	GO:0031323	<1e-4	slr1667	GO:0019752	<1e-4	slr1436	GO:0008171	<1e-4	ssr3410	GO:0044446	<1e-4	slr1438	GO:0005975	0.0275
slr0978	GO:0016054	<1e-4	ssr2318	GO:0009262	<1e-4	slr0294	GO:0022857	<1e-4	sll0481	GO:0044424	<1e-4	slr1577	GO:0005975	0.0275
sll0756	GO:0019362	<1e-4	slr2005	GO:0031326	<1e-4	slr6063	GO:0015399	<1e-4	ssl3615	GO:0044464	<1e-4	slr1170	GO:0005975	0.0275
slr0503	GO:0051188	<1e-4	slr1083	GO:0051716	<1e-4	slr0483	GO:0008171	<1e-4	slr1407	GO:0043226	<1e-4	sll1319	GO:0005975	0.0275
sll0762	GO:0009991	<1e-4	slr1753	GO:0044248	<1e-4	slr6067	GO:0015267	<1e-4	slr1618	GO:0044422	<1e-4	ssr1558	GO:0005975	0.0275

slr0975	GO:0019751	<1e-4	sll0545	GO:0006811	<1e-4	ssr2787	GO:0015291	<1e-4	slr1384	GO:0043226	<1e-4	ssr0336	GO:0005975	0.0275
sll0596	GO:0051347	<1e-4	slr0981	GO:0043170	<1e-4	slr1084	GO:0022892	<1e-4	slr1174	GO:0044422	<1e-4	ssr1425	GO:0005975	0.0275
sll0280	GO:0043648	<1e-4	slr0397	GO:0009117	<1e-4	sll0783	GO:0015077	<1e-4	slr1047	GO:0044464	<1e-4	slr5112	GO:0005975	0.0275
slr0885	GO:0050896	<1e-4	sll1464	GO:0034660	<1e-4	sll0911	GO:0022832	<1e-4	sll1166	GO:0044444	<1e-4	slr0271	GO:0005975	0.0275
sll1472	GO:0044106	<1e-4	ssr1041	GO:0009987	<1e-4	slr5024	GO:0003674	<1e-4	slr5053	GO:0044444	<1e-4	ssl2971	GO:0005975	0.0275
slr0869	GO:0009142	<1e-4	sll0994	GO:0044282	<1e-4	sll1586	GO:0022803	<1e-4	sll5067	GO:0044424	<1e-4	sll1926	GO:0005975	0.0275
ssl1464	GO:0009987	<1e-4	slr6029	GO:0016053	<1e-4	ssr1765	GO:0043167	<1e-4	slr0039	GO:0043226	<1e-4	sll0101	GO:0005975	0.0275
sll0355	GO:0006753	<1e-4	slr1260	GO:0034641	<1e-4	slr1425	GO:0022803	<1e-4	sll1132	GO:0044422	<1e-4	slr1052	GO:0004871	0.0271
slr0300	GO:0046483	<1e-4	sll1109	GO:0072522	<1e-4	sll0858	GO:0003674	<1e-4	ssl7021	GO:0044446	<1e-4	sll1061	GO:0016820	0.0269
slr1437	GO:0006066	<1e-4	slr1690	GO:0055114	<1e-4	slr1493	GO:0042623	<1e-4	slr1033	GO:0043226	<1e-4	sll1262	GO:0016820	0.0269
slr1914	GO:0019222	<1e-4	sll7090	GO:0055086	<1e-4	ssr3570	GO:0060089	<1e-4	sll0923	GO:0044444	<1e-4	sll0243	GO:0016820	0.0269
sll1526	GO:0051171	<1e-4	slr0483	GO:0009161	<1e-4	slr0209	GO:0016818	<1e-4	sll5132	GO:0005575	<1e-4	slr1667	GO:0016820	0.0269
slr1116	GO:0009308	<1e-4	slr0967	GO:0072521	<1e-4	sll1832	GO:0015077	<1e-4	slr2080	GO:0044424	<1e-4	sll1736	GO:0016820	0.0269
slr0586	GO:0080090	<1e-4	sll0691	GO:0031640	<1e-4	slr1573	GO:0015267	<1e-4	sll0871	GO:0044464	<1e-4	slr0668	GO:0016820	0.0269
slr1789	GO:0072524	<1e-4	ssr3467	GO:0009057	<1e-4	sll2011	GO:0015293	<1e-4	slr1032	GO:0005575	<1e-4	slr0204	GO:0016820	0.0269
ssr1258	GO:0009308	<1e-4	ssr5121	GO:0022607	<1e-4	slr1223	GO:0043167	<1e-4	slr6028	GO:0044424	<1e-4	sll1530	GO:0016820	0.0269
slr5101	GO:0009394	<1e-4	slr0217	GO:0065007	<1e-4	slr1565	GO:0015077	<1e-4	ssr6030	GO:0043226	<1e-4	ssr3122	GO:0016820	0.0269
ssr7017	GO:0072521	<1e-4	slr0053	GO:0006732	<1e-4	slr1338	GO:0008171	<1e-4	ssr6030	GO:0044424	<1e-4	slr1073	GO:0016820	0.0269
slr5112	GO:0090304	<1e-4	ssr2422	GO:0043549	<1e-4	sll1319	GO:0005342	<1e-4	slr0964	GO:0044424	<1e-4	slr0294	GO:0016820	0.0269
sll1915	GO:0010468	<1e-4	slr1070	GO:0050794	<1e-4	sll0282	GO:0016817	<1e-4	sll1773	GO:0044424	<1e-4	slr2111	GO:0016820	0.0269
sll7087	GO:0050794	<1e-4	slr1034	GO:0006811	<1e-4	slr1863	GO:0015267	<1e-4	sll0497	GO:0044464	<1e-4	slr1081	GO:0016820	0.0269
slr1819	GO:0055082	<1e-4	sll5067	GO:0009124	<1e-4	slr0642	GO:0022892	<1e-4	sll1321	GO:0005575	<1e-4	slr1648	GO:0016820	0.0269
sll0335	GO:0048522	<1e-4	sll0381	GO:0046164	<1e-4	slr5118	GO:0016817	<1e-4	sll2006	GO:0044424	<1e-4	sll0355	GO:0016820	0.0269
slr0243	GO:0010556	<1e-4	sll0508	GO:0006721	<1e-4	sll0242	GO:0022832	<1e-4	sll7062	GO:0005575	<1e-4	slr0913	GO:0016820	0.0269
ssl3383	GO:0034654	<1e-4	sll0524	GO:0051234	<1e-4	sll0981	GO:0022832	<1e-4	sll0911	GO:0044425	<1e-4	slr2122	GO:0016820	0.0269
sll1319	GO:0006066	<1e-4	slr0103	GO:0019637	<1e-4	sll1785	GO:0015293	<1e-4	sll0909	GO:0044446	<1e-4	sll0010	GO:0016820	0.0269
slr0149	GO:0050794	<1e-4	slr1438	GO:0009262	<1e-4	slr6015	GO:0015291	<1e-4	slr0702	GO:0044424	<1e-4	ssl2807	GO:0016820	0.0269
slr2052	GO:0043170	<1e-4	sll1022	GO:0072524	<1e-4	slr1907	GO:0022832	<1e-4	ssr5092	GO:0044446	<1e-4	slr1880	GO:0016820	0.0269
ssl1417	GO:0042180	<1e-4	sll1218	GO:0051716	<1e-4	ssr0332	GO:0016818	<1e-4	slr1472	GO:0044422	<1e-4	ssr2439	GO:0016820	0.0269
sll0369	GO:0009117	<1e-4	slr1590	GO:0043549	<1e-4	sll0456	GO:0043492	<1e-4	ssr2711	GO:0044425	<1e-4	slr1913	GO:0016820	0.0269
sll0645	GO:0065007	<1e-4	sll1025	GO:0034641	<1e-4	slr1287	GO:0016746	<1e-4	sll8040	GO:0044422	<1e-4	slr1230	GO:0016820	0.0269
sll0732	GO:0043549	<1e-4	ssr1951	GO:0071841	<1e-4	sll0149	GO:0016874	<1e-4	sll0678	GO:0043226	<1e-4	slr0023	GO:0016820	0.0269
sll0843	GO:0051171	<1e-4	slr2011	GO:0006793	<1e-4	slr0038	GO:0015077	<1e-4	slr0967	GO:0044464	<1e-4	ssr2615	GO:0016820	0.0269
slr1944	GO:0051179	<1e-4	sll8027	GO:0006082	<1e-4	sll0564	GO:0015267	<1e-4	slr1391	GO:0044425	<1e-4	slr1999	GO:0016820	0.0269
slr1914	GO:0009889	<1e-4	sll0149	GO:0006091	<1e-4	sll0737	GO:0022890	<1e-4	ssl5129	GO:0044422	<1e-4	slr0364	GO:0016820	0.0269
ssr3402	GO:0042455	<1e-4	slr0780	GO:0009142	<1e-4	sll1054	GO:0060089	<1e-4	sll0995	GO:0044422	<1e-4	sll0325	GO:0016820	0.0269
slr6066	GO:0060255	<1e-4	slr7014	GO:0031326	<1e-4	sll1239	GO:0003674	<1e-4	sll1632	GO:0044464	<1e-4	sll0944	GO:0016820	0.0269
ssl2069	GO:0001932	<1e-4	ssr1552	GO:0019752	<1e-4	slr1900	GO:0022836	<1e-4	sll0847	GO:0005575	<1e-4	sll1942	GO:0016772	0.0268
sll1726	GO:0055082	<1e-4	slr0241	GO:0055114	<1e-4	slr0590	GO:0016817	<1e-4	ssr3409	GO:0044422	<1e-4	slr1023	GO:0016772	0.0268
ssl2733	GO:0048518	<1e-4	slr0038	GO:0006576	<1e-4	ssl0739	GO:0015075	<1e-4	slr1919	GO:0044422	<1e-4	slr1045	GO:0016772	0.0268
slr6074	GO:0009141	<1e-4	sll0269	GO:0006733	<1e-4	slr0742	GO:0022857	<1e-4	slr1789	GO:0044425	<1e-4	slr1391	GO:0016772	0.0268
sll1250	GO:0005996	<1e-4	slr1186	GO:0046907	<1e-4	slr6067	GO:0042623	<1e-4	slr0111	GO:0044446	<1e-4	slr1441	GO:0016772	0.0268
sll0584	GO:0010556	<1e-4	sll0096	GO:0008150	<1e-4	slr0609	GO:0008171	<1e-4	ssl8039	GO:0043226	<1e-4	sll0096	GO:0016772	0.0268
ssl1923	GO:0009394	<1e-4	sll1164	GO:0005996	<1e-4	slr6067	GO:0043167	<1e-4	slr1863	GO:0044444	<1e-4	slr0211	GO:0016772	0.0268
ssr0759	GO:0048522	<1e-4	sll0602	GO:0006811	<1e-4	ssl0242	GO:0022803	<1e-4	slr0407	GO:0043226	<1e-4	slr1398	GO:0016772	0.0268
slr0058	GO:0045184	<1e-4	sll0735	GO:0009144	<1e-4	sll0266	GO:0042623	<1e-4	slr1939	GO:0044422	<1e-4	slr0937	GO:0016772	0.0268
slr1702	GO:0009126	<1e-4	sll5128	GO:0046128	<1e-4	slr1391	GO:0022838	<1e-4	slr0333	GO:0044446	<1e-4	slr0871	GO:0016772	0.0268
slr0587	GO:0046128	<1e-4	sll5090	GO:0006766	<1e-4	sll1401	GO:0043492	<1e-4	sll0361	GO:0005575	<1e-4	sll1433	GO:0016772	0.0268
slr0516	GO:0019219	<1e-4	sll1526	GO:0009141	<1e-4	sll8040	GO:0043167	<1e-4	ssl1690	GO:0005575	<1e-4	sll1218	GO:0016772	0.0268
slr0263	GO:0090304	<1e-4	slr0013	GO:0046164	<1e-4	sll0381	GO:0016817	<1e-4	sll0815	GO:0044464	<1e-4	sll0513	GO:0016772	0.0268
sll0101	GO:0051246	<1e-4	slr7026	GO:0051234	<1e-4	slr0645	GO:0016741	<1e-4	ssl5095	GO:0044424	<1e-4	sll1472	GO:0016772	0.0268
sll1638	GO:0055114	<1e-4	slr1847	GO:0006576	<1e-4	sll1609	GO:0005342	<1e-4	ssl5025	GO:0044424	<1e-4	ssr1698	GO:0016772	0.0268
ssr1552	GO:0055086	<1e-4	slr7082	GO:0006811	<1e-4	sll1766	GO:0015267	<1e-4	sll7029	GO:0044444	<1e-4	slr1900	GO:0016772	0.0268
ssr5019	GO:0034660	<1e-4	sll0911	GO:0071554	<1e-4	sll0298	GO:0016462	<1e-4	slr0708	GO:0005575	<1e-4	sll1135	GO:0016772	0.0268

sll1192	GO:0009142	<1e-4	sll0843	GO:0008150	<1e-4	sll1040	GO:0016818	<1e-4	sll1906	GO:0044446	<1e-4	sll0854	GO:0016772	0.0268
slr2101	GO:0019637	<1e-4	slr2121	GO:0046128	<1e-4	slr1807	GO:0015291	<1e-4	ssl8005	GO:0044424	<1e-4	slr1505	GO:0016772	0.0268
sll1201	GO:0042455	<1e-4	sll0910	GO:0009893	<1e-4	slr7012	GO:0060089	<1e-4	ssl3451	GO:0044425	<1e-4	slr0852	GO:0016772	0.0268
sll1390	GO:0090304	<1e-4	sll1321	GO:0006576	<1e-4	sll1940	GO:0022890	<1e-4	sll1921	GO:0044425	<1e-4	sll1715	GO:0016772	0.0268
slr1660	GO:0072522	<1e-4	slr0845	GO:0006733	<1e-4	ssl1046	GO:0015291	<1e-4	ssl6035	GO:0044446	<1e-4	slr0545	GO:0016772	0.0268
sll1024	GO:0001932	<1e-4	slr1875	GO:0005996	<1e-4	sll1344	GO:0022857	<1e-4	slr0147	GO:0043226	<1e-4	ssl3692	GO:0016772	0.0268
slr2080	GO:0046394	<1e-4	slr0208	GO:0071496	<1e-4	slr0076	GO:0015399	<1e-4	sll0382	GO:0044424	<1e-4	slr0209	GO:0016772	0.0268
ssl0738	GO:0008610	<1e-4	slr0250	GO:0019219	<1e-4	sll0602	GO:0043167	<1e-4	slr0263	GO:0044446	<1e-4	slr1083	GO:0016772	0.0268
slr6103	GO:0009126	<1e-4	slr0400	GO:0046128	<1e-4	sll2011	GO:0022892	<1e-4	slr7058	GO:0044425	<1e-4	ssl1255	GO:0005975	0.0267
slr0606	GO:0018130	<1e-4	sll0614	GO:0051188	<1e-4	ssr1698	GO:0015077	<1e-4	sll0761	GO:0044425	<1e-4	slr0810	GO:0005975	0.0267
sll1025	GO:0050794	<1e-4	sll1217	GO:0019222	<1e-4	ssr1558	GO:0016462	<1e-4	sll0787	GO:0044464	<1e-4	ssr2843	GO:0005975	0.0267
slr0060	GO:0044003	<1e-4	sll0449	GO:0046907	<1e-4	ssr2848	GO:0043167	<1e-4	sll0910	GO:0044424	<1e-4	sll1609	GO:0008168	0.0261
slr1116	GO:0055114	<1e-4	sll0735	GO:0051171	<1e-4	sll0376	GO:0003674	<1e-4	slr0058	GO:0044446	<1e-4	sll1188	GO:0008168	0.0261
slr0815	GO:0044255	<1e-4	slr0813	GO:0051179	<1e-4	ssr2972	GO:0022892	<1e-4	sll1913	GO:0005575	<1e-4	sll1532	GO:0008168	0.0261
sll1442	GO:0051246	<1e-4	sll1775	GO:0009056	<1e-4	slr2010	GO:0022803	<1e-4	slr1819	GO:0044444	<1e-4	slr0976	GO:0008168	0.0261
ssr2009	GO:0046394	<1e-4	ssl7046	GO:0009056	<1e-4	sll5034	GO:0015291	<1e-4	slr7013	GO:0044446	<1e-4	ssr6086	GO:0008168	0.0261
sll0047	GO:0006220	<1e-4	slr6104	GO:0009394	<1e-4	ssr5092	GO:0015267	<1e-4	slr1161	GO:0044422	<1e-4	ssl7051	GO:0008168	0.0261
slr6005	GO:0042451	<1e-4	sll1252	GO:0008150	<1e-4	ssl7074	GO:0015075	<1e-4	slr0317	GO:0044425	<1e-4	slr0271	GO:0008168	0.0261
slr1474	GO:0006733	<1e-4	slr0642	GO:0009056	<1e-4	sll0843	GO:0022892	<1e-4	slr1814	GO:0043226	<1e-4	sll8001	GO:0008168	0.0261
sll1906	GO:0044106	<1e-4	ssl2733	GO:0043436	<1e-4	sll0350	GO:0043492	<1e-4	sll1174	GO:0005575	<1e-4	slr0039	GO:0008168	0.0261
slr1045	GO:0006793	<1e-4	slr1674	GO:0055086	<1e-4	sll1265	GO:0016746	<1e-4	slr1940	GO:0044464	<1e-4	sll0335	GO:0008168	0.0261
ssl1300	GO:0048878	<1e-4	sll5089	GO:0016054	<1e-4	slr0551	GO:0015405	<1e-4	slr1187	GO:0005575	<1e-4	slr0397	GO:0008168	0.0261
sll1954	GO:0055082	<1e-4	sll5003	GO:0006811	<1e-4	slr1170	GO:0016818	<1e-4	slr0852	GO:0044446	<1e-4	sll7069	GO:0008168	0.0261
slr1203	GO:0006576	<1e-4	ssl2733	GO:0006631	<1e-4	sll1319	GO:0022803	<1e-4	slr1062	GO:0005575	<1e-4	sll1979	GO:0008168	0.0261
slr1819	GO:0071841	<1e-4	slr0586	GO:0009199	<1e-4	sll1950	GO:0015293	<1e-4	ssl1045	GO:0044424	<1e-4	slr6106	GO:0008168	0.0261
slr7100	GO:0043549	<1e-4	slr1647	GO:0051186	<1e-4	sll1717	GO:0043167	<1e-4	slr1544	GO:0044424	<1e-4	ssl7038	GO:0008168	0.0261
sll0810	GO:0080090	<1e-4	slr1303	GO:0031640	<1e-4	ssl7007	GO:0016817	<1e-4	slr0598	GO:0044444	<1e-4	slr0285	GO:0008168	0.0261
sll0216	GO:0071496	<1e-4	sll0327	GO:0006091	<1e-4	ssl1690	GO:0016462	<1e-4	ssl5103	GO:0005575	<1e-4	ssl7007	GO:0008168	0.0261
slr1230	GO:0009889	<1e-4	sll1608	GO:0072527	<1e-4	slr5018	GO:0022857	<1e-4	slr1788	GO:0044446	<1e-4	slr6009	GO:0008168	0.0261
sll0732	GO:0009124	<1e-4	slr1116	GO:0016054	<1e-4	slr0871	GO:0005342	<1e-4	slr1807	GO:0043226	<1e-4	ssr2711	GO:0008168	0.0261
slr0013	GO:0090304	<1e-4	sll1715	GO:0006732	<1e-4	slr0871	GO:0022836	<1e-4	sll7077	GO:0044464	<1e-4	slr0270	GO:0008168	0.0261
sll1340	GO:0009141	<1e-4	slr2070	GO:0006091	<1e-4	slr6033	GO:0015075	<1e-4	slr1184	GO:0044424	<1e-4	sll7050	GO:0008168	0.0261
slr0650	GO:0006721	<1e-4	slr1033	GO:0031323	<1e-4	slr7060	GO:0015291	<1e-4	slr1679	GO:0005575	<1e-4	ssl2064	GO:0008168	0.0261
sll0101	GO:0006220	<1e-4	ssr0336	GO:0009889	<1e-4	slr7059	GO:0015399	<1e-4	ssr2333	GO:0044464	<1e-4	ssr5106	GO:0008168	0.0261
sll1785	GO:0042451	<1e-4	slr2018	GO:0031640	<1e-4	slr5013	GO:0015291	<1e-4	slr6007	GO:0044464	<1e-4	sll1381	GO:0008168	0.0261
slr1789	GO:0031323	<1e-4	ssl8003	GO:0006220	<1e-4	slr0318	GO:0015077	<1e-4	slr2003	GO:0044422	<1e-4	sll0101	GO:0008168	0.0261
slr0516	GO:0051246	<1e-4	slr0476	GO:0080090	<1e-4	sll0886	GO:0022891	<1e-4	slr0712	GO:0005575	<1e-4	slr6029	GO:0008168	0.0261
sll0024	GO:0009057	<1e-4	sll2015	GO:0009199	<1e-4	sll1061	GO:0015399	<1e-4	sll0647	GO:0043226	<1e-4	ssr6002	GO:0008168	0.0261
slr1990	GO:0009059	<1e-4	slr0496	GO:0006793	<1e-4	ssr7017	GO:0003674	<1e-4	sll0563	GO:0044425	<1e-4	sll5067	GO:0008168	0.0261
sll0372	GO:0009262	<1e-4	sll0543	GO:0046483	<1e-4	sll0443	GO:0005342	<1e-4	sll0237	GO:0044446	<1e-4	slr1627	GO:0008168	0.0261
slr0517	GO:0009394	<1e-4	ssl7048	GO:0034641	<1e-4	sll1911	GO:0022891	<1e-4	slr0393	GO:0043226	<1e-4	sll1024	GO:0008168	0.0261
sll1773	GO:0006721	<1e-4	sll1188	GO:0072521	<1e-4	slr1441	GO:0015075	<1e-4	slr1854	GO:0044422	<1e-4	slr0416	GO:0008168	0.0261
sll1542	GO:0006576	<1e-4	sll1586	GO:0031326	<1e-4	sll0010	GO:0022832	<1e-4	sll8035	GO:0044424	<1e-4	sll5132	GO:0008168	0.0261
sll0487	GO:0043412	<1e-4	ssr2009	GO:0048519	<1e-4	sll0410	GO:0003674	<1e-4	sll7043	GO:0044424	<1e-4	ssl5100	GO:0008168	0.0261
ssl0242	GO:0072521	<1e-4	slr0059	GO:0019219	<1e-4	slr6022	GO:0016818	<1e-4	sll0265	GO:0044424	<1e-4	slr5053	GO:0008168	0.0261
slr1577	GO:0072522	<1e-4	slr1702	GO:0019637	<1e-4	slr0104	GO:0015077	<1e-4	ssl5129	GO:0005575	<1e-4	sll1714	GO:0008168	0.0261
ssr1499	GO:0009394	<1e-4	sll1906	GO:0009260	<1e-4	slr1484	GO:0015293	<1e-4	sll1372	GO:0044422	<1e-4	ssl2162	GO:0008168	0.0261
slr1450	GO:0051818	<1e-4	slr1079	GO:0044282	<1e-4	sll1132	GO:0022836	<1e-4	slr0545	GO:0044425	<1e-4	slr1079	GO:0008168	0.0261
sll0552	GO:0001932	<1e-4	slr1189	GO:0008150	<1e-4	slr1186	GO:0008171	<1e-4	sll1858	GO:0044422	<1e-4	sll1321	GO:0008168	0.0261
sll0513	GO:0055114	<1e-4	sll1024	GO:0034654	<1e-4	sll0588	GO:0043167	<1e-4	slr1648	GO:0044424	<1e-4	sll8019	GO:0008168	0.0261
slr1276	GO:0006766	<1e-4	slr7026	GO:0046394	<1e-4	slr0270	GO:0015399	<1e-4	slr0172	GO:0044446	<1e-4	slr1670	GO:0008168	0.0261
slr1215	GO:0034654	<1e-4	slr0730	GO:0051716	<1e-4	slr1624	GO:0016817	<1e-4	slr6013	GO:0044464	<1e-4	ssl5015	GO:0008168	0.0261
sll1730	GO:0034654	<1e-4	slr0479	GO:0009059	<1e-4	slr0476	GO:0016741	<1e-4	slr0468	GO:0044422	<1e-4	ssl8039	GO:0008168	0.0261
sgl0001	GO:0009165	<1e-4	slr1999	GO:0043436	<1e-4	slr1799	GO:0015075	<1e-4	sll0456	GO:0043226	<1e-4	sll0984	GO:0008168	0.0261

slr0408	GO:0009165	<1e-4	slr6088	GO:0018130	<1e-4	slr1079	GO:0022803	<1e-4	slr0935	GO:0005575	<1e-4	slr6022	GO:0008168	0.0261
slr0104	GO:0055086	<1e-4	sll7033	GO:0009259	<1e-4	sll0048	GO:0005342	<1e-4	sll1542	GO:0044425	<1e-4	sll0736	GO:0008168	0.0261
sll1680	GO:0043170	<1e-4	ssr3189	GO:0006811	<1e-4	sll1562	GO:0016741	<1e-4	slr1074	GO:0044444	<1e-4	sll0563	GO:0008168	0.0261
slr1083	GO:0071496	<1e-4	slr1056	GO:0031640	<1e-4	sll0350	GO:0016817	<1e-4	slr2049	GO:0044446	<1e-4	slr1704	GO:0008168	0.0261
slr1658	GO:0048522	<1e-4	sll0101	GO:0043412	<1e-4	slr1203	GO:0015291	<1e-4	sll1638	GO:0044444	<1e-4	ssr2975	GO:0008168	0.0261
slr5087	GO:0072522	<1e-4	slr1053	GO:0009892	<1e-4	sll0780	GO:0015293	<1e-4	slr0692	GO:0044444	<1e-4	slr0498	GO:0008168	0.0261
sll0497	GO:0046164	<1e-4	slr1415	GO:0009199	<1e-4	slr1258	GO:0016741	<1e-4	slr0407	GO:0005575	<1e-4	slr7071	GO:0008168	0.0261
sll0263	GO:0048878	<1e-4	sll0595	GO:0019219	<1e-4	sll5097	GO:0042623	<1e-4	sll1751	GO:0044422	<1e-4	sll1191	GO:0008168	0.0261
slr1778	GO:0044003	<1e-4	slr0111	GO:0006091	<1e-4	sll1570	GO:0015291	<1e-4	slr1854	GO:0044444	<1e-4	sll1495	GO:0008168	0.0261
sll1797	GO:0031323	<1e-4	sll7070	GO:0001932	<1e-4	slr0491	GO:0022832	<1e-4	sll0406	GO:0005575	<1e-4	sll7086	GO:0008168	0.0261
ssl2138	GO:0009057	<1e-4	slr2110	GO:0006811	<1e-4	ssl5129	GO:0022892	<1e-4	slr0169	GO:0005575	<1e-4	ssr6099	GO:0008168	0.0261
sll0780	GO:0044248	<1e-4	slr5053	GO:0006753	<1e-4	ssr3122	GO:0016818	<1e-4	slr1800	GO:0044464	<1e-4	slr5087	GO:0008168	0.0261
sll0060	GO:0050789	<1e-4	slr1493	GO:0051716	<1e-4	sll0886	GO:0005342	<1e-4	slr1258	GO:0044444	<1e-4	ssl1255	GO:0016740	0.0261
slr0455	GO:0009117	<1e-4	sll0198	GO:0051188	<1e-4	sll8027	GO:0016746	<1e-4	slr0325	GO:0005575	<1e-4	sll1095	GO:0008168	0.0261
ssl1520	GO:0051186	<1e-4	sll0022	GO:0051179	<1e-4	sll1477	GO:0016818	<1e-4	sll1562	GO:0005575	<1e-4	slr0625	GO:0008168	0.0261
ssr2553	GO:0009199	<1e-4	slr0755	GO:0071496	<1e-4	slr1163	GO:0022838	<1e-4	slr2070	GO:0005575	<1e-4	slr8022	GO:0008168	0.0261
slr1266	GO:0043436	<1e-4	ssl2595	GO:0043436	<1e-4	slr1875	GO:0016462	<1e-4	sll0645	GO:0043226	<1e-4	ssl1918	GO:0008168	0.0261
sll7066	GO:0051234	<1e-4	slr0609	GO:0009142	<1e-4	slr1315	GO:0015405	<1e-4	slr1612	GO:0043226	<1e-4	sll1785	GO:0008168	0.0261
sll0585	GO:0080090	<1e-4	ssl1498	GO:0051347	<1e-4	sll7089	GO:0016746	<1e-4	slr1813	GO:0043226	<1e-4	ssl5068	GO:0008168	0.0261
sll0775	GO:0044255	<1e-4	slr1885	GO:0006220	<1e-4	slr6103	GO:0015077	<1e-4	slr0990	GO:0044464	<1e-4	slr1056	GO:0008168	0.0261
sll1764	GO:0031323	<1e-4	slr0941	GO:0048519	<1e-4	slr1397	GO:0022890	<1e-4	sll0188	GO:0044446	<1e-4	ssr6027	GO:0008168	0.0261
slr1062	GO:0006576	<1e-4	slr1052	GO:0071841	<1e-4	ssl1972	GO:0008171	<1e-4	slr1541	GO:0005575	<1e-4	ssr6030	GO:0008168	0.0261
sll1380	GO:0022607	<1e-4	slr1721	GO:0042180	<1e-4	slr1062	GO:0003674	<1e-4	sll0175	GO:0044425	<1e-4	ssl0410	GO:0008168	0.0261
ssr2317	GO:0009161	<1e-4	slr0668	GO:0048518	<1e-4	ssl3549	GO:0008171	<1e-4	slr1415	GO:0043226	<1e-4	slr7099	GO:0008168	0.0261
ssl8003	GO:0051186	<1e-4	slr0479	GO:0072527	<1e-4	ssl0467	GO:0015267	<1e-4	sll1608	GO:0044424	<1e-4	sll1698	GO:0008168	0.0261
slr1353	GO:0051234	<1e-4	slr6033	GO:0006091	<1e-4	sll0775	GO:0015293	<1e-4	ssl2595	GO:0043226	<1e-4	sll0525	GO:0008168	0.0261
ssr2553	GO:0051716	<1e-4	ssl0483	GO:0009260	<1e-4	ssl5065	GO:0043167	<1e-4	sll0359	GO:0044422	<1e-4	slr7073	GO:0008168	0.0261
sll1853	GO:0019219	<1e-4	slr6021	GO:0009132	<1e-4	sll0098	GO:0022838	<1e-4	sll0397	GO:0044446	<1e-4	slr0651	GO:0008168	0.0261
ssr2754	GO:0034654	<1e-4	slr1195	GO:0080090	<1e-4	slr1614	GO:0016741	<1e-4	slr5037	GO:0043226	<1e-4	sll1052	GO:0008168	0.0261
sll0864	GO:0051818	<1e-4	sll1763	GO:0009987	<1e-4	slr0325	GO:0016746	<1e-4	slr1677	GO:0044425	<1e-4	slr6081	GO:0008168	0.0261
slr1866	GO:0009889	<1e-4	slr0109	GO:0009117	<1e-4	sll7065	GO:0016746	<1e-4	sll7064	GO:0044444	<1e-4	sll0442	GO:0008168	0.0261
slr1052	GO:0018130	<1e-4	slr0151	GO:0009199	<1e-4	slr1513	GO:0015293	<1e-4	slr1906	GO:0044444	<1e-4	ssr0759	GO:0008168	0.0261
sll0174	GO:0072521	<1e-4	ssr0335	GO:0071554	<1e-4	ssr2711	GO:0016746	<1e-4	slr1186	GO:0044425	<1e-4	sll0060	GO:0008168	0.0261
sll0397	GO:0046483	<1e-4	sll1318	GO:0006082	<1e-4	sll0405	GO:0015399	<1e-4	slr0645	GO:0044444	<1e-4	slr5073	GO:0008168	0.0261
slr0554	GO:0006163	<1e-4	slr1789	GO:0006082	<1e-4	ssl7039	GO:0016462	<1e-4	slr0695	GO:0005575	<1e-4	ssr8013	GO:0008168	0.0261
sll1086	GO:0019222	<1e-4	slr1052	GO:0043933	<1e-4	slr1546	GO:0015077	<1e-4	ssr2998	GO:0005575	<1e-4	sll1233	GO:0008168	0.0261
slr6022	GO:0006082	<1e-4	sll7087	GO:0080090	<1e-4	sll0359	GO:0022890	<1e-4	sll0350	GO:0044422	<1e-4	sll1737	GO:0016740	0.0261
slr1519	GO:0009056	<1e-4	sgl0001	GO:0046128	<1e-4	slr0765	GO:0042623	<1e-4	slr1816	GO:0044444	<1e-4	ssr7079	GO:0008168	0.0261
ssl0294	GO:0051818	<1e-4	sll1714	GO:0043549	<1e-4	ssl3573	GO:0015405	<1e-4	slr0921	GO:0044424	<1e-4	slr0708	GO:0008168	0.0261
slr1940	GO:0051171	<1e-4	slr8021	GO:0042180	<1e-4	slr0789	GO:0015293	<1e-4	ssl5099	GO:0044422	<1e-4	slr5111	GO:0008168	0.0261
sll0670	GO:0048522	<1e-4	sll8032	GO:0009889	<1e-4	slr1557	GO:0015399	<1e-4	sll8007	GO:0005575	<1e-4	sll5128	GO:0008168	0.0261
sll0524	GO:0019362	<1e-4	slr7092	GO:0008610	<1e-4	sll0436	GO:0016817	<1e-4	sll1092	GO:0043226	<1e-4	slr1236	GO:0008168	0.0261
slr6064	GO:0009394	<1e-4	ssr1391	GO:0052188	<1e-4	ssl7051	GO:0022836	<1e-4	ssl5096	GO:0005575	<1e-4	slr1944	GO:0008168	0.0261
sll0286	GO:0042451	<1e-4	ssl0739	GO:0019219	<1e-4	slr7083	GO:0043492	<1e-4	sll1415	GO:0044422	<1e-4	slr0813	GO:0008168	0.0261
slr6014	GO:0016053	<1e-4	slr1273	GO:0009059	<1e-4	slr7098	GO:0022892	<1e-4	slr1196	GO:0043226	<1e-4	sll1106	GO:0008168	0.0261
slr0651	GO:0071841	<1e-4	sll0867	GO:0042180	<1e-4	slr2105	GO:0016817	<1e-4	sll5003	GO:0044422	<1e-4	sll0839	GO:0008168	0.0261
slr1613	GO:0055086	<1e-4	ssr2142	GO:0031640	<1e-4	slr1780	GO:0022836	<1e-4	ssr1528	GO:0005575	<1e-4	slr5037	GO:0008168	0.0261
slr1600	GO:0046164	<1e-4	ssl0242	GO:0009142	<1e-4	slr0195	GO:0015267	<1e-4	slr7096	GO:0044464	<1e-4	slr6044	GO:0008168	0.0261
slr7092	GO:0080090	<1e-4	sll5109	GO:0009117	<1e-4	slr1535	GO:0022891	<1e-4	sll1698	GO:0043226	<1e-4	sll1702	GO:0008168	0.0261
ssl2996	GO:0051234	<1e-4	ssl1464	GO:0046128	<1e-4	ssl2733	GO:0043167	<1e-4	sll0286	GO:0044446	<1e-4	slr1819	GO:0008168	0.0261
slr1032	GO:0019219	<1e-4	ssl1004	GO:0034660	<1e-4	slr6073	GO:0008171	<1e-4	ssr1256	GO:0043226	<1e-4	slr0914	GO:0008168	0.0261
sll1254	GO:0019219	<1e-4	slr1174	GO:0009165	<1e-4	ssl3291	GO:0016741	<1e-4	sll1960	GO:0005575	<1e-4	ssr7084	GO:0008168	0.0261
slr1619	GO:0009141	<1e-4	sll0319	GO:0043436	<1e-4	ssl7051	GO:0003674	<1e-4	slr0787	GO:0044422	<1e-4	slr0689	GO:0008168	0.0261
slr0058	GO:0044283	<1e-4	sll7064	GO:0044282	<1e-4	slr0479	GO:0016741	<1e-4	sll1702	GO:0044424	<1e-4	sll7034	GO:0008168	0.0261

sll0858	GO:0052111	<1e-4	slr0318	GO:0050794	<1e-4	slr1070	GO:0016741	<1e-4	slr7014	GO:0044424	<1e-4	sll1192	GO:0008168	0.0261
sll1373	GO:0006721	<1e-4	slr1307	GO:0009132	<1e-4	slr1778	GO:0043167	<1e-4	slr0592	GO:0044425	<1e-4	slr1623	GO:0008168	0.0261
slr1563	GO:0072521	<1e-4	slr0482	GO:0055114	<1e-4	slr0924	GO:0022838	<1e-4	sll0933	GO:0044424	<1e-4	sll0162	GO:0008168	0.0261
ssl1417	GO:0055082	<1e-4	slr1315	GO:0072522	<1e-4	slr0408	GO:0022838	<1e-4	slr0818	GO:0044424	<1e-4	slr1537	GO:0008168	0.0261
sll0702	GO:0042180	<1e-4	slr1913	GO:0006721	<1e-4	slr0092	GO:0022832	<1e-4	slr1340	GO:0005575	<1e-4	sll1089	GO:0008168	0.0261
ssr5074	GO:0006733	<1e-4	slr1425	GO:0009987	<1e-4	sll1632	GO:0022838	<1e-4	slr0270	GO:0005575	<1e-4	slr1206	GO:0008168	0.0261
slr1241	GO:0008610	<1e-4	sll1654	GO:0001932	<1e-4	sll0822	GO:0022836	<1e-4	slr6087	GO:0044444	<1e-4	sll0252	GO:0017111	0.026
sll1390	GO:0043549	<1e-4	ssl5015	GO:0006066	<1e-4	sll1063	GO:0022832	<1e-4	sll0412	GO:0044422	<1e-4	sll1562	GO:0016772	0.0258
sll1541	GO:0009394	<1e-4	slr2084	GO:0051347	<1e-4	slr0423	GO:0015075	<1e-4	sll0419	GO:0043226	<1e-4	slr1847	GO:0016772	0.0258
slr1262	GO:0009117	<1e-4	slr6004	GO:0009057	<1e-4	sll1632	GO:0003674	<1e-4	slr0913	GO:0005575	<1e-4	slr1851	GO:0016772	0.0258
sll5067	GO:0034641	<1e-4	ssl5064	GO:0043648	<1e-4	sll0737	GO:0015075	<1e-4	sll1942	GO:0044464	<1e-4	sll0558	GO:0016772	0.0258
slr7097	GO:0034654	<1e-4	sll0913	GO:0071840	<1e-4	ssl3379	GO:0005342	<1e-4	sll0696	GO:0043226	<1e-4	slr1301	GO:0016772	0.0258
ssr2755	GO:0044106	<1e-4	slr1174	GO:0071841	<1e-4	sll8027	GO:0015399	<1e-4	slr1533	GO:0044464	<1e-4	slr1811	GO:0016772	0.0258
sll1389	GO:0009199	<1e-4	ssl0750	GO:0006793	<1e-4	sll1433	GO:0008171	<1e-4	slr2046	GO:0044446	<1e-4	sll0783	GO:0016772	0.0258
ssl5099	GO:0044003	<1e-4	ssl5007	GO:0051716	<1e-4	sll1250	GO:0015075	<1e-4	slr1920	GO:0044424	<1e-4	sll0183	GO:0016772	0.0258
sll0060	GO:0006631	<1e-4	slr1034	GO:0044283	<1e-4	sgl0001	GO:0015399	<1e-4	sll1036	GO:0005575	<1e-4	sll0505	GO:0016772	0.0258
slr1628	GO:0048522	<1e-4	ssl5025	GO:0046128	<1e-4	ssl2245	GO:0003674	<1e-4	sll5033	GO:0044444	<1e-4	slr1098	GO:0016772	0.0258
slr0065	GO:0051188	<1e-4	ssl1498	GO:0044260	<1e-4	sll1541	GO:0016817	<1e-4	sll1853	GO:0043226	<1e-4	slr0594	GO:0016772	0.0258
sll1009	GO:0006066	<1e-4	slr7073	GO:0051246	<1e-4	slr1885	GO:0015399	<1e-4	sll1306	GO:0044464	<1e-4	sll0614	GO:0004518	0.0255
sll0413	GO:0018130	<1e-4	sll0524	GO:0043170	<1e-4	slr0769	GO:0015405	<1e-4	ssl0410	GO:0005575	<1e-4	sll0588	GO:0004518	0.0255
sll1217	GO:0009056	<1e-4	slr0913	GO:0043648	<1e-4	slr0397	GO:0015077	<1e-4	slr1638	GO:0044425	<1e-4	slr1493	GO:0004518	0.0255
sll1680	GO:0031640	<1e-4	slr1923	GO:0009150	<1e-4	sll1766	GO:0022891	<1e-4	slr1196	GO:0044446	<1e-4	slr1541	GO:0004518	0.0255
sll0473	GO:0010468	<1e-4	slr0498	GO:0051188	<1e-4	slr2003	GO:0015267	<1e-4	slr0655	GO:0044422	<1e-4	slr0924	GO:0004518	0.0255
slr7094	GO:0051171	<1e-4	slr7060	GO:0072521	<1e-4	slr1813	GO:0022832	<1e-4	ssl2420	GO:0044446	<1e-4	sll0319	GO:0004518	0.0255
slr1885	GO:0072521	<1e-4	sll5063	GO:0006066	<1e-4	slr0789	GO:0016462	<1e-4	slr0104	GO:0044425	<1e-4	slr1053	GO:0004518	0.0255
sll7062	GO:0009057	<1e-4	sll1634	GO:0033013	<1e-4	slr1932	GO:0016818	<1e-4	sll1109	GO:0044424	<1e-4	sll7065	GO:0004518	0.0255
ssl3291	GO:0043436	<1e-4	sll0098	GO:0009142	<1e-4	slr0294	GO:0022836	<1e-4	slr1035	GO:0044425	<1e-4	sll1654	GO:0004518	0.0255
slr0517	GO:0033013	<1e-4	sll6055	GO:0051186	<1e-4	slr1353	GO:0016746	<1e-4	slr0196	GO:0044424	<1e-4	slr7024	GO:0004518	0.0255
sll1262	GO:0051246	<1e-4	slr1449	GO:0072527	<1e-4	sll1737	GO:0015399	<1e-4	ssl5098	GO:0044444	<1e-4	sll1350	GO:0004518	0.0255
slr0271	GO:0051246	<1e-4	slr0742	GO:0009150	<1e-4	ssl3615	GO:0016462	<1e-4	slr0888	GO:0044464	<1e-4	sll0359	GO:0004518	0.0255
slr1507	GO:0044260	<1e-4	slr0065	GO:0044271	<1e-4	slr6009	GO:0015077	<1e-4	slr7011	GO:0043226	<1e-4	sll0188	GO:0004518	0.0255
sll0310	GO:0050801	<1e-4	slr1880	GO:0009987	<1e-4	sll0783	GO:0016462	<1e-4	sll1163	GO:0044424	<1e-4	ssr3300	GO:0004518	0.0255
slr0810	GO:0042180	<1e-4	ssl5007	GO:0080090	<1e-4	slr0784	GO:0003674	<1e-4	sll0984	GO:0044446	<1e-4	slr1303	GO:0004518	0.0255
slr2060	GO:0043412	<1e-4	sll1052	GO:0009123	<1e-4	sll0614	GO:0022890	<1e-4	sll0405	GO:0044444	<1e-4	sll1072	GO:0004518	0.0255
slr0976	GO:0050789	<1e-4	slr0172	GO:0044003	<1e-4	sll0499	GO:0042623	<1e-4	slr1704	GO:0044422	<1e-4	slr1940	GO:0004518	0.0255
slr0364	GO:0043412	<1e-4	slr0509	GO:0050789	<1e-4	sll1938	GO:0016817	<1e-4	slr1082	GO:0043226	<1e-4	slr1668	GO:0004518	0.0255
ssr7072	GO:0009987	<1e-4	slr0325	GO:0072527	<1e-4	sll1464	GO:0005342	<1e-4	slr1053	GO:0043226	<1e-4	slr1690	GO:0004518	0.0255
slr1327	GO:0048519	<1e-4	sll0147	GO:0046128	<1e-4	sll0180	GO:0022892	<1e-4	sll0860	GO:0044425	<1e-4	sll1021	GO:0004518	0.0255
sll1424	GO:0009892	<1e-4	slr5111	GO:0042180	<1e-4	sll1979	GO:0022803	<1e-4	slr5118	GO:0044446	<1e-4	sll0888	GO:0004518	0.0255
slr0285	GO:0044281	<1e-4	slr1262	GO:0042180	<1e-4	slr1069	GO:0015293	<1e-4	sll0786	GO:0044464	<1e-4	sll0268	GO:0004518	0.0255
slr6051	GO:0046128	<1e-4	slr1852	GO:0031640	<1e-4	slr0655	GO:0060089	<1e-4	slr1619	GO:0044444	<1e-4	slr0664	GO:0004518	0.0255
slr1378	GO:0022411	<1e-4	ssl2065	GO:0048878	<1e-4	slr0935	GO:0003674	<1e-4	ssl7039	GO:0005575	<1e-4	ssr2754	GO:0004518	0.0255
slr5077	GO:0048878	<1e-4	ssr2998	GO:0046483	<1e-4	sll0068	GO:0022838	<1e-4	ssl2717	GO:0044446	<1e-4	slr1082	GO:0004518	0.0255
slr0586	GO:0008150	<1e-4	slr1431	GO:0034654	<1e-4	slr6091	GO:0015399	<1e-4	slr0964	GO:0005575	<1e-4	sll1225	GO:0004518	0.0255
slr6101	GO:0009889	<1e-4	sll0875	GO:0051186	<1e-4	sll0860	GO:0043167	<1e-4	sll0815	GO:0005575	<1e-4	sll0810	GO:0004518	0.0255
ssl2749	GO:0009056	<1e-4	sll1240	GO:0071842	<1e-4	slr1726	GO:0016817	<1e-4	slr1493	GO:0044446	<1e-4	slr1862	GO:0004518	0.0255
slr5126	GO:0031323	<1e-4	slr1397	GO:0072528	<1e-4	slr0656	GO:0043492	<1e-4	sll1025	GO:0044444	<1e-4	slr1647	GO:0004518	0.0255
slr1644	GO:0006766	<1e-4	sll0639	GO:0065007	<1e-4	slr2121	GO:0043167	<1e-4	slr6029	GO:0044422	<1e-4	sll1411	GO:0004518	0.0255
sll0249	GO:0071554	<1e-4	sll8032	GO:0006163	<1e-4	slr0392	GO:0008171	<1e-4	slr0366	GO:0043226	<1e-4	slr1544	GO:0004518	0.0255
slr2119	GO:0022607	<1e-4	sll6052	GO:0009165	<1e-4	slr7025	GO:0022891	<1e-4	sll0101	GO:0044464	<1e-4	sll0752	GO:0004518	0.0255
sll1960	GO:0043933	<1e-4	slr1613	GO:0044003	<1e-4	slr1484	GO:0016746	<1e-4	sll1164	GO:0044425	<1e-4	slr0172	GO:0004518	0.0255
slr0408	GO:0072521	<1e-4	sll1203	GO:0019751	<1e-4	ssr1375	GO:0005342	<1e-4	sll1293	GO:0044464	<1e-4	ssl0739	GO:0004518	0.0255
slr7058	GO:0009892	<1e-4	slr0909	GO:0043412	<1e-4	ssr5121	GO:0015291	<1e-4	slr2000	GO:0044424	<1e-4	slr1287	GO:0004518	0.0255
slr1914	GO:0071842	<1e-4	sll0984	GO:0043933	<1e-4	sll1109	GO:0022832	<1e-4	slr1951	GO:0044464	<1e-4	sll1201	GO:0004518	0.0255

sll1024	GO:0009126	<1e-4	sll1401	GO:0006066	<1e-4	sll1880	GO:0016746	<1e-4	sll0911	GO:0005575	<1e-4	sll1239	GO:0004518	0.0255
sll0740	GO:0044281	<1e-4	slr0887	GO:0050896	<1e-4	slr2073	GO:0015267	<1e-4	slr0806	GO:0044422	<1e-4	slr0455	GO:0004518	0.0255
sll0669	GO:0009165	<1e-4	sll0658	GO:0009117	<1e-4	slr0765	GO:0022857	<1e-4	ssl5065	GO:0044444	<1e-4	slr0935	GO:0004518	0.0255
slr1809	GO:0006732	<1e-4	slr0374	GO:0019751	<1e-4	slr5077	GO:0022803	<1e-4	sll1426	GO:0043226	<1e-4	sll1752	GO:0004518	0.0255
slr1066	GO:0009161	<1e-4	slr1045	GO:0051347	<1e-4	sll1965	GO:0022838	<1e-4	slr0650	GO:0044464	<1e-4	slr2115	GO:0004518	0.0255
sll0518	GO:0009150	<1e-4	sll0243	GO:0009199	<1e-4	ssl0738	GO:0015405	<1e-4	sll1400	GO:0044464	<1e-4	sll0740	GO:0004518	0.0255
sll1130	GO:0051246	<1e-4	sll0397	GO:0006811	<1e-4	slr0650	GO:0022890	<1e-4	ssr6032	GO:0044424	<1e-4	slr0789	GO:0004518	0.0255
slr0318	GO:0009262	<1e-4	slr1189	GO:0006753	<1e-4	sll5033	GO:0016817	<1e-4	slr1541	GO:0044464	<1e-4	slr0272	GO:0004518	0.0255
slr2049	GO:0051171	<1e-4	slr1260	GO:0009117	<1e-4	sll0761	GO:0022892	<1e-4	slr0913	GO:0044446	<1e-4	sll1344	GO:0004518	0.0255
slr1851	GO:0018130	<1e-4	ssl7007	GO:0022607	<1e-4	slr0729	GO:0015405	<1e-4	ssl1923	GO:0044444	<1e-4	slr0111	GO:0004518	0.0255
sll1193	GO:0065007	<1e-4	slr7092	GO:0034660	<1e-4	slr0146	GO:0016818	<1e-4	slr1383	GO:0043226	<1e-4	sll1173	GO:0004518	0.0255
sll0867	GO:0016052	<1e-4	slr1677	GO:0031326	<1e-4	sll1131	GO:0022803	<1e-4	sll1866	GO:0005575	<1e-4	slr0053	GO:0004518	0.0255
slr0553	GO:0048878	<1e-4	sll1979	GO:0080090	<1e-4	slr1614	GO:0015077	<1e-4	sll0837	GO:0005575	<1e-4	sll0281	GO:0004518	0.0255
ssl5031	GO:0006220	<1e-4	sll8040	GO:0016053	<1e-4	sll0424	GO:0015291	<1e-4	ssl0788	GO:0044425	<1e-4	sll0827	GO:0004518	0.0255
slr1847	GO:0022411	<1e-4	ssl5091	GO:0072528	<1e-4	slr1097	GO:0022857	<1e-4	sll1530	GO:0044464	<1e-4	sll0410	GO:0004518	0.0255
slr1576	GO:0019222	<1e-4	slr0111	GO:0022411	<1e-4	slr1827	GO:0043167	<1e-4	slr1601	GO:0044424	<1e-4	slr1070	GO:0004518	0.0255
sll0547	GO:0065007	<1e-4	sll1222	GO:0044283	<1e-4	slr1397	GO:0015075	<1e-4	slr1726	GO:0044444	<1e-4	sll0996	GO:0044237	0.0254
slr1932	GO:0006721	<1e-4	slr1907	GO:0051234	<1e-4	sll0479	GO:0016817	<1e-4	slr1927	GO:0044446	<1e-4	ssl0109	GO:0044237	0.0254
ssl1255	GO:0044271	<1e-4	slr0049	GO:0044255	<1e-4	sll1656	GO:0022803	<1e-4	ssl0832	GO:0044464	<1e-4	slr0480	GO:0044237	0.0254
ssl0109	GO:0044275	<1e-4	slr1206	GO:0034654	<1e-4	sll0886	GO:0008171	<1e-4	slr1073	GO:0043226	<1e-4	sll1318	GO:0044237	0.0254
sll1960	GO:0010556	<1e-4	sll1446	GO:0031323	<1e-4	ssr2998	GO:0022891	<1e-4	sll0413	GO:0044424	<1e-4	slr1505	GO:0044237	0.0254
sll8027	GO:0009142	<1e-4	sll0297	GO:0043648	<1e-4	sll0048	GO:0008171	<1e-4	ssl1498	GO:0044425	<1e-4	sll1601	GO:0044237	0.0254
sll0487	GO:0009308	<1e-4	sll1651	GO:0042455	<1e-4	ssl5025	GO:0022836	<1e-4	sll1092	GO:0044424	<1e-4	ssr1375	GO:0044237	0.0254
slr1612	GO:0009308	<1e-4	slr0271	GO:0018130	<1e-4	sll1866	GO:0022890	<1e-4	sll0449	GO:0044464	<1e-4	sll0499	GO:0044237	0.0254
slr0656	GO:0048518	<1e-4	slr0887	GO:0051179	<1e-4	ssr3300	GO:0015077	<1e-4	slr2003	GO:0043226	<1e-4	sll1135	GO:0044237	0.0254
ssr1114	GO:0044003	<1e-4	ssr3570	GO:0009056	<1e-4	sll1938	GO:0015399	<1e-4	sll0263	GO:0044424	<1e-4	sll0360	GO:0044237	0.0254
ssr2972	GO:0009263	<1e-4	sll1722	GO:0048878	<1e-4	slr7096	GO:0016462	<1e-4	sll0149	GO:0044446	<1e-4	sll1247	GO:0044237	0.0254
ssl5099	GO:0009126	<1e-4	ssl1577	GO:0009144	<1e-4	sll8032	GO:0043492	<1e-4	slr0300	GO:0044425	<1e-4	sll0547	GO:0044237	0.0254
sll1219	GO:0009132	<1e-4	ssr0536	GO:0019752	<1e-4	sll0192	GO:0042623	<1e-4	slr0645	GO:0044424	<1e-4	slr1127	GO:0044237	0.0254
slr0957	GO:0022607	<1e-4	sll0022	GO:0009141	<1e-4	slr0909	GO:0022891	<1e-4	slr0655	GO:0044444	<1e-4	slr0304	GO:0044237	0.0254
slr2125	GO:0050789	<1e-4	sll0641	GO:0031323	<1e-4	ssl7048	GO:0015399	<1e-4	slr1261	GO:0044464	<1e-4	sll0508	GO:0044237	0.0254
sll1606	GO:0046394	<1e-4	sll0176	GO:0006732	<1e-4	sll0188	GO:0043167	<1e-4	slr0605	GO:0044425	<1e-4	slr0876	GO:0044237	0.0254
slr1914	GO:0006732	<1e-4	ssr0102	GO:0006163	<1e-4	slr1438	GO:0016462	<1e-4	ssr5020	GO:0044425	<1e-4	ssl3692	GO:0044237	0.0254
sll1638	GO:0016054	<1e-4	slr0588	GO:0046128	<1e-4	sll0614	GO:0015291	<1e-4	slr1194	GO:0044424	<1e-4	slr0287	GO:0044237	0.0254
slr0957	GO:0052111	<1e-4	ssr2439	GO:0044271	<1e-4	sll0608	GO:0022838	<1e-4	sll0740	GO:0005575	<1e-4	sll0296	GO:0044237	0.0254
sll1164	GO:0009165	<1e-4	ssr3154	GO:0055082	<1e-4	sll1188	GO:0003674	<1e-4	sll0886	GO:0005575	<1e-4	sll0283	GO:0044237	0.0254
slr1306	GO:0009260	<1e-4	ssr2802	GO:0009394	<1e-4	slr0589	GO:0016818	<1e-4	slr0650	GO:0044444	<1e-4	slr0937	GO:0044237	0.0254
slr5053	GO:0072522	<1e-4	sll0147	GO:0022607	<1e-4	slr2084	GO:0043492	<1e-4	slr1429	GO:0005575	<1e-4	slr0299	GO:0044237	0.0254
slr0725	GO:0043648	<1e-4	slr0625	GO:0019219	<1e-4	slr1301	GO:0015077	<1e-4	ssr6078	GO:0005575	<1e-4	sll0274	GO:0044237	0.0254
slr1906	GO:0009889	<1e-4	sll0932	GO:0043549	<1e-4	sll1979	GO:0043492	<1e-4	slr1069	GO:0044424	<1e-4	sll0854	GO:0044237	0.0254
slr1474	GO:0018130	<1e-4	sll0761	GO:0031323	<1e-4	slr2125	GO:0016746	<1e-4	sll0710	GO:0005575	<1e-4	sll2006	GO:0044237	0.0254
slr1886	GO:0006811	<1e-4	ssl2245	GO:0080090	<1e-4	sll8033	GO:0022838	<1e-4	sll0022	GO:0044464	<1e-4	sll0301	GO:0044237	0.0254
slr0217	GO:0043549	<1e-4	sll0297	GO:0009262	<1e-4	slr7073	GO:0060089	<1e-4	slr1644	GO:0044446	<1e-4	slr1073	GO:0044237	0.0253
sll8002	GO:0050896	<1e-4	sll0238	GO:0051188	<1e-4	ssl1464	GO:0015075	<1e-4	slr0456	GO:0044446	<1e-4	sll1002	GO:0044237	0.0253
ssr6079	GO:0043412	<1e-4	sll1433	GO:0008610	<1e-4	sll0069	GO:0015291	<1e-4	sll7069	GO:0044424	<1e-4	slr1638	GO:0044237	0.0253
sll1691	GO:0006720	<1e-4	ssl0832	GO:0050789	<1e-4	sll8027	GO:0060089	<1e-4	slr0264	GO:0005575	<1e-4	slr0519	GO:0044237	0.0253
sll1433	GO:0051234	<1e-4	slr6068	GO:0052111	<1e-4	slr0651	GO:0022891	<1e-4	sll1681	GO:0044464	<1e-4	slr0386	GO:0044237	0.0253
slr0978	GO:0044281	<1e-4	ssl2920	GO:0006720	<1e-4	sll1530	GO:0043167	<1e-4	sll0984	GO:0005575	<1e-4	sll0314	GO:0044237	0.0253
sll0602	GO:0006163	<1e-4	slr0845	GO:0044275	<1e-4	sll0066	GO:0060089	<1e-4	sll0167	GO:0044425	<1e-4	sll0066	GO:0044237	0.0253
sll1730	GO:0048519	<1e-4	ssr3571	GO:0008150	<1e-4	ssr6099	GO:0022857	<1e-4	ssl3383	GO:0044444	<1e-4	slr1593	GO:0044237	0.0253
slr0455	GO:0071841	<1e-4	ssl0787	GO:0044260	<1e-4	sll1925	GO:0022890	<1e-4	sll0788	GO:0005575	<1e-4	sll0007	GO:0044237	0.0253
sll0482	GO:0009394	<1e-4	slr0480	GO:0009132	<1e-4	slr1614	GO:0016817	<1e-4	sll0314	GO:0044464	<1e-4	slr1262	GO:0044237	0.0253
ssr1375	GO:0051234	<1e-4	ssr5011	GO:0052111	<1e-4	slr0821	GO:0015267	<1e-4	sll0688	GO:0044422	<1e-4	sll1656	GO:0044237	0.0253
ssl2733	GO:0072524	<1e-4	slr1964	GO:0009126	<1e-4	slr0482	GO:0003674	<1e-4	ssl2148	GO:0043226	<1e-4	slr0586	GO:0044237	0.0253

slr0813	GO:0022411	<1e-4	sll1062	GO:0051188	<1e-4	sll0101	GO:0008171	<1e-4	slr1519	GO:0005575	<1e-4	sll1954	GO:0044237	0.0253
slr0637	GO:0042180	<1e-4	slr1900	GO:0006082	<1e-4	sll1949	GO:0022836	<1e-4	sll1381	GO:0005575	<1e-4	sll1530	GO:0044237	0.0253
ssl1690	GO:0006721	<1e-4	slr0058	GO:0080090	<1e-4	slr1223	GO:0003674	<1e-4	slr0489	GO:0043226	<1e-4	sll1061	GO:0044237	0.0253
slr2012	GO:0009394	<1e-4	ssl0461	GO:0072524	<1e-4	sll0656	GO:0015405	<1e-4	slr0053	GO:0044424	<1e-4	slr1648	GO:0044237	0.0253
ssl3291	GO:0055082	<1e-4	slr6012	GO:0048522	<1e-4	sll1472	GO:0022838	<1e-4	sll7069	GO:0005575	<1e-4	sll0191	GO:0044237	0.0253
sll1160	GO:0046483	<1e-4	slr0957	GO:0048878	<1e-4	sll0858	GO:0008171	<1e-4	slr1194	GO:0044464	<1e-4	ssr3122	GO:0044237	0.0253
slr2070	GO:0090304	<1e-4	slr0299	GO:0051234	<1e-4	sll1726	GO:0005342	<1e-4	sll0615	GO:0044446	<1e-4	slr1230	GO:0044237	0.0253
ssl5103	GO:0050794	<1e-4	slr0699	GO:0071554	<1e-4	slr6075	GO:0022892	<1e-4	sll1429	GO:0005575	<1e-4	sll0676	GO:0044237	0.0253
slr6066	GO:0009126	<1e-4	ssl5100	GO:0009259	<1e-4	slr0581	GO:0016746	<1e-4	sll1530	GO:0043226	<1e-4	slr0360	GO:0044237	0.0253
ssr7072	GO:0034660	<1e-4	slr7057	GO:0044260	<1e-4	slr0483	GO:0022803	<1e-4	slr2042	GO:0044424	<1e-4	ssl1577	GO:0044237	0.0253
ssl5114	GO:0034641	<1e-4	slr1657	GO:0019222	<1e-4	slr0821	GO:0022857	<1e-4	slr0755	GO:0044422	<1e-4	slr1572	GO:0044237	0.0253
ssl0467	GO:0006811	<1e-4	slr1958	GO:0009262	<1e-4	slr1258	GO:0022891	<1e-4	sll0590	GO:0044424	<1e-4	sll1315	GO:0044237	0.0253
slr1338	GO:0072524	<1e-4	slr1920	GO:0005996	<1e-4	slr1110	GO:0016818	<1e-4	sll0384	GO:0044425	<1e-4	sll0780	GO:0044237	0.0253
ssr6027	GO:0052111	<1e-4	ssr3532	GO:0072527	<1e-4	sll0586	GO:0015399	<1e-4	sll0309	GO:0044446	<1e-4	sll1675	GO:0044237	0.0253
slr0360	GO:0042180	<1e-4	ssl1046	GO:0044283	<1e-4	ssl0350	GO:0016462	<1e-4	sll1340	GO:0044425	<1e-4	sll0524	GO:0044237	0.0253
sll7047	GO:0009056	<1e-4	sll1880	GO:0042180	<1e-4	sll1414	GO:0016817	<1e-4	sll1722	GO:0005575	<1e-4	sll0497	GO:0044237	0.0253
sll1318	GO:0043933	<1e-4	sgl0002	GO:0044271	<1e-4	sll1609	GO:0015405	<1e-4	sll0670	GO:0044422	<1e-4	sll0360	GO:0016820	0.025
sll8002	GO:0019222	<1e-4	sll5132	GO:0031326	<1e-4	slr0909	GO:0016746	<1e-4	sll1378	GO:0044444	<1e-4	sll1318	GO:0016820	0.025
sll1942	GO:0060255	<1e-4	slr1442	GO:0009126	<1e-4	slr6088	GO:0016741	<1e-4	slr1081	GO:0044446	<1e-4	slr2025	GO:0016820	0.025
slr1495	GO:0009892	<1e-4	sll1426	GO:0051179	<1e-4	sll1267	GO:0043492	<1e-4	sll1573	GO:0005575	<1e-4	slr1417	GO:0016820	0.025
slr0605	GO:0009991	<1e-4	slr1668	GO:0006753	<1e-4	ssr2972	GO:0015267	<1e-4	slr1951	GO:0005575	<1e-4	slr1376	GO:0016820	0.025
sll0241	GO:0006733	<1e-4	sll1239	GO:0019752	<1e-4	ssr1375	GO:0015267	<1e-4	slr7015	GO:0044422	<1e-4	slr1391	GO:0016820	0.025
slr1956	GO:0046128	<1e-4	slr0157	GO:0052111	<1e-4	slr1053	GO:0060089	<1e-4	slr0060	GO:0043226	<1e-4	ssr1698	GO:0016820	0.025
slr7081	GO:0009889	<1e-4	slr1789	GO:0071554	<1e-4	slr1394	GO:0015405	<1e-4	slr0889	GO:0005575	<1e-4	sll1455	GO:0016820	0.025
sll1995	GO:0034641	<1e-4	sll0249	GO:0019219	<1e-4	ssr6083	GO:0022890	<1e-4	ssl2971	GO:0044425	<1e-4	slr1659	GO:0016820	0.025
sll1267	GO:0019362	<1e-4	slr7092	GO:0051716	<1e-4	slr0845	GO:0043492	<1e-4	ssl8008	GO:0005575	<1e-4	sll1218	GO:0016820	0.025
slr1287	GO:0016054	<1e-4	slr0551	GO:0006811	<1e-4	slr1657	GO:0043167	<1e-4	slr5016	GO:0005575	<1e-4	slr0287	GO:0016820	0.025
sll1442	GO:0009260	<1e-4	slr1914	GO:0009259	<1e-4	sll0702	GO:0015267	<1e-4	slr0355	GO:0044424	<1e-4	sll1426	GO:0016820	0.025
sll1123	GO:0072522	<1e-4	sll0174	GO:0008150	<1e-4	slr7014	GO:0015399	<1e-4	sll1979	GO:0044424	<1e-4	sll0382	GO:0016820	0.025
sll0446	GO:0031640	<1e-4	slr6049	GO:0043933	<1e-4	sll0444	GO:0003674	<1e-4	sll0543	GO:0044464	<1e-4	sll0864	GO:0016820	0.025
sll0846	GO:0051716	<1e-4	slr1977	GO:0031323	<1e-4	slr1970	GO:0022832	<1e-4	slr1169	GO:0043226	<1e-4	slr1900	GO:0016820	0.025
ssr3572	GO:0022607	<1e-4	slr1384	GO:0034660	<1e-4	sll1173	GO:0015077	<1e-4	slr1799	GO:0005575	<1e-4	slr0871	GO:0016820	0.025
ssl2920	GO:0001932	<1e-4	sll1601	GO:0071842	<1e-4	ssr8013	GO:0022857	<1e-4	sll0354	GO:0043226	<1e-4	sll1942	GO:0016820	0.025
slr0058	GO:0009141	<1e-4	sll1219	GO:0044106	<1e-4	slr1103	GO:0008171	<1e-4	slr1170	GO:0005575	<1e-4	slr1127	GO:0016820	0.025
sll1715	GO:0009262	<1e-4	sll0995	GO:0009142	<1e-4	slr1259	GO:0022836	<1e-4	slr1327	GO:0044424	<1e-4	slr1896	GO:0016820	0.025
sll0281	GO:0009117	<1e-4	slr1573	GO:0072521	<1e-4	sll0183	GO:0016741	<1e-4	slr0601	GO:0044446	<1e-4	sll2006	GO:0016820	0.025
sll1573	GO:0031323	<1e-4	sll0780	GO:0042455	<1e-4	sll7077	GO:0060089	<1e-4	sll1262	GO:0044444	<1e-4	slr1083	GO:0016820	0.025
slr1623	GO:0044106	<1e-4	ssl6061	GO:0055114	<1e-4	slr7025	GO:0015077	<1e-4	sll1749	GO:0043226	<1e-4	sll0854	GO:0016820	0.025
sll0230	GO:0006220	<1e-4	slr2103	GO:0044281	<1e-4	slr7026	GO:0016462	<1e-4	slr1681	GO:0043226	<1e-4	sll1174	GO:0016820	0.025
slr1623	GO:0044260	<1e-4	sll1485	GO:0071496	<1e-4	ssl5008	GO:0015399	<1e-4	sll1630	GO:0005575	<1e-4	slr0250	GO:0016820	0.025
slr2118	GO:0033013	<1e-4	slr7091	GO:0006733	<1e-4	slr2111	GO:0022892	<1e-4	sll0867	GO:0044446	<1e-4	slr1505	GO:0016820	0.025
sgl0002	GO:0052111	<1e-4	sll7077	GO:0009893	<1e-4	sll1130	GO:0016817	<1e-4	slr0588	GO:0043226	<1e-4	ssr0536	GO:0016820	0.025
slr1704	GO:0010468	<1e-4	ssl2162	GO:0042451	<1e-4	sll0060	GO:0015293	<1e-4	sll1401	GO:0043226	<1e-4	ssr3572	GO:0044237	0.0242
slr1998	GO:0008610	<1e-4	sll1527	GO:0065007	<1e-4	sll0449	GO:0015405	<1e-4	sll8040	GO:0005575	<1e-4	ssr2067	GO:0044237	0.0242
slr1103	GO:0009889	<1e-4	sll1583	GO:0042180	<1e-4	slr0921	GO:0015077	<1e-4	ssr2754	GO:0043226	<1e-4	ssl2162	GO:0044237	0.0242
slr2060	GO:0051818	<1e-4	ssl5096	GO:0034641	<1e-4	slr0516	GO:0015405	<1e-4	sll0913	GO:0043226	<1e-4	ssr1473	GO:0044237	0.0242
sll0286	GO:0048522	<1e-4	sll1203	GO:0065007	<1e-4	ssr2142	GO:0016817	<1e-4	ssl0109	GO:0043226	<1e-4	slr0725	GO:0044237	0.0242
sll0995	GO:0006721	<1e-4	slr0109	GO:0051171	<1e-4	slr0948	GO:0022836	<1e-4	sll1606	GO:0044444	<1e-4	slr0865	GO:0044237	0.0242
ssr2333	GO:0034641	<1e-4	slr1778	GO:0009309	<1e-4	sll1461	GO:0022838	<1e-4	slr1207	GO:0044424	<1e-4	ssr2553	GO:0044237	0.0242
sll0756	GO:0072524	<1e-4	ssr6083	GO:0048523	<1e-4	ssl2064	GO:0016741	<1e-4	slr1599	GO:0044422	<1e-4	slr0416	GO:0044237	0.0242
sll0085	GO:0044275	<1e-4	sll0436	GO:0009309	<1e-4	sll1832	GO:0005342	<1e-4	ssr2060	GO:0043226	<1e-4	ssr6089	GO:0044237	0.0242
sll1092	GO:0048518	<1e-4	slr1110	GO:0016054	<1e-4	slr0588	GO:0015077	<1e-4	sll1873	GO:0005575	<1e-4	slr5127	GO:0044237	0.0242
slr0921	GO:0016054	<1e-4	slr5126	GO:0051246	<1e-4	sll0586	GO:0060089	<1e-4	slr0863	GO:0044422	<1e-4	ssr2047	GO:0044237	0.0242
ssl2064	GO:0019219	<1e-4	slr5012	GO:0065007	<1e-4	sll1730	GO:0043492	<1e-4	sll1717	GO:0043226	<1e-4	ssl3615	GO:0044237	0.0242

slr0801	GO:0052111	<1e-4	slr2105	GO:0034641	<1e-4	sll1925	GO:0060089	<1e-4	slr1906	GO:0044425	<1e-4	slr7010	GO:0044237	0.0242
ssr2333	GO:0009056	<1e-4	slr0207	GO:0006753	<1e-4	slr0305	GO:0016462	<1e-4	ssr1768	GO:0005575	<1e-4	sll1486	GO:0044237	0.0242
slr0594	GO:0043412	<1e-4	slr6075	GO:0009144	<1e-4	sll2011	GO:0016462	<1e-4	sll0703	GO:0043226	<1e-4	ssl5025	GO:0044237	0.0242
slr1591	GO:0046164	<1e-4	sll0810	GO:0031323	<1e-4	slr0059	GO:0022892	<1e-4	sll1765	GO:0005575	<1e-4	sll8001	GO:0044237	0.0242
slr1667	GO:0006732	<1e-4	sll5028	GO:0009889	<1e-4	slr2073	GO:0042623	<1e-4	sll0022	GO:0044446	<1e-4	slr1406	GO:0044237	0.0242
slr0300	GO:0043549	<1e-4	sll0995	GO:0044106	<1e-4	slr1209	GO:0016462	<1e-4	ssr2439	GO:0043226	<1e-4	ssr2060	GO:0044237	0.0242
sll1510	GO:0009394	<1e-4	slr1484	GO:0044281	<1e-4	slr6067	GO:0060089	<1e-4	ssl0467	GO:0044464	<1e-4	sll8019	GO:0044237	0.0242
slr1911	GO:0009056	<1e-4	slr0989	GO:0043549	<1e-4	sll7050	GO:0022857	<1e-4	sll0822	GO:0043226	<1e-4	slr5024	GO:0044237	0.0242
sll0369	GO:0051716	<1e-4	slr0407	GO:0072521	<1e-4	slr2003	GO:0043492	<1e-4	slr0483	GO:0044424	<1e-4	ssr2975	GO:0044237	0.0242
ssl5008	GO:0009199	<1e-4	slr6016	GO:0034654	<1e-4	slr1082	GO:0016817	<1e-4	slr1207	GO:0044444	<1e-4	slr1438	GO:0044237	0.0242
slr1437	GO:0055082	<1e-4	slr7073	GO:0009126	<1e-4	sll0085	GO:0043492	<1e-4	slr0109	GO:0043226	<1e-4	slr5012	GO:0044237	0.0242
sll1193	GO:0009893	<1e-4	slr1659	GO:0042180	<1e-4	sll1630	GO:0022832	<1e-4	slr2037	GO:0044425	<1e-4	slr6075	GO:0044237	0.0242
slr1186	GO:0031323	<1e-4	ssl7074	GO:0051818	<1e-4	sll0410	GO:0016746	<1e-4	sll0189	GO:0044425	<1e-4	slr5016	GO:0044237	0.0242
ssr0657	GO:0034654	<1e-4	sll5028	GO:0016053	<1e-4	ssl5096	GO:0022803	<1e-4	sll5061	GO:0005575	<1e-4	sll0335	GO:0044237	0.0242
sll1062	GO:0022411	<1e-4	slr0250	GO:0031323	<1e-4	ssr3129	GO:0043167	<1e-4	slr0106	GO:0044464	<1e-4	slr0303	GO:0044237	0.0242
slr1117	GO:0048878	<1e-4	slr6074	GO:0048519	<1e-4	slr0888	GO:0042623	<1e-4	ssr5019	GO:0044425	<1e-4	sll0069	GO:0044237	0.0242
ssr6062	GO:0009893	<1e-4	slr0291	GO:0009991	<1e-4	slr0169	GO:0016818	<1e-4	slr2084	GO:0005575	<1e-4	sll0372	GO:0044237	0.0242
ssr1375	GO:0042455	<1e-4	sll1630	GO:0009262	<1e-4	sll5034	GO:0022890	<1e-4	slr1614	GO:0005575	<1e-4	ssl5103	GO:0044237	0.0242
slr0654	GO:0009889	<1e-4	sll0804	GO:0051179	<1e-4	slr0169	GO:0022832	<1e-4	sll0793	GO:0005575	<1e-4	sll1340	GO:0044237	0.0242
sll0274	GO:0006753	<1e-4	sll7067	GO:0006576	<1e-4	sll1638	GO:0016817	<1e-4	slr0712	GO:0044444	<1e-4	slr1752	GO:0044237	0.0242
sll0563	GO:0031323	<1e-4	slr6063	GO:0019362	<1e-4	sll0101	GO:0015077	<1e-4	sll1201	GO:0044424	<1e-4	slr1290	GO:0044237	0.0242
slr0217	GO:0072524	<1e-4	sll1162	GO:0006793	<1e-4	slr1944	GO:0022838	<1e-4	slr0981	GO:0044444	<1e-4	sll0847	GO:0044237	0.0242
slr0742	GO:0065007	<1e-4	sll0595	GO:0043933	<1e-4	slr6015	GO:0043167	<1e-4	sll1036	GO:0044446	<1e-4	slr7073	GO:0044237	0.0242
slr0270	GO:0052188	<1e-4	slr1768	GO:0048519	<1e-4	sll1315	GO:0016817	<1e-4	ssr6020	GO:0044422	<1e-4	sll1321	GO:0044237	0.0242
sll7047	GO:0044260	<1e-4	slr0291	GO:0048519	<1e-4	ssr0336	GO:0003674	<1e-4	sll0023	GO:0044422	<1e-4	sll7028	GO:0044237	0.0242
sll1835	GO:0010468	<1e-4	sll0839	GO:0051347	<1e-4	sll7033	GO:0022836	<1e-4	slr1290	GO:0044422	<1e-4	slr7060	GO:0044237	0.0242
ssl2148	GO:0044271	<1e-4	slr1774	GO:0009132	<1e-4	sll1880	GO:0016818	<1e-4	sll1764	GO:0044444	<1e-4	slr8044	GO:0044237	0.0242
slr0103	GO:0050801	<1e-4	sll0861	GO:0022411	<1e-4	sll1530	GO:0022803	<1e-4	sll1488	GO:0005575	<1e-4	slr1704	GO:0044237	0.0242
slr0516	GO:0009161	<1e-4	slr1114	GO:0006793	<1e-4	slr1103	GO:0003674	<1e-4	slr2110	GO:0044425	<1e-4	slr6088	GO:0044237	0.0242
sll1289	GO:0046907	<1e-4	slr8044	GO:0010468	<1e-4	slr2052	GO:0015077	<1e-4	sll0980	GO:0044422	<1e-4	slr0270	GO:0044237	0.0242
sll1164	GO:0009309	<1e-4	slr0602	GO:0009259	<1e-4	slr1624	GO:0015399	<1e-4	slr1702	GO:0043226	<1e-4	sll0905	GO:0044237	0.0242
slr0845	GO:0072521	<1e-4	slr2048	GO:0022607	<1e-4	slr0356	GO:0008171	<1e-4	slr0872	GO:0044424	<1e-4	slr7094	GO:0044237	0.0242
slr0821	GO:0009259	<1e-4	ssr1766	GO:0006721	<1e-4	sll0597	GO:0016818	<1e-4	slr6045	GO:0044425	<1e-4	slr5037	GO:0044237	0.0242
slr0374	GO:0051716	<1e-4	slr1206	GO:0052188	<1e-4	sll0608	GO:0022890	<1e-4	slr1990	GO:0044425	<1e-4	sll0101	GO:0044237	0.0242
sll0843	GO:0051716	<1e-4	sll0424	GO:0009308	<1e-4	sll0023	GO:0060089	<1e-4	sll1938	GO:0044464	<1e-4	sll0543	GO:0044237	0.0242
slr0172	GO:0072522	<1e-4	slr0575	GO:0010468	<1e-4	ssl1255	GO:0022803	<1e-4	slr0121	GO:0044424	<1e-4	slr1206	GO:0044237	0.0242
sll1021	GO:0043648	<1e-4	slr1911	GO:0042451	<1e-4	ssr2009	GO:0042623	<1e-4	sll0441	GO:0005575	<1e-4	slr1990	GO:0044237	0.0242
slr7096	GO:0009394	<1e-4	slr0598	GO:0031323	<1e-4	sll1563	GO:0015077	<1e-4	slr0937	GO:0044425	<1e-4	sll1009	GO:0044237	0.0242
sll6053	GO:0009259	<1e-4	sll1219	GO:0009991	<1e-4	sll0350	GO:0022857	<1e-4	ssr1407	GO:0044425	<1e-4	slr0326	GO:0044237	0.0242
slr0148	GO:0071842	<1e-4	ssl1498	GO:0008610	<1e-4	sll0361	GO:0060089	<1e-4	sll0005	GO:0044425	<1e-4	sll1319	GO:0044237	0.0242
sll0174	GO:0019637	<1e-4	slr1573	GO:0042180	<1e-4	sll1272	GO:0005342	<1e-4	slr0975	GO:0044446	<1e-4	sll1142	GO:0044237	0.0242
ssl2138	GO:0052111	<1e-4	sll1891	GO:0006811	<1e-4	slr0358	GO:0043492	<1e-4	sll0478	GO:0044424	<1e-4	slr0816	GO:0044237	0.0242
slr0787	GO:0031326	<1e-4	slr0207	GO:0051188	<1e-4	slr5012	GO:0015293	<1e-4	slr1541	GO:0044446	<1e-4	ssr8013	GO:0044237	0.0242
sll0062	GO:0055082	<1e-4	sll1583	GO:0006631	<1e-4	slr0373	GO:0003674	<1e-4	sll7047	GO:0044464	<1e-4	slr0109	GO:0044237	0.0242
slr0304	GO:0005996	<1e-4	slr1636	GO:0016054	<1e-4	sll5090	GO:0022838	<1e-4	slr1023	GO:0044425	<1e-4	sll1036	GO:0044237	0.0242
slr1183	GO:0051716	<1e-4	sll1285	GO:0019362	<1e-4	sll1737	GO:0060089	<1e-4	sll0860	GO:0005575	<1e-4	slr0613	GO:0044237	0.0242
sll1132	GO:0006082	<1e-4	sll0839	GO:0050801	<1e-4	sll1132	GO:0015399	<1e-4	slr1726	GO:0044422	<1e-4	slr0813	GO:0044237	0.0242
ssl0467	GO:0009144	<1e-4	sll0688	GO:0009057	<1e-4	sll1109	GO:0016817	<1e-4	slr7010	GO:0044425	<1e-4	slr1114	GO:0044237	0.0242
sll1583	GO:0006720	<1e-4	slr0380	GO:0044283	<1e-4	sll1072	GO:0016818	<1e-4	sll0361	GO:0043226	<1e-4	slr5112	GO:0044237	0.0242
ssr3341	GO:0009991	<1e-4	slr0594	GO:0042180	<1e-4	ssl3379	GO:0022857	<1e-4	slr1385	GO:0005575	<1e-4	sll6052	GO:0044237	0.0242
slr1926	GO:0048522	<1e-4	slr0587	GO:0019752	<1e-4	sll1956	GO:0022838	<1e-4	sll0630	GO:0044444	<1e-4	sll0442	GO:0044237	0.0242
ssr2755	GO:0080090	<1e-4	slr0607	GO:0006066	<1e-4	slr0521	GO:0022838	<1e-4	ssr1391	GO:0044422	<1e-4	slr1577	GO:0044237	0.0242
sll0867	GO:0044283	<1e-4	sll0068	GO:0065007	<1e-4	sll0547	GO:0022832	<1e-4	sll1446	GO:0043226	<1e-4	sll1191	GO:0044237	0.0242
slr1865	GO:0046164	<1e-4	slr6101	GO:0046394	<1e-4	sll0822	GO:0022857	<1e-4	sll0230	GO:0044444	<1e-4	slr6106	GO:0044237	0.0242

ssl2717	GO:0044260	<1e-4	slr6094	GO:0051716	<1e-4	ssl7064	GO:0016462	<1e-4	sll0602	GO:0044425	<1e-4	sll1785	GO:0044237	0.0242
slr1563	GO:0043436	<1e-4	sll1583	GO:0033013	<1e-4	sll0810	GO:0022832	<1e-4	slr0400	GO:0044464	<1e-4	sll0552	GO:0044237	0.0242
slr1438	GO:0005996	<1e-4	sll7065	GO:0009150	<1e-4	sll1640	GO:0008171	<1e-4	slr0359	GO:0044424	<1e-4	sll1532	GO:0044237	0.0242
sll1510	GO:0006082	<1e-4	sll0319	GO:0009987	<1e-4	slr6081	GO:0022890	<1e-4	sll1232	GO:0043226	<1e-4	slr6016	GO:0044237	0.0242
ssr6020	GO:0043933	<1e-4	sll0513	GO:0009144	<1e-4	slr1537	GO:0005342	<1e-4	slr0110	GO:0044444	<1e-4	slr0667	GO:0044237	0.0242
slr0554	GO:0090304	<1e-4	slr2125	GO:0043436	<1e-4	sll0669	GO:0022857	<1e-4	ssl7042	GO:0005575	<1e-4	slr0914	GO:0044237	0.0242
sll0423	GO:0006732	<1e-4	ssl7048	GO:0048519	<1e-4	slr0586	GO:0022836	<1e-4	sll0243	GO:0044424	<1e-4	ssl7022	GO:0044237	0.0242
slr0241	GO:0048522	<1e-4	slr1033	GO:0006733	<1e-4	slr0810	GO:0022803	<1e-4	sll0355	GO:0044424	<1e-4	ssr5106	GO:0044237	0.0242
sll1396	GO:0009987	<1e-4	slr2070	GO:0044271	<1e-4	slr6074	GO:0015405	<1e-4	sll1853	GO:0005575	<1e-4	sll0157	GO:0044237	0.0242
sll0263	GO:0045184	<1e-4	sll0355	GO:0009199	<1e-4	ssl7022	GO:0015405	<1e-4	slr7080	GO:0005575	<1e-4	ssr2318	GO:0044237	0.0242
sll1902	GO:0046907	<1e-4	ssl5015	GO:0016053	<1e-4	ssl6035	GO:0022838	<1e-4	slr1566	GO:0043226	<1e-4	sll0031	GO:0044237	0.0242
slr0104	GO:0034641	<1e-4	slr1276	GO:0006163	<1e-4	slr0232	GO:0016462	<1e-4	ssl1045	GO:0043226	<1e-4	ssl7030	GO:0044237	0.0242
ssl0109	GO:0034654	<1e-4	sll0327	GO:0051186	<1e-4	sll0886	GO:0022832	<1e-4	sll0641	GO:0044424	<1e-4	slr0285	GO:0044237	0.0242
sll0985	GO:0019222	<1e-4	sll1373	GO:0016053	<1e-4	slr0151	GO:0016741	<1e-4	slr0888	GO:0044422	<1e-4	slr0509	GO:0044237	0.0242
sll0735	GO:0009889	<1e-4	sll0508	GO:0034641	<1e-4	sll0376	GO:0015291	<1e-4	slr6072	GO:0044424	<1e-4	slr0238	GO:0044237	0.0242
ssl6061	GO:0009059	<1e-4	slr1657	GO:0009893	<1e-4	sll0294	GO:0022836	<1e-4	slr0304	GO:0044446	<1e-4	slr1173	GO:0044237	0.0242
slr1568	GO:0010468	<1e-4	ssr7079	GO:0016054	<1e-4	slr0863	GO:0016741	<1e-4	sll0761	GO:0044464	<1e-4	slr1681	GO:0044237	0.0242
sll1151	GO:0071496	<1e-4	slr6100	GO:0009263	<1e-4	sll1583	GO:0060089	<1e-4	sll1381	GO:0044422	<1e-4	sll1784	GO:0044237	0.0242
ssr7072	GO:0050794	<1e-4	slr0514	GO:0044003	<1e-4	slr1773	GO:0022838	<1e-4	slr1056	GO:0044425	<1e-4	ssl2971	GO:0044237	0.0242
slr1813	GO:0009259	<1e-4	slr1187	GO:0009263	<1e-4	slr0751	GO:0022838	<1e-4	slr0971	GO:0005575	<1e-4	sll0565	GO:0044237	0.0242
sll1766	GO:0009144	<1e-4	slr1218	GO:0019637	<1e-4	slr7014	GO:0043167	<1e-4	sll1036	GO:0044425	<1e-4	slr1431	GO:0003723	0.0241
slr1437	GO:0051716	<1e-4	ssl3379	GO:0019637	<1e-4	sll1654	GO:0015267	<1e-4	slr1636	GO:0044422	<1e-4	slr1644	GO:0003723	0.0241
slr6071	GO:0044003	<1e-4	sll0980	GO:0080090	<1e-4	slr2144	GO:0016818	<1e-4	ssl5064	GO:0044425	<1e-4	slr1173	GO:0005515	0.024
slr2125	GO:0072527	<1e-4	slr1923	GO:0008150	<1e-4	slr1568	GO:0022892	<1e-4	slr6103	GO:0044464	<1e-4	slr1752	GO:0005515	0.024
ssr7093	GO:0046907	<1e-4	sll0553	GO:0009165	<1e-4	ssr2422	GO:0022857	<1e-4	sll1693	GO:0044424	<1e-4	sll1388	GO:0005515	0.024
slr1807	GO:0044260	<1e-4	slr0049	GO:0009987	<1e-4	ssl2138	GO:0043167	<1e-4	sll1934	GO:0044424	<1e-4	slr1114	GO:0005515	0.024
ssr6020	GO:0046128	<1e-4	sll1389	GO:0046907	<1e-4	slr0801	GO:0015291	<1e-4	slr0812	GO:0005575	<1e-4	ssr6027	GO:0005515	0.024
sll1106	GO:0043648	<1e-4	sll0688	GO:0046907	<1e-4	slr0596	GO:0015399	<1e-4	sll1680	GO:0044422	<1e-4	slr0453	GO:0005515	0.024
sll1411	GO:0051716	<1e-4	slr1177	GO:0006066	<1e-4	slr1101	GO:0022890	<1e-4	slr5021	GO:0005575	<1e-4	ssr1425	GO:0005515	0.024
slr1441	GO:0031640	<1e-4	sll0024	GO:0019219	<1e-4	sll1063	GO:0043167	<1e-4	sll0314	GO:0043226	<1e-4	slr1875	GO:0005515	0.024
slr7010	GO:0048523	<1e-4	slr7083	GO:0050801	<1e-4	slr2005	GO:0022891	<1e-4	slr7094	GO:0044464	<1e-4	slr0948	GO:0005515	0.024
slr1260	GO:0009889	<1e-4	slr1644	GO:0018130	<1e-4	slr1087	GO:0003674	<1e-4	sll0280	GO:0005575	<1e-4	sll5003	GO:0005515	0.024
sll5097	GO:0006066	<1e-4	sll0639	GO:0005996	<1e-4	slr0337	GO:0015075	<1e-4	sll1654	GO:0044446	<1e-4	ssr1155	GO:0005515	0.024
slr1258	GO:0046394	<1e-4	slr0545	GO:0044275	<1e-4	slr0313	GO:0060089	<1e-4	slr7014	GO:0005575	<1e-4	sll1319	GO:0005515	0.024
slr1407	GO:0009893	<1e-4	slr1087	GO:0009892	<1e-4	sll1681	GO:0022891	<1e-4	sll0449	GO:0005575	<1e-4	ssl7042	GO:0005515	0.024
slr0813	GO:0048878	<1e-4	sll0174	GO:0006631	<1e-4	slr1537	GO:0022890	<1e-4	sll1608	GO:0044446	<1e-4	sll1486	GO:0005515	0.024
slr0569	GO:0050896	<1e-4	sll1062	GO:0009308	<1e-4	sll0861	GO:0022832	<1e-4	ssl0750	GO:0044446	<1e-4	sll1068	GO:0005515	0.024
slr0971	GO:0009117	<1e-4	slr1173	GO:0019222	<1e-4	ssl3379	GO:0022838	<1e-4	slr1811	GO:0044464	<1e-4	slr5073	GO:0005515	0.024
slr1260	GO:0009126	<1e-4	slr1315	GO:0051716	<1e-4	sll5097	GO:0060089	<1e-4	slr5077	GO:0044422	<1e-4	sll7028	GO:0005515	0.024
sll1388	GO:0008610	<1e-4	ssr0335	GO:0051179	<1e-4	slr0588	GO:0022890	<1e-4	ssr5106	GO:0044425	<1e-4	ssl6035	GO:0005515	0.024
ssl0461	GO:0051716	<1e-4	slr0586	GO:0019219	<1e-4	slr5118	GO:0015293	<1e-4	sll0742	GO:0043226	<1e-4	ssr5011	GO:0005515	0.024
sll0625	GO:0052188	<1e-4	sll1348	GO:0042455	<1e-4	slr1611	GO:0015399	<1e-4	slr1107	GO:0044425	<1e-4	slr0416	GO:0005515	0.024
slr0455	GO:0051171	<1e-4	ssl5091	GO:0006793	<1e-4	ssl8028	GO:0015293	<1e-4	slr0654	GO:0044424	<1e-4	slr1142	GO:0005515	0.024
slr1886	GO:0046164	<1e-4	slr0423	GO:0046128	<1e-4	slr6065	GO:0015291	<1e-4	slr0104	GO:0044464	<1e-4	slr1474	GO:0005515	0.024
sll1151	GO:0006793	<1e-4	sll2006	GO:0042180	<1e-4	slr1431	GO:0022891	<1e-4	slr5053	GO:0044424	<1e-4	sll0565	GO:0005515	0.024
ssl1690	GO:0072524	<1e-4	slr0709	GO:0044271	<1e-4	sll0060	GO:0003674	<1e-4	sll0532	GO:0043226	<1e-4	slr1935	GO:0005515	0.024
slr0300	GO:0009150	<1e-4	slr0909	GO:0006811	<1e-4	slr6072	GO:0022857	<1e-4	sll1785	GO:0005575	<1e-4	slr1628	GO:0005515	0.024
slr2103	GO:0043933	<1e-4	ssl0353	GO:0010556	<1e-4	slr0712	GO:0015077	<1e-4	sll1961	GO:0044422	<1e-4	slr2060	GO:0005515	0.024
slr1162	GO:0006066	<1e-4	slr6101	GO:0009161	<1e-4	sll1251	GO:0015077	<1e-4	sll1380	GO:0043226	<1e-4	slr1513	GO:0005515	0.024
sll7086	GO:0022607	<1e-4	sll1348	GO:0051246	<1e-4	slr0334	GO:0015291	<1e-4	slr0456	GO:0043226	<1e-4	ssl2384	GO:0005515	0.024
slr5016	GO:0009144	<1e-4	slr0238	GO:0042180	<1e-4	slr0408	GO:0042623	<1e-4	slr0053	GO:0044446	<1e-4	slr0270	GO:0005515	0.024
sll1691	GO:0051716	<1e-4	ssr6083	GO:0048518	<1e-4	ssr6003	GO:0022890	<1e-4	slr7013	GO:0044424	<1e-4	slr0656	GO:0005515	0.024
slr1814	GO:0019751	<1e-4	slr6091	GO:0042180	<1e-4	slr5112	GO:0042623	<1e-4	sll1730	GO:0044422	<1e-4	slr1790	GO:0005515	0.024
ssr1768	GO:0043436	<1e-4	ssr2802	GO:0044283	<1e-4	slr0699	GO:0015267	<1e-4	slr5102	GO:0005575	<1e-4	ssr7079	GO:0005515	0.024

slr1998	GO:0042180	<1e-4	sll1424	GO:0080090	<1e-4	slr1507	GO:0015405	<1e-4	slr1819	GO:0044422	<1e-4	slr1704	GO:0005515	0.024
slr1206	GO:0051716	<1e-4	slr6101	GO:0006631	<1e-4	slr1103	GO:0042623	<1e-4	sll0441	GO:0044422	<1e-4	slr6091	GO:0005515	0.024
sll7089	GO:0019752	<1e-4	slr6068	GO:0009150	<1e-4	sll0177	GO:0016817	<1e-4	ssl5065	GO:0044425	<1e-4	ssr6030	GO:0005515	0.024
ssl2064	GO:0051171	<1e-4	sll1769	GO:0010556	<1e-4	slr1162	GO:0016818	<1e-4	slr1178	GO:0044444	<1e-4	ssr3129	GO:0005515	0.024
sll0297	GO:0045184	<1e-4	sll1530	GO:0071496	<1e-4	slr0869	GO:0022836	<1e-4	slr0609	GO:0005575	<1e-4	slr8044	GO:0005515	0.024
sll0505	GO:0009394	<1e-4	slr0569	GO:0009309	<1e-4	sll1934	GO:0022892	<1e-4	ssl1464	GO:0044425	<1e-4	sll1609	GO:0005515	0.024
slr0249	GO:0046128	<1e-4	slr1668	GO:0055086	<1e-4	ssr6020	GO:0003674	<1e-4	slr1179	GO:0005575	<1e-4	ssl2162	GO:0005515	0.024
slr0397	GO:0009059	<1e-4	slr7057	GO:0010468	<1e-4	slr7060	GO:0022892	<1e-4	ssl5064	GO:0005575	<1e-4	slr0516	GO:0005515	0.024
ssr2754	GO:0009309	<1e-4	slr0483	GO:0022411	<1e-4	slr1819	GO:0015267	<1e-4	slr1648	GO:0043226	<1e-4	sll1049	GO:0005515	0.024
sll1052	GO:0048878	<1e-4	slr0318	GO:0006732	<1e-4	slr0596	GO:0016818	<1e-4	sll1040	GO:0044444	<1e-4	sll0496	GO:0005515	0.024
slr0263	GO:0071840	<1e-4	sll0756	GO:0050896	<1e-4	ssr6078	GO:0008171	<1e-4	sll0156	GO:0044422	<1e-4	ssl5103	GO:0005515	0.024
sll1654	GO:0009144	<1e-4	sll1609	GO:0009260	<1e-4	slr1258	GO:0015399	<1e-4	sll0266	GO:0005575	<1e-4	sll1464	GO:0005515	0.024
sll1766	GO:0019222	<1e-4	sll0980	GO:0009059	<1e-4	slr1827	GO:0022838	<1e-4	slr1032	GO:0044422	<1e-4	ssl3829	GO:0005515	0.024
slr1619	GO:0042180	<1e-4	slr0408	GO:0008610	<1e-4	slr0712	GO:0022836	<1e-4	slr5119	GO:0044444	<1e-4	ssl6092	GO:0005515	0.024
ssr1473	GO:0009893	<1e-4	ssr1528	GO:0009893	<1e-4	ssr0692	GO:0015077	<1e-4	slr7099	GO:0043226	<1e-4	sll1151	GO:0005515	0.024
slr0287	GO:0051179	<1e-4	slr1537	GO:0048519	<1e-4	slr1690	GO:0005342	<1e-4	sll7043	GO:0044446	<1e-4	slr6068	GO:0005515	0.024
sll0761	GO:0019752	<1e-4	sll1761	GO:0080090	<1e-4	sll0572	GO:0043492	<1e-4	slr7092	GO:0044422	<1e-4	slr0637	GO:0005515	0.024
slr7060	GO:0019637	<1e-4	slr0082	GO:0009141	<1e-4	ssr3402	GO:0022803	<1e-4	slr1391	GO:0044446	<1e-4	slr0300	GO:0005515	0.024
slr5018	GO:0043170	<1e-4	sll1954	GO:0044255	<1e-4	sll0815	GO:0015077	<1e-4	sll7031	GO:0044422	<1e-4	sll6055	GO:0005515	0.024
slr1951	GO:0046907	<1e-4	slr1900	GO:0019752	<1e-4	sll1350	GO:0016741	<1e-4	ssl1577	GO:0044444	<1e-4	slr2110	GO:0005515	0.024
sll1158	GO:0006753	<1e-4	sll0023	GO:0008150	<1e-4	ssr1768	GO:0022838	<1e-4	slr0930	GO:0044464	<1e-4	ssl0350	GO:0005515	0.024
ssr2781	GO:0046128	<1e-4	slr0645	GO:0090304	<1e-4	sll0167	GO:0015267	<1e-4	ssr1041	GO:0043226	<1e-4	slr1886	GO:0005515	0.024
sll6053	GO:0006091	<1e-4	sll1995	GO:0050801	<1e-4	slr1737	GO:0016817	<1e-4	ssr2962	GO:0044422	<1e-4	slr1819	GO:0005515	0.024
slr0978	GO:0043549	<1e-4	sll1250	GO:0006163	<1e-4	sll0405	GO:0015291	<1e-4	sll1853	GO:0044446	<1e-4	slr2103	GO:0005515	0.024
slr0491	GO:0072521	<1e-4	ssl0353	GO:0072527	<1e-4	sll1022	GO:0022890	<1e-4	slr6021	GO:0044422	<1e-4	sll1024	GO:0005515	0.024
sll1233	GO:0034660	<1e-4	sll0286	GO:0048523	<1e-4	slr0285	GO:0015075	<1e-4	sll1049	GO:0005575	<1e-4	slr5087	GO:0005515	0.024
ssr1528	GO:0005996	<1e-4	slr6004	GO:0051179	<1e-4	sll0296	GO:0022838	<1e-4	slr2115	GO:0005575	<1e-4	sll1979	GO:0005515	0.024
ssr2803	GO:0072521	<1e-4	sll1024	GO:0019752	<1e-4	sll8001	GO:0005342	<1e-4	sll1240	GO:0044464	<1e-4	slr2049	GO:0005515	0.024
ssr7017	GO:0009987	<1e-4	slr0376	GO:0042455	<1e-4	sll0063	GO:0016462	<1e-4	slr1886	GO:0044444	<1e-4	ssl2064	GO:0005515	0.024
slr0496	GO:0009056	<1e-4	slr1222	GO:0009161	<1e-4	slr2025	GO:0016817	<1e-4	slr1437	GO:0044425	<1e-4	ssl1300	GO:0005515	0.024
slr1273	GO:0046128	<1e-4	sll7050	GO:0043933	<1e-4	sll0102	GO:0043492	<1e-4	sll0310	GO:0005575	<1e-4	slr1780	GO:0005515	0.024
ssl3451	GO:0090304	<1e-4	sll1586	GO:0051234	<1e-4	slr1384	GO:0022857	<1e-4	ssl2065	GO:0043226	<1e-4	ssr0109	GO:0005515	0.024
ssr2803	GO:0009117	<1e-4	slr7060	GO:0071554	<1e-4	slr0962	GO:0015293	<1e-4	slr1674	GO:0043226	<1e-4	sll7069	GO:0005515	0.024
slr1353	GO:0044260	<1e-4	sll1608	GO:0042180	<1e-4	sll1640	GO:0022836	<1e-4	slr0480	GO:0043226	<1e-4	ssl5008	GO:0005515	0.024
ssr6062	GO:0043436	<1e-4	slr0408	GO:0008150	<1e-4	sll0981	GO:0015075	<1e-4	slr5126	GO:0044422	<1e-4	slr1915	GO:0005515	0.024
slr6005	GO:0034660	<1e-4	sll0414	GO:0050794	<1e-4	slr5101	GO:0008171	<1e-4	sll0062	GO:0044464	<1e-4	sll1640	GO:0005515	0.024
sll0888	GO:0006576	<1e-4	sll0242	GO:0071842	<1e-4	slr0386	GO:0016746	<1e-4	slr0601	GO:0044422	<1e-4	slr1507	GO:0005515	0.024
slr6087	GO:0048878	<1e-4	slr1025	GO:0051234	<1e-4	sll1658	GO:0015291	<1e-4	sll5033	GO:0005575	<1e-4	ssl7022	GO:0005515	0.024
ssl3549	GO:0006631	<1e-4	slr7092	GO:0022607	<1e-4	ssl0467	GO:0015075	<1e-4	ssr1256	GO:0044425	<1e-4	sll1714	GO:0005515	0.024
sll0762	GO:0031323	<1e-4	sll0780	GO:0019751	<1e-4	slr2003	GO:0022836	<1e-4	slr0878	GO:0043226	<1e-4	sll0230	GO:0005515	0.024
ssr2439	GO:0009150	<1e-4	sll0031	GO:0031640	<1e-4	slr1895	GO:0008171	<1e-4	ssr6020	GO:0044446	<1e-4	slr1206	GO:0005515	0.024
slr1513	GO:0044260	<1e-4	slr5016	GO:0009057	<1e-4	ssr6083	GO:0043492	<1e-4	sll0225	GO:0005575	<1e-4	slr0238	GO:0005515	0.024
slr1812	GO:0065007	<1e-4	sll0736	GO:0043170	<1e-4	sll7067	GO:0015405	<1e-4	slr1074	GO:0005575	<1e-4	slr0588	GO:0005515	0.024
slr6064	GO:0046164	<1e-4	slr0971	GO:0050896	<1e-4	sll0930	GO:0015405	<1e-4	sll0658	GO:0043226	<1e-4	ssl5091	GO:0005515	0.024
slr2118	GO:0071554	<1e-4	slr0596	GO:0009144	<1e-4	sll1858	GO:0043492	<1e-4	slr1571	GO:0044446	<1e-4	sll1192	GO:0005515	0.024
sll0905	GO:0046483	<1e-4	sll0860	GO:0044003	<1e-4	ssl7021	GO:0015075	<1e-4	slr0569	GO:0044464	<1e-4	slr0613	GO:0005515	0.024
slr0869	GO:0009308	<1e-4	sll1252	GO:0051818	<1e-4	sll1319	GO:0015405	<1e-4	sll0779	GO:0044424	<1e-4	sll0608	GO:0005515	0.024
slr1222	GO:0010468	<1e-4	slr1396	GO:0044248	<1e-4	slr1464	GO:0015399	<1e-4	ssl2595	GO:0044464	<1e-4	slr1170	GO:0005515	0.024
sll1965	GO:0009165	<1e-4	slr1737	GO:0016053	<1e-4	sll1652	GO:0016746	<1e-4	ssr5121	GO:0005575	<1e-4	slr8021	GO:0005515	0.024
slr1170	GO:0072527	<1e-4	slr1474	GO:0090304	<1e-4	ssr2553	GO:0005342	<1e-4	sll0499	GO:0044464	<1e-4	slr1174	GO:0005515	0.024
sll0103	GO:0006082	<1e-4	sll0505	GO:0006733	<1e-4	sll0327	GO:0008171	<1e-4	sll1541	GO:0044444	<1e-4	slr0459	GO:0005515	0.024
slr1462	GO:0090304	<1e-4	sll0984	GO:0019752	<1e-4	sll0762	GO:0022832	<1e-4	sll1906	GO:0043226	<1e-4	sll7090	GO:0005515	0.024
ssl2420	GO:0001932	<1e-4	slr1169	GO:0006082	<1e-4	sll1526	GO:0016746	<1e-4	sll0410	GO:0044464	<1e-4	slr5013	GO:0005515	0.024
slr0765	GO:0009126	<1e-4	sll0558	GO:0044282	<1e-4	slr1104	GO:0015267	<1e-4	sll7065	GO:0044425	<1e-4	sll1531	GO:0005515	0.024

sll7090	GO:0006066	<1e-4	sll1562	GO:0055114	<1e-4	sll1702	GO:0015075	<1e-4	sll7028	GO:0044444	<1e-4	sll0479	GO:0005515	0.024
ssl0352	GO:0006732	<1e-4	slr8044	GO:0048878	<1e-4	slr0699	GO:0008171	<1e-4	sll0394	GO:0044444	<1e-4	sll0939	GO:0005515	0.024
ssl3829	GO:0071554	<1e-4	sll0102	GO:0046164	<1e-4	sll0360	GO:0022857	<1e-4	sll0188	GO:0043226	<1e-4	slr0730	GO:0005515	0.024
slr0408	GO:0051186	<1e-4	slr6016	GO:0006066	<1e-4	slr2032	GO:0016817	<1e-4	slr1623	GO:0044422	<1e-4	ssl8028	GO:0005515	0.024
sll1272	GO:0050896	<1e-4	slr6015	GO:0044260	<1e-4	sll8001	GO:0015293	<1e-4	slr0634	GO:0044425	<1e-4	sll0905	GO:0005515	0.024
ssr8013	GO:0050794	<1e-4	sll0249	GO:0009059	<1e-4	sll1652	GO:0022803	<1e-4	sll1911	GO:0044422	<1e-4	slr7100	GO:0005515	0.024
sll0243	GO:0009987	<1e-4	ssr1552	GO:0031323	<1e-4	slr0326	GO:0015405	<1e-4	slr6101	GO:0044425	<1e-4	slr0325	GO:0005515	0.024
slr1886	GO:0006576	<1e-4	slr1168	GO:0051234	<1e-4	slr0023	GO:0008171	<1e-4	slr1429	GO:0044464	<1e-4	ssr0761	GO:0005515	0.024
sll0775	GO:0009132	<1e-4	ssr3571	GO:0006721	<1e-4	sll8032	GO:0005342	<1e-4	sll1344	GO:0005575	<1e-4	sll1373	GO:0005515	0.024
slr1073	GO:0009142	<1e-4	slr0989	GO:0042451	<1e-4	slr6101	GO:0043167	<1e-4	sll1121	GO:0005575	<1e-4	sll7006	GO:0005515	0.024
slr0941	GO:0009165	<1e-4	slr1241	GO:0019752	<1e-4	sll0984	GO:0015267	<1e-4	slr0337	GO:0044424	<1e-4	ssl1690	GO:0005515	0.024
sll8027	GO:0071496	<1e-4	slr1230	GO:0065007	<1e-4	sll1638	GO:0016741	<1e-4	sll1321	GO:0043226	<1e-4	slr0273	GO:0005515	0.024
ssl5064	GO:0050794	<1e-4	sll0810	GO:0044271	<1e-4	sll1573	GO:0015075	<1e-4	ssl6061	GO:0044446	<1e-4	sll0098	GO:0005515	0.024
sll1722	GO:0009117	<1e-4	sll1232	GO:0033013	<1e-4	sll0803	GO:0022857	<1e-4	sll0996	GO:0005575	<1e-4	slr1788	GO:0004518	0.0234
sll0564	GO:0009308	<1e-4	slr0730	GO:0006066	<1e-4	ssl7048	GO:0015075	<1e-4	slr6022	GO:0044425	<1e-4	slr1885	GO:0004518	0.0234
sll1573	GO:0006721	<1e-4	slr1935	GO:0060255	<1e-4	slr1169	GO:0022803	<1e-4	slr0479	GO:0044425	<1e-4	slr1230	GO:0004518	0.0234
slr1774	GO:0006082	<1e-4	slr0588	GO:0042455	<1e-4	sll8032	GO:0022891	<1e-4	ssr1473	GO:0005575	<1e-4	sll1613	GO:0004518	0.0234
sll0857	GO:0009263	<1e-4	sll1036	GO:0043648	<1e-4	ssl1046	GO:0015267	<1e-4	sll1938	GO:0005575	<1e-4	sll0944	GO:0004518	0.0234
sll0423	GO:0034641	<1e-4	slr7095	GO:0010556	<1e-4	sll0822	GO:0005342	<1e-4	ssr2802	GO:0044424	<1e-4	slr2011	GO:0004518	0.0234
ssl7039	GO:0019222	<1e-4	slr0609	GO:0019752	<1e-4	slr1315	GO:0003674	<1e-4	slr0039	GO:0044464	<1e-4	ssr1765	GO:0004518	0.0234
slr7094	GO:0006720	<1e-4	sll1658	GO:0006753	<1e-4	slr6091	GO:0022857	<1e-4	sll0443	GO:0005575	<1e-4	slr1266	GO:0004518	0.0234
sll1606	GO:0019219	<1e-4	slr1383	GO:0072521	<1e-4	slr2048	GO:0015077	<1e-4	sll0909	GO:0005575	<1e-4	sll0243	GO:0004518	0.0234
sll0553	GO:0090304	<1e-4	sll1086	GO:0018130	<1e-4	ssr3122	GO:0016741	<1e-4	slr7023	GO:0044425	<1e-4	sll0237	GO:0004518	0.0234
sll0997	GO:0006766	<1e-4	slr0655	GO:0016054	<1e-4	ssr3304	GO:0042623	<1e-4	sll0926	GO:0005575	<1e-4	ssl0352	GO:0004518	0.0234
slr0610	GO:0046128	<1e-4	slr1557	GO:0046394	<1e-4	slr1082	GO:0022836	<1e-4	slr1098	GO:0005575	<1e-4	sll0085	GO:0004518	0.0234
slr1638	GO:0044275	<1e-4	slr0801	GO:0006631	<1e-4	slr1307	GO:0015399	<1e-4	slr0351	GO:0044422	<1e-4	sll1835	GO:0004518	0.0234
slr0870	GO:0006163	<1e-4	slr1544	GO:0050801	<1e-4	sll0413	GO:0043167	<1e-4	sll0298	GO:0044444	<1e-4	sll0785	GO:0004518	0.0234
slr1530	GO:0048519	<1e-4	ssl3383	GO:0009117	<1e-4	ssl2420	GO:0016818	<1e-4	ssl2162	GO:0044464	<1e-4	slr1964	GO:0004518	0.0234
slr0108	GO:0052188	<1e-4	sll0815	GO:0072528	<1e-4	slr0728	GO:0015399	<1e-4	slr1854	GO:0044464	<1e-4	ssr2615	GO:0004518	0.0234
sll1880	GO:0019752	<1e-4	slr7026	GO:0009057	<1e-4	slr1917	GO:0022857	<1e-4	sll0376	GO:0043226	<1e-4	slr0723	GO:0004518	0.0234
sll1009	GO:0044275	<1e-4	sll1380	GO:0006631	<1e-4	slr0876	GO:0016746	<1e-4	slr0962	GO:0005575	<1e-4	slr0941	GO:0004518	0.0234
slr0645	GO:0048878	<1e-4	sll1464	GO:0043412	<1e-4	slr0157	GO:0042623	<1e-4	slr1667	GO:0005575	<1e-4	sll1656	GO:0004518	0.0234
ssl1520	GO:0008150	<1e-4	slr1071	GO:0009987	<1e-4	slr5112	GO:0022838	<1e-4	sll1071	GO:0044425	<1e-4	sll1262	GO:0004518	0.0234
ssl5129	GO:0050789	<1e-4	sll5128	GO:0043412	<1e-4	slr1852	GO:0015399	<1e-4	slr1259	GO:0044464	<1e-4	slr0386	GO:0004518	0.0234
sll1938	GO:0046164	<1e-4	slr1535	GO:0044271	<1e-4	ssl5095	GO:0022892	<1e-4	slr0456	GO:0044424	<1e-4	sll1949	GO:0004518	0.0234
sll0590	GO:0006793	<1e-4	ssr2317	GO:0022411	<1e-4	slr1287	GO:0016741	<1e-4	sll1495	GO:0044444	<1e-4	sll0497	GO:0004518	0.0234
ssl5008	GO:0050896	<1e-4	ssr6019	GO:0010468	<1e-4	sll1072	GO:0022803	<1e-4	ssr6020	GO:0043226	<1e-4	sll1315	GO:0004518	0.0234
sll0325	GO:0006721	<1e-4	slr0291	GO:0046907	<1e-4	slr0921	GO:0022891	<1e-4	slr0769	GO:0005575	<1e-4	slr0006	GO:0004518	0.0234
sll0008	GO:0009150	<1e-4	slr0249	GO:0019222	<1e-4	slr0491	GO:0022857	<1e-4	sll1121	GO:0044464	<1e-4	slr1210	GO:0004518	0.0234
slr0207	GO:0009141	<1e-4	slr2144	GO:0006631	<1e-4	slr5118	GO:0022891	<1e-4	sll1488	GO:0044446	<1e-4	sll0007	GO:0004518	0.0234
sll1960	GO:0006066	<1e-4	slr1917	GO:0009892	<1e-4	sll5132	GO:0015293	<1e-4	slr1513	GO:0043226	<1e-4	sll1006	GO:0004518	0.0234
sll6053	GO:0050896	<1e-4	slr2120	GO:0034641	<1e-4	slr0489	GO:0042623	<1e-4	sll0981	GO:0005575	<1e-4	ssr2962	GO:0004518	0.0234
slr0964	GO:0009132	<1e-4	sll0449	GO:0042451	<1e-4	sll1464	GO:0016817	<1e-4	slr8022	GO:0044424	<1e-4	sll1040	GO:0008233	0.0233
sll0843	GO:0006576	<1e-4	slr1753	GO:0046907	<1e-4	sll1162	GO:0015291	<1e-4	sll0549	GO:0044444	<1e-4	sll1225	GO:0008233	0.0233
ssl1498	GO:0055114	<1e-4	sll0268	GO:0044106	<1e-4	slr7024	GO:0015075	<1e-4	sll8019	GO:0044424	<1e-4	sll0297	GO:0008233	0.0233
sll0473	GO:0006220	<1e-4	slr0489	GO:0019637	<1e-4	sll1002	GO:0042623	<1e-4	slr6012	GO:0044444	<1e-4	slr2115	GO:0008233	0.0233
slr0723	GO:0048522	<1e-4	slr0103	GO:0050794	<1e-4	slr5118	GO:0022857	<1e-4	sll0932	GO:0044464	<1e-4	slr0680	GO:0008233	0.0233
slr2120	GO:0022411	<1e-4	slr1616	GO:0065007	<1e-4	slr0348	GO:0016741	<1e-4	sll1414	GO:0005575	<1e-4	slr1495	GO:0016772	0.0233
slr0655	GO:0034641	<1e-4	sll0414	GO:0019362	<1e-4	slr0263	GO:0022838	<1e-4	sll6054	GO:0044424	<1e-4	ssr2781	GO:0016772	0.0233
ssr3532	GO:0080090	<1e-4	sll1009	GO:0006082	<1e-4	slr0869	GO:0005342	<1e-4	slr0722	GO:0043226	<1e-4	slr1033	GO:0008233	0.0233
sll0198	GO:0006576	<1e-4	ssl3142	GO:0009889	<1e-4	slr7101	GO:0008171	<1e-4	ssr0332	GO:0044464	<1e-4	slr1895	GO:0008233	0.0233
sll1477	GO:0019362	<1e-4	sll0444	GO:0065007	<1e-4	slr2115	GO:0022803	<1e-4	sll0608	GO:0044422	<1e-4	slr0848	GO:0016772	0.0233
slr0397	GO:0044248	<1e-4	sll0886	GO:0016054	<1e-4	slr1576	GO:0016462	<1e-4	sll0872	GO:0044422	<1e-4	sll1424	GO:0016772	0.0233
slr7100	GO:0071840	<1e-4	sll1289	GO:0009124	<1e-4	slr0848	GO:0042623	<1e-4	slr0031	GO:0005575	<1e-4	slr0483	GO:0008233	0.0233

sll0208	GO:0034641	<1e-4	sll8011	GO:0048522	<1e-4	sll7070	GO:0015075	<1e-4	slr5018	GO:0005575	<1e-4	sll1239	GO:0008233	0.0233
slr1169	GO:0050801	<1e-4	slr1854	GO:0010556	<1e-4	slr6063	GO:0003674	<1e-4	slr0954	GO:0005575	<1e-4	sll0449	GO:0008233	0.0233
slr0771	GO:0022607	<1e-4	slr6008	GO:0090304	<1e-4	slr0769	GO:0005342	<1e-4	slr0948	GO:0044444	<1e-4	sll0595	GO:0008233	0.0233
ssl2138	GO:0051188	<1e-4	sll1681	GO:0006631	<1e-4	slr1624	GO:0043167	<1e-4	ssr3571	GO:0043226	<1e-4	sll1352	GO:0008233	0.0233
slr1071	GO:0055082	<1e-4	sll1925	GO:0050801	<1e-4	slr1541	GO:0016462	<1e-4	sll1131	GO:0044464	<1e-4	sll0359	GO:0008233	0.0233
sll0327	GO:0009991	<1e-4	slr0668	GO:0044275	<1e-4	slr1547	GO:0015077	<1e-4	slr0845	GO:0043226	<1e-4	slr0729	GO:0008233	0.0233
slr5087	GO:0022411	<1e-4	slr6100	GO:0043436	<1e-4	slr6104	GO:0022803	<1e-4	slr1681	GO:0005575	<1e-4	sll0787	GO:0008233	0.0233
ssl0109	GO:0009893	<1e-4	slr0919	GO:0031323	<1e-4	slr0006	GO:0022832	<1e-4	slr1957	GO:0044424	<1e-4	ssr2201	GO:0016772	0.0233
sll6052	GO:0010468	<1e-4	sll1265	GO:0044260	<1e-4	slr0581	GO:0016818	<1e-4	slr1970	GO:0044444	<1e-4	sll0688	GO:0008233	0.0233
slr1530	GO:0042180	<1e-4	sll0781	GO:0019222	<1e-4	slr1033	GO:0022890	<1e-4	slr2003	GO:0044444	<1e-4	slr1128	GO:0008233	0.0233
slr0407	GO:0042455	<1e-4	slr1236	GO:0009144	<1e-4	sll1634	GO:0016817	<1e-4	sll1021	GO:0044422	<1e-4	ssl3291	GO:0016772	0.0233
slr1814	GO:0044271	<1e-4	sll1464	GO:0006220	<1e-4	slr0325	GO:0022838	<1e-4	slr0551	GO:0044422	<1e-4	sll1054	GO:0008233	0.0233
sll0478	GO:0010468	<1e-4	sll1095	GO:0006721	<1e-4	sll0309	GO:0005342	<1e-4	sll0775	GO:0044444	<1e-4	sll0763	GO:0016772	0.0233
ssl5095	GO:0016054	<1e-4	slr2003	GO:0031323	<1e-4	slr0689	GO:0003674	<1e-4	slr1276	GO:0044425	<1e-4	sll1119	GO:0016772	0.0233
sll1002	GO:0010556	<1e-4	ssl5099	GO:0006811	<1e-4	sgl0001	GO:0003674	<1e-4	slr2144	GO:0044424	<1e-4	sll1350	GO:0008233	0.0233
slr1799	GO:0009057	<1e-4	ssr6085	GO:0048878	<1e-4	slr6081	GO:0015291	<1e-4	sll0783	GO:0044422	<1e-4	sll0810	GO:0008233	0.0233
slr0318	GO:0009987	<1e-4	ssl2749	GO:0019222	<1e-4	slr0208	GO:0022832	<1e-4	slr1275	GO:0005575	<1e-4	sll1873	GO:0008233	0.0233
slr0651	GO:0065007	<1e-4	slr0818	GO:0044260	<1e-4	sll1571	GO:0022836	<1e-4	slr1315	GO:0043226	<1e-4	sll1752	GO:0008233	0.0233
slr5023	GO:0043933	<1e-4	sll1583	GO:0071554	<1e-4	slr1116	GO:0015267	<1e-4	slr1659	GO:0005575	<1e-4	sll1267	GO:0008233	0.0233
sll0564	GO:0048519	<1e-4	ssl2814	GO:0009893	<1e-4	slr1270	GO:0043167	<1e-4	sll0710	GO:0044425	<1e-4	sll1072	GO:0008233	0.0233
slr1235	GO:0051186	<1e-4	slr0610	GO:0031640	<1e-4	sll0023	GO:0016817	<1e-4	sll0327	GO:0044424	<1e-4	sll0888	GO:0008233	0.0233
sgl0001	GO:0044271	<1e-4	sll0678	GO:0050896	<1e-4	slr1196	GO:0042623	<1e-4	sll1289	GO:0044425	<1e-4	slr1762	GO:0008233	0.0233
ssr2755	GO:0044260	<1e-4	slr1670	GO:0044282	<1e-4	sll1040	GO:0022832	<1e-4	slr0362	GO:0044424	<1e-4	sll1344	GO:0008233	0.0233
sll1702	GO:0009308	<1e-4	sll1131	GO:0052188	<1e-4	sll1541	GO:0022890	<1e-4	slr0376	GO:0005575	<1e-4	slr1827	GO:0008233	0.0233
sll1660	GO:0050794	<1e-4	slr0192	GO:0019222	<1e-4	slr0634	GO:0043492	<1e-4	ssl7038	GO:0044422	<1e-4	sll0156	GO:0008233	0.0233
slr1895	GO:0065007	<1e-4	ssl7042	GO:0009263	<1e-4	slr0263	GO:0016462	<1e-4	sll1095	GO:0044425	<1e-4	sll0614	GO:0008233	0.0233
slr0196	GO:0008610	<1e-4	slr1053	GO:0055086	<1e-4	slr0935	GO:0015291	<1e-4	slr1603	GO:0044446	<1e-4	sll0647	GO:0016772	0.0233
ssl5098	GO:0045184	<1e-4	slr0053	GO:0072528	<1e-4	sll0736	GO:0022890	<1e-4	ssl1690	GO:0044444	<1e-4	slr0789	GO:0008233	0.0233
sll0024	GO:0005996	<1e-4	slr5127	GO:0050801	<1e-4	sll1902	GO:0008171	<1e-4	ssr6099	GO:0044444	<1e-4	slr0654	GO:0008233	0.0233
slr1676	GO:0048518	<1e-4	sll1193	GO:0006753	<1e-4	sll0448	GO:0015399	<1e-4	sll0423	GO:0005575	<1e-4	slr0249	GO:0008233	0.0233
ssr5092	GO:0048519	<1e-4	sll0854	GO:0060255	<1e-4	sll0423	GO:0022892	<1e-4	slr1174	GO:0044444	<1e-4	slr0596	GO:0008233	0.0233
ssr6048	GO:0019219	<1e-4	slr6044	GO:0048522	<1e-4	slr6081	GO:0022838	<1e-4	ssl5103	GO:0044425	<1e-4	sll1925	GO:0008233	0.0233
sll0294	GO:0009991	<1e-4	slr0453	GO:0072521	<1e-4	slr7024	GO:0022832	<1e-4	sll1380	GO:0044424	<1e-4	sll1071	GO:0008233	0.0233
ssr1765	GO:0016054	<1e-4	sll0096	GO:0009144	<1e-4	slr1780	GO:0015405	<1e-4	slr0285	GO:0044425	<1e-4	sll0585	GO:0008233	0.0233
sll1359	GO:0006066	<1e-4	slr1911	GO:0045184	<1e-4	sll6054	GO:0015291	<1e-4	sll1736	GO:0044422	<1e-4	slr0815	GO:0008233	0.0233
sll1939	GO:0006733	<1e-4	sll1060	GO:0060255	<1e-4	slr1505	GO:0016817	<1e-4	sll0577	GO:0044446	<1e-4	slr2018	GO:0016772	0.0233
slr1790	GO:0048878	<1e-4	slr1990	GO:0051171	<1e-4	slr1789	GO:0022891	<1e-4	slr5024	GO:0044446	<1e-4	sll0737	GO:0008233	0.0233
sll0272	GO:0044106	<1e-4	sll1606	GO:0009394	<1e-4	sll8004	GO:0015405	<1e-4	sll6055	GO:0005575	<1e-4	slr0468	GO:0008233	0.0233
slr7083	GO:0006766	<1e-4	slr1161	GO:0016053	<1e-4	sll0293	GO:0022838	<1e-4	sll0661	GO:0043226	<1e-4	ssr2803	GO:0016772	0.0233
sll1737	GO:0048523	<1e-4	ssl0242	GO:0006220	<1e-4	ssl2717	GO:0015291	<1e-4	sll0630	GO:0044422	<1e-4	sll0702	GO:0016772	0.0233
sll1191	GO:0010468	<1e-4	sll1526	GO:0071841	<1e-4	sll0281	GO:0015291	<1e-4	slr1052	GO:0044422	<1e-4	sll0180	GO:0008233	0.0233
slr0318	GO:0048519	<1e-4	ssr2962	GO:0009150	<1e-4	slr0813	GO:0016741	<1e-4	slr1923	GO:0043226	<1e-4	sll1582	GO:0008233	0.0233
sll5028	GO:0006220	<1e-4	sll0451	GO:0042180	<1e-4	slr1194	GO:0022832	<1e-4	sll6052	GO:0044444	<1e-4	sll0752	GO:0008233	0.0233
slr0708	GO:0046164	<1e-4	sll0742	GO:0019222	<1e-4	slr1699	GO:0043167	<1e-4	slr1932	GO:0044464	<1e-4	sll1660	GO:0008233	0.0233
sll1726	GO:0006163	<1e-4	sll0283	GO:0071842	<1e-4	slr0575	GO:0016462	<1e-4	sll8032	GO:0005575	<1e-4	sll1738	GO:0008233	0.0233
slr1699	GO:0071841	<1e-4	sll0147	GO:0055114	<1e-4	sll1068	GO:0043492	<1e-4	slr0058	GO:0044444	<1e-4	ssr1528	GO:0008233	0.0233
slr6064	GO:0006811	<1e-4	sll1135	GO:0009123	<1e-4	slr1875	GO:0008171	<1e-4	sll1121	GO:0044425	<1e-4	slr1767	GO:0044237	0.023
slr2003	GO:0046164	<1e-4	sll5033	GO:0009124	<1e-4	slr5012	GO:0022803	<1e-4	sll1196	GO:0005575	<1e-4	sll0586	GO:0044237	0.023
sll1915	GO:0019752	<1e-4	sll0242	GO:0048522	<1e-4	sll0266	GO:0008171	<1e-4	sll5109	GO:0044424	<1e-4	sll0183	GO:0044237	0.023
ssl3829	GO:0046128	<1e-4	sll1252	GO:0045184	<1e-4	sll1862	GO:0008171	<1e-4	sll0487	GO:0044422	<1e-4	slr0607	GO:0044237	0.023
sll0319	GO:0031326	<1e-4	slr2105	GO:0009117	<1e-4	sll1485	GO:0016741	<1e-4	ssr3189	GO:0044422	<1e-4	sll0192	GO:0044237	0.023
sll0931	GO:0006082	<1e-4	sll0645	GO:0006732	<1e-4	sll1764	GO:0022832	<1e-4	sll1293	GO:0044444	<1e-4	slr1959	GO:0044237	0.023
sll1006	GO:0046164	<1e-4	sll0424	GO:0009165	<1e-4	slr0169	GO:0015267	<1e-4	ssr1766	GO:0044425	<1e-4	slr1098	GO:0044237	0.023
slr1378	GO:0006721	<1e-4	slr2118	GO:0016052	<1e-4	slr0962	GO:0015405	<1e-4	slr1173	GO:0044446	<1e-4	slr1906	GO:0044237	0.023

slr5127	GO:0043170	<1e-4	slr1573	GO:0034660	<1e-4	slr1047	GO:0015267	<1e-4	slr0924	GO:0044424	<1e-4	ssl0739	GO:0042626	0.0228
sll1188	GO:0009132	<1e-4	slr0168	GO:0006220	<1e-4	slr1866	GO:0022891	<1e-4	sll0216	GO:0043226	<1e-4	slr1288	GO:0042626	0.0228
ssr2067	GO:0009117	<1e-4	slr1799	GO:0072521	<1e-4	sll0419	GO:0015399	<1e-4	ssl2069	GO:0044444	<1e-4	slr1809	GO:0042626	0.0228
sll1830	GO:0051818	<1e-4	slr1301	GO:0048523	<1e-4	ssr2047	GO:0016817	<1e-4	slr0207	GO:0044424	<1e-4	sll7065	GO:0042626	0.0228
slr7026	GO:0044281	<1e-4	sll0751	GO:0042451	<1e-4	sll0811	GO:0022891	<1e-4	slr7010	GO:0005575	<1e-4	sll0669	GO:0042626	0.0228
slr0038	GO:0050794	<1e-4	ssr6024	GO:0043549	<1e-4	ssr7035	GO:0015291	<1e-4	slr0400	GO:0044424	<1e-4	sll0688	GO:0042626	0.0228
slr1854	GO:0050794	<1e-4	slr5126	GO:0046164	<1e-4	sll1911	GO:0015293	<1e-4	sll1304	GO:0044444	<1e-4	sll1201	GO:0042626	0.0228
ssr2422	GO:0019751	<1e-4	sll1250	GO:0050789	<1e-4	sll0355	GO:0015291	<1e-4	slr1863	GO:0044422	<1e-4	sll0623	GO:0042626	0.0228
slr0907	GO:0043436	<1e-4	sll1135	GO:0031640	<1e-4	sll0933	GO:0005342	<1e-4	slr1050	GO:0044444	<1e-4	sll0412	GO:0042626	0.0228
ssl0410	GO:0055114	<1e-4	slr0109	GO:0050896	<1e-4	slr1365	GO:0015291	<1e-4	slr0157	GO:0005575	<1e-4	sll0871	GO:0042626	0.0228
slr0023	GO:0019219	<1e-4	sll0181	GO:0043170	<1e-4	slr6088	GO:0015293	<1e-4	slr5102	GO:0043226	<1e-4	slr0742	GO:0042626	0.0228
slr1638	GO:0006766	<1e-4	slr0519	GO:0009161	<1e-4	sll1911	GO:0022803	<1e-4	slr5037	GO:0044424	<1e-4	ssr2009	GO:0042626	0.0228
slr1103	GO:0019222	<1e-4	sll1913	GO:0043648	<1e-4	sll1698	GO:0043167	<1e-4	ssr0761	GO:0044425	<1e-4	slr0272	GO:0042626	0.0228
sll0703	GO:0055114	<1e-4	slr6044	GO:0019637	<1e-4	slr1056	GO:0022832	<1e-4	slr6021	GO:0005575	<1e-4	sll1505	GO:0042626	0.0228
sll0444	GO:0009889	<1e-4	slr1864	GO:0044281	<1e-4	sll0481	GO:0022891	<1e-4	slr6045	GO:0043226	<1e-4	sll1654	GO:0042626	0.0228
slr1603	GO:0071554	<1e-4	slr0888	GO:0009394	<1e-4	slr1240	GO:0022892	<1e-4	sll0614	GO:0043226	<1e-4	sll1512	GO:0042626	0.0228
sll1632	GO:0009394	<1e-4	sll1532	GO:0009991	<1e-4	slr1638	GO:0008171	<1e-4	sll0995	GO:0044444	<1e-4	sll0189	GO:0042626	0.0228
slr1677	GO:0008150	<1e-4	sll0909	GO:0019219	<1e-4	sll0369	GO:0015075	<1e-4	slr1083	GO:0005575	<1e-4	slr0680	GO:0042626	0.0228
ssl5064	GO:0065007	<1e-4	sll0008	GO:0006793	<1e-4	sll1232	GO:0022892	<1e-4	sll0301	GO:0044464	<1e-4	slr1541	GO:0042626	0.0228
slr1600	GO:0046128	<1e-4	slr2032	GO:0009150	<1e-4	slr1260	GO:0043167	<1e-4	slr0606	GO:0044424	<1e-4	slr0172	GO:0042626	0.0228
ssl8028	GO:0043933	<1e-4	sll1135	GO:0072522	<1e-4	sll0242	GO:0022892	<1e-4	ssr2754	GO:0044446	<1e-4	slr1074	GO:0042626	0.0228
slr0271	GO:0090304	<1e-4	ssl2162	GO:0006631	<1e-4	sll1601	GO:0016462	<1e-4	sll0309	GO:0044444	<1e-4	sll0319	GO:0042626	0.0228
slr0589	GO:0055082	<1e-4	ssl5098	GO:0016054	<1e-4	slr0865	GO:0022890	<1e-4	sll0888	GO:0005575	<1e-4	sll1950	GO:0042626	0.0228
sll1267	GO:0045184	<1e-4	slr1419	GO:0019751	<1e-4	sll0372	GO:0016818	<1e-4	ssr1155	GO:0044422	<1e-4	sll1239	GO:0042626	0.0228
ssr1558	GO:0044282	<1e-4	sll1022	GO:0050794	<1e-4	sll0048	GO:0015075	<1e-4	sgl0002	GO:0044422	<1e-4	sll1344	GO:0042626	0.0228
slr8021	GO:0050801	<1e-4	sll0911	GO:0048522	<1e-4	slr1493	GO:0015399	<1e-4	slr1737	GO:0043226	<1e-4	slr0981	GO:0042626	0.0228
sll0451	GO:0016054	<1e-4	sll1832	GO:0009987	<1e-4	slr0601	GO:0016462	<1e-4	slr0491	GO:0005575	<1e-4	slr2115	GO:0042626	0.0228
slr1438	GO:0072522	<1e-4	sll1765	GO:0051179	<1e-4	slr0569	GO:0008171	<1e-4	sll7077	GO:0044422	<1e-4	slr1852	GO:0042626	0.0228
sll1201	GO:0009150	<1e-4	slr1394	GO:0042455	<1e-4	slr1923	GO:0016818	<1e-4	ssr2067	GO:0043226	<1e-4	slr1658	GO:0042626	0.0228
sll0301	GO:0022607	<1e-4	slr0596	GO:0019222	<1e-4	sll0473	GO:0015399	<1e-4	slr2119	GO:0005575	<1e-4	sll1251	GO:0042626	0.0228
slr1677	GO:0009991	<1e-4	slr7071	GO:0009893	<1e-4	slr0514	GO:0016462	<1e-4	sll7055	GO:0044424	<1e-4	sll0590	GO:0042626	0.0228
ssl2733	GO:0071840	<1e-4	slr1074	GO:0055086	<1e-4	sll0414	GO:0022838	<1e-4	ssl6061	GO:0044422	<1e-4	sll0837	GO:0042626	0.0228
sll1609	GO:0008150	<1e-4	slr1210	GO:0060255	<1e-4	slr1819	GO:0015075	<1e-4	slr1918	GO:0005575	<1e-4	sll0781	GO:0042626	0.0228
sll1606	GO:0071554	<1e-4	ssr2201	GO:0019752	<1e-4	sll1606	GO:0016741	<1e-4	sll0149	GO:0044422	<1e-4	sll0827	GO:0042626	0.0228
ssr1256	GO:0006163	<1e-4	sll0532	GO:0008150	<1e-4	sll0710	GO:0022838	<1e-4	slr1885	GO:0005575	<1e-4	sll1738	GO:0042626	0.0228
slr1957	GO:0009892	<1e-4	ssr1698	GO:0071841	<1e-4	ssr6003	GO:0016741	<1e-4	sll1581	GO:0005575	<1e-4	slr0789	GO:0042626	0.0228
slr2011	GO:0044106	<1e-4	slr7073	GO:0034660	<1e-4	sll0442	GO:0015405	<1e-4	sll5026	GO:0044444	<1e-4	sll0752	GO:0042626	0.0228
sll0545	GO:0071554	<1e-4	slr6028	GO:0019219	<1e-4	slr0144	GO:0003674	<1e-4	slr0919	GO:0044446	<1e-4	sll0787	GO:0042626	0.0228
sll1722	GO:0008150	<1e-4	slr1209	GO:0065007	<1e-4	ssr6099	GO:0042623	<1e-4	slr0658	GO:0044446	<1e-4	slr1493	GO:0042626	0.0228
slr1183	GO:0008610	<1e-4	slr7073	GO:0009263	<1e-4	slr7102	GO:0022836	<1e-4	ssl0109	GO:0044425	<1e-4	slr0935	GO:0042626	0.0228
slr1196	GO:0072527	<1e-4	slr8022	GO:0046164	<1e-4	slr1513	GO:0015075	<1e-4	ssr6085	GO:0044425	<1e-4	sll0156	GO:0042626	0.0228
sll1092	GO:0034654	<1e-4	ssr1552	GO:0042180	<1e-4	slr8044	GO:0022836	<1e-4	ssl5103	GO:0044446	<1e-4	slr0483	GO:0042626	0.0228
sll1675	GO:0048878	<1e-4	sll0022	GO:0045184	<1e-4	sll1135	GO:0015399	<1e-4	sll0586	GO:0005575	<1e-4	slr1070	GO:0042626	0.0228
slr1240	GO:0055082	<1e-4	sll6052	GO:0044260	<1e-4	sll8033	GO:0022891	<1e-4	slr0730	GO:0044422	<1e-4	sml0011	GO:0042626	0.0228
sll0875	GO:0016052	<1e-4	sll1915	GO:0034641	<1e-4	sll1164	GO:0060089	<1e-4	sll1532	GO:0044425	<1e-4	sll0740	GO:0042626	0.0228
slr8014	GO:0065007	<1e-4	slr0168	GO:0010556	<1e-4	slr0388	GO:0015399	<1e-4	ssl7045	GO:0043226	<1e-4	sll1938	GO:0042626	0.0228
slr0393	GO:0072521	<1e-4	sll0656	GO:0019751	<1e-4	slr1495	GO:0043492	<1e-4	sll1698	GO:0005575	<1e-4	ssr3300	GO:0042626	0.0228
slr0680	GO:0009394	<1e-4	slr2080	GO:0006721	<1e-4	sll0780	GO:0008171	<1e-4	slr0064	GO:0044424	<1e-4	slr0957	GO:0042626	0.0228
slr6073	GO:0016053	<1e-4	ssr3402	GO:0009124	<1e-4	slr0725	GO:0015405	<1e-4	ssl0483	GO:0044446	<1e-4	slr1258	GO:0042626	0.0228
ssr6020	GO:0042455	<1e-4	sll1072	GO:0072528	<1e-4	sll1355	GO:0022892	<1e-4	slr1652	GO:0005575	<1e-4	sll0188	GO:0042626	0.0228
slr0670	GO:0009056	<1e-4	slr0680	GO:0006721	<1e-4	slr1127	GO:0016741	<1e-4	sll0481	GO:0044464	<1e-4	sll1170	GO:0042626	0.0228
slr0789	GO:0016052	<1e-4	slr2012	GO:0001932	<1e-4	slr1142	GO:0015267	<1e-4	sll0854	GO:0043226	<1e-4	sll0588	GO:0042626	0.0228
sll0335	GO:0055082	<1e-4	slr1918	GO:0009124	<1e-4	slr6067	GO:0008171	<1e-4	ssr3189	GO:0044446	<1e-4	slr1544	GO:0042626	0.0228
ssl1263	GO:0072528	<1e-4	ssl5096	GO:0009260	<1e-4	ssr3300	GO:0022890	<1e-4	slr6074	GO:0044444	<1e-4	slr0491	GO:0008168	0.0224

sll1765	GO:0044283	<1e-4	slr0869	GO:0065007	<1e-4	ssr0657	GO:0005342	<1e-4	ssr6030	GO:0044422	<1e-4	sll1858	GO:0008168	0.0224
slr0249	GO:0006066	<1e-4	sll0995	GO:0060255	<1e-4	sll0857	GO:0003674	<1e-4	sll0405	GO:0044446	<1e-4	slr0810	GO:0008168	0.0224
sll0525	GO:0046483	<1e-4	sll0176	GO:0009259	<1e-4	sll1651	GO:0008171	<1e-4	sll1675	GO:0044464	<1e-4	slr1442	GO:0016740	0.0222
ssr3300	GO:0051246	<1e-4	slr1262	GO:0009123	<1e-4	slr0313	GO:0016746	<1e-4	slr2071	GO:0044444	<1e-4	slr1681	GO:0016740	0.0222
sll0997	GO:0052111	<1e-4	sll0788	GO:0006720	<1e-4	ssl1918	GO:0005342	<1e-4	sll1201	GO:0005575	<1e-4	slr2049	GO:0016740	0.0222
sll1531	GO:0006732	<1e-4	sll1049	GO:0043648	<1e-4	sll0930	GO:0022857	<1e-4	slr7023	GO:0005575	<1e-4	ssr3402	GO:0016740	0.0222
sll6052	GO:0048878	<1e-4	sll1696	GO:0019752	<1e-4	slr1413	GO:0016817	<1e-4	ssr6002	GO:0044446	<1e-4	slr5101	GO:0016740	0.0222
sll0444	GO:0044255	<1e-4	sll1233	GO:0009987	<1e-4	slr1940	GO:0022857	<1e-4	ssl6035	GO:0005575	<1e-4	sll0249	GO:0016740	0.0222
slr1788	GO:0051246	<1e-4	slr1163	GO:0046483	<1e-4	slr1811	GO:0022891	<1e-4	sll0096	GO:0044422	<1e-4	slr1790	GO:0016740	0.0222
ssl3379	GO:0044260	<1e-4	slr1862	GO:0071842	<1e-4	sll1960	GO:0015267	<1e-4	slr0505	GO:0005575	<1e-4	slr2060	GO:0016740	0.0222
sll7077	GO:0009056	<1e-4	slr0059	GO:0043412	<1e-4	slr1778	GO:0015405	<1e-4	ssr5092	GO:0005575	<1e-4	slr7102	GO:0016740	0.0222
sll1158	GO:0006066	<1e-4	slr1066	GO:0043933	<1e-4	slr1275	GO:0016462	<1e-4	slr1627	GO:0044425	<1e-4	sll1158	GO:0016740	0.0222
sll1913	GO:0072524	<1e-4	ssr3570	GO:0009057	<1e-4	slr6088	GO:0022890	<1e-4	slr1195	GO:0044464	<1e-4	sll0925	GO:0016740	0.0222
ssr1407	GO:0006576	<1e-4	slr0103	GO:0072528	<1e-4	sll0910	GO:0016818	<1e-4	ssr7093	GO:0044425	<1e-4	sll0691	GO:0016740	0.0222
slr5112	GO:0009141	<1e-4	slr7071	GO:0009141	<1e-4	sll1769	GO:0022832	<1e-4	slr1464	GO:0043226	<1e-4	sll1785	GO:0016740	0.0222
slr1907	GO:0019752	<1e-4	slr0725	GO:0009889	<1e-4	slr0816	GO:0015399	<1e-4	ssl1377	GO:0005575	<1e-4	slr0575	GO:0016740	0.0222
slr1827	GO:0006793	<1e-4	slr1449	GO:0006732	<1e-4	ssr5106	GO:0022890	<1e-4	sll0499	GO:0043226	<1e-4	slr0634	GO:0016740	0.0222
sll1123	GO:0044248	<1e-4	slr7015	GO:0071842	<1e-4	sll0584	GO:0043167	<1e-4	ssr2142	GO:0044446	<1e-4	ssr3572	GO:0016740	0.0222
sll7030	GO:0006732	<1e-4	slr1927	GO:0010468	<1e-4	sll0909	GO:0022890	<1e-4	sll1095	GO:0044464	<1e-4	sll1233	GO:0016740	0.0222
sll7062	GO:0022411	<1e-4	sll1510	GO:0072527	<1e-4	sll0281	GO:0042623	<1e-4	slr1339	GO:0044424	<1e-4	sll1254	GO:0016740	0.0222
slr1340	GO:0009260	<1e-4	slr7016	GO:0019219	<1e-4	slr0680	GO:0003674	<1e-4	slr0642	GO:0044464	<1e-4	ssr7017	GO:0016740	0.0222
slr2105	GO:0048519	<1e-4	slr0948	GO:0051347	<1e-4	sll1201	GO:0015077	<1e-4	sll1547	GO:0005575	<1e-4	slr1577	GO:0016740	0.0222
slr1468	GO:0046483	<1e-4	sll0354	GO:0044275	<1e-4	sll7033	GO:0016462	<1e-4	slr1819	GO:0005575	<1e-4	slr5053	GO:0016740	0.0222
slr1799	GO:0031640	<1e-4	ssl1377	GO:0031326	<1e-4	slr1210	GO:0022832	<1e-4	sll0585	GO:0005575	<1e-4	sll8019	GO:0016740	0.0222
sll1359	GO:0071841	<1e-4	slr0442	GO:0051246	<1e-4	sll1071	GO:0022892	<1e-4	slr0192	GO:0005575	<1e-4	sll1898	GO:0016740	0.0222
slr0601	GO:0043170	<1e-4	slr0380	GO:0009059	<1e-4	sll0394	GO:0015399	<1e-4	ssr6032	GO:0044422	<1e-4	slr5073	GO:0016740	0.0222
sll1021	GO:0009889	<1e-4	sll0286	GO:0006732	<1e-4	sll0611	GO:0016741	<1e-4	ssr5074	GO:0044425	<1e-4	slr1396	GO:0016740	0.0222
slr0397	GO:0006066	<1e-4	sll0436	GO:0006066	<1e-4	sll7077	GO:0016817	<1e-4	slr6008	GO:0044425	<1e-4	ssl1552	GO:0016740	0.0222
slr1895	GO:0009141	<1e-4	sll1965	GO:0034654	<1e-4	slr2105	GO:0015267	<1e-4	slr1187	GO:0044425	<1e-4	ssl8039	GO:0016740	0.0222
slr1179	GO:0016054	<1e-4	sll0008	GO:0072521	<1e-4	slr2117	GO:0022803	<1e-4	sll0597	GO:0043226	<1e-4	slr2004	GO:0016740	0.0222
sll1680	GO:0008150	<1e-4	sll0847	GO:0044275	<1e-4	slr7071	GO:0015075	<1e-4	slr1394	GO:0043226	<1e-4	slr0013	GO:0016740	0.0222
slr1773	GO:0033013	<1e-4	ssl1690	GO:0072527	<1e-4	sll1606	GO:0022891	<1e-4	sll1775	GO:0043226	<1e-4	ssr8013	GO:0016740	0.0222
sll0985	GO:0005996	<1e-4	sll1749	GO:0044275	<1e-4	slr1195	GO:0060089	<1e-4	ssr0336	GO:0005575	<1e-4	slr0238	GO:0016740	0.0222
ssl5031	GO:0006766	<1e-4	sll1400	GO:0044283	<1e-4	sll0752	GO:0015075	<1e-4	ssl1255	GO:0044444	<1e-4	ssl5113	GO:0016740	0.0222
slr5101	GO:0044003	<1e-4	sll1634	GO:0006576	<1e-4	sll0685	GO:0015399	<1e-4	slr0514	GO:0044425	<1e-4	slr7091	GO:0016740	0.0222
slr1885	GO:0042451	<1e-4	sll1155	GO:0044260	<1e-4	ssl5008	GO:0022836	<1e-4	slr0610	GO:0044424	<1e-4	slr0709	GO:0016740	0.0222
slr1117	GO:0043549	<1e-4	sll0786	GO:0009308	<1e-4	slr7095	GO:0016818	<1e-4	slr0326	GO:0044464	<1e-4	sll0060	GO:0016740	0.0222
slr7025	GO:0046483	<1e-4	sll0497	GO:0008610	<1e-4	ssl0294	GO:0043167	<1e-4	slr5127	GO:0005575	<1e-4	slr1114	GO:0016740	0.0222
sll1785	GO:0080090	<1e-4	sll0737	GO:0006720	<1e-4	ssr5120	GO:0016746	<1e-4	ssr5106	GO:0044446	<1e-4	slr1513	GO:0016740	0.0222
slr1240	GO:0048522	<1e-4	ssr5019	GO:0008610	<1e-4	slr1162	GO:0022803	<1e-4	slr0270	GO:0043226	<1e-4	slr1737	GO:0016740	0.0222
sll0670	GO:0051246	<1e-4	sll0875	GO:0050794	<1e-4	slr1886	GO:0015267	<1e-4	slr2111	GO:0044422	<1e-4	slr7057	GO:0016740	0.0222
slr0503	GO:0051246	<1e-4	slr1025	GO:0051347	<1e-4	ssr2142	GO:0015293	<1e-4	ssl5114	GO:0044446	<1e-4	slr1670	GO:0016740	0.0222
sll1036	GO:0016054	<1e-4	slr0606	GO:0046483	<1e-4	sll5006	GO:0022838	<1e-4	slr1383	GO:0044425	<1e-4	slr1170	GO:0016740	0.0222
ssr2615	GO:0009991	<1e-4	slr7098	GO:0031640	<1e-4	slr1116	GO:0022892	<1e-4	sll7028	GO:0043226	<1e-4	sll0479	GO:0016740	0.0222
sll0688	GO:0019362	<1e-4	slr1557	GO:0044260	<1e-4	sll0685	GO:0015293	<1e-4	sll1174	GO:0044424	<1e-4	slr7060	GO:0016740	0.0222
slr7011	GO:0052111	<1e-4	sll0372	GO:0043648	<1e-4	slr0607	GO:0015399	<1e-4	ssl1004	GO:0044424	<1e-4	ssr6019	GO:0016740	0.0222
sll1455	GO:0046907	<1e-4	sll1399	GO:0044255	<1e-4	slr0852	GO:0042623	<1e-4	sll1442	GO:0005575	<1e-4	ssl7042	GO:0016740	0.0222
slr0082	GO:0008150	<1e-4	slr0725	GO:0009199	<1e-4	slr1790	GO:0015293	<1e-4	slr0423	GO:0044446	<1e-4	slr0516	GO:0016740	0.0222
ssr2317	GO:0022607	<1e-4	slr0613	GO:0065007	<1e-4	slr0392	GO:0022832	<1e-4	slr2101	GO:0044424	<1e-4	slr5126	GO:0016740	0.0222
sll0630	GO:0044281	<1e-4	sll0590	GO:0044106	<1e-4	ssr7035	GO:0022832	<1e-4	ssr7079	GO:0044446	<1e-4	slr6103	GO:0016740	0.0222
sll0310	GO:0006732	<1e-4	sll1858	GO:0043170	<1e-4	ssl8008	GO:0015399	<1e-4	ssr5106	GO:0005575	<1e-4	ssl3829	GO:0016740	0.0222
slr0586	GO:0072522	<1e-4	slr0602	GO:0019752	<1e-4	slr1699	GO:0016746	<1e-4	slr0351	GO:0044464	<1e-4	ssr8047	GO:0016740	0.0222
ssl2781	GO:0044275	<1e-4	slr1177	GO:0009144	<1e-4	ssl1923	GO:0016462	<1e-4	slr1638	GO:0044444	<1e-4	slr2052	GO:0016740	0.0222
ssl2996	GO:0071554	<1e-4	ssl2471	GO:0006220	<1e-4	sll0615	GO:0016818	<1e-4	sll0944	GO:0044444	<1e-4	ssr6086	GO:0016740	0.0222

slr0179	GO:0044260	<1e-4	slr1236	GO:0009150	<1e-4	ssl7046	GO:0022890	<1e-4	sll0272	GO:0044446	<1e-4	ssl5068	GO:0016740	0.0222
ssl0294	GO:0043648	<1e-4	slr0872	GO:0010468	<1e-4	slr1570	GO:0016818	<1e-4	sll0861	GO:0005575	<1e-4	sll1698	GO:0016740	0.0222
slr0655	GO:0019219	<1e-4	sll0743	GO:0043412	<1e-4	slr1800	GO:0060089	<1e-4	sll0482	GO:0044422	<1e-4	ssr1041	GO:0016740	0.0222
ssr6086	GO:0043933	<1e-4	slr1676	GO:0034641	<1e-4	sll1158	GO:0015077	<1e-4	slr7013	GO:0044422	<1e-4	slr0816	GO:0016740	0.0222
sll5028	GO:0033013	<1e-4	sll1880	GO:0072528	<1e-4	slr0272	GO:0015291	<1e-4	sll0266	GO:0044422	<1e-4	sll0047	GO:0016740	0.0222
ssl1690	GO:0016053	<1e-4	sll0863	GO:0055114	<1e-4	slr1862	GO:0022892	<1e-4	ssr3532	GO:0044444	<1e-4	slr5127	GO:0016740	0.0222
slr1290	GO:0045184	<1e-4	slr6067	GO:0048523	<1e-4	slr1623	GO:0022857	<1e-4	ssl1498	GO:0043226	<1e-4	slr1599	GO:0016740	0.0222
ssl3379	GO:0009144	<1e-4	slr5101	GO:0019752	<1e-4	sll7069	GO:0022857	<1e-4	sll0284	GO:0044444	<1e-4	slr5112	GO:0016740	0.0222
slr2012	GO:0019362	<1e-4	slr0241	GO:0019362	<1e-4	slr0217	GO:0016818	<1e-4	sll0007	GO:0044424	<1e-4	slr1570	GO:0016740	0.0222
sll1671	GO:0019219	<1e-4	slr7081	GO:0006793	<1e-4	slr1261	GO:0015399	<1e-4	slr0300	GO:0005575	<1e-4	ssl5091	GO:0016740	0.0222
ssr6026	GO:0006576	<1e-4	ssr3532	GO:0022607	<1e-4	slr0358	GO:0060089	<1e-4	ssl5108	GO:0044446	<1e-4	ssr0336	GO:0016740	0.0222
slr0300	GO:0031323	<1e-4	slr0651	GO:0072527	<1e-4	sll1891	GO:0042623	<1e-4	sll7055	GO:0005575	<1e-4	sll0069	GO:0016740	0.0222
slr1914	GO:0022607	<1e-4	sll1201	GO:0055114	<1e-4	ssr1155	GO:0016741	<1e-4	slr1110	GO:0044425	<1e-4	slr0725	GO:0016740	0.0222
sml0011	GO:0043170	<1e-4	slr5077	GO:0046164	<1e-4	sll0148	GO:0016741	<1e-4	slr0241	GO:0044464	<1e-4	slr0645	GO:0016740	0.0222
sll6052	GO:0031323	<1e-4	ssl1004	GO:0072521	<1e-4	slr1958	GO:0016818	<1e-4	sll0984	GO:0044444	<1e-4	sll1911	GO:0016740	0.0222
ssl2162	GO:0051234	<1e-4	ssl2384	GO:0009199	<1e-4	sll1373	GO:0022832	<1e-4	slr0569	GO:0044425	<1e-4	slr0509	GO:0016740	0.0222
slr6104	GO:0019219	<1e-4	sll0478	GO:0044106	<1e-4	slr0708	GO:0015291	<1e-4	sll0602	GO:0005575	<1e-4	ssl3549	GO:0016740	0.0222
sll1388	GO:0051171	<1e-4	slr6104	GO:0071840	<1e-4	slr0989	GO:0015399	<1e-4	slr2005	GO:0044446	<1e-4	ssl2384	GO:0016740	0.0222
slr0263	GO:0072521	<1e-4	sll0786	GO:0071554	<1e-4	sll1052	GO:0022836	<1e-4	slr8022	GO:0044444	<1e-4	sll8011	GO:0016740	0.0222
slr6064	GO:0072522	<1e-4	slr0664	GO:0071840	<1e-4	sll1222	GO:0043167	<1e-4	slr2125	GO:0043226	<1e-4	slr0271	GO:0016740	0.0222
slr1935	GO:0010468	<1e-4	slr5073	GO:0009987	<1e-4	ssl2996	GO:0016741	<1e-4	slr1406	GO:0044422	<1e-4	ssr6062	GO:0016740	0.0222
slr0250	GO:0031326	<1e-4	slr6047	GO:0048518	<1e-4	sll0793	GO:0022836	<1e-4	sll1396	GO:0005575	<1e-4	slr1628	GO:0016740	0.0222
slr0060	GO:0044255	<1e-4	sll0864	GO:0009308	<1e-4	slr1415	GO:0022803	<1e-4	slr6087	GO:0005575	<1e-4	sll6052	GO:0016740	0.0222
slr1174	GO:0042180	<1e-4	slr1668	GO:0044106	<1e-4	slr0491	GO:0022891	<1e-4	slr1865	GO:0044425	<1e-4	sll0670	GO:0016740	0.0222
slr1206	GO:0055114	<1e-4	slr1815	GO:0044271	<1e-4	slr2018	GO:0015399	<1e-4	sll0147	GO:0044424	<1e-4	slr6104	GO:0016740	0.0222
slr6068	GO:0009991	<1e-4	sll0281	GO:0051188	<1e-4	slr6008	GO:0042623	<1e-4	ssr2755	GO:0044464	<1e-4	ssr2998	GO:0016740	0.0222
ssr2998	GO:0048518	<1e-4	sll0625	GO:0006082	<1e-4	slr1173	GO:0022857	<1e-4	sll0762	GO:0044444	<1e-4	sll0253	GO:0016740	0.0222
sll8001	GO:0043436	<1e-4	sll0763	GO:0006066	<1e-4	sll1053	GO:0022832	<1e-4	slr7073	GO:0044444	<1e-4	sll0847	GO:0016740	0.0222
slr2070	GO:0019362	<1e-4	slr0605	GO:0019222	<1e-4	slr0606	GO:0016741	<1e-4	sll1632	GO:0044425	<1e-4	sll7087	GO:0016740	0.0222
slr1163	GO:0051347	<1e-4	sll1464	GO:0033013	<1e-4	sll1433	GO:0060089	<1e-4	slr1593	GO:0044464	<1e-4	sll0372	GO:0016740	0.0222
ssr6085	GO:0009142	<1e-4	slr0742	GO:0031323	<1e-4	slr6044	GO:0022890	<1e-4	ssr2998	GO:0044422	<1e-4	sll5030	GO:0016740	0.0222
slr0708	GO:0022411	<1e-4	ssl3615	GO:0048878	<1e-4	slr0273	GO:0005342	<1e-4	sll0647	GO:0044422	<1e-4	ssr6078	GO:0016740	0.0222
sll0858	GO:0019637	<1e-4	sll0659	GO:0045184	<1e-4	ssl3291	GO:0015291	<1e-4	ssr2049	GO:0005575	<1e-4	slr0521	GO:0016740	0.0222
sll7067	GO:0009117	<1e-4	slr1507	GO:0009991	<1e-4	sll0597	GO:0043167	<1e-4	slr0359	GO:0044446	<1e-4	sll0563	GO:0016740	0.0222
sll0736	GO:0051818	<1e-4	slr0981	GO:0016052	<1e-4	ssl1378	GO:0015405	<1e-4	slr0300	GO:0044422	<1e-4	sll1095	GO:0016740	0.0222
sll1715	GO:0050801	<1e-4	ssl7007	GO:0009117	<1e-4	slr0975	GO:0022892	<1e-4	slr0172	GO:0044444	<1e-4	sll0394	GO:0016740	0.0222
slr1940	GO:0009991	<1e-4	slr0729	GO:0051818	<1e-4	slr1474	GO:0008171	<1e-4	ssr6086	GO:0043226	<1e-4	sll7067	GO:0016740	0.0222
slr7014	GO:0044260	<1e-4	sll1751	GO:0065007	<1e-4	slr1951	GO:0016817	<1e-4	sll1528	GO:0044424	<1e-4	sll0496	GO:0016740	0.0222
slr8044	GO:0071840	<1e-4	slr0262	GO:0009260	<1e-4	sll1191	GO:0008171	<1e-4	slr0914	GO:0044446	<1e-4	ssl1300	GO:0016740	0.0222
sll0656	GO:0006721	<1e-4	slr0505	GO:0051246	<1e-4	sll5089	GO:0015267	<1e-4	sll1915	GO:0043226	<1e-4	ssl7007	GO:0016740	0.0222
sll1485	GO:0034654	<1e-4	slr1101	GO:0006721	<1e-4	slr1419	GO:0016462	<1e-4	sll8027	GO:0044446	<1e-4	sll1188	GO:0016740	0.0222
slr1668	GO:0019219	<1e-4	sll0355	GO:0051171	<1e-4	slr1069	GO:0042623	<1e-4	ssr2848	GO:0044422	<1e-4	sll1381	GO:0016740	0.0222
sll1002	GO:0048519	<1e-4	sll1921	GO:0046164	<1e-4	sll1652	GO:0022857	<1e-4	slr0172	GO:0044425	<1e-4	slr1623	GO:0016740	0.0222
slr6029	GO:0009260	<1e-4	sll2007	GO:0009141	<1e-4	sll0710	GO:0060089	<1e-4	sll1832	GO:0043226	<1e-4	slr1875	GO:0016740	0.0222
slr1774	GO:0072522	<1e-4	sll1698	GO:0019219	<1e-4	slr7083	GO:0022803	<1e-4	ssl0294	GO:0044422	<1e-4	slr1163	GO:0016740	0.0222
slr1170	GO:0009309	<1e-4	slr2004	GO:0065007	<1e-4	slr1702	GO:0015399	<1e-4	slr5116	GO:0044446	<1e-4	sll0405	GO:0016740	0.0222
sll1785	GO:0009124	<1e-4	sll5089	GO:0046483	<1e-4	ssl0109	GO:0022832	<1e-4	slr0491	GO:0044444	<1e-4	sll1049	GO:0016740	0.0222
sll1426	GO:0008150	<1e-4	sll0984	GO:0010556	<1e-4	sll1570	GO:0005342	<1e-4	sll1510	GO:0044425	<1e-4	sll1765	GO:0042626	0.0221
sll0191	GO:0008150	<1e-4	sll1532	GO:0080090	<1e-4	ssr8013	GO:0005342	<1e-4	slr1442	GO:0005575	<1e-4	slr0362	GO:0042626	0.0221
sll0072	GO:0044260	<1e-4	ssl2064	GO:0043549	<1e-4	slr6091	GO:0022890	<1e-4	slr0575	GO:0044424	<1e-4	sll1400	GO:0042626	0.0221
sll0327	GO:0033013	<1e-4	ssl2162	GO:0019219	<1e-4	ssr2781	GO:0022892	<1e-4	sll1961	GO:0044425	<1e-4	sll0328	GO:0042626	0.0221
sll5026	GO:0080090	<1e-4	slr7092	GO:0016053	<1e-4	slr1880	GO:0008171	<1e-4	sll1630	GO:0044464	<1e-4	sll1862	GO:0042626	0.0221
sll0864	GO:0046907	<1e-4	sll1414	GO:0044106	<1e-4	sll0696	GO:0022890	<1e-4	sll1352	GO:0044424	<1e-4	sll5006	GO:0042626	0.0221
sll1921	GO:0009309	<1e-4	slr7037	GO:0046128	<1e-4	ssr2439	GO:0015075	<1e-4	slr1932	GO:0044424	<1e-4	slr1998	GO:0042626	0.0221

slr0360	GO:0071496	<1e-4	sll1163	GO:0009991	<1e-4	sll1267	GO:0022892	<1e-4	slr1450	GO:0044444	<1e-4	sll1485	GO:0042626	0.0221
sll1902	GO:0072521	<1e-4	slr0960	GO:0009161	<1e-4	sll0265	GO:0022832	<1e-4	sll0742	GO:0044444	<1e-4	slr1104	GO:0042626	0.0221
sll0283	GO:0071554	<1e-4	sll1660	GO:0044283	<1e-4	sll0669	GO:0043167	<1e-4	sll1192	GO:0044446	<1e-4	slr1276	GO:0042626	0.0221
sll0060	GO:0001932	<1e-4	sll1902	GO:0006811	<1e-4	sll0044	GO:0015405	<1e-4	sll1761	GO:0044444	<1e-4	slr7012	GO:0042626	0.0221
slr1419	GO:0046394	<1e-4	slr1442	GO:0046483	<1e-4	slr6045	GO:0003674	<1e-4	slr0607	GO:0044425	<1e-4	sll1053	GO:0042626	0.0221
slr1847	GO:0043412	<1e-4	sll1232	GO:0051716	<1e-4	sll1464	GO:0022836	<1e-4	sll1247	GO:0044444	<1e-4	slr6074	GO:0042626	0.0221
slr5126	GO:0043933	<1e-4	sll7034	GO:0006811	<1e-4	slr1376	GO:0022891	<1e-4	slr1303	GO:0044425	<1e-4	slr0769	GO:0042626	0.0221
ssl1378	GO:0031323	<1e-4	slr2027	GO:0060255	<1e-4	sll1442	GO:0015291	<1e-4	slr1852	GO:0044424	<1e-4	sll1880	GO:0042626	0.0221
slr0211	GO:0046483	<1e-4	slr1864	GO:0046394	<1e-4	sll0837	GO:0022890	<1e-4	slr1927	GO:0044464	<1e-4	sll0481	GO:0042626	0.0221
sll1882	GO:0031323	<1e-4	slr0964	GO:0009394	<1e-4	sll0539	GO:0022832	<1e-4	slr0740	GO:0005575	<1e-4	slr1188	GO:0042626	0.0221
slr1573	GO:0048878	<1e-4	sll0558	GO:0009132	<1e-4	slr1667	GO:0043167	<1e-4	sll0397	GO:0005575	<1e-4	slr0049	GO:0042626	0.0221
sll1109	GO:0065007	<1e-4	ssl3382	GO:0016052	<1e-4	slr7102	GO:0022892	<1e-4	slr0049	GO:0005575	<1e-4	sll6054	GO:0042626	0.0221
slr1122	GO:0009142	<1e-4	sll0208	GO:0019219	<1e-4	sll1715	GO:0022832	<1e-4	slr0695	GO:0043226	<1e-4	ssl3451	GO:0042626	0.0221
sll1142	GO:0051716	<1e-4	sll1726	GO:0006811	<1e-4	slr0479	GO:0008171	<1e-4	slr0397	GO:0044444	<1e-4	sll7064	GO:0042626	0.0221
ssr5011	GO:0044275	<1e-4	slr0243	GO:0044248	<1e-4	sll0524	GO:0043167	<1e-4	slr1977	GO:0044444	<1e-4	ssl2781	GO:0042626	0.0221
ssr7079	GO:0019219	<1e-4	sll1749	GO:0043170	<1e-4	slr5126	GO:0043167	<1e-4	sll1717	GO:0044444	<1e-4	sll0176	GO:0042626	0.0221
slr1533	GO:0019637	<1e-4	ssr6024	GO:0009991	<1e-4	slr0810	GO:0015077	<1e-4	slr0458	GO:0044422	<1e-4	slr6100	GO:0042626	0.0221
sll0518	GO:0065007	<1e-4	slr0299	GO:0044003	<1e-4	sll0543	GO:0042623	<1e-4	sll0888	GO:0043226	<1e-4	slr6039	GO:0042626	0.0221
slr1647	GO:0080090	<1e-4	sll0445	GO:0009262	<1e-4	sll0871	GO:0016462	<1e-4	sll0444	GO:0044422	<1e-4	ssr6020	GO:0042626	0.0221
slr1442	GO:0031640	<1e-4	ssr2422	GO:0031323	<1e-4	slr6080	GO:0015399	<1e-4	ssr6026	GO:0043226	<1e-4	sll0423	GO:0042626	0.0221
slr0606	GO:0044106	<1e-4	sll0645	GO:0044260	<1e-4	sll0071	GO:0015267	<1e-4	slr1674	GO:0044425	<1e-4	slr1566	GO:0042626	0.0221
slr1464	GO:0044271	<1e-4	slr7013	GO:0080090	<1e-4	slr1095	GO:0015291	<1e-4	sll7030	GO:0044422	<1e-4	sll0611	GO:0042626	0.0221
slr1023	GO:0008150	<1e-4	slr0587	GO:0009263	<1e-4	sll0168	GO:0022832	<1e-4	sll1378	GO:0043226	<1e-4	sll0487	GO:0042626	0.0221
slr2049	GO:0043549	<1e-4	sll1218	GO:0034654	<1e-4	slr1807	GO:0060089	<1e-4	sll0905	GO:0005575	<1e-4	sll1921	GO:0042626	0.0221
sll1698	GO:0044275	<1e-4	sll0524	GO:0009123	<1e-4	slr2018	GO:0043492	<1e-4	sll1613	GO:0044444	<1e-4	slr1753	GO:0042626	0.0221
ssl7045	GO:0055082	<1e-4	slr0581	GO:0051186	<1e-4	sll1926	GO:0015077	<1e-4	sll1526	GO:0044444	<1e-4	sll0063	GO:0042626	0.0221
ssr1391	GO:0051246	<1e-4	sll0268	GO:0019222	<1e-4	sll0783	GO:0016817	<1e-4	slr0731	GO:0044446	<1e-4	slr0065	GO:0042626	0.0221
slr1958	GO:0080090	<1e-4	ssr5121	GO:0046164	<1e-4	sll0180	GO:0003674	<1e-4	sll0888	GO:0044425	<1e-4	sll0406	GO:0042626	0.0221
slr6014	GO:0005996	<1e-4	slr1179	GO:0071554	<1e-4	sll8011	GO:0022891	<1e-4	slr2027	GO:0044464	<1e-4	slr1068	GO:0042626	0.0221
slr7081	GO:0046164	<1e-4	slr1854	GO:0009132	<1e-4	sll0702	GO:0016746	<1e-4	sll1239	GO:0005575	<1e-4	sll1884	GO:0042626	0.0221
slr0416	GO:0031640	<1e-4	slr1636	GO:0065007	<1e-4	sll5067	GO:0016817	<1e-4	slr0667	GO:0044424	<1e-4	slr0960	GO:0042626	0.0221
slr0232	GO:0009132	<1e-4	slr0789	GO:0051186	<1e-4	slr0967	GO:0015077	<1e-4	slr5073	GO:0005575	<1e-4	slr0196	GO:0042626	0.0221
sll0588	GO:0009308	<1e-4	sll0503	GO:0009117	<1e-4	ssr5011	GO:0043167	<1e-4	sll1203	GO:0005575	<1e-4	slr1814	GO:0042626	0.0221
sll1698	GO:0009263	<1e-4	sml0011	GO:0071496	<1e-4	ssl2971	GO:0043492	<1e-4	ssl1923	GO:0005575	<1e-4	sll1252	GO:0042626	0.0221
slr1875	GO:0072527	<1e-4	ssl5099	GO:0006766	<1e-4	ssr1765	GO:0022857	<1e-4	sll0237	GO:0044424	<1e-4	ssl5045	GO:0042626	0.0221
sll5030	GO:0009161	<1e-4	sll5089	GO:0051818	<1e-4	slr0751	GO:0008171	<1e-4	sll1488	GO:0044424	<1e-4	slr2010	GO:0042626	0.0221
slr0317	GO:0019222	<1e-4	sll0456	GO:0009262	<1e-4	sll1135	GO:0015267	<1e-4	sll0810	GO:0044425	<1e-4	sll0518	GO:0042626	0.0221
slr1363	GO:0006091	<1e-4	ssr1528	GO:0006732	<1e-4	slr0575	GO:0003674	<1e-4	ssr6027	GO:0044422	<1e-4	slr6063	GO:0042626	0.0221
slr0728	GO:0006091	<1e-4	sll1934	GO:0090304	<1e-4	sll1272	GO:0015405	<1e-4	sll0294	GO:0005575	<1e-4	ssl3142	GO:0042626	0.0221
slr0613	GO:0006631	<1e-4	sll0781	GO:0051171	<1e-4	ssl7048	GO:0016818	<1e-4	slr2025	GO:0044444	<1e-4	sll8002	GO:0042626	0.0221
sll0595	GO:0005996	<1e-4	slr1670	GO:0046907	<1e-4	slr0959	GO:0008171	<1e-4	sll1464	GO:0044464	<1e-4	ssr0335	GO:0042626	0.0221
sll1671	GO:0044271	<1e-4	slr0801	GO:0016053	<1e-4	sll1092	GO:0015075	<1e-4	slr1425	GO:0044424	<1e-4	ssr5117	GO:0042626	0.0221
sll0498	GO:0009987	<1e-4	slr1425	GO:0046128	<1e-4	slr1852	GO:0003674	<1e-4	sll0456	GO:0005575	<1e-4	sll1160	GO:0042626	0.0221
sll1954	GO:0006082	<1e-4	slr0192	GO:0009142	<1e-4	slr7015	GO:0022890	<1e-4	sll0447	GO:0044424	<1e-4	sll0751	GO:0042626	0.0221
sll1151	GO:0071840	<1e-4	slr1593	GO:0019637	<1e-4	ssr2711	GO:0015293	<1e-4	sll0177	GO:0044425	<1e-4	slr6064	GO:0042626	0.0221
ssr3122	GO:0055114	<1e-4	sll0558	GO:0051171	<1e-4	slr0702	GO:0022892	<1e-4	slr0345	GO:0005575	<1e-4	sll0265	GO:0042626	0.0221
sll1217	GO:0006066	<1e-4	slr1053	GO:0050789	<1e-4	slr1163	GO:0015267	<1e-4	sll0638	GO:0044424	<1e-4	slr5018	GO:0042626	0.0221
sll0360	GO:0009889	<1e-4	sll1495	GO:0009056	<1e-4	sll1715	GO:0016741	<1e-4	slr1919	GO:0044424	<1e-4	sll0815	GO:0042626	0.0221
sll0294	GO:0009057	<1e-4	sll0811	GO:0010468	<1e-4	slr1513	GO:0005342	<1e-4	slr0801	GO:0044446	<1e-4	ssl5129	GO:0042626	0.0221
slr1573	GO:0016054	<1e-4	sll1681	GO:0046907	<1e-4	sll0811	GO:0016818	<1e-4	slr2110	GO:0044446	<1e-4	slr7083	GO:0042626	0.0221
slr1056	GO:0009889	<1e-4	sll1956	GO:0009260	<1e-4	slr5118	GO:0016462	<1e-4	sll0301	GO:0005575	<1e-4	sll1025	GO:0042626	0.0221
sll0482	GO:0009262	<1e-4	slr2011	GO:0072521	<1e-4	sll0266	GO:0005342	<1e-4	sll1222	GO:0044446	<1e-4	ssl1972	GO:0042626	0.0221
sll7087	GO:0009057	<1e-4	slr1032	GO:0055114	<1e-4	slr0431	GO:0016462	<1e-4	slr1378	GO:0044422	<1e-4	sll1509	GO:0042626	0.0221
sll0481	GO:0080090	<1e-4	sll0413	GO:0072527	<1e-4	slr1935	GO:0022832	<1e-4	ssl2807	GO:0043226	<1e-4	sll0284	GO:0042626	0.0221

ssl1577	GO:0006720	<1e-4	sll0984	GO:0009142	<1e-4	sll1372	GO:0043167	<1e-4	slr1413	GO:0044425	<1e-4	slr7011	GO:0042626	0.0221
sll0478	GO:0048518	<1e-4	slr1533	GO:0006631	<1e-4	ssl3291	GO:0016462	<1e-4	ssl0410	GO:0044425	<1e-4	ssl2920	GO:0042626	0.0221
ssr6083	GO:0043170	<1e-4	slr6081	GO:0055114	<1e-4	slr7059	GO:0022838	<1e-4	sll0188	GO:0044425	<1e-4	sll0168	GO:0042626	0.0221
ssr1407	GO:0051347	<1e-4	slr0634	GO:0072521	<1e-4	sll1573	GO:0003674	<1e-4	sll0857	GO:0044425	<1e-4	slr0907	GO:0042626	0.0221
slr1815	GO:0043549	<1e-4	slr0589	GO:0006220	<1e-4	slr0059	GO:0016462	<1e-4	slr0919	GO:0043226	<1e-4	sll1691	GO:0042626	0.0221
sll0473	GO:0006631	<1e-4	sll7070	GO:0080090	<1e-4	slr1179	GO:0016741	<1e-4	slr1911	GO:0005575	<1e-4	sll5026	GO:0042626	0.0221
slr0325	GO:0046483	<1e-4	sll1532	GO:0055082	<1e-4	sll0751	GO:0043492	<1e-4	ssl7045	GO:0044464	<1e-4	slr1468	GO:0042626	0.0221
slr1913	GO:0009123	<1e-4	ssr3402	GO:0046483	<1e-4	slr0740	GO:0015293	<1e-4	sll0281	GO:0005575	<1e-4	slr1768	GO:0042626	0.0221
sll0496	GO:0009987	<1e-4	slr0709	GO:0051716	<1e-4	sll0183	GO:0022892	<1e-4	sll0428	GO:0044424	<1e-4	slr7013	GO:0042626	0.0221
ssr6089	GO:0009259	<1e-4	sll0788	GO:0006066	<1e-4	slr2027	GO:0060089	<1e-4	slr0699	GO:0044422	<1e-4	sll0762	GO:0042626	0.0221
slr1778	GO:0071842	<1e-4	slr0551	GO:0006220	<1e-4	slr1056	GO:0016741	<1e-4	sll1916	GO:0044464	<1e-4	slr2105	GO:0042626	0.0221
ssr2554	GO:0048878	<1e-4	sll1388	GO:0072524	<1e-4	sll0488	GO:0005216	<1e-4	slr0491	GO:0044425	<1e-4	slr0380	GO:0042626	0.0221
sll0608	GO:0019219	<1e-4	slr1066	GO:0072522	<1e-4	sll0069	GO:0015267	<1e-4	sll1563	GO:0044425	<1e-4	slr7016	GO:0042626	0.0221
slr1668	GO:0006082	<1e-4	ssr7036	GO:0065007	<1e-4	sll1049	GO:0003674	<1e-4	slr1613	GO:0005575	<1e-4	ssl1004	GO:0042626	0.0221
slr0689	GO:0008150	<1e-4	slr1923	GO:0044003	<1e-4	ssl8039	GO:0005342	<1e-4	slr0294	GO:0044464	<1e-4	sll0846	GO:0042626	0.0221
slr5112	GO:0008150	<1e-4	slr0751	GO:0009394	<1e-4	sll0597	GO:0015291	<1e-4	slr5102	GO:0044446	<1e-4	sll5033	GO:0042626	0.0221
sll0007	GO:0010468	<1e-4	slr6073	GO:0060255	<1e-4	sll0268	GO:0022803	<1e-4	slr1110	GO:0044464	<1e-4	slr0038	GO:0042626	0.0221
slr0755	GO:0019751	<1e-4	slr0318	GO:0019751	<1e-4	slr0613	GO:0015075	<1e-4	sll1389	GO:0005575	<1e-4	sll1062	GO:0042626	0.0221
ssr6079	GO:0018130	<1e-4	sll1433	GO:0046164	<1e-4	ssl0242	GO:0016462	<1e-4	ssr6046	GO:0044446	<1e-4	sll0478	GO:0042626	0.0221
sll0175	GO:0009263	<1e-4	sll1912	GO:0006576	<1e-4	slr5101	GO:0022857	<1e-4	slr1266	GO:0044425	<1e-4	slr0695	GO:0042626	0.0221
slr1468	GO:0051171	<1e-4	sll1036	GO:0052188	<1e-4	ssl0483	GO:0022890	<1e-4	sll0793	GO:0044446	<1e-4	sll1163	GO:0042626	0.0221
sll1192	GO:0044282	<1e-4	sll1769	GO:0051347	<1e-4	sll1940	GO:0022836	<1e-4	slr1142	GO:0044422	<1e-4	ssl8003	GO:0042626	0.0221
slr0092	GO:0072527	<1e-4	slr1753	GO:0090304	<1e-4	sll7028	GO:0022891	<1e-4	slr1184	GO:0043226	<1e-4	ssl1792	GO:0042626	0.0221
ssl0461	GO:0045184	<1e-4	slr5018	GO:0048518	<1e-4	sll1531	GO:0016746	<1e-4	sll1911	GO:0044464	<1e-4	slr7092	GO:0042626	0.0221
sll1934	GO:0051188	<1e-4	sll1232	GO:0046907	<1e-4	ssr7093	GO:0015399	<1e-4	ssr2912	GO:0005575	<1e-4	ssr7072	GO:0042626	0.0221
ssr6085	GO:0042451	<1e-4	sll0446	GO:0006721	<1e-4	slr2018	GO:0060089	<1e-4	sll0614	GO:0044464	<1e-4	sll0419	GO:0042626	0.0221
sll0590	GO:0048519	<1e-4	slr0491	GO:0009987	<1e-4	sll0283	GO:0015291	<1e-4	sll8035	GO:0043226	<1e-4	slr7014	GO:0042626	0.0221
ssl0750	GO:0009117	<1e-4	sll0350	GO:0043933	<1e-4	ssl3379	GO:0015405	<1e-4	sll1415	GO:0044444	<1e-4	slr1407	GO:0042626	0.0221
slr0554	GO:0043549	<1e-4	sll7077	GO:0043412	<1e-4	sll0737	GO:0016746	<1e-4	ssr1499	GO:0044424	<1e-4	sll1651	GO:0042626	0.0221
sll0447	GO:0034654	<1e-4	slr2003	GO:0072521	<1e-4	sll1773	GO:0022890	<1e-4	slr7010	GO:0044424	<1e-4	slr0145	GO:0042626	0.0221
sll0763	GO:0048878	<1e-4	sll1247	GO:0050896	<1e-4	sll1738	GO:0022832	<1e-4	ssr0759	GO:0044422	<1e-4	ssr0332	GO:0042626	0.0221
slr1464	GO:0019222	<1e-4	sll1752	GO:0009124	<1e-4	slr1917	GO:0043492	<1e-4	slr1397	GO:0044422	<1e-4	sll1130	GO:0042626	0.0221
sll0518	GO:0043170	<1e-4	slr6028	GO:0071842	<1e-4	sll0785	GO:0005342	<1e-4	ssr1558	GO:0044425	<1e-4	ssl2009	GO:0042626	0.0221
slr1533	GO:0072522	<1e-4	ssl3142	GO:0043933	<1e-4	slr1932	GO:0016741	<1e-4	slr1362	GO:0043226	<1e-4	slr0619	GO:0042626	0.0221
sll0815	GO:0071842	<1e-4	slr1468	GO:0009893	<1e-4	slr1885	GO:0043167	<1e-4	sll0862	GO:0044446	<1e-4	slr6013	GO:0042626	0.0221
slr1628	GO:0072527	<1e-4	slr1670	GO:0009142	<1e-4	slr1507	GO:0022803	<1e-4	slr1127	GO:0005575	<1e-4	slr1365	GO:0042626	0.0221
slr0269	GO:0048878	<1e-4	slr1062	GO:0048523	<1e-4	slr0241	GO:0015405	<1e-4	sll0740	GO:0044425	<1e-4	slr1914	GO:0042626	0.0221
slr6005	GO:0009991	<1e-4	slr1195	GO:0044255	<1e-4	slr1270	GO:0022892	<1e-4	ssl0467	GO:0044444	<1e-4	slr1206	GO:0016820	0.022
slr8022	GO:0010556	<1e-4	sll0872	GO:0046394	<1e-4	slr1259	GO:0016462	<1e-4	sll1681	GO:0044446	<1e-4	ssr6086	GO:0016820	0.022
slr0609	GO:0009987	<1e-4	sll0749	GO:0051179	<1e-4	sll1319	GO:0015077	<1e-4	sll0980	GO:0044444	<1e-4	sll7067	GO:0016820	0.022
sll0410	GO:0046128	<1e-4	ssl0350	GO:0009124	<1e-4	sll0590	GO:0016746	<1e-4	ssr5092	GO:0044424	<1e-4	sll1891	GO:0016820	0.022
slr1419	GO:0008150	<1e-4	sll1469	GO:0009199	<1e-4	sll0283	GO:0022890	<1e-4	sll0832	GO:0043226	<1e-4	sll8035	GO:0016820	0.022
slr1203	GO:0006733	<1e-4	sll0406	GO:0009987	<1e-4	slr2103	GO:0015293	<1e-4	sll0614	GO:0044424	<1e-4	sll7031	GO:0016820	0.022
slr0619	GO:0044255	<1e-4	sll5109	GO:0043549	<1e-4	slr0609	GO:0005342	<1e-4	slr1385	GO:0044424	<1e-4	ssr0336	GO:0016820	0.022
ssr1041	GO:0019751	<1e-4	slr0787	GO:0044283	<1e-4	slr0569	GO:0015293	<1e-4	slr1674	GO:0005575	<1e-4	slr0453	GO:0016820	0.022
ssr7035	GO:0034654	<1e-4	sll0162	GO:0048522	<1e-4	sll1132	GO:0016746	<1e-4	slr1537	GO:0043226	<1e-4	ssr6083	GO:0016820	0.022
slr0416	GO:0050801	<1e-4	sll0765	GO:0006766	<1e-4	sll1411	GO:0016817	<1e-4	sll1526	GO:0005575	<1e-4	ssr1114	GO:0016874	0.022
slr1624	GO:0048519	<1e-4	slr0872	GO:0009892	<1e-4	sll0309	GO:0043167	<1e-4	ssl2920	GO:0044464	<1e-4	slr0172	GO:0016874	0.022
slr2092	GO:0005996	<1e-4	slr0337	GO:0018130	<1e-4	sll0606	GO:0015405	<1e-4	sll1717	GO:0044422	<1e-4	sll0225	GO:0016874	0.022
sll0473	GO:0072521	<1e-4	slr2052	GO:0050794	<1e-4	slr1935	GO:0022891	<1e-4	slr1069	GO:0005575	<1e-4	ssr1041	GO:0016820	0.022
slr2110	GO:0006163	<1e-4	slr6022	GO:0008610	<1e-4	sll1306	GO:0008171	<1e-4	sll0102	GO:0044446	<1e-4	slr0325	GO:0016820	0.022
slr6039	GO:0051188	<1e-4	slr1419	GO:0050896	<1e-4	ssr1766	GO:0060089	<1e-4	slr7094	GO:0044424	<1e-4	slr2115	GO:0016874	0.022
slr1815	GO:0072528	<1e-4	sll0400	GO:0006732	<1e-4	slr0249	GO:0022890	<1e-4	slr0509	GO:0043226	<1e-4	slr0273	GO:0016820	0.022
sll0282	GO:0072521	<1e-4	slr1513	GO:0044255	<1e-4	sll0577	GO:0015399	<1e-4	slr1780	GO:0044424	<1e-4	sll1717	GO:0016820	0.022

slr6031	GO:0043648	<1e-4	sll0994	GO:0006766	<1e-4	ssr5117	GO:0015291	<1e-4	slr0635	GO:0044422	<1e-4	slr1070	GO:0016874	0.022
slr1462	GO:0051179	<1e-4	sll0400	GO:0065007	<1e-4	sll1863	GO:0016462	<1e-4	slr0287	GO:0005575	<1e-4	sll1573	GO:0016820	0.022
sll0405	GO:0009057	<1e-4	slr0606	GO:0044003	<1e-4	slr1152	GO:0015405	<1e-4	slr2046	GO:0044444	<1e-4	sll1068	GO:0016820	0.022
ssr2843	GO:0051179	<1e-4	ssr1375	GO:0051188	<1e-4	slr0596	GO:0022890	<1e-4	sll1272	GO:0044444	<1e-4	slr0318	GO:0016874	0.022
sll0839	GO:0060255	<1e-4	slr1353	GO:0008150	<1e-4	ssl1378	GO:0015075	<1e-4	sll1036	GO:0043226	<1e-4	sll0585	GO:0016874	0.022
slr1726	GO:0019222	<1e-4	slr0989	GO:0043436	<1e-4	slr1537	GO:0016817	<1e-4	ssr1391	GO:0044446	<1e-4	sll5003	GO:0016820	0.022
slr1450	GO:0019362	<1e-4	sll1396	GO:0051186	<1e-4	slr1866	GO:0016817	<1e-4	slr0680	GO:0044464	<1e-4	ssl3573	GO:0016820	0.022
sll0456	GO:0034660	<1e-4	slr0551	GO:0052188	<1e-4	slr0689	GO:0016817	<1e-4	ssl1300	GO:0043226	<1e-4	sll7069	GO:0016820	0.022
slr0305	GO:0016054	<1e-4	slr2110	GO:0019222	<1e-4	ssl0352	GO:0015293	<1e-4	slr1095	GO:0044444	<1e-4	slr6075	GO:0016820	0.022
slr1768	GO:0044282	<1e-4	ssl2781	GO:0010556	<1e-4	slr1566	GO:0043167	<1e-4	slr0948	GO:0044464	<1e-4	slr0373	GO:0016874	0.022
slr1470	GO:0048518	<1e-4	slr1032	GO:0009259	<1e-4	sll1352	GO:0043492	<1e-4	sll1749	GO:0044422	<1e-4	ssl2595	GO:0016820	0.022
sll0658	GO:0044248	<1e-4	sll0208	GO:0051347	<1e-4	ssr7079	GO:0022890	<1e-4	slr1110	GO:0005575	<1e-4	slr1236	GO:0016820	0.022
ssl3291	GO:0051179	<1e-4	slr2010	GO:0046128	<1e-4	slr1885	GO:0022832	<1e-4	ssr7036	GO:0005575	<1e-4	sll1730	GO:0016874	0.022
slr1425	GO:0042180	<1e-4	ssr1765	GO:0005996	<1e-4	slr0416	GO:0022838	<1e-4	slr1101	GO:0044424	<1e-4	sll0297	GO:0016874	0.022
slr1619	GO:0006732	<1e-4	slr7095	GO:0006721	<1e-4	slr1918	GO:0022832	<1e-4	sll1730	GO:0005575	<1e-4	ssr1425	GO:0016820	0.022
sll1726	GO:0043549	<1e-4	sll1381	GO:0008150	<1e-4	slr0888	GO:0043492	<1e-4	slr6044	GO:0044424	<1e-4	slr1658	GO:0016874	0.022
sll0147	GO:0051186	<1e-4	slr0601	GO:0009117	<1e-4	sll0397	GO:0043492	<1e-4	sll1024	GO:0044425	<1e-4	ssl5100	GO:0016820	0.022
slr0172	GO:0051246	<1e-4	sll0436	GO:0006733	<1e-4	slr1493	GO:0022890	<1e-4	sll0413	GO:0044444	<1e-4	slr1450	GO:0016820	0.022
slr7082	GO:0052188	<1e-4	sll7086	GO:0009889	<1e-4	ssl2595	GO:0022836	<1e-4	sll2006	GO:0043226	<1e-4	ssr6030	GO:0016820	0.022
sll8032	GO:0051188	<1e-4	slr0771	GO:0080090	<1e-4	slr1110	GO:0015291	<1e-4	slr6066	GO:0044422	<1e-4	sll0678	GO:0016820	0.022
sll7067	GO:0072527	<1e-4	slr1811	GO:0009263	<1e-4	slr1864	GO:0016741	<1e-4	ssr5019	GO:0044444	<1e-4	slr6068	GO:0016820	0.022
slr1240	GO:0006631	<1e-4	ssl8039	GO:0009132	<1e-4	sll0253	GO:0022892	<1e-4	sll0588	GO:0044422	<1e-4	ssl2138	GO:0016820	0.022
slr2003	GO:0031326	<1e-4	slr1187	GO:0051188	<1e-4	sll0424	GO:0043492	<1e-4	slr1699	GO:0005575	<1e-4	ssr6019	GO:0016820	0.022
slr5118	GO:0046164	<1e-4	ssl3142	GO:0016053	<1e-4	slr1999	GO:0015075	<1e-4	sll1715	GO:0005575	<1e-4	slr1789	GO:0016820	0.022
slr0380	GO:0009150	<1e-4	slr1573	GO:0090304	<1e-4	sll1355	GO:0022803	<1e-4	sll5034	GO:0044444	<1e-4	sll0691	GO:0016820	0.022
sll0875	GO:0072528	<1e-4	sll0286	GO:0009165	<1e-4	sll1950	GO:0008171	<1e-4	ssr3570	GO:0044425	<1e-4	ssr7079	GO:0016820	0.022
sll0875	GO:0072521	<1e-4	slr1576	GO:0006066	<1e-4	sll0354	GO:0016818	<1e-4	sll7070	GO:0005575	<1e-4	slr5053	GO:0016820	0.022
ssl0788	GO:0043549	<1e-4	sll0994	GO:0005996	<1e-4	slr0317	GO:0042623	<1e-4	slr1230	GO:0044444	<1e-4	slr0605	GO:0016874	0.022
slr0505	GO:0046907	<1e-4	sll0751	GO:0043648	<1e-4	slr1173	GO:0022836	<1e-4	slr0142	GO:0043226	<1e-4	sll1505	GO:0016874	0.022
sll1390	GO:0071554	<1e-4	sll6053	GO:0044248	<1e-4	ssr5120	GO:0015075	<1e-4	sll1570	GO:0044424	<1e-4	slr0664	GO:0016874	0.022
sll1265	GO:0051246	<1e-4	slr1601	GO:0010468	<1e-4	slr8014	GO:0022832	<1e-4	sll1830	GO:0043226	<1e-4	sll7089	GO:0016820	0.022
slr1923	GO:0006753	<1e-4	slr0262	GO:0006793	<1e-4	sll0933	GO:0022803	<1e-4	slr1161	GO:0044464	<1e-4	slr1944	GO:0016820	0.022
sll0982	GO:0051171	<1e-4	sll0473	GO:0034641	<1e-4	ssl2814	GO:0016746	<1e-4	slr1657	GO:0044424	<1e-4	slr1259	GO:0016874	0.022
slr0489	GO:0019362	<1e-4	sll1340	GO:0046483	<1e-4	slr0981	GO:0016818	<1e-4	sll0931	GO:0043226	<1e-4	slr0801	GO:0016874	0.022
sll0710	GO:0044106	<1e-4	slr6072	GO:0001932	<1e-4	sll0553	GO:0015399	<1e-4	slr7016	GO:0005575	<1e-4	slr1087	GO:0016820	0.022
slr1288	GO:0006733	<1e-4	slr6065	GO:0050789	<1e-4	sll1380	GO:0043492	<1e-4	slr0980	GO:0044422	<1e-4	ssl0788	GO:0016820	0.022
slr0742	GO:0034641	<1e-4	ssr2551	GO:0006066	<1e-4	slr1611	GO:0022838	<1e-4	sll0926	GO:0044425	<1e-4	ssl5008	GO:0016820	0.022
slr2092	GO:0031326	<1e-4	slr0780	GO:0031323	<1e-4	sll1123	GO:0015075	<1e-4	slr0815	GO:0005575	<1e-4	slr1911	GO:0016820	0.022
slr0313	GO:0072528	<1e-4	sll0319	GO:0009309	<1e-4	slr0921	GO:0022857	<1e-4	ssl1300	GO:0005575	<1e-4	ssr1558	GO:0016820	0.022
slr0581	GO:0009123	<1e-4	sll0563	GO:0051188	<1e-4	sll0456	GO:0022832	<1e-4	slr0060	GO:0044424	<1e-4	sll1106	GO:0016820	0.022
sll1352	GO:0043170	<1e-4	slr1484	GO:0048523	<1e-4	sll0584	GO:0022890	<1e-4	ssr7017	GO:0044444	<1e-4	sll1873	GO:0016874	0.022
sll0335	GO:0006576	<1e-4	slr1178	GO:0031326	<1e-4	sll1344	GO:0016746	<1e-4	slr2117	GO:0044446	<1e-4	slr1062	GO:0016874	0.022
sll5097	GO:0009059	<1e-4	slr1468	GO:0031326	<1e-4	slr1116	GO:0015293	<1e-4	slr5023	GO:0005575	<1e-4	slr1980	GO:0016820	0.022
sll0359	GO:0016052	<1e-4	ssr6078	GO:0009259	<1e-4	slr1566	GO:0022857	<1e-4	slr1183	GO:0044446	<1e-4	sll0525	GO:0016820	0.022
slr0207	GO:0051716	<1e-4	slr1863	GO:0071840	<1e-4	slr0645	GO:0022857	<1e-4	sll0286	GO:0044422	<1e-4	slr0702	GO:0016874	0.022
sll0518	GO:0042451	<1e-4	ssr2912	GO:0006733	<1e-4	slr1507	GO:0015267	<1e-4	sll0584	GO:0044444	<1e-4	slr0238	GO:0016820	0.022
sll1203	GO:0071841	<1e-4	slr0655	GO:0046483	<1e-4	slr7011	GO:0043167	<1e-4	slr1718	GO:0044425	<1e-4	sll1738	GO:0016874	0.022
slr7037	GO:0009144	<1e-4	sll1911	GO:0044283	<1e-4	slr0142	GO:0015399	<1e-4	sll0174	GO:0005575	<1e-4	sll1254	GO:0016820	0.022
sll0282	GO:0055114	<1e-4	ssr5106	GO:0009262	<1e-4	sll1089	GO:0022836	<1e-4	sll1906	GO:0044422	<1e-4	ssl2162	GO:0016820	0.022
ssr2554	GO:0009165	<1e-4	sll1166	GO:0043933	<1e-4	sll1862	GO:0022857	<1e-4	slr1557	GO:0044425	<1e-4	slr0291	GO:0016820	0.022
slr0729	GO:0006753	<1e-4	slr1590	GO:0043648	<1e-4	sll0775	GO:0022838	<1e-4	sll0702	GO:0044464	<1e-4	sll0031	GO:0016820	0.022
ssl7021	GO:0050789	<1e-4	slr0821	GO:0009142	<1e-4	slr1990	GO:0022890	<1e-4	slr1866	GO:0044425	<1e-4	slr0637	GO:0016820	0.022
sll1949	GO:0009150	<1e-4	ssr1041	GO:0009259	<1e-4	sll0780	GO:0022857	<1e-4	ssr2781	GO:0044422	<1e-4	slr0975	GO:0016874	0.022
slr0325	GO:0060255	<1e-4	sll0230	GO:0051246	<1e-4	sll1222	GO:0015077	<1e-4	slr1215	GO:0005575	<1e-4	slr1544	GO:0016874	0.022

ssl5096	GO:0006082	<1e-4	slr0013	GO:0044281	<1e-4	slr1142	GO:0043167	<1e-4	sll0160	GO:0044464	<1e-4	sll1267	GO:0016874	0.022
sll1949	GO:0009893	<1e-4	sll0198	GO:0042455	<1e-4	sll1106	GO:0060089	<1e-4	sll1411	GO:0043226	<1e-4	slr0967	GO:0016820	0.022
slr1177	GO:0055082	<1e-4	sll0910	GO:0042451	<1e-4	slr1406	GO:0005342	<1e-4	sll1757	GO:0044422	<1e-4	sll1609	GO:0016820	0.022
sll0298	GO:0034641	<1e-4	slr1288	GO:0051347	<1e-4	sll0309	GO:0015293	<1e-4	slr0569	GO:0043226	<1e-4	sll0281	GO:0016874	0.022
ssl5097	GO:0034654	<1e-4	slr6016	GO:0060255	<1e-4	sll1024	GO:0016818	<1e-4	slr0304	GO:0005575	<1e-4	slr0863	GO:0016820	0.022
ssr2142	GO:0034654	<1e-4	sll0189	GO:0051818	<1e-4	ssl7039	GO:0015293	<1e-4	sll1692	GO:0044446	<1e-4	slr6022	GO:0016820	0.022
slr1519	GO:0080090	<1e-4	sll1640	GO:0072521	<1e-4	ssr0109	GO:0043492	<1e-4	sll0525	GO:0044444	<1e-4	sll1040	GO:0016874	0.022
slr1162	GO:0009892	<1e-4	sll0939	GO:0072528	<1e-4	slr6014	GO:0015075	<1e-4	ssr2317	GO:0044425	<1e-4	ssl7039	GO:0016820	0.022
slr1315	GO:0006163	<1e-4	sll1061	GO:0044260	<1e-4	sll0911	GO:0015077	<1e-4	slr0111	GO:0044425	<1e-4	slr2000	GO:0016820	0.022
sll0488	GO:0048518	<1e-4	slr1762	GO:0044260	<1e-4	slr0941	GO:0016818	<1e-4	slr2038	GO:0044464	<1e-4	sml0011	GO:0016874	0.022
ssl1792	GO:0009161	<1e-4	sll0181	GO:0009126	<1e-4	slr0270	GO:0008171	<1e-4	slr6094	GO:0044422	<1e-4	ssr0759	GO:0016820	0.022
sll1504	GO:0072528	<1e-4	slr1462	GO:0006811	<1e-4	ssr2962	GO:0042623	<1e-4	ssr2554	GO:0005575	<1e-4	slr1413	GO:0016874	0.022
sll8033	GO:0006733	<1e-4	slr1659	GO:0009262	<1e-4	slr1752	GO:0043492	<1e-4	slr1472	GO:0044424	<1e-4	ssr3572	GO:0016820	0.022
sll1119	GO:0008610	<1e-4	ssl3615	GO:0055086	<1e-4	ssl5025	GO:0016746	<1e-4	ssl5015	GO:0044444	<1e-4	sll1882	GO:0016820	0.022
sll1866	GO:0043170	<1e-4	slr1819	GO:0006082	<1e-4	sll0982	GO:0016746	<1e-4	sll1654	GO:0044424	<1e-4	sll1681	GO:0016820	0.022
sll1863	GO:0019222	<1e-4	sll1726	GO:0009132	<1e-4	slr0456	GO:0060089	<1e-4	slr0313	GO:0043226	<1e-4	slr1415	GO:0016874	0.022
ssr2912	GO:0009165	<1e-4	sll1835	GO:0072521	<1e-4	slr0695	GO:0016818	<1e-4	ssr2754	GO:0044444	<1e-4	ssl0467	GO:0016820	0.022
sll0505	GO:0006732	<1e-4	sll0499	GO:0009165	<1e-4	slr1958	GO:0043492	<1e-4	slr0610	GO:0005575	<1e-4	slr0104	GO:0016874	0.022
sll1455	GO:0009126	<1e-4	sll1527	GO:0072522	<1e-4	slr1544	GO:0016817	<1e-4	ssl5129	GO:0044464	<1e-4	sll0847	GO:0016820	0.022
slr1813	GO:0044260	<1e-4	ssr2787	GO:0009161	<1e-4	ssl1520	GO:0042623	<1e-4	sll1511	GO:0044425	<1e-4	ssr0761	GO:0016820	0.022
slr1699	GO:0008150	<1e-4	slr1258	GO:0009056	<1e-4	sll8012	GO:0015077	<1e-4	slr0148	GO:0044446	<1e-4	ssr2711	GO:0016820	0.022
sll1583	GO:0008150	<1e-4	slr1648	GO:0031323	<1e-4	ssr3341	GO:0015075	<1e-4	slr1800	GO:0005575	<1e-4	slr5087	GO:0016820	0.022
slr0272	GO:0009142	<1e-4	sll1373	GO:0009059	<1e-4	sll1348	GO:0005342	<1e-4	sll0400	GO:0005575	<1e-4	ssr3402	GO:0016820	0.022
slr0668	GO:0005996	<1e-4	ssl2162	GO:0006720	<1e-4	ssr0336	GO:0022803	<1e-4	slr6075	GO:0043226	<1e-4	sll0479	GO:0016820	0.022
slr1676	GO:0065007	<1e-4	slr2032	GO:0048878	<1e-4	ssr5121	GO:0022891	<1e-4	slr0784	GO:0044425	<1e-4	sll0539	GO:0016874	0.022
ssr6026	GO:0044275	<1e-4	sll1675	GO:0016054	<1e-4	sll1442	GO:0015405	<1e-4	sll1461	GO:0005575	<1e-4	sll1390	GO:0016874	0.022
slr8044	GO:0009308	<1e-4	slr1576	GO:0090304	<1e-4	slr1990	GO:0015399	<1e-4	sll1504	GO:0043226	<1e-4	sll1054	GO:0016874	0.022
slr2103	GO:0072521	<1e-4	sll1866	GO:0046164	<1e-4	sll1541	GO:0042623	<1e-4	sll1352	GO:0043226	<1e-4	sll0376	GO:0016820	0.022
slr0589	GO:0009259	<1e-4	slr6007	GO:0009259	<1e-4	slr1623	GO:0043167	<1e-4	ssl1923	GO:0044446	<1e-4	slr1425	GO:0016820	0.022
sll8004	GO:0034641	<1e-4	sll1095	GO:0006811	<1e-4	ssl1046	GO:0015405	<1e-4	sll0888	GO:0044446	<1e-4	slr1577	GO:0016820	0.022
slr0380	GO:0046907	<1e-4	slr0728	GO:0052188	<1e-4	sll0625	GO:0022832	<1e-4	slr0815	GO:0044464	<1e-4	sll1950	GO:0016874	0.022
sll7078	GO:0051179	<1e-4	slr6066	GO:0009262	<1e-4	sll0174	GO:0043492	<1e-4	slr2103	GO:0005575	<1e-4	ssl3549	GO:0016820	0.022
sll8027	GO:0050789	<1e-4	sll0198	GO:0051171	<1e-4	sll1173	GO:0060089	<1e-4	sll1250	GO:0005575	<1e-4	slr0712	GO:0016820	0.022
sll0162	GO:0009117	<1e-4	slr2119	GO:0019752	<1e-4	slr1235	GO:0015267	<1e-4	sll0926	GO:0044464	<1e-4	slr6088	GO:0016820	0.022
slr0145	GO:0009263	<1e-4	sll0022	GO:0006811	<1e-4	sll0148	GO:0022857	<1e-4	ssl7046	GO:0044464	<1e-4	slr1513	GO:0016820	0.022
sll0847	GO:0008150	<1e-4	slr6013	GO:0043436	<1e-4	sll1021	GO:0022836	<1e-4	ssl0738	GO:0005575	<1e-4	ssl2069	GO:0016820	0.022
slr1762	GO:0046394	<1e-4	slr1210	GO:0008150	<1e-4	sll0102	GO:0005342	<1e-4	slr0914	GO:0044422	<1e-4	slr0634	GO:0016820	0.022
sll0274	GO:0009057	<1e-4	slr0642	GO:0006720	<1e-4	ssr2317	GO:0022803	<1e-4	ssr2067	GO:0005575	<1e-4	slr1290	GO:0016820	0.022
sll1071	GO:0044248	<1e-4	slr0455	GO:0009893	<1e-4	sll0985	GO:0016817	<1e-4	slr0318	GO:0005575	<1e-4	slr0771	GO:0016820	0.022
slr0380	GO:0034654	<1e-4	ssl0750	GO:0034654	<1e-4	sll1192	GO:0022891	<1e-4	sll1250	GO:0044422	<1e-4	slr0181	GO:0016820	0.022
ssr1114	GO:0009165	<1e-4	slr1944	GO:0006720	<1e-4	sll1783	GO:0005342	<1e-4	sll1586	GO:0044422	<1e-4	slr1590	GO:0016874	0.022
sll1921	GO:0045184	<1e-4	sll1769	GO:0071840	<1e-4	slr1951	GO:0022890	<1e-4	sll0424	GO:0044444	<1e-4	ssr2975	GO:0016820	0.022
sll0167	GO:0055082	<1e-4	ssr2333	GO:0006631	<1e-4	sll0539	GO:0015293	<1e-4	slr0645	GO:0005575	<1e-4	slr5127	GO:0016820	0.022
ssl2471	GO:0009144	<1e-4	sll0174	GO:0006082	<1e-4	slr0392	GO:0005342	<1e-4	sll1926	GO:0005575	<1e-4	sll5132	GO:0016820	0.022
sll1830	GO:0006066	<1e-4	sll0584	GO:0042455	<1e-4	slr1618	GO:0015267	<1e-4	slr0948	GO:0005575	<1e-4	slr1101	GO:0016820	0.022
slr1927	GO:0009394	<1e-4	sll5062	GO:0046128	<1e-4	sll0298	GO:0015075	<1e-4	slr1462	GO:0044422	<1e-4	slr7024	GO:0016874	0.022
slr0453	GO:0055082	<1e-4	sll0545	GO:0045184	<1e-4	sll0595	GO:0016818	<1e-4	slr0509	GO:0044446	<1e-4	slr0780	GO:0016820	0.022
sll1866	GO:0009262	<1e-4	ssr0536	GO:0034641	<1e-4	sll1414	GO:0043167	<1e-4	slr0416	GO:0044444	<1e-4	slr0459	GO:0016820	0.022
sll1401	GO:0001932	<1e-4	slr1413	GO:0043170	<1e-4	slr1215	GO:0005342	<1e-4	sll5063	GO:0044446	<1e-4	slr6081	GO:0016820	0.022
sll0997	GO:0048523	<1e-4	slr7099	GO:0006091	<1e-4	slr1069	GO:0015075	<1e-4	ssr1768	GO:0044425	<1e-4	ssr7035	GO:0016820	0.022
slr0503	GO:0071554	<1e-4	ssl5096	GO:0065007	<1e-4	slr0730	GO:0022832	<1e-4	ssl5068	GO:0044446	<1e-4	sll0749	GO:0016820	0.022
slr0065	GO:0006721	<1e-4	sll0284	GO:0009262	<1e-4	ssr0761	GO:0042623	<1e-4	ssl3177	GO:0044446	<1e-4	slr1474	GO:0016820	0.022
sll0615	GO:0031323	<1e-4	slr0590	GO:0006732	<1e-4	sll0238	GO:0005342	<1e-4	slr5037	GO:0044446	<1e-4	sll0787	GO:0016874	0.022
sll1254	GO:0051246	<1e-4	slr1236	GO:0060255	<1e-4	ssl1046	GO:0005342	<1e-4	sll5132	GO:0044464	<1e-4	sll1052	GO:0016820	0.022

slr0765	GO:0022411	<1e-4	slr1444	GO:0006721	<1e-4	slr0431	GO:0015075	<1e-4	ssr2755	GO:0044422	<1e-4	sll8012	GO:0016820	0.022
slr0514	GO:0006733	<1e-4	sll1319	GO:0006732	<1e-4	slr1767	GO:0015267	<1e-4	slr0503	GO:0044422	<1e-4	slr1624	GO:0016820	0.022
sll0183	GO:0051347	<1e-4	sll1381	GO:0071840	<1e-4	slr0453	GO:0016817	<1e-4	slr0383	GO:0044464	<1e-4	slr0650	GO:0016874	0.022
sll1640	GO:0043549	<1e-4	slr1591	GO:0044260	<1e-4	slr1275	GO:0008171	<1e-4	ssr3122	GO:0044425	<1e-4	sll1340	GO:0016820	0.022
sll1241	GO:0006066	<1e-4	sll0565	GO:0006163	<1e-4	slr1811	GO:0016818	<1e-4	slr0398	GO:0044444	<1e-4	sll1911	GO:0016820	0.022
sll1191	GO:0006091	<1e-4	sll1285	GO:0006766	<1e-4	sll0282	GO:0015075	<1e-4	slr0459	GO:0005575	<1e-4	sll1350	GO:0016874	0.022
slr1659	GO:0044260	<1e-4	sll8001	GO:0046394	<1e-4	slr1880	GO:0022803	<1e-4	sll0564	GO:0044446	<1e-4	sll0442	GO:0016820	0.022
ssr0109	GO:0009124	<1e-4	sll0162	GO:0031640	<1e-4	ssl0312	GO:0022892	<1e-4	slr5077	GO:0043226	<1e-4	slr1152	GO:0016874	0.022
slr1721	GO:0009150	<1e-4	slr0397	GO:0008610	<1e-4	slr1809	GO:0008171	<1e-4	sll0839	GO:0044446	<1e-4	slr5112	GO:0016820	0.022
sll0602	GO:0051186	<1e-4	slr0730	GO:0042180	<1e-4	slr1472	GO:0016462	<1e-4	sll0473	GO:0005575	<1e-4	slr0815	GO:0016874	0.022
sll5132	GO:0048878	<1e-4	slr2120	GO:0009987	<1e-4	sll0141	GO:0005342	<1e-4	sll0625	GO:0044444	<1e-4	sll0298	GO:0016820	0.022
sll1691	GO:0009144	<1e-4	sll1608	GO:0018130	<1e-4	sll0615	GO:0022891	<1e-4	sll1319	GO:0044422	<1e-4	sll0069	GO:0016820	0.022
sll0298	GO:0052111	<1e-4	slr0356	GO:0019222	<1e-4	sll1862	GO:0003674	<1e-4	sll0175	GO:0043226	<1e-4	sll1652	GO:0016820	0.022
slr1376	GO:0043933	<1e-4	slr1704	GO:0055086	<1e-4	slr1174	GO:0022836	<1e-4	sll1123	GO:0005575	<1e-4	slr0670	GO:0016820	0.022
slr1864	GO:0009262	<1e-4	slr1398	GO:0043549	<1e-4	ssr0335	GO:0015077	<1e-4	ssl1520	GO:0044425	<1e-4	sll0595	GO:0016874	0.022
slr1852	GO:0034660	<1e-4	ssr1765	GO:0009893	<1e-4	ssl5031	GO:0043492	<1e-4	slr1956	GO:0044422	<1e-4	sll8040	GO:0016820	0.022
slr1053	GO:0009144	<1e-4	slr6067	GO:0022607	<1e-4	slr1114	GO:0022838	<1e-4	ssl7074	GO:0005575	<1e-4	slr1033	GO:0016874	0.022
slr1721	GO:0006091	<1e-4	slr0509	GO:0051818	<1e-4	slr1618	GO:0015293	<1e-4	slr1195	GO:0044446	<1e-4	slr0914	GO:0016820	0.022
sll1834	GO:0009263	<1e-4	slr7097	GO:0042455	<1e-4	sll0010	GO:0008171	<1e-4	sll5069	GO:0044464	<1e-4	slr2103	GO:0016820	0.022
sll0446	GO:0044283	<1e-4	sll1915	GO:0009987	<1e-4	sll0676	GO:0016741	<1e-4	slr0325	GO:0044464	<1e-4	ssr5074	GO:0016820	0.022
sll1632	GO:0045184	<1e-4	slr1425	GO:0051716	<1e-4	sll1934	GO:0022836	<1e-4	sll8011	GO:0005575	<1e-4	ssr1473	GO:0016820	0.022
slr7016	GO:0044283	<1e-4	slr1474	GO:0051234	<1e-4	sll0742	GO:0016746	<1e-4	sll2011	GO:0005575	<1e-4	slr0510	GO:0016874	0.022
slr1472	GO:0048518	<1e-4	sll0761	GO:0006811	<1e-4	sll0839	GO:0060089	<1e-4	sll0861	GO:0044422	<1e-4	slr1915	GO:0016820	0.022
slr2052	GO:0044282	<1e-4	sll0763	GO:0050896	<1e-4	sll1477	GO:0022832	<1e-4	slr0680	GO:0044444	<1e-4	slr1935	GO:0016820	0.022
slr1098	GO:0009123	<1e-4	slr1944	GO:0072521	<1e-4	slr2018	GO:0005342	<1e-4	slr6074	GO:0005575	<1e-4	slr0728	GO:0016820	0.022
ssr3122	GO:0050794	<1e-4	slr1074	GO:0009259	<1e-4	slr5119	GO:0016818	<1e-4	sll0549	GO:0044425	<1e-4	ssr1528	GO:0016874	0.022
sll8007	GO:0019219	<1e-4	slr1177	GO:0072522	<1e-4	ssr6099	GO:0060089	<1e-4	ssr5121	GO:0044464	<1e-4	ssl1918	GO:0016820	0.022
slr0519	GO:0006766	<1e-4	slr2111	GO:0005996	<1e-4	sll0860	GO:0016818	<1e-4	ssr1765	GO:0044422	<1e-4	sll7090	GO:0016820	0.022
ssr1951	GO:0048523	<1e-4	slr5087	GO:0006066	<1e-4	slr2010	GO:0015077	<1e-4	sll0310	GO:0043226	<1e-4	sll0327	GO:0016820	0.022
ssr2802	GO:0009142	<1e-4	ssr2009	GO:0034641	<1e-4	slr1535	GO:0043167	<1e-4	slr6006	GO:0044422	<1e-4	ssl7074	GO:0016820	0.022
sll0552	GO:0009141	<1e-4	sll0787	GO:0006733	<1e-4	slr1206	GO:0016818	<1e-4	slr7073	GO:0044446	<1e-4	sll0141	GO:0016874	0.022
sll0793	GO:0055114	<1e-4	sll1411	GO:0009150	<1e-4	slr1378	GO:0005342	<1e-4	slr0291	GO:0044425	<1e-4	sll1289	GO:0016820	0.022
slr1541	GO:0042180	<1e-4	sll1359	GO:0006576	<1e-4	slr1854	GO:0015267	<1e-4	sll0007	GO:0044444	<1e-4	slr7071	GO:0016820	0.022
ssr2611	GO:0060255	<1e-4	slr1187	GO:0009161	<1e-4	ssr6030	GO:0015405	<1e-4	sll1531	GO:0044464	<1e-4	slr1690	GO:0016874	0.022
slr0272	GO:0051171	<1e-4	sll0645	GO:0072527	<1e-4	slr1638	GO:0015075	<1e-4	slr1544	GO:0005575	<1e-4	slr0812	GO:0016820	0.022
sll1250	GO:0042180	<1e-4	sll1764	GO:0034654	<1e-4	ssl0353	GO:0042623	<1e-4	slr1544	GO:0044464	<1e-4	slr1862	GO:0016874	0.022
ssl3142	GO:0009199	<1e-4	slr0104	GO:0009991	<1e-4	ssr2843	GO:0016746	<1e-4	slr1670	GO:0005575	<1e-4	sll0419	GO:0004518	0.0217
ssl1417	GO:0006163	<1e-4	slr1519	GO:0071842	<1e-4	sll0361	GO:0003674	<1e-4	ssl3383	GO:0005575	<1e-4	sll0328	GO:0004518	0.0217
slr1816	GO:0009150	<1e-4	sll0703	GO:0071841	<1e-4	sll0177	GO:0022891	<1e-4	slr1032	GO:0044446	<1e-4	sll1232	GO:0004518	0.0217
slr0551	GO:0044106	<1e-4	sll0101	GO:0009199	<1e-4	sll1630	GO:0015291	<1e-4	slr1566	GO:0044444	<1e-4	slr7101	GO:0004518	0.0217
sll1163	GO:0006220	<1e-4	sll5003	GO:0019219	<1e-4	ssl3829	GO:0015291	<1e-4	sll7089	GO:0044444	<1e-4	sll1691	GO:0004518	0.0217
slr6072	GO:0006163	<1e-4	sll0253	GO:0009309	<1e-4	slr5013	GO:0015267	<1e-4	sll1659	GO:0005575	<1e-4	sll1306	GO:0004518	0.0217
slr0731	GO:0046128	<1e-4	slr0941	GO:0048523	<1e-4	slr0729	GO:0015291	<1e-4	slr0059	GO:0044424	<1e-4	ssl3383	GO:0004518	0.0217
slr0668	GO:0006766	<1e-4	slr1616	GO:0048522	<1e-4	sll0572	GO:0015077	<1e-4	ssr3154	GO:0044425	<1e-4	sll1160	GO:0004518	0.0217
slr0058	GO:0065007	<1e-4	slr1958	GO:0009141	<1e-4	ssl0750	GO:0043167	<1e-4	ssl2920	GO:0044425	<1e-4	slr6063	GO:0004518	0.0217
sll0301	GO:0046394	<1e-4	slr0937	GO:0044281	<1e-4	slr1702	GO:0003674	<1e-4	ssr5011	GO:0044446	<1e-4	slr0065	GO:0004518	0.0217
sll0861	GO:0051716	<1e-4	ssr0536	GO:0051234	<1e-4	sll1608	GO:0015075	<1e-4	slr1327	GO:0044422	<1e-4	slr1419	GO:0004518	0.0217
slr0863	GO:0016054	<1e-4	sll1530	GO:0072527	<1e-4	slr0459	GO:0005342	<1e-4	ssr2615	GO:0044422	<1e-4	ssr6003	GO:0004518	0.0217
sll0736	GO:0019752	<1e-4	slr1073	GO:0009394	<1e-4	sll1461	GO:0060089	<1e-4	slr0431	GO:0005575	<1e-4	ssl2920	GO:0004518	0.0217
sll1873	GO:0009057	<1e-4	ssl7046	GO:0034654	<1e-4	sll0996	GO:0015267	<1e-4	slr0272	GO:0044464	<1e-4	ssl8003	GO:0004518	0.0217
sll1024	GO:0009309	<1e-4	sll1680	GO:0072522	<1e-4	sll0595	GO:0015267	<1e-4	slr6016	GO:0044444	<1e-4	sll0609	GO:0004518	0.0217
sll0930	GO:0006631	<1e-4	sll0444	GO:0034654	<1e-4	sll1063	GO:0022803	<1e-4	sll1510	GO:0005575	<1e-4	ssl2781	GO:0004518	0.0217
slr0554	GO:0009150	<1e-4	slr1619	GO:0048878	<1e-4	slr1920	GO:0022890	<1e-4	slr0303	GO:0044446	<1e-4	slr6004	GO:0004518	0.0217
slr0521	GO:0072522	<1e-4	ssl2996	GO:0043412	<1e-4	slr0810	GO:0003674	<1e-4	sll0931	GO:0044422	<1e-4	slr1260	GO:0004518	0.0217

ssr1391	GO:0048518	<1e-4	slr0325	GO:0022607	<1e-4	slr5023	GO:0022803	<1e-4	sll0298	GO:0044425	<1e-4	slr0821	GO:0004518	0.0217
ssr1765	GO:0042180	<1e-4	ssl5091	GO:0009124	<1e-4	slr1658	GO:0015077	<1e-4	sll1151	GO:0044464	<1e-4	slr7014	GO:0004518	0.0217
sll0293	GO:0044282	<1e-4	sll0678	GO:0071841	<1e-4	slr2125	GO:0015399	<1e-4	ssl3177	GO:0044422	<1e-4	slr6005	GO:0004518	0.0217
slr1218	GO:0022411	<1e-4	sll1355	GO:0051716	<1e-4	slr1570	GO:0043167	<1e-4	sll1659	GO:0043226	<1e-4	sll5130	GO:0004518	0.0217
ssl5099	GO:0009144	<1e-4	sll1350	GO:0006732	<1e-4	slr1384	GO:0016746	<1e-4	slr0049	GO:0044422	<1e-4	slr1468	GO:0004518	0.0217
sll5090	GO:0046907	<1e-4	slr2119	GO:0046394	<1e-4	slr0637	GO:0015291	<1e-4	sll0532	GO:0005575	<1e-4	ssr5117	GO:0004518	0.0217
sll1135	GO:0043412	<1e-4	sll0572	GO:0044003	<1e-4	slr0468	GO:0015075	<1e-4	slr0625	GO:0044446	<1e-4	slr1178	GO:0004518	0.0217
slr1603	GO:0016054	<1e-4	slr1062	GO:0044283	<1e-4	ssl2069	GO:0022891	<1e-4	slr6014	GO:0005575	<1e-4	slr1464	GO:0004518	0.0217
ssr1698	GO:0051171	<1e-4	sll0932	GO:0046907	<1e-4	slr1880	GO:0005342	<1e-4	slr1258	GO:0044446	<1e-4	sll1355	GO:0004518	0.0217
slr1612	GO:0009150	<1e-4	slr1762	GO:0052111	<1e-4	ssr6062	GO:0022836	<1e-4	ssl0467	GO:0005575	<1e-4	sll1130	GO:0004518	0.0217
slr6106	GO:0009144	<1e-4	slr1464	GO:0080090	<1e-4	slr1534	GO:0022857	<1e-4	sll0283	GO:0005575	<1e-4	sll0846	GO:0004518	0.0217
sll0661	GO:0009144	<1e-4	sll1736	GO:0019751	<1e-4	sll0147	GO:0016818	<1e-4	slr6008	GO:0044464	<1e-4	slr0619	GO:0004518	0.0217
sll0996	GO:0006631	<1e-4	sll0451	GO:0071496	<1e-4	sll1315	GO:0003674	<1e-4	sll0405	GO:0005575	<1e-4	sll1696	GO:0004518	0.0217
ssr5019	GO:0072522	<1e-4	slr0398	GO:0044271	<1e-4	slr2060	GO:0015075	<1e-4	ssl1263	GO:0044422	<1e-4	sll0481	GO:0004518	0.0217
ssl2069	GO:0072521	<1e-4	slr1659	GO:0019219	<1e-4	slr1240	GO:0005342	<1e-4	sll1961	GO:0044464	<1e-4	slr1188	GO:0004518	0.0217
sll1060	GO:0019362	<1e-4	sll0266	GO:0044281	<1e-4	slr1619	GO:0015291	<1e-4	slr0273	GO:0044464	<1e-4	sll0751	GO:0004518	0.0217
slr6031	GO:0031323	<1e-4	sll1512	GO:0072524	<1e-4	ssr2755	GO:0043167	<1e-4	slr0476	GO:0043226	<1e-4	sll5026	GO:0004518	0.0217
sll0658	GO:0019362	<1e-4	sll7077	GO:0044003	<1e-4	sll0269	GO:0022892	<1e-4	sll1500	GO:0044464	<1e-4	ssr2912	GO:0004518	0.0217
slr1533	GO:0006082	<1e-4	ssl2920	GO:0043648	<1e-4	slr1263	GO:0043167	<1e-4	slr1143	GO:0044425	<1e-4	slr8014	GO:0004518	0.0217
sll0703	GO:0008150	<1e-4	slr1110	GO:0048522	<1e-4	slr0380	GO:0015075	<1e-4	slr1045	GO:0044424	<1e-4	slr0695	GO:0004518	0.0217
ssr6019	GO:0051234	<1e-4	ssr2060	GO:0009394	<1e-4	sll1715	GO:0022890	<1e-4	slr7058	GO:0005575	<1e-4	sll0994	GO:0004518	0.0217
slr0313	GO:0019219	<1e-4	slr1114	GO:0048878	<1e-4	ssl0312	GO:0043167	<1e-4	slr1906	GO:0044446	<1e-4	slr1186	GO:0004518	0.0217
sll0007	GO:0009309	<1e-4	sll0060	GO:0044248	<1e-4	slr0358	GO:0015293	<1e-4	slr1056	GO:0005575	<1e-4	ssr2611	GO:0004518	0.0217
slr1152	GO:0006066	<1e-4	sll1659	GO:0009056	<1e-4	sll1170	GO:0042623	<1e-4	slr0913	GO:0044464	<1e-4	sll5090	GO:0004518	0.0217
slr0300	GO:0051716	<1e-4	slr1674	GO:0051347	<1e-4	sll1752	GO:0015399	<1e-4	sll0647	GO:0044444	<1e-4	sgl0002	GO:0004518	0.0217
slr0553	GO:0016054	<1e-4	ssl1300	GO:0006091	<1e-4	ssr1407	GO:0022891	<1e-4	slr1315	GO:0044424	<1e-4	sll0518	GO:0004518	0.0217
slr1307	GO:0051818	<1e-4	ssr1698	GO:0019637	<1e-4	slr0909	GO:0016462	<1e-4	sll1509	GO:0043226	<1e-4	slr6006	GO:0004518	0.0217
sll0853	GO:0033013	<1e-4	ssl3291	GO:0046394	<1e-4	slr0509	GO:0022890	<1e-4	slr2048	GO:0043226	<1e-4	slr1363	GO:0004518	0.0217
slr7025	GO:0051234	<1e-4	sll1464	GO:0065007	<1e-4	sll1658	GO:0015267	<1e-4	slr1648	GO:0005575	<1e-4	sll1401	GO:0004518	0.0217
sll0737	GO:0052188	<1e-4	slr1097	GO:0065007	<1e-4	sll1570	GO:0043492	<1e-4	slr1807	GO:0005575	<1e-4	sll5046	GO:0004518	0.0217
slr1658	GO:0046907	<1e-4	ssl1004	GO:0050789	<1e-4	sll0382	GO:0043492	<1e-4	slr6073	GO:0044422	<1e-4	sll0658	GO:0004518	0.0217
sll0216	GO:0006082	<1e-4	sll8002	GO:0033013	<1e-4	ssr2009	GO:0022892	<1e-4	slr0876	GO:0043226	<1e-4	sll1765	GO:0004518	0.0217
sll0861	GO:0006396	<1e-4	sll1486	GO:0010468	<1e-4	ssl2420	GO:0022803	<1e-4	slr0709	GO:0005575	<1e-4	slr1069	GO:0004518	0.0217
sll0428	GO:0009308	<1e-4	slr0818	GO:0009165	<1e-4	sll1218	GO:0015291	<1e-4	slr8014	GO:0005575	<1e-4	sll1485	GO:0004518	0.0217
sll1219	GO:0043648	<1e-4	slr1699	GO:0019362	<1e-4	slr1301	GO:0022892	<1e-4	ssr5020	GO:0043226	<1e-4	sll7063	GO:0004518	0.0217
slr1025	GO:0006733	<1e-4	slr0769	GO:0051716	<1e-4	slr0334	GO:0022890	<1e-4	slr1177	GO:0044424	<1e-4	slr7059	GO:0004518	0.0217
sll0266	GO:0006793	<1e-4	sll0839	GO:0072524	<1e-4	sll0446	GO:0022832	<1e-4	sll1730	GO:0044464	<1e-4	ssr6048	GO:0004518	0.0217
slr0816	GO:0050789	<1e-4	slr1599	GO:0009141	<1e-4	sll1061	GO:0043167	<1e-4	slr0482	GO:0005575	<1e-4	ssl5031	GO:0004518	0.0217
slr6045	GO:0016053	<1e-4	ssl0312	GO:0071841	<1e-4	slr0287	GO:0015075	<1e-4	sll0266	GO:0044464	<1e-4	sll1378	GO:0004518	0.0217
slr2005	GO:0046164	<1e-4	sll1004	GO:0042180	<1e-4	sll1071	GO:0022832	<1e-4	sgl0001	GO:0005575	<1e-4	sll0241	GO:0004518	0.0217
ssr0536	GO:0006163	<1e-4	sll0192	GO:0009889	<1e-4	sll1162	GO:0043492	<1e-4	ssr7036	GO:0044464	<1e-4	sll0742	GO:0004518	0.0217
sll1726	GO:0072527	<1e-4	slr7094	GO:0019222	<1e-4	slr8014	GO:0060089	<1e-4	ssl2996	GO:0005575	<1e-4	slr0059	GO:0004518	0.0217
slr5102	GO:0009892	<1e-4	sll1372	GO:0042455	<1e-4	slr6088	GO:0022832	<1e-4	slr0243	GO:0044425	<1e-4	sll0293	GO:0004518	0.0217
sll0595	GO:0022411	<1e-4	slr7095	GO:0051171	<1e-4	sll7028	GO:0015399	<1e-4	slr1415	GO:0044444	<1e-4	sll0762	GO:0004518	0.0217
slr1970	GO:0006766	<1e-4	ssl2971	GO:0009056	<1e-4	ssl7042	GO:0043167	<1e-4	sll0669	GO:0044424	<1e-4	slr2101	GO:0004518	0.0217
slr1533	GO:0065007	<1e-4	slr1932	GO:0048523	<1e-4	slr1056	GO:0043167	<1e-4	slr0964	GO:0044446	<1e-4	sll0860	GO:0004518	0.0217
sll1652	GO:0051716	<1e-4	sll0827	GO:0048878	<1e-4	ssr7017	GO:0043492	<1e-4	sll0243	GO:0044446	<1e-4	ssr3189	GO:0004518	0.0217
slr0594	GO:0018130	<1e-4	slr0483	GO:0016052	<1e-4	slr1726	GO:0015291	<1e-4	ssr2998	GO:0043226	<1e-4	slr1576	GO:0004518	0.0217
sll1965	GO:0043412	<1e-4	slr1127	GO:0005996	<1e-4	ssl7045	GO:0022838	<1e-4	sll0980	GO:0043226	<1e-4	sll0597	GO:0004518	0.0217
slr6066	GO:0071496	<1e-4	slr0971	GO:0019362	<1e-4	sll1606	GO:0015399	<1e-4	sll1765	GO:0044446	<1e-4	ssl3451	GO:0004518	0.0217
sll0394	GO:0045184	<1e-4	slr1800	GO:0042180	<1e-4	ssl6035	GO:0003674	<1e-4	sll0846	GO:0044422	<1e-4	slr1116	GO:0004518	0.0217
slr0305	GO:0001932	<1e-4	slr1188	GO:0044271	<1e-4	slr0989	GO:0022891	<1e-4	slr0232	GO:0044464	<1e-4	slr1998	GO:0004518	0.0217
ssl3573	GO:0009142	<1e-4	slr1659	GO:0006811	<1e-4	slr0271	GO:0043167	<1e-4	sll0294	GO:0044422	<1e-4	sll0176	GO:0004518	0.0217
slr1163	GO:0016052	<1e-4	slr1572	GO:0009991	<1e-4	slr7060	GO:0022803	<1e-4	sll1656	GO:0044425	<1e-4	ssl1498	GO:0004518	0.0217

slr1150	GO:0009126	<1e-4	sll1396	GO:0051246	<1e-4	ssr3129	GO:0060089	<1e-4	slr0594	GO:0044464	<1e-4	slr1104	GO:0004518	0.0217
sll0446	GO:0034654	<1e-4	sll1002	GO:0006082	<1e-4	slr1547	GO:0005342	<1e-4	sll0442	GO:0005575	<1e-4	sll0630	GO:0004518	0.0217
sll0325	GO:0009117	<1e-4	slr7015	GO:0009263	<1e-4	sll0911	GO:0022891	<1e-4	slr0664	GO:0043226	<1e-4	sll5061	GO:0004518	0.0217
ssl5025	GO:0006732	<1e-4	sll0382	GO:0051347	<1e-4	sll0742	GO:0015399	<1e-4	slr1122	GO:0005575	<1e-4	slr0592	GO:0004518	0.0217
sll1532	GO:0008610	<1e-4	ssr2611	GO:0005996	<1e-4	slr0326	GO:0022832	<1e-4	sll0505	GO:0044444	<1e-4	slr1362	GO:0004518	0.0217
slr1177	GO:0009987	<1e-4	slr1648	GO:0080090	<1e-4	ssr1041	GO:0022892	<1e-4	sll0564	GO:0005575	<1e-4	sll0803	GO:0004518	0.0217
sll0241	GO:0046164	<1e-4	slr1397	GO:0050896	<1e-4	ssr0759	GO:0022836	<1e-4	slr0318	GO:0044444	<1e-4	ssl5098	GO:0004518	0.0217
slr1593	GO:0006811	<1e-4	ssr1258	GO:0009059	<1e-4	slr1863	GO:0016817	<1e-4	slr0770	GO:0044464	<1e-4	ssl1004	GO:0004518	0.0217
slr1573	GO:0034641	<1e-4	ssl5103	GO:0046394	<1e-4	sll1832	GO:0016818	<1e-4	slr2118	GO:0044425	<1e-4	slr1677	GO:0004518	0.0217
sll0609	GO:0006576	<1e-4	slr0013	GO:0009124	<1e-4	slr1768	GO:0022891	<1e-4	slr7014	GO:0044422	<1e-4	sll1960	GO:0004518	0.0217
slr2121	GO:0006091	<1e-4	slr0569	GO:0006733	<1e-4	slr1530	GO:0015267	<1e-4	slr1047	GO:0005575	<1e-4	ssl1520	GO:0004518	0.0217
ssr1766	GO:0072527	<1e-4	sll0157	GO:0045184	<1e-4	ssl6061	GO:0060089	<1e-4	slr6009	GO:0044424	<1e-4	slr2032	GO:0004518	0.0217
ssr2803	GO:0080090	<1e-4	slr0453	GO:0055086	<1e-4	ssr1473	GO:0015293	<1e-4	slr1815	GO:0044422	<1e-4	slr1162	GO:0004518	0.0217
sll0545	GO:0050794	<1e-4	ssl5027	GO:0071840	<1e-4	slr1601	GO:0015405	<1e-4	sll0376	GO:0044446	<1e-4	slr7023	GO:0004518	0.0217
ssl2471	GO:0071842	<1e-4	slr0821	GO:0010556	<1e-4	sll0670	GO:0015075	<1e-4	slr0658	GO:0044425	<1e-4	ssr2422	GO:0004518	0.0217
slr2119	GO:0006163	<1e-4	slr1847	GO:0044281	<1e-4	ssl0109	GO:0015293	<1e-4	sll1411	GO:0044444	<1e-4	slr0655	GO:0004518	0.0217
sll0496	GO:0050896	<1e-4	slr6009	GO:0048878	<1e-4	sll1926	GO:0016817	<1e-4	sgl0002	GO:0044424	<1e-4	sll0872	GO:0004518	0.0217
sll1749	GO:0044260	<1e-4	slr0243	GO:0018130	<1e-4	ssr2754	GO:0022832	<1e-4	sll0493	GO:0044444	<1e-4	ssr7036	GO:0004518	0.0217
slr1895	GO:0009165	<1e-4	slr1668	GO:0043933	<1e-4	slr0483	GO:0022832	<1e-4	sll1002	GO:0044422	<1e-4	ssr0335	GO:0004518	0.0217
slr6075	GO:0055114	<1e-4	slr1977	GO:0044275	<1e-4	sll1939	GO:0016741	<1e-4	sll0602	GO:0044464	<1e-4	slr1753	GO:0004518	0.0217
slr0208	GO:0044260	<1e-4	sll1510	GO:0019362	<1e-4	slr0594	GO:0060089	<1e-4	slr1932	GO:0043226	<1e-4	sll1921	GO:0004518	0.0217
slr2025	GO:0031640	<1e-4	slr0689	GO:0019222	<1e-4	sll1021	GO:0015405	<1e-4	ssl0832	GO:0044425	<1e-4	sll5032	GO:0004518	0.0217
sll0208	GO:0001932	<1e-4	slr1667	GO:0006082	<1e-4	ssr0761	GO:0015075	<1e-4	slr0960	GO:0044446	<1e-4	slr0869	GO:0004518	0.0217
sll0446	GO:0019637	<1e-4	slr1790	GO:0071842	<1e-4	sll1942	GO:0016741	<1e-4	slr1568	GO:0044444	<1e-4	slr1535	GO:0004518	0.0217
ssl5064	GO:0005996	<1e-4	sll0543	GO:0009059	<1e-4	sll1068	GO:0015405	<1e-4	sll0827	GO:0005575	<1e-4	slr1600	GO:0004518	0.0217
slr2032	GO:0006631	<1e-4	slr0971	GO:0009141	<1e-4	slr6074	GO:0022891	<1e-4	slr0364	GO:0005575	<1e-4	slr1923	GO:0004518	0.0217
slr6016	GO:0044260	<1e-4	slr7092	GO:0019222	<1e-4	slr1513	GO:0022832	<1e-4	sll0185	GO:0044424	<1e-4	slr0356	GO:0004518	0.0217
ssl2065	GO:0006163	<1e-4	slr1179	GO:0044003	<1e-4	slr0885	GO:0016817	<1e-4	slr0249	GO:0044424	<1e-4	sll1022	GO:0004518	0.0217
ssr5020	GO:0010556	<1e-4	sll8032	GO:0044003	<1e-4	ssl2733	GO:0022838	<1e-4	ssl3549	GO:0044464	<1e-4	slr6101	GO:0004518	0.0217
sll0785	GO:0010556	<1e-4	ssr2615	GO:0009117	<1e-4	sll1426	GO:0015267	<1e-4	slr0059	GO:0044444	<1e-4	ssr6085	GO:0004518	0.0217
sll1472	GO:0042451	<1e-4	sll0335	GO:0019752	<1e-4	sll0298	GO:0016741	<1e-4	slr0059	GO:0005575	<1e-4	sll1004	GO:0004518	0.0217
ssl2717	GO:0006732	<1e-4	sll5004	GO:0016053	<1e-4	slr1814	GO:0022838	<1e-4	slr2004	GO:0044424	<1e-4	slr7092	GO:0004518	0.0217
sll0761	GO:0009123	<1e-4	sll0263	GO:0051171	<1e-4	ssr5121	GO:0022890	<1e-4	sll7064	GO:0044424	<1e-4	ssl2717	GO:0004518	0.0217
slr1127	GO:0019222	<1e-4	slr2027	GO:0071841	<1e-4	sll0141	GO:0022891	<1e-4	slr1507	GO:0044425	<1e-4	sll0853	GO:0004518	0.0217
ssl7074	GO:0050794	<1e-4	sll1373	GO:0009889	<1e-4	slr1223	GO:0016818	<1e-4	sll1763	GO:0044464	<1e-4	slr1273	GO:0004518	0.0217
ssl3382	GO:0071554	<1e-4	slr0692	GO:0042180	<1e-4	sll8012	GO:0022803	<1e-4	slr1939	GO:0043226	<1e-4	sll7047	GO:0004518	0.0217
slr1425	GO:0080090	<1e-4	sll0875	GO:0080090	<1e-4	sll1219	GO:0022890	<1e-4	slr5127	GO:0044422	<1e-4	slr0168	GO:0004518	0.0217
slr0619	GO:0006766	<1e-4	sll1024	GO:0046907	<1e-4	sll0984	GO:0022857	<1e-4	slr0313	GO:0044422	<1e-4	sll0498	GO:0004518	0.0217
ssr2802	GO:0006732	<1e-4	slr1573	GO:0072528	<1e-4	sll1730	GO:0060089	<1e-4	slr0625	GO:0044422	<1e-4	ssl5007	GO:0004518	0.0217
sll0268	GO:0016053	<1e-4	slr1619	GO:0071840	<1e-4	sll0732	GO:0022838	<1e-4	sll1862	GO:0043226	<1e-4	slr0552	GO:0004518	0.0217
sll0406	GO:0034641	<1e-4	sll1766	GO:0072524	<1e-4	ssl0109	GO:0015075	<1e-4	sll0267	GO:0043226	<1e-4	slr7012	GO:0004518	0.0217
sll0216	GO:0009142	<1e-4	sll1511	GO:0044283	<1e-4	slr0514	GO:0016817	<1e-4	sll7006	GO:0043226	<1e-4	ssr5120	GO:0004518	0.0217
slr6045	GO:0009141	<1e-4	slr1470	GO:0009308	<1e-4	slr1196	GO:0022890	<1e-4	sll0310	GO:0044464	<1e-4	ssr6020	GO:0004518	0.0217
sll1873	GO:0044255	<1e-4	sll1934	GO:0006082	<1e-4	sll0763	GO:0003674	<1e-4	slr0168	GO:0005575	<1e-4	ssl1923	GO:0004518	0.0217
sll1225	GO:0009124	<1e-4	slr1864	GO:0046128	<1e-4	slr1406	GO:0022891	<1e-4	slr5017	GO:0044464	<1e-4	sll0611	GO:0004518	0.0217
slr1990	GO:0009892	<1e-4	slr0294	GO:0005996	<1e-4	slr0602	GO:0022890	<1e-4	slr1513	GO:0044422	<1e-4	slr0598	GO:0004518	0.0217
sll0735	GO:0055086	<1e-4	slr0519	GO:0043436	<1e-4	sll1830	GO:0022857	<1e-4	slr0516	GO:0044425	<1e-4	sll0710	GO:0004518	0.0217
slr0111	GO:0044271	<1e-4	slr6064	GO:0051234	<1e-4	slr0262	GO:0022890	<1e-4	ssl7022	GO:0044422	<1e-4	sll7043	GO:0004518	0.0217
ssl2064	GO:0034641	<1e-4	sll0023	GO:0043170	<1e-4	slr1618	GO:0022857	<1e-4	slr6005	GO:0005575	<1e-4	slr0108	GO:0005515	0.0216
slr1530	GO:0001932	<1e-4	sll0496	GO:0008610	<1e-4	sll1761	GO:0003674	<1e-4	slr0765	GO:0044444	<1e-4	sll1995	GO:0005515	0.0216
slr6007	GO:0051716	<1e-4	ssl3829	GO:0006163	<1e-4	slr0482	GO:0022803	<1e-4	slr0876	GO:0044422	<1e-4	ssr3154	GO:0005515	0.0216
ssr6089	GO:0009308	<1e-4	slr0149	GO:0042180	<1e-4	slr1163	GO:0015077	<1e-4	slr1571	GO:0005575	<1e-4	sll0656	GO:0005515	0.0216
ssr1375	GO:0065007	<1e-4	slr1819	GO:0051246	<1e-4	slr6013	GO:0016818	<1e-4	ssl1255	GO:0043226	<1e-4	sll2006	GO:0004518	0.0213
sll1751	GO:0044255	<1e-4	sll1250	GO:0031326	<1e-4	ssr2912	GO:0022890	<1e-4	slr7095	GO:0044422	<1e-4	ssr3532	GO:0004518	0.0213

slr1127	GO:0043170	<1e-4	slr0262	GO:0051188	<1e-4	sll0909	GO:0022803	<1e-4	sll1002	GO:0043226	<1e-4	ssr1698	GO:0004518	0.0213
sll1504	GO:0051246	<1e-4	slr1699	GO:0009199	<1e-4	slr0887	GO:0015077	<1e-4	slr2119	GO:0044424	<1e-4	slr1196	GO:0004518	0.0213
sll1239	GO:0050789	<1e-4	sll1583	GO:0080090	<1e-4	sll0274	GO:0015293	<1e-4	slr0699	GO:0044444	<1e-4	sll1218	GO:0004518	0.0213
slr0053	GO:0006631	<1e-4	sll1188	GO:0016052	<1e-4	slr1307	GO:0015291	<1e-4	slr1083	GO:0044424	<1e-4	sll1433	GO:0004518	0.0213
slr0287	GO:0044282	<1e-4	slr1342	GO:0034654	<1e-4	sll0871	GO:0060089	<1e-4	slr1628	GO:0005575	<1e-4	slr1533	GO:0004518	0.0213
sll1736	GO:0006720	<1e-4	sll0487	GO:0009892	<1e-4	slr8044	GO:0022890	<1e-4	slr6073	GO:0005575	<1e-4	ssl0787	GO:0004518	0.0213
sll0242	GO:0019637	<1e-4	slr0145	GO:0052188	<1e-4	slr0962	GO:0022892	<1e-4	sll0272	GO:0005575	<1e-4	slr0503	GO:0004518	0.0213
sll1632	GO:0034660	<1e-4	slr1338	GO:0009144	<1e-4	sll1830	GO:0022892	<1e-4	slr1923	GO:0044425	<1e-4	slr1674	GO:0004518	0.0213
slr2000	GO:0019752	<1e-4	slr1676	GO:0009126	<1e-4	ssl0242	GO:0015077	<1e-4	sll0181	GO:0044444	<1e-4	sll1934	GO:0004518	0.0213
ssr3532	GO:0055086	<1e-4	slr0179	GO:0009059	<1e-4	sll0597	GO:0022803	<1e-4	sll0508	GO:0044446	<1e-4	sll0854	GO:0004518	0.0213
slr1110	GO:0010468	<1e-4	slr1927	GO:0009142	<1e-4	slr5077	GO:0022836	<1e-4	ssl2148	GO:0044444	<1e-4	slr0876	GO:0004518	0.0213
sll1735	GO:0009893	<1e-4	slr0305	GO:0009056	<1e-4	sll0218	GO:0016818	<1e-4	ssr1256	GO:0044446	<1e-4	slr1353	GO:0004518	0.0213
slr1095	GO:0055114	<1e-4	sll0982	GO:0072522	<1e-4	sll0181	GO:0015075	<1e-4	ssr6078	GO:0044446	<1e-4	slr1194	GO:0004518	0.0213
slr1260	GO:0042451	<1e-4	sll0253	GO:0044248	<1e-4	sll0499	GO:0015293	<1e-4	sll1825	GO:0044446	<1e-4	sll0508	GO:0004518	0.0213
sll1654	GO:0065007	<1e-4	ssr3572	GO:0043549	<1e-4	slr0431	GO:0015293	<1e-4	slr1737	GO:0044424	<1e-4	slr0250	GO:0004518	0.0213
sll1979	GO:0009123	<1e-4	sll0294	GO:0050794	<1e-4	slr0695	GO:0022857	<1e-4	slr1618	GO:0044425	<1e-4	sll1942	GO:0004518	0.0213
sll0980	GO:0044003	<1e-4	sll0354	GO:0051716	<1e-4	slr1677	GO:0005342	<1e-4	sll0760	GO:0005575	<1e-4	slr0937	GO:0004518	0.0213
ssr0109	GO:0009889	<1e-4	ssr2333	GO:0060255	<1e-4	ssr6086	GO:0016818	<1e-4	ssl6061	GO:0044444	<1e-4	sll0382	GO:0004518	0.0213
slr0376	GO:0019219	<1e-4	ssr3467	GO:0090304	<1e-4	slr8014	GO:0015399	<1e-4	slr2012	GO:0044464	<1e-4	slr1066	GO:0004518	0.0213
sll0625	GO:0006220	<1e-4	sll0265	GO:0019751	<1e-4	sll0444	GO:0015291	<1e-4	slr6101	GO:0044444	<1e-4	sll1586	GO:0004518	0.0213
slr0887	GO:0045184	<1e-4	sll1965	GO:0045184	<1e-4	slr1470	GO:0015405	<1e-4	sll0314	GO:0044444	<1e-4	slr0423	GO:0004518	0.0213
slr7012	GO:0048519	<1e-4	sll0939	GO:0034641	<1e-4	slr0923	GO:0016817	<1e-4	ssr2060	GO:0005575	<1e-4	sll1174	GO:0004518	0.0213
sll0997	GO:0046164	<1e-4	sll0096	GO:0051818	<1e-4	slr0023	GO:0016746	<1e-4	sll0735	GO:0044464	<1e-4	sll1528	GO:0005975	0.0212
slr5102	GO:0019219	<1e-4	sll0780	GO:0042451	<1e-4	slr1342	GO:0016746	<1e-4	sll8033	GO:0005575	<1e-4	sll1272	GO:0005975	0.0212
sll0661	GO:0016052	<1e-4	ssr0657	GO:0050801	<1e-4	slr1865	GO:0016817	<1e-4	slr0821	GO:0044425	<1e-4	ssr3154	GO:0005975	0.0212
slr1999	GO:0034654	<1e-4	sll1942	GO:0009987	<1e-4	slr1644	GO:0022832	<1e-4	sll7047	GO:0044446	<1e-4	sll0488	GO:0003723	0.0211
slr5087	GO:0046164	<1e-4	slr5018	GO:0044281	<1e-4	slr6028	GO:0015293	<1e-4	sll0911	GO:0044444	<1e-4	sll0822	GO:0016820	0.0208
sll0479	GO:0044275	<1e-4	sll1004	GO:0009124	<1e-4	sll0283	GO:0015293	<1e-4	slr1081	GO:0044422	<1e-4	slr1851	GO:0016820	0.0208
sll1926	GO:0001932	<1e-4	slr0930	GO:0008610	<1e-4	slr0812	GO:0008171	<1e-4	slr2084	GO:0044464	<1e-4	sll0183	GO:0016820	0.0208
slr1690	GO:0044248	<1e-4	slr0589	GO:0051179	<1e-4	sll1950	GO:0015405	<1e-4	sll1702	GO:0005575	<1e-4	slr0923	GO:0016820	0.0208
ssr7084	GO:0009892	<1e-4	slr1660	GO:0008150	<1e-4	slr6103	GO:0015291	<1e-4	slr6090	GO:0044425	<1e-4	ssl2733	GO:0016820	0.0208
sll2007	GO:0033013	<1e-4	slr1023	GO:0050789	<1e-4	ssr2962	GO:0016746	<1e-4	sll0048	GO:0005575	<1e-4	slr1047	GO:0016820	0.0208
slr0960	GO:0009259	<1e-4	sll1692	GO:0043412	<1e-4	ssl5096	GO:0015267	<1e-4	slr1066	GO:0044444	<1e-4	slr0978	GO:0016820	0.0208
slr2118	GO:0009892	<1e-4	sll0400	GO:0031323	<1e-4	sll1692	GO:0008171	<1e-4	slr1290	GO:0005575	<1e-4	sll0577	GO:0016820	0.0208
slr0642	GO:0006811	<1e-4	ssl0350	GO:0009262	<1e-4	slr1807	GO:0043492	<1e-4	ssr6079	GO:0044422	<1e-4	sll1526	GO:0016820	0.0208
slr0147	GO:0048878	<1e-4	ssl2733	GO:0072521	<1e-4	slr1619	GO:0015075	<1e-4	slr1260	GO:0044444	<1e-4	sll0022	GO:0016820	0.0208
sll2011	GO:0022411	<1e-4	sll1680	GO:0051818	<1e-4	ssr7093	GO:0042623	<1e-4	sll0444	GO:0044446	<1e-4	sll0175	GO:0016820	0.0208
slr1407	GO:0006631	<1e-4	slr0651	GO:0009309	<1e-4	slr0209	GO:0005342	<1e-4	sll7006	GO:0005575	<1e-4	slr1444	GO:0016820	0.0208
sll0703	GO:0046128	<1e-4	slr0680	GO:0052111	<1e-4	sll1866	GO:0022891	<1e-4	sll0263	GO:0005575	<1e-4	slr0496	GO:0016820	0.0208
ssr7093	GO:0044281	<1e-4	slr0496	GO:0051818	<1e-4	sll0293	GO:0003674	<1e-4	slr6022	GO:0005575	<1e-4	slr0589	GO:0016820	0.0208
sll1222	GO:0009126	<1e-4	sll1203	GO:0080090	<1e-4	slr1266	GO:0016462	<1e-4	sll0172	GO:0044425	<1e-4	sll0361	GO:0016820	0.0208
slr1258	GO:0006220	<1e-4	sll7077	GO:0009117	<1e-4	slr0789	GO:0016818	<1e-4	slr0709	GO:0044446	<1e-4	slr1431	GO:0004871	0.0206
slr1097	GO:0052188	<1e-4	sll1880	GO:0051234	<1e-4	slr1699	GO:0016462	<1e-4	ssr1698	GO:0044464	<1e-4	slr1493	GO:0006259	0.02
slr0480	GO:0006811	<1e-4	slr1789	GO:0052188	<1e-4	slr1807	GO:0016741	<1e-4	sll1068	GO:0044425	<1e-4	slr1209	GO:0006259	0.02
slr1150	GO:0009987	<1e-4	slr0038	GO:0009142	<1e-4	slr1752	GO:0015293	<1e-4	slr0816	GO:0005575	<1e-4	sll1938	GO:0006259	0.02
slr1266	GO:0046164	<1e-4	ssr6019	GO:0050789	<1e-4	slr1590	GO:0043167	<1e-4	ssl5008	GO:0044424	<1e-4	sll0225	GO:0006259	0.02
sll0192	GO:0008150	<1e-4	ssl5099	GO:0042455	<1e-4	sll6055	GO:0022890	<1e-4	slr1342	GO:0044422	<1e-4	slr1690	GO:0006259	0.02
sll1702	GO:0031326	<1e-4	sll0909	GO:0009394	<1e-4	sll1109	GO:0005342	<1e-4	sll0781	GO:0005575	<1e-4	sll1350	GO:0006259	0.02
slr0287	GO:0009262	<1e-4	sll0732	GO:0009260	<1e-4	sll1233	GO:0022857	<1e-4	ssl3692	GO:0043226	<1e-4	sll0572	GO:0006259	0.02
ssl1378	GO:0019751	<1e-4	slr1917	GO:0050896	<1e-4	ssr7017	GO:0015293	<1e-4	ssl2717	GO:0044444	<1e-4	sll0737	GO:0006259	0.02
slr0479	GO:0006720	<1e-4	sll1130	GO:0046394	<1e-4	slr1222	GO:0003674	<1e-4	ssr1114	GO:0044425	<1e-4	slr1383	GO:0006259	0.02
sll0678	GO:0016052	<1e-4	slr0325	GO:0006163	<1e-4	sll1155	GO:0016817	<1e-4	ssl1378	GO:0005575	<1e-4	sll0827	GO:0006259	0.02
ssl0294	GO:0051234	<1e-4	sll8019	GO:0046394	<1e-4	slr7013	GO:0015077	<1e-4	sll0925	GO:0044422	<1e-4	sll0981	GO:0006259	0.02
sll0933	GO:0009144	<1e-4	slr0291	GO:0034654	<1e-4	sll0985	GO:0016788	<1e-4	slr1644	GO:0044422	<1e-4	sll1634	GO:0006259	0.02

sll1135	GO:0034660	<1e-4	slr6006	GO:0001932	<1e-4	sll0984	GO:0016818	<1e-4	sll0743	GO:0044425	<1e-4	sll1399	GO:0006259	0.02
sll0691	GO:0010468	<1e-4	sll1006	GO:0009889	<1e-4	ssr2781	GO:0022838	<1e-4	sll1769	GO:0044422	<1e-4	sml0011	GO:0006259	0.02
sll7030	GO:0065007	<1e-4	sll0822	GO:0016054	<1e-4	slr1444	GO:0015291	<1e-4	ssr7084	GO:0044424	<1e-4	slr1762	GO:0006259	0.02
sll0861	GO:0072522	<1e-4	sll0788	GO:0016054	<1e-4	sll0249	GO:0042623	<1e-4	sll0584	GO:0005575	<1e-4	slr1303	GO:0006259	0.02
sll0481	GO:0044106	<1e-4	sll1527	GO:0055082	<1e-4	slr6101	GO:0022836	<1e-4	slr0517	GO:0044464	<1e-4	slr7024	GO:0006259	0.02
ssl2781	GO:0051171	<1e-4	slr1813	GO:0048878	<1e-4	ssr7035	GO:0015267	<1e-4	slr1474	GO:0044464	<1e-4	slr0815	GO:0006259	0.02
slr0157	GO:0065007	<1e-4	slr0325	GO:0009132	<1e-4	slr0907	GO:0022891	<1e-4	slr1593	GO:0044446	<1e-4	slr1071	GO:0006259	0.02
slr1222	GO:0009132	<1e-4	ssr1558	GO:0006721	<1e-4	sll1634	GO:0016741	<1e-4	sll0853	GO:0044464	<1e-4	ssl0483	GO:0006259	0.02
sll1359	GO:0072522	<1e-4	slr7092	GO:0048878	<1e-4	sll0335	GO:0022832	<1e-4	sll0310	GO:0044444	<1e-4	sll0268	GO:0006259	0.02
slr1854	GO:0009165	<1e-4	ssl1498	GO:0009165	<1e-4	sll0448	GO:0022832	<1e-4	slr0590	GO:0044446	<1e-4	sll0156	GO:0006259	0.02
sll1062	GO:0051246	<1e-4	sll1252	GO:0006721	<1e-4	ssr6024	GO:0016462	<1e-4	slr1628	GO:0044422	<1e-4	sll0669	GO:0006259	0.02
sll5128	GO:0043549	<1e-4	slr1557	GO:0005996	<1e-4	slr1613	GO:0022891	<1e-4	slr0348	GO:0044422	<1e-4	slr1603	GO:0016772	0.0192
slr1932	GO:0008150	<1e-4	sll1965	GO:0033013	<1e-4	ssr2611	GO:0043167	<1e-4	slr7098	GO:0044464	<1e-4	sll1737	GO:0016772	0.0192
sll0149	GO:0006733	<1e-4	sll0532	GO:0006721	<1e-4	slr1066	GO:0016818	<1e-4	ssr5117	GO:0044424	<1e-4	slr5112	GO:0016772	0.019
slr6066	GO:0052111	<1e-4	ssr5074	GO:0051818	<1e-4	sll0584	GO:0003674	<1e-4	slr1188	GO:0044425	<1e-4	ssl5108	GO:0016772	0.019
slr1699	GO:0044260	<1e-4	sll1611	GO:0051188	<1e-4	sll0325	GO:0016741	<1e-4	slr0334	GO:0044422	<1e-4	sll1388	GO:0016772	0.019
sll1737	GO:0009117	<1e-4	slr7015	GO:0072524	<1e-4	slr1704	GO:0016817	<1e-4	sll1222	GO:0043226	<1e-4	ssr6099	GO:0016772	0.019
sll1717	GO:0052188	<1e-4	sll1388	GO:0031326	<1e-4	sll1217	GO:0016462	<1e-4	slr0871	GO:0044422	<1e-4	sll0372	GO:0016772	0.019
sll0410	GO:0050789	<1e-4	slr0712	GO:0006811	<1e-4	slr8022	GO:0022891	<1e-4	slr1179	GO:0044444	<1e-4	slr0334	GO:0016772	0.019
sll0602	GO:0009199	<1e-4	sll6052	GO:0043170	<1e-4	slr6009	GO:0015293	<1e-4	slr6047	GO:0044464	<1e-4	sll5132	GO:0016772	0.019
slr1614	GO:0043933	<1e-4	slr1864	GO:0019751	<1e-4	slr1303	GO:0016818	<1e-4	sll1372	GO:0005575	<1e-4	slr1990	GO:0016772	0.019
ssl2384	GO:0006220	<1e-4	slr7060	GO:0009262	<1e-4	sll0532	GO:0016818	<1e-4	sll1643	GO:0044425	<1e-4	slr0816	GO:0016772	0.019
ssr2972	GO:0006163	<1e-4	ssl2471	GO:0006733	<1e-4	slr1069	GO:0016746	<1e-4	slr1847	GO:0044446	<1e-4	ssl7021	GO:0016772	0.019
ssr1114	GO:0009124	<1e-4	sll0518	GO:0045184	<1e-4	sll0010	GO:0022836	<1e-4	ssr3467	GO:0044446	<1e-4	sll1915	GO:0016772	0.019
slr0975	GO:0045184	<1e-4	slr0554	GO:0009893	<1e-4	ssl5065	GO:0005342	<1e-4	sll0360	GO:0005575	<1e-4	ssl7042	GO:0016772	0.019
sll0925	GO:0019637	<1e-4	slr1394	GO:0009124	<1e-4	sll0487	GO:0015077	<1e-4	slr1429	GO:0043226	<1e-4	ssl6061	GO:0016772	0.019
slr6106	GO:0071841	<1e-4	slr1863	GO:0016053	<1e-4	slr1207	GO:0008171	<1e-4	sll7090	GO:0044422	<1e-4	sll5063	GO:0016772	0.019
sll0858	GO:0044275	<1e-4	slr7013	GO:0042180	<1e-4	sll1166	GO:0043492	<1e-4	sll0793	GO:0044425	<1e-4	sll8035	GO:0016772	0.019
sll0208	GO:0009259	<1e-4	sll0225	GO:0008150	<1e-4	slr2111	GO:0015291	<1e-4	slr1474	GO:0043226	<1e-4	slr0271	GO:0016772	0.019
slr6005	GO:0010468	<1e-4	slr0157	GO:0006793	<1e-4	slr1365	GO:0022836	<1e-4	sll0497	GO:0043226	<1e-4	sll0552	GO:0016772	0.019
sll0188	GO:0031323	<1e-4	sll1218	GO:0044281	<1e-4	sll0498	GO:0003674	<1e-4	sll1040	GO:0044424	<1e-4	ssl6092	GO:0016772	0.019
ssl8005	GO:0042180	<1e-4	sll1289	GO:0009161	<1e-4	slr2092	GO:0015077	<1e-4	ssl7046	GO:0044422	<1e-4	slr7060	GO:0016772	0.019
slr0651	GO:0031323	<1e-4	slr0243	GO:0006082	<1e-4	sll0218	GO:0015075	<1e-4	slr1442	GO:0044444	<1e-4	sll1068	GO:0016772	0.019
slr1951	GO:0009987	<1e-4	sll8011	GO:0060255	<1e-4	slr6051	GO:0022838	<1e-4	slr0341	GO:0044425	<1e-4	ssr6086	GO:0016772	0.019
slr1194	GO:0042451	<1e-4	ssr2551	GO:0009141	<1e-4	slr2073	GO:0016818	<1e-4	ssl1552	GO:0044422	<1e-4	slr1290	GO:0016772	0.019
slr1222	GO:0072522	<1e-4	sll0168	GO:0009056	<1e-4	ssl2384	GO:0022891	<1e-4	slr1384	GO:0044464	<1e-4	sll0162	GO:0016772	0.019
ssr2962	GO:0042451	<1e-4	slr1117	GO:0072524	<1e-4	ssr2611	GO:0016818	<1e-4	ssr3572	GO:0044422	<1e-4	sll1714	GO:0016772	0.019
slr1260	GO:0071840	<1e-4	sll0839	GO:0009144	<1e-4	ssl0832	GO:0022803	<1e-4	slr6101	GO:0005575	<1e-4	slr0082	GO:0016772	0.019
slr0398	GO:0051188	<1e-4	sll7028	GO:0051186	<1e-4	slr1913	GO:0043492	<1e-4	sll1132	GO:0044444	<1e-4	slr0645	GO:0016772	0.019
slr2049	GO:0009057	<1e-4	sll0350	GO:0072524	<1e-4	sll1681	GO:0022832	<1e-4	sll0860	GO:0044424	<1e-4	ssr6024	GO:0016772	0.019
sll0216	GO:0031326	<1e-4	sll1730	GO:0019751	<1e-4	slr1196	GO:0015405	<1e-4	slr0345	GO:0043226	<1e-4	slr6081	GO:0016772	0.019
slr6067	GO:0016054	<1e-4	ssr6086	GO:0072521	<1e-4	sll1761	GO:0042623	<1e-4	slr1033	GO:0044422	<1e-4	ssl7048	GO:0016772	0.019
sll7064	GO:0046128	<1e-4	ssl0787	GO:0009394	<1e-4	sll1766	GO:0015291	<1e-4	sll1318	GO:0044424	<1e-4	ssr6062	GO:0016772	0.019
sll0071	GO:0044283	<1e-4	sll0641	GO:0005976	<1e-4	sll1764	GO:0022836	<1e-4	slr2037	GO:0044424	<1e-4	ssl8028	GO:0016772	0.019
slr0157	GO:0033013	<1e-4	ssr2554	GO:0048519	<1e-4	ssr3570	GO:0043167	<1e-4	sll1389	GO:0043226	<1e-4	slr5111	GO:0016772	0.019
ssl1923	GO:0001932	<1e-4	slr1577	GO:0006163	<1e-4	sll5003	GO:0022892	<1e-4	slr0771	GO:0005575	<1e-4	sll0044	GO:0016772	0.019
sll0328	GO:0019362	<1e-4	sll0802	GO:0034660	<1e-4	slr2084	GO:0022836	<1e-4	sll0314	GO:0044446	<1e-4	slr7102	GO:0016772	0.019
slr7059	GO:0043412	<1e-4	sll0410	GO:0072524	<1e-4	sll7062	GO:0043167	<1e-4	ssl5108	GO:0044444	<1e-4	sll1531	GO:0016772	0.019
slr0431	GO:0009161	<1e-4	sll0096	GO:0044281	<1e-4	slr1854	GO:0016818	<1e-4	sll0354	GO:0044424	<1e-4	sll0098	GO:0016772	0.019
sll0676	GO:0042451	<1e-4	sll7066	GO:0006163	<1e-4	sll1254	GO:0016746	<1e-4	sll5004	GO:0044424	<1e-4	ssr7079	GO:0016772	0.019
slr0924	GO:0034654	<1e-4	sll1659	GO:0044255	<1e-4	slr1495	GO:0022838	<1e-4	sll1671	GO:0043226	<1e-4	sll5089	GO:0016772	0.019
sll0147	GO:0043412	<1e-4	ssl0738	GO:0090304	<1e-4	sll0062	GO:0005342	<1e-4	sll1021	GO:0044464	<1e-4	slr1789	GO:0016772	0.019
ssr1552	GO:0009132	<1e-4	slr0386	GO:0044281	<1e-4	slr1788	GO:0015399	<1e-4	sll0267	GO:0044424	<1e-4	slr0181	GO:0016772	0.019
ssl1577	GO:0051171	<1e-4	ssr6026	GO:0044271	<1e-4	slr6087	GO:0003674	<1e-4	ssl2471	GO:0044464	<1e-4	slr7094	GO:0016772	0.019

slr6031	GO:0006091	<1e-4	ssr8013	GO:0009991	<1e-4	slr0634	GO:0022891	<1e-4	sll0280	GO:0044425	<1e-4	slr0637	GO:0016772	0.019
slr0337	GO:0043412	<1e-4	slr1095	GO:0009124	<1e-4	slr0590	GO:0005342	<1e-4	sll1835	GO:0044464	<1e-4	sll1681	GO:0016772	0.019
sll1350	GO:0072528	<1e-4	sll0860	GO:0042180	<1e-4	ssr6026	GO:0022890	<1e-4	slr1875	GO:0005575	<1e-4	slr0509	GO:0016772	0.019
sll0047	GO:0009126	<1e-4	slr1462	GO:0019362	<1e-4	slr0503	GO:0015291	<1e-4	sll0606	GO:0044446	<1e-4	ssr6030	GO:0016772	0.019
slr1815	GO:0010556	<1e-4	slr0184	GO:0009308	<1e-4	sll5097	GO:0003674	<1e-4	slr0948	GO:0043226	<1e-4	sll1891	GO:0016772	0.019
sll0780	GO:0044283	<1e-4	slr1052	GO:0009987	<1e-4	slr1170	GO:0015399	<1e-4	slr0179	GO:0005575	<1e-4	sll1164	GO:0016772	0.019
slr0147	GO:0080090	<1e-4	sll1698	GO:0050801	<1e-4	sll0394	GO:0022857	<1e-4	slr1438	GO:0044444	<1e-4	sll1486	GO:0016772	0.019
sll0735	GO:0051188	<1e-4	slr1816	GO:0031323	<1e-4	sll1898	GO:0016741	<1e-4	ssl8039	GO:0044444	<1e-4	ssr2975	GO:0016772	0.019
ssr3572	GO:0051716	<1e-4	sll2015	GO:0006082	<1e-4	sll1155	GO:0043492	<1e-4	slr1261	GO:0044444	<1e-4	slr0498	GO:0016772	0.019
sll0007	GO:0008150	<1e-4	sll0732	GO:0046128	<1e-4	sll1092	GO:0016746	<1e-4	ssr2049	GO:0043226	<1e-4	slr0613	GO:0016772	0.019
ssr0761	GO:0048518	<1e-4	sll0543	GO:0052188	<1e-4	slr0362	GO:0022892	<1e-4	slr1425	GO:0044444	<1e-4	ssr7084	GO:0016772	0.019
slr0863	GO:0051179	<1e-4	slr0865	GO:0009165	<1e-4	sll1797	GO:0003674	<1e-4	sll0350	GO:0005575	<1e-4	ssr0336	GO:0016772	0.019
slr0358	GO:0046483	<1e-4	slr0300	GO:0034654	<1e-4	ssl2138	GO:0015399	<1e-4	sll0997	GO:0044424	<1e-4	sll0479	GO:0016772	0.019
slr1301	GO:0009893	<1e-4	ssr8047	GO:0006720	<1e-4	slr6049	GO:0022803	<1e-4	slr6005	GO:0043226	<1e-4	slr8022	GO:0016772	0.019
slr0876	GO:0060255	<1e-4	slr0645	GO:0009123	<1e-4	slr0468	GO:0003674	<1e-4	sll2015	GO:0044424	<1e-4	slr0634	GO:0016772	0.019
slr5018	GO:0019751	<1e-4	sll0413	GO:0006091	<1e-4	sll1265	GO:0015291	<1e-4	sll1355	GO:0044422	<1e-4	sll1832	GO:0016772	0.019
slr1915	GO:0065007	<1e-4	slr0204	GO:0009126	<1e-4	slr0196	GO:0043167	<1e-4	slr0870	GO:0005575	<1e-4	ssr0109	GO:0016772	0.019
slr0667	GO:0045184	<1e-4	slr1906	GO:0051716	<1e-4	sll1188	GO:0016462	<1e-4	sll5047	GO:0043226	<1e-4	slr1935	GO:0016772	0.019
sll1267	GO:0006066	<1e-4	sll1272	GO:0044281	<1e-4	ssl0832	GO:0003674	<1e-4	sll1307	GO:0044444	<1e-4	slr6016	GO:0016772	0.019
ssr5020	GO:0071554	<1e-4	slr1636	GO:0006220	<1e-4	ssr2047	GO:0016818	<1e-4	slr0722	GO:0044422	<1e-4	slr0291	GO:0016772	0.019
sll0488	GO:0071840	<1e-4	ssl1923	GO:0009150	<1e-4	ssl1255	GO:0022857	<1e-4	sll0445	GO:0044446	<1e-4	ssl3573	GO:0016772	0.019
slr5111	GO:0072528	<1e-4	ssl7007	GO:0052111	<1e-4	slr6044	GO:0015075	<1e-4	ssl3383	GO:0043226	<1e-4	slr7071	GO:0016772	0.019
sll0545	GO:0019362	<1e-4	ssl5114	GO:0006220	<1e-4	slr1789	GO:0015293	<1e-4	slr0468	GO:0044464	<1e-4	slr5013	GO:0016772	0.019
sll1862	GO:0044106	<1e-4	slr0273	GO:0009893	<1e-4	sll1898	GO:0015291	<1e-4	slr1885	GO:0044446	<1e-4	slr0728	GO:0016772	0.019
slr0865	GO:0071841	<1e-4	sll1442	GO:0019222	<1e-4	sll1979	GO:0022891	<1e-4	slr1926	GO:0043226	<1e-4	slr1915	GO:0016772	0.019
slr1425	GO:0010468	<1e-4	slr0575	GO:0042451	<1e-4	slr1827	GO:0022803	<1e-4	ssl7074	GO:0044422	<1e-4	slr2117	GO:0016772	0.019
sll0811	GO:0090304	<1e-4	slr0149	GO:0043549	<1e-4	sll5003	GO:0016746	<1e-4	slr6063	GO:0044464	<1e-4	slr0521	GO:0016772	0.019
ssl2064	GO:0051716	<1e-4	sll5030	GO:0046907	<1e-4	sll0218	GO:0043167	<1e-4	slr1101	GO:0043226	<1e-4	ssl7022	GO:0016772	0.019
sll1925	GO:0009124	<1e-4	slr0890	GO:0051188	<1e-4	sll1285	GO:0043167	<1e-4	sll1251	GO:0043226	<1e-4	sll0442	GO:0016772	0.019
ssr3410	GO:0042455	<1e-4	slr1865	GO:0051186	<1e-4	sll1652	GO:0016817	<1e-4	sll0268	GO:0044444	<1e-4	slr0813	GO:0016772	0.019
sll0525	GO:0019637	<1e-4	sll0645	GO:0071840	<1e-4	slr1444	GO:0043492	<1e-4	sll0369	GO:0044444	<1e-4	slr0610	GO:0016772	0.019
ssr6048	GO:0008610	<1e-4	sll0867	GO:0050794	<1e-4	slr1852	GO:0016741	<1e-4	slr1047	GO:0044424	<1e-4	ssl6035	GO:0016772	0.019
sll1954	GO:0051186	<1e-4	slr0729	GO:0055086	<1e-4	slr6080	GO:0003674	<1e-4	slr7057	GO:0044425	<1e-4	sll1583	GO:0016772	0.019
slr1624	GO:0009144	<1e-4	sll0188	GO:0006720	<1e-4	sll1510	GO:0016818	<1e-4	ssr5020	GO:0044424	<1e-4	sll0327	GO:0016772	0.019
sll8002	GO:0052111	<1e-4	sll0732	GO:0031640	<1e-4	ssr2009	GO:0022836	<1e-4	slr1616	GO:0005575	<1e-4	slr2003	GO:0016772	0.019
sll0241	GO:0019222	<1e-4	slr0962	GO:0052111	<1e-4	ssr7035	GO:0022891	<1e-4	slr1365	GO:0005575	<1e-4	sll8001	GO:0016772	0.019
sll8032	GO:0045184	<1e-4	slr0345	GO:0019222	<1e-4	sll0189	GO:0016817	<1e-4	slr1378	GO:0044425	<1e-4	slr1173	GO:0016772	0.019
sll0505	GO:0034654	<1e-4	slr6075	GO:0044275	<1e-4	sll0445	GO:0022891	<1e-4	sll0810	GO:0044446	<1e-4	sll2011	GO:0016772	0.019
sll1106	GO:0050896	<1e-4	sll1763	GO:0009117	<1e-4	sll0096	GO:0022832	<1e-4	slr0698	GO:0044444	<1e-4	slr1513	GO:0016772	0.019
slr1907	GO:0006576	<1e-4	slr0108	GO:0071496	<1e-4	sll1858	GO:0022892	<1e-4	sll0886	GO:0044424	<1e-4	slr5077	GO:0016772	0.019
ssr2754	GO:0008610	<1e-4	slr5087	GO:0051347	<1e-4	sll0414	GO:0060089	<1e-4	slr0907	GO:0044444	<1e-4	slr6106	GO:0016772	0.019
slr1196	GO:0034654	<1e-4	sll0751	GO:0009987	<1e-4	sll1232	GO:0015267	<1e-4	slr1207	GO:0043226	<1e-4	slr6088	GO:0016772	0.019
slr0326	GO:0006631	<1e-4	ssr6079	GO:0009123	<1e-4	ssl0738	GO:0042623	<1e-4	slr6063	GO:0005575	<1e-4	ssl2595	GO:0016772	0.019
ssr1698	GO:0051234	<1e-4	sll0361	GO:0009124	<1e-4	sll0177	GO:0022832	<1e-4	sll0863	GO:0043226	<1e-4	ssr7017	GO:0016772	0.019
sll0847	GO:0044003	<1e-4	slr0769	GO:0046128	<1e-4	sll0321	GO:0015075	<1e-4	ssl0352	GO:0044424	<1e-4	ssr0761	GO:0016772	0.019
slr0976	GO:0019752	<1e-4	slr2018	GO:0006220	<1e-4	sll0230	GO:0008171	<1e-4	sll0549	GO:0005575	<1e-4	slr5037	GO:0016772	0.019
slr1152	GO:0048522	<1e-4	sll0423	GO:0009124	<1e-4	slr0103	GO:0015075	<1e-4	slr0634	GO:0044464	<1e-4	sll0625	GO:0016772	0.019
sll1218	GO:0009987	<1e-4	sll1950	GO:0006631	<1e-4	ssr0759	GO:0015405	<1e-4	sll1995	GO:0043226	<1e-4	ssl7007	GO:0016772	0.019
slr1073	GO:0072524	<1e-4	sll0996	GO:0006753	<1e-4	slr2118	GO:0022857	<1e-4	ssl5096	GO:0044422	<1e-4	ssl2971	GO:0016772	0.019
slr1493	GO:0072528	<1e-4	slr1163	GO:0019222	<1e-4	sll0445	GO:0005342	<1e-4	sll5090	GO:0044444	<1e-4	slr0300	GO:0016772	0.019
ssr5120	GO:0071554	<1e-4	ssr3467	GO:0006732	<1e-4	sll1273	GO:0022892	<1e-4	ssl1378	GO:0044422	<1e-4	slr0575	GO:0016772	0.019
sll1965	GO:0009150	<1e-4	slr2070	GO:0006163	<1e-4	sll0837	GO:0016817	<1e-4	sll0068	GO:0043234	<1e-4	slr5102	GO:0016772	0.019
slr0921	GO:0009141	<1e-4	slr7016	GO:0031640	<1e-4	sll0436	GO:0042623	<1e-4	sll1390	GO:0044464	<1e-4	ssl5114	GO:0016772	0.019
ssl7038	GO:0071496	<1e-4	slr0948	GO:0019219	<1e-4	sll1654	GO:0003674	<1e-4	sll1563	GO:0044446	<1e-4	slr6044	GO:0016772	0.019

sll0932	GO:0008610	<1e-4	slr1917	GO:0006811	<1e-4	ssr2067	GO:0015291	<1e-4	slr7082	GO:0043226	<1e-4	sll1285	GO:0016772	0.019
sll1583	GO:0051234	<1e-4	slr0815	GO:0006811	<1e-4	slr2049	GO:0016818	<1e-4	sll1477	GO:0044425	<1e-4	ssr2553	GO:0016772	0.019
sll1250	GO:0044260	<1e-4	sll0286	GO:0019751	<1e-4	slr0852	GO:0043167	<1e-4	slr1034	GO:0044424	<1e-4	sll1940	GO:0016772	0.019
sll0539	GO:0052188	<1e-4	sll0584	GO:0009991	<1e-4	slr1721	GO:0022890	<1e-4	slr0606	GO:0043226	<1e-4	slr0238	GO:0016772	0.019
sll0496	GO:0050801	<1e-4	ssr2972	GO:0018130	<1e-4	sll0499	GO:0015075	<1e-4	sll0995	GO:0044446	<1e-4	sll0310	GO:0044237	0.0188
slr1667	GO:0006766	<1e-4	slr5037	GO:0019751	<1e-4	slr1530	GO:0016817	<1e-4	slr0476	GO:0005575	<1e-4	ssl3291	GO:0044237	0.0188
slr0723	GO:0005996	<1e-4	sll1735	GO:0008610	<1e-4	slr6029	GO:0022832	<1e-4	sll5004	GO:0005575	<1e-4	sll0286	GO:0044237	0.0188
slr1062	GO:0042180	<1e-4	slr1215	GO:0031640	<1e-4	sll0181	GO:0008171	<1e-4	sll0414	GO:0044464	<1e-4	slr0408	GO:0044237	0.0188
slr5024	GO:0072524	<1e-4	slr0195	GO:0006066	<1e-4	slr0789	GO:0022838	<1e-4	sll0482	GO:0044464	<1e-4	ssr1258	GO:0044237	0.0188
sll0609	GO:0009259	<1e-4	slr1053	GO:0052188	<1e-4	slr0816	GO:0043492	<1e-4	slr0199	GO:0044464	<1e-4	slr0848	GO:0044237	0.0188
slr1566	GO:0009262	<1e-4	sll0630	GO:0071554	<1e-4	sll1884	GO:0042623	<1e-4	sll0310	GO:0044424	<1e-4	slr0919	GO:0044237	0.0188
sll1571	GO:0031640	<1e-4	slr0398	GO:0048522	<1e-4	sll6052	GO:0015267	<1e-4	sll0615	GO:0043226	<1e-4	slr2084	GO:0044237	0.0188
sll7090	GO:0022607	<1e-4	slr0594	GO:0009132	<1e-4	slr0913	GO:0016462	<1e-4	sll7077	GO:0005575	<1e-4	sll0269	GO:0044237	0.0188
sll1714	GO:0006082	<1e-4	sll0085	GO:0042180	<1e-4	ssl0350	GO:0022838	<1e-4	ssl0312	GO:0044422	<1e-4	sll1613	GO:0008233	0.0187
sll0447	GO:0065007	<1e-4	slr7023	GO:0072524	<1e-4	slr1677	GO:0022838	<1e-4	slr1601	GO:0044422	<1e-4	slr0586	GO:0008233	0.0187
slr1875	GO:0009262	<1e-4	ssr3129	GO:0009126	<1e-4	slr2004	GO:0022857	<1e-4	sll1160	GO:0005575	<1e-4	slr1081	GO:0008233	0.0187
sll0424	GO:0009132	<1e-4	sll0513	GO:0009141	<1e-4	sll0423	GO:0008171	<1e-4	slr0108	GO:0005575	<1e-4	sll0280	GO:0008233	0.0187
sll1217	GO:0009893	<1e-4	slr1964	GO:0010468	<1e-4	sll1541	GO:0008171	<1e-4	sll1884	GO:0044446	<1e-4	sll1262	GO:0008233	0.0187
sll1749	GO:0055114	<1e-4	slr1907	GO:0009142	<1e-4	slr0545	GO:0015405	<1e-4	ssl5045	GO:0005575	<1e-4	slr1778	GO:0008233	0.0187
slr0552	GO:0009987	<1e-4	slr0304	GO:0006793	<1e-4	slr1811	GO:0022836	<1e-4	ssr0336	GO:0044422	<1e-4	slr0392	GO:0008233	0.0187
slr6012	GO:0006766	<1e-4	slr1177	GO:0008150	<1e-4	ssl1263	GO:0022838	<1e-4	sll0785	GO:0044422	<1e-4	sll0198	GO:0008233	0.0187
sll0910	GO:0009991	<1e-4	sll7069	GO:0034660	<1e-4	sll0532	GO:0022838	<1e-4	slr1855	GO:0005575	<1e-4	sll0314	GO:0008233	0.0187
slr0930	GO:0072527	<1e-4	slr0601	GO:0071842	<1e-4	sll0743	GO:0022891	<1e-4	slr0294	GO:0043226	<1e-4	slr2121	GO:0008233	0.0187
slr5119	GO:0048523	<1e-4	slr1809	GO:0042455	<1e-4	slr1568	GO:0015075	<1e-4	sll0428	GO:0044422	<1e-4	slr1262	GO:0008233	0.0187
slr0668	GO:0006066	<1e-4	sll0253	GO:0001932	<1e-4	ssr2142	GO:0022836	<1e-4	sll1638	GO:0044425	<1e-4	ssl2807	GO:0008233	0.0187
sll0406	GO:0048522	<1e-4	sll0400	GO:0009117	<1e-4	sll1424	GO:0015405	<1e-4	slr1396	GO:0044422	<1e-4	ssr2787	GO:0008233	0.0187
slr0376	GO:0001932	<1e-4	slr1886	GO:0051188	<1e-4	slr0112	GO:0060089	<1e-4	sll0691	GO:0044424	<1e-4	slr1084	GO:0008233	0.0187
ssr2142	GO:0034641	<1e-4	slr1886	GO:0009124	<1e-4	slr1507	GO:0008171	<1e-4	sll0930	GO:0044444	<1e-4	slr0845	GO:0008233	0.0187
ssl2384	GO:0006811	<1e-4	sll1902	GO:0009141	<1e-4	slr1186	GO:0022892	<1e-4	sll1671	GO:0044425	<1e-4	slr0144	GO:0008233	0.0187
ssr2009	GO:0033013	<1e-4	sll0588	GO:0019752	<1e-4	slr6065	GO:0003674	<1e-4	slr1098	GO:0044446	<1e-4	sll0524	GO:0008233	0.0187
slr0416	GO:0055114	<1e-4	sll1913	GO:0044248	<1e-4	sll0176	GO:0015405	<1e-4	slr6106	GO:0005575	<1e-4	slr0103	GO:0008233	0.0187
sll1757	GO:0009308	<1e-4	sll0160	GO:0060255	<1e-4	sll1949	GO:0015293	<1e-4	slr6071	GO:0044444	<1e-4	sll0645	GO:0008233	0.0187
slr1628	GO:0006811	<1e-4	ssr0761	GO:0046128	<1e-4	sll0167	GO:0016818	<1e-4	slr7026	GO:0043226	<1e-4	slr1667	GO:0008233	0.0187
sll1002	GO:0009056	<1e-4	ssl0312	GO:0006766	<1e-4	slr1074	GO:0016818	<1e-4	ssl7022	GO:0044446	<1e-4	ssl0242	GO:0008233	0.0187
slr1648	GO:0006753	<1e-4	slr1958	GO:0072521	<1e-4	ssl7007	GO:0015077	<1e-4	sll1166	GO:0005575	<1e-4	slr1530	GO:0008233	0.0187
sll1160	GO:0019219	<1e-4	sll0785	GO:0051716	<1e-4	slr1852	GO:0015405	<1e-4	ssl3382	GO:0005575	<1e-4	sll1632	GO:0008233	0.0187
sll0659	GO:0034660	<1e-4	slr1940	GO:0048518	<1e-4	sll0265	GO:0015291	<1e-4	sll1634	GO:0044444	<1e-4	sll0243	GO:0008233	0.0187
ssl3291	GO:0042455	<1e-4	slr0325	GO:0044260	<1e-4	slr0291	GO:0015405	<1e-4	slr0468	GO:0005575	<1e-4	ssr2615	GO:0008233	0.0187
sll1761	GO:0009132	<1e-4	slr0848	GO:0044106	<1e-4	slr7012	GO:0016817	<1e-4	sll1089	GO:0044464	<1e-4	sll1656	GO:0008233	0.0187
sll0361	GO:0048522	<1e-4	sll0827	GO:0034641	<1e-4	slr1790	GO:0060089	<1e-4	sll2007	GO:0044444	<1e-4	slr1964	GO:0008233	0.0187
ssl1300	GO:0065007	<1e-4	ssr0332	GO:0051171	<1e-4	sll1095	GO:0022803	<1e-4	sll1006	GO:0044446	<1e-4	sll0944	GO:0008233	0.0187
sll0481	GO:0072527	<1e-4	slr2144	GO:0051171	<1e-4	slr1864	GO:0015293	<1e-4	slr0287	GO:0044424	<1e-4	ssl2065	GO:0008233	0.0187
sll1659	GO:0034641	<1e-4	sll0513	GO:0009124	<1e-4	sll0984	GO:0022836	<1e-4	ssr3154	GO:0005575	<1e-4	slr1676	GO:0008233	0.0187
sll7067	GO:0065007	<1e-4	slr2105	GO:0034660	<1e-4	sll0749	GO:0022832	<1e-4	slr6008	GO:0005575	<1e-4	sll0785	GO:0008233	0.0187
sll0243	GO:0051818	<1e-4	sll0925	GO:0009057	<1e-4	sll1528	GO:0015293	<1e-4	slr0552	GO:0044422	<1e-4	sll1949	GO:0008233	0.0187
sgl0002	GO:0051186	<1e-4	sll1315	GO:0044275	<1e-4	slr0975	GO:0042623	<1e-4	sll5047	GO:0005575	<1e-4	sll0216	GO:0008233	0.0187
slr1074	GO:0009260	<1e-4	slr0596	GO:0019362	<1e-4	slr0579	GO:0015075	<1e-4	slr0650	GO:0044424	<1e-4	slr0348	GO:0008233	0.0187
ssr2317	GO:0042180	<1e-4	ssr2975	GO:0009117	<1e-4	slr5023	GO:0016817	<1e-4	sll0985	GO:0044425	<1e-4	sll0023	GO:0008233	0.0187
sll1307	GO:0044260	<1e-4	sll7034	GO:0009987	<1e-4	slr0725	GO:0015291	<1e-4	slr6021	GO:0044464	<1e-4	sll1835	GO:0008233	0.0187
sll5004	GO:0009126	<1e-4	sll7090	GO:0044260	<1e-4	slr0442	GO:0015405	<1e-4	slr1462	GO:0044446	<1e-4	slr1932	GO:0008233	0.0187
slr0147	GO:0009126	<1e-4	ssr1528	GO:0048522	<1e-4	slr1990	GO:0022857	<1e-4	sll1321	GO:0044446	<1e-4	sll1608	GO:0008233	0.0187
sll5132	GO:0009117	<1e-4	sll0804	GO:0006753	<1e-4	ssl1498	GO:0022832	<1e-4	ssr5019	GO:0044464	<1e-4	sll1830	GO:0008233	0.0187
sll1025	GO:0010556	<1e-4	sll0765	GO:0043412	<1e-4	sll1715	GO:0022803	<1e-4	ssl0738	GO:0044464	<1e-4	sll1446	GO:0016740	0.0186
sll0249	GO:0006631	<1e-4	sll0563	GO:0008610	<1e-4	sll1381	GO:0022838	<1e-4	slr1103	GO:0044446	<1e-4	sll0656	GO:0016740	0.0186

ssr0109	GO:0072527	<1e-4	slr0959	GO:0055114	<1e-4	sll0602	GO:0022803	<1e-4	slr0031	GO:0044425	<1e-4	ssr0692	GO:0016740	0.0186
slr0142	GO:0044003	<1e-4	ssr0336	GO:0009892	<1e-4	slr1209	GO:0022857	<1e-4	sll1611	GO:0005575	<1e-4	sll0944	GO:0042626	0.0185
slr6063	GO:0043412	<1e-4	sll1319	GO:0044281	<1e-4	slr7096	GO:0015293	<1e-4	slr5119	GO:0044464	<1e-4	sll1442	GO:0042626	0.0185
slr0964	GO:0072527	<1e-4	ssl1378	GO:0052188	<1e-4	ssr3122	GO:0003674	<1e-4	slr0651	GO:0044422	<1e-4	slr0941	GO:0042626	0.0185
slr0250	GO:0019752	<1e-4	sll1632	GO:0060255	<1e-4	slr1376	GO:0022836	<1e-4	slr2027	GO:0044446	<1e-4	slr1210	GO:0042626	0.0185
sll1054	GO:0050801	<1e-4	ssl6061	GO:0016052	<1e-4	slr1262	GO:0015405	<1e-4	sll0744	GO:0005575	<1e-4	sll1658	GO:0042626	0.0185
slr1079	GO:0006631	<1e-4	slr7097	GO:0010468	<1e-4	slr1301	GO:0042623	<1e-4	slr0655	GO:0044446	<1e-4	ssr1765	GO:0042626	0.0185
sll1289	GO:0034641	<1e-4	slr1535	GO:0009889	<1e-4	slr1110	GO:0022803	<1e-4	ssr3154	GO:0044464	<1e-4	sll0325	GO:0042626	0.0185
slr1932	GO:0065007	<1e-4	sll1052	GO:0009259	<1e-4	sll1531	GO:0015075	<1e-4	slr1415	GO:0044446	<1e-4	sll0524	GO:0042626	0.0185
slr6051	GO:0018130	<1e-4	slr2121	GO:0042455	<1e-4	sll1053	GO:0043167	<1e-4	sll0023	GO:0005575	<1e-4	slr2121	GO:0042626	0.0185
slr2018	GO:0050801	<1e-4	ssl7046	GO:0044283	<1e-4	slr2004	GO:0005342	<1e-4	slr1484	GO:0005575	<1e-4	sll1736	GO:0042626	0.0185
sll1692	GO:0018130	<1e-4	sll0218	GO:0019752	<1e-4	sll7034	GO:0022857	<1e-4	sll0283	GO:0044424	<1e-4	sll0185	GO:0042626	0.0185
slr1071	GO:0042455	<1e-4	sll7070	GO:0006811	<1e-4	slr7101	GO:0015267	<1e-4	sll1902	GO:0044425	<1e-4	ssl2807	GO:0042626	0.0185
slr0651	GO:0080090	<1e-4	sll1726	GO:0006720	<1e-4	sll1424	GO:0016746	<1e-4	sll1429	GO:0044464	<1e-4	sll0756	GO:0042626	0.0185
slr0888	GO:0009132	<1e-4	slr0967	GO:0051171	<1e-4	sll1757	GO:0016818	<1e-4	ssl0739	GO:0005575	<1e-4	sll0780	GO:0042626	0.0185
slr0712	GO:0033013	<1e-4	ssl8005	GO:0080090	<1e-4	slr1636	GO:0016817	<1e-4	slr0112	GO:0044422	<1e-4	sll0309	GO:0042626	0.0185
sll1106	GO:0006163	<1e-4	ssr6089	GO:0010556	<1e-4	slr0317	GO:0015291	<1e-4	slr1288	GO:0044422	<1e-4	sll0023	GO:0042626	0.0185
slr0581	GO:0046164	<1e-4	slr6072	GO:0050794	<1e-4	slr1206	GO:0008171	<1e-4	sll1749	GO:0005575	<1e-4	sll1061	GO:0042626	0.0185
ssl1498	GO:0006066	<1e-4	sll0853	GO:0046483	<1e-4	sll1285	GO:0022836	<1e-4	sll0932	GO:0044425	<1e-4	slr1177	GO:0042626	0.0185
slr7097	GO:0006082	<1e-4	sll0281	GO:0019752	<1e-4	slr7081	GO:0022838	<1e-4	slr0975	GO:0043226	<1e-4	ssr2615	GO:0042626	0.0185
sll0071	GO:0006766	<1e-4	sll1583	GO:0006163	<1e-4	slr1914	GO:0022890	<1e-4	sll1949	GO:0044425	<1e-4	sll1763	GO:0005515	0.0185
sll1378	GO:0048523	<1e-4	slr6064	GO:0009126	<1e-4	sll0787	GO:0015267	<1e-4	slr1104	GO:0044464	<1e-4	sll0350	GO:0005515	0.0185
sll0181	GO:0051818	<1e-4	slr0888	GO:0031640	<1e-4	sll1773	GO:0015267	<1e-4	sll2011	GO:0043226	<1e-4	sll1656	GO:0042626	0.0185
slr0060	GO:0006721	<1e-4	sll0839	GO:0016054	<1e-4	sll0394	GO:0003674	<1e-4	ssr6020	GO:0005575	<1e-4	slr0668	GO:0042626	0.0185
sll1135	GO:0055082	<1e-4	slr1307	GO:0009126	<1e-4	sll0765	GO:0060089	<1e-4	slr1142	GO:0044444	<1e-4	slr2073	GO:0042626	0.0185
slr0702	GO:0009150	<1e-4	ssr3300	GO:0034654	<1e-4	slr0589	GO:0008171	<1e-4	sll0996	GO:0044422	<1e-4	sll0355	GO:0042626	0.0185
slr6073	GO:0009991	<1e-4	slr1340	GO:0043549	<1e-4	slr1287	GO:0008171	<1e-4	ssr0335	GO:0005575	<1e-4	sll0497	GO:0042626	0.0185
slr0065	GO:0009117	<1e-4	sll0545	GO:0052111	<1e-4	sll0157	GO:0022838	<1e-4	ssl1417	GO:0043226	<1e-4	sll0414	GO:0042626	0.0185
sll0886	GO:0006793	<1e-4	slr1384	GO:0031323	<1e-4	sll0487	GO:0022836	<1e-4	sll0208	GO:0044424	<1e-4	slr0386	GO:0042626	0.0185
sll1763	GO:0008150	<1e-4	sll0676	GO:0016052	<1e-4	sll5004	GO:0042623	<1e-4	slr7098	GO:0043226	<1e-4	ssr6079	GO:0016874	0.0184
sll0563	GO:0071496	<1e-4	slr1444	GO:0042180	<1e-4	slr1142	GO:0008171	<1e-4	ssr2787	GO:0044444	<1e-4	slr7023	GO:0016874	0.0184
sll0853	GO:0046907	<1e-4	sll0843	GO:0006811	<1e-4	slr0519	GO:0015405	<1e-4	sll1652	GO:0005575	<1e-4	slr1927	GO:0016874	0.0184
ssl2245	GO:0006753	<1e-4	ssl1577	GO:0010468	<1e-4	sll5128	GO:0022832	<1e-4	ssr2975	GO:0044444	<1e-4	ssr1407	GO:0016874	0.0184
sll1835	GO:0009161	<1e-4	slr0304	GO:0044283	<1e-4	slr6080	GO:0022891	<1e-4	ssr6019	GO:0044425	<1e-4	slr0960	GO:0016874	0.0184
sll1773	GO:0042451	<1e-4	sll0281	GO:0010468	<1e-4	sll1389	GO:0015267	<1e-4	slr5073	GO:0044422	<1e-4	slr6074	GO:0016874	0.0184
ssr5120	GO:0080090	<1e-4	slr7060	GO:0051246	<1e-4	slr0195	GO:0022892	<1e-4	sll0685	GO:0043226	<1e-4	slr0769	GO:0016874	0.0184
slr7080	GO:0046164	<1e-4	ssl5027	GO:0009057	<1e-4	slr1196	GO:0022891	<1e-4	slr0299	GO:0044464	<1e-4	slr1384	GO:0016874	0.0184
sll5034	GO:0009057	<1e-4	slr2012	GO:0051818	<1e-4	sll1640	GO:0015267	<1e-4	slr7094	GO:0043226	<1e-4	ssl1464	GO:0016874	0.0184
slr1958	GO:0031640	<1e-4	sll1193	GO:0046907	<1e-4	sll8002	GO:0016741	<1e-4	sll1130	GO:0044424	<1e-4	sll0846	GO:0016874	0.0184
slr1773	GO:0046907	<1e-4	ssr7017	GO:0009259	<1e-4	slr1261	GO:0008171	<1e-4	sll0024	GO:0044464	<1e-4	ssr6003	GO:0016874	0.0184
slr1647	GO:0009991	<1e-4	slr0885	GO:0019222	<1e-4	slr0810	GO:0022838	<1e-4	slr0250	GO:0044425	<1e-4	sll1651	GO:0016874	0.0184
sll1142	GO:0043412	<1e-4	sll0191	GO:0019362	<1e-4	ssr1258	GO:0008171	<1e-4	ssr1768	GO:0044446	<1e-4	sll1004	GO:0016874	0.0184
slr0483	GO:0044283	<1e-4	sll7055	GO:0006766	<1e-4	sll0787	GO:0015405	<1e-4	ssr2009	GO:0044446	<1e-4	slr5018	GO:0016874	0.0184
slr7096	GO:0051171	<1e-4	slr0199	GO:0044255	<1e-4	sll1400	GO:0022838	<1e-4	sll1092	GO:0044425	<1e-4	sll1509	GO:0016874	0.0184
slr1230	GO:0009132	<1e-4	slr0582	GO:0009263	<1e-4	ssl2384	GO:0043492	<1e-4	slr2010	GO:0044446	<1e-4	slr1660	GO:0016874	0.0184
sll0406	GO:0048518	<1e-4	sll0875	GO:0009161	<1e-4	sll2006	GO:0016817	<1e-4	slr7073	GO:0044422	<1e-4	sll1265	GO:0016874	0.0184
slr7023	GO:0052188	<1e-4	slr1340	GO:0031323	<1e-4	slr1266	GO:0015293	<1e-4	slr0885	GO:0044424	<1e-4	ssl2920	GO:0016874	0.0184
sll0103	GO:0019222	<1e-4	sll0843	GO:0065007	<1e-4	slr0610	GO:0042623	<1e-4	slr0601	GO:0044424	<1e-4	slr7081	GO:0016874	0.0184
ssl8008	GO:0006066	<1e-4	ssl1923	GO:0009142	<1e-4	sll1461	GO:0015267	<1e-4	slr1406	GO:0044425	<1e-4	sll1696	GO:0016874	0.0184
ssl7007	GO:0044271	<1e-4	sll0397	GO:0048878	<1e-4	slr0356	GO:0043167	<1e-4	slr0695	GO:0044464	<1e-4	slr1306	GO:0016874	0.0184
slr1258	GO:0009142	<1e-4	slr7071	GO:0010556	<1e-4	sll1476	GO:0022857	<1e-4	sll0645	GO:0044444	<1e-4	slr1546	GO:0016874	0.0184
sll1505	GO:0045184	<1e-4	sll0505	GO:0031323	<1e-4	sll0252	GO:0022891	<1e-4	sll0274	GO:0044446	<1e-4	sll8007	GO:0016874	0.0184
slr1537	GO:0010468	<1e-4	slr0865	GO:0006733	<1e-4	sll1344	GO:0022890	<1e-4	slr0271	GO:0005575	<1e-4	slr1535	GO:0016874	0.0184
slr6090	GO:0071496	<1e-4	slr1444	GO:0055086	<1e-4	sll7033	GO:0015291	<1e-4	ssl2717	GO:0044422	<1e-4	ssr0335	GO:0016874	0.0184

slr2052	GO:0034654	<1e-4	slr6087	GO:0019752	<1e-4	sll0727	GO:0016817	<1e-4	ssr1391	GO:0043226	<1e-4	slr0581	GO:0016874	0.0184
sll1247	GO:0034641	<1e-4	slr1813	GO:0009117	<1e-4	slr6101	GO:0042623	<1e-4	slr0990	GO:0044446	<1e-4	slr1619	GO:0016874	0.0184
slr1816	GO:0050801	<1e-4	sll0910	GO:0006220	<1e-4	slr1362	GO:0016741	<1e-4	sll0926	GO:0044446	<1e-4	sll0762	GO:0016874	0.0184
sll0488	GO:0045184	<1e-4	ssl0461	GO:0005996	<1e-4	slr0521	GO:0015399	<1e-4	slr1444	GO:0005575	<1e-4	sll1092	GO:0016874	0.0184
sll1763	GO:0006082	<1e-4	slr0989	GO:0065007	<1e-4	slr7013	GO:0016746	<1e-4	sll0638	GO:0044464	<1e-4	slr6090	GO:0016874	0.0184
sll1455	GO:0051179	<1e-4	slr6101	GO:0008610	<1e-4	sll0372	GO:0015267	<1e-4	slr5116	GO:0044424	<1e-4	slr0971	GO:0016874	0.0184
sll1714	GO:0043170	<1e-4	slr7101	GO:0050801	<1e-4	slr0656	GO:0016818	<1e-4	sll0609	GO:0044422	<1e-4	sll1132	GO:0016874	0.0184
slr0755	GO:0034641	<1e-4	slr1462	GO:0009987	<1e-4	sll1191	GO:0022836	<1e-4	ssr1256	GO:0005575	<1e-4	slr7011	GO:0016874	0.0184
sll0482	GO:0046164	<1e-4	slr6039	GO:0009259	<1e-4	slr1544	GO:0015077	<1e-4	sll1582	GO:0044464	<1e-4	slr6013	GO:0016874	0.0184
slr5053	GO:0005996	<1e-4	slr1262	GO:0009144	<1e-4	sll1318	GO:0022832	<1e-4	sll0775	GO:0044446	<1e-4	ssl2717	GO:0016874	0.0184
sll0048	GO:0031640	<1e-4	slr0981	GO:0006066	<1e-4	sll1306	GO:0016462	<1e-4	ssl5108	GO:0044422	<1e-4	slr1068	GO:0016874	0.0184
sll0804	GO:0009259	<1e-4	slr0780	GO:0010556	<1e-4	ssl2814	GO:0043167	<1e-4	sll0871	GO:0044422	<1e-4	slr1768	GO:0016874	0.0184
slr1677	GO:0044260	<1e-4	sll2015	GO:0043933	<1e-4	ssl0738	GO:0008171	<1e-4	sll1916	GO:0044444	<1e-4	sll1130	GO:0016874	0.0184
slr1150	GO:0009309	<1e-4	slr0962	GO:0051188	<1e-4	sll0752	GO:0022891	<1e-4	slr1788	GO:0005575	<1e-4	slr0912	GO:0016874	0.0184
slr0554	GO:0009991	<1e-4	slr1168	GO:0080090	<1e-4	sll1882	GO:0015267	<1e-4	ssr1391	GO:0005575	<1e-4	sll1862	GO:0016874	0.0184
slr1815	GO:0051347	<1e-4	sll1464	GO:0050789	<1e-4	slr1863	GO:0022891	<1e-4	slr1862	GO:0044464	<1e-4	sll0478	GO:0016874	0.0184
sll0503	GO:0006721	<1e-4	slr1690	GO:0009126	<1e-4	sll0572	GO:0022890	<1e-4	slr1788	GO:0044424	<1e-4	ssl3383	GO:0016874	0.0184
slr0921	GO:0009394	<1e-4	slr0625	GO:0009144	<1e-4	sll0810	GO:0016818	<1e-4	ssl2064	GO:0044424	<1e-4	sll0611	GO:0016874	0.0184
ssl7045	GO:0071842	<1e-4	slr1315	GO:0006720	<1e-4	sll5128	GO:0043492	<1e-4	sll1832	GO:0044422	<1e-4	slr2010	GO:0016874	0.0184
sll5063	GO:0043933	<1e-4	sll0780	GO:0043170	<1e-4	sll0497	GO:0022890	<1e-4	ssr2912	GO:0044422	<1e-4	slr6080	GO:0016874	0.0184
slr2101	GO:0006721	<1e-4	sll8012	GO:0031326	<1e-4	slr2092	GO:0022891	<1e-4	slr6106	GO:0044446	<1e-4	slr1657	GO:0016874	0.0184
sll1378	GO:0009987	<1e-4	sll8019	GO:0009889	<1e-4	slr1450	GO:0015399	<1e-4	ssr5019	GO:0043226	<1e-4	slr6005	GO:0016874	0.0184
slr0400	GO:0044106	<1e-4	slr1052	GO:0010468	<1e-4	slr0204	GO:0022832	<1e-4	sll1562	GO:0044446	<1e-4	slr0362	GO:0016874	0.0184
sll1509	GO:0048878	<1e-4	slr0664	GO:0052111	<1e-4	ssr5121	GO:0016818	<1e-4	ssr3341	GO:0044446	<1e-4	slr0388	GO:0016874	0.0184
slr5024	GO:0071554	<1e-4	sll5004	GO:0006576	<1e-4	slr1721	GO:0016746	<1e-4	sll6052	GO:0044446	<1e-4	slr1263	GO:0016874	0.0184
slr5127	GO:0016053	<1e-4	sll1873	GO:0019362	<1e-4	slr6072	GO:0022838	<1e-4	sll0552	GO:0044464	<1e-4	ssl1498	GO:0016874	0.0184
slr0740	GO:0031323	<1e-4	sll1455	GO:0043549	<1e-4	slr0505	GO:0008171	<1e-4	sll1188	GO:0005575	<1e-4	sll7043	GO:0016874	0.0184
sll1530	GO:0009141	<1e-4	ssr1155	GO:0090304	<1e-4	sll0756	GO:0015405	<1e-4	ssr6048	GO:0044425	<1e-4	ssr5020	GO:0016874	0.0184
slr6100	GO:0031323	<1e-4	sll0230	GO:0043933	<1e-4	slr2117	GO:0060089	<1e-4	slr1276	GO:0044422	<1e-4	ssr2611	GO:0016874	0.0184
slr0871	GO:0046128	<1e-4	sll2006	GO:0044106	<1e-4	slr0146	GO:0003674	<1e-4	sll8027	GO:0043226	<1e-4	slr0049	GO:0016874	0.0184
sll1217	GO:0071840	<1e-4	sll1472	GO:0006793	<1e-4	sll1773	GO:0022892	<1e-4	sll1898	GO:0043226	<1e-4	ssl7046	GO:0016874	0.0184
slr1659	GO:0071554	<1e-4	sll0762	GO:0044260	<1e-4	sll0783	GO:0042623	<1e-4	slr1362	GO:0044464	<1e-4	slr1270	GO:0016874	0.0184
ssr2803	GO:0051818	<1e-4	ssl1255	GO:0044003	<1e-4	slr1704	GO:0003674	<1e-4	slr0181	GO:0044424	<1e-4	slr1362	GO:0016874	0.0184
sll0325	GO:0006733	<1e-4	slr1880	GO:0048523	<1e-4	slr1127	GO:0015267	<1e-4	sll1233	GO:0044464	<1e-4	slr6008	GO:0016874	0.0184
slr0192	GO:0046394	<1e-4	sll8002	GO:0051234	<1e-4	slr0065	GO:0022836	<1e-4	ssl2384	GO:0044425	<1e-4	ssl1520	GO:0016874	0.0184
sll8004	GO:0072524	<1e-4	sll0423	GO:0006163	<1e-4	sll7047	GO:0015399	<1e-4	sll0853	GO:0043226	<1e-4	slr0907	GO:0016874	0.0184
ssl1417	GO:0046907	<1e-4	slr0305	GO:0044283	<1e-4	ssl5065	GO:0022836	<1e-4	slr2049	GO:0005575	<1e-4	ssl3142	GO:0016874	0.0184
sll0361	GO:0009117	<1e-4	slr1752	GO:0009123	<1e-4	slr0919	GO:0060089	<1e-4	ssl3142	GO:0043226	<1e-4	slr1407	GO:0016874	0.0184
slr1431	GO:0006720	<1e-4	ssr3341	GO:0019219	<1e-4	sll1089	GO:0015293	<1e-4	slr0712	GO:0044424	<1e-4	sll5090	GO:0016874	0.0184
slr0789	GO:0031640	<1e-4	sll0008	GO:0034654	<1e-4	slr1812	GO:0016746	<1e-4	ssl3382	GO:0043226	<1e-4	sll1447	GO:0016874	0.0184
slr0092	GO:0052111	<1e-4	sll1749	GO:0050801	<1e-4	sll1388	GO:0022857	<1e-4	slr0654	GO:0044444	<1e-4	slr8014	GO:0016874	0.0184
slr6014	GO:0051186	<1e-4	slr1263	GO:0031640	<1e-4	sll1060	GO:0016741	<1e-4	sll1654	GO:0044422	<1e-4	slr1914	GO:0016874	0.0184
slr2144	GO:0009056	<1e-4	sll1638	GO:0044248	<1e-4	sll1262	GO:0022836	<1e-4	slr1920	GO:0005575	<1e-4	sll0775	GO:0016874	0.0184
sll1913	GO:0022607	<1e-4	sll0147	GO:0018130	<1e-4	sll1601	GO:0022857	<1e-4	sll0293	GO:0043226	<1e-4	slr1907	GO:0016874	0.0184
slr0586	GO:0048519	<1e-4	sll0982	GO:0009987	<1e-4	slr1753	GO:0022857	<1e-4	sll0448	GO:0044425	<1e-4	slr0199	GO:0016874	0.0184
sll1455	GO:0071554	<1e-4	ssl2138	GO:0044271	<1e-4	sll1036	GO:0016818	<1e-4	slr0887	GO:0044444	<1e-4	slr0821	GO:0016874	0.0184
sll6052	GO:0048518	<1e-4	slr0919	GO:0050789	<1e-4	slr1102	GO:0022838	<1e-4	ssl5068	GO:0044444	<1e-4	sll0802	GO:0016874	0.0184
slr6066	GO:0031640	<1e-4	sll1630	GO:0034654	<1e-4	slr6063	GO:0015077	<1e-4	slr1660	GO:0044425	<1e-4	sll1396	GO:0016874	0.0184
slr0157	GO:0009892	<1e-4	ssl0739	GO:0048519	<1e-4	slr1875	GO:0022891	<1e-4	ssr2975	GO:0044422	<1e-4	ssr3410	GO:0016874	0.0184
sll0763	GO:0006811	<1e-4	slr1648	GO:0006576	<1e-4	slr0645	GO:0015267	<1e-4	ssl7022	GO:0044424	<1e-4	slr1464	GO:0016874	0.0184
slr1230	GO:0009150	<1e-4	slr1612	GO:0009144	<1e-4	sll1873	GO:0015077	<1e-4	slr0876	GO:0005575	<1e-4	sll0419	GO:0016874	0.0184
slr1914	GO:0044271	<1e-4	slr7092	GO:0006720	<1e-4	slr0479	GO:0016818	<1e-4	sll2011	GO:0044464	<1e-4	slr0489	GO:0016874	0.0184
sll1749	GO:0019222	<1e-4	sll0513	GO:0009892	<1e-4	sll1797	GO:0016746	<1e-4	slr0885	GO:0044446	<1e-4	sll7066	GO:0016874	0.0184
ssl5015	GO:0009394	<1e-4	ssl0738	GO:0034660	<1e-4	slr0006	GO:0015075	<1e-4	slr5119	GO:0005575	<1e-4	slr0606	GO:0016874	0.0184

ssl3573	GO:0051171	<1e-4	slr1188	GO:0009259	<1e-4	sll0858	GO:0015405	<1e-4	slr1383	GO:0044444	<1e-4	ssr2317	GO:0016874	0.0184
sll6053	GO:0051818	<1e-4	slr1944	GO:0018130	<1e-4	slr0975	GO:0022836	<1e-4	sll1613	GO:0044464	<1e-4	slr7092	GO:0016874	0.0184
sll0760	GO:0051179	<1e-4	ssl7048	GO:0044106	<1e-4	sll0319	GO:0043167	<1e-4	slr2070	GO:0044422	<1e-4	slr5119	GO:0016874	0.0184
ssr2067	GO:0065007	<1e-4	slr0519	GO:0006733	<1e-4	slr1070	GO:0016746	<1e-4	slr1327	GO:0044446	<1e-4	slr7013	GO:0016874	0.0184
slr1827	GO:0009263	<1e-4	slr0142	GO:0046394	<1e-4	slr2049	GO:0015075	<1e-4	slr5018	GO:0044446	<1e-4	slr1677	GO:0016874	0.0184
sll0442	GO:0006721	<1e-4	slr1189	GO:0048522	<1e-4	sll1783	GO:0022832	<1e-4	ssl0750	GO:0043226	<1e-4	slr6004	GO:0016874	0.0184
sll0647	GO:0051179	<1e-4	slr7092	GO:0044283	<1e-4	slr1339	GO:0015293	<1e-4	sll0625	GO:0005575	<1e-4	sll1921	GO:0016874	0.0184
sll1542	GO:0044003	<1e-4	slr1591	GO:0006631	<1e-4	sll1004	GO:0043492	<1e-4	slr0551	GO:0044424	<1e-4	sll0487	GO:0016874	0.0184
sll0867	GO:0016054	<1e-4	sll0788	GO:0051347	<1e-4	sll0372	GO:0022836	<1e-4	ssr7093	GO:0043226	<1e-4	sll1109	GO:0016874	0.0184
sll1698	GO:0043412	<1e-4	sll0736	GO:0044271	<1e-4	ssr2439	GO:0016462	<1e-4	sll0243	GO:0044425	<1e-4	slr1468	GO:0016874	0.0184
slr1618	GO:0044281	<1e-4	slr2070	GO:0009117	<1e-4	slr0238	GO:0015267	<1e-4	slr0498	GO:0044464	<1e-4	slr7012	GO:0016874	0.0184
ssl7045	GO:0045184	<1e-4	sll1696	GO:0043170	<1e-4	ssl1378	GO:0022857	<1e-4	sll0781	GO:0044425	<1e-4	slr6021	GO:0016874	0.0184
slr1534	GO:0006793	<1e-4	sll0864	GO:0006732	<1e-4	slr1851	GO:0043167	<1e-4	ssl2733	GO:0005575	<1e-4	sll1203	GO:0016874	0.0184
ssl1923	GO:0051171	<1e-4	slr1807	GO:0006811	<1e-4	sll0269	GO:0043167	<1e-4	slr1658	GO:0044446	<1e-4	sll0659	GO:0016874	0.0184
slr6100	GO:0048523	<1e-4	ssl0242	GO:0016052	<1e-4	sll1949	GO:0016818	<1e-4	sll0577	GO:0044424	<1e-4	sll0688	GO:0006950	0.0183
sll1563	GO:0048522	<1e-4	sll0787	GO:0019752	<1e-4	sll0785	GO:0022892	<1e-4	slr1768	GO:0044425	<1e-4	slr1762	GO:0006950	0.0183
ssl2245	GO:0009259	<1e-4	sll2013	GO:0019752	<1e-4	sll2006	GO:0022836	<1e-4	slr7096	GO:0005575	<1e-4	sll0980	GO:0006950	0.0183
slr6104	GO:0031326	<1e-4	sll0760	GO:0031640	<1e-4	slr7026	GO:0022832	<1e-4	slr1470	GO:0043226	<1e-4	slr2080	GO:0006950	0.0183
ssl6061	GO:0043170	<1e-4	sll1652	GO:0009142	<1e-4	sll0102	GO:0016817	<1e-4	sll0596	GO:0044424	<1e-4	ssr1528	GO:0006950	0.0183
slr0023	GO:0044003	<1e-4	sll0910	GO:0050789	<1e-4	sll0147	GO:0015267	<1e-4	slr2018	GO:0043226	<1e-4	slr0207	GO:0006950	0.0183
sll1025	GO:0006066	<1e-4	slr1811	GO:0009124	<1e-4	sll1119	GO:0016741	<1e-4	sll1272	GO:0044425	<1e-4	slr1338	GO:0006950	0.0183
slr0304	GO:0046164	<1e-4	sll0930	GO:0046128	<1e-4	sll1004	GO:0042623	<1e-4	slr0250	GO:0044444	<1e-4	slr0702	GO:0006950	0.0183
slr1546	GO:0052188	<1e-4	slr1406	GO:0009117	<1e-4	sll1702	GO:0016817	<1e-4	slr1186	GO:0005575	<1e-4	sll0319	GO:0006950	0.0183
slr1895	GO:0034654	<1e-4	sll1119	GO:0009262	<1e-4	slr1999	GO:0022838	<1e-4	sll1063	GO:0005575	<1e-4	sll0359	GO:0006950	0.0183
sll0780	GO:0051818	<1e-4	sll1106	GO:0055086	<1e-4	slr1449	GO:0015075	<1e-4	slr1218	GO:0005575	<1e-4	sll1634	GO:0006950	0.0183
sll1191	GO:0019362	<1e-4	slr1644	GO:0006066	<1e-4	slr0076	GO:0022838	<1e-4	slr0637	GO:0044446	<1e-4	sll0810	GO:0006950	0.0183
sll5003	GO:0043412	<1e-4	sll7086	GO:0050801	<1e-4	sll8027	GO:0022892	<1e-4	sll0688	GO:0044464	<1e-4	slr2048	GO:0006950	0.0183
ssr7093	GO:0009124	<1e-4	sll0703	GO:0006811	<1e-4	slr7095	GO:0043167	<1e-4	sll7077	GO:0044425	<1e-4	slr1240	GO:0006950	0.0183
slr0147	GO:0042180	<1e-4	slr6047	GO:0044260	<1e-4	ssr2615	GO:0015291	<1e-4	slr0146	GO:0005575	<1e-4	slr0272	GO:0006950	0.0183
sll1092	GO:0009059	<1e-4	ssl5091	GO:0044106	<1e-4	ssr3532	GO:0015077	<1e-4	ssl8008	GO:0044446	<1e-4	slr0053	GO:0006950	0.0183
sll5003	GO:0071554	<1e-4	ssr8013	GO:0016052	<1e-4	sll0445	GO:0042623	<1e-4	slr1827	GO:0044422	<1e-4	slr1209	GO:0006950	0.0183
ssl1046	GO:0034654	<1e-4	slr1616	GO:0019637	<1e-4	slr1493	GO:0022838	<1e-4	slr0551	GO:0005575	<1e-4	slr0172	GO:0006950	0.0183
slr2118	GO:0009165	<1e-4	sll0656	GO:0009150	<1e-4	sll0241	GO:0016746	<1e-4	sll0448	GO:0005575	<1e-4	slr0456	GO:0006950	0.0183
slr0104	GO:0006576	<1e-4	sll0473	GO:0071840	<1e-4	slr8014	GO:0022857	<1e-4	ssl3692	GO:0005575	<1e-4	sll0737	GO:0006950	0.0183
ssl2920	GO:0051234	<1e-4	slr7071	GO:0034654	<1e-4	ssr3532	GO:0043167	<1e-4	sll0406	GO:0043226	<1e-4	ssl0352	GO:0006950	0.0182
sll1273	GO:0043648	<1e-4	ssl0353	GO:0006766	<1e-4	sll1960	GO:0022891	<1e-4	slr1790	GO:0005575	<1e-4	sll1954	GO:0006950	0.0182
slr6039	GO:0044255	<1e-4	sll8011	GO:0031323	<1e-4	sll0843	GO:0016746	<1e-4	slr0517	GO:0005575	<1e-4	sll0309	GO:0006950	0.0182
slr1563	GO:0046128	<1e-4	slr1210	GO:0052188	<1e-4	sll0496	GO:0043492	<1e-4	slr0990	GO:0005575	<1e-4	slr1854	GO:0006950	0.0182
sll1763	GO:0009262	<1e-4	sll0586	GO:0043170	<1e-4	slr1533	GO:0022832	<1e-4	slr0780	GO:0044464	<1e-4	slr0386	GO:0006950	0.0182
slr6047	GO:0052188	<1e-4	slr1262	GO:0019222	<1e-4	slr6014	GO:0016817	<1e-4	slr0509	GO:0005575	<1e-4	slr1275	GO:0006950	0.0182
ssr6003	GO:0042451	<1e-4	ssr1407	GO:0019362	<1e-4	slr0341	GO:0016462	<1e-4	slr5012	GO:0044464	<1e-4	ssr2755	GO:0006950	0.0182
sll0272	GO:0055086	<1e-4	slr0111	GO:0009259	<1e-4	slr0587	GO:0060089	<1e-4	sll0252	GO:0005575	<1e-4	ssr1765	GO:0006950	0.0182
sll1241	GO:0048518	<1e-4	ssr2975	GO:0006732	<1e-4	slr1339	GO:0043492	<1e-4	ssl3291	GO:0005575	<1e-4	slr0770	GO:0006950	0.0182
slr1259	GO:0072522	<1e-4	sll0174	GO:0042455	<1e-4	sll0661	GO:0016741	<1e-4	slr0050	GO:0005575	<1e-4	slr0723	GO:0006950	0.0182
sll0939	GO:0072521	<1e-4	slr1068	GO:0065007	<1e-4	ssr1407	GO:0042623	<1e-4	sll0102	GO:0043226	<1e-4	slr0023	GO:0006950	0.0182
slr0742	GO:0046394	<1e-4	slr1203	GO:0051186	<1e-4	slr0885	GO:0043167	<1e-4	sll1399	GO:0044446	<1e-4	sll1262	GO:0006950	0.0182
sll2007	GO:0006721	<1e-4	slr0609	GO:0010468	<1e-4	sll0780	GO:0015267	<1e-4	sll0267	GO:0005575	<1e-4	slr1081	GO:0006950	0.0182
slr0059	GO:0008150	<1e-4	slr1032	GO:0006811	<1e-4	slr1644	GO:0016829	<1e-4	ssr6085	GO:0005575	<1e-4	slr1913	GO:0006950	0.0182
sll0585	GO:0044003	<1e-4	slr1415	GO:0009059	<1e-4	slr1436	GO:0022803	<1e-4	slr0870	GO:0043226	<1e-4	slr1593	GO:0006950	0.0182
slr0147	GO:0009124	<1e-4	slr0634	GO:0009056	<1e-4	sll1925	GO:0016462	<1e-4	ssl1377	GO:0044424	<1e-4	sll1630	GO:0006950	0.0182
ssr7093	GO:0055086	<1e-4	sll0564	GO:0006163	<1e-4	slr1442	GO:0016746	<1e-4	slr1338	GO:0005575	<1e-4	sll0606	GO:0016820	0.0182
sll1239	GO:0033013	<1e-4	ssl2920	GO:0048518	<1e-4	sll1638	GO:0022891	<1e-4	sll1783	GO:0005575	<1e-4	ssr2201	GO:0016820	0.0182
sll0066	GO:0046394	<1e-4	ssl2420	GO:0051716	<1e-4	slr1800	GO:0016741	<1e-4	sll2006	GO:0044444	<1e-4	ssr2803	GO:0016820	0.0182
slr1951	GO:0009165	<1e-4	ssr7093	GO:0072522	<1e-4	sll0361	GO:0016817	<1e-4	slr0924	GO:0044422	<1e-4	sll0647	GO:0016820	0.0182

slr0702	GO:0044271	<1e-4	ssl5096	GO:0051171	<1e-4	sll0481	GO:0016817	<1e-4	slr1978	GO:0044422	<1e-4	slr1032	GO:0016820	0.0182
slr1218	GO:0043412	<1e-4	ssl2069	GO:0009126	<1e-4	sll0188	GO:0016817	<1e-4	slr1053	GO:0044464	<1e-4	sll0272	GO:0016820	0.0182
sll1526	GO:0022607	<1e-4	slr1436	GO:0072521	<1e-4	slr0771	GO:0015399	<1e-4	sll1651	GO:0044444	<1e-4	sll0103	GO:0016820	0.0182
sll0785	GO:0065007	<1e-4	slr6006	GO:0031640	<1e-4	slr6044	GO:0016818	<1e-4	slr7094	GO:0005575	<1e-4	sll0932	GO:0016820	0.0182
slr1442	GO:0031326	<1e-4	slr0609	GO:0018130	<1e-4	slr7102	GO:0022803	<1e-4	sll6053	GO:0043226	<1e-4	sll1763	GO:0016740	0.0181
sll1530	GO:0016053	<1e-4	sll8032	GO:0018130	<1e-4	sll0176	GO:0015075	<1e-4	slr1278	GO:0005575	<1e-4	slr1614	GO:0016740	0.0181
slr0692	GO:0051188	<1e-4	ssl2717	GO:0009144	<1e-4	slr0505	GO:0022892	<1e-4	slr6068	GO:0044422	<1e-4	slr1699	GO:0005515	0.018
slr0813	GO:0072522	<1e-4	sll0098	GO:0019219	<1e-4	sll0736	GO:0016746	<1e-4	slr1203	GO:0044425	<1e-4	slr1812	GO:0005515	0.018
slr0695	GO:0044248	<1e-4	slr0605	GO:0043412	<1e-4	sll0372	GO:0022803	<1e-4	ssl1255	GO:0044425	<1e-4	slr1444	GO:0004518	0.0177
sll7066	GO:0034641	<1e-4	sll0497	GO:0016054	<1e-4	slr6022	GO:0022832	<1e-4	sll1009	GO:0044424	<1e-4	slr1098	GO:0004518	0.0177
slr1307	GO:0044260	<1e-4	ssr5020	GO:0034654	<1e-4	slr6021	GO:0022891	<1e-4	sll0252	GO:0044425	<1e-4	slr1865	GO:0004518	0.0177
sll0886	GO:0051818	<1e-4	slr0909	GO:0043170	<1e-4	slr1082	GO:0015291	<1e-4	ssl1918	GO:0005575	<1e-4	sll1751	GO:0004518	0.0177
slr1306	GO:0010556	<1e-4	slr0751	GO:0060255	<1e-4	sll0447	GO:0016462	<1e-4	sll5069	GO:0043226	<1e-4	sll0910	GO:0004518	0.0177
sll1942	GO:0006732	<1e-4	ssr2047	GO:0010468	<1e-4	sll8035	GO:0022832	<1e-4	sll0236	GO:0044464	<1e-4	slr1726	GO:0004518	0.0177
slr1977	GO:0044255	<1e-4	sll0815	GO:0046907	<1e-4	ssl7022	GO:0022890	<1e-4	slr1150	GO:0043226	<1e-4	slr0479	GO:0004518	0.0177
slr1534	GO:0009124	<1e-4	ssr0336	GO:0006066	<1e-4	slr1406	GO:0015077	<1e-4	ssr2615	GO:0005575	<1e-4	slr1047	GO:0004518	0.0177
sll5026	GO:0019637	<1e-4	ssl2717	GO:0052188	<1e-4	sll1526	GO:0015291	<1e-4	slr1210	GO:0005575	<1e-4	sll0361	GO:0004518	0.0177
slr1235	GO:0019751	<1e-4	slr7101	GO:0044260	<1e-4	slr1534	GO:0015291	<1e-4	slr0870	GO:0044425	<1e-4	ssl1377	GO:0004518	0.0177
sll2015	GO:0048522	<1e-4	slr0104	GO:0008150	<1e-4	sll1372	GO:0022838	<1e-4	slr1895	GO:0044464	<1e-4	ssl2996	GO:0004518	0.0177
sll1563	GO:0031326	<1e-4	sll0175	GO:0045184	<1e-4	slr1885	GO:0022857	<1e-4	slr0146	GO:0044424	<1e-4	sll1251	GO:0006807	0.0176
ssr2047	GO:0051234	<1e-4	sll1472	GO:0046164	<1e-4	slr1613	GO:0015267	<1e-4	slr0110	GO:0044425	<1e-4	sll0319	GO:0006807	0.0176
slr0890	GO:0043170	<1e-4	slr5118	GO:0009987	<1e-4	sll0263	GO:0022857	<1e-4	slr0238	GO:0044425	<1e-4	sll0281	GO:0006807	0.0176
ssl1918	GO:0009161	<1e-4	slr2105	GO:0019752	<1e-4	sll0843	GO:0015077	<1e-4	ssr2142	GO:0005575	<1e-4	sll1240	GO:0019538	0.0176
sll1761	GO:0051179	<1e-4	ssr5019	GO:0009199	<1e-4	sll1233	GO:0015267	<1e-4	sll5069	GO:0044425	<1e-4	slr0172	GO:0006807	0.0176
slr1800	GO:0006721	<1e-4	slr5116	GO:0009260	<1e-4	ssl2138	GO:0008171	<1e-4	slr0285	GO:0044422	<1e-4	slr1303	GO:0006807	0.0176
slr0111	GO:0072527	<1e-4	sll0743	GO:0071842	<1e-4	sll0931	GO:0015293	<1e-4	sll0761	GO:0005575	<1e-4	slr0104	GO:0006807	0.0176
sll1201	GO:0009142	<1e-4	sll0702	GO:0009260	<1e-4	ssr5117	GO:0022892	<1e-4	slr0664	GO:0044444	<1e-4	slr1852	GO:0019538	0.0176
sll5028	GO:0009150	<1e-4	ssl1263	GO:0006811	<1e-4	slr0264	GO:0016817	<1e-4	slr6067	GO:0044425	<1e-4	slr0053	GO:0019538	0.0176
slr1413	GO:0044260	<1e-4	slr1169	GO:0051347	<1e-4	sll1562	GO:0015291	<1e-4	sll1921	GO:0044424	<1e-4	slr0654	GO:0006807	0.0176
slr7058	GO:0051179	<1e-4	sll1765	GO:0006732	<1e-4	slr1566	GO:0015077	<1e-4	sll1160	GO:0044422	<1e-4	sll0156	GO:0006807	0.0176
slr1362	GO:0050794	<1e-4	sll1191	GO:0043549	<1e-4	sll1163	GO:0016741	<1e-4	sll0994	GO:0044444	<1e-4	slr1122	GO:0006807	0.0176
ssr1698	GO:0046164	<1e-4	slr1484	GO:0044106	<1e-4	ssl8003	GO:0042623	<1e-4	slr0680	GO:0044424	<1e-4	sll0781	GO:0006807	0.0176
sll0775	GO:0009141	<1e-4	sll0382	GO:0052188	<1e-4	sll1476	GO:0005342	<1e-4	slr0006	GO:0044424	<1e-4	slr1287	GO:0019538	0.0176
sll0481	GO:0009123	<1e-4	slr0964	GO:0006766	<1e-4	slr2003	GO:0016462	<1e-4	slr6100	GO:0044446	<1e-4	sll0787	GO:0006807	0.0176
slr1668	GO:0006066	<1e-4	slr1032	GO:0042455	<1e-4	ssr2843	GO:0015293	<1e-4	slr1563	GO:0005575	<1e-4	sll0981	GO:0019538	0.0176
sll1400	GO:0046164	<1e-4	slr0914	GO:0065007	<1e-4	slr0769	GO:0043492	<1e-4	sll1411	GO:0044422	<1e-4	sll1054	GO:0019538	0.0176
slr1815	GO:0043648	<1e-4	sll0752	GO:0006753	<1e-4	ssl1520	GO:0022838	<1e-4	sll1429	GO:0044444	<1e-4	ssr2754	GO:0019538	0.0176
sgl0002	GO:0006576	<1e-4	slr1958	GO:0042451	<1e-4	slr0013	GO:0022803	<1e-4	ssl1417	GO:0044424	<1e-4	slr1413	GO:0006807	0.0176
ssl1004	GO:0044260	<1e-4	slr0199	GO:0044106	<1e-4	sll1021	GO:0022857	<1e-4	sll1891	GO:0043226	<1e-4	sll1752	GO:0006807	0.0176
slr0784	GO:0009126	<1e-4	slr0442	GO:0048523	<1e-4	sll0405	GO:0043492	<1e-4	slr0468	GO:0043226	<1e-4	sll1541	GO:0006807	0.0176
sll1995	GO:0008150	<1e-4	slr1117	GO:0044260	<1e-4	sll0751	GO:0016817	<1e-4	sll0252	GO:0044444	<1e-4	sll1173	GO:0006807	0.0176
slr6006	GO:0043436	<1e-4	ssl8005	GO:0031326	<1e-4	slr0695	GO:0008171	<1e-4	slr0374	GO:0044444	<1e-4	sll0156	GO:0019538	0.0176
slr1270	GO:0050801	<1e-4	slr1768	GO:0006082	<1e-4	sll1638	GO:0022838	<1e-4	ssr7017	GO:0005575	<1e-4	slr0650	GO:0006807	0.0176
sll1188	GO:0034641	<1e-4	slr1530	GO:0009132	<1e-4	slr1288	GO:0022836	<1e-4	slr1258	GO:0044464	<1e-4	sll7065	GO:0019538	0.0176
slr1862	GO:0019222	<1e-4	ssl5098	GO:0046164	<1e-4	sll1961	GO:0022838	<1e-4	slr0887	GO:0043226	<1e-4	sll1634	GO:0019538	0.0176
sll0060	GO:0016053	<1e-4	slr6087	GO:0034660	<1e-4	slr0801	GO:0016741	<1e-4	ssr6062	GO:0044444	<1e-4	ssl3382	GO:0019538	0.0176
ssl8003	GO:0010556	<1e-4	slr1084	GO:0044281	<1e-4	slr0195	GO:0016741	<1e-4	slr0919	GO:0044424	<1e-4	sll0688	GO:0006807	0.0176
slr1572	GO:0019222	<1e-4	slr1398	GO:0051171	<1e-4	slr1471	GO:0015405	<1e-4	slr0821	GO:0044446	<1e-4	sll1201	GO:0019538	0.0176
slr0313	GO:0009126	<1e-4	ssl1552	GO:0009056	<1e-4	slr1102	GO:0060089	<1e-4	sll0553	GO:0044444	<1e-4	sll0888	GO:0019538	0.0176
slr7083	GO:0071842	<1e-4	slr8022	GO:0009394	<1e-4	slr1259	GO:0016817	<1e-4	slr0553	GO:0044444	<1e-4	slr1544	GO:0019538	0.0176
slr0780	GO:0009987	<1e-4	slr0294	GO:0071841	<1e-4	slr1378	GO:0016741	<1e-4	slr7102	GO:0044424	<1e-4	sll1352	GO:0019538	0.0176
sll1775	GO:0001932	<1e-4	slr1573	GO:0044255	<1e-4	sll1942	GO:0015291	<1e-4	sll0827	GO:0043226	<1e-4	sll1873	GO:0006807	0.0176
sll0853	GO:0051171	<1e-4	sll1095	GO:0043412	<1e-4	sll1956	GO:0003674	<1e-4	ssl1972	GO:0043226	<1e-4	sll1071	GO:0019538	0.0176
ssl1792	GO:0019752	<1e-4	slr0358	GO:0008150	<1e-4	slr1436	GO:0060089	<1e-4	ssl7067	GO:0005575	<1e-4	slr1895	GO:0019538	0.0176

sll0532	GO:0080090	<1e-4	sll0072	GO:0055082	<1e-4	sll7066	GO:0005342	<1e-4	slr0670	GO:0005575	<1e-4	slr0729	GO:0019538	0.0176
slr1762	GO:0009987	<1e-4	slr1854	GO:0016053	<1e-4	sll0543	GO:0022891	<1e-4	slr7016	GO:0044425	<1e-4	slr1062	GO:0019538	0.0176
slr1533	GO:0009123	<1e-4	slr1396	GO:0072527	<1e-4	slr0211	GO:0022836	<1e-4	slr0771	GO:0043226	<1e-4	sll0871	GO:0006807	0.0176
sll1737	GO:0006732	<1e-4	slr2101	GO:0009394	<1e-4	ssr6002	GO:0016741	<1e-4	ssr2711	GO:0043226	<1e-4	sll1660	GO:0019538	0.0176
sll1884	GO:0006220	<1e-4	slr0770	GO:0052188	<1e-4	slr6045	GO:0043492	<1e-4	slr0921	GO:0005575	<1e-4	sll0737	GO:0006807	0.0176
slr0989	GO:0042180	<1e-4	sll0933	GO:0044238	<1e-4	sll1531	GO:0015405	<1e-4	sll0497	GO:0044444	<1e-4	slr1616	GO:0006807	0.0176
sll1142	GO:0006721	<1e-4	sll1702	GO:0048878	<1e-4	sll8012	GO:0016746	<1e-4	sll7030	GO:0044424	<1e-4	slr0111	GO:0019538	0.0176
slr0989	GO:0016053	<1e-4	slr6064	GO:0009309	<1e-4	ssl3692	GO:0022892	<1e-4	sll0102	GO:0044444	<1e-4	slr1762	GO:0019538	0.0176
slr0981	GO:0051188	<1e-4	ssr1114	GO:0009889	<1e-4	ssl2814	GO:0016817	<1e-4	slr1396	GO:0044444	<1e-4	slr0887	GO:0006807	0.0176
ssr7079	GO:0031640	<1e-4	sll0861	GO:0006766	<1e-4	sll0647	GO:0022838	<1e-4	sll1853	GO:0044425	<1e-4	slr2125	GO:0019538	0.0176
sll1751	GO:0031323	<1e-4	sll1757	GO:0022607	<1e-4	sll1726	GO:0016462	<1e-4	sll1770	GO:0005575	<1e-4	slr1647	GO:0006807	0.0176
slr2119	GO:0043933	<1e-4	ssl1552	GO:0009309	<1e-4	ssr2711	GO:0022838	<1e-4	sll0148	GO:0043226	<1e-4	ssr1114	GO:0019538	0.0176
ssl1377	GO:0022411	<1e-4	slr0924	GO:0071840	<1e-4	slr1774	GO:0043167	<1e-4	sll1191	GO:0043226	<1e-4	sll1512	GO:0019538	0.0176
sll0310	GO:0006066	<1e-4	ssr2009	GO:0042455	<1e-4	sll1348	GO:0043167	<1e-4	slr1572	GO:0005575	<1e-4	ssl0739	GO:0006807	0.0176
slr0334	GO:0048523	<1e-4	slr1098	GO:0016053	<1e-4	slr0053	GO:0016818	<1e-4	ssl3549	GO:0044446	<1e-4	slr2125	GO:0006807	0.0176
sll0641	GO:0080090	<1e-4	sll1401	GO:0006811	<1e-4	sll0060	GO:0022857	<1e-4	slr6075	GO:0044424	<1e-4	sll0359	GO:0019538	0.0176
slr1616	GO:0031326	<1e-4	ssl7021	GO:0010468	<1e-4	slr0709	GO:0015077	<1e-4	sll0319	GO:0044422	<1e-4	slr1152	GO:0019538	0.0176
slr6006	GO:0048878	<1e-4	ssr1375	GO:0006811	<1e-4	slr0300	GO:0022892	<1e-4	slr0238	GO:0005575	<1e-4	sll0188	GO:0019538	0.0176
sll1532	GO:0016052	<1e-4	sll0525	GO:0080090	<1e-4	slr6006	GO:0022832	<1e-4	ssr2802	GO:0044422	<1e-4	slr1074	GO:0006807	0.0176
slr0586	GO:0009117	<1e-4	slr7026	GO:0009199	<1e-4	sll0524	GO:0016746	<1e-4	slr1218	GO:0044446	<1e-4	sll1956	GO:0019538	0.0176
slr1702	GO:0043170	<1e-4	sll1247	GO:0044271	<1e-4	slr5073	GO:0022836	<1e-4	slr1170	GO:0044424	<1e-4	sll1660	GO:0006807	0.0176
slr0038	GO:0044281	<1e-4	ssr1528	GO:0060255	<1e-4	slr0702	GO:0043167	<1e-4	sll1389	GO:0044446	<1e-4	sll1352	GO:0006807	0.0176
slr1142	GO:0006721	<1e-4	sll0508	GO:0009141	<1e-4	slr1816	GO:0015077	<1e-4	slr6088	GO:0044424	<1e-4	ssr2009	GO:0006807	0.0176
sll1769	GO:0009893	<1e-4	sll1123	GO:0009132	<1e-4	ssr2439	GO:0022890	<1e-4	sll0563	GO:0044446	<1e-4	slr0815	GO:0006807	0.0176
sll0096	GO:0019222	<1e-4	sll0608	GO:0044260	<1e-4	slr1537	GO:0015293	<1e-4	slr1681	GO:0044464	<1e-4	slr0112	GO:0019538	0.0176
ssr1768	GO:0009892	<1e-4	ssl6061	GO:0009199	<1e-4	sll0394	GO:0022891	<1e-4	slr0755	GO:0005575	<1e-4	slr7024	GO:0019538	0.0176
slr1413	GO:0072522	<1e-4	slr0108	GO:0050801	<1e-4	sll0174	GO:0015075	<1e-4	slr1436	GO:0044444	<1e-4	sll1239	GO:0019538	0.0176
sll0837	GO:0009987	<1e-4	slr0610	GO:0006631	<1e-4	ssl7038	GO:0015267	<1e-4	sll1390	GO:0043226	<1e-4	slr1259	GO:0006807	0.0176
slr0168	GO:0006066	<1e-4	sll1469	GO:0006793	<1e-4	slr0049	GO:0016462	<1e-4	slr1752	GO:0005575	<1e-4	sll0585	GO:0006807	0.0176
ssl8003	GO:0051246	<1e-4	slr1767	GO:0006732	<1e-4	ssr8047	GO:0015293	<1e-4	slr0232	GO:0005575	<1e-4	sll0268	GO:0019538	0.0176
ssr7036	GO:0031326	<1e-4	sll0325	GO:0019222	<1e-4	sll1352	GO:0016817	<1e-4	sll0872	GO:0044424	<1e-4	sll1505	GO:0019538	0.0176
slr0362	GO:0071841	<1e-4	slr1753	GO:0009394	<1e-4	ssr2972	GO:0016741	<1e-4	slr0374	GO:0005575	<1e-4	slr1062	GO:0006807	0.0176
slr1168	GO:0006066	<1e-4	ssl5099	GO:0019751	<1e-4	sll1797	GO:0016462	<1e-4	sll1766	GO:0005575	<1e-4	sll0827	GO:0006807	0.0176
slr0545	GO:0051179	<1e-4	slr5119	GO:0046483	<1e-4	slr1396	GO:0015293	<1e-4	ssr0335	GO:0044424	<1e-4	sll0297	GO:0019538	0.0176
ssl0832	GO:0046164	<1e-4	slr1659	GO:0016052	<1e-4	slr2101	GO:0022892	<1e-4	sll1586	GO:0044425	<1e-4	slr1977	GO:0006807	0.0176
slr1152	GO:0043648	<1e-4	ssl0461	GO:0043549	<1e-4	slr6008	GO:0015267	<1e-4	sll2013	GO:0044446	<1e-4	sll1344	GO:0019538	0.0176
sll1173	GO:0072522	<1e-4	slr0006	GO:0055086	<1e-4	sll5006	GO:0015075	<1e-4	sll0446	GO:0044444	<1e-4	slr1762	GO:0006807	0.0176
slr1107	GO:0009259	<1e-4	sll1834	GO:0009141	<1e-4	sll1092	GO:0015077	<1e-4	sll0588	GO:0044464	<1e-4	sll1021	GO:0006807	0.0176
sll0296	GO:0051179	<1e-4	sll1461	GO:0005996	<1e-4	slr2121	GO:0005342	<1e-4	sll0172	GO:0044464	<1e-4	sll0787	GO:0019538	0.0176
slr1306	GO:0009893	<1e-4	sll1571	GO:0006720	<1e-4	sll1164	GO:0015075	<1e-4	slr0345	GO:0044425	<1e-4	sll1938	GO:0006807	0.0176
ssr3572	GO:0009057	<1e-4	slr0755	GO:0009262	<1e-4	slr0941	GO:0022836	<1e-4	sll0539	GO:0043226	<1e-4	sll0623	GO:0006807	0.0176
slr2018	GO:0009259	<1e-4	sll0253	GO:0009056	<1e-4	slr0049	GO:0060089	<1e-4	sll1241	GO:0044422	<1e-4	sll0225	GO:0006807	0.0176
slr1215	GO:0043549	<1e-4	ssr2711	GO:0006576	<1e-4	sll1638	GO:0003674	<1e-4	slr2080	GO:0005575	<1e-4	sll0843	GO:0019538	0.0176
ssl5008	GO:0042180	<1e-4	sll0241	GO:0006631	<1e-4	sll7066	GO:0015291	<1e-4	slr2049	GO:0044464	<1e-4	sll0188	GO:0006807	0.0176
slr1932	GO:0050794	<1e-4	sll0539	GO:0009124	<1e-4	slr2118	GO:0008171	<1e-4	slr0552	GO:0043226	<1e-4	sll1934	GO:0042626	0.0176
slr1535	GO:0051186	<1e-4	sgl0001	GO:0042180	<1e-4	ssr2554	GO:0022890	<1e-4	slr0144	GO:0005575	<1e-4	sll1166	GO:0042626	0.0176
slr1484	GO:0042455	<1e-4	slr1601	GO:0048519	<1e-4	sll0335	GO:0015077	<1e-4	sll1526	GO:0044464	<1e-4	slr1187	GO:0008233	0.0176
sll0241	GO:0006732	<1e-4	slr1462	GO:0034641	<1e-4	slr0456	GO:0003674	<1e-4	slr0498	GO:0043226	<1e-4	ssr2848	GO:0042626	0.0176
sll5004	GO:0006631	<1e-4	slr6039	GO:0034660	<1e-4	slr0642	GO:0015291	<1e-4	sll1192	GO:0044422	<1e-4	slr0243	GO:0042626	0.0176
ssr1552	GO:0006811	<1e-4	ssr1041	GO:0046128	<1e-4	sll0442	GO:0022803	<1e-4	slr1593	GO:0044444	<1e-4	sll0241	GO:0008233	0.0176
slr1767	GO:0043933	<1e-4	slr0813	GO:0006631	<1e-4	slr6106	GO:0015075	<1e-4	ssr2318	GO:0043226	<1e-4	sll7064	GO:0008233	0.0176
sll0518	GO:0009309	<1e-4	ssr0761	GO:0006811	<1e-4	slr7080	GO:0016741	<1e-4	slr6057	GO:0044425	<1e-4	slr0601	GO:0008233	0.0176
slr1236	GO:0080090	<1e-4	slr0334	GO:0034641	<1e-4	sll0446	GO:0015293	<1e-4	slr2005	GO:0005575	<1e-4	sll0063	GO:0008233	0.0176
slr1025	GO:0044283	<1e-4	slr6068	GO:0006163	<1e-4	sll1696	GO:0015293	<1e-4	ssl0350	GO:0044425	<1e-4	ssr6048	GO:0008233	0.0176

sll0149	GO:0044237	<1e-4	slr2012	GO:0045184	<1e-4	slr0588	GO:0022832	<1e-4	sll1476	GO:0044422	<1e-4	sll0727	GO:0008233	0.0176
sll0274	GO:0009144	<1e-4	sll1373	GO:0046164	<1e-4	sll0369	GO:0016462	<1e-4	slr0348	GO:0044464	<1e-4	sll0857	GO:0008233	0.0176
ssl0109	GO:0009057	<1e-4	sll0786	GO:0080090	<1e-4	sll0864	GO:0016818	<1e-4	slr5101	GO:0005575	<1e-4	slr0545	GO:0042626	0.0176
slr1788	GO:0009199	<1e-4	sll0191	GO:0043412	<1e-4	sll0449	GO:0016741	<1e-4	slr1071	GO:0044424	<1e-4	sll1130	GO:0008233	0.0176
sll1217	GO:0006220	<1e-4	slr1218	GO:0048518	<1e-4	sll0606	GO:0008171	<1e-4	ssr6002	GO:0044422	<1e-4	ssr0536	GO:0042626	0.0176
sll8004	GO:0042451	<1e-4	sll0381	GO:0051171	<1e-4	sll1442	GO:0043167	<1e-4	sll0265	GO:0043226	<1e-4	ssr1766	GO:0008233	0.0176
slr0872	GO:0060255	<1e-4	sll2011	GO:0009124	<1e-4	slr0888	GO:0016741	<1e-4	ssr1528	GO:0044446	<1e-4	slr0355	GO:0008233	0.0176
slr0810	GO:0071842	<1e-4	sll1089	GO:0009056	<1e-4	sll0068	GO:0008171	<1e-4	slr0489	GO:0044446	<1e-4	sll0994	GO:0008233	0.0176
sll1192	GO:0009161	<1e-4	slr6028	GO:0009117	<1e-4	sll1052	GO:0015291	<1e-4	slr1206	GO:0044464	<1e-4	sll0266	GO:0008233	0.0176
slr6094	GO:0034660	<1e-4	ssr7093	GO:0055082	<1e-4	ssl2595	GO:0016462	<1e-4	sll0744	GO:0044424	<1e-4	sll8004	GO:0008233	0.0176
slr0964	GO:0052188	<1e-4	sll1606	GO:0048518	<1e-4	sll0625	GO:0016746	<1e-4	sll1350	GO:0043226	<1e-4	slr6073	GO:0008233	0.0176
sll8035	GO:0034654	<1e-4	slr0337	GO:0042451	<1e-4	slr1397	GO:0022803	<1e-4	ssr6019	GO:0005575	<1e-4	sll6054	GO:0008233	0.0176
slr0699	GO:0022607	<1e-4	sll1151	GO:0009144	<1e-4	ssr2060	GO:0015267	<1e-4	ssl2138	GO:0005575	<1e-4	sll5047	GO:0008233	0.0176
slr1143	GO:0072528	<1e-4	slr1813	GO:0044255	<1e-4	sll1926	GO:0022803	<1e-4	slr5053	GO:0044446	<1e-4	sll1651	GO:0008233	0.0176
ssl1263	GO:0009987	<1e-4	sll2013	GO:0016053	<1e-4	ssl2064	GO:0015075	<1e-4	slr1704	GO:0044425	<1e-4	sll1186	GO:0042626	0.0176
sll1160	GO:0044281	<1e-4	ssr1528	GO:0006163	<1e-4	slr0601	GO:0015293	<1e-4	sll0595	GO:0044422	<1e-4	sll2015	GO:0008233	0.0176
sll1532	GO:0006721	<1e-4	sll0861	GO:0044281	<1e-4	ssr2803	GO:0022832	<1e-4	slr6091	GO:0044444	<1e-4	slr0092	GO:0008233	0.0176
slr1425	GO:0006753	<1e-4	sll1486	GO:0006163	<1e-4	sll0822	GO:0022891	<1e-4	slr0948	GO:0044422	<1e-4	sll1400	GO:0008233	0.0176
ssl7022	GO:0006576	<1e-4	ssr5120	GO:0009262	<1e-4	slr1970	GO:0043167	<1e-4	sll0543	GO:0044425	<1e-4	sll5034	GO:0008233	0.0176
slr1914	GO:0043170	<1e-4	sll1651	GO:0046394	<1e-4	sll0590	GO:0015267	<1e-4	ssl0294	GO:0005575	<1e-4	sll1601	GO:0042626	0.0176
sll1239	GO:0044283	<1e-4	slr1932	GO:0044271	<1e-4	slr0592	GO:0042623	<1e-4	slr1927	GO:0005575	<1e-4	slr7023	GO:0008233	0.0176
sll0280	GO:0019752	<1e-4	slr1338	GO:0050789	<1e-4	slr1288	GO:0022890	<1e-4	slr1070	GO:0044422	<1e-4	sll1252	GO:0008233	0.0176
slr0586	GO:0016054	<1e-4	slr0458	GO:0016054	<1e-4	sll1738	GO:0015293	<1e-4	slr6066	GO:0005575	<1e-4	sll1696	GO:0008233	0.0176
slr1301	GO:0051234	<1e-4	slr0575	GO:0051188	<1e-4	ssr6048	GO:0042623	<1e-4	slr1573	GO:0005575	<1e-4	sll1414	GO:0008233	0.0176
slr1178	GO:0016052	<1e-4	sll1131	GO:0009123	<1e-4	sll1052	GO:0022892	<1e-4	ssr2962	GO:0044464	<1e-4	ssr6026	GO:0008233	0.0176
sll0176	GO:0009892	<1e-4	sll1830	GO:0006721	<1e-4	slr5119	GO:0015405	<1e-4	slr1737	GO:0044422	<1e-4	sll1764	GO:0008233	0.0176
slr1397	GO:0048522	<1e-4	slr0337	GO:0009892	<1e-4	sll1388	GO:0015267	<1e-4	sll0481	GO:0005575	<1e-4	slr1195	GO:0008233	0.0176
sll1233	GO:0080090	<1e-4	sll0479	GO:0080090	<1e-4	slr0964	GO:0022890	<1e-4	slr1407	GO:0005575	<1e-4	slr7096	GO:0008233	0.0176
sll5004	GO:0006066	<1e-4	slr0065	GO:0044260	<1e-4	slr6065	GO:0022891	<1e-4	sll0625	GO:0043226	<1e-4	sll8002	GO:0008233	0.0176
slr1577	GO:0006576	<1e-4	slr1505	GO:0052111	<1e-4	ssl0350	GO:0008171	<1e-4	sll0615	GO:0044422	<1e-4	slr1814	GO:0008233	0.0176
slr5119	GO:0009056	<1e-4	sll0982	GO:0051179	<1e-4	sll0930	GO:0015267	<1e-4	slr0954	GO:0044446	<1e-4	slr1535	GO:0008233	0.0176
ssl1520	GO:0046128	<1e-4	slr1215	GO:0048878	<1e-4	ssl3451	GO:0016818	<1e-4	slr1143	GO:0005575	<1e-4	slr1342	GO:0008233	0.0176
slr1066	GO:0044260	<1e-4	sll1372	GO:0009893	<1e-4	slr0609	GO:0022832	<1e-4	ssl5015	GO:0044446	<1e-4	sll0996	GO:0042626	0.0176
slr5087	GO:0019362	<1e-4	ssl7022	GO:0048519	<1e-4	slr0589	GO:0015075	<1e-4	sll1582	GO:0044425	<1e-4	sll1218	GO:0042626	0.0176
sll0659	GO:0009124	<1e-4	sll1563	GO:0042455	<1e-4	slr0582	GO:0015267	<1e-4	sll1225	GO:0043226	<1e-4	slr0870	GO:0008233	0.0176
slr0605	GO:0009161	<1e-4	sll1092	GO:0006576	<1e-4	slr1262	GO:0015399	<1e-4	sll1906	GO:0044424	<1e-4	sll0630	GO:0008233	0.0176
ssr1528	GO:0046164	<1e-4	ssl2733	GO:0018130	<1e-4	slr0668	GO:0060089	<1e-4	ssr0332	GO:0043226	<1e-4	slr1407	GO:0008233	0.0176
slr6063	GO:0051171	<1e-4	sll8001	GO:0044260	<1e-4	slr1471	GO:0046873	<1e-4	slr0680	GO:0044446	<1e-4	sll0802	GO:0008233	0.0176
slr0291	GO:0009059	<1e-4	sll0238	GO:0009057	<1e-4	slr0514	GO:0008171	<1e-4	sll0788	GO:0044424	<1e-4	slr1674	GO:0042626	0.0176
sll0749	GO:0009259	<1e-4	ssl1972	GO:0006091	<1e-4	sll0505	GO:0016746	<1e-4	sll1162	GO:0044425	<1e-4	slr1566	GO:0008233	0.0176
sll1273	GO:0009987	<1e-4	slr0362	GO:0031326	<1e-4	sll0281	GO:0016462	<1e-4	sll1135	GO:0005575	<1e-4	sll0481	GO:0008233	0.0176
ssl2148	GO:0006733	<1e-4	slr6014	GO:0044271	<1e-4	slr5018	GO:0015399	<1e-4	slr0980	GO:0044464	<1e-4	sll1884	GO:0008233	0.0176
ssr0536	GO:0009889	<1e-4	slr0208	GO:0006091	<1e-4	slr6004	GO:0043167	<1e-4	sll0428	GO:0005575	<1e-4	slr7083	GO:0008233	0.0176
slr0964	GO:0046907	<1e-4	sll0661	GO:0009132	<1e-4	sll0608	GO:0015077	<1e-4	sll0167	GO:0044446	<1e-4	slr1956	GO:0008233	0.0176
sll7070	GO:0060255	<1e-4	sll0886	GO:0022411	<1e-4	slr1519	GO:0060089	<1e-4	ssl7046	GO:0044446	<1e-4	slr7081	GO:0008233	0.0176
sll7067	GO:0022411	<1e-4	slr0262	GO:0090304	<1e-4	sll0858	GO:0016741	<1e-4	sll0547	GO:0005575	<1e-4	sll0096	GO:0042626	0.0176
slr0479	GO:0042451	<1e-4	slr6008	GO:0009144	<1e-4	sll0410	GO:0022803	<1e-4	slr6039	GO:0044422	<1e-4	sll1348	GO:0008233	0.0176
slr1668	GO:0009124	<1e-4	sll0558	GO:0009126	<1e-4	sll0296	GO:0015077	<1e-4	slr1614	GO:0044422	<1e-4	sll0913	GO:0008233	0.0176
sll0198	GO:0072521	<1e-4	sll0274	GO:0034641	<1e-4	slr0609	GO:0003674	<1e-4	slr1624	GO:0044422	<1e-4	slr5119	GO:0008233	0.0176
slr1807	GO:0019222	<1e-4	slr1033	GO:0006163	<1e-4	sll0174	GO:0015267	<1e-4	sll1396	GO:0043226	<1e-4	sll0282	GO:0008233	0.0176
ssr7084	GO:0048522	<1e-4	slr1161	GO:0009144	<1e-4	sll0788	GO:0015291	<1e-4	sll1025	GO:0044464	<1e-4	slr1505	GO:0042626	0.0176
ssl2781	GO:0072521	<1e-4	sll0451	GO:0009987	<1e-4	sll0659	GO:0015291	<1e-4	ssl1972	GO:0044424	<1e-4	sll1880	GO:0008233	0.0176
ssl2595	GO:0009150	<1e-4	slr1396	GO:0043549	<1e-4	slr0232	GO:0060089	<1e-4	slr1546	GO:0005575	<1e-4	slr7059	GO:0008233	0.0176
sll0361	GO:0044106	<1e-4	slr1240	GO:0071496	<1e-4	slr1419	GO:0022836	<1e-4	sll1643	GO:0005575	<1e-4	slr1600	GO:0008233	0.0176

slr1070	GO:0051188	<1e-4	slr2103	GO:0009142	<1e-4	slr0980	GO:0015075	<1e-4	slr1957	GO:0044464	<1e-4	ssr1499	GO:0008233	0.0176
ssl5099	GO:0006576	<1e-4	slr6014	GO:0042451	<1e-4	slr1260	GO:0015077	<1e-4	ssl3692	GO:0044424	<1e-4	sll1433	GO:0042626	0.0176
slr6008	GO:0006091	<1e-4	sll1414	GO:0022607	<1e-4	sll0354	GO:0015405	<1e-4	sll0863	GO:0044464	<1e-4	ssl2717	GO:0008233	0.0176
sll0676	GO:0051246	<1e-4	slr5024	GO:0016053	<1e-4	sll0905	GO:0022832	<1e-4	ssl8028	GO:0044425	<1e-4	slr1150	GO:0008233	0.0176
slr1815	GO:0034641	<1e-4	sll8001	GO:0042180	<1e-4	sll0905	GO:0015293	<1e-4	slr1340	GO:0044424	<1e-4	sll0008	GO:0008233	0.0176
slr1468	GO:0009161	<1e-4	slr1066	GO:0006091	<1e-4	sll0984	GO:0015291	<1e-4	slr1767	GO:0044446	<1e-4	ssl0312	GO:0008233	0.0176
slr7025	GO:0043549	<1e-4	ssr2067	GO:0034654	<1e-4	ssr6026	GO:0016817	<1e-4	slr2103	GO:0044444	<1e-4	ssl2148	GO:0008233	0.0176
ssl2920	GO:0006576	<1e-4	slr1128	GO:0051188	<1e-4	slr1301	GO:0043492	<1e-4	slr7058	GO:0044464	<1e-4	sll0174	GO:0008233	0.0176
slr0366	GO:0072527	<1e-4	ssr8013	GO:0034654	<1e-4	sll0284	GO:0015399	<1e-4	ssr1425	GO:0044444	<1e-4	sll1961	GO:0008233	0.0176
sll1009	GO:0009165	<1e-4	ssr1951	GO:0022607	<1e-4	sll1769	GO:0016817	<1e-4	sll7089	GO:0044446	<1e-4	slr7101	GO:0008233	0.0176
sll1751	GO:0042451	<1e-4	slr0702	GO:0042451	<1e-4	slr0318	GO:0022836	<1e-4	slr0039	GO:0005575	<1e-4	slr0869	GO:0008233	0.0176
sll7066	GO:0006721	<1e-4	ssl5100	GO:0046483	<1e-4	sll0230	GO:0043167	<1e-4	slr0184	GO:0044425	<1e-4	ssr2554	GO:0008233	0.0176
sll7033	GO:0050794	<1e-4	sll1401	GO:0051171	<1e-4	sll7089	GO:0015291	<1e-4	slr0751	GO:0044444	<1e-4	ssr3341	GO:0008233	0.0176
sll1738	GO:0009892	<1e-4	sll1155	GO:0010556	<1e-4	sll2013	GO:0022838	<1e-4	slr0483	GO:0044444	<1e-4	ssl2009	GO:0008233	0.0176
slr0303	GO:0009199	<1e-4	ssr5020	GO:0090304	<1e-4	sll5097	GO:0043167	<1e-4	slr7092	GO:0044446	<1e-4	ssl2245	GO:0008233	0.0176
sll0298	GO:0044275	<1e-4	sll1634	GO:0016054	<1e-4	slr1638	GO:0022890	<1e-4	sll1219	GO:0043226	<1e-4	slr0489	GO:0008233	0.0176
slr0729	GO:0051186	<1e-4	ssl8008	GO:0009394	<1e-4	slr0812	GO:0022891	<1e-4	sll0384	GO:0005575	<1e-4	slr0503	GO:0042626	0.0176
sll0325	GO:0043436	<1e-4	slr0602	GO:0008150	<1e-4	slr2084	GO:0015291	<1e-4	sll0328	GO:0044446	<1e-4	slr1397	GO:0008233	0.0176
slr1778	GO:0009259	<1e-4	sll1691	GO:0034641	<1e-4	ssl0788	GO:0015075	<1e-4	slr1957	GO:0044446	<1e-4	sll0864	GO:0042626	0.0176
sll0172	GO:0043412	<1e-4	slr0238	GO:0043549	<1e-4	slr0013	GO:0016818	<1e-4	ssl0294	GO:0044444	<1e-4	sll1571	GO:0008233	0.0176
sll1350	GO:0006793	<1e-4	slr1081	GO:0019222	<1e-4	sll0775	GO:0016817	<1e-4	slr0145	GO:0044424	<1e-4	ssl1972	GO:0008233	0.0176
sll0815	GO:0050896	<1e-4	sll0451	GO:0072527	<1e-4	slr1951	GO:0043167	<1e-4	slr1081	GO:0044444	<1e-4	slr1468	GO:0008233	0.0176
slr6029	GO:0043170	<1e-4	sll0853	GO:0043933	<1e-4	sll0189	GO:0022832	<1e-4	sll0008	GO:0044425	<1e-4	slr1194	GO:0042626	0.0176
slr0232	GO:0019637	<1e-4	sll0676	GO:0046164	<1e-4	slr2103	GO:0016818	<1e-4	ssl0312	GO:0005575	<1e-4	sll0443	GO:0008233	0.0176
slr1215	GO:0009987	<1e-4	sll0858	GO:0072521	<1e-4	slr0588	GO:0016817	<1e-4	sll1135	GO:0044444	<1e-4	sll0048	GO:0008233	0.0176
slr0771	GO:0009199	<1e-4	slr6014	GO:0051716	<1e-4	slr0818	GO:0015293	<1e-4	slr7073	GO:0043226	<1e-4	slr1162	GO:0008233	0.0176
slr7012	GO:0019222	<1e-4	slr1087	GO:0043549	<1e-4	slr0596	GO:0022832	<1e-4	slr0890	GO:0044425	<1e-4	ssl2920	GO:0008233	0.0176
sll1036	GO:0072527	<1e-4	ssr6083	GO:0051171	<1e-4	slr1033	GO:0042623	<1e-4	sll0864	GO:0043226	<1e-4	slr1306	GO:0008233	0.0176
sll1949	GO:0016052	<1e-4	sll1735	GO:0042455	<1e-4	ssl3379	GO:0015399	<1e-4	sll0815	GO:0043226	<1e-4	sll0498	GO:0008233	0.0176
sll0898	GO:0065007	<1e-4	sll7028	GO:0009308	<1e-4	slr1179	GO:0060089	<1e-4	sll0208	GO:0005575	<1e-4	slr7098	GO:0008233	0.0176
slr0270	GO:0009059	<1e-4	slr1505	GO:0052188	<1e-4	sll1106	GO:0015399	<1e-4	sll1609	GO:0044464	<1e-4	slr0157	GO:0042626	0.0176
ssr2912	GO:0031640	<1e-4	sll1714	GO:0009059	<1e-4	sll0085	GO:0015293	<1e-4	slr0941	GO:0005575	<1e-4	slr1533	GO:0042626	0.0176
sll0446	GO:0006631	<1e-4	slr7073	GO:0009892	<1e-4	sll0742	GO:0022832	<1e-4	slr1128	GO:0043226	<1e-4	slr1391	GO:0042626	0.0176
sll1613	GO:0072528	<1e-4	sll1835	GO:0043170	<1e-4	sll1510	GO:0015293	<1e-4	sll1659	GO:0044444	<1e-4	slr0065	GO:0008233	0.0176
ssl1004	GO:0071842	<1e-4	ssl0832	GO:0006066	<1e-4	ssr8047	GO:0022891	<1e-4	slr1210	GO:0044425	<1e-4	slr1378	GO:0008233	0.0176
slr7102	GO:0044003	<1e-4	slr0199	GO:0006811	<1e-4	ssr0332	GO:0022838	<1e-4	ssl8028	GO:0005575	<1e-4	ssr0332	GO:0008233	0.0176
sll1531	GO:0046394	<1e-4	sll0815	GO:0009987	<1e-4	ssr1256	GO:0016817	<1e-4	slr2111	GO:0005575	<1e-4	slr1619	GO:0008233	0.0176
ssr6086	GO:0044271	<1e-4	slr0271	GO:0051234	<1e-4	slr2000	GO:0022892	<1e-4	ssr6062	GO:0044425	<1e-4	ssr5120	GO:0008233	0.0176
ssr8047	GO:0044003	<1e-4	slr1648	GO:0051818	<1e-4	sll5034	GO:0008171	<1e-4	slr0590	GO:0005575	<1e-4	sll1942	GO:0042626	0.0176
sll7086	GO:0033013	<1e-4	slr1169	GO:0009150	<1e-4	ssl2471	GO:0005342	<1e-4	slr5077	GO:0044425	<1e-4	ssl1520	GO:0008233	0.0176
ssr1256	GO:0044003	<1e-4	slr5016	GO:0051234	<1e-4	ssr3154	GO:0005342	<1e-4	slr0050	GO:0044425	<1e-4	sll0872	GO:0008233	0.0176
slr1726	GO:0042180	<1e-4	ssr1256	GO:0080090	<1e-4	sll0242	GO:0060089	<1e-4	sll7031	GO:0044424	<1e-4	sll8007	GO:0008233	0.0176
sll1530	GO:0034641	<1e-4	slr0476	GO:0009123	<1e-4	sll0558	GO:0003674	<1e-4	sll1954	GO:0043226	<1e-4	sll1062	GO:0008233	0.0176
slr1513	GO:0044248	<1e-4	sll1072	GO:0009308	<1e-4	ssr2201	GO:0008171	<1e-4	sll0493	GO:0043226	<1e-4	sll5032	GO:0008233	0.0176
slr1847	GO:0034654	<1e-4	slr0145	GO:0019752	<1e-4	sll1396	GO:0015291	<1e-4	slr1241	GO:0044464	<1e-4	slr1799	GO:0042626	0.0176
ssl2996	GO:0050896	<1e-4	slr1778	GO:0042455	<1e-4	slr0313	GO:0015293	<1e-4	slr1956	GO:0044424	<1e-4	sll1109	GO:0008233	0.0176
slr1611	GO:0006720	<1e-4	ssl2971	GO:0019222	<1e-4	ssr1766	GO:0022836	<1e-4	slr0386	GO:0005575	<1e-4	sll0863	GO:0008233	0.0176
sll0188	GO:0006082	<1e-4	slr7102	GO:0044275	<1e-4	sll1155	GO:0022832	<1e-4	sll0024	GO:0043226	<1e-4	slr1097	GO:0008233	0.0176
sll0208	GO:0050801	<1e-4	sll5069	GO:0008150	<1e-4	slr1406	GO:0043167	<1e-4	sll5034	GO:0044425	<1e-4	ssr7036	GO:0008233	0.0176
slr1441	GO:0051171	<1e-4	sll0994	GO:0031640	<1e-4	sll0839	GO:0022838	<1e-4	slr2010	GO:0044422	<1e-4	sll1769	GO:0008233	0.0176
slr1690	GO:0046907	<1e-4	sll2013	GO:0065007	<1e-4	slr1391	GO:0060089	<1e-4	sll7064	GO:0005575	<1e-4	slr1464	GO:0008233	0.0176
slr1396	GO:0009057	<1e-4	sll0871	GO:0006576	<1e-4	slr0238	GO:0016462	<1e-4	slr0195	GO:0005575	<1e-4	sll1396	GO:0008233	0.0176
sll0847	GO:0043549	<1e-4	sll0996	GO:0071496	<1e-4	slr0345	GO:0015077	<1e-4	sll0274	GO:0005575	<1e-4	sll1455	GO:0042626	0.0176
sll8032	GO:0006091	<1e-4	slr1958	GO:0051186	<1e-4	sll0062	GO:0015267	<1e-4	sll7067	GO:0044425	<1e-4	sll1766	GO:0008233	0.0176

sll1222	GO:0016053	<1e-4	slr0423	GO:0072521	<1e-4	slr0148	GO:0022832	<1e-4	slr1173	GO:0005575	<1e-4	slr1116	GO:0008233	0.0176
slr2103	GO:0019362	<1e-4	ssl0788	GO:0065007	<1e-4	slr0981	GO:0016741	<1e-4	ssr0759	GO:0044424	<1e-4	slr0076	GO:0042626	0.0176
sll1476	GO:0016052	<1e-4	sll0925	GO:0009262	<1e-4	sll1925	GO:0043167	<1e-4	sll0447	GO:0044444	<1e-4	sll0854	GO:0042626	0.0176
slr1800	GO:0044283	<1e-4	slr1534	GO:0046907	<1e-4	sll1123	GO:0016746	<1e-4	slr1603	GO:0005575	<1e-4	sll0846	GO:0008233	0.0176
slr1365	GO:0009057	<1e-4	slr1544	GO:0009308	<1e-4	slr1074	GO:0015399	<1e-4	slr0742	GO:0005575	<1e-4	sll0181	GO:0008233	0.0176
slr0589	GO:0050794	<1e-4	sll0910	GO:0034660	<1e-4	ssr1155	GO:0022891	<1e-4	sll0862	GO:0044444	<1e-4	slr0551	GO:0042626	0.0176
sll0563	GO:0050794	<1e-4	sll0761	GO:0065007	<1e-4	sll1630	GO:0005342	<1e-4	slr1753	GO:0005575	<1e-4	sll1232	GO:0008233	0.0176
slr1699	GO:0071840	<1e-4	sll0785	GO:0043412	<1e-4	sll1232	GO:0015077	<1e-4	sll0007	GO:0044446	<1e-4	sll5130	GO:0008233	0.0176
sll0414	GO:0009161	<1e-4	slr6090	GO:0009259	<1e-4	sll1495	GO:0043492	<1e-4	slr0455	GO:0044444	<1e-4	ssl3692	GO:0042626	0.0176
slr0870	GO:0009889	<1e-4	ssr2060	GO:0009132	<1e-4	sll0263	GO:0015291	<1e-4	slr5119	GO:0044446	<1e-4	sll0301	GO:0042626	0.0176
slr0360	GO:0009309	<1e-4	sll0071	GO:0018130	<1e-4	slr6044	GO:0015267	<1e-4	slr1932	GO:0005575	<1e-4	sll1485	GO:0008233	0.0176
ssr5020	GO:0005996	<1e-4	sll1691	GO:0044281	<1e-4	slr0147	GO:0022890	<1e-4	sll0787	GO:0005575	<1e-4	slr5023	GO:0008233	0.0176
slr1676	GO:0031323	<1e-4	sll1306	GO:0006733	<1e-4	sll0658	GO:0022892	<1e-4	sll1632	GO:0044446	<1e-4	sll1461	GO:0008233	0.0176
slr1865	GO:0072524	<1e-4	slr1450	GO:0044106	<1e-4	slr0606	GO:0043167	<1e-4	sll0424	GO:0005575	<1e-4	slr1419	GO:0008233	0.0176
ssr6078	GO:0044255	<1e-4	sll0442	GO:0051246	<1e-4	slr0468	GO:0015291	<1e-4	sll8040	GO:0044446	<1e-4	slr1484	GO:0008233	0.0176
sll1634	GO:0034654	<1e-4	slr2011	GO:0051179	<1e-4	sll0910	GO:0015293	<1e-4	sll0984	GO:0044422	<1e-4	slr7011	GO:0008233	0.0176
sll0174	GO:0048518	<1e-4	slr1034	GO:0048519	<1e-4	ssr3410	GO:0016462	<1e-4	slr1353	GO:0044444	<1e-4	sll0487	GO:0008233	0.0176
slr2025	GO:0044003	<1e-4	sll6054	GO:0001932	<1e-4	ssl0294	GO:0016462	<1e-4	slr1923	GO:0005575	<1e-4	slr1263	GO:0008233	0.0176
sll1203	GO:0008150	<1e-4	sll0735	GO:0048522	<1e-4	slr1464	GO:0016462	<1e-4	sll7033	GO:0005575	<1e-4	slr0569	GO:0008233	0.0176
slr6015	GO:0009117	<1e-4	ssr1766	GO:0009059	<1e-4	slr1590	GO:0015075	<1e-4	slr0503	GO:0044444	<1e-4	slr0751	GO:0008233	0.0176
slr2003	GO:0009057	<1e-4	sll1486	GO:0009059	<1e-4	sll1265	GO:0003674	<1e-4	sll0606	GO:0043226	<1e-4	slr1659	GO:0042626	0.0176
slr1944	GO:0090304	<1e-4	ssl0483	GO:0046483	<1e-4	slr1127	GO:0022836	<1e-4	slr1464	GO:0044446	<1e-4	slr1398	GO:0042626	0.0176
ssl5027	GO:0009161	<1e-4	sll0410	GO:0016053	<1e-4	ssl3383	GO:0016741	<1e-4	sll0263	GO:0043226	<1e-4	sll1265	GO:0008233	0.0176
slr1398	GO:0052188	<1e-4	slr0333	GO:0009199	<1e-4	ssl6092	GO:0016818	<1e-4	ssl5045	GO:0044424	<1e-4	sll1060	GO:0042626	0.0176
ssr1558	GO:0046128	<1e-4	slr1195	GO:0034654	<1e-4	slr1704	GO:0005342	<1e-4	sll7028	GO:0044464	<1e-4	slr0168	GO:0008233	0.0176
slr6071	GO:0022607	<1e-4	sll0487	GO:0006720	<1e-4	sll0181	GO:0016462	<1e-4	slr1116	GO:0044444	<1e-4	sll0444	GO:0008233	0.0176
slr1774	GO:0044003	<1e-4	sll1380	GO:0009161	<1e-4	sll0702	GO:0003674	<1e-4	slr1023	GO:0043226	<1e-4	sgl0001	GO:0008233	0.0176
slr0769	GO:0009309	<1e-4	slr1572	GO:0009057	<1e-4	sll5026	GO:0015291	<1e-4	sll1106	GO:0044422	<1e-4	slr0818	GO:0008168	0.0174
slr0789	GO:0009123	<1e-4	slr1143	GO:0033013	<1e-4	sll1267	GO:0016462	<1e-4	sll0384	GO:0044422	<1e-4	sll1222	GO:0008168	0.0174
ssr6024	GO:0048878	<1e-4	sll0265	GO:0071840	<1e-4	ssl7038	GO:0042623	<1e-4	sll1188	GO:0044446	<1e-4	slr1773	GO:0008168	0.0174
sll1526	GO:0042455	<1e-4	slr1690	GO:0071496	<1e-4	sll0564	GO:0022857	<1e-4	sll1054	GO:0044422	<1e-4	slr1591	GO:0008168	0.0174
sll1072	GO:0005996	<1e-4	ssr7079	GO:0051186	<1e-4	sll1956	GO:0022832	<1e-4	slr0109	GO:0044444	<1e-4	sll1527	GO:0005975	0.0173
slr2118	GO:0048518	<1e-4	sll1054	GO:0019637	<1e-4	sll1532	GO:0043167	<1e-4	sll0249	GO:0005575	<1e-4	sll0350	GO:0005975	0.0173
sll1873	GO:0009309	<1e-4	sll1109	GO:0019222	<1e-4	sll1486	GO:0060089	<1e-4	slr1563	GO:0044425	<1e-4	ssr3571	GO:0016874	0.0173
ssl0352	GO:0071496	<1e-4	slr0208	GO:0009056	<1e-4	slr0845	GO:0005342	<1e-4	slr2012	GO:0044425	<1e-4	slr1230	GO:0016874	0.0173
sll7065	GO:0009124	<1e-4	sll1135	GO:0033013	<1e-4	slr0888	GO:0016817	<1e-4	slr0607	GO:0043226	<1e-4	sll0456	GO:0016874	0.0173
sll1505	GO:0043933	<1e-4	sll0572	GO:0006721	<1e-4	sll1530	GO:0005342	<1e-4	sll1429	GO:0044425	<1e-4	sll1835	GO:0016874	0.0173
slr1263	GO:0080090	<1e-4	sll0785	GO:0034660	<1e-4	slr2115	GO:0043167	<1e-4	sll1390	GO:0005575	<1e-4	sll1656	GO:0016874	0.0173
sll1510	GO:0052111	<1e-4	ssr3122	GO:0019362	<1e-4	sll1775	GO:0016818	<1e-4	sll0558	GO:0005575	<1e-4	slr0872	GO:0016874	0.0173
sll0688	GO:0055082	<1e-4	sll1071	GO:0019751	<1e-4	slr1788	GO:0016462	<1e-4	slr1240	GO:0044444	<1e-4	sll1608	GO:0016874	0.0173
ssr1951	GO:0006631	<1e-4	slr1956	GO:0009132	<1e-4	slr1419	GO:0043167	<1e-4	slr7011	GO:0044446	<1e-4	slr1084	GO:0016874	0.0173
sll1130	GO:0065007	<1e-4	sll1681	GO:0009893	<1e-4	sll0625	GO:0022892	<1e-4	ssr3532	GO:0044464	<1e-4	ssr2755	GO:0016874	0.0173
slr6087	GO:0044248	<1e-4	sll0479	GO:0006220	<1e-4	slr0656	GO:0022892	<1e-4	sll0982	GO:0044425	<1e-4	slr1593	GO:0016874	0.0173
sll1251	GO:0072527	<1e-4	ssl1690	GO:0050896	<1e-4	slr1557	GO:0016462	<1e-4	slr1342	GO:0044424	<1e-4	sll1630	GO:0016874	0.0173
sll0564	GO:0046164	<1e-4	ssr2711	GO:0006793	<1e-4	slr1177	GO:0015399	<1e-4	slr0431	GO:0044444	<1e-4	slr0103	GO:0016874	0.0173
sll7033	GO:0051179	<1e-4	sll1348	GO:0009161	<1e-4	sll1609	GO:0015291	<1e-4	sll1225	GO:0044425	<1e-4	slr1778	GO:0016874	0.0173
ssl7022	GO:0006793	<1e-4	sll0676	GO:0051347	<1e-4	sll5061	GO:0015405	<1e-4	ssl1520	GO:0005575	<1e-4	slr0294	GO:0016874	0.0173
sll1424	GO:0009057	<1e-4	sll8032	GO:0009309	<1e-4	ssl6035	GO:0043167	<1e-4	slr6044	GO:0044422	<1e-4	sll0177	GO:0016874	0.0173
ssr7036	GO:0006753	<1e-4	slr1677	GO:0071842	<1e-4	sll0372	GO:0003674	<1e-4	sll1934	GO:0043226	<1e-4	ssl0352	GO:0016874	0.0173
slr5112	GO:0019222	<1e-4	sll1956	GO:0031640	<1e-4	slr1627	GO:0015405	<1e-4	sll0183	GO:0044424	<1e-4	slr1932	GO:0016874	0.0173
sll0864	GO:0009124	<1e-4	slr0815	GO:0043648	<1e-4	sll1995	GO:0022890	<1e-4	slr0313	GO:0005575	<1e-4	sll1658	GO:0016874	0.0173
slr1875	GO:0050801	<1e-4	sll0319	GO:0008610	<1e-4	sll1486	GO:0008171	<1e-4	sll1442	GO:0043226	<1e-4	ssl0242	GO:0016874	0.0173
sll1068	GO:0009309	<1e-4	sll7033	GO:0044282	<1e-4	sll0044	GO:0060089	<1e-4	sll1880	GO:0043226	<1e-4	slr1073	GO:0016874	0.0173
sll5047	GO:0051818	<1e-4	sll0442	GO:0019752	<1e-4	slr1768	GO:0022857	<1e-4	sll7086	GO:0044444	<1e-4	slr0144	GO:0016874	0.0173

slr0553	GO:0051186	<1e-4	sll1531	GO:0050896	<1e-4	ssl1972	GO:0043167	<1e-4	slr0594	GO:0044422	<1e-4	sll1965	GO:0016874	0.0173
ssl7022	GO:0051186	<1e-4	slr1852	GO:0044282	<1e-4	slr1544	GO:0022836	<1e-4	sll0160	GO:0005575	<1e-4	sll0314	GO:0016874	0.0173
ssl3383	GO:0006733	<1e-4	slr0907	GO:0043648	<1e-4	ssr3467	GO:0003674	<1e-4	sll8019	GO:0044446	<1e-4	slr0770	GO:0016874	0.0173
slr1917	GO:0044248	<1e-4	sll1586	GO:0034654	<1e-4	sll1751	GO:0005342	<1e-4	slr1652	GO:0044425	<1e-4	slr0431	GO:0016874	0.0173
sll1009	GO:0065007	<1e-4	sll1086	GO:0052188	<1e-4	slr0408	GO:0016462	<1e-4	sll0932	GO:0043226	<1e-4	sll1761	GO:0016874	0.0173
slr0888	GO:0072527	<1e-4	slr1117	GO:0050794	<1e-4	slr1033	GO:0016818	<1e-4	sll1350	GO:0044425	<1e-4	slr0723	GO:0016874	0.0173
sll1552	GO:0009117	<1e-4	sll0827	GO:0009123	<1e-4	slr1977	GO:0008171	<1e-4	slr1566	GO:0044425	<1e-4	sll0085	GO:0016874	0.0173
ssr2803	GO:0051188	<1e-4	sll5109	GO:0006066	<1e-4	sll1880	GO:0015405	<1e-4	slr0142	GO:0005575	<1e-4	ssr2615	GO:0016874	0.0173
slr2027	GO:0044248	<1e-4	slr1168	GO:0009893	<1e-4	ssr6026	GO:0016462	<1e-4	ssl0788	GO:0044464	<1e-4	sll0237	GO:0016874	0.0173
sll5046	GO:0034641	<1e-4	slr0168	GO:0019751	<1e-4	slr1276	GO:0043167	<1e-4	slr0374	GO:0044422	<1e-4	sll1006	GO:0016874	0.0173
ssr0332	GO:0019219	<1e-4	slr5119	GO:0009987	<1e-4	slr1062	GO:0005342	<1e-4	slr1307	GO:0044446	<1e-4	slr0941	GO:0016874	0.0173
sll1926	GO:0072521	<1e-4	slr1431	GO:0009262	<1e-4	sll1765	GO:0022890	<1e-4	slr6007	GO:0005575	<1e-4	slr0023	GO:0016874	0.0173
slr6088	GO:0006163	<1e-4	sll5067	GO:0048518	<1e-4	slr1188	GO:0015291	<1e-4	ssr2009	GO:0044424	<1e-4	sll1442	GO:0016874	0.0173
sll0595	GO:0009309	<1e-4	slr0981	GO:0048522	<1e-4	sll0762	GO:0015291	<1e-4	ssr2551	GO:0005575	<1e-4	slr0360	GO:0016874	0.0173
sll0335	GO:0019637	<1e-4	sll1396	GO:0022411	<1e-4	sll0609	GO:0015077	<1e-4	slr1926	GO:0044464	<1e-4	sll1563	GO:0016874	0.0173
slr7023	GO:0009263	<1e-4	slr0287	GO:0006753	<1e-4	sll0552	GO:0016462	<1e-4	sll0010	GO:0044444	<1e-4	slr1436	GO:0016874	0.0173
ssr5019	GO:0009057	<1e-4	sll0192	GO:0051171	<1e-4	sll1285	GO:0015291	<1e-4	sll1191	GO:0044444	<1e-4	sll0756	GO:0016874	0.0173
slr0645	GO:0019219	<1e-4	slr0975	GO:0008150	<1e-4	sll0558	GO:0008171	<1e-4	slr1907	GO:0044446	<1e-4	sll0176	GO:0006950	0.0167
slr1177	GO:0071496	<1e-4	sll0243	GO:0044282	<1e-4	slr0978	GO:0022832	<1e-4	sll1735	GO:0005575	<1e-4	slr5023	GO:0006950	0.0167
ssr5106	GO:0006082	<1e-4	slr1790	GO:0006732	<1e-4	slr1025	GO:0022832	<1e-4	sll1912	GO:0044444	<1e-4	sll0710	GO:0006950	0.0167
slr0962	GO:0048522	<1e-4	slr0888	GO:0071840	<1e-4	slr1913	GO:0008171	<1e-4	slr0806	GO:0005575	<1e-4	ssr2422	GO:0006950	0.0167
slr2105	GO:0009259	<1e-4	sll0243	GO:0009124	<1e-4	slr5102	GO:0043492	<1e-4	sll1763	GO:0044424	<1e-4	sll1511	GO:0006950	0.0167
sll1009	GO:0048878	<1e-4	ssr6030	GO:0043436	<1e-4	slr1056	GO:0022803	<1e-4	sll1749	GO:0044424	<1e-4	sll0518	GO:0006950	0.0167
ssl5096	GO:0051246	<1e-4	sll0216	GO:0006793	<1e-4	sll1882	GO:0022832	<1e-4	slr0364	GO:0044464	<1e-4	slr1162	GO:0006950	0.0167
slr7095	GO:0044003	<1e-4	slr7026	GO:0031323	<1e-4	slr1397	GO:0043167	<1e-4	sll0862	GO:0044424	<1e-4	slr6015	GO:0006950	0.0167
sll1830	GO:0009259	<1e-4	slr0887	GO:0009893	<1e-4	sll1511	GO:0015291	<1e-4	sll1717	GO:0005575	<1e-4	ssl5007	GO:0006950	0.0167
sll1735	GO:0009126	<1e-4	sll1158	GO:0071840	<1e-4	slr7012	GO:0015399	<1e-4	sll1217	GO:0005575	<1e-4	slr0912	GO:0006950	0.0167
sll1938	GO:0009124	<1e-4	sll1024	GO:0048518	<1e-4	ssr2912	GO:0022832	<1e-4	ssl1498	GO:0005575	<1e-4	sll1691	GO:0006950	0.0167
ssr2611	GO:0090304	<1e-4	slr0607	GO:0051716	<1e-4	sll1151	GO:0016741	<1e-4	sll0456	GO:0044425	<1e-4	sll5062	GO:0006950	0.0167
sll0448	GO:0034660	<1e-4	sll1400	GO:0052111	<1e-4	ssr3572	GO:0042623	<1e-4	sll0442	GO:0044422	<1e-4	sll1469	GO:0006950	0.0167
slr1222	GO:0009056	<1e-4	slr0483	GO:0006793	<1e-4	sll1956	GO:0015399	<1e-4	sll1062	GO:0044425	<1e-4	slr0146	GO:0006950	0.0167
slr0482	GO:0044260	<1e-4	sll0473	GO:0009150	<1e-4	slr6008	GO:0060089	<1e-4	ssl0739	GO:0044446	<1e-4	sll1757	GO:0006950	0.0167
slr1340	GO:0019752	<1e-4	slr1468	GO:0043436	<1e-4	slr6065	GO:0016462	<1e-4	ssl0461	GO:0044446	<1e-4	sll0024	GO:0006950	0.0167
sll1934	GO:0009308	<1e-4	sll0372	GO:0051186	<1e-4	sll0314	GO:0022803	<1e-4	sll5062	GO:0005575	<1e-4	slr1660	GO:0006950	0.0167
sll1698	GO:0042451	<1e-4	slr1907	GO:0050801	<1e-4	slr0440	GO:0043167	<1e-4	ssr2998	GO:0044444	<1e-4	slr1546	GO:0006950	0.0167
slr0217	GO:0019752	<1e-4	sll0189	GO:0051347	<1e-4	sll1251	GO:0015293	<1e-4	slr0935	GO:0044464	<1e-4	ssl0461	GO:0006950	0.0167
slr0498	GO:0009142	<1e-4	ssr2802	GO:0065007	<1e-4	ssr1256	GO:0016462	<1e-4	slr5013	GO:0044425	<1e-4	slr0151	GO:0006950	0.0167
sll1570	GO:0006720	<1e-4	slr1162	GO:0051186	<1e-4	slr0104	GO:0015405	<1e-4	slr7010	GO:0044422	<1e-4	sll1401	GO:0006950	0.0167
slr0407	GO:0001932	<1e-4	slr1657	GO:0006091	<1e-4	slr1676	GO:0015267	<1e-4	slr2073	GO:0043226	<1e-4	slr0398	GO:0006950	0.0167
ssr6062	GO:0016054	<1e-4	sll0297	GO:0031326	<1e-4	slr1886	GO:0022836	<1e-4	ssr3572	GO:0043226	<1e-4	sll0658	GO:0006950	0.0167
slr0582	GO:0006066	<1e-4	ssl7042	GO:0043170	<1e-4	sll0985	GO:0015399	<1e-4	ssl3382	GO:0044446	<1e-4	slr7080	GO:0006950	0.0167
sll0505	GO:0051716	<1e-4	slr0273	GO:0043648	<1e-4	sll0606	GO:0015293	<1e-4	ssr0109	GO:0044464	<1e-4	sll5034	GO:0006950	0.0167
sll1254	GO:0080090	<1e-4	slr1025	GO:0005996	<1e-4	slr1507	GO:0016746	<1e-4	sll0913	GO:0005575	<1e-4	slr6014	GO:0006950	0.0167
slr0517	GO:0050794	<1e-4	slr1572	GO:0005996	<1e-4	sll1162	GO:0015077	<1e-4	sll1411	GO:0044446	<1e-4	ssr3341	GO:0006950	0.0167
slr0668	GO:0031323	<1e-4	slr2103	GO:0009263	<1e-4	sll0296	GO:0016746	<1e-4	slr0249	GO:0005575	<1e-4	sll1378	GO:0006950	0.0167
sll0558	GO:0019362	<1e-4	sll0266	GO:0009889	<1e-4	ssr2615	GO:0003674	<1e-4	slr1397	GO:0044464	<1e-4	slr0337	GO:0006950	0.0167
sll0939	GO:0009165	<1e-4	sll1193	GO:0006082	<1e-4	sll0063	GO:0015075	<1e-4	ssl5064	GO:0044464	<1e-4	sll1025	GO:0006950	0.0167
sll8007	GO:0033013	<1e-4	sll7089	GO:0022607	<1e-4	slr1577	GO:0016462	<1e-4	slr1576	GO:0044444	<1e-4	ssl8005	GO:0006950	0.0167
slr5102	GO:0009889	<1e-4	sll1853	GO:0043549	<1e-4	sll1052	GO:0043492	<1e-4	sll0615	GO:0005575	<1e-4	slr0195	GO:0006950	0.0167
slr5016	GO:0065007	<1e-4	sll8019	GO:0016054	<1e-4	sll0931	GO:0016462	<1e-4	sll1825	GO:0044425	<1e-4	sll8004	GO:0006950	0.0167
slr1752	GO:0045184	<1e-4	slr1462	GO:0008150	<1e-4	slr0147	GO:0060089	<1e-4	sll1447	GO:0044444	<1e-4	slr2010	GO:0006950	0.0167
slr2000	GO:0006811	<1e-4	slr0264	GO:0019752	<1e-4	sll0543	GO:0016746	<1e-4	slr1762	GO:0044464	<1e-4	sll0802	GO:0006950	0.0167
sll0585	GO:0019751	<1e-4	slr0801	GO:0009892	<1e-4	sll0861	GO:0022836	<1e-4	sll0641	GO:0005575	<1e-4	slr0606	GO:0006950	0.0167
sll0678	GO:0043412	<1e-4	slr2060	GO:0006720	<1e-4	ssl2807	GO:0022891	<1e-4	sll0751	GO:0005575	<1e-4	slr6013	GO:0006950	0.0167

slr0366	GO:0052188	<1e-4	ssl0109	GO:0051347	<1e-4	slr0291	GO:0043167	<1e-4	ssl5114	GO:0043226	<1e-4	sll1092	GO:0006950	0.0167
slr0479	GO:0046907	<1e-4	slr1799	GO:0033013	<1e-4	slr1674	GO:0015077	<1e-4	slr1169	GO:0044422	<1e-4	slr0333	GO:0006950	0.0167
sll1784	GO:0050896	<1e-4	slr6016	GO:0009142	<1e-4	sll0263	GO:0022832	<1e-4	slr7095	GO:0005575	<1e-4	slr6100	GO:0006950	0.0167
sll1884	GO:0051188	<1e-4	ssl2420	GO:0044283	<1e-4	sll0547	GO:0022838	<1e-4	slr1573	GO:0044444	<1e-4	slr6049	GO:0006950	0.0167
sll1915	GO:0046128	<1e-4	ssr3409	GO:0072521	<1e-4	slr1413	GO:0022838	<1e-4	slr1174	GO:0005575	<1e-4	ssr3189	GO:0006950	0.0167
ssr6024	GO:0009123	<1e-4	slr7016	GO:0052111	<1e-4	sll1381	GO:0016818	<1e-4	sll1203	GO:0043226	<1e-4	slr0059	GO:0006950	0.0167
sll0584	GO:0060255	<1e-4	slr1940	GO:0006766	<1e-4	sll0843	GO:0015291	<1e-4	slr0962	GO:0044425	<1e-4	slr1068	GO:0006950	0.0167
sll0280	GO:0006811	<1e-4	sll0860	GO:0016054	<1e-4	slr0816	GO:0015293	<1e-4	ssl1378	GO:0044464	<1e-4	sll0815	GO:0006950	0.0167
ssl5100	GO:0051347	<1e-4	sll1531	GO:0009132	<1e-4	slr1097	GO:0008171	<1e-4	sll7050	GO:0044424	<1e-4	slr1957	GO:0006950	0.0167
slr0848	GO:0046164	<1e-4	sll0532	GO:0009141	<1e-4	slr0602	GO:0016741	<1e-4	sll0864	GO:0044425	<1e-4	sll2015	GO:0006950	0.0167
sll1954	GO:0006753	<1e-4	ssr2067	GO:0043648	<1e-4	slr1128	GO:0016462	<1e-4	ssl2420	GO:0044464	<1e-4	sll5046	GO:0006950	0.0167
slr0404	GO:0009059	<1e-4	ssr2975	GO:0009987	<1e-4	slr0476	GO:0015267	<1e-4	slr1566	GO:0005575	<1e-4	slr1186	GO:0006950	0.0167
sll0780	GO:0009889	<1e-4	sll0225	GO:0034641	<1e-4	sll0488	GO:0016817	<1e-4	sll1969	GO:0044424	<1e-4	sll1130	GO:0006950	0.0167
sll0310	GO:0072527	<1e-4	sll1652	GO:0033013	<1e-4	ssr6027	GO:0015291	<1e-4	slr1062	GO:0044446	<1e-4	sll0760	GO:0006950	0.0167
slr5087	GO:0055082	<1e-4	sll1222	GO:0050794	<1e-4	ssr2422	GO:0043167	<1e-4	sll0498	GO:0044425	<1e-4	ssr0332	GO:0006950	0.0167
ssl2065	GO:0001932	<1e-4	sll1089	GO:0016053	<1e-4	sll1455	GO:0015291	<1e-4	slr1223	GO:0005575	<1e-4	slr0655	GO:0006950	0.0167
ssr1698	GO:0072522	<1e-4	ssr2781	GO:0009987	<1e-4	ssr0692	GO:0015399	<1e-4	slr6101	GO:0044446	<1e-4	slr1557	GO:0016874	0.0167
slr0053	GO:0008150	<1e-4	sll8033	GO:0009124	<1e-4	sll0863	GO:0016818	<1e-4	slr0423	GO:0005575	<1e-4	slr0076	GO:0016874	0.0167
sll0732	GO:0043933	<1e-4	sll0327	GO:0009124	<1e-4	ssr6086	GO:0022832	<1e-4	slr0270	GO:0044424	<1e-4	slr1127	GO:0016874	0.0167
sll0191	GO:0009260	<1e-4	slr1657	GO:0009259	<1e-4	ssr5106	GO:0008171	<1e-4	sll1902	GO:0005575	<1e-4	sll0360	GO:0016874	0.0167
slr1376	GO:0051179	<1e-4	sll1352	GO:0006091	<1e-4	slr1236	GO:0015291	<1e-4	ssr1768	GO:0044422	<1e-4	sll1472	GO:0016874	0.0167
slr0730	GO:0048523	<1e-4	sll0172	GO:0048522	<1e-4	slr0870	GO:0016746	<1e-4	sll1062	GO:0043226	<1e-4	sll1222	GO:0016772	0.0167
sll0428	GO:0001932	<1e-4	slr1081	GO:0090304	<1e-4	sll0867	GO:0016741	<1e-4	slr0787	GO:0044444	<1e-4	slr0545	GO:0016874	0.0167
slr1674	GO:0006066	<1e-4	sll0022	GO:0008150	<1e-4	slr1599	GO:0003674	<1e-4	sll0923	GO:0005575	<1e-4	sll1247	GO:0016874	0.0167
slr6088	GO:0046394	<1e-4	ssr1375	GO:0006733	<1e-4	sll1252	GO:0015075	<1e-4	slr1768	GO:0005575	<1e-4	sll0735	GO:0016772	0.0167
sll0743	GO:0042455	<1e-4	ssl0312	GO:0050801	<1e-4	ssl1255	GO:0016462	<1e-4	sll0265	GO:0044446	<1e-4	slr1353	GO:0016874	0.0167
slr1658	GO:0033013	<1e-4	ssr2803	GO:0042180	<1e-4	sll0803	GO:0005342	<1e-4	slr0709	GO:0044444	<1e-4	slr1612	GO:0016874	0.0167
slr1288	GO:0042180	<1e-4	slr6029	GO:0019637	<1e-4	sll0410	GO:0008171	<1e-4	sll0031	GO:0005575	<1e-4	sll1426	GO:0016874	0.0167
slr2011	GO:0009260	<1e-4	slr1275	GO:0042180	<1e-4	ssr3532	GO:0022857	<1e-4	sll1267	GO:0043226	<1e-4	sll1601	GO:0016874	0.0167
sll0263	GO:0051818	<1e-4	sll1797	GO:0051347	<1e-4	slr0592	GO:0043492	<1e-4	slr0514	GO:0005575	<1e-4	slr1918	GO:0016874	0.0167
sll0023	GO:0046394	<1e-4	slr1449	GO:0044271	<1e-4	slr1122	GO:0060089	<1e-4	sll7069	GO:0043226	<1e-4	slr1196	GO:0016874	0.0167
slr1557	GO:0006720	<1e-4	sll1858	GO:0006732	<1e-4	slr0209	GO:0022832	<1e-4	sll1318	GO:0044446	<1e-4	sll0382	GO:0016874	0.0167
slr1444	GO:0034654	<1e-4	ssl1923	GO:0009991	<1e-4	slr1110	GO:0008171	<1e-4	slr1956	GO:0044425	<1e-4	ssl0787	GO:0016874	0.0167
sll0737	GO:0050789	<1e-4	sll0175	GO:0006732	<1e-4	slr0169	GO:0015291	<1e-4	slr7016	GO:0043226	<1e-4	slr1066	GO:0016874	0.0167
sll0572	GO:0055114	<1e-4	ssl8028	GO:0050801	<1e-4	sll5028	GO:0003674	<1e-4	sll0176	GO:0044424	<1e-4	slr1083	GO:0016874	0.0167
slr0476	GO:0055086	<1e-4	sll8002	GO:0009144	<1e-4	sll0781	GO:0022836	<1e-4	slr1862	GO:0044422	<1e-4	slr0818	GO:0016772	0.0167
slr0816	GO:0009141	<1e-4	slr0729	GO:0009987	<1e-4	slr0784	GO:0043167	<1e-4	sll0241	GO:0044425	<1e-4	slr1674	GO:0016874	0.0167
slr0521	GO:0048522	<1e-4	sll5047	GO:0048523	<1e-4	slr0730	GO:0015267	<1e-4	sll0354	GO:0005575	<1e-4	slr1866	GO:0016874	0.0167
slr0217	GO:0044003	<1e-4	sll7033	GO:0031640	<1e-4	slr2118	GO:0016746	<1e-4	sll0482	GO:0005575	<1e-4	slr0287	GO:0016874	0.0167
slr7092	GO:0006576	<1e-4	slr1152	GO:0051716	<1e-4	ssr2998	GO:0015405	<1e-4	slr0333	GO:0044464	<1e-4	slr1023	GO:0016874	0.0167
slr1261	GO:0006766	<1e-4	sll0191	GO:0043436	<1e-4	sll0319	GO:0022890	<1e-4	slr1339	GO:0044464	<1e-4	slr1441	GO:0016874	0.0167
slr1611	GO:0009308	<1e-4	slr0362	GO:0051171	<1e-4	slr1932	GO:0042623	<1e-4	sll1009	GO:0044425	<1e-4	sll0547	GO:0016874	0.0167
slr1658	GO:0051186	<1e-4	slr6028	GO:0051716	<1e-4	ssl6061	GO:0043492	<1e-4	ssr2843	GO:0044444	<1e-4	slr0423	GO:0016874	0.0167
slr0217	GO:0071841	<1e-4	slr2012	GO:0042455	<1e-4	sll0910	GO:0022892	<1e-4	slr0273	GO:0043226	<1e-4	sll0913	GO:0006807	0.0166
slr0142	GO:0031326	<1e-4	slr2118	GO:0043549	<1e-4	slr0935	GO:0043167	<1e-4	sll0864	GO:0005575	<1e-4	slr1462	GO:0006807	0.0166
ssl5008	GO:0006732	<1e-4	slr6067	GO:0048519	<1e-4	slr1194	GO:0022892	<1e-4	sll0645	GO:0044424	<1e-4	sll7062	GO:0006807	0.0166
sll1832	GO:0055114	<1e-4	sll1609	GO:0001932	<1e-4	slr1263	GO:0022832	<1e-4	sll1696	GO:0044444	<1e-4	slr1657	GO:0006807	0.0166
slr1789	GO:0042451	<1e-4	slr0264	GO:0006733	<1e-4	sll7028	GO:0015267	<1e-4	sll1315	GO:0044444	<1e-4	sll0545	GO:0006807	0.0166
sll0858	GO:0001932	<1e-4	sll0786	GO:0019752	<1e-4	ssl5113	GO:0016462	<1e-4	slr5087	GO:0044424	<1e-4	sll5034	GO:0006807	0.0166
sll1528	GO:0072528	<1e-4	sll0007	GO:0006066	<1e-4	slr0924	GO:0043167	<1e-4	sll0031	GO:0044422	<1e-4	sll1359	GO:0006807	0.0166
slr6103	GO:0018130	<1e-4	sll0888	GO:0034654	<1e-4	slr0092	GO:0015291	<1e-4	ssr6089	GO:0044444	<1e-4	slr0333	GO:0006807	0.0166
slr0730	GO:0046164	<1e-4	sll0225	GO:0051716	<1e-4	slr6106	GO:0043492	<1e-4	sll7067	GO:0044444	<1e-4	ssl5099	GO:0006807	0.0166
slr1935	GO:0008610	<1e-4	slr1210	GO:0048518	<1e-4	slr1290	GO:0015291	<1e-4	slr1846	GO:0005575	<1e-4	sll5026	GO:0006807	0.0166
ssl2148	GO:0022411	<1e-4	slr7026	GO:0055082	<1e-4	sll0284	GO:0005342	<1e-4	slr0698	GO:0043226	<1e-4	ssl5007	GO:0006807	0.0166

slr0848	GO:0046394	<1e-4	sll0497	GO:0006066	<1e-4	sll0861	GO:0008171	<1e-4	slr5115	GO:0044446	<1e-4	sll1853	GO:0006807	0.0166
sll1692	GO:0019752	<1e-4	ssl0739	GO:0019637	<1e-4	slr0587	GO:0022890	<1e-4	slr0487	GO:0044446	<1e-4	sll6054	GO:0006807	0.0166
ssr3189	GO:0016053	<1e-4	sll0294	GO:0072527	<1e-4	sll5062	GO:0060089	<1e-4	ssr7084	GO:0044422	<1e-4	slr5119	GO:0006807	0.0166
slr0935	GO:0006066	<1e-4	sll0932	GO:0009126	<1e-4	slr0592	GO:0003674	<1e-4	ssl2420	GO:0005575	<1e-4	ssr7036	GO:0006807	0.0166
slr0468	GO:0052111	<1e-4	ssr6027	GO:0016052	<1e-4	slr0650	GO:0022803	<1e-4	ssr2781	GO:0044464	<1e-4	slr0869	GO:0006807	0.0166
slr5037	GO:0008150	<1e-4	sll1024	GO:0051171	<1e-4	sll1446	GO:0042623	<1e-4	sll0168	GO:0044425	<1e-4	ssr3409	GO:0006807	0.0166
slr0147	GO:0065007	<1e-4	slr0655	GO:0019637	<1e-4	ssl3142	GO:0043167	<1e-4	sll1925	GO:0044425	<1e-4	ssl3383	GO:0006807	0.0166
slr1866	GO:0080090	<1e-4	sll0496	GO:0006720	<1e-4	slr0023	GO:0015267	<1e-4	slr1790	GO:0044446	<1e-4	slr1863	GO:0006807	0.0166
ssl2920	GO:0031640	<1e-4	slr0637	GO:0044260	<1e-4	slr6014	GO:0015405	<1e-4	slr1999	GO:0044424	<1e-4	slr1195	GO:0006807	0.0166
sll0218	GO:0046128	<1e-4	sll5033	GO:0046483	<1e-4	slr0742	GO:0022838	<1e-4	sll1912	GO:0044425	<1e-4	slr1998	GO:0006807	0.0166
slr0517	GO:0080090	<1e-4	sll1552	GO:0009144	<1e-4	slr1394	GO:0003674	<1e-4	slr0780	GO:0044425	<1e-4	sll2015	GO:0006807	0.0166
slr1307	GO:0043170	<1e-4	slr0890	GO:0006220	<1e-4	ssr1951	GO:0016817	<1e-4	sll0760	GO:0044446	<1e-4	slr1186	GO:0006807	0.0166
slr1338	GO:0009199	<1e-4	slr5024	GO:0009132	<1e-4	slr1816	GO:0008171	<1e-4	ssl0467	GO:0044422	<1e-4	slr0505	GO:0006807	0.0166
slr0294	GO:0016053	<1e-4	sll0588	GO:0006720	<1e-4	sll1940	GO:0043492	<1e-4	ssl2148	GO:0044464	<1e-4	slr6039	GO:0006807	0.0166
sll0846	GO:0009394	<1e-4	slr5101	GO:0046483	<1e-4	slr1471	GO:0016746	<1e-4	sll1573	GO:0044444	<1e-4	ssl1004	GO:0006807	0.0166
sll0031	GO:0050794	<1e-4	slr1062	GO:0051234	<1e-4	ssl3615	GO:0016818	<1e-4	slr0521	GO:0044444	<1e-4	sll0762	GO:0006807	0.0166
slr1638	GO:0009394	<1e-4	slr0664	GO:0043412	<1e-4	sll1542	GO:0003674	<1e-4	slr1935	GO:0005575	<1e-4	slr1419	GO:0006807	0.0166
ssl0483	GO:0009991	<1e-4	sll0268	GO:0044260	<1e-4	slr0723	GO:0008171	<1e-4	slr0398	GO:0044422	<1e-4	sll1691	GO:0006807	0.0166
slr1263	GO:0022411	<1e-4	ssr6085	GO:0044255	<1e-4	slr1266	GO:0015405	<1e-4	slr5073	GO:0044444	<1e-4	sll1773	GO:0006807	0.0166
slr5116	GO:0072527	<1e-4	slr1571	GO:0044275	<1e-4	sll7086	GO:0022838	<1e-4	slr1241	GO:0044422	<1e-4	slr6007	GO:0006807	0.0166
slr0702	GO:0042455	<1e-4	slr1087	GO:0044282	<1e-4	slr0876	GO:0022803	<1e-4	slr1288	GO:0044424	<1e-4	sll5033	GO:0006807	0.0166
ssl0312	GO:0072527	<1e-4	slr7083	GO:0046394	<1e-4	sll0047	GO:0022832	<1e-4	sll1939	GO:0044446	<1e-4	ssr0335	GO:0006807	0.0166
sll0298	GO:0009117	<1e-4	slr5119	GO:0072524	<1e-4	slr1507	GO:0016741	<1e-4	slr7073	GO:0005575	<1e-4	slr2119	GO:0006807	0.0166
sll0886	GO:0009987	<1e-4	slr0787	GO:0009057	<1e-4	sll0176	GO:0016818	<1e-4	sll1359	GO:0044422	<1e-4	slr7096	GO:0006807	0.0166
sll1681	GO:0051186	<1e-4	sll1862	GO:0009132	<1e-4	ssr1951	GO:0016741	<1e-4	slr1278	GO:0044424	<1e-4	ssr2912	GO:0006807	0.0166
sll0414	GO:0006163	<1e-4	slr0317	GO:0016053	<1e-4	sll1072	GO:0015293	<1e-4	slr6050	GO:0043226	<1e-4	slr7101	GO:0006807	0.0166
sll1131	GO:0005996	<1e-4	sll0749	GO:0072521	<1e-4	sll0980	GO:0060089	<1e-4	slr0725	GO:0044446	<1e-4	slr5116	GO:0006807	0.0166
sll0008	GO:0051186	<1e-4	slr0196	GO:0006811	<1e-4	slr1032	GO:0003674	<1e-4	sll0822	GO:0044425	<1e-4	sll1400	GO:0006807	0.0166
slr1178	GO:0019362	<1e-4	sll0381	GO:0048878	<1e-4	slr7016	GO:0042623	<1e-4	sll0625	GO:0044446	<1e-4	ssl1498	GO:0006807	0.0166
ssl0832	GO:0055086	<1e-4	slr0184	GO:0090304	<1e-4	slr2144	GO:0022890	<1e-4	sll0595	GO:0005575	<1e-4	slr1468	GO:0006807	0.0166
sll1486	GO:0045184	<1e-4	sll1401	GO:0055114	<1e-4	sll1691	GO:0003674	<1e-4	slr0106	GO:0044446	<1e-4	slr7083	GO:0006807	0.0166
ssl7039	GO:0006066	<1e-4	sll1583	GO:0006766	<1e-4	sll1188	GO:0022892	<1e-4	sll0446	GO:0044425	<1e-4	sll0775	GO:0006807	0.0166
sll0775	GO:0008150	<1e-4	slr0731	GO:0048518	<1e-4	sll1131	GO:0015075	<1e-4	ssr1499	GO:0044422	<1e-4	sll0886	GO:0006807	0.0166
sll0532	GO:0050794	<1e-4	slr6029	GO:0050789	<1e-4	slr1194	GO:0022838	<1e-4	sll1512	GO:0044464	<1e-4	sll1447	GO:0006807	0.0166
slr1537	GO:0019751	<1e-4	slr2060	GO:0009260	<1e-4	slr1577	GO:0022891	<1e-4	ssr7035	GO:0044422	<1e-4	sll0181	GO:0006807	0.0166
slr7013	GO:0006066	<1e-4	slr0569	GO:0031323	<1e-4	slr5127	GO:0008171	<1e-4	sll0310	GO:0044446	<1e-4	slr6067	GO:0006807	0.0166
sll8012	GO:0009059	<1e-4	slr1547	GO:0019362	<1e-4	sll1293	GO:0016746	<1e-4	sll0274	GO:0044422	<1e-4	sll1062	GO:0006807	0.0166
slr1413	GO:0009142	<1e-4	slr0941	GO:0043648	<1e-4	slr1218	GO:0015075	<1e-4	slr0398	GO:0043226	<1e-4	sll1722	GO:0006807	0.0166
slr0304	GO:0048518	<1e-4	sll5090	GO:0046164	<1e-4	slr0923	GO:0008171	<1e-4	slr2119	GO:0044444	<1e-4	sll0658	GO:0006807	0.0166
sll1891	GO:0044106	<1e-4	sll0406	GO:0019219	<1e-4	ssl8039	GO:0016462	<1e-4	slr1677	GO:0044424	<1e-4	ssr1407	GO:0006807	0.0166
ssr7035	GO:0051716	<1e-4	slr0455	GO:0044271	<1e-4	sll5062	GO:0016462	<1e-4	ssl1300	GO:0044464	<1e-4	slr7080	GO:0006807	0.0166
sll1401	GO:0072521	<1e-4	slr2000	GO:0016054	<1e-4	slr0980	GO:0016746	<1e-4	sll1163	GO:0044464	<1e-4	sll1509	GO:0006807	0.0166
slr1951	GO:0009394	<1e-4	slr0458	GO:0034641	<1e-4	slr1623	GO:0015293	<1e-4	slr1173	GO:0044464	<1e-4	ssl2717	GO:0006807	0.0166
sll1036	GO:0044275	<1e-4	sll0354	GO:0009394	<1e-4	slr1612	GO:0008171	<1e-4	sll1072	GO:0044424	<1e-4	sll0815	GO:0006807	0.0166
sll0930	GO:0008610	<1e-4	sll0760	GO:0009124	<1e-4	ssl7051	GO:0008171	<1e-4	slr2060	GO:0044424	<1e-4	ssr3189	GO:0006807	0.0166
slr0092	GO:0022411	<1e-4	ssr1528	GO:0016054	<1e-4	sll1764	GO:0022891	<1e-4	ssl8008	GO:0043226	<1e-4	slr1566	GO:0006807	0.0166
ssr6099	GO:0005996	<1e-4	ssl2162	GO:0009394	<1e-4	sll0424	GO:0022890	<1e-4	ssl3177	GO:0044424	<1e-4	slr0407	GO:0006807	0.0166
sll0007	GO:0046164	<1e-4	ssr2615	GO:0072528	<1e-4	ssl1004	GO:0022892	<1e-4	slr7025	GO:0044444	<1e-4	ssr2554	GO:0006807	0.0166
slr1263	GO:0048518	<1e-4	sll0863	GO:0009987	<1e-4	slr0082	GO:0060089	<1e-4	slr0082	GO:0044444	<1e-4	slr7082	GO:0006807	0.0166
sll0609	GO:0046483	<1e-4	slr0264	GO:0050801	<1e-4	sll1692	GO:0015077	<1e-4	slr1207	GO:0005575	<1e-4	slr7095	GO:0006807	0.0166
slr0053	GO:0009117	<1e-4	sll1477	GO:0044106	<1e-4	slr0300	GO:0016817	<1e-4	ssr2787	GO:0044425	<1e-4	slr6047	GO:0006807	0.0166
slr5023	GO:0044248	<1e-4	sll0939	GO:0009161	<1e-4	ssr6027	GO:0015075	<1e-4	sll0180	GO:0044425	<1e-4	slr1306	GO:0006807	0.0166
ssl3829	GO:0010556	<1e-4	slr0404	GO:0006066	<1e-4	sll0563	GO:0016746	<1e-4	sll1547	GO:0044444	<1e-4	sll0487	GO:0006807	0.0166
sll1188	GO:0006082	<1e-4	slr6063	GO:0009165	<1e-4	sll1426	GO:0016817	<1e-4	ssl1792	GO:0005575	<1e-4	sll0284	GO:0006807	0.0166

sll0274	GO:0065007	<1e-4	sll0803	GO:0043648	<1e-4	sll0376	GO:0015077	<1e-4	sll0066	GO:0044424	<1e-4	slr6073	GO:0006807	0.0166
sll0269	GO:0051716	<1e-4	slr2048	GO:0006811	<1e-4	ssr2317	GO:0015405	<1e-4	sll0098	GO:0044464	<1e-4	sll0478	GO:0006807	0.0166
sll1054	GO:0031640	<1e-4	slr6087	GO:0008150	<1e-4	slr0765	GO:0022838	<1e-4	slr1752	GO:0044424	<1e-4	slr6013	GO:0006807	0.0166
slr0179	GO:0006793	<1e-4	slr1619	GO:0009893	<1e-4	sll1640	GO:0016817	<1e-4	sll0314	GO:0044422	<1e-4	ssl2781	GO:0006807	0.0166
sll1773	GO:0009056	<1e-4	slr1069	GO:0055086	<1e-4	sll1006	GO:0042623	<1e-4	slr1718	GO:0044422	<1e-4	ssl8005	GO:0006807	0.0166
sll1691	GO:0008610	<1e-4	sll1321	GO:0006066	<1e-4	sll1726	GO:0022838	<1e-4	sll1072	GO:0044464	<1e-4	ssl2471	GO:0006807	0.0166
sll0243	GO:0072522	<1e-4	slr6007	GO:0044271	<1e-4	slr1911	GO:0008171	<1e-4	slr1303	GO:0044444	<1e-4	slr1429	GO:0006807	0.0166
slr1493	GO:0009123	<1e-4	sll0982	GO:0051818	<1e-4	slr1768	GO:0015077	<1e-4	sll0149	GO:0005575	<1e-4	sll8007	GO:0006807	0.0166
sll0335	GO:0060255	<1e-4	ssr1391	GO:0043170	<1e-4	slr0112	GO:0015399	<1e-4	slr1944	GO:0043226	<1e-4	slr1815	GO:0006807	0.0166
ssl1972	GO:0010468	<1e-4	slr2004	GO:0046164	<1e-4	sll0007	GO:0008171	<1e-4	sll1504	GO:0044424	<1e-4	sll0168	GO:0006259	0.0161
ssr2554	GO:0009987	<1e-4	sll1071	GO:0009142	<1e-4	sll0010	GO:0015293	<1e-4	slr6074	GO:0044464	<1e-4	sll0282	GO:0006259	0.0161
slr1163	GO:0055082	<1e-4	sll0994	GO:0050801	<1e-4	slr6008	GO:0015075	<1e-4	sll1464	GO:0044424	<1e-4	slr1677	GO:0006259	0.0161
ssr1951	GO:0006091	<1e-4	sll0069	GO:0009056	<1e-4	sll0737	GO:0016817	<1e-4	slr0398	GO:0044446	<1e-4	sll1485	GO:0006259	0.0161
slr0272	GO:0042451	<1e-4	sll0611	GO:0009123	<1e-4	sll1025	GO:0016818	<1e-4	sll1834	GO:0044425	<1e-4	sll0167	GO:0006259	0.0161
sll0931	GO:0009893	<1e-4	sll1891	GO:0043648	<1e-4	slr1110	GO:0022832	<1e-4	sll1378	GO:0044464	<1e-4	slr0262	GO:0006259	0.0161
slr0960	GO:0065007	<1e-4	sll1022	GO:0034641	<1e-4	slr1218	GO:0043167	<1e-4	sll5032	GO:0044422	<1e-4	slr1187	GO:0006259	0.0161
ssr3300	GO:0072527	<1e-4	slr1623	GO:0033013	<1e-4	slr0333	GO:0022803	<1e-4	slr1275	GO:0044425	<1e-4	slr0038	GO:0006259	0.0161
ssr1041	GO:0048519	<1e-4	sll0611	GO:0042451	<1e-4	ssl2009	GO:0008171	<1e-4	slr1263	GO:0044446	<1e-4	slr1815	GO:0006259	0.0161
slr0516	GO:0034654	<1e-4	sll0843	GO:0046128	<1e-4	sll0793	GO:0015077	<1e-4	sll0174	GO:0044444	<1e-4	slr1468	GO:0006259	0.0161
sll1191	GO:0009991	<1e-4	sll1307	GO:0019362	<1e-4	sll1783	GO:0008171	<1e-4	slr0598	GO:0044425	<1e-4	sll0397	GO:0006259	0.0161
sll0238	GO:0072521	<1e-4	ssl3379	GO:0055086	<1e-4	sll1285	GO:0022803	<1e-4	sll1797	GO:0044464	<1e-4	slr2032	GO:0006259	0.0161
slr1103	GO:0080090	<1e-4	slr2117	GO:0071840	<1e-4	ssr6046	GO:0008171	<1e-4	slr1811	GO:0044425	<1e-4	slr5018	GO:0006259	0.0161
ssl2920	GO:0008150	<1e-4	sll1671	GO:0055114	<1e-4	slr1449	GO:0022838	<1e-4	slr0393	GO:0005575	<1e-4	sll1155	GO:0006259	0.0161
sll1752	GO:0065007	<1e-4	slr1306	GO:0006220	<1e-4	sll1095	GO:0005342	<1e-4	sll0022	GO:0044424	<1e-4	slr2119	GO:0006259	0.0161
sll1583	GO:0065007	<1e-4	slr6029	GO:0009263	<1e-4	ssl0739	GO:0016818	<1e-4	slr1278	GO:0044444	<1e-4	ssr0102	GO:0006259	0.0161
ssr6086	GO:0043436	<1e-4	sll1166	GO:0009142	<1e-4	slr0455	GO:0060089	<1e-4	slr0664	GO:0005575	<1e-4	slr1273	GO:0006259	0.0161
sll0266	GO:0050801	<1e-4	sll0802	GO:0043549	<1e-4	slr2052	GO:0016817	<1e-4	sll0762	GO:0005575	<1e-4	slr1958	GO:0006259	0.0161
sll0815	GO:0043170	<1e-4	sll8032	GO:0051246	<1e-4	slr0876	GO:0005342	<1e-4	slr1263	GO:0044424	<1e-4	slr2101	GO:0006259	0.0161
slr6044	GO:0009142	<1e-4	slr1413	GO:0051234	<1e-4	ssr1768	GO:0022891	<1e-4	sll6055	GO:0044464	<1e-4	slr1914	GO:0006259	0.0161
ssl0788	GO:0044003	<1e-4	sll0394	GO:0022607	<1e-4	slr1398	GO:0015291	<1e-4	sll1251	GO:0044424	<1e-4	sll0853	GO:0006259	0.0161
sll0658	GO:0006721	<1e-4	slr6008	GO:0019752	<1e-4	ssl0294	GO:0015399	<1e-4	sml0011	GO:0005575	<1e-4	slr1768	GO:0006259	0.0161
slr0053	GO:0050896	<1e-4	slr1851	GO:0045184	<1e-4	slr1471	GO:0016741	<1e-4	sll1380	GO:0044444	<1e-4	sll0284	GO:0006259	0.0161
slr5053	GO:0009057	<1e-4	slr1425	GO:0055086	<1e-4	sll0069	GO:0005342	<1e-4	ssl5113	GO:0044464	<1e-4	slr0151	GO:0006259	0.0161
ssl2065	GO:0044283	<1e-4	sll1764	GO:0006721	<1e-4	ssr3154	GO:0016818	<1e-4	slr0553	GO:0044464	<1e-4	slr1660	GO:0006259	0.0161
sll1763	GO:0019362	<1e-4	slr1920	GO:0009056	<1e-4	slr0360	GO:0016817	<1e-4	slr2119	GO:0043226	<1e-4	sll5006	GO:0006259	0.0161
slr0053	GO:0019637	<1e-4	sll0558	GO:0052188	<1e-4	sll1609	GO:0016818	<1e-4	sll1186	GO:0044464	<1e-4	slr6006	GO:0006259	0.0161
ssl0788	GO:0042455	<1e-4	sll1009	GO:0042455	<1e-4	slr1116	GO:0016741	<1e-4	sll0181	GO:0005575	<1e-4	slr6080	GO:0006259	0.0161
sll0678	GO:0051171	<1e-4	slr0699	GO:0090304	<1e-4	slr0670	GO:0016741	<1e-4	slr1306	GO:0005575	<1e-4	sll0545	GO:0006259	0.0161
sll0428	GO:0009150	<1e-4	slr0318	GO:0019222	<1e-4	ssr0109	GO:0060089	<1e-4	slr7082	GO:0044422	<1e-4	slr1568	GO:0006259	0.0161
slr0334	GO:0009161	<1e-4	sll0101	GO:0006631	<1e-4	sll1068	GO:0003674	<1e-4	slr0869	GO:0044424	<1e-4	sgl0001	GO:0006259	0.0161
sll0886	GO:0046483	<1e-4	slr1116	GO:0046128	<1e-4	sll0225	GO:0022890	<1e-4	slr6013	GO:0044446	<1e-4	slr2010	GO:0006259	0.0161
sll0911	GO:0051246	<1e-4	ssl0739	GO:0009142	<1e-4	sll0272	GO:0022892	<1e-4	sll8001	GO:0044446	<1e-4	slr0569	GO:0006259	0.0161
sll0547	GO:0009309	<1e-4	sll7078	GO:0050794	<1e-4	sll1954	GO:0015293	<1e-4	slr6012	GO:0005575	<1e-4	sll0611	GO:0006259	0.0161
slr6012	GO:0006066	<1e-4	sll0198	GO:0043436	<1e-4	slr1307	GO:0015267	<1e-4	slr1261	GO:0005575	<1e-4	slr7081	GO:0006259	0.0161
ssr1256	GO:0009144	<1e-4	slr1128	GO:0043170	<1e-4	sll7050	GO:0016746	<1e-4	sll0779	GO:0044444	<1e-4	ssr1766	GO:0006259	0.0161
ssl7074	GO:0006811	<1e-4	slr0755	GO:0051234	<1e-4	slr0912	GO:0060089	<1e-4	sll1571	GO:0044425	<1e-4	slr1097	GO:0006259	0.0161
slr7083	GO:0043412	<1e-4	sll1446	GO:0072527	<1e-4	sll0785	GO:0022857	<1e-4	slr1258	GO:0044425	<1e-4	slr0552	GO:0006259	0.0161
slr1071	GO:0046907	<1e-4	sll1834	GO:0009987	<1e-4	slr2103	GO:0043167	<1e-4	slr1668	GO:0005575	<1e-4	sll1884	GO:0006259	0.0161
slr1391	GO:0009057	<1e-4	slr6066	GO:0009892	<1e-4	ssr6078	GO:0022857	<1e-4	slr0334	GO:0044446	<1e-4	slr7101	GO:0006259	0.0161
slr0376	GO:0019222	<1e-4	sll0732	GO:0016054	<1e-4	slr7098	GO:0015293	<1e-4	slr1095	GO:0005575	<1e-4	slr0092	GO:0006259	0.0161
slr7097	GO:0016052	<1e-4	slr0238	GO:0031323	<1e-4	ssr3341	GO:0043492	<1e-4	slr2052	GO:0005575	<1e-4	slr6063	GO:0006259	0.0161
slr5012	GO:0006721	<1e-4	sll1319	GO:0055082	<1e-4	ssr0657	GO:0043167	<1e-4	sll1106	GO:0005575	<1e-4	slr6005	GO:0006259	0.0161
slr1178	GO:0009144	<1e-4	slr2101	GO:0051716	<1e-4	sll0762	GO:0043492	<1e-4	slr1495	GO:0044446	<1e-4	slr1183	GO:0006259	0.0161
slr0392	GO:0008610	<1e-4	sll1060	GO:0042455	<1e-4	slr1083	GO:0016741	<1e-4	slr1444	GO:0044425	<1e-4	sll8004	GO:0006259	0.0161

sll0762	GO:0005996	<1e-4	slr0300	GO:0009308	<1e-4	sll1319	GO:0016741	<1e-4	slr1927	GO:0044425	<1e-4	slr6072	GO:0006259	0.0161
sll5034	GO:0072524	<1e-4	ssr1114	GO:0022411	<1e-4	ssl7039	GO:0060089	<1e-4	sll0696	GO:0044425	<1e-4	ssr2912	GO:0006259	0.0161
sll7030	GO:0071496	<1e-4	slr1170	GO:0080090	<1e-4	ssl1417	GO:0005342	<1e-4	sll1570	GO:0005575	<1e-4	slr0264	GO:0006259	0.0161
slr1468	GO:0060255	<1e-4	ssl0109	GO:0051234	<1e-4	slr2025	GO:0008171	<1e-4	sll0513	GO:0044446	<1e-4	sll8033	GO:0006259	0.0161
sll1660	GO:0034654	<1e-4	sll0298	GO:0009165	<1e-4	sll0294	GO:0008171	<1e-4	sll0293	GO:0044424	<1e-4	ssl5129	GO:0006259	0.0161
sll0678	GO:0051179	<1e-4	ssl2595	GO:0016054	<1e-4	sll5026	GO:0022890	<1e-4	slr0554	GO:0044444	<1e-4	slr0971	GO:0006259	0.0161
sll0444	GO:0031323	<1e-4	slr1926	GO:0009117	<1e-4	slr0602	GO:0022838	<1e-4	sll0854	GO:0044425	<1e-4	slr1263	GO:0006259	0.0161
slr1753	GO:0008610	<1e-4	sll0669	GO:0043170	<1e-4	sll0839	GO:0008171	<1e-4	slr0386	GO:0044424	<1e-4	ssr2787	GO:0006259	0.0159
slr1188	GO:0006082	<1e-4	slr1923	GO:0006163	<1e-4	sll0752	GO:0016817	<1e-4	sll0930	GO:0005575	<1e-4	sll0524	GO:0006259	0.0159
ssr3129	GO:0034660	<1e-4	sll0625	GO:0051347	<1e-4	slr8021	GO:0022891	<1e-4	ssl0352	GO:0043226	<1e-4	slr1885	GO:0006259	0.0159
slr1573	GO:0006220	<1e-4	sll0447	GO:0006082	<1e-4	slr7095	GO:0022891	<1e-4	slr1964	GO:0043226	<1e-4	sll0066	GO:0006259	0.0159
ssr1768	GO:0010556	<1e-4	sll1130	GO:0090304	<1e-4	sll1135	GO:0016818	<1e-4	slr0885	GO:0043226	<1e-4	ssl2807	GO:0006259	0.0159
slr1122	GO:0006576	<1e-4	sll1163	GO:0009142	<1e-4	slr1644	GO:0022803	<1e-4	slr2046	GO:0005575	<1e-4	ssr1765	GO:0006259	0.0159
sll7089	GO:0019219	<1e-4	sll7030	GO:0008150	<1e-4	sll0419	GO:0042623	<1e-4	sll1062	GO:0044444	<1e-4	slr1999	GO:0006259	0.0159
sll1696	GO:0006082	<1e-4	slr7100	GO:0048518	<1e-4	sll0803	GO:0016746	<1e-4	sll0167	GO:0044464	<1e-4	slr0941	GO:0006259	0.0159
sll0930	GO:0010468	<1e-4	ssl8039	GO:0072524	<1e-4	slr0498	GO:0022892	<1e-4	slr1568	GO:0005575	<1e-4	ssr2962	GO:0006259	0.0159
slr6091	GO:0006793	<1e-4	sll1583	GO:0048519	<1e-4	slr0076	GO:0015291	<1e-4	slr0049	GO:0044446	<1e-4	slr0921	GO:0006259	0.0159
slr1628	GO:0006082	<1e-4	sll0414	GO:0044003	<1e-4	slr1163	GO:0005342	<1e-4	sll0183	GO:0005575	<1e-4	slr1571	GO:0006259	0.0159
sll1509	GO:0051716	<1e-4	ssl0312	GO:0006733	<1e-4	slr1628	GO:0022838	<1e-4	sll0867	GO:0044424	<1e-4	slr0294	GO:0006259	0.0159
ssl2807	GO:0046164	<1e-4	slr1287	GO:0006733	<1e-4	sll0096	GO:0043167	<1e-4	ssl3829	GO:0044422	<1e-4	slr0386	GO:0006259	0.0159
slr0606	GO:0048519	<1e-4	slr0151	GO:0048522	<1e-4	sll1373	GO:0022857	<1e-4	slr0625	GO:0043226	<1e-4	slr2011	GO:0006259	0.0159
ssr7072	GO:0006720	<1e-4	ssr3189	GO:0009117	<1e-4	sll1061	GO:0022892	<1e-4	sll0676	GO:0044464	<1e-4	sll1949	GO:0006807	0.0155
sll1307	GO:0051716	<1e-4	slr0695	GO:0034654	<1e-4	sll1163	GO:0042623	<1e-4	sll0413	GO:0044446	<1e-4	sll0007	GO:0006807	0.0155
slr1187	GO:0090304	<1e-4	slr0981	GO:0006721	<1e-4	slr0287	GO:0022892	<1e-4	sll1109	GO:0044446	<1e-4	slr0845	GO:0006807	0.0155
slr1407	GO:0044255	<1e-4	sll7087	GO:0044275	<1e-4	ssr7072	GO:0022857	<1e-4	slr1846	GO:0044444	<1e-4	sll0280	GO:0006807	0.0155
sll0775	GO:0044283	<1e-4	ssl7022	GO:0044275	<1e-4	slr1262	GO:0003674	<1e-4	sll1765	GO:0044425	<1e-4	ssl0242	GO:0006807	0.0155
sll7062	GO:0048522	<1e-4	slr0845	GO:0019637	<1e-4	ssl1300	GO:0022803	<1e-4	ssr2611	GO:0044444	<1e-4	slr2144	GO:0006807	0.0155
sll1911	GO:0019752	<1e-4	sll1485	GO:0016052	<1e-4	sll1381	GO:0060089	<1e-4	slr1690	GO:0044464	<1e-4	slr1230	GO:0006807	0.0155
slr1365	GO:0051716	<1e-4	slr1768	GO:0009889	<1e-4	slr0967	GO:0005342	<1e-4	slr1623	GO:0005575	<1e-4	sll1675	GO:0006807	0.0155
slr7059	GO:0016052	<1e-4	slr1644	GO:0006793	<1e-4	slr0479	GO:0015399	<1e-4	slr2105	GO:0005575	<1e-4	slr0404	GO:0006807	0.0155
ssr1558	GO:0051171	<1e-4	slr1218	GO:0009259	<1e-4	sll1006	GO:0016818	<1e-4	sll1656	GO:0044464	<1e-4	slr1638	GO:0006807	0.0155
slr0740	GO:0043933	<1e-4	slr0755	GO:0009892	<1e-4	ssl0353	GO:0022836	<1e-4	sll0802	GO:0005575	<1e-4	sll1830	GO:0006807	0.0155
sll1447	GO:0019219	<1e-4	slr0869	GO:0009889	<1e-4	sll1912	GO:0003674	<1e-4	sll1321	GO:0044444	<1e-4	slr0431	GO:0006807	0.0155
slr2027	GO:0048518	<1e-4	sll1072	GO:0090304	<1e-4	slr0740	GO:0015399	<1e-4	ssl2245	GO:0005575	<1e-4	sll0676	GO:0006807	0.0155
slr0975	GO:0046164	<1e-4	slr0408	GO:0044282	<1e-4	sll1355	GO:0016462	<1e-4	sll0930	GO:0044446	<1e-4	slr1573	GO:0006807	0.0155
sll1446	GO:0009263	<1e-4	slr0609	GO:0048518	<1e-4	slr7026	GO:0042623	<1e-4	sll1954	GO:0044425	<1e-4	ssr2787	GO:0006807	0.0155
sll2006	GO:0006163	<1e-4	ssl5095	GO:0043933	<1e-4	slr1572	GO:0015293	<1e-4	sll0925	GO:0044446	<1e-4	sll0532	GO:0006807	0.0155
sll1765	GO:0065007	<1e-4	slr1647	GO:0044283	<1e-4	slr2073	GO:0015291	<1e-4	sll6053	GO:0044425	<1e-4	slr0360	GO:0006807	0.0155
sll5033	GO:0051234	<1e-4	slr0730	GO:0072524	<1e-4	slr1614	GO:0022838	<1e-4	sll1606	GO:0043226	<1e-4	slr0144	GO:0006807	0.0155
sll1072	GO:0044248	<1e-4	slr1737	GO:0009117	<1e-4	ssr5092	GO:0022832	<1e-4	slr2122	GO:0043226	<1e-4	slr0872	GO:0006807	0.0155
sll0101	GO:0006163	<1e-4	sll5128	GO:0051179	<1e-4	sll0930	GO:0022892	<1e-4	sll1233	GO:0005575	<1e-4	slr1913	GO:0006807	0.0155
ssr2553	GO:0006793	<1e-4	slr0845	GO:0065007	<1e-4	slr0459	GO:0016817	<1e-4	ssl0788	GO:0005575	<1e-4	sll0804	GO:0044237	0.015
slr6021	GO:0044282	<1e-4	sll0793	GO:0051171	<1e-4	slr6021	GO:0015399	<1e-4	slr0325	GO:0044425	<1e-4	slr0491	GO:0044237	0.015
ssr1698	GO:0006091	<1e-4	sll1433	GO:0006766	<1e-4	sll1736	GO:0016741	<1e-4	slr1468	GO:0044464	<1e-4	sll0272	GO:0004518	0.015
slr0699	GO:0052188	<1e-4	ssr3467	GO:0046164	<1e-4	slr1957	GO:0022890	<1e-4	slr0404	GO:0044444	<1e-4	sll1119	GO:0004518	0.015
ssr3467	GO:0019219	<1e-4	sll0586	GO:0065007	<1e-4	ssr6089	GO:0015291	<1e-4	slr1266	GO:0044444	<1e-4	slr2018	GO:0004518	0.015
slr7081	GO:0022411	<1e-4	slr1397	GO:0009991	<1e-4	sll1350	GO:0022832	<1e-4	slr0813	GO:0044424	<1e-4	slr1032	GO:0004518	0.015
slr1774	GO:0071841	<1e-4	sll1570	GO:0008610	<1e-4	ssr2611	GO:0015267	<1e-4	sll1233	GO:0044444	<1e-4	ssr1258	GO:0004518	0.015
slr0442	GO:0046907	<1e-4	ssr1155	GO:0022411	<1e-4	slr0625	GO:0016741	<1e-4	ssr1375	GO:0044446	<1e-4	slr0408	GO:0004518	0.015
slr1444	GO:0009123	<1e-4	sll0423	GO:0009150	<1e-4	ssr0692	GO:0060089	<1e-4	slr0269	GO:0044422	<1e-4	sll0606	GO:0004518	0.015
sll0563	GO:0051171	<1e-4	slr5116	GO:0009165	<1e-4	slr1235	GO:0042623	<1e-4	slr1103	GO:0044444	<1e-4	ssr2803	GO:0004518	0.015
sll1611	GO:0009893	<1e-4	slr1215	GO:0043933	<1e-4	sll1304	GO:0022803	<1e-4	slr0871	GO:0044464	<1e-4	sll1912	GO:0004518	0.015
ssr6020	GO:0009144	<1e-4	slr1847	GO:0009892	<1e-4	slr0362	GO:0015293	<1e-4	slr1800	GO:0044422	<1e-4	sll0071	GO:0004518	0.015
sll1307	GO:0009260	<1e-4	slr0263	GO:0051246	<1e-4	slr1101	GO:0015077	<1e-4	sll1570	GO:0044422	<1e-4	slr0937	GO:0006807	0.0149

slr0521	GO:0009144	<1e-4	ssr0657	GO:0009150	<1e-4	slr0731	GO:0016746	<1e-4	slr1846	GO:0044424	<1e-4	ssr0536	GO:0006807	0.0149
slr1163	GO:0060255	<1e-4	ssl2245	GO:0044260	<1e-4	slr1958	GO:0015267	<1e-4	slr0151	GO:0005575	<1e-4	slr0871	GO:0006807	0.0149
slr0712	GO:0031640	<1e-4	slr0006	GO:0001932	<1e-4	slr1122	GO:0015267	<1e-4	sll0010	GO:0044422	<1e-4	sll1218	GO:0006807	0.0149
slr1069	GO:0019219	<1e-4	slr7058	GO:0009124	<1e-4	sll1318	GO:0016746	<1e-4	slr2073	GO:0044444	<1e-4	sll0996	GO:0006807	0.0149
sll0263	GO:0048522	<1e-4	sll1164	GO:0055082	<1e-4	sll0443	GO:0060089	<1e-4	ssl2749	GO:0005575	<1e-4	sll1135	GO:0006807	0.0149
slr1081	GO:0051246	<1e-4	slr0376	GO:0046128	<1e-4	sll1527	GO:0022857	<1e-4	sll1158	GO:0044444	<1e-4	slr1083	GO:0006807	0.0149
slr0334	GO:0046128	<1e-4	ssl5031	GO:0009141	<1e-4	slr7098	GO:0016462	<1e-4	slr6103	GO:0044422	<1e-4	sll0743	GO:0006807	0.0149
sll1222	GO:0009059	<1e-4	ssr7035	GO:0009144	<1e-4	slr0579	GO:0043167	<1e-4	sll0854	GO:0005575	<1e-4	slr0503	GO:0006807	0.0149
slr0423	GO:0043933	<1e-4	sll1071	GO:0008610	<1e-4	sll1956	GO:0022890	<1e-4	sll0913	GO:0044444	<1e-4	slr0553	GO:0006807	0.0149
sll0843	GO:0009987	<1e-4	sll0167	GO:0008610	<1e-4	sll1717	GO:0022857	<1e-4	sll1476	GO:0043226	<1e-4	slr0299	GO:0006807	0.0149
slr7101	GO:0042451	<1e-4	sll0658	GO:0046483	<1e-4	sll1722	GO:0022891	<1e-4	slr0989	GO:0043226	<1e-4	slr1222	GO:0006807	0.0149
slr1774	GO:0019222	<1e-4	slr6073	GO:0031326	<1e-4	ssr0657	GO:0016741	<1e-4	sll1072	GO:0005575	<1e-4	slr1659	GO:0006807	0.0149
slr1273	GO:0019752	<1e-4	slr1053	GO:0006082	<1e-4	slr1270	GO:0015291	<1e-4	ssr2422	GO:0005575	<1e-4	slr0545	GO:0006807	0.0149
ssl3549	GO:0042455	<1e-4	sll1289	GO:0043933	<1e-4	sll0710	GO:0043167	<1e-4	slr1862	GO:0005575	<1e-4	slr1557	GO:0006807	0.0149
sll0803	GO:0009165	<1e-4	sll1656	GO:0009161	<1e-4	ssr6024	GO:0022857	<1e-4	sll1222	GO:0005575	<1e-4	slr0243	GO:0006807	0.0149
sll0359	GO:0009987	<1e-4	sll1304	GO:0034641	<1e-4	ssr1558	GO:0015291	<1e-4	sll1969	GO:0005575	<1e-4	sll0513	GO:0006807	0.0149
slr1799	GO:0043412	<1e-4	slr0423	GO:0052188	<1e-4	sll1784	GO:0016818	<1e-4	sll0939	GO:0044424	<1e-4	sll0296	GO:0006807	0.0149
sll0376	GO:0080090	<1e-4	sll0781	GO:0043170	<1e-4	sll0588	GO:0022836	<1e-4	sll7033	GO:0044464	<1e-4	sll1934	GO:0006950	0.0147
slr1570	GO:0046394	<1e-4	slr1084	GO:0065007	<1e-4	slr0582	GO:0060089	<1e-4	slr0505	GO:0044444	<1e-4	slr1391	GO:0006950	0.0147
sll0445	GO:0006886	<1e-4	sll0802	GO:0046394	<1e-4	slr2049	GO:0015291	<1e-4	slr6057	GO:0044446	<1e-4	slr1659	GO:0006950	0.0147
slr1470	GO:0009123	<1e-4	slr1624	GO:0051188	<1e-4	slr0913	GO:0022836	<1e-4	ssl2781	GO:0044424	<1e-4	slr0937	GO:0006950	0.0147
sll0297	GO:0009126	<1e-4	sll1717	GO:0006576	<1e-4	ssl2162	GO:0008171	<1e-4	slr0142	GO:0044424	<1e-4	slr0852	GO:0006950	0.0147
slr1541	GO:0009126	<1e-4	slr6015	GO:0044271	<1e-4	slr0211	GO:0016741	<1e-4	ssl5103	GO:0043226	<1e-4	ssr0536	GO:0006950	0.0147
slr7057	GO:0034641	<1e-4	ssr2611	GO:0052188	<1e-4	ssl1004	GO:0042623	<1e-4	slr0810	GO:0044422	<1e-4	ssr1375	GO:0006950	0.0147
slr0852	GO:0071496	<1e-4	slr1413	GO:0048519	<1e-4	sll1061	GO:0016818	<1e-4	slr0453	GO:0044422	<1e-4	slr1612	GO:0006950	0.0147
ssl1972	GO:0044281	<1e-4	slr0491	GO:0048519	<1e-4	sll0183	GO:0022803	<1e-4	slr7081	GO:0005575	<1e-4	sll0547	GO:0006950	0.0147
slr0605	GO:0010556	<1e-4	sll6055	GO:0071496	<1e-4	slr1827	GO:0043492	<1e-4	slr0957	GO:0044422	<1e-4	ssl0787	GO:0006950	0.0147
sll5028	GO:0051179	<1e-4	sll0985	GO:0051179	<1e-4	sll1698	GO:0015405	<1e-4	slr0496	GO:0044444	<1e-4	ssr1698	GO:0006950	0.0147
sll1477	GO:0043648	<1e-4	slr2117	GO:0019751	<1e-4	slr1384	GO:0022832	<1e-4	sll1913	GO:0044444	<1e-4	ssl3692	GO:0006950	0.0147
sll1119	GO:0001932	<1e-4	sll0572	GO:0016053	<1e-4	ssr3154	GO:0008171	<1e-4	slr1391	GO:0044464	<1e-4	slr1441	GO:0006950	0.0147
sll1658	GO:0008610	<1e-4	sll1285	GO:0050801	<1e-4	sll1390	GO:0003674	<1e-4	sll1306	GO:0044422	<1e-4	slr0545	GO:0006950	0.0147
sll1092	GO:0009394	<1e-4	sll0181	GO:0048523	<1e-4	slr6006	GO:0015405	<1e-4	slr1398	GO:0044422	<1e-4	sll1426	GO:0008233	0.0145
slr1178	GO:0016054	<1e-4	slr2025	GO:0055114	<1e-4	sll0611	GO:0022803	<1e-4	sll7064	GO:0043226	<1e-4	ssl0787	GO:0008233	0.0145
sll0837	GO:0046128	<1e-4	sll0237	GO:0072528	<1e-4	slr0408	GO:0043167	<1e-4	slr1920	GO:0044446	<1e-4	sll1601	GO:0008233	0.0145
sll7086	GO:0044106	<1e-4	slr1593	GO:0048518	<1e-4	slr0243	GO:0015399	<1e-4	slr1495	GO:0044422	<1e-4	slr0305	GO:0008233	0.0145
slr0587	GO:0009259	<1e-4	ssl7007	GO:0042455	<1e-4	slr2110	GO:0015293	<1e-4	sll5130	GO:0044446	<1e-4	sll1166	GO:0008233	0.0145
slr0930	GO:0044255	<1e-4	slr7016	GO:0055114	<1e-4	sll0272	GO:0015293	<1e-4	slr1998	GO:0044464	<1e-4	slr2025	GO:0008233	0.0145
slr0601	GO:0006732	<1e-4	slr0103	GO:0009132	<1e-4	sll1247	GO:0016818	<1e-4	slr0887	GO:0044446	<1e-4	sll0743	GO:0008233	0.0145
slr2117	GO:0033013	<1e-4	slr1425	GO:0009056	<1e-4	ssr3189	GO:0015267	<1e-4	slr1918	GO:0044422	<1e-4	slr1045	GO:0008233	0.0145
slr0479	GO:0044282	<1e-4	sll0532	GO:0006720	<1e-4	sll1025	GO:0016746	<1e-4	ssl5027	GO:0044424	<1e-4	slr1196	GO:0008233	0.0145
slr1128	GO:0052188	<1e-4	sll1752	GO:0044260	<1e-4	slr0356	GO:0022890	<1e-4	sll1757	GO:0044464	<1e-4	sll1060	GO:0008233	0.0145
slr1880	GO:0051347	<1e-4	sll1086	GO:0019637	<1e-4	slr6004	GO:0060089	<1e-4	sll1547	GO:0044422	<1e-4	sll0596	GO:0008233	0.0145
sll0875	GO:0044248	<1e-4	slr1263	GO:0051818	<1e-4	sll1835	GO:0022803	<1e-4	sll1675	GO:0005575	<1e-4	sll0513	GO:0008233	0.0145
slr1174	GO:0051716	<1e-4	sll1160	GO:0016054	<1e-4	slr1203	GO:0015405	<1e-4	sll1785	GO:0044424	<1e-4	sll0424	GO:0008233	0.0145
sll1656	GO:0046164	<1e-4	slr0082	GO:0051188	<1e-4	sll0853	GO:0016817	<1e-4	slr0262	GO:0044444	<1e-4	sll1186	GO:0008233	0.0145
ssl0410	GO:0006721	<1e-4	sll1411	GO:0019752	<1e-4	slr7059	GO:0015267	<1e-4	slr1547	GO:0043226	<1e-4	slr1866	GO:0008233	0.0145
ssl5008	GO:0048878	<1e-4	slr0848	GO:0031323	<1e-4	ssr3154	GO:0043167	<1e-4	slr0238	GO:0043226	<1e-4	slr0304	GO:0008233	0.0145
sll1469	GO:0009161	<1e-4	sll0751	GO:0008610	<1e-4	sll1219	GO:0022836	<1e-4	sll1834	GO:0005575	<1e-4	slr0545	GO:0008233	0.0145
sll0381	GO:0043436	<1e-4	slr2110	GO:0042451	<1e-4	sll1381	GO:0016462	<1e-4	sll2013	GO:0044425	<1e-4	ssr0536	GO:0008233	0.0145
slr1911	GO:0072521	<1e-4	sll5128	GO:0009987	<1e-4	ssl5100	GO:0016818	<1e-4	sll1131	GO:0043226	<1e-4	slr1417	GO:0008233	0.0145
sll1866	GO:0048523	<1e-4	ssl1923	GO:0034641	<1e-4	ssl5045	GO:0022890	<1e-4	slr1601	GO:0043226	<1e-4	slr0551	GO:0008233	0.0145
sll7070	GO:0009057	<1e-4	slr1762	GO:0050896	<1e-4	slr0269	GO:0022838	<1e-4	sll1464	GO:0044422	<1e-4	sll0354	GO:0008233	0.0145
slr1866	GO:0044282	<1e-4	slr0272	GO:0051716	<1e-4	ssr6032	GO:0015267	<1e-4	slr1616	GO:0043226	<1e-4	slr0937	GO:0008233	0.0145
slr6103	GO:0042180	<1e-4	slr0455	GO:0048878	<1e-4	slr0971	GO:0015399	<1e-4	slr1343	GO:0043226	<1e-4	sll0499	GO:0008233	0.0145

sll1526	GO:0042180	<1e-4	slr0870	GO:0009126	<1e-4	slr8022	GO:0015077	<1e-4	sll7070	GO:0044422	<1e-4	sll1433	GO:0008233	0.0145
slr1638	GO:0019219	<1e-4	sll0997	GO:0005996	<1e-4	slr0456	GO:0016817	<1e-4	slr7097	GO:0044444	<1e-4	sll0931	GO:0005975	0.0144
slr1505	GO:0065007	<1e-4	slr6104	GO:0022411	<1e-4	slr0168	GO:0043492	<1e-4	sll0268	GO:0044446	<1e-4	sll0068	GO:0005515	0.0142
ssr2615	GO:0006811	<1e-4	sll0505	GO:0016053	<1e-4	slr0333	GO:0022832	<1e-4	ssr3304	GO:0044464	<1e-4	sll0596	GO:0006259	0.0141
slr1398	GO:0016052	<1e-4	sll0786	GO:0065007	<1e-4	sll1433	GO:0022803	<1e-4	ssr2317	GO:0044422	<1e-4	sll1166	GO:0006259	0.0141
ssr2803	GO:0072524	<1e-4	sll1160	GO:0006576	<1e-4	sll1775	GO:0005342	<1e-4	slr0317	GO:0043226	<1e-4	sll1318	GO:0006259	0.0141
slr0689	GO:0009117	<1e-4	sll1426	GO:0071840	<1e-4	sll7077	GO:0015399	<1e-4	slr1266	GO:0044422	<1e-4	sll1913	GO:0006259	0.0141
ssr1558	GO:0051188	<1e-4	slr1384	GO:0072524	<1e-4	sll8040	GO:0015267	<1e-4	sll0638	GO:0043226	<1e-4	sll0447	GO:0006259	0.0141
slr6007	GO:0006720	<1e-4	slr0787	GO:0009987	<1e-4	sll0871	GO:0022857	<1e-4	slr1537	GO:0044425	<1e-4	slr0287	GO:0006259	0.0141
slr0483	GO:0016053	<1e-4	sll0101	GO:0043549	<1e-4	slr0655	GO:0016741	<1e-4	sll1388	GO:0043226	<1e-4	sll0547	GO:0006259	0.0141
ssl5064	GO:0019752	<1e-4	slr1900	GO:0043436	<1e-4	slr2111	GO:0060089	<1e-4	ssr6046	GO:0005575	<1e-4	slr1799	GO:0006259	0.0141
sll1960	GO:0055082	<1e-4	slr1576	GO:0048878	<1e-4	sll1542	GO:0015399	<1e-4	sll1505	GO:0044422	<1e-4	sll0508	GO:0006259	0.0141
sll1092	GO:0016052	<1e-4	sll1528	GO:0001932	<1e-4	sll0553	GO:0016818	<1e-4	slr1079	GO:0044422	<1e-4	sll0513	GO:0006259	0.0141
sll0981	GO:0009394	<1e-4	sll0060	GO:0050801	<1e-4	slr7102	GO:0043167	<1e-4	sll1089	GO:0044422	<1e-4	sll0743	GO:0006259	0.0141
sll1630	GO:0009308	<1e-4	sll7070	GO:0009263	<1e-4	slr1762	GO:0015075	<1e-4	sll0669	GO:0044444	<1e-4	sll0354	GO:0019538	0.014
slr0108	GO:0006576	<1e-4	sll0238	GO:0009987	<1e-4	ssl5015	GO:0022803	<1e-4	slr1612	GO:0044444	<1e-4	slr0287	GO:0019538	0.014
slr7071	GO:0051246	<1e-4	slr1068	GO:0009144	<1e-4	slr0479	GO:0015405	<1e-4	slr0147	GO:0005575	<1e-4	sll0854	GO:0019538	0.014
slr0393	GO:0008150	<1e-4	slr0590	GO:0072521	<1e-4	sll0586	GO:0016462	<1e-4	sll1749	GO:0044446	<1e-4	sll1601	GO:0019538	0.014
sll0355	GO:0052111	<1e-4	slr0976	GO:0009987	<1e-4	sll0412	GO:0022892	<1e-4	slr1752	GO:0043226	<1e-4	slr0376	GO:0019538	0.014
slr0594	GO:0044283	<1e-4	ssr2975	GO:0051171	<1e-4	sll1562	GO:0016462	<1e-4	slr1951	GO:0044446	<1e-4	sll0263	GO:0019538	0.014
ssr5120	GO:0009142	<1e-4	slr5018	GO:0051171	<1e-4	slr1599	GO:0015293	<1e-4	sll0781	GO:0043226	<1e-4	ssl0109	GO:0019538	0.014
slr2120	GO:0072527	<1e-4	sll1552	GO:0048523	<1e-4	slr0204	GO:0015267	<1e-4	slr2115	GO:0044422	<1e-4	slr1107	GO:0019538	0.014
sll0982	GO:0034641	<1e-4	slr8022	GO:0009991	<1e-4	sll0023	GO:0005342	<1e-4	slr1670	GO:0044446	<1e-4	sll0296	GO:0019538	0.014
ssr0102	GO:0046483	<1e-4	slr1073	GO:0019219	<1e-4	sll0412	GO:0016462	<1e-4	slr1530	GO:0005575	<1e-4	sll1426	GO:0019538	0.014
sll1541	GO:0034660	<1e-4	sll1251	GO:0009117	<1e-4	sll1319	GO:0043167	<1e-4	slr1442	GO:0044446	<1e-4	ssr0536	GO:0019538	0.014
slr1074	GO:0042455	<1e-4	sll1447	GO:0008150	<1e-4	ssl1918	GO:0015399	<1e-4	slr7101	GO:0044464	<1e-4	ssr2848	GO:0019538	0.014
slr6094	GO:0009161	<1e-4	sll1411	GO:0033013	<1e-4	sll0659	GO:0042623	<1e-4	slr0771	GO:0044446	<1e-4	sll1186	GO:0019538	0.014
sll0189	GO:0031326	<1e-4	sll1526	GO:0006732	<1e-4	slr1032	GO:0016817	<1e-4	sll0513	GO:0044422	<1e-4	sll0846	GO:0019538	0.0139
sll0172	GO:0009144	<1e-4	slr1263	GO:0022607	<1e-4	sll7086	GO:0015291	<1e-4	sll1254	GO:0044425	<1e-4	slr6014	GO:0019538	0.0139
sll1155	GO:0009199	<1e-4	slr7058	GO:0009309	<1e-4	ssr1528	GO:0005342	<1e-4	ssr6032	GO:0044425	<1e-4	ssr2422	GO:0019538	0.0139
sll7086	GO:0006766	<1e-4	slr0695	GO:0006753	<1e-4	slr0975	GO:0008171	<1e-4	sll1446	GO:0005575	<1e-4	sll1921	GO:0019538	0.0139
slr1340	GO:0006066	<1e-4	slr0789	GO:0009165	<1e-4	ssl1300	GO:0022857	<1e-4	slr1083	GO:0044444	<1e-4	sll0481	GO:0019538	0.0139
slr1398	GO:0006163	<1e-4	ssr3402	GO:0048519	<1e-4	slr6074	GO:0016817	<1e-4	sll0586	GO:0043226	<1e-4	slr0695	GO:0019538	0.0139
ssl5100	GO:0080090	<1e-4	slr1342	GO:0022607	<1e-4	ssl8039	GO:0042623	<1e-4	ssl2064	GO:0044422	<1e-4	sll0886	GO:0019538	0.0139
sll1426	GO:0006066	<1e-4	slr0519	GO:0009124	<1e-4	sll1355	GO:0015405	<1e-4	slr0582	GO:0044464	<1e-4	sll0293	GO:0019538	0.0139
sll0481	GO:0042455	<1e-4	slr1474	GO:0044248	<1e-4	ssr6003	GO:0016818	<1e-4	sll7028	GO:0005575	<1e-4	slr6004	GO:0019538	0.0139
slr5053	GO:0016053	<1e-4	sll0283	GO:0051186	<1e-4	sll0321	GO:0043167	<1e-4	slr5115	GO:0044425	<1e-4	ssr6026	GO:0019538	0.0139
sll1472	GO:0048878	<1e-4	sll1095	GO:0044248	<1e-4	slr1541	GO:0022892	<1e-4	slr1218	GO:0044464	<1e-4	sll1414	GO:0019538	0.0139
sll0354	GO:0052188	<1e-4	slr2080	GO:0071841	<1e-4	slr1081	GO:0015075	<1e-4	sll0180	GO:0044444	<1e-4	sll1162	GO:0019538	0.0139
sll1583	GO:0006721	<1e-4	slr6038	GO:0031640	<1e-4	sll1053	GO:0015267	<1e-4	slr1207	GO:0044464	<1e-4	slr0407	GO:0019538	0.0139
ssr2318	GO:0009161	<1e-4	slr6067	GO:0051716	<1e-4	sll1477	GO:0005342	<1e-4	sll0085	GO:0044464	<1e-4	sll1396	GO:0019538	0.0139
slr5037	GO:0044003	<1e-4	slr1288	GO:0051188	<1e-4	slr0272	GO:0015267	<1e-4	slr0575	GO:0044444	<1e-4	slr1566	GO:0019538	0.0139
sll0354	GO:0060255	<1e-4	ssl2384	GO:0044106	<1e-4	slr5018	GO:0043167	<1e-4	sll1170	GO:0044446	<1e-4	slr2101	GO:0019538	0.0139
slr0581	GO:0019362	<1e-4	ssr0761	GO:0044003	<1e-4	sll1651	GO:0043167	<1e-4	sll1954	GO:0005575	<1e-4	slr7014	GO:0019538	0.0139
slr5087	GO:0034641	<1e-4	slr0505	GO:0008150	<1e-4	sll0447	GO:0016746	<1e-4	slr1431	GO:0005575	<1e-4	sll1552	GO:0019538	0.0139
ssl3549	GO:0009991	<1e-4	sll1192	GO:0071496	<1e-4	sll0985	GO:0042623	<1e-4	sll1054	GO:0044444	<1e-4	ssl1464	GO:0019538	0.0139
sll0623	GO:0071842	<1e-4	slr0299	GO:0009987	<1e-4	slr1413	GO:0022803	<1e-4	slr7024	GO:0005575	<1e-4	slr1362	GO:0019538	0.0139
sll0793	GO:0008610	<1e-4	sll0756	GO:0009394	<1e-4	sll1163	GO:0008171	<1e-4	sll7063	GO:0043226	<1e-4	ssr6079	GO:0019538	0.0139
sll1832	GO:0006082	<1e-4	sll0249	GO:0019637	<1e-4	sml0011	GO:0015075	<1e-4	slr1914	GO:0044425	<1e-4	ssl5095	GO:0019538	0.0139
sll1472	GO:0009059	<1e-4	ssr2318	GO:0072524	<1e-4	slr0287	GO:0015267	<1e-4	slr0871	GO:0044424	<1e-4	slr0598	GO:0019538	0.0139
sll1219	GO:0065007	<1e-4	sll0995	GO:0042451	<1e-4	sll1072	GO:0043492	<1e-4	slr1045	GO:0044422	<1e-4	slr6065	GO:0019538	0.0139
ssl5096	GO:0009057	<1e-4	sll1401	GO:0009056	<1e-4	slr1537	GO:0060089	<1e-4	sll1170	GO:0044464	<1e-4	ssl3142	GO:0019538	0.0139
ssl2814	GO:0044281	<1e-4	slr1023	GO:0009141	<1e-4	sll1749	GO:0022803	<1e-4	slr1958	GO:0044446	<1e-4	sll0400	GO:0019538	0.0139
slr0605	GO:0006576	<1e-4	slr1468	GO:0072522	<1e-4	slr1591	GO:0016462	<1e-4	sll0085	GO:0005575	<1e-4	sll7063	GO:0019538	0.0139

ssr0759	GO:0044260	<1e-4	sll0596	GO:0009150	<1e-4	slr0326	GO:0016746	<1e-4	slr0204	GO:0043226	<1e-4	slr5118	GO:0019538	0.0139
sll1691	GO:0019222	<1e-4	ssr5019	GO:0044106	<1e-4	sll0539	GO:0022891	<1e-4	sll1608	GO:0043226	<1e-4	slr2032	GO:0019538	0.0139
sll1350	GO:0043412	<1e-4	slr1591	GO:0006766	<1e-4	slr1847	GO:0015077	<1e-4	slr0209	GO:0044422	<1e-4	slr7058	GO:0019538	0.0139
sll5046	GO:0046483	<1e-4	ssl3291	GO:0006720	<1e-4	sll1160	GO:0042623	<1e-4	slr0105	GO:0044446	<1e-4	sll0863	GO:0019538	0.0139
ssr0657	GO:0043549	<1e-4	ssr3129	GO:0019219	<1e-4	slr0092	GO:0022857	<1e-4	sll0639	GO:0044444	<1e-4	sll1939	GO:0019538	0.0139
sll0394	GO:0006091	<1e-4	ssl0832	GO:0042451	<1e-4	sll0787	GO:0022803	<1e-4	ssr6024	GO:0044425	<1e-4	sll1217	GO:0019538	0.0139
sll1680	GO:0072521	<1e-4	sll1304	GO:0046483	<1e-4	sll0181	GO:0042623	<1e-4	slr1161	GO:0044444	<1e-4	slr1189	GO:0019538	0.0139
sll1174	GO:0009987	<1e-4	slr0264	GO:0045184	<1e-4	slr0147	GO:0016746	<1e-4	slr0784	GO:0044444	<1e-4	slr6007	GO:0019538	0.0139
slr1519	GO:0044281	<1e-4	sll1915	GO:0006720	<1e-4	sll5033	GO:0022803	<1e-4	slr1398	GO:0043226	<1e-4	slr1815	GO:0019538	0.0139
slr1800	GO:0022411	<1e-4	slr1183	GO:0043170	<1e-4	slr1773	GO:0022892	<1e-4	sll7078	GO:0005575	<1e-4	sll0436	GO:0019538	0.0139
sll0449	GO:0034654	<1e-4	slr0975	GO:0044260	<1e-4	slr6088	GO:0022838	<1e-4	ssr2787	GO:0044464	<1e-4	slr0971	GO:0019538	0.0139
sll1632	GO:0071840	<1e-4	sll1651	GO:0046907	<1e-4	slr0708	GO:0042623	<1e-4	sll0763	GO:0044446	<1e-4	sll1022	GO:0019538	0.0139
slr1450	GO:0019219	<1e-4	slr1917	GO:0009126	<1e-4	sll1109	GO:0043167	<1e-4	slr1956	GO:0005575	<1e-4	ssl2749	GO:0019538	0.0139
sll0361	GO:0045184	<1e-4	slr0207	GO:0006811	<1e-4	sll0410	GO:0015291	<1e-4	ssr3402	GO:0005575	<1e-4	ssr3410	GO:0019538	0.0139
ssr5092	GO:0009126	<1e-4	ssr8047	GO:0009132	<1e-4	slr1025	GO:0042623	<1e-4	ssl3451	GO:0044424	<1e-4	ssr0332	GO:0019538	0.0139
slr0821	GO:0071496	<1e-4	sll1461	GO:0031323	<1e-4	sll0985	GO:0016462	<1e-4	slr6071	GO:0005575	<1e-4	ssl8005	GO:0019538	0.0139
sll1658	GO:0051716	<1e-4	slr0821	GO:0006082	<1e-4	ssr2781	GO:0008171	<1e-4	ssl0750	GO:0044422	<1e-4	slr1907	GO:0019538	0.0139
slr0967	GO:0042451	<1e-4	slr6072	GO:0034654	<1e-4	sll1135	GO:0015075	<1e-4	sll1132	GO:0044464	<1e-4	slr5021	GO:0019538	0.0139
sll1352	GO:0044260	<1e-4	slr2012	GO:0043549	<1e-4	sll1942	GO:0043167	<1e-4	sll0177	GO:0044444	<1e-4	slr1814	GO:0019538	0.0139
ssl8039	GO:0043933	<1e-4	ssl0350	GO:0009165	<1e-4	slr0337	GO:0022890	<1e-4	sll0760	GO:0044444	<1e-4	slr6067	GO:0019538	0.0139
sll0147	GO:0009142	<1e-4	ssr1473	GO:0044255	<1e-4	sll1583	GO:0016818	<1e-4	sll0861	GO:0044446	<1e-4	slr1315	GO:0019538	0.0139
sll1396	GO:0009126	<1e-4	slr7094	GO:0006733	<1e-4	sll8019	GO:0015075	<1e-4	slr0480	GO:0044424	<1e-4	sll5062	GO:0019538	0.0139
slr0241	GO:0006220	<1e-4	slr1079	GO:0009987	<1e-4	sll0297	GO:0005342	<1e-4	ssr2554	GO:0044424	<1e-4	sll1461	GO:0019538	0.0139
slr1183	GO:0051179	<1e-4	slr1762	GO:0050801	<1e-4	slr0264	GO:0015293	<1e-4	slr7011	GO:0005575	<1e-4	sll0284	GO:0019538	0.0139
slr1816	GO:0006811	<1e-4	sll0414	GO:0009260	<1e-4	slr0625	GO:0015077	<1e-4	slr1441	GO:0005575	<1e-4	slr1546	GO:0019538	0.0139
sll0444	GO:0060255	<1e-4	sll0913	GO:0006721	<1e-4	sll1714	GO:0015405	<1e-4	ssl5099	GO:0043226	<1e-4	ssl1004	GO:0019538	0.0139
sll1192	GO:0019752	<1e-4	ssl2971	GO:0051716	<1e-4	ssr2067	GO:0003674	<1e-4	slr1572	GO:0044444	<1e-4	slr1340	GO:0019538	0.0139
slr1195	GO:0048523	<1e-4	sll0443	GO:0071842	<1e-4	ssl5091	GO:0022836	<1e-4	ssl3451	GO:0044422	<1e-4	ssl2781	GO:0019538	0.0139
sll5130	GO:0034641	<1e-4	sll0208	GO:0043412	<1e-4	ssl8007	GO:0043492	<1e-4	slr6065	GO:0044422	<1e-4	sll0659	GO:0019538	0.0139
sll0563	GO:0042180	<1e-4	slr1852	GO:0009161	<1e-4	sll1251	GO:0022891	<1e-4	sll5047	GO:0044446	<1e-4	ssr2554	GO:0019538	0.0139
sll1714	GO:0009308	<1e-4	sll5062	GO:0046907	<1e-4	slr7094	GO:0016818	<1e-4	sll1188	GO:0043226	<1e-4	ssl2420	GO:0019538	0.0139
slr1970	GO:0009987	<1e-4	slr1495	GO:0006082	<1e-4	sll1071	GO:0016741	<1e-4	slr6103	GO:0044424	<1e-4	sll1155	GO:0019538	0.0139
sll5004	GO:0048519	<1e-4	sll0827	GO:0046128	<1e-4	slr1162	GO:0022836	<1e-4	slr0553	GO:0044425	<1e-4	sll1092	GO:0019538	0.0139
sll1866	GO:0051186	<1e-4	slr1690	GO:0022607	<1e-4	sll0382	GO:0015405	<1e-4	ssr3402	GO:0044422	<1e-4	slr7097	GO:0019538	0.0139
sll0249	GO:0022411	<1e-4	slr0483	GO:0042455	<1e-4	slr1493	GO:0022892	<1e-4	ssl3615	GO:0005575	<1e-4	sll0498	GO:0019538	0.0139
sll0447	GO:0043648	<1e-4	slr0551	GO:0009142	<1e-4	slr0780	GO:0016462	<1e-4	slr0602	GO:0005575	<1e-4	sll1160	GO:0019538	0.0139
ssr2802	GO:0044275	<1e-4	sll0815	GO:0045184	<1e-4	ssr6083	GO:0016817	<1e-4	sll1638	GO:0005575	<1e-4	sll1651	GO:0019538	0.0139
slr1398	GO:0006091	<1e-4	sll0350	GO:0031326	<1e-4	slr7092	GO:0022891	<1e-4	ssr0336	GO:0043226	<1e-4	sll5047	GO:0019538	0.0139
sll1586	GO:0031640	<1e-4	slr1767	GO:0019637	<1e-4	slr1082	GO:0016462	<1e-4	sll1510	GO:0044422	<1e-4	slr0393	GO:0019538	0.0139
slr1104	GO:0019752	<1e-4	sll1164	GO:0010556	<1e-4	sll0181	GO:0015293	<1e-4	sll1613	GO:0044425	<1e-4	ssl5007	GO:0019538	0.0139
sll1232	GO:0046128	<1e-4	ssr0761	GO:0051716	<1e-4	sll7069	GO:0042623	<1e-4	slr0302	GO:0044422	<1e-4	ssl0461	GO:0019538	0.0139
slr6073	GO:0044282	<1e-4	sll1541	GO:0019222	<1e-4	slr5018	GO:0015077	<1e-4	slr1122	GO:0044446	<1e-4	sll1696	GO:0019538	0.0139
slr0725	GO:0019752	<1e-4	sll0803	GO:0046394	<1e-4	sll1315	GO:0005342	<1e-4	ssr3571	GO:0044446	<1e-4	sll0710	GO:0019538	0.0139
slr0333	GO:0009132	<1e-4	sll1949	GO:0034654	<1e-4	slr0545	GO:0015075	<1e-4	sll7033	GO:0044444	<1e-4	sll1401	GO:0019538	0.0139
sll0775	GO:0009165	<1e-4	slr0978	GO:0048518	<1e-4	sll1350	GO:0042623	<1e-4	ssr3409	GO:0044464	<1e-4	sll0265	GO:0019538	0.0139
sll1411	GO:0043436	<1e-4	sll1119	GO:0006753	<1e-4	ssr2962	GO:0022803	<1e-4	sll0703	GO:0005575	<1e-4	sll1722	GO:0019538	0.0139
sll0780	GO:0046483	<1e-4	slr1958	GO:0051246	<1e-4	slr1565	GO:0015405	<1e-4	slr0076	GO:0044424	<1e-4	slr1384	GO:0019538	0.0139
slr1327	GO:0009991	<1e-4	slr1032	GO:0042451	<1e-4	sll1722	GO:0016817	<1e-4	sll0565	GO:0044422	<1e-4	sll5026	GO:0019538	0.0139
slr1396	GO:0055082	<1e-4	slr0305	GO:0034654	<1e-4	ssr6086	GO:0016817	<1e-4	slr1907	GO:0005575	<1e-4	slr1161	GO:0019538	0.0139
sll0691	GO:0009056	<1e-4	slr6067	GO:0009126	<1e-4	sll0085	GO:0022857	<1e-4	slr0038	GO:0005575	<1e-4	slr0168	GO:0019538	0.0139
slr1045	GO:0051716	<1e-4	ssr5020	GO:0009124	<1e-4	slr1977	GO:0015399	<1e-4	slr1301	GO:0044446	<1e-4	sll1355	GO:0019538	0.0139
slr1534	GO:0019751	<1e-4	slr0238	GO:0055114	<1e-4	slr0058	GO:0005342	<1e-4	slr5111	GO:0044446	<1e-4	sll0423	GO:0019538	0.0139
sll0847	GO:0019751	<1e-4	slr0579	GO:0009132	<1e-4	ssl5095	GO:0022803	<1e-4	sll0539	GO:0005575	<1e-4	sll0473	GO:0019538	0.0139
sll1765	GO:0051171	<1e-4	slr0270	GO:0006091	<1e-4	ssl1498	GO:0015293	<1e-4	sll1660	GO:0044444	<1e-4	ssl2717	GO:0019538	0.0139

ssl3379	GO:0042180	<1e-4	slr2011	GO:0080090	<1e-4	slr1534	GO:0022838	<1e-4	slr1964	GO:0044446	<1e-4	slr0769	GO:0019538	0.0139
sll1785	GO:0051179	<1e-4	sll0436	GO:0016054	<1e-4	sll1006	GO:0015075	<1e-4	slr0169	GO:0043226	<1e-4	sll1348	GO:0019538	0.0139
slr0458	GO:0009889	<1e-4	slr1223	GO:0031323	<1e-4	slr1690	GO:0016741	<1e-4	sll1715	GO:0044446	<1e-4	slr0606	GO:0019538	0.0139
ssr0335	GO:0046128	<1e-4	ssl3379	GO:0046128	<1e-4	slr6033	GO:0015291	<1e-4	sll0743	GO:0044444	<1e-4	ssr1951	GO:0006396	0.0137
sll0565	GO:0044260	<1e-4	slr1547	GO:0042180	<1e-4	sll1722	GO:0015077	<1e-4	ssr3189	GO:0044444	<1e-4	ssl3451	GO:0006396	0.0137
sll8032	GO:0009308	<1e-4	slr0358	GO:0009987	<1e-4	slr7091	GO:0022857	<1e-4	sll1775	GO:0044444	<1e-4	sll0545	GO:0006396	0.0137
sll0263	GO:0006793	<1e-4	sll0590	GO:0072521	<1e-4	slr0725	GO:0022832	<1e-4	sll1853	GO:0044422	<1e-4	ssl5099	GO:0006396	0.0137
slr0699	GO:0009394	<1e-4	slr5118	GO:0034654	<1e-4	ssr3129	GO:0016818	<1e-4	ssr1041	GO:0044464	<1e-4	sll0167	GO:0006396	0.0137
sll1130	GO:0006631	<1e-4	sll0736	GO:0055086	<1e-4	sll0558	GO:0015075	<1e-4	sll1025	GO:0043226	<1e-4	slr7014	GO:0006396	0.0137
sll1570	GO:0009260	<1e-4	slr1774	GO:0080090	<1e-4	slr0144	GO:0016741	<1e-4	ssr6085	GO:0044422	<1e-4	sll1378	GO:0006396	0.0137
slr0519	GO:0046128	<1e-4	ssl0832	GO:0071841	<1e-4	ssl8003	GO:0016741	<1e-4	slr2103	GO:0044422	<1e-4	sll1252	GO:0006396	0.0137
slr0651	GO:0009308	<1e-4	slr5016	GO:0008150	<1e-4	ssl0832	GO:0022857	<1e-4	slr1854	GO:0044424	<1e-4	sll0436	GO:0006396	0.0137
slr1034	GO:0046164	<1e-4	sll1252	GO:0016054	<1e-4	sll1961	GO:0015267	<1e-4	slr8021	GO:0005575	<1e-4	slr1472	GO:0006396	0.0137
sll0837	GO:0009123	<1e-4	slr0184	GO:0016052	<1e-4	sll1761	GO:0060089	<1e-4	sll0445	GO:0044444	<1e-4	ssr6079	GO:0006396	0.0137
ssl2920	GO:0046164	<1e-4	slr1535	GO:0009141	<1e-4	slr1977	GO:0022838	<1e-4	slr6005	GO:0044424	<1e-4	slr0569	GO:0006396	0.0137
sll0590	GO:0009260	<1e-4	sll1239	GO:0009132	<1e-4	slr0269	GO:0043492	<1e-4	sll5090	GO:0044446	<1e-4	sll0487	GO:0006396	0.0137
sll0176	GO:0052111	<1e-4	ssl3692	GO:0006733	<1e-4	slr7025	GO:0008171	<1e-4	slr1534	GO:0044422	<1e-4	slr0146	GO:0006396	0.0137
slr0709	GO:0009893	<1e-4	slr1915	GO:0055114	<1e-4	sll1233	GO:0042623	<1e-4	slr0142	GO:0044446	<1e-4	slr1568	GO:0006396	0.0137
slr0634	GO:0071842	<1e-4	slr0890	GO:0009132	<1e-4	sll1715	GO:0015399	<1e-4	ssl1792	GO:0044444	<1e-4	slr7095	GO:0006396	0.0137
sll0875	GO:0019751	<1e-4	sll0676	GO:0006766	<1e-4	slr1699	GO:0015077	<1e-4	slr1273	GO:0005575	<1e-4	slr0971	GO:0006396	0.0137
ssr2611	GO:0006163	<1e-4	ssr6083	GO:0009141	<1e-4	slr1178	GO:0015291	<1e-4	ssr6086	GO:0005575	<1e-4	sll0448	GO:0006396	0.0137
sll1656	GO:0016054	<1e-4	sll0174	GO:0071496	<1e-4	sll1965	GO:0022836	<1e-4	slr1087	GO:0005575	<1e-4	sll0872	GO:0006396	0.0137
ssr1114	GO:0051716	<1e-4	sll5028	GO:0072524	<1e-4	slr1102	GO:0042623	<1e-4	slr1087	GO:0044424	<1e-4	sll2013	GO:0006396	0.0137
sll0238	GO:0048878	<1e-4	sll1380	GO:0009059	<1e-4	sll7087	GO:0015267	<1e-4	slr6007	GO:0044424	<1e-4	ssl5095	GO:0006396	0.0137
sll0781	GO:0044260	<1e-4	sll1722	GO:0019222	<1e-4	sll1979	GO:0005342	<1e-4	sll1509	GO:0044422	<1e-4	slr0264	GO:0006396	0.0137
slr1069	GO:0009259	<1e-4	ssr0761	GO:0043933	<1e-4	slr1342	GO:0003674	<1e-4	sll0148	GO:0044425	<1e-4	sll1638	GO:0006396	0.0137
slr2071	GO:0048523	<1e-4	ssl2384	GO:0051171	<1e-4	ssr7084	GO:0015075	<1e-4	sll0867	GO:0043226	<1e-4	ssl1417	GO:0006396	0.0137
ssl2749	GO:0009141	<1e-4	sll0803	GO:0009142	<1e-4	sll0230	GO:0022891	<1e-4	sll1608	GO:0005575	<1e-4	sll0008	GO:0006396	0.0137
slr2010	GO:0050789	<1e-4	sll1832	GO:0051179	<1e-4	sll0230	GO:0016746	<1e-4	ssl3573	GO:0044464	<1e-4	sll5032	GO:0006396	0.0137
sll0863	GO:0052188	<1e-4	slr1819	GO:0019219	<1e-4	sll1630	GO:0022803	<1e-4	slr0755	GO:0044464	<1e-4	ssr0102	GO:0006396	0.0137
sll1763	GO:0050789	<1e-4	slr1670	GO:0044106	<1e-4	ssr7072	GO:0015293	<1e-4	slr1340	GO:0044425	<1e-4	ssr0335	GO:0006396	0.0137
sll0066	GO:0033013	<1e-4	sll1352	GO:0065007	<1e-4	sll1563	GO:0015399	<1e-4	slr1811	GO:0005575	<1e-4	ssl0312	GO:0006396	0.0137
sll0172	GO:0019752	<1e-4	slr6005	GO:0009259	<1e-4	slr1601	GO:0022803	<1e-4	slr2037	GO:0043226	<1e-4	slr1306	GO:0006396	0.0137
slr0885	GO:0009987	<1e-4	ssr5020	GO:0051188	<1e-4	sll0060	GO:0016817	<1e-4	sll1938	GO:0044425	<1e-4	sll0762	GO:0006396	0.0137
ssr7072	GO:0051188	<1e-4	sll1680	GO:0009987	<1e-4	slr1396	GO:0015267	<1e-4	slr1618	GO:0044444	<1e-4	slr0038	GO:0006396	0.0137
sll1381	GO:0034660	<1e-4	slr1547	GO:0055086	<1e-4	slr1611	GO:0043167	<1e-4	ssl7038	GO:0044464	<1e-4	sll0913	GO:0006396	0.0137
slr1863	GO:0009893	<1e-4	slr1896	GO:0044106	<1e-4	sll0847	GO:0043492	<1e-4	sll5089	GO:0044444	<1e-4	slr1365	GO:0006396	0.0137
ssl2245	GO:0072521	<1e-4	slr0291	GO:0019222	<1e-4	slr1023	GO:0015291	<1e-4	sll0780	GO:0044425	<1e-4	slr2118	GO:0006396	0.0137
slr0930	GO:0072522	<1e-4	slr0294	GO:0050789	<1e-4	ssr2998	GO:0022836	<1e-4	slr0655	GO:0043226	<1e-4	sll5097	GO:0006396	0.0137
ssr2781	GO:0009123	<1e-4	sll1702	GO:0009394	<1e-4	slr0241	GO:0005342	<1e-4	slr0491	GO:0044464	<1e-4	slr0145	GO:0006396	0.0137
slr0692	GO:0006163	<1e-4	slr1168	GO:0051179	<1e-4	sll0473	GO:0015291	<1e-4	slr6065	GO:0044446	<1e-4	slr1814	GO:0006396	0.0137
slr1577	GO:0048518	<1e-4	ssl0788	GO:0018130	<1e-4	sll0479	GO:0022892	<1e-4	slr1034	GO:0043226	<1e-4	slr1470	GO:0006396	0.0137
sll1131	GO:0044283	<1e-4	sll0230	GO:0055114	<1e-4	slr0521	GO:0016741	<1e-4	ssl0294	GO:0043226	<1e-4	sll1062	GO:0006396	0.0137
ssr2972	GO:0006753	<1e-4	slr1097	GO:0006811	<1e-4	sll1882	GO:0022803	<1e-4	sll1702	GO:0044464	<1e-4	sll0168	GO:0006396	0.0137
slr0006	GO:0016052	<1e-4	slr0521	GO:0043549	<1e-4	sll0141	GO:0015077	<1e-4	slr7092	GO:0044464	<1e-4	slr1464	GO:0006396	0.0137
ssl5025	GO:0009394	<1e-4	slr1737	GO:0050801	<1e-4	slr0664	GO:0008171	<1e-4	slr0105	GO:0044422	<1e-4	slr7097	GO:0006396	0.0137
sll0412	GO:0044248	<1e-4	sll7034	GO:0043549	<1e-4	slr0912	GO:0022832	<1e-4	sll1006	GO:0044444	<1e-4	slr1178	GO:0006396	0.0137
sll1832	GO:0072527	<1e-4	ssr2333	GO:0042451	<1e-4	slr1505	GO:0015405	<1e-4	ssr2333	GO:0044444	<1e-4	ssl0750	GO:0016772	0.0137
slr0769	GO:0071841	<1e-4	sll1340	GO:0034654	<1e-4	slr1383	GO:0022857	<1e-4	sll8007	GO:0043226	<1e-4	slr1812	GO:0016740	0.0136
sll1527	GO:0071841	<1e-4	sll1769	GO:0019752	<1e-4	slr1601	GO:0022892	<1e-4	slr0459	GO:0044425	<1e-4	sll0413	GO:0016740	0.0136
sll1060	GO:0009893	<1e-4	sll0445	GO:0044260	<1e-4	slr0232	GO:0022832	<1e-4	slr2038	GO:0044425	<1e-4	slr1811	GO:0016874	0.0135
sll1381	GO:0050801	<1e-4	slr1152	GO:0071841	<1e-4	slr0076	GO:0043492	<1e-4	slr0058	GO:0005575	<1e-4	slr0978	GO:0016874	0.0135
slr1611	GO:0046164	<1e-4	slr5012	GO:0046394	<1e-4	sll7031	GO:0060089	<1e-4	slr0103	GO:0044424	<1e-4	slr1098	GO:0016874	0.0135
sll7031	GO:0009262	<1e-4	slr0294	GO:0009893	<1e-4	slr1398	GO:0022892	<1e-4	ssl1417	GO:0044422	<1e-4	slr1851	GO:0016874	0.0135

sll0156	GO:0055086	<1e-4	slr1338	GO:0071841	<1e-4	ssl5095	GO:0043167	<1e-4	slr1394	GO:0044464	<1e-4	slr0909	GO:0016874	0.0135
slr0937	GO:0044271	<1e-4	slr1628	GO:0019637	<1e-4	sll0282	GO:0022838	<1e-4	slr1161	GO:0005575	<1e-4	slr1767	GO:0016874	0.0135
sll1054	GO:0071841	<1e-4	sll1193	GO:0022607	<1e-4	sll1882	GO:0005342	<1e-4	sll1609	GO:0044424	<1e-4	sll0361	GO:0016874	0.0135
sll0327	GO:0043436	<1e-4	slr2010	GO:0009165	<1e-4	sll1123	GO:0060089	<1e-4	sll0446	GO:0043226	<1e-4	sll0910	GO:0016874	0.0135
sll0443	GO:0042180	<1e-4	sll1571	GO:0009142	<1e-4	sll0497	GO:0015293	<1e-4	sll1562	GO:0043226	<1e-4	slr0589	GO:0016874	0.0135
slr0554	GO:0009117	<1e-4	slr0169	GO:0043648	<1e-4	sll1862	GO:0016818	<1e-4	sll1488	GO:0044444	<1e-4	slr1800	GO:0016874	0.0135
slr0303	GO:0050801	<1e-4	sll1562	GO:0009892	<1e-4	sll0168	GO:0022890	<1e-4	sll0451	GO:0005575	<1e-4	slr0263	GO:0016874	0.0135
slr1866	GO:0050794	<1e-4	sll7066	GO:0044106	<1e-4	slr1790	GO:0015267	<1e-4	sll0174	GO:0044446	<1e-4	sll0414	GO:0019538	0.0133
sll1769	GO:0009142	<1e-4	slr1851	GO:0044271	<1e-4	sll0763	GO:0015399	<1e-4	slr0272	GO:0044422	<1e-4	slr0872	GO:0019538	0.0133
slr7100	GO:0051186	<1e-4	sll0623	GO:0060255	<1e-4	sll0615	GO:0022836	<1e-4	slr2018	GO:0005575	<1e-4	sll1632	GO:0019538	0.0133
sll0751	GO:0044260	<1e-4	ssr2060	GO:0034654	<1e-4	sll1021	GO:0022890	<1e-4	sll1109	GO:0005575	<1e-4	slr1618	GO:0019538	0.0133
slr1275	GO:0044275	<1e-4	ssr6002	GO:0044003	<1e-4	sll0176	GO:0016746	<1e-4	slr8014	GO:0044422	<1e-4	ssr3571	GO:0019538	0.0133
slr1391	GO:0051716	<1e-4	sll8040	GO:0052188	<1e-4	slr1210	GO:0042623	<1e-4	sll1446	GO:0044424	<1e-4	ssr2551	GO:0019538	0.0133
sll0751	GO:0009893	<1e-4	slr6051	GO:0072527	<1e-4	slr1544	GO:0016462	<1e-4	ssl3451	GO:0044464	<1e-4	sll0198	GO:0019538	0.0133
slr0625	GO:0050801	<1e-4	slr1957	GO:0031323	<1e-4	slr6090	GO:0008171	<1e-4	sll1681	GO:0043226	<1e-4	sll0237	GO:0019538	0.0133
sll1004	GO:0009987	<1e-4	slr0509	GO:0052188	<1e-4	ssr3129	GO:0022832	<1e-4	ssr7017	GO:0044446	<1e-4	slr2073	GO:0019538	0.0133
sll0168	GO:0042180	<1e-4	sll0369	GO:0008150	<1e-4	sll1174	GO:0043167	<1e-4	sll0157	GO:0005575	<1e-4	ssr2439	GO:0019538	0.0133
ssr2318	GO:0006066	<1e-4	slr1778	GO:0010468	<1e-4	ssr6030	GO:0016817	<1e-4	sll0031	GO:0044424	<1e-4	slr0913	GO:0019538	0.0133
slr1222	GO:0019362	<1e-4	slr1547	GO:0071840	<1e-4	ssl0738	GO:0060089	<1e-4	sll0189	GO:0044422	<1e-4	slr1073	GO:0019538	0.0133
sll1486	GO:0080090	<1e-4	ssr5106	GO:0044260	<1e-4	sll1891	GO:0022838	<1e-4	sll5026	GO:0005575	<1e-4	slr1676	GO:0019538	0.0133
slr0930	GO:0009987	<1e-4	ssr6089	GO:0009132	<1e-4	slr0907	GO:0022857	<1e-4	slr1814	GO:0044446	<1e-4	slr2011	GO:0019538	0.0133
slr7083	GO:0009126	<1e-4	sll1304	GO:0009262	<1e-4	slr2119	GO:0015293	<1e-4	slr0773	GO:0044425	<1e-4	slr1230	GO:0019538	0.0133
sll0564	GO:0016052	<1e-4	sll0847	GO:0009987	<1e-4	slr1601	GO:0022857	<1e-4	slr8021	GO:0044446	<1e-4	slr0668	GO:0019538	0.0133
sll1355	GO:0044260	<1e-4	slr6063	GO:0072527	<1e-4	sll1582	GO:0015291	<1e-4	slr1079	GO:0043226	<1e-4	slr1436	GO:0019538	0.0133
sll1775	GO:0051818	<1e-4	ssr3129	GO:0042180	<1e-4	slr1920	GO:0043492	<1e-4	ssl1464	GO:0005575	<1e-4	sll1949	GO:0019538	0.0133
sll0756	GO:0042180	<1e-4	sll0372	GO:0009165	<1e-4	slr0476	GO:0022836	<1e-4	sll1390	GO:0044425	<1e-4	ssr2615	GO:0019538	0.0133
sll0846	GO:0009059	<1e-4	slr0914	GO:0051179	<1e-4	ssl0787	GO:0015293	<1e-4	sll1485	GO:0044464	<1e-4	sll0325	GO:0019538	0.0133
slr0655	GO:0072528	<1e-4	slr0480	GO:0009259	<1e-4	slr1935	GO:0015077	<1e-4	sll0282	GO:0044444	<1e-4	slr1573	GO:0019538	0.0133
slr1907	GO:0044283	<1e-4	slr0845	GO:0080090	<1e-4	slr0579	GO:0015293	<1e-4	sll1658	GO:0005575	<1e-4	sll1656	GO:0019538	0.0133
sll0996	GO:0009124	<1e-4	slr1590	GO:0044260	<1e-4	slr1338	GO:0015405	<1e-4	sll1006	GO:0043226	<1e-4	slr1177	GO:0019538	0.0133
sll0482	GO:0048518	<1e-4	sll0296	GO:0006631	<1e-4	slr1726	GO:0022890	<1e-4	slr1474	GO:0044422	<1e-4	slr0360	GO:0019538	0.0133
ssl6061	GO:0034654	<1e-4	sll1630	GO:0071496	<1e-4	sll1109	GO:0022892	<1e-4	sll0238	GO:0005575	<1e-4	ssr1765	GO:0019538	0.0133
slr5023	GO:0055114	<1e-4	sll1586	GO:0055114	<1e-4	sll0252	GO:0016818	<1e-4	sll0253	GO:0044424	<1e-4	slr0364	GO:0019538	0.0133
sll0588	GO:0006753	<1e-4	ssl2064	GO:0006733	<1e-4	sll1512	GO:0060089	<1e-4	sll0932	GO:0005575	<1e-4	slr0404	GO:0019538	0.0133
ssr7035	GO:0055086	<1e-4	slr7037	GO:0009165	<1e-4	slr1383	GO:0022890	<1e-4	slr7025	GO:0044422	<1e-4	slr0006	GO:0019538	0.0133
slr0142	GO:0071840	<1e-4	sll1505	GO:0006721	<1e-4	slr1865	GO:0016462	<1e-4	slr1117	GO:0044424	<1e-4	slr0386	GO:0019538	0.0133
ssl7039	GO:0072528	<1e-4	slr1266	GO:0048518	<1e-4	slr1287	GO:0003674	<1e-4	sll0062	GO:0044425	<1e-4	sll1262	GO:0019538	0.0133
slr0147	GO:0009123	<1e-4	sll1068	GO:0044282	<1e-4	slr1450	GO:0022891	<1e-4	slr1812	GO:0044425	<1e-4	slr0204	GO:0019538	0.0133
slr1854	GO:0009117	<1e-4	slr0392	GO:0051347	<1e-4	sll0802	GO:0043492	<1e-4	slr0587	GO:0044446	<1e-4	sll0355	GO:0019538	0.0133
sll1880	GO:0060255	<1e-4	sll0394	GO:0009260	<1e-4	slr0890	GO:0022857	<1e-4	slr6009	GO:0005575	<1e-4	sll1761	GO:0019538	0.0133
slr2084	GO:0009259	<1e-4	slr1940	GO:0044271	<1e-4	slr1657	GO:0015267	<1e-4	slr1270	GO:0005575	<1e-4	slr1638	GO:0019538	0.0133
slr1613	GO:0051347	<1e-4	slr1926	GO:0044260	<1e-4	slr0959	GO:0016741	<1e-4	sll1531	GO:0005575	<1e-4	sll0066	GO:0019538	0.0133
slr8021	GO:0072521	<1e-4	ssr6020	GO:0009132	<1e-4	sll0445	GO:0022803	<1e-4	slr6080	GO:0044424	<1e-4	slr1084	GO:0019538	0.0133
sll0216	GO:0016054	<1e-4	sll1509	GO:0008150	<1e-4	ssl5096	GO:0016741	<1e-4	sll0442	GO:0044444	<1e-4	sll0871	GO:0006396	0.0132
ssl8039	GO:0044282	<1e-4	ssr6030	GO:0001932	<1e-4	slr2000	GO:0015291	<1e-4	slr0351	GO:0044425	<1e-4	slr2005	GO:0006396	0.0132
ssr6099	GO:0009263	<1e-4	slr5102	GO:0006766	<1e-4	slr0482	GO:0022892	<1e-4	sll0098	GO:0044446	<1e-4	slr1074	GO:0006396	0.0132
slr0967	GO:0034654	<1e-4	sll1411	GO:0048518	<1e-4	sll8033	GO:0015293	<1e-4	sll1089	GO:0005575	<1e-4	sll0614	GO:0006396	0.0132
slr7096	GO:0006733	<1e-4	ssl1577	GO:0055086	<1e-4	slr7101	GO:0005342	<1e-4	slr0587	GO:0005575	<1e-4	slr1383	GO:0006396	0.0132
slr1189	GO:0080090	<1e-4	sll0787	GO:0019222	<1e-4	slr0112	GO:0022832	<1e-4	sll1344	GO:0044422	<1e-4	sll0268	GO:0006396	0.0132
slr0975	GO:0050801	<1e-4	slr2011	GO:0060255	<1e-4	sll1737	GO:0022832	<1e-4	sll0423	GO:0044446	<1e-4	slr1209	GO:0006396	0.0132
slr1287	GO:0048522	<1e-4	sll8012	GO:0044283	<1e-4	sll0265	GO:0022857	<1e-4	sll0238	GO:0044425	<1e-4	sll0669	GO:0006396	0.0132
slr0207	GO:0071554	<1e-4	sll1174	GO:0051246	<1e-4	slr0151	GO:0015267	<1e-4	slr0076	GO:0005575	<1e-4	slr0172	GO:0006396	0.0132
sll0864	GO:0006576	<1e-4	sll0780	GO:0009141	<1e-4	sll1318	GO:0015075	<1e-4	slr0169	GO:0044424	<1e-4	sll0319	GO:0006396	0.0132
sll1052	GO:0065007	<1e-4	slr0695	GO:0019222	<1e-4	sll1757	GO:0015405	<1e-4	slr1056	GO:0044444	<1e-4	slr2080	GO:0006396	0.0132

sll1217	GO:0055086	<1e-4	sll0547	GO:0009889	<1e-4	sll1726	GO:0008171	<1e-4	slr0852	GO:0044422	<1e-4	slr1852	GO:0006396	0.0132
sll1866	GO:0009057	<1e-4	sll0335	GO:0009259	<1e-4	sll1401	GO:0003674	<1e-4	sll0095	GO:0044425	<1e-4	slr0053	GO:0006396	0.0132
slr1470	GO:0043436	<1e-4	slr1790	GO:0009123	<1e-4	ssl2595	GO:0043167	<1e-4	slr1896	GO:0044464	<1e-4	sll1390	GO:0006396	0.0132
sll0188	GO:0009991	<1e-4	ssl1972	GO:0019752	<1e-4	slr1614	GO:0016818	<1e-4	sll5128	GO:0044446	<1e-4	slr0358	GO:0006396	0.0132
slr2119	GO:0051818	<1e-4	slr1913	GO:0050801	<1e-4	sll0995	GO:0005342	<1e-4	sll1053	GO:0044424	<1e-4	slr0272	GO:0006396	0.0132
slr1807	GO:0044248	<1e-4	slr1215	GO:0051179	<1e-4	slr1603	GO:0008171	<1e-4	sll8027	GO:0005575	<1e-4	sll1634	GO:0006396	0.0132
slr0345	GO:0022411	<1e-4	ssl0294	GO:0006721	<1e-4	slr2003	GO:0022838	<1e-4	ssl0787	GO:0044446	<1e-4	slr0975	GO:0006396	0.0132
sll0822	GO:0071841	<1e-4	slr0271	GO:0009262	<1e-4	slr1915	GO:0015291	<1e-4	sll0294	GO:0044446	<1e-4	slr0060	GO:0006396	0.0132
ssr3410	GO:0006066	<1e-4	slr1647	GO:0009123	<1e-4	sll1340	GO:0042623	<1e-4	ssr6024	GO:0005575	<1e-4	sll0539	GO:0006396	0.0132
sll1388	GO:0072521	<1e-4	slr1658	GO:0051716	<1e-4	sll1510	GO:0022838	<1e-4	sll0858	GO:0044425	<1e-4	slr1415	GO:0006396	0.0132
sll1504	GO:0055114	<1e-4	slr0407	GO:0046483	<1e-4	slr6033	GO:0016746	<1e-4	ssr5074	GO:0044464	<1e-4	slr1152	GO:0006396	0.0132
slr1704	GO:0043648	<1e-4	slr0848	GO:0006082	<1e-4	slr1188	GO:0015293	<1e-4	slr1095	GO:0044425	<1e-4	slr0104	GO:0006396	0.0132
sll0984	GO:0009889	<1e-4	ssl1378	GO:0019752	<1e-4	ssl3382	GO:0016818	<1e-4	slr1935	GO:0044444	<1e-4	ssl0467	GO:0042626	0.0132
sll0062	GO:0044275	<1e-4	ssr1155	GO:0044248	<1e-4	slr1702	GO:0015291	<1e-4	sll0678	GO:0044446	<1e-4	sll1476	GO:0042626	0.0132
slr0582	GO:0009893	<1e-4	slr0869	GO:0034641	<1e-4	slr1599	GO:0005342	<1e-4	sll0839	GO:0044425	<1e-4	slr1577	GO:0042626	0.0132
sll1765	GO:0050801	<1e-4	ssr6026	GO:0008150	<1e-4	sll1164	GO:0022891	<1e-4	slr0455	GO:0005575	<1e-4	ssl2162	GO:0042626	0.0132
slr1944	GO:0009161	<1e-4	sll0787	GO:0009150	<1e-4	sll0309	GO:0060089	<1e-4	ssl2814	GO:0005575	<1e-4	slr0816	GO:0042626	0.0132
sll0183	GO:0009262	<1e-4	slr1541	GO:0006721	<1e-4	sll0103	GO:0060089	<1e-4	sll7090	GO:0044424	<1e-4	slr1628	GO:0042626	0.0132
sll0208	GO:0044260	<1e-4	ssl3692	GO:0006163	<1e-4	slr1353	GO:0022892	<1e-4	sll1130	GO:0044464	<1e-4	ssr6002	GO:0042626	0.0132
slr0937	GO:0034641	<1e-4	sll0775	GO:0080090	<1e-4	ssr0536	GO:0022836	<1e-4	sll1092	GO:0005575	<1e-4	sll0552	GO:0042626	0.0132
slr1591	GO:0072528	<1e-4	ssl2595	GO:0008150	<1e-4	slr6028	GO:0015077	<1e-4	slr2046	GO:0044422	<1e-4	ssr2975	GO:0042626	0.0132
slr0919	GO:0050801	<1e-4	sll5034	GO:0009889	<1e-4	sll0294	GO:0016818	<1e-4	slr2004	GO:0005575	<1e-4	ssr0109	GO:0042626	0.0132
slr2110	GO:0055086	<1e-4	ssr0759	GO:0031323	<1e-4	sll0505	GO:0015293	<1e-4	slr0453	GO:0005575	<1e-4	sll0160	GO:0042626	0.0132
ssr2843	GO:0010556	<1e-4	slr1819	GO:0006163	<1e-4	slr2010	GO:0015405	<1e-4	ssr1558	GO:0005575	<1e-4	slr1173	GO:0042626	0.0132
slr0059	GO:0008610	<1e-4	slr0582	GO:0072522	<1e-4	slr5023	GO:0016462	<1e-4	slr0262	GO:0005575	<1e-4	sll2011	GO:0042626	0.0132
slr1260	GO:0006720	<1e-4	slr0935	GO:0009987	<1e-4	slr1619	GO:0015399	<1e-4	slr0304	GO:0044444	<1e-4	sll5132	GO:0042626	0.0132
sll1071	GO:0051234	<1e-4	slr0610	GO:0009059	<1e-4	slr6044	GO:0008171	<1e-4	slr0498	GO:0044422	<1e-4	sll1570	GO:0042626	0.0132
ssr6085	GO:0034660	<1e-4	slr6044	GO:0051186	<1e-4	slr0740	GO:0043492	<1e-4	slr2027	GO:0043226	<1e-4	slr0645	GO:0042626	0.0132
slr7037	GO:0044275	<1e-4	slr1737	GO:0072524	<1e-4	sll0846	GO:0015267	<1e-4	ssl2148	GO:0044446	<1e-4	ssr2060	GO:0042626	0.0132
slr0503	GO:0009262	<1e-4	slr6066	GO:0055086	<1e-4	slr0211	GO:0016817	<1e-4	sll1163	GO:0005575	<1e-4	slr0334	GO:0042626	0.0132
ssr1391	GO:0080090	<1e-4	ssl2807	GO:0022411	<1e-4	slr0318	GO:0043167	<1e-4	slr0109	GO:0044422	<1e-4	ssr2553	GO:0042626	0.0132
slr0579	GO:0050896	<1e-4	sll1106	GO:0044281	<1e-4	slr0510	GO:0016741	<1e-4	sll8011	GO:0044444	<1e-4	slr0285	GO:0042626	0.0132
slr0217	GO:0042451	<1e-4	sll0608	GO:0080090	<1e-4	sll1632	GO:0043167	<1e-4	slr0912	GO:0044424	<1e-4	slr6091	GO:0042626	0.0132
slr0358	GO:0055086	<1e-4	sll1240	GO:0006576	<1e-4	slr0468	GO:0022892	<1e-4	sll1946	GO:0005575	<1e-4	slr8021	GO:0042626	0.0132
slr1098	GO:0044282	<1e-4	slr0058	GO:0051188	<1e-4	sll5033	GO:0022892	<1e-4	sll0098	GO:0005575	<1e-4	ssr8013	GO:0042626	0.0132
ssl7042	GO:0009056	<1e-4	sll1378	GO:0006163	<1e-4	sll0010	GO:0060089	<1e-4	slr1827	GO:0005575	<1e-4	sll5003	GO:0042626	0.0132
slr1886	GO:0009987	<1e-4	sll1372	GO:0006576	<1e-4	ssl1498	GO:0022892	<1e-4	sll0172	GO:0044446	<1e-4	ssl7045	GO:0042626	0.0132
sll1131	GO:0055082	<1e-4	slr6047	GO:0031640	<1e-4	slr1327	GO:0015405	<1e-4	slr0392	GO:0044422	<1e-4	slr0651	GO:0042626	0.0132
sll0886	GO:0006631	<1e-4	slr0919	GO:0019751	<1e-4	ssl1464	GO:0022803	<1e-4	slr1206	GO:0044425	<1e-4	sll1142	GO:0042626	0.0132
ssl3142	GO:0051179	<1e-4	ssr2422	GO:0031326	<1e-4	sll0793	GO:0022803	<1e-4	sll0479	GO:0043226	<1e-4	slr0325	GO:0042626	0.0132
slr1927	GO:0009059	<1e-4	slr1307	GO:0009889	<1e-4	ssr6048	GO:0005342	<1e-4	slr1990	GO:0043226	<1e-4	slr1780	GO:0042626	0.0132
ssr2009	GO:0043412	<1e-4	slr6049	GO:0019219	<1e-4	slr6033	GO:0016741	<1e-4	slr0885	GO:0044444	<1e-4	ssr6024	GO:0042626	0.0132
sll0360	GO:0031326	<1e-4	slr1303	GO:0080090	<1e-4	slr1944	GO:0005342	<1e-4	slr0064	GO:0044446	<1e-4	slr0521	GO:0042626	0.0132
sll7069	GO:0044260	<1e-4	sll0553	GO:0033013	<1e-4	sll0243	GO:0015293	<1e-4	sll1486	GO:0043226	<1e-4	slr1507	GO:0042626	0.0132
slr0924	GO:0009123	<1e-4	slr0712	GO:0051179	<1e-4	sll1562	GO:0022891	<1e-4	slr1619	GO:0044425	<1e-4	slr0509	GO:0042626	0.0132
sll0382	GO:0044275	<1e-4	slr1537	GO:0008150	<1e-4	sll1638	GO:0015077	<1e-4	sll1191	GO:0005575	<1e-4	sll1882	GO:0042626	0.0132
slr0109	GO:0044281	<1e-4	sll0497	GO:0005996	<1e-4	sll0843	GO:0022836	<1e-4	sll1606	GO:0044424	<1e-4	ssl2971	GO:0042626	0.0132
sll1965	GO:0008150	<1e-4	slr1074	GO:0009165	<1e-4	ssl0312	GO:0022803	<1e-4	sll1158	GO:0043226	<1e-4	slr2012	GO:0042626	0.0132
slr0305	GO:0005996	<1e-4	slr1780	GO:0046164	<1e-4	sll0282	GO:0005342	<1e-4	slr1230	GO:0044425	<1e-4	slr7071	GO:0042626	0.0132
ssl7039	GO:0072521	<1e-4	slr5013	GO:0009987	<1e-4	slr0552	GO:0022891	<1e-4	ssl5095	GO:0005575	<1e-4	ssr1473	GO:0042626	0.0132
sll0861	GO:0044271	<1e-4	slr0978	GO:0006066	<1e-4	ssl2471	GO:0015293	<1e-4	sll1880	GO:0044446	<1e-4	ssr7035	GO:0042626	0.0132
slr1813	GO:0009059	<1e-4	ssr2972	GO:0044106	<1e-4	slr1827	GO:0015293	<1e-4	slr1327	GO:0044444	<1e-4	slr0039	GO:0042626	0.0132
ssr1258	GO:0006766	<1e-4	sll0216	GO:0050896	<1e-4	sll0242	GO:0016741	<1e-4	slr1659	GO:0044422	<1e-4	slr1819	GO:0042626	0.0132
slr1647	GO:0044255	<1e-4	slr1275	GO:0009259	<1e-4	sll5128	GO:0042623	<1e-4	ssr3341	GO:0044444	<1e-4	slr7094	GO:0042626	0.0132

sll5030	GO:0006066	<1e-4	ssl2807	GO:0009056	<1e-4	slr1657	GO:0016746	<1e-4	sll5130	GO:0044424	<1e-4	ssl5103	GO:0042626	0.0132
slr6071	GO:0009263	<1e-4	sll0996	GO:0022607	<1e-4	slr1444	GO:0016818	<1e-4	sll0606	GO:0044444	<1e-4	sll1373	GO:0042626	0.0132
slr1223	GO:0050794	<1e-4	ssr3571	GO:0006753	<1e-4	sll0335	GO:0043492	<1e-4	slr1535	GO:0044425	<1e-4	slr0667	GO:0042626	0.0132
slr6081	GO:0031323	<1e-4	ssr2615	GO:0009893	<1e-4	slr0582	GO:0015405	<1e-4	slr1082	GO:0044422	<1e-4	slr7091	GO:0042626	0.0132
sll7087	GO:0006753	<1e-4	slr1616	GO:0071496	<1e-4	ssl1046	GO:0022803	<1e-4	sll0827	GO:0044425	<1e-4	sll0765	GO:0042626	0.0132
sll1188	GO:0009987	<1e-4	slr5012	GO:0009124	<1e-4	slr0695	GO:0042623	<1e-4	slr0598	GO:0005575	<1e-4	slr0588	GO:0042626	0.0132
sll0532	GO:0072522	<1e-4	slr1647	GO:0006066	<1e-4	slr6067	GO:0015399	<1e-4	slr1142	GO:0043226	<1e-4	slr0459	GO:0042626	0.0132
ssr2972	GO:0072522	<1e-4	ssr1499	GO:0045184	<1e-4	sll0658	GO:0022836	<1e-4	slr0924	GO:0044425	<1e-4	ssr7079	GO:0042626	0.0132
sll1609	GO:0006220	<1e-4	slr1690	GO:0050794	<1e-4	slr2011	GO:0015405	<1e-4	ssr2060	GO:0044444	<1e-4	sll1532	GO:0042626	0.0132
slr0392	GO:0009132	<1e-4	ssr3189	GO:0006766	<1e-4	ssr5121	GO:0016746	<1e-4	slr0602	GO:0044424	<1e-4	sll0847	GO:0042626	0.0132
sll0181	GO:0042455	<1e-4	sll1915	GO:0034654	<1e-4	sll0735	GO:0015399	<1e-4	ssr6027	GO:0043226	<1e-4	sll1464	GO:0042626	0.0132
slr8044	GO:0006811	<1e-4	sll0860	GO:0009059	<1e-4	sll0160	GO:0015293	<1e-4	sll0031	GO:0044464	<1e-4	sll1486	GO:0042626	0.0132
slr0364	GO:0046907	<1e-4	slr1045	GO:0051818	<1e-4	slr1657	GO:0022803	<1e-4	sll1736	GO:0043226	<1e-4	sll1785	GO:0042626	0.0132
sll1447	GO:0006732	<1e-4	ssl3615	GO:0080090	<1e-4	sll1738	GO:0016818	<1e-4	slr0355	GO:0044444	<1e-4	ssr2802	GO:0042626	0.0132
sll0596	GO:0071496	<1e-4	slr0376	GO:0044255	<1e-4	sll1135	GO:0015405	<1e-4	sll8035	GO:0005575	<1e-4	sll1915	GO:0042626	0.0132
ssr7072	GO:0019222	<1e-4	slr1918	GO:0055114	<1e-4	sll1749	GO:0060089	<1e-4	slr2018	GO:0044446	<1e-4	slr6045	GO:0042626	0.0132
ssr6083	GO:0006720	<1e-4	sll0756	GO:0006720	<1e-4	sll8035	GO:0016462	<1e-4	ssl5091	GO:0044424	<1e-4	sll6055	GO:0042626	0.0132
ssr2439	GO:0051818	<1e-4	slr1378	GO:0044260	<1e-4	sll1884	GO:0016817	<1e-4	sll0749	GO:0044446	<1e-4	slr0082	GO:0042626	0.0132
sll0615	GO:0022411	<1e-4	slr0172	GO:0031640	<1e-4	sll0508	GO:0005342	<1e-4	slr8044	GO:0044444	<1e-4	sll8035	GO:0042626	0.0132
ssl1255	GO:0009126	<1e-4	slr1070	GO:0045184	<1e-4	sll0997	GO:0022892	<1e-4	sll1660	GO:0005575	<1e-4	sll1573	GO:0042626	0.0132
sll0735	GO:0019752	<1e-4	sll0656	GO:0006732	<1e-4	slr1612	GO:0060089	<1e-4	sll0493	GO:0044425	<1e-4	ssl1552	GO:0042626	0.0132
sll0355	GO:0008150	<1e-4	slr1886	GO:0009141	<1e-4	slr1449	GO:0022857	<1e-4	sll0141	GO:0044425	<1e-4	slr0397	GO:0042626	0.0132
sll1477	GO:0051234	<1e-4	slr0521	GO:0043412	<1e-4	slr0184	GO:0016462	<1e-4	sll1092	GO:0044446	<1e-4	ssl6092	GO:0042626	0.0132
ssl2420	GO:0006753	<1e-4	slr1101	GO:0006793	<1e-4	slr6091	GO:0022892	<1e-4	sll0815	GO:0044446	<1e-4	slr0184	GO:0042626	0.0132
sll1702	GO:0050801	<1e-4	slr1362	GO:0055082	<1e-4	sll7070	GO:0022836	<1e-4	sll0898	GO:0044425	<1e-4	slr1449	GO:0042626	0.0132
sll1504	GO:0006066	<1e-4	slr1025	GO:0044003	<1e-4	ssr0692	GO:0022803	<1e-4	slr5024	GO:0044464	<1e-4	sll0670	GO:0042626	0.0132
sll0428	GO:0048519	<1e-4	ssr3341	GO:0043170	<1e-4	sll0505	GO:0016817	<1e-4	sll1766	GO:0044424	<1e-4	slr1681	GO:0042626	0.0132
sll1528	GO:0044003	<1e-4	sll0268	GO:0009150	<1e-4	slr6029	GO:0022891	<1e-4	slr5112	GO:0044464	<1e-4	slr0712	GO:0042626	0.0132
sll1858	GO:0018130	<1e-4	ssr6020	GO:0008610	<1e-4	ssr5020	GO:0016818	<1e-4	slr0334	GO:0005575	<1e-4	sll0376	GO:0042626	0.0132
sll0424	GO:0009987	<1e-4	sll1355	GO:0010468	<1e-4	slr1546	GO:0022890	<1e-4	slr1875	GO:0043226	<1e-4	slr6075	GO:0042626	0.0132
slr6009	GO:0031323	<1e-4	sll1472	GO:0051186	<1e-4	sll0071	GO:0015075	<1e-4	slr1288	GO:0044446	<1e-4	sll0678	GO:0042626	0.0132
slr1162	GO:0006753	<1e-4	slr1261	GO:0043170	<1e-4	sll1586	GO:0022890	<1e-4	sll5089	GO:0005575	<1e-4	ssr3467	GO:0042626	0.0132
sll1389	GO:0050896	<1e-4	slr5077	GO:0051347	<1e-4	sll1350	GO:0015291	<1e-4	slr0217	GO:0043226	<1e-4	slr6016	GO:0042626	0.0132
slr0581	GO:0001932	<1e-4	slr1396	GO:0009889	<1e-4	sll7033	GO:0015293	<1e-4	sll1785	GO:0044446	<1e-4	slr0863	GO:0042626	0.0132
slr6071	GO:0051188	<1e-4	ssl5031	GO:0071496	<1e-4	sll0321	GO:0015293	<1e-4	slr5053	GO:0044422	<1e-4	sll5028	GO:0042626	0.0132
slr0667	GO:0022607	<1e-4	ssl1690	GO:0071841	<1e-4	sll0810	GO:0043167	<1e-4	slr1814	GO:0005575	<1e-4	slr2071	GO:0042626	0.0132
sll5006	GO:0009117	<1e-4	sll1233	GO:0042451	<1e-4	slr7082	GO:0060089	<1e-4	slr1122	GO:0044422	<1e-4	ssl5015	GO:0042626	0.0132
slr5053	GO:0042180	<1e-4	slr0872	GO:0006082	<1e-4	slr5024	GO:0043167	<1e-4	sll0641	GO:0044425	<1e-4	ssr2998	GO:0042626	0.0132
slr1442	GO:0019222	<1e-4	ssl7042	GO:0048878	<1e-4	slr1568	GO:0003674	<1e-4	sll0997	GO:0044464	<1e-4	slr0637	GO:0042626	0.0132
slr0366	GO:0009126	<1e-4	slr1240	GO:0006576	<1e-4	sll1511	GO:0022838	<1e-4	ssl7045	GO:0044424	<1e-4	sll0031	GO:0042626	0.0132
sll1749	GO:0051818	<1e-4	sll1651	GO:0019752	<1e-4	sll0096	GO:0016741	<1e-4	sll7043	GO:0043226	<1e-4	sll0294	GO:0042626	0.0132
slr0712	GO:0072524	<1e-4	slr1752	GO:0018130	<1e-4	slr1956	GO:0022891	<1e-4	ssr3571	GO:0044425	<1e-4	slr1601	GO:0042626	0.0132
sll1106	GO:0042455	<1e-4	slr0964	GO:0071554	<1e-4	sll0606	GO:0015399	<1e-4	slr0552	GO:0044464	<1e-4	slr0730	GO:0042626	0.0132
slr1572	GO:0006066	<1e-4	ssr8013	GO:0090304	<1e-4	ssl3549	GO:0022890	<1e-4	slr0326	GO:0044444	<1e-4	ssl7021	GO:0042626	0.0132
slr1315	GO:0009309	<1e-4	sll0898	GO:0019222	<1e-4	slr0606	GO:0015077	<1e-4	slr1866	GO:0044422	<1e-4	slr1944	GO:0042626	0.0132
slr1338	GO:0071554	<1e-4	slr1816	GO:0044282	<1e-4	slr0249	GO:0015291	<1e-4	sll1527	GO:0005575	<1e-4	sll0691	GO:0042626	0.0132
slr0109	GO:0043648	<1e-4	sll0982	GO:0019637	<1e-4	sll7034	GO:0060089	<1e-4	ssl0353	GO:0044464	<1e-4	slr6104	GO:0042626	0.0132
slr2103	GO:0031640	<1e-4	slr1681	GO:0006082	<1e-4	slr0273	GO:0042623	<1e-4	sll0286	GO:0005575	<1e-4	ssl2138	GO:0042626	0.0132
slr5017	GO:0050801	<1e-4	slr1406	GO:0072521	<1e-4	slr0516	GO:0022891	<1e-4	ssr5011	GO:0043226	<1e-4	ssl7051	GO:0042626	0.0132
sll1880	GO:0042451	<1e-4	slr0491	GO:0044282	<1e-4	sll1940	GO:0015077	<1e-4	slr1658	GO:0044444	<1e-4	ssr6062	GO:0042626	0.0132
ssl3829	GO:0006721	<1e-4	ssl7074	GO:0043170	<1e-4	ssr0335	GO:0015293	<1e-4	slr6047	GO:0005575	<1e-4	slr2070	GO:0042626	0.0132
sll0237	GO:0051716	<1e-4	sll1222	GO:0042451	<1e-4	slr1774	GO:0022857	<1e-4	ssr2972	GO:0044425	<1e-4	sll1926	GO:0042626	0.0132
slr0680	GO:0019752	<1e-4	slr1590	GO:0009161	<1e-4	slr1098	GO:0022832	<1e-4	sll5063	GO:0005575	<1e-4	ssr2067	GO:0042626	0.0132
sll7069	GO:0051347	<1e-4	ssr1499	GO:0009991	<1e-4	sll0314	GO:0022832	<1e-4	slr1083	GO:0044425	<1e-4	slr7102	GO:0042626	0.0132

ssl1377	GO:0009987	<1e-4	sll1359	GO:0009124	<1e-4	slr1505	GO:0016818	<1e-4	slr0318	GO:0044446	<1e-4	ssr2711	GO:0042626	0.0132
sll0062	GO:0043436	<1e-4	slr0689	GO:0051186	<1e-4	slr1690	GO:0008171	<1e-4	slr0729	GO:0005575	<1e-4	slr0590	GO:0042626	0.0132
sll8004	GO:0009059	<1e-4	ssl2971	GO:0009991	<1e-4	sll0572	GO:0015399	<1e-4	sll0615	GO:0044424	<1e-4	slr1174	GO:0042626	0.0132
slr1306	GO:0009308	<1e-4	slr0589	GO:0009889	<1e-4	sll0265	GO:0022803	<1e-4	sll0751	GO:0044422	<1e-4	slr1394	GO:0042626	0.0132
ssr6078	GO:0006733	<1e-4	sll1188	GO:0009259	<1e-4	slr1611	GO:0022836	<1e-4	slr0147	GO:0044464	<1e-4	slr1425	GO:0042626	0.0132
slr0699	GO:0050789	<1e-4	slr0273	GO:0019219	<1e-4	sll0641	GO:0022803	<1e-4	sll0785	GO:0044424	<1e-4	sll7070	GO:0042626	0.0132
ssr0102	GO:0031326	<1e-4	sll7087	GO:0044271	<1e-4	slr7080	GO:0015399	<1e-4	ssr7079	GO:0005575	<1e-4	ssr6089	GO:0042626	0.0132
ssl0353	GO:0065007	<1e-4	slr0937	GO:0006091	<1e-4	slr1431	GO:0003674	<1e-4	slr1788	GO:0044422	<1e-4	slr5112	GO:0042626	0.0132
ssl7021	GO:0009309	<1e-4	sll0909	GO:0065007	<1e-4	slr0082	GO:0015399	<1e-4	sll1571	GO:0044446	<1e-4	slr0013	GO:0042626	0.0132
ssr1041	GO:0006733	<1e-4	slr0157	GO:0009991	<1e-4	ssr0102	GO:0016462	<1e-4	slr1668	GO:0043226	<1e-4	slr5024	GO:0042626	0.0132
sll0932	GO:0044275	<1e-4	sll0456	GO:0051246	<1e-4	sll1155	GO:0015399	<1e-4	slr0373	GO:0044422	<1e-4	sll8001	GO:0042626	0.0132
slr1363	GO:0019751	<1e-4	sll5003	GO:0043933	<1e-4	sll0381	GO:0022838	<1e-4	slr0731	GO:0044425	<1e-4	slr0670	GO:0042626	0.0132
sll1166	GO:0043648	<1e-4	slr1142	GO:0009987	<1e-4	slr0602	GO:0003674	<1e-4	ssr7035	GO:0043226	<1e-4	sll1131	GO:0042626	0.0132
slr7101	GO:0009057	<1e-4	ssr1765	GO:0080090	<1e-4	sll1702	GO:0016818	<1e-4	sll1634	GO:0005575	<1e-4	sll5063	GO:0042626	0.0132
slr1241	GO:0034654	<1e-4	ssl0294	GO:0048522	<1e-4	sll1131	GO:0022838	<1e-4	sll0839	GO:0044464	<1e-4	sll0563	GO:0042626	0.0132
sll0980	GO:0045184	<1e-4	sll0103	GO:0019219	<1e-4	slr1073	GO:0003674	<1e-4	sll1601	GO:0043226	<1e-4	slr0516	GO:0042626	0.0132
sll5032	GO:0065007	<1e-4	sll1222	GO:0009132	<1e-4	slr2084	GO:0016818	<1e-4	sll0497	GO:0005575	<1e-4	sll7069	GO:0042626	0.0132
sll1834	GO:0031326	<1e-4	slr1384	GO:0006753	<1e-4	slr1690	GO:0042623	<1e-4	slr1203	GO:0044446	<1e-4	sll0062	GO:0042626	0.0132
slr0516	GO:0008150	<1e-4	slr1507	GO:0034660	<1e-4	sll0846	GO:0043167	<1e-4	slr1628	GO:0044464	<1e-4	slr0273	GO:0042626	0.0132
slr0771	GO:0009144	<1e-4	ssr7036	GO:0009308	<1e-4	sll0499	GO:0022836	<1e-4	slr5118	GO:0044425	<1e-4	slr0313	GO:0042626	0.0132
slr2052	GO:0050896	<1e-4	sll0310	GO:0006733	<1e-4	sll0564	GO:0016818	<1e-4	slr7083	GO:0044425	<1e-4	sll1254	GO:0042626	0.0132
sll0997	GO:0044248	<1e-4	sll0525	GO:0051246	<1e-4	sll5004	GO:0043492	<1e-4	sll5067	GO:0044444	<1e-4	sll0101	GO:0042626	0.0132
slr6066	GO:0046394	<1e-4	sll1009	GO:0022607	<1e-4	sll1049	GO:0015075	<1e-4	ssr3341	GO:0044422	<1e-4	ssl5068	GO:0042626	0.0132
slr1097	GO:0051246	<1e-4	ssr3571	GO:0006631	<1e-4	ssr1375	GO:0003674	<1e-4	ssr1425	GO:0044446	<1e-4	sll7090	GO:0042626	0.0132
slr1236	GO:0051186	<1e-4	slr0509	GO:0043436	<1e-4	sll0685	GO:0016818	<1e-4	slr0065	GO:0044422	<1e-4	ssr7017	GO:0042626	0.0132
slr0455	GO:0009309	<1e-4	sll1542	GO:0006732	<1e-4	slr5119	GO:0015291	<1e-4	slr0318	GO:0043226	<1e-4	sll7033	GO:0042626	0.0132
sll1218	GO:0001932	<1e-4	sll1174	GO:0034654	<1e-4	ssl3549	GO:0016817	<1e-4	sll1715	GO:0044422	<1e-4	sll5089	GO:0042626	0.0132
ssr6030	GO:0006576	<1e-4	slr1815	GO:0022607	<1e-4	slr1122	GO:0015399	<1e-4	ssr1951	GO:0044425	<1e-4	ssr2781	GO:0016874	0.013
slr0273	GO:0065007	<1e-4	slr1143	GO:0046164	<1e-4	sll5063	GO:0016818	<1e-4	slr1327	GO:0005575	<1e-4	sll1775	GO:0016874	0.013
slr0514	GO:0046483	<1e-4	slr6008	GO:0009056	<1e-4	sll0488	GO:0022890	<1e-4	slr0888	GO:0005575	<1e-4	sll1526	GO:0042626	0.013
slr0780	GO:0031640	<1e-4	sll1938	GO:0044282	<1e-4	slr6014	GO:0015077	<1e-4	sll0252	GO:0003824	0.9436	slr0408	GO:0016874	0.013
ssr3410	GO:0051716	<1e-4	sll0761	GO:0050789	<1e-4	ssr5120	GO:0015405	<1e-4	sll0985	GO:0003824	0.9024	ssl0353	GO:0042626	0.013
sll1797	GO:0044283	<1e-4	sll0543	GO:0016053	<1e-4	slr1143	GO:0016817	<1e-4	slr0602	GO:0003824	0.8497	slr0594	GO:0042626	0.013
slr1084	GO:0006091	<1e-4	slr1854	GO:0065007	<1e-4	sll1054	GO:0005342	<1e-4	sll0242	GO:0003824	0.819	sll0763	GO:0016874	0.013
slr0111	GO:0006066	<1e-4	sll1469	GO:0016054	<1e-4	slr1636	GO:0015399	<1e-4	sll0445	GO:0003824	0.8041	sll0783	GO:0042626	0.013
slr0169	GO:0006721	<1e-4	slr0326	GO:0044248	<1e-4	sll0266	GO:0043167	<1e-4	sll0641	GO:0003824	0.8041	slr2084	GO:0016874	0.013
slr1603	GO:0006811	<1e-4	sll0525	GO:0006091	<1e-4	sll1203	GO:0005342	<1e-4	slr1644	GO:0003824	0.794	slr1811	GO:0042626	0.013
sll1469	GO:0034654	<1e-4	slr6067	GO:0034641	<1e-4	slr7082	GO:0022891	<1e-4	slr1215	GO:0003824	0.7824	sll0732	GO:0042626	0.013
sll1698	GO:0072522	<1e-4	slr1932	GO:0009132	<1e-4	sll1289	GO:0015399	<1e-4	sll0861	GO:0008152	0.7678	sll0647	GO:0016874	0.013
slr1163	GO:0009117	<1e-4	slr1117	GO:0009199	<1e-4	slr1066	GO:0043492	<1e-4	slr1812	GO:0003824	0.758	sll0022	GO:0042626	0.013
sll1505	GO:0009142	<1e-4	sll0602	GO:0006220	<1e-4	sll1752	GO:0015077	<1e-4	slr1636	GO:0003824	0.758	slr0589	GO:0042626	0.013
ssl6035	GO:0019222	<1e-4	slr7082	GO:0006732	<1e-4	slr6100	GO:0022890	<1e-4	sll0931	GO:0003824	0.758	slr1032	GO:0016874	0.013
slr1932	GO:0009144	<1e-4	ssl0750	GO:0051186	<1e-4	sll0867	GO:0042623	<1e-4	ssl0294	GO:0003824	0.7324	slr1047	GO:0042626	0.013
sll0496	GO:0052111	<1e-4	sll0765	GO:0009263	<1e-4	slr1533	GO:0016746	<1e-4	sll0584	GO:0003824	0.7324	slr0442	GO:0016874	0.013
sll0656	GO:0019637	<1e-4	slr2120	GO:0009161	<1e-4	sll0449	GO:0022836	<1e-4	sll1763	GO:0003824	0.7324	sll0361	GO:0042626	0.013
slr1614	GO:0009308	<1e-4	slr1918	GO:0050801	<1e-4	sll0630	GO:0015405	<1e-4	sll1293	GO:0003824	0.7324	sll0910	GO:0042626	0.013
ssl6061	GO:0043648	<1e-4	sll0191	GO:0072522	<1e-4	ssl0738	GO:0022832	<1e-4	sll1527	GO:0003824	0.7324	sll1250	GO:0016874	0.013
sll0098	GO:0044275	<1e-4	sll1873	GO:0006163	<1e-4	slr6021	GO:0060089	<1e-4	sll0861	GO:0016020	0.7212	slr1800	GO:0042626	0.013
slr0148	GO:0009308	<1e-4	slr0250	GO:0044106	<1e-4	slr1276	GO:0022891	<1e-4	slr0765	GO:0003824	0.6998	slr1301	GO:0042626	0.013
sll6053	GO:0043549	<1e-4	ssr2754	GO:0009057	<1e-4	sll0872	GO:0015293	<1e-4	sll0735	GO:0003824	0.6998	sll0272	GO:0016874	0.013
slr0708	GO:0006576	<1e-4	sll1004	GO:0031323	<1e-4	sll0539	GO:0043492	<1e-4	sll1222	GO:0003824	0.6998	sll0933	GO:0004871	0.0127
sll1873	GO:0016054	<1e-4	slr1753	GO:0072528	<1e-4	sll1858	GO:0022803	<1e-4	slr1774	GO:0003824	0.6998	slr1444	GO:0006807	0.0126
sll1186	GO:0072524	<1e-4	slr0780	GO:0006082	<1e-4	sll8035	GO:0015291	<1e-4	sll1528	GO:0003824	0.6998	ssl2996	GO:0006807	0.0126
sll0060	GO:0031323	<1e-4	sll0369	GO:0071841	<1e-4	slr1628	GO:0015077	<1e-4	sll1446	GO:0003824	0.6998	slr1767	GO:0006807	0.0126

slr1572	GO:0009117	<1e-4	sll1570	GO:0071496	<1e-4	sll0175	GO:0003674	<1e-4	ssr3154	GO:0003824	0.6998	ssr1391	GO:0006807	0.0126
sll1570	GO:0043549	<1e-4	sll1912	GO:0046483	<1e-4	ssr3300	GO:0015291	<1e-4	sll1272	GO:0003824	0.6998	slr0496	GO:0006807	0.0126
slr1074	GO:0044281	<1e-4	slr2073	GO:0006066	<1e-4	sll1749	GO:0005342	<1e-4	slr0108	GO:0003824	0.6998	ssl1377	GO:0006807	0.0126
sll0645	GO:0046907	<1e-4	slr1493	GO:0009161	<1e-4	slr1670	GO:0043167	<1e-4	sll0252	GO:0016020	0.696	sll0577	GO:0006807	0.0126
slr0398	GO:0019222	<1e-4	ssr2047	GO:0044003	<1e-4	slr1681	GO:0016462	<1e-4	slr2092	GO:0003824	0.6589	sll0822	GO:0006807	0.0126
sll7086	GO:0043648	<1e-4	ssl2781	GO:0052111	<1e-4	slr1547	GO:0016817	<1e-4	slr0491	GO:0003824	0.6589	sll0183	GO:0006807	0.0126
slr1218	GO:0009161	<1e-4	sll0265	GO:0071496	<1e-4	sll0547	GO:0022857	<1e-4	ssr2843	GO:0003824	0.6589	sll1526	GO:0006807	0.0126
slr1570	GO:0050801	<1e-4	sll0804	GO:0016052	<1e-4	slr2052	GO:0022838	<1e-4	ssr2049	GO:0003824	0.6589	slr0978	GO:0006807	0.0126
slr0921	GO:0050801	<1e-4	slr0356	GO:0051246	<1e-4	sll0156	GO:0060089	<1e-4	sll0661	GO:0003824	0.6589	sll0192	GO:0006807	0.0126
sll1267	GO:0071842	<1e-4	sll1163	GO:0009132	<1e-4	slr1495	GO:0022857	<1e-4	slr0579	GO:0003824	0.6226	sll1611	GO:0006807	0.0126
slr6049	GO:0046164	<1e-4	sll1608	GO:0006163	<1e-4	sll7031	GO:0016462	<1e-4	sll1912	GO:0003824	0.6226	ssr2781	GO:0008233	0.0125
ssr1256	GO:0044271	<1e-4	sll0412	GO:0009263	<1e-4	slr6088	GO:0043492	<1e-4	slr0442	GO:0003824	0.6226	slr0919	GO:0008233	0.0125
slr0458	GO:0019752	<1e-4	slr1537	GO:0006811	<1e-4	slr1612	GO:0022890	<1e-4	sll0763	GO:0003824	0.6226	slr0208	GO:0008233	0.0125
ssr2142	GO:0016052	<1e-4	ssl1378	GO:0046483	<1e-4	slr0569	GO:0015075	<1e-4	sll1749	GO:0003824	0.6226	sll0932	GO:0008233	0.0125
slr1436	GO:0009117	<1e-4	slr8044	GO:0022607	<1e-4	sll0641	GO:0015399	<1e-4	slr1495	GO:0003824	0.6226	sll1119	GO:0008233	0.0125
slr1676	GO:0071554	<1e-4	slr1394	GO:0051188	<1e-4	slr0023	GO:0022803	<1e-4	slr0919	GO:0003824	0.6226	sll0647	GO:0008233	0.0125
sll0412	GO:0010556	<1e-4	slr1391	GO:0008150	<1e-4	slr0729	GO:0016462	<1e-4	sll0269	GO:0003824	0.6226	slr1565	GO:0008233	0.0125
ssl3573	GO:0009126	<1e-4	slr5073	GO:0050794	<1e-4	ssl5008	GO:0022892	<1e-4	sll0103	GO:0003824	0.6226	sll1749	GO:0008233	0.0125
slr0103	GO:0016053	<1e-4	sll0428	GO:0009199	<1e-4	slr2073	GO:0008171	<1e-4	sll0702	GO:0003824	0.6226	slr0263	GO:0006259	0.0124
sll1979	GO:0006163	<1e-4	sll1601	GO:0019219	<1e-4	sll0157	GO:0022832	<1e-4	sll0932	GO:0003824	0.6226	slr1811	GO:0006259	0.0124
slr6101	GO:0009394	<1e-4	sll0263	GO:0090304	<1e-4	slr6049	GO:0022891	<1e-4	ssr2803	GO:0003824	0.6226	ssl1377	GO:0006259	0.0124
slr0456	GO:0043436	<1e-4	sll6053	GO:0009199	<1e-4	sll0406	GO:0015291	<1e-4	ssr2781	GO:0003824	0.6226	slr0609	GO:0006259	0.0124
slr1218	GO:0009059	<1e-4	sll0611	GO:0072528	<1e-4	sll1698	GO:0022836	<1e-4	slr0980	GO:0003824	0.6226	sll1526	GO:0006259	0.0124
ssr5092	GO:0046128	<1e-4	ssr2317	GO:0034654	<1e-4	slr2073	GO:0022892	<1e-4	sll0272	GO:0003824	0.6226	sll1611	GO:0006259	0.0124
slr1819	GO:0071840	<1e-4	sll0647	GO:0046394	<1e-4	slr1023	GO:0005342	<1e-4	sll1775	GO:0003824	0.6226	slr1767	GO:0006259	0.0124
sll0984	GO:0044282	<1e-4	sll0909	GO:0009126	<1e-4	ssl5045	GO:0003674	<1e-4	ssl0832	GO:0003824	0.6226	sll0910	GO:0019538	0.0123
ssl1255	GO:0006576	<1e-4	ssr1528	GO:0019222	<1e-4	sll0446	GO:0016817	<1e-4	slr0848	GO:0003824	0.6226	sll1751	GO:0019538	0.0123
sll0218	GO:0051171	<1e-4	slr0885	GO:0005996	<1e-4	sll1660	GO:0015075	<1e-4	slr0208	GO:0003824	0.6226	slr1926	GO:0019538	0.0123
slr8021	GO:0034654	<1e-4	slr1636	GO:0009142	<1e-4	ssr2848	GO:0015399	<1e-4	slr1565	GO:0003824	0.6226	sll0558	GO:0019538	0.0123
slr1920	GO:0006576	<1e-4	slr1547	GO:0009124	<1e-4	slr0816	GO:0022857	<1e-4	sll1250	GO:0003824	0.6226	slr1811	GO:0019538	0.0123
slr0498	GO:0006163	<1e-4	sll0496	GO:0019362	<1e-4	sll1660	GO:0022838	<1e-4	sll0985	GO:0016020	0.6145	sll0022	GO:0019538	0.0123
slr2004	GO:0044271	<1e-4	slr1864	GO:0009132	<1e-4	sll0590	GO:0015405	<1e-4	slr0589	GO:0003824	0.573	slr1865	GO:0019538	0.0123
slr5037	GO:0071842	<1e-4	slr1644	GO:0006721	<1e-4	slr1958	GO:0043167	<1e-4	sll0022	GO:0003824	0.573	slr0978	GO:0019538	0.0123
slr1816	GO:0071841	<1e-4	slr7098	GO:0031326	<1e-4	slr0038	GO:0016817	<1e-4	slr0607	GO:0003824	0.573	slr1307	GO:0019538	0.0123
sll0985	GO:0042455	<1e-4	slr0303	GO:0080090	<1e-4	slr6016	GO:0015267	<1e-4	slr1444	GO:0003824	0.573	sll0788	GO:0019538	0.0123
slr0337	GO:0019637	<1e-4	sll1193	GO:0080090	<1e-4	slr0456	GO:0015405	<1e-4	sll0910	GO:0003824	0.573	slr1959	GO:0019538	0.0123
sll1613	GO:0019222	<1e-4	slr1827	GO:0022607	<1e-4	slr6008	GO:0022836	<1e-4	slr1301	GO:0003824	0.573	slr0496	GO:0019538	0.0123
sll1254	GO:0009132	<1e-4	slr1612	GO:0065007	<1e-4	sll0185	GO:0015075	<1e-4	slr0479	GO:0003824	0.573	slr1444	GO:0019538	0.0123
slr1800	GO:0080090	<1e-4	slr5116	GO:0009057	<1e-4	slr1601	GO:0003674	<1e-4	sll0783	GO:0003824	0.573	slr0263	GO:0019538	0.0123
sll0543	GO:0042180	<1e-4	sll1162	GO:0051234	<1e-4	ssr6027	GO:0015405	<1e-4	slr1307	GO:0003824	0.573	sll0586	GO:0019538	0.0123
slr0325	GO:0019222	<1e-4	slr2071	GO:0006163	<1e-4	slr0634	GO:0015291	<1e-4	slr0263	GO:0003824	0.573	ssl2733	GO:0019538	0.0123
sll1447	GO:0009308	<1e-4	slr7024	GO:0009309	<1e-4	slr1774	GO:0042623	<1e-4	sll0788	GO:0003824	0.573	slr1847	GO:0019538	0.0123
sll1608	GO:0065007	<1e-4	sll0822	GO:0009259	<1e-4	ssr2201	GO:0015405	<1e-4	slr0496	GO:0003824	0.573	sll1858	GO:0004518	0.012
slr0196	GO:0043170	<1e-4	ssl1972	GO:0009889	<1e-4	slr2010	GO:0015291	<1e-4	ssr1391	GO:0003824	0.573	ssl1255	GO:0004518	0.012
sll1151	GO:0051186	<1e-4	sll0309	GO:0046128	<1e-4	sll1262	GO:0016817	<1e-4	sll0732	GO:0003824	0.573	slr1385	GO:0004518	0.012
slr0498	GO:0071840	<1e-4	ssr1765	GO:0048523	<1e-4	sll5090	GO:0022803	<1e-4	slr1098	GO:0003824	0.573	sll1355	GO:0015031	0.0118
slr0848	GO:0048519	<1e-4	slr0148	GO:0042180	<1e-4	ssl2996	GO:0015399	<1e-4	slr0514	GO:0003824	0.573	ssl3379	GO:0015031	0.0118
slr0865	GO:0045184	<1e-4	slr0521	GO:0044248	<1e-4	sll1446	GO:0003674	<1e-4	sll0183	GO:0003824	0.573	slr1535	GO:0015031	0.0118
sll1681	GO:0044260	<1e-4	sll0473	GO:0051818	<1e-4	slr1920	GO:0015293	<1e-4	sll0505	GO:0003824	0.573	sll8033	GO:0015031	0.0118
sll5063	GO:0044260	<1e-4	sll1191	GO:0009124	<1e-4	sll1766	GO:0015399	<1e-4	sll0175	GO:0003824	0.573	slr0569	GO:0015031	0.0118
slr1442	GO:0034641	<1e-4	ssr1391	GO:0046128	<1e-4	sll1477	GO:0022857	<1e-4	slr0594	GO:0003824	0.573	sll1862	GO:0015031	0.0118
sll7066	GO:0005996	<1e-4	sll0198	GO:0072528	<1e-4	ssr2781	GO:0016741	<1e-4	slr0740	GO:0003824	0.573	slr1619	GO:0015031	0.0118
slr1189	GO:0065007	<1e-4	sll1511	GO:0006631	<1e-4	sll1164	GO:0015293	<1e-4	slr1767	GO:0003824	0.573	sll0436	GO:0015031	0.0118
sll1232	GO:0071554	<1e-4	sll1562	GO:0051186	<1e-4	sll0572	GO:0015267	<1e-4	sll1526	GO:0003824	0.573	sll0443	GO:0015031	0.0118

slr6013	GO:0034654	<1e-4	ssr1473	GO:0044271	<1e-4	ssl1552	GO:0022891	<1e-4	ssl0353	GO:0003824	0.573	sll1939	GO:0015031	0.0118
slr0821	GO:0044260	<1e-4	sll1247	GO:0080090	<1e-4	sll1135	GO:0005342	<1e-4	ssl1377	GO:0003824	0.573	sll0167	GO:0015031	0.0118
slr0751	GO:0052111	<1e-4	sll5067	GO:0009123	<1e-4	slr6103	GO:0015405	<1e-4	slr1865	GO:0003824	0.573	slr7025	GO:0015031	0.0118
slr1425	GO:0055114	<1e-4	slr0888	GO:0009309	<1e-4	sll0875	GO:0015399	<1e-4	sll1751	GO:0003824	0.573	slr0264	GO:0015031	0.0118
slr7010	GO:0044271	<1e-4	ssl5113	GO:0072524	<1e-4	slr1188	GO:0015267	<1e-4	slr0609	GO:0003824	0.573	slr1104	GO:0015031	0.0118
sll1240	GO:0034654	<1e-4	ssr6002	GO:0044106	<1e-4	slr0729	GO:0060089	<1e-4	slr1431	GO:0008152	0.5561	slr1920	GO:0015031	0.0118
slr1023	GO:0009893	<1e-4	slr0765	GO:0044255	<1e-4	slr6029	GO:0016741	<1e-4	slr0602	GO:0016020	0.5475	ssr1951	GO:0015031	0.0118
ssr6062	GO:0009142	<1e-4	sll5004	GO:0065007	<1e-4	sll1086	GO:0022803	<1e-4	sll0068	GO:0008152	0.5474	slr0655	GO:0015031	0.0118
slr1363	GO:0005996	<1e-4	slr0784	GO:0008610	<1e-4	sll0931	GO:0016746	<1e-4	slr1045	GO:0003824	0.5363	slr1263	GO:0015031	0.0118
slr1470	GO:0034654	<1e-4	sll0022	GO:0051171	<1e-4	slr1362	GO:0016817	<1e-4	sll0447	GO:0003824	0.5363	slr2118	GO:0015031	0.0118
ssl5027	GO:0055114	<1e-4	ssl5100	GO:0051171	<1e-4	slr6073	GO:0016818	<1e-4	slr1023	GO:0003824	0.5363	ssr5120	GO:0015031	0.0118
slr1419	GO:0044281	<1e-4	sll0595	GO:0044106	<1e-4	slr0243	GO:0022892	<1e-4	slr1918	GO:0003824	0.5363	slr0179	GO:0015031	0.0118
slr1862	GO:0010556	<1e-4	sll0281	GO:0009165	<1e-4	sll0822	GO:0016746	<1e-4	slr0376	GO:0003824	0.5363	sll0609	GO:0015031	0.0118
sll1158	GO:0008150	<1e-4	sll0984	GO:0048519	<1e-4	slr6029	GO:0043492	<1e-4	sll0354	GO:0003824	0.5363	slr6072	GO:0015031	0.0118
slr1778	GO:0044282	<1e-4	ssr2803	GO:0009142	<1e-4	sll0997	GO:0016818	<1e-4	ssl3692	GO:0003824	0.5363	slr1923	GO:0015031	0.0118
ssl0739	GO:0009150	<1e-4	sll0243	GO:0008150	<1e-4	sll0625	GO:0015291	<1e-4	sll1218	GO:0003824	0.5363	sll1797	GO:0015031	0.0118
sll1609	GO:0019219	<1e-4	slr0294	GO:0019222	<1e-4	slr0305	GO:0015075	<1e-4	sll0274	GO:0003824	0.5363	ssr6079	GO:0015031	0.0118
sll7028	GO:0006732	<1e-4	slr1778	GO:0051246	<1e-4	slr0959	GO:0022891	<1e-4	ssr1375	GO:0003824	0.5363	sll1414	GO:0015031	0.0118
sll0888	GO:0052188	<1e-4	slr1235	GO:0045184	<1e-4	slr0680	GO:0015077	<1e-4	slr1557	GO:0003824	0.5363	slr1768	GO:0015031	0.0118
sll1912	GO:0006066	<1e-4	slr1396	GO:0009309	<1e-4	sll2006	GO:0003674	<1e-4	slr1196	GO:0003824	0.5363	sgl0001	GO:0015031	0.0118
sll0737	GO:0022607	<1e-4	sll0614	GO:0009394	<1e-4	ssl2420	GO:0016817	<1e-4	ssl0787	GO:0003824	0.5363	sll0024	GO:0015031	0.0118
slr0006	GO:0009308	<1e-4	sll0625	GO:0009165	<1e-4	slr0505	GO:0016462	<1e-4	sll0596	GO:0003824	0.5363	slr1273	GO:0015031	0.0118
slr1235	GO:0006766	<1e-4	slr0654	GO:0009124	<1e-4	slr0483	GO:0022836	<1e-4	sll1934	GO:0003824	0.5363	sll1232	GO:0015031	0.0118
sll0098	GO:0051188	<1e-4	slr0876	GO:0044248	<1e-4	sll1022	GO:0043167	<1e-4	sll1715	GO:0003824	0.5363	sll0553	GO:0015031	0.0118
slr7094	GO:0010468	<1e-4	sll1472	GO:0022607	<1e-4	slr1143	GO:0015405	<1e-4	slr1222	GO:0003824	0.5363	slr1419	GO:0015031	0.0118
sll0225	GO:0071840	<1e-4	sll0103	GO:0044248	<1e-4	slr0510	GO:0015077	<1e-4	slr0304	GO:0003824	0.5363	slr7014	GO:0015031	0.0118
slr5012	GO:0046483	<1e-4	sll0497	GO:0006163	<1e-4	sll0857	GO:0015267	<1e-4	slr1917	GO:0003824	0.5363	slr1534	GO:0015031	0.0118
sll0872	GO:0006066	<1e-4	slr0650	GO:0006066	<1e-4	sll0839	GO:0022832	<1e-4	sll0096	GO:0003824	0.5363	slr0049	GO:0015031	0.0118
slr6068	GO:0044282	<1e-4	slr0664	GO:0046907	<1e-4	slr1464	GO:0060089	<1e-4	slr1025	GO:0003824	0.5363	sll1552	GO:0015031	0.0118
sll5026	GO:0051234	<1e-4	ssr2060	GO:0008610	<1e-4	ssr3570	GO:0015405	<1e-4	slr1519	GO:0003824	0.5363	slr0821	GO:0015031	0.0118
ssr7035	GO:0044248	<1e-4	sll1289	GO:0046164	<1e-4	ssl5031	GO:0022857	<1e-4	ssr2848	GO:0003824	0.5363	slr6005	GO:0015031	0.0118
sll8001	GO:0009889	<1e-4	slr1415	GO:0051246	<1e-4	slr2048	GO:0015399	<1e-4	sll0743	GO:0003824	0.5363	slr6047	GO:0015031	0.0118
sll1388	GO:0009987	<1e-4	slr5073	GO:0051347	<1e-4	sll0596	GO:0015267	<1e-4	sll1942	GO:0003824	0.5363	sll1511	GO:0015031	0.0118
sll1680	GO:0050896	<1e-4	slr0634	GO:0048522	<1e-4	sll8033	GO:0060089	<1e-4	sll1318	GO:0003824	0.5363	ssl1498	GO:0015031	0.0118
sll0810	GO:0006082	<1e-4	slr0924	GO:0009057	<1e-4	slr0082	GO:0022892	<1e-4	slr0551	GO:0003824	0.5363	slr1384	GO:0015031	0.0118
slr0249	GO:0043412	<1e-4	sll0656	GO:0009165	<1e-4	slr7071	GO:0022838	<1e-4	sll0148	GO:0003824	0.5363	sll0446	GO:0016820	0.0118
slr1923	GO:0043648	<1e-4	slr6090	GO:0006091	<1e-4	sll0590	GO:0060089	<1e-4	slr0876	GO:0003824	0.5363	ssr0692	GO:0016820	0.0118
sll1671	GO:0006576	<1e-4	slr1195	GO:0044260	<1e-4	slr0865	GO:0003674	<1e-4	sll0360	GO:0003824	0.5363	ssr3154	GO:0016820	0.0118
ssl1792	GO:0044248	<1e-4	slr0664	GO:0050801	<1e-4	slr1471	GO:0016462	<1e-4	slr1398	GO:0003824	0.5363	sll1455	GO:0006396	0.0117
sll1160	GO:0090304	<1e-4	sll0062	GO:0019752	<1e-4	ssr7036	GO:0015405	<1e-4	ssl0109	GO:0003824	0.5363	slr0480	GO:0006396	0.0117
slr6081	GO:0044282	<1e-4	sll7066	GO:0009126	<1e-4	ssr6062	GO:0022892	<1e-4	slr0305	GO:0003824	0.5363	sll0499	GO:0006396	0.0117
ssl0242	GO:0022411	<1e-4	ssr3409	GO:0052111	<1e-4	slr1964	GO:0022832	<1e-4	ssr0536	GO:0003824	0.5363	sll0615	GO:0006396	0.0117
slr2117	GO:0071842	<1e-4	sll0676	GO:0055086	<1e-4	ssl3692	GO:0008171	<1e-4	sll1433	GO:0003824	0.5363	sll0447	GO:0006396	0.0117
slr1034	GO:0065007	<1e-4	slr0553	GO:0055082	<1e-4	slr0103	GO:0015405	<1e-4	slr0211	GO:0003824	0.5363	slr1196	GO:0006396	0.0117
sll5004	GO:0044271	<1e-4	ssr0759	GO:0009893	<1e-4	slr2092	GO:0016462	<1e-4	slr0852	GO:0003824	0.5363	sll1934	GO:0006396	0.0117
sll1926	GO:0009161	<1e-4	sll0783	GO:0044281	<1e-4	ssr6030	GO:0015267	<1e-4	slr0250	GO:0003824	0.5363	ssr0536	GO:0006396	0.0117
ssl0738	GO:0065007	<1e-4	ssl7045	GO:0048878	<1e-4	sll0787	GO:0022832	<1e-4	slr1659	GO:0003824	0.5363	slr0305	GO:0006396	0.0117
sll0284	GO:0071496	<1e-4	slr0625	GO:0009123	<1e-4	slr6068	GO:0016462	<1e-4	sll1186	GO:0003824	0.5363	slr1353	GO:0006396	0.0117
ssr1951	GO:0051234	<1e-4	slr1384	GO:0009199	<1e-4	slr1677	GO:0016741	<1e-4	sll0508	GO:0003824	0.5363	sll1318	GO:0006396	0.0117
slr1023	GO:0080090	<1e-4	sll1862	GO:0031323	<1e-4	slr0317	GO:0022832	<1e-4	sll0513	GO:0003824	0.5363	slr0948	GO:0004518	0.0117
sll0167	GO:0090304	<1e-4	sll1505	GO:0009394	<1e-4	slr0305	GO:0015405	<1e-4	slr0287	GO:0003824	0.5363	slr0373	GO:0008324	0.0117
slr0249	GO:0022607	<1e-4	sll0658	GO:0043933	<1e-4	slr6103	GO:0016741	<1e-4	slr1066	GO:0003824	0.5363	sll0101	GO:0004518	0.0117
sll1022	GO:0052188	<1e-4	slr5021	GO:0072524	<1e-4	slr1571	GO:0022803	<1e-4	slr1674	GO:0003824	0.5363	sll1698	GO:0004518	0.0117
slr1162	GO:0008150	<1e-4	slr1168	GO:0009199	<1e-4	sll0218	GO:0022803	<1e-4	sll0301	GO:0003824	0.5363	sll1582	GO:0008324	0.0117

ssr2611	GO:0006066	<1e-4	ssr2439	GO:0051171	<1e-4	sll1022	GO:0022803	<1e-4	slr0157	GO:0003824	0.5363	slr1790	GO:0004518	0.0117
sll0783	GO:0048878	<1e-4	sll1106	GO:0042451	<1e-4	ssr6062	GO:0042623	<1e-4	slr1127	GO:0003824	0.5363	slr8021	GO:0004518	0.0117
sll1915	GO:0044248	<1e-4	sll0410	GO:0010556	<1e-4	ssr2975	GO:0016462	<1e-4	sll1455	GO:0003824	0.5363	sll5028	GO:0004518	0.0117
ssr7079	GO:0055086	<1e-4	ssr3341	GO:0051347	<1e-4	sll0160	GO:0015075	<1e-4	slr1391	GO:0003824	0.5363	ssl2138	GO:0004518	0.0117
sll1225	GO:0006163	<1e-4	slr0341	GO:0009394	<1e-4	sll1934	GO:0022857	<1e-4	ssr1698	GO:0003824	0.5363	ssr2067	GO:0004518	0.0117
slr1920	GO:0019751	<1e-4	slr0980	GO:0019637	<1e-4	sll1634	GO:0015399	<1e-4	slr1376	GO:0003824	0.5363	sll1730	GO:0008324	0.0117
ssl7007	GO:0071840	<1e-4	slr0810	GO:0006631	<1e-4	slr1851	GO:0022891	<1e-4	sll0424	GO:0003824	0.5363	sll0394	GO:0004518	0.0117
slr1505	GO:0043436	<1e-4	sll7077	GO:0046164	<1e-4	sll0167	GO:0043492	<1e-4	slr0545	GO:0003824	0.5363	ssr6024	GO:0004518	0.0117
slr1572	GO:0046164	<1e-4	slr0169	GO:0022607	<1e-4	sll0174	GO:0022803	<1e-4	sll0854	GO:0003824	0.5363	slr7071	GO:0004518	0.0117
slr0313	GO:0034654	<1e-4	slr6016	GO:0072521	<1e-4	sll1702	GO:0022892	<1e-4	slr0871	GO:0003824	0.5363	slr1668	GO:0008324	0.0117
sll1882	GO:0065007	<1e-4	ssl1792	GO:0072521	<1e-4	slr0111	GO:0015267	<1e-4	sll0547	GO:0003824	0.5363	ssr1558	GO:0004518	0.0117
sll1173	GO:0009991	<1e-4	slr0723	GO:0034654	<1e-4	ssl5098	GO:0043167	<1e-4	sll0413	GO:0008152	0.5353	sll1072	GO:0008324	0.0117
ssl1300	GO:0043549	<1e-4	sll0263	GO:0006631	<1e-4	slr0364	GO:0015267	<1e-4	slr1812	GO:0008152	0.5353	slr2110	GO:0004518	0.0117
sll1979	GO:0008150	<1e-4	ssr1473	GO:0055114	<1e-4	slr0989	GO:0022892	<1e-4	sll0641	GO:0016020	0.522	slr0610	GO:0004518	0.0117
slr6063	GO:0009259	<1e-4	slr2070	GO:0034641	<1e-4	sll0241	GO:0016818	<1e-4	sll0314	GO:0003824	0.5134	slr1911	GO:0004518	0.0117
slr2118	GO:0072524	<1e-4	ssl2733	GO:0048878	<1e-4	slr6004	GO:0016817	<1e-4	slr1913	GO:0003824	0.5134	slr1303	GO:0008324	0.0117
slr0921	GO:0033013	<1e-4	slr0609	GO:0009132	<1e-4	sll7065	GO:0005342	<1e-4	sll0309	GO:0003824	0.5134	sll0047	GO:0004518	0.0117
ssr2333	GO:0019752	<1e-4	sll1469	GO:0022411	<1e-4	sll0584	GO:0016741	<1e-4	slr1999	GO:0003824	0.5134	slr1450	GO:0004518	0.0117
slr1681	GO:0009987	<1e-4	ssr2318	GO:0008150	<1e-4	slr1104	GO:0022838	<1e-4	slr0404	GO:0003824	0.5134	sll1464	GO:0004518	0.0117
sll5067	GO:0046483	<1e-4	ssl2245	GO:0065007	<1e-4	sll1163	GO:0015077	<1e-4	slr1593	GO:0003824	0.5134	ssl0483	GO:0008324	0.0117
slr7102	GO:0006631	<1e-4	ssl7021	GO:0018130	<1e-4	slr0575	GO:0043167	<1e-4	ssr2755	GO:0003824	0.5134	slr2115	GO:0008324	0.0117
sll1217	GO:0010468	<1e-4	sll0381	GO:0072522	<1e-4	slr0380	GO:0022832	<1e-4	slr1778	GO:0003824	0.5134	sll1049	GO:0004518	0.0117
slr5101	GO:0006721	<1e-4	slr6065	GO:0051246	<1e-4	slr1519	GO:0022890	<1e-4	ssr1765	GO:0003824	0.5134	slr0957	GO:0008324	0.0117
ssl3573	GO:0048523	<1e-4	ssl8008	GO:0071842	<1e-4	sll0098	GO:0022890	<1e-4	slr0348	GO:0003824	0.5134	sll1251	GO:0008324	0.0117
slr1116	GO:0031326	<1e-4	slr6009	GO:0044282	<1e-4	ssl5129	GO:0016817	<1e-4	sll1315	GO:0003824	0.5134	sll0685	GO:0008324	0.0117
sll0406	GO:0051716	<1e-4	slr0845	GO:0071496	<1e-4	ssr2803	GO:0016818	<1e-4	slr0519	GO:0003824	0.5134	slr0654	GO:0008324	0.0117
slr1266	GO:0071841	<1e-4	sgl0001	GO:0010556	<1e-4	slr1851	GO:0008171	<1e-4	slr1880	GO:0003824	0.5134	sll8012	GO:0004518	0.0117
sll0793	GO:0001932	<1e-4	slr0865	GO:0031323	<1e-4	ssl7046	GO:0043492	<1e-4	sll1630	GO:0003824	0.5134	slr1383	GO:0008324	0.0117
sll0400	GO:0009141	<1e-4	ssr2711	GO:0043436	<1e-4	slr1150	GO:0042623	<1e-4	slr1275	GO:0003824	0.5134	sll0249	GO:0004518	0.0117
sll1009	GO:0044281	<1e-4	slr0148	GO:0043648	<1e-4	sll0350	GO:0015291	<1e-4	slr1573	GO:0003824	0.5134	sll0909	GO:0008324	0.0117
sll1511	GO:0016054	<1e-4	sll1002	GO:0034641	<1e-4	sll1485	GO:0015075	<1e-4	sll1835	GO:0003824	0.5134	sll0572	GO:0008324	0.0117
sll0496	GO:0009260	<1e-4	slr1142	GO:0043436	<1e-4	sll0846	GO:0022892	<1e-4	sll0524	GO:0003824	0.5134	slr0728	GO:0004518	0.0117
sll1191	GO:0046128	<1e-4	ssl1378	GO:0051818	<1e-4	sll7064	GO:0008171	<1e-4	slr1081	GO:0003824	0.5134	sll0359	GO:0008324	0.0117
slr2004	GO:0042451	<1e-4	sll0183	GO:0034641	<1e-4	slr1177	GO:0022891	<1e-4	sll0066	GO:0003824	0.5134	ssl8028	GO:0004518	0.0117
slr0172	GO:0009132	<1e-4	slr1223	GO:0001932	<1e-4	ssr6026	GO:0022891	<1e-4	slr1932	GO:0003824	0.5134	sll1319	GO:0004518	0.0117
sll0282	GO:0080090	<1e-4	sll1717	GO:0042451	<1e-4	slr1484	GO:0022803	<1e-4	slr1885	GO:0003824	0.5134	sll1201	GO:0008324	0.0117
sll1021	GO:0008150	<1e-4	sll0410	GO:0019752	<1e-4	slr1915	GO:0015405	<1e-4	slr2122	GO:0003824	0.5134	sll0479	GO:0004518	0.0117
sll0160	GO:0048519	<1e-4	slr1915	GO:0009132	<1e-4	sll0749	GO:0015077	<1e-4	slr1854	GO:0003824	0.5134	ssr7084	GO:0004518	0.0117
slr7060	GO:0043436	<1e-4	slr1206	GO:0031323	<1e-4	sll1652	GO:0043167	<1e-4	sll0185	GO:0003824	0.5134	ssl5113	GO:0004518	0.0117
sll0478	GO:0065007	<1e-4	slr1431	GO:0043436	<1e-4	slr1303	GO:0016462	<1e-4	slr0386	GO:0003824	0.5134	sll1926	GO:0004518	0.0117
ssl5031	GO:0006721	<1e-4	ssl2162	GO:0009132	<1e-4	sll1698	GO:0015291	<1e-4	slr2121	GO:0003824	0.5134	slr1290	GO:0004518	0.0117
slr0871	GO:0065007	<1e-4	slr1376	GO:0006576	<1e-4	ssr6046	GO:0022892	<1e-4	slr0921	GO:0003824	0.5134	sll7077	GO:0004518	0.0117
sll0853	GO:0051188	<1e-4	sll1738	GO:0050801	<1e-4	slr1342	GO:0022803	<1e-4	slr0845	GO:0003824	0.5134	ssr1155	GO:0004518	0.0117
ssr2060	GO:0016052	<1e-4	ssl0739	GO:0050896	<1e-4	ssl0788	GO:0016741	<1e-4	slr0723	GO:0003824	0.5134	sll1832	GO:0004518	0.0117
slr1363	GO:0051818	<1e-4	slr0680	GO:0006733	<1e-4	slr1102	GO:0016462	<1e-4	slr1788	GO:0003824	0.5134	ssl3615	GO:0004518	0.0117
slr0552	GO:0048518	<1e-4	sll8033	GO:0048518	<1e-4	sll0354	GO:0005342	<1e-4	sll0497	GO:0003824	0.5134	slr0650	GO:0008324	0.0117
sll0149	GO:0016070	<1e-4	slr1327	GO:0006721	<1e-4	ssl5100	GO:0022836	<1e-4	sll1965	GO:0003824	0.5134	slr0981	GO:0008324	0.0117
slr6047	GO:0019222	<1e-4	slr0271	GO:0009987	<1e-4	slr1209	GO:0043167	<1e-4	slr1676	GO:0003824	0.5134	sll0740	GO:0008324	0.0117
ssr1473	GO:0072527	<1e-4	ssl1046	GO:0010468	<1e-4	sll1571	GO:0016818	<1e-4	slr0023	GO:0003824	0.5134	sll1151	GO:0004518	0.0117
ssl0109	GO:0055114	<1e-4	sll0864	GO:0009142	<1e-4	slr1862	GO:0015405	<1e-4	sll1530	GO:0003824	0.5134	sll1654	GO:0008324	0.0117
sll2013	GO:0051716	<1e-4	slr0634	GO:0009987	<1e-4	slr0388	GO:0015405	<1e-4	sll0177	GO:0003824	0.5134	slr2052	GO:0004518	0.0117
slr0967	GO:0046128	<1e-4	sll1995	GO:0033013	<1e-4	slr0059	GO:0022803	<1e-4	sll0756	GO:0003824	0.5134	sll0044	GO:0004518	0.0117
slr7060	GO:0009991	<1e-4	ssr2142	GO:0009262	<1e-4	sll0284	GO:0016818	<1e-4	slr1530	GO:0003824	0.5134	sll1505	GO:0008324	0.0117
sll1912	GO:0010556	<1e-4	sll1109	GO:0006066	<1e-4	sll0661	GO:0022803	<1e-4	slr0431	GO:0003824	0.5134	slr0587	GO:0004518	0.0117

sll0310	GO:0045184	<1e-4	slr1449	GO:0048522	<1e-4	slr0656	GO:0015293	<1e-4	slr1571	GO:0003824	0.5134	sll0625	GO:0004518	0.0117
slr1900	GO:0009308	<1e-4	slr1940	GO:0022607	<1e-4	slr6051	GO:0003674	<1e-4	sll1736	GO:0003824	0.5134	slr1062	GO:0008324	0.0117
sll0218	GO:0045184	<1e-4	ssl2064	GO:0009132	<1e-4	sll0735	GO:0022857	<1e-4	ssr2787	GO:0003824	0.5134	slr1977	GO:0008324	0.0117
ssr2067	GO:0042451	<1e-4	sll0564	GO:0006766	<1e-4	sll0192	GO:0015399	<1e-4	slr0668	GO:0003824	0.5134	slr0699	GO:0004518	0.0117
slr1958	GO:0009132	<1e-4	slr7081	GO:0019222	<1e-4	slr0610	GO:0015075	<1e-4	slr0360	GO:0003824	0.5134	slr1082	GO:0008324	0.0117
slr0366	GO:0006082	<1e-4	ssl5100	GO:0006163	<1e-4	sll0565	GO:0022838	<1e-4	ssr3122	GO:0003824	0.5134	slr1816	GO:0004518	0.0117
slr0594	GO:0019637	<1e-4	slr0517	GO:0090304	<1e-4	sll1765	GO:0022838	<1e-4	ssr2551	GO:0003824	0.5134	slr0625	GO:0004518	0.0117
sll5046	GO:0072521	<1e-4	slr5126	GO:0051186	<1e-4	sll0238	GO:0016746	<1e-4	sll1761	GO:0003824	0.5134	slr0989	GO:0008324	0.0117
slr6031	GO:0009260	<1e-4	slr1383	GO:0071841	<1e-4	slr7059	GO:0022891	<1e-4	slr2073	GO:0003824	0.5134	ssl5015	GO:0004518	0.0117
slr0489	GO:0048518	<1e-4	slr1070	GO:0044003	<1e-4	slr1636	GO:0016462	<1e-4	sll0780	GO:0003824	0.5134	slr7100	GO:0004518	0.0117
slr1079	GO:0009123	<1e-4	slr1493	GO:0008150	<1e-4	slr0362	GO:0015399	<1e-4	slr1203	GO:0003824	0.5134	slr1327	GO:0004518	0.0117
slr1025	GO:0034641	<1e-4	slr6029	GO:0006720	<1e-4	slr1600	GO:0016817	<1e-4	slr1437	GO:0003824	0.5134	sll0062	GO:0004518	0.0117
slr1623	GO:0046907	<1e-4	slr1069	GO:0022411	<1e-4	slr0637	GO:0015405	<1e-4	ssr2439	GO:0003824	0.5134	sll1476	GO:0004518	0.0117
sll1730	GO:0050801	<1e-4	sll0446	GO:0009161	<1e-4	sll0023	GO:0016746	<1e-4	sll0381	GO:0003824	0.5134	slr1886	GO:0004518	0.0117
slr1163	GO:0044248	<1e-4	slr0103	GO:0044271	<1e-4	sll0156	GO:0022803	<1e-4	slr1572	GO:0003824	0.5134	sll0925	GO:0004518	0.0117
slr6087	GO:0006811	<1e-4	sll0478	GO:0006091	<1e-4	slr1799	GO:0043167	<1e-4	ssl2065	GO:0003824	0.5134	sll0670	GO:0004518	0.0117
slr7081	GO:0019752	<1e-4	sll1862	GO:0010468	<1e-4	sll1784	GO:0005342	<1e-4	sll0676	GO:0003824	0.5134	sll1289	GO:0004518	0.0117
slr1429	GO:0009394	<1e-4	slr0948	GO:0043549	<1e-4	slr0876	GO:0015291	<1e-4	sll0085	GO:0003824	0.5134	slr0181	GO:0004518	0.0117
slr1413	GO:0009394	<1e-4	slr1276	GO:0051179	<1e-4	sll1186	GO:0043492	<1e-4	slr0364	GO:0003824	0.5134	sll0180	GO:0008324	0.0117
slr0358	GO:0044255	<1e-4	sll0447	GO:0043436	<1e-4	sll1390	GO:0015075	<1e-4	sll1563	GO:0003824	0.5134	ssl2069	GO:0004518	0.0117
slr7015	GO:0009126	<1e-4	sll1400	GO:0034641	<1e-4	slr1935	GO:0015399	<1e-4	slr1073	GO:0003824	0.5134	sll2011	GO:0004518	0.0117
slr1025	GO:0033013	<1e-4	slr1659	GO:0031326	<1e-4	sll1289	GO:0003674	<1e-4	sll1262	GO:0003824	0.5134	slr0334	GO:0004518	0.0117
slr5127	GO:0008150	<1e-4	sll1446	GO:0090304	<1e-4	slr0740	GO:0005342	<1e-4	sll1442	GO:0003824	0.5134	slr0013	GO:0004518	0.0117
ssl2384	GO:0048522	<1e-4	slr1327	GO:0009161	<1e-4	slr0964	GO:0043167	<1e-4	ssr3571	GO:0003824	0.5134	ssr2975	GO:0004518	0.0117
ssr2439	GO:0019752	<1e-4	sll0369	GO:0010468	<1e-4	slr1327	GO:0015075	<1e-4	slr0192	GO:0003824	0.5134	sll0688	GO:0008324	0.0117
sll1388	GO:0055086	<1e-4	slr1441	GO:0071496	<1e-4	sll1764	GO:0005342	<1e-4	slr1177	GO:0003824	0.5134	slr0241	GO:0004518	0.0117
sll0742	GO:0009892	<1e-4	slr7014	GO:0009161	<1e-4	sll1307	GO:0043492	<1e-4	ssl0352	GO:0003824	0.5134	slr0975	GO:0008324	0.0117
sll0590	GO:0018130	<1e-4	slr0103	GO:0031323	<1e-4	sll0327	GO:0016741	<1e-4	slr0770	GO:0003824	0.5134	ssr3572	GO:0004518	0.0117
sll0309	GO:0034641	<1e-4	sll0192	GO:0009892	<1e-4	slr0770	GO:0042623	<1e-4	sll0532	GO:0003824	0.5134	sll7070	GO:0004518	0.0117
sll1344	GO:0071841	<1e-4	sll0803	GO:0009124	<1e-4	sll1835	GO:0022857	<1e-4	slr0144	GO:0003824	0.5134	slr1875	GO:0004518	0.0117
slr1544	GO:0006082	<1e-4	slr1657	GO:0048523	<1e-4	sll1062	GO:0022832	<1e-4	slr1084	GO:0003824	0.5134	slr0885	GO:0004518	0.0117
ssr6027	GO:0044260	<1e-4	slr1812	GO:0046128	<1e-4	slr6014	GO:0015291	<1e-4	slr2111	GO:0003824	0.5134	sll5128	GO:0004518	0.0117
slr0325	GO:0009142	<1e-4	sll1834	GO:0034641	<1e-4	slr0784	GO:0022890	<1e-4	ssr2962	GO:0003824	0.5134	slr0291	GO:0004518	0.0117
sll0198	GO:0044260	<1e-4	slr6013	GO:0055082	<1e-4	slr6106	GO:0022803	<1e-4	ssr2142	GO:0016020	0.5087	slr0613	GO:0004518	0.0117
slr0264	GO:0071841	<1e-4	slr1101	GO:0009892	<1e-4	slr7082	GO:0008171	<1e-4	slr1431	GO:0016020	0.5087	slr0510	GO:0008324	0.0117
slr1854	GO:0006220	<1e-4	sll5030	GO:0031326	<1e-4	ssl5098	GO:0022857	<1e-4	sll0584	GO:0008152	0.5033	ssl7021	GO:0004518	0.0117
sll0982	GO:0019362	<1e-4	slr1122	GO:0052188	<1e-4	slr0058	GO:0016817	<1e-4	sll0068	GO:0016020	0.5033	slr0483	GO:0008324	0.0117
sll1509	GO:0048518	<1e-4	slr7024	GO:0060255	<1e-4	sll5132	GO:0015399	<1e-4	sll0688	GO:0003824	0.4943	sll1089	GO:0004518	0.0117
slr0667	GO:0008150	<1e-4	slr1638	GO:0009150	<1e-4	sll0414	GO:0015267	<1e-4	sll0180	GO:0003824	0.4943	sll1979	GO:0004518	0.0117
slr1391	GO:0009262	<1e-4	sll5033	GO:0043170	<1e-4	ssl7048	GO:0016741	<1e-4	sll0297	GO:0003824	0.4943	sll6052	GO:0004518	0.0117
slr1507	GO:0006082	<1e-4	slr5013	GO:0009142	<1e-4	slr0362	GO:0005342	<1e-4	slr0957	GO:0003824	0.4943	slr0605	GO:0008324	0.0117
sll1995	GO:0043436	<1e-4	sll5033	GO:0051818	<1e-4	sll0931	GO:0015267	<1e-4	sll1239	GO:0003824	0.4943	sll1925	GO:0008324	0.0117
slr0872	GO:0071496	<1e-4	slr1179	GO:0048519	<1e-4	sll1509	GO:0016817	<1e-4	sll1659	GO:0003824	0.4943	ssl3382	GO:0008324	0.0117
slr0521	GO:0006066	<1e-4	slr1397	GO:0009987	<1e-4	slr5013	GO:0022803	<1e-4	sll0359	GO:0003824	0.4943	slr0976	GO:0004518	0.0117
sll1389	GO:0009124	<1e-4	slr0930	GO:0048523	<1e-4	slr1619	GO:0042623	<1e-4	sll0810	GO:0003824	0.4943	sll0412	GO:0008324	0.0117
sll1485	GO:0071840	<1e-4	sll0286	GO:0046907	<1e-4	sll5090	GO:0015077	<1e-4	slr1809	GO:0003824	0.4943	sll0608	GO:0004518	0.0117
sll1414	GO:0071842	<1e-4	sll6055	GO:0052111	<1e-4	slr0959	GO:0015267	<1e-4	slr1590	GO:0003824	0.4943	slr0812	GO:0004518	0.0117
sll5028	GO:0072528	<1e-4	sll0864	GO:0051246	<1e-4	sll1401	GO:0015405	<1e-4	ssr1528	GO:0003824	0.4943	slr2003	GO:0004518	0.0117
sll1660	GO:0044260	<1e-4	sll0982	GO:0051716	<1e-4	slr0348	GO:0043167	<1e-4	sll1938	GO:0003824	0.4943	sll7078	GO:0004518	0.0117
slr0476	GO:0006766	<1e-4	slr6094	GO:0009117	<1e-4	slr1534	GO:0015267	<1e-4	slr1895	GO:0003824	0.4943	ssl7038	GO:0004518	0.0117
slr1353	GO:0009161	<1e-4	slr1034	GO:0008150	<1e-4	sll0905	GO:0022836	<1e-4	sll1660	GO:0003824	0.4943	slr1056	GO:0004518	0.0117
slr0376	GO:0044248	<1e-4	sll1696	GO:0006066	<1e-4	slr8044	GO:0003674	<1e-4	slr1033	GO:0003824	0.4943	sll1381	GO:0004518	0.0117
sll1002	GO:0008610	<1e-4	sll0860	GO:0009309	<1e-4	sll0238	GO:0022891	<1e-4	sll0141	GO:0003824	0.4943	slr0082	GO:0004518	0.0117
sll1389	GO:0019219	<1e-4	slr1854	GO:0044260	<1e-4	sll0609	GO:0015291	<1e-4	sll0685	GO:0003824	0.4943	ssr3300	GO:0008324	0.0117

ssr6078	GO:0009892	<1e-4	ssl5008	GO:0009059	<1e-4	slr2004	GO:0015267	<1e-4	sll1072	GO:0003824	0.4943	sll1352	GO:0008324	0.0117
sll1681	GO:0006720	<1e-4	slr1547	GO:0009150	<1e-4	slr1789	GO:0022832	<1e-4	sll0871	GO:0003824	0.4943	sll0321	GO:0004518	0.0117
sll1021	GO:0022607	<1e-4	slr0575	GO:0048523	<1e-4	sll8027	GO:0022836	<1e-4	sll7065	GO:0003824	0.4943	slr0397	GO:0004518	0.0117
ssr6027	GO:0080090	<1e-4	ssr2318	GO:0006793	<1e-4	slr1107	GO:0015075	<1e-4	ssr2754	GO:0003824	0.4943	slr6081	GO:0004518	0.0117
ssr2962	GO:0009987	<1e-4	slr0249	GO:0009987	<1e-4	ssr2802	GO:0022891	<1e-4	slr1288	GO:0003824	0.4943	sll5003	GO:0004518	0.0117
slr1767	GO:0009057	<1e-4	sll1730	GO:0006091	<1e-4	slr1914	GO:0022832	<1e-4	slr1493	GO:0003824	0.4943	slr0358	GO:0008324	0.0117
ssr5020	GO:0046483	<1e-4	ssl7007	GO:0005996	<1e-4	sll7031	GO:0016746	<1e-4	slr1658	GO:0003824	0.4943	ssl0410	GO:0004518	0.0117
sll0608	GO:0009308	<1e-4	sll1307	GO:0009161	<1e-4	sll1954	GO:0015267	<1e-4	sll0669	GO:0003824	0.4943	sll0160	GO:0004518	0.0117
ssl3573	GO:0044260	<1e-4	ssl5025	GO:0071840	<1e-4	sll1225	GO:0016462	<1e-4	sll1201	GO:0003824	0.4943	sll0871	GO:0008324	0.0117
sll7077	GO:0080090	<1e-4	ssl8008	GO:0044275	<1e-4	sll1527	GO:0022803	<1e-4	slr2005	GO:0003824	0.4943	ssr0336	GO:0004518	0.0117
sll0802	GO:0072527	<1e-4	slr1670	GO:0022411	<1e-4	sll0761	GO:0060089	<1e-4	sll1873	GO:0003824	0.4943	ssl1690	GO:0004518	0.0117
sll1193	GO:0019637	<1e-4	slr1173	GO:0071840	<1e-4	sll5090	GO:0016462	<1e-4	slr1668	GO:0003824	0.4943	ssl0467	GO:0004518	0.0117
sll0630	GO:0009893	<1e-4	sll0309	GO:0019222	<1e-4	sll1254	GO:0022803	<1e-4	slr0989	GO:0003824	0.4943	ssl2971	GO:0004518	0.0117
sll1530	GO:0019752	<1e-4	sll0867	GO:0046128	<1e-4	slr0845	GO:0015075	<1e-4	slr0272	GO:0003824	0.4943	slr0967	GO:0004518	0.0117
sll0609	GO:0055082	<1e-4	sll0615	GO:0009132	<1e-4	ssr3571	GO:0015075	<1e-4	slr0789	GO:0003824	0.4943	sll6055	GO:0004518	0.0117
ssl5027	GO:0051347	<1e-4	sll1267	GO:0009150	<1e-4	slr2048	GO:0022803	<1e-4	sll0888	GO:0003824	0.4943	slr1852	GO:0008324	0.0117
ssl2595	GO:0019752	<1e-4	sll1232	GO:0019222	<1e-4	slr0519	GO:0016817	<1e-4	sll0837	GO:0003824	0.4943	ssl5025	GO:0004518	0.0117
slr1230	GO:0043933	<1e-4	sll0658	GO:0019751	<1e-4	sll1318	GO:0015293	<1e-4	slr0060	GO:0003824	0.4943	ssl1263	GO:0004518	0.0117
sll0867	GO:0006753	<1e-4	sll1164	GO:0080090	<1e-4	sll0853	GO:0022836	<1e-4	sll0740	GO:0003824	0.4943	ssl5068	GO:0004518	0.0117
sll5062	GO:0044283	<1e-4	slr1957	GO:0009059	<1e-4	sll0525	GO:0005342	<1e-4	slr0053	GO:0003824	0.4943	sll1192	GO:0004518	0.0117
slr0723	GO:0042455	<1e-4	slr1618	GO:0006576	<1e-4	slr1438	GO:0016817	<1e-4	sll0911	GO:0003824	0.4943	sll0875	GO:0004518	0.0117
slr1087	GO:0044271	<1e-4	slr7098	GO:0006793	<1e-4	ssr2553	GO:0015077	<1e-4	slr0664	GO:0003824	0.4943	sll1036	GO:0004518	0.0117
slr1236	GO:0006091	<1e-4	ssl3379	GO:0046164	<1e-4	slr1970	GO:0015267	<1e-4	slr0456	GO:0003824	0.4943	sll1873	GO:0008324	0.0117
slr6022	GO:0006721	<1e-4	sll0496	GO:0034654	<1e-4	slr7011	GO:0015291	<1e-4	sml0011	GO:0003824	0.4943	ssl0350	GO:0004518	0.0117
sll0572	GO:0043549	<1e-4	sll1186	GO:0019222	<1e-4	sll0803	GO:0015293	<1e-4	ssr1552	GO:0003824	0.4943	slr6087	GO:0004518	0.0117
slr0482	GO:0048518	<1e-4	sll1764	GO:0034660	<1e-4	sll0167	GO:0015405	<1e-4	sll1170	GO:0003824	0.4943	sll1891	GO:0004518	0.0117
sll1696	GO:0048878	<1e-4	sll1119	GO:0044275	<1e-4	ssl0461	GO:0022892	<1e-4	slr0742	GO:0003824	0.4943	slr1613	GO:0008324	0.0117
slr6012	GO:0065007	<1e-4	slr1391	GO:0005996	<1e-4	sll0518	GO:0008171	<1e-4	slr1762	GO:0003824	0.4943	sll8019	GO:0004518	0.0117
sll0930	GO:0009260	<1e-4	slr0818	GO:0071841	<1e-4	sll0249	GO:0015399	<1e-4	slr1977	GO:0003824	0.4943	ssr2998	GO:0004518	0.0117
sll0359	GO:0010468	<1e-4	sll0630	GO:0055082	<1e-4	slr0625	GO:0042623	<1e-4	sll0827	GO:0003824	0.4943	ssl3829	GO:0004518	0.0117
sll2011	GO:0009141	<1e-4	sll1938	GO:0072527	<1e-4	sll1766	GO:0016741	<1e-4	sll0752	GO:0003824	0.4943	slr6104	GO:0004518	0.0117
sll1511	GO:0048523	<1e-4	sll0898	GO:0051234	<1e-4	sll1752	GO:0022838	<1e-4	slr0358	GO:0003824	0.4943	sll0496	GO:0004518	0.0117
sll6053	GO:0006811	<1e-4	slr1811	GO:0010556	<1e-4	ssr6079	GO:0022891	<1e-4	sll1956	GO:0003824	0.4943	slr0318	GO:0008324	0.0117
sll0751	GO:0006721	<1e-4	sll0524	GO:0006082	<1e-4	sll1119	GO:0042623	<1e-4	slr1209	GO:0003824	0.4943	sll0564	GO:0008324	0.0117
slr0818	GO:0051347	<1e-4	sll0659	GO:0006811	<1e-4	ssr2318	GO:0016818	<1e-4	slr0680	GO:0003824	0.4943	slr1413	GO:0015031	0.0116
sll0283	GO:0009263	<1e-4	slr1194	GO:0050794	<1e-4	slr0581	GO:0015399	<1e-4	slr1258	GO:0003824	0.4943	slr0442	GO:0006807	0.0116
slr0145	GO:0051171	<1e-4	sll0249	GO:0009394	<1e-4	ssl8003	GO:0015267	<1e-4	ssr3300	GO:0003824	0.4943	ssl0832	GO:0006807	0.0116
slr1095	GO:0050794	<1e-4	ssr2998	GO:0051188	<1e-4	slr1425	GO:0015267	<1e-4	slr0650	GO:0003824	0.4943	sll1372	GO:0015031	0.0116
sll0888	GO:0006811	<1e-4	sll0423	GO:0031323	<1e-4	sll0252	GO:0015405	<1e-4	sll1512	GO:0003824	0.4943	sll0763	GO:0006807	0.0116
sll1949	GO:0050801	<1e-4	ssr8013	GO:0009059	<1e-4	ssl2807	GO:0015405	<1e-4	ssl0483	GO:0003824	0.4943	ssr1258	GO:0006807	0.0116
slr1107	GO:0006576	<1e-4	slr1442	GO:0044271	<1e-4	ssl0787	GO:0005342	<1e-4	slr0111	GO:0003824	0.4943	sll0572	GO:0015031	0.0116
ssr3300	GO:0051179	<1e-4	slr6104	GO:0043170	<1e-4	sll0872	GO:0005342	<1e-4	slr0962	GO:0003824	0.4943	slr1288	GO:0015031	0.0116
slr0702	GO:0051171	<1e-4	slr0468	GO:0051818	<1e-4	sll0225	GO:0015075	<1e-4	slr1413	GO:0003824	0.4943	sll1938	GO:0015031	0.0116
sll1921	GO:0019752	<1e-4	sll1630	GO:0009057	<1e-4	slr0109	GO:0022836	<1e-4	sll0995	GO:0003824	0.4943	slr1287	GO:0015031	0.0116
slr7080	GO:0051246	<1e-4	ssl3451	GO:0046483	<1e-4	sll5004	GO:0015399	<1e-4	sll0449	GO:0003824	0.4943	slr1383	GO:0015031	0.0116
slr0039	GO:0072522	<1e-4	sll1862	GO:0034654	<1e-4	ssr2060	GO:0043167	<1e-4	sll0564	GO:0003824	0.4943	sll0787	GO:0015031	0.0116
sll8002	GO:0019751	<1e-4	sll0473	GO:0048519	<1e-4	slr6101	GO:0015405	<1e-4	sll0602	GO:0003824	0.4943	slr1940	GO:0015031	0.0116
slr6064	GO:0042180	<1e-4	sll0238	GO:0009126	<1e-4	sll0585	GO:0015267	<1e-4	slr0654	GO:0003824	0.4943	sll1304	GO:0015031	0.0116
sll0498	GO:0034641	<1e-4	slr0334	GO:0050896	<1e-4	slr1464	GO:0022891	<1e-4	sll0539	GO:0003824	0.4943	sll1424	GO:0006807	0.0116
ssl1792	GO:0046164	<1e-4	ssr8047	GO:0072524	<1e-4	ssl0350	GO:0042623	<1e-4	sll1730	GO:0003824	0.4943	slr2125	GO:0015031	0.0116
sll1378	GO:0044282	<1e-4	ssl8005	GO:0065007	<1e-4	sll0688	GO:0016741	<1e-4	slr0605	GO:0003824	0.4943	slr1690	GO:0015031	0.0116
slr1210	GO:0048523	<1e-4	sll5026	GO:0042180	<1e-4	ssl0788	GO:0022832	<1e-4	slr0596	GO:0003824	0.4943	slr0605	GO:0015031	0.0116
sll0487	GO:0009394	<1e-4	sll1763	GO:0046907	<1e-4	ssr1951	GO:0060089	<1e-4	slr1062	GO:0003824	0.4943	sll0412	GO:0015031	0.0116
slr7098	GO:0043933	<1e-4	slr1223	GO:0006163	<1e-4	slr1150	GO:0015293	<1e-4	slr0510	GO:0003824	0.4943	slr7024	GO:0015031	0.0116

slr1206	GO:0045184	<1e-4	slr0374	GO:0072528	<1e-4	ssl5108	GO:0015075	<1e-4	slr0815	GO:0003824	0.4943	sll0269	GO:0006807	0.0116
sll0558	GO:0046483	<1e-4	slr0650	GO:0009308	<1e-4	slr0810	GO:0043492	<1e-4	sll1267	GO:0003824	0.4943	sll0103	GO:0006807	0.0116
sll7031	GO:0072528	<1e-4	sll0647	GO:0044275	<1e-4	slr0334	GO:0043167	<1e-4	sll0410	GO:0003824	0.4943	sll0909	GO:0015031	0.0116
sll0688	GO:0044260	<1e-4	slr2080	GO:0006793	<1e-4	slr0667	GO:0022832	<1e-4	ssr2009	GO:0003824	0.4943	ssr2201	GO:0006807	0.0116
sll0413	GO:0009309	<1e-4	ssl7038	GO:0048878	<1e-4	ssr2142	GO:0043492	<1e-4	slr1940	GO:0003824	0.4943	sll5026	GO:0008324	0.0114
sll0781	GO:0071554	<1e-4	sll0602	GO:0042455	<1e-4	sll0659	GO:0015075	<1e-4	ssl8008	GO:0003824	0.4943	slr5023	GO:0008324	0.0114
sll0397	GO:0009199	<1e-4	sll0793	GO:0043412	<1e-4	sll1285	GO:0016818	<1e-4	slr1128	GO:0003824	0.4943	slr0740	GO:0008233	0.0114
ssr2755	GO:0034654	<1e-4	slr1614	GO:0043648	<1e-4	slr6021	GO:0016818	<1e-4	slr1169	GO:0003824	0.4943	ssr6020	GO:0008324	0.0114
sll5030	GO:0044281	<1e-4	sll7030	GO:0052111	<1e-4	sll0008	GO:0015075	<1e-4	slr0924	GO:0003824	0.4943	slr1619	GO:0008324	0.0114
slr0516	GO:0051234	<1e-4	slr0569	GO:0009259	<1e-4	ssl1918	GO:0015291	<1e-4	sll1240	GO:0003824	0.4943	sll0361	GO:0008233	0.0114
slr7091	GO:0006720	<1e-4	sll1752	GO:0009308	<1e-4	sll1698	GO:0016462	<1e-4	ssl0739	GO:0003824	0.4943	sll0048	GO:0008324	0.0114
slr0692	GO:0016052	<1e-4	ssl3692	GO:0055082	<1e-4	sll0167	GO:0022891	<1e-4	sll0590	GO:0003824	0.4943	sll1389	GO:0008324	0.0114
sll0085	GO:0051234	<1e-4	slr1914	GO:0006721	<1e-4	ssr8047	GO:0043492	<1e-4	slr1152	GO:0003824	0.4943	sll0803	GO:0008324	0.0114
sll1411	GO:0019222	<1e-4	sll7063	GO:0009263	<1e-4	sll0063	GO:0015399	<1e-4	sll1241	GO:0003824	0.4943	sll1722	GO:0008324	0.0114
slr0431	GO:0019362	<1e-4	slr1611	GO:0051171	<1e-4	sll0445	GO:0015399	<1e-4	slr0318	GO:0003824	0.4943	slr1768	GO:0008324	0.0114
slr2103	GO:0048522	<1e-4	sll0505	GO:0065007	<1e-4	slr1541	GO:0043167	<1e-4	slr2125	GO:0003824	0.4943	slr8014	GO:0008324	0.0114
slr0907	GO:0051818	<1e-4	slr1270	GO:0009394	<1e-4	slr1385	GO:0015077	<1e-4	sll1752	GO:0003824	0.4943	slr6021	GO:0008324	0.0114
slr1866	GO:0019222	<1e-4	ssr5106	GO:0048878	<1e-4	slr1301	GO:0022836	<1e-4	sll1950	GO:0003824	0.4943	slr1162	GO:0008324	0.0114
sll5062	GO:0065007	<1e-4	slr1183	GO:0043436	<1e-4	sll0456	GO:0016462	<1e-4	slr1240	GO:0003824	0.4943	sll1961	GO:0008324	0.0114
slr1970	GO:0051347	<1e-4	slr0592	GO:0009893	<1e-4	sll1757	GO:0022892	<1e-4	sll1251	GO:0003824	0.4943	slr0151	GO:0008324	0.0114
slr2010	GO:0050896	<1e-4	slr2052	GO:0052188	<1e-4	ssr3402	GO:0015405	<1e-4	sll0225	GO:0003824	0.4943	slr0333	GO:0008324	0.0114
sll1135	GO:0080090	<1e-4	sll0740	GO:0008610	<1e-4	ssr7093	GO:0015293	<1e-4	sll0909	GO:0003824	0.4943	slr1069	GO:0008324	0.0114
ssl2064	GO:0046128	<1e-4	slr1789	GO:0009141	<1e-4	ssr0335	GO:0008171	<1e-4	slr1122	GO:0003824	0.4943	slr0594	GO:0008233	0.0114
slr1998	GO:0016053	<1e-4	sll1608	GO:0006732	<1e-4	sll0781	GO:0005342	<1e-4	sll0189	GO:0003824	0.4943	ssl2009	GO:0008324	0.0114
slr0789	GO:0009892	<1e-4	slr0272	GO:0006066	<1e-4	slr0440	GO:0042623	<1e-4	slr0149	GO:0003824	0.4943	ssr2611	GO:0008324	0.0114
sll0564	GO:0031323	<1e-4	slr0468	GO:0044106	<1e-4	slr7082	GO:0022832	<1e-4	sll0413	GO:0016020	0.4927	slr1068	GO:0008324	0.0114
sll7089	GO:0034654	<1e-4	sll1563	GO:0019752	<1e-4	sll0645	GO:0016746	<1e-4	slr1699	GO:0016020	0.4927	slr1914	GO:0008324	0.0114
ssr7036	GO:0034641	<1e-4	sll5109	GO:0048518	<1e-4	sll5032	GO:0008171	<1e-4	slr1636	GO:0016020	0.4927	slr1104	GO:0008324	0.0114
ssr0761	GO:0045184	<1e-4	slr6073	GO:0019752	<1e-4	slr2038	GO:0022836	<1e-4	slr1614	GO:0016020	0.4872	slr0964	GO:0008324	0.0114
slr1601	GO:0008610	<1e-4	slr1186	GO:0006811	<1e-4	sll1866	GO:0016817	<1e-4	ssl0750	GO:0016020	0.4872	sll0762	GO:0008324	0.0114
sll0996	GO:0044260	<1e-4	ssr2611	GO:0046164	<1e-4	slr1303	GO:0015291	<1e-4	sll0779	GO:0016020	0.4872	ssr6085	GO:0008324	0.0114
sll0103	GO:0046164	<1e-4	slr1087	GO:0006766	<1e-4	ssl5031	GO:0015291	<1e-4	sll1763	GO:0016020	0.4872	ssl1377	GO:0008233	0.0114
slr5102	GO:0034654	<1e-4	sll0997	GO:0001932	<1e-4	sll0909	GO:0042623	<1e-4	slr1591	GO:0008152	0.4775	sll7043	GO:0008324	0.0114
sll1853	GO:0072527	<1e-4	sll0740	GO:0009132	<1e-4	sll1797	GO:0043492	<1e-4	sll1446	GO:0008152	0.4775	ssl2733	GO:0008233	0.0114
slr1270	GO:0008150	<1e-4	slr1533	GO:0080090	<1e-4	sll0361	GO:0016746	<1e-4	slr0147	GO:0008152	0.4775	slr1306	GO:0008324	0.0114
ssr2201	GO:0044281	<1e-4	sll0069	GO:0044003	<1e-4	slr2122	GO:0043167	<1e-4	slr1773	GO:0008152	0.4775	sll1769	GO:0008324	0.0114
slr0755	GO:0071842	<1e-4	slr0821	GO:0046128	<1e-4	slr7059	GO:0022836	<1e-4	slr1774	GO:0008152	0.4775	slr1847	GO:0008233	0.0114
sll0478	GO:0009126	<1e-4	sll0860	GO:0009987	<1e-4	sll0448	GO:0042623	<1e-4	slr0108	GO:0008152	0.4775	sll0857	GO:0008324	0.0114
slr7010	GO:0005996	<1e-4	ssl3615	GO:0051246	<1e-4	slr7100	GO:0008171	<1e-4	sll1528	GO:0016020	0.474	slr0263	GO:0008233	0.0114
sll1717	GO:0009308	<1e-4	sll1939	GO:0031323	<1e-4	sll0172	GO:0015293	<1e-4	sll0446	GO:0016020	0.474	sll8033	GO:0008324	0.0114
ssl1464	GO:0048519	<1e-4	sll8007	GO:0022607	<1e-4	ssl2717	GO:0016817	<1e-4	slr1978	GO:0016020	0.474	slr1168	GO:0008324	0.0114
ssr2554	GO:0045184	<1e-4	slr0038	GO:0044248	<1e-4	slr1444	GO:0022838	<1e-4	slr1773	GO:0016020	0.474	slr1800	GO:0008233	0.0114
slr0919	GO:0046164	<1e-4	sll0406	GO:0051246	<1e-4	ssr6019	GO:0008171	<1e-4	ssr0692	GO:0016020	0.474	sll0241	GO:0008324	0.0114
slr0602	GO:0046907	<1e-4	sll5003	GO:0008150	<1e-4	slr0941	GO:0060089	<1e-4	sll1995	GO:0016020	0.474	slr1363	GO:0008324	0.0114
slr0815	GO:0055114	<1e-4	slr0598	GO:0009199	<1e-4	sll0148	GO:0015075	<1e-4	sll1272	GO:0016020	0.474	sll5033	GO:0008324	0.0114
ssr3122	GO:0071841	<1e-4	sll0102	GO:0045184	<1e-4	slr6063	GO:0008171	<1e-4	slr0765	GO:0016020	0.474	slr1813	GO:0008324	0.0114
ssr2551	GO:0006631	<1e-4	slr5012	GO:0034654	<1e-4	sll1289	GO:0042623	<1e-4	slr0147	GO:0016020	0.474	sll1562	GO:0008233	0.0114
slr0195	GO:0051179	<1e-4	sll1062	GO:0051179	<1e-4	ssr7093	GO:0022832	<1e-4	slr0818	GO:0016020	0.474	ssr6032	GO:0008324	0.0114
slr1223	GO:0009991	<1e-4	slr0702	GO:0046164	<1e-4	ssr2060	GO:0022891	<1e-4	sll1214	GO:0016020	0.474	ssl5031	GO:0008324	0.0114
slr1546	GO:0044275	<1e-4	sll1132	GO:0006576	<1e-4	sll0268	GO:0060089	<1e-4	sll0898	GO:0016020	0.474	slr1273	GO:0008324	0.0114
slr0645	GO:0019752	<1e-4	sll0293	GO:0031323	<1e-4	sll0872	GO:0043492	<1e-4	slr0642	GO:0016020	0.474	sll1866	GO:0008324	0.0114
sll0414	GO:0006720	<1e-4	ssr6019	GO:0009262	<1e-4	sll1866	GO:0022857	<1e-4	sll1858	GO:0016020	0.4431	slr0978	GO:0008233	0.0114
ssr3572	GO:0019362	<1e-4	sll0763	GO:0009262	<1e-4	slr0149	GO:0016462	<1e-4	sll2007	GO:0016020	0.4431	slr1611	GO:0008324	0.0114
sll1160	GO:0051179	<1e-4	sll0543	GO:0009893	<1e-4	slr0151	GO:0005342	<1e-4	sll0804	GO:0016020	0.4431	slr1178	GO:0008324	0.0114

slr7100	GO:0010468	<1e-4	slr0960	GO:0046907	<1e-4	slr0013	GO:0022892	<1e-4	sll0661	GO:0016020	0.4431	slr1195	GO:0008324	0.0114
ssl3615	GO:0006720	<1e-4	slr1977	GO:0055086	<1e-4	sll0177	GO:0022838	<1e-4	ssl1255	GO:0016020	0.4431	slr0909	GO:0008233	0.0114
slr1854	GO:0016054	<1e-4	slr5016	GO:0051179	<1e-4	ssl2595	GO:0015293	<1e-4	slr1702	GO:0016020	0.4431	slr1397	GO:0008324	0.0114
sll0354	GO:0046483	<1e-4	ssr1041	GO:0044106	<1e-4	sll7006	GO:0060089	<1e-4	slr0359	GO:0016020	0.4431	sll1880	GO:0008324	0.0114
slr1956	GO:0071841	<1e-4	ssr1765	GO:0008150	<1e-4	sll1247	GO:0022891	<1e-4	slr0810	GO:0016020	0.4431	sll1396	GO:0008324	0.0114
[truncated: 351,676 more chars]
